# Exploring online consumer behavior on fraudulent energy-saving products

**DOI:** 10.1038/s41598-024-65210-1

**Published:** 2024-06-21

**Authors:** Pingfei Li, Dingwei Zheng, Li Yan, Qianxi Zhou

**Affiliations:** 1 School of Automobile and Transportation, Xihua University, Chengdu, 610039 China; 2 Vehicle Measurement Control and Safety Key Laboratory of Sichuan Province, Xihua University, Chengdu, 610039 China

**Keywords:** Fake energy-saving products, Sustainable development, Energy-saving behavior, Purchase intention, Energy and society, Psychology and behaviour, Sustainability

## Abstract

Purchasing energy-saving products is key for public participation in energy conservation and sustainable development. However, the sale of fraudulent energy-saving products has boomed through online shopping, with little research on these products and consumer demands. This study explored the underlying factors driving consumer purchases of fraudulent energy-saving products and measured their impact on environmental awareness. Sales data for such products from four major online shopping platforms were collected. Results suggested unique demand characteristics from consumers who unknowingly purchase fraudulent energy-saving products, referred to as “hidden energy savers”, including a preference for moderately priced products, a desire for straightforward energy-saving explanations, and a tendency to seek multiple additional features, even if they conflict with the core functionality. Perceived installation and usage difficulty significantly influences purchasing behavior. A practical survey of freight companies and individual transporters’ demand for freight energy-saving products was conducted as a case study to validate the practical application of this research. This study presents a novel perspective on public energy-saving behavior, aiding in creating true energy-saving products, boosting public energy conservation interest, and reducing the negative impact of fraudulent products on environmental awareness. It also sheds light on hidden consumer needs, guiding the development of authentic energy-saving products.

## Introduction

Energy-saving behavior is an effective method for mitigating the negative environmental impacts of human energy-generating activities^1^. Governments worldwide have implemented policies to promote energy-saving behaviors among citizens and integrate these practices into everyday life. Significant initiatives, such as the Paris Agreement^2^, and carbon reduction targets the European Union^3^ and China^4^ set, exemplify these efforts. The energy-saving behaviors of individuals are a key component of these strategies, as home energy users in Europe, such as TVs, fridges, and air conditioners, account for more than 30% of the total energy used^5^. The urgency of these behaviors has been amplified due to recent energy crises, precipitated by events such as the Russo-Ukrainian War and disruptions in natural gas supplies^1^.

**Figure 1:**
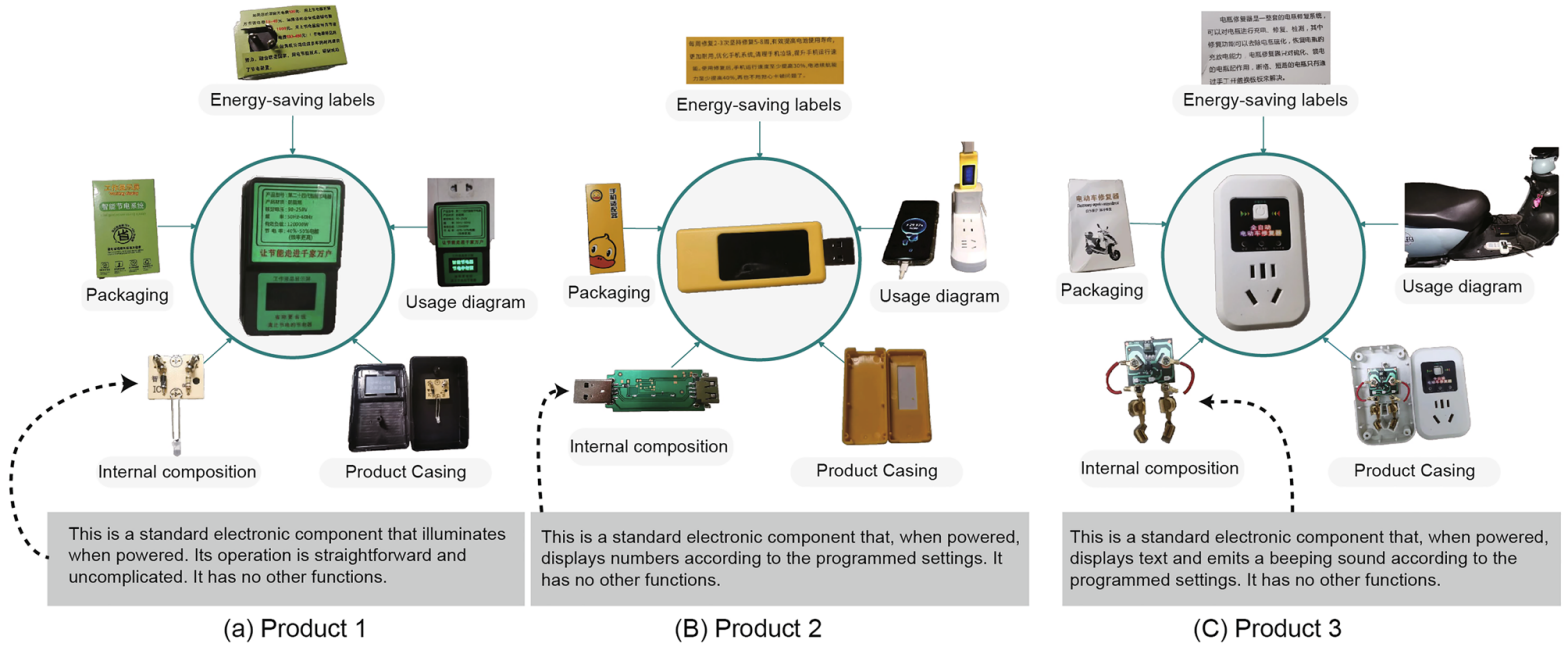
Detailed view of three counterfeit energy-saving products and their core components.

As the demand for energy conservation grows, the market has seen a rise in energy-saving products. However, there is a troubling increase in the sale of fraudulent energy-saving products online, with significant trading volumes. Unlike counterfeit products, these fraudulent energy-saving products do not imitate any genuine energy-saving products but instead are based on fabricated features, fictional energy-saving theories, or false descriptions, thus establishing a unique branch of energy-saving products. This prevents them from being mistaken for counterfeit goods and restricted by e-commerce platforms. Figure 1 presents the disassembled views of counterfeit energy-saving products commonly sold online, each with sales exceeding 10,000 pieces. The internal components of these products are simplistic, primarily designed to power an LED light, display digits, or increase electrical resistance. Consequently, these products lack any genuine energy-saving capabilities. Consumers who unwittingly purchase these ineffective products are called hidden energy savers. Their efforts at energy conservation are often wasted because they do not result in real energy savings. Nevertheless, these hidden consumers have a real desire to save energy and are willing to incur certain financial expenditures to achieve such savings. Unfortunately, the market lacks genuine energy-saving products that meet their specific needs. Hence, exploring the purchasing behavior of these hidden energy savers and the characteristics of fraudulent products will help online sales platforms identify and ban these fraudulent products. This can also encourage manufacturers to develop products that more closely align with consumer demands, reducing the market share of fraudulent energy-saving products, protecting consumer interests, and promoting energy conservation and emission reduction.

Moreover, the scale of counterfeit energy-saving products is considerable: According to the author’s research data, in just the first half of 2023, over 512,511 fraudulent energy-saving products were sold on China’s four major online shopping platforms: Taobao, Jingdong, Pinduoduo, and Tik Tok (China). In contrast, based on the same research data, genuine energy-saving products of similar types accounted for only 269,025 sales during the same period. These counterfeit products mislead consumers and exploit weaknesses in online retail systems, capturing a market share that should belong to genuine energy-saving products. Additionally, they may undermine public commitment to environmental conservation and indirectly prompt increased energy use by giving a false impression of energy efficiency. Despite these issues, there is still little research focusing on counterfeit energy-saving products.)

There has been extensive research on energy-saving behaviors and patterns of the public. In a standard consumer decision-making process, consumers begin by evaluating factors such as product quality, price, and service, which leads to a purchase decision that meets their specific needs. During this process, a range of internal factors such as consumption habits, perceived effectiveness, price sensitivity, social responsibility, and knowledge of environmental issues, along with external factors such as the social environment, personal norms, and guidance from external information, influence enthusiasm and initiative towards energy-saving consumption^6^. The surge in online shopping platforms has led to a broader focus on online consumer behaviors. Compared to traditional shopping, online shopping decisions are often influenced by additional factors, including online reviews^7^, product labels, return policies, and visual information from product images and videos.

However, the studies typically face two issues: First, it is difficult to obtain actual sales data for analysis on products that are desired for their alleged energy-saving features, as well as consumer preferences for these non-existent functions. This reliance makes it challenging to genuinely understand the public’s behavior patterns and demands for non-existent energy-saving products, leaving potential energy-saving behavior patterns and needs largely unexplored. Second, in existing research, purchasers of fraudulent energy-saving products have been largely overlooked, despite forming a significant market segment. Their behavior differs from that of the broader consumer pool because the product descriptions on websites are filled with contradictions, inaccuracies, and false claims. A deeper investigation into the buyers of these fraudulent products can help rapidly expand the energy-saving market and reduce energy waste caused by the sale and use of fraudulent energy-saving products. By analyzing the public’s behavior in response to these fraudulent products, researchers can gain insights into the latent expectations and demands for energy-saving products, thereby guiding the development of actual energy-saving products that meet these needs and promote energy conservation. Additionally, assessing the extent to which fraudulent energy-saving products erode environmental consciousness can complement existing research on the relationship between environmental awareness and energy-saving behavior, leading to more accurate predictions regarding environmental behaviors and the market for eco-friendly products.

This article aims to identify the behavioral traits and influencing factors of hidden energy savers in purchasing energy-saving products, thereby expanding the scope of energy-efficiency promotion. Specifically, the objectives are divided into four points: (a) to identify the latent needs of potential energy-saving product consumers regarding product function and price; (b) to identify the factors influencing the purchase behaviors of fraudulent energy-saving products and establish a predictive framework for such consumer behavior; (c) to estimate the current energy wastage caused by fraudulent energy-saving products; and (d) through comparing our research findings on energy-saving product demands with survey data from freight companies and individual transporters, we aim to validate the practical application value of our research findings.

The study can provide recommendations on consumer demand for the development of new energy-saving products, which will assist manufacturers in launching products that better meet market demands. It also aids in transitioning consumers of fraudulent energy-saving products into actual energy-saving products, expanding the market for energy-saving products and promoting the development of energy conservation awareness. Furthermore, it can contribute to promoting energy conservation and the development of a sustainable society.

Key innovations include: (a) augmenting energy-saving behavior research by exploring new subjects (hidden energy savers) and new behaviors (online purchasing of counterfeit energy-saving products), uncovering the potential energy-saving needs, behaviors, and influencing factors of a frequently overlooked group. This contributes to expanding the new energy-saving market; (b) utilizing the Random Forest-Boruta algorithm instead of the commonly used linear regression analysis to unearth the nonlinear relationships between product characteristics and energy-saving behaviors; (c) proposing a novel everyday energy-saving approach, namely curbing the sale of counterfeit products, and estimating their energy-saving effects; and (d) estimating the hidden product demand, to fill gaps left by survey questionnaires and actual behavior studies, offering valuable insights for incentive policy research across multiple fields.

The contributions of this paper include: (a) identifying the consumption behavior characteristics of consumers of fraudulent energy-saving products, a previously overlooked group, which sheds light on neglected demand directions for the development of new energy-saving products; (b) aiding policymakers and online shopping platform operators in more accurately identifying and regulating these fraudulent products, thereby reducing the waste of energy and depletion of conservation sentiment associated with such products; (c) providing recommendations for market expansion and consumer incentives for various products, including but not limited to new energy vehicles; and (d) enhancing the theoretical research on green product consumption behavior, covering aspects such as demographic characteristics, product features, and functional design.

## Literature review

Research identifies five main factors influencing the purchase of online energy-saving products: Price, Online Reviews, Product Attributes, Energy Efficiency Labels, and Shopping Experience.

*Price* Price directly influences the purchase of green products. Studies indicate that individuals with lower energy-saving awareness are more price-sensitive, often choosing cheaper, less efficient products over pricier, energy-saving alternatives^8^. Furthermore, the influence of income on green purchasing implies the important role of price: Income is a significant barrier to energy-saving behaviors^9^. Specifically, income inequality inhibits the improvement of carbon emission efficiency^10^, while higher income promotes the adoption of energy-efficient behaviors^11^. Furthermore, when people have a more optimistic perception of having a higher future income, the time between forming low-carbon intentions and engaging in low-carbon behaviors is reduced^12^. This provides a possible explanation for the consumption of fraudulent energy-saving products, as these products may appear to offer a better quality/price ratio.

*Online reviews* Positive online customer reviews increase the likelihood of purchase, while in the face of negative reviews, perceived credibility and perceived diagnostic significantly impact purchasing decisions^13^. The number of reviews boosts early sales, while pricing strategies influence later stages, and polarized reviews positively impact sales at all stages^14^. Although direct research is lacking, some studies imply behaviors related to fraudulent green consumption: consumers usually lack the knowledge and skills to identify false reviews, which can lead them to rely on positive (albeit fake) reviews when making purchasing decisions^15^. However, it should be noted that overwhelmingly positive reviews can sometimes raise suspicion among consumers, improving their perceived ability to detect fake reviews. Despite this, in our context, hidden energy savers, who are usually less critical and more trusting of online reviews, are more likely to be influenced by positive reviews, as they are often seeking low-cost solutions and may overlook inconsistencies in reviews.

Hence, we proposed our first hypothesis:

### H1

Low prices and positive reviews encourage hidden energy savers to buy counterfeit products.

*Product attributes* Product Attributes such as energy efficiency level, control modes, usage methods, and types of functions suggest a significant impact on purchasing behavior^16^.

*Energy efficiency labels* Consumers are more likely to buy products with better energy efficiency, indicating high sensitivity to these labels, but the labels can sometimes mislead consumers into using the products more frequently, potentially negating energy savings or even increasing energy consumption^5^. This also suggests that buyers of counterfeit energy-saving products may misinterpret the information on energy labels, leading to the purchase of products that, despite being labeled as energy-efficient, still consume substantial amounts of energy, thereby creating a different kind of energy waste. Furthermore, the willingness to pay for products with different energy efficiency labels varies among different demographic groups: The impact of energy efficiency labels on the willingness to pay is less significant among less educated individuals; and higher education levels in first-level cities do not necessarily correlate with a higher willingness to pay^17^. This suggests potential characteristics of consumers of fraudulent energy-saving products-consumers of such products are likely to be less educated (as they are more susceptible to being deceived by simple fraudulent claims), hence energy efficiency labels, especially the data and specific meanings they contain, may not influence their willingness to pay.

Hence, we proposed our second hypothesis:

### H2

Product attributes and the specific values of energy efficiency labels do not influence the purchasing decisions of hidden energy savers.

## Methodology

### Research framework

To identify potential energy-saving behaviors among the hidden energy savers, the study is organized into four key parts: (a) text clustering on descriptions of fake energy-saving products to categorize and analyze the characteristics of different types; (b) statistical analysis to reveal features of product functionality, and the relationship between price, energy-saving labels, reviews, and sales volume; and utilizing Random Forest and Boruta algorithms to explore whether and how price, reviews, product attributes, and energy labels influence hidden energy savers’ purchasing behaviors; (c) comparing the adoption demands predicted based on this study’s conclusions with those from survey data to validate the effectiveness and significance of the study; and (d) validating the accuracy of this study’s analysis through the examination of actual demand data for freight energy-saving products and energy-saving measures from freight companies and individual transporters. The reason for using survey data from the freight companies and individual transporters groups for validation is that in Section “Results”, we explored the characteristics of the hidden energy savers group and identified certain similarities with the freight companies and individual transporters groups, making them a relevant reference. The detailed reasons for this selection are provided in Section “Validation and implications”.

**Figure 2:**
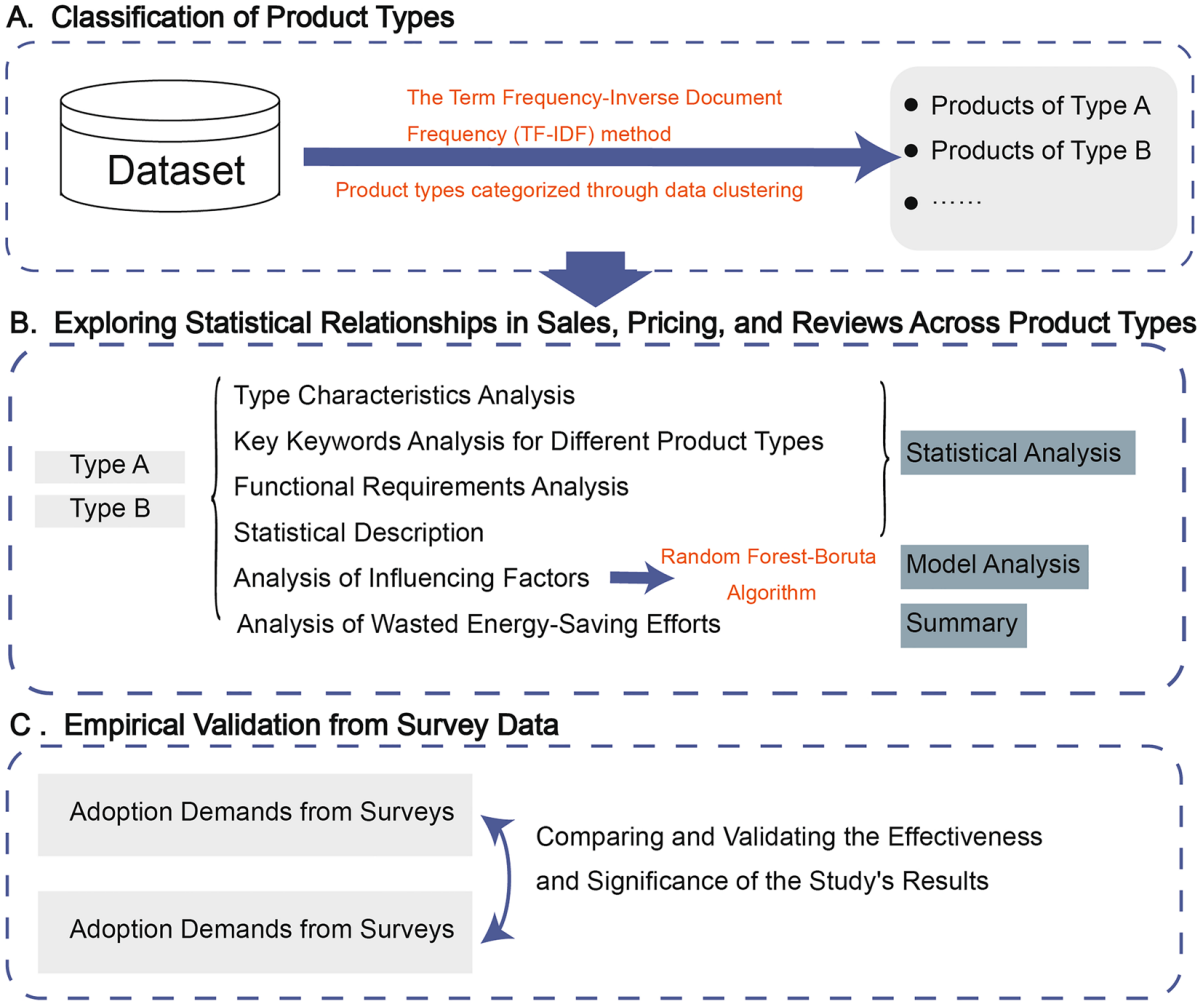
Research framework.

The research idea is also shown in Fig. 2 below:

### The term frequency-inverse document frequency (TF-IDF) method

The TF-IDF algorithm is a widely used weighting function in text clustering and information retrieval. It evaluates the significance of a word in a document or a collection of documents^18^. TF-IDF primarily comprises two components: (a) TF (Term Frequency), which counts the occurrence of each term in a document; and (b) IDF (Inverse Document Frequency), which adjusts the term’s weight by assuming its importance is inversely proportional to its frequency across all documents in a corpus^19^. The TF, IDF, and TF-IDF values for word *i* in document *j* are expressed in Eqs. (1)–(3):

1 $$T{F}_{ij}=\frac{{n}_{ij}}{Dj}$$

2 $$ID{F}_{i}=\mathit{lg}\left(\frac{D}{{N}_{i}}\right)$$

3 $$TF-ID{F}_{ij}=T{F}_{ji}\cdot ID{F}_{i}$$

where *D*_*j*_ represents document *j* and *D* is the total number of documents; *n*_*ij*_ is the frequency of word *i* in document *j*, and *N*_*i*_ is the total frequency of word *i* in all documents.

After calculating the TF-IDF values for each word in document *j*, these values serve as the feature vector for the document. We use the k-means clustering method to cluster documents based on their feature vectors, completing the TF-IDF text clustering process. Additionally, to address the limitation of k-means clustering in objectively determining the number of clusters, the elbow method was incorporated to identify the optimal cluster count.

### Random Forest-Boruta algorithm

The Random Forest (RF) method is a commonly used machine learning method for fitting nonlinear relationships. It builds upon decision tree models, integrating multiple, relatively uncorrelated decision trees to mitigate overfitting and local convergence issues^20^. The model includes two randomization processes: bootstrapping, addressing data imbalance through resampling, and random feature selection, preventing potential correlations between the base trees. The final prediction is based on aggregated decisions of numerous trees, employing majority voting for classification and numerical aggregation (like averaging) for regression.

The reason for using the RF regression model rather than conventional linear regression models is: (a) The study of counterfeit energy-saving products and their sales involves a dataset with many variables and high dimensionality, as our research focused on products rather than buyers’ data. Compared to traditional methods, RF is more suitable for high-dimensional datasets; (b) predictably, the attributes and functions of products might exhibit multicollinearity, significantly reducing or even invalidating the model’s predictive accuracy. Linear regression would involve the subjective elimination of collinear variables, affecting our response strategy for hidden energy savers. The RF method, however, is less sensitive to multicollinearity and does not require variable elimination. Therefore, compared to traditional regression methods, employing Random Forest (RF) will more effectively address the issues of multicollinearity among product attributes and functions. It allows for the analysis of the influence of multiple indicators with multicollinearity, without the need to eliminate any indicators; (c) more importantly, in the RF regression model, potential interactions between variables are measured simultaneously, closer to the decision-making process of hidden energy savers when purchasing energy-saving products. As a comparison, typical significance test or sensitivity analysis will alter the value of one explanatory variable and then estimate the change of output at one time. This also makes our study more closely aligned with real-world conditions.

However, the limitation of the RF regression model is its weak interpretability, making it difficult to clearly reflect the significance of variables. Hence, the Boruta algorithm is employed as a complement. It validates the importance of explanatory variables through comparison with their randomly shuffled counterparts, achieving stable and clear results through multiple iterations. The Boruta algorithm includes the following steps: First, each feature in the original dataset is randomly permuted to create “shadow features”. These shadow features have the same value distribution as the original features but are randomly shuffled. Then, a random forest model is trained using both the original features and the shadow features. Then, the importance of all features, including both the original and shadow features, is computed using the random forest model. The primary measure used is the Mean Decrease in Accuracy (MDA), which measures the average reduction in accuracy caused by permuting the feature, averaged over all trees in the forest.

For each original feature *X*_*j*_, the specific formula is:

4 $$MDA\left({X}_{j}\right)=\frac{1}{N}\sum_{i-1}^{N}({Accuracy}_{original}-{Accuracy}_{permuted})$$

where Accuracy_original_ is the model accuracy using the original features, Accuracy_permuted_ is the model accuracy after permuting feature *X*_*j*_, and Nis the number of trees in the random forest. The same method is used to calculate the importance of the shadow features.

Finally, The importance of each original feature *I*(*X*_*j*_) is compared with the maximum importance among the shadow features *I*(*S*_*max*_), to determine whether the variable *X*_*j*_ is an important factor:

- If *I*(*X*_*j*_) > *I*(*S*_*max*_), the feature *X*_*j*_ is classified as “important”;
- If *I*(*X*_*j*_) < *I*(*S*_*max*_), the feature *X*_*j*_ is classified as “unimportant”;
- If *I*(*X*_*j*_) ≈ *I*(*S*_*max*_), the feature *X*_*j*_ is classified as “tentative”;

## Data sources

### Sales data of fraudulent energy-saving products

The data on fraudulent energy-saving products was collected in two stages: first, identifying the fraudulent energy-saving products on online shopping platforms, and second, gathering sales data for these fraudulent products.

*Identification of fraudulent energy-saving products:* During the data collection phase, we identified fraudulent energy-saving products by conducting manual searches on major online shopping platforms using relevant keywords such as “energy saver”, “energy-saving magic”, “battery repair”, and “energy-saving miracle”. We sorted the results by price to identify low-cost products. Next, we reviewed the product descriptions to check for unrealistic energy-saving functions, significant errors, and exaggerated claims. Products with such descriptions were flagged and included in our data collection. For products where it was unclear whether they were fraudulent, such as those describing real energy-saving functions but priced significantly lower than similar genuine products, we purchased and tested these items. Thanks to fair online shopping return policies, we were able to return most of the fraudulent products we bought, keeping a few as samples. It is also worth noting that online shopping platforms generally do not allow the sale of such fraudulent products, so despite their high total sales volume, the actual number of these products is relatively low, making manual screening feasible. This thorough process ensured the accuracy and reliability of the data used in our study.

*Sales data of fraudulent energy-saving products* By using keywords such as ‘save energy’, ‘save electricity’, and ‘reduce energy consumption’, we extracted data on products, sales, and customer reviews from the four largest online shopping platforms in China: Taobao, Jingdong, Pinduoduo and TikTok (China). We collected information on 301 fraudulent energy-saving products with a combined sales volume exceeding 1,469,396 units, along with 139,562 customer reviews. Detailed data can be found in Supplementary Tables 1-5. A statistical overview of the basic data is presented in Table 1 below:

**Table 1:** Descriptive statistics of product sales data.

Metric	Mean	Max.	Min.	S.D.
Sales	1389.22	112,544	0	8009.00
Number of reviews	107.98	5671	0	443.08
Price	82.97	2680.0	1.01	240.21
Proportion/%				
Online shopping platforms	1	Taobao	40.43%	
	2	Pinduoduo	25.88%	
	3	Jingdong	24.53%	
	4	Tik Tok (China)	9.16%	
Average electricity saving rate (claimed)	1	0–20%	76.88%	
	2	20–30%	9.14%	
	3	30–40%	3.76%	
	4	40–50%	10.22%	
	5	More than 50%	0.00%	
The repair rate of used batteries (claimed)^a^	1	0–50%	69.19%	
	2	50–100%	20.00%	
	3	100–200%	8.65%	
	4	More than 200%	2.16%	

^a^The repair rate of used batteries shows The percentage increase in battery lifespan claimed by the product. Calculated as: $\frac{{T_{3} - T_{2} }}{{T_{1} }} \times 100{\text{\% }}$, where *T*_1_ is the original lifespan of a new battery, *T*_2_ is the reduced lifespan after use, and *T*_3_ is the extended lifespan after using the product. These fraudulent products often deceive consumers by claiming high repair rates without being accountable for actual performance. As a result, some products’ repair rates can exceed 100%

## Results

### Analysis of the hidden energy-saving demands

#### Product categorization

**Figure 3:**
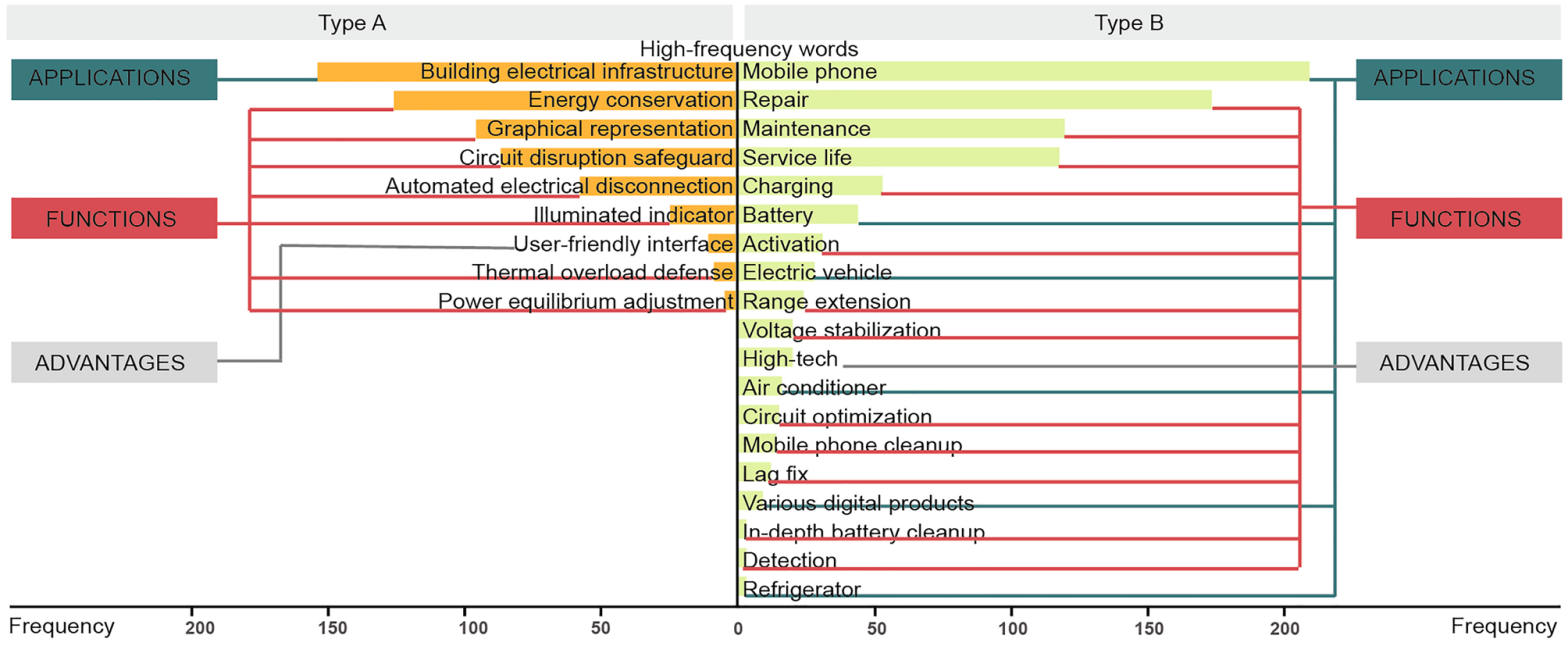
Distribution of high-frequency words in text clustering.

The Term Frequency-Inverse Document Frequency (TF-IDF) method was used for text clustering on the product descriptions of the fraudulent energy-saving items gathered. The analysis identified that two was the optimal number of clusters. Figure 3 displays the primary keywords (those with a frequency above two) and their distribution within the two clusters identified.

The cluster analysis shows that fake energy-saving products fall into two main types:

- Type A: These products are utilized in building circuits, primarily to reduce energy consumption both in the circuits themselves and in devices connected to these circuits within the buildings.
- Type B: These are primarily designed for battery-powered phones, electric bikes, and common high-energy appliances (like air conditioners and refrigerators). Their main functions are to repair or extend battery life and reduce the energy consumption of high-power items. They are typically installed at battery interfaces or directly on the appliance plugs.

#### Analysis of functional requirements

**Figure 4:**
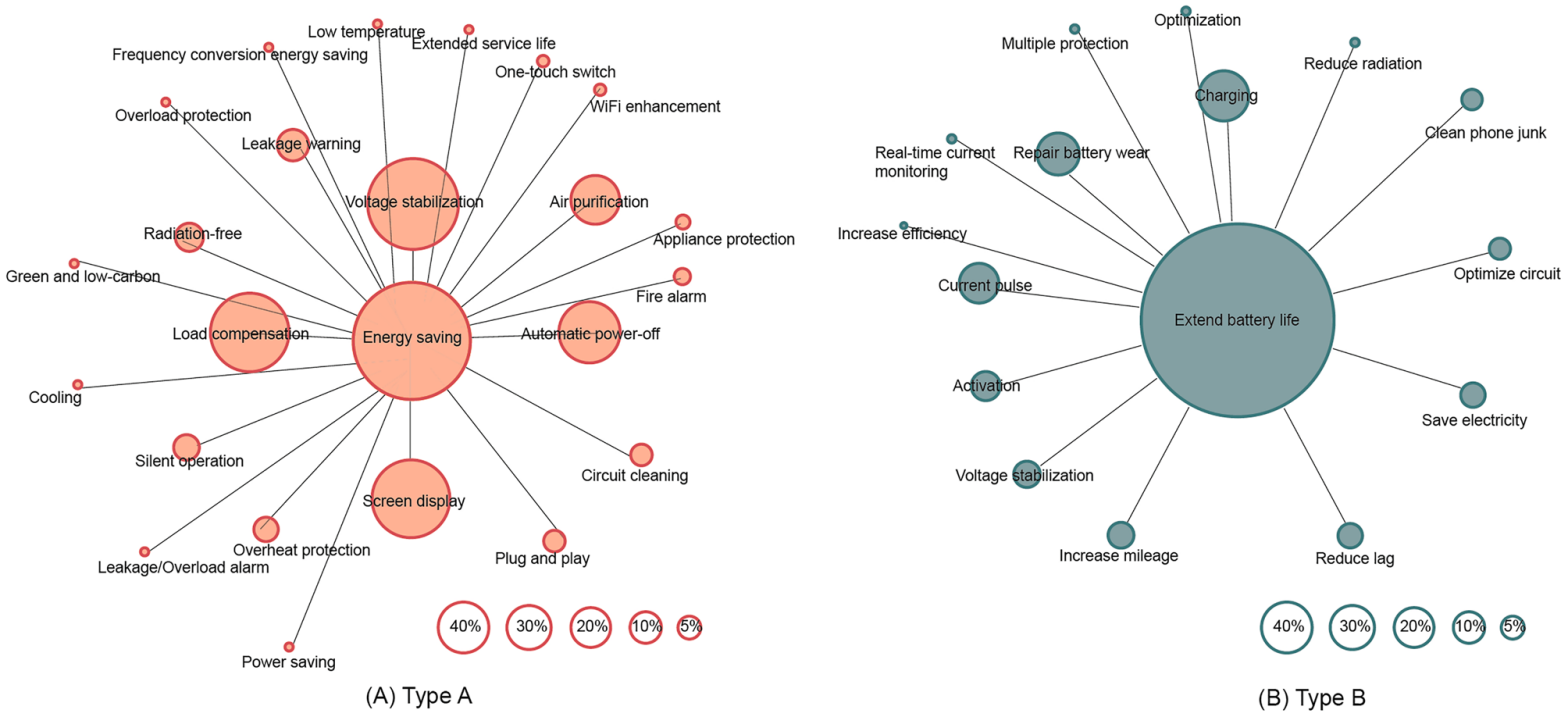
Product functions and their sales shares.

The key functions of both product types and the sales share of each product’s additional functions are shown in Fig. 4 below, with larger bubbles indicating higher proportions. It’s important to note that each product might have several additional functions. Additionally, certain functions are either non-existent, impractical (including functions like circuit optimization, current pulse battery repair, and load compensation), or meaningless (such as ‘Activation’, ‘Optimization’, and ‘Multiple protection’).

For Type A, Voltage Stabilization and Load Compensation stand out as the additional functions with the highest representation. This is likely because sellers need to provide a scientific basis for how their products save energy, suggesting that by stabilizing voltage via Load Compensation, they can reduce energy wastage and enhance efficiency in related devices. This reflects the public’s interest in understanding the rationale behind energy-saving measures. The popularity of the Screen Display function indicates consumers’ desire for straightforward feedback on energy saved. Interestingly, the Air Purification stands out with a high proportion of 23.53%, an unrelated function that paradoxically consumes electricity instead of conserving it. This trend might highlight consumers’ preference for additional special functions, even when they conflict with energy conservation, a point further evidenced by the presence of functions like air purification, ultrasonic pest repelling, and Wi-Fi boosting. Moreover, the ‘Plug and Play’ function suggests a preference for devices that are easy to install and use. The perceived difficulty of use significantly impacts adoption decisions. A case in point is the challenge with adopting home solar panels: their large size and the need for circuit modifications make them seem too complex to use, which has greatly affected their uptake^21^, even when some sellers offer free installation.

For Type B, aside from the function to delay/repair battery wear, the additional Charging feature has the highest proportion at 38.92%. This feature, essentially transforming the product into a charger, bridges the power source and battery-containing devices. Two reasons likely contribute to this feature’s inclusion: first, it underscores the product’s ease of installation and use, mirroring familiar chargers; second, it aids in elucidating the principle of battery repair, especially through the ‘Current Pulse’ function (24.32%). This aligns with findings for Type A, where consumers prefer understandable energy-saving principles and user-friendly products. Notably, Type B has fewer additional functions with lower proportions than Type A. This may be due to differing usage environments, with Type A intended for more attentive indoor areas, whereas Type B’s diverse application contexts potentially diminish the appeal of extra features.

#### Statistical relationships between sales, price, energy efficiency labels, and reviews

**Figure 5:**
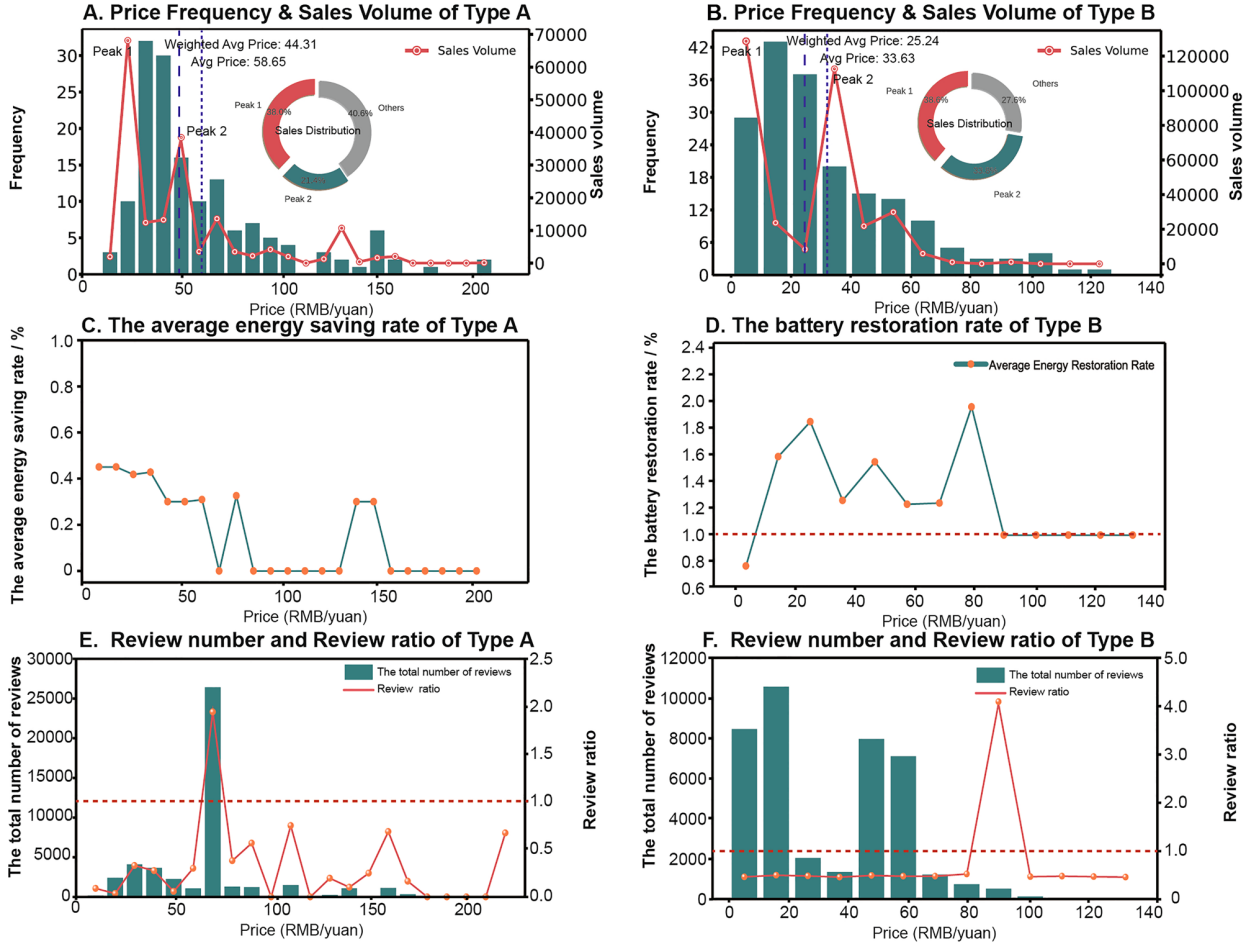
Statistical relationships between sales, price, energy efficiency labels, and reviews.

The distribution of online prices for Type A and B products is depicted in Fig. 5A,B, respectively, with sales volumes across various price ranges shown using line charts. Moreover, the average energy-saving and battery restoration rates in different price segments are illustrated in Fig. 5C,D. The total number of reviews and the review rates for different price segments are presented in Fig. 5E,F. Notably, the review rate indicates the ratio of total reviews to total sales within the price range. A review rate exceeding 1 suggests a high prevalence of fabricated reviews, as the total number of reviews should not surpass total sales.

The above illustrations reveal that:

1. Counterfeit energy-saving products are predominantly low-priced, with sales-weighted average prices of 44.31 and 25.24 yuan, respectively. However, the sales data also show that the highest sales volumes are not at the lowest price points. This suggests that hidden energy savers do not always choose the cheapest option, as excessively low prices might lead them to question the product’s authenticity. It is supported by the sales peaks: energy-saving devices have two peaks in the [10, 20] and [40, 50] price ranges, with sales of 68,153 and 38,461 units, respectively. Battery restorers also exhibit two peaks, in the [0, 10] and [30, 40] ranges, with sales of 128,609 and 112,612 units, respectively. This points to two consumer types: (a) those solely price-motivated, selecting the least expensive product that fits their requirements, and (b) prudent consumers guided by price, skeptical of rock-bottom prices, and more prone to select moderately priced goods. This notion is further supported by the second sales peak in both categories, which corresponds closely with the average prices on the platform.
2. Despite the primary consumer needs being energy conservation or delaying/repairing battery wear, there’s no significant correlation between energy-saving rates, restoration rates, and sales volumes. Additionally, the distribution of these core rates doesn’t appear to relate to pricing. Both suggest a certain level of impulsiveness in consumer choice, with less emphasis on the highest efficiency or restoration rates. On the one hand, vendors often inflate these core parameters due to no consideration for actual functionality, with the lowest claimed energy-saving rate at 1.25% and battery restoration rate at 73.5%, which potentially have met consumers’ needs. On the other hand, considering products claiming battery restoration rates exceeding 100% (accounting for 73.13% of all such products), some consumers may lack understanding of these parameters or disregard them entirely.
3. An interesting point about pricing is that consumers prefer to “fix” their phone batteries rather than just replace them with new ones, even when replacement costs are low. For e-bikes, the costs of replacing batteries are high, but many sellers offer a one-time free replacement. Meanwhile, the claimed battery restoration rate doesn’t significantly sway product sales. It’s indicated that the attraction of these products is enhanced by their promises to solve other prevalent issues with battery-powered items. Referring to the Product Functions in Fig. 2, these concerns include phone lag, battery overheating, phone radiation, batteries not charging after long periods of disuse, and insufficient e-bike mileage.
4. Preliminary analysis from the statistical graphs indicates a correlation between the number of reviews and sales volumes. However, a clear presence of fabricated reviews (indicated by review rates exceeding 1) does not appear to affect sales volumes.

#### Identifying the importance of influencing factors

**Figure 6:**
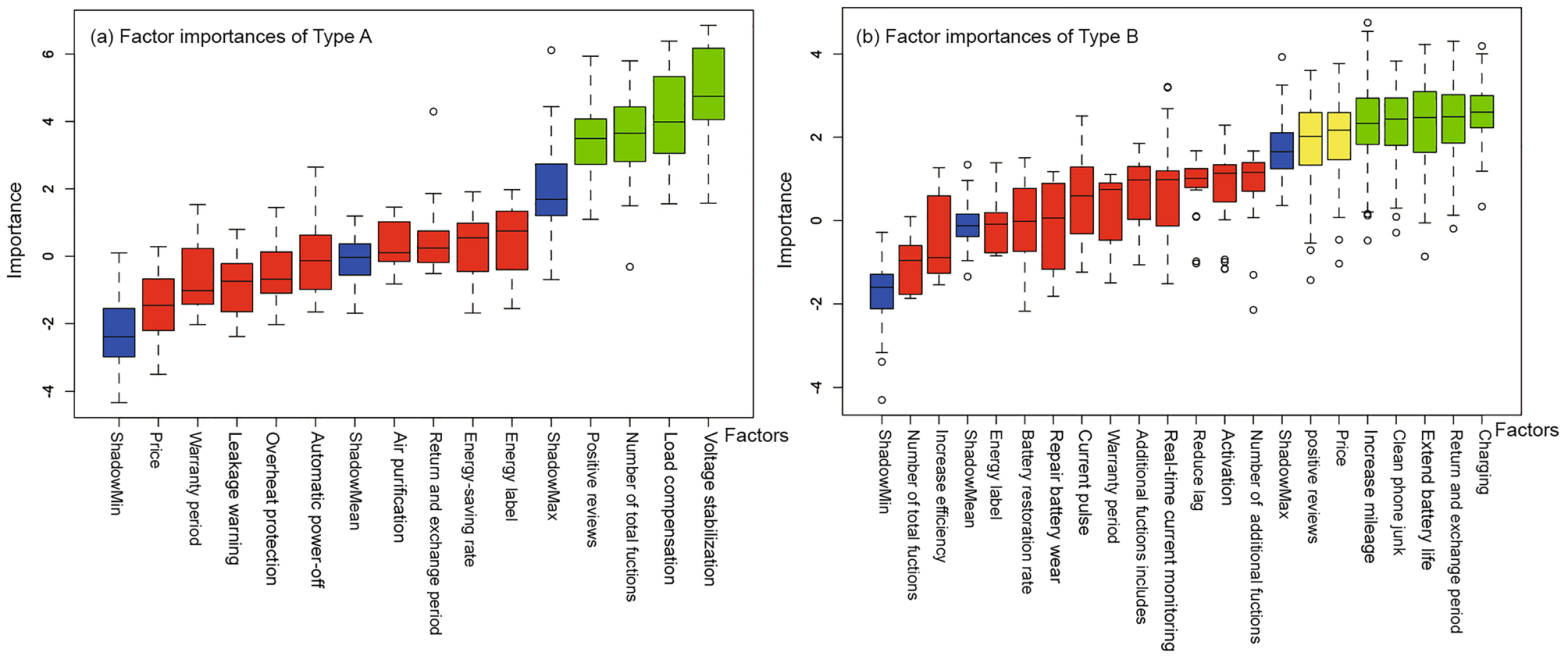
Identification of factor importance.

The study utilizes an RF-Boruta regression model to identify key factors influencing the sales volume of fake energy-saving products. This model considers sales volume as the dependent variable, and the independent variables include price, energy labels, energy efficiency, number of positive reviews, and main product functions. The product function factors are transformed into dummy variables, and those representing a small, non-representative portion are omitted. Statistical descriptions for each variable across the two product categories are presented in Table 2. The outcomes of the important factor identification are shown in Fig. 6, where red indicates no significant importance, green signifies significant importance, and yellow represents uncertainty.

**Table 2:** Statistical descriptions of independent and dependent variables for type A and type B products.

	Type A				Type B			
	Mean	Max	Min	S.D.	Mean	Max	Min	S.D.
Candidate variables								
Price	58.65	218	9.9	43.46	33.52	128.5	1.01	26.23
Positive reviews	192.72	4000	0	449.64	216.54	5671	0	609.21
Return and exchange period	62.54	365	7	64.47	22.41	180	7	41.81
Warranty period	2.86	10	1	2.16	1.36	10	1	1.65
Number of total functions	1.80	6	0	1.16	4.02	6	1	1.44
Energy-saving rate/Battery restoration rate	0.10	0.45	0	0.17	0.48	6	0	0.95
Dependent variable								
Sales volume	1172.98	50,000	0	4889.53	1800.18	100,000	0	10,420.46

The results reveal:

- In both product categories, the significance of price and reviews on sales volume is affirmed, supporting Hypothesis 1. This suggests that the purchasing decisions of hidden energy savers may be influenced by economic investment and product online reviews. Simultaneously, the findings rejected the importance of energy efficiency labels and the energy-saving rate/battery repair rate in both categories, supporting Hypothesis 2. This implies that despite their energy-saving goals, hidden energy savers may not pay close attention to the energy efficiency parameters of products.
- A notable discovery is the confirmed importance of product features: The most influential factors on Type A sales are the presence of Voltage stabilization and Load compensation features, along with the total number of features. This aligns with our analysis in Section “Analysis of functional requirements”, ‘Analysis of Functional Requirements’, suggesting that hidden energy savers require functions that elucidate the reasons for energy saving and prefer a greater variety of features. Moreover, considering the insignificant importance of energy-saving parameters, this finding suggests that descriptions of energy savings in textual form, rather than specific parameters, are more appealing to hidden energy savers.
- For Type B products, the significance of the charging function is reemphasized. Additionally, the return and exchange period is found to be important, indicating that online return and exchange policies might encourage hidden energy savers to experiment with purchasing energy-saving products. Furthermore, anxiety about the lifespan of battery products is a significant factor in purchasing Type B energy-saving products.

#### Characteristics of hidden energy-saving demand

Moreover, based on the previous discussion, the characteristics of hidden energy savers when purchasing energy efficiency-enhancing products can be summarized in Table 3:

**Table 3:** Characteristics of hidden energy-saving demand.

Category	Demand characteristics	Description
Price preference	Highly cost-effective	Preference for lower-priced products or those priced around the media in their market categories
Purchase threshold	Ease of acquisition and use	Significant preference for products that are easy to acquire, install, and use. Higher perceived complexity leads to lower purchase intent. Flexible return and exchange policies online can motivate the purchase of energy-saving products
Energy efficiency labels	Simplified information	Favoring text-based energy saving descriptions over specific technical parameters
Feature preference	Functional innovations	Marked interest in additional functionalities, especially those enhancing usage environment adaptability or offering unique improvements to existing features
Other characteristics	Clarity in efficiency principles	Preference for clear and concise explanations of the principles behind improvements in energy efficiency

### Wasted energy-saving efforts

Wasted Energy-Saving Efforts refer to the futile attempts at conserving energy made by individuals who purchase and use counterfeit energy-saving products. These hidden energy savers, believing they are reducing their energy consumption, are instead misled by fake products that not only fail to save energy but may actually consume more. As a result, their genuine efforts to conserve energy are rendered ineffective, leading to what is termed as Wasted Energy-Saving Efforts.

We categorize the wasted energy-saving efforts into two types, with specific descriptions, reasons for energy wastage, and identification methods as shown below:

- *Positive waste* These are individuals who trust the efficacy of counterfeit energy-saving products and are satisfied with their purchases.
  - *Reasons* (a) The actual energy-saving effects are not achieved, but the use of fake energy-saving products leads to greater energy wastage. (b) Consumers may believe they’ve conserved energy and, as a result, might reduce their current energy-saving measures, leading to more significant energy wastage.
  - *Identification method* Identify product reviews that demonstrate positive emotions and include at least one of the following aspects in their content: belief in achieving energy savings, willingness to repurchase, or readiness to recommend to others.
- *Negative waste* These are individuals who recognize the falseness of the product features and feel negative about their experiences.
  - *Reasons* This group may become more cautious about, or even reject, the use of energy-saving products, potentially affecting the usage rate of genuine energy-saving items.
  - *Identification method* Search for product reviews that express negative emotions and contain at least one of the following in their content: skepticism about the effectiveness and purpose of energy-saving products, a clear stance on not purchasing similar products in the future.

After analyzing 20,063 review contents, we derived the proportions of wasted energy-saving efforts, as shown in Table 4.

**Table 4:** The proportions of wasted energy-saving efforts.

Products		Quantity	Percentage of total reviews
Type A (*N* = 11,406)	Positive waste	5093	44.65%
	Negative waste	214	1.88%
	Total	5307	47%
Type B (*N* = 8657)	Positive waste	957	11.06%
	Negative waste	124	1.43%
	Total	1081	12%
Total		6388	31.84%

It indicates that 31.84% of the reviews point to Wasted Energy-Saving Efforts. It also implies that controlling and eliminating fake energy-saving products can result in substantial energy savings.

Moreover, Type A products (building energy-saving) result in more waste than Type B products (battery repair products), both in terms of quantity and percentage, especially from Positive Waste. A possible explanation is that energy savings in buildings are harder and take longer to detect. Typically, energy conservation in buildings can only be approximated by comparing electricity usage, which varies daily and seasonally, thus making accurate estimations challenging.

## Validation and implications

Based on the characteristics of hidden energy savers identified in Section “Results”, this study chose to use survey data from freight companies and individual transporters to analyze their preferences for adopting energy-saving measures in freight transportation, as a practical application and validation case for promoting energy-saving products, similar to the approach used for fraudulent energy-saving products. Here, individual transporters refer to individuals who provide transportation services independently.

Freight companies and individual transporters exhibit characteristics similar to hidden energy savers: price sensitivity, lack of sufficient scientific information, and the need for solutions that fit their regular daily activities. Of course, these groups are not identical to the hidden energy savers analyzed in this study, but this analysis serves as practical support for the research findings.

Our study examines freight companies and individual transporters, comparing two sets of demand data. One set comes from survey data, exploring their needs for freight products and measures that enhance energy efficiency, like adopting new energy vehicles, vans, container trucks, and eco-driving practices. The other set is based on demand estimates from our research findings, assessing the preferences of our subjects for these estimated demands through surveys.

For set 1, we conducted an online survey of 1450 freight companies and individual transporters in Zhejiang Province in May 2021. After discarding responses that were incomplete, nonsensical, or incorrect, we obtained 980 valid questionnaires. Detailed survey results and data are available in^12^.

For the hidden demand investigation (set 2), we reconnected with the same respondents using their contact details gathered from the initial survey. Notably, in the first survey, each respondent was asked to provide four of their needs when purchasing freight energy-saving products. In the second survey, we asked them to choose the four most attractive measures from a set of twenty options: ten response actions suggested in our study, and ten demands noted in the previous survey. It’s important to highlight that we did not use a rating scale for this inquiry. A total of 543 respondents submitted valid responses in the follow-up survey, and the findings are detailed in Tables 5 and 6.

**Table 5:** Proportions of adoption demands from surveys.

Specific demands	Category	Proportion^a^ (*N* = 981)
Increased number of qualified drivers	Purchase threshold (ease of use)	55.76%
Expanded charging infrastructure	Purchase threshold (ease of use)	50.36%
Extended battery range	Functions preference	44.24%
Reduced acquisition cost	Price preference	38.33%
Enhanced power output	Functions preference	31.80%
Improved maintenance services	Functions preference	24.97%
Increased Cargo capacity	Functions preference	22.94%
More convenient and reliable purchasing methods	Purchase threshold (ease of acquisition)	13.05%
Enhanced transportation safety	Functions preference	9.79%

^a^“Proportion” refers to the percentage of all respondents who selected this option.

**Table 6:** Proportions of adoption demands from revisited surveys.

Category	Predicted specific hidden demands	Proportion (*N* = 543)
Functions preference	Increased Cargo capacity^a^	52.49%
Purchase threshold	Unconditional return and exchange	45.30%
Purchase threshold	Product usage training	34.81%
Purchase threshold	On-site display and one-stop installation services	28.55%
Purchase threshold	Average purchase cost for similar products	20.63%
Functions preference	Access to extensive freight information	20.07%
Energy efficiency labels	Simple explanation of economical savings from promotional policies	10.87%
Energy efficiency labels	Simple explanation of energy efficiency and cost reduction	8.29%
Functions preference	Exquisite appearance	8.29%
Other characteristics	Manual demonstration of energy efficiency principles	6.63%
These demands were gathered through survey questionnaires and have also been included in the list of requirements for callbacks. This allows for a clearer comparison of the respondents’ support rates for needs identified through survey questionnaires versus those identified through predictions	Increased Cargo capacity^a^	52.49%
	Increased number of qualified drivers	34.62%
	Expanded charging infrastructure	32.23%
	Extended battery range	27.26%
	More convenient and reliable purchasing methods	19.34%
	Improved maintenance services	18.78%
	Enhanced power output	13.26%
	Reduced acquisition cost	10.31%
	Enhanced transportation safety	8.29%

^a^Duplicated options in the questionnaire.

The survey revealed that freight companies and individual transporters primarily focus on improving the existing functions and availability of products while showing limited interest in energy efficiency label information. Specifically, 55.76% and 50.36% of respondents selected “Increased Number of Qualified Drivers” and “Expanded Charging Infrastructure”, respectively, both of which relate to the product availability. Additionally, “Extended Battery Range”, “Reduced Acquisition Cost”, and three feature preferences (“Enhanced Power Output”, “Improved Maintenance Services”, and “Increased Cargo Capacity”) were also highly selected. In contrast, there was very little interest in energy efficiency label information.

This illustrates that while function preference and purchase threshold continue to be focal points of interest, our study can help to identify more specific and additional requirements, such as a returnable sales mode (45.30%), and product usage training (34.81%). Moreover, there is increased support for more clearer explanations of energy efficiency information.

Consequently, this research partially validates the conclusions drawn from the analysis of latent energy-saving needs, demonstrating the practical value of these findings. Specifically, it reveals that more hidden demand information can be gleaned than what is typically obtained through survey questionnaires. The practical and theoretical implications are as follows:

*Practical implications* The conclusions from this study on purchasing preferences can provide valuable recommendations for promoting various energy-saving products. These recommendations can be applied to areas such as energy-saving kitchen equipment in the catering industry, heat recycling in small manufacturing, and photovoltaic roofs in rural areas. Our research can assist promoters of energy conservation in these industries (including governments, managers, and practitioners) by providing a clearer understanding of potential issues and areas for optimization in product promotion. For example, adjusting marketing strategies and providing a wider range of features can attract hidden energy savers to shift from purchasing fraudulent energy-saving products to genuine ones. It also offers support in product design for manufacturers of various energy-saving products, allowing them to have a clearer direction of target consumer demands even in the absence of tangible products, thereby producing more appealing energy-saving products. Furthermore, the findings can contribute to the design of current incentive policies for energy-saving products.

Secondly, our study estimates the energy wastage stemming from counterfeit energy-saving products and proposes a new direction for energy conservation: reducing the waste of societal energy-saving efforts using fake energy-saving products. The analysis of the characteristics of these counterfeit products also aids online shopping platforms in more accurately identifying and combating such products.

The practical implications outlined above will ultimately contribute to the healthy development of energy-saving products, alternative energy products, and energy-conserving practices, thereby promoting energy conservation and the creation of a sustainable environment.

*Theoretical implications* The consumption behaviors of energy-saving consumers have been explored in multiple studies. This paper builds upon this foundation by supplementing it with research on the often-ignored purchasing behaviors of consumers of fraudulent energy-saving products. This enriches our understanding of the factors influencing the adoption of energy-saving products. For instance, unlike other energy-saving consumers, the group purchasing fraudulent energy-saving products tends to favor mid-range prices over low prices and often disregards energy labels and product attributes. This theoretically supplements our understanding of how energy labels and product attributes can drive energy-saving consumption and expands the categories of consumers that energy-saving product strategies should consider. Moreover, the findings contribute to research on the environmental sustainability impacts of online shopping by addressing the gap in assessing how intention-driven energy-saving behaviors in e-commerce can paradoxically increase energy consumption. Furthermore, this paper provides a new predictive framework to forecast the consumer behaviors of a currently hidden potential group.

## Discussion

Differing from other analyses of energy-saving products and behaviors, our study focuses on the purchasing behavior of a hidden group toward energy-saving products. Similar to other research, we find price and reviews significantly influence decisions^7^. However, a unique aspect is that both low and mid-range prices attract hidden energy savers, not just low prices. This might be due to the lower manufacturing costs of counterfeit energy-saving products. Additionally, hidden energy savers disregard energy labels and efficiency parameters, even when blatantly incorrect. This contrasts with most findings on general energy-saving consumers, who value these parameters and labels^22^, even if they sometimes misinterpret them^5^.

For the segmented group of hidden energy savers, product price and reviews influence their purchasing behavior differently compared to general energy-saving consumers. This suggests that studying energy-saving consumers as a single group may overlook the behavioral preferences of certain subgroups. This highlights the importance of categorizing energy-saving consumers into distinct groups, an aspect largely overlooked in most studies.

Another notable difference is that hidden energy savers pay attention to additional features, even if they contradict the core function, like energy-saving vs air purification features. Consumers’ pursuit of extra functionalities often stems from a desire to maximize benefits and differentiate from competitors^23^. Additionally, reliance on consumer knowledge and subjective interpretation^24^ can explain contradictions in core functionality. Another potential contributing factor is the sense of freshness from online products’ additional features, thereby stimulating their purchase intention^25^. Although these reasons have been proven in previous research, they have hardly exerted such a significant impact on the purchase intention of a specific consumer group, further highlighting the significance of analyzing the green purchasing behavior of hidden energy savers.

A noteworthy discovery is that energy-saving descriptions in textual form are more appealing to latent energy savers than precise energy-saving parameters. This suggests that market development in this area require more general and less technically demanding energy-saving descriptions. This aligns with research on displaying digital products: a combination of specific text and detailed images has a significant impact on consumers who prefer visual information processing over verbal processing, effectively evoking consumers’ mental imagery^26^. A novel idea proposed by the researchers of this paper is to use live streaming for the introduction and promotion of authentic energy-saving products. This perspective is supported by^26^, whose research on customer purchasing tendencies in live commerce suggests that the quality of streaming interaction and the consistency of comments significantly influence customer engagement. Live streaming, potentially more understandable and visually engaging than textual descriptions, allows for interactive elements that can help potential consumers fully grasp the product’s features.

While there are currently no specific energy-saving products tailored to latent energy savers, the behavior analysis of this group can be applied to the promotion of energy-saving products in other domains, as demonstrated by the case studies. This approach offers a new perspective on energy efficiency promotion: enticing the public to engage in energy-saving behaviors, rather than solely enhancing the appeal of energy efficiency from a rational, scientific standpoint. This is crucial as the irrational, gullible, and less informed public should also be considered an integral part of energy conservation and emission reduction efforts.

## Conclusions

This paper uncovers the hidden energy-saving behavior traits and needs of individuals who have purchased counterfeit energy-saving products. Despite their economic investment and efforts to save energy, these consumers do not achieve any energy savings due to the use of inauthentic products. This not only wastes their environmental consciousness but may also lead to greater energy wastage. The two research hypotheses are validated, and the case studies demonstrate the effectiveness and practical significance of this research. The primary conclusions of the study are:

- The specific characteristics of hidden energy savers include a tendency to prefer the lowest or mid-range prices, significant influence from product reviews, a preference for textual descriptions over specific energy-saving parameters, and an affinity for additional features.
- Regarding the purchase of energy-saving products, features like Voltage stabilization and Load compensation, as well as the total number of functions, have the most significant impact. For battery repair products, the focus is more on the charging function and the return and exchange policies.
- The two research hypotheses are validated, and the case studies demonstrate the effectiveness and practical significance of this research.

In summary, this study addresses the existing gap in research regarding the unrealized energy efficiency improvement purchasing behavior of latent energy savers. Our findings can be applied by government policy makers, energy product manufacturers, and sellers to meet the hidden demands of energy-conscious consumers. The research conclusions also contribute to the analysis of the impact of online shopping patterns on daily sustainability. All these implications collectively aid in advancing the achievement of carbon neutrality goals and promoting energy conservation, emissions reduction, and sustainable development in everyday life.

While this study provides valuable insights, it also has certain limitations. First, due to the protection of personal privacy, we could not access specific individual characteristics and purchase data of each buyer. Our analysis was thus limited to product-based evaluations, preventing a precise characterization of latent energy savers’ traits. Secondly, we performed an initial removal of fake reviews on online platforms, but those written by AI or individually crafted by real people may not have been completely identified. Additionally, our analysis assumes that all hidden energy savers cannot discern false reviews, which may introduce some inaccuracies. Moreover, the diversity in the functionalities of fraudulent energy-saving products and the uncertain likelihood of their actual usage by buyers make it difficult to estimate the exact energy waste caused by these products. Furthermore, a potential trend is that these fraudulent products might attempt to replicate some features of genuine products to confuse energy-saving product buyers. Although addressing this situation holds significant practical value, it does not align with the objectives of this study and is therefore less emphasized in our research.

### Supplementary Information

Supplementary Table 1.

Supplementary Table 2.

Supplementary Table 3.

Supplementary Table 4.

Supplementary Table 5.

Supplementary Information 1.

Supplementary Information 2.

Supplementary Information 3.

Supplementary Information 4.

Supplementary Information 5.

## Data Availability

The datasets used and analyzed during the current study available from the corresponding author on reasonable request.

## References

[1] Corbos R-A, Bunea O-I, Jiroveanu D-C (2023). The effects of the energy crisis on the energy-saving behavior of young people. Energy Strategy Rev..

[2] United Nations. The Paris Agreement|UNFCCC. https://unfccc.int/process-and-meetings/the-paris-agreement (2015).

[3] Liobikienė G, Butkus M (2017). The European Union possibilities to achieve targets of Europe 2020 and Paris agreement climate policy. Renew. Energy.

[4] XNA (Xinhua News Agency). *Central Government of the People’s Republic of China: ‘The Central Committee of the Communist Party of China, the State Council about the Complete and Accurate to Fully Implement the New Concept of Development to Do a Good Job of Carbon of Peak Carbon Neutral Opinion’*.

[5] Waechter S, Sütterlin B, Siegrist M (2015). The misleading effect of energy efficiency information on perceived energy friendliness of electric goods. J. Clean. Prod..

[6] Ma Y, Liu C (2023). Emotional or rational choice: The influence of individual personality on energy-saving behavior. Energy Econ..

[7] Ma G (2022). Customer behavior in purchasing energy-saving products: Big data analytics from online reviews of e-commerce. Energy Policy.

[8] De Temmerman J, Heeremans E, Slabbinck H, Vermeir I (2021). The impact of the nutri-score nutrition label on perceived healthiness and purchase intentions. Appetite.

[9] Matthies E, Merten MJ (2022). High-income Households—Damned to consume or free to engage in high-impact energy-saving behaviours?. J. Environ. Psychol..

[10] Wang Q, Li L, Li R (2023). Uncovering the impact of income inequality and population aging on carbon emission efficiency: An empirical analysis of 139 countries. Sci. Total Environ..

[11] Wu C, Xiao L, Hu Z, Zhou Y (2022). Modeling the low-carbon behaviors’ development paths of freight enterprises based on a survey in Zhejiang, China. Sustain. Cities Soc..

[12] Wu C, Li P, Zhou H, Zhou Y (2023). The changing adoption behaviors on electric trucks over time during the intention-purchase stage: Insights from freight enterprises’ states and perception features. J. Clean. Prod..

[13] Guo J, Wang X, Wu Y (2020). Positive emotion bias: Role of emotional content from online customer reviews in purchase decisions. J. Retail. Consum. Serv..

[14] Yuhsiang L, Lichung J (2024). The impact of consumer heterogeneity in the product life cycle on the diffusion patterns of user reviews and sales. J. Retail. Consum. Serv..

[15] Costa Filho M, Nogueira Rafael D, Salmonson Guimarães Barros L, Mesquita E (2023). Mind the fake reviews! Protecting consumers from deception through persuasion knowledge acquisition. J. Bus. Res..

[16] Zhang Y, Tao W (2020). Will energy efficiency affect appliance price? An empirical analysis of refrigerators in China based on hedonic price model. Energy Policy.

[17] Duan H, He B, Song J, Li W, Liu Z (2023). Preference of consumers for higher-grade energy-saving appliances in hierarchical Chinese cities. J. Environ. Manag..

[18] Aizawa A (2003). An information-theoretic perspective of TF–IDF measures. Inf. Process. Manag..

[19] Ali M, Mohamed Y (2017). A method for clustering unlabeled BIM objects using entropy and TF-IDF with RDF encoding. Adv. Eng. Inform..

[20] Breiman L (2001). Random forests. Mach. Learn..

[21] Lundheim SH, Vesely S, Nayum A, Klöckner CA (2021). From vague interest to strong intentions to install solar panels on private homes in the North: An analysis of psychological drivers. Renew. Energy.

[22] Zhang Y, Song B (2023). Does energy-efficiency label affect appliance price? Empirical analysis of the new national standard air conditioners in China. Energy.

[23] Chen P, Hu H (2010). How determinant attributes of service quality influence customer-perceived value: An empirical investigation of the Australian coffee outlet industry. Int. J. Contemp. Hosp. Manag..

[24] Viana MM, Silva VLS, Deliza R, Trindade MA (2016). The use of an online completion test to reveal important attributes in consumer choice: An empirical study on frozen burgers. Food Qual. Prefer..

[25] Frasquet M, Ieva M, Mollá-Descals A (2024). Customer inspiration in retailing: The role of perceived novelty and customer loyalty across offline and online channels. J. Retail. Consum. Serv..

[26] Cheng P, Zhang C (2023). Show me insides: Investigating the influences of product exploded view on consumers’ mental imagery, comprehension, attitude, and purchase intention. J. Retail. Consum. Serv..

