# Supplementary material for: Exploring online consumer behavior on fraudulent energy-saving products
Source: Sci Rep. 2024 Jun 21;14:14304. doi: 10.1038/s41598-024-65210-1 (PMC11192901; doi:10.1038/s41598-024-65210-1)
Supplement: Supplementary file 1 — Supplementary Table 1. [file 41598_2024_65210_MOESM1_ESM.pdf]

Product name	Features	For whom
Battery fixer	Extend battery life；<br>fix phone jams；<br>Check the current voltage	Cell PHONE
Battery repair artifact	Check the current voltage；<br>repair and maintain the battery；<br>to improve the life	Various digital products
Battery fixer	Repair；<br>prolongs life；<br>cleans up mobile phone rubbish；<br>reduces radiation	Various mobile phones；<br>power bank；<br>laptop
Battery repair artifact	Pulse repair battery capacity；<br>maintenance；<br>life prolonging	Various digital products
Battery repair artifact	Pulse repair battery；<br>maintenance；<br>life extension	Battery cart
Battery repair artifact	Prolong life；<br>battery repair；<br>Fix Caton；<br>Clean up cell phone trash	Multiple devices compatible；<br>mobile tablet
Battery repair artifact	Improve efficiency；<br>charging；<br>maintenance；<br>Fix Catton Clean up litter	Multiple devices compatible；<br>mobile tablet
Battery repair artifact	Prolonging life；<br>battery repair；<br>repair Caton；<br>Clean up cell phone litter；<br>activation	Multiple devices compatible；<br>mobile tablet
Battery repair artifact	Prolong life；<br>battery repair；<br>Fix Caton；<br>Clean up cell phone trash	Smart phone
Battery repair artifact	Prolong life；<br>battery repair；<br>Fix Caton；<br>Clean up cell phone trash	Smart phone
Battery repair artifact	Prolong life；<br>battery repair；<br>maintenance；<br>Repair Caton；<br>Clean up your phone trash	Huawei；<br>apple；<br>Xiaomi ；<br>oppo；<br>vivo mobile phone
Battery fixer	Prolonging life；<br>battery repair；<br>maintenance	All mobile phones
Battery repair artifact	Prolong life；<br>battery repair；<br>Fix Caton；<br>Clean up cell phone trash	Smart phone
Battery repair artifact	Charging fix；<br>fix Caton；<br>Clean up cell phone trash	Multiple devices compatible；<br>mobile tablet
Battery fixer	Repair；<br>charging；<br>maintenance；<br>repair Caton；<br>Clean up cell phone junk	Multiple devices compatible；<br>mobile tablet
Battery fixer	Pulse repair；<br>charging；<br>maintenance；<br>Repair Caton；<br>Clean up cell phone junk	Huawei；<br>apple；<br>Xiaomi ；<br>oppo；<br>vivo mobile phone
Battery fixer	Charging repair；<br>repair Caton；<br>aging；<br>machine hot；<br>Clean up mobile phone garbage	Smart phone
Electric vehicle intelligent pulse regulator	Battery；<br>repair；<br>maintenance；<br>activation；<br>prolong life；<br>battery deep cleaning；<br>Detect the current	Electric car
New super Wei battery repair device	battery;<br>repair;<br>maintenance;<br>mileage extension;<br>life extension;<br>battery deep cleaning;<br>detects the current	Electric car
Battery repairer	Pulse repair；<br>charging；<br>maintenance；<br>increase mileage；<br>life extension battery 3-5 years	Fully autonomous electric vehicle
Battery repairer	Pulse repair；<br>charging；<br>maintenance；<br>activation；<br>increase mileage；<br>life extension battery 3-5 years	Fully autonomous electric vehicle
Battery fixer	Charging repair；<br>repair Caton；<br>aging；<br>machine hot；<br>Clean up mobile phone garbage	Smart phone

Battery repairer	Pulse repair；<br>charging；<br>maintenance；<br>activation；<br>increase mileage；<br>prolong life	Fully autonomous electric vehicle
conditioner intelligent inverter repair dev	Electrical repair；<br>energy saving；<br>voltage regulation；<br>circuit optimization	air conditioner.
conditioner intelligent inverter repair dev	Electrical repair；<br>energy saving；<br>voltage regulation；<br>circuit optimization	air conditioner.
Battery repairer	Battery；<br>repair；<br>maintenance；<br>activation；<br>prolong life；<br>battery deep cleaning；<br>Detect the current	Electric car
Battery fixer	Prolonging life；<br>battery repair；<br>charging	Multiple devices compatible；<br>mobile tablet
conditioner intelligent inverter repair dev	Electrical repair；<br>energy saving；<br>voltage regulation；<br>circuit optimization	air conditioner.
conditioner intelligent inverter repair dev	Electrical repair；<br>energy saving；<br>voltage regulation；<br>circuit optimization	air conditioner.
conditioner intelligent inverter repair dev	Electrical repair；<br>energy saving；<br>voltage regulation；<br>circuit optimization	air conditioner.
conditioner intelligent inverter repair dev	Electrical repair；<br>energy saving；<br>voltage regulation；<br>circuit optimization	air conditioner.
Battery fixer	Prolonging life；<br>battery repair；<br>charging	Multiple devices compatible；<br>mobile tablet
Battery repairer	Pulse repair；<br>charging；<br>maintenance；<br>activation；<br>increase mileage；<br>prolong life	Fully autonomous electric vehicle
1st generation battery repairer	Life extension；<br>repair	Multiple devices compatible；<br>mobile tablet
2nd generation Battery repairer	Life extension；<br>repair	Multiple devices compatible；<br>mobile tablet
3rd generation Battery Repairer	Life extension；<br>repair	Multiple devices compatible；<br>mobile tablet
Classic Black	Battery；<br>repair；<br>maintenance；<br>activation；<br>increase mileage	Various digital products
Moonlight White	Battery；<br>repair；<br>maintenance；<br>activation；<br>increase mileage	Various digital products
Lemon Yellow	Repair；<br>prolongs life	Various digital products
Lemon yellow	Pulse repair；<br>battery；<br>maintenance；<br>life extension	Smartphone universal
Lemon yellow	Repair；<br>prolongs life	Various digital products
Battery repairer	Pulse repair；<br>charging；<br>maintenance；<br>activation；<br>increase mileage；<br>prolong life battery	Fully autonomous electric vehicle
Battery fixer	Pulse repair battery capacity；<br>maintenance；<br>Activate；<br>life extension battery	Various digital products
Battery fixer	Pulse repair；<br>life extension	Multiple devices compatible；<br>mobile tablet
Battery fixer	Pulse repair；<br>life extension；<br>Charging	All mobile phones
Battery fixer	Pulse repair；<br>life extension；<br>Charging	Multiple devices compatible；<br>mobile tablet
New super Wei battery repair device	Battery；<br>repair；<br>maintenance；<br>range extension；<br>life extension；<br>activation	Electric car

New super Wei battery repair device	Battery；<br>repair；<br>maintenance；<br>range extension；<br>life extension；<br>activation	Electric car
New super Wei battery repair device	Battery；<br>repair；<br>maintenance；<br>range extension；<br>life extension；<br>activation	Electric car
Battery fixer	Pulse repair battery capacity；<br>maintenance；<br>life prolonging	Smart phone
Battery fixer	Pulse repair；<br>maintenance；<br>life extension	Multiple devices compatible；<br>mobile tablet
Battery fixer	Low temperature pulse repair；<br>maintenance；<br>life extension	Multiple devices compatible；<br>mobile tablet
Battery repairer	Pulse repair；<br>charging；<br>maintenance；<br>activation；<br>increase mileage；<br>life extension battery 3-5 years	Fully autonomous electric vehicle
Battery repairer	Pulse repair；<br>charging；<br>maintenance；<br>activation；<br>increase mileage；<br>life extension battery 3-5 years	Fully autonomous electric vehicle
Battery fixer	Optimize；<br>charging；<br>superconducting pulse repair；<br>maintenance	Huawei；<br>apple；<br>Xiaomi ；<br>oppo；<br>vivo mobile phone
Battery fixer	Optimize；<br>charging；<br>superconducting pulse repair；<br>maintenance	Huawei；<br>apple；<br>Xiaomi ；<br>oppo；<br>vivo mobile phone
Battery fixer	Charging；<br>superconducting pulse repair；<br>maintenance	Multiple devices compatible；<br>mobile tablet
Battery repairer	Pulse repair；<br>increase mileage；<br>improve life；<br>Monitor current in real time	Electric car
Battery repairer	Pulse repair；<br>increase mileage；<br>improve life；<br>Monitor current in real time	Electric car
Battery fixer	Life extension；<br>battery repair	Multiple devices compatible；<br>mobile tablet
Battery fixer	Life extension；<br>battery repair	Various digital products
Battery fixer	Battery；<br>Pulse repair；<br>Life extension	All mobile phones
Battery fixer	Battery；<br>Pulse repair；<br>Life extension	Multiple devices compatible；<br>mobile tablet
Electric car repair tool	Silent charging；<br>battery；<br>repair；<br>maintenance；<br>activation	Electric car
Repair Adapter	Charging；<br>repair；<br>repair Caton；<br>lower radiation	Smart phone
Battery fixer	Battery；<br>repair；<br>maintenance；<br>range extension；<br>life extension；<br>activation	Smart phone
Cell phone battery adapter	Battery；<br>repair；<br>maintenance；<br>range extension；<br>life extension；<br>activation	Multiple devices compatible；<br>mobile tablet
Cell phone battery adapter	Charging repair；<br>prolongs life	Multiple devices compatible；<br>mobile tablet
Cell phone battery adapter	Charging repair；<br>prolongs life	Multiple devices compatible；<br>mobile tablet
Cell phone battery adapter	Charging repair；<br>prolongs life	Multiple devices compatible；<br>mobile tablet
Battery fixer	Pulse repair；<br>maintenance；<br>battery；<br>life prolonging	Multiple devices compatible；<br>mobile tablet
conditioner intelligent inverter repair dev	Electrical repair；<br>energy saving；<br>voltage regulation；<br>circuit optimization	air conditioner.

conditioner intelligent inverter repair dev	Electrical repair；<br>energy saving；<br>voltage regulation；<br>circuit optimization	1.5bhp air conditioner
conditioner intelligent inverter repair dev	Electrical repair；<br>energy saving；<br>voltage regulation；<br>circuit optimization	Air-conditioned refrigerator
conditioner intelligent inverter repair dev	Electrical repair；<br>energy saving；<br>voltage regulation；<br>circuit optimization	Air-conditioned refrigerator
conditioner intelligent inverter repair dev	Electrical repair；<br>energy saving；<br>voltage regulation；<br>circuit optimization	Air-conditioned refrigerator
Electric car repair tool	Repair；<br>activate；<br>charging	Electric car
Electric car repair tool	Auto repair；<br>silent charging；<br>Maintenance；<br>activate battery；<br>prolonging life	Electric car
Battery fixer	Battery；<br>repair；<br>maintenance；<br>range extension；<br>life extension；<br>activation	Multiple devices compatible；<br>mobile tablet
Battery fixer	Battery；<br>repair；<br>maintenance；<br>life extension	Multiple devices compatible；<br>mobile tablet
Battery fixer	Battery；<br>repair；<br>maintenance；<br>life extension	Multiple devices compatible；<br>mobile tablet
Battery fixer	Battery；<br>repair；<br>maintenance；<br>life extension	Multiple devices compatible；<br>mobile tablet
Battery fixer	Charging repair；<br>life extension；<br>maintenance	All mobile phones
Battery fixer	Charging repair；<br>life extension；<br>maintenance	All mobile phones
Battery fixer	Charging repair；<br>life extension；<br>maintenance	All mobile phones
Battery fixer	Charging repair；<br>life extension；<br>maintenance	All mobile phones
Battery fixer	Charging repair；<br>life extension；<br>maintenance	All mobile phones
Battery fixer	Charging repair；<br>life extension；<br>maintenance	All mobile phones
Battery fixer	Charging repair；<br>life extension；<br>maintenance	All mobile phones
Battery fixer	Pulse charging repair；<br>life extension；<br>maintenance；<br>battery	Multiple devices compatible；<br>mobile tablet
Battery fixer	Pulse charging repair；<br>life extension；<br>maintenance；<br>battery	Multiple devices compatible；<br>mobile tablet
Battery fixer	Pulse charging repair；<br>life extension；<br>maintenance；<br>battery	Multiple devices compatible；<br>mobile tablet
Battery fixer	Pulse charging repair；<br>life extension；<br>maintenance；<br>battery	Multiple devices compatible；<br>mobile tablet
Battery fixer	Repair battery；<br>to extend life	none
Battery fixer	Repair battery；<br>to extend life	none
Battery repair artifact	Activate；<br>repair battery；<br>to extend life	Multiple devices compatible；<br>mobile tablet
Battery repair artifact	Activate；<br>repair battery；<br>to extend life	Multiple devices compatible；<br>mobile tablet
Battery fixer	Repair battery；<br>to extend life	Multiple devices compatible；<br>mobile tablet
Battery fixer	Repair battery；<br>to extend life	Multiple devices compatible；<br>mobile tablet
Battery fixer	Repair battery；<br>to extend life	Multiple devices compatible；<br>mobile tablet
Automatic battery repair artifact	Repair battery；<br>extends battery life；<br>multiple protection；<br>maintenance	Multiple devices compatible；<br>mobile tablet

Automatic battery repair artifact	Repair battery；<br>extends battery life；<br>multiple protection；<br>maintenance	Multiple devices compatible；<br>mobile tablet
Battery repairer	Battery；<br>repair；<br>maintenance；<br>range extension；<br>life extension；<br>activation	Electric car
Battery repairer	Battery；<br>repair；<br>maintenance；<br>range extension；<br>life extension；<br>activation	Electric car
Battery repairer	Battery；<br>repair；<br>maintenance；<br>range extension；<br>life extension；<br>activation	Electric car
Battery repairer	Charging；<br>repair；<br>maintenance；<br>activation	Electric scooter
Battery fixer	Charge；<br>repair；<br>maintenance	All mobile phones
Battery fixer	Charging；<br>repair；<br>maintenance；<br>activation	All mobile phones
Battery fixer	Charging；<br>repair；<br>maintenance；<br>activation	All mobile phones
Phone adapter	Charge；<br>repair；<br>maintenance	All mobile phones
Phone adapter	Charge；<br>repair；<br>maintenance	All mobile phones
Phone adapter	Charge；<br>repair；<br>maintenance	All mobile phones
Cell phone battery fixer	Charge；<br>repair；<br>maintenance	All mobile phones
Cell phone battery fixer	Charging；<br>repair；<br>maintenance；<br>activation	All mobile phones
Cell phone battery fixer	Charging；<br>repair；<br>maintenance；<br>activation	All mobile phones
Battery fixer	Charge；<br>repair；<br>maintenance	All mobile phones
Battery fixer	Charge；<br>repair；<br>maintenance	All mobile phones
Battery fixer	Charging；<br>repair；<br>maintenance；<br>activation	Android phone
Battery fixer	Charging；<br>repair；<br>maintenance；<br>activation	iPhone
Battery fixer	Battery；<br>pulse repair；<br>maintenance；<br>life prolonging	An iPhone or tablet is fine
Battery fixer	Battery；<br>pulse repair；<br>maintenance；<br>life prolonging	An iPhone or tablet is fine
Battery fixer	Battery；<br>pulse repair；<br>maintenance；<br>life prolonging	An iPhone or tablet is fine
Battery fixer	Pulse repair；<br>charging；<br>maintenance；<br>life extension	Huawei；<br>apple；<br>Xiaomi ；<br>oppo；<br>vivo mobile phone
Battery fixer	Battery；<br>pulse repair；<br>maintenance；<br>life prolonging	Multiple devices compatible；<br>mobile tablet
Battery fixer	Battery；<br>pulse repair；<br>maintenance；<br>life prolonging	Multiple devices compatible；<br>mobile tablet
Battery fixer	Battery；<br>pulse repair；<br>maintenance；<br>life prolonging	Multiple devices compatible；<br>mobile tablet

Battery fixer	Battery；<br>pulse repair；<br>maintenance；<br>life prolonging	Multiple devices compatible；<br>mobile tablet
Battery fixer	Battery；<br>pulse repair；<br>maintenance；<br>life prolonging	Multiple devices compatible；<br>mobile tablet
Tram adapter	Battery；<br>repair；<br>maintenance；<br>activation；<br>increase mileage	Tram
Tram adapter	Battery；<br>repair；<br>maintenance；<br>activation；<br>increase mileage	Tram
Phone adapter	Battery；<br>pulse repair；<br>maintenance；<br>life prolonging	Huawei；<br>apple；<br>Xiaomi ；<br>oppo；<br>vivo mobile phone
Phone adapter	Battery；<br>pulse repair；<br>maintenance；<br>life prolonging	Huawei；<br>apple；<br>Xiaomi ；<br>oppo；<br>vivo mobile phone
Air conditioning adapter	Electrical repair；<br>energy saving；<br>voltage regulation；<br>circuit optimization	air conditioner.
Air conditioning adapter	Electrical repair；<br>energy saving；<br>voltage regulation；<br>circuit optimization	air conditioner.
Air conditioning adapter	Electrical repair；<br>energy saving；<br>voltage regulation；<br>circuit optimization	air conditioner.
Battery fixer	Repair；<br>prolongs life	Huawei；<br>xiaomi ；<br>vivo；<br>oppo honor；<br>Meizu；<br>OnePlus
Battery fixer	Battery；<br>pulse repair；<br>maintenance；<br>life prolonging	Multiple devices compatible；<br>mobile tablet
Battery fixer	Pulse repair；<br>battery；<br>maintenance；<br>life extension	Multiple devices compatible；<br>mobile tablet
Battery fixer	Pulse repair；<br>battery；<br>maintenance；<br>life extension	Multiple devices compatible；<br>mobile tablet
Battery fixer	Pulse repair；<br>battery；<br>maintenance；<br>life extension	Multiple devices compatible；<br>mobile tablet
Battery fixer	Pulse repair；<br>battery；<br>maintenance；<br>life extension	Multiple devices compatible；<br>mobile tablet
Battery fixer	Pulse repair；<br>battery；<br>maintenance；<br>life extension	Multiple devices compatible；<br>mobile tablet
Electric vehicle intelligent pulse regulator	Repair battery；<br>prolong life；<br>maintenance	Electric scooter
Electric vehicle intelligent pulse regulator	Repair battery；<br>prolong life；<br>maintenance	Electric scooter
Electric vehicle intelligent pulse regulator	Repair battery；<br>prolong life；<br>maintenance	Electric scooter
Battery fixer	Charging；<br>pulse repair；<br>maintenance	Multiple devices compatible；<br>mobile tablet
Small cell phone battery butler	Charging；<br>pulse repair；<br>prolonging life	Multiple devices compatible；<br>mobile tablet
Small cell phone battery butler	Maintenance；<br>repair；<br>prolongs life	Multiple devices compatible；<br>mobile tablet
Small cell phone battery butler	Maintenance；<br>repair；<br>prolongs life	Multiple devices compatible；<br>mobile tablet
Phone adapter	Charging；<br>pulse repair；<br>maintenance；<br>life extension	Multiple devices compatible；<br>mobile tablet
Phone adapter	Repair battery；<br>prolong life；<br>maintenance	Phones, watches, earphones

Phone adapter	Charging；<br>pulse repair；<br>maintenance	Multiple devices compatible；<br>mobile tablet
Phone adapter	Charging；<br>pulse repair；<br>maintenance；<br>life extension	Multiple devices compatible；<br>mobile tablet
Air conditioning adapter	Repair；<br>power saving；<br>voltage regulator；<br>optimize the circuit	air conditioner.
Multi-functional fixer	Charging；<br>pulse repair；<br>maintenance；<br>life extension	Compatible
Apple Cord + Fixer	Charging；<br>pulse repair；<br>maintenance；<br>life extension	Compatible
Small cell phone battery butler	Charging；<br>pulse repair；<br>maintenance	Multiple devices compatible；<br>mobile tablet
Smartphone fixer	Repair battery；<br>prolong life；<br>maintenance	Multiple devices compatible；<br>mobile tablet
Smartphone fixer	Repair battery；<br>prolong life；<br>maintenance	Multiple devices compatible；<br>mobile tablet
Smartphone fixer	Repair battery；<br>prolong life；<br>maintenance	Multiple devices compatible；<br>mobile tablet
Air conditioning adapter	Electrical repair；<br>power saving；<br>voltage regulator	Appliances；<br>Air conditioning
Air conditioning adapter	Electrical repair；<br>power saving；<br>voltage regulator	Appliances；Air conditioning
High quality phone restorer	Charging；<br>pulse repair；<br>maintenance	Multiple devices compatible；<br>mobile tablet
High quality phone restorer	Charging；<br>pulse repair；<br>maintenance	Multiple devices compatible；<br>mobile tablet
High quality phone restorer	Charging；<br>pulse repair；<br>maintenance	Multiple devices compatible；<br>mobile tablet
High quality phone restorer	Charging；<br>pulse repair；<br>maintenance；<br>life extension	Multiple devices compatible；<br>mobile tablet
High quality phone restorer	Charging；<br>pulse repair；<br>maintenance；<br>life extension	Multiple devices compatible；<br>mobile tablet
High quality phone restorer	Charging；<br>pulse repair；<br>maintenance；<br>life extension	Multiple devices compatible；<br>mobile tablet
Fully automatic phone restorer	Charging；<br>pulse repair；<br>maintenance	Multiple devices compatible；<br>mobile tablet
Battery repairer	Battery；<br>repair；<br>maintenance；<br>range extension；<br>life extension；<br>activation	Electric scooter
Battery repairer	Battery；<br>repair；<br>maintenance；<br>range extension；<br>life extension；<br>activation	Electric scooter
Battery repairer	Battery；<br>repair；<br>maintenance；<br>range extension；<br>life extension；<br>activation	Electric scooter
Battery fixer	Charging；<br>pulse repair；<br>maintenance	Multiple devices compatible；<br>mobile tablet
Phone adapter	Battery repair；<br>prolong life；<br>maintenance	Multiple devices compatible；<br>mobile tablet
Phone adapter	Battery repair；<br>prolong life；<br>maintenance	Multiple devices compatible；<br>mobile tablet
Phone adapter	Battery repair；<br>prolong life；<br>maintenance	Multiple devices compatible；<br>mobile tablet
Air conditioning adapter	Electrical repair；<br>power saving；<br>voltage regulator	Special for air conditioning
Battery fixer	Prolong life；<br>battery repair；<br>Fix Caton；<br>Clean up cell phone trash	All cell phones

Battery fixer	Extend battery life ；<br>fix phone jams ；<br>Check the current voltage	Cell PHONE
Air conditioning adapter	Electrical repair ；<br>power saving ；<br>voltage regulator	Special for air conditioning
Battery fixer	Prolong life ；<br>battery repair ；<br>Fix Caton ；<br>Clean up cell phone trash	All cell phones
Battery fixer	Prolonging life ；<br>battery repair ；<br>charging	All cell phones
Air conditioning adapter	Electrical repair ；<br>power saving ；<br>voltage regulator	Special for air conditioning
Battery fixer	Charging ；<br>repair ；<br>maintenance ；<br>life extension	All cell phones
Battery fixer	Charging ；<br>pulse repair ；<br>maintenance ；<br>life extension	Various digital products
Green intelligent energy-saving applianc	On-site compensation ；<br>overload and overpressure protection ；	Home commercial circuit
Green intelligent energy-saving applianc	Indicator ；<br>overload overpressure protection ；<br>local compensation	Home commercial circuit
Smart Appliance Saver	Screen control ；<br>overload overpressure protection ；<br>local compensation	Home commercial circuit
Concentrated energy Province	Overpressure protection ；<br>local compensation	Home commercial circuit
Power saver	Indicator light ；<br>overload overpressure protection ；<br>overheat protection	Home commercial circuit
Concentrated energy Province	none	Home commercial circuit
White smart appliance saver	Overload and overpressure protection ；<br>on-site compensation ；	Home commercial circuit
Smart Butler	none	Home commercial circuit
Smart energy saving Butler	Screen control ；<br>overload overpressure protection ；<br>local compensation	Home commercial circuit
Power saver	Overload and overpressure protection ；<br>on-site compensation ；	Home commercial circuit
automatic intelligent energy saving appl	Voltage regulator ；<br>overload overpressure protection ；<br>local compensation ；<br>indicator light	Home commercial circuit
Smart Province	Voltage regulator ；<br>overload and overpressure protection ；<br>active local compensation	Home commercial circuit
Power saver	Indicator light ；<br>overload overpressure protection ；<br>overheat protection	Home commercial circuit
Concentrated energy Province	Screen control ；<br>overload overpressure protection ；<br>local compensation	Home commercial circuit
automatic intelligent energy saving appl	Stable voltage ；<br>overload overvoltage protection ；<br>local compensation	Home commercial circuit
Green intelligent energy-saving applianc	Screen control ；<br>overload overpressure protection ；<br>local compensation	Home commercial circuit
Power saver	Indicator light ；<br>overload overpressure protection ；<br>overheat protection	Home commercial circuit
Green intelligent energy-saving applianc	Screen control ；<br>overload overpressure protection ；<br>local compensation	Home commercial circuit
Smart Appliance Saver	Plug and play ；<br>voltage display	Home commercial circuit
Electrodoc	Screen control ；<br>overload overvoltage protection ；<br>local compensation ；<br>indicator light	Home commercial circuit
On-site compensation saver	Screen control ；<br>overload overpressure protection ；<br>local compensation	Home commercial circuit
Power Saver	Voltage regulation ；<br>life extension ；<br>screen control ；<br>overload overpressure protection ；<br>in-situ compensation ；<br>overheat protection	Home commercial circuit
Green intelligent energy-saving applianc	Screen control ；<br>overload overpressure protection ；<br>reactive power compensation	Home commercial circuit
Green intelligent energy-saving applianc	Screen control ；<br>overload overpressure protection ；<br>local compensation	Home commercial circuit
White smart appliance saver	none	Home commercial circuit
On-site compensation saver	Overheat protection Automatic power off	Home commercial circuit

Local compensation saver	Indicator ；<br>overload overpressure protection ；<br>in situ compensation ；<br>overheat protection	Home commercial circuit
Smart Appliance Saver	none	Home commercial circuit
Smart Appliance Saver	Plug and play ；<br>voltage display	Home commercial circuit
Power saver	Overload overpressure protection ；<br>local compensation ；<br>indicator light	Home commercial circuit
Power saver	none	Home commercial circuit
Smart Appliance Saver	Plug and play ；<br>simple operation	Home commercial circuit
Power saver	The one-button switch ；<br>has an indicator light	Home commercial circuit
Power saver	Overload and overpressure protection ；<br>active local compensation ；<br>indicator light	Home commercial circuit
Smart Appliance Saver	Voltage regulator ；<br>screen display control ；<br>overload overpressure protection ；<br>local compensation	Home commercial circuit
Green intelligent energy-saving appliance	none	Home commercial circuit
Green intelligent energy-saving appliance	Overload and overpressure protection ；<br>active local compensation	Home commercial circuit
Smart Appliance Saver	Indicator ；<br>overvoltage ；<br>overheat ；<br>short circuit ；<br>overcurrent protection	Home commercial circuit
Power saver	Indicator ；<br>screen control ；<br>overload and overpressure protection ；<br>local compensation	Home commercial circuit
Smart Appliance Saver	Plug and play ；<br>voltage display	Home commercial circuit
Concentrated energy Province	Plug and play ；<br>simple operation	Home commercial circuit
On-site compensation saver	Overheat protection Automatic power off	Home commercial circuit
Local compensation saver	Voltage regulator ；<br>overload overpressure protection ；<br>local compensation ；<br>indicator light	Home commercial circuit
Power saver	Overheat protection ；<br>indicator light	Home commercial circuit
Power saving expert	Screen control ；<br>overload overpressure protection ；<br>in situ compensation ；<br>independent fuse	Home commercial circuit
On-site compensation saver	Screen control ；<br>overload overpressure protection ；<br>local compensation ；<br>overheat protection automatic power off	Home commercial circuit
Power Saving King	Screen control ；<br>overload overpressure protection ；<br>in situ compensation ；<br>independent fuse	Home commercial circuit
Ultrasonic Power Saver	none	Home commercial circuit
White smart appliance saver	Screen control ；<br>overload overpressure protection ；<br>local compensation	Home commercial circuit
Power Saver	none	Home commercial circuit
Green intelligent energy-saving appliance	Overvoltage ；<br>overload ；<br>overheat ；<br>overcurrent ；<br>short circuit protection ；<br>indicator light	Home commercial circuit
Energy Saver	Plug and play ；<br>overvoltage protection	Home commercial circuit
Power saver	none	Home commercial circuit
Power saver	Indicator ；<br>screen display control ；<br>overload overpressure protection ；<br>on the spot compensation ；<br>independent safety tube	Home commercial circuit
Smart Appliance Saver	Screen control ；<br>overload overpressure protection ；<br>local compensation	Home commercial circuit
Green intelligent energy-saving appliance	none	Home commercial circuit
White smart appliance saver	Screen control ；<br>overload overpressure protection ；<br>local compensation	Home commercial circuit
Power saver	Screen control ；<br>overload overpressure protection ；<br>local compensation	Home commercial circuit
Green intelligent energy-saving appliance	none	Home commercial circuit
White smart appliance saver	Screen control ；<br>overload overpressure protection ；<br>local compensation	Home commercial circuit
Power Saving King	Screen control ；<br>overload overpressure protection ；<br>in situ compensation ；<br>independent fuse	Home commercial circuit
Smart Appliance Saver	Screen control ；<br>overload and overpressure protection ；<br>local compensation ；<br>independent safety tube	Home commercial circuit

Power Saving King	Screen control ；<br>overload and overpressure protection ；<br>local compensation ；<br>independent safety tube	Home commercial circuit
Red power saving expert	Screen control ；<br>overload overpressure protection ；<br>in situ compensation ；<br>independent fuse	Home commercial circuit
Smart Power Saver	Screen control ；<br>overload overpressure protection ；<br>reactive power compensation	Home commercial circuit
Power saver	Overload and overpressure protection ；<br>active local compensation	Home commercial circuit
Green intelligent energy-saving appliance	none	Home commercial circuit
Smart Appliance Saver	none	Home commercial circuit
Smart Power saver	Screen control ；<br>overload and overpressure protection ；<br>local compensation ；<br>independent safety tube	Home commercial circuit
White smart appliance saver	Screen control ；<br>overload overpressure protection ；<br>local compensation	Home commercial circuit
Local compensation saver	Voltage regulator ；<br>indicator ；<br>overload overpressure protection ；<br>local compensation	Home commercial circuit
Power Saving King	Voltage regulator ；<br>screen display control ；<br>overload overpressure protection ；<br>local compensation ；<br>independent fuse	Home commercial circuit
Power-saving devices	Screen control ；<br>overload overpressure protection ；<br>in situ compensation ；<br>independent fuse	Home commercial circuit
Red power saving expert	Screen control ；<br>overload and overpressure protection ；<br>local compensation ；<br>independent safety tube	Home commercial circuit
Red power saving expert	Screen control ；<br>overload overpressure protection ；<br>local compensation	Home commercial circuit
Power saving expert	Liquid crystal display ；<br>overload overpressure protection ；<br>local compensation ；<br>independent fuse	Home commercial circuit
Power Saving King	Screen control ；<br>overload overpressure protection ；<br>in situ compensation ；<br>independent fuse	Home commercial circuit
Power Saving King	LCD display	Home commercial circuit
Local compensation saver	Indicator light ；<br>local compensation	Home commercial circuit
Smart Appliance Saver	none	Home commercial circuit
Power saver	Indicator light ；<br>local compensation	Home commercial circuit
Smart Power saving Manager	Visual screen	Home commercial circuit
Power Saver	none	Home commercial circuit
Smart energy saving Butler	none	Home commercial circuit
Smart Appliance Saver	Screen control ；<br>overload and overpressure protection	Home commercial circuit
Power Saving King	Screen control ；<br>overload overpressure protection ；<br>in situ compensation ；<br>independent fuse	Home commercial circuit
Smart Power Saver	Screen control ；<br>overload overpressure protection ；<br>reactive power compensation	Home commercial circuit
Smart Power saver	Reactive power compensation No heat<br>overload protection for low temperature operation	Home commercial circuit
Power Saving King	Screen control ；<br>overload overpressure protection ；<br>in situ compensation ；<br>independent fuse	Home commercial circuit
Power Saving King	Screen control ；<br>independent fuse	Home commercial circuit
Power Saving King	Screen control ；<br>overload overpressure protection ；<br>local compensation	Home commercial circuit
Power Saving King	Screen control ；<br>overload overpressure protection ；<br>in situ compensation ；<br>independent fuse	Home commercial circuit
Power Saving King	Liquid crystal display ；<br>overload overpressure protection ；<br>local compensation ；<br>independent fuse	Home commercial circuit
Power Saving King	Screen control ；<br>overload overpressure protection ；<br>in situ compensation ；<br>independent fuse	Home commercial circuit
Power Saving King	Screen control ；<br>overload overpressure protection ；<br>in situ compensation ；<br>independent fuse	Home commercial circuit
Power Saving King	Screen control ；<br>overload overpressure protection ；<br>local compensation	Home commercial circuit
Smart Power saver	none	Home commercial circuit

Power Saving King	Voltage regulator；<br>screen display control；<br>overload overpressure protection；<br>local compensation；<br>independent fuse	Home commercial circuit
Power Saving King	Screen control；<br>overload and overpressure protection	Home commercial circuit
Power Saving King	Indicator；<br>screen control；<br>independent fuse	Home commercial circuit
Power Saving King	Plug and play；<br>voltage display	Home commercial circuit
Power Saver	Voltage regulator；<br>screen display control；<br>overload overpressure protection；<br>local compensation；<br>independent fuse	Home commercial circuit
Smart Power saver	none	Home commercial circuit
Power Saving King	none	Home commercial circuit
Power Saving King	Voltage regulator；<br>indicator；<br>screen display control；<br>independent fuse	Home commercial circuit
Save electricity	Screen display control；<br>independent fuse	Home commercial circuit
Gold power saving expert	Voltage regulator；<br>screen display control；<br>overload overpressure protection；<br>local compensation；<br>independent fuse	Home commercial circuit
Gold power saving expert	Screen control；<br>overload overpressure protection；<br>local compensation	Home commercial circuit
Power saver	Screen control；<br>overload overpressure protection；<br>local compensation	Home commercial circuit
Whole House power Saver	Screen control；<br>overload and overpressure protection；<br>local compensation；<br>independent safety tube	Home commercial circuit
Huang saves electricity	Screen control；<br>overload and overpressure protection；<br>local compensation；<br>independent safety tube	Home commercial circuit
Purple power saving expert	Screen control；<br>overload and overpressure protection；<br>local compensation；<br>independent safety tube	Home commercial circuit
Power Saver	Screen control；<br>overload and overpressure protection；<br>local compensation；<br>independent safety tube	Home commercial circuit
Gold power saving expert	Indicator；<br>screen control；<br>independent fuse	Home commercial circuit
Gold power saving expert	Voltage regulator；<br>screen display control；<br>overload and overpressure protection；<br>local compensation；<br>independent safety tube	Home commercial circuit
Smart Power saver	Screen control；<br>overload and overpressure protection；<br>local compensation；<br>independent safety tube	Home commercial circuit
Power saving expert	Voltage regulator；<br>screen display control；<br>overload overpressure protection；<br>local compensation	Home commercial circuit
Smart power saving expert	LCD screen display	Home commercial circuit
Power Saving King	Screen control；<br>overload overpressure protection；<br>in situ compensation；<br>independent fuse	Home commercial circuit
Power saving expert	Screen control；<br>independent fuse	Home commercial circuit
Smart Power saver	Voltage regulator；<br>screen display control；<br>overload overpressure protection；<br>local compensation	Home commercial circuit
Gold power saving expert	Screen control；<br>overload overpressure protection；<br>local compensation	Home commercial circuit
Power Saving King	Voltage regulator；<br>screen display control；<br>overload overpressure protection；<br>local compensation；<br>independent fuse	Home commercial circuit
Power Saving King	Screen control；<br>overload overpressure protection；<br>in situ compensation；<br>independent fuse	Home commercial circuit
Platinum power saving expert	Screen control；<br>independent fuse	Home commercial circuit
Lake Blue power saving expert	Screen display control；<br>independent fuse	Home commercial circuit
Black power saving expert	Screen display control；<br>overheat and overload protection；<br>local compensation	Home commercial circuit
Smart power saving expert	LCD screen display	Home commercial circuit
ntelligent frequency conversion high pow	none	Home commercial circuit

Smart power saving expert	LCD screen display	Home commercial circuit
Electrical Officer	Screen control；<br>overload and overpressure protection；<br>local compensation；<br>independent safety tube	Home commercial circuit
Aijia intelligent energy-saving appliance	none	Home commercial circuit
Save electricity	Screen control；<br>overload overpressure protection；<br>in situ compensation；<br>independent fuse	Home commercial circuit
Light blue saves electricity	Screen control；<br>overload overpressure protection；<br>in situ compensation；<br>independent fuse	Home commercial circuit
(Three phase) Electricity officer	Individual safety tube	Home commercial circuit
High power super saver	Voltage regulator；<br>frequency conversion consumption reduction；<br>reactive power compensation	Home commercial circuit
Power Saving King	Screen monitoring；<br>overload overpressure protection；<br>independent fuse；<br>local compensation	Home commercial circuit
Blue power saving expert	Voltage regulator；<br>screen display control；<br>overload and overpressure protection；<br>local compensation；<br>independent safety tube	Home commercial circuit
Deep Blue power saving expert	Screen control；<br>overload and overpressure protection；<br>local compensation；<br>independent safety tube	Home commercial circuit
Power Saving King	Screen control；<br>overload and overpressure protection；<br>local compensation；<br>independent safety tube	Home commercial circuit
Blue power saving expert	Screen control；<br>overload and overpressure protection；<br>local compensation；<br>independent safety tube	Home commercial circuit
Deep Blue power saving expert	Screen control；<br>overload overpressure protection；<br>in situ compensation；<br>independent fuse	Home commercial circuit
Electric Butler	none	Home commercial circuit
Gold power saving expert	Screen control；<br>overload overpressure protection；<br>in situ compensation；<br>independent fuse	Home commercial circuit
Copper power saving expert	Indicator；<br>screen display control；<br>overload overpressure protection；<br>on the spot compensation；<br>independent safety tube	Home commercial circuit
Blue power saving expert	Indicator；<br>screen display control；<br>overload overpressure protection；<br>on the spot compensation；<br>independent safety tube	Home commercial circuit
Power saving expert	Screen control；<br>overload overpressure protection；<br>in situ compensation；<br>independent fuse	Home commercial circuit
Electrical Officer	Screen control；<br>overload and overpressure protection；<br>local compensation；<br>independent safety tube	Home commercial circuit
Power Saving King	Screen control	Home commercial circuit
Power Saving King	Voltage regulator；<br>indicator；<br>screen display control；<br>independent fuse	Home commercial circuit
High power super power saving	The screen control；<br>voltage can be seen	Home commercial circuit
Deep blue saves electricity	Screen control；<br>overvoltage protection；<br>in situ compensation；<br>independent fuse	Home commercial circuit
Red power saving expert	Screen control；<br>overload overpressure protection；<br>in situ compensation；<br>independent fuse	Home commercial circuit
