# Supplementary material for: Exploring online consumer behavior on fraudulent energy-saving products
Source: Sci Rep. 2024 Jun 21;14:14304. doi: 10.1038/s41598-024-65210-1 (PMC11192901; doi:10.1038/s41598-024-65210-1)
Supplement: Supplementary file 2 — Supplementary Table 2. [file 41598_2024_65210_MOESM2_ESM.pdf]

source	Merchant name	Product name	Price	Sales volume	Number of Reviews	Warranty Period	Features	Characteristics	arameter	Applicable	Size (cm)	Additional features	Frequency of use
Taobao	Overseas high-end care	Battery restorers	9.8	500.00	79	90-day free trial<br>with 2-year free renewal lifetime service	Extend battery life<br>fix phone jams<br>check current voltage	none	200%	Cell phone	none	none	none
Taobao	Niu People Living appliances	Battery repair artifact	18.8	73.00	54	none	Detect current<br>voltage<br>repair<br>maintain battery<br>improve life	Plug<br>play<br>compact<br>convenient	200%	Various digital products	none	none	none
Taobao	Niu People Living appliances	Battery restorers	19.9	16.00	11	90 day free trial with refund	Fix<br>prolong life<br>clean up cell phone trash<br>lower radiation	Plug<br>play<br>display<br>over voltage<br>over current<br>over power<br>mute<br>short circuit<br>temperature protection	0	Various cell phones<br>power banks<br>laptops	53 * 24 * 40	Intelligent Voice broadcasting	none
Taobao	Niu People Living appliances	Battery repair artifact	49.7	500.00	300	none	Pulse repair Battery capacity<br>maintenance<br>life extension	High heat dissipation<br>fix stuck<br>clean up litter	0	Various digital products	none	none	none
Taobao	Niu People Living appliances	Battery repair artifact	59.9	1.00	0	none	Pulse Repair battery<br>maintenance<br>Life extension	Solve the battery is full at a charge<br>charging does not turn the lamp<br>battery expansion<br>lack of uphill strength<br>battery life reduction<br>heat<br>maintenance<br>lack of power	0	Battery Cart	none	none	none
Taobao	Ri In Department Store	Battery repair artifact	26	1000.00	400	30 days no reason to return<br>100 days to replace 10 years free repair	Life extension<br>Battery repair<br>Fix Stuck<br>Clean up phone trash	Solve heat<br>fast power consumption<br>slow charging<br>check the current voltage	200%	Multi-device compatibility<br>mobile phone tablet	67 * 23.8 * 14.3	none	none
Taobao	Viet Ba Flagship Store	Battery repair artifact	1.8-49.	200.00	44	90-day free trial<br>with 2-year free renewal lifetime service	Boost efficiency<br>charge<br>upkeep<br>fix stuck<br>clean up litter	Check current voltage<br>high heat dissipation	400%	Multi-device compatibility<br>mobile phone tablet	76 * 45 * 22	none	Battery repair for 3 hours<br>1-2 times a week for 5-8 weeks
Taobao	Fenyue Electronics	Battery repair artifact	13.8	700.00	100	none	Life extension<br>Battery repair<br>Fix caton<br>Clean up mobile phone litter<br>activate	Reduce radiation<br>check current voltage	0	Multi-device compatibility<br>mobile phone tablet	76 * 45 * 22	none	Battery repair for 3 hours<br>1-2 times a week for 5-8 weeks
Taobao	Siluwang flagship store	Battery repair artifact	48	400.00	82	none	Life extension<br>Battery repair<br>Fix Stuck<br>Clean up phone trash	Check the current voltage<br>over voltage<br>over current<br>over power<br>mute<br>short circuit<br>temperature protection	0	Smart phone	75 * 25 * 13	Intelligent Voice broadcasting	Battery repair for 3 hours<br>1-2 times a week for 5-8 weeks
Taobao	Chuyi Life Hall	Battery repair artifact	11.8	800.00	300	30 days no reason to return<br>100 days to replace 10 years free repair	Life extension<br>Battery repair<br>Fix Stuck<br>Clean up phone trash	Solve heat<br>fast power consumption<br>slow charging<br>check the current voltage	200%	Smart phone	76 * 45 * 22	none	Battery repair for 3 hours<br>1-2 times a week for 5-8 weeks
Taobao	L Science<br>Technology Life Hall	Battery repair artifact	23.9	100.00	100	none	Life extension<br>Battery repair<br>maintenance<br>fix stuck<br>Clean up mobile phone litter	none	0	Huawei<br>Apple<br>Xiaomi<br>oppo<br>vivo phone	none	none	none
Taobao	Tianxing Jianyi	Battery restorers	17	52.00	38	none	Life extension<br>battery repair<br>maintenance	Check current voltage	0	All cell phones	none	none	none
Taobao	Xumbe flagship store	Battery repair artifact	10	200.00	100	none	Life extension<br>Battery repair<br>Fix Stuck<br>Clean up phone trash	Real-time monitoring of current voltage	0	Smart phone	8.2 * 29.9 * 16.	Intelligent Voice broadcasting	Battery repair for 3 hours<br>1-2 times a week for 5-8 weeks
Taobao	Xumbe flagship store	Battery repair artifact	19.9	1000.00	500	none	Charge repair<br>Fix stuck<br>Clean up phone litter	Real-time monitoring of current voltage<br>efficient heat dissipation	0	Multi-device compatibility<br>mobile phone tablet	76 * 45 * 22	none	Battery repair for 3 hours<br>1-2 times a week for 5-8 weeks
Taobao	Zhiheng Technology	Battery restorers	6.8	15.00	0	none	Repair<br>Charge<br>Maintenance<br>repair caton<br>Clean up mobile phone litter	Ultra long battery life<br>check the current voltage	0	Multi-device compatibility<br>mobile phone tablet	76 * 45 * 22	none	Battery repair for 3 hours<br>1-2 times a week for 5-8 weeks
Taobao	Zhiheng Technology	Battery restorers	22	200.00	44	none	Pulse repair<br>charging<br>maintenance<br>repair stuck<br>clean up mobile phone litter	Real-time monitoring of current voltage	0	Huawei<br>Apple<br>Xiaomi<br>oppo<br>vivo phone	6.6 * 24.7 * 11.	none	none
Taobao	Xiaoyu Summer Trade Shop	Battery restorers	13.8	100.00	35	none	Charge repair<br>repair of stuck<br>aging<br>machine hot<br>clean up mobile phone garbage	none	0	Smart phone	none	none	none

Taobao	Hang Seng Enterprise Store	Smart pulse regulator for electric vehicles	69	2000.00	300	none	Battery<br>repair<br>maintenance<br>activation<br>life extension<br>battery deep cleaning<br>current detection	Solve the battery is full at a charge<br>charging does not turn the lamp<br>battery expansion<br>lack of uphill strength<br>battery life reduction<br>heat<br>maintenance<br>lack of power	100%	EVS	none	none	none
Taobao	Dream Home Franchise store	New Super Wei battery restorer	49.9	600.00	57	none	Battery<br>repair<br>maintenance<br>mileage<br>life extension<br>battery deep cleaning<br>current detection	Solve the battery is full at a charge<br>charging does not turn the lamp<br>battery expansion<br>lack of uphill strength<br>battery life reduction<br>heat<br>maintenance<br>lack of power	100%	EVS	none	none	none
Taobao	Exuberant Scientific Research Museum	Battery restorer	28	100.00	28	90 day free trial with refund	Pulse repair<br>charge<br>maintenance to increase mileage<br>extend battery life for 3-5 years	Automatic power off<br>increase battery life<br>protect the battery silent charging<br>real-time monitoring of current<br>voltage	0	Fully automatic electric vehicl	110 * 34.5 * 57	none	none
Taobao	Luk Moon Auspicious	Battery restorer	28	100.00	13	90 days free return<br>exchange	Pulse repair<br>charge<br>maintenance<br>activation to increase mileage<br>extend life of battery for 3-5 years	One key repair<br>automatic power off to increase battery life<br>protect the battery silent charging<br>real-time monitoring of current<br>voltage	0	Fully automatic electric vehicl	110 * 34.5 * 57	none	none
Taobao	Luk Moon Auspicious	Battery restorers	23	10.00	5	30 days no reason to return<br>100 days to replace 10 years free<br>repair	Charge repair<br>repair of stuck<br>aging<br>machine hot<br>clean up mobile phone garbage	Extend battery life<br>check current voltage	0	Smart phone	none	none	none
Taobao	Kuyasu Health Don	Battery restorer	29.9	19.00	2	90 days no reason to return	Pulse repair<br>charge<br>maintenance<br>activation to increase mileage<br>and prolong life	Automatic power off<br>increase battery life<br>boost power<br>silent charging<br>check current voltage	0	Fully automatic electric vehicl	110 * 34.5 * 57	none	none
Taobao	Shunfeng Shunshuiyuan Merchandise store	Air conditioner intelligent frequency conversion repairer	8.41	1000.00	100	none	Electrical repair<br>energy saving<br>voltage regulation<br>optimization circuit	none	0	air conditioner.	none	none	none
Taobao	Tik Tok Boom specials	Air conditioner intelligent frequency conversion repairer	15.8	200.00	29	none	Electrical repair<br>energy saving<br>voltage regulation<br>optimization circuit	none	0	air conditioner.	none	none	none
Taobao	Tik Tok Boom specials	Battery restorer	45.8	600.00	4	none	Battery<br>repair<br>maintenance<br>activation<br>life extension<br>battery deep cleaning<br>current detection	Solve the battery is full at a charge<br>charging does not turn the lamp<br>battery expansion<br>lack of uphill strength<br>battery life reduction<br>heat<br>maintenance<br>lack of power	100%	EVS	none	none	none
Taobao	Dianli flagship store	Battery restorers	28.8	14.00	6	none	Life extension<br>Battery repair<br>Charging	Real-time monitoring of current voltage<br>repair of stuck<br>clean up mobile phone litter	0	Multi-device compatibility<br>mobile phone tablet	76 * 45 * 22	none	Battery repair for 3 hours<br>1-2 times a week for 5-8 weeks
Taobao	Dianli flagship store	Air conditioner intelligent frequency conversion repairer	28.8	200.00	53	none	Electrical repair<br>energy saving<br>voltage regulation<br>optimization circuit	none	0	air conditioner.	none	none	none
Taobao	Huiren Science Technology Museum	Air conditioner intelligent frequency conversion repairer	28	100.00	79	90 day free trial with refund	Electrical repair<br>energy saving<br>voltage regulation<br>optimization circuit	none	0	air conditioner.	none	none	none
Taobao	Huiren Science Technology Museum	Air conditioner intelligent frequency conversion repairer	21	33.00	9	90 day free trial with refund	Electrical repair<br>energy saving<br>voltage regulation<br>optimization circuit	none	0	air conditioner.	none	none	none
Taobao	Huiren Science Technology Museum	Air conditioner intelligent frequency conversion repairer	21	33.00	22	90 day free trial with refund	Electrical repair<br>energy saving<br>voltage regulation<br>optimization circuit	none	0	air conditioner.	none	none	none
Taobao	Huiren Science Technology Museum	Battery restorers	23	200.00	100	30 days no reason to return<br>100 days to replace 10 years free<br>repair	Life extension<br>Battery repair<br>Charging	Real-time monitoring of current voltage<br>repair of stuck<br>clean up mobile phone litter	0	Multi-device compatibility<br>mobile phone tablet	none	none	none
Taobao	Huiren Science Technology Museum	Battery restorer	35	17.00	1	91-day free trial<br>with refund guaranteed	Pulse repair<br>charge<br>maintenance<br>activation to increase mileage<br>and prolong life	Automatic power off<br>increase battery life<br>boost power<br>silent charging<br>check current voltage	0	Fully automatic electric vehicl	110 * 34.5 * 57	none	none
Taobao	altt flagship store	1st generation Battery Restorer	54.9	4000.00	1200	none	Life Extension<br>Repair	Fix cell phone jams<br>Check current voltage<br>Clean up cell phone trash	200%	Multi-device compatibility<br>mobile phone tablet	76 * 45 * 22	none	Battery repair for 3 hours<br>1-2 times a week for 5-8 weeks
Taobao	altt flagship store	2nd generation battery fixer	64.9	2000.00	600	none	Life Extension Repair	Fix cell phone jams<br>Check current voltage<br>Clean up cell phone trash	2	Multi-device compatibility<br>mobile phone tablet	76 * 45 * 22	none	Battery repair for 3 hours<br>1-2 times a week for 5-8 weeks
Taobao	altt flagship store	3rd generation Battery Repairer	72.9	1000.00	200	none	Life Extension Repair	Fix cell phone jams<br>Check current voltage<br>Clean up cell phone trash	2	Multi-device compatibility<br>mobile phone tablet	76 * 45 * 22	none	Battery repair for 3 hours<br>1-2 times a week for 5-8 weeks

Taobao	blesi flagship store	Classic Black	29.8	100.00	62	none	Battery<br>Repair<br>Maintenance<br>Activation<br>Increased mileage	Solve the battery is full at a charge<br>charging does not turn the lamp<br>battery expansion<br>insufficient uphill force<br>ionized water crystallization<br>deep cleaning<br>real-time monitoring of current<br>full stop	200%	Various digital products	76 * 45 * 22	none	Battery repair for 3 hours<br>1-2 times a week for 5-8 weeks
Taobao	blesi flagship store	Moonlight White	29.8	88.00	60	none	Battery<br>Repair<br>Maintenance<br>Activation<br>Increased mileage	Solve the battery is full at a charge<br>charging does not turn the lamp<br>battery expansion<br>insufficient uphill force<br>ionized water crystallization<br>deep cleaning<br>real-time monitoring of current<br>full stop	200%	Various digital products	76 * 45 * 22	none	Battery repair for 3 hours<br>1-2 times a week for 5-8 weeks
Taobao	blesi flagship store	Lemon Yellow	11.8	64.00	50	none	Repair<br>Life extension	Detect current<br>voltage in real time<br>repair cell phone jams<br>clean up cell phone garbage	80%	Various digital products	76 * 45 * 22	none	Battery repair for 3 hours<br>1-2 times a week for 5-8 weeks
Taobao	blesi flagship store	Lemon Yellow	16.8	55.00	40	none	Pulse repair<br>battery<br>maintenance<br>life extension	Detect current<br>voltage in real time<br>repair cell phone jams<br>clean up cell phone garbage	90%	Smartphone Universal	76 * 45 * 22	none	Battery repair for 3 hours<br>1-2 times a week for 5-8 weeks
Taobao	blesi flagship store	Lemon Yellow	19.8	34.00	30	none	Repair<br>Life extension	Real-time detection of current<br>voltage power consumption<br>repair of mobile phone jams<br>clean up mobile phone garbage	100%	Various digital products	76 * 45 * 22	none	Battery repair for 3 hours<br>1-2 times a week for 5-8 weeks
Pinduoduo	Xinyun Shopkeepers	Battery restorer	58.2	6227.00	1134	5 years warranty free renewal	Pulse repair<br>charge<br>maintenance<br>activation<br>increase mileage<br>prolong life battery	Battery deep cleaning<br>real-time monitoring of current<br>automatic power off<br>overvoltage<br>overheating<br>short circuit<br>insurance protection	100%	Fully automatic electric vehicl	none	none	none
Pinduoduo	Catebon Technology	Battery restorers	49	2.00	5671	none	Pulse Repair Battery Capacity<br>Maintenance<br>Activation<br>Life Extension battery	Real-time monitoring of current<br>voltage<br>repair of stuck<br>clean up mobile phone garbage<br>release memory	0	Various digital products	76 * 45 * 22	none	Battery repair for 3 hours<br>1-2 times a week for 5-8 weeks
Pinduoduo	Catebon Technology	Battery restorers	51.3	5534.00	2295	Free data cable	Pulse repair<br>life extension	Real-time monitoring of current<br>voltage<br>repair of stuck<br>clean up mobile phone garbage<br>release memory	0	Multi-device compatibility<br>mobile phone tablet	76 * 45 * 22	none	Battery repair for 3 hours<br>1-2 times a week for 5-8 weeks
Pinduoduo	Baitong Selection	Battery restorers	8.8	2.00	3028	No results guaranteed return	Pulse repair<br>Life extension<br>charging	Fix the stuck<br>Clean up the phone litter	100%	All cell phones	75 * 25 * 13	none	none
Pinduoduo	Baitong Selection	Battery restorers	5.9	947.00	251	none	Pulse repair<br>Life extension<br>charging	Fix the stuck<br>Clean up the phone litter	0	Multi-device compatibility<br>mobile phone tablet	75 * 25 * 13	none	none
Pinduoduo	Jiu<br>Mei	New Super Wei battery restorer	52.79	5000.00	600	Suitable for 2-wheeled battery cars	Battery<br>repair<br>maintenance<br>range<br>life extension<br>activation	Battery deep cleaning<br>real-time monitoring of current<br>automatic power off<br>overvoltage<br>overheating<br>short circuit<br>insurance protection	100%	EVS	none	none	none
Pinduoduo	JiuMei	New Super Wei battery restorer	58.79	1000.00	400	For 3-wheeled battery cars	Battery<br>repair<br>maintenance<br>range<br>life extension<br>activation	Battery deep cleaning<br>real-time monitoring of current<br>automatic power off<br>overvoltage<br>overheating<br>short circuit<br>insurance protection	1	EVS	none	none	none
Pinduoduo	JiuMei	New Super Wei battery restorer	62.79	1800.00	221	Suitable for 4-wheel battery car	Battery<br>repair<br>maintenance<br>range<br>life extension<br>activation	Battery deep cleaning<br>real-time monitoring of current<br>automatic power off<br>overvoltage<br>overheating<br>short circuit<br>insurance protection	1	EVS	none	none	none
Pinduoduo	Friendly objects	Battery restorers	13.2	7677.00	3135	Free data cable	Pulse repair Battery capacity<br>maintenance<br>life extension	Monitor current<br>voltage in real time<br>fix stuck<br>clean up mobile phone litter<br>improve battery efficiency	0	Smart phone	76 * 45 * 22	none	none
Pinduoduo	Friendly objects	Battery restorers	25.5	366.00	116	Free data cable	Pulse repair<br>maintenance<br>life extension	Monitor current<br>voltage in real time<br>fix stuck<br>clean up mobile phone litter<br>improve battery efficiency	0	Multi-device compatibility<br>mobile phone tablet	76 * 45 * 22	none	Battery repair for 3 hours<br>1-2 times a week for 5-8 weeks
Pinduoduo	Friendly objects	Battery restorers	48.6	1160.00	449	Free data cable	Low temperature pulse repair<br>maintenance<br>life extension	Monitor current<br>voltage in real time<br>fix stuck<br>clean up mobile phone litter<br>improve battery efficiency	0	Multi-device compatibility<br>mobile phone tablet	76 * 45 * 22	none	Battery repair for 3 hours<br>1-2 times a week for 5-8 weeks

Pinduoduo	Friendly objects	Battery restorer	58	5200.00	949	none	Pulse repair<br>charge<br>maintenance<br>activation to increase mileage<br>and extend life of battery for 3-5 years	One key repair<br>automatic power off to increase battery life<br>protect the battery silent charging<br>real-time monitoring of current<br>voltage	0	Fully automatic<br>electric vehicle	none	Fire, heat<br>drop resistant	none
Pinduoduo	Friendly objects	Battery restorer	58	218.00	120	none	Pulse repair<br>charge<br>maintenance<br>activation to increase mileage<br>and extend life of battery for 3-5 years	One key repair<br>automatic power off to increase battery life<br>protect the battery silent charging<br>real-time monitoring of current<br>voltage	0	Fully automatic<br>electric vehicle	none	Fire, heat<br>drop resistant	none
Pinduoduo	Henrui new energy repair technology	Battery restorers	25	2.00	202	90 day free trial with refund	Optimization<br>charging<br>superconducting pulse repair<br>maintenance	Fix the stuck<br>clean up the phone garbage<br>check the current voltage<br>free up the memory	0	Huawei<br>Apple<br>Xiaomi<br>oppo<br>vivo phone	76 * 45 * 22	Full program temperature control	Battery repair for 3 hours<br>1-2 times a week for 5-8 weeks
Pinduoduo	Henrui new energy repair technology	Battery restorers	35	0.00	0	none	Optimization<br>charging<br>superconducting pulse repair<br>maintenance	Fix the stuck<br>clean up the phone garbage<br>check the current voltage<br>free up the memory	0	Huawei<br>Apple<br>Xiaomi<br>oppo<br>vivo phone	76 * 45 * 22	Full program temperature control	Battery repair for 3 hours<br>1-2 times a week for 5-8 weeks
Pinduoduo	Henrui new energy repair technology	Battery restorers	27.79	5104.00	518	none	Charge<br>Superconducting pulse repair<br>maintenance	Monitor current<br>voltage in real time<br>fix stuck<br>clean up mobile phone litter<br>improve battery efficiency	0	Multi-device compatibility<br>mobile phone tablet	76 * 45 * 22	none	Battery repair for 3 hours<br>1-2 times a week for 5-8 weeks
Pinduoduo	German Tech Tech	Battery restorer	40.1	10000.00	72	none	Pulse repair<br>increased mileage<br>improved life<br>real-time monitoring of current	Solve the battery is full at a charge<br>charging does not turn the lamp<br>battery expansion<br>lack of uphill strength<br>battery life reduction<br>heat<br>maintenance<br>lack of power	100%	EVS	none	none	none
Pinduoduo	German Tech Tech	Battery restorer	40.1	5026.00	100	none	Pulse repair<br>increased mileage<br>improved life<br>real-time monitoring of current	Solve the battery is full at a charge<br>charging does not turn the lamp<br>battery expansion<br>lack of uphill strength<br>battery life reduction<br>heat<br>maintenance<br>lack of power	100%	EVS	none	none	none
Pinduoduo	Wang Wang Digital	Battery restorers	49	3020.00	1015	none	Life Extension<br>Battery repair	Fix the stuck<br>check the current voltage<br>clean up the cell phone garbage<br>improve battery efficiency	200%	Multi-device compatibility<br>mobile phone tablet	76 * 45 * 22	none	Battery repair for 3 hours<br>1-2 times a week for 5-8 weeks
Pinduoduo	Royal Preference	Battery restorers	4.34	100000.00	3.7	none	Life Extension<br>Battery repair	Real-time monitoring of current voltage<br>repair of stuck<br>clean up mobile phone litter	0	Various digital products	none	none	none
Pinduoduo	Starry Night Life Hall	Battery restorers	6.16	6926.00	437	No results guaranteed return	Battery<br>Pulse Repair<br>Life Extension	Real-time monitoring of current voltage<br>repair of stuck<br>clean up mobile phone litter	100%	All cell phones	76 * 45 * 22	none	Battery repair for 3 hours<br>1-2 times a week for 5-8 weeks
Pinduoduo	A good thing is a good thing	Battery restorers	39.9	1940.00	0	none	Battery<br>Pulse Repair<br>Life Extension	Real-time monitoring of current voltage<br>repair of stuck<br>clean up mobile phone litter	100%	Multi-device compatibility<br>mobile phone tablet	76 * 45 * 22	none	Battery repair for 3 hours<br>1-2 times a week for 5-8 weeks
Pinduoduo	Mukeyi digital selection	Electric vehicle restoration artifact	32.4	183.00	43	none	Silent charging<br>Battery<br>repair<br>maintenance<br>activation	Automatic power off<br>increase battery life<br>boost power<br>silent charging<br>check current voltage	0	EVS	110 * 34.5 * 57	Fire, heat<br>drop resistant	none
Pinduoduo	Flower Rich Big Store	Repair Adapter	18.97	128.00	1	Free 180-day trial<br>refund guaranteed	Charge<br>Repair<br>Fix Stuck<br>Reduce radiation	Over voltage<br>over current<br>over power<br>mute<br>short circuit<br>temperature protection<br>free up memory<br>clean up phone junk	0	Smart phone	53 * 24 * 40	Intelligent Voice broadcasting	none
Pinduoduo	Smart 3C Digital furniture AD	Battery restorers	19.9	4190.00	1690	none	Battery<br>repair<br>maintenance<br>range<br>life extension<br>activation	Improve efficiency<br>detect current<br>voltage	0	Smart phone	none	none	none
Pinduoduo	Home living equipment	Cell phone battery adapter	23.92	35.00	1	Free 180-day trial<br>refund guaranteed	Battery<br>repair<br>maintenance<br>range<br>life extension<br>activation	Battery deep cleaning<br>real-time monitoring of current<br>automatic power off<br>overvoltage<br>overheating<br>short circuit<br>insurance protection	0	Multi-device compatibility<br>mobile phone tablet	53 * 24 * 40	Intelligent Voice broadcasting	none
Pinduoduo	Home living equipment	Cell phone battery adapter	23.92	1.00	0	Free 180-day trial<br>refund guaranteed	Charging Repair<br>Life extension	Fix the stuck<br>clean up the phone garbage<br>check the current voltage<br>free up the memory	0	Multi-device compatibility<br>mobile phone tablet	53 * 24 * 40	Intelligent Voice broadcasting	none
Pinduoduo	Home living equipment	Cell phone battery adapter	23.52	5.00	0	Free 180-day trial<br>refund guaranteed	Charging Repair<br>Life extension	Fix the stuck<br>clean up the phone garbage<br>check the current voltage<br>free up the memory	0	Multi-device compatibility<br>mobile phone tablet	53 * 24 * 40	Intelligent Voice broadcasting	none

Pinduoduo	Home living equipment	Cell phone battery adapter	23.52	108.00	57	Free 180-day trial<br>refund guaranteed	Charging Repair<br>Life extension	Fix the stuck<br>clean up the phone garbage<br>check the current voltage<br>free up the memory	0	Multi-device compatibility<br>mobile phone tablet	53 * 24 * 40	Intelligent Voice broadcasting	none
Pinduoduo	Joban outdoor equipment<br>franchise store	Battery restorers	75.5	0.00	0	5-year warranty	Pulse repair<br>maintenance<br>Battery<br>Life extension	Fix stuck<br>improve efficiency<br>Check current voltage	200%	Multi-device compatibility<br>mobile phone tablet	76 * 45 * 22	none	Battery repair for 3 hours<br>1-2 times a week for 5-8 weeks
Pinduoduo	Xiaohai Home<br>Department Store	Air conditioner intelligent<br>frequency conversion repairer	6.47	396.00	94	none	Electrical repair<br>energy saving<br>voltage regulation<br>optimization circuit	Overload protection<br>lightning protection<br>Electrical surge protection	0	air conditioner.	none	none	none
Pinduoduo	Xinshuo Black Ma	Air conditioner intelligent<br>frequency conversion repairer	16.88	127.00	21	none	Electrical repair<br>energy saving<br>voltage regulation<br>optimization circuit	none	0	1.5pc air conditioner	none	none	none
Pinduoduo	Xinshuo Black Ma	Air conditioner intelligent<br>frequency conversion repairer	19.99	14.00	6	none	Electrical repair<br>energy saving<br>voltage regulation<br>optimization circuit	none	0	Air-conditioned refrigerator	none	none	none
Pinduoduo	Xinshuo Black Ma	Air conditioner intelligent<br>frequency conversion repairer	18.88	32.00	7	none	Electrical repair<br>energy saving<br>voltage regulation<br>optimization circuit	none	0	Air-conditioned refrigerator	none	none	none
Pinduoduo	Research beauty care	Air conditioner intelligent<br>frequency conversion repairer	37.9	191.00	33	none	Electrical repair<br>energy saving<br>voltage regulation<br>optimization circuit	none	0	Air-conditioned refrigerator	none	Mute	none
Pinduoduo	Research beauty care	Electric vehicle restoration artifact	99	974.00	115	none	Fix<br>Activate<br>Charge	Overcurrent<br>short circuit<br>overvoltage<br>overheat<br>reverse protection<br>detect voltage charge	0	EVS	110 * 34.5 * 57	Fire, heat<br>drop resistant	none
Pinduoduo	Geeky goodies	Electric vehicle restoration artifact	58	355.00	104	none	Auto repair<br>Silent charging<br>Maintenance<br>activating battery<br>Life extension	Detect current voltage	0	EVS	110 * 34.5 * 58	Fire, heat<br>drop resistant	none
Pinduoduo	Tekulla Smart	Battery restorers	35.6	100000.00	443	30-day trial<br>3-year warranty	Battery<br>repair<br>maintenance<br>range<br>life extension<br>activation	Real-time monitoring of current voltage<br>repair of stuck<br>clean up mobile phone litter	0	Multi-device compatibility<br>mobile phone tablet	76 * 45 * 22	none	Battery repair for 3 hours<br>1-2 times a week for 5-8 weeks
Pinduoduo	Year Explosive Store	Battery restorers	36.72	200.00	7	Zero yuan order support trial	Battery<br>repair<br>maintenance<br>life extension	Real-time monitoring of current voltage<br>repair of stuck<br>clean up mobile phone litter	0	Multi-device compatibility<br>mobile phone tablet	none	none	none
Pinduoduo	Year Explosive Store	Battery restorers	28.79	98.00	5	Zero yuan order support trial	Battery<br>repair<br>maintenance<br>life extension	Real-time monitoring of current voltage<br>repair of stuck<br>clean up mobile phone litter	0	Multi-device compatibility<br>mobile phone tablet	none	none	none
Pinduoduo	Year Explosive Store	Battery restorers	12.95	78.00	2	Zero yuan order support trial	Battery<br>repair<br>maintenance<br>life extension	Real-time monitoring of current voltage<br>repair of stuck<br>clean up mobile phone litter	0	Multi-device compatibility<br>mobile phone tablet	none	none	none
Pinduoduo	Wan Xuan Technology	Battery restorers	15.9	247.00	87	Free 180-day trial<br>refund guaranteed	Charge repair<br>Life extension<br>Maintenance	Detection of current<br>voltage power consumption<br>heat dissipation<br>repair of mobile phone stuck<br>clean up mobile phone garbage	1%	All cell phones	none	none	none
Pinduoduo	Wan Xuan Technology	Battery restorers	16.9	32.00	7	Free 180-day trial refund guaranteed	Charge repair<br>Life extension<br>Maintenance	Detection of current<br>voltage power consumption<br>heat dissipation<br>repair of mobile phone stuck<br>clean up mobile phone garbage	50%	All cell phones	none	none	none
Pinduoduo	Wan Xuan Technology	Battery restorers	18.9	4495.00	1764	Free 180-day trial refund guaranteed	Charge repair<br>Life extension<br>Maintenance	Detection of current<br>voltage power consumption<br>heat dissipation<br>repair of mobile phone stuck<br>clean up mobile phone garbage	100%	All cell phones	none	none	none
Pinduoduo	Wan Xuan Technology	Battery restorers	22.9	21.00	9	Free 180-day trial refund guaranteed	Charge repair<br>Life extension<br>Maintenance	Detection of current<br>voltage power consumption<br>heat dissipation<br>repair of mobile phone stuck<br>clean up mobile phone garbage	0	All cell phones	none	none	none
Pinduoduo	Good things are best	Battery restorers	1.09	13.00	4	none	Charge repair<br>Life extension<br>Maintenance	Detection of current<br>voltage power consumption<br>heat dissipation<br>repair of mobile phone stuck<br>clean up mobile phone garbage	10%	All cell phones	none	none	none
Pinduoduo	Good things are best	Battery restorers	1.12	193.00	26	none	Charge repair<br>Life extension<br>Maintenance	Detection of current<br>voltage power consumption<br>heat dissipation<br>repair of mobile phone stuck<br>clean up mobile phone garbage	60%	All cell phones	none	none	none

Pinduoduo	Good things are best	Battery restorers	2.96	45.00	8	none	Charge repair<br>Life extension<br>Maintenance	Detection of current<br>voltage power consumption<br>heat dissipation<br>repair of mobile phone stuck<br>clean up mobile phone garbage	200%	All cell phones	none	none	none
Pinduoduo	Good things are best	Battery restorers	3.53	308.00	73	The effect is obvious after 5-8 weeks	Pulse charge repair<br>life extension<br>maintenance<br>battery	Detect current<br>voltage<br>High speed<br>low temperature repair<br>repair mobile phone stuck<br>clean up mobile phone garbage	80%	Multi-device compatibility<br>mobile phone tablet	76 * 45 * 22	none	Battery repair for 3 hours<br>1-2 times a week for 5-8 weeks
Pinduoduo	Good things are best	Battery restorers	4.56	13.00	3	The effect is obvious after 5-8 weeks	Pulse charge repair<br>life extension<br>maintenance<br>battery	Detection of current<br>voltage power consumption<br>heat dissipation<br>repair of mobile phone stuck<br>clean up mobile phone garbage	90%	Multi-device compatibility<br>mobile phone tablet	76 * 45 * 22	none	Battery repair for 3 hours<br>1-2 times a week for 5-8 weeks
Pinduoduo	Good things are best	Battery restorers	5.86	19.00	7	The effect is obvious after 5-8 weeks	Pulse charge repair<br>life extension<br>maintenance<br>battery	Detection of current<br>voltage power consumption<br>heat dissipation<br>repair of mobile phone stuck<br>clean up mobile phone garbage	100%	Multi-device compatibility<br>mobile phone tablet	76 * 45 * 22	none	Battery repair for 3 hours<br>1-2 times a week for 5-8 weeks
Pinduoduo	Good things are best	Battery restorers	9.28	10.00	0	none	Pulse charge repair<br>life extension<br>maintenance<br>battery	Detect current<br>voltage<br>High speed<br>low temperature repair<br>repair mobile phone stuck<br>clean up mobile phone garbage	0	Multi-device compatibility<br>mobile phone tablet	76 * 45 * 22	none	Battery repair for 3 hours<br>1-2 times a week for 5-8 weeks
Pinduoduo	No matter what<br>No matter what	Battery restorers	1.35	2360.00	665	none	Fix battery<br>extend life	Fix cell phone jams<br>Clean up cell phone trash	0	none	none	none	none
Pinduoduo	No matter what<br>No matter what	Battery restorers	12.88	1371.00	567	none	Fix battery extend life	Fix cell phone jams<br>Clean up cell phone trash	0	none	none	none	none
Pinduoduo	Everything Xing Technology	Battery repair artifact	9.98	1229.00	491	none	Activate<br>Repair battery<br>Extend life	Free up memory<br>fix cell phone stuck<br>Clean up cell phone trash<br>Lower radiation	0	Multi-device compatibility<br>mobile phone tablet	53 * 24 * 40	Intelligent Voice broadcasting	none
Pinduoduo	Everything Xing Technology	Battery repair artifact	19.7	446.00	165	none	Activate<br>Repair battery<br>Extend life	Free up memory<br>fix cell phone stuck<br>Clean up cell phone trash<br>Lower radiation	0	Multi-device compatibility<br>mobile phone tablet	53 * 24 * 40	Intelligent Voice broadcasting	none
Pinduoduo	Everything Xing Technology	Battery restorers	4.92	4558.00	1874	none	Fix battery<br>extend life	Detect current<br>voltage<br>High speed<br>low temperature repair<br>repair mobile phone stuck<br>clean up mobile phone garbage	0	Multi-device compatibility<br>mobile phone tablet	none	none	none
Pinduoduo	Everything Xing Technology	Battery restorers	6.62	251.00	20	none	Fix battery<br>extend life	Detect current<br>voltage<br>High speed<br>low temperature repair<br>repair mobile phone stuck<br>clean up mobile phone garbage	0	Multi-device compatibility<br>mobile phone tablet	none	none	none
Pinduoduo	Everything Xing Technology	Battery restorers	12.38	37.00	18	none	Fix battery<br>extend life	Real-time detection of current<br>voltage<br>high-speed<br>low temperature repair<br>repair of mobile phone jams<br>clean up mobile phone garbage	0	Multi-device compatibility<br>mobile phone tablet	none	none	none
Pinduoduo	Everything Xing Technology	Automatic repair battery artifact	52.02	96.00	67	none	Battery repair<br>Life extension<br>Multiple protection<br>maintenance	Detect the current voltage<br>fix the phone stuck<br>clean up the phone garbage<br>high heat dissipation	0	Multi-device compatibility<br>mobile phone tablet	6.6 * 24.7 * 11.	none	none
Pinduoduo	Everything Xing Technology	Automatic repair battery artifact	43.41	14.00	7	none	Battery repair<br>Life extension<br>Multiple protection<br>maintenance	Detect the current voltage<br>fix the phone stuck<br>clean up the phone garbage<br>high heat dissipation	0	Multi-device compatibility<br>mobile phone tablet	6.6 * 24.7 * 11.	none	none
Pinduoduo	Everything Xing Technology	Battery restorer	59.75	1179.00	150	Suitable for 2-wheeled battery cars	Battery<br>repair<br>maintenance<br>range<br>life extension<br>activation	Solve the battery is full at a charge<br>charging does not turn the lamp<br>battery expansion<br>insufficient uphill force<br>ionized water crystallization<br>deep cleaning<br>real-time monitoring of current<br>full stop	100%	EVS	none	none	none
Pinduoduo	Everything Xing Technology	Battery restorer	57.32	942.00	60	For 3-wheeled battery cars	Battery<br>repair<br>maintenance<br>range<br>life extension<br>activation	Solve the battery is full at a charge<br>charging does not turn the lamp<br>battery expansion<br>insufficient uphill force<br>ionized water crystallization<br>deep cleaning<br>real-time monitoring of current<br>full stop	1	EVS	none	none	none
Pinduoduo	Everything Xing Technology	Battery restorer	44.38	421.00	50	Suitable for 4-wheel battery car	Battery<br>repair<br>maintenance<br>range<br>life extension<br>activation	Solve the battery is full at a charge<br>charging does not turn the lamp<br>battery expansion<br>insufficient uphill force<br>ionized water crystallization<br>deep cleaning<br>real-time monitoring of current<br>full stop	1	EVS	none	none	none

Pinduoduo	Everything Xing Technology	Battery restorer	51.93	126.00	18	2023 New upgrade model	Charge<br>Repair<br>Maintenance<br>Activation	Detect current voltage<br>lithium battery disabled	0	EVS	160 * 180 * 100	none	none
Pinduoduo	UOHCGFA Digital Appliances Flagship store	Battery restorers	1.7	440.00	57	none	Charge<br>Repair<br>Maintenance	Detect current<br>voltage in real time<br>repair cell phone jams<br>clean up cell phone garbage	0	All cell phones	none	none	none
Pinduoduo	UOHCGFA Digital Appliances Flagship store	Battery restorers	4.95	74.00	21	none	Charge<br>Repair<br>Maintenance	Detect current<br>voltage in real time<br>repair cell phone jams<br>clean up cell phone garbage	0	All cell phones	none	none	none
Pinduoduo	UOHCGFA Digital Appliances Flagship store	Battery restorers	19.38	942.00	252	none	Charge<br>Repair<br>Maintenance	Detect current<br>voltage in real time<br>repair cell phone jams<br>clean up cell phone garbage	0	All cell phones	none	none	none
Pinduoduo	Fu Yi Living Hall	Phone adapter	22.01	38.00	0	none	Charge<br>Repair<br>Maintenance	Detect current<br>voltage<br>High speed<br>low temperature repair<br>repair mobile phone stuck<br>clean up mobile phone garbage	0	All cell phones	76 * 45 * 22	none	Battery repair for 3 hours<br>1-2 times a week for 5-8 weeks
Pinduoduo	Fu Yi Living Hall	Phone adapter	37	2966.00	0	none	Charge<br>Repair<br>Maintenance	Detect current<br>voltage<br>High speed<br>low temperature repair<br>repair mobile phone stuck<br>clean up mobile phone garbage	0	All cell phones	76 * 45 * 22	none	Battery repair for 3 hours<br>1-2 times a week for 5-8 weeks
Pinduoduo	Fu Yi Living Hall	Phone adapter	38.9	4975.00	0	none	Charge<br>Repair<br>Maintenance	Detect current<br>voltage<br>High speed<br>low temperature repair<br>repair mobile phone stuck<br>clean up mobile phone garbage	0	All cell phones	76 * 45 * 22	none	Battery repair for 3 hours<br>1-2 times a week for 5-8 weeks
Pinduoduo	Puning Mouth	Cell phone battery restorer	1.02	2058.00	433	none	Charge<br>Repair<br>Maintenance	Detect current<br>voltage<br>High speed<br>low temperature repair<br>repair mobile phone stuck<br>clean up mobile phone garbage	1%	All cell phones	none	none	none
Pinduoduo	Puning Mouth	Cell phone battery restorer	1.01	309.00	107	none	Charge<br>Repair<br>Maintenance	Detect current<br>voltage<br>High speed<br>low temperature repair<br>repair mobile phone stuck<br>clean up mobile phone garbage	60%	All cell phones	none	none	none
Pinduoduo	Puning Mouth	Cell phone battery restorer	10.08	21.00	4	none	Charge<br>Repair<br>Maintenance	Detect current<br>voltage<br>High speed<br>low temperature repair<br>repair mobile phone stuck<br>clean up mobile phone garbage	100%	All cell phones	none	none	none
Tiktok	Ming Shengda Digital	Battery restorers	28.8	1.00	1	none	Charge<br>Repair<br>Maintenance	Detect current<br>voltage<br>High speed<br>low temperature repair<br>repair mobile phone stuck<br>clean up mobile phone garbage	0	All cell phones	none	none	none
Tiktok	Ming Shengda Digital	Battery restorers	29.9	13.00	1	none	Charge<br>Repair<br>Maintenance	Detect current<br>voltage<br>High speed<br>low temperature repair<br>repair mobile phone stuck<br>clean up mobile phone garbage	1%	All cell phones	none	none	none
Tiktok	Ming Shengda Digital	Battery restorers	69.9	0.00	0	none	Charge<br>Repair<br>Maintenance	Detect current<br>voltage<br>High speed<br>low temperature repair<br>repair mobile phone stuck<br>clean up mobile phone garbage	100%	Android phone	none	none	none
Tiktok	Ming Shengda Digital	Battery restorers	89.9	0.00	0	none	Charge<br>Repair<br>Maintenance	Detect current<br>voltage<br>High speed<br>low temperature repair<br>repair mobile phone stuck<br>clean up mobile phone garbage	0	iPhone	none	none	none
Tiktok	A small individual shop of Lang Insurance Electronics firm in Chancheng district, Foshan City	Battery restorers	8.28	4.00	1	none	Battery<br>pulse repair<br>maintenance<br>life extension	Real-time detection of current voltage	1%	Mobile phones<br>tablets can be used	none	none	none
Tiktok	A small individual shop of Lang Insurance Electronics firm in Chancheng district, Foshan City	Battery restorers	14.4	2.00	1	none	Battery<br>pulse repair<br>maintenance<br>life extension	Real-time detection of current voltage	50%	Mobile phones<br>tablets can be used	none	none	none
Tiktok	A small individual shop of Lang Insurance Electronics firm in Chancheng district, Foshan City	Battery restorers	19.38	1.00	1	none	Battery<br>pulse repair<br>maintenance<br>life extension	Real-time detection of current voltage	100%	Mobile phones<br>tablets can be used	none	none	none

Tiktok	Victory Charge Digital flagship store	Battery restorers	7.9	38.00	8	none	Pulse Repair<br>Charging<br>Maintenance<br>Life extension	Automatic cleaning repair<br>repair cell phone stuck<br>deep cleaning garbage<br>check current voltage	0	Huawei<br>Apple<br>Xiaomi<br>oppo<br>vivo phone	76 * 45 * 22	none	Battery repair for 3 hours<br>2-3 times a week for 5-8 weeks
Tiktok	One Day Store	Battery restorers	4.01	24.00	1	none	Battery<br>pulse repair<br>maintenance<br>life extension	Detect current<br>voltage<br>High speed<br>low temperature repair<br>repair mobile phone stuck<br>clean up mobile phone garbage	10%	Multi-device compatibility<br>mobile phone tablet	76 * 45 * 22	none	Battery repair for 3 hours<br>2-3 times a week for 5-8 weeks
Tiktok	One Day Store	Battery restorers	8.9	15.00	1	none	Battery<br>pulse repair<br>maintenance<br>life extension	Detect current<br>voltage<br>High speed<br>low temperature repair<br>repair mobile phone stuck<br>clean up mobile phone garbage	50%	Multi-device compatibility<br>mobile phone tablet	76 * 45 * 22	none	Battery repair for 3 hours<br>2-3 times a week for 5-8 weeks
Tiktok	One Day Store	Battery restorers	11.9	15.00	1	none	Battery<br>pulse repair<br>maintenance<br>life extension	Detect current<br>voltage<br>High speed<br>low temperature repair<br>repair mobile phone stuck<br>clean up mobile phone garbage	100%	Multi-device compatibility<br>mobile phone tablet	76 * 45 * 22	none	Battery repair for 3 hours<br>2-3 times a week for 5-8 weeks
Tiktok	One Day Store	Battery restorers	14.8	10.00	1	none	Battery<br>pulse repair<br>maintenance<br>life extension	Detect current<br>voltage<br>High speed<br>low temperature repair<br>repair mobile phone stuck<br>clean up mobile phone garbage	200%	Multi-device compatibility<br>mobile phone tablet	76 * 45 * 22	none	Battery repair for 3 hours<br>2-3 times a week for 5-8 weeks
Tiktok	Yuan Bu Department Store	Battery restorers	9.9	5906.00	0	none	Battery<br>pulse repair<br>maintenance<br>life extension	Detect current<br>voltage<br>High speed<br>low temperature repair<br>repair mobile phone stuck<br>clean up mobile phone garbage	0	Multi-device compatibility<br>mobile phone tablet	none	none	none
Tiktok	Xinhua Yuanxuan	Tram adapter	20.8	2.00	1	none	Battery<br>Repair<br>Maintenance<br>Activation<br>Increased mileage	Fix cell phone jams<br>Deep trash cleaning<br>check current voltage<br>overcurrent<br>overload<br>overvoltage<br>high temperature<br>short circuit<br>interference protection	30%	Trolleys	none	none	none
Tiktok	Xinhua Yuanxuan	Tram adapter	31.9	1.00	1	none	Battery<br>Repair<br>Maintenance<br>Activation<br>Increased mileage	Fix cell phone jams<br>Deep trash cleaning<br>check current voltage<br>overcurrent<br>overload<br>overvoltage<br>high temperature<br>short circuit<br>interference protection	80%	Trolleys	none	none	none
Tiktok	Xinhua Yuanxuan	Phone adapter	21.9	9.00	0	none	Battery<br>pulse repair<br>maintenance<br>life extension	Detecting current voltage<br>fixing cell phone jams<br>Cleaning up cell phone trash	0	Huawei<br>Apple<br>Xiaomi<br>oppo<br>vivo phone	none	none	none
Tiktok	Xinhua Yuanxuan	Phone adapter	32.9	9.00	5	none	Battery<br>pulse repair<br>maintenance<br>life extension	Detecting current voltage<br>fixing cell phone jams<br>Cleaning up cell phone trash	0	Huawei<br>Apple<br>Xiaomi<br>oppo<br>vivo phone	none	none	none
Tiktok	Mumu select business	Air conditioning Adapter	14.7	2.00	0	No cents for bad results	Electrical repair<br>energy saving<br>voltage regulation<br>optimization circuit	Plug<br>play	68%	air conditioner.	none	none	none
Tiktok	Mumu select business	Air conditioning Adapter	34.58	1.00	0	No cents for bad results	Electrical repair<br>energy saving<br>voltage regulation<br>optimization circuit	Plugplay	128%	air conditioner.	none	none	none
Tiktok	Mumu select business	Air conditioning Adapter	54.52	1.00	0	No cents for bad results	Electrical repair<br>energy saving<br>voltage regulation<br>optimization circuit	Plugplay	168%	air conditioner.	none	none	none
Tiktok	Illusion straight supply	Battery restorers	19.9	7.00	0	none	Repair<br>Life extension	none	0	Huawei<br>Xiaomi<br>vivo<br>oppo Honor<br>Meizu<br>OnePlus	none	none	none
Tiktok	Illusion straight supply	Battery restorers	29.9	37.00	0	none	Battery<br>pulse repair<br>maintenance<br>life extension	Detecting current voltage<br>fixing cell phone jams<br>Cleaning up cell phone trash	0	Multi-device compatibility<br>mobile phone tablet	76 * 45 * 22	none	Battery repair for 3 hours<br>1-2 times a week for 5-8 weeks

JD.com	Hami Bedroom Furniture specialty store	Battery restorers	12	20.00	10	7 days no reason to return	Pulse repair<br>battery<br>maintenance<br>life extension	Check the current voltage<br>improve the battery efficiency<br>high heat dissipation<br>repair the mobile phone jam<br>clean the mobile phone garbage<br>release the memory of the mobile phone	0	Multi-device compatibility<br>mobile phone tablet	76 * 45 * 22	none	Battery repair for 3 hours<br>2-3 times a week for 5-8 weeks
JD.com	Hami Bedroom Furniture specialty store	Battery restorers	13.4	15.00	5	7 days no reason to return	Pulse repair<br>battery<br>maintenance<br>life extension	Check the current voltage<br>improve the battery efficiency<br>high heat dissipation<br>repair the mobile phone jam<br>clean the mobile phone garbage<br>release the memory of the mobile phone	600%	Multi-device compatibility<br>mobile phone tablet	76 * 45 * 22	none	Battery repair for 3 hours<br>2-3 times a week for 5-8 weeks
JD.com	Hami Bedroom Furniture specialty store	Battery restorers	13.4	10.00	3	7 days no reason to return	Pulse repair<br>battery<br>maintenance<br>life extension	Check the current voltage<br>improve the battery efficiency<br>high heat dissipation<br>repair the mobile phone jam<br>clean the mobile phone garbage<br>release the memory of the mobile phone	100%	Multi-device compatibility<br>mobile phone tablet	76 * 45 * 22	none	Battery repair for 3 hours<br>2-3 times a week for 5-8 weeks
JD.com	Hami Bedroom Furniture specialty store	Battery restorers	24	5.00	1	7 days no reason to return	Pulse repair<br>battery<br>maintenance<br>life extension	Check the current voltage<br>improve the battery efficiency<br>high heat dissipation<br>repair the mobile phone jam<br>clean the mobile phone garbage<br>release the memory of the mobile phone	500%	Multi-device compatibility<br>mobile phone tablet	76 * 45 * 22	none	Battery repair for 3 hours<br>2-3 times a week for 5-8 weeks
JD.com	Hami Bedroom Furniture specialty store	Battery restorers	100.8	0.00	0	7 days no reason to return	Pulse repair<br>battery<br>maintenance<br>life extension	Check the current voltage<br>improve the battery efficiency<br>high heat dissipation<br>repair the mobile phone jam<br>clean the mobile phone garbage<br>release the memory of the mobile phone	0	Multi-device compatibility<br>mobile phone tablet	76 * 45 * 22	none	Battery repair for 3 hours<br>2-3 times a week for 5-8 weeks
JD.com	Hami Bedroom Furniture specialty store	Smart pulse regulator for electric vehicles	89.6	12.00	9	Suitable for 2-wheeled battery cars	Repair the battery<br>prolong life<br>maintenance	Battery deep cleaning<br>real-time monitoring of current<br>full stop<br>low voltage<br>overcurrent<br>short circuit<br>overheat protection	100%	EVS	none	none	none
JD.com	Hami Bedroom Furniture specialty store	Smart pulse regulator for electric vehicles	102.2	5.00	1	For 3-wheeled battery cars	Repair the battery<br>prolong life<br>maintenance	Battery deep cleaning<br>real-time monitoring of current<br>full stop<br>low voltage<br>overcurrent<br>short circuit<br>overheat protection	1	EVS	none	none	none
JD.com	Hami Bedroom Furniture specialty store	Smart pulse regulator for electric vehicles	111.3	8.00	1	Suitable for 4-wheel battery car	Repair the battery<br>prolong life<br>maintenance	Battery deep cleaning<br>real-time monitoring of current<br>full stop<br>low voltage<br>overcurrent<br>short circuit<br>overheat protection	1	EVS	none	none	none
JD.com	Electric chief flagship store	Battery restorers	39.9	0.00	3	none	Charge<br>Pulse Repair<br>Maintenance	Real-time detection of current<br>voltage power consumption<br>repair of mobile phone jams<br>cleaning mobile phone garbage<br>high heat dissipation	0	Multi-device compatibility<br>mobile phone tablet	none	none	none
JD.com	Electric chief flagship store	Mobile phone battery small housekeeper	48.8	0.00	100	none	Charging<br>Pulse repair<br>Life extension	Real-time detection of current<br>voltage power consumption	0	Multi-device compatibility<br>mobile phone tablet	none	none	none
JD.com	Electric chief flagship store	Mobile phone battery small housekeeper	79	0.00	500	none	Maintenance<br>repair<br>life extension	Detect the current voltage<br>high heat dissipation<br>repair the mobile phone stuck<br>clean up the mobile phone rubbish	0	Multi-device compatibility<br>mobile phone tablet	none	Night Light	none
JD.com	Electric chief flagship store	Mobile phone battery small housekeeper	89	0.00	500	none	Maintenance<br>repair<br>life extension	Detect the current voltage<br>high heat dissipation<br>repair the mobile phone stuck<br>clean up the mobile phone rubbish	0	Multi-device compatibility<br>mobile phone tablet	none	Night Light	none
JD.com	Hejun Furniture Franchise store	Phone adapter	15.84	0.00	62	none	Charging<br>Pulse repair<br>Maintenance<br>Life extension	Detect the current voltage<br>high heat dissipation<br>repair the mobile phone stuck<br>clean up the mobile phone rubbish	600%	Multi-device compatibility<br>mobile phone tablet	76 * 45 * 22	none	Battery repair for 3 hours<br>2-3 times a week for 5-8 weeks
JD.com	Hejun Furniture Franchise store	Phone adapter	11	0.00	55	none	Repair the battery<br>prolong life<br>maintenance	Fix game stuttering<br>hair perm fever	0	Mobile phone watch<br>headphones are used	none	none	none
JD.com	Hejun Furniture Franchise store	Phone adapter	17.1	0.00	38	none	Charge<br>Pulse Repair<br>Maintenance	Real-time detection of current<br>voltage power consumption<br>repair of mobile phone jams<br>clean up mobile phone garbage	0	Multi-device compatibility<br>mobile phone tablet	none	none	none
JD.com	Hejun Furniture Franchise store	Phone adapter	23.39	0.00	62	none	Charging<br>Pulse repair<br>Maintenance<br>Life extension	Real-time detection of current<br>voltage power consumption<br>repair of mobile phone jams<br>clean up mobile phone garbage	0	Multi-device compatibility<br>mobile phone tablet	76 * 45 * 22	none	Battery repair for 3 hours<br>2-3 times a week for 5-8 weeks
JD.com	Hejun Furniture Franchise store	Air conditioning Adapter	35	0.00	200	none	Repair<br>power saving<br>voltage regulation<br>optimization circuit	none	0	air conditioner.	none	Save power<br>regulate voltage	none

JD.com	Samuel Electrician Shop	Multifunctional Restorer	68.1	0.00	29	none	Charging<br>Pulse repair<br>Maintenance<br>Life extension	Real-time detection of current<br>voltage power consumption<br>repair of mobile phone jams<br>cleaning mobile phone garbage<br>high heat dissipation	0	Compatible	none	Night Light	none
JD.com	Samuel Electrician Shop	Apple Cord + Fixer	77.1	0.00	29	none	Charging<br>Pulse repair<br>Maintenance<br>Life extension	Real-time detection of current<br>voltage power consumption<br>repair of mobile phone jams<br>cleaning mobile phone garbage<br>high heat dissipation	0	Compatible	none	none	none
JD.com	Samuel Electrician Shop	Mobile phone battery small housekeeper	43.92	0.00	3	none	Charge<br>Pulse Repair<br>Maintenance	Real-time detection of current<br>voltage power consumption<br>repair of mobile phone jams<br>cleaning mobile phone garbage<br>high heat dissipation	0	Multi-device compatibility<br>mobile phone tablet	none	none	none
JD.com	Lingfan Furniture Franchise	Smartphone restorer	6.9	956.00	567	none	Repair the battery<br>prolong life<br>maintenance	Real-time detection of current<br>voltage power consumption<br>repair of mobile phone stuck<br>clean up mobile phone garbage<br>high-speed<br>low temperature repair	0	Multi-device compatibility<br>mobile phone tablet	76 * 45 * 22	none	Battery repair for 3 hours<br>2-3 times a week for 5-8 weeks
JD.com	Lingfan Furniture Franchise	Smartphone restorer	12.5	356.00	256	none	Repair the battery<br>prolong life<br>maintenance	Real-time detection of current<br>voltage power consumption<br>repair of mobile phone stuck<br>clean up mobile phone garbage<br>high-speed<br>low temperature repair	0	Multi-device compatibility<br>mobile phone tablet	76 * 45 * 22	none	Battery repair for 3 hours<br>2-3 times a week for 5-8 weeks
JD.com	Lingfan Furniture Franchise	Smartphone restorer	15.3	153.00	213	none	Repair the battery<br>prolong life<br>maintenance	Real-time detection of current<br>voltage power consumption<br>repair of mobile phone stuck<br>clean up mobile phone garbage<br>high-speed<br>low temperature repair	0	Multi-device compatibility<br>mobile phone tablet	76 * 45 * 22	none	Battery repair for 3 hours<br>2-3 times a week for 5-8 weeks
JD.com	Lingfan Furniture Franchise	Air conditioning Adapter	17.5	0.00	0	none	Electrical repair<br>power saving<br>voltage regulation	Optimize appliances<br>plug<br>play	0	Appliances<br>Air conditioning	none	none	none
JD.com	Lingfan Furniture Franchise	Air conditioning Adapter	23.8	0.00	0	none	Electrical repair<br>power saving<br>voltage regulation	Optimize appliancesplugplay	0	AppliancesAir conditioning	none	none	none
JD.com	Lingfan Furniture Franchise	High quality phone restorer	31.9	0.00	0	none	Charge<br>Pulse Repair<br>Maintenance	Detect the current voltage<br>high heat dissipation<br>repair the mobile phone stuck<br>clean up the mobile phone garbage	0	Multi-device compatibility<br>mobile phone tablet	76 * 45 * 22	none	Battery repair for 3 hours<br>2-3 times a week for 5-8 weeks
JD.com	Lingfan Furniture Franchise	High quality phone restorer	47.3	0.00	0	none	Charge<br>Pulse Repair<br>Maintenance	Detect the current voltage<br>high heat dissipation<br>repair the mobile phone stuck<br>clean up the mobile phone garbage	0	Multi-device compatibility<br>mobile phone tablet	76 * 45 * 22	none	Battery repair for 3 hours<br>2-3 times a week for 5-8 weeks
JD.com	Lingfan Furniture Franchise	High quality phone restorer	62.7	0.00	0	none	Charge<br>Pulse Repair<br>Maintenance	Detect the current voltage<br>high heat dissipation<br>repair the mobile phone stuck<br>clean up the mobile phone garbage	0	Multi-device compatibility<br>mobile phone tablet	76 * 45 * 22	none	Battery repair for 3 hours<br>2-3 times a week for 5-8 weeks
JD.com	Lingfan Furniture Franchise	High quality phone restorer	78.1	0.00	0	none	Charging<br>Pulse repair<br>Maintenance<br>Life extension	Detect current voltage<br>high heat dissipation<br>Repair mobile phone stuck<br>Clean mobile phone garbage<br>release memory	0	Multi-device compatibility<br>mobile phone tablet	76 * 45 * 22	none	Battery repair for 3 hours<br>2-3 times a week for 5-8 weeks
JD.com	Lingfan Furniture Franchise	High quality phone restorer	93.5	0.00	0	none	Charging<br>Pulse repair<br>Maintenance<br>Life extension	Detect the current voltage<br>high heat dissipation<br>repair the mobile phone stuck<br>clean up the mobile phone garbage	0	Multi-device compatibility<br>mobile phone tablet	76 * 45 * 22	none	Battery repair for 3 hours<br>2-3 times a week for 5-8 weeks
JD.com	Lingfan Furniture Franchise	High quality phone restorer	108.9	0.00	0	none	Charging<br>Pulse repair<br>Maintenance<br>Life extension	Detect the current voltage<br>high heat dissipation<br>repair the mobile phone stuck<br>clean up the mobile phone garbage	0	Multi-device compatibility<br>mobile phone tablet	76 * 45 * 22	none	Battery repair for 3 hours<br>2-3 times a week for 5-8 weeks
JD.com	Lingfan Furniture Franchise	Automatic mobile phone repair device	10.8	0.00	1000	none	Charge<br>Pulse Repair<br>Maintenance	Detect the current voltage<br>high heat dissipation<br>repair the mobile phone stuck<br>clean up the mobile phone garbage	0	Multi-device compatibility<br>mobile phone tablet	76 * 45 * 22	none	Battery repair for 3 hours<br>2-3 times a week for 5-8 weeks
JD.com	Easy snow home<br>franchise store	Battery restorer	93.5	45.00	13	Suitable for 2-wheeled battery cars	Battery<br>repair<br>maintenance<br>range<br>life extension<br>activation	Solve the battery is full at a charge<br>charging does not turn the lamp<br>battery expansion<br>insufficient uphill force<br>ionized water crystallization<br>deep cleaning<br>real-time monitoring of current<br>full stop	100%	EVS	none	none	none
JD.com	Easy snow home<br>franchise store	Battery restorer	107.5	0.00	0	For 3-wheeled battery cars	Battery<br>repair<br>maintenance<br>range<br>life extension<br>activation	Solve the battery is full at a charge<br>charging does not turn the lamp<br>battery expansion<br>insufficient uphill force<br>ionized water crystallization<br>deep cleaning<br>real-time monitoring of current<br>full stop	1	EVS	none	none	none

JD.com	Easy snow home franchise store	Battery restorer	128.5	0.00	0	Suitable for 4-wheel battery car	Battery<br>repair<br>maintenance<br>range<br>life extension<br>activation	Solve the battery is full at a charge<br>charging does not turn the lamp<br>battery expansion<br>insufficient uphill force<br>ionized water crystallization<br>deep cleaning<br>real-time monitoring of current<br>full stop	1	EVS	none	none	none
JD.com	Han Ou Fei home building materials flagship store	Battery restorers	30	0.00	22	none	Charge<br>Pulse Repair<br>Maintenance	Detect the current voltage<br>high heat dissipation<br>repair the mobile phone stuck<br>clean up the mobile phone rubbish	0	Multi-device compatibility<br>mobile phone tablet	76 * 45 * 22	none	Battery repair for 3 hours<br>2-3 times a week for 5-8 weeks
JD.com	Xuanjin home textile franchise store	Phone adapter	63	11.00	2	none	Battery repair<br>life extension<br>maintenance	Real-time detection of current<br>voltage power consumption<br>repair of mobile phone jams<br>clean up mobile phone garbage	0	Multi-device compatibility<br>mobile phone tablet	none	none	none
JD.com	Xuanjin home textile franchise store	Phone adapter	66	5.00	1	none	Battery repair<br>life extension<br>maintenance	Real-time detection of current<br>voltage power consumption<br>repair of mobile phone jams<br>clean up mobile phone garbage	0	Multi-device compatibility<br>mobile phone tablet	none	none	none
JD.com	Xuanjin home textile franchise store	Phone adapter	66	3.00	1	none	Battery repair<br>life extension<br>maintenance	Real-time detection of current<br>voltage power consumption<br>repair of mobile phone jams<br>clean up mobile phone garbage	0	Multi-device compatibility<br>mobile phone tablet	none	none	none
JD.com	IKUN Daily Life Shop	Air conditioning Adapter	30.8	1534.00	500	90-day trial	Electrical repair<br>power saving<br>voltage regulation	Optimize appliances<br>plug<br>play	0	Special for air conditioning	none	none	none
JD.com	Lao Four small shop	Battery restorers	35.8	356.00	100	30 days no reason to return<br>100 days to replace 10 years free repair	Life extension<br>Battery repair<br>Fix Stuck<br>Clean up phone trash	Solve heat<br>fast power consumption<br>slow charging<br>check the current voltage	0	All cell phones	none	none	none
JD.com	Lao Four small shop	Battery restorers	28.8	45.00	2	90-day free trial<br>with 2-year free renewal lifetime service	Extend battery life<br>fix phone jams<br>check current voltage	Smooth game<br>speed up download<br>lower radiation<br>improve performance	0	Cell phone	none	none	none
JD.com	Monsisi's Little Shop	Air conditioning Adapter	28.8	59.00	13	60-day free trial	Electrical repair<br>power saving<br>voltage regulation	Optimize appliances<br>plug<br>play	0	Special for air conditioning	none	none	none
JD.com	Monsisi's Little Shop	Battery restorers	48	51.00	14	none	Life extension<br>Battery repair<br>Fix Stuck<br>Clean up phone trash	Solve heat<br>fast power consumption<br>slow charging<br>check the current voltage	0	All cell phones	none	none	none
JD.com	Monsisi's Little Shop	Battery restorers	69	125.00	62	none	Life extension<br>Battery repair<br>Charging	Solve heat<br>fast power consumption<br>slow charging<br>check the current voltage	0	All cell phones	none	none	none
JD.com	Little Orange Shop	Air conditioning Adapter	28.8	5.00	1	none	Electrical repair<br>power saving<br>voltage regulation	Optimize appliances<br>plug<br>play	0	Special for air conditioning	none	none	none
JD.com	Little Orange Shop	Battery restorers	28	178.00	49	30 days no reason to return<br>100 days to replace 10 years free repair	Charge<br>Repair<br>maintenance<br>Life extension	Smooth game<br>speed up download<br>lower radiation<br>improve performance	0	All cell phones	none	none	none
JD.com	Little Orange Shop	Battery restorers	31	39.00	13	none	Charging<br>Pulse repair<br>Maintenance<br>Life extension	Detect the current voltage<br>high heat dissipation<br>repair the mobile phone stuck<br>clean up the mobile phone rubbish	200%	Various digital products	none	none	none
