# Supplementary material for: Exploring online consumer behavior on fraudulent energy-saving products
Source: Sci Rep. 2024 Jun 21;14:14304. doi: 10.1038/s41598-024-65210-1 (PMC11192901; doi:10.1038/s41598-024-65210-1)
Supplement: Supplementary file 3 — Supplementary Table 3. [file 41598_2024_65210_MOESM3_ESM.pdf]

source	Merchant name	Product name	Price	Sales volume	Number of Reviews	Warranty Period	Parameters	Payload (W	Dimensions (mm)	Power saving rat	Range of use	Characteristics	Main features	Additional features
Taobao	Black technology electrician Electrical	Green Smart Appliance Saver	9.9	900	76	100 Day Trial	Material: flame retardant<br>voltage: 90-250V<br>frequency: 50-60Hz	120000	none	45%	Home commercial	On-site compensation<br>overload overpressure<br>protection	Power saving	none
Taobao	Black technology electrician Electrical	Green Smart Appliance Saver	9.9	46	2	90 day trial 180 days warranty	Material: flame retardant<br>voltage: 90-250V<br>frequency: 50-60Hz	120000	none	45.00%	Home commercial	Indicator light<br>overload overpressure<br>protection<br>on-site compensation	Power saving	none
Taobao	Sundian Technology	Smart Appliances	9.9	1000	89	Guaranteed for 10 years<br>buy 2 hair 3	Material: flame retardant<br>voltage: 90-250V<br>frequency: 50-60Hz	110000	87 * 57* 26	0	Home commercial	Screen control<br>overload overpressure<br>protection<br>local compensation	Power saving	Fire and lightning Protection
Pinduoduo	Wangxin Electronic digital	Concentrated Energy Province	11.8	5167	1229	none	none	none	none	0	Home commercial	Overpressure protection<br>local compensation	Power saving	none
Pinduoduo	Bajin Junjun	Appliance saving	12.8	50000	353	none	Material: flame retardant<br>voltage: 90-250V<br>frequency: 50-60Hz	none	none	0	Home commercial	Indicator light<br>overload overpressure<br>protection<br>overheat protection	Power saving	none
Pinduoduo	Shangru Appliances	Concentrated Energy Province	13.15	699	178	none	none	none	none	0	Home commercial	none	Power saving	none
Pinduoduo	Shangru Appliances	White intelligent electric saving	13.8	5	1	Unsatisfied no reason to return for a refund	Material: flame retardant<br>voltage: 90-250V<br>frequency: 50-60Hz	110000	82 * 55 * 20	45%	Home commercial	Overload overpressure<br>protection<br>in situ compensation	Power saving	none
Pinduoduo	Lai Xiaoxiao Boutique shop	Smart Butler	17.8	1338	303	none	none	none	none	0	Home commercial	none	Power saving	none
Pinduoduo	Lai Xiaoxiao Boutique shop	Smart Energy saving Steward	17.8	762	187	10 years warranty	Material: flame retardant<br>voltage: 90-250V<br>frequency: 50-60Hz	none	none	0	Home commercial	Screen control<br>overload overpressure<br>protection<br>local compensation	Power saving	Fire Protection
Pinduoduo	Lai Xiaoxiao Boutique shop	Appliance saving	18.8	9	1	90 day free trial warranty 5 years<br>replacement only without repair	Material: ABS flame retardant<br>voltage: 90-250V<br>frequency: 50-60Hz	28000	58 * 29* 98	0	Home commercial	Overload overpressure<br>protection<br>in situ compensation	Power saving	Protection of<br>electrical appliances
Pinduoduo	Lai Xiaoxiao Boutique shop	Automatic intelligent economizer	18.9	100	74	90-day free trial<br>2-year renewal<br>lifetime service	none	none	none	0	Home commercial	Voltage regulation<br>overload overpressure<br>protection<br>local compensation<br>indicator light	Power saving	Clean circuit
Taobao	Zhongyuan Pharmaceutical	Smart Province	19	73	23	100 day free trial 5 years<br>with 10 years warranty	Material: flame retardant<br>voltage: 90-250V<br>frequency: 50-60Hz	none	none	0	Home commercial	Voltage regulation<br>overload and overpressure<br>protection<br>active local compensation	Power saving	Leakage alarm<br>fireproof green without<br>radiation
Taobao	Zhongyuan Pharmaceutical	Appliance saving	19.8	10000	80	none	Material: flame retardant<br>voltage: 90-250V<br>frequency: 50-60Hz	none	none	0	Home commercial	Indicator light<br>overload overpressure<br>protection<br>overheat protection	Power saving	none
JD.com	Xuanjin Home Textile franchise store	Concentrated Energy Province	21	0	1	none	none	none	none	0	Home commercial	Screen control<br>overload overpressure<br>protection<br>local compensation	Power saving	Leakage alarm
JD.com	Xuanjin Home Textile franchise store	Automatic intelligent economizer	22.8	32	6	90-day free trial<br>2-year renewal<br>lifetime service	none	none	none	0	Home commercial	Stable voltage<br>overload overvoltage<br>protection	Power saving	Clean circuit<br>protect electrical appliances<br>green and low carbon
JD.com	Longlong good quality goods store	Green Smart Appliance Saver	22.97	0	1000	90 days free trial 5 years warranty for life	Material: flame retardant<br>voltage: 90-250V<br>frequency: 50-60Hz	150000	none	45%	Home commercial	Screen control<br>overload overpressure<br>protection<br>local compensation	Power saving	none
JD.com	Longlong good quality goods store	Appliance saving	23.5	1000	20	none	Material: flame retardant<br>voltage: 90-250V<br>frequency: 50-60Hz	none	none	0	Home commercial	Indicator light<br>overload overpressure<br>protection<br>overheat protection	Power saving	none
JD.com	IXCHSQ flagship store	Green Smart Appliance Saver	24.48	0	100	Useless<br>return	Material: flame retardant<br>voltage: 90-250V<br>frequency: 50-60Hz	110000	82 * 55 * 20	45%	Home commercial	Screen control<br>overload overpressure<br>protection<br>local compensation	Power saving	none
Tiktok	Longhua Creative Supply Chain	Smart Appliances	24.49	725	68	100-day 2-year trial with 5-year warranty	Material: flame retardant<br>voltage: 90-250V<br>frequency: 50-60Hz	110000	82 * 55 * 20	45%	Home commercial	Plug and play<br>voltage display	Power saving	none
Taobao	New Generation Science and Technology Museur	Dr Electricity	24.6	200	100	none	Material: ABS flame retardant<br>voltage: 90-250V<br>frequency: 50-60Hz	28000	58 * 29* 98	0	Home commercial	Screen control<br>overload overvoltage<br>protection<br>local compensation	Power saving	none
Taobao	New Generation Science and Technology Museur	On-site compensation saver	24.8	0	5	5 years warranty	Rated voltage 220V<br>frequency: 50-60Hz	none	98 * 58 * 29	0	Home commercial	Screen control<br>overload overpressure<br>protection<br>local compensation	Power saving	none
JD.com	Sidwei flagship store	Appliances Saver	25.8	0	100	none	Material: flame retardant<br>voltage: 90-250V<br>frequency: 50-60Hz	none	98 * 58 * 29	0	Home commercial	Voltage stabilization<br>life extension<br>Screen control<br>overload overpressure<br>protection<br>in-place compensation<br>overheat protection	Power saving	none
JD.com	Power Saver small shop	Green Smart Appliance Saver	26	0	82	A refund is guaranteed<br>if you don't save electricity	Material: ABS flame retardant<br>voltage: 90-250V<br>frequency: 50-60Hz	110000	57 * 87	40%	Home commercial	Screen control<br>overload overpressure<br>protection<br>reactive power compensation	Power saving	none
JD.com	Energy-saving technology shop	Green Smart Appliance Saver	26.18	0	32	90 days free trial 5 years warranty for life	Material: flame retardant<br>voltage: 90-250V<br>frequency: 50-60Hz	110000	57 * 87	40%	Home commercial	Screen control<br>overload overpressure<br>protection<br>local compensation	Power saving	none
JD.com	Energy-saving technology shop	White Smart Saver	26.18	0	7	90 days free trial 5 years warranty for life	Material: flame retardant<br>voltage: 90-250V<br>frequency: 50-60Hz	150000	82 * 55 * 20	45%	Home commercial	none	Power saving	none
JD.com	Energy-saving technology shop	On-site compensation saver	26.18	0	29	90 days free trial 5 years warranty for life	Material: ABS flame retardant<br>voltage: 90-250V<br>frequency: 50-60Hz	28000	98 * 58 * 29	0	Home commercial	Overheat protection<br>automatic power off	Power saving	none
Pinduoduo	Duxin Home Department Store	Local compensation economizer	26.8	990	365	Unsatisfied no reason to return for a refund	Material: flame retardant<br>voltage: 90-250V<br>frequency: 50-60Hz	28000	99 * 58 * 26	0	Home commercial	Indicator light<br>overload overpressure<br>protection<br>in-place compensation<br>overheat protection	Power saving	Radiation-free
Tiktok	Lao Fourth daily shop	Smart Appliances	26.8	22	3	90 days trial 5 years only change not repair	Material: flame retardant<br>voltage: 90-250V<br>frequency: 50-60Hz	150000	none	45%	Home commercial	none	Power saving	none

Tiktok	Lao Fourth daily shop	Smart Appliances	26.8	22	3	90 days trial 5 years only change not repair	Material: flame retardant<br>voltage: 90-250V<br>frequency: 50-60Hz	150000	none	45%	Home commercial	Plug and play<br>voltage display	Power saving	none
Taobao	Super Digital dedicated store	Appliance saving	27.6	4000	100	none	none	none	none	0	Home commercial	Overload overpressure protection<br>local compensation<br>indicator light	Power saving	none
Taobao	Super Digital dedicated store	Appliance saving	27.6	400	200	100-day free trial with 5-year warranty	Rated voltage 220V<br>frequency: 50-60Hz	none	59 * 98 * 32	0	Home commercial	none	Power saving	none
Tiktok	Quality U choose small shop	Smart Appliances	27.8	51	0	100 days trial 5 years guarantee	none	none	none	0	Home commercial	Plug and play<br>easy to operate	Power saving	none
Pinduoduo	Lubao Boutique	Appliance saving	27.9	3264	1162	Three years warranty	Voltage: 90-240V<br>frequency: 50-60Hz	10000	none	30%	Home commercial	One-button switch<br>with indicator light	Power saving	Noise-cancelling no radiation
Pinduoduo	Lubao Boutique	Appliance saving	28	65	13	90 day free trial warranty<br>5 years replacement only without repair	none	none	none	0	Home commercial	Overload and overpressure protection<br>active in situ compensation<br>indicator light	Power saving	none
Pinduoduo	Lubao Boutique	Smart Appliances	28	100	22	Free 120-day trial<br>full refund if invalid	Material: flame retardant<br>voltage: 90-250V<br>frequency: 50-60Hz	110000	82 * 55 * 20	45%	Home commercial	Voltage regulator<br>screen control<br>overload and overpressure protection<br>local compensation	Power saving	none
JD.com	Happy little Fan shop	Green Smart Appliance Saver	28.1	0	200	60 days refundable<br>2 years warranty	Material: flame retardant<br>voltage: 90-250V<br>frequency: 50-60Hz	110000	none	40%	Home commercial	none	Power saving	none
Taobao	Every day to explode single 8762	Green Smart Appliance Saver	28.8	300	35	90 day free trial	Material: ABS flame retardant<br>voltage: 90-250V<br>frequency: 50-60Hz	150000	57 * 87	45%	Home commercial	Overload overpressure protection<br>active on-site compensation	Power saving	none
Taobao	Every day to explode single 8762	Smart Appliances	28.8	1000	400	none	Material: flame retardant<br>voltage: 90-250V<br>frequency: 50-60Hz	150000	82 * 55 * 20	45%	Home commercial	Indicator light<br>overvoltage<br>overheat<br>short circuit<br>overcurrent protection	Power saving	none
Taobao	IXCHSQ flagship store	Appliance saving	28.8	6	3	Free 120-day trial<br>full refund if invalid	none	none	none	30%	Home commercial	Indicator light<br>screen control<br>overload and overpressure protection<br>on-site compensation	Power saving	none
Tiktok	Daily Good Garden Shop	Smart Appliances	28.8	32	1	120 days free trial 5 years<br>Replace only replace not repair	Material: high quality materials<br>voltage: 90-250V<br>frequency: 50-60Hz	none	82 * 55 * 20	0	Home commercial	Plug and play<br>voltage display	Power saving	none
Tiktok	Haruki Winter	Concentrated Energy Province	28.8	128	10	5 years warranty	none	none	none	0	Home commercial	Plug and play<br>easy to operate	Power saving	none
Tiktok	Haruki Winter	On-site compensation saver	28.8	0	0	90 day free trial with 5 year warranty	none	none	none	0	Home commercial	Overheat protection<br>automatic power off	Power saving	none
Tiktok	Haruki Winter	Local compensation economizer	29.9	15	6	none	Material: flame retardant<br>voltage: 90-250V<br>frequency: 50-60Hz	28000	58 * 99 *26	0	Home commercial	Voltage regulation<br>overload overpressure protection<br>local compensation<br>indicator light	Power saving	Silent no radiation
Taobao	Crooked Tech	Appliance saving	29.9	56	27	90 day free trial warranty 5 years replacement only without repair	Material: ABS flame retardant<br>voltage: 90-250V<br>frequency: 50-60Hz	28000	58 * 29* 98	0	Home commercial	Overheat protection<br>indicator light	Power saving	none
Taobao	Crooked Tech	Power saving expert	29.9	2	0	none	Material: Aluminum alloy<br>voltage: 90-250V<br>frequency: 50-60Hz	none	118 * 65 * 40	0	Home commercial	Screen control<br>overload overpressure protection<br>in-place compensation<br>independent fuse	Power saving	none
Taobao	Crooked Tech	On-site compensation saver	30	0	68	90 days free trial 5 years warranty for life	Material: ABS flame retardant<br>voltage: 90-250V<br>frequency: 50-60Hz	28000	98 * 58 * 29	0	Home commercial	Screen control<br>overload overpressure protection<br>on-site compensation<br>overheat protection automatic power off	Power saving	none
Taobao	Crooked Tech	Power Saver	30.9	2000	100	none	Material: Aluminum alloy<br>voltage: 90-250V<br>frequency: 50-60Hz	none	130 * 65 * 40	0	Home commercial	Screen control<br>overload overpressure protection<br>in-place compensation<br>independent fuse	Power saving	Air purification
Taobao	Crooked Tech	Ultrasonic economizer	31	0	1	Useless<br>return	none	none	none	0	Home commercial	none	Power saving	Leakage alarm voice control ultrasonic insecticidal wifi enhancement
Taobao	Crooked Tech	White Smart Saver	32.2	0	8	5 years warranty and refund	Material: flame retardant<br>voltage: 90-250V<br>frequency: 50-60Hz	150000	none	45%	Home commercial	Screen control<br>overload overpressure protection<br>local compensation	Power saving	none
Taobao	Crooked Tech	Appliances Saver	32.2	0	24	90 day free trial with 5 year warranty	none	none	none	0	Home commercial	none	Power saving	none
Taobao	Shindu Flagship store	Green Smart Appliance Saver	33.6	5000	800	Free 100-day trial	Material: flame retardant<br>voltage: 90-250V<br>frequency: 50-60Hz	110000	none	45%	Home commercial	Overvoltage<br>overload<br>overheat<br>overcurrent<br>short circuit protection<br>indicator light	Power saving	none
Tiktok	Piwanle Daily Store	Energy Saving King	33.8	49	5	5 years warranty for 180 day trial	none	none	98 * 58 * 29	0	Home commercial	Plug and play<br>overvoltage protection	Power saving	none
Tiktok	Piwanle Daily Store	Power saver	34	0	23	none	Material: flame retardant<br>voltage: 90-250V<br>frequency: 50-60Hz	none	none	0	Home commercial	none	Power saving	Leakage alarm voice control ultrasonic insecticidal wifi enhancement
Taobao	Sidway flagship store	Appliance saving	34.8	100	33	90 day free trial warranty 5 years replacement only without repair	none	none	none	0	Home commercial	Indicator light<br>screen control<br>overload overpressure protection<br>in place compensation<br>independent fuse	Power saving	none
Taobao	Sidway flagship store	Smart Appliances	34.8	6	0	none	Material: flame retardant<br>voltage: 90-250V<br>frequency: 50-60Hz	110000	82 * 55 * 20	45%	Home commercial	Screen control<br>overload overpressure protection<br>local compensation	Power saving	none
Taobao	Diyuan Electric flagship store	Green Smart Appliance Saver	34.8	29	9	Free 100-day trial<br>no reason to return for a refund if you are not satisfied	Material: flame retardant<br>voltage: 90-250V<br>frequency: 50-60Hz	110000	82 * 55 * 20	45%	Home commercial	none	Power saving	none

Taobao	Diyuan Electric flagship store	White intelligent electric saving	34.8	16	13	Dissatisfaction or refund	Material: flame retardant<br>voltage: 90-250V<br>frequency: 50-60Hz	150000	82 * 55 * 20	45%	Home commercial	Screen control<br>overload overpressure<br>protection<br>local compensation	Power saving	none
Taobao	Diyuan Electric flagship store	Appliance saving	34.8	22	6	Dissatisfaction or refund	Rated voltage: 220V<br>frequency: 50-60Hz	none	98 * * 32 59	0	Home commercial	Screen control<br>overload overpressure<br>protection<br>local compensation	Power saving	none
JD.com	Good luck again and again shop	Green Smart Appliance Saver	35	0	1000	60 days refundable<br>2 years warranty	Material: flame retardant<br>voltage: 90-250V<br>frequency: 50-60Hz	110000	none	40%	Home commercial	none	Power saving	none
JD.com	Good luck again and again shop	White Smart Saver	35	0	500	60 days refundable<br>2 years warranty	Material: flame retardant<br>voltage: 90-250V<br>frequency: 50-60Hz	150000	57 * 87	45%	Home commercial	Screen control<br>overload overpressure<br>protection<br>local compensation	Power saving	none
JD.com	Good luck again and again shop	Power Saver	35.6	3	1	Free trial	Material: Aluminum alloy<br>voltage: 90-250V<br>frequency: 50-60Hz	none	130 * 65 * 40	0	Home commercial	Screen control<br>overload overpressure<br>protection<br>in-place compensation<br>independent fuse	Power saving	Air purification
JD.com	Good luck again and again shop	Smart Appliances	35.8	100	68	30-day trial<br>2-year free renewal for 8-year life	Material: Aluminum alloy<br>voltage: 90-250V<br>frequency: 50-60Hz	1100000	110 * 35 * 60	0	Home commercial	Screen control<br>overload overpressure<br>protection<br>local compensation<br>independent safety tube	Power saving	none
Pinduoduo	Energy and appliance saving	Power Saver	35.94	4106	107	60-day trial with 2-year warranty	none	none	none	0	Home commercial	Screen control<br>overload overpressure<br>protection<br>local compensation<br>independent safety tube	Power saving	Air purification
Taobao	Jintianyuan Smart Home	Red power saving expert	36	100	500	60 day trial<br>2 year warranty	Material: Aluminum alloy<br>voltage: 90-250V<br>frequency: 50-60Hz	300000	130 * 65 * 40	0	Home commercial	Screen control<br>overload overpressure<br>protection<br>in-place compensation<br>independent fuse	Power saving	Air purification
Taobao	Jintianyuan Smart Home	Smart power saving	36	0	0	If it doesn't work<br>return it	none	none	none	0	Home commercial	Screen control<br>overload overpressure<br>protection<br>reactive power compensation	Power saving	Leakage alarm zero<br>noise zero radiation
Taobao	Jintianyuan Smart Home	Appliance saving	36.6	500	2000	90 day free trial warranty 5 years<br>replacement only without repair	Material: flame retardant<br>voltage: 90-250V<br>frequency: 50-60Hz	28000	58 * 29* 98	0	Home commercial	Overload overpressure<br>protection<br>active on-site compensation	Power saving	none
Taobao	Sru King Flagship store	Green Smart Appliance Saver	36.8	500	83	none	Material: flame retardant<br>voltage: 90-250V<br>frequency: 50-60Hz	none	none	0	Home commercial	none	Power saving	none
Taobao	Smart Appliances	Smart Appliances	36.8	85	20	none	Material: flame retardant<br>voltage: 90-250V<br>frequency: 50-60Hz	none	82 * 55 * 20	0	Home commercial	none	Power saving	none
Taobao	Smart Appliances	Smart economizer	38	300	24	none	Rated voltage: 220V<br>frequency: 50-60Hz	none	150 * 90 * 60	0	Home commercial	Screen control<br>overload overpressure<br>protection<br>local compensation<br>independent safety tube	Power saving	Leakage alarm no<br>noise no radiation
Taobao	Smart Appliances	White Smart Saver	38.8	0	11	180-day trial with 5-year warranty	Material: flame retardant<br>voltage: 90-250V<br>frequency: 50-60Hz	150000	none	45%	Home commercial	Screen control<br>overload overpressure<br>protection<br>local compensation	Power saving	none
Pinduoduo	Shuxin Daily Goods Store	Local compensation economizer	38.9	22	1	Unsatisfied no reason to return for a refund	Voltage: 90-250V<br>frequency: 50-60Hz	28000	99 * 58 * 26	0	Home commercial	Voltage regulator<br>indicator light<br>overload overpressure<br>protection<br>local compensation	Power saving	Silent no radiation
Taobao	Rong Tongcheng Technology	Power Saver	39	200	200	Free 90-day trial warranty for 5 years	Material: Metallic aluminum alloy	none	128 * 65 * 40	0	Home commercial	Voltage regulator<br>screen control<br>overload overpressure<br>protection<br>in-place compensation<br>independent fuse	Power saving	Clean circuit protection<br>appliances<br>air purification
Taobao	Rong Tongcheng Technology	Power-saving devices	39	10	38	60-day trial	Material: Aluminum alloy<br>voltage: 90-250V<br>frequency: 50-60Hz	90000	118 * 65 * 40	30%	Home commercial	Screen control<br>overload overpressure<br>protection<br>in-place compensation<br>independent fuse	Power saving	none
Taobao	Rong Tongcheng Technology	Red power saving expert	39.9	88	100	60 day free trial with 2-year warranty	Material: Aluminum alloy<br>voltage: 90-250V<br>frequency: 50-60Hz	300000	130 * 65 * 40	0	Home commercial	Screen control<br>overload overpressure<br>protection<br>local compensation<br>independent safety tube	Power saving	Air purification
Taobao	Rong Tongcheng Technology	Red power saving expert	39.9	21	14	60-day free trial with 3-year warranty	Material: Aluminum alloy case	none	none	0	Home commercial	Screen control<br>overload overpressure<br>protection<br>local compensation	Power saving	none
Taobao	Rong Tongcheng Technology	Power saving expert	40	0	6	1 year replacement	Material: Aluminum alloy<br>voltage: 90-250V<br>frequency: 50-60Hz	none	118 * 65 * 40	0	Home commercial	LCD<br>overload overpressure<br>protection<br>in situ compensation<br>individual fuse	Power saving	none
Taobao	Edison Tech Factory Store	Power Saver	41.8	200	82	60-day free trial 30-day<br>guarantee with 5-year warranty	Material: Aluminum alloy<br>voltage: 90-250V<br>frequency: 50-60Hz	none	130 * 65 * 40	30%	Home commercial	Screen control<br>overload overpressure<br>protection<br>in-place compensation<br>independent fuse	Power saving	Air purification
Taobao	Edison Tech Factory Store	King of Power Saving	42	0	92	Useless<br>return	Material: Aluminum alloy case	none	none	0	Home commercial	Liquid crystal display	Power saving	Air purification
Taobao	Edison Tech Factory Store	Local compensation economizer	42.6	30000	53	100 day free trial for 5 years warranty	Voltage: 110-260V<br>frequency: 50Hz	none	85 * 55 * 30	0	Home commercial	Indicator light<br>compensation in place	Power saving	none
Taobao	Edison Tech Factory Store	Smart Appliances	43.8	5000	500	100-day free trial with 5-year warranty	Voltage: 90-250V<br>frequency: 50-60Hz	none	59 * 98 * 32	0	Home commercial	none	Power saving	none
Taobao	Edison Tech Factory Store	Appliance saving	43.8	100	29	100-day free trial with 5-year warranty	Voltage: 90-250V<br>frequency: 50-60Hz	none	59 * 98 * 32	0	Home commercial	Indicator light<br>compensation in place	Power saving	none

Taobao	Edison Tech Factory Store	Smart power saver	43.8	1000	100	5 years to replace<br>buy 2 hair 3<br>lifetime warranty	Voltage: 90-250V<br>frequency: 50-60Hz	none	87 * 55 * 25	0	Home commercial	Visual screen	Power saving	none
Taobao	Edison Tech Factory Store	Appliance Saver	43.8	100	67	100-day free trial with 5-year warranty	Rated voltage: 220V<br>rated frequency: 50Hz	none	57 * 98 * 25	0	Home commercial	none	Power saving	none
Taobao	Edison Tech Factory Store	Smart Energy saving Steward	43.8	76	37	100 days free trial 5 years replacement<br>lifetime warranty	Rated voltage: 220V<br>frequency: 50-60Hz	none	none	0	Home commercial	none	Power saving	none
Taobao	Edison Tech Factory Store	Smart Appliances	45	200	29	Unsatisfied no reason to return for a refund	none	none	none	0	Home commercial	Screen control<br>overload and overpressure<br>protection	Power saving	Leakage alarm
Taobao	Edison Tech Factory Store	King of Power Saving	46	0	93	2 years warranty	Material: Aluminum alloy<br>voltage: 90-250V<br>frequency: 50-60Hz	none	130 * 65 * 40	0	Home commercial	Screen control<br>overload overpressure<br>protection<br>in-place compensation<br>individual fuse	Power saving	Air purification
Taobao	Edison Tech Factory Store	Smart power saving	46	0	0	If it doesn't work<br>return it	none	none	none	0	Home commercial	Screen control<br>overload overpressure<br>protection<br>reactive power compensation	Power saving	Leakage alarm zero<br>noise zero radiation
Pinduoduo	Ningde Kechuang Technology	Smart economizer	47	1014	35	365 day trial<br>10 year warranty	Rated voltage: 220V<br>rated frequency: 50Hz	60000	150 * 90 * 60	0	Home commercial	Reactive power compensation<br>low temperature work<br>without heating overload<br>protection	Power saving	Zero radiation/noise
Taobao	New technology of intelligent power saving	Power Saver	49	71	54	3 years warranty for 60 days trial	Material: Aluminum alloy<br>voltage: 90-250V<br>frequency: 50-60Hz	150000	130 * 65 * 40	30.00%	Home commercial	Screen control<br>overload overpressure<br>protection<br>in-place compensation<br>independent fuse	Power saving	Air purification
JD.com	Doraemon Store of Treasures	King of Power Saving	49.3	0	500	90 days no reason to return	Material: Aluminum alloy case	none	none	30%	Home commercial	Screen control<br>individual fuse	Power saving	none
JD.com	Doraemon Store of Treasures	Power Saver	49.9	700	600	90 day free trial 5 years warranty	Material: Aluminum alloy case	none	128 * 65 * 40	0	Home commercial	Screen control<br>overload overpressure<br>protection<br>local compensation	Power saving	Air purification<br>Clean the circuit
JD.com	Doraemon Store of Treasures	King of Power Saving	53.55	0	8	90 days no reason to return	Material: Aluminum alloy case	none	none	0	Home commercial	Screen control<br>overload overpressure<br>protection<br>in-place compensation<br>individual fuse	Power saving	Air purification
JD.com	Doraemon Store of Treasures	King of Power Saving	54.1	0	500	60 days refundable<br>3 years warranty	Material: Aluminum alloy case	none	none	30%	Home commercial	LCD<br>overload overpressure<br>protection<br>in situ compensation<br>individual fuse	Power saving	Air purification
JD.com	Doraemon Store of Treasures	Power Saver	55	86	27	Free renewal in 5 years	Material: Aluminum alloy<br>voltage: 90-250V<br>frequency: 50-60Hz	none	127 * 65 * 40	30%	Home commercial	Screen control<br>overload overpressure<br>protection<br>in-place compensation<br>independent fuse	Power saving	Air purification
JD.com	Doraemon Store of Treasures	King of Power Saving	55.8	0	200	5 year warranty	Material: Aluminum alloy case	none	107 * 65 * 40	0	Home commercial	Screen control<br>overload overpressure<br>protection<br>in-place compensation<br>individual fuse	Power saving	none
JD.com	Doraemon Store of Treasures	Power Saver	56	40	38	60 days trial 3 years free<br>replacement only replacement not repair	Material: Metal alloy	none	none	30%	Home commercial	Screen control<br>overload overpressure<br>protection<br>local compensation	Power saving	Air purification
Pinduoduo	Novi Energy Saving Technology	Smart economizer	56	3357	75	365 day trial<br>10 year warranty	none	none	none	0	Home commercial	none	Power saving	Leakage alarm
Pinduoduo	Novi Energy Saving Technology	King of Power Saving	57.8	0	200	120 day free trial	Material: Aluminum alloy<br>voltage: 90-250V<br>frequency: 50-60Hz	none	107 * 65 * 40	30%	Home commercial	Voltage regulator<br>screen control<br>overload overpressure<br>protection<br>in-place compensation<br>individual fuse	Power saving	Air purification
Pinduoduo	Novi Energy Saving Technology	Power Saver	58	14	9	90 days no reason to return or exchange	none	none	none	0	Home commercial	Screen control<br>overload and overpressure<br>protection	Power saving	Air purification
Pinduoduo	Novi Energy Saving Technology	Power Saver	58	46	12	Free 120-day trial<br>full refund if invalid	Material: Aluminum alloy<br>voltage: 90-250V<br>frequency: 50-60Hz	none	127 * 65 * 40	30%	Home commercial	Indicator light<br>screen control<br>individual fuse	Power saving	Air purification
Tiktok	Roselle commissary	Power Saver	58	2	0	Free 120-day trial with warranty	none	none	107 * 65 * 40	30%	Home commercial	Plug and play<br>voltage display	Power saving	Air purification
Tiktok	Roselle commissary	Power Saver	62	7	4	60 day trial<br>2 year warranty	Material: Aluminum alloy<br>voltage: 90-250V<br>frequency: 50-60Hz	300000	130 * 65 * 40	0	Home commercial	Voltage regulator<br>screen control<br>overload overpressure<br>protection<br>in-place compensation<br>independent fuse	Power saving	Air purification
Pinduoduo	Gao Lixin Technology	Smart economizer	62.51	1254	71	No power saved<br>return guaranteed	Material: Metal alloy	none	none	0	Home commercial	none	Power saving	none
Pinduoduo	Gao Lixin Technology	King of Power Saving	63	0	100	90 days no reason to return	Material: Aluminum alloy case	none	none	30%	Home commercial	none	Power saving	Air purification
Pinduoduo	Gao Lixin Technology	Power Saver	63.8	6	4	none	Material: Aluminum alloy<br>voltage: 90-250V<br>frequency: 50-60Hz	none	127 * 65 * 40	30%	Home commercial	Voltage regulator<br>indicator light<br>screen control<br>individual fuse	Power saving	Air purification
JD.com	Jinfeng Hongyuan flagship store	Power saving	67.15	0	20<br>000	60 days refundable<br>2 years warranty	Material: Aluminum alloy<br>voltage: 90-250V<br>frequency: 50-60Hz	98000	108 * 65	0	Home commercial	Screen control<br>individual safety tube	Power saving	none
JD.com	Jinfeng Hongyuan flagship store	Gold Power saving expert	68	11	6	Free 90-day trial warranty for 5 years	Material: Aluminum alloy<br>voltage: 90-250V<br>frequency: 50-60Hz	99000	110 * 41 * 68	0	Home commercial	Voltage regulator<br>screen control<br>overload overpressure<br>protection<br>in-place compensation<br>independent fuse	Power saving	Air purification
Taobao	Hida Smart	Gold Power saving expert	68	22	11	90-day free trial 5-year warranty<br>100-800° applicable	Material: Aluminum alloy<br>voltage: 90-250V<br>frequency: 50-60Hz	none	63 * 112	0	Home commercial	Screen control<br>overload overpressure<br>protection<br>local compensation	Power saving	none

Taobao	Hida Smart	Appliance saving	68	4000	2000	Free 90-day trial warranty for 5 years	Material: Aluminum alloy<br>voltage: 90-250V<br>frequency: 50-60Hz	150000	63 * 112	0	Home commercial	Screen control<br>overload overpressure<br>protection<br>local compensation	Power saving	none
Taobao	Hida Smart	Power savings throughout the house	68.6	1	1	none	Material: flame retardant	none	none	0	Home commercial	Screen control<br>overload overpressure<br>protection<br>local compensation<br>independent safety tube	Power saving	none
Taobao	Jinfeng Hongyuan flagship store	Huang Saving Electricity	69	8000	4000	60-day trial<br>2-year warranty<br>100-300° applicable	Material: Aluminum alloy<br>voltage: 90-250V<br>frequency: 50-60Hz	98000	65 * 108	0	Home commercial	Screen control<br>overload overpressure<br>protection<br>local compensation<br>independent safety tube	Power saving	none
Taobao	Jinfeng Hongyuan flagship store	Violet Power Saving expert	69	99	48	60 day trial<br>2 years warranty	Material: Aluminum alloy<br>voltage: 90-250V<br>frequency: 50-60Hz	98000	135 * 68 * 41	0	Home commercial	Screen control<br>overload overpressure<br>protection<br>local compensation<br>independent safety tube	Power saving	none
Taobao	Jinfeng Hongyuan flagship store	Power Saver	69	100	100	90 day free trial Warranty 2<br>years replacement only no repair	Material: Aluminum alloy<br>voltage: 90-250V	300000	110 * 40 * 70	32.50%	Home commercial	Screen control<br>overload overpressure<br>protection<br>local compensation<br>independent safety tube	Power saving	Air purification
Taobao	Bon Union Digital Code franchise	Gold Power saving expert	69	100	66	none	Material: Aluminum alloy case	none	none	0	Home commercial	Indicator light<br>screen control<br>individual fuse	Power saving	none
Taobao	Bon Union Digital Code franchise	Gold Power saving expert	76	800	200	5 years with a 90-day return guarantee	Material: Aluminum alloy<br>voltage: 90-250V<br>frequency: 50-60Hz	none	110 * 41 * 64	0	Home commercial	Voltage regulation<br>screen control<br>overload overpressure<br>protection<br>local compensation<br>independent safety tube	Power saving	Air purification
Taobao	Bon Union Digital Code franchise	Smart economizer	78	22	2	365 days trial<br>5 years warranty	none	none	none	0	Home commercial	Screen control<br>overload overpressure<br>protection<br>local compensation<br>independent safety tube	Power saving	none
Taobao	Bon Union Digital Code franchise	Power saving expert	78	29	8	60-day trial with 2-year warranty	Material: Aluminum alloy<br>voltage: 90-250V<br>frequency: 50-60Hz	none	110 * 40 * 65	0	Home commercial	Voltage regulator<br>screen control<br>overload and overpressure<br>protection<br>local compensation	Power saving	Clean circuit
Taobao	Bon Union Digital Code franchise	Intelligent power saving expert	78	0	1	60-day trial with 2-year renewal	Material: Aluminum alloy<br>voltage: 90-250V<br>frequency: 50-60Hz	none	none	0	Home commercial	LCD screen display	Power saving	none
Pinduoduo	Mustang Overseas Technology	Power Saver	78.98	2631	612	none	Material: Aluminum alloy<br>voltage: 90-250V<br>frequency: 50-60Hz	none	130 * 40 * 65	0	Home commercial	Screen control<br>overload overpressure<br>protection<br>in-place compensation<br>independent fuse	Power saving	Air purification
Pinduoduo	Mustang Overseas Technology	Power saving expert	79.1	0	500	60-day trial with 2-year warranty	Material: Metal alloy<br>voltage: 90-250V<br>frequency: 50-60Hz	98000	none	0	Home commercial	Screen control<br>individual fuse	Power saving	none
Pinduoduo	Mustang Overseas Technology	Smart economizer	82	2000	1000	90 day free trial 5 years warranty	Material: Aluminum alloy<br>voltage: 90-250V<br>frequency: 50-60Hz	300000	110 * 41 * 68	0	Home commercial	Voltage regulator<br>screen control<br>overload and overpressure<br>protection<br>local compensation	Power saving	Leakage alarm clean circuit
Pinduoduo	Mustang Overseas Technology	Gold Power saving expert	88	9	3	Free 90-day trial warranty for 5 years	Material: Aluminum alloy<br>voltage: 90-250V<br>frequency: 50-60Hz	none	110 * 41 * 64	0	Home commercial	Screen control<br>overload overpressure<br>protection<br>local compensation	Power saving	Air purification
Pinduoduo	Mustang Overseas Technology	Power Saver	88	15	9	none	Material: Aluminum alloy case	none	128 * 65 * 40	30%	Home commercial	Voltage regulator<br>screen control<br>overload overpressure<br>protection<br>in-place compensation<br>independent fuse	Power saving	Air purification
Pinduoduo	Mustang Overseas Technology	Power Saver	88	100	200	Free 90-day trial warranty for 5 years	Material: Aluminum alloy case	none	none	0	Home commercial	Screen control<br>overload overpressure<br>protection<br>in-place compensation<br>independent fuse	Power saving	Air purification
Pinduoduo	Mustang Overseas Technology	Platinum Power Saving Expert	89	64	22	60-day trial 2-year warranty<br>send 5 insurance tubes<br>500-800° applicable	Material: Aluminum alloy<br>voltage: 90-250V<br>frequency: 50-60Hz	99000	108 * 63 * 38	35%	Home commercial	Screen control<br>separate fuse	Power saving	none
Pinduoduo	Mustang Overseas Technology	Lake Blue Power saving expert	89	26	14	60 day trial<br>2 years warranty	Material: Aluminum alloy<br>voltage: 90-250V<br>frequency: 50-60Hz	98000	108 * 63 * 38	0	Home commercial	Screen control<br>independent safety tube	Power saving	none
Pinduoduo	Mustang Overseas Technology	Black Power Saving expert	89	5	4	none	Material: Aluminum alloy<br>voltage: 90-250V<br>frequency: 50-60Hz		150 * 95	0	Home commercial	Screen control<br>overheat and overload<br>protection<br>on-site compensation	Power saving	Leakage/overload alarm
Pinduoduo	Mustang Overseas Technology	Intelligent power saving expert	93	0	0	60-day trial with 3-year renewal	Material: Aluminum alloy<br>voltage: 90-250V<br>frequency: 50-60Hz	none	none	0	Home commercial	LCD screen display	Power saving	none
Pinduoduo	Mustang Overseas Technology	Intelligent frequency conversion high power	96.6	4106	0	none	none	none	none	0	Home commercial	none	Power saving	Leakage overload alarm
Pinduoduo	Mustang Overseas Technology	Intelligent power saving expert	97	0	0	60 days trial<br>4 years warranty	Material: Aluminum alloy<br>voltage: 90-250V<br>frequency: 50-60Hz	none	none	0	Home commercial	LCD screen display	Power saving	none
Pinduoduo	Zichen Technology Digital	Electric Officer	98	5	0	60-day trial<br>3-year warranty	Material: Metal alloy<br>voltage: 90-250V<br>frequency: 50-60Hz	110000	110 * 40 * 65	0	Home commercial	Screen control<br>overload overpressure<br>protection<br>local compensation<br>independent safety tube	Power saving	Air purification
Pinduoduo	Zichen Technology Digital	Love home intelligent energy-saving applianc	99	100	17	60-day free trial with 5-year warranty	none	none	none	0	Home commercial	none	Power saving	none
Pinduoduo	Zichen Technology Digital	Power saving	105.2	0	500	60 days refundable<br>3 years warranty<br>5-8 years life	Material: Metal alloy<br>voltage: 90-250V<br>frequency: 50-60Hz	99000	110 * 40 * 65	0	Home commercial	Screen control<br>overload overpressure<br>protection<br>in-place compensation<br>individual fuse	Power saving	none

Pinduoduo	Zichen Technology Digital	Light blue saves electricity	108	2000	1000	60-day trial<br>2-year warranty<br>300-500° applicable	Material: Aluminum alloy<br>voltage: 90-250V<br>frequency: 50-60Hz	99000	68 * 41 * 110	0	Home commercial	Screen control<br>overload overpressure protection<br>in-place compensation<br>independent fuse	Power saving	none
Pinduoduo	Zichen Technology Digital	(Three phase) Electrical officer	109	2	2	Warranty 5 years	Material: Aluminum alloy<br>voltage: 110-250V<br>frequency: 50-60Hz	none	190 * 120 * 55	0	Home commercial	Individual fuse	Power saving	none
Pinduoduo	Zichen Technology Digital	High power super economizer	109	0	0	90-day free trial	Material: Aluminum alloy<br>voltage: 90-250V<br>frequency: 50-60Hz	none	none	0	Home commercial	Voltage stabilization<br>variable frequency<br>consumption reduction<br>reactive power compensation	Power saving	Leakage alarm<br>short circuit automatic stop
Pinduoduo	Zichen Technology Digital	Power Saver	128	1000	100	90 day free trial 5 years warranty	Material: Aluminum alloy case	none	none	0	Home commercial	Screen monitoring<br>overload and overpressure protection<br>independent fuse<br>local compensation	Power saving	none
Pinduoduo	Zichen Technology Digital	Blue Power Saving Expert	128	69	49	90 day free trial 5 year warranty<br>100-1500° applicable	Material: Aluminum alloy case	none	177 * 110 * 52	0	Home commercial	Voltage regulation<br>screen control<br>overload overpressure protection<br>local compensation<br>independent safety tube	Power saving	Air purification leakage alarm Voice control ultrasonic insecticide
Pinduoduo	Zichen Technology Digital	Deep Blue Power saving expert	129	200	100	90 days free trial 5 years warranty<br>send 5 fuse	Material: Aluminum alloy<br>voltage: 90-250V<br>frequency: 50-60Hz	14000	177 * 110 * 52	0	Home commercial	Screen control<br>overload overpressure protection<br>local compensation<br>independent safety tube	Power saving	Air purification
Taobao	Pinsheng Electronic Technology	Power Saver	138	10000	800	90-day free trial with 3-year renewal	Material: Aluminum alloy<br>voltage: 90-250V<br>frequency: 50-60Hz	98000	135 * 68 * 41	0	Home commercial	Screen control<br>overload overpressure protection<br>local compensation<br>independent safety tube	Power saving	none
Taobao	Pinsheng Electronic Technology	Blue Power Saving Expert	139	700	300	90-day free trial Warranty 5 years<br>100-1800° applicable	Material: Aluminum alloy<br>voltage: 90-250V<br>frequency: 50-60Hz	300000	145 * 95 * 55	0	Home commercial	Screen control<br>overload overpressure protection<br>local compensation<br>independent safety tube	Power saving	Air purification leakage alarm Voice control ultrasonic insecticide
Taobao	Pinsheng Electronic Technology	Deep Blue Power saving expert	148	400	100	60-day trial 2-year warranty<br>send 5 insurance tubes<br>500-800° applicable	Material: Aluminum alloy<br>voltage: 90-250V<br>frequency: 50-60Hz	none	57 * 94 * 145	0	Home commercial	Screen control<br>overload overpressure protection<br>in-place compensation<br>independent fuse	Power saving	none
Taobao	Pinsheng Electronic Technology	Electric Butler	153	2	0	Lifetime guarantee<br>10 for every fake	none	none	none	30%	Home commercial	none	Power saving	none
Taobao	Pinsheng Electronic Technology	Gold Power saving expert	158	32	23	60 day trial<br>2 year warranty	Material: Aluminum alloy<br>voltage: 90-250V<br>frequency: 50-60Hz	110000	96 * 145 * 56	0	Home commercial	Screen control<br>overload overpressure protection<br>in-place compensation<br>independent fuse	Power saving	none
Taobao	Enogre Specialty Store	Copper power saving specialist	158	800	500	100-500° applies	Material: Aluminum alloy<br>voltage: 90-250V<br>frequency: 50-60Hz	110000	55 * 135 * 95	0	Home commercial	Indicator light<br>screen control<br>overload overpressure protection<br>in place compensation<br>independent fuse	Power saving	Cooling<br>cleaning to save electricity
Taobao	Enogre Specialty Store	Blue Power Saving Expert	158	800	400	60 day trial<br>2 year warranty	Material: Aluminum alloy<br>voltage: 90-250V<br>frequency: 50-60Hz	110000	145 * 94 * 57	0	Home commercial	Indicator light<br>screen control<br>overload overpressure protection<br>in place compensation<br>independent fuse	Power saving	none
Taobao	Enogre Specialty Store	Power saving expert	159.2	0	200	60-day trial<br>2-year warranty<br>5-8 years	Material: Metal alloy<br>voltage: 90-250V<br>frequency: 50-60Hz	110000	145 * 96 * 56	0	Home commercial	Screen control<br>overload overpressure protection<br>in-place compensation<br>individual fuse	Power saving	none
Taobao	Enogre Specialty Store	Electric Officer	159.8	2	0	none	none	none	none	0	Home commercial	Screen control<br>overload and overpressure protection<br>local compensation<br>independent safety tube	Power saving	Leakage alarm
Taobao	Enogre Specialty Store	Power Saver	168	2000	300	none	none	none	none	0	Home commercial	Screen control	Power saving	none
Taobao	Enogre Specialty Store	Power Saver	168	58	40	none	Material: Aluminum alloy<br>voltage: 90-250V<br>frequency: 50-60Hz	none	127 * 65 * 40	30%	Home commercial	Voltage regulator<br>indicator light<br>screen control<br>individual fuse	Power saving	Air purification
Taobao	Enogre Specialty Store	High power super power saving	188	1	0	365 days trial 10 years for new lifetime warranty<br>send 5 insurance pipe 2 hooks	none	none	none	0	Home commercial	Screen control<br>voltage visible	Power saving	Leakage alarm
Taobao	Enogre Specialty Store	Deep blue saves electricity	218	100	69	60 day trial<br>2 years warranty	Material: Aluminum alloy<br>voltage: 90-250V<br>frequency: 50-60Hz	none	80 * 41 * 120	0	Home commercial	Screen control<br>overvoltage protection<br>in-place compensation<br>individual fuse	Power saving	none
Taobao	Enogre Specialty Store	Red power saving expert	218	16	9	60 days refundable<br>2 years warranty<br>800-1400° applicable	Material: Metal alloy<br>voltage: 90-250V<br>frequency: 50-60Hz	none	80 * 41 * 120	0	Home commercial	Screen control<br>overload overpressure protection<br>in-place compensation<br>independent fuse	Power saving	none
