# Supplementary material for: Exploring online consumer behavior on fraudulent energy-saving products
Source: Sci Rep. 2024 Jun 21;14:14304. doi: 10.1038/s41598-024-65210-1 (PMC11192901; doi:10.1038/s41598-024-65210-1)
Supplement: Supplementary file 6 — Supplementary Information 1. [file 41598_2024_65210_MOESM6_ESM.pdf]

Supplementary Table 1: Text Clustering Data

This data table primarily illustrates the types, functions, and applicable uses of common fraudulent energy-saving products. By performing text clustering on the collected descriptions of these products, we identified two optimal clusters, referred to as Class A and Class B products.
