# Supplementary material for: Exploring online consumer behavior on fraudulent energy-saving products
Source: Sci Rep. 2024 Jun 21;14:14304. doi: 10.1038/s41598-024-65210-1 (PMC11192901; doi:10.1038/s41598-024-65210-1)
Supplement: Supplementary file 7 — Supplementary Information 2. [file 41598_2024_65210_MOESM7_ESM.pdf]

Supplementary Table 2: Sales Data of Class A Products

Class A products encompass various types of economizers designed for use in building circuits, aiming to reduce the energy consumption of the circuits themselves and the connected equipment. This dataset provides insights into the advantages, functions, features, and applications of Class A products. It reflects consumer preferences, user needs, and energy-saving intentions, thereby supporting the research on energy conservation behaviors.
