# Supplementary material for: Exploring online consumer behavior on fraudulent energy-saving products
Source: Sci Rep. 2024 Jun 21;14:14304. doi: 10.1038/s41598-024-65210-1 (PMC11192901; doi:10.1038/s41598-024-65210-1)
Supplement: Supplementary file 8 — Supplementary Information 3. [file 41598_2024_65210_MOESM8_ESM.pdf]

Supplementary Table 3: Sales Data of Class B Products

Class B products include a range of mobile phone air conditioning energy-saving devices. These products are primarily used for electrical repair or to extend battery life and reduce the energy consumption of high-power items. This dataset offers information on the advantages, functions, characteristics, and uses of Class B products. Consistent with the survey results of Category A consumers, this data indicates a preference for comprehensible energy-saving principles and user-friendly products, thus informing research on energy conservation intentions.
