# Supplementary material for: Exploring online consumer behavior on fraudulent energy-saving products
Source: Sci Rep. 2024 Jun 21;14:14304. doi: 10.1038/s41598-024-65210-1 (PMC11192901; doi:10.1038/s41598-024-65210-1)
Supplement: Supplementary file 9 — Supplementary Information 4. [file 41598_2024_65210_MOESM9_ESM.pdf]

Supplementary Table 4: Class A Product Evaluations

User evaluations of Class A products encompass experiences with various energy-saving appliances. These evaluations, based on personal practical experience and psychological expectations, provide potential consumers with real and reliable product information. This data helps consumers understand the actual performance, energy-saving effects, and potential issues of the products, enabling more informed purchasing decisions. As consumers expect these products to deliver tangible energy-saving benefits, they are more likely to purchase and use them, thereby promoting societal energy conservation and emission reduction. The evaluation data also reflect consumer intentions and consumption trends, which are valuable for researchers, while enhancing consumer awareness of energy conservation.
