# Supplementary material for: Exploring online consumer behavior on fraudulent energy-saving products
Source: Sci Rep. 2024 Jun 21;14:14304. doi: 10.1038/s41598-024-65210-1 (PMC11192901; doi:10.1038/s41598-024-65210-1)
Supplement: Supplementary file 10 — Supplementary Information 5. [file 41598_2024_65210_MOESM10_ESM.pdf]

Supplementary Table 5: Class B Product Evaluations

User evaluations of Class B products include experiences with various mobile phone air conditioning energy-saving devices. These evaluations, similarly based on personal practical experience and psychological expectations, provide authentic and reliable product information for potential consumers. This information aids consumers in understanding the actual performance, energy-saving effects, and potential problems of the products, facilitating more informed purchasing decisions. Given the expectation that these products will yield actual energy-saving results, consumers are more inclined to buy and use them, thus advancing the process of energy conservation and emission reduction across society. The evaluation data offer insights into consumer intentions and consumption trends for researchers, while also improving consumer awareness of energy conservation.
