# Supplementary material for: Exploring online consumer behavior on fraudulent energy-saving products
Source: Sci Rep. 2024 Jun 21;14:14304. doi: 10.1038/s41598-024-65210-1 (PMC11192901; doi:10.1038/s41598-024-65210-1)
Supplement: Supplementary file 5 — Supplementary Table 5. [file 41598_2024_65210_MOESM5_ESM.pdf]

Store Name	Product Name	Review	Source
Overseas high-end care	Battery Reconditioner	It's no good.	Taobao Marketplace, a Chinese website for online shopping
Overseas high-end care	Battery Reconditioner	Arrived quickly, well packaged and works great!	Taobao Marketplace, a Chinese website for online shopping
Overseas high-end care	Battery Reconditioner	Things have received yet to use it hope that the quality of the drop exactly the same as the Internet said after the use of it to come back!	Taobao Marketplace, a Chinese website for online shopping
Overseas high-end care	Battery Reconditioner	Tried it at home and it did repair the cell phone battery.	Taobao Marketplace, a Chinese website for online shopping
Overseas high-end care	Battery Reconditioner	Well packed, logistics and delivery are satisfactory. The battery restorer has been used for a while and feels good.	Taobao Marketplace, a Chinese website for online shopping
Overseas high-end care	Battery Reconditioner	Oh, yeah.	Taobao Marketplace, a Chinese website for online shopping
Overseas high-end care	Battery Reconditioner	Realistically, after using it a few times, the charging time has become exceptionally slow; it was full in half an hour or so, but now it won't charge in an hour, it takes about two hours. Other than that, I don't feel much.	Taobao Marketplace, a Chinese website for online shopping
Overseas high-end care	Battery Reconditioner	It's not very effective in practice.	Taobao Marketplace, a Chinese website for online shopping
Overseas high-end care	Battery Reconditioner	It's so bad, it's been broken for days	Taobao Marketplace, a Chinese website for online shopping
Overseas high-end care	Battery Reconditioner	The quality is very poor, the skewer comes loose after a few days of use, and there is no repair function at all, cheating!	Taobao Marketplace, a Chinese website for online shopping
Overseas high-end care	Battery Reconditioner	Charging is too slow.	Taobao Marketplace, a Chinese website for online shopping
Overseas high-end care	Battery Reconditioner	I received it early and am trying it out.	Taobao Marketplace, a Chinese website for online shopping
Overseas high-end care	Battery Reconditioner	same as described, wait for the use of a period of time to comment.	Taobao Marketplace, a Chinese website for online shopping
Overseas high-end care	Battery Reconditioner	Cover goods size detained large, use rain or very good, baby received good very satisfied, the boss is also very good waterproof file phoenix effect. Very good! Good quality and cheap, next time also come to this buy.	Taobao Marketplace, a Chinese website for online shopping
Overseas high-end care	Battery Reconditioner	not clear; I am now fooled, it is better to white has searched for how to brush brush, brush out of it is a new phone ！！！！！！	Taobao Marketplace, a Chinese website for online shopping
Overseas high-end care	Battery Reconditioner	Feed cell stable feed Cell phone battery also not hot I saw the internal circuit board and other $4 a board the same Seller should send the internal board diagrams description really imported parts?	Taobao Marketplace, a Chinese website for online shopping
Overseas high-end care	Battery Reconditioner	Only one day after the arrival of the goods, according to the requirements of the phone allowed electricity, seems to be a little effect, but not very obvious, and then follow up later. Fast logistics.	Taobao Marketplace, a Chinese website for online shopping
Overseas high-end care	Battery Reconditioner	The goods have been received! I don't know how well it works. Logistics is also very fast! It's still beautiful.	Taobao Marketplace, a Chinese website for online shopping
Overseas high-end care	Battery Reconditioner	It won't solve anything it's just a fast charger	Taobao Marketplace, a Chinese website for online shopping
Overseas high-end care	Battery Reconditioner	The merchant was quick to ship the item! The service attitude is also very enthusiastic! After using this cell phone repairer, the cell phone really fast network speed is also fast not jamming yo ！！！！	Taobao Marketplace, a Chinese website for online shopping
Overseas high-end care	Battery Reconditioner	Not a good picture.	Taobao Marketplace, a Chinese website for online shopping
Overseas high-end care	Battery Reconditioner	IQ tax!	Taobao Marketplace, a Chinese website for online shopping
Overseas high-end care	Battery Reconditioner	Customer service is very patient and good attitude, already in use, looking forward to good results!	Taobao Marketplace, a Chinese website for online shopping
Overseas high-end care	Battery Reconditioner	Fast shipping and happy with the quality.	Taobao Marketplace, a Chinese website for online shopping
Overseas high-end care	Battery Reconditioner	Copy that. It's good.	Taobao Marketplace, a Chinese website for online shopping
Overseas high-end care	Battery Reconditioner	This****isall****-free	Taobao Marketplace, a Chinese website for online shopping
Overseas high-end care	Battery Reconditioner	The logistics is very fast the phone is on very satisfied	Taobao Marketplace, a Chinese website for online shopping
Overseas high-end care	Battery Reconditioner	Baby received, used the second good use, before the flush a night are not rinsed, now six or seven hours on the full flush	Taobao Marketplace, a Chinese website for online shopping
Overseas high-end care	Battery Reconditioner	the more you use it, the more stuck it gets	Taobao Marketplace, a Chinese website for online shopping
Overseas high-end care	Battery Reconditioner	Good review!	Taobao Marketplace, a Chinese website for online shopping
Overseas high-end care	Battery Reconditioner	Good point, not so good with it😅	Taobao Marketplace, a Chinese website for online shopping
Overseas high-end care	Battery Reconditioner	It's not bad for a trial.	Taobao Marketplace, a Chinese website for online shopping

Overseas high-end care	Battery Reconditioner	Cell phone battery repair is fantastic, it's a godsend! Kudos👍👍👍👍	Taobao Marketplace, a Chinese website for online shopping
Overseas high-end care	Battery Reconditioner	In-kind and the same as described, the quality is quite good, the seller's attitude is also good, ask questions and answer them, the speed of delivery bar, worth buying Oh!	Taobao Marketplace, a Chinese website for online shopping
Overseas high-end care	Battery Reconditioner	Repairer to now use to repair the cell phone battery4times, the phone battery has been somewhat improved, the battery life is longer than before some good, the feeling is still good	Taobao Marketplace, a Chinese website for online shopping
Overseas high-end care	Battery Reconditioner	📩been wanting it for a long time, I decided to go for it, I feel great after using it, it's genuine and the delivery is great, ititsname👍	Taobao Marketplace, a Chinese website for online shopping
Overseas high-end care	Battery Reconditioner	Cell phone battery repairer received, the quality is very good, there is a need to rest assured that the purchase!	Taobao Marketplace, a Chinese website for online shopping
Overseas high-end care	Battery Reconditioner	System default comments	Taobao Marketplace, a Chinese website for online shopping
Overseas high-end care	Battery Reconditioner	The logistics speed is awesome and the packaging is great. The pattern is cute, the design is awesome, and it's easy to use	Taobao Marketplace, a Chinese website for online shopping
Overseas high-end care	Battery Reconditioner	Been using it consistently for a while now, it's really good, optimizes the phone's system and the battery lasts longer.	Taobao Marketplace, a Chinese website for online shopping
Overseas high-end care	Battery Reconditioner	Cell phone battery repair activator received is genuine, easy to use, repair battery effect is good, satisfied👍	Taobao Marketplace, a Chinese website for online shopping
Overseas high-end care	Battery Reconditioner	Cell phone battery repair after a few times, today has been able to use a whole day The effect is still OK Easy to use too	Taobao Marketplace, a Chinese website for online shopping
Overseas high-end care	Battery Reconditioner	Received it, it's very nice and is working.	Taobao Marketplace, a Chinese website for online shopping
Overseas high-end care	Battery Reconditioner	System default comments	Taobao Marketplace, a Chinese website for online shopping
Overseas high-end care	Battery Reconditioner	the cell phone battery has become stronger, the battery is durable, and the power consumption has become slower and more energy efficient.	Taobao Marketplace, a Chinese website for online shopping
Overseas high-end care	Battery Reconditioner	Cell phone with a long time, the battery is more and more bad, the loss of this replenishment of power God ah, otherwise I am worried about death.	Taobao Marketplace, a Chinese website for online shopping
Overseas high-end care	Battery Reconditioner	It works really well, the charge is full and durable awesome!	Taobao Marketplace, a Chinese website for online shopping
Overseas high-end care	Battery Reconditioner	Product Reviews:Things received, the repair effect is really good, need friends rest assured to buy	Taobao Marketplace, a Chinese website for online shopping
Overseas high-end care	Battery Reconditioner	The packaging is very sturdy and the stuff is well protected, so it's worth a good review	Taobao Marketplace, a Chinese website for online shopping
Overseas high-end care	Battery Reconditioner	cell phone battery gaga good use, high security, genuine good use	Taobao Marketplace, a Chinese website for online shopping
Overseas high-end care	Battery Reconditioner	The phone drops power a little fast, the battery is not enough, see this to try, the result is still effective, the value of high effect, adhere to the instructions, I believe it will be used for a few more years ha!	Taobao Marketplace, a Chinese website for online shopping
Overseas high-end care	Battery Reconditioner	The manual has phone optimization tips and the board is clean and tidy(twoLEDbulbs a transistor and a few small parts)	Taobao Marketplace, a Chinese website for online shopping
Overseas high-end care	Battery Reconditioner	The stuff received, good quality, affordable, fast shipping, good service, easy to use, good results, thank you.	Taobao Marketplace, a Chinese website for online shopping
Overseas high-end care	Battery Reconditioner	Baby received, the seller shipping speed is quite fast. The cell phone is used for a long time, the battery is really very power consumption, now after using the repair can be better, can use longer time.	Taobao Marketplace, a Chinese website for online shopping
Overseas high-end care	Battery Reconditioner	The battery restorer arrived, good quality, used it and it's fine.	Taobao Marketplace, a Chinese website for online shopping
Overseas high-end care	Battery Reconditioner	Cell phone battery repairer quality is very good, strong function, easy to use, effectively clean up the garbage, is genuine, worth buying!	Taobao Marketplace, a Chinese website for online shopping
Overseas high-end care	Battery Reconditioner	Used it for a day to repair the cell phone battery, feel the battery is much more durable, satisfied!	Taobao Marketplace, a Chinese website for online shopping
Overseas high-end care	Battery Reconditioner	Commodity evaluation:cell phone battery repairer received, the quality is very good, the need for sisters to rest assured that purchase	Taobao Marketplace, a Chinese website for online shopping
Overseas high-end care	Battery Reconditioner	Baby quality is great, practical and good, repair preferred, good choice	Taobao Marketplace, a Chinese website for online shopping
Overseas high-end care	Battery Reconditioner	Cell phone battery repairer received is genuine, easy to use, a variety of models of cell phones universal, good	Taobao Marketplace, a Chinese website for online shopping
Overseas high-end care	Battery Reconditioner	Improve cell phone battery life and more durable, and clean up cell phone garbage, currently used for more than ten days, can obviously feel the effect, the price is too worth it	Taobao Marketplace, a Chinese website for online shopping
Overseas high-end care	Battery Reconditioner	the battery, after repairing the phone with a little longer, and does not heat up, and also automatically clean up the phone system cache garbage, the phone has a significantly smooth, the boss said that often used to repair will be a little better. Does not hurt the phone, the appearance is very beautiful, but also equipped with a display, it is very convenient to use!	Taobao Marketplace, a Chinese website for online shopping

Overseas high-end care	Battery Reconditioner	really late love, used a few days to feel the phone than before lagging a little better, and not too hot, thank you boss and wish your company prosperous business, more and more good!	Taobao Marketplace, a Chinese website for online shopping
Overseas high-end care	Battery Reconditioner	How longrecharge How do I know it'？	Taobao Marketplace, a Chinese website for online shopping
Overseas high-end care	Battery Reconditioner	There's no way anyone thinks it's fixable, is there?	Taobao Marketplace, a Chinese website for online shopping
Overseas high-end care	Battery Reconditioner	Is this an IQ detector?	Taobao Marketplace, a Chinese website for online shopping
Overseas high-end care	Battery Reconditioner	Does it really fix battery drain?	Taobao Marketplace, a Chinese website for online shopping
Overseas high-end care	Battery Reconditioner	ineffective	Taobao Marketplace, a Chinese website for online shopping
Overseas high-end care	Battery Reconditioner	Repair the water ingress🧠?	Taobao Marketplace, a Chinese website for online shopping
Overseas high-end care	Battery Reconditioner	Can you fix your brain?	Taobao Marketplace, a Chinese website for online shopping
Overseas high-end care	Battery Reconditioner	That's a big mistake.	Taobao Marketplace, a Chinese website for online shopping
Overseas high-end care	Battery Reconditioner	Sure it's not an IQ tax?	Taobao Marketplace, a Chinese website for online shopping
Overseas high-end care	Battery Reconditioner	Fixing the brain?	Taobao Marketplace, a Chinese website for online shopping
Overseas high-end care	Battery Reconditioner	Will anyone really buy it?	Taobao Marketplace, a Chinese website for online shopping
Overseas high-end care	Battery Reconditioner	Will you be able to ascend to immortality if you buy it?	Taobao Marketplace, a Chinese website for online shopping
Overseas high-end care	Battery Reconditioner	Nothing works.	Taobao Marketplace, a Chinese website for online shopping
Overseas high-end care	Battery Reconditioner	ineffective	Taobao Marketplace, a Chinese website for online shopping
Overseas high-end care	Battery Reconditioner	Enough to buy before the phone charge always remind the battery is not working, need to replace the battery, after using this product charge there is no reminder.	Taobao Marketplace, a Chinese website for online shopping
Overseas high-end care	Battery Reconditioner	should not	Taobao Marketplace, a Chinese website for online shopping
Overseas high-end care	Battery Reconditioner	For old cell phones that won't charge there are suburban fruit	Taobao Marketplace, a Chinese website for online shopping
Overseas high-end care	Battery Reconditioner	It's not.	Taobao Marketplace, a Chinese website for online shopping
Overseas high-end care	Battery Reconditioner	It's for old cell phones that won't charge and fix it	Taobao Marketplace, a Chinese website for online shopping
Overseas high-end care	Battery Reconditioner	No offense, my old phone was going to be given to a recycler and I was able to keep it because of this product!	Taobao Marketplace, a Chinese website for online shopping
Overseas high-end care	Battery Reconditioner	IQ tax	Taobao Marketplace, a Chinese website for online shopping
Overseas high-end care	Battery Reconditioner	(nonverbal grunt as interjection)	Taobao Marketplace, a Chinese website for online shopping
Overseas high-end care	Battery Reconditioner	No kidding! (gently sarcastic)	Taobao Marketplace, a Chinese website for online shopping
Overseas high-end care	Battery Reconditioner	It's just a toy. It doesn't work. It breaks.	Taobao Marketplace, a Chinese website for online shopping
Cowman Life Electric	Battery Repairer	Cell phone battery repairer is very practical, the business online communication is timely, value for money!	Taobao Marketplace, a Chinese website for online shopping
Cowman Life Electric	Battery Repairer	I'll give you a good review first, and then I'll follow up after I've used it	Taobao Marketplace, a Chinese website for online shopping
Cowman Life Electric	Battery Repairer	rare	Taobao Marketplace, a Chinese website for online shopping
Cowman Life Electric	Battery Repairer	serviceable	Taobao Marketplace, a Chinese website for online shopping
Cowman Life Electric	Battery Repairer	I haven't started using it yet, and I don't know yet if it works well or not.	Taobao Marketplace, a Chinese website for online shopping
Cowman Life Electric	Battery Repairer	How can I not give 5 stars for coming to Taiwan？	Taobao Marketplace, a Chinese website for online shopping
Cowman Life Electric	Battery Repairer	Very good positive reviews	Taobao Marketplace, a Chinese website for online shopping
Cowman Life Electric	Battery Repairer	,***	Taobao Marketplace, a Chinese website for online shopping
Cowman Life Electric	Battery Repairer	The old man with a cell phone is always charging there will be a little problem, this special repair, with a very satisfied, but also really save a little power, first praise.	Taobao Marketplace, a Chinese website for online shopping
Cowman Life Electric	Battery Repairer	Received and used, genuine, really can save electricity,, especially satisfied with the	Taobao Marketplace, a Chinese website for online shopping
Cowman Life Electric	Battery Repairer	I haven't used it yet, let's give it a good review first	Taobao Marketplace, a Chinese website for online shopping
Cowman Life Electric	Battery Reconditioner	goody	Taobao Marketplace, a Chinese website for online shopping

Cowman Life Electric	Battery Reconditioner	Goods received, the quality is good, logistics is fast, after using in the evaluation.	Taobao Marketplace, a Chinese website for online shopping
Cowman Life Electric	Battery Repairer	The quality is good, the logistics is quite fast, I do not know whether it works or not	Taobao Marketplace, a Chinese website for online shopping
Cowman Life Electric	Battery Repairer	👍	Taobao Marketplace, a Chinese website for online shopping
Cowman Life Electric	Battery Repairer	higher price, looking forward to the results, if good will come back!	Taobao Marketplace, a Chinese website for online shopping
Cowman Life Electric	Battery Repairer	Didn't work. Bought it and paid the IQ tax.	Taobao Marketplace, a Chinese website for online shopping
Cowman Life Electric	Battery Repairer	temperature is low, there is no heating phenomenon, the repair effect on the cell phone battery can not be seen, good service, fast logistics!👍👍👍	Taobao Marketplace, a Chinese website for online shopping
Cowman Life Electric	Battery Repairer	rare	Taobao Marketplace, a Chinese website for online shopping
Cowman Life Electric	Battery Repairer	Trying it out.	Taobao Marketplace, a Chinese website for online shopping
Cowman Life Electric	Battery Repairer	It can be used to monitor the charging performance, and the repair effect seems to be testable.	Taobao Marketplace, a Chinese website for online shopping
Cowman Life Electric	Battery Repairer	formula 1 (math.)	Taobao Marketplace, a Chinese website for online shopping
Cowman Life Electric	Battery Repairer	Been using it for a while now and it doesn't feel like it's working at all! The good reviews should all be swiped by someone, ugh! Another IQ tax paid.	Taobao Marketplace, a Chinese website for online shopping
Cowman Life Electric	Battery Repairer	ineffective	Taobao Marketplace, a Chinese website for online shopping
Cowman Life Electric	Battery Repairer	The restorer has been received, the logistics is fast, is the genuine article	Taobao Marketplace, a Chinese website for online shopping
Cowman Life Electric	Battery Repairer	Cell phone charging repairer has received, logistics is very fast, is genuine, repair battery effect is obvious	Taobao Marketplace, a Chinese website for online shopping
Cowman Life Electric	Battery Repairer	an excellent	Taobao Marketplace, a Chinese website for online shopping
Cowman Life Electric	Battery Repairer	The goods have been received and I'm very happy with them.	Taobao Marketplace, a Chinese website for online shopping
Cowman Life Electric	Battery Repairer	Frequent delays in delivery	Taobao Marketplace, a Chinese website for online shopping
Cowman Life Electric	Battery Repairer	Conscientious businessman, return if not satisfied! Good review!	Taobao Marketplace, a Chinese website for online shopping
Cowman Life Electric	Battery Repairer	phoney	Taobao Marketplace, a Chinese website for online shopping
Cowman Life Electric	Battery Repairer	didn't work much I've been using it for almost 10 days now It's really useful for repairing batteries Now the battery works a little better than it did before I hope it gets better and better from now on It's like a new machine	Taobao Marketplace, a Chinese website for online shopping
Cowman Life Electric	Battery Repairer	I don't see any effect.	Taobao Marketplace, a Chinese website for online shopping
Cowman Life Electric	Battery Repairer	Practical and good.	Taobao Marketplace, a Chinese website for online shopping
Cowman Life Electric	Battery Repairer	(of an unmarried couple) be close	Taobao Marketplace, a Chinese website for online shopping
Cowman Life Electric	Battery Repairer	I don't know if it works, but I bought it on a fluke, in case it works. Is it?	Taobao Marketplace, a Chinese website for online shopping
Cowman Life Electric	Battery Repairer	phone doesn't store power! I hope people buy cell phone fixers!	Taobao Marketplace, a Chinese website for online shopping
Cowman Life Electric	Battery Repairer	Use it and then comment.	Taobao Marketplace, a Chinese website for online shopping
Cowman Life Electric	Battery Repairer	No visible effect	Taobao Marketplace, a Chinese website for online shopping
Cowman Life Electric	Battery Repairer	average	Taobao Marketplace, a Chinese website for online shopping
Cowman Life Electric	Battery Repairer	an excellent	Taobao Marketplace, a Chinese website for online shopping
Cowman Life Electric	Battery Repairer	can	Taobao Marketplace, a Chinese website for online shopping
Cowman Life Electric	Battery Repairer	I'll give you five stars.	Taobao Marketplace, a Chinese website for online shopping
Cowman Life Electric	Battery Repairer	Fake and fraudulent can't be fixed at all	Taobao Marketplace, a Chinese website for online shopping
Cowman Life Electric	Battery Repairer	The product does not function as described.	Taobao Marketplace, a Chinese website for online shopping
Cowman Life Electric	Battery Repairer	The product began to use did not find any effect, but after about ten days of use a slight effect of	Taobao Marketplace, a Chinese website for online shopping
Cowman Life Electric	Battery Repairer	The quality is good.	Taobao Marketplace, a Chinese website for online shopping
Cowman Life Electric	Battery Repairer	Both charging and repairing, safe and solid, heat dissipation performance is particularly good. The packaging is exquisite and tight👍.	Taobao Marketplace, a Chinese website for online shopping

Cowman Life Electric	Battery Repairer	Used it for two days, charged it five times, no effect.	Taobao Marketplace, a Chinese website for online shopping
Cowman Life Electric	Battery Repairer	Repaired battery Long standby time Easy to operate Very practical In No need to change the battery Satisfaction	Taobao Marketplace, a Chinese website for online shopping
Cowman Life Electric	Battery Repairer	maintenance role, the quality is very good, it is worth recommending to the majority of customers who have a cell phone to buy, repair ten maintenance of your cell phone battery.	Taobao Marketplace, a Chinese website for online shopping
Cowman Life Electric	Battery Repairer	Battery repair is very good, very satisfied, improve the life of durability well	Taobao Marketplace, a Chinese website for online shopping
Cowman Life Electric	Battery Repairer	The quality is great, the battery lasts longer than it did, and it works very well.	Taobao Marketplace, a Chinese website for online shopping
Cowman Life Electric	Battery Repairer	Nice stuff.	Taobao Marketplace, a Chinese website for online shopping
Cowman Life Electric	Battery Repairer	Repair effect is very good, the shape of the small portable, very practical	Taobao Marketplace, a Chinese website for online shopping
Cowman Life Electric	Battery Repairer	Cell phone battery repairer to try the effect is good old Samsung put4.5years with other chargers charging half an hour useless with this5minutes to start charging, as for the repair effect to be used to see. Favorable comments.	Taobao Marketplace, a Chinese website for online shopping
Cowman Life Electric	Battery Repairer	The quality is quite good, cost-effective, worth buying	Taobao Marketplace, a Chinese website for online shopping
Cowman Life Electric	Battery Repairer	Cell Phone Repair Magic is mini and delicate, easy to use, good repair effect, especially satisfied.	Taobao Marketplace, a Chinese website for online shopping
Cowman Life Electric	Battery Repairer	The first time to buy this, affordable and good, fast shipping, the store is very good Oh, worth buying!	Taobao Marketplace, a Chinese website for online shopping
Cowman Life Electric	Battery Repairer	Just received the goods, have not yet used, first give positive feedback, customer service commitment60days invalid can be returned unconditionally,	Taobao Marketplace, a Chinese website for online shopping
Cowman Life Electric	Battery Repairer	It's very convenient to carry it outside, doesn't hurt the battery, efficient heat dissipation, much more durable than before, very satisfactory	Taobao Marketplace, a Chinese website for online shopping
Cowman Life Electric	Battery Repairer	Used a few times to feel a little effect, especially the old phone is more obvious, my Huawei cell phone shutdown after using the phone immediately active open for some reason, other brands do not have this situation, in short, after the use of the battery feel more durable.	Taobao Marketplace, a Chinese website for online shopping
Cowman Life Electric	Battery Repairer	Cell phone battery repairer quality is very good, strong function, easy to use, effectively clean up the garbage, is genuine, worth buying!	Taobao Marketplace, a Chinese website for online shopping
Cowman Life Electric	Battery Repairer	Good, good, a few hours to repair the success of it, and consistent with the description, it is worth buying!	Taobao Marketplace, a Chinese website for online shopping
Cowman Life Electric	Battery Repairer	Use a long time basically does not heat up, the phone battery has become stronger, the battery is durable, the power consumption has become slower, more energy-saving	Taobao Marketplace, a Chinese website for online shopping
Cowman Life Electric	Battery Repairer	Practical, good quality, no longer worry about charging can not be charged in, satisfaction	Taobao Marketplace, a Chinese website for online shopping
Cowman Life Electric	Battery Repairer	Good quality, practical, as described by customer service, cost-effective	Taobao Marketplace, a Chinese website for online shopping
Cowman Life Electric	Battery Repairer	Shipping logistics speed are quite fast, packaging is very good, the store has a great service attitude, arrival contact customer service to teach how to use, the phone battery is much more durable than before, very satisfied!	Taobao Marketplace, a Chinese website for online shopping
Cowman Life Electric	Battery Repairer	can	Taobao Marketplace, a Chinese website for online shopping
Cowman Life Electric	Battery Repairer	Strong fixes, not expensive, fast	Taobao Marketplace, a Chinese website for online shopping
Cowman Life Electric	Battery Repairer	Received, as described thanks boss! Fixed Immediately the problem of hot phone is solved, and the slow response has been significantly improved.	Taobao Marketplace, a Chinese website for online shopping
Cowman Life Electric	Battery Repairer	Really useful, thought there is no rescue, bought a back in accordance with the tutorial to use really work, the battery is twice as durable, really great value for money!	Taobao Marketplace, a Chinese website for online shopping
Cowman Life Electric	Battery Repairer	Cell phone battery repair device has got, logistics is fast, well-packed, repair is very good, the original phone shutdown, charging for a while has been turned on!	Taobao Marketplace, a Chinese website for online shopping
Cowman Life Electric	Battery Repairer	Cell phone battery repair artifact has received, with this repair artifact on the cell phone battery repair is better, the battery use time is longer than the original, good value for money, give five-star praise!	Taobao Marketplace, a Chinese website for online shopping
Cowman Life Electric	Battery Repairer	There is a cell phone at home for too many years, the battery is really not durable, the power with the same readings, a moment on the shutdown, it is full of previous memories, throw and do not want to throw, the old powerless shutdown, I use this battery repairer, the battery has been a very good boost, and now can be used for two or three hours, the price is more affordable, very satisfied with the recommended purchase!	Taobao Marketplace, a Chinese website for online shopping

Cowman Life Electric	Battery Repairer	Cell phone battery repairer after hours of charging and on a cell phone that can not be charged into the repair, a few hours to repair the success, kudos! Commodity description is consistent, logistics is so fast, worth buying!	Taobao Marketplace, a Chinese website for online shopping
Cowman Life Electric	Battery Repairer	Received product! Have used it! Works very well! I am very satisfied! Refer more customers with you guys! Thanks 🙏	Taobao Marketplace, a Chinese website for online shopping
Cowman Life Electric	Battery Repairer	repaired... Used a few days to evaluate, the effect is still there, one of the cell phone original charging charging can not be charged, with this now can be charged very quickly, there is a cell phone must be plugged in all the time to be able to use, not plugged in the power will be automatically shut down, after repairing several times, not plugged in the power can be used for a period of time, on the cell phone lagging problem, is still in the test. All in all it still works, not bad not bad.	Taobao Marketplace, a Chinese website for online shopping
Cowman Life Electric	Battery Repairer	Really effective, I thought there is no rescue, my OPPO Now barbaric hold to try the mentality to buy a back, according to the tutorials do, but also really have a role, the battery is twice as durable, really too cost-effective!	Taobao Marketplace, a Chinese website for online shopping
Cowman Life Electric	Battery Repairer	Affordable price, great quality, very good shopping, next time come back!	Taobao Marketplace, a Chinese website for online shopping
Cowman Life Electric	Battery Repairer	Baby received, the packaging is very strong, should be a good thing, first give a favorable comment	Taobao Marketplace, a Chinese website for online shopping
Cowman Life Electric	Battery Repairer	Received the goods, waiting to verify if it really works!	Taobao Marketplace, a Chinese website for online shopping
Cowman Life Electric	Battery Repairer	How can I not give 5 stars for coming to Taiwan?	Taobao Marketplace, a Chinese website for online shopping
Cowman Life Electric	Battery Repairer	Repairing your cell phone is powerful, you can clean the trash in your cell phone, easy to use.	Taobao Marketplace, a Chinese website for online shopping
Cowman Life Electric	Battery Repairer	Does it really work? Is it an IQ tax?	Taobao Marketplace, a Chinese website for online shopping
Cowman Life Electric	Battery Repairer	Is it working, guys?	Taobao Marketplace, a Chinese website for online shopping
Cowman Life Electric	Battery Repairer	It's not like it's gonna put a virus in your cell phone.	Taobao Marketplace, a Chinese website for online shopping
Cowman Life Electric	Battery Repairer	Will you pay the IQ tax?	Taobao Marketplace, a Chinese website for online shopping
Cowman Life Electric	Battery Repairer	Is it really fixable?	Taobao Marketplace, a Chinese website for online shopping
Cowman Life Electric	Battery Repairer	Haven't used it yet.	Taobao Marketplace, a Chinese website for online shopping
Cowman Life Electric	Battery Repairer	I've used it once, but I don't feel it.	Taobao Marketplace, a Chinese website for online shopping
Cowman Life Electric	Battery Repairer	Also known at this time	Taobao Marketplace, a Chinese website for online shopping
Cowman Life Electric	Battery Repairer	an excellent	Taobao Marketplace, a Chinese website for online shopping
Cowman Life Electric	Battery Repairer	should not	Taobao Marketplace, a Chinese website for online shopping
Cowman Life Electric	Battery Repairer	The quality of the goods is not good. When charging it's cord is hot. It feels like it's going to catch fire someday. I don't trust it.	Taobao Marketplace, a Chinese website for online shopping
Cowman Life Electric	Battery Repairer	Goods received.	Taobao Marketplace, a Chinese website for online shopping
Liren Department [illegible]	Battery Repairer	The store went out of business.	Taobao Marketplace, a Chinese website for online shopping
Flagship Store	Battery Repairer	It's okay.	Taobao Marketplace, a Chinese website for online shopping
Flagship Store	Battery Repairer	Cell phone battery repairer has received, try a little, can not see the battery repair to what effect, first use to see it, and then come back later to follow up!	Taobao Marketplace, a Chinese website for online shopping
Flagship Store	Battery Repairer	[illegible] described.	Taobao Marketplace, a Chinese website for online shopping
Flagship Store	Battery Repairer	Let's wait and see how it works after the restoration	Taobao Marketplace, a Chinese website for online shopping
Flagship Store	Battery Repairer	Good review.	Taobao Marketplace, a Chinese website for online shopping
Flagship Store	Battery Repairer	[illegible] results.	Taobao Marketplace, a Chinese website for online shopping
Flagship Store	Battery Repairer	Received immediately opened to see, very good, superb quality, is using the effect is not sure, but feel quite good, recommended recommended.	Taobao Marketplace, a Chinese website for online shopping
Flagship Store	Battery Repairer	phone in all aspects of the feeling good charge long time not afraid of the battery rushed bad price is a little expensive, or good	Taobao Marketplace, a Chinese website for online shopping
Flagship Store	Battery Repairer	It's really good and effective, before I bought a fake one I bought this one and threw that one away.	Taobao Marketplace, a Chinese website for online shopping
Flagship Store	Battery Repairer	Needless to say too much, this is truly a godsend, a device accelerating death godsend!	Taobao Marketplace, a Chinese website for online shopping
Flagship Store	Battery Repairer	Good!	Taobao Marketplace, a Chinese website for online shopping
Flagship Store	Battery Repairer	incompetent	Taobao Marketplace, a Chinese website for online shopping

Flagship Store	Battery Repairer	It is genuine very good, the price is much cheaper, cost-effective, really special cost-effective, packaging is also very high-end atmosphere.	Taobao Marketplace, a Chinese website for online shopping
Flagship Store	Battery Repairer	very good	Taobao Marketplace, a Chinese website for online shopping
Flagship Store	Battery Repairer	Can't see the effect.	Taobao Marketplace, a Chinese website for online shopping
Flagship Store	Battery Repairer	Received the item and used it for a couple days can only tell it's charging! Don't know how it shows up as fixed! I hope the seller boss give a reply okay? Thank you!	Taobao Marketplace, a Chinese website for online shopping
Flagship Store	Battery Repairer	The seller's attitude is also good, ask questions and answer them, the speed of delivery is great, it is worth buying Oh worth buying the product, the workmanship is very fine, better than expected, recommended to try good!	Taobao Marketplace, a Chinese website for online shopping
Flagship Store	Battery Repairer	very good	Taobao Marketplace, a Chinese website for online shopping
Flagship Store	Battery Repairer	A nice item, keep repurchasing.	Taobao Marketplace, a Chinese website for online shopping
Flagship Store	Battery Repairer	Not bad. Nice.	Taobao Marketplace, a Chinese website for online shopping
Flagship Store	Battery Repairer	The restorer should work, it's been used a few times and the battery lasts longer than before	Taobao Marketplace, a Chinese website for online shopping
Flagship Store	Battery Repairer	淘寶網 Convenient, fast Quality Assurance Economical Home Supplies Electrical Brands Hygiene and Safety All 淘寶網	Taobao Marketplace, a Chinese website for online shopping
Flagship Store	Battery Repairer	淘寶網 Convenient, fast Quality Assurance Economical Home Supplies Electrical Brands Hygiene and Safety All 淘寶網	Taobao Marketplace, a Chinese website for online shopping
Flagship Store	Battery Repairer	Cell phone battery repairer received, looks good, not yet used, there are a few old cell phones and tablets at home, wait for the test and then follow up.	Taobao Marketplace, a Chinese website for online shopping
Flagship Store	Battery Repairer	Effective in restoring battery capacity.	Taobao Marketplace, a Chinese website for online shopping
Flagship Store	Battery Repairer	It's not much use.	Taobao Marketplace, a Chinese website for online shopping
Flagship Store	Battery Repairer	two days, but did not see the phone battery has improved, the use of time and the original as long. Replaced an old Apple cell phone, repair twice, each time about 8 hours, also did not feel the effect, or the same as the original. Is this pulse repair really useful?	Taobao Marketplace, a Chinese website for online shopping
Flagship Store	Battery Repairer	The appearance looks premium. Haven't used it yet, so I hope it works as well as it looks and will post a follow up review of the results then.	Taobao Marketplace, a Chinese website for online shopping
Flagship Store	Battery Repairer	I just got it. I'm not using it.	Taobao Marketplace, a Chinese website for online shopping
Flagship Store	Battery Repairer	IQ tax.	Taobao Marketplace, a Chinese website for online shopping
Flagship Store	Battery Repairer	Charged, has been changing the number, very technological sense, received on the home phone with, now adhere to the time does last longer than before, very good products	Taobao Marketplace, a Chinese website for online shopping
Flagship Store	Battery Repairer	Really quite effective, charging to 100%, and then continuous brush video, from 17:16, until 19:41, 2 hours and 20 minutes, before at least 50%, this time the electricity used only a little more than 30%, I believe that few more times with this, the effect will be better!	Taobao Marketplace, a Chinese website for online shopping
Flagship Store	Battery Repairer	Charged, has been changing the number, very technological sense, received on the home phone with, now adhere to the time does last longer than before, very good products	Taobao Marketplace, a Chinese website for online shopping
Flagship Store	Battery Repairer	Chose this higher specification of the magic phone electric slack repair, late to try those old phones and tablets, expect to be effective, and then follow up later!	Taobao Marketplace, a Chinese website for online shopping
Flagship Store	Battery Repairer	Charged up the old cell phone and it's not bad	Taobao Marketplace, a Chinese website for online shopping
Flagship Store	Battery Repairer	(of an unmarried couple) be close	Taobao Marketplace, a Chinese website for online shopping
Flagship Store	Battery Repairer	The battery adapter works, the battery now lasts longer than before and playing games is much smoother!	Taobao Marketplace, a Chinese website for online shopping
Flagship Store	Battery Repairer	It is worth buying is genuine, and the same as the physical store, the price is also favorable, decisively turn the powder cost-effective very high?the	Taobao Marketplace, a Chinese website for online shopping
Flagship Store	Battery Repairer	Great product, love it, order and buy if you like it, Punch Punch Punch!!!!	Taobao Marketplace, a Chinese website for online shopping
Flagship Store	Battery Repairer	Just follow the seller's instructions, it works well and has a restorative effect on the battery!	Taobao Marketplace, a Chinese website for online shopping
Flagship Store	Battery Repairer	Simple to use, the effect is good, Apple 6s repair effect is very good, this time do not have to change the battery, it is worth having, and still multi-device compatibility, especially after the power is very beautiful, and finally, I wish the boss a prosperous business	Taobao Marketplace, a Chinese website for online shopping
Flagship Store	Battery Repairer	Baby received, very surprised, the quality is very good, very satisfied with a shopping. I will continue to buy back, the store is trustworthy!	Taobao Marketplace, a Chinese website for online shopping
Flagship Store	Battery Repairer	too.	Taobao Marketplace, a Chinese website for online shopping
Flagship Store	Battery Repairer	Looked very cow, technology, used a few times the phone battery is obviously more durable	Taobao Marketplace, a Chinese website for online shopping
Flagship Store	Battery Repairer	I've been using it for a while and it still has some battery reconditioning effect	Taobao Marketplace, a Chinese website for online shopping
Flagship Store	Battery Repairer	Looking forward to the results	Taobao Marketplace, a Chinese website for online shopping

Flagship Store	Battery Repairer	No effect. No.	Taobao Marketplace, a Chinese website for online shopping
Flagship Store	Battery Repairer	Used it. Works well. Battery use was noticeably longer today, amazing results!	Taobao Marketplace, a Chinese website for online shopping
Flagship Store	Battery Repairer	Being repaired, see the display jumped the number, tried several times the phone has the effect, praise!	Taobao Marketplace, a Chinese website for online shopping
Flagship Store	Battery Repairer	Fast shipping and good service	Taobao Marketplace, a Chinese website for online shopping
Flagship Store	Battery Repairer	very good, good quality, no longer need to worry about the battery charge is not enough to use it, highly recommended to buy this item!	Taobao Marketplace, a Chinese website for online shopping
Flagship Store	Battery Repairer	Repair cell phone battery life, just started using it, see how it works, if it's good, introduce it to friends to buy.	Taobao Marketplace, a Chinese website for online shopping
Flagship Store	Battery Repairer	Fast shipping and good service	Taobao Marketplace, a Chinese website for online shopping
Flagship Store	Battery Repairer	very good, good quality, no longer need to worry about the battery charge is not enough to use it, highly recommended to buy this item!	Taobao Marketplace, a Chinese website for online shopping
Flagship Store	Battery Repairer	This cell phone charging repairer is very good, my cell phone charging repair, the repairer shows from the beginning of 000 has been charging to show 500 dial down, try to try the phone a lot faster, not card stop, charging show more than 500 words, the better the effect, it seems that this repairer really has the effect of very good, five-star praise.	Taobao Marketplace, a Chinese website for online shopping
Flagship Store	Battery Repairer	this thing, has been skeptical of its efficacy and role as similar products described as magical."To know the taste of pears should be tasted in person", in line with this point of view, it is no longer entangled, and decisively chose the baby of your store. Now is the initial experimental charging (repair) of the phone, only to see the repairer's display screen numbers jumping (I do not know what the changing numbers represent?), only to see the repairer's display screen numbers jumping (I do not know what the changing numbers represent?). I just hope that through a few repairs can improve (increase) the battery's length of use, but also not in vain I wrote these words (evaluation).	Taobao Marketplace, a Chinese website for online shopping
Flagship Store	Battery Repairer	Look at the night off to charge tomorrow to see the effect with, to see if it's an IQ tax or a real effect	Taobao Marketplace, a Chinese website for online shopping
Flagship Store	Battery Repairer	Used three days three times, the conclusion came, there is no effect, used also put my battery drop use time, pay IQ tax, no no friends never buy.	Taobao Marketplace, a Chinese website for online shopping
Flagship Store	Battery Repairer	Very satisfied with a shopping, logistics is very fast, the quality is very good and very practical	Taobao Marketplace, a Chinese website for online shopping
Flagship Store	Battery Repairer	of time to evaluate, the effect is very good, the battery use time obviously increased a lot, in accordance with the instructions required to shut down the repair effect bar, without interference!	Taobao Marketplace, a Chinese website for online shopping
Flagship Store	Battery Repairer	Used it for a few days, can't see it doing anything, phone charging and discharging time is the same as the original. Maybe it's not for my phone.	Taobao Marketplace, a Chinese website for online shopping
Flagship Store	Battery Repairer	The seller's attitude is also good, ask questions and answer them, the speed of delivery is great, it is worth buying Oh worth buying the product, the workmanship is very fine, better than expected, recommended to try good!	Taobao Marketplace, a Chinese website for online shopping
Flagship Store	Battery Repairer	description is consistent, affordable price, good quality, praise.	Taobao Marketplace, a Chinese website for online shopping
Flagship Store	Battery Repairer	This battery restorer is very good, it is genuine can't wait to get it back can't wait to use it Good quality. Charging stability. Need to buy again.	Taobao Marketplace, a Chinese website for online shopping
Flagship Store	Battery Repairer	After a few days of testing the phone's battery temperature has dropped, the battery is slightly extended, or constantly have to be repaired, may only achieve some results.	Taobao Marketplace, a Chinese website for online shopping
Flagship Store	Battery Repairer	Cell phone fixer is worth buying, it improves the battery life of your cell phone, and also fixes problems such as lagging, making your cell phone more durable	Taobao Marketplace, a Chinese website for online shopping
Flagship Store	Battery Repairer	This cell phone battery repairer was received very quickly! Its quality, really good! This baby, its appearance, exquisite production, fashionable technology, small and beautiful, convenient and good use! Efficient top quality, repair effect, worth looking forward to and buy!	Taobao Marketplace, a Chinese website for online shopping
Flagship Store	Battery Repairer	Received goods, not used, later evaluation	Taobao Marketplace, a Chinese website for online shopping
Flagship Store	Battery Repairer	Used it. Works well. Battery use was noticeably longer today, amazing results!	Taobao Marketplace, a Chinese website for online shopping
Flagship Store	Battery Repairer	Old cell phone, after restoration, standby for a day, and more than sixty power, rare good product, worth buying	Taobao Marketplace, a Chinese website for online shopping
Flagship Store	Battery Repairer	It can be used now it works well and lasts longer than before	Taobao Marketplace, a Chinese website for online shopping
Flagship Store	Battery Repairer	You need to read the instructions carefully to use it, and the service is great!	Taobao Marketplace, a Chinese website for online shopping
Flagship Store	Battery Repairer	It's not bad, following the instructions the phone is already more durable than before	Taobao Marketplace, a Chinese website for online shopping

Flagship Store	Battery Repairer	This repairer after using the phone directly newborn, it is very good, the phone will not be hot, the response are becoming sensitive, have the ability to do it all can be completed, the battery is quite durable, the capacity is particularly large, the response effect is also super agile, charging effect is also super fast!	Taobao Marketplace, a Chinese website for online shopping
Flagship Store	Battery Repairer	I received the cell phone repairer and used it a few times before commenting It works well The battery is durable and the cell phone doesn't get stuck I'm very satisfied	Taobao Marketplace, a Chinese website for online shopping
Flagship Store	Battery Repairer	Battery Repairer received, fast logistics, the use of a period of time to evaluate, the effect is very good, the battery use time increased significantly a lot, in accordance with the instructions required by the shutdown repair effect, without interference, the effect can reach more than ninety percent!	Taobao Marketplace, a Chinese website for online shopping
Flagship Store	Battery Repairer	phone battery repairer after repairing the cell phone battery is obviously faster than before when charging, logistics is also very fast.	Taobao Marketplace, a Chinese website for online shopping
Flagship Store	Battery Repairer	Quality is very good, the old cell phone battery used for a long time, repair cell phone battery is effective, charging speed has become faster, value for money, cost-effective.	Taobao Marketplace, a Chinese website for online shopping
Flagship Store	Battery Repairer	I thought this thing was another IQ tax, but I was really wrong this time, it works!	Taobao Marketplace, a Chinese website for online shopping
Flagship Store	Battery Repairer	display is normal Looking forward to a better experience effect!	Taobao Marketplace, a Chinese website for online shopping
Flagship Store	Battery Repairer	The quality of the goods is very good, the charging display is being repaired, used a few days the effect is good looking forward to better later.	Taobao Marketplace, a Chinese website for online shopping
Flagship Store	Battery Repairer	Good to use, the previous cell phone charging is particularly difficult to repair cell phone stores to replace the battery are very expensive, with this cell phone battery repairer, no longer need to spend money, and do not have to beg, since they can do, not only charging stable in the fast, but also to care for cell phone batteries do not hurt the phone, the use of security is also reliable, there is a need for you to get!	Taobao Marketplace, a Chinese website for online shopping
Flagship Store	Battery Repairer	Received and tried once before coming to evaluate, feel standby time is indeed longer than before, adhere to the instructions to use, I hope that after two months will have better results, look forward to!	Taobao Marketplace, a Chinese website for online shopping
Flagship Store	Battery Repairer	the battery, after repairing the phone with a little longer, and does not heat up, and also automatically clean up the phone system cache garbage, the phone has a significantly smooth, the boss said that often used to repair will be a little better. Does not hurt the phone, the appearance is very beautiful, but also equipped with a display, it is very convenient to use!	Taobao Marketplace, a Chinese website for online shopping
Flagship Store	Battery Repairer	phone battery. It is a tool for cruising cell phone battery life. Although the complaint has been used but I believe it will be effective. I would recommend it to my friends if it works well.	Taobao Marketplace, a Chinese website for online shopping
Flagship Store	Battery Repairer	Not bad, not bad, the color and very match, really good, quite satisfied with the	Taobao Marketplace, a Chinese website for online shopping
Flagship Store	Battery Repairer	Cell phone fixer is worth buying, it improves the battery life of your cell phone and also fixes problems such as lagging, making your cell phone more durable.	Taobao Marketplace, a Chinese website for online shopping
Flagship Store	Battery Repairer	Cell phone battery repairer friends to recommend, very good, easy to use, cost-effective, satisfactory	Taobao Marketplace, a Chinese website for online shopping
Flagship Store	Battery Repairer	Received and tried once before coming to evaluate, feel standby time is indeed longer than before, adhere to the instructions to use, I hope that after two months will have better results, look forward to!	Taobao Marketplace, a Chinese website for online shopping
Flagship Store	Battery Repairer	What exactly do the numbers on the display do? It's not in the manual.	Taobao Marketplace, a Chinese website for online shopping
Flagship Store	Battery Repairer	Does it really work?	Taobao Marketplace, a Chinese website for online shopping
Flagship Store	Battery Repairer	How come my phone doesn't automatically power off after charging or repairing is complete?	Taobao Marketplace, a Chinese website for online shopping
Flagship Store	Battery Repairer	Not so clear. I'm sorry!	Taobao Marketplace, a Chinese website for online shopping
Flagship Store	Battery Repairer	I don't feel it yet.	Taobao Marketplace, a Chinese website for online shopping
Flagship Store	Battery Repairer	will not (act, happen etc)	Taobao Marketplace, a Chinese website for online shopping
Finn Yueh Electronics	Battery Repairer	know if it's good until I use it. I'm not sure if it's good or not, but I'm used to it. I haven't seen this fixer yet. I've never seen it before, so I'll have to use it to know if it's any good.	Taobao Marketplace, a Chinese website for online shopping
Finn Yueh Electronics	Battery Repairer	The quality can be, quite good, satisfied, good comments,	Taobao Marketplace, a Chinese website for online shopping
Slu King Flagship Store	Battery Repairer	Not bad, it still works better.	Taobao Marketplace, a Chinese website for online shopping
Slu King Flagship Store	Battery Repairer	pretty good	Taobao Marketplace, a Chinese website for online shopping

Slu King Flagship Store	Battery Repairer	It's a trick, isn't it? It doesn't feel like it's working.	Taobao Marketplace, a Chinese website for online shopping
Slu King Flagship Store	Battery Repairer	It doesn't do anything at all, the display doesn't do much, and you can't tell the extent of the repair from anywhere	Taobao Marketplace, a Chinese website for online shopping
Slu King Flagship Store	Battery Repairer	Just received, wait for a few days to try to evaluate_x005f_x0001_ very good, very satisfied	Taobao Marketplace, a Chinese website for online shopping
Slu King Flagship Store	Battery Repairer	online that this stuff didn't work. Already used it, officially announced. This thing is too good, the battery repair full, after charging can be used for more than 30hours, there is a cell phone battery can not be charged, and now has been rushed, the power is very durable, play music hard, put into more than 20% of time, and then repair once again (is charging the electricity). A small suggestion: the workmanship can be a little more fine, always feel that the plug this small piece will wiggle, so wrapped a circle with tape.	Taobao Marketplace, a Chinese website for online shopping
Slu King Flagship Store	Battery Repairer	Received! Looking forward to the results! 5points	Taobao Marketplace, a Chinese website for online shopping
Slu King Flagship Store	Battery Repairer	favorable criticism	Taobao Marketplace, a Chinese website for online shopping
Slu King Flagship Store	Battery Repairer	Received it, tried it 2days, it didn't work!	Taobao Marketplace, a Chinese website for online shopping
Slu King Flagship Store	Battery Repairer	It doesn't feel like it's working.	Taobao Marketplace, a Chinese website for online shopping
Slu King Flagship Store	Battery Repairer	incompetent	Taobao Marketplace, a Chinese website for online shopping
Slu King Flagship Store	Battery Repairer	Baby has received, good business service, fast logistics	Taobao Marketplace, a Chinese website for online shopping
Slu King Flagship Store	Battery Repairer	I've been using it for a while now, but I haven't seen any results.	Taobao Marketplace, a Chinese website for online shopping
Slu King Flagship Store	Battery Repairer	It's really what the customer said, I'll comment after I've used it for a while	Taobao Marketplace, a Chinese website for online shopping
Slu King Flagship Store	Battery Repairer	Defective and poor quality. Cannot be used. Rip off!	Taobao Marketplace, a Chinese website for online shopping
Slu King Flagship Store	Battery Repairer	This item falls apart and cannot be used.	Taobao Marketplace, a Chinese website for online shopping
Slu King Flagship Store	Battery Repairer	Follow up review after use.	Taobao Marketplace, a Chinese website for online shopping
Slu King Flagship Store	Battery Repairer	I've used it a few times and it doesn't feel as divine as advertised	Taobao Marketplace, a Chinese website for online shopping
Slu King Flagship Store	Battery Repairer	marks, good quality and good use full marks, good quality and good use full marks, good quality and good use full marks, good quality and good use full marks, good quality and good use full marks, good quality and good use full marks, good quality and good use full marks, good quality and good use full marks, good quality and good use full marks, good quality and good use full marks, good quality and good use full marks, good quality and good use full marks, good quality and good use full marks, good quality and good use full marks.	Taobao Marketplace, a Chinese website for online shopping
Slu King Flagship Store	Battery Repairer	Express very fast packaging is very good, the quality is good, the effect is quite good	Taobao Marketplace, a Chinese website for online shopping
Slu King Flagship Store	Battery Repairer	Baby has received. Just used it once, the effect is not too obvious. Wait a few more times to see the effect. Battery charging is too slow, full need a whole night time, last night charged 4-5hours. Anyway, the effect has a little. Give praise	Taobao Marketplace, a Chinese website for online shopping
Slu King Flagship Store	Battery Repairer	The phone is a bit lagging and hot, so I bought this fixer, have received it, is using it, looking forward to solving the hot and lagging! The effect of additional praise	Taobao Marketplace, a Chinese website for online shopping
Slu King Flagship Store	Battery Repairer	It may be a quality issue, after charging for a long time, the plug gets hot and burns like crazy. I'll go with the highest priced and said new model.	Taobao Marketplace, a Chinese website for online shopping
Slu King Flagship Store	Battery Repairer	Getting it back is a bad one, a total waiver.	Taobao Marketplace, a Chinese website for online shopping
Slu King Flagship Store	Battery Repairer	Great value and awesome!	Taobao Marketplace, a Chinese website for online shopping
Slu King Flagship Store	Battery Repairer	Mine is an OPPO that has worked a few times with no effect at all.	Taobao Marketplace, a Chinese website for online shopping
Slu King Flagship Store	Battery Repairer	No function	Taobao Marketplace, a Chinese website for online shopping
Slu King Flagship Store	Battery Repairer	Nice stuff, easy to use.	Taobao Marketplace, a Chinese website for online shopping
Slu King Flagship Store	Battery Repairer	of the same, the effect of the use of only know, logistics is very fast.	Taobao Marketplace, a Chinese website for online shopping
Slu King Flagship Store	Battery Repairer	Very good use of logistics very fast quality is well worth buying good	Taobao Marketplace, a Chinese website for online shopping
Slu King Flagship Store	Battery Repairer	Unscrupulous businessmen, who use such ugly faces to make money, are bound to attract**.	Taobao Marketplace, a Chinese website for online shopping
Slu King Flagship Store	Battery Repairer	No results yet, use to observe.	Taobao Marketplace, a Chinese website for online shopping

Slu King Flagship Store	Battery Repairer	Let's try it and see what happens.	Taobao Marketplace, a Chinese website for online shopping
Slu King Flagship Store	Battery Repairer	Great quality, great value!	Taobao Marketplace, a Chinese website for online shopping
Slu King Flagship Store	Battery Repairer	Just used it and don't understand it yet. I'll find out in a couple days!	Taobao Marketplace, a Chinese website for online shopping
Slu King Flagship Store	Battery Repairer	acceptable	Taobao Marketplace, a Chinese website for online shopping
Slu King Flagship Store	Battery Repairer	The quality is great I love it, the customer service is great I love it.	Taobao Marketplace, a Chinese website for online shopping
Slu King Flagship Store	Battery Repairer	It works great.	Taobao Marketplace, a Chinese website for online shopping
Slu King Flagship Store	Battery Repairer	It doesn't work well.	Taobao Marketplace, a Chinese website for online shopping
Slu King Flagship Store	Battery Repairer	I've just been using this cell phone battery repair device to see how it works, and will continue to follow up later!	Taobao Marketplace, a Chinese website for online shopping
Slu King Flagship Store	Battery Repairer	I used a little time did not have much effect best not to buy someone rated good maybe they operate just spend tens of dollars for nothing regretted	Taobao Marketplace, a Chinese website for online shopping
Slu King Flagship Store	Battery Repairer	I'm still trying it out. I don't know if it works.	Taobao Marketplace, a Chinese website for online shopping
Slu King Flagship Store	Battery Repairer	Good review!	Taobao Marketplace, a Chinese website for online shopping
Slu King Flagship Store	Battery Repairer	I'll comment after using it.	Taobao Marketplace, a Chinese website for online shopping
Slu King Flagship Store	Battery Repairer	Opened the package and the contents fell apart!	Taobao Marketplace, a Chinese website for online shopping
Slu King Flagship Store	Battery Repairer	This first time I ordered it didn't use it, I can only know by charging it and then using my cell phone I'll comment after I use it thank you.'l	Taobao Marketplace, a Chinese website for online shopping
Slu King Flagship Store	Battery Repairer	The logistics is fast. Merchant service is too good.	Taobao Marketplace, a Chinese website for online shopping
Slu King Flagship Store	Battery Repairer	Received it and tried it out, it's great, great quality and worth having! Five stars praise!	Taobao Marketplace, a Chinese website for online shopping
Slu King Flagship Store	Battery Repairer	I bought one a few days ago and felt it was still somewhat useful, so now I'm buying another one with voice prompts	Taobao Marketplace, a Chinese website for online shopping
Slu King Flagship Store	Battery Repairer	To be honest, I bought one with the idea of paying IQ tax, and after using it for two days, I feel that it is still effective, and I have already collected it.	Taobao Marketplace, a Chinese website for online shopping
Slu King Flagship Store	Battery Repairer	Used a few days, the effect is quite good, this product is still worth supporting, the quality is still quite reliable!	Taobao Marketplace, a Chinese website for online shopping
Slu King Flagship Store	Battery Repairer	Quite good to use, my cell phone electric charge can not be charged into, every time full soon light up the red light, showing no power,, this time the tube more than a day, there are half of the power, the power of the cell phone.	Taobao Marketplace, a Chinese website for online shopping
Slu King Flagship Store	Battery Repairer	Cell phone battery repair artifact quality is very good, last night after repairing3hours has received the effect, feel the battery durable a lot, according to the instructions three times a week, continuous repair5-8weeks after the battery will be better, it is worth recommending the purchase!	Taobao Marketplace, a Chinese website for online shopping
Slu King Flagship Store	Battery Repairer	after replacing the batteries last year, the new batteries dropped points fast, got hot, and have been rebooting frequently lately. So I bought new phones and one phone was turned off and not used. Recently pulled out this baby. Dusty half a year phone on charging, I did not expect a miracle: fully charged, continuously watching21/2 hours of movies, the machine does not heat up, and does not reboot. If I had known this baby, I would not have bought a new cell phone. Thank you for the business, so that the fault idle cell phone back to life!	Taobao Marketplace, a Chinese website for online shopping
Slu King Flagship Store	Battery Repairer	good, the store has a great service attitude, the arrival contact customer service to teach how to use, go out and carry very convenient, does not hurt the battery, efficient heat dissipation, the phone battery is much more durable than before, very satisfied!	Taobao Marketplace, a Chinese website for online shopping
Slu King Flagship Store	Battery Repairer	, very durable, the workmanship is also very exquisite, smooth plugging and unplugging, the response is very fast, and the charging current is also very stable. Cost-effective is quite high. The most crucial thing is that the charging is really quite fast, more satisfied.	Taobao Marketplace, a Chinese website for online shopping
Slu King Flagship Store	Battery Repairer	phone battery repair device is small and easy to use, to be used for a period of time to see the effectiveness of observation!	Taobao Marketplace, a Chinese website for online shopping
Slu King Flagship Store	Battery Repairer	Things received a few days, did not go to comment, and now also used, very good, android system phones are not warped, with this charge can also be used, very good!	Taobao Marketplace, a Chinese website for online shopping
Slu King Flagship Store	Battery Repairer	charging and everything!Straight cry the range is superb, full blooded!	Taobao Marketplace, a Chinese website for online shopping

Slu King Flagship Store	Battery Repairer	Cell phone battery repairer received, I was watching the promotional ads shot, I have a weak cell phone battery, I want to repair it through this repairer, used a week, the battery life to improve, the cell phone running speed is also faster	Taobao Marketplace, a Chinese website for online shopping
Slu King Flagship Store	Battery Repairer	Cell phone battery repairer after hours of charging and on a cell phone that can not be charged into the repair, a few hours to repair the success, kudos! Commodity description is consistent, logistics is so fast worth buying!	Taobao Marketplace, a Chinese website for online shopping
Slu King Flagship Store	Battery Repairer	Cell phone repairer has been received, well packaged, just opened to try, feel very good, recommend, need can rest assured that the order!	Taobao Marketplace, a Chinese website for online shopping
Slu King Flagship Store	Battery Repairer	the phone really fast, the original to jump out of the phone battery to repair the text message is also gone, really useful ay.	Taobao Marketplace, a Chinese website for online shopping
Slu King Flagship Store	Battery Repairer	plugged in and also eliminates a lot of junk, this is a satisfying purchase and I'll be back when I need it in the future.	Taobao Marketplace, a Chinese website for online shopping
Slu King Flagship Store	Battery Repairer	According to customer service guidance to insert the charging head connected to the data cable can be slow charging fast power consumption or charging into the power of the cell phone to repair, the effect is really good!	Taobao Marketplace, a Chinese website for online shopping
Slu King Flagship Store	Battery Repairer	Cell phone battery repairer after hours of charging and on a cell phone that can not be charged into the repair, a few hours to repair the success, kudos! Commodity description is consistent, logistics is so fast, worth buying!	Taobao Marketplace, a Chinese website for online shopping
Slu King Flagship Store	Battery Repairer	Battery Repairer received, fast logistics, the use of a period of time to evaluate, the effect is very good, the battery use time increased significantly a lot, in accordance with the instructions required by the shutdown repair effect, without interference, the effect can reach more than ninety percent!	Taobao Marketplace, a Chinese website for online shopping
Slu King Flagship Store	Battery Repairer	barbaric hold to try the mentality to buy a back, in accordance with the tutorial to do, but also really have a role, the battery is twice as durable, really too cost-effective!	Taobao Marketplace, a Chinese website for online shopping
Slu King Flagship Store	Battery Repairer	The family's old cell phone waiting in line to be repaired, the effect is still there, one of the cell phone original charging charging can not be charged, with this now can be charged very quickly, there is a cell phone must always be plugged in to use, not plugged in will automatically shut down, after repairing a few times, not plugged in can be used for a period of time, on the cell phone lagging problem, is still in the test. All in all it still works, not bad not bad.	Taobao Marketplace, a Chinese website for online shopping
Slu King Flagship Store	Battery Repairer	Cell phone battery repair device received, small and exquisite lovely, very good quality, flat plate has three years, can not open, ready to sell, heard downstairs neighbor recommended this repair device, to buy the link to try, received today plugged into the power less than3hours to repair to90, is still repairing, small things great use, the price is also cheap, worth the need to pro start	Taobao Marketplace, a Chinese website for online shopping
Slu King Flagship Store	Battery Repairer	phenomenon of heat reduction, lagging or better, continue to use according to the requirements of the manual, the battery repair.	Taobao Marketplace, a Chinese website for online shopping
Slu King Flagship Store	Battery Repairer	Cell phone repairer received, the quality is very good, received home on the use, really a good product, we can rest assured to buy it! Give merchants a big praise.	Taobao Marketplace, a Chinese website for online shopping
Slu King Flagship Store	Battery Repairer	Cell phone battery repair artifact has received, with this repair artifact on the cell phone battery repair is better, the battery use time is longer than the original, good value for money, give five-star praise!	Taobao Marketplace, a Chinese website for online shopping
Slu King Flagship Store	Battery Repairer	adapter was received, plugged into the charger, connecting the charging cable and cell phone, it has effect on the delayed battery life of the cell phone;the logisticsalso very fast, will recommend it to my friends!	Taobao Marketplace, a Chinese website for online shopping
Slu King Flagship Store	Battery Repairer	The product is very good, the use of the effect is also very good, good value for money to buy value, logistics is very fast, pro can get into it!	Taobao Marketplace, a Chinese website for online shopping
Slu King Flagship Store	Battery Repairer	Shipped very quickly, immediately after receiving the trial, because the phone battery has been used for three years, currently a full charge can only be used for three or four hours, would like to use this adapter to repair.	Taobao Marketplace, a Chinese website for online shopping
Slu King Flagship Store	Battery Repairer	very good it cell phone charging very quickly and more than normal charger charging electricity resistant cell phone battery and repair function is very good ah special good small a role of a great!	Taobao Marketplace, a Chinese website for online shopping
Slu King Flagship Store	Battery Repairer	Doesn't the repair restore the phone to its outgoing settings?	Taobao Marketplace, a Chinese website for online shopping
Slu King Flagship Store	Battery Repairer	Is it an IQ tax?	Taobao Marketplace, a Chinese website for online shopping

Slu King Flagship Store	Battery Repairer	Do you have to be discharged from the hospital to buy it?	Taobao Marketplace, a Chinese website for online shopping
Slu King Flagship Store	Battery Repairer	Is this a brainwave detector?	Taobao Marketplace, a Chinese website for online shopping
Slu King Flagship Store	Battery Repairer	Can you fix my brain dropo_	Taobao Marketplace, a Chinese website for online shopping
Slu King Flagship Store	Battery Repairer	Does anyone really buy this?	Taobao Marketplace, a Chinese website for online shopping
Slu King Flagship Store	Battery Repairer	pointless	Taobao Marketplace, a Chinese website for online shopping
Slu King Flagship Store	Battery Repairer	Yes, it is.	Taobao Marketplace, a Chinese website for online shopping
Slu King Flagship Store	Battery Repairer	You can buy it without leaving the hospital/:^_^	Taobao Marketplace, a Chinese website for online shopping
Slu King Flagship Store	Battery Repairer	It's a cell phone battery repairer.	Taobao Marketplace, a Chinese website for online shopping
Slu King Flagship Store	Battery Repairer	The effect is confusing and not obvious.	Taobao Marketplace, a Chinese website for online shopping
Slu King Flagship Store	Battery Repairer	It's not much use.	Taobao Marketplace, a Chinese website for online shopping
Chor Yee Life Center	Battery Repairer	Charge fixes that work.	Taobao Marketplace, a Chinese website for online shopping
Chor Yee Life Center	Battery Repairer	Didn't work, charging it for a couple days didn't help at all, always on the mend. No repair at all much worse than the last one I bought.	Taobao Marketplace, a Chinese website for online shopping
Chor Yee Life Center	Battery Repairer	Junk product, fake!!!	Taobao Marketplace, a Chinese website for online shopping
Chor Yee Life Center	Battery Repairer	It's ok, it's a bit tricky to operate, I thought it would be eliminated by tapping on it. It works, but there is a mark.	Taobao Marketplace, a Chinese website for online shopping
Chor Yee Life Center	Battery Repairer	IQ tax, useless!	Taobao Marketplace, a Chinese website for online shopping
Chor Yee Life Center	Battery Repairer	Logistics lost one, the merchant sent a replacement, praise.	Taobao Marketplace, a Chinese website for online shopping
Chor Yee Life Center	Battery Repairer	I don't want to talk about it. IQ tax.	Taobao Marketplace, a Chinese website for online shopping
Chor Yee Life Center	Battery Repairer	Pay IQ tax, received it yesterday afternoon, started to use it at night, today I am going to charge it in a different outlet, unplugged it is two halves, whether it can repair the battery, do not know for the time being, because it has only been used once. To be observedit……	Taobao Marketplace, a Chinese website for online shopping
Chor Yee Life Center	Battery Repairer	same as me stupid, those who commented that the logistics is very fast, I do not understand, logistics and product quality has what to do?	Taobao Marketplace, a Chinese website for online shopping
Chor Yee Life Center	Battery Repairer	The quality is terrible!	Taobao Marketplace, a Chinese website for online shopping
Chor Yee Life Center	Battery Repairer	Not bad!	Taobao Marketplace, a Chinese website for online shopping
Chor Yee Life Center	Battery Repairer	I feel like I'm buying an IQ tax.	Taobao Marketplace, a Chinese website for online shopping
Chor Yee Life Center	Battery Repairer	Made in China craftsmanship design sense strong sense of repair effect in serious observation should be good	Taobao Marketplace, a Chinese website for online shopping
Chor Yee Life Center	Battery Repairer	👍	Taobao Marketplace, a Chinese website for online shopping
Chor Yee Life Center	Battery Repairer	A proper IQ tax, followed the instructions for a couple days and it didn't help at all.	Taobao Marketplace, a Chinese website for online shopping
Chor Yee Life Center	Battery Repairer	The restoration is unsatisfactory.	Taobao Marketplace, a Chinese website for online shopping
Chor Yee Life Center	Battery Repairer	Exquisite and compact, the delivery is very timely, tried it, it works well, praise five points.	Taobao Marketplace, a Chinese website for online shopping
Chor Yee Life Center	Battery Repairer	……	Taobao Marketplace, a Chinese website for online shopping
Chor Yee Life Center	Battery Repairer	Fake I bought one that broke after two or three days of use contacted the merchant and ignored it	Taobao Marketplace, a Chinese website for online shopping
Chor Yee Life Center	Battery Repairer	The goods have been received and match the description. Logistics is also very fast! Full five points praise!	Taobao Marketplace, a Chinese website for online shopping
Chor Yee Life Center	Battery Repairer	It didn't help at all, scammy stuff, the battery didn't have any gas at all.	Taobao Marketplace, a Chinese website for online shopping
Chor Yee Life Center	Battery Repairer	It's not much use.	Taobao Marketplace, a Chinese website for online shopping
Chor Yee Life Center	Battery Repairer	It doesn't work.	Taobao Marketplace, a Chinese website for online shopping
Chor Yee Life Center	Battery Repairer	Very poor quality product and a total rip-off	Taobao Marketplace, a Chinese website for online shopping
Chor Yee Life Center	Battery Repairer	IQ tax product, designed by Chinese talent, to purchase please see chart for analysis!	Taobao Marketplace, a Chinese website for online shopping
Chor Yee Life Center	Battery Repairer	Very good goods.	Taobao Marketplace, a Chinese website for online shopping
Chor Yee Life Center	Battery Repairer	It didn't work as well as presented.	Taobao Marketplace, a Chinese website for online shopping

Chor Yee Life Center	Battery Repairer	The owner has a good service attitude	Taobao Marketplace, a Chinese website for online shopping
Chor Yee Life Center	Battery Repairer	pay an IQ tax	Taobao Marketplace, a Chinese website for online shopping
Chor Yee Life Center	Battery Repairer	The service attitude is very good, logistics is very fast, and the description is the same, use a period of time to see the effect and then follow up!	Taobao Marketplace, a Chinese website for online shopping
Chor Yee Life Center	Battery Repairer	no good	Taobao Marketplace, a Chinese website for online shopping
Chor Yee Life Center	Battery Repairer	It doesn't match the physical ad.	Taobao Marketplace, a Chinese website for online shopping
Chor Yee Life Center	Battery Repairer	It's not gonna help.	Taobao Marketplace, a Chinese website for online shopping
Chor Yee Life Center	Battery Repairer	Shoot three, use it for a while to see the effect, it should not be bad	Taobao Marketplace, a Chinese website for online shopping
Chor Yee Life Center	Battery Repairer	Cell phone battery repair instrument received, has not been used, do not know how the effect, estimated that the internal simplicity of this thing, such as size of an ordinary U disk, buy a back to try, first to give habitual positive feedback, to be tried after the follow-up.	Taobao Marketplace, a Chinese website for online shopping
Chor Yee Life Center	Battery Repairer	It didn't work at all.	Taobao Marketplace, a Chinese website for online shopping
Chor Yee Life Center	Battery Repairer	Just started using it and will comment later.	Taobao Marketplace, a Chinese website for online shopping
Chor Yee Life Center	Battery Repairer	Can't see it working for a while, rough workmanship.	Taobao Marketplace, a Chinese website for online shopping
Chor Yee Life Center	Battery Repairer	Customer service was dishonest and promised to pass on enlargement instructions that were never done.	Taobao Marketplace, a Chinese website for online shopping
Chor Yee Life Center	Battery Repairer	Fast logistics, good customer service	Taobao Marketplace, a Chinese website for online shopping
Chor Yee Life Center	Battery Repairer	The socket is loose and feels bad quality	Taobao Marketplace, a Chinese website for online shopping
Chor Yee Life Center	Battery Repairer	This cell phone battery repairer is great	Taobao Marketplace, a Chinese website for online shopping
Chor Yee Life Center	Battery Repairer	Real fake. Doesn't fix the cell phone battery at all. Even the display is fake.	Taobao Marketplace, a Chinese website for online shopping
Chor Yee Life Center	Battery Repairer	I don't know if it'll work.	Taobao Marketplace, a Chinese website for online shopping
Chor Yee Life Center	Battery Repairer	Taote all know to send a text message to notify the pickup code, Taobao this melon B let you guess!	Taobao Marketplace, a Chinese website for online shopping
Chor Yee Life Center	Battery Repairer	It's a lie. It doesn't work at all.	Taobao Marketplace, a Chinese website for online shopping
Chor Yee Life Center	Battery Repairer	Doesn't work at all, scam never buy.	Taobao Marketplace, a Chinese website for online shopping
Chor Yee Life Center	Battery Repairer	I don't feel anything.	Taobao Marketplace, a Chinese website for online shopping
Chor Yee Life Center	Battery Repairer	The print on the instructions is too small, everything else works fine so far, the rest later!	Taobao Marketplace, a Chinese website for online shopping
Chor Yee Life Center	Battery Repairer	After almost 2 fixing its still set to see results, not as good as advertised.	Taobao Marketplace, a Chinese website for online shopping
Chor Yee Life Center	Battery Repairer	Great service and fast shipping.	Taobao Marketplace, a Chinese website for online shopping
Chor Yee Life Center	Battery Repairer	Rough, not very effective	Taobao Marketplace, a Chinese website for online shopping
Chor Yee Life Center	Battery Repairer	Wait for the effect	Taobao Marketplace, a Chinese website for online shopping
Chor Yee Life Center	Battery Repairer	no good	Taobao Marketplace, a Chinese website for online shopping
Chor Yee Life Center	Battery Repairer	Not shown in the fix	Taobao Marketplace, a Chinese website for online shopping
Chor Yee Life Center	Battery Repairer	Baby received, immediately use, repair 3 hours, the phone is not hot, good use. Very satisfied!	Taobao Marketplace, a Chinese website for online shopping
Chor Yee Life Center	Battery Repairer	Repairer received immediately use 3 hours the phone is not hot at all always show repair, good to give five-star praise!	Taobao Marketplace, a Chinese website for online shopping
Chor Yee Life Center	Battery Repairer	I just received it without using it, so I'll give it a medium rating first and comment again after using it.	Taobao Marketplace, a Chinese website for online shopping
Chor Yee Life Center	Battery Repairer	Baby received, small and exquisite. Have not yet used, after using the follow-up evaluation	Taobao Marketplace, a Chinese website for online shopping
Chor Yee Life Center	Battery Repairer	all. Merchant attitude is still very good, made a refund processing.	Taobao Marketplace, a Chinese website for online shopping
Chor Yee Life Center	Battery Repairer	Not seeing the desired results	Taobao Marketplace, a Chinese website for online shopping
Chor Yee Life Center	Battery Repairer	Fast shipping, good quality, really works, very satisfied, love it, thanks.	Taobao Marketplace, a Chinese website for online shopping
Chor Yee Life Center	Battery Repairer	Bought 2, a own use, a gift to friends, used a few days, the effect is really not bad, I am personally quite satisfied, the details are also done in place, looks quite upscale, overall cost-effective, it is worth getting.	Taobao Marketplace, a Chinese website for online shopping

Chor Yee Life Center	Battery Repairer	battery is not hot, the use of much smoother, pro assured purchase it!	Taobao Marketplace, a Chinese website for online shopping
Chor Yee Life Center	Battery Repairer	Good to use, the previous cell phone charging is particularly difficult to repair cell phone stores to replace the battery are very expensive, with this cell phone battery repairer, no longer need to spend money, and do not have to beg, since they can do, not only charging stable in the fast, but also to care for cell phone batteries do not hurt the phone, the use of security is also reliable, there is a need for you to get it.	Taobao Marketplace, a Chinese website for online shopping
Chor Yee Life Center	Battery Repairer	days to evaluate, the effect is still there, one of the cell phone original charging charging can not be charged into, with this now can be charged very quickly, there is a cell phone must always be plugged in order to use, not plugged in the power will be automatically shut down, after repairing a few times, not plugged in can be used for a period of time, on the cell phone lagging problem, is still in the test. Anyway, it's still there.	Taobao Marketplace, a Chinese website for online shopping
Chor Yee Life Center	Battery Repairer	Once, the next day the cell phone battery is much more durable. From the appearance and workmanship is very satisfied, and the business description is consistent with the customer service attitude is very good, account for a very clear, after the use of charging armor effect is good, the speed is also very fast, the quality is very good, this does not have to change the battery, thanks to the business.	Taobao Marketplace, a Chinese website for online shopping
Chor Yee Life Center	Battery Repairer	battery is not hot, the use of much smoother, pro assured purchase it!	Taobao Marketplace, a Chinese website for online shopping
Chor Yee Life Center	Battery Repairer	do not have to change the battery, after repairing the phone with a longer time some, also do not heat up, and also can automatically clean up the phone system cache garbage, the phone has obvious smooth, the boss said often used to repair will be better.	Taobao Marketplace, a Chinese website for online shopping
Chor Yee Life Center	Battery Repairer	Pool Repairer has received, the phone used for a long time too many background programs, lagging, heat, battery loss fast, give people annoyed, not moving the phone on the power shutdown, the use of cell phone battery repair device, simple to connect the charging plug and charging cable, a charging immediately on the cell phone battery repair, can extend the life of the optimization of the cell phone system, the price is not only cheap, but also easy to use, businessmen fast delivery, logistics and distribution timely, give strength	Taobao Marketplace, a Chinese website for online shopping
Chor Yee Life Center	Battery Repairer	is quite good, millet phone for three years, the battery is seriously depleted, brush video brush four hours on the red, with this product, you can now brush six hours, continue to use to see if it can be improved, the future charging will rely on it!	Taobao Marketplace, a Chinese website for online shopping
Chor Yee Life Center	Battery Repairer	The first day of use, the effect is good, the phone is much more sensitive than before, and not lagging, after charging standby time gradually extended, this charging repairer is really good, worth buying, give five stars!	Taobao Marketplace, a Chinese website for online shopping
Chor Yee Life Center	Battery Repairer	To the baby has been a few days, specially tried a few days, feel or a little effect, before the day to charge twice, now a charge basically can use a day!	Taobao Marketplace, a Chinese website for online shopping
Chor Yee Life Center	Battery Repairer	phone battery of the gods, but also a good fast charger, does not hurt the phone, charging fast, the appearance is very beautiful, but also equipped with a display, it is very convenient to use. The same as described on the product introduction, and the logistics is very fast, thoughtful service, very satisfied!	Taobao Marketplace, a Chinese website for online shopping
Chor Yee Life Center	Battery Repairer	changing a battery before electricity can only be charged to25% did not expect to use this small thing actually miraculously full of electricity at first thought will not be a scam did not expect really have the effect of wishing the boss a prosperous business!	Taobao Marketplace, a Chinese website for online shopping
Chor Yee Life Center	Battery Repairer	This repairer after using the phone directly newborn, it is very good, the phone will not be hot, the response are becoming sensitive, with hands-on can be completed, the battery is quite durable, the capacity is particularly large, the response effect is also super agile, the phone does not appear to be lagging phenomenon, the charging effect is also super fast, and there is no mismatch.	Taobao Marketplace, a Chinese website for online shopping
Chor Yee Life Center	Battery Repairer	Buyer does not comment within15days	Taobao Marketplace, a Chinese website for online shopping
Chor Yee Life Center	Battery Repairer	Anyway, I didn't see any results after I bought it and used it, I didn't know it would be so much better!	Taobao Marketplace, a Chinese website for online shopping
Chor Yee Life Center	Battery Repairer	This is useful.	Taobao Marketplace, a Chinese website for online shopping
Chor Yee Life Center	Battery Repairer	Is it true?	Taobao Marketplace, a Chinese website for online shopping

Chor Yee Life Center	Battery Repairer	Can it be used foriPadbatteries?	Taobao Marketplace, a Chinese website for online shopping
Chor Yee Life Center	Battery Repairer	Is it an IQ tax?	Taobao Marketplace, a Chinese website for online shopping
Chor Yee Life Center	Battery Repairer	lit. is aware of each other sleeping	Taobao Marketplace, a Chinese website for online shopping
Chor Yee Life Center	Battery Repairer	Do the batteries in the Bluetooth headset work?	Taobao Marketplace, a Chinese website for online shopping
Chor Yee Life Center	Battery Repairer	Is this thing an IQ tax?	Taobao Marketplace, a Chinese website for online shopping
Chor Yee Life Center	Battery Repairer	It's not very effective.	Taobao Marketplace, a Chinese website for online shopping
Chor Yee Life Center	Battery Repairer	It's not very effective.	Taobao Marketplace, a Chinese website for online shopping
Chor Yee Life Center	Battery Repairer	ineffective	Taobao Marketplace, a Chinese website for online shopping
Chor Yee Life Center	Battery Repairer	(onom.) hahaha	Taobao Marketplace, a Chinese website for online shopping
Chor Yee Life Center	Battery Repairer	Heh IQ tax	Taobao Marketplace, a Chinese website for online shopping
Chor Yee Life Center	Battery Repairer	I've never tried it.	Taobao Marketplace, a Chinese website for online shopping
Chor Yee Life Center	Battery Repairer	It didn't help at all and the merchant has refunded it.	Taobao Marketplace, a Chinese website for online shopping
L Technology Life Center	Battery Repairer	It doesn't feel useful! IQ Tax!	Taobao Marketplace, a Chinese website for online shopping
L Technology Life Center	Battery Repairer	The quality is superb, I'm very happy with my purchase, it's a great value for money!	Taobao Marketplace, a Chinese website for online shopping
L Technology Life Center	Battery Repairer	Good quality, affordable price, strong logistics	Taobao Marketplace, a Chinese website for online shopping
L Technology Life Center	Battery Repairer	Overall rating: not working	Taobao Marketplace, a Chinese website for online shopping
L Technology Life Center	Battery Repairer	Junk products that don't work	Taobao Marketplace, a Chinese website for online shopping
L Technology Life Center	Battery Repairer	used once on the bad, pit people ah! Friends must not be fooled!!!	Taobao Marketplace, a Chinese website for online shopping
L Technology Life Center	Battery Repairer	? ?	Taobao Marketplace, a Chinese website for online shopping
L Technology Life Center	Battery Repairer	I just bought it and don't know how it works yet. Asked to use it off, but my phone turns on automatically when I charge it	Taobao Marketplace, a Chinese website for online shopping
L Technology Life Center	Battery Repairer	Merchant service was quite good, I bought my adapter with no display and no response when I pressed the switch. I was so angry that I contacted the merchant. The merchant said to send a replacement tomorrow. This makes us feel relieved to buy.	Taobao Marketplace, a Chinese website for online shopping
L Technology Life Center	Battery Repairer	the goods are not made to measure up	Taobao Marketplace, a Chinese website for online shopping
L Technology Life Center	Battery Repairer	Item received in good condition.	Taobao Marketplace, a Chinese website for online shopping
L Technology Life Center	Battery Repairer	The goods have been received, thank you boss	Taobao Marketplace, a Chinese website for online shopping
L Technology Life Center	Battery Repairer	It's the ideal product, it works well and the logistics are fast, so it deserves kudos!	Taobao Marketplace, a Chinese website for online shopping
L Technology Life Center	Battery Repairer	I haven't used it yet, I'll comment after I use it!	Taobao Marketplace, a Chinese website for online shopping
L Technology Life Center	Battery Repairer	the more you do better and better, very good shopping experience!	Taobao Marketplace, a Chinese website for online shopping
L Technology Life Center	Battery Repairer	Overall rating: I can	Taobao Marketplace, a Chinese website for online shopping
L Technology Life Center	Battery Repairer	Overall rating: on hand, after the restoration of the phone, responsive flexible and cast a lot of memory very good!!!!	Taobao Marketplace, a Chinese website for online shopping
L Technology Life Center	Battery Repairer	Finally received the goods, very, very much like, exquisite and beautiful workmanship, has been a lot of people praise, really too happy, is already a loyal fan of his family, will continue to buy buy buy buy!	Taobao Marketplace, a Chinese website for online shopping
L Technology Life Center	Battery Repairer	m looking forward to the results, so I'll give you a good review first😉	Taobao Marketplace, a Chinese website for online shopping
L Technology Life Center	Battery Repairer	Good! Very good! Good quality, fast charging, no heat, same as the physical store, it's my favorite!	Taobao Marketplace, a Chinese website for online shopping
L Technology Life Center	Battery Repairer	Appearance:ok	Taobao Marketplace, a Chinese website for online shopping
L Technology Life Center	Battery Repairer	Overall rating:ok Smoothness:ok Running speed:ok Form and appearance: nice	Taobao Marketplace, a Chinese website for online shopping
L Technology Life Center	Battery Repairer	Fast shipping. Good quality. Very good and practical. Very satisfied.	Taobao Marketplace, a Chinese website for online shopping
L Technology Life Center	Battery Repairer	The quality is quite good, the value is high, the child likes it very much	Taobao Marketplace, a Chinese website for online shopping
L Technology Life Center	Battery Repairer	There are effects, but they are not obvious.	Taobao Marketplace, a Chinese website for online shopping

L Technology Life Center	Battery Repairer	Very happy with the baby, will repurchase again, great value for money!	Taobao Marketplace, a Chinese website for online shopping
L Technology Life Center	Battery Repairer	Fast delivery, also good to use	Taobao Marketplace, a Chinese website for online shopping
L Technology Life Center	Battery Repairer	It's working well.	Taobao Marketplace, a Chinese website for online shopping
L Technology Life Center	Battery Repairer	It's good. It's good. It's good.	Taobao Marketplace, a Chinese website for online shopping
L Technology Life Center	Battery Repairer	The product is quite good oh! Like can buy, a very pleasant shopping!	Taobao Marketplace, a Chinese website for online shopping
L Technology Life Center	Battery Repairer	smartphones at home with bad batteries and took the time to fix them.	Taobao Marketplace, a Chinese website for online shopping
Skywalker I (1937-), Hong Kong actor and politician, President of the Republic of China from 2008	Battery Reconditioner	The store went out of business.	Taobao Marketplace, a Chinese website for online shopping
Seeking Shell Flagship Store	Battery Repairer	It's the right length and charges well.	Taobao Marketplace, a Chinese website for online shopping
Seeking Shell Flagship Store	Battery Repairer	The data cable is received, it looks beautiful, the color is very like, it works very well	Taobao Marketplace, a Chinese website for online shopping
Seeking Shell Flagship Store	Battery Repairer	The quality is great the charging is fast!	Taobao Marketplace, a Chinese website for online shopping
Seeking Shell Flagship Store	Battery Repairer	Got it. It's good. It's worth every penny.	Taobao Marketplace, a Chinese website for online shopping
Seeking Shell Flagship Store	Battery Repairer	much cheaper than the physical store, the overall feeling is okay	Taobao Marketplace, a Chinese website for online shopping
Seeking Shell Flagship Store	Battery Repairer	handy	Taobao Marketplace, a Chinese website for online shopping
Seeking Shell Flagship Store	Battery Repairer	recommendation,👍👍👍👍the same asdescription of the quality of the bar	Taobao Marketplace, a Chinese website for online shopping
Seeking Shell Flagship Store	Battery Repairer	The quality is good ah, soft silicone material head is also well protected. Cheap texture is good	Taobao Marketplace, a Chinese website for online shopping
Seeking Shell Flagship Store	Battery Repairer	The seller has a good attitude, attentive service, and the quality of the product is also good	Taobao Marketplace, a Chinese website for online shopping
Seeking Shell Flagship Store	Battery Repairer	The data cable is quite good, 3 in 1, quite convenient	Taobao Marketplace, a Chinese website for online shopping
Seeking Shell Flagship Store	Battery Repairer	Merchants shipping fast Received the goods Quality is very good Worth buying	Taobao Marketplace, a Chinese website for online shopping
Seeking Shell Flagship Store	Battery Repairer	Nice, good material, fine workmanship, fast charging	Taobao Marketplace, a Chinese website for online shopping
Seeking Shell Flagship Store	Battery Repairer	Received, packaging is very good, the color is good, the price is also very suitable, shipping is still fast, satisfied!	Taobao Marketplace, a Chinese website for online shopping
Seeking Shell Flagship Store	Battery Repairer	Baby received, the goods are awesome, inexpensive, logistics is very fast, business service attitude is also very good	Taobao Marketplace, a Chinese website for online shopping
Seeking Shell Flagship Store	Battery Repairer	Fast charging Good use	Taobao Marketplace, a Chinese website for online shopping
Seeking Shell Flagship Store	Battery Repairer	👌never thoughtcould get suchgood babyfor such an affordable price😍very good value for money, I love it😃oh😃	Taobao Marketplace, a Chinese website for online shopping
Seeking Shell Flagship Store	Battery Repairer	The quality is pretty good! Inflates quickly too!	Taobao Marketplace, a Chinese website for online shopping
Seeking Shell Flagship Store	Battery Repairer	Data cable received Charging is fast Works great Recommended	Taobao Marketplace, a Chinese website for online shopping
Seeking Shell Flagship Store	Battery Repairer	The item was received, the seller was friendly and the shipping logistics were fast. The packaging is very good. The baby is consistent with my description, the same as normal charging. Just connect it, the charging effect is better than before too amazing, the product is practical and worth buying.	Taobao Marketplace, a Chinese website for online shopping
Seeking Shell Flagship Store	Battery Repairer	Great price, great stuff so easy to use.	Taobao Marketplace, a Chinese website for online shopping
Seeking Shell Flagship Store	Battery Repairer	product is the same as described. Works well and is very affordable.	Taobao Marketplace, a Chinese website for online shopping
Seeking Shell Flagship Store	Battery Repairer	Good quality, also good to use not expensive satisfaction	Taobao Marketplace, a Chinese website for online shopping
Seeking Shell Flagship Store	Battery Repairer	Fast shipping and tight packaging Received in perfect condition, looks good quality.	Taobao Marketplace, a Chinese website for online shopping
Seeking Shell Flagship Store	Battery Repairer	Sort of works a little bit, not as awesome as advertised.	Taobao Marketplace, a Chinese website for online shopping
Seeking Shell Flagship Store	Battery Repairer	**No use	Taobao Marketplace, a Chinese website for online shopping
Seeking Shell Flagship Store	Battery Repairer	Quality is good, very good, previously did not know that there is such a product, friends recommended over, wish the seller a prosperous business.	Taobao Marketplace, a Chinese website for online shopping
Seeking Shell Flagship Store	Battery Repairer	Didn't change the charging on the cell phone, his description doesn't match, with the scam.	Taobao Marketplace, a Chinese website for online shopping

Seeking Shell Flagship Store	Battery Repairer	Things are good, convenient and easy to use, the socket on the left and right side is more humanized, just use a few days effect is not yet seen.	Taobao Marketplace, a Chinese website for online shopping
Seeking Shell Flagship Store	Battery Repairer	It's just a digital display. The phone does nothing at all. Fake 。。。。。	Taobao Marketplace, a Chinese website for online shopping
Seeking Shell Flagship Store	Battery Repairer	Haven't used it yet don't know the effect	Taobao Marketplace, a Chinese website for online shopping
Seeking Shell Flagship Store	Battery Repairer	Good.	Taobao Marketplace, a Chinese website for online shopping
Seeking Shell Flagship Store	Battery Repairer	After a couple of flushes, it seems to be working a bit.	Taobao Marketplace, a Chinese website for online shopping
Seeking Shell Flagship Store	Battery Repairer	It works and is worth every penny	Taobao Marketplace, a Chinese website for online shopping
Seeking Shell Flagship Store	Battery Repairer	Apple phone can not be used, you also need to buy your own conversion head or buy the old charger before ✎The product has been destroyed, not recommended!	Taobao Marketplace, a Chinese website for online shopping
Seeking Shell Flagship Store	Battery Repairer	also charged, used more than one time on the charging can not be charged, shutdown charging a day is not enough to charge, but also said what repair the battery? Junk stuff! Wasted20dollars for nothing.	Taobao Marketplace, a Chinese website for online shopping
Seeking Shell Flagship Store	Battery Repairer	Able to display rechargeable battery current and voltage……it is not possible to repair the battery.	Taobao Marketplace, a Chinese website for online shopping
Seeking Shell Flagship Store	Battery Repairer	favorable criticism	Taobao Marketplace, a Chinese website for online shopping
Seeking Shell Flagship Store	Battery Repairer	The seller is friendly! Quick shipping logistics! Simple packaging, stable and safe! Baby is the same as my description, same as normal charging, just have to connect it, after charging the effect is better than before, it's amazing! The product is practical, satisfied with the praise!	Taobao Marketplace, a Chinese website for online shopping
Seeking Shell Flagship Store	Battery Repairer	Pretty good very happy with it very much like the authenticity guarantee quality is very good very happy with it.	Taobao Marketplace, a Chinese website for online shopping
Seeking Shell Flagship Store	Battery Repairer	Rip-off stuff, not only the phone battery did not repair, but instead a phone completely scrapped!	Taobao Marketplace, a Chinese website for online shopping
Seeking Shell Flagship Store	Battery Repairer	genuine goods at fair prices	Taobao Marketplace, a Chinese website for online shopping
Seeking Shell Flagship Store	Battery Repairer	Not good for that!	Taobao Marketplace, a Chinese website for online shopping
Seeking Shell Flagship Store	Battery Repairer	It doesn't work. It's a lie.	Taobao Marketplace, a Chinese website for online shopping
Seeking Shell Flagship Store	Battery Repairer	Apple phone charging used20days, and eulogized a little, does not work	Taobao Marketplace, a Chinese website for online shopping
Seeking Shell Flagship Store	Battery Repairer	Nice, satisfied.	Taobao Marketplace, a Chinese website for online shopping
Seeking Shell Flagship Store	Battery Repairer	After using it for so many days, I don't feel like it's doing anything, whether it's an IQ tax or not I don't know, and when I asked the owner, he couldn't tell me.	Taobao Marketplace, a Chinese website for online shopping
Seeking Shell Flagship Store	Battery Repairer	an excellent	Taobao Marketplace, a Chinese website for online shopping
Seeking Shell Flagship Store	Battery Repairer	dissatisfied	Taobao Marketplace, a Chinese website for online shopping
Seeking Shell Flagship Store	Battery Repairer	It's what I want.	Taobao Marketplace, a Chinese website for online shopping
Seeking Shell Flagship Store	Battery Repairer	Fast logistics, some restorative effects, five stars.	Taobao Marketplace, a Chinese website for online shopping
Seeking Shell Flagship Store	Battery Repairer	Its amazingthat you can fix an Apple7base generation fryer	Taobao Marketplace, a Chinese website for online shopping
Seeking Shell Flagship Store	Battery Repairer	of no use	Taobao Marketplace, a Chinese website for online shopping
Seeking Shell Flagship Store	Battery Repairer	Doesn't work well, doesn't work	Taobao Marketplace, a Chinese website for online shopping
Seeking Shell Flagship Store	Battery Repairer	I don't know how effective it is, use it for a while and see	Taobao Marketplace, a Chinese website for online shopping
Seeking Shell Flagship Store	Battery Repairer	can	Taobao Marketplace, a Chinese website for online shopping
Seeking Shell Flagship Store	Battery Repairer	I bought and used a few days, no effect at all, and now I have thrown it aside. Three no products, not even a manual. Pure fraud. Don't be fooled again!	Taobao Marketplace, a Chinese website for online shopping
Seeking Shell Flagship Store	Battery Repairer	Received the goods! Quite satisfied! Thanks!	Taobao Marketplace, a Chinese website for online shopping
Seeking Shell Flagship Store	Battery Repairer	Feeling ripped off, posing as abar**like card	Taobao Marketplace, a Chinese website for online shopping
Seeking Shell Flagship Store	Battery Repairer	Very satisfied with the store service, charging the phone normally, worth having!	Taobao Marketplace, a Chinese website for online shopping
Seeking Shell Flagship Store	Battery Repairer	A basically useless thing that doesn't work, anyone who purchased this product is paying an IQ tax.	Taobao Marketplace, a Chinese website for online shopping
Seeking Shell Flagship Store	Battery Repairer	Able to repair the battery and increase standby time, good product, worth buying	Taobao Marketplace, a Chinese website for online shopping
Seeking Shell Flagship Store	Battery Repairer	An experience of paying the IQ tax! Speechless!	Taobao Marketplace, a Chinese website for online shopping

Seeking Shell Flagship Store	Battery Repairer	I'm using it, just to see if it works	Taobao Marketplace, a Chinese website for online shopping
Seeking Shell Flagship Store	Battery Repairer	Goods received, packaging effect is very good, very delicate, logistics is very fast, satisfied!	Taobao Marketplace, a Chinese website for online shopping
Seeking Shell Flagship Store	Battery Repairer	serviceable	Taobao Marketplace, a Chinese website for online shopping
Seeking Shell Flagship Store	Battery Repairer	It doesn't help at all.	Taobao Marketplace, a Chinese website for online shopping
Seeking Shell Flagship Store	Battery Repairer	It doesn't help at all.	Taobao Marketplace, a Chinese website for online shopping
Seeking Shell Flagship Store	Battery Repairer	No effect at all, instead of charging hot, strongly request to return the product	Taobao Marketplace, a Chinese website for online shopping
Seeking Shell Flagship Store	Battery Repairer	Haven't used it yet, don't know how it works	Taobao Marketplace, a Chinese website for online shopping
Seeking Shell Flagship Store	Battery Repairer	Scammy stuff. Not recommended to buy. This thing is just a power monitor, not a battery replicator.	Taobao Marketplace, a Chinese website for online shopping
Seeking Shell Flagship Store	Battery Repairer	No response yet, wait a little longer.	Taobao Marketplace, a Chinese website for online shopping
Seeking Shell Flagship Store	Battery Repairer	It's no good. It's no use.	Taobao Marketplace, a Chinese website for online shopping
Seeking Shell Flagship Store	Battery Repairer	A proper IQ tax. It won't work to fix the battery.	Taobao Marketplace, a Chinese website for online shopping
Seeking Shell Flagship Store	Battery Repairer	Habitual praise	Taobao Marketplace, a Chinese website for online shopping
Seeking Shell Flagship Store	Battery Repairer	can	Taobao Marketplace, a Chinese website for online shopping
Seeking Shell Flagship Store	Battery Repairer	Cell Phone Battery Repair Magic Received Looking forward to the results	Taobao Marketplace, a Chinese website for online shopping
Seeking Shell Flagship Store	Battery Repairer	Use and then experience the feeling, now there is no special, packaging is tight, the courier is also strong.	Taobao Marketplace, a Chinese website for online shopping
Seeking Shell Flagship Store	Battery Repairer	I haven't used it yet, I'll come back with a follow-up comment after I use it	Taobao Marketplace, a Chinese website for online shopping
Seeking Shell Flagship Store	Battery Repairer	Good review!	Taobao Marketplace, a Chinese website for online shopping
Seeking Shell Flagship Store	Battery Repairer	Looking forward to the results	Taobao Marketplace, a Chinese website for online shopping
Seeking Shell Flagship Store	Battery Repairer	(of an unmarried couple) be close	Taobao Marketplace, a Chinese website for online shopping
Seeking Shell Flagship Store	Battery Repairer	Received the baby, the second time to buy, very good	Taobao Marketplace, a Chinese website for online shopping
Seeking Shell Flagship Store	Battery Repairer	Does it work or not, I can't believe online shopping now.	Taobao Marketplace, a Chinese website for online shopping
Seeking Shell Flagship Store	Battery Repairer	Not as good as described, use it for a while and see what happens.	Taobao Marketplace, a Chinese website for online shopping
Seeking Shell Flagship Store	Battery Repairer	I'm using it to see how it works and then I'll review it.	Taobao Marketplace, a Chinese website for online shopping
Seeking Shell Flagship Store	Battery Repairer	It seems basically useless!	Taobao Marketplace, a Chinese website for online shopping
Seeking Shell Flagship Store	Battery Repairer	(of an unmarried couple) be close	Taobao Marketplace, a Chinese website for online shopping
Seeking Shell Flagship Store	Battery Repairer	Not sure what it does, but it's durable.	Taobao Marketplace, a Chinese website for online shopping
Seeking Shell Flagship Store	Battery Repairer	review after use	Taobao Marketplace, a Chinese website for online shopping
Seeking Shell Flagship Store	Battery Repairer	I'm used to good reviews, I didn't feel any restorative effects.	Taobao Marketplace, a Chinese website for online shopping
Seeking Shell Flagship Store	Battery Repairer	It doesn't seem to be working very well.	Taobao Marketplace, a Chinese website for online shopping
Seeking Shell Flagship Store	Battery Repairer	There is no difference between the item and the picture, the charging rate and just bought the item charged with nothing at all, it is simply deceptive.	Taobao Marketplace, a Chinese website for online shopping
Seeking Shell Flagship Store	Battery Repairer	As described, there is no switch on the side, you can only pull it out if you want to turn it off	Taobao Marketplace, a Chinese website for online shopping
Seeking Shell Flagship Store	Battery Repairer	It's useless. It's all bragging.	Taobao Marketplace, a Chinese website for online shopping
Seeking Shell Flagship Store	Battery Repairer	Roger that. - Thanks! Used it for two days now. It's pretty good. Continue to use. Thanks to the store👍👍👍👍	Taobao Marketplace, a Chinese website for online shopping
Seeking Shell Flagship Store	Battery Repairer	The effect is not obvious.	Taobao Marketplace, a Chinese website for online shopping
Seeking Shell Flagship Store	Battery Repairer	It's working!	Taobao Marketplace, a Chinese website for online shopping
Seeking Shell Flagship Store	Battery Repairer	It's okay to use it.	Taobao Marketplace, a Chinese website for online shopping
Seeking Shell Flagship Store	Battery Repairer	faster than the original, the product is exquisite workmanship, beautiful, good quality. Thank you for the seller's baby, the seller has a good service attitude, praise one.	Taobao Marketplace, a Chinese website for online shopping
Seeking Shell Flagship Store	Battery Repairer	Already started using it Waiting for results Hopefully the charge will last longer	Taobao Marketplace, a Chinese website for online shopping

Seeking Shell Flagship Store	Battery Repairer	It feels like something that turns fast charging into slow charging.	Taobao Marketplace, a Chinese website for online shopping
Seeking Shell Flagship Store	Battery Repairer	I've used it many times and only now I'm evaluating it, it does have a stabilizing effect when charging, but repairing a damaged battery is not perceptible.	Taobao Marketplace, a Chinese website for online shopping
Seeking Shell Flagship Store	Battery Repairer	No effect, this kind of pure fraud! You do not fall for it!	Taobao Marketplace, a Chinese website for online shopping
Seeking Shell Flagship Store	Battery Repairer	I've been using it for two or three days and I can't see any results so far.	Taobao Marketplace, a Chinese website for online shopping
Seeking Shell Flagship Store	Battery Repairer	Nothing works.	Taobao Marketplace, a Chinese website for online shopping
Seeking Shell Flagship Store	Battery Repairer	Just received is using it should be good. Waiting for a follow up review.	Taobao Marketplace, a Chinese website for online shopping
Seeking Shell Flagship Store	Battery Repairer	I don't know.	Taobao Marketplace, a Chinese website for online shopping
Seeking Shell Flagship Store	Battery Repairer	OK	Taobao Marketplace, a Chinese website for online shopping
Seeking Shell Flagship Store	Battery Repairer	Used twice and normal charging the same, feel a little effect than before using standby time has improved. Stick to the end of use to see the effect is estimated to be good.	Taobao Marketplace, a Chinese website for online shopping
Seeking Shell Flagship Store	Battery Repairer	I've received the item, let's use it for a while and then we'll follow up.	Taobao Marketplace, a Chinese website for online shopping
Seeking Shell Flagship Store	Battery Repairer	Repairing the battery is not very effective	Taobao Marketplace, a Chinese website for online shopping
Seeking Shell Flagship Store	Battery Repairer	Logistics, service attitude is good. But the product has no manual and no origin, which makes people feel cheated, or ask the dealer to explain.	Taobao Marketplace, a Chinese website for online shopping
Seeking Shell Flagship Store	Battery Repairer	Shipping was quick, no comment as to whether the battery was repaired or not.	Taobao Marketplace, a Chinese website for online shopping
Seeking Shell Flagship Store	Battery Repairer	I was apprehensive before I received it, but I tried it out immediately after receiving it, and it's really good!	Taobao Marketplace, a Chinese website for online shopping
Seeking Shell Flagship Store	Battery Repairer	used it twice now and it hasn't fixed anything, as usual, when I charge it, it prompts me that the battery needs to be serviced.	Taobao Marketplace, a Chinese website for online shopping
Seeking Shell Flagship Store	Battery Repairer	very happy	Taobao Marketplace, a Chinese website for online shopping
Seeking Shell Flagship Store	Battery Repairer	Arrived quickly, the product is exquisite, the effect of how to wait tosee_x005f_x0001_service is not a problem	Taobao Marketplace, a Chinese website for online shopping
Seeking Shell Flagship Store	Battery Repairer	Baby received, hopefully worth the money.	Taobao Marketplace, a Chinese website for online shopping
Seeking Shell Flagship Store	Battery Repairer	fast delivery	Taobao Marketplace, a Chinese website for online shopping
Seeking Shell Flagship Store	Battery Repairer	Can not repair the battery and the business is not the same as the introduction of There is no use ah can not turn on the phone how to charge also still can not turn on The battery is either bad or put more than a year only Suggest that I see my evaluation do not buy This is just a means of earning money	Taobao Marketplace, a Chinese website for online shopping
Seeking Shell Flagship Store	Battery Repairer	After2days ofuse, the phone no longer gets hot while charging, other effects to be discovered in the future.	Taobao Marketplace, a Chinese website for online shopping
Seeking Shell Flagship Store	Battery Repairer	I haven't used it yet, so let's give it a good review first.	Taobao Marketplace, a Chinese website for online shopping
Seeking Shell Flagship Store	Battery Repairer	It does charge fast, the power like enough to use for a long time. It is worth buying. The courier arrived the next day very quickly. Thank you.	Taobao Marketplace, a Chinese website for online shopping
Seeking Shell Flagship Store	Battery Repairer	bad, what, buy less is looked down on ah! Then why do you still sell a single, close it early, so as not to disgust people again!	Taobao Marketplace, a Chinese website for online shopping
Seeking Shell Flagship Store	Battery Repairer	(of an unmarried couple) be close	Taobao Marketplace, a Chinese website for online shopping
Seeking Shell Flagship Store	Battery Repairer	Give it a good review, tried it for a while, not much change, but the phone is less hot.	Taobao Marketplace, a Chinese website for online shopping
Seeking Shell Flagship Store	Battery Repairer	It didn't work, got ripped off.	Taobao Marketplace, a Chinese website for online shopping
Seeking Shell Flagship Store	Battery Repairer	Already used Very good	Taobao Marketplace, a Chinese website for online shopping
Seeking Shell Flagship Store	Battery Repairer	while now, it doesn't work, plugging it in is redundant! Not recommended.	Taobao Marketplace, a Chinese website for online shopping
Seeking Shell Flagship Store	Battery Repairer	It's taxable.	Taobao Marketplace, a Chinese website for online shopping
Seeking Shell Flagship Store	Battery Repairer	Truly useless!	Taobao Marketplace, a Chinese website for online shopping
Seeking Shell Flagship Store	Battery Repairer	Baby has received, how exactly to use after the follow-up comment.	Taobao Marketplace, a Chinese website for online shopping
Seeking Shell Flagship Store	Battery Repairer	It's really effective and easy to use.	Taobao Marketplace, a Chinese website for online shopping
Seeking Shell Flagship Store	Battery Repairer	A proper IQ tax!	Taobao Marketplace, a Chinese website for online shopping

Seeking Shell Flagship Store	Battery Repairer	Repairing the battery for another 10years is pure****, don't believe their bullshit, I've been using it for a long time now and it's not working.	Taobao Marketplace, a Chinese website for online shopping
Seeking Shell Flagship Store	Battery Repairer	Already used it and how it works to see what happens.	Taobao Marketplace, a Chinese website for online shopping
Seeking Shell Flagship Store	Battery Repairer	It doesn't work. It's a scam. I fell for it.	Taobao Marketplace, a Chinese website for online shopping
Seeking Shell Flagship Store	Battery Repairer	Very good👍Shipped quickly and well packaged. Product quality is very good👍good review	Taobao Marketplace, a Chinese website for online shopping
Seeking Shell Flagship Store	Battery Repairer	Very good👍Shipped quickly and well packaged. Product quality is very good👍good review	Taobao Marketplace, a Chinese website for online shopping
Seeking Shell Flagship Store	Battery Repairer	Try it, there's one where you can at least see the current voltage. But, the miracle hasn't happened yet.	Taobao Marketplace, a Chinese website for online shopping
Seeking Shell Flagship Store	Battery Repairer	No effect No effect No improvement Threw it away for fear of hurting the machine	Taobao Marketplace, a Chinese website for online shopping
Seeking Shell Flagship Store	Battery Repairer	Totally trippy product. Doesn't work at all.	Taobao Marketplace, a Chinese website for online shopping
Seeking Shell Flagship Store	Battery Repairer	I've been using it for so long and it's not working.	Taobao Marketplace, a Chinese website for online shopping
Seeking Shell Flagship Store	Battery Repairer	1	Taobao Marketplace, a Chinese website for online shopping
Seeking Shell Flagship Store	Battery Repairer	favorable criticism	Taobao Marketplace, a Chinese website for online shopping
Seeking Shell Flagship Store	Battery Repairer	Fine workmanship. Socket is tight and not hot. Not bad.	Taobao Marketplace, a Chinese website for online shopping
Seeking Shell Flagship Store	Battery Repairer	Received and in use, looking forward to the effect	Taobao Marketplace, a Chinese website for online shopping
Seeking Shell Flagship Store	Battery Repairer	miracle of a fix that I can't be sure of until three months later.	Taobao Marketplace, a Chinese website for online shopping
Seeking Shell Flagship Store	Battery Repairer	Haven't used it yet, trust the seller's description.	Taobao Marketplace, a Chinese website for online shopping
Seeking Shell Flagship Store	Battery Repairer	It works great!	Taobao Marketplace, a Chinese website for online shopping
Seeking Shell Flagship Store	Battery Repairer	I feel like it's working a little bit.	Taobao Marketplace, a Chinese website for online shopping
Seeking Shell Flagship Store	Battery Repairer	Good stuff, fast logistics, hope it works, looking forward to the effect!	Taobao Marketplace, a Chinese website for online shopping
Seeking Shell Flagship Store	Battery Repairer	favorable criticism	Taobao Marketplace, a Chinese website for online shopping
Seeking Shell Flagship Store	Battery Repairer	Copy that. It's good!	Taobao Marketplace, a Chinese website for online shopping
Seeking Shell Flagship Store	Battery Repairer	Got it. Thanks, boss.	Taobao Marketplace, a Chinese website for online shopping
Seeking Shell Flagship Store	Battery Repairer	It's not working.	Taobao Marketplace, a Chinese website for online shopping
Seeking Shell Flagship Store	Battery Repairer	favorable criticism	Taobao Marketplace, a Chinese website for online shopping
Seeking Shell Flagship Store	Battery Repairer	Baby has arrived today, has not yet been used, do not know how the effect, such as the use of in the follow-up comment.	Taobao Marketplace, a Chinese website for online shopping
Seeking Shell Flagship Store	Battery Repairer	ve been using it for a couple days and it feels like the same**. Shipping was pretty quick. No labels, certificates of compliance, manuals, etc.	Taobao Marketplace, a Chinese website for online shopping
Seeking Shell Flagship Store	Battery Repairer	The data cable is very good quality, soft and non-tangle 3heads color differentiation easy to use	Taobao Marketplace, a Chinese website for online shopping
Seeking Shell Flagship Store	Battery Repairer	Have not yet used, after using in the follow-up review oh.	Taobao Marketplace, a Chinese website for online shopping
Seeking Shell Flagship Store	Battery Repairer	Received the item, still testing it	Taobao Marketplace, a Chinese website for online shopping
Seeking Shell Flagship Store	Battery Repairer	No effect, surface scratches, obviously a re-sale item	Taobao Marketplace, a Chinese website for online shopping
Seeking Shell Flagship Store	Battery Repairer	Not bad, it's working.	Taobao Marketplace, a Chinese website for online shopping
Seeking Shell Flagship Store	Battery Repairer	It doesn't do much good.	Taobao Marketplace, a Chinese website for online shopping
Seeking Shell Flagship Store	Battery Repairer	The previous Huawei phone always mentioned that my phone battery performance declined, as soon as possible to customer service testing! After using this baby, it no longer prompts. Just haven't obviously felt the standby time is longer, try it again for a while!Try iton66Wfast charger, it doesn't support fast charging, you should use it on a regular charger.	Taobao Marketplace, a Chinese website for online shopping
Seeking Shell Flagship Store	Battery Repairer	Not much use.	Taobao Marketplace, a Chinese website for online shopping
Seeking Shell Flagship Store	Battery Repairer	in use	Taobao Marketplace, a Chinese website for online shopping
Seeking Shell Flagship Store	Battery Repairer	Baby received, used a few days the effect has improved, satisfied.	Taobao Marketplace, a Chinese website for online shopping

Seeking Shell Flagship Store	Battery Repairer	The goods have been received, and the arrival package is intact. Used a few days to feel repaired, delayed cell phone use time function. Previously used two other, the effect is not as good as this. In short, it is worth buying.	Taobao Marketplace, a Chinese website for online shopping
Seeking Shell Flagship Store	Battery Repairer	Great quality, affordable, easy to use and good.	Taobao Marketplace, a Chinese website for online shopping
Seeking Shell Flagship Store	Battery Repairer	It works. I don't know about the repair function.	Taobao Marketplace, a Chinese website for online shopping
Seeking Shell Flagship Store	Battery Repairer	It is being used slowly! Effectiveness to be tested	Taobao Marketplace, a Chinese website for online shopping
Seeking Shell Flagship Store	Battery Repairer	First time buyer don't know how well it works, try it first	Taobao Marketplace, a Chinese website for online shopping
Seeking Shell Flagship Store	Battery Repairer	Trying it out, not sure how well it works. Hope it really works.	Taobao Marketplace, a Chinese website for online shopping
Seeking Shell Flagship Store	Battery Repairer	It doesn't do anything! IQ tax identified.	Taobao Marketplace, a Chinese website for online shopping
Seeking Shell Flagship Store	Battery Repairer	It's not working.	Taobao Marketplace, a Chinese website for online shopping
Seeking Shell Flagship Store	Battery Repairer	Nothing works, though it's not expensive, it's still a bit upsetting.	Taobao Marketplace, a Chinese website for online shopping
Seeking Shell Flagship Store	Battery Repairer	The messenger is ok and should have a restorative effect on the battery.	Taobao Marketplace, a Chinese website for online shopping
Seeking Shell Flagship Store	Battery Repairer	exact same, very satisfied, really like, completely exceeded expectations, the delivery speed is very fast, the packaging is very careful, strict, the logistics company has a very good service attitude, the delivery speed is very fast, very satisfied with the shopping!	Taobao Marketplace, a Chinese website for online shopping
Seeking Shell Flagship Store	Battery Repairer	very fast, express delivery is strong thank you boss to give full stars.	Taobao Marketplace, a Chinese website for online shopping
Seeking Shell Flagship Store	Battery Repairer	Hard to say good or bad. Dead, old batteries can't be fixed, and generally cell phones are still the same after charging and fixing, don't think it's much of an improvement. I don't know how others use it.	Taobao Marketplace, a Chinese website for online shopping
Seeking Shell Flagship Store	Battery Repairer	It's an IQ tax. It's useless. Whoever buys it regrets it.	Taobao Marketplace, a Chinese website for online shopping
Seeking Shell Flagship Store	Battery Repairer	Can't see the effect at the moment. Waiting for a few days to add	Taobao Marketplace, a Chinese website for online shopping
Seeking Shell Flagship Store	Battery Repairer	The goods have been received and will be evaluated after use.	Taobao Marketplace, a Chinese website for online shopping
Seeking Shell Flagship Store	Battery Repairer	Received the goods, packaging is very good, things compact, good value, stock up on good, kudos.	Taobao Marketplace, a Chinese website for online shopping
Seeking Shell Flagship Store	Battery Repairer	First praise, in the trial, if the use of matters according to customer service said if the effect, I will come back to the follow-up review, and then recommend the purchase.	Taobao Marketplace, a Chinese website for online shopping
Seeking Shell Flagship Store	Battery Repairer	Can't see the effect	Taobao Marketplace, a Chinese website for online shopping
Seeking Shell Flagship Store	Battery Repairer	The customer service lady was very patient in answering questions and speaking very carefully.	Taobao Marketplace, a Chinese website for online shopping
Seeking Shell Flagship Store	Battery Repairer	Very good quality, very satisfied, value for money, worth buying, value for money	Taobao Marketplace, a Chinese website for online shopping
Seeking Shell Flagship Store	Battery Repairer	(of an unmarried couple) be close	Taobao Marketplace, a Chinese website for online shopping
Seeking Shell Flagship Store	Battery Repairer	It's really quite useful yea✌, after fixing it, the phone has a longer standby time, and it's also easy to operate, it's quite good and inexpensive.	Taobao Marketplace, a Chinese website for online shopping
Seeking Shell Flagship Store	Battery Repairer	I haven't seen the results yet, so I'll give you a good review first.	Taobao Marketplace, a Chinese website for online shopping
Seeking Shell Flagship Store	Battery Repairer	Fooled, the cell phone battery is still the same, no repair at all.	Taobao Marketplace, a Chinese website for online shopping
Seeking Shell Flagship Store	Battery Repairer	I'll review after I use it, 5 stars first	Taobao Marketplace, a Chinese website for online shopping
Seeking Shell Flagship Store	Battery Repairer	I hope it works.	Taobao Marketplace, a Chinese website for online shopping
Seeking Shell Flagship Store	Battery Repairer	Doesn't help at all, was80before,still80aftercharging with this for well over3overnight. flaky stuff, what's with all the positive reviews?	Taobao Marketplace, a Chinese website for online shopping
Seeking Shell Flagship Store	Battery Repairer	This thing charges the phone a few times and feels like it doesn't work at all, I feel like I'm being ignored	Taobao Marketplace, a Chinese website for online shopping
Seeking Shell Flagship Store	Battery Repairer	Looking forward to the results	Taobao Marketplace, a Chinese website for online shopping
Seeking Shell Flagship Store	Battery Repairer	Great quality, affordable, sturdy, and value for money!	Taobao Marketplace, a Chinese website for online shopping
Seeking Shell Flagship Store	Battery Repairer	It's all new, haven't started using it yet, don't know if it's as god as advertised, good to be praising.	Taobao Marketplace, a Chinese website for online shopping
Seeking Shell Flagship Store	Battery Repairer	Feels like paying an IQ tax! Not recommended to buy!	Taobao Marketplace, a Chinese website for online shopping
Seeking Shell Flagship Store	Battery Repairer	It doesn't feel like much.	Taobao Marketplace, a Chinese website for online shopping

Seeking Shell Flagship Store	Battery Repairer	Great quality and great value. The cell phone battery gets a big boost with this.	Taobao Marketplace, a Chinese website for online shopping
Seeking Shell Flagship Store	Battery Repairer	The logistics is very fast Just arrived Use it first to try to see if it is good We will review it later	Taobao Marketplace, a Chinese website for online shopping
Seeking Shell Flagship Store	Battery Repairer	Recommended by a friend, looks pretty good, looking forward to the results!	Taobao Marketplace, a Chinese website for online shopping
Seeking Shell Flagship Store	Battery Repairer	It didn't work, just an ammeter and a voltmeter.	Taobao Marketplace, a Chinese website for online shopping
Seeking Shell Flagship Store	Battery Repairer	The goods received, especially like, once received, can not wait to open, the family is also very satisfied, this shopping experience is good!	Taobao Marketplace, a Chinese website for online shopping
Seeking Shell Flagship Store	Battery Repairer	normally, as for the repair effect on the battery how, because only use, no right to speak, to be used for a period of time after the follow-up.	Taobao Marketplace, a Chinese website for online shopping
Seeking Shell Flagship Store	Battery Repairer	I've been looking for a long time, I finally bought it, the quality is fine and it's easy to use.	Taobao Marketplace, a Chinese website for online shopping
Seeking Shell Flagship Store	Battery Repairer	Very compact one, easy to carry and use. The restorer is stable in charging and very practical.	Taobao Marketplace, a Chinese website for online shopping
Seeking Shell Flagship Store	Battery Repairer	The old cell phone battery is not working, use this to restore the effect is still good!	Taobao Marketplace, a Chinese website for online shopping
Seeking Shell Flagship Store	Battery Repairer	I tried it and didn't get the advertised results yet	Taobao Marketplace, a Chinese website for online shopping
Seeking Shell Flagship Store	Battery Repairer	Originally do not intend to use to get this, the effect is the same as the introduction, good can also👍	Taobao Marketplace, a Chinese website for online shopping
Seeking Shell Flagship Store	Battery Repairer	Good quality and fine workmanship	Taobao Marketplace, a Chinese website for online shopping
Seeking Shell Flagship Store	Battery Repairer	It really works.	Taobao Marketplace, a Chinese website for online shopping
Seeking Shell Flagship Store	Battery Repairer	Quality is very over the top, quality is guaranteed, quite a good choice, a lot of friends to see me use all want to buy, I recommend it!	Taobao Marketplace, a Chinese website for online shopping
Seeking Shell Flagship Store	Battery Repairer	the appearance and workmanship is very satisfied, and the business description is consistent with the customer service attitude is very good, the account is very clear, the use of charging effect is good, the speed is also very fast, the quality is very good, this does not have to change the battery, thanks to the business.	Taobao Marketplace, a Chinese website for online shopping
Seeking Shell Flagship Store	Battery Repairer	Shipping was fast, received it quickly, worked well as the seller said, and has a restorative effect on the battery!	Taobao Marketplace, a Chinese website for online shopping
Seeking Shell Flagship Store	Battery Repairer	Baby received, love it, worth buying.	Taobao Marketplace, a Chinese website for online shopping
Seeking Shell Flagship Store	Battery Repairer	Haven't started using it yet.	Taobao Marketplace, a Chinese website for online shopping
Seeking Shell Flagship Store	Battery Repairer	Things received, the product appearance is cool, multi-device compatibility, especially after powering up is very beautiful, physical and description consistent, good quality, easy to use, the effect is good, the cell phone battery repair is improved, it is worth having. Favorable comments Oh, wish the boss a prosperous business.	Taobao Marketplace, a Chinese website for online shopping
Seeking Shell Flagship Store	Battery Repairer	battery is very durable. It is genuine. Logistics is also very fast, business service attitude is good, very satisfied, worth buying!	Taobao Marketplace, a Chinese website for online shopping
Seeking Shell Flagship Store	Battery Repairer	The logistics is very fast, the quality of the goods is very good, the magic weapon to repair the cell phone battery, but also a good fast charger, does not hurt the phone, fast charging, the appearance is very beautiful, but also equipped with a display, it is very convenient to use. The same as described on the product introduction, and the logistics is very fast, thoughtful service, praise.	Taobao Marketplace, a Chinese website for online shopping
Seeking Shell Flagship Store	Battery Repairer	I just started using it and I don't know how well it works.	Taobao Marketplace, a Chinese website for online shopping
Seeking Shell Flagship Store	Battery Repairer	It matches the description. It's good.	Taobao Marketplace, a Chinese website for online shopping
Seeking Shell Flagship Store	Battery Repairer	quite good, and the same as described by the seller, the business service is very attentive, this product can extend the battery life and standby time, this product praise, very satisfied.	Taobao Marketplace, a Chinese website for online shopping
Seeking Shell Flagship Store	Battery Repairer	through the trial did find that can effectively repair the old cell phone old battery, is the old cell phone old battery protector cell phone used after also not hot, with a long time, it is worth buying.	Taobao Marketplace, a Chinese website for online shopping
Seeking Shell Flagship Store	Battery Repairer	The battery does last a bit longer after use, recommended	Taobao Marketplace, a Chinese website for online shopping
Seeking Shell Flagship Store	Battery Repairer	Cell phone battery repairer received, logistics is very fast, the physical look is very good, fine workmanship, the effect of gradual recovery of the cell phone battery, very like.	Taobao Marketplace, a Chinese website for online shopping

Seeking Shell Flagship Store	Battery Repairer	out of use for a long time and can't be charged properly can activate the wake-up and charge normally). Battery Repair (battery capacity greatly reduced) function is still in testing.	Taobao Marketplace, a Chinese website for online shopping
Seeking Shell Flagship Store	Battery Repairer	I just received it and haven't used it yet? How effective is it yet to know? Waiting for evaluation	Taobao Marketplace, a Chinese website for online shopping
Seeking Shell Flagship Store	Battery Repairer	by buying this? I've been using it for a long time and it's draining the battery just like before? It doesn't work at all ？？？？	Taobao Marketplace, a Chinese website for online shopping
Seeking Shell Flagship Store	Battery Repairer	particularly difficult to repair cell phone stores to replace the battery are very expensive, with this cell phone battery repairer, no longer need to spend money, and do not have to beg, since they can do, not only charging stable in the fast, but also to care for cell phone batteries do not hurt the phone, the use of security is also reliable, there is a need for you to get it.	Taobao Marketplace, a Chinese website for online shopping
Seeking Shell Flagship Store	Battery Repairer	The effect is very good, samsungnote8repair effect is very good, this time do not have to change the battery, after repairing the phone with a longer time some, not heat, and also automatically clean up the cell phone system cache garbage, the phone has a significantly smoother, the boss said that often used to repair will be better	Taobao Marketplace, a Chinese website for online shopping
Seeking Shell Flagship Store	Battery Repairer	consistent with the description, cost-effective, good quality, compact and convenient, full of technology, charging speed is very fast, the cell phone battery repair is a great help, customer service attitude is particularly good, I like it, the next time I will buy back.	Taobao Marketplace, a Chinese website for online shopping
Seeking Shell Flagship Store	Battery Repairer	really can repair the old cell phone battery, in the past only40%, now89%, and the repairer is stable charging, clear display, and has a certain repair function for the battery. The cell phone is much smoother than before, it's really good value for money.	Taobao Marketplace, a Chinese website for online shopping
Seeking Shell Flagship Store	Battery Repairer	Already fixed several phones, Apple and Android can be used, my family's cell phone let me fix once, and now it can be used when usually charging.	Taobao Marketplace, a Chinese website for online shopping
Seeking Shell Flagship Store	Battery Repairer	Logistics and packaging are fine, quality is good, price is ok, customer service is good.	Taobao Marketplace, a Chinese website for online shopping
Seeking Shell Flagship Store	Battery Repairer	I'm very happy with the fact that I can fix it myself at home without having to go to a cell phone store and get screwed out of money.	Taobao Marketplace, a Chinese website for online shopping
Seeking Shell Flagship Store	Battery Repairer	use a lot of days, there is a screen display, according to customer service guidance to insert the charging head to connect the data line can be slow charging power consumption fast or charging into the power of the cell phone repair, but also one time to repair the two cell phones, the first time to repair the phone shutdown, used after the effect of the effect is indeed, and now my two cell phones do not have the previous power consumption.	Taobao Marketplace, a Chinese website for online shopping
Seeking Shell Flagship Store	Battery Repairer	See small sweet potato recommended, I this cell phone a day loss of power consumption is too fast, with this cell phone battery repairer, the battery amount restored to more than90%, truly recommended to the need of people.	Taobao Marketplace, a Chinese website for online shopping
Seeking Shell Flagship Store	Battery Repairer	This repairer used the phone directly after the newborn, very good, the phone will not be hot, the response are becoming sensitive, have the ability to do it all can be completed, the battery is quite durable, the capacity is particularly large, the response effect is also super snappy, the phone will not appear lag phenomenon, the charging effect is also super fast, the use of particularly convenient, praise.	Taobao Marketplace, a Chinese website for online shopping
Seeking Shell Flagship Store	Battery Repairer	could only be plugged into the power supply to boot, with this charge a few times, you can leave the power supply to boot a eight or nine hours, there has been a big progress, in more than a few times will be better, customer service attitude is also very good, sent me the use of the method, very satisfied with o.	Taobao Marketplace, a Chinese website for online shopping
Seeking Shell Flagship Store	Battery Repairer	After customer service explanation and use now can, the battery capacity can be charged to 100%, things are very good thank you boss	Taobao Marketplace, a Chinese website for online shopping
Seeking Shell Flagship Store	Battery Repairer	I've just used it twice, it feels good, my phone's battery doesn't drop as fast, so I'll use it again.	Taobao Marketplace, a Chinese website for online shopping
Seeking Shell Flagship Store	Battery Repairer	Goods received good quality worth buying	Taobao Marketplace, a Chinese website for online shopping
Seeking Shell Flagship Store	Battery Repairer	Repair device quickly received, workmanship is relatively fine, just used a little, can be charged, if you can repair the damaged appliances that would be worth it, first to give a favorable review, not the back of the follow-up!	Taobao Marketplace, a Chinese website for online shopping
Seeking Shell Flagship Store	Battery Repairer	Baby received, small and exquisite, it works well	Taobao Marketplace, a Chinese website for online shopping

Seeking Shell Flagship Store	Battery Repairer	Fast shipping, three types of interface is very practical, no longer need for no suitable charging cable headache, tried charging is also fast!	Taobao Marketplace, a Chinese website for online shopping
Seeking Shell Flagship Store	Battery Repairer	💌finally made a move on it, the face value is really no words❤, the material is very good, good value, take advantage of the event to stock up	Taobao Marketplace, a Chinese website for online shopping
Seeking Shell Flagship Store	Battery Repairer	Received the goods, the quality is good, the next need to come back to buy!	Taobao Marketplace, a Chinese website for online shopping
Seeking Shell Flagship Store	Battery Repairer	Got it. It's good quality.	Taobao Marketplace, a Chinese website for online shopping
Seeking Shell Flagship Store	Battery Repairer	Does this work well for those who have bought it? Does it work for those who have used it?	Taobao Marketplace, a Chinese website for online shopping
Seeking Shell Flagship Store	Battery Repairer	Isopphone an android only model?	Taobao Marketplace, a Chinese website for online shopping
Seeking Shell Flagship Store	Battery Repairer	Anyone who has bought it, honestly does it really work?	Taobao Marketplace, a Chinese website for online shopping
Seeking Shell Flagship Store	Battery Repairer	Are you paying an IQ tax to buy this?	Taobao Marketplace, a Chinese website for online shopping
Seeking Shell Flagship Store	Battery Repairer	Is it a product that pays an IQ tax?	Taobao Marketplace, a Chinese website for online shopping
Seeking Shell Flagship Store	Battery Repairer	How's the restoration going?	Taobao Marketplace, a Chinese website for online shopping
Seeking Shell Flagship Store	Battery Repairer	I can go up five flights of stairs in one breath without any trouble.	Taobao Marketplace, a Chinese website for online shopping
Seeking Shell Flagship Store	Battery Repairer	You can get water out of the river even if you drain the pump.	Taobao Marketplace, a Chinese website for online shopping
Seeking Shell Flagship Store	Battery Repairer	Does it work? How does it work?	Taobao Marketplace, a Chinese website for online shopping
Seeking Shell Flagship Store	Battery Repairer	Does it really work?	Taobao Marketplace, a Chinese website for online shopping
Seeking Shell Flagship Store	Battery Repairer	Personally, I find it useful.	Taobao Marketplace, a Chinese website for online shopping
Seeking Shell Flagship Store	Battery Repairer	Works on both Android and Apple	Taobao Marketplace, a Chinese website for online shopping
Seeking Shell Flagship Store	Battery Repairer	Anyway, I've been using almost a month nowand it's not working	Taobao Marketplace, a Chinese website for online shopping
Seeking Shell Flagship Store	Battery Repairer	essentially	Taobao Marketplace, a Chinese website for online shopping
Seeking Shell Flagship Store	Battery Repairer	I've been using it for a few days and it's not working yet I'll know in a few days.	Taobao Marketplace, a Chinese website for online shopping
Seeking Shell Flagship Store	Battery Repairer	No visible effect	Taobao Marketplace, a Chinese website for online shopping
Seeking Shell Flagship Store	Battery Repairer	You're thinking too much.	Taobao Marketplace, a Chinese website for online shopping
Seeking Shell Flagship Store	Battery Repairer	I don't know what you're asking.	Taobao Marketplace, a Chinese website for online shopping
Seeking Shell Flagship Store	Battery Repairer	didn't work for me anyway But it's pretty techy to use	Taobao Marketplace, a Chinese website for online shopping
Seeking Shell Flagship Store	Battery Repairer	I've been using it for a couple of days now, and it's not really working.	Taobao Marketplace, a Chinese website for online shopping
Seeking Shell Flagship Store	Battery Repairer	This product is a good fit for its price point	Taobao Marketplace, a Chinese website for online shopping
Seeking Shell Flagship Store	Battery Repairer	The seller shipped quickly and the quality of the thread is excellent and recommended.	Taobao Marketplace, a Chinese website for online shopping
Seeking Shell Flagship Store	Battery Repairer	Convenient, good quality, good customer service and fast shipping!	Taobao Marketplace, a Chinese website for online shopping
Seeking Shell Flagship Store	Battery Repairer	Fast logistics Good service	Taobao Marketplace, a Chinese website for online shopping
Seeking Shell Flagship Store	Battery Repairer	very good	Taobao Marketplace, a Chinese website for online shopping
Seeking Shell Flagship Store	Battery Repairer	The packaging is very sturdy and the stuff is well protected, so it's worth a good review	Taobao Marketplace, a Chinese website for online shopping
Seeking Shell Flagship Store	Battery Repairer	The impulse cord is good and the logistics is fast	Taobao Marketplace, a Chinese website for online shopping
Seeking Shell Flagship Store	Battery Repairer	Good quality, charges pretty fast, works well, and the three heads are easy to use!	Taobao Marketplace, a Chinese website for online shopping
Seeking Shell Flagship Store	Battery Repairer	Great quality, 3 in 1, easy to use and extra fast charging!	Taobao Marketplace, a Chinese website for online shopping
Seeking Shell Flagship Store	Battery Repairer	charging does not heat up, easy to carry out of the house, fast delivery,👍	Taobao Marketplace, a Chinese website for online shopping
Seeking Shell Flagship Store	Battery Repairer	Fast shipping, the quality is pretty good, the price is really good! Charging is easy and works well, no more taking a bunch of chargers😋😋😋😋	Taobao Marketplace, a Chinese website for online shopping
Seeking Shell Flagship Store	Battery Repairer	Have been using this one, charging especially fast, the line thickness is also enough, the material is very solid, charging time will not be hot, very durable, very good data cable, next time will buy back!	Taobao Marketplace, a Chinese website for online shopping
Seeking Shell Flagship Store	Battery Repairer	Very good, fast charging	Taobao Marketplace, a Chinese website for online shopping

Seeking Shell Flagship Store	Battery Repairer	texture is quite good The charging speed is quite fast It works quite well	Taobao Marketplace, a Chinese website for online shopping
Seeking Shell Flagship Store	Battery Repairer	also very strong,customer service attitude is excellent, very patient, give a person a sense of affinity, good like. There are beautifully packaged, high-end atmosphere upscale;see the business is very attentive. Baby is really good, and the picture is consistent, there is no difference, it is really value for money, good value for money ah. The quality is very good, next time will again patronize!Express is very powerful, praise! Fast shipping cost-effective	Taobao Marketplace, a Chinese website for online shopping
Seeking Shell Flagship Store	Battery Repairer	Logistics is quite fast, packaging is tight, cheap, there is a need to come back!	Taobao Marketplace, a Chinese website for online shopping
Seeking Shell Flagship Store	Battery Repairer	The practicality and value are awesome, I love it and will be back for more!	Taobao Marketplace, a Chinese website for online shopping
Seeking Shell Flagship Store	Battery Repairer	Good quality, very satisfied. Charging is fast too.	Taobao Marketplace, a Chinese website for online shopping
Seeking Shell Flagship Store	Battery Repairer	Fast shipping good packaging, affordable cost-effective👌, good	Taobao Marketplace, a Chinese website for online shopping
Seeking Shell Flagship Store	Battery Repairer	Good quality, works well	Taobao Marketplace, a Chinese website for online shopping
Seeking Shell Flagship Store	Battery Repairer	Works great, charges fast	Taobao Marketplace, a Chinese website for online shopping
Seeking Shell Flagship Store	Battery Repairer	Baby received it👌, really great ah!🌈The quality can't be beat, very satisfied!🆗Happy happy happy😃	Taobao Marketplace, a Chinese website for online shopping
Seeking Shell Flagship Store	Battery Repairer	Received the goods, very good, the price is cheap straight enough to buy!	Taobao Marketplace, a Chinese website for online shopping
Seeking Shell Flagship Store	Battery Repairer	Recommended by a friend, very good	Taobao Marketplace, a Chinese website for online shopping
Seeking Shell Flagship Store	Battery Repairer	Baby received, very surprised, the quality is very good, very satisfied with a shopping. I will continue to buy back, the store is trustworthy!	Taobao Marketplace, a Chinese website for online shopping
Seeking Shell Flagship Store	Battery Repairer	The data cable charges the phone pretty fast and the color is nice.	Taobao Marketplace, a Chinese website for online shopping
Seeking Shell Flagship Store	Battery Repairer	Shipping was fast, the quality is genuinely good charging quickly, and it's recommended!	Taobao Marketplace, a Chinese website for online shopping
Seeking Shell Flagship Store	Battery Repairer	The stuff works great Very good quality and workmanship Very easy to use	Taobao Marketplace, a Chinese website for online shopping
Seeking Shell Flagship Store	Battery Repairer	Good quality, very satisfied, logistics is also very fast, good service attitude, worth buying.	Taobao Marketplace, a Chinese website for online shopping
Seeking Shell Flagship Store	Battery Repairer	Charging is pretty fast, and the store ships quickly, so that's good~~	Taobao Marketplace, a Chinese website for online shopping
Seeking Shell Flagship Store	Battery Repairer	Received, shipped quickly packed tightly, used well very satisfied!	Taobao Marketplace, a Chinese website for online shopping
Seeking Shell Flagship Store	Battery Repairer	Baby is good, good quality, fine workmanship, and the same as described, good value for money	Taobao Marketplace, a Chinese website for online shopping
Seeking Shell Flagship Store	Battery Repairer	sockets are very convenient to use, fast charging and not hot, good!	Taobao Marketplace, a Chinese website for online shopping
Seeking Shell Flagship Store	Battery Repairer	Shipping is quite fast, packaging with care , received intact, the overall feeling is good! Customer service are quite good, communication is pleasant!	Taobao Marketplace, a Chinese website for online shopping
Seeking Shell Flagship Store	Battery Repairer	Logistics is quite fast, business service attitude is also good	Taobao Marketplace, a Chinese website for online shopping
Seeking Shell Flagship Store	Battery Repairer	Works great, will come back if needed.	Taobao Marketplace, a Chinese website for online shopping
Seeking Shell Flagship Store	Battery Repairer	Fast shipping, fast charging, not too expensive, happy with it	Taobao Marketplace, a Chinese website for online shopping
Seeking Shell Flagship Store	Battery Repairer	Pretty good quality, affordable price, fast shipping	Taobao Marketplace, a Chinese website for online shopping
Seeking Shell Flagship Store	Battery Repairer	Very fast charging, very durable and very cost effective.	Taobao Marketplace, a Chinese website for online shopping
Seeking Shell Flagship Store	Battery Repairer	The quality is great and the shipping was fast the price was very affordable.	Taobao Marketplace, a Chinese website for online shopping
Seeking Shell Flagship Store	Battery Repairer	It's good. It works.	Taobao Marketplace, a Chinese website for online shopping
Seeking Shell Flagship Store	Battery Repairer	It works well. Recommended.	Taobao Marketplace, a Chinese website for online shopping
Seeking Shell Flagship Store	Battery Repairer	The data cable received, the quality is very good, charging speed is very fast, the price is affordable, worth buying.	Taobao Marketplace, a Chinese website for online shopping
Seeking Shell Flagship Store	Battery Repairer	Affordable price, fast delivery, satisfied!	Taobao Marketplace, a Chinese website for online shopping
Seeking Shell Flagship Store	Battery Repairer	It's authentic and not too expensive, it's great to use, I use it all the time and it's great🎉✨	Taobao Marketplace, a Chinese website for online shopping
Seeking Shell Flagship Store	Battery Repairer	trial	Taobao Marketplace, a Chinese website for online shopping
Seeking Shell Flagship Store	Battery Repairer	Fast shipping, packaged with care, great customer service, and affordable.	Taobao Marketplace, a Chinese website for online shopping
Seeking Shell Flagship Store	Battery Repairer	Fast shipping, cost effective, good quality for battery repair, good quality.	Taobao Marketplace, a Chinese website for online shopping

Seeking Shell Flagship Store	Battery Repairer	I bought a wrong product I bought a fixer and he sent me an impulse line and didn't reply to my messages.	Taobao Marketplace, a Chinese website for online shopping
Seeking Shell Flagship Store	Battery Repairer	Currently just used, and then use again to evaluate_x005f_x0001_ good	Taobao Marketplace, a Chinese website for online shopping
Seeking Shell Flagship Store	Battery Repairer	The logistics were fast, well packaged, and the price was affordable.	Taobao Marketplace, a Chinese website for online shopping
Seeking Shell Flagship Store	Battery Repairer	The color is beautiful and it works oh so well.	Taobao Marketplace, a Chinese website for online shopping
Seeking Shell Flagship Store	Battery Repairer	very happy	Taobao Marketplace, a Chinese website for online shopping
Seeking Shell Flagship Store	Battery Repairer	This is really good, the material and workmanship is particularly exquisite, the workmanship is really very good, I think I can recommend to my friends to buy	Taobao Marketplace, a Chinese website for online shopping
Seeking Shell Flagship Store	Battery Repairer	Logistics to give a strong, tried it, charging speed is fast, good quality!	Taobao Marketplace, a Chinese website for online shopping
Seeking Shell Flagship Store	Battery Repairer	Very good quality, satisfied with a shopping	Taobao Marketplace, a Chinese website for online shopping
Seeking Shell Flagship Store	Battery Repairer	Fast shipping Good quality Cost effective	Taobao Marketplace, a Chinese website for online shopping
Seeking Shell Flagship Store	Battery Repairer	Works pretty well. Charges quickly. Satisfied	Taobao Marketplace, a Chinese website for online shopping
Seeking Shell Flagship Store	Battery Repairer	I bought this to protect the phone battery, but the instructions say how to remove the phone's stored garbage is not necessary, or give a good review!	Taobao Marketplace, a Chinese website for online shopping
Seeking Shell Flagship Store	Battery Repairer	Baby received, thank you boss, the quality is very good, very happy with a shopping next time will come back to patronize!	Taobao Marketplace, a Chinese website for online shopping
Seeking Shell Flagship Store	Battery Repairer	Nice, good quality, well made	Taobao Marketplace, a Chinese website for online shopping
Seeking Shell Flagship Store	Battery Repairer	customer service is very attentive very good recommended to buy😍😍😍😍	Taobao Marketplace, a Chinese website for online shopping
Seeking Shell Flagship Store	Battery Repairer	Pretty good workmanship, fast charging speed, satisfactory	Taobao Marketplace, a Chinese website for online shopping
Seeking Shell Flagship Store	Battery Repairer	Baby has been received. Worth having. The 3 in 1 works great. Buy with confidence if you need it	Taobao Marketplace, a Chinese website for online shopping
Seeking Shell Flagship Store	Battery Repairer	Got the stuff, pretty good quality just arrived and tried it out. It works well.	Taobao Marketplace, a Chinese website for online shopping
Seeking Shell Flagship Store	Battery Repairer	The price is not expensive. Just what was needed, I will be watching your house and will come back if needed.	Taobao Marketplace, a Chinese website for online shopping
Seeking Shell Flagship Store	Battery Repairer	Fast delivery, great stuff, good quality, good price, worth buying.	Taobao Marketplace, a Chinese website for online shopping
Seeking Shell Flagship Store	Battery Repairer	quality The color is also very nice charging is also relatively fast	Taobao Marketplace, a Chinese website for online shopping
Seeking Shell Flagship Store	Battery Repairer	Data cable received no bad, all can be used, the night to use the charging	Taobao Marketplace, a Chinese website for online shopping
Seeking Shell Flagship Store	Battery Repairer	Exquisite workmanship, cost-effective, very satisfactory	Taobao Marketplace, a Chinese website for online shopping
Seeking Shell Flagship Store	Battery Repairer	Received, very good, I bought back several times, really good	Taobao Marketplace, a Chinese website for online shopping
Seeking Shell Flagship Store	Battery Repairer	Good quality, battery repair effect is obvious, the price is friendly, cost-effective, worth recommending the purchase, satisfied.	Taobao Marketplace, a Chinese website for online shopping
Seeking Shell Flagship Store	Battery Repairer	Very good quality and affordable	Taobao Marketplace, a Chinese website for online shopping
Seeking Shell Flagship Store	Battery Repairer	It doesn't help at all.	Taobao Marketplace, a Chinese website for online shopping
Seeking Shell Flagship Store	Battery Repairer	Fast logistics, good quality, works well, good customer service	Taobao Marketplace, a Chinese website for online shopping
Seeking Shell Flagship Store	Battery Repairer	Baby received, so surprised, the quality is very good, cost-effective, really out of my expectation!	Taobao Marketplace, a Chinese website for online shopping
Seeking Shell Flagship Store	Battery Repairer	Logistics is fast, quality is good, business service attitude is very good, satisfied!	Taobao Marketplace, a Chinese website for online shopping
Seeking Shell Flagship Store	Battery Repairer	Professional Cell Phone Battery Repair👍Received and tried it The effect is good prolonged the battery🔋life	Taobao Marketplace, a Chinese website for online shopping
Seeking Shell Flagship Store	Battery Repairer	Satisfied🈵Good and cheap!	Taobao Marketplace, a Chinese website for online shopping
Seeking Shell Flagship Store	Battery Repairer	Cell phone battery repair device, very good, used a few days before coming to evaluation	Taobao Marketplace, a Chinese website for online shopping
Seeking Shell Flagship Store	Battery Repairer	Recommended by a friend, good quality at a good price, very satisfied	Taobao Marketplace, a Chinese website for online shopping
Seeking Shell Flagship Store	Battery Repairer	Good quality and fast shipping	Taobao Marketplace, a Chinese website for online shopping
Seeking Shell Flagship Store	Battery Repairer	Received the goods, the data cable quality is great, very satisfied.	Taobao Marketplace, a Chinese website for online shopping
Seeking Shell Flagship Store	Battery Repairer	The stuff is affordable and beautiful. Merchants are good too.	Taobao Marketplace, a Chinese website for online shopping
Seeking Shell Flagship Store	Battery Repairer	Average, not as good as you guys say, I've charged it several times and the phone is still the same as it was before.	Taobao Marketplace, a Chinese website for online shopping
Seeking Shell Flagship Store	Battery Repairer	Charge quickly not hot machine Easy to carry, first-class quality, packaging is also good, logistics is very fast	Taobao Marketplace, a Chinese website for online shopping

Seeking Shell Flagship Store	Battery Repairer	The data cable has a nice color, charges quickly, has several ports, and is reasonably priced.	Taobao Marketplace, a Chinese website for online shopping
Seeking Shell Flagship Store	Battery Repairer	Received, good quality, fast charging, cheap price too	Taobao Marketplace, a Chinese website for online shopping
Seeking Shell Flagship Store	Battery Repairer	The cell phone repair charger works great and the battery lasts longer than before!	Taobao Marketplace, a Chinese website for online shopping
Seeking Shell Flagship Store	Battery Repairer	Baby received, especially good, repair effect is good, recommended to buy!	Taobao Marketplace, a Chinese website for online shopping
Seeking Shell Flagship Store	Battery Repairer	The data cable charges quickly, is of good quality, easy to use, cost-effective, very satisfied.	Taobao Marketplace, a Chinese website for online shopping
Seeking Shell Flagship Store	Battery Repairer	Good quality, fast charging, cheap	Taobao Marketplace, a Chinese website for online shopping
Seeking Shell Flagship Store	Battery Repairer	Works pretty well, come back if you need to.	Taobao Marketplace, a Chinese website for online shopping
Seeking Shell Flagship Store	Battery Repairer	Data cable feels great and charges quickly Doesn't get hot Good review	Taobao Marketplace, a Chinese website for online shopping
Seeking Shell Flagship Store	Battery Repairer	the old cell phone battery with a long time, value for money, cost-effective	Taobao Marketplace, a Chinese website for online shopping
Seeking Shell Flagship Store	Battery Repairer	The old phone that was previously shutting down while charging seems to have improved a bit, so I can play for relatively long periods of time, so I hope to build on that!	Taobao Marketplace, a Chinese website for online shopping
Seeking Shell Flagship Store	Battery Repairer	Nice, works pretty good, pretty good, fast shipping	Taobao Marketplace, a Chinese website for online shopping
Seeking Shell Flagship Store	Battery Repairer	Nice, recommended by a friend. Trustworthy.	Taobao Marketplace, a Chinese website for online shopping
Seeking Shell Flagship Store	Battery Repairer	Very good good worth buying fast logistics good service reasonable price very satisfied overall very like it	Taobao Marketplace, a Chinese website for online shopping
Seeking Shell Flagship Store	Battery Repairer	packaging is intact, the design is novel, small and exquisite, the repair is very good, the phone does not lag, the price is also affordable	Taobao Marketplace, a Chinese website for online shopping
Seeking Shell Flagship Store	Battery Repairer	Received good quality, fast charging, nice and satisfied.	Taobao Marketplace, a Chinese website for online shopping
Seeking Shell Flagship Store	Battery Repairer	The product was received, good quality, beautiful color and fast charging.	Taobao Marketplace, a Chinese website for online shopping
Seeking Shell Flagship Store	Battery Repairer	carry does not take up space, has been used to feel good, satisfied.	Taobao Marketplace, a Chinese website for online shopping
Seeking Shell Flagship Store	Battery Repairer	Received, the courier is quite fast, opened and looked at, the quality can be, not yet used, used to say again	Taobao Marketplace, a Chinese website for online shopping
Seeking Shell Flagship Store	Battery Repairer	pretty good	Taobao Marketplace, a Chinese website for online shopping
Seeking Shell Flagship Store	Battery Repairer	Baby received, the quality is very good, easy to use, good value for money, worth buying!	Taobao Marketplace, a Chinese website for online shopping
Seeking Shell Flagship Store	Battery Repairer	Friends introduced to buy, very good, logistics is also very fast!	Taobao Marketplace, a Chinese website for online shopping
Seeking Shell Flagship Store	Battery Repairer	Really super like it, very good quality, as described by the seller!	Taobao Marketplace, a Chinese website for online shopping
Seeking Shell Flagship Store	Battery Repairer	Fast charging, silicone, soft and unbreakable	Taobao Marketplace, a Chinese website for online shopping
Seeking Shell Flagship Store	Battery Repairer	It is very easy to use! Worth recommending! Works great!	Taobao Marketplace, a Chinese website for online shopping
Seeking Shell Flagship Store	Battery Repairer	Baby received, very satisfied with a shopping, will come back again.	Taobao Marketplace, a Chinese website for online shopping
Seeking Shell Flagship Store	Battery Repairer	It is very good ah, good to use, logistics is also very fast, the boss has a good service attitude, praise!	Taobao Marketplace, a Chinese website for online shopping
Seeking Shell Flagship Store	Battery Repairer	It has arrived some days ago and is really good quality, very satisfied. The seller shipped quickly.	Taobao Marketplace, a Chinese website for online shopping
Seeking Shell Flagship Store	Battery Repairer	Not bad, the delivery is quite fast, the packaging is quite good, the stuff is good	Taobao Marketplace, a Chinese website for online shopping
Seeking Shell Flagship Store	Battery Repairer	Baby received, the effect is very good, affordable, recommended to buy!	Taobao Marketplace, a Chinese website for online shopping
Seeking Shell Flagship Store	Battery Repairer	Fast shipping, works well	Taobao Marketplace, a Chinese website for online shopping
Seeking Shell Flagship Store	Battery Repairer	Received the stuff, well packaged, tried it out and it worked pretty well👍	Taobao Marketplace, a Chinese website for online shopping
Seeking Shell Flagship Store	Battery Repairer	no good	Taobao Marketplace, a Chinese website for online shopping
Seeking Shell Flagship Store	Battery Repairer	Baby arrived really good. The price/performance ratio is superb and the logistics are great!	Taobao Marketplace, a Chinese website for online shopping
Seeking Shell Flagship Store	Battery Repairer	The data cable is beautiful and delicate, three charging heads can meet different needs, charging is also fast fantastic!	Taobao Marketplace, a Chinese website for online shopping
Seeking Shell Flagship Store	Battery Repairer	Express received, very good, very convenient, the quality is also good	Taobao Marketplace, a Chinese website for online shopping
Seeking Shell Flagship Store	Battery Repairer	Works great, tried it a couple times and fast shipping!	Taobao Marketplace, a Chinese website for online shopping
Seeking Shell Flagship Store	Battery Repairer	Great material, good quality and arrived quickly!	Taobao Marketplace, a Chinese website for online shopping
Seeking Shell Flagship Store	Battery Repairer	Pretty good, fast shipping, fine workmanship pretty good, used it2times, put a lot of durability before.	Taobao Marketplace, a Chinese website for online shopping

Seeking Shell Flagship Store	Battery Repairer	Received it, logistics and delivery fast. Repair Magic is really great, 👍, the effect is obvious, solve a lot of things. Awesome! 🏵🏵🏵🏵🏵🏵	Taobao Marketplace, a Chinese website for online shopping
Seeking Shell Flagship Store	Battery Repairer	Good quality, easy to use and affordable.	Taobao Marketplace, a Chinese website for online shopping
Seeking Shell Flagship Store	Battery Repairer	The data cable is of good quality, charges quickly and is easy to use	Taobao Marketplace, a Chinese website for online shopping
Seeking Shell Flagship Store	Battery Repairer	Baby! Nice. Love it.	Taobao Marketplace, a Chinese website for online shopping
Seeking Shell Flagship Store	Battery Repairer	I received the item, but I don't know what it will do?	Taobao Marketplace, a Chinese website for online shopping
Seeking Shell Flagship Store	Battery Repairer	Goods received, good quality, fast charging, very satisfied.	Taobao Marketplace, a Chinese website for online shopping
Seeking Shell Flagship Store	Battery Repairer	Very good, bought with the intention of trying, received the quality is very good, worth buying!	Taobao Marketplace, a Chinese website for online shopping
Seeking Shell Flagship Store	Battery Repairer	Baby received, love it, worth buying.	Taobao Marketplace, a Chinese website for online shopping
Seeking Shell Flagship Store	Battery Repairer	Small, easy to carry, quality is quite good, the price is also affordable, satisfied with the	Taobao Marketplace, a Chinese website for online shopping
Seeking Shell Flagship Store	Battery Repairer	Baby is basically the same as the description, fast shipping, worth it!	Taobao Marketplace, a Chinese website for online shopping
Seeking Shell Flagship Store	Battery Repairer	Nice and fast charging	Taobao Marketplace, a Chinese website for online shopping
Seeking Shell Flagship Store	Battery Repairer	Good quality, fast logistics, like	Taobao Marketplace, a Chinese website for online shopping
Seeking Shell Flagship Store	Battery Repairer	Baby received, tested it and it works well. Extend the life of cell phone battery.	Taobao Marketplace, a Chinese website for online shopping
Seeking Shell Flagship Store	Battery Repairer	Slow charging. Electricity won't charge.	Taobao Marketplace, a Chinese website for online shopping
Seeking Shell Flagship Store	Battery Repairer	Customer service, the electricity won't charge into the phone these days. What to do?	Taobao Marketplace, a Chinese website for online shopping
Seeking Shell Flagship Store	Battery Repairer	Received the goods, used it, it works well, the quality is very good, recommended to buy.	Taobao Marketplace, a Chinese website for online shopping
Seeking Shell Flagship Store	Battery Repairer	Packaging is intact, quality is guaranteed, practical and convenient, will buy back!	Taobao Marketplace, a Chinese website for online shopping
Seeking Shell Flagship Store	Battery Repairer	Packaging is very solid, logistics is also particularly fast, things get very satisfied, consistent with the description. Overall, a very satisfied shopping.	Taobao Marketplace, a Chinese website for online shopping
Seeking Shell Flagship Store	Battery Repairer	The charging cable arrived, a 3-in-1 universal product for Android and Apple, and the cable is long enough. Satisfied	Taobao Marketplace, a Chinese website for online shopping
Seeking Shell Flagship Store	Battery Repairer	Received the quality is very good very like very satisfied with a shopping!	Taobao Marketplace, a Chinese website for online shopping
Seeking Shell Flagship Store	Battery Repairer	Works well. Charged the battery a couple times. Battery life is better than before. Satisfied with the shopping experience.	Taobao Marketplace, a Chinese website for online shopping
Seeking Shell Flagship Store	Battery Repairer	The current activated charger is pretty good quality! Tried out the repair and it works well!	Taobao Marketplace, a Chinese website for online shopping
Seeking Shell Flagship Store	Battery Repairer	Just started to use, whether it is good or not, after a period of time to know, the shape is quite good-looking.	Taobao Marketplace, a Chinese website for online shopping
Seeking Shell Flagship Store	Battery Repairer	Baby has been received and is charging, will comment after use. First5out5for fast shipping, etc.	Taobao Marketplace, a Chinese website for online shopping
Seeking Shell Flagship Store	Battery Repairer	Not bad, charging quite fast, work in the office to use quite good	Taobao Marketplace, a Chinese website for online shopping
Seeking Shell Flagship Store	Battery Repairer	Overall it is still very good, the store has a very good service attitude, the logistics is also very fast good use	Taobao Marketplace, a Chinese website for online shopping
Seeking Shell Flagship Store	Battery Repairer	Express has been received, the thing is really great, better than expected in addition to the packaging intact, worth buying, good value!	Taobao Marketplace, a Chinese website for online shopping
Seeking Shell Flagship Store	Battery Repairer	Stylish design, extensive practicality, nice and good, and excellent quality	Taobao Marketplace, a Chinese website for online shopping
Seeking Shell Flagship Store	Battery Repairer	Battery Repair Magic received, very good, logistics is also very fast.	Taobao Marketplace, a Chinese website for online shopping
Seeking Shell Flagship Store	Battery Repairer	The cable is great too, I forgot to comment on it, follow up follow up😛😛😛😛😛😛😛😛😛	Taobao Marketplace, a Chinese website for online shopping
Seeking Shell Flagship Store	Battery Repairer	Very professional, very satisfied	Taobao Marketplace, a Chinese website for online shopping
Seeking Shell Flagship Store	Battery Repairer	Seller service is very good, very satisfied with a shopping	Taobao Marketplace, a Chinese website for online shopping
Seeking Shell Flagship Store	Battery Repairer	The quality is good, charging quite fast, and three plugs are very convenient, good value for money	Taobao Marketplace, a Chinese website for online shopping
Seeking Shell Flagship Store	Battery Repairer	Baby arrived in good quality, very satisfied, cost-effective logistics is also strong	Taobao Marketplace, a Chinese website for online shopping
Seeking Shell Flagship Store	Battery Repairer	Received the goods, the quality is very good, worth recommending to everyone.	Taobao Marketplace, a Chinese website for online shopping
Seeking Shell Flagship Store	Battery Repairer	Very good use, logistics is also very fast, very practical, satisfactory	Taobao Marketplace, a Chinese website for online shopping
Seeking Shell Flagship Store	Battery Repairer	fast, the effect is quite good, easy to use, you can carry with you!	Taobao Marketplace, a Chinese website for online shopping
Seeking Shell Flagship Store	Battery Repairer	Logistics is very fast, things look good, wait until after the use of follow-up comments	Taobao Marketplace, a Chinese website for online shopping

Seeking Shell Flagship Store	Battery Repairer	Very good, very satisfied, love it, worth buying.	Taobao Marketplace, a Chinese website for online shopping
Seeking Shell Flagship Store	Battery Repairer	better than expected Packaging intact, special praise need to hurry to buy it	Taobao Marketplace, a Chinese website for online shopping
Seeking Shell Flagship Store	Battery Repairer	As described by the store, fast shipping good quality, affordable price very good use	Taobao Marketplace, a Chinese website for online shopping
Seeking Shell Flagship Store	Battery Repairer	The goods received and unpacked to use, the quality is very good	Taobao Marketplace, a Chinese website for online shopping
Seeking Shell Flagship Store	Battery Repairer	♡Looks good, packaged carefully, very fair price, will continue to buy and buy! ✌	Taobao Marketplace, a Chinese website for online shopping
Seeking Shell Flagship Store	Battery Repairer	not be easy to break Cell phone battery maintenance is really good Very much like this cell phone battery repair magic weapon Really love it	Taobao Marketplace, a Chinese website for online shopping
Seeking Shell Flagship Store	Battery Repairer	Finally received the baby I need, the stuff is very good, good price. Thanks to the shopkeeper!	Taobao Marketplace, a Chinese website for online shopping
Seeking Shell Flagship Store	Battery Repairer	I can't tell if it's an IQ tax until I use it, and I'll evaluate it accurately later.	Taobao Marketplace, a Chinese website for online shopping
Seeking Shell Flagship Store	Battery Repairer	The logistics were fast, well packaged, and the price was affordable.	Taobao Marketplace, a Chinese website for online shopping
Seeking Shell Flagship Store	Battery Repairer	Sticking with cell phones does play longer than before	Taobao Marketplace, a Chinese website for online shopping
Seeking Shell Flagship Store	Battery Repairer	connected to this restorer can be normal charging, very useful, good stuff!	Taobao Marketplace, a Chinese website for online shopping
Seeking Shell Flagship Store	Battery Repairer	an excellent	Taobao Marketplace, a Chinese website for online shopping
Seeking Shell Flagship Store	Battery Repairer	Received, the logistics is very strong, the quality is very good, used to know it's good, the price is not expensive, it is worth having!	Taobao Marketplace, a Chinese website for online shopping
Seeking Shell Flagship Store	Battery Repairer	You need to read the instructions carefully to use it, and the service is great!	Taobao Marketplace, a Chinese website for online shopping
Seeking Shell Flagship Store	Battery Repairer	That cell phone battery repairer, lightweight, new style, shopping satisfaction.	Taobao Marketplace, a Chinese website for online shopping
Seeking Shell Flagship Store	Battery Repairer	Received, good quality, 3 in 1 is very convenient, home a go out with one.	Taobao Marketplace, a Chinese website for online shopping
Seeking Shell Flagship Store	Battery Repairer	Fast shipping, pretty good quality, as described by the seller, very satisfied!	Taobao Marketplace, a Chinese website for online shopping
Seeking Shell Flagship Store	Battery Repairer	It's not bad, following the instructions the phone is already more durable than before	Taobao Marketplace, a Chinese website for online shopping
Seeking Shell Flagship Store	Battery Repairer	Old cell phone, after restoration, standby for a day, and more than sixty power, rare good product, worth buying	Taobao Marketplace, a Chinese website for online shopping
Seeking Shell Flagship Store	Battery Repairer	With this, the phone battery now plays longer than before	Taobao Marketplace, a Chinese website for online shopping
Seeking Shell Flagship Store	Battery Repairer	Good value for money and good reviews!	Taobao Marketplace, a Chinese website for online shopping
Seeking Shell Flagship Store	Battery Repairer	time I felt there was a problem. After communicating with the store, it was realized that the product itself structure. Try again, can be used now use the effect is good, use longer than before!	Taobao Marketplace, a Chinese website for online shopping
Seeking Shell Flagship Store	Battery Repairer	It's a little on the expensive side for the price and I'm too lazy to return it	Taobao Marketplace, a Chinese website for online shopping
Seeking Shell Flagship Store	Battery Repairer	Good packaging and quality, nice style, very sensitive contact, fast charging!	Taobao Marketplace, a Chinese website for online shopping
Seeking Shell Flagship Store	Battery Repairer	Got it. It's working fine so far.	Taobao Marketplace, a Chinese website for online shopping
Seeking Shell Flagship Store	Battery Repairer	favorable criticism	Taobao Marketplace, a Chinese website for online shopping
Seeking Shell Flagship Store	Battery Repairer	is also very good, very satisfied with the next will continue to buy!	Taobao Marketplace, a Chinese website for online shopping
Seeking Shell Flagship Store	Battery Repairer	It's pretty good and the color is nice, av 🍉milkshake like	Taobao Marketplace, a Chinese website for online shopping
Seeking Shell Flagship Store	Battery Repairer	Quality is very good🍆, cost-effective goods🏵, like the pro can be directly under the hand do not hesitate Oh🍸	Taobao Marketplace, a Chinese website for online shopping
Seeking Shell Flagship Store	Battery Repairer	Fast shipping, good quality, good value	Taobao Marketplace, a Chinese website for online shopping
Seeking Shell Flagship Store	Battery Repairer	Packed carefully, shipped quickly, pretty good quality	Taobao Marketplace, a Chinese website for online shopping
Seeking Shell Flagship Store	Battery Repairer	Received it, used it as instructed and it worked, worth the money!	Taobao Marketplace, a Chinese website for online shopping
Seeking Shell Flagship Store	Battery Repairer	Baby received, thank you boss lady, the quality is very good, logistics is also very fast, very satisfied and very happy with a shopping, the next time will patronize!	Taobao Marketplace, a Chinese website for online shopping
Seeking Shell Flagship Store	Battery Repairer	The quality is particularly good, inexpensive, the shopkeeper has a very good attitude towards service	Taobao Marketplace, a Chinese website for online shopping
Seeking Shell Flagship Store	Battery Repairer	It still feels effective, stick to3capsules2timesa week.	Taobao Marketplace, a Chinese website for online shopping
Seeking Shell Flagship Store	Battery Repairer	The cable works great.	Taobao Marketplace, a Chinese website for online shopping

Seeking Shell Flagship Store	Battery Repairer	Durable and good quality!	Taobao Marketplace, a Chinese website for online shopping
Seeking Shell Flagship Store	Battery Repairer	Very good! Charges quickly!	Taobao Marketplace, a Chinese website for online shopping
Seeking Shell Flagship Store	Battery Repairer	Received, logistics is very fast, the seller service is also very good, worth buying!	Taobao Marketplace, a Chinese website for online shopping
Seeking Shell Flagship Store	Battery Repairer	Baby received it, quite good looking this color, quality or no words, quite satisfied, ready to buy a few more	Taobao Marketplace, a Chinese website for online shopping
Seeking Shell Flagship Store	Battery Repairer	Fast shipping and undamaged packaging	Taobao Marketplace, a Chinese website for online shopping
Seeking Shell Flagship Store	Battery Repairer	Fast shipping, great stuff.	Taobao Marketplace, a Chinese website for online shopping
Seeking Shell Flagship Store	Battery Repairer	Goods received, the quality is very good like, test is effective, the price is also very reasonable, good quality, fast logistics, worth recommending!	Taobao Marketplace, a Chinese website for online shopping
Seeking Shell Flagship Store	Battery Repairer	The quality is okay! Affordable price, come back if you need	Taobao Marketplace, a Chinese website for online shopping
Seeking Shell Flagship Store	Battery Repairer	Received, the quality is quite good, charging quickly, satisfied.	Taobao Marketplace, a Chinese website for online shopping
Seeking Shell Flagship Store	Battery Repairer	The quality is exceptionally well made and detailed. Shipping is fast.	Taobao Marketplace, a Chinese website for online shopping
Seeking Shell Flagship Store	Battery Repairer	It's a flim-flam, don't believe it!	Taobao Marketplace, a Chinese website for online shopping
Seeking Shell Flagship Store	Battery Repairer	After several days of testing and searching for several related test videos on the Internet, I have come to the conclusion that you should not believe this advertisement!	Taobao Marketplace, a Chinese website for online shopping
Seeking Shell Flagship Store	Battery Repairer	Goods have been received, logistics delivery is also very fast, the seller's service attitude is also very good, very satisfied with a shopping.	Taobao Marketplace, a Chinese website for online shopping
Seeking Shell Flagship Store	Battery Repairer	Fast shipping, great stuff, overall very happy.	Taobao Marketplace, a Chinese website for online shopping
Seeking Shell Flagship Store	Battery Repairer	Good review!	Taobao Marketplace, a Chinese website for online shopping
Seeking Shell Flagship Store	Battery Repairer	Things have been received, the quality is very good, fine workmanship, inexpensive, fast shipping, good service attitude	Taobao Marketplace, a Chinese website for online shopping
Seeking Shell Flagship Store	Battery Repairer	The item has been received and is of good quality and works well. Express delivery is strong	Taobao Marketplace, a Chinese website for online shopping
Seeking Shell Flagship Store	Battery Repairer	Nice, good quality, affordable, will be back for more.	Taobao Marketplace, a Chinese website for online shopping
Seeking Shell Flagship Store	Battery Repairer	IQ tax, people, don't fall for it!	Taobao Marketplace, a Chinese website for online shopping
Seeking Shell Flagship Store	Battery Repairer	Express has been received, the thing is really great, better than expected in addition to the packaging intact, worth buying, good value!	Taobao Marketplace, a Chinese website for online shopping
Seeking Shell Flagship Store	Battery Repairer	It's okay, you can see the voltage and current! Looks good too.	Taobao Marketplace, a Chinese website for online shopping
Seeking Shell Flagship Store	Battery Repairer	The store shipped quite quickly, received and tried to repair the effect is quite good!	Taobao Marketplace, a Chinese website for online shopping
Seeking Shell Flagship Store	Battery Repairer	Good quality at an affordable price	Taobao Marketplace, a Chinese website for online shopping
Seeking Shell Flagship Store	Battery Repairer	Baby received, good price, trustworthy business!	Taobao Marketplace, a Chinese website for online shopping
Seeking Shell Flagship Store	Battery Repairer	Doesn't work or has no noticeable effect. Idle.	Taobao Marketplace, a Chinese website for online shopping
Seeking Shell Flagship Store	Battery Repairer	It's too much trouble. It's too complicated.	Taobao Marketplace, a Chinese website for online shopping
Seeking Shell Flagship Store	Battery Repairer	Fast shipping and great quality for the money!	Taobao Marketplace, a Chinese website for online shopping
Seeking Shell Flagship Store	Battery Repairer	This is a three-less product, the merchant only said refund, Taobao also only give a refund, bought a three-less product, so I am very unhappy to deal with it. This kind of three-nothing product without 3C certification, you dare to give the phone to use? Do not recommend it to everyone.	Taobao Marketplace, a Chinese website for online shopping
Seeking Shell Flagship Store	Battery Repairer	Don't buy it, it's a three-no product, it's a scam! You find 12315 Trade and Industry Bureau is also useless, the Trade and Industry Bureau does not give a solution!	Taobao Marketplace, a Chinese website for online shopping
Seeking Shell Flagship Store	Battery Repairer	I've used it a couple times and it doesn't feel like it's working.	Taobao Marketplace, a Chinese website for online shopping
Seeking Shell Flagship Store	Battery Repairer	Charging is particularly fast, and it is also distinguished by different colors, which is particularly easy to distinguish. The workmanship is quite exquisite.	Taobao Marketplace, a Chinese website for online shopping
Seeking Shell Flagship Store	Battery Repairer	Fast charging, affordable, easy to use, very good packaging, a pleasant shopping	Taobao Marketplace, a Chinese website for online shopping
Seeking Shell Flagship Store	Battery Repairer	Arrived quickly, meticulous workmanship, test results, good to come back!	Taobao Marketplace, a Chinese website for online shopping
Seeking Shell Flagship Store	Battery Repairer	Baby received, the logistics is great, the quality is also very good!	Taobao Marketplace, a Chinese website for online shopping
Seeking Shell Flagship Store	Battery Repairer	Exceptional quality, very fast charging, nice	Taobao Marketplace, a Chinese website for online shopping

Seeking Shell Flagship Store	Battery Repairer	Shipping is quite fast, things are received,andpicture described in line with the customer service is very patient, ask and answer, very satisfied with a shopping.	Taobao Marketplace, a Chinese website for online shopping
Seeking Shell Flagship Store	Battery Repairer	The goods have been received, as described, first praise. Enthusiastic service, fast delivery, full marks.	Taobao Marketplace, a Chinese website for online shopping
Seeking Shell Flagship Store	Battery Repairer	Just received the goods, after use to see the effect of follow-up comments	Taobao Marketplace, a Chinese website for online shopping
Seeking Shell Flagship Store	Battery Repairer	Charger received, good quality, fine workmanship, beautiful appearance	Taobao Marketplace, a Chinese website for online shopping
Seeking Shell Flagship Store	Battery Repairer	Fast shipping and logistics, 3-in-1 data cable is very good quality, affordable price, buy a good deal!	Taobao Marketplace, a Chinese website for online shopping
Seeking Shell Flagship Store	Battery Repairer	Logistics so fast, the quality of workmanship is good, charging very quickly, with a safe, affordable price, very satisfied, recommended to buy.	Taobao Marketplace, a Chinese website for online shopping
Seeking Shell Flagship Store	Battery Repairer	Cell phone repair charger is good, after cell phone repair charging, cell phone use longer, worth to buy a good charger.	Taobao Marketplace, a Chinese website for online shopping
Seeking Shell Flagship Store	Battery Repairer	Great quality, easy to use, very satisfied.	Taobao Marketplace, a Chinese website for online shopping
Seeking Shell Flagship Store	Battery Repairer	dissatisfied	Taobao Marketplace, a Chinese website for online shopping
Seeking Shell Flagship Store	Battery Repairer	It's fast. It's good.	Taobao Marketplace, a Chinese website for online shopping
Seeking Shell Flagship Store	Battery Repairer	I've tried it, I don't know if it's working anymore, during which the odd performance occurs.	Taobao Marketplace, a Chinese website for online shopping
Seeking Shell Flagship Store	Battery Repairer	Tried it out and it works well it's affordable.	Taobao Marketplace, a Chinese website for online shopping
Seeking Shell Flagship Store	Battery Repairer	Quality is fine	Taobao Marketplace, a Chinese website for online shopping
Seeking Shell Flagship Store	Battery Repairer	Fast charging line received, logistics is very fast, charging line quality is very good, fine workmanship, a line of multi-purpose, can charge Android, Apple, Huawei, fantastic, cheap price is worth buying.	Taobao Marketplace, a Chinese website for online shopping
Seeking Shell Flagship Store	Battery Repairer	Whether or not it achieves the ability to repair batteries will have to be evaluated after using it for a while!	Taobao Marketplace, a Chinese website for online shopping
Seeking Shell Flagship Store	Battery Repairer	A line of multi-charging, the cable is an extended version, charging extra fast	Taobao Marketplace, a Chinese website for online shopping
Seeking Shell Flagship Store	Battery Repairer	Fast shipping, tight packaging, the quality of goods is very good, much cheaper than the counter, customer service attitude is also particularly good, the main thing is that the charge is very fast Good comment	Taobao Marketplace, a Chinese website for online shopping
Seeking Shell Flagship Store	Battery Repairer	It works fine and the battery lasts a bit longer than the previousone~	Taobao Marketplace, a Chinese website for online shopping
Seeking Shell Flagship Store	Battery Repairer	The quality is great and the colors look good!	Taobao Marketplace, a Chinese website for online shopping
Seeking Shell Flagship Store	Battery Repairer	Received the goods, the price is good, repurchase 2times. Convenient to take with you when you go out.	Taobao Marketplace, a Chinese website for online shopping
Seeking Shell Flagship Store	Battery Repairer	It's not bad, it's quite practical, it's worth buying	Taobao Marketplace, a Chinese website for online shopping
Seeking Shell Flagship Store	Battery Repairer	Received, happy with the purchase, will be back.	Taobao Marketplace, a Chinese website for online shopping
Seeking Shell Flagship Store	Battery Repairer	It's great quality and worth having, just try it!	Taobao Marketplace, a Chinese website for online shopping
Seeking Shell Flagship Store	Battery Repairer	Consider it when you need it.	Taobao Marketplace, a Chinese website for online shopping
Seeking Shell Flagship Store	Battery Repairer	Logistics is very fast, the packaging is intact, the quality is good, cost-effective recommended!	Taobao Marketplace, a Chinese website for online shopping
Seeking Shell Flagship Store	Battery Repairer	The quality of the cord is great, the charging is stable, and the shipping was fast!	Taobao Marketplace, a Chinese website for online shopping
Seeking Shell Flagship Store	Battery Repairer	Logistics is particularly convenient, the seller's service attitude is good	Taobao Marketplace, a Chinese website for online shopping
Seeking Shell Flagship Store	Battery Repairer	Very good Good quality Easy to use Three heads Any type will work	Taobao Marketplace, a Chinese website for online shopping
Seeking Shell Flagship Store	Battery Repairer	It's good. It's good.	Taobao Marketplace, a Chinese website for online shopping
Seeking Shell Flagship Store	Battery Repairer	praise! Boss shipping is also god speed, how to feel just bought it arrived	Taobao Marketplace, a Chinese website for online shopping
Seeking Shell Flagship Store	Battery Repairer	Charges quickly, ships fast, great quality!	Taobao Marketplace, a Chinese website for online shopping
Seeking Shell Flagship Store	Battery Repairer	The cell phone battery reconditioning is quite good, after using it, the charge is much faster than before. The price is not expensive and the quality is pretty great.	Taobao Marketplace, a Chinese website for online shopping
Seeking Shell Flagship Store	Battery Repairer	Shipping was fast, the quality is exceptional, and it's a great deal for the money.	Taobao Marketplace, a Chinese website for online shopping
Seeking Shell Flagship Store	Battery Repairer	The workmanship is great! Customer service is very good! Logistics is very fast oh!	Taobao Marketplace, a Chinese website for online shopping
Seeking Shell Flagship Store	Battery Repairer	Really good, good use of the effect, good customer service, logistics is also fast!	Taobao Marketplace, a Chinese website for online shopping
Seeking Shell Flagship Store	Battery Repairer	The quality is great!	Taobao Marketplace, a Chinese website for online shopping

Seeking Shell Flagship Store	Battery Repairer	Fast charging, good quality, more affordable price	Taobao Marketplace, a Chinese website for online shopping
Seeking Shell Flagship Store	Battery Repairer	Shipped quickly, works well, great customer service!	Taobao Marketplace, a Chinese website for online shopping
Seeking Shell Flagship Store	Battery Repairer	The quality of the product is really awesome and works well.	Taobao Marketplace, a Chinese website for online shopping
Seeking Shell Flagship Store	Battery Repairer	More than satisfied, haven't looked at the inverted school fruit yet.	Taobao Marketplace, a Chinese website for online shopping
Seeking Shell Flagship Store	Battery Repairer	The quality is great and it works very well.	Taobao Marketplace, a Chinese website for online shopping
Seeking Shell Flagship Store	Battery Repairer	Nice🉐, great quality, great customer service👌!	Taobao Marketplace, a Chinese website for online shopping
Seeking Shell Flagship Store	Battery Repairer	Look quite high-tech, follow the instructions to operate, look forward to effect	Taobao Marketplace, a Chinese website for online shopping
Seeking Shell Flagship Store	Battery Repairer	Item was received in perfect condition and is being tested for effectiveness.	Taobao Marketplace, a Chinese website for online shopping
Seeking Shell Flagship Store	Battery Repairer	Three interface design, convenient and safe. Favorable price, cost-effective, good customer service, good, love it!	Taobao Marketplace, a Chinese website for online shopping
Seeking Shell Flagship Store	Battery Repairer	Very good. Logistics was fast.	Taobao Marketplace, a Chinese website for online shopping
Seeking Shell Flagship Store	Battery Repairer	Arrived and could not wait to open, really did not let me disappoint, the quality is particularly good, the value is also quite high, satisfied with a shopping	Taobao Marketplace, a Chinese website for online shopping
Seeking Shell Flagship Store	Battery Repairer	It's good quality.	Taobao Marketplace, a Chinese website for online shopping
Seeking Shell Flagship Store	Battery Repairer	I just got it. I haven't tried it yet.	Taobao Marketplace, a Chinese website for online shopping
Seeking Shell Flagship Store	Battery Repairer	This quality is really unexpected, the real thing is really good, touch is very good, very recommended Oh~	Taobao Marketplace, a Chinese website for online shopping
Seeking Shell Flagship Store	Battery Repairer	Good quality, fast shipping, beautiful, works well.	Taobao Marketplace, a Chinese website for online shopping
Seeking Shell Flagship Store	Battery Repairer	Great quality, works well, fast shipping	Taobao Marketplace, a Chinese website for online shopping
Seeking Shell Flagship Store	Battery Repairer	Received, feel can, good workmanship, easy to use, repair function is good, satisfaction	Taobao Marketplace, a Chinese website for online shopping
Seeking Shell Flagship Store	Battery Repairer	Easy to operate and the battery life is really long	Taobao Marketplace, a Chinese website for online shopping
Seeking Shell Flagship Store	Battery Repairer	Affordable and practical, great!	Taobao Marketplace, a Chinese website for online shopping
Seeking Shell Flagship Store	Battery Repairer	Received it, works great, easy to use, fast charging.	Taobao Marketplace, a Chinese website for online shopping
Seeking Shell Flagship Store	Battery Repairer	tried, the effect has been improved. The quality is quite good, the basic function is consistent with the store description, the charging data is clear, and the quality is stable. Good review.	Taobao Marketplace, a Chinese website for online shopping
Seeking Shell Flagship Store	Battery Repairer	Shipping and logistics are quite strong, the packaging is very careful not to damage, three lines in one is very convenient. Very good to go out with one is enough	Taobao Marketplace, a Chinese website for online shopping
Seeking Shell Flagship Store	Battery Repairer	Fired up.	Taobao Marketplace, a Chinese website for online shopping
Seeking Shell Flagship Store	Battery Repairer	🎁Baby received it and the texture is also very nice and simple looking!😍It's what I wanted! Will be back next time!	Taobao Marketplace, a Chinese website for online shopping
Seeking Shell Flagship Store	Battery Repairer	The goods received, especially like, once received, can not wait to open, the family is also very satisfied, this shopping experience is good!	Taobao Marketplace, a Chinese website for online shopping
Seeking Shell Flagship Store	Battery Repairer	Used it. Works well. Battery use was noticeably longer today, amazing results!	Taobao Marketplace, a Chinese website for online shopping
Seeking Shell Flagship Store	Battery Repairer	Great, fast logistics, great stuff, great value for money!	Taobao Marketplace, a Chinese website for online shopping
Seeking Shell Flagship Store	Battery Repairer	There's no need to do anything else, is there?	Taobao Marketplace, a Chinese website for online shopping
Seeking Shell Flagship Store	Battery Repairer	Good quality, good packaging, fast charging, good	Taobao Marketplace, a Chinese website for online shopping
Seeking Shell Flagship Store	Battery Repairer	I received the item and am very happy with it and will continue to patronize the store.	Taobao Marketplace, a Chinese website for online shopping
Seeking Shell Flagship Store	Battery Repairer	It's nice and charges quickly.	Taobao Marketplace, a Chinese website for online shopping
Seeking Shell Flagship Store	Battery Repairer	charging speed is also very fast, very satisfied, recommended to buy!	Taobao Marketplace, a Chinese website for online shopping
Seeking Shell Flagship Store	Battery Repairer	additional comments, thanks to the seller to send the data line	Taobao Marketplace, a Chinese website for online shopping
Seeking Shell Flagship Store	Battery Repairer	There is always a process of acclimatization, followed by a period of slow battery restoration of the product with a weak battery according to the procedure in the manual	Taobao Marketplace, a Chinese website for online shopping
Seeking Shell Flagship Store	Battery Repairer	The baby is really good, it works very well love it, the quality is great!	Taobao Marketplace, a Chinese website for online shopping
Seeking Shell Flagship Store	Battery Repairer	Multi-functional simplicity and convenience at a reasonable price.	Taobao Marketplace, a Chinese website for online shopping

Seeking Shell Flagship Store	Battery Repairer	Fast shipping, very good quality, great value for money, love it, just what I needed, will keep an eye on it, kudos.	Taobao Marketplace, a Chinese website for online shopping
Seeking Shell Flagship Store	Battery Repairer	nil	Taobao Marketplace, a Chinese website for online shopping
Seeking Shell Flagship Store	Battery Repairer	Honestly, I didn't feel out the battery durability, so I'll give it a good review out of habit.	Taobao Marketplace, a Chinese website for online shopping
Seeking Shell Flagship Store	Battery Repairer	Nice The material is ok Much cheaper than what I bought in the store Good value for money 👍	Taobao Marketplace, a Chinese website for online shopping
Seeking Shell Flagship Store	Battery Repairer	Baby received, the large group of quite fast,😆😆😆😆😆Overall feel the quality is quite good, with a smooth,😂😂😂😂cost-effective comparison!	Taobao Marketplace, a Chinese website for online shopping
Seeking Shell Flagship Store	Battery Repairer	Didn't see any real results charging with two phones! Habitual positive feedback😒	Taobao Marketplace, a Chinese website for online shopping
Seeking Shell Flagship Store	Battery Repairer	against the merchant for cheating consumers!!!😤😤😤😤😤😤😤😤	Taobao Marketplace, a Chinese website for online shopping
Seeking Shell Flagship Store	Battery Repairer	Very good, great packaging, looks good, next time, arrived very quickly	Taobao Marketplace, a Chinese website for online shopping
Seeking Shell Flagship Store	Battery Repairer	Data cable received, charging speed is very fast, very satisfied with a shopping	Taobao Marketplace, a Chinese website for online shopping
Seeking Shell Flagship Store	Battery Repairer	The quality of the cable is pretty good	Taobao Marketplace, a Chinese website for online shopping
Seeking Shell Flagship Store	Battery Repairer	Fast logistics, value for money, good quality stuff, high-tech👍	Taobao Marketplace, a Chinese website for online shopping
Seeking Shell Flagship Store	Battery Repairer	I just used it today, I'll come back with a follow-up comment if it works!	Taobao Marketplace, a Chinese website for online shopping
Seeking Shell Flagship Store	Battery Repairer	Cell phone battery repair device, exquisite workmanship, compact, beautiful appearance, looking forward to the effect, first use, such as the effect is good and then follow up! Buyers good service, five-star praise!	Taobao Marketplace, a Chinese website for online shopping
Seeking Shell Flagship Store	Battery Repairer	Data cable received quite good, the color is also very beautiful, charging up can.	Taobao Marketplace, a Chinese website for online shopping
Seeking Shell Flagship Store	Battery Repairer	not be charged Buy this kind of one tow three charging cable, of course, to adapt to the different charging port electronic products, the purpose of the purchase is a line of multi-purpose, or else you can just buy ordinary fast charging cable! Why buy this slow charging thing? Functionally defective surprisingly no warranty and compensation, said the post-sale, now the after-sales to do seven days? The five-year warranty for electronic products was reduced to two years, and finally a general one-year warranty. Now 7days warranty? Garbage products, five or six dollars to buy seven-day warranty things, I think it is better to buy a bag of yogurt, fermented things than his shelf life are longer.	Taobao Marketplace, a Chinese website for online shopping
Seeking Shell Flagship Store	Battery Repairer	Baby arrived, logistics is very fast, very good, the price is also very affordable, worth recommending.	Taobao Marketplace, a Chinese website for online shopping
Seeking Shell Flagship Store	Battery Repairer	Logistics is fast, very technological, good	Taobao Marketplace, a Chinese website for online shopping
Seeking Shell Flagship Store	Battery Repairer	It's not bad, charging is pretty efficient.	Taobao Marketplace, a Chinese website for online shopping
Seeking Shell Flagship Store	Battery Repairer	Arrived fast, good quality, as described, affordable, satisfied.	Taobao Marketplace, a Chinese website for online shopping
Seeking Shell Flagship Store	Battery Repairer	I've got it. Good, good, good. Thank you.	Taobao Marketplace, a Chinese website for online shopping
Seeking Shell Flagship Store	Battery Repairer	Good quality, affordable price, and prompt shipping!	Taobao Marketplace, a Chinese website for online shopping
Seeking Shell Flagship Store	Battery Repairer	charges quickly and works well, and the color I like it too. Customer service attitude is also good, ask questions and answer them. Very satisfied with this purchase ,	Taobao Marketplace, a Chinese website for online shopping
Seeking Shell Flagship Store	Battery Repairer	I don't know if it works. I'm using it.	Taobao Marketplace, a Chinese website for online shopping
Seeking Shell Flagship Store	Battery Repairer	Baby has been received, very good baby	Taobao Marketplace, a Chinese website for online shopping
Seeking Shell Flagship Store	Battery Repairer	Seller service is very good, very satisfied with a shopping	Taobao Marketplace, a Chinese website for online shopping
Seeking Shell Flagship Store	Battery Repairer	Affordable price, great quality, very good shopping, next time come back!	Taobao Marketplace, a Chinese website for online shopping
Seeking Shell Flagship Store	Battery Repairer	Good quality, thick line charging fast, extended version of a line of multi-charging, interface stable, very satisfied	Taobao Marketplace, a Chinese website for online shopping
Seeking Shell Flagship Store	Battery Repairer	Seller shipped quickly, have received the item and it works as described by the seller.	Taobao Marketplace, a Chinese website for online shopping
Seeking Shell Flagship Store	Battery Repairer	High-tech quality is good, easy to use, well-packaged fast delivery	Taobao Marketplace, a Chinese website for online shopping
Seeking Shell Flagship Store	Battery Repairer	I haven't used it yet, I'll comment afterward	Taobao Marketplace, a Chinese website for online shopping
Seeking Shell Flagship Store	Battery Repairer	Charged my cell phone twice, didn't notice any noticeable effect, try it for a few more days and then follow up. I'll give it a good review first!	Taobao Marketplace, a Chinese website for online shopping

Seeking Shell Flagship Store	Battery Repairer	Haven't used it yet Don't know if it works	Taobao Marketplace, a Chinese website for online shopping
Seeking Shell Flagship Store	Battery Repairer	The product received, the quality is strong, logistics speed, packaging is also intact, great!	Taobao Marketplace, a Chinese website for online shopping
Seeking Shell Flagship Store	Battery Repairer	Fast logistics, good quality, affordable price, satisfied with a shopping.	Taobao Marketplace, a Chinese website for online shopping
Seeking Shell Flagship Store	Battery Repairer	Indeed have the effect, before playing the cell phone power drop very quickly, now much better than before, very good, satisfied, recommended to friends!	Taobao Marketplace, a Chinese website for online shopping
Seeking Shell Flagship Store	Battery Repairer	It works pretty well, it's worth the money, I'm very happy with it	Taobao Marketplace, a Chinese website for online shopping
Seeking Shell Flagship Store	Battery Repairer	Being repaired, see the display jumped the number, tried several times the phone has the effect, praise!	Taobao Marketplace, a Chinese website for online shopping
Seeking Shell Flagship Store	Battery Repairer	Not good, not ideal, almost as bad as the charger, bad, bad, bad	Taobao Marketplace, a Chinese website for online shopping
Seeking Shell Flagship Store	Battery Repairer	was good and the shipping was pretty fast, all in all it was awesome!	Taobao Marketplace, a Chinese website for online shopping
Seeking Shell Flagship Store	Battery Repairer	Great quality, very practical and very affordable, worth buying.	Taobao Marketplace, a Chinese website for online shopping
Seeking Shell Flagship Store	Battery Repairer	♡Looks good, packed carefully and very fairly priced! ✌	Taobao Marketplace, a Chinese website for online shopping
Seeking Shell Flagship Store	Battery Repairer	Logistics is fast, received the goods, high-tech ah, good quality	Taobao Marketplace, a Chinese website for online shopping
Seeking Shell Flagship Store	Battery Repairer	link to buy it, it really works, before my friend had to charge it twice a day, but now it only needs to be charged once.	Taobao Marketplace, a Chinese website for online shopping
Seeking Shell Flagship Store	Battery Repairer	It works very well, charges quickly, is effective, and is recommended.	Taobao Marketplace, a Chinese website for online shopping
Seeking Shell Flagship Store	Battery Repairer	Not bad. Good value.	Taobao Marketplace, a Chinese website for online shopping
Seeking Shell Flagship Store	Battery Repairer	The seller shipped quickly, packed tightly, easy to use, cost-effective, recommendable	Taobao Marketplace, a Chinese website for online shopping
Seeking Shell Flagship Store	Battery Repairer	Fast shipping and good quality.	Taobao Marketplace, a Chinese website for online shopping
Seeking Shell Flagship Store	Battery Repairer	good, ready to use for a period of time, hope to repair the phone battery	Taobao Marketplace, a Chinese website for online shopping
Seeking Shell Flagship Store	Battery Repairer	Baby in use, with the seller described the same, now the phone is more durable than before	Taobao Marketplace, a Chinese website for online shopping
Seeking Shell Flagship Store	Battery Repairer	Works great, charges quickly, and is exactly what I wanted!	Taobao Marketplace, a Chinese website for online shopping
Seeking Shell Flagship Store	Battery Repairer	It works.	Taobao Marketplace, a Chinese website for online shopping
Seeking Shell Flagship Store	Battery Repairer	I've used it for a few days and it feels good, so I'll charge it for a month and see what happens.	Taobao Marketplace, a Chinese website for online shopping
Seeking Shell Flagship Store	Battery Repairer	Battery repair is very good, used it for a week, feels pretty good	Taobao Marketplace, a Chinese website for online shopping
Seeking Shell Flagship Store	Battery Repairer	Customer service attitude is good. Shipping is also fast, cheaper than brick and mortar stores, cost-effective	Taobao Marketplace, a Chinese website for online shopping
Seeking Shell Flagship Store	Battery Repairer	Data cable looks good, nice color, works well, affordable, love it!	Taobao Marketplace, a Chinese website for online shopping
Seeking Shell Flagship Store	Battery Repairer	It's pretty good and flushes pretty fast as expected. It was full in a minute.	Taobao Marketplace, a Chinese website for online shopping
Seeking Shell Flagship Store	Battery Repairer	I'm not sure how well it works, I'll know after the restoration, it looks like it should be fine!	Taobao Marketplace, a Chinese website for online shopping
Seeking Shell Flagship Store	Battery Repairer	In-kind and the same as described, the quality is quite good, the seller's attitude is also good, ask questions and answer questions, the speed of delivery bar, it is worth buying!	Taobao Marketplace, a Chinese website for online shopping
Seeking Shell Flagship Store	Battery Repairer	The quality of the stuff is very good, the object matches the description of the picture, you can get your hands on it, and the price is not expensive, haha😄	Taobao Marketplace, a Chinese website for online shopping
Seeking Shell Flagship Store	Battery Repairer	I'm fixing it, I can see the numbers on the display and it feels smart.	Taobao Marketplace, a Chinese website for online shopping
Seeking Shell Flagship Store	Battery Repairer	Charging speed is very fast, half an hour can charge 80% ofthe power, the price is also preferential, can be used for a long time, logistics is also very fast, the packaging is tight, no damage, two meters is long enough, casually used	Taobao Marketplace, a Chinese website for online shopping
Seeking Shell Flagship Store	Battery Repairer	True to its name, it works well for battery reconditioning purposes	Taobao Marketplace, a Chinese website for online shopping
Seeking Shell Flagship Store	Battery Repairer	Looked very cow, technology, used a few times the phone battery is obviously more durable	Taobao Marketplace, a Chinese website for online shopping
Seeking Shell Flagship Store	Battery Repairer	It's good quality.	Taobao Marketplace, a Chinese website for online shopping
Seeking Shell Flagship Store	Battery Repairer	Baby is good, as described, good quality, value for money	Taobao Marketplace, a Chinese website for online shopping
Seeking Shell Flagship Store	Battery Repairer	Quality is very good, charging speed is very fast, the price is not expensive and affordable, worth buying	Taobao Marketplace, a Chinese website for online shopping
Seeking Shell Flagship Store	Battery Repairer	Charging quickly, battery repair is effective, worth buying	Taobao Marketplace, a Chinese website for online shopping

Seeking Shell Flagship Store	Battery Repairer	First time to buy, do not know whether it is good or not, look at the quality is good, delivery is also fast!	Taobao Marketplace, a Chinese website for online shopping
Seeking Shell Flagship Store	Battery Repairer	I'm using it, let's see how it works, I think it should work.	Taobao Marketplace, a Chinese website for online shopping
Seeking Shell Flagship Store	Battery Repairer	It looks good, it's a little slow to fix, but it works pretty well, still very good.	Taobao Marketplace, a Chinese website for online shopping
Seeking Shell Flagship Store	Battery Repairer	Great fixer, detailed instructions, easy to use, plug and play, full and stop!	Taobao Marketplace, a Chinese website for online shopping
Seeking Shell Flagship Store	Battery Repairer	an effect, the quality is also good quite good, give five stars!	Taobao Marketplace, a Chinese website for online shopping
Seeking Shell Flagship Store	Battery Repairer	The cell phone battery is indeed more durable than before, genuine, quite good.	Taobao Marketplace, a Chinese website for online shopping
Seeking Shell Flagship Store	Battery Repairer	The logistics were fast and well packaged.	Taobao Marketplace, a Chinese website for online shopping
Seeking Shell Flagship Store	Battery Repairer	You can clearly see the amount of current, the repair is stable and very convenient	Taobao Marketplace, a Chinese website for online shopping
Seeking Shell Flagship Store	Battery Repairer	Shipping is fast, the charge restorer is compact and looks good, 5 stars first(not practical then follow up review)	Taobao Marketplace, a Chinese website for online shopping
Seeking Shell Flagship Store	Battery Repairer	it for half a month, I feel that the boot and running speed has improved, and the duration of dirty time has also increased.	Taobao Marketplace, a Chinese website for online shopping
Seeking Shell Flagship Store	Battery Repairer	I've been using it for a while and it still has some battery reconditioning effect	Taobao Marketplace, a Chinese website for online shopping
Seeking Shell Flagship Store	Battery Repairer	high-tech products, there is always a process of adaptation, the next will be in accordance with the instructions of the program on the virtual power of the product for a period of time the battery slowly repair	Taobao Marketplace, a Chinese website for online shopping
Seeking Shell Flagship Store	Battery Repairer	The merchant shipped very quickly!Packed well without damage!Customer service is very enthusiastic!	Taobao Marketplace, a Chinese website for online shopping
Seeking Shell Flagship Store	Battery Repairer	Charging is stable☺and blah,perfect forold📱at home, no need to change the battery anymore♡	Taobao Marketplace, a Chinese website for online shopping
Seeking Shell Flagship Store	Battery Repairer	Charged up, has been changing numbers, very technological, should be good, not good then chase evaluation	Taobao Marketplace, a Chinese website for online shopping
Seeking Shell Flagship Store	Battery Repairer	Look at the effect, according to the business operation requirements, adhere to two months should be able to achieve the business description!	Taobao Marketplace, a Chinese website for online shopping
Seeking Shell Flagship Store	Battery Repairer	Arrived, fast shipping, things look good, quite a sense of technology, try to see if it works!	Taobao Marketplace, a Chinese website for online shopping
Seeking Shell Flagship Store	Battery Repairer	The restorer should work, it's been used a few times and the battery lasts longer than before	Taobao Marketplace, a Chinese website for online shopping
Seeking Shell Flagship Store	Battery Repairer	Received the goods very quickly and good service, looking forward to good results!	Taobao Marketplace, a Chinese website for online shopping
Seeking Shell Flagship Store	Battery Repairer	The repairer has been here for a few days, I used it on my home phone as soon as I received it, and now it's already lasting longer than before!	Taobao Marketplace, a Chinese website for online shopping
Seeking Shell Flagship Store	Battery Repairer	Received the goods, followed the instructions a few times, it works and feels good! Hope it gets better and better and saves money on battery replacement	Taobao Marketplace, a Chinese website for online shopping
Seeking Shell Flagship Store	Battery Repairer	It is a very good repair battery charger, the effect is very obvious, it is worth having, charging speed is quite fast.	Taobao Marketplace, a Chinese website for online shopping
Seeking Shell Flagship Store	Battery Repairer	Good service, fast logistics, the product works well, just do not know how long it can be used	Taobao Marketplace, a Chinese website for online shopping
Seeking Shell Flagship Store	Battery Repairer	Logistics and shipping are fast, also study this specialty, the theory is really good for the battery, and compared with the replacement of the battery is very affordable	Taobao Marketplace, a Chinese website for online shopping
Seeking Shell Flagship Store	Battery Repairer	Think the stuff is good, the quality is good, the seller's customer service attitude from start to finish is very good, the express delivery speed is super fast!	Taobao Marketplace, a Chinese website for online shopping
Seeking Shell Flagship Store	Battery Repairer	Good quality, affordable price, fast shipping, repair should be fine	Taobao Marketplace, a Chinese website for online shopping
Seeking Shell Flagship Store	Battery Repairer	Baby received and matches the picture. Hopefully it will be ideal as described. Customer service is very good, there will not be a quick answer to the answer!	Taobao Marketplace, a Chinese website for online shopping
Seeking Shell Flagship Store	Battery Repairer	Also used it for a while, the battery is lasts longer than before, overall not too bad	Taobao Marketplace, a Chinese website for online shopping
Seeking Shell Flagship Store	Battery Repairer	Customer service is very good, there is no understanding of the place are also very quickly answered!	Taobao Marketplace, a Chinese website for online shopping
Seeking Shell Flagship Store	Battery Repairer	Baby received, and the picture matches, tried to find really can repair, before the power loss can not open the machine can be charged into the customer service attitude is good, there will not be able to reply in time, satisfied!	Taobao Marketplace, a Chinese website for online shopping
Seeking Shell Flagship Store	Battery Repairer	The seller shipped quickly, the service attitude is also very good, the price of goods is more affordable, the need for friends can get, the next time will come back to patronize.	Taobao Marketplace, a Chinese website for online shopping
Seeking Shell Flagship Store	Battery Repairer	It's okay, the cell phone battery is more durable than before, the express delivery is also fast!	Taobao Marketplace, a Chinese website for online shopping
Seeking Shell Flagship Store	Battery Repairer	The restorer was received and works well, I wish the merchant a prosperous business and smooth sailing to the next level.	Taobao Marketplace, a Chinese website for online shopping

Seeking Shell Flagship Store	Battery Repairer	It works, the battery doesn't die as fast after using it	Taobao Marketplace, a Chinese website for online shopping
Seeking Shell Flagship Store	Battery Repairer	Received to try a few days feel the quality is very good, fast charging, interface stable, quite convenient to use, the price is also affordable.	Taobao Marketplace, a Chinese website for online shopping
Seeking Shell Flagship Store	Battery Repairer	It works, the battery doesn't die as fast after using it	Taobao Marketplace, a Chinese website for online shopping
Seeking Shell Flagship Store	Battery Repairer	It looks good, charging with voltage display.	Taobao Marketplace, a Chinese website for online shopping
Seeking Shell Flagship Store	Battery Repairer	the restorer is currently charging stable and helpful to the battery	Taobao Marketplace, a Chinese website for online shopping
Seeking Shell Flagship Store	Battery Repairer	It's great to try it out, a few old phones and tablets are fully charged, hopefully a few more times and these old phones will be durable again!	Taobao Marketplace, a Chinese website for online shopping
Seeking Shell Flagship Store	Battery Repairer	Used for a period of time, my ipad can only be plugged into the power supply to boot, with this charging a few times, you can leave the power supply to boot a ten minutes, is also considered an improvement, praise it, I hope that a few more times to charge will be better.	Taobao Marketplace, a Chinese website for online shopping
Seeking Shell Flagship Store	Battery Repairer	My friend introduced it to me. I used it, and I still feel that it works.	Taobao Marketplace, a Chinese website for online shopping
Seeking Shell Flagship Store	Battery Repairer	It has been charged twice, as instructed, and it does work!	Taobao Marketplace, a Chinese website for online shopping
Seeking Shell Flagship Store	Battery Repairer	The family's cell phone, tablet used for a long time, the battery does not work, online to see this battery repairer, ordered, but also try, if it can be repaired, than the replacement of the battery can be much more appropriate, customer service is good, ask questions and answer them!	Taobao Marketplace, a Chinese website for online shopping
Seeking Shell Flagship Store	Battery Repairer	Tried the cell phone battery maintenance, it does have the effect, is now trying to repair the stereo lithium batteries, the effect is good if you will come back!	Taobao Marketplace, a Chinese website for online shopping
Seeking Shell Flagship Store	Battery Repairer	Is it an IQ tax?	Taobao Marketplace, a Chinese website for online shopping
Seeking Shell Flagship Store	Battery Repairer	Does this really activate cell phone batteries?	Taobao Marketplace, a Chinese website for online shopping
Seeking Shell Flagship Store	Battery Repairer	Does battery reconditioning work?	Taobao Marketplace, a Chinese website for online shopping
Seeking Shell Flagship Store	Battery Repairer	Does this really fix cell phone batteries?	Taobao Marketplace, a Chinese website for online shopping
Seeking Shell Flagship Store	Battery Repairer	Does this work? How does it work?	Taobao Marketplace, a Chinese website for online shopping
Seeking Shell Flagship Store	Battery Repairer	The fixer is what fixes the battery, you can't fix it by just buying the cable!	Taobao Marketplace, a Chinese website for online shopping
Seeking Shell Flagship Store	Battery Repairer	This is still being tested.	Taobao Marketplace, a Chinese website for online shopping
Seeking Shell Flagship Store	Battery Repairer	acceptable	Taobao Marketplace, a Chinese website for online shopping
Seeking Shell Flagship Store	Battery Repairer	I also bought it to try it out.	Taobao Marketplace, a Chinese website for online shopping
Seeking Shell Flagship Store	Battery Repairer	The effect is less obvious	Taobao Marketplace, a Chinese website for online shopping
Jihyeong Technologies Co., Ltd.	Battery Reconditioner	not have	Taobao Marketplace, a Chinese website for online shopping
Jihyeong Technologies Co., Ltd.	Battery Reconditioner	It's off the shelves.	Taobao Marketplace, a Chinese website for online shopping
Jade Summer Trading Shop	Battery Reconditioner	Cell phone adapter receipt, immediately used, with the previous charger charging phone heat, with this phone does not heat, into the power is just as fast.	Taobao Marketplace, a Chinese website for online shopping
Jade Summer Trading Shop	Battery Reconditioner	Just used it and it's not bad.	Taobao Marketplace, a Chinese website for online shopping
Jade Summer Trading Shop	Battery Reconditioner	It won't even fit in the socket.	Taobao Marketplace, a Chinese website for online shopping
Jade Summer Trading Shop	Battery Reconditioner	Battery Repair is very effective, I only charged it once last night and today it is noticeable that the battery is more durable! It's really a miracle tool for repairing batteries!	Taobao Marketplace, a Chinese website for online shopping
Jade Summer Trading Shop	Battery Reconditioner	merchant's attitude is still very good, and a replacement color can be sent, but after receiving the goods is still not good to contact again without further reply. The merchant also admitted that there was a quality problem with the product! As the price is not expensive, no energy to negotiate with the merchant again. So comment poorly, prompting other consumers to pay attention not to buy poor quality products to raise vigilance!	Taobao Marketplace, a Chinese website for online shopping
Jade Summer Trading Shop	Battery Reconditioner	The cell phone adapter is beautifully packaged, new and chic, and satisfying to use. Thanks for receiving!	Taobao Marketplace, a Chinese website for online shopping
Jade Summer Trading Shop	Battery Reconditioner	Very effective in restoration, very well satisfied!	Taobao Marketplace, a Chinese website for online shopping

Jade Summer Trading Shop	Battery Reconditioner	Unavailable.	Taobao Marketplace, a Chinese website for online shopping
Jade Summer Trading Shop	Battery Reconditioner	There is no instruction manual, according to customer service instructions for a week, no effect, the battery is even worse, often can not be charged into the power, charging speed slows down, pure IQ tax!	Taobao Marketplace, a Chinese website for online shopping
Jade Summer Trading Shop	Battery Reconditioner	I've charged it three times already, before I went to work and it was dead by noon, today I was able to go to work at night without charging it at noon, so that means it's working!	Taobao Marketplace, a Chinese website for online shopping
Jade Summer Trading Shop	Battery Reconditioner	I don't know how to do this stuff.	Taobao Marketplace, a Chinese website for online shopping
Jade Summer Trading Shop	Battery Reconditioner	Fast shipping, beautifully packaged, good value, looking forward to the restoration, good reviews!	Taobao Marketplace, a Chinese website for online shopping
Jade Summer Trading Shop	Battery Reconditioner	and beautiful. Logistics is very fast, the service is very good.	Taobao Marketplace, a Chinese website for online shopping
Jade Summer Trading Shop	Battery Reconditioner	The effect is really great, the cell phone battery repair can be extended for a long time!	Taobao Marketplace, a Chinese website for online shopping
Jade Summer Trading Shop	Battery Reconditioner	poor quality	Taobao Marketplace, a Chinese website for online shopping
Jade Summer Trading Shop	Battery Reconditioner	Great service and fast delivery	Taobao Marketplace, a Chinese website for online shopping
Jade Summer Trading Shop	Battery Reconditioner	Great service, fast delivery, I'm good with this product	Taobao Marketplace, a Chinese website for online shopping
Jade Summer Trading Shop	Battery Reconditioner	I just got it. I don't know what it's like yet. Started plugging it in and charging it, hopefully it will work.	Taobao Marketplace, a Chinese website for online shopping
Jade Summer Trading Shop	Battery Reconditioner	Things to buy, have received the goods, look good, just do not know with a period of time has no effect?	Taobao Marketplace, a Chinese website for online shopping
Jade Summer Trading Shop	Battery Reconditioner	connect a fewLEDs, illuminating the repair of three fake words, the cost is only one or two cents! There is no tassel, selling fake goods!	Taobao Marketplace, a Chinese website for online shopping
Jade Summer Trading Shop	Battery Reconditioner	Still has a certain effect, the phone consumes power slower, not particularly hot	Taobao Marketplace, a Chinese website for online shopping
Jade Summer Trading Shop	Battery Reconditioner	Received on& ldquo;bloom& rdquo;, the quality is worrying ah!	Taobao Marketplace, a Chinese website for online shopping
Jade Summer Trading Shop	Battery Reconditioner	and exquisite, easy to use, effective relief of cell phone lag, very🈵intention, customer service attitude is very good👍👍👍👍, logistics is also fast, the next time to buy oh😮	Taobao Marketplace, a Chinese website for online shopping
Jade Summer Trading Shop	Battery Reconditioner	Fake doesn't work. It's a lie.	Taobao Marketplace, a Chinese website for online shopping
Jade Summer Trading Shop	Battery Reconditioner	The facts don't match what was stated, I bought it and used it to no avail.	Taobao Marketplace, a Chinese website for online shopping
Jade Summer Trading Shop	Battery Reconditioner	The battery restorer is not bad, tried it and it works.	Taobao Marketplace, a Chinese website for online shopping
Jade Summer Trading Shop	Battery Reconditioner	Habitually favorable reviews. Used to be unreliable.	Taobao Marketplace, a Chinese website for online shopping
Jade Summer Trading Shop	Battery Reconditioner	This cell phone adapter works great, I've used it for a day longer than usual, and my phone doesn't spin around!	Taobao Marketplace, a Chinese website for online shopping
Jade Summer Trading Shop	Battery Reconditioner	I charged my phone yesterday when I received it and did see a number added to the battery health, so it seems to be useful, hopefully it works well.	Taobao Marketplace, a Chinese website for online shopping
Jade Summer Trading Shop	Battery Reconditioner	🥰	Taobao Marketplace, a Chinese website for online shopping
Jade Summer Trading Shop	Battery Reconditioner	Repair is indeed very good to use, charging enemy enemy A fall, only to repair five minutes with the new phone exactly the same, super cost-effective good goods very powerful, faster than the original charging several times faster than the high-speed rail is much more powerful than the Chinese goods is good to give. Rest assured that the order, better than buying a new phone. Five stars praise solid good goods	Taobao Marketplace, a Chinese website for online shopping
Jade Summer Trading Shop	Battery Reconditioner	And the store described the same, used a few times to feel the battery is not hot, the use of much smoother, pro assured purchase it!	Taobao Marketplace, a Chinese website for online shopping
Jade Summer Trading Shop	Battery Reconditioner	I used it very very well, the phone doesn't lag anymore. The battery is back to normal.	Taobao Marketplace, a Chinese website for online shopping
Jade Summer Trading Shop	Battery Reconditioner	Received the cell phone battery repairer. I've seen a lot of live broadcasts about it, and it was saying that plugging it in would fix the battery. I tried it with my old cell phone, it originally took more than ten hours to be fully charged, this time it only took more than four hours to be full.	Taobao Marketplace, a Chinese website for online shopping
Jade Summer Trading Shop	Battery Reconditioner	The battery repairer is convenient and applicable, good quality, good price, is a good product, the cell phone battery tube soon have a better repair effect	Taobao Marketplace, a Chinese website for online shopping
Jade Summer Trading Shop	Battery Reconditioner	The product received is trying, before I charge, the phone old power outage yesterday charged once, the phone does not power up, good use, we can rest assured that the purchase.	Taobao Marketplace, a Chinese website for online shopping

Jade Summer Trading Shop	Battery Reconditioner	Yes, my cell phone broke down, I thought I had to replace the screen, it wouldn't turn on, my friend used this repair charger to charge it, hey, hey, it turned on, thank God, so I decided to buy one!	Taobao Marketplace, a Chinese website for online shopping
Jade Summer Trading Shop	Battery Reconditioner	Baby received today received the upgraded version of the quality is very good, I will introduce friends over to buy.	Taobao Marketplace, a Chinese website for online shopping
Jade Summer Trading Shop	Battery Reconditioner	Will you grow a bag in your head if you buy this?	Taobao Marketplace, a Chinese website for online shopping
Jade Summer Trading Shop	Battery Reconditioner	Does it really fix batteries? An IQ tax?	Taobao Marketplace, a Chinese website for online shopping
Jade Summer Trading Shop	Battery Reconditioner	Is there a noticeable effect? How long does the battery last longer than before?	Taobao Marketplace, a Chinese website for online shopping
Jade Summer Trading Shop	Battery Reconditioner	Is this thing gonna work or not?	Taobao Marketplace, a Chinese website for online shopping
Jade Summer Trading Shop	Battery Reconditioner	What do you mean? Doesn't it work?	Taobao Marketplace, a Chinese website for online shopping
Jade Summer Trading Shop	Battery Reconditioner	So just a psychological comfort, the actual people do not have the means, the instrument can be checked and tested out of the more fruit, self-deception only.	Taobao Marketplace, a Chinese website for online shopping
Jade Summer Trading Shop	Battery Reconditioner	It should lastabout2longer than before.	Taobao Marketplace, a Chinese website for online shopping
Jade Summer Trading Shop	Battery Reconditioner	No, I've been duped.	Taobao Marketplace, a Chinese website for online shopping
Hang Seng Enterprise Store	Intelligent Pulse Voltage Regulator for Electric Vehicles	This new ChaoWei intelligent pulse voltage stabilizer let me say goodbye to the problem of unstable voltage, and the size is small and easy to carry, where it is unstable to put it!The description matches the condition and the same, and I will comment tomorrow after using it!	Taobao Marketplace, a Chinese website for online shopping
Hang Seng Enterprise Store	Intelligent Pulse Voltage Regulator for Electric Vehicles	Reliable quality, still maintains good repair effect after using for some time. Multifunctional design, not only can repair the battery, but also can detect the health of the battery. Safe and reliable, with over-charging, over-discharging, short-circuit protection, use more assured.	Taobao Marketplace, a Chinese website for online shopping
Hang Seng Enterprise Store	Intelligent Pulse Voltage Regulator for Electric Vehicles	It is genuine, good quality, charged overnight, the next day the tram did ride faster and more vigorously than before. Intend to buy back again, give merchants praise, this price is cheaper than other homes. Also send adapter, praise	Taobao Marketplace, a Chinese website for online shopping
Hang Seng Enterprise Store	Intelligent Pulse Voltage Regulator for Electric Vehicles	Packaging is intact, logistics is also very fast, the appearance of good design, looks quite advanced, advanced, more use a few times to see the effect of the tram running more powerful, very good!	Taobao Marketplace, a Chinese website for online shopping
Hang Seng Enterprise Store	Intelligent Pulse Voltage Regulator for Electric Vehicles	Charged the battery three or four times in a row and it didn't charge. I can't even leave the house.	Taobao Marketplace, a Chinese website for online shopping
Hang Seng Enterprise Store	Intelligent Pulse Voltage Regulator for Electric Vehicles	service, good attitude,.Logistics is very fast, give five-star praise	Taobao Marketplace, a Chinese website for online shopping
Hang Seng Enterprise Store	Intelligent Pulse Voltage Regulator for Electric Vehicles	I just received it, I don't know how effective it is, it's not good to comment.	Taobao Marketplace, a Chinese website for online shopping
Hang Seng Enterprise Store	Intelligent Pulse Voltage Regulator for Electric Vehicles	Usage: followed instructions, only shows battery repair, but never changes.	Taobao Marketplace, a Chinese website for online shopping
Hang Seng Enterprise Store	Intelligent Pulse Voltage Regulator for Electric Vehicles	Don't be fooled, it doesn't work at all, I took it apart to see, you can compare it with the regular battery charger, simple can't be any simpler, absolutely shoddy and fake!	Taobao Marketplace, a Chinese website for online shopping
Hang Seng Enterprise Store	Intelligent Pulse Voltage Regulator for Electric Vehicles	Usage: is not a mistake, not matching at all	Taobao Marketplace, a Chinese website for online shopping
Hang Seng Enterprise Store	Intelligent Pulse Voltage Regulator for Electric Vehicles	liars	Taobao Marketplace, a Chinese website for online shopping

Hang Seng Enterprise Store	Intelligent Pulse Voltage Regulator for Electric Vehicles	The service is too bad, the return does not give shipping costs, bought goods in Yiwu, return to pay for their own shipping costs, but also sent to Shaanxi, really speechless.	Taobao Marketplace, a Chinese website for online shopping
Hang Seng Enterprise Store	Intelligent Pulse Voltage Regulator for Electric Vehicles	There's not even a manual. What the hell.	Taobao Marketplace, a Chinese website for online shopping
Hang Seng Enterprise Store	Intelligent Pulse Voltage Regulator for Electric Vehicles	It's not working.	Taobao Marketplace, a Chinese website for online shopping
Hang Seng Enterprise Store	Intelligent Pulse Voltage Regulator for Electric Vehicles	Repairer is really good, the business is also free to send the adapter, just right, one step, do not have to match my own again, a penny a penny, the quality is also very good, everyone rest assured that the next order	Taobao Marketplace, a Chinese website for online shopping
Hang Seng Enterprise Store	Intelligent Pulse Voltage Regulator for Electric Vehicles	Repair effect is good, bought for the battery car battery charging, small size is very convenient to use, charging repair effect is also very good, a very good intelligent pulse voltage stabilizer	Taobao Marketplace, a Chinese website for online shopping
Hang Seng Enterprise Store	Intelligent Pulse Voltage Regulator for Electric Vehicles	I've been using it for a few days now and it's not working	Taobao Marketplace, a Chinese website for online shopping
Hang Seng Enterprise Store	Intelligent Pulse Voltage Regulator for Electric Vehicles	Interface does not match, can not be used, before buying to determine whether there is no shipping insurance, or return is very troublesome. Later, I wanted to buy a transfer interface has not been used, put on the table was directly plugged into the colleague burned, can only be thrown away, so this point should also pay attention to.	Taobao Marketplace, a Chinese website for online shopping
Hang Seng Enterprise Store	Intelligent Pulse Voltage Regulator for Electric Vehicles	A very good product, genuine.	Taobao Marketplace, a Chinese website for online shopping
Hang Seng Enterprise Store	Intelligent Pulse Voltage Regulator for Electric Vehicles	Fast shipping, good business service, the description matches the price.	Taobao Marketplace, a Chinese website for online shopping
Hang Seng Enterprise Store	Intelligent Pulse Voltage Regulator for Electric Vehicles	It's genuine, worth having, will repurchase.	Taobao Marketplace, a Chinese website for online shopping
Hang Seng Enterprise Store	Intelligent Pulse Voltage Regulator for Electric Vehicles	Not good for that.	Taobao Marketplace, a Chinese website for online shopping
Hang Seng Enterprise Store	Intelligent Pulse Voltage Regulator for Electric Vehicles	Battery charging is effective, not a big improvement on the battery, overall it's ok, when buying pay attention to your needs, starving battery can be repaired, did not try.	Taobao Marketplace, a Chinese website for online shopping
Hang Seng Enterprise Store	Intelligent Pulse Voltage Regulator for Electric Vehicles	Affordable and worth buying!👍👍👍👍👍👍👍👍👍👍👍👍👍👍👍👍👍👍👍👍👍👍	Taobao Marketplace, a Chinese website for online shopping
Hang Seng Enterprise Store	Intelligent Pulse Voltage Regulator for Electric Vehicles	Used it once, the effect of the battery will have to wait a year later	Taobao Marketplace, a Chinese website for online shopping
Hang Seng Enterprise Store	Intelligent Pulse Voltage Regulator for Electric Vehicles	Originally bought the charger I do not know is the reason for the battery or charger reasons, charging a night also shows no power (battery display icon does not show), today with just received the baby re-charging, eh, immediately show that there is power (icon shows that there is a grid of electricity), indeed high-tech products Oh, (intelligent pulse repair machine, full of self-stopping) and save a few hundred oceans! Favorable comments, all five stars!	Taobao Marketplace, a Chinese website for online shopping

Hang Seng Enterprise Store	Intelligent Pulse Voltage Regulator for Electric Vehicles	The service attitude of the owner is really good, and the delivery is very fast. The quality of the goods is also quite good. Baby is good, exactly as described, like can rest assured to buy.	Taobao Marketplace, a Chinese website for online shopping
Hang Seng Enterprise Store	Intelligent Pulse Voltage Regulator for Electric Vehicles	The store put five-star positive feedback card back to the red packets in the courier, positive feedback finished, the results of the store does not recognize the activity, said this activity, not this activity also put positive feedback to cash to do what, really can not figure out the brain was kicked by a donkey, it is really speechless**to the speechless to open the door to speechless to the house, the	Taobao Marketplace, a Chinese website for online shopping
Hang Seng Enterprise Store	Intelligent Pulse Voltage Regulator for Electric Vehicles	Just received the goods, look at the quality is okay, activate the function to try to see how the	Taobao Marketplace, a Chinese website for online shopping
Hang Seng Enterprise Store	Intelligent Pulse Voltage Regulator for Electric Vehicles	an excellent	Taobao Marketplace, a Chinese website for online shopping
Hang Seng Enterprise Store	Intelligent Pulse Voltage Regulator for Electric Vehicles	Baby received on the can't wait to prepare for the repair work, my minivan forgot to turn off the key a few days later only to find, hit the car a little response is not, multimeter measure only5.6volts, today repair six hours to13.5volts, very good repair machine	Taobao Marketplace, a Chinese website for online shopping
Hang Seng Enterprise Store	Intelligent Pulse Voltage Regulator for Electric Vehicles	It's a pulse charger.	Taobao Marketplace, a Chinese website for online shopping
Hang Seng Enterprise Store	Intelligent Pulse Voltage Regulator for Electric Vehicles	Overall rating: good Value for money: good Appearance and workmanship: good	Taobao Marketplace, a Chinese website for online shopping
Hang Seng Enterprise Store	Intelligent Pulse Voltage Regulator for Electric Vehicles	Overall evaluation: good Use: this intelligent pulse repair machine has not been used, wait for the use of in the evaluation	Taobao Marketplace, a Chinese website for online shopping
Hang Seng Enterprise Store	Intelligent Pulse Voltage Regulator for Electric Vehicles	This charger doesn't have smart features, and it bulged a good battery on the first charge	Taobao Marketplace, a Chinese website for online shopping
Hang Seng Enterprise Store	Intelligent Pulse Voltage Regulator for Electric Vehicles	good to use, received the goods to the battery charged, save the money to change the battery, and the volume of things is not big, usually storage is also convenient, good. Customer service is very patient to explain to me how to use, but also sent me a video recorded by their staff to explain, very detailed, follow the operation is good!	Taobao Marketplace, a Chinese website for online shopping
Hang Seng Enterprise Store	Intelligent Pulse Voltage Regulator for Electric Vehicles	Appearance workmanship: good	Taobao Marketplace, a Chinese website for online shopping
Hang Seng Enterprise Store	Intelligent Pulse Voltage Regulator for Electric Vehicles	Charging it up does nothing, the battery is still dropping out and not charging in, I don't feel like the fix is working	Taobao Marketplace, a Chinese website for online shopping
Hang Seng Enterprise Store	Intelligent Pulse Voltage Regulator for Electric Vehicles	, the old iron door, this repair machine directly can not be used, can not be repaired, and the same as the original, the purchase of regret, I hope not to be fooled!	Taobao Marketplace, a Chinese website for online shopping
Hang Seng Enterprise Store	Intelligent Pulse Voltage Regulator for Electric Vehicles	Overall Rating:: Timely delivery, baby received, according to the description of the manual for the operation of the test charging, the effect is better, is the family has an electric bike a necessary equipment.	Taobao Marketplace, a Chinese website for online shopping
Hang Seng Enterprise Store	Intelligent Pulse Voltage Regulator for Electric Vehicles	Have not used it yet, this is powered up and functioning properly, allow for a review later.	Taobao Marketplace, a Chinese website for online shopping

Hang Seng Enterprise Store	Intelligent Pulse Voltage Regulator for Electric Vehicles	The Radical Talking Battery is about the same, the repair function is IQ, it's better to use it as a charger.	Taobao Marketplace, a Chinese website for online shopping
Hang Seng Enterprise Store	Intelligent Pulse Voltage Regulator for Electric Vehicles	Overall assessment: quality	Taobao Marketplace, a Chinese website for online shopping
Hang Seng Enterprise Store	Intelligent Pulse Voltage Regulator for Electric Vehicles	atmospheric appearance, a test and the manual to solve the introduction of the exact match, should be genuine undoubtedly. Customer service staff attitude is very good can be very patient to help solve the various problems encountered. Logistics is also much faster than the beginning of the idea. Subsequent use will also follow up!	Taobao Marketplace, a Chinese website for online shopping
Hang Seng Enterprise Store	Intelligent Pulse Voltage Regulator for Electric Vehicles	use, activate and then repair, step by step to follow the merchant sent me a video, the operation is also simple, each piece I have repaired two hours, combined, feel the car is very strong, a start, stay away from the distance is also long-lasting, it's really good, the merchant is also a comparison of a number of home before placing an order, my choice is not wrong!	Taobao Marketplace, a Chinese website for online shopping
Hang Seng Enterprise Store	Intelligent Pulse Voltage Regulator for Electric Vehicles	After using it, it feels good to repair the battery, the capacity has increased, it's genuine, recommended!	Taobao Marketplace, a Chinese website for online shopping
Hang Seng Enterprise Store	Intelligent Pulse Voltage Regulator for Electric Vehicles	Pulse repair all-in-one machine received very good use, the old battery into a new battery, start a very strong, run more kilometers. The quality is superb, please feel free to buy. Product description of the same, the business is also very patient, has been to explain to me how to use, to me also sent a video of the operation, follow the steps to operate up very convenient! Give a six-star praise.	Taobao Marketplace, a Chinese website for online shopping
Hang Seng Enterprise Store	Intelligent Pulse Voltage Regulator for Electric Vehicles	Usage: the logistics is okay, the charger is also good	Taobao Marketplace, a Chinese website for online shopping
Hang Seng Enterprise Store	Intelligent Pulse Voltage Regulator for Electric Vehicles	Good review!	Taobao Marketplace, a Chinese website for online shopping
Hang Seng Enterprise Store	Intelligent Pulse Voltage Regulator for Electric Vehicles	Baby has received, is what I want, feel very good, the quality is also quite good, there are problems customer service is also quickly resolved, the attitude is very good, must praise!	Taobao Marketplace, a Chinese website for online shopping
Hang Seng Enterprise Store	Intelligent Pulse Voltage Regulator for Electric Vehicles	It's okay, but the cord on the charger is so flimsy that I'm afraid it'll burn out over time.	Taobao Marketplace, a Chinese website for online shopping
Hang Seng Enterprise Store	Intelligent Pulse Voltage Regulator for Electric Vehicles	Nice and convenient Authentic card card no problem Will try later	Taobao Marketplace, a Chinese website for online shopping
Hang Seng Enterprise Store	Intelligent Pulse Voltage Regulator for Electric Vehicles	The goods arrived and used it, charging an hour and a half to13.25vanother three hours after full. Charger workmanship fine appearance, very good machine.	Taobao Marketplace, a Chinese website for online shopping
Hang Seng Enterprise Store	Intelligent Pulse Voltage Regulator for Electric Vehicles	charging, the family has an old-fashioned charger, see the Internet has a new type of intelligent charger can be full of power since the stop, think this can save energy, when the shipment of logistics is also not bad quickly received, this charger to get the details are still very good, the shell beautifully good-looking small small one does not take up space, clamps are also enough to not be afraid of charging will be loosened when charging, charging current according to the battery Charging current according to the battery to adjust the current charging. Capacity of small batteries quickly filled, in general, this is still more convenient,	Taobao Marketplace, a Chinese website for online shopping

Hang Seng Enterprise Store	Intelligent Pulse Voltage Regulator for Electric Vehicles	pretty good	Taobao Marketplace, a Chinese website for online shopping
Hang Seng Enterprise Store	Intelligent Pulse Voltage Regulator for Electric Vehicles	Intelligent pulse repair is very good to use plugged into the electric selection on the positioning of the automatic then, small and lightweight, repair a little bit, the battery has a lot of strength.	Taobao Marketplace, a Chinese website for online shopping
Hang Seng Enterprise Store	Intelligent Pulse Voltage Regulator for Electric Vehicles	Baby has received, small and exquisite, inexpensive and beautiful, worth having!	Taobao Marketplace, a Chinese website for online shopping
Hang Seng Enterprise Store	Intelligent Pulse Voltage Regulator for Electric Vehicles	Electric car in the home put did not ride, the recent warm weather, want to ride to work, uphill no strength, accidentally found this product, activate the restoration of uphill with strength, kilometers than before, the money spent on the people feel like doing the job, it's really good.	Taobao Marketplace, a Chinese website for online shopping
Hang Seng Enterprise Store	Intelligent Pulse Voltage Regulator for Electric Vehicles	Buyer does not comment within15days	Taobao Marketplace, a Chinese website for online shopping
Hang Seng Enterprise Store	Intelligent Pulse Voltage Regulator for Electric Vehicles	Fake goods. Don't be fooled.	Taobao Marketplace, a Chinese website for online shopping
Hang Seng Enterprise Store	Intelligent Pulse Voltage Regulator for Electric Vehicles	Buyer does not comment within15days	Taobao Marketplace, a Chinese website for online shopping
Hang Seng Enterprise Store	Intelligent Pulse Voltage Regulator for Electric Vehicles	This dealer is a fake and doesn't care	Taobao Marketplace, a Chinese website for online shopping
Hang Seng Enterprise Store	Intelligent Pulse Voltage Regulator for Electric Vehicles	fix60Vbattery	Taobao Marketplace, a Chinese website for online shopping
Hang Seng Enterprise Store	Intelligent Pulse Voltage Regulator for Electric Vehicles	Will it charge72volt battery car?	Taobao Marketplace, a Chinese website for online shopping
Hang Seng Enterprise Store	Intelligent Pulse Voltage Regulator for Electric Vehicles	Still25%after6hourssummer repair	Taobao Marketplace, a Chinese website for online shopping
Hang Seng Enterprise Store	Intelligent Pulse Voltage Regulator for Electric Vehicles	Is it a pure copper motor?	Taobao Marketplace, a Chinese website for online shopping
Hang Seng Enterprise Store	Intelligent Pulse Voltage Regulator for Electric Vehicles	Can you charge five batteries? It's6OV	Taobao Marketplace, a Chinese website for online shopping
Hang Seng Enterprise Store	Intelligent Pulse Voltage Regulator for Electric Vehicles	Pro, does this fix the battery?	Taobao Marketplace, a Chinese website for online shopping
Hang Seng Enterprise Store	Intelligent Pulse Voltage Regulator for Electric Vehicles	terrestrial electric vehicle (TEMV)	Taobao Marketplace, a Chinese website for online shopping

Hang Seng Enterprise Store	Intelligent Pulse Voltage Regulator for Electric Vehicles	How long does a restored battery last?	Taobao Marketplace, a Chinese website for online shopping
Hang Seng Enterprise Store	Intelligent Pulse Voltage Regulator for Electric Vehicles	Can a water battery be repaired?	Taobao Marketplace, a Chinese website for online shopping
Hang Seng Enterprise Store	Intelligent Pulse Voltage Regulator for Electric Vehicles	Will it work fine after one fix?	Taobao Marketplace, a Chinese website for online shopping
Hang Seng Enterprise Store	Intelligent Pulse Voltage Regulator for Electric Vehicles	Is it a solid brass movement?	Taobao Marketplace, a Chinese website for online shopping
Hang Seng Enterprise Store	Intelligent Pulse Voltage Regulator for Electric Vehicles	How many hours can it take to charge the battery?	Taobao Marketplace, a Chinese website for online shopping
Hang Seng Enterprise Store	Intelligent Pulse Voltage Regulator for Electric Vehicles	Can you detect current and voltage?	Taobao Marketplace, a Chinese website for online shopping
Hang Seng Enterprise Store	Intelligent Pulse Voltage Regulator for Electric Vehicles	Is it an IQ tax?	Taobao Marketplace, a Chinese website for online shopping
Hang Seng Enterprise Store	Intelligent Pulse Voltage Regulator for Electric Vehicles	Can you recharge lithium batteries	Taobao Marketplace, a Chinese website for online shopping
Hang Seng Enterprise Store	Intelligent Pulse Voltage Regulator for Electric Vehicles	Can a flushed drum battery be repaired?	Taobao Marketplace, a Chinese website for online shopping
Hang Seng Enterprise Store	Intelligent Pulse Voltage Regulator for Electric Vehicles	Can this thing be wrapped?	Taobao Marketplace, a Chinese website for online shopping
Hang Seng Enterprise Store	Intelligent Pulse Voltage Regulator for Electric Vehicles	For those who have used it, does it really fix the battery? Enthusiastic friends to answer, to seek the truth. After all, we are all consumers	Taobao Marketplace, a Chinese website for online shopping
Hang Seng Enterprise Store	Intelligent Pulse Voltage Regulator for Electric Vehicles	Who has bought it and how does it work?	Taobao Marketplace, a Chinese website for online shopping
Hang Seng Enterprise Store	Intelligent Pulse Voltage Regulator for Electric Vehicles	Can the Super Yee Wee battery be repaired?	Taobao Marketplace, a Chinese website for online shopping
Hang Seng Enterprise Store	Intelligent Pulse Voltage Regulator for Electric Vehicles	Will a72volt32Ado?	Taobao Marketplace, a Chinese website for online shopping
Hang Seng Enterprise Store	Intelligent Pulse Voltage Regulator for Electric Vehicles	Can you fix a starved battery?	Taobao Marketplace, a Chinese website for online shopping

Hang Seng Enterprise Store	Intelligent Pulse Voltage Regulator for Electric Vehicles	Can youfix6batteries in parallel?	Taobao Marketplace, a Chinese website for online shopping
Hang Seng Enterprise Store	Intelligent Pulse Voltage Regulator for Electric Vehicles	Can you fix a starved battery?	Taobao Marketplace, a Chinese website for online shopping
Hang Seng Enterprise Store	Intelligent Pulse Voltage Regulator for Electric Vehicles	Does the lithium battery work?	Taobao Marketplace, a Chinese website for online shopping
Hang Seng Enterprise Store	Intelligent Pulse Voltage Regulator for Electric Vehicles	should not	Taobao Marketplace, a Chinese website for online shopping
Hang Seng Enterprise Store	Intelligent Pulse Voltage Regulator for Electric Vehicles	I bought the unused, can not charge lithium batteries, single battery charging, the maximum can charge200	Taobao Marketplace, a Chinese website for online shopping
Hang Seng Enterprise Store	Intelligent Pulse Voltage Regulator for Electric Vehicles	I regretted buying it when I received the goods, bragging is not a capital offense	Taobao Marketplace, a Chinese website for online shopping
Hang Seng Enterprise Store	Intelligent Pulse Voltage Regulator for Electric Vehicles	No pure copper for smartphones	Taobao Marketplace, a Chinese website for online shopping
Hang Seng Enterprise Store	Intelligent Pulse Voltage Regulator for Electric Vehicles	Personally, I don't see any difference between adding this charge and not adding it, the	Taobao Marketplace, a Chinese website for online shopping
Hang Seng Enterprise Store	Intelligent Pulse Voltage Regulator for Electric Vehicles	should not	Taobao Marketplace, a Chinese website for online shopping
Hang Seng Enterprise Store	Intelligent Pulse Voltage Regulator for Electric Vehicles	It doesn't work. It only works as a charger. It doesn't work with a repaired battery	Taobao Marketplace, a Chinese website for online shopping
Hang Seng Enterprise Store	Intelligent Pulse Voltage Regulator for Electric Vehicles	There is no way it can be repaired, it charges very slowly, and it's okay for sub-handling at home.	Taobao Marketplace, a Chinese website for online shopping
Hang Seng Enterprise Store	Intelligent Pulse Voltage Regulator for Electric Vehicles	I'm the water battery.	Taobao Marketplace, a Chinese website for online shopping
Hang Seng Enterprise Store	Intelligent Pulse Voltage Regulator for Electric Vehicles	How can it be thatamazing/:802/:802/:802	Taobao Marketplace, a Chinese website for online shopping
Hang Seng Enterprise Store	Intelligent Pulse Voltage Regulator for Electric Vehicles	I'm not an insider, so I don't know.	Taobao Marketplace, a Chinese website for online shopping
Hang Seng Enterprise Store	Intelligent Pulse Voltage Regulator for Electric Vehicles	It doesn't work. IQ tax.	Taobao Marketplace, a Chinese website for online shopping

Hang Seng Enterprise Store	Intelligent Pulse Voltage Regulator for Electric Vehicles	can	Taobao Marketplace, a Chinese website for online shopping
Hang Seng Enterprise Store	Intelligent Pulse Voltage Regulator for Electric Vehicles	Let's be polite. My father asked me to buy this, so we can't refuse, right?	Taobao Marketplace, a Chinese website for online shopping
Hang Seng Enterprise Store	Intelligent Pulse Voltage Regulator for Electric Vehicles	(usually used in the negative) have the possibility of	Taobao Marketplace, a Chinese website for online shopping
Hang Seng Enterprise Store	Intelligent Pulse Voltage Regulator for Electric Vehicles	Repair good also not good use can not run for two kilometers, it is dead not good use, do not buy change a piece of two change battery better is only10yuan20or so than the repair of the battery is too much better	Taobao Marketplace, a Chinese website for online shopping
Hang Seng Enterprise Store	Intelligent Pulse Voltage Regulator for Electric Vehicles	Is it all-inclusive?	Taobao Marketplace, a Chinese website for online shopping
Hang Seng Enterprise Store	Intelligent Pulse Voltage Regulator for Electric Vehicles	Useless but the merchant service is also very not work, received a return without talking about the refund platform intervention before refunding, the shipping cost is also their own out, and then the platform advances, can only buy things on Taobao.	Taobao Marketplace, a Chinese website for online shopping
Hang Seng Enterprise Store	Intelligent Pulse Voltage Regulator for Electric Vehicles	No effect at all, charged it3times and fixed3times purely without taking anything.	Taobao Marketplace, a Chinese website for online shopping
Hang Seng Enterprise Store	Intelligent Pulse Voltage Regulator for Electric Vehicles	I'm just using it now. It'll be a couple days before I get any results.	Taobao Marketplace, a Chinese website for online shopping
Hang Seng Enterprise Store	Intelligent Pulse Voltage Regulator for Electric Vehicles	I don't know.	Taobao Marketplace, a Chinese website for online shopping
Hang Seng Enterprise Store	Intelligent Pulse Voltage Regulator for Electric Vehicles	It can be repaired but a repaired battery won't last more than two kilometers You can buy it as a charger at home	Taobao Marketplace, a Chinese website for online shopping
Hang Seng Enterprise Store	Intelligent Pulse Voltage Regulator for Electric Vehicles	should not	Taobao Marketplace, a Chinese website for online shopping
Hang Seng Enterprise Store	Intelligent Pulse Voltage Regulator for Electric Vehicles	I don't know. Never bought one.	Taobao Marketplace, a Chinese website for online shopping
Hang Seng Enterprise Store	Intelligent Pulse Voltage Regulator for Electric Vehicles	No, only lead acid.	Taobao Marketplace, a Chinese website for online shopping
Have a dream home franchise	New Ultraviolet [illegible]	I have not yet experienced the effect! There is no use for Grace! IQ tax!	Taobao Marketplace, a Chinese website for online shopping
Have a dream home franchise	New Ultraviolet [illegible]	I've used it three times for four or five hours each time and it just blew up the battery, is that a problem with the usage?	Taobao Marketplace, a Chinese website for online shopping
Have a dream home franchise	New Ultraviolet [illegible]	The service was extremely poor!	Taobao Marketplace, a Chinese website for online shopping
Have a dream home franchise	New Ultraviolet [illegible]	The courier's too slow.	Taobao Marketplace, a Chinese website for online shopping
Have a dream home franchise	New Ultraviolet Battery Repairer	The courier has been confiscated, but also did not call, and later found that it has been returned to the business with the merchant business also signed, but has not been refunded, and then the business ignored me, equal to spend money to buy a lonely, this is a what is the reasoning?	Taobao Marketplace, a Chinese website for online shopping

Have a dream home franchise	New Ultraviolet B[illegible]	Spend a couple bucks on a piece of crap	Taobao Marketplace, a Chinese website for online shopping
Have a dream home franchise	New Ultraviolet B[illegible]	It doesn't work, what a fix, it's a rip-off.	Taobao Marketplace, a Chinese website for online shopping
Have a dream home franchise	New Ultraviolet B[illegible]	nothing good be said about it	Taobao Marketplace, a Chinese website for online shopping
Have a dream home franchise	New Ultraviolet Battery Repairer	I have nothing to comment on and I can't blame the store. All I can say is that those shameful scammers will not end well. People do what they do and it will never last. Does this so called fixer work? I have used it at least three times now and it does amount to putting a*.	Taobao Marketplace, a Chinese website for online shopping
Have a dream home franchise	New Ultraviolet B[illegible]	special, and messages to customer service were not returned or addressed!	Taobao Marketplace, a Chinese website for online shopping
Have a dream home franchise	New Ultraviolet B[illegible]	serviceable	Taobao Marketplace, a Chinese website for online shopping
Have a dream home franchise	New Ultraviolet Battery Repairer	The feeling of stepping on the pit, received the appearance of the goods became so, not smooth, the instructions Liao Liao a few sentences, so that every month to repair one to two times, do not see the effect, feel paid IQ tax.	Taobao Marketplace, a Chinese website for online shopping
Have a dream home franchise	New Ultraviolet Battery R[illegible]	Good battery, after using this contraption, the charger keeps showing it is not full and will not cut off automatically, causing the battery to bulge! Please don't ever buy one!	Taobao Marketplace, a Chinese website for online shopping
Have a dream home franchise	New Ultraviolet B[illegible]	I have not used it yet and will comment afterward.	Taobao Marketplace, a Chinese website for online shopping
Have a dream home franchise	New Ultraviolet B[illegible]	I haven't used it yet.	Taobao Marketplace, a Chinese website for online shopping
Have a dream home franchise	New Ultraviolet B[illegible]	garbage	Taobao Marketplace, a Chinese website for online shopping
Have a dream home franchise	New Ultraviolet B[illegible]	Yes, you can.	Taobao Marketplace, a Chinese website for online shopping
Have a dream home franchise	New Ultraviolet Battery Repairer	The product did not use the instructions, a moment to say that I forgot to put, a moment to say that there is no said that there is no instruction manual, but the box is written clearly, before using the detailed reading of the instruction manual, I raised the issue of no one to respond to, let alone if it is dealt with, it is very irresponsible.	Taobao Marketplace, a Chinese website for online shopping
Have a dream home franchise	New Ultraviolet B[illegible]	The goods have been received and are of good quality and are being used.	Taobao Marketplace, a Chinese website for online shopping
Have a dream home franchise	New Ultraviolet Battery Repairer	Not good to use! I used three times, the first charging like the manufacturer said more than seven hours charger is also automatically disconnected, but ride up a go or the second before the same as a grid, the second and third time charging' more than twelve hours or not!!!! Suggest that you do not buy a bit of a flimflam.	Taobao Marketplace, a Chinese website for online shopping
Have a dream home franchise	New Ultraviolet Battery R[illegible]	announcements, the fan does not work, there is no pass inside the air intake grill, no more work response, I observed for an hour does not heat up, the	Taobao Marketplace, a Chinese website for online shopping
Have a dream home franchise	New Ultraviolet B[illegible]	There is no production license, no three C certification, purely three products.	Taobao Marketplace, a Chinese website for online shopping
Have a dream home franchise	New Ultraviolet B[illegible]	Haven't tested the results yet, we'll see in a week.	Taobao Marketplace, a Chinese website for online shopping
Have a dream home franchise	New Ultraviolet Battery Repairer	I went to the pickup point yesterday to pick up the goods back, open the package to try, feel the quality can be, I am more satisfied, thanks to the service of the business, if I later use the bad look at the situation to buy again!	Taobao Marketplace, a Chinese website for online shopping
Have a dream home franchise	New Ultraviolet B[illegible]	Very effective.	Taobao Marketplace, a Chinese website for online shopping
Have a dream home franchise	New Ultraviolet B[illegible]	Not yet used, first praise	Taobao Marketplace, a Chinese website for online shopping
Have a dream home franchise	New Ultraviolet Battery Repairer	In the jittery voice was planted, the first time to buy, after receiving the check is genuine, to see the effect after use, so far with the feeling of texture is good,, and came to Taobao to buy a guarantee, things can be good to receive the goods is what I want, the	Taobao Marketplace, a Chinese website for online shopping
Have a dream home franchise	New Ultraviolet B[illegible]	Genuine product guarantee good price good value for money worth having	Taobao Marketplace, a Chinese website for online shopping
Have a dream home franchise	New Ultraviolet Battery Repairer	It is genuine, very good, very good, easy to use, the seller patiently explains, the price is not expensive, the battery repair is very good. More powerful than before. In-kind and the same as described, the quality is quite good, the seller's attitude is also good, ask questions and answer them, the speed of delivery bar, worth buying Oh shipping very quickly, have received the goods, the goods and the description is almost the same, the quality is no problem, the key price is also appropriate, buy here is still very assured, praise!	Taobao Marketplace, a Chinese website for online shopping
Have a dream home franchise	New Ultraviolet Battery R[illegible]	The first time to buy online, but also look at the evaluation before buying, the quality is not bad, but also very cheap, the need for friends can rest assured to buy!	Taobao Marketplace, a Chinese website for online shopping

Have a dream home franchise	New Ultraviolet Battery Repairer	before and it works great. See Taobao also have. And the price is cheap. Just buy to try. The effect is just as good, and it is also genuine. The date is the latest, which is very safe ingredients. It's still a good deal to buy here, you can save as much money as you want, I'm buying back again, very good product!	Taobao Marketplace, a Chinese website for online shopping
Have a dream home franchise	New Ultraviolet B	Is it good?	Taobao Marketplace, a Chinese website for online shopping
Have a dream home franchise	New Ultraviolet B	Isn't it the same as a rechargeable battery?	Taobao Marketplace, a Chinese website for online shopping
Have a dream home franchise	New Ultraviolet B	Will my battery work without adding sulfuric acid?	Taobao Marketplace, a Chinese website for online shopping
Have a dream home franchise	New Ultraviolet B	I see a lot of people say it doesn't work, who has used it?	Taobao Marketplace, a Chinese website for online shopping
Have a dream home franchise	New Ultraviolet B	My battery car won't charge, will a battery restorer help?	Taobao Marketplace, a Chinese website for online shopping
Have a dream home franchise	New Ultraviolet Battery B	Does this battery restorer really fix batteries? The battery is now running out of juice after a very short run! Can I improve my mileage after using it?	Taobao Marketplace, a Chinese website for online shopping
Have a dream home franchise	New Ultraviolet B	What model to choose60volts20amps	Taobao Marketplace, a Chinese website for online shopping
Have a dream home franchise	New Ultraviolet B	Can you really fix a battery? Can you fix a battery that won't charge anymore?	Taobao Marketplace, a Chinese website for online shopping
Have a dream home franchise	New Ultraviolet B	It doesn't seem to be working.	Taobao Marketplace, a Chinese website for online shopping
Have a dream home franchise	New Ultraviolet B	Almost. I haven't used it yet. It's at home.	Taobao Marketplace, a Chinese website for online shopping
Have a dream home franchise	New Ultraviolet B	can	Taobao Marketplace, a Chinese website for online shopping
Have a dream home franchise	New Ultraviolet B	I don't feel it's very effective.	Taobao Marketplace, a Chinese website for online shopping
Have a dream home franchise	New Ultraviolet B	use sparingly	Taobao Marketplace, a Chinese website for online shopping
Have a dream home franchise	New Ultraviolet B	It works, the battery can't be charged with this one.	Taobao Marketplace, a Chinese website for online shopping
Have a dream home franchise	New Ultraviolet B	Applicableto36_96	Taobao Marketplace, a Chinese website for online shopping
Have a dream home franchise	New Ultraviolet B	Just used it once, not sure about the specifics	Taobao Marketplace, a Chinese website for online shopping
Wangsheng Research Center	Battery Repairer	parked for a long time, the battery is slowly in a state of loss, and it is good to use the charger to fill up the battery regularly. Compared to the price of replacing the battery, this charger is much more cost-effective. It can also repair and desulfurize the battery, and the battery can be used for two more years, which is good!	Taobao Marketplace, a Chinese website for online shopping
Wangsheng Research Center	Battery Repairer	Baby has been received, feel quite strong, hope to improve the battery sooner, long time not ride the battery starved to death, good	Taobao Marketplace, a Chinese website for online shopping
Wangsheng Research Center	Battery Repairer	The repair has been used for two days and the results are already showing, the distance traveled has increased significantly, great, a very good product, will stick with it!	Taobao Marketplace, a Chinese website for online shopping
Wangsheng Research Center	Battery Repairer	Each receipt is very pleasant, first of all, the price is good, the quality is good, the delivery is good, the logistics is quite fast, customer service.	Taobao Marketplace, a Chinese website for online shopping
Wangsheng Research Center	Battery Repairer	The product is good, this charging will be assured, there is a stabilized voltage, not afraid of burning the line, the night charging overnight, and have the function of repairing the battery, very satisfied!	Taobao Marketplace, a Chinese website for online shopping
Wangsheng Research Center	Battery Repairer	Has received, battery loss improvement, power enough, charging is also very fast and stable, very satisfied with the restorer	Taobao Marketplace, a Chinese website for online shopping
Wangsheng Research Center	Battery Repairer	Received, immediately used, packaging intact, logistics is very fast, the instructions say it takes several weeks to see the effect, first use it, see the effect is good, the	Taobao Marketplace, a Chinese website for online shopping
Wangsheng Research Center	Battery Repairer	good product. My batteries are over a year old and feel weak and this charger improves them. Pretty happy with it and will stick with it!	Taobao Marketplace, a Chinese website for online shopping
Wangsheng Research Center	Battery Repairer	Baby received, the quality is very good, recommended to buy, I charge the car's battery, charging a night, the original 9vold battery is also repaired, is now12.7v,the battery all normal	Taobao Marketplace, a Chinese website for online shopping
Wangsheng Research Center	Battery Repairer	is useful, a42Vbattery originally charged with electricity, the first repair is still not charged, the second repair can be almost full.	Taobao Marketplace, a Chinese website for online shopping
Wangsheng Research Center	Battery Repairer	Merchants shipping fast, the battery is full compared to the previous full charge after also ran more than 10 kilometers, very good product!	Taobao Marketplace, a Chinese website for online shopping
Wangsheng Research Center	Battery Repairer	System default comments	Taobao Marketplace, a Chinese website for online shopping

Wangsheng Research Center	Battery Repairer	Shipping is very fast, the baby received after the trial, indeed can effectively repair the old battery, with a long time, it is worth buying!	Taobao Marketplace, a Chinese website for online shopping
Wangsheng Research Center	Battery Repairer	effect is really awesome, I don't realize that the range has increased a lot!	Taobao Marketplace, a Chinese website for online shopping
Wangsheng Research Center	Battery Repairer	Already used it, consistent with the description, works well and is powerful, it does run longer compared to before	Taobao Marketplace, a Chinese website for online shopping
Wangsheng Research Center	Battery Repairer	Product is very satisfied, good quality, easy to use.The electric car played a very good protection, battery repair is more durable than before, logistics is also fast!	Taobao Marketplace, a Chinese website for online shopping
Wangsheng Research Center	Battery Repairer	I keep my trolley plugged in when charging, usually a charge can be used for two days, now a charge can be used for four days, you can run farther and longer, the speed is faster than before, do not need to change the battery.	Taobao Marketplace, a Chinese website for online shopping
Wangsheng Research Center	Battery Repairer	The seller shipped quickly, the baby is of good quality, after using the repair function, I feel that the electric car has strength and runs farther, praise!Purposefully used for two days before coming to the evaluation	Taobao Marketplace, a Chinese website for online shopping
Wangsheng Research Center	Battery Repairer	Repairer compact size, convenient and easy to use, affordable, is what I want, this time do not have to change the battery, to spend hundreds of dollars, is genuine, there are instructions. This time to buy the mind super value.	Taobao Marketplace, a Chinese website for online shopping
Wangsheng Research Center	Battery Repairer	compatible, especially after the power is very beautiful, good quality, simple to use the effect is good, the battery repair is improved, it is worth having, I wish the boss a prosperous business!	Taobao Marketplace, a Chinese website for online shopping
Wangsheng Research Center	Battery Repairer	The car runs longer and farther than before after using it, the battery works, and the logistics are fast!	Taobao Marketplace, a Chinese website for online shopping
Wangsheng Research Center	Battery Repairer	times. The charger is lightweight, simple and practical, simple and easy to use, smart automatic adjustment, perfect!Worth buying	Taobao Marketplace, a Chinese website for online shopping
Wangsheng Research Center	Battery Repairer	Very good and the quality of the product is OK. the item was received for a few days straight without coming to evaluate. I have charged and repaired 3 batteries and the charging is good	Taobao Marketplace, a Chinese website for online shopping
Wangsheng Research Center	Battery Repairer	and motorcycle charging, charging in the garage on the line, it is very convenient does not take up space, but also can be put in the trunk with the car with the car, ready to buy a home for the elderly to use, not complicated, easy to operate, plug in on the line, there are also repair functions, simply too convenient!	Taobao Marketplace, a Chinese website for online shopping
Wangsheng Research Center	Battery Repairer	Very qualitative, electric car put a long time lead to power consumption too fast battery, has not run much far on the power, with this after a very stable, power consumption has been reduced, genuine!	Taobao Marketplace, a Chinese website for online shopping
Wangsheng Research Center	Battery Repairer	Looks very good. The appearance is small and compact. It was a very satisfying purchase. Customer service is very patient. Logistics is also very fast. When the goods were received, I immediately tried it. The charging speed is very fast. It works much better than the charger I bought some years ago.	Taobao Marketplace, a Chinese website for online shopping
Wangsheng Research Center	Battery Repairer	The seller shipped quickly, the baby is very good quality, after using the repair function feels the electric car has strength, praise!used two days before coming to evaluate!	Taobao Marketplace, a Chinese website for online shopping
Wangsheng Research Center	Battery Repairer	Repair period quality is really good, repair electric car battery is really good electric car also run faster and durable without having to replace the battery, worth buying!	Taobao Marketplace, a Chinese website for online shopping
Wangsheng Research Center	Battery Repairer	appearance of good design, looks quite advanced, advanced, more use a few times will be able to see the effect, very good!	Taobao Marketplace, a Chinese website for online shopping
Wangsheng Research Center	Battery Repairer	convenient and easy to use electric car repair device received, logistics is very fast quality is good, listen to the introduction of friends to buy, get can't wait to use the repair effect is quite good, good value for money is really worth buying!	Taobao Marketplace, a Chinese website for online shopping
Wangsheng Research Center	Battery Repairer	The price is not expensive, much cheaper than the jitterbug Good service attitude Cost-effective, really good, but also anti-drumming, automatic power-off, pulse repair, the battery used for a long time, really need this to repair it	Taobao Marketplace, a Chinese website for online shopping
Wangsheng Research Center	Battery Repairer	The product is superb, very good shopping experience, electric car battery repairer received, tried, before my electric car due to power loss, very intolerant to run, with about half a month, obviously feel the battery loss of the repair, than the usual probably more than 20 kilometers, in accordance with the businessman said the longer the use of the time, the better the repair, the mileage will also be increased, the product is very satisfied, it is the genuine article, value for money!	Taobao Marketplace, a Chinese website for online shopping

Wangsheng Research Center	Battery Repairer	second-hand electric car, before the day up to run 6.7hours on the power, now when the charge plugged into this repairer, today ranmore than10hours, and there is still power left a little bit, solved my charging troubles!!!!The product is really great!	Taobao Marketplace, a Chinese website for online shopping
Wangsheng Research Center	Battery Repairer	used to consult customer service, under the guidance of customer service, successfully put into use, as to whether the battery can be repaired, according to customer service, a short time to test not to be2a3weeks to see the effect of the logistics is very fast on Friday afternoon under the order, Sunday on the arrival of customer service attitude is very good to have do not understand the place, very satisfied with five-star praise!	Taobao Marketplace, a Chinese website for online shopping
Wangsheng Research Center	Battery Repairer	Good to use good to use, stick to the use of it, now the battery is completely good, before can not charge at all, save the money to change the battery!	Taobao Marketplace, a Chinese website for online shopping
Wangsheng Research Center	Battery Repairer	Did it work?	Taobao Marketplace, a Chinese website for online shopping
Wangsheng Research Center	Battery Repairer	Does it automatically cut off the power?	Taobao Marketplace, a Chinese website for online shopping
Wangsheng Research Center	Battery Repairer	This battery repair device is still useful, I used it for some days to automatically cut off the power, these are all in line with the function of this product said!	Taobao Marketplace, a Chinese website for online shopping
Wangsheng Research Center	Battery Repairer	I bought this one and have been using it for so many days now, it really works quite well, it can auto power off and the battery life is long lasting, overall it's great.	Taobao Marketplace, a Chinese website for online shopping
Lu Yue Xiang Xiang	Battery Repairer	It's the same whether you use it or not.	Taobao Marketplace, a Chinese website for online shopping
Lu Yue Xiang Xiang	Battery Repairer	False propaganda, simply, where so many positive reviews, there is no chip in it at all, it is a direct socket, ordinary a single-head socket are heavier than it! And what automatic power-off function?When we fool ah! Buy a bull socket are stronger than it. Pay the IQ tax once. Don't be fooled!	Taobao Marketplace, a Chinese website for online shopping
Lu Yue Xiang Xiang	Battery Repairer	Very good quality and value for money	Taobao Marketplace, a Chinese website for online shopping
Lu Yue Xiang Xiang	Battery Repairer	The quality is so bad that charging it doesn't help at all and it won't recover the battery.	Taobao Marketplace, a Chinese website for online shopping
Lu Yue Xiang Xiang	Battery Repairer	I don't know how long it takes to get results	Taobao Marketplace, a Chinese website for online shopping
Lu Yue Xiang Xiang	Battery Repairer	Special to get a power loss for a long time to repair the battery, the beginning of the error, a moment to start repairing, everything is normal, the meter test is also normal, it is really a godsend, the repair effect is good!	Taobao Marketplace, a Chinese website for online shopping
Lu Yue Xiang Xiang	Battery Repairer	Received, immediately used, well-packaged, logistics is very fast, the instructions say that it takes weeks to see the effect, first use it, see the effect is good, will buy back!	Taobao Marketplace, a Chinese website for online shopping
Lu Yue Xiang Xiang	Battery Repairer	second-hand electric car, before the day up to run 6.7hours on the power, now when the charge plugged into this repairer, today ranmore than10hours, and there is still power left a little bit, solved my charging troubles! The product is really great!	Taobao Marketplace, a Chinese website for online shopping
Lu Yue Xiang Xiang	Battery Repairer	used to consult customer service, under the guidance of customer service, successfully put into use, as for whether the battery can be repaired, according to customer service, a short period of time to test not to be2a3weeks to see the effect of the logistics is very fast on Friday afternoon under the order, Sunday on the arrival of customer service attitude is very good to have do not understand the place, is very satisfied with five-star praise!	Taobao Marketplace, a Chinese website for online shopping
Lu Yue Xiang Xiang	Battery Repairer	good product. My batteries are over a year old and feel weak and this charger improves them. Pretty happy with it and will stick with it!	Taobao Marketplace, a Chinese website for online shopping
Lu Yue Xiang Xiang	Battery Repairer	electric car battery repairer received, tried, before my electric car due to power loss, very intolerant to run, with about half a month, obviously feel the battery loss of the repair, than the usual probably more than 20kilometers, in accordance with the businessman said the longer the use of the time, the better the repair, the mileage will also be increased, the product is very satisfied, it is genuine, value for money!	Taobao Marketplace, a Chinese website for online shopping
Lu Yue Xiang Xiang	Battery Repairer	Tram repair range extender received, the effect is the same as expected, did not let me disappointed, very good, than the usual also have the strength, the effect is too good, good.	Taobao Marketplace, a Chinese website for online shopping
Lu Yue Xiang Xiang	Battery Repairer	after the repair is obviously farther than before, good five-star praise!	Taobao Marketplace, a Chinese website for online shopping

Lu Yue Xiang Xiang	Battery Repairer	battery has failed, each time the charge has to be a long time, and can not run far, and now bought this repair fast-charging conversion socket, obviously feel the electric car much better than before charging fast, but also run far, is indeed a good thing to recommend!	Taobao Marketplace, a Chinese website for online shopping
Lu Yue Xiang Xiang	Battery Repairer	Is it lithium or lead acid?	Taobao Marketplace, a Chinese website for online shopping
Lu Yue Xiang Xiang	Battery Repairer	leaded	Taobao Marketplace, a Chinese website for online shopping
Lu Yue Xiang Xiang	Battery Reconditioner	phone is still heating up, nothing has changed, what is the reason.	Taobao Marketplace, a Chinese website for online shopping
Lu Yue Xiang Xiang	Battery Reconditioner	With the role, do not enter!	Taobao Marketplace, a Chinese website for online shopping
Lu Yue Xiang Xiang	Battery Reconditioner	Repair device received, complete accessories, customer service is very patient to teach me how to use, after repairing the effect is very good, full of electricity resistance is not very hot, it is worth buying!	Taobao Marketplace, a Chinese website for online shopping
Lu Yue Xiang Xiang	Battery Reconditioner	Got it for half a month, it works very well. Appearance design is novel and generous, used to repair Huawei cell phone, so that the power has been effectively restored, good stuff.	Taobao Marketplace, a Chinese website for online shopping
Lu Yue Xiang Xiang	Battery Reconditioner	Received the baby has been a few days, specially tried a few days, feel or a little effect, before the day to charge twice, now charge a power basically can use a day , good	Taobao Marketplace, a Chinese website for online shopping
Lu Yue Xiang Xiang	Battery Reconditioner	battery life of the time to grow a little, repeated a number of times will certainly have better results, has been recommended to friends!	Taobao Marketplace, a Chinese website for online shopping
Jiu'an Health Hall	Battery Repairer	Comments	Taobao Marketplace, a Chinese website for online shopping
Jiu'an Health Hall	Battery Repairer	After using the repair effect is very good, very satisfied, worth recommending!	Taobao Marketplace, a Chinese website for online shopping
Jiu'an Health Hall	Battery Repairer	The product packaging is exquisite and beautiful atmosphere, currently used very good, real evaluation, I hope to help the set of beauty!	Taobao Marketplace, a Chinese website for online shopping
Jiu'an Health Hall	Battery Repairer	Goods received very good quality very like	Taobao Marketplace, a Chinese website for online shopping
Jiu'an Health Hall	Battery Repairer	times. The charger is lightweight, simple and practical, simple and easy to use, smart automatic adjustment, perfect! Worth buying	Taobao Marketplace, a Chinese website for online shopping
Jiu'an Health Hall	Battery Repairer	Tried, pulse repair effect is very good, the battery was not used for a month power loss, start charging is not normal, repair a few hours of normal charging charged a few hours, full blood resurrection, trustworthy!	Taobao Marketplace, a Chinese website for online shopping
Shunfeng Shunshuiyuan Merchandise Store	Air Conditioner Intelligent Inverter P	store closure	Taobao Marketplace, a Chinese website for online shopping
Jitterbug Specialty Shop	Air Conditioner Intelligent Inverter P	(of an unmarried couple) be close	Taobao Marketplace, a Chinese website for online shopping
Jitterbug Specialty Shop	Air Conditioner Intelligent Inverter P	plate	Taobao Marketplace, a Chinese website for online shopping
Jitterbug Specialty Shop	Air Conditioner Intelligent Inverter P	This is very effective in computers, air conditioners... electric fans... coils with inductors are effective.	Taobao Marketplace, a Chinese website for online shopping
Jitterbug Specialty Shop	Air Conditioner Intelligent Inverter P	The jack doesn't fit. It won't go in.	Taobao Marketplace, a Chinese website for online shopping
Jitterbug Specialty Shop	Air Conditioner Intelligent Inverter P	Swiped from Jitterbug I hope it's really power efficient	Taobao Marketplace, a Chinese website for online shopping
Jitterbug Specialty Shop	Air Conditioner Intelligent Inverter P	I hope it's not an IQ tax.	Taobao Marketplace, a Chinese website for online shopping
Jitterbug Specialty Shop	Air Conditioner Intelligent Inverter P	general	Taobao Marketplace, a Chinese website for online shopping
Jitterbug Specialty Shop	Air Conditioner Intelligent Inverter P	general	Taobao Marketplace, a Chinese website for online shopping

Jitterbug Specialty Shop	Air Conditioner Intelligent Inverter	Don't know the exact effect yet, how to comment	Taobao Marketplace, a Chinese website for online shopping
Jitterbug Specialty Shop	Air Conditioner Intelligent Inverter	Logistics is fast, the use of the function to be observed!	Taobao Marketplace, a Chinese website for online shopping
Jitterbug Specialty Shop	Air Conditioner Intelligent Inverter	save electricity not careful will also have electrical hazards three holes a no grounding wire grounding wire is mainly to prevent electrocution15buy return10bucks shipping really boltedQ	Taobao Marketplace, a Chinese website for online shopping
Jitterbug Specialty Shop	Air Conditioner Intelligent Inverter	I don't know if it will save electricity, it's already being used.	Taobao Marketplace, a Chinese website for online shopping
Jitterbug Specialty Shop	Air Conditioner Intelligent Inverter	I'll comment after using it.	Taobao Marketplace, a Chinese website for online shopping
Jitterbug Specialty Shop	Air Conditioner Intelligent Inverter	Buyer does not comment within15days	Taobao Marketplace, a Chinese website for online shopping
Jitterbug Specialty Shop	Air Conditioner Intelligent Inverter	You this product is completely fraudulent, a saving what electricity, inside is a circuit, except nothing, this time I have been deceived.	Taobao Marketplace, a Chinese website for online shopping
Jitterbug Specialty Shop	Air Conditioner Intelligent Inverter	Can you save electricity?	Taobao Marketplace, a Chinese website for online shopping
Jitterbug Specialty Shop	Air Conditioner Intelligent Inverter	Does an air conditioner energy saver save electricity	Taobao Marketplace, a Chinese website for online shopping
Jitterbug Specialty Shop	Air Conditioner Intelligent Inverter	Is this good?	Taobao Marketplace, a Chinese website for online shopping
Jitterbug Specialty Shop	Air Conditioner Intelligent Inverter	We don't know yet.	Taobao Marketplace, a Chinese website for online shopping
Jitterbug Specialty Shop	Air Conditioner Intelligent Inverter	I didn't pay attention to the meter either. I won't know until next month.	Taobao Marketplace, a Chinese website for online shopping
Jitterbug Specialty Shop	Air Conditioner Intelligent Inverter	I didn't pay attention to the meter either. I won't know until next month.	Taobao Marketplace, a Chinese website for online shopping
Jitterbug Specialty Shop	Battery Repairer	Good review!	Taobao Marketplace, a Chinese website for online shopping
Jitterbug Specialty Shop	Battery Repairer	(of an unmarried couple) be close	Taobao Marketplace, a Chinese website for online shopping
Jitterbug Specialty Shop	Battery Repairer	(of an unmarried couple) be close	Taobao Marketplace, a Chinese website for online shopping
Jitterbug Specialty Shop	Battery Repairer	Great customer service, very patient with my questions, great product quality. The product labeling is grossly incorrect!s actuallyDC12--96V(DC) Labeled for AC	Taobao Marketplace, a Chinese website for online shopping
Jitterbug Specialty Shop	Battery Repairer	It's very effective.	Taobao Marketplace, a Chinese website for online shopping
Jitterbug Specialty Shop	Battery Repairer	Pretty good, battery life jumped	Taobao Marketplace, a Chinese website for online shopping
Jitterbug Specialty Shop	Battery Repairer	It is not used yet, and will be further evaluated after it is used. Merchants shipping fast, logistics is also very fast.	Taobao Marketplace, a Chinese website for online shopping
Jitterbug Specialty Shop	Battery Repairer	Can I use96V	Taobao Marketplace, a Chinese website for online shopping
Jitterbug Specialty Shop	Battery Repairer	work for allVdegree sizes?	Taobao Marketplace, a Chinese website for online shopping
Jitterbug Specialty Shop	Battery Repairer	Is it an IQ tax?	Taobao Marketplace, a Chinese website for online shopping
Jitterbug Specialty Shop	Battery Repairer	Maximum is72volts	Taobao Marketplace, a Chinese website for online shopping
Jitterbug Specialty Shop	Battery Repairer	The fix isn't as noticeable as it could be, I'd recommend replacing the battery, the	Taobao Marketplace, a Chinese website for online shopping
Jitterbug Specialty Shop	Battery Repairer	It's working a bit, charging is slower now	Taobao Marketplace, a Chinese website for online shopping

Dali Flagship Store	Battery Reconditioner	Very good quality, very satisfied with the purchase. Customer service is very professional with questions and answers	Taobao Marketplace, a Chinese website for online shopping
Dali Flagship Store	Battery Reconditioner	Very well packaged, good value and no problems with quality	Taobao Marketplace, a Chinese website for online shopping
Dali Flagship Store	Battery Reconditioner	Experience: nice and effective	Taobao Marketplace, a Chinese website for online shopping
Dali Flagship Store	Battery Reconditioner	too many background programs, lagging, heat, battery loss fast, to people annoyed, not moving the phone on the power shutdown, the use of cell phone battery repairer, simple to connect the charging plug and charging cable, a charging immediately on the cell phone battery repair, can extend the service life, optimize the cell phone system, the price is not only cheap, but also easy to use, businessmen fast delivery, logistics and distribution in a timely manner, and the battery repairer can be used to repair the battery. Powerful.	Taobao Marketplace, a Chinese website for online shopping
Dali Flagship Store	Battery Reconditioner	Cell phone battery repairer after hours of charging and on a cell phone that can not be charged into the repair, a few hours to repair the success, kudos! Commodity description is consistent, logistics is so fast, worth buying!	Taobao Marketplace, a Chinese website for online shopping
Dali Flagship Store	Battery Reconditioner	Cell phone repairer received, the quality is very good, received home on the use, really a good product, everyone rest assured to buy it! Give merchants point a big praise	Taobao Marketplace, a Chinese website for online shopping
Dali Flagship Store	Battery Reconditioner	The use of the effect is quite good, after repairing the battery obviously more power, very satisfied.	Taobao Marketplace, a Chinese website for online shopping
Dali Flagship Store	Battery Reconditioner	Very good quality, very satisfied with the purchase. Customer service is very professional with questions and answers	Taobao Marketplace, a Chinese website for online shopping
Dali Flagship Store	Air Conditioner Intelligent Inverter	I just tried it, and the refrigerator looks good when plugged into the meter, and the meter doesn't jump as much as it used to.	Taobao Marketplace, a Chinese website for online shopping
Dali Flagship Store	Air Conditioner Intelligent Inverter	The power saving effect is very good, consistent with the description, worth buying	Taobao Marketplace, a Chinese website for online shopping
Dali Flagship Store	Air Conditioner Intelligent Inverter	It's a little useful.	Taobao Marketplace, a Chinese website for online shopping
Dali Flagship Store	Air Conditioner Intelligent Inverter	garbage	Taobao Marketplace, a Chinese website for online shopping
Dali Flagship Store	Air Conditioner Intelligent Inverter	Great value for money, very affordable, feels great to use, perfect.^◡^.	Taobao Marketplace, a Chinese website for online shopping
Dali Flagship Store	Air Conditioner Intelligent Inverter	It's a scam, it doesn't work at all, don't fall for it	Taobao Marketplace, a Chinese website for online shopping
Dali Flagship Store	Air Conditioner Intelligent Inverter	Other people use it at home, the power saving effect is good, I also buy a try, used a few days the effect is good!	Taobao Marketplace, a Chinese website for online shopping
Dali Flagship Store	Air Conditioner Intelligent Inverter	It didn't work!	Taobao Marketplace, a Chinese website for online shopping
Dali Flagship Store	Air Conditioner Intelligent Inverter	Power-saving effect: poor appearance style: poor workmanship material: poor is a fake, fooled, buy back plugged into the original power does not power, and toys, the same, useless , , , ,	Taobao Marketplace, a Chinese website for online shopping
Dali Flagship Store	Air Conditioner Intelligent Inverter	inserted in the house, look quite upscale, exquisite workmanship, affordable, get up in the morning to see indeed save a lot of, will insist on the use of the good will continue to repurchase!	Taobao Marketplace, a Chinese website for online shopping
Dali Flagship Store	Air Conditioner Intelligent Inverter	Received, well packaged, not yet used, looking forward to the effect	Taobao Marketplace, a Chinese website for online shopping
Dali Flagship Store	Air Conditioner Intelligent Inverter	I hope you don't information this product, it's purely a scam, bull*blowing* all over the place. False products.	Taobao Marketplace, a Chinese website for online shopping
Dali Flagship Store	Air Conditioner Intelligent Inverter	liars	Taobao Marketplace, a Chinese website for online shopping

Dali Flagship Store	Air Conditioner Intelligent Inverter R[illegible]	Power saving effect: no feeling	Taobao Marketplace, a Chinese website for online shopping
Dali Flagship Store	Air Conditioner Intelligent Inverter R[illegible]	Power saving effect: okay Price/performance ratio: very high Using experience: very good	Taobao Marketplace, a Chinese website for online shopping
Dali Flagship Store	Air Conditioner Intelligent Inverter R[illegible]	I've been using it since I got back, I forgot to give a good review, but today I remembered to give a good review, it's still pretty good, I really saved a lot of money in the summer with this.	Taobao Marketplace, a Chinese website for online shopping
Dali Flagship Store	Air Conditioner Intelligent Inverter Repairer	Power saving effect: disposable, simply too late to see the power saving or not Workmanship material: super garbage, used once broken, but also burned my home jack Use experience: disposable experience, but also backward a mopping plate	Taobao Marketplace, a Chinese website for online shopping
Dali Flagship Store	Air Conditioner Intelligent Inverter R[illegible]	A very trashy product, belonging to the disposable kind of it, but also reverse compensation, bought back and used a burned out, but also burned my jack can not be used, separate and buy the towing plate	Taobao Marketplace, a Chinese website for online shopping
Dali Flagship Store	Air Conditioner Intelligent Inverter R[illegible]	I'm using it, I don't know how well it works yet, I'll comment after I use it.	Taobao Marketplace, a Chinese website for online shopping
Dali Flagship Store	Air Conditioner Intelligent Inverter R[illegible]	received	Taobao Marketplace, a Chinese website for online shopping
Dali Flagship Store	Air Conditioner Intelligent Inverter Repairer	I was in the same store to buy two yellow duck air conditioning adapter received the goods first give all five-star praise, but today used to plug in the panel use found out no power unplugged3feet concave two feet quality is too bad bad store, or air conditioning adapter quality is too bad?	Taobao Marketplace, a Chinese website for online shopping
Dali Flagship Store	Air Conditioner Intelligent Inverter R[illegible]	Terrible quality, bad store.	Taobao Marketplace, a Chinese website for online shopping
Dali Flagship Store	Air Conditioner Intelligent Inverter R[illegible]	Power saving effect: fake does not work, can not save power. Used a few days in the evaluation, this product does not save electricity.	Taobao Marketplace, a Chinese website for online shopping
Dali Flagship Store	Air Conditioner Intelligent Inverter R[illegible]	Experience: this product can not save electricity, installed on the use of a few days a same, fake not save electricity	Taobao Marketplace, a Chinese website for online shopping
Dali Flagship Store	Air Conditioner Intelligent Inverter R[illegible]	Garbage products, after trying, a little power-saving effect is not, in fact, is a socket only, cheating customers, selling at high prices, bad business, illegal business!	Taobao Marketplace, a Chinese website for online shopping
Dali Flagship Store	Air Conditioner Intelligent Inverter R[illegible]	I've been using it for a while now and it feels good, watching the meter really saves electricity, it works great!	Taobao Marketplace, a Chinese website for online shopping
Dali Flagship Store	Air Conditioner Intelligent Inverter Repairer	actual test is effective!My home every day to open their own air conditioning, almost a month to use more than500dollars of electricity, and now use this after more than400will be enough, although I do not understand what the reasoning, but it is true and then give me money to save money to save money on electricity, very!Very good power-saving effect: good cost-effective: good appearance and style: good workmanship materials: good experience: good	Taobao Marketplace, a Chinese website for online shopping
Dali Flagship Store	Air Conditioner Intelligent Inverter R[illegible]	Recommended by a friend who said it was very good and practical	Taobao Marketplace, a Chinese website for online shopping
Dali Flagship Store	Air Conditioner Intelligent Inverter R[illegible]	Quality is quite good, the next time you need to come to your store to buy, this is a very good shopping, my friend asked me where to buy, I said in your store to buy, to the time he came to your store to buy to give a five-star!	Taobao Marketplace, a Chinese website for online shopping
Dali Flagship Store	Air Conditioner Intelligent Inverter R[illegible]	Exceptional value very well made and good value for money	Taobao Marketplace, a Chinese website for online shopping
Dali Flagship Store	Air Conditioner Intelligent Inverter R[illegible]	inserted in the house, look quite upscale, exquisite workmanship, affordable, get up in the morning to see indeed save a lot of, will insist on the use of the good will continue to repurchase!	Taobao Marketplace, a Chinese website for online shopping

Dali Flagship Store	Air Conditioner Intelligent Inverter [illegible]	Summer air conditioning consumes store electricity, specially bought, that is, plug and play, optimize the power consumption, the effect is very good	Taobao Marketplace, a Chinese website for online shopping
Dali Flagship Store	Air Conditioner Intelligent Inverter [illegible]	Back to use the effect is good, especially good satisfaction worth buying	Taobao Marketplace, a Chinese website for online shopping
Dali Flagship Store	Air Conditioner Intelligent Inverter Repairer	In-kind and the same as described, the quality is quite good, summer air conditioning power consumption, with this power saving and stable voltage on the appliance is also better, the seller's service attitude is good, ask questions and answer them, the delivery speed of the bar	Taobao Marketplace, a Chinese website for online shopping
Dali Flagship Store	Air Conditioner Intelligent Inverter Repairer	the power, and it does save compared to when I didn't use this adapter before. Can be seen. The air conditioner is used a lot in summer, you should still have more at home, and the refrigerator is also used. It's great to have a proper power saving.	Taobao Marketplace, a Chinese website for online shopping
Dali Flagship Store	Air Conditioner Intelligent Inverter Repairer	In-kind and the same as described, the quality is quite good, summer air conditioning power consumption, with this power saving and stable voltage on the appliance is also better, the seller's service attitude is good, ask questions and answer them, the delivery speed of the bar	Taobao Marketplace, a Chinese website for online shopping
Dali Flagship Store	Air Conditioner Intelligent Inverter Repairer	of very simple, plug into the socket on the summer air conditioning power is too large, electricity is also expensive, with this product directly on the electricity bill to save half, has been purchased a few more, the effect is too awesome!	Taobao Marketplace, a Chinese website for online shopping
Dali Flagship Store	Air Conditioner Intelligent Inverter [illegible]	Small, convenient and power saving, great value for money!👍👍	Taobao Marketplace, a Chinese website for online shopping
Dali Flagship Store	Air Conditioner Intelligent Inverter Repairer	very good, very satisfied, with this air conditioner saves much more electricity, and the summer use can also stabilize the voltage. That is, it is plugged in, very convenient, big brand. Very safe, rest assured with, very good, save a lot of money. Very satisfied	Taobao Marketplace, a Chinese website for online shopping
Dali Flagship Store	Air Conditioner Intelligent Inverter [illegible]	How many kilowatts a night does this adapter save?	Taobao Marketplace, a Chinese website for online shopping
Dali Flagship Store	Air Conditioner Intelligent Inverter [illegible]	Does it really work? Is it safe?	Taobao Marketplace, a Chinese website for online shopping
Dali Flagship Store	Air Conditioner Intelligent Inverter [illegible]	Ineffective safety, doesn't save power, effectively just an outlet	Taobao Marketplace, a Chinese website for online shopping
Dali Flagship Store	Air Conditioner Intelligent Inverter [illegible]	Invalid security, actually just a podcast seat	Taobao Marketplace, a Chinese website for online shopping
Huiren Science and Technology Center	Air Conditioner Intelligent Inverter Repairer	up, I do not know did not light up to play a role? However, there is no reduction in the electricity bill, according to the original payment time comparison, basically it is about the same time as the original payment time, no reduction in the electricity bill, so it is not effective and can only be used as a plug-in board.	Taobao Marketplace, a Chinese website for online shopping
Huiren Science and Technology Center	Air Conditioner Intelligent Inverter [illegible]	The quality of the product is very good, the customer service is very good, and the reputation of the business is very good!	Taobao Marketplace, a Chinese website for online shopping
Huiren Science and Technology Center	Air Conditioner Intelligent Inverter [illegible]	Received on the use of the goods, the use of these days the power is indeed much less than before, good quality, the product and the expression of the same, the home must be recommended!	Taobao Marketplace, a Chinese website for online shopping
Huiren Science and Technology Center	Air Conditioner Intelligent Inverter [illegible]	Fast shipping, good quality, really works, very satisfied, love it, thanks!	Taobao Marketplace, a Chinese website for online shopping
Huiren Science and Technology Center	Air Conditioner Intelligent Inverter [illegible]	Value for money, great store service and fast shipping, genuine.	Taobao Marketplace, a Chinese website for online shopping
Huiren Science and Technology Center	Air Conditioner Intelligent Inverter [illegible]	Baby favorite for a long time, take advantage of the activities when to buy really too cost-effective, indeed more power than before, there is a need for friends do not hesitate ah, good value for money!	Taobao Marketplace, a Chinese website for online shopping

Huiren Science and Technology Center	Air Conditioner Intelligent Inverter [illegible]	Class1lowenergy/:085/:085/:0, has anyone taken	Taobao Marketplace, a Chinese website for online shopping
Huiren Science and Technology Center	Air Conditioner Intelligent Inverter [illegible]	have nothing	Taobao Marketplace, a Chinese website for online shopping
Huiren Science and Technology Center	Air Conditioner Intelligent Inverter [illegible]	not have	Taobao Marketplace, a Chinese website for online shopping
Huiren Science and Technology Center	Air Conditioner Intelligent Inverter [illegible]	favorable criticism	Taobao Marketplace, a Chinese website for online shopping
Huiren Science and Technology Center	Air Conditioner Intelligent Inverter [illegible]	Summer air conditioning consumes store electricity, specially bought, that is, plug and play, optimize the power consumption, the effect is very good	Taobao Marketplace, a Chinese website for online shopping
Huiren Science and Technology Center	Air Conditioner Intelligent Inverter [illegible]	Used almost a month, can see the obvious effect, is genuine, very good	Taobao Marketplace, a Chinese website for online shopping
Huiren Science and Technology Center	Air Conditioner Intelligent Inverter [illegible]	Bought back on the use, now the electricity bill once to more than 50 cents, with this power saver a month to save a hundred dollars is also very good!	Taobao Marketplace, a Chinese website for online shopping
Huiren Science and Technology Center	Air Conditioner Intelligent Inverter [illegible]	Received the goods is very ideal Indeed very power saving Really buy the right thing Good review	Taobao Marketplace, a Chinese website for online shopping
Huiren Science and Technology Center	Air Conditioner Intelligent Inverter [illegible]	Fast shipping, good service attitude, back to install and use the effect is good, especially good!	Taobao Marketplace, a Chinese website for online shopping
Huiren Science and Technology Center	Air Conditioner Intelligent Inverter [illegible]	Baby received, root business description of the same, the quality is very good price is also very affordable, very practical and very good, logistics is also very fast!	Taobao Marketplace, a Chinese website for online shopping
Huiren Science and Technology Center	Air Conditioner Intelligent Inverter [illegible]	Received on the use of the goods, the use of these days the power is indeed much less than before, good quality, the product and the expression of the same, the home must be recommended!	Taobao Marketplace, a Chinese website for online shopping
Huiren Science and Technology Center	Air Conditioner Intelligent Inverter [illegible]	works great It saves power when you use Unintentionally bought it It saves a lot of money on the electric bill It makes us happy It looks great too It's worth the buy!	Taobao Marketplace, a Chinese website for online shopping
Huiren Science and Technology Center	Air Conditioner Intelligent Inverter [illegible]	After receiving the goods, plug in the socket, feel the socket plug is quite good, also quite good plug, shell look beautiful, in short, quite satisfied	Taobao Marketplace, a Chinese website for online shopping
Huiren Science and Technology Center	Battery Reconditioner	Battery Recovery Receiver received, the goods are real, repair effect is good, the phone battery becomes strong!	Taobao Marketplace, a Chinese website for online shopping
Huiren Science and Technology Center	Battery Reconditioner	Long battery life Adaptation level: very perfect	Taobao Marketplace, a Chinese website for online shopping
Huiren Science and Technology Center	Battery Reconditioner	only fast logistics, but also good customer service, the most critical is the product quality satisfaction. The phone is not hot	Taobao Marketplace, a Chinese website for online shopping
Huiren Science and Technology Center	Battery Reconditioner	good, the battery use time increased significantly, in accordance with the instructions required to repair the effect of bar, without interference, the effect can reach more than ninety percent!	Taobao Marketplace, a Chinese website for online shopping
Huiren Science and Technology Center	Battery Reconditioner	Heat: I have an old cell phone, charging hot, with our repairer is not hot, the family has an old receiver and charging hot, slow to enter the power consider getting!	Taobao Marketplace, a Chinese website for online shopping
Huiren Science and Technology Center	Battery Reconditioner	only fast logistics, but also good customer service, the most critical is the product quality satisfaction. The phone is not hot	Taobao Marketplace, a Chinese website for online shopping
Huiren Science and Technology Center	Battery Reconditioner	It's as described.	Taobao Marketplace, a Chinese website for online shopping
Huiren Science and Technology Center	Battery Reconditioner	Too fake!	Taobao Marketplace, a Chinese website for online shopping

Huiren Science and Technology Center	Battery Reconditioner	It's in use and I don't know how to see the results. Good review first, will follow up in the future.	Taobao Marketplace, a Chinese website for online shopping
Huiren Science and Technology Center	Battery Reconditioner	Good for you.	Taobao Marketplace, a Chinese website for online shopping
Huiren Science and Technology Center	Battery Reconditioner	Good.	Taobao Marketplace, a Chinese website for online shopping
Huiren Science and Technology Center	Battery Reconditioner	Fake, there are only3LEDsin it, don't buy it	Taobao Marketplace, a Chinese website for online shopping
Huiren Science and Technology Center	Battery Reconditioner	The product is good and we will see how it works after using it.	Taobao Marketplace, a Chinese website for online shopping
Huiren Science and Technology Center	Battery Reconditioner	The stuff has been received and feels ok. Store service is very good! Logistics is very fast! Good reviews.	Taobao Marketplace, a Chinese website for online shopping
Huiren Science and Technology Center	Battery Reconditioner	intact. Used a few days to feel repaired, delayed cell phone use time function. I have used two other kinds before, the effect is not as good as this one. In short, it is worth buying	Taobao Marketplace, a Chinese website for online shopping
Huiren Science and Technology Center	Battery Reconditioner	Cell phone repairer received, the quality is very good, received home on the use, really a good product, everyone rest assured to buy it! Give merchants point a big praise	Taobao Marketplace, a Chinese website for online shopping
Huiren Science and Technology Center	Battery Reconditioner	punch, the quality of the product is good, can repair the cell phone battery and cell phone diffuse card quality is good satisfaction👍	Taobao Marketplace, a Chinese website for online shopping
Huiren Science and Technology Center	Battery Reconditioner	There is a liquid crystal display, An customer service guide to insert the charging head connected to the data line can be slow charging fast power consumption or charging into the power of the cell phone to repair, the effect is really good!	Taobao Marketplace, a Chinese website for online shopping
Huiren Science and Technology Center	Battery Reconditioner	Really of have effect results, I thought it was hopeless, myOPPOis now barring the mindset of trying to buy a back, in accordance with the tutorials to do, but also really have a role, the battery durability doubled, the really of is too plan	Taobao Marketplace, a Chinese website for online shopping
Huiren Science and Technology Center	Battery Reconditioner	It works well, the phone won't be so hot even if you use it for a long time, it's good and convenient!	Taobao Marketplace, a Chinese website for online shopping
Huiren Science and Technology Center	Battery Reconditioner	Product quality is very good, next time I have to buy	Taobao Marketplace, a Chinese website for online shopping
Huiren Science and Technology Center	Battery Reconditioner	with other chargers charging is very slow, go to the customer service repair people also said that the model is too aging, need to change the battery, I bought this slowly repair, now very durable	Taobao Marketplace, a Chinese website for online shopping
Huiren Science and Technology Center	Battery Reconditioner	Just used once, the next day the cell phone battery durable a lot. From the appearance and workmanship is very satisfied, and the business description is consistent with the customer service attitude is very good, the account is very clear, after the use of charging armor effect is good, speed is also very fast, the quality is very good, this does not have to change the battery, thanks to the business!	Taobao Marketplace, a Chinese website for online shopping
Huiren Science and Technology Center	Battery Reconditioner	Baby received, it works great the battery does last a lot longer, kudos.	Taobao Marketplace, a Chinese website for online shopping
Huiren Science and Technology Center	Battery Reconditioner	most important thing is to have the effect, the battery is more durable than before, very satisfied with this effect, praise.	Taobao Marketplace, a Chinese website for online shopping
Huiren Science and Technology Center	Battery Reconditioner	Just used once, the next day the cell phone battery is much more durable. From the appearance and workmanship is very satisfied, and the business description is consistent with the customer service attitude is very good, the account is very clear, the use of the charging armor effect is good, the speed is also very fast, the quality is very good, this time do not have to change the battery, thanks to the business.	Taobao Marketplace, a Chinese website for online shopping
Huiren Science and Technology Center	Battery Reconditioner	Did it work?	Taobao Marketplace, a Chinese website for online shopping
Huiren Science and Technology Center	Battery Reconditioner	Does it really work? Is the phone resistant to electricity after charging?	Taobao Marketplace, a Chinese website for online shopping
Huiren Science and Technology Center	Battery Reconditioner	Paid the IQ tax.	Taobao Marketplace, a Chinese website for online shopping
Huiren Science and Technology Center	Battery Reconditioner	of no use	Taobao Marketplace, a Chinese website for online shopping

Huiren Science and Technology Center	Battery Repairer	times. The charger is lightweight, simple and practical, simple and easy to use, smart and automatic adjustment. It's perfect!	Taobao Marketplace, a Chinese website for online shopping
Huiren Science and Technology Center	Battery Repairer	dissatisfied	Taobao Marketplace, a Chinese website for online shopping
Huiren Science and Technology Center	Battery Repairer	originally the battery charge is not enough, starved for too long, directly starved to death, now has gradually improved, great!	Taobao Marketplace, a Chinese website for online shopping
Huiren Science and Technology Center	Battery Repairer	Now the battery is no longer working, every time you charge a long time, and can not run far, now buy this repair fast-charging conversion socket, obviously feel much better than the electric car, faster than before charging, but also run far, is indeed a good thing, recommend!	Taobao Marketplace, a Chinese website for online shopping
Huiren Science and Technology Center	Battery Repairer	Tram repair range extender received, the effect is the same as expected, did not let me disappointed, very good, than the usual also have the strength, the effect is too good, good.	Taobao Marketplace, a Chinese website for online shopping
Huiren Science and Technology Center	Battery Repairer	The smart charger has a beautiful appearance, fine workmanship, the seller's description is consistent, the logistics is very fast, satisfied!	Taobao Marketplace, a Chinese website for online shopping
Huiren Science and Technology Center	Battery Repairer	entity store affordable, their own home can repair the battery, good value for money, the need for friends can rest assured that the order!	Taobao Marketplace, a Chinese website for online shopping
Huiren Science and Technology Center	Battery Repairer	Very satisfied, good quality, easy to use.The electric car played a very good protection, the battery was repaired more durable than before, the service attitude is very good, logistics is also very fast. Buy again also to your store.	Taobao Marketplace, a Chinese website for online shopping
Huiren Science and Technology Center	Battery Repairer	used to consult customer service, under the guidance of customer service, successfully put into use, as to whether the battery can be repaired, according to customer service, a short period of time to test not to be2a3weeks to see the effect	Taobao Marketplace, a Chinese website for online shopping
Huiren Science and Technology Center	Battery Repairer	lasts two days, now a charge lasts four days, I can run farther , and faster than before, I don't need to change the battery anymore	Taobao Marketplace, a Chinese website for online shopping
Huiren Science and Technology Center	Battery Repairer	Quality is very over the top, quality is guaranteed, quite a good choice, a lot of friends to see me use all want to buy	Taobao Marketplace, a Chinese website for online shopping
Huiren Science and Technology Center	Battery Repairer	The use of very simple, my family is an electric car, charging is not very good, quite good, do not have to change the battery, this effect is good, and easy to use	Taobao Marketplace, a Chinese website for online shopping
Huiren Science and Technology Center	Battery Repairer	Electric car repairer received in the afternoon, well-packed, open the package immediately began to use, looks good quality, and charging effect is also good, repair should not be bad, useful will buy again to send someone!	Taobao Marketplace, a Chinese website for online shopping
Huiren Science and Technology Center	Battery Repairer	the car is not often used, resulting in a loss of battery power, use this to regularly charge, after using it, then follow up reviews	Taobao Marketplace, a Chinese website for online shopping
Huiren Science and Technology Center	Battery Repairer	Charger quality is very good, can be completely full of car batteries, the courier speed is amazing three days to hand, the product price is very competitive, you can start!	Taobao Marketplace, a Chinese website for online shopping
Huiren Science and Technology Center	Battery Repairer	The product is quite satisfactory, good quality, easy to use.The electric car is well protected, the battery repair is more durable than before, and the logistics is fast!	Taobao Marketplace, a Chinese website for online shopping
Huiren Science and Technology Center	Battery Repairer	Very qualitative, electric car put a long time lead to power consumption too fast battery, has not run much far on the power, with this after a very stable, power consumption has been reduced, genuine!	Taobao Marketplace, a Chinese website for online shopping
Huiren Science and Technology Center	Battery Repairer	The repair has been used for two days and the results are already showing, the distance traveled has increased significantly, great, a very good product, will stick with it!	Taobao Marketplace, a Chinese website for online shopping
Huiren Science and Technology Center	Battery Repairer	The seller shipped quickly, the baby is very good quality, after using the repair function feels the electric car has strength, praise!used two days before coming to evaluate!	Taobao Marketplace, a Chinese website for online shopping
Huiren Science and Technology Center	Battery Repairer	The sockets are really good, safe and have range boosting ability	Taobao Marketplace, a Chinese website for online shopping
Huiren Science and Technology Center	Battery Repairer	Shipped quickly, baby received after the trial, indeed can effectively repair the old battery, with a long time, it is worth buying .	Taobao Marketplace, a Chinese website for online shopping
altt flagship store	Battery Reconditioner	Very mediocre results	Taobao Marketplace, a Chinese website for online shopping
altt flagship store	Battery Reconditioner	delivery, customer service attitude is very good, a good shopping	Taobao Marketplace, a Chinese website for online shopping
altt flagship store	Battery Reconditioner	Fast enough delivery, good enough quality, great service, 5 stars!	Taobao Marketplace, a Chinese website for online shopping
altt flagship store	Battery Reconditioner	The effect is really good	Taobao Marketplace, a Chinese website for online shopping

altt flagship store	Battery Reconditioner	Very practical.	Taobao Marketplace, a Chinese website for online shopping
altt flagship store	Battery Reconditioner	results yet. Hopefully the fix will be as good as it looks after a few weeks. Habitually favorable comments! _x005f_x0001_ Good review!	Taobao Marketplace, a Chinese website for online shopping
altt flagship store	Battery Reconditioner	Fixed two days of no battery boost	Taobao Marketplace, a Chinese website for online shopping
altt flagship store	Battery Reconditioner	very strong, the use of the effect is very good, will be a can not start the cell phone battery repair charging to full power95%, practice proves that this new product is trustworthy, and the description of the business is consistent.	Taobao Marketplace, a Chinese website for online shopping
altt flagship store	Battery Reconditioner	Works great Good quality	Taobao Marketplace, a Chinese website for online shopping
altt flagship store	Battery Reconditioner	Why don't you buy the equipment stay to buy the hero ah beyond expectations, the quality is very good, fine workmanship, satisfied with	Taobao Marketplace, a Chinese website for online shopping
altt flagship store	Battery Reconditioner	Cell phone battery repair artifact received, inserted intoUSbcharging head, and then connected to the phone, a number of industry immediately have a change, from5Voutput to pulse charging1Vtrickle charging, play a role in repairing, protecting the battery. Fully charged, the phone is more than the usual power for half a day. Small things play a big role. It is a good new product.	Taobao Marketplace, a Chinese website for online shopping
altt flagship store	Battery Reconditioner	Very good quality, well made and good value for money.	Taobao Marketplace, a Chinese website for online shopping
altt flagship store	Battery Reconditioner	A very pleasant shopping. This battery restorer is very good, it's genuine can't wait to get it back can't wait to use it Good quality. Charging stability. Need to buy again.	Taobao Marketplace, a Chinese website for online shopping
altt flagship store	Battery Reconditioner	Purchased for the second time, the quality is still poor, the first time I bought it back less than a month it was broken, this time I do not know how many days it can be	Taobao Marketplace, a Chinese website for online shopping
altt flagship store	Battery Reconditioner	It doesn't work at all. It's a scam	Taobao Marketplace, a Chinese website for online shopping
altt flagship store	Battery Reconditioner	A friend referred it to me, it's pretty good and good value for money!	Taobao Marketplace, a Chinese website for online shopping
altt flagship store	Battery Reconditioner	j	Taobao Marketplace, a Chinese website for online shopping
altt flagship store	Battery Reconditioner	Quality is very good, the old cell phone battery used for a long time, repair cell phone battery is effective, charging speed has become faster, value for money, cost-effective.	Taobao Marketplace, a Chinese website for online shopping
altt flagship store	Battery Reconditioner	This cell phone battery repairer was received very quickly! Its quality, really good! This baby, its appearance, exquisite production, fashionable technology, small and beautiful, convenient and good use! Efficient top quality, repair effect, worth looking forward to and buying!	Taobao Marketplace, a Chinese website for online shopping
altt flagship store	Battery Reconditioner	It's basically an IQ tax.	Taobao Marketplace, a Chinese website for online shopping
altt flagship store	Battery Reconditioner	Works well. Charged the battery a couple times. Battery life is better than before. Satisfied with the shopping experience.	Taobao Marketplace, a Chinese website for online shopping
altt flagship store	Battery Reconditioner	Something that's confusing.	Taobao Marketplace, a Chinese website for online shopping
altt flagship store	Battery Reconditioner	appearance of the material is also very good, the charging speed is slow	Taobao Marketplace, a Chinese website for online shopping
altt flagship store	Battery Reconditioner	phone battery repairer after repairing the cell phone battery is obviously faster than before when charging, logistics is also very fast.	Taobao Marketplace, a Chinese website for online shopping
altt flagship store	Battery Reconditioner	Cell phone battery repairer received, looked good, as described, there are instructions have the use of methods, plugged in the words of the repair, I hope the effect is obvious, or to give five-star praise.	Taobao Marketplace, a Chinese website for online shopping
altt flagship store	Battery Reconditioner	It's as if there's no counter-image!	Taobao Marketplace, a Chinese website for online shopping
altt flagship store	Battery Reconditioner	I don't know if it works.	Taobao Marketplace, a Chinese website for online shopping
altt flagship store	Battery Reconditioner	Don't sell the fakes. Fakes.	Taobao Marketplace, a Chinese website for online shopping
altt flagship store	Battery Reconditioner	convenient and easy to use	Taobao Marketplace, a Chinese website for online shopping
altt flagship store	Battery Reconditioner	Baby received, very good, satisfied!	Taobao Marketplace, a Chinese website for online shopping
altt flagship store	Battery Reconditioner	durable, by cheap recommended to buy this fixer, did not expect to use cell phone is indeed smooth the battery feels durable to, do not have to always take the charging treasure, very satisfied with an online shopping , there is a need to also come to buy.	Taobao Marketplace, a Chinese website for online shopping
altt flagship store	Battery Reconditioner	Received, don't know if it works, try it.	Taobao Marketplace, a Chinese website for online shopping

altt flagship store	Battery Reconditioner	It's working. The batteries do last longer than before.	Taobao Marketplace, a Chinese website for online shopping
altt flagship store	Battery Reconditioner	After using it for a week, there is a problem A fully charged phone turns around and looks at it and it is only 70% charged And sometimes it is only 80% charged even after charging all night It can't even charge 100% for 7 hours**I thought I was charging a battery car	Taobao Marketplace, a Chinese website for online shopping
altt flagship store	Battery Reconditioner	What****really These positive reviews are either**or charged Since using this phone are a little bit stuck and charging is not enough Not plugged in this is two hours to be full	Taobao Marketplace, a Chinese website for online shopping
altt flagship store	Battery Reconditioner	I've used it twice and it works quite well and stretches out when I use it	Taobao Marketplace, a Chinese website for online shopping
altt flagship store	Battery Reconditioner	No restorative effect	Taobao Marketplace, a Chinese website for online shopping
altt flagship store	Battery Reconditioner	IQ tax. It doesn't work.	Taobao Marketplace, a Chinese website for online shopping
altt flagship store	Battery Reconditioner	Cell phone battery repairer after hours of charging and on a cell phone that can not be charged into the repair, a few hours to repair the success, kudos! Commodity description is consistent, logistics is so fast, worth buying!	Taobao Marketplace, a Chinese website for online shopping
altt flagship store	Battery Reconditioner	Cell phone battery repair device has been received, with this repair device on the cell phone battery repair comparative	Taobao Marketplace, a Chinese website for online shopping
altt flagship store	Battery Reconditioner	I just received it last night and will evaluate it after using it, giving it 5 stars for now. But the logistics is very fast.	Taobao Marketplace, a Chinese website for online shopping
altt flagship store	Battery Reconditioner	ineffective	Taobao Marketplace, a Chinese website for online shopping
altt flagship store	Battery Reconditioner	The product is fake and only has one head, it's like a battery saver, it doesn't increase the charge of the phone at all like he said it would. I have used it a few times and it does what he says, it does not make the phone charge the appliance like he says it does. Sincerely caution all my friends online, make sure you don't believe his bullshit. Charging speed: too bad Contact sensitivity: too bad Appearance material: general	Taobao Marketplace, a Chinese website for online shopping
altt flagship store	Battery Reconditioner	It's a crock of shit, I bought $69 and it doesn't charge at all.	Taobao Marketplace, a Chinese website for online shopping
altt flagship store	Battery Reconditioner	It's pretty good, the fix is estimated to take a couple days. I'll use it first.	Taobao Marketplace, a Chinese website for online shopping
altt flagship store	Battery Reconditioner	Very good, satisfied!	Taobao Marketplace, a Chinese website for online shopping
altt flagship store	Battery Reconditioner	It's a good product.	Taobao Marketplace, a Chinese website for online shopping
altt flagship store	Battery Reconditioner	acceptable	Taobao Marketplace, a Chinese website for online shopping
altt flagship store	Battery Reconditioner	Only after using it to evaluate, the appearance is exquisite, charging speed is very fast, satisfied with the	Taobao Marketplace, a Chinese website for online shopping
altt flagship store	Battery Reconditioner	Just use, have not yet seen the effect of use, wait for a period of time to see if there is effect then make a follow-up comment! First give habitual favorable comments!	Taobao Marketplace, a Chinese website for online shopping
altt flagship store	Battery Reconditioner	Baby's not bad.	Taobao Marketplace, a Chinese website for online shopping
altt flagship store	Battery Reconditioner	I said it was good when I bought it, but it's just a lie and doesn't work	Taobao Marketplace, a Chinese website for online shopping
altt flagship store	Battery Reconditioner	Yes.Use to charge and turn off as instructed, 2times battery health back up4%, use again in a few days.	Taobao Marketplace, a Chinese website for online shopping
altt flagship store	Battery Reconditioner	Average, not very effective.	Taobao Marketplace, a Chinese website for online shopping
altt flagship store	Battery Reconditioner	favorable criticism	Taobao Marketplace, a Chinese website for online shopping
altt flagship store	Battery Reconditioner	?	Taobao Marketplace, a Chinese website for online shopping
altt flagship store	Battery Reconditioner	Battery Repairer received, fast logistics, the use of a period of time to evaluate, the effect is very good, the battery use time increased significantly a lot, in accordance with the instructions required by the shutdown repair effect, without interference, the effect can reach more than ninety percent!	Taobao Marketplace, a Chinese website for online shopping
altt flagship store	Battery Reconditioner	Baby safe delivery, packaging is quite good, first try to use, do not know how the effect habit of praise!	Taobao Marketplace, a Chinese website for online shopping
altt flagship store	Battery Reconditioner	I didn't realize this contraption was so useful, I just charged it twice yesterday and the reminder icon on my phone is gone, it's really amazing! Thanks for the high tech and thanks to the store owner!	Taobao Marketplace, a Chinese website for online shopping
altt flagship store	Battery Reconditioner	I thought this thing was another IQ tax, but I was really wrong this time, it works!	Taobao Marketplace, a Chinese website for online shopping
altt flagship store	Battery Reconditioner	Used for a period of time, from the appearance and workmanship is very satisfied, and the business description-consistent, have effect, and now charging has not been hot	Taobao Marketplace, a Chinese website for online shopping
altt flagship store	Battery Reconditioner	Do not use this charging okay, with this charging charging head and this hot, easy to accident!	Taobao Marketplace, a Chinese website for online shopping

altt flagship store	Battery Reconditioner	Goods to receive, good or bad wait for a period of time to evaluate, first give a five-star praise.	Taobao Marketplace, a Chinese website for online shopping
altt flagship store	Battery Reconditioner	It doesn't work, I recommend not buying it and honestly changing the battery is ok!	Taobao Marketplace, a Chinese website for online shopping
altt flagship store	Battery Reconditioner	It's working. It looks good now.	Taobao Marketplace, a Chinese website for online shopping
altt flagship store	Battery Reconditioner	I've been using it for two weeks and can't see any results.	Taobao Marketplace, a Chinese website for online shopping
altt flagship store	Battery Reconditioner	Works well charging process doesn't burn your hands anymore. Effective and noticeable! Thanks for the kudos!	Taobao Marketplace, a Chinese website for online shopping
altt flagship store	Battery Reconditioner	Repairer shipment received. Going to try it on an unused phone to see how it works.	Taobao Marketplace, a Chinese website for online shopping
altt flagship store	Battery Reconditioner	According to the description of the introduction is very good, good service, heat less often, especially once open the King of Glory, the game opens faster, not bad dozens of things	Taobao Marketplace, a Chinese website for online shopping
altt flagship store	Battery Reconditioner	Appearance material: good Charging speed: fast In use, see the effect and then make evaluation	Taobao Marketplace, a Chinese website for online shopping
altt flagship store	Battery Reconditioner	Been using it for two weeks now and it doesn't feel like it's working! Repairing batteries🔋can't see the results……	Taobao Marketplace, a Chinese website for online shopping
altt flagship store	Battery Reconditioner	Not bad👍pretty good👌good price for	Taobao Marketplace, a Chinese website for online shopping
altt flagship store	Battery Reconditioner	The goods have been received, and I will comment after using it.	Taobao Marketplace, a Chinese website for online shopping
altt flagship store	Battery Reconditioner	Poor quality, broke in less than a month	Taobao Marketplace, a Chinese website for online shopping
altt flagship store	Battery Reconditioner	It's well made, but personally I feel it should be an IQ tax	Taobao Marketplace, a Chinese website for online shopping
altt flagship store	Battery Reconditioner	Fast shipping, good quality Contact s:good sensitivity Appearance material:comfortable feel Charging speed:very fast	Taobao Marketplace, a Chinese website for online shopping
altt flagship store	Battery Reconditioner	slow, charged. I see from yesterday on 12:30 charging repair, rushed to this morning 7:30, showing that the charge is ninety-five percent. Use it first to see what the effect is behind.	Taobao Marketplace, a Chinese website for online shopping
altt flagship store	Battery Reconditioner	Logistics is very fast, product quality can be, satisfied	Taobao Marketplace, a Chinese website for online shopping
altt flagship store	Battery Reconditioner	Doesn't feel like it'll do much to fix your cell phone's battery.	Taobao Marketplace, a Chinese website for online shopping
altt flagship store	Battery Reconditioner	I used it only a few times and thought it was ineffective, but after a few more times I seem to be seeing a lot of improvement in my battery.	Taobao Marketplace, a Chinese website for online shopping
altt flagship store	Battery Reconditioner	Just started to use bad evaluation	Taobao Marketplace, a Chinese website for online shopping
altt flagship store	Battery Reconditioner	thankfully	Taobao Marketplace, a Chinese website for online shopping
altt flagship store	Battery Reconditioner	Thank you; thank you; thank you very much……	Taobao Marketplace, a Chinese website for online shopping
altt flagship store	Battery Reconditioner	Thank you; thank you; thank you very much……	Taobao Marketplace, a Chinese website for online shopping
altt flagship store	Battery Reconditioner	The lens is also protected by a raised design, and it's easy to remove and install, so I'm very satisfied!	Taobao Marketplace, a Chinese website for online shopping
altt flagship store	Battery Reconditioner	There is value in using Convenient Good to use !	Taobao Marketplace, a Chinese website for online shopping
altt flagship store	Battery Reconditioner	quality of workmanship is exquisite.trying it out for a few days now, and not sure how well it works.But the old Bluetooth speakers are showing signs of life.	Taobao Marketplace, a Chinese website for online shopping
altt flagship store	Battery Reconditioner	Pretty good.	Taobao Marketplace, a Chinese website for online shopping
altt flagship store	Battery Reconditioner	The restorer was received well and I hope it works now.	Taobao Marketplace, a Chinese website for online shopping
altt flagship store	Battery Reconditioner	Black technology, good, customer service, good.	Taobao Marketplace, a Chinese website for online shopping
altt flagship store	Battery Reconditioner	This thing needs to be restored to factory settings to work, I haven't used it yet, I'm a little worried about it	Taobao Marketplace, a Chinese website for online shopping
altt flagship store	Battery Reconditioner	It arrived fine.	Taobao Marketplace, a Chinese website for online shopping
altt flagship store	Battery Reconditioner	one point, other than that it's really good and the battery lasts significantly longer than before after I followed the instructions.	Taobao Marketplace, a Chinese website for online shopping
altt flagship store	Battery Reconditioner	thank you! As described in the workmanship looks good rules standard! The effect of the test charging in the future to evaluate it! Satisfaction! Thank you for the speed of delivery, I wish the boss business is getting better and better, health and happiness!	Taobao Marketplace, a Chinese website for online shopping
altt flagship store	Battery Reconditioner	Doesn't work well, slow charging, no fixes! Poor rating!	Taobao Marketplace, a Chinese website for online shopping
altt flagship store	Battery Reconditioner	Charging speed:(good	Taobao Marketplace, a Chinese website for online shopping

altt flagship store	Battery Reconditioner	I've been using it for over a month now and it's not working at all, and now I don't know about charging it up	Taobao Marketplace, a Chinese website for online shopping
altt flagship store	Battery Reconditioner	(of an unmarried couple) be close	Taobao Marketplace, a Chinese website for online shopping
altt flagship store	Battery Reconditioner	Things received last night to take me a cell phone can not be charged into the try, charging a night today actually can turn on, surprise, things should be good!	Taobao Marketplace, a Chinese website for online shopping
altt flagship store	Battery Reconditioner	Here's my4sthat wouldn't turn on for a couple years, fixed it for half an hour and it turned on automatically!	Taobao Marketplace, a Chinese website for online shopping
altt flagship store	Battery Reconditioner	I don't know how effective it is, and I can't see how cell phone battery repair.	Taobao Marketplace, a Chinese website for online shopping
altt flagship store	Battery Reconditioner	description. It's really a black technology, it's breathtaking.	Taobao Marketplace, a Chinese website for online shopping
altt flagship store	Battery Reconditioner	I've been using it for a while and don't think it's effective, so I don't know where all the positive reviews come from. Just use it as a monitor! Not recommended!	Taobao Marketplace, a Chinese website for online shopping
altt flagship store	Battery Reconditioner	before buying and the seller negotiated well within a week not satisfied can be returned, but think about a pack of cigarettes money, do not intend to return, just want to wait forthe results after8weeks, if still not useful, and then ask the relatives, this kind of product can still be on the platform!	Taobao Marketplace, a Chinese website for online shopping
altt flagship store	Battery Reconditioner	So far, no results.	Taobao Marketplace, a Chinese website for online shopping
altt flagship store	Battery Reconditioner	can	Taobao Marketplace, a Chinese website for online shopping
altt flagship store	Battery Reconditioner	It should be fine after a few uses.	Taobao Marketplace, a Chinese website for online shopping
altt flagship store	Battery Reconditioner	I've used it twice now, and I haven't seen any effect at all.	Taobao Marketplace, a Chinese website for online shopping
altt flagship store	Battery Reconditioner	Bought cell phone battery repairer received more**, have been using, the effect is good, the seller's service is hospitable, after receiving do not know how to use the seller also sent a detailed description, trustworthy business.	Taobao Marketplace, a Chinese website for online shopping
altt flagship store	Battery Reconditioner	The results are much better than what you can buy at other places!	Taobao Marketplace, a Chinese website for online shopping
altt flagship store	Battery Reconditioner	replacing the battery last year, the new battery dropped points fast, got hot, and recently rebooted frequently. So I bought a new phone and turned the Apple12off and didn't use it. Recently in Taobao pulled out this baby. Dusty half a year of Apple12open charging, did not expect a miracle: fully charged, continuously watching21/2 hours of movies, the machine does not heat up, and does not reboot. If I had known this baby, I would not have bought a new cell phone. Thank you to the business, so that the faulty idle cell phone back to	Taobao Marketplace, a Chinese website for online shopping
altt flagship store	Battery Reconditioner	Good review!	Taobao Marketplace, a Chinese website for online shopping
altt flagship store	Battery Reconditioner	no obvious effect, and then use it after the follow-up comment!	Taobao Marketplace, a Chinese website for online shopping
altt flagship store	Battery Reconditioner	There's no effect. Merchants do what's on their conscience.	Taobao Marketplace, a Chinese website for online shopping
altt flagship store	Battery Reconditioner	with the power off, and I can't charge it with the phone on, sometimes it doesn't charge enough, and I don't know what the reason is.	Taobao Marketplace, a Chinese website for online shopping
altt flagship store	Battery Reconditioner	Battery reconditioner doesn't do much good, it burns out after 3 or 4 uses	Taobao Marketplace, a Chinese website for online shopping
altt flagship store	Battery Reconditioner	It shows repair in progress without the cable connected, is it fixing a ghost? Obviously it's something for the Mabian people, think about it if you want to buy it!	Taobao Marketplace, a Chinese website for online shopping
altt flagship store	Battery Reconditioner	The goods have been received, everything is normal, the packaging is very good, the cell phone repairer is good quite good, is genuine!	Taobao Marketplace, a Chinese website for online shopping
altt flagship store	Battery Reconditioner	Battery Repairer is particularly good, cost-effective, like you can get it!	Taobao Marketplace, a Chinese website for online shopping
altt flagship store	Battery Reconditioner	was good and the shipping was pretty fast, all in all it was awesome!	Taobao Marketplace, a Chinese website for online shopping
altt flagship store	Battery Reconditioner	In use, the display is normal, now with the store description of the match, friends have the effect before buying, with a longer time than before	Taobao Marketplace, a Chinese website for online shopping
altt flagship store	Battery Reconditioner	Things have been received can not wait to start using the effect is quite good, just use the phone to see the effect.	Taobao Marketplace, a Chinese website for online shopping
altt flagship store	Battery Reconditioner	The battery adapter works, the battery now lasts longer than before and playing games is much smoother!	Taobao Marketplace, a Chinese website for online shopping
altt flagship store	Battery Reconditioner	Logistics is very fast, and the power off and charge, really effective, full of power on, charging is not enough should be in the repair of the battery!	Taobao Marketplace, a Chinese website for online shopping

altt flagship store	Battery Reconditioner	Not recommended to buy, no effect, from the beginning of the battery loss a few days a sharp decline, do not dare to use again, this is a test current and voltage machine only, there is no possibility of repairing the battery.	Taobao Marketplace, a Chinese website for online shopping
altt flagship store	Battery Reconditioner	Baby received, good quality, cost-effective, give a five-star praise	Taobao Marketplace, a Chinese website for online shopping
altt flagship store	Battery Reconditioner	Logistics is fast, the product was received quickly, better than expected, good good! I hope it will be durable.	Taobao Marketplace, a Chinese website for online shopping
altt flagship store	Battery Reconditioner	charging can always monitor the charging voltage, current, according to the instructions, there is a clear effect of repairing the battery. Good customer service and fast logistics.	Taobao Marketplace, a Chinese website for online shopping
altt flagship store	Battery Reconditioner	The charging effect is OK	Taobao Marketplace, a Chinese website for online shopping
altt flagship store	Battery Reconditioner	Cool looking, hope it works for a while	Taobao Marketplace, a Chinese website for online shopping
altt flagship store	Battery Reconditioner	The baby is just as introduced, the texture is very good, powerful, and it feels good to use it	Taobao Marketplace, a Chinese website for online shopping
altt flagship store	Battery Reconditioner	The seller shipped quickly,received and immediately installed,very good.	Taobao Marketplace, a Chinese website for online shopping
altt flagship store	Battery Reconditioner	Received it very quickly, very nice, satisfied	Taobao Marketplace, a Chinese website for online shopping
altt flagship store	Battery Reconditioner	In use, so far I have not felt any restorative effect on the battery!	Taobao Marketplace, a Chinese website for online shopping
altt flagship store	Battery Reconditioner	It doesn't seem to have much effect. Instead, it's a bit of a drain on the battery and doesn't hold up very well.	Taobao Marketplace, a Chinese website for online shopping
altt flagship store	Battery Reconditioner	Simple and easy works great love it!	Taobao Marketplace, a Chinese website for online shopping
altt flagship store	Battery Reconditioner	Good quality, fine workmanship, standard size, affordable price, fast logistics, good service attitude, satisfied.	Taobao Marketplace, a Chinese website for online shopping
altt flagship store	Battery Reconditioner	Battery Repairer is very good, now the cell phone battery is also much more durable, very good, recommended!	Taobao Marketplace, a Chinese website for online shopping
altt flagship store	Battery Reconditioner	Slow charging, so-called"repair"is onlypaper, believe it or not, try it!	Taobao Marketplace, a Chinese website for online shopping
altt flagship store	Battery Reconditioner	Fooled again,no improvement at all3charges.	Taobao Marketplace, a Chinese website for online shopping
altt flagship store	Battery Reconditioner	only charging85%now, what the****.	Taobao Marketplace, a Chinese website for online shopping
altt flagship store	Battery Reconditioner	Try it out and give it a good review.	Taobao Marketplace, a Chinese website for online shopping
altt flagship store	Battery Reconditioner	It doesn't work. I'd advise against it.	Taobao Marketplace, a Chinese website for online shopping
altt flagship store	Battery Reconditioner	Slow to charge, but still works well and has already lasted longer than it originally did	Taobao Marketplace, a Chinese website for online shopping
altt flagship store	Battery Reconditioner	pretty good	Taobao Marketplace, a Chinese website for online shopping
altt flagship store	Battery Reconditioner	Used a few days feel good, charging the number of times significantly less, directly pushed to a few good friends with the purchase, the price is also very affordable, the need to hurry up to prepare a ha!	Taobao Marketplace, a Chinese website for online shopping
altt flagship store	Battery Reconditioner	(of an unmarried couple) be close	Taobao Marketplace, a Chinese website for online shopping
altt flagship store	Battery Reconditioner	(of an unmarried couple) be close	Taobao Marketplace, a Chinese website for online shopping
altt flagship store	Battery Reconditioner	Good review!	Taobao Marketplace, a Chinese website for online shopping
altt flagship store	Battery Reconditioner	Just received the goods, not yet used, use and then evaluation	Taobao Marketplace, a Chinese website for online shopping
altt flagship store	Battery Reconditioner	OK	Taobao Marketplace, a Chinese website for online shopping
altt flagship store	Battery Reconditioner	Particularly good repairer, the quality of workmanship can not be said, very delicate, the only drawback is that the charging is slow, but there are effects that line!	Taobao Marketplace, a Chinese website for online shopping
altt flagship store	Battery Reconditioner	I'm using it, I don't know the effect yet, I'll comment later!	Taobao Marketplace, a Chinese website for online shopping
altt flagship store	Battery Reconditioner	It's an IQ tax. Whoever buys it gets screwed.	Taobao Marketplace, a Chinese website for online shopping
altt flagship store	Battery Reconditioner	I do not know if it is good, I just got the goods, with two days of additional reviews	Taobao Marketplace, a Chinese website for online shopping
altt flagship store	Battery Reconditioner	Let's try it for a while.	Taobao Marketplace, a Chinese website for online shopping
altt flagship store	Battery Reconditioner	It's useless. Whoever buys it will be fooled.	Taobao Marketplace, a Chinese website for online shopping
altt flagship store	Battery Reconditioner	Good review!	Taobao Marketplace, a Chinese website for online shopping
altt flagship store	Battery Reconditioner	an old cell phone, often frequent shutdown and reboot, all want to throw away do not want! I bought back this fixer and charged it first, and it really did the job of fixing it! Now it shuts off once in a day.	Taobao Marketplace, a Chinese website for online shopping

altt flagship store	Battery Reconditioner	personally used it, not only did it not work, I couldn't repair any of the batteries, 17%when I forked the battery, butonly16%after12hoursforked the, and it didn't even function as a forked battery, it kept decreasing the battery power. the merchant, who told me to pay another ￥25buy another one. I asked the merchant whether the battery recovery function is really effective after I ordered another new recuperator, but the merchant didn't answer the question positively! I think there is no honesty again, I've suffered a loss once!	Taobao Marketplace, a Chinese website for online shopping
altt flagship store	Battery Reconditioner	Test it out for a while and then come back with a review	Taobao Marketplace, a Chinese website for online shopping
altt flagship store	Battery Reconditioner	Effective, used once or twice, consistent with the owner's description, next time to buy again	Taobao Marketplace, a Chinese website for online shopping
altt flagship store	Battery Reconditioner	It works. Works well.	Taobao Marketplace, a Chinese website for online shopping
altt flagship store	Battery Reconditioner	I hope it works.	Taobao Marketplace, a Chinese website for online shopping
altt flagship store	Battery Reconditioner	The phone really doesn't heat up when charging with this one	Taobao Marketplace, a Chinese website for online shopping
altt flagship store	Battery Reconditioner	battery restorer, I want to shoot a try. After a few days of trial, feel the battery performance significantly improved, see the effect, greatly enhanced my confidence in this baby, the battery is saved!	Taobao Marketplace, a Chinese website for online shopping
altt flagship store	Battery Reconditioner	I bought this baby, my cell phone battery is much more durable than the original, my friend came to my house and saw it also want to buy one.	Taobao Marketplace, a Chinese website for online shopping
altt flagship store	Battery Reconditioner	It feels good to use and can!	Taobao Marketplace, a Chinese website for online shopping
altt flagship store	Battery Reconditioner	This product has been tried. Tried out three old cell phones and they felt much better than they were, and the internet access was sped up on all of them. Now trying an old laptop to see how it works.	Taobao Marketplace, a Chinese website for online shopping
altt flagship store	Battery Reconditioner	I haven't used it yet, so let's give it a good review!	Taobao Marketplace, a Chinese website for online shopping
altt flagship store	Battery Reconditioner	Tried it for a few days before coming to evaluate, the service is very good. My old cell phone could not be used for an hour, after using this restorer once it really works, now it can be used for more than two hours!	Taobao Marketplace, a Chinese website for online shopping
altt flagship store	Battery Reconditioner	Charging is steady, and you can also take care of your cell phone battery without hurting your phone.	Taobao Marketplace, a Chinese website for online shopping
altt flagship store	Battery Reconditioner	From the packaging of the product, to the texture of the product in my hand, I think it's a great product. Look at its work seems to be balancing the current! Should be a good product. Later in the supplement battery repair effect it!	Taobao Marketplace, a Chinese website for online shopping
altt flagship store	Battery Reconditioner	Really quite effective, charged to100%and then swiped the video continuously from17:16until19:41,2hoursand 20minutes, the power used upa little over 50%. I'm sure it will work even better with a few more uses of this!	Taobao Marketplace, a Chinese website for online shopping
altt flagship store	Battery Reconditioner	It's okay! The first time I received a little problem with the restorer, and the store reacted, to re-exchange a normal! As for the repair effect has to be a period of time in order to see whether there is no effect, and then follow up later!	Taobao Marketplace, a Chinese website for online shopping
altt flagship store	Battery Reconditioner	What to say, used a few days did not feel anything special, according to the store said in the trial to see it, customary praise.	Taobao Marketplace, a Chinese website for online shopping
altt flagship store	Battery Reconditioner	Baby received, fast shipping, and the seller described the same, very satisfied!	Taobao Marketplace, a Chinese website for online shopping
altt flagship store	Battery Reconditioner	The restorer charges steadily, has fixed the battery somewhat, and the phone is smoother than it was a few days ago, and plays much longer than it did before time	Taobao Marketplace, a Chinese website for online shopping
altt flagship store	Battery Reconditioner	Charging speed: very fast Delivery: door-to-door, very good attitude!	Taobao Marketplace, a Chinese website for online shopping
altt flagship store	Battery Reconditioner	Baby received and works great._x005f_x0001_ Merchant shipped quickly and promptly.	Taobao Marketplace, a Chinese website for online shopping
altt flagship store	Battery Reconditioner	alttcell phone battery repairer has been received unused, first to the business five praise, the courier is also very fast.	Taobao Marketplace, a Chinese website for online shopping
altt flagship store	Battery Reconditioner	The goods are very good and durable, the quality is very good at first glance also will buy back, very like	Taobao Marketplace, a Chinese website for online shopping
altt flagship store	Battery Reconditioner	Good service, fast shipping and logistics for orders!	Taobao Marketplace, a Chinese website for online shopping
altt flagship store	Battery Reconditioner	I've been using it for a while now and it works pretty well.	Taobao Marketplace, a Chinese website for online shopping
altt flagship store	Battery Reconditioner	Good quality, pretty responsive, sensitive contact.	Taobao Marketplace, a Chinese website for online shopping
altt flagship store	Battery Reconditioner	Just started using the effect can not be seen yet, use a period of time to see.	Taobao Marketplace, a Chinese website for online shopping
altt flagship store	Battery Reconditioner	Try it out before you comment, it's very cartoonish looking.	Taobao Marketplace, a Chinese website for online shopping

altt flagship store	Battery Reconditioner	👍	Taobao Marketplace, a Chinese website for online shopping
altt flagship store	Battery Reconditioner	The store shipped very quickly, baby received after the trial, feel well-made, easy to use, small and exquisite. It is the protector of old cell phones and old batteries.	Taobao Marketplace, a Chinese website for online shopping
altt flagship store	Battery Reconditioner	repair old batteries of old cell phones and improve the efficiency of use.	Taobao Marketplace, a Chinese website for online shopping
altt flagship store	Battery Reconditioner	This product has been tried. Tried three old cell phones and felt like they opened and surfed the web faster than they did. Now trying an old laptop to see how it works. The product does not fail to deliver.	Taobao Marketplace, a Chinese website for online shopping
altt flagship store	Battery Reconditioner	Have just tested it on my laptop and the results have improved. Booting up is faster than it was, opening web pages, files, videos have all sped up a bit. Going to test a couple more times to see if there is any more improvement.	Taobao Marketplace, a Chinese website for online shopping
altt flagship store	Battery Reconditioner	Exquisite packaging, design texture, fast delivery, with detailed instructions for use in English and Chinese.	Taobao Marketplace, a Chinese website for online shopping
altt flagship store	Battery Reconditioner	Last night, shut down, try2phones, one of the phone, in front of the use of a moment on the heatmore than30degrees, the first repair, obviously feel the heat problem has improved, should be the goods really, and then repair one or two to see if the real phone is obviously not hot, it should be true	Taobao Marketplace, a Chinese website for online shopping
altt flagship store	Battery Reconditioner	This is really useful stuff to follow along with by looking at said name book!	Taobao Marketplace, a Chinese website for online shopping
altt flagship store	Battery Reconditioner	Just received it, haven't used it yet	Taobao Marketplace, a Chinese website for online shopping
altt flagship store	Battery Reconditioner	Finally received the baby I need, the stuff is very good, the boss attitude is very professional and enthusiastic, ask questions and reply quickly, I asked a lot of questions, he does not feel annoyed, they will seriously answer me.	Taobao Marketplace, a Chinese website for online shopping
altt flagship store	Battery Reconditioner	At first did not use according to the instructions, the effect is not obvious, contact the owner of the shop to understand how to use, and now has been more durable than before	Taobao Marketplace, a Chinese website for online shopping
altt flagship store	Battery Reconditioner	Works well, good review!	Taobao Marketplace, a Chinese website for online shopping
altt flagship store	Battery Reconditioner	Baby received, the quality is very good, logistics is also very fast, the material is thick and environmentally friendly, it is worth recommending!	Taobao Marketplace, a Chinese website for online shopping
altt flagship store	Battery Reconditioner	It's not working at all.	Taobao Marketplace, a Chinese website for online shopping
altt flagship store	Battery Reconditioner	Good quality and fast logistics	Taobao Marketplace, a Chinese website for online shopping
altt flagship store	Battery Reconditioner	Have not used it yet, will comment later.	Taobao Marketplace, a Chinese website for online shopping
altt flagship store	Battery Reconditioner	Baby received, has used once, now can not see the effect, hope it is good.	Taobao Marketplace, a Chinese website for online shopping
altt flagship store	Battery Reconditioner	Cell phone repairer received. Bought it and turned it off and fully charged it. Turn on the phone much smoother. I do not know how much the battery repair, I am now according to 2-3timesweekto charge a repair battery, has been used longer	Taobao Marketplace, a Chinese website for online shopping
altt flagship store	Battery Reconditioner	The seller was nice and shipped quickly. Exactly as described on the web, with a fresh production date. The stuff is good value for money, good price, affordable and trustworthy. A satisfactory online shopping.	Taobao Marketplace, a Chinese website for online shopping
altt flagship store	Battery Reconditioner	What cell phone battery repairer, don't be fooled. The more you charge, the more you deplete the life of your cell phone battery. Pictures as evidence. Everyone should think clearly.l. Wasted more than fifty dollars not to mention. Also depletes the life of the cell phone battery capacity.	Taobao Marketplace, a Chinese website for online shopping
altt flagship store	Battery Reconditioner	I received it and used it for a while, but I don't know if it works.	Taobao Marketplace, a Chinese website for online shopping
altt flagship store	Battery Reconditioner	IQ tax	Taobao Marketplace, a Chinese website for online shopping
altt flagship store	Battery Reconditioner	I've been using it for a few days now, it's all charged with the power off, it works well and it's not too expensive, it's worth buying!	Taobao Marketplace, a Chinese website for online shopping
altt flagship store	Battery Reconditioner	Fixer received, looking forward to the results	Taobao Marketplace, a Chinese website for online shopping
altt flagship store	Battery Reconditioner	It's working fine.	Taobao Marketplace, a Chinese website for online shopping
altt flagship store	Battery Reconditioner	Fast logistics, affordable price, so good it can only be described as perfect! Highly recommended!	Taobao Marketplace, a Chinese website for online shopping
altt flagship store	Battery Reconditioner	pretty good	Taobao Marketplace, a Chinese website for online shopping
altt flagship store	Battery Reconditioner	also very good, delivery is timely, very satisfied, will buy back!	Taobao Marketplace, a Chinese website for online shopping
altt flagship store	Battery Reconditioner	Used a few days, now the phone battery is also much more durable, very good product, recommended!	Taobao Marketplace, a Chinese website for online shopping

altt flagship store	Battery Reconditioner	Boss is very good, logistics is also particularly fast, I am liberation East Road 141China Express is particularly bad, always send my courier to the victory of the road, so that I - an80-year-old woman more than a hundred meters away, and is not handed over to my own hands, to the China Express I can only give him3points to forget!	Taobao Marketplace, a Chinese website for online shopping
altt flagship store	Battery Reconditioner	The goods have been received and tried.	Taobao Marketplace, a Chinese website for online shopping
altt flagship store	Battery Reconditioner	Well packaged, fast shipping, good customer service, recommend buying.	Taobao Marketplace, a Chinese website for online shopping
altt flagship store	Battery Reconditioner	Logistics was also very strong! Received the item very quickly! First give a favorable comment! Other use and then evaluate!	Taobao Marketplace, a Chinese website for online shopping
altt flagship store	Battery Reconditioner	Used it for a couple days and didn't feel much. Or maybe there's nothing wrong with your own phone	Taobao Marketplace, a Chinese website for online shopping
altt flagship store	Battery Reconditioner	Can't wait to open the package and the business description of the same , charging speed is also very fast , compact and portable, very satisfied with a shopping.	Taobao Marketplace, a Chinese website for online shopping
altt flagship store	Battery Reconditioner	Looking forward to the results Charging Speed:Fast	Taobao Marketplace, a Chinese website for online shopping
altt flagship store	Battery Reconditioner	The quality is fine, as described, it lasted longer than before	Taobao Marketplace, a Chinese website for online shopping
altt flagship store	Battery Reconditioner	That's good!	Taobao Marketplace, a Chinese website for online shopping
altt flagship store	Battery Reconditioner	Logistics is super super fast! The quality is also very good, indeed value for money Logistics is super super fast!	Taobao Marketplace, a Chinese website for online shopping
altt flagship store	Battery Reconditioner	Nothing works. It doesn't work at all.	Taobao Marketplace, a Chinese website for online shopping
altt flagship store	Battery Reconditioner	shopkeeper has a very good service attitude, satisfied with the	Taobao Marketplace, a Chinese website for online shopping
altt flagship store	Battery Reconditioner	Logistics fast, using this battery is really still durable and new batteries, the same, effective ha. Customer service attitude is very good, we support the store has a quality assurance Oh.2023quickly come to buy it.	Taobao Marketplace, a Chinese website for online shopping
altt flagship store	Battery Reconditioner	To be evaluated after use.	Taobao Marketplace, a Chinese website for online shopping
altt flagship store	Battery Reconditioner	Logistics is very fast Received very like shopkeeper service attitude is very good	Taobao Marketplace, a Chinese website for online shopping
altt flagship store	Battery Reconditioner	It's good quality.	Taobao Marketplace, a Chinese website for online shopping
altt flagship store	Battery Reconditioner	Good repair, good quality	Taobao Marketplace, a Chinese website for online shopping
altt flagship store	Battery Reconditioner	IQ sleep	Taobao Marketplace, a Chinese website for online shopping
altt flagship store	Battery Reconditioner	Battery Reconditioner received, very good quality, the effect is very obvious!	Taobao Marketplace, a Chinese website for online shopping
altt flagship store	Battery Reconditioner	(of an unmarried couple) be close	Taobao Marketplace, a Chinese website for online shopping
altt flagship store	Battery Reconditioner	Charged three times, feel no effect, continue to observe	Taobao Marketplace, a Chinese website for online shopping
altt flagship store	Battery Reconditioner	Got it. Good.	Taobao Marketplace, a Chinese website for online shopping
altt flagship store	Battery Reconditioner	Fast shipping, the price is much cheaper than the physical store, very satisfied with a shopping.	Taobao Marketplace, a Chinese website for online shopping
altt flagship store	Battery Reconditioner	Workmanship is more exquisite, can display data, can repair the battery effect to be observed	Taobao Marketplace, a Chinese website for online shopping
altt flagship store	Battery Reconditioner	The quality of the item is very good and worth buying the product.	Taobao Marketplace, a Chinese website for online shopping
altt flagship store	Battery Reconditioner	The quality is very good, quite satisfied.	Taobao Marketplace, a Chinese website for online shopping
altt flagship store	Battery Reconditioner	customer service attitude is also good, there are problems are solved, the next wait to try the effect is good if not in the follow-up.	Taobao Marketplace, a Chinese website for online shopping
altt flagship store	Battery Reconditioner	Great service, convenient, effective and affordable!	Taobao Marketplace, a Chinese website for online shopping
altt flagship store	Battery Reconditioner	Really cheating, charging a night is less than half, a few times are like this, the next day early powerless mischief, alas really pitiful, we must not buy!	Taobao Marketplace, a Chinese website for online shopping
altt flagship store	Battery Reconditioner	Restorer arrived, just used a short time, a little effect, may not be all eucalyptus instructions to do, the next will be in accordance with the specification of the next	Taobao Marketplace, a Chinese website for online shopping
altt flagship store	Battery Reconditioner	Doesn't do much, still half a day of charging.	Taobao Marketplace, a Chinese website for online shopping
altt flagship store	Battery Reconditioner	Doesn't help It should power down fast or it should power down fast Doesn't make any difference Charging speed: okay	Taobao Marketplace, a Chinese website for online shopping
altt flagship store	Battery Reconditioner	Exterior material: compact Charging speed: okay	Taobao Marketplace, a Chinese website for online shopping

altt flagship store	Battery Reconditioner	Seller shipped quickly, good quality, good repair, good service, very satisfied	Taobao Marketplace, a Chinese website for online shopping
altt flagship store	Battery Reconditioner	That's good!	Taobao Marketplace, a Chinese website for online shopping
altt flagship store	Battery Reconditioner	Fast shipping, good service, great product quality, good price, good value, recommendable.	Taobao Marketplace, a Chinese website for online shopping
altt flagship store	Battery Reconditioner	Try it is very good, before the old phone can not be charged into the power can be charged to full, I hope to charge a few more times, the old phone can be durable again, praise.	Taobao Marketplace, a Chinese website for online shopping
altt flagship store	Battery Reconditioner	Baby received, in line with the description, logistics is very fast, quality and service attitude is good.	Taobao Marketplace, a Chinese website for online shopping
altt flagship store	Battery Reconditioner	The effect is not obvious, habitually favorable.	Taobao Marketplace, a Chinese website for online shopping
altt flagship store	Battery Reconditioner	understand if it has any restorative effect on cell phone batteries.	Taobao Marketplace, a Chinese website for online shopping
altt flagship store	Battery Reconditioner	the merchant, things do work well, and after charging a few times, it does feel like it is able to repair the previous non-durable.	Taobao Marketplace, a Chinese website for online shopping
altt flagship store	Battery Reconditioner	Cell phone battery repairer has been used for a period of time, not bad, not IQ tax, more durable than before.	Taobao Marketplace, a Chinese website for online shopping
altt flagship store	Battery Reconditioner	Repair cell phone charger used for a few days, also asked customer service how to use the repairer, according to customer service said rushed a few times, a little effect, the next ready to continue to operate according to this, should be able to hold on for quite a long time	Taobao Marketplace, a Chinese website for online shopping
altt flagship store	Battery Reconditioner	charge my cell phone2days, all of which were charged after turning off the phone. I feel that after using it, the charging process becomes clear, from this point of view, this is a good voltage and current regulator, after using it to extend the life of the battery to improve the charging effect is indeed beneficial.	Taobao Marketplace, a Chinese website for online shopping
altt flagship store	Battery Reconditioner	Baby received, good quality, very good, cost-effective, worth the purchase! Satisfied with a shopping	Taobao Marketplace, a Chinese website for online shopping
altt flagship store	Battery Reconditioner	high-tech products, there must be a process of adaptation, the next will be in accordance with the instructions of the program on the virtual power of the product for a period of time the battery slowly repair	Taobao Marketplace, a Chinese website for online shopping
altt flagship store	Battery Reconditioner	Used a few days, think there is effect, should be able to, just do not know the above non-stop changes in the number of all represent what it means. I don't use it during the day because I can't turn it off during the day, but when I go to bed at night, I turn it off and charge it, and it still works.	Taobao Marketplace, a Chinese website for online shopping
altt flagship store	Battery Reconditioner	Appearance Material:1Very Satisfied	Taobao Marketplace, a Chinese website for online shopping
altt flagship store	Battery Reconditioner	Baby received, logistics is fast, effective, not bad.	Taobao Marketplace, a Chinese website for online shopping
altt flagship store	Battery Reconditioner	Indeed, the repair effect is good, used a few days to follow up the evaluation, the phone electric pool obviously used for a longer period of time	Taobao Marketplace, a Chinese website for online shopping
altt flagship store	Battery Reconditioner	battery power was more than sufficient than before. Standby time is longer.	Taobao Marketplace, a Chinese website for online shopping
altt flagship store	Battery Reconditioner	battery power was more than sufficient than before. Standby time is longer.	Taobao Marketplace, a Chinese website for online shopping
altt flagship store	Battery Reconditioner	Phone case received, good quality, good workmanship, good customer service, five praise!	Taobao Marketplace, a Chinese website for online shopping
altt flagship store	Battery Reconditioner	Quite small and exquisite, the effect should be awesome, full of sense of technology	Taobao Marketplace, a Chinese website for online shopping
altt flagship store	Battery Reconditioner	Received, fast shipping, high battery repair utilization, comparable, good quality	Taobao Marketplace, a Chinese website for online shopping
altt flagship store	Battery Reconditioner	features such as details of the picture description is true, repair works!	Taobao Marketplace, a Chinese website for online shopping
altt flagship store	Battery Reconditioner	The quality is very good, the battery repair is very satisfied, very good	Taobao Marketplace, a Chinese website for online shopping
altt flagship store	Battery Reconditioner	Tried and tested, no effect at all, bragging rights.	Taobao Marketplace, a Chinese website for online shopping
altt flagship store	Battery Reconditioner	Very nice, fast shipping and the price was right!	Taobao Marketplace, a Chinese website for online shopping
altt flagship store	Battery Reconditioner	Fast logistics, good quality, fast charging. Satisfied	Taobao Marketplace, a Chinese website for online shopping
altt flagship store	Battery Reconditioner	Baby received quality is quite good 5 stars praise	Taobao Marketplace, a Chinese website for online shopping
altt flagship store	Battery Reconditioner	It works. It's good.	Taobao Marketplace, a Chinese website for online shopping
altt flagship store	Battery Reconditioner	Good quality, pretty good material	Taobao Marketplace, a Chinese website for online shopping
altt flagship store	Battery Reconditioner	Very good service, fast shipping, packaging intact, product looks very good, effective, satisfied with the purchase.	Taobao Marketplace, a Chinese website for online shopping

altt flagship store	Battery Reconditioner	🆒It's authentic, it's the same as the ones you buy in the store, it's easy and fast to buy online here, I'm assured of the quality, I'll be back!!.^◡^.	Taobao Marketplace, a Chinese website for online shopping
altt flagship store	Battery Reconditioner	It's pretty good, fast charging, and customer service is pretty good, satisfied.	Taobao Marketplace, a Chinese website for online shopping
altt flagship store	Battery Reconditioner	Compact and easy to use, cell phone power time are extended, good use	Taobao Marketplace, a Chinese website for online shopping
altt flagship store	Battery Reconditioner	Battery Repairer received, used a few days the effect is very good, my cell phone was fully charged can only be used less than half a day, the use of this repair device, after charging an hour will be able to use a day, the effect is magical!	Taobao Marketplace, a Chinese website for online shopping
altt flagship store	Battery Reconditioner	The logistics is very fast and the packaging is intact.	Taobao Marketplace, a Chinese website for online shopping
altt flagship store	Battery Reconditioner	The quality of this is really unexpected, the physical beauty, touch is very good, very recommended Oh~	Taobao Marketplace, a Chinese website for online shopping
altt flagship store	Battery Reconditioner	Charging speed: good👌	Taobao Marketplace, a Chinese website for online shopping
altt flagship store	Battery Reconditioner	Fast shipping, great repair, one hour more than before the repair, satisfied!	Taobao Marketplace, a Chinese website for online shopping
altt flagship store	Battery Reconditioner	house are old, battery life is lacking, looking forward to fixing it.	Taobao Marketplace, a Chinese website for online shopping
altt flagship store	Battery Reconditioner	Aftershutting down and charging for3hours and restartingas instructed, it took longer than usual to use, so it seems to be working.	Taobao Marketplace, a Chinese website for online shopping
altt flagship store	Battery Reconditioner	Fast shipping, good service, cell phone battery lasts longer than before	Taobao Marketplace, a Chinese website for online shopping
altt flagship store	Battery Reconditioner	the repair is good, the effect is powerful, now the battery is not so fast dead, before the full charge can only be used for two hours, now you can brush6to7hours of video. Dozens of dollars can extend the battery life, more cost-effective than changing the battery!	Taobao Marketplace, a Chinese website for online shopping
altt flagship store	Battery Reconditioner	slow, but extended battery life should be good too, good review.	Taobao Marketplace, a Chinese website for online shopping
altt flagship store	Battery Reconditioner	I bought it under the inquiry of seeing Collage in use, and his family used it with great results. Immediately asked for the link and is testing.	Taobao Marketplace, a Chinese website for online shopping
altt flagship store	Battery Reconditioner	Charging speed: average speed, didn't feel like it worked much, battery is still the same	Taobao Marketplace, a Chinese website for online shopping
altt flagship store	Battery Reconditioner	Charging is really slow, it takesabout10hours, I don't feel any effect, don't be fooled	Taobao Marketplace, a Chinese website for online shopping
altt flagship store	Battery Reconditioner	null	Taobao Marketplace, a Chinese website for online shopping
altt flagship store	Battery Reconditioner	Logistics is very fast, customer service reply promptly told the repair method, with a period of time should be able to see the repair effect.	Taobao Marketplace, a Chinese website for online shopping
altt flagship store	Battery Reconditioner	Good use, the old phone Apple8with3useno power, after using this repairer, now can almost watch fast5hours it, receive calls1day are no problem	Taobao Marketplace, a Chinese website for online shopping
altt flagship store	Battery Reconditioner	The automatic fix, just follow the instructions, works great and saves you from having to go to the repair store.	Taobao Marketplace, a Chinese website for online shopping
altt flagship store	Battery Reconditioner	fashion, compact and portable, now according to the instructions have begun to charge the experiment, I hope to be able to receive satisfactory results according to the instructions.	Taobao Marketplace, a Chinese website for online shopping
altt flagship store	Battery Reconditioner	This battery restorer is really good, after using it for a few days, it feels great. Charging speed is obviously much faster after repairing. Satisfied	Taobao Marketplace, a Chinese website for online shopping
altt flagship store	Battery Reconditioner	Repair effect is very good, this time do not need to change the battery, after repairing the phone with a longer time, the boss said often used to repair will be better.	Taobao Marketplace, a Chinese website for online shopping
altt flagship store	Battery Reconditioner	Genuine, buy with confidence, the price is fair, satisfied!	Taobao Marketplace, a Chinese website for online shopping
altt flagship store	Battery Reconditioner	As described, most charged devices can be repaired. Should increase battery life.	Taobao Marketplace, a Chinese website for online shopping
altt flagship store	Battery Reconditioner	Very nice.	Taobao Marketplace, a Chinese website for online shopping
altt flagship store	Battery Reconditioner	Charging speed: good	Taobao Marketplace, a Chinese website for online shopping
altt flagship store	Battery Reconditioner	Baby is good, great value, trying it out	Taobao Marketplace, a Chinese website for online shopping
altt flagship store	Battery Reconditioner	simulacrum	Taobao Marketplace, a Chinese website for online shopping
altt flagship store	Battery Reconditioner	Received it and used it, I hope it will help repair the cell phone battery and increase the power level	Taobao Marketplace, a Chinese website for online shopping
altt flagship store	Battery Reconditioner	Just received Unknown first use 5starsfor fast logistics	Taobao Marketplace, a Chinese website for online shopping

altt flagship store	Battery Reconditioner	Charging with cell phone repairer, the phone does not get hot, originally a few minutes of use in a fully charged state will consume the battery, after a few minutes of use with the repairer or a fully charged state	Taobao Marketplace, a Chinese website for online shopping
altt flagship store	Battery Reconditioner	You need to read the instructions carefully to use it, and the service is great!	Taobao Marketplace, a Chinese website for online shopping
altt flagship store	Battery Reconditioner	The goods have been received, fast logistics, good quality, very satisfied, give the store praise!	Taobao Marketplace, a Chinese website for online shopping
altt flagship store	Battery Reconditioner	Cell phone battery repairer received, logistics is very fast, the product is very good workmanship, used for a period of time, the effect is good.	Taobao Marketplace, a Chinese website for online shopping
altt flagship store	Battery Reconditioner	Received in perfect condition. Unpacked and started to try according to the instructions. Effectiveness, try a period of time after the follow-up evaluation.	Taobao Marketplace, a Chinese website for online shopping
altt flagship store	Battery Reconditioner	Started charging fast with it, after a few days of using it the charging slowed down, I feel the battery storage has improved, keep using it to see how it works out!	Taobao Marketplace, a Chinese website for online shopping
altt flagship store	Battery Reconditioner	Shipped quickly and well packaged for the right price!	Taobao Marketplace, a Chinese website for online shopping
altt flagship store	Battery Reconditioner	Baby received, the installation is relatively simple charging fast, long battery life, satisfied with	Taobao Marketplace, a Chinese website for online shopping
altt flagship store	Battery Reconditioner	pretty good	Taobao Marketplace, a Chinese website for online shopping
altt flagship store	Battery Reconditioner	Shipping was fast Packaging was good Charging was pretty fast	Taobao Marketplace, a Chinese website for online shopping
altt flagship store	Battery Reconditioner	A very pleasant shopping, charging the phone to try a few hours, repair effect is obvious	Taobao Marketplace, a Chinese website for online shopping
altt flagship store	Battery Reconditioner	Received the goods, small and exquisite, easy to use, look at the appearance of particularly good, after charging with this, the phone can be used to extend the time!	Taobao Marketplace, a Chinese website for online shopping
altt flagship store	Battery Reconditioner	The result is very good, the cell phone use time has become longer, very satisfied	Taobao Marketplace, a Chinese website for online shopping
altt flagship store	Battery Reconditioner	The phone has been used for three years, the battery is not durable, after the repair is much more durable, now you do not need to replace the phone, save a sum of money!	Taobao Marketplace, a Chinese website for online shopping
altt flagship store	Battery Reconditioner	The seller shipped super fast, the product is exactly the same as the business description, repair a few times, the phone than before the repair of more than half an hour, I believe that adhere to the use of the effect will be better!	Taobao Marketplace, a Chinese website for online shopping
altt flagship store	Battery Reconditioner	Baby has received, hurry to try to charge, I do not know how the effect, to be evaluated!	Taobao Marketplace, a Chinese website for online shopping
altt flagship store	Battery Reconditioner	days the effect is very satisfied, is-a cost-effective product.	Taobao Marketplace, a Chinese website for online shopping
altt flagship store	Battery Reconditioner	I've been using it since I received it, it lasts a long time and the phone runs much more comfortably.	Taobao Marketplace, a Chinese website for online shopping
altt flagship store	Battery Reconditioner	Baby received, the quality is good, it is worth recommending the purchase, five-star praise support	Taobao Marketplace, a Chinese website for online shopping
altt flagship store	Battery Reconditioner	I'll comment after I use it and give you a good review if it works.r.	Taobao Marketplace, a Chinese website for online shopping
altt flagship store	Battery Reconditioner	The stuff is oh so good, love it.	Taobao Marketplace, a Chinese website for online shopping
altt flagship store	Battery Reconditioner	It's really a lie.	Taobao Marketplace, a Chinese website for online shopping
altt flagship store	Battery Reconditioner	Waiting to use it for a while before evaluating it!	Taobao Marketplace, a Chinese website for online shopping
altt flagship store	Battery Reconditioner	Don't ever buy it, used it a couple times wai, no homework at all, garbage, garbage	Taobao Marketplace, a Chinese website for online shopping
altt flagship store	Battery Reconditioner	Merchants fast shipping, the product has been tried once, the effect of waiting for a few more times to follow up!	Taobao Marketplace, a Chinese website for online shopping
altt flagship store	Battery Reconditioner	Good quality, good value for money, bought the right one. Good review.	Taobao Marketplace, a Chinese website for online shopping
altt flagship store	Battery Reconditioner	Fast shipping logistics, packaging is perfect, the value is very high, feel value for money, customer service is also very patient and meticulous	Taobao Marketplace, a Chinese website for online shopping
altt flagship store	Battery Reconditioner	the charge can not be charged, with this battery repairer three hours(for the first time), unplugged after the boot has been to full power one hundred percent, very praise this product, solve the trouble, do not want to dismantle the cell phone to repair and replace the battery, after all, dismantling will hurt the phone Yuanqi, this product is according to theGBis the production of the national standard, I would like to tell you that there is a role in recommending the acquisition of the.	Taobao Marketplace, a Chinese website for online shopping
altt flagship store	Battery Reconditioner	Used for a period of time about a month or so, on the phone battery capacity and power drop has a very big improvement, is a godsend, not in vain the name of the godsend.	Taobao Marketplace, a Chinese website for online shopping
altt flagship store	Battery Reconditioner	The material is awesome, didn't let me down yo, very satisfied with the purchase!	Taobao Marketplace, a Chinese website for online shopping

altt flagship store	Battery Reconditioner	really can repair the same, before the loss of a point can not open the machine can be charged into the customer service attitude is good, there will not be able to reply in a timely manner, satisfied!	Taobao Marketplace, a Chinese website for online shopping
altt flagship store	Battery Reconditioner	Received the goods, not yet used	Taobao Marketplace, a Chinese website for online shopping
altt flagship store	Battery Reconditioner	Charging speed: pretty fast	Taobao Marketplace, a Chinese website for online shopping
altt flagship store	Battery Reconditioner	Received. Shipping was fast. Overall very satisfied with the workmanship. Material is awesome👍cheaper than brick and mortar. It's worth it.	Taobao Marketplace, a Chinese website for online shopping
altt flagship store	Battery Reconditioner	The quality is good, the price is cheap, the seller also fast delivery, good service, worth buying!	Taobao Marketplace, a Chinese website for online shopping
altt flagship store	Battery Reconditioner	The physical quality of the battery is very good, the packaging is sturdy, tried it for a few days🔋very useful	Taobao Marketplace, a Chinese website for online shopping
altt flagship store	Battery Reconditioner	The seller shipped and packed tightly, as described by customer service, satisfied	Taobao Marketplace, a Chinese website for online shopping
altt flagship store	Battery Reconditioner	Good, like, cost-effective, will come back often	Taobao Marketplace, a Chinese website for online shopping
altt flagship store	Battery Reconditioner	It was shipped quickly and packaged well, much better than expected for the price!	Taobao Marketplace, a Chinese website for online shopping
altt flagship store	Battery Reconditioner	Very nice, the material is very good, the quality can be	Taobao Marketplace, a Chinese website for online shopping
altt flagship store	Battery Reconditioner	Very good quality, well made, very sensitive, fast charging	Taobao Marketplace, a Chinese website for online shopping
altt flagship store	Battery Reconditioner	Baby arrived, charged twice, works great!	Taobao Marketplace, a Chinese website for online shopping
altt flagship store	Battery Reconditioner	Size fit, no scratches, very clear good review.	Taobao Marketplace, a Chinese website for online shopping
altt flagship store	Battery Reconditioner	Contact sensitivity: great item, great service!	Taobao Marketplace, a Chinese website for online shopping
altt flagship store	Battery Reconditioner	It's okay. I'll tell you after I use it.	Taobao Marketplace, a Chinese website for online shopping
altt flagship store	Battery Reconditioner	A very good shopping experience, attentive service, fast logistics, delivery and good attitude, praise!	Taobao Marketplace, a Chinese website for online shopping
altt flagship store	Battery Reconditioner	666	Taobao Marketplace, a Chinese website for online shopping
altt flagship store	Battery Reconditioner	Contact sensitivity: not bad	Taobao Marketplace, a Chinese website for online shopping
altt flagship store	Battery Reconditioner	Pingback: Can't charge my cell phone?	Taobao Marketplace, a Chinese website for online shopping
altt flagship store	Battery Reconditioner	off the Department of power consumption function, the pit ! ! ! !	Taobao Marketplace, a Chinese website for online shopping
altt flagship store	Battery Reconditioner	Goods received for inspection	Taobao Marketplace, a Chinese website for online shopping
altt flagship store	Battery Reconditioner	As described by the seller!	Taobao Marketplace, a Chinese website for online shopping
altt flagship store	Battery Reconditioner	My Apple8has not been used for a long time, the charging state can be turned on, a dial off on the powerless, used the store products to charge a few times useless, directly replace the battery, no use, threw away the utility cabinet!	Taobao Marketplace, a Chinese website for online shopping
altt flagship store	Battery Reconditioner	Used it for a few days and it didn't do much. The logistics is quite fast	Taobao Marketplace, a Chinese website for online shopping
altt flagship store	Battery Reconditioner	I'm using it, let's see how it works later. I hope it works.	Taobao Marketplace, a Chinese website for online shopping
altt flagship store	Battery Reconditioner	Not seeing the effects of the restoration that	Taobao Marketplace, a Chinese website for online shopping
altt flagship store	Battery Reconditioner	Terrible quality, broke after a few days of use……	Taobao Marketplace, a Chinese website for online shopping
altt flagship store	Battery Reconditioner	IQ cargo	Taobao Marketplace, a Chinese website for online shopping
altt flagship store	Battery Reconditioner	rubbish	Taobao Marketplace, a Chinese website for online shopping
altt flagship store	Battery Reconditioner	Baby received, logistics is very fast, the description is consistent, more satisfactory	Taobao Marketplace, a Chinese website for online shopping
altt flagship store	Battery Reconditioner	Think things are good, baby quality is very good, is genuine and the page description writtenbasic-consistent, the seller's customer service attitude from start to finish is very good, the express delivery speed is super fast	Taobao Marketplace, a Chinese website for online shopping
altt flagship store	Battery Reconditioner	Customer service is very good, there is no understanding of the place are also very quickly answered!	Taobao Marketplace, a Chinese website for online shopping
altt flagship store	Battery Reconditioner	Contact sensitivity:	Taobao Marketplace, a Chinese website for online shopping
altt flagship store	Battery Reconditioner	Contact sensitivity: not bad, the repair effect is not obvious, maybe I have to restore the factory settings a few more tries to give a favorable review	Taobao Marketplace, a Chinese website for online shopping
altt flagship store	Battery Reconditioner	Complete and utter IQ tax!	Taobao Marketplace, a Chinese website for online shopping

altt flagship store	Battery Reconditioner	It's not working.	Taobao Marketplace, a Chinese website for online shopping
altt flagship store	Battery Reconditioner	increased dramatically, the original day two punch has become a three-charge, not accustomed to bad reviews, counting the general bar	Taobao Marketplace, a Chinese website for online shopping
altt flagship store	Battery Reconditioner	This cell phone charging repairer of yours, does not hold Apple phone charging oh?	Taobao Marketplace, a Chinese website for online shopping
altt flagship store	Battery Reconditioner	Contact sensitivity: fast Appearance material: good Charging speed: fast	Taobao Marketplace, a Chinese website for online shopping
altt flagship store	Battery Reconditioner	After receiving it, I charged it once and felt no change in the phone!	Taobao Marketplace, a Chinese website for online shopping
altt flagship store	Battery Reconditioner	Received the goods very quickly, the service is also good, try the cell phone battery maintenance, now trying to repair the stereo lithium batteries, looking forward to the effect of good will also come!	Taobao Marketplace, a Chinese website for online shopping
altt flagship store	Battery Reconditioner	Being repaired, see the display jumping numbers, tried several times the phone has effect, looking forward to the	Taobao Marketplace, a Chinese website for online shopping
altt flagship store	Battery Reconditioner	Packaging is good, the courier is also very fast, good quality, enthusiastic service, good, the repairer is very good, with a longer time than before the	Taobao Marketplace, a Chinese website for online shopping
altt flagship store	Battery Reconditioner	Goods have been received, logistics is also very fast, very satisfied with a shopping, five stars!	Taobao Marketplace, a Chinese website for online shopping
altt flagship store	Battery Reconditioner	using it15days and it's not working	Taobao Marketplace, a Chinese website for online shopping
altt flagship store	Battery Reconditioner	I don't know if it works or not.	Taobao Marketplace, a Chinese website for online shopping
altt flagship store	Battery Reconditioner	Don't be fooled I've been using it4doesn't work at all I've paid my IQ tax for	Taobao Marketplace, a Chinese website for online shopping
altt flagship store	Battery Reconditioner	Charge old slow, but so full than before to use longer, the store also has instructions, in general it is okay	Taobao Marketplace, a Chinese website for online shopping
altt flagship store	Battery Reconditioner	It's not as good as it sounds.	Taobao Marketplace, a Chinese website for online shopping
altt flagship store	Battery Reconditioner	It doesn't work at all, it's a scam, don't buy it!	Taobao Marketplace, a Chinese website for online shopping
altt flagship store	Battery Reconditioner	of power in about60minutesbefore, but now it lastsabout90minutes	Taobao Marketplace, a Chinese website for online shopping
altt flagship store	Battery Reconditioner	After trying the phone according to the steps in the manual, the phone runs soothingly, and I believe that the effect will be basically the same as the description after charging5-8timesa monthSmartphones are laggy and slow after a long time, the battery does not last is a common phenomenon, with this can be used for a long time!	Taobao Marketplace, a Chinese website for online shopping
altt flagship store	Battery Reconditioner	In use	Taobao Marketplace, a Chinese website for online shopping
altt flagship store	Battery Reconditioner	Average! Charging is slow! All major video platforms are advertised by one person!	Taobao Marketplace, a Chinese website for online shopping
altt flagship store	Battery Reconditioner	It doesn't work , IQ tax	Taobao Marketplace, a Chinese website for online shopping
altt flagship store	Battery Reconditioner	The repairer has been here for a few days, I used it on my home phone as soon as I received it, and now it's already lasting longer than before!	Taobao Marketplace, a Chinese website for online shopping
altt flagship store	Battery Reconditioner	Contact sensitivity: good Appearance material: good Charging speed: good	Taobao Marketplace, a Chinese website for online shopping
altt flagship store	Battery Reconditioner	It works, the battery doesn't die as fast after using it	Taobao Marketplace, a Chinese website for online shopping
altt flagship store	Battery Reconditioner	I just started using it now, and will follow up after a while to see the results.	Taobao Marketplace, a Chinese website for online shopping
altt flagship store	Battery Reconditioner	So far, so good.	Taobao Marketplace, a Chinese website for online shopping
altt flagship store	Battery Reconditioner	have no effect	Taobao Marketplace, a Chinese website for online shopping
altt flagship store	Battery Reconditioner	Good quality, buy with confidence	Taobao Marketplace, a Chinese website for online shopping
altt flagship store	Battery Reconditioner	IQ tax	Taobao Marketplace, a Chinese website for online shopping
altt flagship store	Battery Reconditioner	Contact sensitivity: good! Appearance material: good! Charging speed: I received it in the morning and will add more when I try it in the evening.	Taobao Marketplace, a Chinese website for online shopping
altt flagship store	Battery Reconditioner	It's not even real.	Taobao Marketplace, a Chinese website for online shopping
altt flagship store	Battery Reconditioner	using it for a couple of days. It doesn't do anything for you	Taobao Marketplace, a Chinese website for online shopping
altt flagship store	Battery Reconditioner	I haven't used it yet, I'm not sure. After all these years of shopping, I'm surprised that I bought it only after careful consideration. The boss has a pretty good grasp of human nature and understands the IQ tax rate, so I admit that I'm trapped! Of course, I expect it to work, but if it doesn't, I'll admit defeat and pay the tuition once.	Taobao Marketplace, a Chinese website for online shopping

altt flagship store	Battery Reconditioner	Contact sensitivity: good on sensitivity!	Taobao Marketplace, a Chinese website for online shopping
altt flagship store	Battery Reconditioner	It's not much use.	Taobao Marketplace, a Chinese website for online shopping
altt flagship store	Battery Reconditioner	Good! All five stars!	Taobao Marketplace, a Chinese website for online shopping
altt flagship store	Battery Reconditioner	Logistics is very strong, not used, used to good reviews!	Taobao Marketplace, a Chinese website for online shopping
altt flagship store	Battery Reconditioner	Paid the IQ tax once.	Taobao Marketplace, a Chinese website for online shopping
altt flagship store	Battery Reconditioner	It's good quality.	Taobao Marketplace, a Chinese website for online shopping
altt flagship store	Battery Reconditioner	The effect is not very obvious, use it for a while and then evaluate.	Taobao Marketplace, a Chinese website for online shopping
altt flagship store	Battery Reconditioner	I've been using it for a while and it doesn't work, it's a rip-off	Taobao Marketplace, a Chinese website for online shopping
altt flagship store	Battery Reconditioner	It feels OK and is in use.	Taobao Marketplace, a Chinese website for online shopping
altt flagship store	Battery Reconditioner	It's in use, just sold on, and the cell phone battery isn't responding yet.	Taobao Marketplace, a Chinese website for online shopping
altt flagship store	Battery Reconditioner	good as the picture described Satisfied	Taobao Marketplace, a Chinese website for online shopping
altt flagship store	Battery Reconditioner	I received it early, tried it for a week, still testing it.	Taobao Marketplace, a Chinese website for online shopping
altt flagship store	Battery Reconditioner	IQ tax!!! Hex is broken!	Taobao Marketplace, a Chinese website for online shopping
altt flagship store	Battery Reconditioner	Received okay worth the purchase. Give you a good review after use.	Taobao Marketplace, a Chinese website for online shopping
altt flagship store	Battery Reconditioner	Contact sensitivity: very good Appearance material: good-looking Charging speed: OK	Taobao Marketplace, a Chinese website for online shopping
altt flagship store	Battery Reconditioner	First of all, after using it, charging is very slow, and the current data has been jumping, and after using it for almost a week and also charging for more than three hours each time as required, the result is that the battery power 62% before, and a week later it is still 62% not at all repairing effect.	Taobao Marketplace, a Chinese website for online shopping
altt flagship store	Battery Reconditioner	It doesn't work. Don't fall for it.	Taobao Marketplace, a Chinese website for online shopping
altt flagship store	Battery Reconditioner	Just used it! Haven't seen results yet!	Taobao Marketplace, a Chinese website for online shopping
altt flagship store	Battery Reconditioner	I don't know the effect yet, but I'll give you a good review.	Taobao Marketplace, a Chinese website for online shopping
altt flagship store	Battery Reconditioner	It's okay. It's fast.	Taobao Marketplace, a Chinese website for online shopping
altt flagship store	Battery Reconditioner	Good service, fast logistics, the product works well, just don't know how long it will last?	Taobao Marketplace, a Chinese website for online shopping
altt flagship store	Battery Reconditioner	Good review.	Taobao Marketplace, a Chinese website for online shopping
altt flagship store	Battery Reconditioner	It's good quality.	Taobao Marketplace, a Chinese website for online shopping
altt flagship store	Battery Reconditioner	serviceable	Taobao Marketplace, a Chinese website for online shopping
altt flagship store	Battery Reconditioner	Very good quality, feel free to use it	Taobao Marketplace, a Chinese website for online shopping
altt flagship store	Battery Reconditioner	It doesn't work at all. The light was out two days after I got it.	Taobao Marketplace, a Chinese website for online shopping
altt flagship store	Battery Reconditioner	Use it for a while and then evaluate	Taobao Marketplace, a Chinese website for online shopping
altt flagship store	Battery Reconditioner	Not good Scam IQ tax!	Taobao Marketplace, a Chinese website for online shopping
altt flagship store	Battery Reconditioner	It works very well, I hope everyone will buy it	Taobao Marketplace, a Chinese website for online shopping
altt flagship store	Battery Reconditioner	Not a very obvious fix for cell phones	Taobao Marketplace, a Chinese website for online shopping
altt flagship store	Battery Reconditioner	Appearance Material: Follow-up evaluation after use	Taobao Marketplace, a Chinese website for online shopping
altt flagship store	Battery Reconditioner	favorable criticism	Taobao Marketplace, a Chinese website for online shopping
altt flagship store	Battery Reconditioner	technology, plugged in to show the current and voltage changes, repaired and rebooted, is indeed more durable than the previous	Taobao Marketplace, a Chinese website for online shopping
altt flagship store	Battery Reconditioner	I'm not sure how well it works, I'll know after the restoration, it looks like it should be fine!	Taobao Marketplace, a Chinese website for online shopping
altt flagship store	Battery Reconditioner	Whoever uses it knows! The numbers on this item jump around one by one, ㊖results……check out the pictures	Taobao Marketplace, a Chinese website for online shopping

altt flagship store	Battery Reconditioner	Used for a period of time, on the bad battery is not useful, I have a cell phone can not turn on the machine is not useful, on the electric capacity of a little less or a little bit of use, myipadcan only be plugged in the power supply in order to turn on the power supply, with this charging a few times, can leave the power supply to turn on a few minutes, is also considered to be an improvement, positive feedback, hope that more than a few times will be a little bit better.	Taobao Marketplace, a Chinese website for online shopping
altt flagship store	Battery Reconditioner	Received to try a few days feel the quality is very good, fast charging, interface stable, quite convenient to use, the price is also affordable.	Taobao Marketplace, a Chinese website for online shopping
altt flagship store	Battery Reconditioner	home is just right for use, never need to change the battery again.	Taobao Marketplace, a Chinese website for online shopping
altt flagship store	Battery Reconditioner	Absolutely positive feedback, the store public cell phone vacation starvation, electricity can not be charged into, this immediately can be charged into the	Taobao Marketplace, a Chinese website for online shopping
altt flagship store	Battery Reconditioner	Fake! Doesn't match the description at all, almost charged up my cell phone	Taobao Marketplace, a Chinese website for online shopping
altt flagship store	Battery Reconditioner	effect, use a period of time, see the effect and then follow up!	Taobao Marketplace, a Chinese website for online shopping
altt flagship store	Battery Reconditioner	It's great to try it out, a few old phones and tablets are fully charged, hopefully a few more times and these old phones will be durable again!	Taobao Marketplace, a Chinese website for online shopping
altt flagship store	Battery Reconditioner	Performance is great and after two uses, the phone doesn't heat up as much as it used to, obviously.	Taobao Marketplace, a Chinese website for online shopping
altt flagship store	Battery Reconditioner	The effect is not obvious	Taobao Marketplace, a Chinese website for online shopping
altt flagship store	Battery Reconditioner	It's not much use.	Taobao Marketplace, a Chinese website for online shopping
altt flagship store	Battery Reconditioner	It doesn't work very well.	Taobao Marketplace, a Chinese website for online shopping
altt flagship store	Battery Reconditioner	no good	Taobao Marketplace, a Chinese website for online shopping
altt flagship store	Battery Reconditioner	Charging speed: fast	Taobao Marketplace, a Chinese website for online shopping
altt flagship store	Battery Reconditioner	It works.	Taobao Marketplace, a Chinese website for online shopping
altt flagship store	Battery Reconditioner	Unclear descriptions to confuse customers	Taobao Marketplace, a Chinese website for online shopping
altt flagship store	Battery Reconditioner	Theoretically it is feasible, cell phone battery repairer use two days, there is an effect, charging, current voltage changes, looking forward to a week after the effect. The store shipped quickly, logistics is also fast, praise!	Taobao Marketplace, a Chinese website for online shopping
altt flagship store	Battery Reconditioner	light and its middle side will have VandAvalue display, the quality looks good, the appearance of the workmanship is not sloppy there is no flaws, is to use the repair device will become a slow charging, if you want to test the effect of the pre a little more time	Taobao Marketplace, a Chinese website for online shopping
altt flagship store	Battery Reconditioner	Not bad, in use, do not know if it is possible to repair the battery it, to see the effect and then follow up!	Taobao Marketplace, a Chinese website for online shopping
altt flagship store	Battery Reconditioner	Used it for a few days, didn't notice any difference, charge - for a month and see.	Taobao Marketplace, a Chinese website for online shopping
altt flagship store	Battery Reconditioner	terrific	Taobao Marketplace, a Chinese website for online shopping
altt flagship store	Battery Reconditioner	I hope it works well!	Taobao Marketplace, a Chinese website for online shopping
altt flagship store	Battery Reconditioner	Looking forward to the results	Taobao Marketplace, a Chinese website for online shopping
altt flagship store	Battery Reconditioner	Received delivery in the afternoon and will apply it in the evening	Taobao Marketplace, a Chinese website for online shopping
altt flagship store	Battery Reconditioner	The baby was received in perfect condition.	Taobao Marketplace, a Chinese website for online shopping
altt flagship store	Battery Reconditioner	I received it, I believe it should be good.	Taobao Marketplace, a Chinese website for online shopping
altt flagship store	Battery Reconditioner	Still watching……	Taobao Marketplace, a Chinese website for online shopping
altt flagship store	Battery Reconditioner	Tried it at home for days and it didn't work at all. It's a total rip-off.	Taobao Marketplace, a Chinese website for online shopping
altt flagship store	Battery Reconditioner	Used it three times didn't see any results	Taobao Marketplace, a Chinese website for online shopping
altt flagship store	Battery Reconditioner	The effect is a little bit, just average.	Taobao Marketplace, a Chinese website for online shopping
altt flagship store	Battery Reconditioner	Today received the goods then opened to try, two charging not into the power of the old cell phone has a charging into the power can be normal power on and use, the other cell phone may be a battery problem has been charging not into the power, indicating that this cell phone battery repairer is very good👍! Worthy of praise and promotion.	Taobao Marketplace, a Chinese website for online shopping

altt flagship store	Battery Reconditioner	This is still useful.	Taobao Marketplace, a Chinese website for online shopping
altt flagship store	Battery Reconditioner	Don't be fooled. I've been using it for a month and doesn't work.	Taobao Marketplace, a Chinese website for online shopping
altt flagship store	Battery Reconditioner	I've been using this product for two days, and the digital display, it's broken, and this product sucks.	Taobao Marketplace, a Chinese website for online shopping
altt flagship store	Battery Reconditioner	It's okay to use, as for the effect in use, later to follow up the evaluation	Taobao Marketplace, a Chinese website for online shopping
altt flagship store	Battery Reconditioner	First time buyer, don't know if it's good yet, looks good quality	Taobao Marketplace, a Chinese website for online shopping
altt flagship store	Battery Reconditioner	Disappointed that you have to restore factory settings for it to work, and it's still only30%effective. Right to use as a charging head.	Taobao Marketplace, a Chinese website for online shopping
altt flagship store	Battery Reconditioner	Very good Contact sensitivity: very good	Taobao Marketplace, a Chinese website for online shopping
altt flagship store	Battery Reconditioner	I'll comment in a few days, just got it today	Taobao Marketplace, a Chinese website for online shopping
altt flagship store	Battery Reconditioner	not very good	Taobao Marketplace, a Chinese website for online shopping
altt flagship store	Battery Reconditioner	The item has been received, I opened it and tried it when I received it, and it didn't work well, not as advertised......!	Taobao Marketplace, a Chinese website for online shopping
altt flagship store	Battery Reconditioner	It didn't work.	Taobao Marketplace, a Chinese website for online shopping
altt flagship store	Battery Reconditioner	It's not bad, an extra charger plug was given	Taobao Marketplace, a Chinese website for online shopping
altt flagship store	Battery Reconditioner	Charged the repair once and it was slow, not sure how well it works yet.	Taobao Marketplace, a Chinese website for online shopping
altt flagship store	Battery Reconditioner	It doesn't feel useful. It's an IQ tax.	Taobao Marketplace, a Chinese website for online shopping
altt flagship store	Battery Reconditioner	Got it. Good.	Taobao Marketplace, a Chinese website for online shopping
altt flagship store	Battery Reconditioner	After charging3hours, it feels like the power consumption is much slower than before	Taobao Marketplace, a Chinese website for online shopping
altt flagship store	Battery Reconditioner	It's okay, use it for a while and then evaluate	Taobao Marketplace, a Chinese website for online shopping
altt flagship store	Battery Reconditioner	The goods have been received, the logistics is very fast	Taobao Marketplace, a Chinese website for online shopping
altt flagship store	Battery Reconditioner	favorable criticism	Taobao Marketplace, a Chinese website for online shopping
altt flagship store	Battery Reconditioner	Customer service is very good shipping is also very fast, the baby quality is very good Oh, it is very easy to use, ten days later to evaluate.	Taobao Marketplace, a Chinese website for online shopping
altt flagship store	Battery Reconditioner	Used it for a while and it didn't help at all. It's a lie.	Taobao Marketplace, a Chinese website for online shopping
altt flagship store	Battery Reconditioner	(of an unmarried couple) be close	Taobao Marketplace, a Chinese website for online shopping
altt flagship store	Battery Reconditioner	The stuff looks ok, I'll follow up after I use it!	Taobao Marketplace, a Chinese website for online shopping
altt flagship store	Battery Reconditioner	It has a restorative effect, matches the description and is very satisfactory.	Taobao Marketplace, a Chinese website for online shopping
altt flagship store	Battery Reconditioner	an excellent	Taobao Marketplace, a Chinese website for online shopping
altt flagship store	Battery Reconditioner	Contact sensitivity: fast Exterior material: nice Charging speed: fast	Taobao Marketplace, a Chinese website for online shopping
altt flagship store	Battery Reconditioner	Don't be fooled, it doesn't work at all (I rushed it and plugged it in for a day with no power at all)	Taobao Marketplace, a Chinese website for online shopping
altt flagship store	Battery Reconditioner	Baby should be great and I'll buy it again next time I need it.	Taobao Marketplace, a Chinese website for online shopping
altt flagship store	Battery Reconditioner	Good quality👍👍👍!	Taobao Marketplace, a Chinese website for online shopping
altt flagship store	Battery Reconditioner	Charging speed: It doesn't seem to work.	Taobao Marketplace, a Chinese website for online shopping
altt flagship store	Battery Reconditioner	Baby is worth having and trustworthy	Taobao Marketplace, a Chinese website for online shopping
altt flagship store	Battery Reconditioner	I wonder if it's an IQ tax?	Taobao Marketplace, a Chinese website for online shopping
altt flagship store	Battery Reconditioner	The goods have been received.	Taobao Marketplace, a Chinese website for online shopping
altt flagship store	Battery Reconditioner	Regardless of the fact that it can't fix the phone battery, I've been charging it for three days now and it won't charge at all and won't turn on.	Taobao Marketplace, a Chinese website for online shopping
altt flagship store	Battery Reconditioner	I don't see the effect.	Taobao Marketplace, a Chinese website for online shopping
altt flagship store	Battery Reconditioner	Received it very quickly after ordering, kudos to the merchant for their service.	Taobao Marketplace, a Chinese website for online shopping
altt flagship store	Battery Reconditioner	Seller shipped promptly and courier delivered to home. Good review!	Taobao Marketplace, a Chinese website for online shopping
altt flagship store	Battery Reconditioner	The baby was received in perfect condition.	Taobao Marketplace, a Chinese website for online shopping

altt flagship store	Battery Reconditioner	Is it good? Use it for a while before evaluating	Taobao Marketplace, a Chinese website for online shopping
altt flagship store	Battery Reconditioner	👌	Taobao Marketplace, a Chinese website for online shopping
altt flagship store	Battery Reconditioner	Quite good looking good, charging with voltage display, do not know with a period of time really will not repair the battery, but hopefully it can, can only try a period of time to see.	Taobao Marketplace, a Chinese website for online shopping
altt flagship store	Battery Reconditioner	Five out of five full praise.	Taobao Marketplace, a Chinese website for online shopping
altt flagship store	Battery Reconditioner	It is worth recommending the purchase.	Taobao Marketplace, a Chinese website for online shopping
altt flagship store	Battery Reconditioner	I've been using it for three months and it's not working.	Taobao Marketplace, a Chinese website for online shopping
altt flagship store	Battery Reconditioner	Goods have been received, charged once, feel and the original punch speed is almost the same, can repair the battery power is not yet known, first habitual praise.	Taobao Marketplace, a Chinese website for online shopping
altt flagship store	Battery Reconditioner	Looking forward to the results	Taobao Marketplace, a Chinese website for online shopping
altt flagship store	Battery Reconditioner	not work, the holiday also do not notify a little, obviously the goods have arrived at the outlets, prefer not to notify!notto	Taobao Marketplace, a Chinese website for online shopping
altt flagship store	Battery Reconditioner	not bad	Taobao Marketplace, a Chinese website for online shopping
altt flagship store	Battery Reconditioner	It's good quality.	Taobao Marketplace, a Chinese website for online shopping
altt flagship store	Battery Reconditioner	Looks good, use it for a while and see if it's any good.	Taobao Marketplace, a Chinese website for online shopping
altt flagship store	Battery Reconditioner	Thanks to the boss also sent a charging head, charging very quickly, have battery repair effect, worth buying!	Taobao Marketplace, a Chinese website for online shopping
altt flagship store	Battery Reconditioner	Turns out it's all an IQ tax.	Taobao Marketplace, a Chinese website for online shopping
altt flagship store	Battery Reconditioner	I haven't used it yet, I don't know how it is.	Taobao Marketplace, a Chinese website for online shopping
altt flagship store	Battery Reconditioner	It's not working.	Taobao Marketplace, a Chinese website for online shopping
altt flagship store	Battery Reconditioner	rare	Taobao Marketplace, a Chinese website for online shopping
altt flagship store	Battery Reconditioner	I haven't used it yet, so I'll give it a good review first!	Taobao Marketplace, a Chinese website for online shopping
altt flagship store	Battery Reconditioner	I've been using it for a while now and it's really good! I used to have to charge my phone after half a day, but now I can use it for a day. Suggested shutdown fix is the best, there's a noticeable improvement.	Taobao Marketplace, a Chinese website for online shopping
altt flagship store	Battery Reconditioner	The battery is getting less and less durable, the phone is not durable after a month or two, now with this magic tool can finally take good care of my phone battery!	Taobao Marketplace, a Chinese website for online shopping
altt flagship store	Battery Reconditioner	Buy this and try it. See my follow up review	Taobao Marketplace, a Chinese website for online shopping
altt flagship store	Battery Reconditioner	Baby received, used it once didn't notice any noticeable effect!	Taobao Marketplace, a Chinese website for online shopping
altt flagship store	Battery Reconditioner	Fine workmanship quality is very good, very good, worth buying.	Taobao Marketplace, a Chinese website for online shopping
altt flagship store	Battery Reconditioner	Works well, not too expensive, and continues to be used	Taobao Marketplace, a Chinese website for online shopping
altt flagship store	Battery Reconditioner	Nice material! Looking forward to the effect! Adding later. Good review!	Taobao Marketplace, a Chinese website for online shopping
altt flagship store	Battery Reconditioner	I don't know if it will fix the battery yet, but the exterior design is beautiful.	Taobao Marketplace, a Chinese website for online shopping
altt flagship store	Battery Reconditioner	The battery restorer was received, but there is no instruction manual, I don't know if it is a negligence of the store, I hope to arrange it as soon as possible, even if the electronic file is OK. Otherwise, do not know how to use, in case of problems, do not know how to deal with. Thanks to the store.	Taobao Marketplace, a Chinese website for online shopping
altt flagship store	Battery Reconditioner	Not yet used	Taobao Marketplace, a Chinese website for online shopping
altt flagship store	Battery Reconditioner	Contact sensitivity:, high Appearance material: good Charging speed: slow	Taobao Marketplace, a Chinese website for online shopping
altt flagship store	Battery Reconditioner	(of an unmarried couple) be close	Taobao Marketplace, a Chinese website for online shopping
altt flagship store	Battery Reconditioner	Can't see any special features😎	Taobao Marketplace, a Chinese website for online shopping
altt flagship store	Battery Reconditioner	Also used it for a while, the downside is that it's slow to charge, the upside is that it lasts longer than before, overall it's not too bad	Taobao Marketplace, a Chinese website for online shopping
altt flagship store	Battery Reconditioner	*Use all of them, scammy stuff!	Taobao Marketplace, a Chinese website for online shopping
altt flagship store	Battery Reconditioner	Contact Sensitivity: Sensitive and fast contact Appearance Material: Beautiful and aesthetic small and convenient Charging Speed: Fast charging speed	Taobao Marketplace, a Chinese website for online shopping

altt flagship store	Battery Reconditioner	All fives._x005f_x0001_ good	Taobao Marketplace, a Chinese website for online shopping
altt flagship store	Battery Reconditioner	Logistics was fast, workmanship was pretty good, now charging as required.	Taobao Marketplace, a Chinese website for online shopping
altt flagship store	Battery Reconditioner	Really super like it, very good quality, as described by the seller!	Taobao Marketplace, a Chinese website for online shopping
altt flagship store	Battery Reconditioner	Contact sensitivity: very good Appearance material: fine Charging speed: suitable	Taobao Marketplace, a Chinese website for online shopping
altt flagship store	Battery Reconditioner	Satisfied with the positive reviews! Use it for a while first and then evaluate	Taobao Marketplace, a Chinese website for online shopping
altt flagship store	Battery Reconditioner	Logistics is fast! The baby arrived and the repair and maintenance is great! Effective! Quality is very good! Cell phones and tablets can be! Very satisfied! Everyone is well assured to recommend the purchase! Five stars praise!	Taobao Marketplace, a Chinese website for online shopping
altt flagship store	Battery Reconditioner	Contact sensitivity: good Appearance material: good Charging speed: did not try it out. After reading the manual, I think it's a bit complicated, and I have to restore the factory settings! I'll try it again when I'm on spring break. I'll try it again when I'm on Spring Break.	Taobao Marketplace, a Chinese website for online shopping
altt flagship store	Battery Reconditioner	it for half a month, I feel that the boot and running speed has improved, and the duration of dirty time has also increased.	Taobao Marketplace, a Chinese website for online shopping
altt flagship store	Battery Reconditioner	Charging speed: received with, charging good fast, worth buying👍	Taobao Marketplace, a Chinese website for online shopping
altt flagship store	Battery Reconditioner	I use it for a while, I hope it's not an IQ tax, and I'll follow up later.	Taobao Marketplace, a Chinese website for online shopping
altt flagship store	Battery Reconditioner	Thanks to the seller's sincere service, the purchased item has arrived. Good quality, easy logistics and fast delivery. Must give a nod of praise. The seller's customer service is professional and patient and detailed introduction, giving people a home away from home. Must five-star praise !!!!	Taobao Marketplace, a Chinese website for online shopping
altt flagship store	Battery Reconditioner	Waiting for days for express delivery.	Taobao Marketplace, a Chinese website for online shopping
altt flagship store	Battery Reconditioner	After using it for a few days, the phone doesn't get hot anymore and the battery life is longer, which is good.	Taobao Marketplace, a Chinese website for online shopping
altt flagship store	Battery Reconditioner	favorable criticism	Taobao Marketplace, a Chinese website for online shopping
altt flagship store	Battery Reconditioner	The effect is not obvious	Taobao Marketplace, a Chinese website for online shopping
altt flagship store	Battery Reconditioner	pretty good	Taobao Marketplace, a Chinese website for online shopping
altt flagship store	Battery Reconditioner	Look at the effect, according to the business operation requirements, adhere to two months should be able to achieve the business description!	Taobao Marketplace, a Chinese website for online shopping
altt flagship store	Battery Reconditioner	desirable	Taobao Marketplace, a Chinese website for online shopping
altt flagship store	Battery Reconditioner	Logistics and shipping are very fast, also study this specialty, the theory is really good for the battery, compared with the replacement of the battery is very affordable!	Taobao Marketplace, a Chinese website for online shopping
altt flagship store	Battery Reconditioner	Quality is very good, exactly the same as the picture, details of the workmanship is also particularly good, very like, very much to my liking	Taobao Marketplace, a Chinese website for online shopping
altt flagship store	Battery Reconditioner	link to buy it, it really works, before my friend had to charge it twice a day, but now it only needs to be charged once.	Taobao Marketplace, a Chinese website for online shopping
altt flagship store	Battery Reconditioner	Shipping is fast. Logistics was fast too. The stuff is good. Practical. Will come back to buy. Satisfied	Taobao Marketplace, a Chinese website for online shopping
altt flagship store	Battery Reconditioner	Great fixer, easy to use, plug and play, full and stop.	Taobao Marketplace, a Chinese website for online shopping
altt flagship store	Battery Reconditioner	The feeling of being fooled again, after using the battery of their cell phone is still confused, I do not know how much help this kind of tiresome operation to improve the performance of the cell phone battery in the end!	Taobao Marketplace, a Chinese website for online shopping
altt flagship store	Battery Reconditioner	Charger received, works well, I wish the merchant business is prosperous, smooth sailing, and more to the next level.	Taobao Marketplace, a Chinese website for online shopping
altt flagship store	Battery Reconditioner	Fast shipping, good packaging, good quality, cost-effective, worth buying very satisfied!	Taobao Marketplace, a Chinese website for online shopping
altt flagship store	Battery Reconditioner	Big brand, with a very assured, the quality is very good, in time for the event to buy very cost,customer service is really very good Oh ~!	Taobao Marketplace, a Chinese website for online shopping
altt flagship store	Battery Reconditioner	Can see the current level, the repair is not bad, fast charging speed	Taobao Marketplace, a Chinese website for online shopping
altt flagship store	Battery Reconditioner	Baby received and matches the picture. I hope it can be as ideal as the introduction. Customer service is very good, there will not be a quick answer to the answer	Taobao Marketplace, a Chinese website for online shopping
altt flagship store	Battery Reconditioner	Good quality, affordable price, fast shipping, repair should be fine	Taobao Marketplace, a Chinese website for online shopping

altt flagship store	Battery Reconditioner	I couldn't wait to open it when it arrived, and it really didn't let me down, the quality is especially good, and the value is also quite high	Taobao Marketplace, a Chinese website for online shopping
altt flagship store	Battery Reconditioner	Very durable Charging is also very good and economical	Taobao Marketplace, a Chinese website for online shopping
altt flagship store	Battery Reconditioner	True to its name, it works well for battery reconditioning purposes	Taobao Marketplace, a Chinese website for online shopping
altt flagship store	Battery Reconditioner	Watching friends use it and ask for a link to buy it, it's really practical, not an IQ tax, it's pretty awesome	Taobao Marketplace, a Chinese website for online shopping
altt flagship store	Battery Reconditioner	Packed tightly, logistics is very fast, fine workmanship, material is very good, satisfied with the	Taobao Marketplace, a Chinese website for online shopping
altt flagship store	Battery Reconditioner	Works great, good quality, good value for money	Taobao Marketplace, a Chinese website for online shopping
altt flagship store	Battery Reconditioner	Baby received, the product is basically consistent with the description, good business service, fast delivery, logistics is also strong, five praise!	Taobao Marketplace, a Chinese website for online shopping
altt flagship store	Battery Reconditioner	The merchant shipped very quickly!Packed well without damage!Customer service is very enthusiastic!	Taobao Marketplace, a Chinese website for online shopping
altt flagship store	Battery Reconditioner	Charged up, has been changing numbers, very technological, should be good, not good then chase evaluation	Taobao Marketplace, a Chinese website for online shopping
altt flagship store	Battery Reconditioner	Shipping is fast, the charge restorer is compact and looks good, 5 stars first(not practical then follow up review)	Taobao Marketplace, a Chinese website for online shopping
altt flagship store	Battery Reconditioner	Tightly packaged, inexpensive, very satisfied	Taobao Marketplace, a Chinese website for online shopping
altt flagship store	Battery Reconditioner	Shipping was fast, stuff was well packaged, great price, I'm happy with👍and will repurchase	Taobao Marketplace, a Chinese website for online shopping
altt flagship store	Battery Reconditioner	Excellent workmanship of electronic products, I really think that do not buy those cheap defective products for the sake of cheap, very damaging cell phone	Taobao Marketplace, a Chinese website for online shopping
altt flagship store	Battery Reconditioner	it's good, it does last longer than it did before the restoration	Taobao Marketplace, a Chinese website for online shopping
altt flagship store	Battery Reconditioner	Logistics is very fast, the product began to use, not bad!	Taobao Marketplace, a Chinese website for online shopping
altt flagship store	Battery Reconditioner	The goods have arrived, with a good well, worth buying!	Taobao Marketplace, a Chinese website for online shopping
altt flagship store	Battery Reconditioner	The color looks pretty high and techy, should be good	Taobao Marketplace, a Chinese website for online shopping
altt flagship store	Battery Reconditioner	Your merchants received the goods, yesterday tried to feel some effect, high-tech products, there is always a process of adaptation, the next will be in accordance with the instructions of the program on the virtual power of the product for a period of time the battery is slowly repaired	Taobao Marketplace, a Chinese website for online shopping
altt flagship store	Battery Reconditioner	Very satisfied, it's genuine, well restored, just like the original, complete accessories, recommended to buy🌹	Taobao Marketplace, a Chinese website for online shopping
altt flagship store	Battery Reconditioner	The quality is great, the details are well done and it's easy to use.	Taobao Marketplace, a Chinese website for online shopping
altt flagship store	Battery Reconditioner	The quality is quite good, the delivery is fast, really good, satisfied!	Taobao Marketplace, a Chinese website for online shopping
altt flagship store	Battery Reconditioner	You can clearly see the amount of current, the repair is stable and very convenient	Taobao Marketplace, a Chinese website for online shopping
altt flagship store	Battery Reconditioner	good, ready to use for a period of time, hope to repair the phone battery	Taobao Marketplace, a Chinese website for online shopping
altt flagship store	Battery Reconditioner	recommended, when the experience was good, is too expensive, looking for the same model searched, the price is not expensive, the arrival is also very fast, it is genuine, pretty awesome!	Taobao Marketplace, a Chinese website for online shopping
altt flagship store	Battery Reconditioner	The first time to buy online, but also look at the evaluation before buying, the quality is not bad, but also very cheap, the need for friends can rest assured to buy!	Taobao Marketplace, a Chinese website for online shopping
altt flagship store	Battery Reconditioner	The quality is very good, very good, start the store service attitude, shipping speed👍	Taobao Marketplace, a Chinese website for online shopping
altt flagship store	Battery Reconditioner	Great quality, great stuff, well made, very happy with it.	Taobao Marketplace, a Chinese website for online shopping
altt flagship store	Battery Reconditioner	As great as described by the seller, great charging effect, strong sensitivity, very practical	Taobao Marketplace, a Chinese website for online shopping
altt flagship store	Battery Reconditioner	For those who have used it, how did it work? Please tell me the truth.	Taobao Marketplace, a Chinese website for online shopping
altt flagship store	Battery Reconditioner	Did it work?	Taobao Marketplace, a Chinese website for online shopping
altt flagship store	Battery Reconditioner	Will it work on any cell phone?	Taobao Marketplace, a Chinese website for online shopping
altt flagship store	Battery Reconditioner	Has it worked for those who have bought it?	Taobao Marketplace, a Chinese website for online shopping
altt flagship store	Battery Reconditioner	Does this thing kill people with laughter?	Taobao Marketplace, a Chinese website for online shopping
altt flagship store	Battery Reconditioner	Is it an IQ tax product?	Taobao Marketplace, a Chinese website for online shopping

altt flagship store	Battery Reconditioner	Does it work?	Taobao Marketplace, a Chinese website for online shopping
altt flagship store	Battery Reconditioner	What is a 3-in-1 data cable?	Taobao Marketplace, a Chinese website for online shopping
altt flagship store	Battery Reconditioner	Please ask your friends if this stuff is an IQ tax and if it really works	Taobao Marketplace, a Chinese website for online shopping
altt flagship store	Battery Reconditioner	Can the problem of capacity drop caused by irreversible side reactions within lithium batteries be repaired?	Taobao Marketplace, a Chinese website for online shopping
altt flagship store	Battery Reconditioner	Please is there any use?	Taobao Marketplace, a Chinese website for online shopping
altt flagship store	Battery Reconditioner	Which one is better to shoot?	Taobao Marketplace, a Chinese website for online shopping
altt flagship store	Battery Reconditioner	Will the original count be cleared after the fix?	Taobao Marketplace, a Chinese website for online shopping
altt flagship store	Battery Reconditioner	Does it work as he advertises?	Taobao Marketplace, a Chinese website for online shopping
altt flagship store	Battery Reconditioner	Is this thing paying an IQ tax?	Taobao Marketplace, a Chinese website for online shopping
altt flagship store	Battery Reconditioner	Can I save myold cell phonenot1year and is not charging and is dead?	Taobao Marketplace, a Chinese website for online shopping
altt flagship store	Battery Reconditioner	Does it really work?	Taobao Marketplace, a Chinese website for online shopping
altt flagship store	Battery Reconditioner	How effective is battery recovery ?	Taobao Marketplace, a Chinese website for online shopping
altt flagship store	Battery Reconditioner	Is it effective? Can it really repair batteries?	Taobao Marketplace, a Chinese website for online shopping
altt flagship store	Battery Reconditioner	It really works.	Taobao Marketplace, a Chinese website for online shopping
altt flagship store	Battery Reconditioner	Those of you who have used it! Does it work?	Taobao Marketplace, a Chinese website for online shopping
altt flagship store	Battery Reconditioner	Are they the kind of electronics that pay an IQ tax?	Taobao Marketplace, a Chinese website for online shopping
altt flagship store	Battery Reconditioner	Doesn't such a high-tech new energy vehicle eliminate the need to hemorrhage money on battery replacements?	Taobao Marketplace, a Chinese website for online shopping
altt flagship store	Battery Reconditioner	Will a Huawei phone also work?	Taobao Marketplace, a Chinese website for online shopping
altt flagship store	Battery Reconditioner	The effect is not yet known?	Taobao Marketplace, a Chinese website for online shopping
altt flagship store	Battery Reconditioner	Not much use.	Taobao Marketplace, a Chinese website for online shopping
altt flagship store	Battery Reconditioner	My family uses Huawei and Samsung cell phones, not sure about other brands.	Taobao Marketplace, a Chinese website for online shopping
altt flagship store	Battery Reconditioner	It's working.	Taobao Marketplace, a Chinese website for online shopping
altt flagship store	Battery Reconditioner	so that	Taobao Marketplace, a Chinese website for online shopping
altt flagship store	Battery Reconditioner	Bought just used not long ago, the effect is not obvious, but colleagues before buying according to the instructions to do, the effect is not bad, should be able to	Taobao Marketplace, a Chinese website for online shopping
altt flagship store	Battery Reconditioner	in the process of using	Taobao Marketplace, a Chinese website for online shopping
altt flagship store	Battery Reconditioner	3-in-1 data cable is3connectors:apple, android,type-c, basic market can be universal	Taobao Marketplace, a Chinese website for online shopping
altt flagship store	Battery Reconditioner	I don't know. I haven't used it yet.	Taobao Marketplace, a Chinese website for online shopping
altt flagship store	Battery Reconditioner	It doesn't feel like much.	Taobao Marketplace, a Chinese website for online shopping
altt flagship store	Battery Reconditioner	It doesn't work. Nothing works.	Taobao Marketplace, a Chinese website for online shopping
altt flagship store	Battery Reconditioner	It's not much use.	Taobao Marketplace, a Chinese website for online shopping
altt flagship store	Battery Reconditioner	No, but the repair process is always slow.	Taobao Marketplace, a Chinese website for online shopping
altt flagship store	Battery Reconditioner	It doesn't work. It's all a lie.	Taobao Marketplace, a Chinese website for online shopping
altt flagship store	Battery Reconditioner	I don't know.	Taobao Marketplace, a Chinese website for online shopping
altt flagship store	Battery Reconditioner	general	Taobao Marketplace, a Chinese website for online shopping
altt flagship store	Battery Reconditioner	of no great use	Taobao Marketplace, a Chinese website for online shopping
altt flagship store	Battery Reconditioner	this one, this one is more affordable, according to the instructions, from the original charge twice a day, now charge once a day	Taobao Marketplace, a Chinese website for online shopping
altt flagship store	Battery Reconditioner	I've been using it for two days and it hasn't worked yet	Taobao Marketplace, a Chinese website for online shopping
altt flagship store	Battery Reconditioner	High-tech stuff, can't read it, but when I first bought my phone, the original charger was charging at50.01percent, and now it's charging50percent with this tool of his	Taobao Marketplace, a Chinese website for online shopping

altt flagship store	Battery Reconditioner	I don't think it's gonna help.	Taobao Marketplace, a Chinese website for online shopping
altt flagship store	Battery Reconditioner	Kind of. Wouldn't it be a great invention if it actually straightened out the battery?youthink/:813	Taobao Marketplace, a Chinese website for online shopping
altt flagship store	Battery Reconditioner	IQ tax! No you use! Has been broken! Don't buy it!	Taobao Marketplace, a Chinese website for online shopping
altt flagship store	Battery Reconditioner	night. I don't know if it will solve the battery problem either.	Taobao Marketplace, a Chinese website for online shopping
blesi flagship store	Battery Reconditioner	Just the right size, easy to use.	Taobao Marketplace, a Chinese website for online shopping
blesi flagship store	Battery Reconditioner	very good	Taobao Marketplace, a Chinese website for online shopping
blesi flagship store	Battery Reconditioner	Worth buying the product, very fine workmanship, better than expected, recommended to try	Taobao Marketplace, a Chinese website for online shopping
blesi flagship store	Battery Reconditioner	Cell phone battery repair god received, repair effect is very good, cost-effective	Taobao Marketplace, a Chinese website for online shopping
blesi flagship store	Battery Reconditioner	Works well and is very affordable.	Taobao Marketplace, a Chinese website for online shopping
blesi flagship store	Battery Reconditioner	The packaging is very sturdy and the stuff is well protected, so it's worth a good review	Taobao Marketplace, a Chinese website for online shopping
blesi flagship store	Battery Reconditioner	Works for the first time, keep trying	Taobao Marketplace, a Chinese website for online shopping
blesi flagship store	Battery Reconditioner	It works.	Taobao Marketplace, a Chinese website for online shopping
blesi flagship store	Battery Reconditioner	Very satisfied, really like, completely exceeded expectations, very fast shipping, packaging is very careful, strict, logistics company service attitude is very good, fast delivery, very satisfied with a shopping	Taobao Marketplace, a Chinese website for online shopping
blesi flagship store	Battery Reconditioner	didn't see any effect after two days of use, the previous83%battery is now so no□point change.	Taobao Marketplace, a Chinese website for online shopping
blesi flagship store	Battery Reconditioner	Packaging is very careful. Tight, logistics company service attitude is very good, the delivery speed is very fast, very satisfied with a shopping	Taobao Marketplace, a Chinese website for online shopping
blesi flagship store	Battery Reconditioner	This cell phone battery repairer, it is of good quality! This baby, it's appearance, exquisitely crafted, efficiently top-quality, repairing effect, worth waiting and buying!	Taobao Marketplace, a Chinese website for online shopping
blesi flagship store	Battery Reconditioner	The store has a very good service attitude, the quality is very good, I received it	Taobao Marketplace, a Chinese website for online shopping
blesi flagship store	Battery Reconditioner	basically has no effect, and the current is weakened, and I phonsmart phone is affected, and the battery stops often, and then it is unplugged and plugged in again, and it still can't be full, and it is an IQ tax! The battery's wear and tear capacity is reduced and can't be repaired from scratch, so the next day we send it to the service department to replace it with a new one, and it's a real rebirth! _x005f_x0001_Good	Taobao Marketplace, a Chinese website for online shopping
blesi flagship store	Battery Reconditioner	It works.	Taobao Marketplace, a Chinese website for online shopping
blesi flagship store	Battery Reconditioner	Great quality as always, a very favorite product, great! o(*￣︶￣*)o	Taobao Marketplace, a Chinese website for online shopping
blesi flagship store	Battery Reconditioner	Product quality is very good, fine workmanship, design style atmospheric fashion, and the same as the business description.	Taobao Marketplace, a Chinese website for online shopping
blesi flagship store	Battery Reconditioner	Expected baby received, very satisfied and love it. Very good quality, recommend it!	Taobao Marketplace, a Chinese website for online shopping
blesi flagship store	Battery Reconditioner	In-kind and the same as described, the quality is quite good, the seller's attitude is also good, ask questions and answer them, the speed of delivery bar, worth buying Oh!	Taobao Marketplace, a Chinese website for online shopping
blesi flagship store	Battery Reconditioner	A domestically produced charger is labeled by the business as a cell phone battery repair miracle imported from Germany.	Taobao Marketplace, a Chinese website for online shopping
blesi flagship store	Battery Reconditioner	Expected baby received, very satisfied and love it, the quality is very good, worth buying.	Taobao Marketplace, a Chinese website for online shopping
blesi flagship store	Battery Reconditioner	The product is great to use, well made, great price and quality!	Taobao Marketplace, a Chinese website for online shopping
blesi flagship store	Battery Reconditioner	After charging and using does have a restorative effect, the phone battery than before there is a significant continuation of the length of time, and not card, very practical	Taobao Marketplace, a Chinese website for online shopping
blesi flagship store	Battery Reconditioner	Expected baby received, very satisfied, also like the quality is very good, worth buying!	Taobao Marketplace, a Chinese website for online shopping
blesi flagship store	Battery Reconditioner	Very satisfied, really like, completely exceeded expectations, very fast shipping, packaging is very careful, strict, logistics company service attitude is very good, fast delivery, very satisfied with a shopping	Taobao Marketplace, a Chinese website for online shopping
blesi flagship store	Battery Reconditioner	It is genuine.Quite good,simple and generous shape and the description is not bad.	Taobao Marketplace, a Chinese website for online shopping
blesi flagship store	Battery Reconditioner	Logistics is also fast, very can. Logistics attitude is also very can, the price is too good value	Taobao Marketplace, a Chinese website for online shopping
blesi flagship store	Battery Reconditioner	continue to repurchase! All five points! Ness Ness ~ it's authentic!	Taobao Marketplace, a Chinese website for online shopping
blesi flagship store	Battery Reconditioner	the more you do better and better, very good shopping experience!	Taobao Marketplace, a Chinese website for online shopping

blesi flagship store	Battery Reconditioner	The appearance is small and exquisite, and it is quite practical to use.	Taobao Marketplace, a Chinese website for online shopping
blesi flagship store	Battery Reconditioner	Used it. Works well. Battery use was noticeably longer today, amazing results!	Taobao Marketplace, a Chinese website for online shopping
blesi flagship store	Battery Reconditioner	Being repaired, see the display jumped the number, tried several times the phone has the effect, praise!	Taobao Marketplace, a Chinese website for online shopping
blesi flagship store	Battery Reconditioner	Good quality, well-packed, logistics is quite fast, the price is fair, very practical, and very satisfied!	Taobao Marketplace, a Chinese website for online shopping
blesi flagship store	Battery Reconditioner	Take advantage of the offer, hurry to get it, it is genuine, the packaging is also very good, no damage, and the quality is not to speak of????	Taobao Marketplace, a Chinese website for online shopping
blesi flagship store	Battery Reconditioner	Very good, great packaging, looks good, next time, arrived very quickly good product, good quality, good service, baby is really worth the price!	Taobao Marketplace, a Chinese website for online shopping
blesi flagship store	Battery Reconditioner	I thought this thing was another IQ tax, but I was really wrong this time, it works!	Taobao Marketplace, a Chinese website for online shopping
blesi flagship store	Battery Reconditioner	Expected baby received, very satisfied and love it, the quality is very good, worth buying.	Taobao Marketplace, a Chinese website for online shopping
blesi flagship store	Battery Reconditioner	Good has been here to buy this, the activities of the price is very beautiful, the delivery speed thief fast, good quality, should be genuine	Taobao Marketplace, a Chinese website for online shopping
blesi flagship store	Battery Reconditioner	very favorite store, I want to crazy recommend to friends things quality is okay, the speed of the courier is also very strong!	Taobao Marketplace, a Chinese website for online shopping
blesi flagship store	Battery Reconditioner	Old cell phone, after restoration, standby for a day, and more than sixty power, rare good product, worth buying	Taobao Marketplace, a Chinese website for online shopping
blesi flagship store	Battery Reconditioner	You can use it. It's working well now.	Taobao Marketplace, a Chinese website for online shopping
blesi flagship store	Battery Reconditioner	continue to repurchase! All five points! Ness Ness ~ it's authentic!	Taobao Marketplace, a Chinese website for online shopping
blesi flagship store	Battery Reconditioner	The quality is great, the baby is completely in line with the seller's description, recommended to get. Express logistics is also very strong, conscientious business!	Taobao Marketplace, a Chinese website for online shopping
blesi flagship store	Battery Reconditioner	Good quality of goods, details, quality, workmanship are very good, shipping thief, express delivery,~	Taobao Marketplace, a Chinese website for online shopping
blesi flagship store	Battery Reconditioner	The product is made of thick material, fine workmanship, very good to use and good quality!	Taobao Marketplace, a Chinese website for online shopping
blesi flagship store	Battery Reconditioner	The appearance of the atmosphere, easy to carry, very good, worth buying	Taobao Marketplace, a Chinese website for online shopping
blesi flagship store	Battery Reconditioner	Packaging is very careful. Tight, logistics company service attitude is very good, the delivery speed is very fast, very satisfied with a shopping	Taobao Marketplace, a Chinese website for online shopping
blesi flagship store	Battery Reconditioner	Expected baby received, very satisfied and love it. The quality is very good, recommend chanting	Taobao Marketplace, a Chinese website for online shopping
blesi flagship store	Battery Reconditioner	Very courteous and the customer service lady returned my queries in seconds. hehehehehehehehehehehehehehe, I will repurchase next time.	Taobao Marketplace, a Chinese website for online shopping
blesi flagship store	Battery Reconditioner	After a few days of testing the phone's battery temperature has dropped, the battery is slightly extended, or constantly have to be repaired, may only achieve some results.	Taobao Marketplace, a Chinese website for online shopping
blesi flagship store	Battery Reconditioner	Expected baby received, very satisfied, also like, the quality is very good, worth buying!	Taobao Marketplace, a Chinese website for online shopping
blesi flagship store	Battery Reconditioner	This repairer after using the phone directly newborn, it is very good, the phone will not be hot, the response are becoming sensitive, have the ability to do it all can be completed, the battery is quite durable, the capacity is particularly large, the response effect is also super agile, charging effect is also super fast!	Taobao Marketplace, a Chinese website for online shopping
blesi flagship store	Battery Reconditioner	This battery restorer is very good, it is genuine can't wait to get it back can't wait to use it Good quality. Charging stability. Need to buy again.	Taobao Marketplace, a Chinese website for online shopping
blesi flagship store	Battery Reconditioner	After a few days of testing the phone's battery temperature has dropped, the battery is slightly extended, or constantly have to be repaired, may only achieve some results.	Taobao Marketplace, a Chinese website for online shopping
blesi flagship store	Battery Reconditioner	This repairer after using the phone directly newborn, it is very good, the phone will not be hot, the response are becoming sensitive, have the ability to do it all can be completed, the battery is quite durable, the capacity is particularly large, the response effect is also super agile, charging effect is also super fast!	Taobao Marketplace, a Chinese website for online shopping
blesi flagship store	Battery Reconditioner	battery, after repairing the phone with a little longer, and does not heat up, but also automatically clean up the phone system cache garbage, the phone has a significantly smooth, the boss said that often used to repair will be a little better. Does not hurt the phone, the appearance is very beautiful, but also equipped with a display, it is very convenient to use!	Taobao Marketplace, a Chinese website for online shopping

blesi flagship store	Battery Reconditioner	Battery Repairer received, fast logistics, the use of the evaluation, the effect is very good, the battery time significantly increased a lot, in accordance with the instructions required by the shutdown repair effect, without interference, the effect can be up to ninety percent or more!	Taobao Marketplace, a Chinese website for online shopping
blesi flagship store	Battery Reconditioner	The seller's attitude is also good, ask questions and answer them, the speed of delivery is great, it is worth buying Oh worth buying the product, the workmanship is very fine, better than expected, recommended to try good!	Taobao Marketplace, a Chinese website for online shopping
New Rhythm Shopkeeper	Battery Repairer	Products off the shelves	JD.com
Cajepont Technology	Battery Reconditioner	Can this thing can can be fixed?	JD.com
Cajepont Technology	Battery Reconditioner	Express delivery arrived in three days, used it, okay, satisfactory	JD.com
Cajepont Technology	Battery Reconditioner	Received, not yet used, effect unknown.	JD.com
Cajepont Technology	Battery Reconditioner	Fast logistics, well packaged, very practical	JD.com
Cajepont Technology	Battery Reconditioner	Charging: three plugs,<br>Charging speed: pretty much the same.	JD.com
Cajepont Technology	Battery Reconditioner	Try it and see how it goes.	JD.com
Cajepont Technology	Battery Reconditioner	This cell phone repairer is quite convenient to use, while charging and repairing, yesterday repaired once, today is repairing again, after the cell phone is repaired, the electricity is more durable than before,, and the charging speed is fast and safe, this is the second time I buy it, give it to my siblings. I will recommend it to my friends later.	JD.com
Cajepont Technology	Battery Reconditioner	It feels like the phone battery is more durable and lasts longer	JD.com
Cajepont Technology	Battery Reconditioner	(of an unmarried couple) be close	JD.com
Cajepont Technology	Battery Reconditioner	Baby received, logistics is very fast, the design is very good, charging speed, very satisfied!	JD.com
Cajepont Technology	Battery Reconditioner	Works great. Great results.	JD.com
Cajepont Technology	Battery Reconditioner	phone system for Android series and Apple series. Effect to be evaluated.	JD.com
Cajepont Technology	Battery Reconditioner	I got it. I'll try it before I give it a good review.	JD.com
Cajepont Technology	Battery Reconditioner	Very good, the baby is very high, the quality is very good, it looks like it gives a sense of high class, the price is also suitable, this online shopping is very pleasant, big praise 👍	JD.com
Cajepont Technology	Battery Reconditioner	It would be nice if the restorer could fix the battery.	JD.com
Cajepont Technology	Battery Reconditioner	Beautifully styled and easy to use, love this product. 👍✌	JD.com
Cajepont Technology	Battery Reconditioner	Shipping was fast, stuff was well packaged, great price, I'm happy with👍and will repurchase	JD.com
Cajepont Technology	Battery Reconditioner	using, the effect is good or not, after using a follow-up comment.	JD.com
Cajepont Technology	Battery Reconditioner	Goods received, my cell phone is always charging very slowly, and easy to lag, this time to buy this, can solve this problem, great! This time also thanks to the Shentong Express staff to deliver the goods to the home, to solve my legs and feet difficult to pick up is not! Thank you so much!	JD.com
Cajepont Technology	Battery Reconditioner	Been using it for a few days now and really the phone doesn't get hot. Fixed the battery to be continued to comment again.	JD.com
Cajepont Technology	Battery Reconditioner	I just received it and will comment later, hopefully it lives up to the hype.	JD.com
Cajepont Technology	Battery Reconditioner	an excellent	JD.com
Cajepont Technology	Battery Reconditioner	Works well and the cell phone battery is noticeably more durable.	JD.com
Cajepont Technology	Battery Reconditioner	It feels good. - It feels good. - It feels good.	JD.com
Cajepont Technology	Battery Reconditioner	Fast charging speed	JD.com
Cajepont Technology	Battery Reconditioner	Fast logistics, good quality, trustworthy.	JD.com
Cajepont Technology	Battery Reconditioner	Received, a very good restorer	JD.com
Cajepont Technology	Battery Reconditioner	It works great The repairs	JD.com
Cajepont Technology	Battery Reconditioner	Good, strong repair, still feels good	JD.com
Cajepont Technology	Battery Reconditioner	Great value for money and just as described by the merchant, this is a great product!	JD.com

Cajepont Technology	Battery Reconditioner	I've been using it for a while and I feel it's still working!	JD.com
Cajepont Technology	Battery Reconditioner	Goods logistics is very fast! The goods are also good value for money worth buying! Recommended to you! It does work well! Give merchants favorable comments! You can get it if you need it! Kudos to support!	JD.com
Cajepont Technology	Battery Reconditioner	Things received, small and exquisite, beautiful and generous, can not wait to try, do not know how to use, should be good, with a few times to follow up!	JD.com
Cajepont Technology	Battery Reconditioner	The style is new and fine workmanship, used a little, and will not see if there is no effect. Anyway, the heart is satisfied.	JD.com
Cajepont Technology	Battery Reconditioner	Look so lofty, do not know how to repair the effect, use a period of time to see the battery effect	JD.com
Cajepont Technology	Battery Reconditioner	It works very well, after that I don't have to worry about the battery not lasting any longer, the battery is full of health. Very satisfied	JD.com
Cajepont Technology	Battery Reconditioner	Very happy with the cell phone fixer, nice and good.	JD.com
Cajepont Technology	Battery Reconditioner	(of an unmarried couple) be close	JD.com
Cajepont Technology	Battery Reconditioner	Am following the instructions and will submit a review later.	JD.com
Cajepont Technology	Battery Reconditioner	Repair effect is particularly good, charging speed is very fast, conscientious business, worth buying	JD.com
Cajepont Technology	Battery Reconditioner	Very good fast shipping good courier service good item good value for money	JD.com
Cajepont Technology	Battery Reconditioner	Works well and extends battery life	JD.com
Cajepont Technology	Battery Reconditioner	Expect it to work.	JD.com
Cajepont Technology	Battery Reconditioner	It is being restored for use!	JD.com
Cajepont Technology	Battery Reconditioner	It's in the process of being fixed. It feels good.	JD.com
Cajepont Technology	Battery Reconditioner	Didn't have time to pick it up at the delivery point! Hope it works. I'm used to good reviews.	JD.com
Cajepont Technology	Battery Reconditioner	an excellent	JD.com
Cajepont Technology	Battery Reconditioner	the display screen numbers have been changing, I do not know if it is repairing the battery, looked at several models before choosing this one, I feel that the quality of this one is the best.	JD.com
Cajepont Technology	Battery Reconditioner	The first time to use, do not know how it is, first give some praise, give five stars, packaging in all aspects are very good, logistics is also very fast.	JD.com
Cajepont Technology	Battery Reconditioner	Fast delivery, good service, good results for a one time fix, keep tracking the follow up results, recommend it.	JD.com
Cajepont Technology	Battery Reconditioner	Cell phone battery is not durable, bought this item. It is in use, the effect of use to be followed up.	JD.com
Cajepont Technology	Battery Reconditioner	The quality of the negotiation is very good, the charging and repairing effect is very good, I like it very much.	JD.com
Cajepont Technology	Battery Reconditioner	see the numbers jumping. We'll see what we can do when we run out.	JD.com
Cajepont Technology	Battery Reconditioner	Baby got it. It works.	JD.com
Cajepont Technology	Battery Reconditioner	The effect is very good, I used to charge this phone very slowly, just repair once, charging much faster!	JD.com
Cajepont Technology	Battery Reconditioner	Fast brother delivery is very fast, goods received, very satisfied	JD.com
Cajepont Technology	Battery Reconditioner	It's good. It's pretty good.	JD.com
Cajepont Technology	Battery Reconditioner	The effect is very good, after receiving the goods, immediately go to try the effect is good, the next time there are friends to your home to recommend the purchase of good	JD.com
Cajepont Technology	Battery Reconditioner	This is a good look, it still works	JD.com
Cajepont Technology	Battery Reconditioner	Received it. It's what I wanted to buy.	JD.com
Cajepont Technology	Battery Reconditioner	favorable criticism	JD.com
Cajepont Technology	Battery Reconditioner	Cell phone battery repairer received, do not know whether it can be repaired effectively look forward to the effect, but still give five-star praise!	JD.com
Cajepont Technology	Battery Reconditioner	Very satisfied with the purchase	JD.com

Cajepont Technology	Battery Reconditioner	I feel that the battery life of the phone has improved significantly, and the power consumption is not so fast when using the phone, and the power change display is very clear when charging, and I will continue to use it in anticipation of getting a better experience! Good review!!!	JD.com
Cajepont Technology	Battery Reconditioner	Just received and used, do not know if it is good ah wait ah good in the evaluation	JD.com
Cajepont Technology	Battery Reconditioner	Product received, quite good, good quality	JD.com
Cajepont Technology	Battery Reconditioner	Goods are very good, well-packed not damaged at all, logistics is fast and trustworthy!	JD.com
Cajepont Technology	Battery Reconditioner	Smart Fix little machine received, small and compact, works great!	JD.com
Cajepont Technology	Battery Reconditioner	This charging repairer is very good, my cell phone can not turn off the power, and a long time the phone is hot, use this repairer a few days later, the phone this phenomenon has significantly improved, it is worth having.	JD.com
Cajepont Technology	Battery Reconditioner	Used a few days do not know, too old phone is impossible to repair, because the battery has been bad or deep sleep, there is a Huawei phone with6years in the middle of the original factory replaced the battery with three years, so far can not see, need to observe, and then tell you	JD.com
Cajepont Technology	Battery Reconditioner	The goods have been received,,, fine workmanship, beautiful appearance, good quality, I am satisfied. I am satisfied.	JD.com
Cajepont Technology	Battery Reconditioner	I received the loan this morning, and I am using it, it should be effective, everyone says it's good, and I'll follow up after a while![Good][Kudos]	JD.com
Cajepont Technology	Battery Reconditioner	Fast delivery, well packaged, good value, works great, thanks!	JD.com
Cajepont Technology	Battery Reconditioner	The product is good and effective……can be worth having!	JD.com
Cajepont Technology	Battery Reconditioner	It's good. Just used it yesterday. I'm using it. I'll buy a couple more when I'm done.	JD.com
Cajepont Technology	Battery Reconditioner	Product received. Good quality. Shipping was fast. Better than the original purchase. One price for one item. Hope it helps. Good reviews in advance.	JD.com
Cajepont Technology	Battery Reconditioner	Used it a couple of times with some results, will comment after a few more days of use.	JD.com
Cajepont Technology	Battery Reconditioner	It works very well, the cell phone battery life is really extended, high tech!	JD.com
Cajepont Technology	Battery Reconditioner	Boss:I ordered to buy a cell phone repair device has been received, the description matches the physical object, the logistics is strong, to see how the effect of the use of the cycle? Favorable comments satisfied5points.	JD.com
Cajepont Technology	Battery Reconditioner	The item works, it's affordable and works well, and it has a generous appearance	JD.com
Cajepont Technology	Battery Reconditioner	in use	JD.com
Cajepont Technology	Battery Reconditioner	economical	JD.com
Cajepont Technology	Battery Reconditioner	Goods have been received, business services, packaging logistics Yu is very satisfied, thank you!	JD.com
Cajepont Technology	Battery Reconditioner	This user did not fill in a rating	JD.com
Cajepont Technology	Battery Reconditioner	Baby is good, value for money, business integrity, customer service Yujia, logistics!	JD.com
Cajepont Technology	Battery Reconditioner	The baby was received and given 5 stars!	JD.com
Cajepont Technology	Battery Reconditioner	It works.	JD.com
Cajepont Technology	Battery Reconditioner	Very good, is repurchasing again. Functions are powerful.	JD.com
Cajepont Technology	Battery Reconditioner	Cell phone repairer received not only the effect of how to make good use of the chemical continue to repurchase the first five-star praise!	JD.com
Cajepont Technology	Battery Reconditioner	The goods have received the packaging can be very good to give a praise!	JD.com
Cajepont Technology	Battery Reconditioner	Used it. Pretty good. I give it 5 stars.	JD.com
Cajepont Technology	Battery Reconditioner	Received the goods and used it once Don't know the exact effect yet It should be fine	JD.com
Cajepont Technology	Battery Reconditioner	The item was received, good quality, easy to use and good delivery.	JD.com
Cajepont Technology	Battery Reconditioner	The product is affordable. The quality is in use.	JD.com
Cajepont Technology	Battery Reconditioner	Works well, no more hot charging	JD.com

Cajepont Technology	Battery Reconditioner	Fast charging and the phone is less likely to heat up	JD.com
Cajepont Technology	Battery Reconditioner	Received the cell phone battery repairer, looks very formal, good quality, but have not tried, to be used after the follow-up comment, the business service is thoughtful, very patient[kudos]logistics is very fast	JD.com
Cajepont Technology	Battery Reconditioner	Tried it for three days, no obvious effect, wait for time to test it!	JD.com
Cajepont Technology	Battery Reconditioner	Received a cell phone battery repairer, asked customer service to use the method according to the instructions began to do repair work, showing repair data in the change, I hope that after5-8weeks to repair my cell phone battery performance is improved, five points.	JD.com
Cajepont Technology	Battery Reconditioner	I'm very satisfied with this fixer, my cell phone doesn't get stuck anymore.	JD.com
Cajepont Technology	Battery Reconditioner	Fast logistics, good price and good service.	JD.com
Cajepont Technology	Battery Reconditioner	Good quality and fast logistics, trustworthy[kudos][kudos][kudos][kudos	JD.com
Cajepont Technology	Battery Reconditioner	The restorer is of good quality, looks great, well made, and looks forward to refreshing the battery.	JD.com
Cajepont Technology	Battery Reconditioner	I received the cell phone repairer, and I repaired it once, but I didn't feel how effective it is? Need to repair many times to see the effect, right? The logistics is fast.	JD.com
Cajepont Technology	Battery Reconditioner	I used it once, I don't know if it worked or not. I'll use it again in a few days!	JD.com
Cajepont Technology	Battery Reconditioner	Fine workmanship, affordable, clear power display	JD.com
Cajepont Technology	Battery Reconditioner	give you a good review and see if it works.	JD.com
Cajepont Technology	Battery Reconditioner	Great results, quality assurance, great service, peace of mind!	JD.com
Cajepont Technology	Battery Reconditioner	This smart charge is very good indeed is the same as advertised above, the service and good rest assured to buy!	JD.com
Cajepont Technology	Battery Reconditioner	Battery Repairer Skewer, can effectively improve the battery life more durable clean up cell phone garbage, improve the running speed of the phone. Very good.	JD.com
Cajepont Technology	Battery Reconditioner	Received and rated 5 stars	JD.com
Cajepont Technology	Battery Reconditioner	cheap and affordableandworks[big love]big love][big	JD.com
Cajepont Technology	Battery Reconditioner	Really trying it out and will submit a review after it operates.	JD.com
Cajepont Technology	Battery Reconditioner	Baby received, the logistics is very fast, the product quality is very good, as described, especially customer service Miss is very provincial patience, very enthusiastic.	JD.com
Cajepont Technology	Battery Reconditioner	It's working well. Keep using it.	JD.com
Cajepont Technology	Battery Reconditioner	Goods have been received, well-packed, logistics is also very fast, used two days the phone is indeed not hot before, the speed is also faster[praise]	JD.com
Cajepont Technology	Battery Reconditioner	Nice product, trustworthy……	JD.com
Cajepont Technology	Battery Reconditioner	That's good. That's good.	JD.com
Cajepont Technology	Battery Reconditioner	Okay!	JD.com
Cajepont Technology	Battery Reconditioner	product doesn't restore the battery to its original state in one go you have to keep using it for a while to get better results!	JD.com
Cajepont Technology	Battery Reconditioner	It looks good, try charging it twice, pulse charging activates the battery, it should be reliable.	JD.com
Cajepont Technology	Battery Reconditioner	two back to power, now rushed-back can use a day, so give five stars.	JD.com
Cajepont Technology	Battery Reconditioner	Cell phone battery board capacity is insufficient, buy this repairer to repair it, the effect is not clear, but also need to repair a period of time to see the effect, looking forward to, I hope a good result.	JD.com
Cajepont Technology	Battery Reconditioner	Cell phone repairer just received, get back to install to the phone charged, do not know how to repair the effect, the Internet also has a cheaper than this are there, I bought this is to be a little more expensive, I hope to have the effect of the cell phone has a big help.	JD.com
Cajepont Technology	Battery Reconditioner	I used it as soon as I received it and read the instructions to use it for a couple of weeks before I could see any results, so I'm looking forward to it!	JD.com

Cajepont Technology	Battery Reconditioner	Baby received, logistics is very fast, well packaged! Tried it in the comments! Repair hand machine works great! Now the phone good not card! Customer service attitude is very very good too powerful! Affordable	JD.com
Cajepont Technology	Battery Reconditioner	The line universal charger, like, genuine[love][love][love][love][love][love]	JD.com
Cajepont Technology	Battery Reconditioner	The product received, the appearance is good, just used two days look forward to the effect!	JD.com
Cajepont Technology	Battery Reconditioner	It feels like it should work. Relatively1%longer to watch videos on electricity	JD.com
Cajepont Technology	Battery Reconditioner	I use after the evaluation, charging once or twice can not see the more fruitful	JD.com
Cajepont Technology	Battery Reconditioner	Received the goods, used it feels pretty good.	JD.com
Cajepont Technology	Battery Reconditioner	Used it right away, flickering changes, works well. Good review!	JD.com
Cajepont Technology	Battery Reconditioner	Trying it out.	JD.com
Cajepont Technology	Battery Reconditioner	Received the goods is quite good, worth buying	JD.com
Cajepont Technology	Battery Reconditioner	an excellent	JD.com
Cajepont Technology	Battery Reconditioner	Received the restorer, not sure if it works.	JD.com
Cajepont Technology	Battery Reconditioner	Just as described.	JD.com
Cajepont Technology	Battery Reconditioner	good quality. Received the goods I immediately used to charge, the result is really cell phone one day down the power than not use the restorer when more very each, is true, for you to praise.	JD.com
Cajepont Technology	Battery Reconditioner	Display experience: very good<br>Product design: very good<br>Product appearance: very good<br>Rechargeable battery: very good<br>Product function: very good<br>Carrying description: very convenient	JD.com
Cajepont Technology	Battery Reconditioner	I am very satisfied with this product, please recognize this product to buy with confidence.	JD.com
Cajepont Technology	Battery Reconditioner	I just received the baby today, it charges quickly and the phone doesn't get hot!	JD.com
Cajepont Technology	Battery Reconditioner	The product was received, the logistics is very fast, the product quality is guaranteed!	JD.com
Cajepont Technology	Battery Reconditioner	Received the product, the logistics is very fast, after the trial and then give a follow-up comment!	JD.com
Cajepont Technology	Battery Reconditioner	Got it, it's great, I love it, awesome.	JD.com
Cajepont Technology	Battery Reconditioner	The stuff works, I've been using itmore than2great	JD.com
Cajepont Technology	Battery Reconditioner	Looked good quality, don't know how well it works yet, just received the goods.	JD.com
Cajepont Technology	Battery Reconditioner	Uses a good general courier suit	JD.com
Cajepont Technology	Battery Reconditioner	effect is also good, the next time will be back to buy, I wish the boss business is booming, the source of wealth to make a fortune.	JD.com
Cajepont Technology	Battery Reconditioner	Baby received, as described, looks great, first five star review a...	JD.com
Cajepont Technology	Battery Reconditioner	Works great, inexpensive, won't know how it works until a few days later	JD.com
Cajepont Technology	Battery Reconditioner	Received!ll follow up with a review after I use it[grimace]	JD.com
Cajepont Technology	Battery Reconditioner	Just started using it, seems to be working, use it and see what happens!	JD.com
Cajepont Technology	Battery Reconditioner	Received it, looks good, will follow up later.	JD.com
Cajepont Technology	Battery Reconditioner	Purposefully used a few days before evaluation, can can	JD.com
Cajepont Technology	Battery Reconditioner	thing, but also feel good play, but also good-looking, ask the customer service attitude is also good, use a few days to evaluate.	JD.com
Cajepont Technology	Battery Reconditioner	The baby has been received, the first time to use a little bit of the effect is okay	JD.com
Cajepont Technology	Battery Reconditioner	Received it, used it once and looking forward to satisfactory results.	JD.com
Cajepont Technology	Battery Reconditioner	Logistics fast service attitude is good, the arrival of timely, intact packaging, the physical and publicity of the full compliance, special to give praise!	JD.com

Cajepont Technology	Battery Reconditioner	Hello: Received the goods, very satisfied ready to repurchase.	JD.com
Cajepont Technology	Battery Reconditioner	The restorer was received and works well after a couple of uses.	JD.com
Cajepont Technology	Battery Reconditioner	In a repair cell phone battery, really works, I think the cell phone battery is longer than before, not so hot!	JD.com
Cajepont Technology	Battery Reconditioner	I received it and couldn't wait to try it out, it felt good!	JD.com
Cajepont Technology	Battery Reconditioner	Not bad.<br>It	JD.com
Cajepont Technology	Battery Reconditioner	well-packed, logistics is also very fast. Full five-star praise!	JD.com
Cajepont Technology	Battery Reconditioner	Things received, intact, courier service attitude is good, the effect is how, to be used and then evaluate it.	JD.com
Cajepont Technology	Battery Reconditioner	Goods received good quality, logistics is also very fast	JD.com
Cajepont Technology	Battery Reconditioner	Received the product and will follow up with a review after use.	JD.com
Cajepont Technology	Battery Reconditioner	The restorer looks good and displays the numbers clearly, so it's recommended!	JD.com
Cajepont Technology	Battery Reconditioner	Cell phone fixer is good, worth buying.	JD.com
Cajepont Technology	Battery Reconditioner	Cell phone battery repairer received, logistics is fast, well-packed, compact, easy to use, for repairing cell phone batteries have a greater role.	JD.com
Cajepont Technology	Battery Reconditioner	Fast shipping. After receiving the goods, check the packaging is intact, open the package to take out the device, the device looks good, easy to use, according to the instructions guide immediately inserted into the power supply, the device smoothly into the normal working state. Kudos.	JD.com
Cajepont Technology	Battery Reconditioner	Baby received, love it👍with it the phone is easy, thanks to the business! Good luck with business👌👌👌👌👌	JD.com
Cajepont Technology	Battery Reconditioner	Will follow up after a few days of use	JD.com
Cajepont Technology	Battery Reconditioner	I got it. It feels good.	JD.com
Cajepont Technology	Battery Reconditioner	Baby received, good quality, easy to use, easy to use, express safe and reliable, worth buying!	JD.com
Cajepont Technology	Battery Reconditioner	Charging speed: fast<br>Aesthetics: beautiful<br>Display experience: good!	JD.com
Cajepont Technology	Battery Reconditioner	Try it first and if it works, will repurchase!	JD.com
Cajepont Technology	Battery Reconditioner	Fast charging, after fixing it, the phone is much faster and not laggy anymore	JD.com
Cajepont Technology	Battery Reconditioner	Works great, fast shipping, will buy from here again next time!	JD.com
Cajepont Technology	Battery Reconditioner	I just received it and tried it, it's ok, worth buying.	JD.com
Cajepont Technology	Battery Reconditioner	Received the item and used it once with good results.	JD.com
Cajepont Technology	Battery Reconditioner	Product Appearance: cool shape<br>Rechargeable Battery: quick charging<br>Product Function: consistent with the description of the advertisement, give five stars.	JD.com
Cajepont Technology	Battery Reconditioner	Logistics is very fast, the service attitude is very good, immediately after receiving the goods to take an old cell phone to try to fix it, the	JD.com
Cajepont Technology	Battery Reconditioner	pretty good	JD.com
Cajepont Technology	Battery Reconditioner	Cell phone repairer has received,, fine workmanship,, affordable, on the cell phone charging repair function is very good, the phone is also very good with the packaging is very good, the businessmen shipment fast, logistics speed, on time, service attitude are very good, logistics courier hard work thank you, rated five-pointed star, worth buying, I wish the businessmen to prosper and develop their business.	JD.com
Cajepont Technology	Battery Reconditioner	Multi-functional cell phone battery repairer received, the quality is very good, I like it very much.	JD.com
Cajepont Technology	Battery Reconditioner	Baby received, very good, packaging is very good no defects, with the description in line. Baby is very beautiful, can repair the cell phone battery, I have a flat version, charging can not be charged, with this baby, bidding can make the electric signal shine, this baby is quite good, like.<br>Thank you for mentioning the good goods, thank you courier boy, good service attitude, fast delivery. Thank you!	JD.com
Cajepont Technology	Battery Reconditioner	It's really good, just bought it and used it	JD.com

Cajepont Technology	Battery Reconditioner	As described by Dodo, the product is of good quality and easy to use!	JD.com
Cajepont Technology	Battery Reconditioner	Beautifully packaged, beautiful appearance, reliable quality, easy to use, worth having.	JD.com
Cajepont Technology	Battery Reconditioner	At work, ask customer service if you don't understand something and get back in seconds[kudos]	JD.com
Cajepont Technology	Battery Reconditioner	I'm using it to see how it works.	JD.com
Cajepont Technology	Battery Reconditioner	I got it. It's a nice view.	JD.com
Cajepont Technology	Battery Reconditioner	with facts, it is really good, it can eliminate a lot of traffic. It's a great way to optimize your phone while repairing it, and it also has a function to organize the debris in your phone.	JD.com
Cajepont Technology	Battery Reconditioner	pick up the courier, see the effect, late in the additional evaluation	JD.com
Cajepont Technology	Battery Reconditioner	Well made, nice looking, pretty good.	JD.com
Cajepont Technology	Battery Reconditioner	works, use it and then say, if it's good, I highly recommend it!	JD.com
Cajepont Technology	Battery Reconditioner	The goods have been received, immediately used to try, feel or repair effect.	JD.com
Cajepont Technology	Battery Reconditioner	The stuff is great, it protects your phone, the stuff is great	JD.com
Cajepont Technology	Battery Reconditioner	I don't know if it works or not, I'll evaluate it in a few days after I use it.	JD.com
Cajepont Technology	Battery Reconditioner	Beautiful appearance, affordable, strong sense of technology, the power display is clear, charging very quickly, the goods are practical, beautiful appearance, powerful, innovative design, used a few days before the parity, it is very good to use, the phone is hot, lag can be ordered!	JD.com
Cajepont Technology	Battery Reconditioner	Logistics was fast! Good value for money! Novel shape!	JD.com
Cajepont Technology	Battery Reconditioner	The goods have been received, well packaged, to be tried to see how the effect!	JD.com
Cajepont Technology	Battery Reconditioner	Received quickly after ordering, customer service patience and guidance, good quality material! Repair batteries fast!	JD.com
Cajepont Technology	Battery Reconditioner	Received the goods, quite good, looking forward to a while to repair the battery	JD.com
Cajepont Technology	Battery Reconditioner	The goods have been received, the logistics is very fast, the packaging is intact. It is being used and hopefully it will fix my cell phone.	JD.com
Cajepont Technology	Battery Reconditioner	the4 cell phone charging repair, the effect is quite ideal, which has a cell phone has been unable to charge into the electricity, charging10hours10%of the way, last night with this charging repairer2hours or so on the char98% of thespeed of fast at the same time on the cell phone to pull the garbage cleanup and do not Katun, really straight to buy a good product, the full five-star praise!	JD.com
Cajepont Technology	Battery Reconditioner	an excellent	JD.com
Cajepont Technology	Battery Reconditioner	I'm using it now on a charge, charge it a few times and see if it works	JD.com
Cajepont Technology	Battery Reconditioner	Received it and used it! Should be just as the store said it would be! 5 stars!	JD.com
Cajepont Technology	Battery Reconditioner	Received the goods yesterday, the evening phone charged to try, feel charged more slightly faster, but also more secure, continue to experience, should be fixed better.	JD.com
Cajepont Technology	Battery Reconditioner	Received. It's good. I'll comment after I use it.	JD.com
Cajepont Technology	Battery Reconditioner	Battery Reconditioner received, now looks good, can repair the old battery, try and then follow up!	JD.com
Cajepont Technology	Battery Reconditioner	Good product quality, easy to carry, with environmental protection for the battery less pollution of the environment.	JD.com
Cajepont Technology	Battery Reconditioner	This has been received, feels great, fast delivery, will see how it works when I charge it at night.	JD.com
Cajepont Technology	Battery Reconditioner	Fast logistics and good after-sales service	JD.com
Cajepont Technology	Battery Reconditioner	The cell phone battery repairer is easy to use and works well.	JD.com
Cajepont Technology	Battery Reconditioner	I've received it, but I don't know what it's like to repair the battery.	JD.com
Cajepont Technology	Battery Reconditioner	Used two days, feel the quality is still very good, before charging the phone will be hot, after using this repairer there is no heat, the phone is not stuck, in short, purchased this product value, businessmen shipment is fast, courier brother delivery is also timely, must give five stars.	JD.com
Cajepont Technology	Battery Reconditioner	Practical! Worth having!	JD.com

Cajepont Technology	Battery Reconditioner	Tried, because the time is short did not see the effect of it, charging are normal, it should be no problem, I wish the boss prosperous business ah!	JD.com
Cajepont Technology	Battery Reconditioner	can	JD.com
Cajepont Technology	Battery Reconditioner	It's very good. It's a lot better.	JD.com
Cajepont Technology	Battery Reconditioner	Works great, fixes cell phones	JD.com
Cajepont Technology	Battery Reconditioner	ve used2times and it's supposed to be effective and sm	JD.com
Cajepont Technology	Battery Reconditioner	Dad said that the introduction said that it is very good, try it first, the follow-up evaluation	JD.com
Cajepont Technology	Battery Reconditioner	It works great.	JD.com
Cajepont Technology	Battery Reconditioner	Harvest is used, the value of jumping, the surrounding bright flash, feel in the non-stop work, the past charge can only be up to ninety like, now can reach one hundred, and speed up, and then charge a few days certainly have the effect!	JD.com
Cajepont Technology	Battery Reconditioner	After turning it off, I charged my phone and it felt good!	JD.com
Cajepont Technology	Battery Reconditioner	Used it, works well, value for money	JD.com
Cajepont Technology	Battery Reconditioner	Very nice looking Well made Not bad	JD.com
Cajepont Technology	Battery Reconditioner	Works great, feels great, and is very well made	JD.com
Cajepont Technology	Battery Reconditioner	Fast delivery, well-packed, try to feel good, looking forward to the later practical effects	JD.com
Cajepont Technology	Battery Reconditioner	Nice stuff, good material, charges fast.	JD.com
Cajepont Technology	Battery Reconditioner	Product Appearance: very sci-fi<br>Charging Battery: not much change for now<br>Carrying Description: very convenient<br>Product Design: very like<br>Display Experience: display is very clear	JD.com
Cajepont Technology	Battery Reconditioner	carry, repair powerful, cost-effective, inexpensive, has been shared with friends and family, worth recommending and repurchase.	JD.com
Cajepont Technology	Battery Reconditioner	Received a few days to evaluate the goods, the service attitude is very good has been collected store auxiliary	JD.com
Cajepont Technology	Battery Reconditioner	I received the goods and used it, it is more durable than before, and the phone is not so bad.	JD.com
Cajepont Technology	Battery Reconditioner	The logistics is fast, the quality of the product should be tried before evaluating.	JD.com
Cajepont Technology	Battery Reconditioner	Good quality to repair cell phone batteries	JD.com
Cajepont Technology	Battery Reconditioner	Very good. Satisfied.	JD.com
Cajepont Technology	Battery Reconditioner	On the mend, not sure how well it works? After using it, I will come back to make a follow-up comment. After-sales service is very good, trustworthy.	JD.com
Cajepont Technology	Battery Reconditioner	Looks good.	JD.com
Cajepont Technology	Battery Reconditioner	The goods and the description of[smile]in the charging of the data is constantly changing[smile]logistics is also very fast[smile]customer service is warm and thoughtful[smile]this style is also very beautiful[cool]	JD.com
Cajepont Technology	Battery Reconditioner	Goods have been received took home to open to try the effect I do not understand what it means only see the digital display keeps changing[smile]wait foruse of the effect is good then give you a follow-up review	JD.com
Cajepont Technology	Battery Reconditioner	contact is not good, immediately send a replacement, thank you!	JD.com
Cajepont Technology	Battery Reconditioner	This product is the same as described on the video, I received it to try it works great!	JD.com
Cajepont Technology	Battery Reconditioner	The product was received and the logistics was fast. The effect is quite good.	JD.com
Cajepont Technology	Battery Reconditioner	Restorer, received.	JD.com
Cajepont Technology	Battery Reconditioner	Just received the goods, packaging is good, exquisite workmanship, fast logistics, good service, give praise!	JD.com
Cajepont Technology	Battery Reconditioner	This is a real high tech product, with it you don't have to worry about changing batteries!	JD.com
Cajepont Technology	Battery Reconditioner	The goods have arrived[smile], on the use of good will be introduced to friends, I hope it works well,	JD.com
Cajepont Technology	Battery Reconditioner	The shape of the product is unique, the box is intact, the repairer will repair the cell phone battery.	JD.com

Cajepont Technology	Battery Reconditioner	The appearance of the new trend, a sense of weight, try it out first, wait for a few times after charging to see the effect and then follow up.	JD.com
Cajepont Technology	Battery Reconditioner	Commodities received, look and feel good logistics is also very fast service attitude is very good, first give five-star praise, make additional comments after the completion of the	JD.com
Cajepont Technology	Battery Reconditioner	Fast shipping, affordable price, good value for money, 5 stars!	JD.com
Cajepont Technology	Battery Reconditioner	Yes, the first time I used it, I saw results.	JD.com
Cajepont Technology	Battery Reconditioner	Yesterday after receiving the goods to test an old cell phone, repair3hoursand 20minutes, I feel that the Internet Internet speed is slightly faster than before, and then continue to repair in two days, two months after the follow-up evaluation.	JD.com
Cajepont Technology	Battery Reconditioner	Goods received, very good, and the description of the same, value for money, courier boy is also very strong!	JD.com
Cajepont Technology	Battery Reconditioner	The logistics is fast, well packed, and I will comment after using it.	JD.com
Cajepont Technology	Battery Reconditioner	It feels good to use it. Keep it up.	JD.com
Cajepont Technology	Battery Reconditioner	This user did not fill in a rating	JD.com
Cajepont Technology	Battery Reconditioner	Goods have received the quality is very good, will repurchase again!	JD.com
Cajepont Technology	Battery Reconditioner	It's running.	JD.com
Cajepont Technology	Battery Reconditioner	The product has been received as described-the product is trustworthy	JD.com
Cajepont Technology	Battery Reconditioner	Following the instructions will complete the repair.	JD.com
Cajepont Technology	Battery Reconditioner	High-tech products! Digital display! In use! Works well in continue to repurchase reviews!	JD.com
Cajepont Technology	Battery Reconditioner	Received the goods, should be genuine satisfaction	JD.com
Cajepont Technology	Battery Reconditioner	It was received very well packaged with no damage.	JD.com
Cajepont Technology	Battery Reconditioner	The goods have been received.	JD.com
Cajepont Technology	Battery Reconditioner	Feel pretty good Oh, firsttrywith the very old Apple6, the effect is good, if my family Apple10and11are optimized, so you do not have to buy12la!s really good👍	JD.com
Cajepont Technology	Battery Reconditioner	I received it today and I started using it then and it is indeed a good product[kudos][kudos]! Worth having!	JD.com
Cajepont Technology	Battery Reconditioner	Item received, logistics is very fast, try it out, in the chase comment.	JD.com
Cajepont Technology	Battery Reconditioner	Good! This has a data display, look is technology, wait to use some time to appreciate the benefits it brings to the phone, fast delivery, praise!	JD.com
Cajepont Technology	Battery Reconditioner	Fast shipping, pretty good quality, as described by the seller, very satisfied!	JD.com
Cajepont Technology	Battery Reconditioner	properly	JD.com
Cajepont Technology	Battery Reconditioner	Fast logistics, convenient and practical, fast charging!	JD.com
Cajepont Technology	Battery Reconditioner	The stuff is really good, repair the battery is pretty good, need friends go buy it!	JD.com
Cajepont Technology	Battery Reconditioner	Good quality and exceptionally fast collection.	JD.com
Cajepont Technology	Battery Reconditioner	Very good use of logistics is also very fast, 亻, give you praise five red stars	JD.com
Cajepont Technology	Battery Reconditioner	New style and easy to use.	JD.com
Cajepont Technology	Battery Reconditioner	It's good. I just used it. I don't know what it's like.	JD.com
Cajepont Technology	Battery Reconditioner	effect, to be used after the evaluation, looking forward to good results.	JD.com
Cajepont Technology	Battery Reconditioner	The item is very good, the logistics is fast, honoring the sincerity of the promise, deserves praise.	JD.com
Cajepont Technology	Battery Reconditioner	Battery Repairer received, logistics is also very fast, used a few days is very good, with the business description of the same, the battery is durable, give praise.	JD.com
Cajepont Technology	Battery Reconditioner	Baby has received quite good and match the description of the seller's good service attitude fast shipping!	JD.com
Cajepont Technology	Battery Reconditioner	I got it back and started fixing a cell phone. I won't know the results until a few days later.	JD.com
Cajepont Technology	Battery Reconditioner	The logistics is fast, the instrument is small and compact, and the quality is good.	JD.com

Cajepont Technology	Battery Reconditioner	Small and exquisite, repairing cell phone batteries, a good helper for cell phone batteries.	JD.com
Cajepont Technology	Battery Reconditioner	I just got it. It feels good.	JD.com
Cajepont Technology	Battery Reconditioner	I've received the goods, let's use it first	JD.com
Cajepont Technology	Battery Reconditioner	Received it and used it for two days! The battery does have a little more standby time than before I didn't use it!	JD.com
Cajepont Technology	Battery Reconditioner	Design evaluation: the first time to see how to use, there are instructions to look at the will be	JD.com
Cajepont Technology	Battery Reconditioner	Commodities received, the appearance of the exact same as described, is testing, if indeed the same as the business said, and then follow up, logistics is very fast service attitude is good, give five-star praise.	JD.com
Cajepont Technology	Battery Reconditioner	Fast logistics, good attitude, fine packaging, immediately use, after use7Ncomments	JD.com
Cajepont Technology	Battery Reconditioner	Baby just received and used it right away, charging is four times faster.	JD.com
Cajepont Technology	Battery Reconditioner	Express received, very fast charging is also quite good results	JD.com
Cajepont Technology	Battery Reconditioner	Received the goods, logistics is very fast, customer service is also very patient, very enthusiastic, tell how to use the detailed steps, the effect must be good!	JD.com
Cajepont Technology	Battery Reconditioner	Good quality, fast logistics and good service.	JD.com
Cajepont Technology	Battery Reconditioner	Very good quality, fixes cell phone batteries fast!	JD.com
Cajepont Technology	Battery Reconditioner	I don't know if it's working yet. It's charging.	JD.com
Cajepont Technology	Battery Reconditioner	Repairer is indeed very useful, just bought back and used once, the phone used to feel the battery life a lot longer, very recommended to use!	JD.com
Cajepont Technology	Battery Reconditioner	Things received a good use, logistics fast quality, good service praise	JD.com
Cajepont Technology	Battery Reconditioner	Goods have been received, the product quality is good, I'm this is a repurchase, worth promoting to give you praise!	JD.com
Cajepont Technology	Battery Reconditioner	Cell phone battery repairer has been received to try a moment to try a few more times to say!	JD.com
Cajepont Technology	Battery Reconditioner	Good quality, as advertised, logistics is also fast, back to start using, the use of the effect will be evaluated later!	JD.com
Cajepont Technology	Battery Reconditioner	The product has been received, tried it, very good, good cell phone battery repair, and there are voltage and current display, really good.	JD.com
Cajepont Technology	Battery Reconditioner	Received, the logistics is very fast, the courier boy good service attitude, this is very satisfied!	JD.com
Cajepont Technology	Battery Reconditioner	This is a great way to fix your cell phone battery! The battery is charged for hours!	JD.com
Cajepont Technology	Battery Reconditioner	Really useful, I a Pingguo, a Hua办, used formore than3years, would have to charge every day, with the battery repairer two days before charging, good goods!	JD.com
Cajepont Technology	Battery Reconditioner	Received, very good quality, satisfied with the use, praise, praise, praise.	JD.com
Cajepont Technology	Battery Reconditioner	Small and compact very good giving 5 stars.	JD.com
Cajepont Technology	Battery Reconditioner	delivery, packaging is very good, logistics is also very fast, five-star praise to a, the use of good results and then additional comments.	JD.com
Cajepont Technology	Battery Reconditioner	The goods have been received, the logistics is very fast, as the merchant said	JD.com
Cajepont Technology	Battery Reconditioner	every penny, I chose this more expensive one. I hope it's worth it.	JD.com
Cajepont Technology	Battery Reconditioner	Logistics is very fast, the quality is good use good next time to come back	JD.com
Cajepont Technology	Battery Reconditioner	Battery restorer received. Good quality, good price. Logistics is also very fast, kudos.	JD.com
Cajepont Technology	Battery Reconditioner	Received the cell phone battery repairer is great, and the business description of the same, thanks to the business, thanks to customer service, thanks to the courier!	JD.com
Cajepont Technology	Battery Reconditioner	Portable, inexpensive, and a godsend for fixing batteries on your own.	JD.com
Cajepont Technology	Battery Reconditioner	Good stuff received the goods to use after the evaluation	JD.com
Cajepont Technology	Battery Reconditioner	We'll talk about it after we use it.	JD.com
Cajepont Technology	Battery Reconditioner	The restorer is great, with a five year warranty, so feel free to use it.	JD.com
Cajepont Technology	Battery Reconditioner	I'm trying it out and will comment later	JD.com

Cajepont Technology	Battery Reconditioner	I've got the goods.	JD.com
Cajepont Technology	Battery Reconditioner	That's good!	JD.com
Cajepont Technology	Battery Reconditioner	continue to follow the instructions and then use a few times, really can achieve the desired effect, but also really save time and money.	JD.com
Cajepont Technology	Battery Reconditioner	I just received the item, tried it out and the results are still unknown.	JD.com
Cajepont Technology	Battery Reconditioner	I'll review it later.	JD.com
Cajepont Technology	Battery Reconditioner	Cell phone battery repair, buy and try how effective	JD.com
Cajepont Technology	Battery Reconditioner	The stuff is good and affordable. Save on battery replacement. The phone does not get stuck and the logistics are fast. Shipped on the same day.	JD.com
Cajepont Technology	Battery Reconditioner	Goods have been received, can charge the quality is very good	JD.com
Cajepont Technology	Battery Reconditioner	The baby is quite good, I like the charging is also very fast. Packaging is also very good	JD.com
Cajepont Technology	Battery Reconditioner	Very good product powering up with display pulse powering up	JD.com
Cajepont Technology	Battery Reconditioner	Very satisfied with the product Digital Function Display The streamlined look of the car.	JD.com
Cajepont Technology	Battery Reconditioner	The cell phone battery recovery is good and recommendable to buy.	JD.com
Cajepont Technology	Battery Reconditioner	It's good, the logistics is quite fast	JD.com
Cajepont Technology	Battery Reconditioner	Things received, good-looking, well-packed, as described, worth recommending the purchase.	JD.com
Cajepont Technology	Battery Reconditioner	The logistics is fast, the stuff is really good, and it's recommendable.	JD.com
Cajepont Technology	Battery Reconditioner	This cell phone repairer is small, exquisite appearance, easy to use, worth buying.	JD.com
Cajepont Technology	Battery Reconditioner	packaging is very good, received immediately to the cell phone battery repair for three hours, feel the phone mom like clear, thanks to customer service is not afraid of trouble to answer Yan how to plug in the original how to use, so give five-star praise.	JD.com
Cajepont Technology	Battery Reconditioner	The goods have been received, fast shipping, tightly packaged, must praise!!!!	JD.com
Cajepont Technology	Battery Reconditioner	That's good[Smiles]	JD.com
Cajepont Technology	Battery Reconditioner	丶幻单确认收货全给5star好评	JD.com
Cajepont Technology	Battery Reconditioner	Fast logistics, good service attitude, fine products, compact Linglong.	JD.com
Cajepont Technology	Battery Reconditioner	The quality of the product is very good, the idea is good, the service	JD.com
Cajepont Technology	Battery Reconditioner	Fast logistics Beautiful appearance Good service	JD.com
Cajepont Technology	Battery Reconditioner	Quite a cool product with stable voltage output	JD.com
Cajepont Technology	Battery Reconditioner	Looked good👍equipment exquisite good-looking, easy to use. Just do not know how the effect, wait until after the use of evaluation Oh[smile]look forward to the effect Oh!	JD.com
Cajepont Technology	Battery Reconditioner	Not sure about the fix for now, use it for a while and see[cool]	JD.com
Cajepont Technology	Battery Reconditioner	Cell phone battery repair summerizer received. Use it to see how the effect is, but then again, it is very good. Good service, fast logistics, five-star praise.	JD.com
Cajepont Technology	Battery Reconditioner	Cell phone battery repairer received, the product packaging is exquisite, the appearance of beautiful quality is very good.	JD.com
Cajepont Technology	Battery Reconditioner	Charger is very bright atmosphere atmosphere things also fast should be a good charger	JD.com
Cajepont Technology	Battery Reconditioner	The product is pretty good, good experience with my phone.	JD.com
Cajepont Technology	Battery Reconditioner	Received, used well recommended to friends	JD.com
Cajepont Technology	Battery Reconditioner	Received it, it's great, trying it out.	JD.com
Cajepont Technology	Battery Reconditioner	Role experience: good<br>Display experience: very good<br>Appearance description: good-looking<br>Design evaluation: good<br>Rechargeable battery: should be good	JD.com
Cajepont Technology	Battery Reconditioner	The quality of this product is guaranteed to be good and inexpensive, so you can trust and buy the product.	JD.com

Cajepont Technology	Battery Reconditioner	Received [Universal] cell phone battery repairer to extend battery life optimize the system without lagging voltage regulator charger, the use of the results, it is really great[kudos] [clap] [handshake] [shake]	JD.com
Cajepont Technology	Battery Reconditioner	This stuff is great, I've already recommended it to my friends	JD.com
Cajepont Technology	Battery Reconditioner	This is a good product. Kudos.	JD.com
Cajepont Technology	Battery Reconditioner	This product is very satisfied very practical	JD.com
Cajepont Technology	Battery Reconditioner	Tried it out for a day it seems like the range is a little longer, so I'll see if I can use it again for a while.	JD.com
Cajepont Technology	Battery Reconditioner	of repairing, hope it is a good experience, looking forward to it!	JD.com
Cajepont Technology	Battery Reconditioner	Very good use,, really good, logistics is also very fast very satisfied	JD.com
Cajepont Technology	Battery Reconditioner	Cell phone battery repairer received, well-packaged, good style and quality, small and exquisite very practical, the cell phone battery to play a role in repairing and protecting the role of the phone, it is worth buying, the logistics courier is very timely, give five-star praise.	JD.com
Cajepont Technology	Battery Reconditioner	Very interesting stuff to protect your phone battery kudos for bookmarking.	JD.com
Cajepont Technology	Battery Reconditioner	The goods have been received, the packaging is intact, logistics is very fast.<br>Cell phone battery repairer, beautiful appearance, easy to use, good quality, fast repair, five-star praise!	JD.com
Cajepont Technology	Battery Reconditioner	The product is good!	JD.com
Cajepont Technology	Battery Reconditioner	Battery Repairer received, the product appearance exquisite, first give five-star praise, service to see the effect of giving a follow-up.	JD.com
Cajepont Technology	Battery Reconditioner	The goods have been received, the packaging is intact, the product quality is very good. The logistics is very fast, the service attitude is very good	JD.com
Cajepont Technology	Battery Reconditioner	Sent quickly, well-packed, charging repairer appearance of good workmanship, just when the phone battery is not durable, see if there is no use.	JD.com
Cajepont Technology	Battery Reconditioner	Things have been received without damage such as pieces, repair device work is very peculiar, feel very good, there is also a display, for the genuine, to be used after the follow-up comment, to the merchant praise, five-star praise.	JD.com
Cajepont Technology	Battery Reconditioner	The outside off is very delicate, generous and beautiful. It is worth buying!	JD.com
Cajepont Technology	Battery Reconditioner	Super good use, logistics is fast, the merchants keep their word, worth buying!	JD.com
Cajepont Technology	Battery Reconditioner	Works very well, excellent quality.	JD.com
Cajepont Technology	Battery Reconditioner	I don't know if it works well.	JD.com
Cajepont Technology	Battery Reconditioner	The product is compact, affordable, durable and effective! Good value for money. Shipping is timely and on point.	JD.com
Cajepont Technology	Battery Reconditioner	Bought this charger to repair my cell phone battery. Not using it now, don't know how it is yet. After using it, I'll give you a follow up review. First give a 5-star praise.	JD.com
Cajepont Technology	Battery Reconditioner	It's good and affordable.	JD.com
Cajepont Technology	Battery Reconditioner	It's great, I'll give it a good review first and add a positive review later.	JD.com
Cajepont Technology	Battery Reconditioner	view, found that the outer packaging (box) is intact, the repair device is also very good, no damage phenomenon, as for how to repair the effect, to be used to follow up, very satisfied with a shopping.	JD.com
Cajepont Technology	Battery Reconditioner	I don't know if it's okay.	JD.com
Cajepont Technology	Battery Reconditioner	The item was received in time, logistics was fast and satisfactory.	JD.com
Cajepont Technology	Battery Reconditioner	It's so good! Solves phone lagging problems!	JD.com
Cajepont Technology	Battery Reconditioner	Goods and business publicity and promotion of the same, fast logistics, business service is good.	JD.com
Cajepont Technology	Battery Reconditioner	I hope it works[cool]	JD.com
Cajepont Technology	Battery Reconditioner	It charges quickly and the battery is durable.	JD.com
Cajepont Technology	Battery Reconditioner	Good goods are using the logistics is very fast next time to buy!	JD.com
Cajepont Technology	Battery Reconditioner	Good service attitude, received the goods immediately tried, charging quickly, good use	JD.com

Cajepont Technology	Battery Reconditioner	Cell phone battery repairer received quality very good logistics fast next time to buy again	JD.com
Cajepont Technology	Battery Reconditioner	I just received it and will comment after I use it.	JD.com
Cajepont Technology	Battery Reconditioner	In the first charging, do not know if it is good or not in the back in the additional comments.	JD.com
Cajepont Technology	Battery Reconditioner	Tried, the quality is very good, logistics is also very fast, the business attitude is good everyone rest assured to buy it!	JD.com
Cajepont Technology	Battery Reconditioner	days, the phone battery improved quite a lot, is a good product!	JD.com
Cajepont Technology	Battery Reconditioner	Goods have been received logistics quickly and good quality	JD.com
Cajepont Technology	Battery Reconditioner	I've been using it since I got it back. I really hope it works.	JD.com
Cajepont Technology	Battery Reconditioner	The results are great!	JD.com
Cajepont Technology	Battery Reconditioner	Baby got it. It looks good.	JD.com
Cajepont Technology	Battery Reconditioner	solid, the repair effect will come back to the review after the test	JD.com
Cajepont Technology	Battery Reconditioner	The logistics is very fast and the customer service after-sales attitude is very good.	JD.com
Cajepont Technology	Battery Reconditioner	The Electric Fan Repairer has been collected. Very cry gas, feel Ming Rong Qi Hall's also very elastic fine.	JD.com
Cajepont Technology	Battery Reconditioner	Charge protection device has been received, installed after charging very satisfied! Able to protect the phone battery is very good and practical, worth buying five stars and beautiful appearance with digital display very satisfied!	JD.com
Cajepont Technology	Battery Reconditioner	Due to busy courier guy put it in the delivery box didn't have time to pick it up on the day. After two days to get. To charge[stay][skimming][skimming][skimming][skimming][skimming]to get back to plug to the charging treasure light is normal. I hope it works to repair the battery	JD.com
Cajepont Technology	Battery Reconditioner	Can, quality can, cell phone repair electric board	JD.com
Cajepont Technology	Battery Reconditioner	It's nice and compact, and it's a great fix.	JD.com
Cajepont Technology	Battery Reconditioner	[Lots of love][Lots of love]	JD.com
Cajepont Technology	Battery Reconditioner	The product is exquisitely packaged, easy and quick to use, and has outstanding functionality. Value for money.	JD.com
Cajepont Technology	Battery Reconditioner	Cell phone battery repairer received to use four hours, now do not know how effective, with a period of time in the follow-up, customer attitude is very good point👍	JD.com
Cajepont Technology	Battery Reconditioner	Item received try it works great thanks to seller service.	JD.com
Cajepont Technology	Battery Reconditioner	shipping quite fast thanks, in the jitterbug inside to see this repairer is very useful and convenient, received the goods plugged in all normal, repair the phone is not a day or two, rushed for a period of time in order to have the effect, looking forward to.	JD.com
Cajepont Technology	Battery Reconditioner	Let's use it first.	JD.com
Cajepont Technology	Battery Reconditioner	save money to buy, do not hesitate Baby is very good, I have to say, the customer service attitude is also too good, right! A very good shopping experience, attentive service, fast logistics, delivery is also very thoughtful, attitude is also very good, praise!	JD.com
Cajepont Technology	Battery Reconditioner	Satisfied with the item, good value, economical and timely delivery.	JD.com
Cajepont Technology	Battery Reconditioner	Baby received, very satisfied, thank you pro.	JD.com
Cajepont Technology	Battery Reconditioner	It works.	JD.com
Cajepont Technology	Battery Reconditioner	Batteries are dead in the house, gotta take out the phones that don't work one by one and check them!	JD.com
Cajepont Technology	Battery Reconditioner	The long awaited thing finally received, how long customer service help!	JD.com
Cajepont Technology	Battery Reconditioner	I wonder how it works?	JD.com
Cajepont Technology	Battery Reconditioner	goods years ago, the courier company to get somewhere else to get back.	JD.com
Cajepont Technology	Battery Reconditioner	Great Great Cheap Try it out and come back for more	JD.com

Cajepont Technology	Battery Reconditioner	The product packaging is exquisite, beautiful appearance, good quality, clear and accurate digital display, the trial of the cell phone battery has a certain repair effect, easy to use, the phone life time is obviously long, very practical sense of technology, it is worth recommending!	JD.com
Cajepont Technology	Battery Reconditioner	The real thing matches the picture.	JD.com
Cajepont Technology	Battery Reconditioner	used, no evaluation of the most excellent merchants and product quality assiduous, fast shipping, service attitude communication is very good, thank you, please. [hugs] [hugs] [hugs] [hugs] [kudos] [kudos][kudos	JD.com
Cajepont Technology	Battery Reconditioner	Logistics is very fast, the price is affordable, the stuff is very good, five stars, next time to come back!	JD.com
Cajepont Technology	Battery Reconditioner	The goods have been received and the logistics is very fast	JD.com
Cajepont Technology	Battery Reconditioner	I received this product and tried it with it, it's very practical and useful for cell phones.	JD.com
Cajepont Technology	Battery Reconditioner	Fast logistics, good attitude, will recommend friends to buy, good quality, good price, effective, good looking, cost-effective, good use.	JD.com
Cajepont Technology	Battery Reconditioner	[heehee] [] [hee] [hee]cell phone charging repairer received, logistics is very fast well-packed, I wish business is prosperous, the financial resources of the retailers much more	JD.com
Cajepont Technology	Battery Reconditioner	This product is great, if you need it, buy it quickly!	JD.com
Cajepont Technology	Battery Reconditioner	Received the goods, logistics is very fast, back to try quite good, worth buying.	JD.com
Cajepont Technology	Battery Reconditioner	Logistics is very fast, the item is exquisite, after getting the goods and try to feel very good, customer service personnel attitude is very good	JD.com
Cajepont Technology	Battery Reconditioner	Good quality of goods, details, quality, workmanship are very good, shipping thief, express delivery, great~❀❀❀❀❀	JD.com
Cajepont Technology	Battery Reconditioner	phone battery has been undercharged, each time only charged to90%can not be charged, now finally can be fully charged, adhere to the requirements to do, looking forward to better results.	JD.com
Cajepont Technology	Battery Reconditioner	Battery Repairer received, well-packaged, good-looking, data at a glance, I hope it can be useful to repair the battery, customer service attitude is very good, logistics is fast.	JD.com
Cajepont Technology	Battery Reconditioner	Great purchase received great quality!	JD.com
Cajepont Technology	Battery Reconditioner	With it, my Huawei and Apple phones are considered beneficial, from full charge to99%is about ten minutes longer than before, which can be given a good review!	JD.com
Cajepont Technology	Battery Reconditioner	During use, the punch function looks like an authentic product!	JD.com
Cajepont Technology	Battery Reconditioner	Cell phone battery repairer received, I used it to repair the glory and Apple, used3days to evaluate, off the charge is really effective! Merchants shipping very quickly logistics is also fast, five-star praise!	JD.com
Cajepont Technology	Battery Reconditioner	It's pretty good, good value for money. Extremely easy to use, recommend buying	JD.com
Cajepont Technology	Battery Reconditioner	Did not start the baby, you can place an order to buy a to try, repair your second wife, female second husband cutie, cell phone📱battery repairer, repair battery and lagging, October listing of the artifacts, hahaha😄	JD.com
Cajepont Technology	Battery Reconditioner	Merchant shipped quickly, good value, 5 stars.	JD.com
Cajepont Technology	Battery Reconditioner	The logistics is fast, the quality of the stuff is awesome, and it's in use.	JD.com
Cajepont Technology	Battery Reconditioner	the instructions are very detailed. Logistics is very fast, praise.	JD.com
Cajepont Technology	Battery Reconditioner	will definitely follow up with a review if it works well! Recommended!	JD.com
Cajepont Technology	Battery Reconditioner	described. Great quality, great logistics, great customer service, great.	JD.com
Cajepont Technology	Battery Reconditioner	effect to be a few days to know, so in the past ten days to follow up!	JD.com
Cajepont Technology	Battery Reconditioner	Nice baby, fine workmanship, arrived on the use of , looking good, the seller shipped quickly,5star praise of the	JD.com
Cajepont Technology	Battery Reconditioner	couldn't wait to test the goods when I received them, now charging🔋phone is not hot; it's a genuine product worthy of recommendation and praise!	JD.com
Cajepont Technology	Battery Reconditioner	rare	JD.com
Cajepont Technology	Battery Reconditioner	The quality is very good, thank you merchant, thank you so much.	JD.com

Cajepont Technology	Battery Reconditioner	Well just received expecting results, waiting for the repair to work!	JD.com
Cajepont Technology	Battery Reconditioner	really works, I thought there was no saving, my OPPO is now barreling along.one back with the intention of trying it out, followed the tutorial and it really worked, the battery lasted twice as long and last 3 weeks, really great deal	JD.com
Cajepont Technology	Battery Reconditioner	to change the battery, after repairing the phone with a longer time some, and does not heat up, and also automatically clean up the phone system cache garbage, the phone has a significantly smooth, the boss said that often used to repair will be better.	JD.com
Cajepont Technology	Battery Reconditioner	So far it works fine, charges quickly, looks great and is well made.	JD.com
Cajepont Technology	Battery Reconditioner	The cell phone battery automatically shuts off when it reaches 20%, I hope to improve the battery	JD.com
Cajepont Technology	Battery Reconditioner	Goods received! Very good quality👍[kudos][kudos][kudos[hugs][hugs][hugs]	JD.com
Cajepont Technology	Battery Reconditioner	Cell phone repairer received, tried it out, asked the merchant to use it after a few times to be effective, and if it doesn't work 90 days return. Warranty period 5 years.[Kudos]	JD.com
Cajepont Technology	Battery Reconditioner	Charge Fix feels pretty good and likes it. Use a few days to see the effect. Thank you for the customer service enthusiastic service, five-star praise!	JD.com
Cajepont Technology	Battery Reconditioner	Because the product I bought was not a brand that broke after a few days, this one will be reviewed after a while	JD.com
Cajepont Technology	Battery Reconditioner	The smart phone battery repairer works very well. The logistics is very fast. The service attitude is very good.	JD.com
Cajepont Technology	Battery Reconditioner	Cell phone repairer received immediately try to charge fast, and then repair in.	JD.com
Cajepont Technology	Battery Reconditioner	This one's okay. It works just like he said it would.	JD.com
Cajepont Technology	Battery Reconditioner	I'll give you 5 stars for now and follow up if the fix doesn't work out	JD.com
Cajepont Technology	Battery Reconditioner	First time buyer, used it right after receiving it, felt like the phone lasted a little longer than it should have, and the temperature of the phone came down while I was using it, so I'm sure it will be better in a few weeks! Giving five stars!	JD.com
Cajepont Technology	Battery Reconditioner	Express delivery arrived on time! Baby received! Experience it first and then follow up! Congratulations!	JD.com
Cajepont Technology	Battery Reconditioner	Good logistics	JD.com
Cajepont Technology	Battery Reconditioner	Cell phone repairer received, the ability to repair the battery and charging speed is very fast, the quality is very good, very practical, repair effect is very good, prolong the life, can be suitable for a variety of models of cell phone repair, the future no longer need to change the battery often, good value for money, you can rest assured that the purchase!	JD.com
Cajepont Technology	Battery Reconditioner	to use that has good results, reflecting quickly, screen clarity has improved. Kudos to science and technology innovation.	JD.com
Cajepont Technology	Battery Reconditioner	It's okay it works fine, it's okay it's practical	JD.com
Cajepont Technology	Battery Reconditioner	Pro, the loan received, beautiful fine quality of the little baby, the packaging is intact, the boss service attitude is also quite good, thank you boss good products, wish you prosperous business💯💰💰💰💰💯	JD.com
Cajepont Technology	Battery Reconditioner	This user feels the product is better	JD.com
Cajepont Technology	Battery Reconditioner	The quality of goods is very good, logistics is quite fast, very satisfied!	JD.com
Cajepont Technology	Battery Reconditioner	Received, clear values, simple and easy to use, with three charging heads, more thoughtful.	JD.com
Cajepont Technology	Battery Reconditioner	This user feels the product is better	JD.com
Cajepont Technology	Battery Reconditioner	Goods received good quality, good results I am very satisfied!	JD.com
Cajepont Technology	Battery Reconditioner	time<br>Compatibility: can work with any charging head<br>Charging speed: quite fast<br>Safety: automatic disconnection after charging	JD.com
Cajepont Technology	Battery Reconditioner	The product is as described by the merchant, charging quickly, good quality, kudos.	JD.com
Cajepont Technology	Battery Reconditioner	Battery life is extended	JD.com
Cajepont Technology	Battery Reconditioner	Customer service is slow to reply, what is asked is not answered, this cell phone fixer basically does not work, very unhappy to receive it, asked to return it for a refund	JD.com

Cajepont Technology	Battery Reconditioner	Cell phone repairer is very good logistics very quickly I tried to give with five-star praise	JD.com
Cajepont Technology	Battery Reconditioner	Baby has been received, better than expected, customer service is also very good, looking forward to the battery recovery	JD.com
Cajepont Technology	Battery Reconditioner	Quality and price, packaging is very good, logistics is very fast, after use and then follow up!	JD.com
Cajepont Technology	Battery Reconditioner	The goods have been received, have the effect then update the follow-up evaluation	JD.com
Cajepont Technology	Battery Reconditioner	Can't tell if it's working or not	JD.com
Cajepont Technology	Battery Reconditioner	Nothing works! Used the batteries just like the original, they don't last long	JD.com
Cajepont Technology	Battery Reconditioner	This user thinks the item is great and gives it a 5 star rating.	JD.com
Cajepont Technology	Battery Reconditioner	Goods have been received very satisfied with the quality of good trustworthy conscientious business is in use	JD.com
Cajepont Technology	Battery Reconditioner	This user feels the product is better	JD.com
Cajepont Technology	Battery Reconditioner	dissatisfied	JD.com
Cajepont Technology	Battery Reconditioner	It works.	JD.com
Cajepont Technology	Battery Reconditioner	Cell phone battery repairer is very good, after the phone can be used for a long time, can extend the battery usage, very cost-effective, worth buying!	JD.com
Cajepont Technology	Battery Reconditioner	I got it back and started to try it out, I'm in the process of fixing it, let's see how it works out after the fix!	JD.com
Cajepont Technology	Battery Reconditioner	after use; logistics service is also very good, I am very satisfied!	JD.com
Cajepont Technology	Battery Reconditioner	I'll tell you when I've used it.	JD.com
Cajepont Technology	Battery Reconditioner	Just came back beautifully packaged and sized but hopefully the battery health will be back to 100% sooner than later	JD.com
Cajepont Technology	Battery Reconditioner	It's very effective.	JD.com
Cajepont Technology	Battery Reconditioner	It's good, it's very good. Buy it with confidence.	JD.com
Cajepont Technology	Battery Reconditioner	I used it when I received it and expect it to work well.	JD.com
Cajepont Technology	Battery Reconditioner	Very good, using it right after receiving it, great customer service, very satisfied.	JD.com
Cajepont Technology	Battery Reconditioner	one night of use, use it for a few more days and see what happens	JD.com
Cajepont Technology	Battery Reconditioner	First time purchasing this product, hopefully it will be great!	JD.com
Cajepont Technology	Battery Reconditioner	an excellent	JD.com
Cajepont Technology	Battery Reconditioner	Beautiful appearance, fast charging, fine workmanship, clear display	JD.com
Cajepont Technology	Battery Reconditioner	Safety: very good	JD.com
Cajepont Technology	Battery Reconditioner	Cell phone repairer received, logistics is very fast, home delivery, good service! Before using it to consult with customer service, customer service is very enthusiastic and patient to give guidance and help, thank you very much! With this repair machine, the future of the phone can be well maintained, extend the life of the battery, may the cell phone repair machine for the public to make a greater contribution!	JD.com
Cajepont Technology	Battery Reconditioner	I've been using it for a week and it's not working.	JD.com
Cajepont Technology	Battery Reconditioner	phone is not as hot as before, thank you boss for inventing this product.	JD.com
Cajepont Technology	Battery Reconditioner	Baby received, well-packed, fast delivery, thanks to all involved, you hard la🙏🙏look forward to good results, I wish the boss business is booming, prosperity.👍👍👍👍👍⭐⭐⭐⭐⭐🎉🎉🌹🌹🤗🤗👌	JD.com
Cajepont Technology	Battery Reconditioner	Already in use. Hope it's as good as the reviews. Able to change. Battery drain issue with the phone. Go for it!	JD.com
Cajepont Technology	Battery Reconditioner	I wonder if it comes with a restoration? The quality is very good! 5 star reviews!	JD.com
Cajepont Technology	Battery Reconditioner	The product is good!	JD.com
Cajepont Technology	Battery Reconditioner	It's good. I'm charging my old cell phone.	JD.com
Cajepont Technology	Battery Reconditioner	have started to use, the effect is good, charged once, it feels longer than before, according to the instructions, more than once on the increase of half an hour, should be able to, trustworthy products	JD.com

Cajepont Technology	Battery Reconditioner	Cell phone repairer received, looks delicate, compact, good quality, very good[Kudos] [Kudos] [Kudos] [Kudos][Kudos	JD.com
Cajepont Technology	Battery Reconditioner	Goods have been received, the quality is very good. Express,customer service attitude is very good.	JD.com
Cajepont Technology	Battery Reconditioner	[cool]It worked well, and the recovery was pretty quick	JD.com
Cajepont Technology	Battery Reconditioner	I just got it and I'll comment after I use it I think it's good	JD.com
Cajepont Technology	Battery Reconditioner	Workmanship quality is very very good, the boss has a good service attitude, looking forward to using the results.	JD.com
Cajepont Technology	Battery Reconditioner	It's still okay to look at, but I haven't used it yet	JD.com
Cajepont Technology	Battery Reconditioner	Cell phone repairer received, yesterday tried, feel pretty good than the previous smooth quite a lot!	JD.com
Cajepont Technology	Battery Reconditioner	address, test standards, production date and other certificates of conformity:QCpassed. It is genuine, worth buying	JD.com
Cajepont Technology	Battery Reconditioner	Nice purchase, hopefully it will return to its normal charging speed.	JD.com
Cajepont Technology	Battery Reconditioner	Battery charging: received it and used it, good, satisfactory	JD.com
Cajepont Technology	Battery Reconditioner	Currently in normal use	JD.com
Cajepont Technology	Battery Reconditioner	I haven't felt the effect yet.	JD.com
Cajepont Technology	Battery Reconditioner	Logistics is fast. The product is good. Repair is also good.	JD.com
Cajepont Technology	Battery Reconditioner	well, well, well, well, well, well, well, well, well, well, well.	JD.com
Cajepont Technology	Battery Reconditioner	Durable battery after charging	JD.com
Cajepont Technology	Battery Reconditioner	So far it works fine, charges quickly, looks great and is a great buy!	JD.com
Cajepont Technology	Battery Reconditioner	This user feels the product is better	JD.com
Cajepont Technology	Battery Reconditioner	The cell phone repairer has been received, not yet used, after the use of follow-up comments. Prompt shipment and delivery by courier boy! Attentive service! Hard work! Five star rating!	JD.com
Cajepont Technology	Battery Reconditioner	Very good. Good value for money.	JD.com
Cajepont Technology	Battery Reconditioner	Baby received, just got home and used up, feel very good, charging can be,[praise][praise]praisepraise[praise] [praise] [praise] [praise] [looks like the small baby is good, satisfied	JD.com
Cajepont Technology	Battery Reconditioner	Very good, restored like new batteries	JD.com
Cajepont Technology	Battery Reconditioner	Good product quality	JD.com
Cajepont Technology	Battery Reconditioner	Product appearance: beautiful<br>Hand experience: very good<br>Battery charging: fast	JD.com
Cajepont Technology	Battery Reconditioner	Use it first, first payoff try, thanks to the merchant, 5 stars!	JD.com
Cajepont Technology	Battery Reconditioner	The quality of the product is very good	JD.com
Cajepont Technology	Battery Reconditioner	This user feels the product is better	JD.com
Cajepont Technology	Battery Reconditioner	Received the battery charge restorer. Immediately used once. Charging quickly, cell phone battery repair has effect, give five stars praise	JD.com
Cajepont Technology	Battery Reconditioner	Satisfied with a purchase.	JD.com
Cajepont Technology	Battery Reconditioner	quickly, battery durability, comfortable feel, fine workmanship, beautiful shape, functional and practical, beautiful items	JD.com
Cajepont Technology	Battery Reconditioner	Cell phone repair artifact received, logistics is very fast, the design is small and beautiful, try to use good quality, screen clear, very like.	JD.com
Cajepont Technology	Battery Reconditioner	I bought the$57.24one, after charging my phone, it's much faster than before, I used to get stuck a lot, but now I don't have to change my phone, thanks to the store.	JD.com
Cajepont Technology	Battery Reconditioner	This user feels the product is better	JD.com
Cajepont Technology	Battery Reconditioner	Great quality, easy to use, right price, good repair, very satisfied.	JD.com
Cajepont Technology	Battery Reconditioner	We'll see how it goes.	JD.com
Cajepont Technology	Battery Reconditioner	is very obvious, the phone is no longer lagging, it is worth having.	JD.com

Cajepont Technology	Battery Reconditioner	The product is well packaged, small and fine, the effect to be good!	JD.com
Cajepont Technology	Battery Reconditioner	Use it for a while and then follow up	JD.com
Cajepont Technology	Battery Reconditioner	It's consistent with the ads and it's real and effective.	JD.com
Cajepont Technology	Battery Reconditioner	Received the goods in the try to fix the charger, see	JD.com
Cajepont Technology	Battery Reconditioner	Plugging this in gives me fast internet speeds.	JD.com
Cajepont Technology	Battery Reconditioner	This product works great! I've been wanting to buy one for a long time! I bought it today! I'll be using it a lot!	JD.com
Cajepont Technology	Battery Reconditioner	It is as described and looks good.	JD.com
Cajepont Technology	Battery Reconditioner	Good appearance, still trying, fast logistics	JD.com
Cajepont Technology	Battery Reconditioner	Good quality, good charging repair, straight to buy, suggest the need can buy this product, the	JD.com
Cajepont Technology	Battery Reconditioner	Good use is the cell phone clean, has a lot of space network faster to receive the収很快, is that I have no worries, thank you for putting out such a good product. Thank you again.	JD.com
Cajepont Technology	Battery Reconditioner	The packaging is intact, small, convenient and practical, and will come back if needed!	JD.com
Cajepont Technology	Battery Reconditioner	Very well packed, fast logistics, very satisfied!	JD.com
Cajepont Technology	Battery Reconditioner	Fast logistics, beautiful appearance, received today to try, see how effective. Just wait a few days.	JD.com
Cajepont Technology	Battery Reconditioner	The stuff was delivered quickly, have not yet used it, use it and then add evaluation	JD.com
Cajepont Technology	Battery Reconditioner	I've been using it for more than 10 days now, but the power is still dropping, so it seems that I've paid for my IQ sleep	JD.com
Cajepont Technology	Battery Reconditioner	Product received, fast charging. Good quality.	JD.com
Cajepont Technology	Battery Reconditioner	The cell phone has been used for five or six years, the battery is not good, I wanted to change the battery, accidentally saw this ad, so I bought it to try.	JD.com
Cajepont Technology	Battery Reconditioner	Cell phone battery repairer packaging is very good, the effect of repair is yet to be proved. First give five-star praise.	JD.com
Cajepont Technology	Battery Reconditioner	This user did not fill in a rating	JD.com
Cajepont Technology	Battery Reconditioner	The delivery service is top notch and the equipment is optimized for the benefit of the people! Awesome!👍	JD.com
Cajepont Technology	Battery Reconditioner	It looks good, I don't know how effective it is, I'll wait for some time to use it and then give a good review[hee hee]	JD.com
Cajepont Technology	Battery Reconditioner	serviceable	JD.com
Cajepont Technology	Battery Reconditioner	very good, shipping quickly, well packaged, intact, small and exquisite, inexpensive, affordable, and the picture description is consistent with the store's good service attitude, never tire of asking. Pros in need! Hurry up to place an order.	JD.com
Cajepont Technology	Battery Reconditioner	The item is beautiful, practical and effective with good functionality.	JD.com
Cajepont Technology	Battery Reconditioner	use, faster than fast charging, good-looking, atmosphere, the courier brother speed, for the boss and fast brother brother praise!	JD.com
Cajepont Technology	Battery Reconditioner	Very good! Very prompt! Logistics and merchants very fast! Very satisfied! Thank you very much!	JD.com
Cajepont Technology	Battery Reconditioner	Baby received and loved it, will follow up after I use it next time I will come back.	JD.com
Cajepont Technology	Battery Reconditioner	The item has been received and I look forward to the results.	JD.com
Cajepont Technology	Battery Reconditioner	Repair period used twice, convenient and practical, can repair for a variety of cell phones, the future no longer need to change the battery often, value for money, like the small partners together with the order order it!	JD.com
Cajepont Technology	Battery Reconditioner	The cell phone repairer is safe and can repair the battery so that the battery can extend its life, it works very well, the	JD.com
Cajepont Technology	Battery Reconditioner	workmanship, good quality, body shape trend cool technology creating art, easy to carry outside, efficient heat dissipation, safety and security, try to charge a few times, looking forward to the repair effect, such as the effect is significant, then, will continue to buy, to the full five stars, thank you business!	JD.com
Cajepont Technology	Battery Reconditioner	Beautiful, like a high-tech product[love it]	JD.com
Cajepont Technology	Battery Reconditioner	Received it early, used it a couple times and it's ok	JD.com

Cajepont Technology	Battery Reconditioner	Cell phone battery repairer received, according to customer service to give the use of methods and instructions, used a few times, the effect is very good, continue to use!	JD.com
Cajepont Technology	Battery Reconditioner	Easy to carry beautiful and practical.	JD.com
Cajepont Technology	Battery Reconditioner	Logistics is very fast, the description is the same as the real thing, use to change the life of the battery, the	JD.com
Cajepont Technology	Battery Reconditioner	Repair battery device received, well-packaged, fast logistics, especially good customer service, questions and answers, explaining the description of the special clear. Expect to work as well as the live introduction.	JD.com
Cajepont Technology	Battery Reconditioner	Battery Reconditioner received, opened it pretty good	JD.com
Cajepont Technology	Battery Reconditioner	Just received the goods, this product I am using, the effect is very good!	JD.com
Cajepont Technology	Battery Reconditioner	The quality is great and after charging the phone twice, it lasted longer than it did.	JD.com
Cajepont Technology	Battery Reconditioner	After using it for a while, it does work. Just writing a review today. Recommended to use	JD.com
Cajepont Technology	Battery Reconditioner	I don't know if it's fixable yet, I hope I'm not paying an IQ tax to try it out first	JD.com
Cajepont Technology	Battery Reconditioner	Cell phone battery repairer has been received. Trial effect is good, the display is clear, charging is very stable, the style is also good, it is worth recommending!	JD.com
Cajepont Technology	Battery Reconditioner	Rechargeable Battery:[Smile]The goods have been received safely[Smile][Smile] [Smile]Thank you[Smile]Try it out[Smile][Smile's good before you buy it.[smilesmile][smile][smile]	JD.com
Cajepont Technology	Battery Reconditioner	Baby received. Packaging is intact Baby is small and in good shape, trying it out.	JD.com
Cajepont Technology	Battery Reconditioner	Baby received very satisfied thank you boss	JD.com
Cajepont Technology	Battery Reconditioner	This user feels the product is better	JD.com
Cajepont Technology	Battery Reconditioner	In use.	JD.com
Cajepont Technology	Battery Reconditioner	Very good, the repairer received, small and exquisite, easy to carry, very convenient to use, according to the instructions, the effect is good.	JD.com
Cajepont Technology	Battery Reconditioner	Quality assurance, cheap price, worth buying	JD.com
Cajepont Technology	Battery Reconditioner	I got it.	JD.com
Cajepont Technology	Battery Reconditioner	This user feels the product is better	JD.com
Cajepont Technology	Battery Reconditioner	I received this product in perfect condition, and I believe that the business is really as advertised, so I'd like to give it a five-star review.	JD.com
Cajepont Technology	Battery Reconditioner	Beautiful and generous, clear font, has a certain effect on the repair of cell phone batteries has been used for four days, not bad, looking forward to the repairer durable. If you want to have friends worth getting.	JD.com
Cajepont Technology	Battery Reconditioner	Great quality, 5 stars to the boss	JD.com
Cajepont Technology	Battery Reconditioner	Received, used it today is very good, look at the repair of the battery is good in the follow-up comment!	JD.com
Cajepont Technology	Battery Reconditioner	battery needs to be slowly repaired, there should be no problem, after a period of time with the follow-up speak, praise!	JD.com
Cajepont Technology	Battery Reconditioner	It works very well very well every one of them is practical	JD.com
Cajepont Technology	Battery Reconditioner	One side of the plug is deformed, I don't know if it works.	JD.com
Cajepont Technology	Battery Reconditioner	It's really great, I'm very happy with it, thanks to the merchant	JD.com
Cajepont Technology	Battery Reconditioner	I really like this flusher, the ability to repair the batteries and charging speed is also fast, (I should have come to share this with you earlier, considering that I have to do a few more days of experience before my heart is in the right place) so I'm late to the party. Believe me no problem......!	JD.com
Cajepont Technology	Battery Reconditioner	Baby received, used the effect is good, still quite satisfied[smile]	JD.com
Cajepont Technology	Battery Reconditioner	Charge charge charge, cell phone speed, specific operation has a video, slowly read the instructions, well	JD.com
Cajepont Technology	Battery Reconditioner	It's working.	JD.com
Cajepont Technology	Battery Reconditioner	Cell phone battery repairer, works great very accurate.	JD.com
Cajepont Technology	Battery Reconditioner	The stuff is pretty good, you can get it can get it, it's pretty easy for me to use it	JD.com

Cajepont Technology	Battery Reconditioner	Charging is fine.	JD.com
Cajepont Technology	Battery Reconditioner	through the five-star praise[praise] [praise] [praise] [praise] [praise]	JD.com
Cajepont Technology	Battery Reconditioner	Goods to receive, can, customer service attitude is very good, logistics is very fast!	JD.com
Cajepont Technology	Battery Reconditioner	Goods have been received product quality can be very good	JD.com
Cajepont Technology	Battery Reconditioner	The product has been received and is very good	JD.com
Cajepont Technology	Battery Reconditioner	Received the goods immediately tried to see the number is jumping, should be in the repair it	JD.com
Cajepont Technology	Battery Reconditioner	A good, practical and well-matched product is a good product.	JD.com
Cajepont Technology	Battery Reconditioner	I've received the goods and I'll comment after using it.	JD.com
Cajepont Technology	Battery Reconditioner	Product looks cool and has instructions for use. Have installed and used it according to the instructions.	JD.com
Cajepont Technology	Battery Reconditioner	Received by express mail. Packaging is complete. Unpacked and looked at the bright design. Easy to use, genuine quality!	JD.com
Cajepont Technology	Battery Reconditioner	Baby received, beautiful and practical, with some restorative effects, great!	JD.com
Cajepont Technology	Battery Reconditioner	Just received. Don't know to. Will it fix the cell phone battery. Not evaluating now.	JD.com
Cajepont Technology	Battery Reconditioner	Use it first and see what works.	JD.com
Cajepont Technology	Battery Reconditioner	Good appearance, fast charging, fast logistics, so far normal use.	JD.com
Cajepont Technology	Battery Reconditioner	Cell phone electromagnetic repair instrument received, small and exquisite, standard look practical, logistics is very fast, worth buying.	JD.com
Cajepont Technology	Battery Reconditioner	The item was received and I couldn't wait to use it and it felt fine. It is a good shopping. I will introduce my friends to buy it when I have the chance in the future.	JD.com
Cajepont Technology	Battery Reconditioner	Good The logistics was fast I'm satisfied	JD.com
Cajepont Technology	Battery Reconditioner	The cell phone repairer has been used twice, the cell phone battery life is longer, the quality of the repairer is very good, it is a very good product. Give👍	JD.com
Cajepont Technology	Battery Reconditioner	Water machine battery repairer, there is an effect, the speed has improved, logistics fast business service attitude!	JD.com
Cajepont Technology	Battery Reconditioner	Merchant customer service attitude is very good The logistics speed is also very fast Very satisfied with a shopping Good Value for Good things are worth promoting Kudos to the merchant I hope that everyone will come to see the	JD.com
Cajepont Technology	Battery Reconditioner	charging the phone is full, than before with the time is more___some, is straight to buy, thanks to the seller, someone needs to buy back.	JD.com
Cajepont Technology	Battery Reconditioner	Goods received. Logistics is very fast, the quality is very good. Worth embracing this has a good product. Will recommend this product to friends.	JD.com
Cajepont Technology	Battery Reconditioner	Just received , not yet used, the boss service is very patient and timely, there are questions and answers Use simple, fast logistics, intact	JD.com
Cajepont Technology	Battery Reconditioner	Great quality, easy to use and I'm very happy with it!	JD.com
Cajepont Technology	Battery Reconditioner	Good appearance, good use, charging quite fast, logistics is very fast, the quality is very good,, the current use of normal, the service is quite good, good value for money, packaging is very good, cost-effective, feel very good	JD.com
Cajepont Technology	Battery Reconditioner	It's nice looking[big love][big love]love[bigbigbig[love[big[big[big[big[big[big[][biglove,,good	JD.com
Cajepont Technology	Battery Reconditioner	Baby received, and merchants described the same, trustworthy!	JD.com
Cajepont Technology	Battery Reconditioner	Fast shipping is good, I'll follow up after using it	JD.com
Cajepont Technology	Battery Reconditioner	phone these two days without using the use of automatic shutdown.	JD.com
Cajepont Technology	Battery Reconditioner	The goods have been received and customer service described the same charging quite fast need pro worth having!	JD.com
Cajepont Technology	Battery Reconditioner	Baby received Used Very good Will be back for more	JD.com
Cajepont Technology	Battery Reconditioner	I've only used it once and it feels good, I'll comment after a few more uses	JD.com
Cajepont Technology	Battery Reconditioner	It's very good. It's good.	JD.com
Cajepont Technology	Battery Reconditioner	serviceable	JD.com

Cajepont Technology	Battery Reconditioner	The goods have been received, tried for two days, and continue to	JD.com
Cajepont Technology	Battery Reconditioner	The cell phone repairer has been received and is well packaged. Easy to use.	JD.com
Cajepont Technology	Battery Reconditioner	Just used it once don't know how long the fix	JD.com
Cajepont Technology	Battery Reconditioner	Simple operation and good logistics is also very fast	JD.com
Cajepont Technology	Battery Reconditioner	Thought it was pretty good, just a higher sex price overall very happy with it.	JD.com
Cajepont Technology	Battery Reconditioner	Express delivery was fast, arrived in four or five days for New Year's Eve. Now the battery is dead, fix it quickly. Extend the time. You extend is to do a few hours, the battery is dead slowly recover, can be timed, slowly will be durable, a little longer, I used to buy a new battery there to change, do not change the battery and discount.	JD.com
Cajepont Technology	Battery Reconditioner	effect, look at the instructions of this is what power-saving devices, not what cell phone battery repair device ah, feel a little cheated, hopefully not fooled can be a little bit of effect. First give a four-star, after using additional comments	JD.com
Cajepont Technology	Battery Reconditioner	Nice, pretty fast delivery, charging now, looking forward to good use![Smiles][Smiles][Smiles]	JD.com
Cajepont Technology	Battery Reconditioner	Works well, charges quickly, works fine so far, service is pretty good, the	JD.com
Cajepont Technology	Battery Reconditioner	Cell phone battery repairer received. The logistics is very fast, the appearance is good, the workmanship is fine, and the overall feeling is very good!	JD.com
Cajepont Technology	Battery Reconditioner	The effect is quite good', the light is colorful, praise	JD.com
Cajepont Technology	Battery Reconditioner	Very cool! Love it, tried it out and it works like a charm![Smiles]	JD.com
Cajepont Technology	Battery Reconditioner	Feels good to fix the battery	JD.com
Cajepont Technology	Battery Reconditioner	Cell phone repairer, small and exquisite, good quality, good price, cost-effective, very satisfied. Full praise!	JD.com
Cajepont Technology	Battery Reconditioner	Just as described, very good quality, very well packaged, very fast delivery, so nice[smile]	JD.com
Cajepont Technology	Battery Reconditioner	Value for money worth it, exactly as described in kind, worth buying	JD.com
Cajepont Technology	Battery Reconditioner	Works great Will recommend friends to buy next time	JD.com
Cajepont Technology	Battery Reconditioner	The quality is great, the service is cuddly, and it arrived.	JD.com
Cajepont Technology	Battery Reconditioner	Things have been received, just received can not wait to plug in and use, good use[kudos]. The effect of using it will be reviewed tomorrow. Please look forward to.	JD.com
Cajepont Technology	Battery Reconditioner	The logistics were fast!	JD.com
Cajepont Technology	Battery Reconditioner	This cell phone battery repairer received immediately plugged in to try the effect, the effect is still relatively good, to see if the future can repair the battery, the effect is good to buy a few more to send friends!	JD.com
Cajepont Technology	Battery Reconditioner	(of an unmarried couple) be close	JD.com
Cajepont Technology	Battery Reconditioner	Another IQ tax and not even a manual.	JD.com
Cajepont Technology	Battery Reconditioner	This item is a great, cell phone repairer that protects the battery.	JD.com
Cajepont Technology	Battery Reconditioner	ok ! I'm very happy with it.	JD.com
Cajepont Technology	Battery Reconditioner	The goods have been received, everything is normal, the packaging is very good, the cell phone repairer is good, 挺 good, is genuine, logistics is very fast.	JD.com
Cajepont Technology	Battery Reconditioner	I've received the item, it's very delicate and has a digital display on it.	JD.com
Cajepont Technology	Battery Reconditioner	The cell phone repairer is good. Adopting high efficient heat dissipation material, safety is guaranteed and beautiful shape. Fix the phone lag, optimize the battery step by step and extend the battery life. Five-year warranty period, can be used with confidence.	JD.com
Cajepont Technology	Battery Reconditioner	Battery repair on the phone does not lag, it's smooth, and it takes longer than the original	JD.com
Cajepont Technology	Battery Reconditioner	Fast logistics, great customer service, compact device, consistent with description	JD.com
Cajepont Technology	Battery Reconditioner	Just received the shipment and used it. Do not know how effective. Good will continue to buy.	JD.com

Cajepont Technology	Battery Reconditioner	Received the goods, the packaging is intact, open immediately tried, can be used, very satisfied with the, high-tech[kudos]to five-star praise, has been concerned about the store, recommended circle of friends	JD.com
Cajepont Technology	Battery Reconditioner	Today received the repair smartphone function universal baby, very like, very satisfied with the quality is very good.	JD.com
Cajepont Technology	Battery Reconditioner	Baby is great!Charges fast and as described by the merchant!May my cell phone battery be fixed soon!Thank you boss!	JD.com
Cajepont Technology	Battery Reconditioner	charging quite fast, unique shape, beautiful and practical, can be suitable for various models of cell phone repair charging use, now in use, looking forward to using 5to8weeks after the obvious results.	JD.com
Cajepont Technology	Battery Reconditioner	Repair charger received, logistics is very fast,, the product is very good, the color is consistent with the picture, the price is very affordable, the video image is clear, the need for friends can rest assured that the order!	JD.com
Cajepont Technology	Battery Reconditioner	After using it for a few days, it feels like a cover-up device. Genuinely skeptical of the people who rave about it……	JD.com
Cajepont Technology	Battery Reconditioner	I received it and it feels good, I'll use it in a while and then I'll know	JD.com
Cajepont Technology	Battery Reconditioner	Just received, and the picture is the same, directly plugged in the first use to see the effect.	JD.com
Cajepont Technology	Battery Reconditioner	the same, have not yet used. After the use of the follow-up evaluation	JD.com
Cajepont Technology	Battery Reconditioner	Works great, great price, worth the money.	JD.com
Cajepont Technology	Battery Reconditioner	A very good cell phone battery repairer, worth buying!	JD.com
Cajepont Technology	Battery Reconditioner	Great. Thank you. Happy New Year, everyone.	JD.com
Cajepont Technology	Battery Reconditioner	Good service attitude of merchants, courier brother hard work	JD.com
Cajepont Technology	Battery Reconditioner	Can be good next time to repurchase logistics quickly[smile]	JD.com
Cajepont Technology	Battery Reconditioner	Shipped quickly during the outbreak, goods received	JD.com
Cajepont Technology	Battery Reconditioner	Haven't used it yet don't know how it will turn out	JD.com
Cajepont Technology	Battery Reconditioner	The item is as described by the merchant A great product	JD.com
Cajepont Technology	Battery Reconditioner	Commodities to receive, courier service is very good, not yet used after waiting in the praise!	JD.com
Cajepont Technology	Battery Reconditioner	Now that I've got it, there's no effect of using it now!	JD.com
Cajepont Technology	Battery Reconditioner	This express I received, not bad, logistics is also quite fast, this cell phone repairer, looks very good, quite good	JD.com
Cajepont Technology	Battery Reconditioner	The goods have been received, the courier arrived quickly, praise!	JD.com
Cajepont Technology	Battery Reconditioner	Goods have long been received, after two trials of the phone is now used very well, but also durable, thanks to the patient guidance of customer service, collection in order to introduce to friends to buy!	JD.com
Cajepont Technology	Battery Reconditioner	It's not bad by the looks of it.	JD.com
Cajepont Technology	Battery Reconditioner	Good, cheap and practical	JD.com
Cajepont Technology	Battery Reconditioner	The shipment was received in good condition.	JD.com
Cajepont Technology	Battery Reconditioner	Baby has received, the appearance is very delicate, is charging, try to see how the effect, and then follow up!	JD.com
Cajepont Technology	Battery Reconditioner	Cell phone battery repairer is really good, with it you don't need to change the cell phone battery. Thank you!	JD.com
Cajepont Technology	Battery Reconditioner	works, more than the usual two hours or so, try a week and then follow up!	JD.com
Cajepont Technology	Battery Reconditioner	It's good. I'll buy it again.	JD.com
Cajepont Technology	Battery Reconditioner	It won't work without a cable.	JD.com
Cajepont Technology	Battery Reconditioner	Baby received, feel pretty good. Full-featured instructions are clearly described.	JD.com
Cajepont Technology	Battery Reconditioner	Cell Phone Repairer for Android Apple Tablet without lagging to extend battery life to stabilize the charger current and voltage Received, the logistics is very fast, the price is right, the shape is good, I hope it is effective, follow the instructions in the manual to do it, and insist on using it5to8weeks, and then we will talk about it!	JD.com
Cajepont Technology	Battery Reconditioner	Packaging is intact, has been used, continue to see how the effect? The effect is good and then continue to follow up!	JD.com

Cajepont Technology	Battery Reconditioner	Baby is nice, looks beautiful, love it.	JD.com
Cajepont Technology	Battery Reconditioner	Baby received it👌, really great ah!🌈The quality is really nothing, very satisfied!🆗Happy happy happy😃Logistics and products are good, cost-effective, praise praise praise👏👏👏👏👏👏👏	JD.com
Cajepont Technology	Battery Reconditioner	Merchants with logistics very quickly, the cell phone repair device received, baby small and exquisite, with an old cell phone charging repair, repair device light digital jumps, it seems that there should be a repair battery effect, with a few weeks of battery repair how the most positive feedback.	JD.com
Cajepont Technology	Battery Reconditioner	Just started using it, feels good	JD.com
Cajepont Technology	Battery Reconditioner	I don't know if it works.	JD.com
Cajepont Technology	Battery Reconditioner	The quality is excellent, value for money, and by practical charging does add alkyne.	JD.com
Cajepont Technology	Battery Reconditioner	Fast logistics, good customer service, worth buying!	JD.com
Cajepont Technology	Battery Reconditioner	It works pretty well, fixes the battery pretty well, and it's better to suggest that you turn off your phone when you use it	JD.com
Cajepont Technology	Battery Reconditioner	tightly, high screen resolution, convenient size, convenient size	JD.com
Cajepont Technology	Battery Reconditioner	The quality is very good, especially satisfied with the praise!	JD.com
Cajepont Technology	Battery Reconditioner	满星满星满星满星满星满星，质量不错，希望能达到预期效果，使用一段时间再来真实追评，到时候希望能帮到大家想入手的。	JD.com
Cajepont Technology	Battery Reconditioner	The stuff is great , very techy , haven't used it yet, hope it fixes the battery.	JD.com
Cajepont Technology	Battery Reconditioner	I got it. It feels good. I'm using it.	JD.com
Cajepont Technology	Battery Reconditioner	Charges pretty fast, pretty much as described	JD.com
Cajepont Technology	Battery Reconditioner	The logistics were quick and I look forward to the recovery!	JD.com
Cajepont Technology	Battery Reconditioner	Fast delivery, very affordable and easy to carry around.	JD.com
Cajepont Technology	Battery Reconditioner	Great quality forwarded to friends to buy	JD.com
Cajepont Technology	Battery Reconditioner	The product is good, the battery repair is effective, true technology Blackstar.	JD.com
Cajepont Technology	Battery Reconditioner	The accessories are perfect.	JD.com
Cajepont Technology	Battery Reconditioner	Looks good has the weight [love it]specific performance with feedback after use	JD.com
Cajepont Technology	Battery Reconditioner	Logistics is very fast The package is intact How long does it take to have an effect	JD.com
Cajepont Technology	Battery Reconditioner	Quality is good, very fine workmanship, good expensive, to 2see the effect, first praise, effective and then comment	JD.com
Cajepont Technology	Battery Reconditioner	This user did not fill in a rating	JD.com
Cajepont Technology	Battery Reconditioner	Baby received, fast shipping, perfect packaging, high profile, awesome!	JD.com
Cajepont Technology	Battery Reconditioner	Display has been spaced changes, looking for a barely stored power machine in the test, the effect of the test and then see, the repairer appearance, workmanship is good, the overall feeling is quite good	JD.com

Cajepont Technology	Battery Reconditioner	the smoothness of the phone. After he fixed it, the phone's boot screen was much shorter than it was, and the speed run was noticeable. The speed of running is also obviously improved. As for the repair function of the battery, it has not yet been found, can only wait for a long time to slowly see if there will be a time extension. Changing the cell phone battery is more troublesome a job, and then also on the dead horse, use this to see if it works, after all, more than a year of cell phones, the battery is also sure to have wear and tear. Everyone then change a new battery, its capacity is also the cure is not the root cause, never too durable, and the original battery is to generally speaking, the evaluation is much better. Still decided to use this to try to repair a bit, see if the battery can be a little more durable, and then this thing, it looks like it is through the constant application of current. It looks like it activates the battery by constantly applying different sizes of electric current to form a pulse-like structure. The so-called can optimize the running speed of the phone, may be it has a certain intelligent chip inside, and the phone is connected to an external storage space. It could be that it has a smart chip inside that is connected to the phone's external storage space and carries out certain repairs or implantation of certain programs. Specifically we do not understand, anyway, this thing is to say that if you can get the battery, it is indeed also very worthwhile and recommended to buy, anyway, on the running speed is improved, indeed this feeling. The phone and the new one is almost the	JD.com
Cajepont Technology	Battery Reconditioner	I'm trying it out and it should work well. Recommend this product to friends if it's good!	JD.com
Cajepont Technology	Battery Reconditioner	Easton received the product and it works as advertised.	JD.com
Cajepont Technology	Battery Reconditioner	I'm in the process of organizing, looking forward to the follow-up evaluation, and looking at this number, I feel like it's somewhat useful	JD.com
Cajepont Technology	Battery Reconditioner	After using it, I feel that it has a certain repair effect on cell phone range, in addition to charging voltage, current at a glance, a good cell phone battery maintainer.	JD.com
Cajepont Technology	Battery Reconditioner	The cell phone repairer has been received for a good two days, and I have been trying it out, and it does extend or repair the life of old cell phone batteries when operated according to the instructions.	JD.com
Cajepont Technology	Battery Reconditioner	Repairing the cell phone is more effective. Two years of cell phone power drop, after a few days of repair, recovered a lot. Already recommended friends to buy	JD.com
Cajepont Technology	Battery Reconditioner	Fast logistics, beautiful appearance, true description.	JD.com
Cajepont Technology	Battery Reconditioner	The item was received and the quality is super positive! Very satisfied!	JD.com
Cajepont Technology	Battery Reconditioner	This user did not fill in a rating	JD.com
Cajepont Technology	Battery Reconditioner	and practical, after receiving the goods, I can not wait to load the repairer to start charging repair la! In the power repair, the repairer light digital keep flashing changes, stable and normal work, I thought of our family has two old cell phone battery durability problems, finally saved. When you can also save money for the family, it can be said that the best of both worlds. If you need it, you can rest assured that you can buy it.	JD.com
Cajepont Technology	Battery Reconditioner	Cell phone battery repair charger received, the delivery is very fast, the packaging is very good, intact. Repair charger fine workmanship, small and exquisite, good quality, convenient and practical, worth recommending the purchase.	JD.com
Cajepont Technology	Battery Reconditioner	First time to use, see a lot of positive feedback to try to see, use a good and then come back to the follow-up review	JD.com
Cajepont Technology	Battery Reconditioner	Packed well without damage, small and exquisite easy to carry, fast charging speed	JD.com
Cajepont Technology	Battery Reconditioner	Not bad.	JD.com
Cajepont Technology	Battery Reconditioner	Comfortable in the hand, beautiful, great converter[love it], everything functions properly, looks great	JD.com
Cajepont Technology	Battery Reconditioner	Logistics is very fast, the packaging is very good, and the manufacturer describes the same, very satisfied with a shopping, and will follow up after use.	JD.com
Cajepont Technology	Battery Reconditioner	Received it, hope this works to repair the battery!	JD.com
Cajepont Technology	Battery Reconditioner	Currently using normal, comfortable feel, all the functions are normal, sensitive, good experience, good effect on battery repair, the shape is also more beautiful, charging normal, recommended to buy!	JD.com

Cajepont Technology	Battery Reconditioner	Purchase repair cell phone battery charger received, logistics is very fast, color digital display beautiful and exquisite, repair the battery more durable, thank you business, this product praise praise praise praise praise praise praise	JD.com
Cajepont Technology	Battery Reconditioner	comment, only to try twice before confirming receipt, the first fully charged, the minimum value is 0.04A, the second fully charged when the minimum value is 0.00A, I think this change should be the effect of cell phone battery repair it. I think this change should be the effect of cell phone battery repair.	JD.com
Cajepont Technology	Battery Reconditioner	Very good. Satisfied.	JD.com
Cajepont Technology	Battery Reconditioner	Cell phone battery repair charger received, well packaged without damage, the business logistics delivery is very fast, the courier brother to the door, thank you.	JD.com
Cajepont Technology	Battery Reconditioner	don't last long, plus this does work after charging! Good review!	JD.com
Cajepont Technology	Battery Reconditioner	It's working fine so far, feels comfortable in the hand, and the converter is very nice	JD.com
Cajepont Technology	Battery Reconditioner	This user did not fill in a rating	JD.com
Cajepont Technology	Battery Reconditioner	The item works well, is small and compact, and is practical to promote the purchase.	JD.com
Cajepont Technology	Battery Reconditioner	Received it, great quality, will be back for more next time.	JD.com
Cajepont Technology	Battery Reconditioner	Good packaging, fast logistics, and as described.[Smile]	JD.com
Cajepont Technology	Battery Reconditioner	Trying it out right now! Don't know if it works yet!	JD.com
Cajepont Technology	Battery Reconditioner	Received and will review after use. Five stars first.	JD.com
Cajepont Technology	Battery Reconditioner	not heat up👍👍👍👍👍My cell phone Huawei P40Charging with the original charger from20%-80%every time you charge the phone is hot with this does not heat at all👍high-tech products who does not know, slowly repair to see the effect of it……merchant service is thoughtful, logistics is particularly fast worth buying👍……	JD.com
Cajepont Technology	Battery Reconditioner	[Big Love]The quality of the item is excellent. Fine workmanship. Appearance is superb. The restoration is excellent. Great quality as well.	JD.com
Cajepont Technology	Battery Reconditioner	This fixer is very good, cell phone repair, everyone go buy this fixer, this fixer is very good, cell phone repair is very good, the effect is very good, and cheap and affordable, really good.[Smile][Smile][Smile][Smile][Smile].	JD.com
Cajepont Technology	Battery Reconditioner	Plugs are tight, still testing them, first a positive review	JD.com
Cajepont Technology	Battery Reconditioner	Goods have been received, has been used, do not know the effect, the effect is good then will add evaluation!	JD.com
Cajepont Technology	Battery Reconditioner	The baby received thanks to the logistics is also very	JD.com
Cajepont Technology	Battery Reconditioner	house are old, battery life is lacking, looking forward to fixing it.	JD.com
Cajepont Technology	Battery Reconditioner	I hope it really has a battery repair function, I'll comment after trying it out.	JD.com
Cajepont Technology	Battery Reconditioner	Baby received yesterday, the seller's service is enthusiastic, delivery is fast. Yesterday tried, feel quite good, is genuine is a good seller!	JD.com
Cajepont Technology	Battery Reconditioner	Roger that. Techy.	JD.com
Cajepont Technology	Battery Reconditioner	NB	JD.com
Cajepont Technology	Battery Reconditioner	It feels good.It's the right product.good.	JD.com
Cajepont Technology	Battery Reconditioner	It's nice to look at, charging is not yet seen to be useful in restoring the battery, but it's good for charging the phone	JD.com
Cajepont Technology	Battery Reconditioner	I bought it and used it right away it works great give it 5 stars!	JD.com
Cajepont Technology	Battery Reconditioner	After using it, the cell phone battery drains faster, and it doesn't feel like it's doing much good	JD.com
Cajepont Technology	Battery Reconditioner	Great charger, not sure if it really fixes batteries, will comment after use!	JD.com
Cajepont Technology	Battery Reconditioner	Fast shipping Exquisite workmanship Renewing the life of a cell phone Haha	JD.com
Cajepont Technology	Battery Reconditioner	The charger is ticket bright and works well, will buy again when needed.	JD.com

Cajepont Technology	Battery Reconditioner	treasure because it can not store electricity, with this repair and can be used normally, cell phone battery repair to be used for a period of time and then evaluate, first give five-star praise.	JD.com
Cajepont Technology	Battery Reconditioner	Just received the package looks good, has not been used, after use to give follow-up comments.	JD.com
Cajepont Technology	Battery Reconditioner	and the battery feels like a charge is a little longer than before use.	JD.com
Cajepont Technology	Battery Reconditioner	bought it to try. Hopefully the battery will be restored as new!	JD.com
Cajepont Technology	Battery Reconditioner	I just started using it today, and I see the numbers keep changing, so I think it's working, and I'll follow up after I use it for half a month to see if there's any effect!	JD.com
Cajepont Technology	Battery Reconditioner	The service is quite good,, the logistics is good,, the appearance is exquisite, the packaging is intact. Waiting for the trial and then follow up.	JD.com
Cajepont Technology	Battery Reconditioner	Well-packed, fast logistics. Take out the multifunctional cell phone battery repairer, the shape of the clever design, read the instructions, then plugged in to the cell phone battery charging repair, such as charging the battery to use after the effect of post-evaluation.	JD.com
Cajepont Technology	Battery Reconditioner	Received the product after using the feeling of battery durability a lot, kudos	JD.com
Cajepont Technology	Battery Reconditioner	inexpensive, thanks to the shopkeeper! Honestly, this is my Taobao shopping to let me very satisfied with a shopping whether the boss's attitude or the items, I am very satisfied. Cabinet attitude is very professional and enthusiastic, ask questions, reply quickly, I asked a lot of questions, he does not feel annoyed, will seriously answer me, this point I express my heartfelt respect to the cabinet, such a good cabinet can not be more. Then say baby, is what I need, received when the packaging is complete, after opening my surprise, baby than I thought it was even better! Have to have to give a thumbs up. The next time I need to come back, when the trouble boss to give a discount Oh!	JD.com
Cajepont Technology	Battery Reconditioner	Exquisite appearance, clever design, workmanship is particularly good, good logistics, first praise	JD.com
Cajepont Technology	Battery Reconditioner	Looks good, first time using it, ready to shut down and charge to fix. Come back later to comment	JD.com
Cajepont Technology	Battery Reconditioner	The packaging is great and the car model hides the connectors.	JD.com
Cajepont Technology	Battery Reconditioner	Functional and practical, easy to install, easy to use. The price is right, worth having. Fast delivery is strong, praise all five stars.	JD.com
Cajepont Technology	Battery Reconditioner	few years, the battery degradation sharp, I used the repairer test charging once, feel a little effect, read the instructions need to be charged seven, eight times, I hope to have satisfactory results.	JD.com
Cajepont Technology	Battery Reconditioner	The logistics is very fast, the packaging is intact, the cell phone battery repairer received, has been used, first praise!	JD.com
Cajepont Technology	Battery Reconditioner	Received the product first positive review!	JD.com
Cajepont Technology	Battery Reconditioner	The material is quite good, the logistics is good, the appearance is exquisite, and the effect is clear.	JD.com
Cajepont Technology	Battery Reconditioner	This thing is kinda useful, have a redmi note 9pro. I don't know why it won't turn on, but after charging it for 6 or 7 hours with another charger, it still won't turn on. no choice but to buy this charger to try, unexpectedly with this charger4hours, actually can turn on the power, the power is89%, and then charged for2hours,100%. It is said that the Redmi cell phone this can not turn on more problems	JD.com
Cajepont Technology	Battery Reconditioner	Good product, maintains cell phone battery. Worth recommending to buy	JD.com
Cajepont Technology	Battery Reconditioner	A high tech product, hopefully it will improve the battery life and the phone won't lag.	JD.com
Cajepont Technology	Battery Reconditioner	Nice stuff[Smiles][Smiles][Smiles]	JD.com
Cajepont Technology	Battery Reconditioner	Plugged in to charge my cell phone and it felt great, the numbers jumped in to fix the charging cell phone battery!	JD.com
Cajepont Technology	Battery Reconditioner	of the battery, after using the battery than before, much more durable, very good to recommend to everyone, very satisfied with a shopping.	JD.com
Cajepont Technology	Battery Reconditioner	I purposely took a couple of old cell phones to try it out and it still does work. It's great.	JD.com
Cajepont Technology	Battery Reconditioner	Received the item and am in the process of test charging it, but hopefully it will be restorative.	JD.com
Cajepont Technology	Battery Reconditioner	The quality of the customer service is very good next time to buy back!	JD.com

Cajepont Technology	Battery Reconditioner	It's pretty good, the phone charges noticeably faster and performance is improved	JD.com
Cajepont Technology	Battery Reconditioner	The quality of the goods is very good, the customer service is warm and attentive, and the logistics is also very powerful. Favorable comments, praise👍👍👍👍	JD.com
Cajepont Technology	Battery Reconditioner	value for money. Small and exquisite, easy to carry. Visual appearance and quality are very good, because I just received the goods have not been used, after use and then the effect of follow-up comments.	JD.com
Cajepont Technology	Battery Reconditioner	Product received, very satisfied, thanks[hee][hee][hee hee][hee	JD.com
Cajepont Technology	Battery Reconditioner	Goods are good, logistics is fast	JD.com
Cajepont Technology	Battery Reconditioner	I received it yesterday, charged my cell phone at night, and used it today, and it really didn't drain as much, it worked'	JD.com
Cajepont Technology	Battery Reconditioner	I tried it once and it did extend by 10%, let's see the result after half a month.	JD.com
Cajepont Technology	Battery Reconditioner	Received it very well, 5 stars.	JD.com
Cajepont Technology	Battery Reconditioner	Very happy with it,[cool][cool][cool]It feels pretty sci-fi[cool]	JD.com
Cajepont Technology	Battery Reconditioner	that charge for two or three minutes and fill up, and use the phone for an hour and then die. I hope it can be repaired with this. First use a period of time. Look at the effect. If there is an effect then come back to the review, no effect then directly return.	JD.com
Cajepont Technology	Battery Reconditioner	Good thing to support the repair after the later good or not next time continue to come back to buy[big love][big love][love[biglove] [big][big	JD.com
Cajepont Technology	Battery Reconditioner	quality is really good very good to use convenient is also really work!	JD.com
Cajepont Technology	Battery Reconditioner	The case is good, not expensive, so far it is working fine, but hopefully it will be effective in repairing the battery life of the phone.	JD.com
Cajepont Technology	Battery Reconditioner	Fast results, inexpensive, normal use so far, great range, normal use so far	JD.com
Cajepont Technology	Battery Reconditioner	Bought it for my parents, reply charging works great!	JD.com
Cajepont Technology	Battery Reconditioner	Works well, easy to use, and the batteries charge up with noticeable results and durability!	JD.com
Cajepont Technology	Battery Reconditioner	I've received the goods, looks cool, try the effect again!	JD.com
Cajepont Technology	Battery Reconditioner	Nice product, works very well	JD.com
Cajepont Technology	Battery Reconditioner	very happy	JD.com
Cajepont Technology	Battery Reconditioner	Cell phone battery repairer has been received, well-packed, fast logistics, satisfied!	JD.com
Cajepont Technology	Battery Reconditioner	Baby received, logistics is very fast, the degree of technology is high, try it out, very feel, the effect should be a period of time to come back to the follow-up Oh!	JD.com
Cajepont Technology	Battery Reconditioner	Logistics is very fast, customer service reply promptly told the repair method, with a period of time should be able to see the repair effect.	JD.com
Cajepont Technology	Battery Reconditioner	Repair function to be tested, charging is still normal, beautiful appearance, safe and reliable to use!	JD.com
Cajepont Technology	Battery Reconditioner	Fast charging is fast and looks great	JD.com
Cajepont Technology	Battery Reconditioner	Got it[smile]nice👌fast charging, straight to own👍......	JD.com
Cajepont Technology	Battery Reconditioner	The battery restorer has been received the styling is very technological!	JD.com
Cajepont Technology	Battery Reconditioner	Just started to use, the shape is cool, the effect of how to use according to the instructions to know later, first praise.	JD.com
Cajepont Technology	Battery Reconditioner	Well well well, very good product, very good quality and functional!	JD.com
Cajepont Technology	Battery Reconditioner	Good use really good use oh!	JD.com
Cajepont Technology	Battery Reconditioner	at night when plugged in, the product digital display constantly jumping, the phone will be very stable repair, no other adverse reactions, continue to try to see, later in the follow-up comment!	JD.com
Cajepont Technology	Battery Reconditioner	really works, I thought there was no saving, my OPPO is now barreling along. one back with the intention of trying it out, followed the tutorial and it really worked, the battery lasted twice as long and last 3 weeks, really great deal	JD.com

Cajepont Technology	Battery Reconditioner	Repair is very good, the effect is powerful, the use of the battery is not so fast dead, dozens of dollars can be repaired, more cost-effective than the replacement of the phone, hahaha save a lot of money, is the genuine it!	JD.com
Cajepont Technology	Battery Reconditioner	use of the effect is very obvious, it is indeed a very cost-effective thing to save thousands of dollars to change the phone, the operation is also particularly easy, not for a while on the good recommended!	JD.com
Cajepont Technology	Battery Reconditioner	Sleeping at night plugged in can, after repairing used for a week, can charge and repair, battery life did not fall over, 10 the King of Glory cell phone is dead, currently can be opened to 13, continued to use, look at the effect of maximization can be up to how much!	JD.com
Cajepont Technology	Battery Reconditioner	Very good to use, the old phone Apple 8 with 3 hours of no power, after using this restorer, now almost can watch fast 5 hours it, receive calls 1 day no problem it, this phone is very old, can use so long, really happy. This is a genuine product, I really recommend it!	JD.com
Cajepont Technology	Battery Reconditioner	Apple X battery life before a month to drop 5 percent, with the previous 80 percent, used 4 weeks, is still 80 percent, really awesome, recommend recommended!	JD.com
Cajepont Technology	Battery Reconditioner	Nice product, plugs in and charges quickly, batteries seem to last a bit longer.	JD.com
Cajepont Technology	Battery Reconditioner	Just received it today, haven't used it yet, don't know how it works. After a few days to follow up the evaluation.	JD.com
Cajepont Technology	Battery Reconditioner	Looks great, looking forward to having results	JD.com
Cajepont Technology	Battery Reconditioner	Battery Repairer is as described, well packaged without damage, fast logistics.	JD.com
Cajepont Technology	Battery Reconditioner	I charged it today, and it should help if it charges a little faster.	JD.com
Cajepont Technology	Battery Reconditioner	On Trial.	JD.com
Cajepont Technology	Battery Reconditioner	Cell phone battery repair instrument has been received, tried twice, there are effects of good quality, businessmen shipments in a timely manner express delivery, as a cell phone users are worth having, recommended the use of the purchase, spend a small amount of money to protect big money.	JD.com
Perten Select	Battery Reconditioner	The store's gone.	JD.com
old but beautiful	New Ultraviolet	I'll comment again when I've tried it for a while.	JD.com
old but beautiful	New Ultraviolet	It works.	JD.com
old but beautiful	New Ultraviolet	This user feels the product is better	JD.com
old but beautiful	New Ultraviolet	Used a few times with no results, scammy stuff	JD.com
old but beautiful	New Ultraviolet	The electric car restorer is very good quality and cheap, I will buy it again next time.	JD.com
old but beautiful	New Ultraviolet	Logistics very quickly well-packaged, did not use, used to come back to the review	JD.com
old but beautiful	New Ultraviolet	This user feels the product is better	JD.com
old but beautiful	New Ultraviolet	First use the results in the review first five-star praise!	JD.com
old but beautiful	New Ultraviolet	Logistics is very fast, and see the same belt voice, worth buying, has begun to use the	JD.com
old but beautiful	New Ultraviolet	It's good to revitalize the old eutectic.	JD.com
old but beautiful	New Ultraviolet	Logistics is fast, the goods have been very good is the plug is not the right number	JD.com
old but beautiful	New Ultraviolet	I'll review after I use it.	JD.com
old but beautiful	New Ultraviolet	I haven't used it yet. I'll come back after I use it.	JD.com
old but beautiful	New Ultraviolet	I don't know yet. I'll come back when I've used it.	JD.com
old but beautiful	New Ultraviolet Battery	positive comment after I use it, I'll give a five-star praise first, if it's really good, I'll introduce my friends to buy from your store	JD.com
good friend	Battery Reconditioner	Very good, good service and fast logistics!	JD.com
good friend	Battery Reconditioner	This user feels the product is better	JD.com
good friend	Battery Reconditioner	Runs fine, fine workmanship, nice appearance	JD.com
good friend	Battery Reconditioner	Runs fine Goods received, not bad, can be charged! Really good!	JD.com

good friend	Battery Reconditioner	pretty good	JD.com
good friend	Battery Reconditioner	This user feels the product is better	JD.com
good friend	Battery Reconditioner	It could be good.	JD.com
good friend	Battery Reconditioner	This user feels the product is better	JD.com
good friend	Battery Reconditioner	I had a juicer that wouldn't charge and I used it to charge it and this fixer really worked.	JD.com
good friend	Battery Reconditioner	It feels as if it's going to charge a little faster than before, and it's also slightly more durable, but the store said that it should be charged every three days, and there will be a more noticeable effect 5~8 weeksin a row, so I initially feel that it's OK, and I'll follow up with a review later on.	JD.com
good friend	Battery Reconditioner	Just received the goods, wait for a few days to use before evaluating! Logistics is fast!	JD.com
good friend	Battery Reconditioner	Has been charged for three days feel much better want to buy the relatives can rest assured to buy it wish the boss business prosperity five-star praise!	JD.com
good friend	Battery Reconditioner	Gahhhh good.	JD.com
good friend	Battery Reconditioner	Good review. It works.	JD.com
good friend	Battery Reconditioner	Works great so happy with it.	JD.com
good friend	Battery Reconditioner	ve been using it for a while and it feels good I just can't read it myself	JD.com
good friend	Battery Reconditioner	It's okay.	JD.com
good friend	Battery Reconditioner	not bad	JD.com
good friend	Battery Reconditioner	This user feels the product is better	JD.com
good friend	Battery Reconditioner	Why is it nothing like the one pictured?	JD.com
good friend	Battery Reconditioner	Works great and worth having	JD.com
good friend	Battery Reconditioner	The stuff is genuinely good and recommendable	JD.com
good friend	Battery Reconditioner	Fast mail and very good quality. Highly recommended! [Cool]	JD.com
good friend	Battery Reconditioner	Fix it and see, looking forward to the results!	JD.com
good friend	Battery Reconditioner	Nice stuff very nice ready to make another order	JD.com
good friend	Battery Reconditioner	I bought it, turned it off and tried it, and it was average.	JD.com
good friend	Battery Reconditioner	I'll buy it when it works.	JD.com
good friend	Battery Reconditioner	a bargain	JD.com
good friend	Battery Reconditioner	Convenience: well well well well well	JD.com
good friend	Battery Reconditioner	goods to open the first time to see, it is very good, very satisfied, customer service also have a question and answer, thank you	JD.com
good friend	Battery Reconditioner	Very good, I like, good make is really genuine, have the opportunity to buy again!	JD.com
good friend	Battery Reconditioner	Good! Very good! Good quality, the same as the physical store, the color is correct, I like it!	JD.com
good friend	Battery Reconditioner	I just received it. I don't know if it's any good.	JD.com
good friend	Battery Reconditioner	Beautiful appearance, fine workmanship, running normally	JD.com
good friend	Battery Reconditioner	Very satisfied, five stars will introduce to friends (.˘ε˘.)	JD.com
good friend	Battery Reconditioner	Poor Poor Average Good Very Good Satisfactory	JD.com
good friend	Battery Reconditioner	This user feels the product is better	JD.com
good friend	Battery Reconditioner	serviceable	JD.com
good friend	Battery Reconditioner	I'm satisfied every time, and I'm even more satisfied with the baby I chose this time, and I'll buy again as always!	JD.com

good friend	Battery Reconditioner	Quite good[cool]use to now the phone power consumption has been considered quite slow[hee hee]consumption is not that fast, not to use before that called a speechless[cover face]a not yet to half a day on the power[tears]and not used when the power consumption of30%, you have not yet gone out of the house on the power[two ha] [two ha] [two[ha]two]ha]twotwo[two[] [two ha] [two ha] [two ha] [two ha] [two ha] [two ha] [two ha] [ha]	JD.com
good friend	Battery Reconditioner	Baby received good use next time to buy	JD.com
good friend	Battery Reconditioner	Product Appearance: pretty good looking<br>Battery Charging: very good<br>Carrying Description: very good	JD.com
good friend	Battery Reconditioner	Good quality and easy to carry	JD.com
good friend	Battery Reconditioner	This user did not fill in a rating	JD.com
good friend	Battery Reconditioner	The quality is great.	JD.com
good friend	Battery Reconditioner	Thank you very much. Thanks for your hard work.	JD.com
good friend	Battery Reconditioner	Good quality, simple and convenient, good service attitude, fast logistics.	JD.com
good friend	Battery Reconditioner	This user feels the product is better	JD.com
good friend	Battery Reconditioner	Logistics is very fast quality is very good service attitude is very good	JD.com
good friend	Battery Reconditioner	Logistics is very fast, has been used for a few days, feel the effect is not very big.	JD.com
good friend	Battery Reconditioner	I'm using it, I don't know how it works yet, we'll see. First of all, I'd like to give you a good review.	JD.com
good friend	Battery Reconditioner	Just arrived and couldn't wait to open it, love it!	JD.com
good friend	Battery Reconditioner	‘	JD.com
good friend	Battery Reconditioner	This user feels the product is better	JD.com
good friend	Battery Reconditioner	We'll see if it's any good tomorrow.	JD.com
good friend	Battery Reconditioner	The item was received, shipped quickly, packaged very complete, very satisfied!	JD.com
good friend	Battery Reconditioner	Goods have been received appearance is very good ready to try also will follow up the review[smile] [smile] [smile] [smile][smile]	JD.com
good friend	Battery Reconditioner	Not bad, pretty good, thanks to the merchant	JD.com
good friend	Battery Reconditioner	Arrived quickly, good quality. Thank you boss 👍	JD.com
good friend	Battery Reconditioner	This user feels the product is better	JD.com
good friend	Battery Reconditioner	Feels good, looks full of technology, try it out and then follow up.	JD.com
good friend	Battery Reconditioner	This user feels the product is better	JD.com
good friend	Battery Reconditioner	It's especially good. This stuff's old-fashioned.	JD.com
good friend	Battery Reconditioner	pretty good	JD.com
good friend	Battery Reconditioner	After using it, the phone doesn't get as hot when charging.	JD.com
good friend	Battery Reconditioner	finally arrived. Inside that blazing excitement of the energy surging up to stop can not stop!!!! Think things are good I will give a favorable review of the baby product quality is very good, is genuine. And the page description written basically the same, the seller's customer service attitude from start to finish are very good, express delivery speed super fast!	JD.com
good friend	Battery Reconditioner	Great service, excellent appearance. Restoration evident.	JD.com
good friend	Battery Reconditioner	The package has a surprise[hee]effect,how touse after the complementary comment[cool]	JD.com
good friend	Battery Reconditioner	Goods received the logistics guy is very good, is good or bad try to come to the review	JD.com
good friend	Battery Reconditioner	good. Hope it can bring good results. Give the merchant a good review.	JD.com
good friend	Battery Reconditioner	It's very good. It's very good.	JD.com
good friend	Battery Reconditioner	This user did not fill in a rating	JD.com

good friend	Battery Reconditioner	This user feels the product is better	JD.com
good friend	Battery Reconditioner	Just received it, tried it out and it looks good.	JD.com
good friend	Battery Reconditioner	This user feels the product is better	JD.com
good friend	Battery Reconditioner	I have to praise, the quality is really good, and the packaging logistics are also good. Really like it, won't make you regret after getting it!	JD.com
good friend	Battery Reconditioner	description is almost the same, the quality is no problem, the key price is also appropriate, buy here or very assured, praise!	JD.com
good friend	Battery Reconditioner	It's good. It's the same thing as in Jitterbug.	JD.com
good friend	Battery Reconditioner	The item was received, thanks to the courier guy!	JD.com
good friend	Battery Reconditioner	The baby has received have not yet used first give a favorable comment	JD.com
good friend	Battery Reconditioner	Just received.	JD.com
good friend	Battery Reconditioner	Cell phone battery repair device received, modeling like a small toy of gold steel, not yet used to know how to use the effect? Waiting for the use of additional comments, thank you boss round-trip courier hard!	JD.com
good friend	Battery Reconditioner	The logistics is fast and the quality looks good	JD.com
good friend	Battery Reconditioner	Satisfied Wish the merchant a prosperous business!	JD.com
good friend	Battery Reconditioner	I've got the goods.	JD.com
good friend	Battery Reconditioner	Battery Repairer received, ready to start to study the usage in a while, hope to have a repair effect on my cell phone battery, will follow up!	JD.com
good friend	Battery Reconditioner	[Smiles] [Skimming] [Big Love] [Nerdish] [Cool]	JD.com
good friend	Battery Reconditioner	The function is very good, fast charging. There is a repair function, the business owner a courier hard work! [rose] [rose] [rose] [rose]	JD.com
good friend	Battery Reconditioner	Works great, can be purchased consistently and fixes quickly.	JD.com
good friend	Battery Reconditioner	This thing is quite useful, so you don't have to worry about falling asleep while charging your cell phone anymore.	JD.com
good friend	Battery Reconditioner	Finally received the baby I need, the stuff is very good, the price is good and cheap, thanks to!The owner's attitude is very professional and enthusiastic, ask questions and reply quickly, I asked a lot of questions, he does not feel annoyed, they will seriously answer me. And then say baby, just what I need, when I received the packaging is complete, after opening the surprise, baby than I imagined even better!Had to have to give a thumbs up. I will come back next time I need it! [smile] [smile[smile]smilesmile[smile] [smile] [smile] [smile] [smile] [smile] [smile] [smile] [smile] [smile] [smile][smile	JD.com
good friend	Battery Reconditioner	Very good, after charging once, the phone battery is obviously much more durable than the original	JD.com
good friend	Battery Reconditioner	other similar products, of course the price is also more expensive, if it really can repair the battery, I will repurchase. Thank you🙏	JD.com
good friend	Battery Reconditioner	The goods have been received do not know if it is as you say can repair cell phone batteries	JD.com
good friend	Battery Reconditioner	The goods have been received, the logistics is also very fast, it's okay	JD.com
good friend	Battery Reconditioner	[Lots of love] [Lots of love]	JD.com
good friend	Battery Reconditioner	[Big Love]Logistics is fast	JD.com
good friend	Battery Reconditioner	This user feels the product is better	JD.com
good friend	Battery Reconditioner	This user feels the product is better	JD.com
good friend	Battery Reconditioner	Smartphone cell phone fixer has been received, works well, affordable price? It's a smartphone fixer good helper.	JD.com
good friend	Battery Reconditioner	Very good, the baby is very high, the quality is very good, it looks like it gives a sense of high class, the price is also suitable, this online shopping is very pleasant, big praise👍	JD.com
good friend	Battery Reconditioner	service how to use it? Customer service was very patient for me. But each fix takes two or three hours, so. The effect is looking forward to.	JD.com

good friend	Battery Reconditioner	I received the product, I don't know if it's good or not, but I'll know if I've had it.	JD.com
good friend	Battery Reconditioner	Really much good use.	JD.com
good friend	Battery Reconditioner	Goods have been received, the outer packaging is intact, the package is complete.	JD.com
good friend	Battery Reconditioner	[Smiles]	JD.com
good friend	Battery Reconditioner	Baby received, good service, fast logistics. Feels good, use a few times to follow up.	JD.com
good friend	Battery Reconditioner	Products you can trust[smile]	JD.com
good friend	Battery Reconditioner	Appearance Description:[Big Love]Beautiful Role Experience: good charging	JD.com
good friend	Battery Reconditioner	good, green light is very dazzling. Connected computer direct charging, the speed is much faster than expected, and wired charging is almost the same. Cost-effective is quite high, recommended to buy	JD.com
good friend	Battery Reconditioner	Finish the battery quite quickly, only five weeks time cell phone battery life increased to10%now has been as high as90%of the quite good, the positive sun is quite good, I am particularly satisfied with	JD.com
good friend	Battery Reconditioner	Pro restorers are really good and powerful, good reviews are a must!	JD.com
good friend	Battery Reconditioner	This user feels the product is better	JD.com
good friend	Battery Reconditioner	Watching a few screens of numbers running, does not affect the fast charging at all, the specific effect to be used after a complementary one	JD.com
good friend	Battery Reconditioner	This user feels the product is better	JD.com
good friend	Battery Reconditioner	It is as described, genuine and works well.	JD.com
good friend	Battery Reconditioner	Baby received, logistics is very fast , the quality is very good, very satisfied	JD.com
good friend	Battery Reconditioner	serviceable	JD.com
good friend	Battery Reconditioner	Goods have been received, the quality is very good, using the effect is quite good	JD.com
good friend	Battery Reconditioner	Don't know if it works. Try it first.	JD.com
good friend	Battery Reconditioner	Just used it, whether there is any effect use a few days to say.	JD.com
good friend	Battery Reconditioner	It works.	JD.com
good friend	Battery Reconditioner	ineffective	JD.com
good friend	Battery Reconditioner	It's the ideal product💝, good quality and fast logistics, worth the kudos ‼ the effect is not yet known[rose]but it feels better than the average da[love it] [rose] [rose] [rose]	JD.com
good friend	Battery Reconditioner	I used it for two days after receiving it before taking delivery, and it helped with the battery recovery restoration.	JD.com
good friend	Battery Reconditioner	serviceable	JD.com
good friend	Battery Reconditioner	This baby works really well, unfortunately the phone must be restored to factory settings for it to work.	JD.com
good friend	Battery Reconditioner	Not bad Not bad It looks awesome, we'll see when we use it.	JD.com
good friend	Battery Reconditioner	try to see the attitude to buy this baby, and then received the instructions to operate, the phone standby time is much longer, very good! I want to continue to introduce friends to buy and use.	JD.com
good friend	Battery Reconditioner	Rechargeable Battery: the goods received, feel good, hope the phone battery can reply[smile]	JD.com
good friend	Battery Reconditioner	Very good. Great. Good. Trustworthy.	JD.com
good friend	Battery Reconditioner	I don't know if it's effective, use it for a while and see	JD.com
good friend	Battery Reconditioner	[cool]cool look, the design of the sports car, very comfortable, began to use the expected results yo[cool]	JD.com
good friend	Battery Reconditioner	It's here. We'll just have to see if it works.	JD.com
good friend	Battery Reconditioner	Satisfied with the merchandise, great service, and very smooth logistics.	JD.com
good friend	Battery Reconditioner	Goods received the quality is very good logistics service is also very good	JD.com
good friend	Battery Reconditioner	I just bought it, and I'm looking forward to evaluating the results after a while!	JD.com

good friend	Battery Reconditioner	I almost didn't receive the goods, and it took two days to find it. The casing was also dismantled and the inside is the same as the picture. Boring.	JD.com
good friend	Battery Reconditioner	It looks good but I don't know how it works after use.	JD.com
good friend	Battery Reconditioner	There is a feeling effect. A few more fixes work better. Will recommend friends to buy.	JD.com
good friend	Battery Reconditioner	Looks quite high-tech, just received the goods to use it first, I hope there are results!	JD.com
good friend	Battery Reconditioner	battery repair situation and then follow up, so far it looks pretty good	JD.com
good friend	Battery Reconditioner	Works great, works great, excellent customer service, prompt answers, good reviews, would recommend to friends!	JD.com
good friend	Battery Reconditioner	receipt of the very like, the seller's service attitude is also very good.	JD.com
good friend	Battery Reconditioner	serviceable	JD.com
good friend	Battery Reconditioner	it, the result is good, I continue to use it again to watch the effect!	JD.com
good friend	Battery Reconditioner	Good quality, genuine, repair cell phone batteries have effect	JD.com
good friend	Battery Reconditioner	Fine workmanship, high-end, atmospheric, upscale, I hope it works!	JD.com
good friend	Battery Reconditioner	Charging speed is ok, first time, a few more times to see how it works!	JD.com
good friend	Battery Reconditioner	The goods have been received, thank you.	JD.com
good friend	Battery Reconditioner	atmospheric class zanzanzanzanzanzanzanzanzanzanzanzanzanzanzanzanzanz	JD.com
good friend	Battery Reconditioner	I just received it. I'll see what I can do with it.	JD.com
good friend	Battery Reconditioner	Goods received. Fast logistics, exactly as described in the picture. Quite good.	JD.com
good friend	Battery Reconditioner	This user did not fill in a rating	JD.com
good friend	Battery Reconditioner	Nice and very satisfied with the seller's service attitude.	JD.com
good friend	Battery Reconditioner	Cell phone battery charging repairer received, exquisite and beautiful, logistics is very fast.	JD.com
good friend	Battery Reconditioner	Logistics is very fast, beautiful appearance, easy to operate, easy to use, good repair effect, good business service.	JD.com
good friend	Battery Reconditioner	The item was received very quickly, 5 stars to the merchant!	JD.com
good friend	Battery Reconditioner	Very satisfied with a shopping, recommend everyone to buy!	JD.com
good friend	Battery Reconditioner	The Cell Phone Battery Repairer is so good! The quality is very good! Repair the battery very quickly. The appearance is atmospheric and beautiful, and the workmanship is perfect! Running for three hours, the data rose very quickly!	JD.com
good friend	Battery Reconditioner	Things received, looks good quality Express is also very powerful, received is being used in the subsequent use of the situation to assess the follow-up	JD.com
good friend	Battery Reconditioner	The stuff was received and the logistics was pretty fast.	JD.com
good friend	Battery Reconditioner	Item has been received in perfect condition. See how the restoration goes before giving a pre-evaluation.	JD.com
good friend	Battery Reconditioner	Received the goods on the repair of cell phone batteries, there is a digital display for easy observation, easy to carry, beautiful design, logistics is very fast!	JD.com
good friend	Battery Reconditioner	Fast shipping Quality available	JD.com
good friend	Battery Reconditioner	Fast logistics, reliable quality, recommended to buy	JD.com
good friend	Battery Reconditioner	Charges quickly and works well	JD.com
good friend	Battery Reconditioner	Good service attitude, good product quality and fast logistics. This time is a repurchase.	JD.com
good friend	Battery Reconditioner	Good quality, fast logistics, enthusiastic service	JD.com
good friend	Battery Reconditioner	It works great.	JD.com
good friend	Battery Reconditioner	Nice. Nice looking.	JD.com
good friend	Battery Reconditioner	The product is very beautiful and inexpensive, the logistics is very fast this is very good to use worth buying	JD.com
good friend	Battery Reconditioner	Works great, myIphoneand Huaweiof many yearshave improved time	JD.com
good friend	Battery Reconditioner	I'm sorry to use it, it took a few days to evaluate it works very well good	JD.com

good friend	Battery Reconditioner	It works really well and fixes well, and the battery now lasts longer than before.	JD.com
good friend	Battery Reconditioner	Logistics is very fast, beautiful, good quality, I wish the boss a prosperous business prosperity.	JD.com
good friend	Battery Reconditioner	This user feels the product is better	JD.com
good friend	Battery Reconditioner	good-looking design, and it feels good to use it for a few days, which is consistent with the description. There will be better results in the future, and then evaluate. Logistics is also very fast.	JD.com
good friend	Battery Reconditioner	Received, the logistics is very fast thanks, this charge is very good	JD.com
good friend	Battery Reconditioner	Fast logistics, good service, beautiful appearance, fine workmanship	JD.com
good friend	Battery Reconditioner	This is good stuff, much better than it used to be. Shoot if you need to.	JD.com
good friend	Battery Reconditioner	It's good. It's just starting to work. We'll talk about it in a few days.	JD.com
good friend	Battery Reconditioner	Good service from the merchant! Shipping was fast! It really works so well!	JD.com
good friend	Battery Reconditioner	Very powerful. The logistics were fast too.	JD.com
good friend	Battery Reconditioner	Baby received, small and exquisite, quite good, worth buying	JD.com
good friend	Battery Reconditioner	Not as ideal as advertised Only20points.	JD.com
good friend	Battery Reconditioner	doesn't feel like it's doing anything after using it.	JD.com
good friend	Battery Reconditioner	The stuff is great. The courier's nice.	JD.com
good friend	Battery Reconditioner	The charger has been received, I do not know how well it works first a good comment	JD.com
good friend	Battery Reconditioner	I just received it. I haven't used it yet.	JD.com
good friend	Battery Reconditioner	Great service attitude, fast logistics, good value for money, recommendable.	JD.com
good friend	Battery Reconditioner	Being used, to see if there is no effect, have the effect there and then follow up!	JD.com
good friend	Battery Reconditioner	The goods have been received, thanks to the business service product quality is very good thank you!	JD.com
good friend	Battery Reconditioner	Received the goods, used it once, felt good	JD.com
good friend	Battery Reconditioner	Things received or very satisfied with the pending Sega effect	JD.com
good friend	Battery Reconditioner	Baby received and matches the description.	JD.com
good friend	Battery Reconditioner	Paid the IQ tax and it didn't work!	JD.com
good friend	Battery Reconditioner	Good quality, genuine, repair cell phone battery has effect![Kudos][Kudos][KudosGood packaging, intact, good service attitude, fast shipping, good value, worth getting[Kudos]	JD.com
good friend	Battery Reconditioner	This is good, use to come quite good, will come back. Buy it, you won't regret it! All five points.	JD.com
good friend	Battery Reconditioner	Role experience: things work well, fast charging, merchants are also very good	JD.com
good friend	Battery Reconditioner	The logistics is fast and the package is intact	JD.com
good friend	Battery Reconditioner	Repairer received the goods, easy to use, effective,, electricity disappeared quickly, using the repairer punch fast, the socket does not heat up, renewable power security	JD.com
good friend	Battery Reconditioner	That's good.	JD.com
good friend	Battery Reconditioner	The cell phone battery repairer has been received, looks great, five stars, thanks to the business.	JD.com
good friend	Battery Reconditioner	I received the item, but I won't know how it works until I use it.	JD.com
good friend	Battery Reconditioner	Neighbor collects for you. Come back if there's a problem.	JD.com
good friend	Battery Reconditioner	buddy and I will be putting together an order for the next time.	JD.com
good friend	Battery Reconditioner	This is good stuff, don't know how well it works yet.	JD.com
good friend	Battery Reconditioner	Things are good very good have not yet to charge the phone should be useful it first give a positive feedback all five points after a period of time in the follow-up evaluation	JD.com
good friend	Battery Reconditioner	The item matches the picture, the logistics is fast and the customer service is excellent.	JD.com

good friend	Battery Reconditioner	I got it. I didn't try it.	JD.com
good friend	Battery Reconditioner	This user feels the product is better	JD.com
good friend	Battery Reconditioner	Received, very good quality, began to repair, the phone is fully charged, battery recovery is very good	JD.com
good friend	Battery Reconditioner	Multifunctional cell phone battery repairer is of good quality and fine workmanship! The appearance is beautiful and upscale.	JD.com
good friend	Battery Reconditioner	Haven't used it yet! I don't know how effective it is	JD.com
good friend	Battery Reconditioner	The data plug is bad.	JD.com
good friend	Battery Reconditioner	: Unpacked and intact, item is in great condition straight to the credit,...	JD.com
good friend	Battery Reconditioner	Express delivery was delivered, finished undamaged, give 5 stars!	JD.com
good friend	Battery Reconditioner	Appearance can be whether or not it can really fix the battery to be later Wu tried to comment again	JD.com
good friend	Battery Reconditioner	Visual quite good-looking heavy hope useful[smile],goods found that the data line can not be inserted into the millet phone,the seller free of charge and send a supplement to send me,conscientious seller thank you	JD.com
good friend	Battery Reconditioner	the appearance in the design is kinda sci-fi, the function is the same as described on the web page, there is no difference. My cell phone battery power drop drop very strong, just do not know whether this repairer can really repair my cell phone battery. First five-star praise, to be used for a period of time in the future and then see.	JD.com
good friend	Battery Reconditioner	Received the cell phone dot pool repairer, will follow up after using it!	JD.com
good friend	Battery Reconditioner	2great quality, fast delivery and service.	JD.com
good friend	Battery Reconditioner	I'm trying it out and haven't seen any results yet, so I'll follow up after using it for a while.	JD.com
good friend	Battery Reconditioner	It's good quality.	JD.com
good friend	Battery Reconditioner	It's awesome. It fixes batteries.	JD.com
good friend	Battery Reconditioner	Good use, the shot is Huawei, but I shot the wrong (Android), and customer service contact, immediately shipped over, thank you very much!	JD.com
good friend	Battery Reconditioner	Started fixing it, my phone was fully charged and died in a little over an hour, hopefully the fix will work well, kudos!	JD.com
good friend	Battery Reconditioner	Very happy with it, very easy to use and works well.	JD.com
good friend	Battery Reconditioner	Looks good, give it a try, buy again if it works well	JD.com
good friend	Battery Reconditioner	Baby received, very like, logistics is very fast, good service attitude!	JD.com
good friend	Battery Reconditioner	were a great value. The seller also shipped much earlier than expected.	JD.com
good friend	Battery Reconditioner	The item was received, the description and the object matches the introduction, it looks good and has a modern look.	JD.com
good friend	Battery Reconditioner	Received, well packaged, whether it works or not, use it for a while to see, the	JD.com
good friend	Battery Reconditioner	The logistics is very fast, first give a 5-star praise, use it and then say	JD.com
good friend	Battery Reconditioner	Just picked it up Haven't used it yet Will let you know when I do	JD.com
good friend	Battery Reconditioner	Just got in, feel pretty good. Appearance style is very innovative, use for a period of time to know how the effect! If the effect is good, will come back to highly recommended, I hope the boss more recommended good goods to everyone!	JD.com
good friend	Battery Reconditioner	New style, fine workmanship, fast logistics, looking forward to the effect!	JD.com
good friend	Battery Reconditioner	Fast shipping, pretty good quality, as described by the seller, very satisfied!	JD.com
good friend	Battery Reconditioner	Goods received. Packaging is very good. Logistics quickly. Merchant service attitude is very good.	JD.com
good friend	Battery Reconditioner	Fast shipping, pretty good quality, as described by the seller, very satisfied!	JD.com
good friend	Battery Reconditioner	Baby is good, use it well continue	JD.com
good friend	Battery Reconditioner	This user feels the product is better	JD.com
good friend	Battery Reconditioner	can	JD.com

good friend	Battery Reconditioner	The product can be,feel the battery is really better than before, use a period of time to follow up on the evaluation	JD.com
good friend	Battery Reconditioner	Goods have been received, received the goods have begun to test, a short period of time can not be seen the effect, and so have the effect and then follow up.	JD.com
good friend	Battery Reconditioner	The feeling of using it after receiving it is that it slowly repairs the cell phone battery and should do the trick.	JD.com
good friend	Battery Reconditioner	The goods have been received, the logistics is very fast, very good, the battery recovery is very good	JD.com
good friend	Battery Reconditioner	Repairing the charger cable works great👍	JD.com
good friend	Battery Reconditioner	I have received the item I purchased. Thank you.	JD.com
good friend	Battery Reconditioner	[Lots of love] [Lots of love] [Lotsof love]	JD.com
good friend	Battery Reconditioner	Repairing cell phone batteries takes time, black technology is powerful	JD.com
good friend	Battery Reconditioner	The logistics is very fast, the packaging is very good, the workmanship is fine, the quality is good.	JD.com
good friend	Battery Reconditioner	Got it! Nice! Saw the instructions to remind the method! I hope it works.	JD.com
good friend	Battery Reconditioner	Fast logistics and good customer service!	JD.com
good friend	Battery Reconditioner	This item works very well worth recommending everyone to buy it.	JD.com
good friend	Battery Reconditioner	Matches the description well, it works after using it once, will keep using it for a while, the phone is saved[love it]	JD.com
good friend	Battery Reconditioner	Goods have been received logistics very fast service is very good	JD.com
good friend	Battery Reconditioner	Received the item, works great, following the store.	JD.com
good friend	Battery Reconditioner	Packaging can be	JD.com
good friend	Battery Reconditioner	Very nice👍👍👍😌😌😌👍😌👌😌😌	JD.com
good friend	Battery Reconditioner	The stuff is very good, fast delivery, very satisfied, next time to buy things in your home.	JD.com
good friend	Battery Reconditioner	This goods delivered in a timely manner, there is no use I do not know yet, waiting for practice test, then comment on the good or bad!	JD.com
good friend	Battery Reconditioner	Haven't used it yet, don't know if it works	JD.com
good friend	Battery Reconditioner	What a great deal to have. protects the phone	JD.com
good friend	Battery Reconditioner	I'll comment after I use it.	JD.com
good friend	Battery Reconditioner	Fast shipping, pretty good quality, as described by the seller, very satisfied!	JD.com
good friend	Battery Reconditioner	Repair quickly Goods are good Charge quickly	JD.com
good friend	Battery Reconditioner	fitted it. First time I felt something was wrong. After communicating with the store, I realized that the product itself structure. Try again, can be used. To be used later effect.	JD.com
good friend	Battery Reconditioner	Received and charged better.	JD.com
good friend	Battery Reconditioner	Things work well, before the phone battery does not work, it is very good to operate, in the future, no longer need to be afraid of the battery is not enough, praise!	JD.com
good friend	Battery Reconditioner	old phone charged seven or eight hours, today there is still quite a lot of electricity, and the bluetooth headset is also charged, than the first few days with a lot of time, and the battery is very stable, good, [Big Love] [Big Love] [Big Love] [Big Love] [Big Love]	JD.com
good friend	Battery Reconditioner	Goods have been received, charged for a while do not know how the effect? First give a favorable comment, the effect is good later and then add a favorable comment!	JD.com
good friend	Battery Reconditioner	Good price, good quality and very practical.	JD.com
good friend	Battery Reconditioner	The product was received and was very satisfied with the positive feedback, thank you.	JD.com
good friend	Battery Reconditioner	Being used, results to follow	JD.com
good friend	Battery Reconditioner	I don't know how it works, but I'll comment later! Anyway, I'll give it a 5-star review first!	JD.com
good friend	Battery Reconditioner	Cell phone battery repairer received! Logistics is fast, easy to use, cell phone battery back to normal!	JD.com

good friend	Battery Reconditioner	Goods received, the seller shipped quickly, logistics is also fast, well wrapped, beautiful appearance, is trying, I hope it is the same as described, looking forward to the effect.	JD.com
good friend	Battery Reconditioner	The look is a bit handsome Oh, voltage and current uninterrupted real-time monitoring, there are some codes, do not know what it means, it looks so professional look, with a few days and then follow up the evaluation[cool]	JD.com
good friend	Battery Reconditioner	The goods have been received and are both easy and quite good to use.	JD.com
good friend	Battery Reconditioner	It's easy to use.	JD.com
good friend	Battery Reconditioner	Things received ah, the effect of it, try to see at night, the boss attitude is very good, I believe that the goods must also be very good!	JD.com
good friend	Battery Reconditioner	Cell phone battery repairer has been received, the effect is good	JD.com
good friend	Battery Reconditioner	Very good and merchants describe the same is straight trust merchants are genuine have to dare to act quickly!	JD.com
good friend	Battery Reconditioner	Looking forward to the results and restoring my phone	JD.com
good friend	Battery Reconditioner	I bought online to extend the life of the battery goods, in today's time finally received, logistics is very fast, the business attitude is very good, I like the goods, the business is also a gift of data line, recently my cell phone is fully charged when the power is consumed very quickly, so I purchased online to repair the battery goods to use a little bit of the quality of the very good, if there is a like the goods on the relatives! Do not hesitate, hurry to order it!	JD.com
good friend	Battery Reconditioner	Good👌	JD.com
good friend	Battery Reconditioner	Customer service is very good, the delivery is not bad, I hope the boss business is getting better and better!!!! Shipping is fast, arrived in two or three days, this color I love, cost-effective, verynice	JD.com
good friend	Battery Reconditioner	Battery restorer and charger all received in good condition. The owner is enthusiastic and courteous shipping is very fast and the quality of the product is good. Give kudos and 5star excellent rating.	JD.com
good friend	Battery Reconditioner	Very good shopping experience good reviews	JD.com
good friend	Battery Reconditioner	Good review and fast shipping.	JD.com
good friend	Battery Reconditioner	The logistics is fast the quality is very good, I like it very much.	JD.com
good friend	Battery Reconditioner	Good quality! Worth recommending! The effect is used to follow up the evaluation!	JD.com
good friend	Battery Reconditioner	All other aspects are good, more beautiful and generous, but I feel that the charging speed is too slow, a night is not enough to charge a cell phone.	JD.com
good friend	Battery Reconditioner	Finally received the baby I need, the stuff is very good, the price is good and cheap, thanks to!The owner's attitude is very professional and enthusiastic, ask questions and reply quickly, I asked a lot of questions, he does not feel annoyed, they will seriously answer me. And then say baby, just what I need, when I received the packaging is complete, after opening the surprise, baby than I imagined even better!Had to have to give a thumbs up. I will come back next time I need it!	JD.com
good friend	Battery Reconditioner	Not bad. It works.	JD.com
good friend	Battery Reconditioner	This user feels the product is better	JD.com
good friend	Battery Reconditioner	technology, I hope the cell phone battery repair effect will be a surprise.	JD.com
good friend	Battery Reconditioner	Baby's quality is great.	JD.com
good friend	Battery Reconditioner	It works great, still charges quickly, and has a screen display feels like reliable quality.	JD.com
good friend	Battery Reconditioner	Trying it out and waiting to see how it works!	JD.com
good friend	Battery Reconditioner	Baby has received, logistics is very fast, packaging is very good, now in the process of restoration, give five stars[kudos][kudos[kudoskudos[] [kudos] [kudos] [kudos] [kudos] [kudos] [kudos] [kudos] [kudos][kudos] [k	JD.com
good friend	Battery Reconditioner	Repairing cell phone batteries is very good! Prompt shipping and fast logistics!	JD.com
good friend	Battery Reconditioner	Just received the goods. Logistics is fast. Packaging is very good. It is genuine.	JD.com

good friend	Battery Reconditioner	Fast logistics, good packaging, and instructions, I'll try it tonight[smile]	JD.com
good friend	Battery Reconditioner	I received the baby and hope to achieve the desired results.	JD.com
good friend	Battery Reconditioner	Very satisfied Quality is very good this phone used to charge very well No problems at all	JD.com
good friend	Battery Reconditioner	It's very good, the battery is especially durable after using it, very satisfied!	JD.com
good friend	Battery Reconditioner	This user feels the product is better	JD.com
good friend	Battery Reconditioner	Just as introduced, good packaging and pretty fast logistics.	JD.com
good friend	Battery Reconditioner	adhere to a period of time to see the effect, and then follow up!	JD.com
good friend	Battery Reconditioner	I just received the goods this morning, immediately to try, there are different numbers, we do not know what it is, but still look forward to good results.	JD.com
good friend	Battery Reconditioner	Logistics is very fast, the product and the introduction of complementary products good monitor	JD.com
good friend	Battery Reconditioner	The stuff works great.	JD.com
good friend	Battery Reconditioner	I'll give you a good review first, and then come back after I've used it for a while	JD.com
good friend	Battery Reconditioner	Logistics is fast![Smile] [Smile[Sm] [Smile] [Smile]Good value for money![Smile]Smile[Smile]Smile] [Smile] [Smile]	JD.com
good friend	Battery Reconditioner	Goods to receive, logistics is very fast, I am very satisfied, give five stars.	JD.com
good friend	Battery Reconditioner	It should be able to repair the cell phone battery, wait for a period of time to use it and then come back to follow up the effect of the use of the repairer.	JD.com
good friend	Battery Reconditioner	The product received wait to see laugh through and then follow up.	JD.com
good friend	Battery Reconditioner	I'm very happy with it! I am very satisfied, thank you!	JD.com
good friend	Battery Reconditioner	The product is great, the phone is as good as new after charging it	JD.com
good friend	Battery Reconditioner	This user feels the product is better	JD.com
good friend	Battery Reconditioner	Energized and connected, running normally. Appearance design looks good,[smile]as for the effect of what, a moment to say, slightly expensive, service, fast delivery.	JD.com
good friend	Battery Reconditioner	Very detailed instructions, well packaged	JD.com
good friend	Battery Reconditioner	Received it and will try it out first.	JD.com
good friend	Battery Reconditioner	It's good.	JD.com
good friend	Battery Reconditioner	Logistics is very fast oh, next time will come back to buy[smile] [smile] [smile[smile] [smile]	JD.com
good friend	Battery Reconditioner	Logistics is fast, service is thoughtful, as described, recommendable	JD.com
good friend	Battery Reconditioner	afternoon, butwouldn'tfor half an hour at60%, then unplugged it, restarted charging, and thenfully charged it in about1hour. If I run out of power and then recharge will it fully charge, the	JD.com
good friend	Battery Reconditioner	The object received matches the description of the store, as for the effect, we have to wait until after the use to confirm whether it is useful.	JD.com
good friend	Battery Reconditioner	Cell Phone Battery Repair Magic is nice and well packaged.	JD.com
good friend	Battery Reconditioner	Merchant shipment is very, the courier received, the packaging is very good and the same as described, to be used and then evaluate, will recommend friends to buy, be five-star praise.	JD.com
good friend	Battery Reconditioner	Good value for money! The goods are real! Follow up review after use! 5 stars!	JD.com
good friend	Battery Reconditioner	Very good quality and good results Original Genuine	JD.com
good friend	Battery Reconditioner	Cell Phone Battery Repair Magic Universal Extended Battery Life Charger received, the packaging is intact, the quality is good, fine workmanship, exquisite appearance, repair effect is good, give full five-star praise.	JD.com
good friend	Battery Reconditioner	Received, the appearance looks good, tried charging, basically normal, just do not know how to repair the battery effect? Waiting for a period of time and then follow up.	JD.com
good friend	Battery Reconditioner	Received today, looking forward to the effect, compact and easy to carry, worth buying!	JD.com
good friend	Battery Reconditioner	Product quality is good, logistics is very fast, is genuine, expect everyone to rest assured that the purchase!	JD.com

good friend	Battery Reconditioner	、	JD.com
good friend	Battery Reconditioner	Looks high tech I don't know if it works I'll comment after I use	JD.com
good friend	Battery Reconditioner	This user feels the product is better	JD.com
good friend	Battery Reconditioner	Baby received yet do not know whether it is good to use after the use of the follow-up comment!	JD.com
good friend	Battery Reconditioner	Things received use for a period of time is not very good still give you a good review hope you guys do better and better congratulations on wealth	JD.com
good friend	Battery Reconditioner	I hope my cell phone battery will be full soon[hee][hee][hee hee][hee	JD.com
good friend	Battery Reconditioner	more than three years a day charging to charge several times the battery is quickly dead, I saw the video ads say this repairer charging can be repaired battery, I bought a business service is very good, very patient to teach me how to use it, the quality is very good, like the battery is quickly dead you can buy a to try, the price concessions wish the boss prosperous business, Happy New Year!	JD.com
good friend	Battery Reconditioner	The quality of the item is very good, well made, superb appearance, and the restoration is excellent.	JD.com
good friend	Battery Reconditioner	Baby received, logistics is very fast, the boss shipped in time, the phone battery is not used, buy to repair the old cell phone battery, can repair, the phone battery than before with a long time, this time do not have to change the phone, repairer is worth recommending.	JD.com
good friend	Battery Reconditioner	Just received the goods, the logistics is very strong, have not yet tried.	JD.com
good friend	Battery Reconditioner	It's good.	JD.com
good friend	Battery Reconditioner	service is very good, logistics is also very fast, five-star praise!	JD.com
good friend	Battery Reconditioner	Tried itfor1month and our batteries take longer to charge than before anyway. Whatever the principle is, it's good if it works good?	JD.com
good friend	Battery Reconditioner	Used two two charging3have six hours, the effect is still some, see the back of the use and then Lei evaluation, but I hope that as described useful, first to give a good review	JD.com
good friend	Battery Reconditioner	Good to use originally fully charged with3hours, yesterday just started to use Doting two hours. Thank you for the express customer service teaching and understanding, stage yi have staff, in special times adhere to the work. Wish the boss a prosperous business and prosperity!	JD.com
good friend	Battery Reconditioner	Baby high usage, comparable, applicable to all types of cell phones, the store has a good service attitude, has recommended the baby to friends, thanks to the store! Five-star praise!	JD.com
good friend	Battery Reconditioner	Over the past few days, according to the business and the instructions to come up to operate, not only the phone can be repaired, even my Xiaomi Tablet can be repaired. This is still my experience with the merchant on the customer service, and it is really good! The service patience and attitude of the merchant is great!	JD.com
good friend	Battery Reconditioner	Battery Repairer is okay, it's just a little bit slightly troublesome to do, it can fix the battery and cell phone lagging, in general it's okay	JD.com
good friend	Battery Reconditioner	Express received, the quality of goods is also very good, the packaging is very strong, no damage, style and	JD.com
good friend	Battery Reconditioner	In kind is very good, with the seller's description of the exact same match, logistics fast seller's service attitude is also good, the most important quality is very good, this is a very satisfied with the shopping.	JD.com
good friend	Battery Reconditioner	Because of online shopping for fear of return and replacement trouble, so it is directly consult the customer service to buy, customer service is very good recommended very good!	JD.com
good friend	Battery Reconditioner	The first time to buy things in this, of course, is still very hesitant, did not expect to receive a very pleasant surprise, the goods are consistent with the description, the logistics is also very powerful, give a favorable review to support.	JD.com
good friend	Battery Reconditioner	Merchants are very good to talk to oh, very polite ah, wish merchants more and more prosperous business.	JD.com
good friend	Battery Reconditioner	Fixer2weeks now, it's working great and the phone lag problem is solved	JD.com
good friend	Battery Reconditioner	Jitterbug someone sells288, Poundlandmore than70, you know, whether it works or not, saving money is happy	JD.com
good friend	Battery Reconditioner	Racer brush over, Racer178a, heremore than70dollars a, really Pinduoduo cheap ah haha	JD.com

good friend	Battery Reconditioner	Indeed very good, is genuine, with2weeks, the effect is very obvious, the battery repair is very good	JD.com
good friend	Battery Reconditioner	Really can be oh, good stuff to come, good comments	JD.com
good friend	Battery Reconditioner	It's noticeable after a week of use, the range is longer, good review!	JD.com
good friend	Battery Reconditioner	Things are good, is why the boss does not sell higher, a few cheap make people feel can not believe that there is an effect, in general it does work!	JD.com
good friend	Battery Reconditioner	Repair the battery good helper, the old phone can also be used, the price is not expensive, than spend thousands of dollars to buy a cell phone is worth it!	JD.com
good friend	Battery Reconditioner	Thought it was aUSBflash drive, but it turned out to be a black technology to repair the battery, it is really nothing strange, I have to say, a little effect	JD.com
good friend	Battery Reconditioner	Poor can't afford a new phone, stuff tested, should the phone last another2years, hey the mouthpiece issue is hurting me	JD.com
good friend	Battery Reconditioner	continue to use for a period of time, good use in the come to buy	JD.com
good friend	Battery Reconditioner	use of the effect is very obvious, it is indeed a very cost-effective thing to save thousands of dollars to change the phone, the operation is also particularly easy, not for a while on the good recommended!	JD.com
good friend	Battery Reconditioner	using this restorer, now almost can watch fast5hours it, receive calls1day no problem it, this phone is very old, can use so long, really happy. This is a genuine product, I really recommend it!	JD.com
good friend	Battery Reconditioner	the hands of a, indeed the battery with a long time, the phone will not be so stuck, the boss said with90days no effect can be returned, something useful on the return, the times are changing ah, technology in the development of our countryside are an eye-opener	JD.com
good friend	Battery Reconditioner	to change the battery, after repairing the phone with a longer time some, and does not heat up, and also automatically clean up the phone system cache garbage, the phone has a significantly smooth, the boss said that often used to repair will be better.	JD.com
good friend	Battery Reconditioner	AppleXbattery life before a month to drop5percent, with the previous80percent, used4weeks, is still80percent, really awesome, recommend recommended	JD.com
good friend	Battery Reconditioner	High black technology products, the store said it can protect the phone battery by controlling the current, in fact, it is right, many times the voltage is not stable we do not know. Now the cell phone battery with a little bit of the next began to decline in performance, that is, the current is not stable, this try a day is indeed very good.	JD.com
good friend	Battery Reconditioner	Got it, looks like a nice package. I hope it really works.	JD.com
good friend	Battery Reconditioner	This user feels the product is better	JD.com
good friend	Battery Reconditioner	Got the stuff and looking forward to the results. Kudos!	JD.com
good friend	Battery Reconditioner	This user feels the product is better	JD.com
good friend	Battery Reconditioner	This user feels the product is better	JD.com
good friend	Battery Reconditioner	This user feels the product is better	JD.com
good friend	Battery Reconditioner	Baby has arrived, the quality is very good, next time continue to buy	JD.com
good friend	Battery Reconditioner	The goods have been received, exactly as described, quite good.	JD.com
good friend	Battery Reconditioner	This user feels the product is better	JD.com
good friend	Battery Reconditioner	This user feels the product is better	JD.com
good friend	Battery Reconditioner	I haven't used it yet, I'll comment afterward.	JD.com
good friend	Battery Reconditioner	liars	JD.com
good friend	Battery Reconditioner	Rip-off<br>Not as good as a regular charging device, deluded	JD.com
good friend	Battery Reconditioner	This user feels the product is better	JD.com
good friend	Battery Reconditioner	This user feels the product is better	JD.com
good friend	Battery Reconditioner	(of an unmarried couple) be close	JD.com

good friend	Battery Reconditioner	Baby received, very good workmanship, logistics is very fast,, packaging intact! Next time if you need, and then buy this home goods, for merchants and logistics points[love][love][love][love] [love][love][love] [love] [love] [love] [love] [love] [love] [love] [love] [love] [love] [love] [love] [love] [love][love] [love] [love	JD.com
good friend	Battery Reconditioner	Look at the evaluation of the effect	JD.com
good friend	Battery Reconditioner	This user feels the product is better	JD.com
good friend	Battery Reconditioner	Rechargeable battery: have started to use, looking forward to be able to repair the battery<br>Product appearance: the appearance of the design is very good	JD.com
good friend	Battery Reconditioner	Good and practical, cheap and easy to use	JD.com
good friend	Battery Reconditioner	This user feels the product is better	JD.com
good friend	Battery Reconditioner	This user feels the product is better	JD.com
good friend	Battery Reconditioner	This user feels the product is better	JD.com
good friend	Battery Reconditioner	It's a lie. It doesn't work.	JD.com
good friend	Battery Reconditioner	Not yet used, not yet know how the effect, first praise.	JD.com
good friend	Battery Reconditioner	impostor	JD.com
good friend	Battery Reconditioner	genuine goods at fair prices	JD.com
good friend	Battery Reconditioner	I got it.	JD.com
good friend	Battery Reconditioner	This user feels the product is better	JD.com
good friend	Battery Reconditioner	The stuff works. It works better than the previous one. Logistics is also fast. Praise praise praise!	JD.com
good friend	Battery Reconditioner	This user feels the product is better	JD.com
good friend	Battery Reconditioner	This user feels the product is better	JD.com
good friend	Battery Reconditioner	This user feels the product is better	JD.com
good friend	Battery Reconditioner	The goods have been received, intact, the effect to use in the evaluation.	JD.com
good friend	Battery Reconditioner	The cell phone repairer is very well packaged, the logistics is very fast, and I used it right after I received it.	JD.com
good friend	Battery Reconditioner	Give him a good review. A very good review.	JD.com
good friend	Battery Reconditioner	This product is very good, really good, straight to buy, the	JD.com
good friend	Battery Reconditioner	[Lots of love][Lots of love][Lotsof love]	JD.com
good friend	Battery Reconditioner	The product has been received, have not yet used it, do not know if there is any effect, to be used to know.	JD.com
good friend	Battery Reconditioner	This user feels the product is better	JD.com
good friend	Battery Reconditioner	Delivered to the door of the shipment arrived relatively quickly, first give a positive review of the good to make wait for the next chase flat	JD.com
good friend	Battery Reconditioner	The quality is superb and the material is very comfortable to wear. Very happy shopping!	JD.com
good friend	Battery Reconditioner	This user feels the product is better	JD.com
good friend	Battery Reconditioner	I've been using it for a while now and haven't seen any change in my phone's battery.	JD.com
good friend	Battery Reconditioner	It looks good, it's very high class, and it's easy to carry it out of the house.	JD.com
good friend	Battery Reconditioner	Let's hope it works! Will follow up with a review!	JD.com
good friend	Battery Reconditioner	This user feels the product is better	JD.com
good friend	Battery Reconditioner	used a few days to evaluate. My cell phone battery with the battery is particularly easy to get hot, standby time is much less than the original, according to the store's instructions for several consecutive nights before going to bed to turn off the phone after charging, and now the phone battery is better than before, and not as hot as it used to be, I will continue to follow the battery repair instructions for battery repair, so that the battery is repaired to a certain degree, and the battery is repaired to a certain degree, and the battery is repaired to a certain degree.	JD.com

good friend	Battery Reconditioner	This user feels the product is better	JD.com
good friend	Battery Reconditioner	Received Haven't used it yet Good shape Corded with outlet Covered Good review	JD.com
good friend	Battery Reconditioner	I'm using it, we'll see after a while, how it works!	JD.com
good friend	Battery Reconditioner	The outer packaging is very good! I believe it's authentic, it must work well and the quality is definitely good! I'll follow up after using it!	JD.com
good friend	Battery Reconditioner	It's not a bad fixer-upper.	JD.com
good friend	Battery Reconditioner	experience: good<br>Display experience: clear<br>Product features: well	JD.com
good friend	Battery Reconditioner	inserted after seeing the data flash, three hours later they pulled out, check the phone is simply a surprise, the phone is the same as new, very smooth, really deeply in love with you, the appearance of small and beautiful, according to the store's requirements a week to clear it 2-3times, really friends clear after the cell phone is not stuck very smooth, this is the most satisfied with the purchase, the cell phone card card friends rest assured I'm very happy with this purchase, so don't worry about it!	JD.com
good friend	Battery Reconditioner	Repairing the phone is quite good, the phone is not stuck and not hot, fast charging, fast shipping, fast logistics.	JD.com
good friend	Battery Reconditioner	Goods have been received, look good, charging today to try, feel okay, tomorrow and then charging to see	JD.com
good friend	Battery Reconditioner	Really good use. You can buy it with confidence if you need[smile][smile]	JD.com
good friend	Battery Reconditioner	I have tried it and it feels good, I will follow up after using it for a while.	JD.com
good friend	Battery Reconditioner	This user feels the product is better	JD.com
good friend	Battery Reconditioner	Cell Phone Battery, Repair Magic received and started to use, trustworthy, the	JD.com
good friend	Battery Reconditioner	Fine and beautiful workmanship, space is very practical, fully functional, durable battery, good appearance, easy to use, good appearance	JD.com
good friend	Battery Reconditioner	Works well and is beautifully crafted	JD.com
good friend	Battery Reconditioner	The logistics is quite fast first give five stars to use in the additional comments	JD.com
good friend	Battery Reconditioner	This user feels the product is better	JD.com
good friend	Battery Reconditioner	This user feels the product is better	JD.com
good friend	Battery Reconditioner	Cell phone charging repairer received, the effect is good, the boss's attitude is very good, the delivery is very fast, to want to buy friends grab go buy it, it is really good!	JD.com
good friend	Battery Reconditioner	This user feels the product is better	JD.com
good friend	Battery Reconditioner	This user did not fill in a rating	JD.com
good friend	Battery Reconditioner	evening, I hope the product can be useful to repair the phone and battery.	JD.com
good friend	Battery Reconditioner	It's not bad to use! Can be! I'll buy it again.	JD.com
good friend	Battery Reconditioner	This user feels the product is better	JD.com
good friend	Battery Reconditioner	I don't know if it works I don't feel any difference for now	JD.com
good friend	Battery Reconditioner	This user feels the product is better	JD.com
good friend	Battery Reconditioner	Goods have been received, the appearance of a look very beautiful. Have not tried to use repair and then evaluate. Logistics is also very fast, thank you business owner.	JD.com
good friend	Battery Reconditioner	What kind of medicine is this? I don't see any dead bugs. It's killing my cat.	JD.com
good friend	Battery Reconditioner	Try it out and then comment!	JD.com
good friend	Battery Reconditioner	Got the stuff, tried it pretty well, good quality.	JD.com
good friend	Battery Reconditioner	clear, after the use of a more obvious effect, it seems that this product is worth buying, use a period of time and then follow up.	JD.com
good friend	Battery Reconditioner	's okay, average, recommended purchase[love it][love it][love it][loveit]	JD.com

good friend	Battery Reconditioner	Received the item, tried it today and it's good 🌹	JD.com
good friend	Battery Reconditioner	This user feels the product is better	JD.com
good friend	Battery Reconditioner	Affordable and good for the price.	JD.com
good friend	Battery Reconditioner	Battery Repair Magic received, the quality is very good, hope to have the effect will be back to buy again!	JD.com
good friend	Battery Reconditioner	This user feels the product is better	JD.com
good friend	Battery Reconditioner	Baby received great logistics quickly delivered to the door	JD.com
good friend	Battery Reconditioner	Very satisfied with the product	JD.com
good friend	Battery Reconditioner	Received the goods have not yet used, should be good	JD.com
good friend	Battery Reconditioner	hassles	JD.com
good friend	Battery Reconditioner	It's okay. It's easy to carry around. It's small.	JD.com
good friend	Battery Reconditioner	This user feels the product is better	JD.com
good friend	Battery Reconditioner	This user feels the product is better	JD.com
good friend	Battery Reconditioner	I want to return it. I want to return it. I want to return it.	JD.com
good friend	Battery Reconditioner	This user feels the product is better	JD.com
good friend	Battery Reconditioner	fancy this, good, used for a period of time, will, as always, continue to support, logistics is also very fast service, shipping very fast, packaging very carefully, the space is very practical, energy saving and power saving, with a very cost-effective, in kind and everyone describes is the same, the brand is worthy of trust love, recently too busy to confirm the late, the quality of the good, the express point of praise , praise must be praised, the service is in place	JD.com
good friend	Battery Reconditioner	This user feels the product is better	JD.com
good friend	Battery Reconditioner	Good quality goods, fast logistics	JD.com
good friend	Battery Reconditioner	Commodity received, very satisfied, especially like, the quality is very good, fast delivery, repair the phone fast, after repairing the phone in also not lagging, very good.	JD.com
good friend	Battery Reconditioner	This user feels the product is better	JD.com
good friend	Battery Reconditioner	This user feels the product is better	JD.com
good friend	Battery Reconditioner	Good quality, fast logistics, good value for money	JD.com
good friend	Battery Reconditioner	This user feels the product is better	JD.com
good friend	Battery Reconditioner	acceptable	JD.com
good friend	Battery Reconditioner	Just used it for three days, the effect is not obvious	JD.com
good friend	Battery Reconditioner	originally my cell phone battery is not very good,, now buy this charger to repair the cell phone battery is the cell phone electricity is good!	JD.com
good friend	Battery Reconditioner	The quality of the stuff is okay and the speed of the delivery is also very powerful	JD.com
good friend	Battery Reconditioner	Has received the battery repairer, very beautiful now. Accessories full, very satisfied!	JD.com
good friend	Battery Reconditioner	This user feels the product is better	JD.com
good friend	Battery Reconditioner	Fine and beautiful workmanship	JD.com
good friend	Battery Reconditioner	I'll comment after I've used it.	JD.com
good friend	Battery Reconditioner	Good quality and quantity of goods, fast logistics. Courier boy good service	JD.com
good friend	Battery Reconditioner	Logistics is very fast, packaging is exquisite, baby received, fully functional, repair effect is good, will recommend friends and relatives to buy.	JD.com
good friend	Battery Reconditioner	This user feels the product is better	JD.com
good friend	Battery Reconditioner	This user feels the product is better	JD.com
good friend	Battery Reconditioner	This user feels the product is better	JD.com

good friend	Battery Reconditioner	an excellent	JD.com
good friend	Battery Reconditioner	This user did not fill in a rating	JD.com
good friend	Battery Reconditioner	just used	JD.com
good friend	Battery Reconditioner	I haven't used it yet, I'll comment afterward.	JD.com
good friend	Battery Reconditioner	Hello everyone:This black technology, repair while charging, cell phone repairer, good-looking, good repair effect, I used the phone runs faster, please have the need to friends rest assured that the purchase and use……	JD.com
good friend	Battery Reconditioner	This user feels the product is better	JD.com
good friend	Battery Reconditioner	This user feels the product is better	JD.com
good friend	Battery Reconditioner	Logistics was fast. The product is very good. Introduced to renew the purchase in the future.	JD.com
good friend	Battery Reconditioner	Satisfied with the merchandise[smile]	JD.com
good friend	Battery Reconditioner	Received the goods[cool]pretty good[cool]give 5 stars[cool]	JD.com
good friend	Battery Reconditioner	The item was received, the delivery was fast and the stuff is good, you can consider repurchasing!	JD.com
good friend	Battery Reconditioner	This user feels the product is better	JD.com
good friend	Battery Reconditioner	This user feels the product is better	JD.com
good friend	Battery Reconditioner	This user feels the product is better	JD.com
good friend	Battery Reconditioner	This user feels the product is better	JD.com
good friend	Battery Reconditioner	The item has been received and the item was shipped with fast and good logistics.	JD.com
good friend	Battery Reconditioner	Looks good, haven't used it yet, should work well	JD.com
good friend	Battery Reconditioner	(of an unmarried couple) be close	JD.com
good friend	Battery Reconditioner	It works.	JD.com
good friend	Battery Reconditioner	Fast charging, good quality, restorative effect on batteries, very satisfactory	JD.com
good friend	Battery Reconditioner	This user feels the product is better	JD.com
good friend	Battery Reconditioner	This user feels the product is better	JD.com
good friend	Battery Reconditioner	This user feels the product is better	JD.com
good friend	Battery Reconditioner	I received the goods, it's very good, I'll introduce my friends to buy it	JD.com
good friend	Battery Reconditioner	Boss baby received, the boss service is very good, I am using a little. I wish the boss a prosperous business. I give the boss five-star praise.	JD.com
good friend	Battery Reconditioner	Used it as soon as I received it. Take your time to see the effect	JD.com
good friend	Battery Reconditioner	This user did not fill in a rating	JD.com
good friend	Battery Reconditioner	The goods have been received, the quality is very good, the logistics is very fast, the merchant has a good reputation.	JD.com
good friend	Battery Reconditioner	This user feels the product is better	JD.com
good friend	Battery Reconditioner	Twopricesone dayin the morning, $53 in the afternoon. What's the point of charging $10 more? It makes sense	JD.com
good friend	Battery Reconditioner	It's not very effective.	JD.com
good friend	Battery Reconditioner	This user feels the product is better	JD.com
good friend	Battery Reconditioner	Quite good, good-looking and practical, the phone has been used for many years, the battery storage function is not good, for many reasons do not want to replace the new phone, see this good thing really solved a big problem for me, love it!	JD.com
good friend	Battery Reconditioner	This user feels the product is better	JD.com

good friend	Battery Reconditioner	Show experience: charging head plugged in battery▯Fixer in<br><br>Battery charging: cell phone battery▯Problems well<br>Product design: test it out<br>Next time sell fixer$60 is too expensive<br>Service Chat Bonus Reward$3	JD.com
good friend	Battery Reconditioner	Got it. It's pretty good.	JD.com
good friend	Battery Reconditioner	This user feels the product is better	JD.com
good friend	Battery Reconditioner	This user feels the product is better	JD.com
good friend	Battery Reconditioner	This user feels the product is better	JD.com
good friend	Battery Reconditioner	Received the item, it does work well and can pass it on to friends to buy.	JD.com
good friend	Battery Reconditioner	This user feels the product is better	JD.com
good friend	Battery Reconditioner	This user feels the product is better	JD.com
good friend	Battery Reconditioner	This user feels the product is better	JD.com
good friend	Battery Reconditioner	phone battery is not very good, not long ago I was going to go to change the battery, but I heard that after changing the battery, the phone is not very valuable. Better to fix it with this black technology!	JD.com
good friend	Battery Reconditioner	This user feels the product is better	JD.com
good friend	Battery Reconditioner	It doesn't match what the internet says.	JD.com
good friend	Battery Reconditioner	(of an unmarried couple) be close	JD.com
good friend	Battery Reconditioner	This user feels the product is better	JD.com
good friend	Battery Reconditioner	This user feels the product is better	JD.com
good friend	Battery Reconditioner	I haven't used it yet.	JD.com
good friend	Battery Reconditioner	Used it for a few days to evaluate, no effect for the time being	JD.com
good friend	Battery Reconditioner	This user feels the product is better	JD.com
good friend	Battery Reconditioner	Don't fall for it, it doesn't work at all, it doesn't work for two days, the plug is too loose, the first day there is fast charging, the second day is slow charging, and then the plug is too loose, it can't be charged.	JD.com
good friend	Battery Reconditioner	This user feels the product is better	JD.com
good friend	Battery Reconditioner	It's very technological, and it's high-tech black, and it's particularly effective.	JD.com
good friend	Battery Reconditioner	This user feels the product is better	JD.com
good friend	Battery Reconditioner	Just received, the appearance of this good, the specific effect of the use of after evaluation	JD.com
good friend	Battery Reconditioner	This user feels the product is better	JD.com
good friend	Battery Reconditioner	It's real, it works, so buy it now!	JD.com
good friend	Battery Reconditioner	This user feels the product is better	JD.com
good friend	Battery Reconditioner	Smart phone battery universal restorer received, look beautiful, compact, easy to use, good repair effect, long warranty period! Digital display parameters are clear, after the cell phone battery will have a doctor, can make the battery sub-health state can be normal recovery!	JD.com
good friend	Battery Reconditioner	Take back the night to an old phone, repair a moment, to the next morning, really an advertisement are not, don't five-star praise, quickly, logistics courier hard work!	JD.com
good friend	Battery Reconditioner	Have aniphone4s, we all know, very classic, the battery repair effect is very good, praise	JD.com
good friend	Battery Reconditioner	I just started using it10days and brought it in to recharge, and I feel as if the battery has lengthened a bit.	JD.com
good friend	Battery Reconditioner	Things are very satisfied Oh, beautiful and stylish atmosphere, customer service is super patient, service is meticulous and attentive, the big brands are not the same!	JD.com
good friend	Battery Reconditioner	resurrection, originally wanted to change the phone now do not have to change!	JD.com

good friend	Battery Reconditioner	The product received, genuine, the use of the effect is very good, fine workmanship and beautiful, Overseas Chinese Gog affordable, thoughtful service, has given 5stars	JD.com
good friend	Battery Reconditioner	Bought this hoping it works so I don't have to spend more money on a new phone	JD.com
good friend	Battery Reconditioner	have time, my cell phone battery drains strangely, up to 30 or 40 pcs of power will drop off at once, it's just annoying, and there's still a problem with charging it when it's turned off, the	JD.com
good friend	Battery Reconditioner	After using the reconditioned battery two or three times, it still feels better than when it was not reconditioned, so I hope it will be better in the future!	JD.com
good friend	Battery Reconditioner	prompted to replace, no power to boot can not open, used the repairer to repair, but also can charge and boot up, really can be	JD.com
good friend	Battery Reconditioner	Vivocell phone a grid of electricity is also gone, can not charge into the electricity is this battery repair really activate the phone, very happy thanks to the business of good products! May this good product can help more people in need, I wish the boss a prosperous business!	JD.com
good friend	Battery Reconditioner	before anyway. Whatever the principle is, it's good if it works well	JD.com
good friend	Battery Reconditioner	the effect is not very obvious, I hope that after a few more times will have the effect, the later with a good will recommend to friends.	JD.com
good friend	Battery Reconditioner	Is a repairer just bought back, always thought can not have no effect, but after the boss general patience guide, charge twice a week, more than three weeks on the effect, and now it is true that the cell phone is more durable than the previous battery, it is worth buying!	JD.com
good friend	Battery Reconditioner	Used two two charging3have six hours, the effect is still some, see the back of the use and then Lei evaluation, but I hope that as described useful, first to give a good review	JD.com
good friend	Battery Reconditioner	Good to use originally fully charged with 3hours, yesterday just started to use Doting two hours. Thank you for the express customer service teaching and understanding, stage yi have staff, in special times adhere to the work. Wish the boss a prosperous business and prosperity!	JD.com
good friend	Battery Reconditioner	Baby high usage, comparable, applicable to all types of cell phones, the store has a good service attitude, has recommended the baby to friends, thanks to the store! Five-star praise!	JD.com
good friend	Battery Reconditioner	Over the past few days, according to the business and the instructions to come up to operate, not only the phone can be repaired, even my Xiaomi Tablet can be repaired. This is still my experience with the merchant on the customer service, and it is really good! The service patience and attitude of the merchant is great!	JD.com
good friend	Battery Reconditioner	Battery Repairer is okay, it's just a little bit slightly troublesome to do, it can fix the battery and cell phone lagging, in general it's okay	JD.com
good friend	Battery Reconditioner	After charging it once I feel that my cell phone standby time has increased. Hopefully it will work very well after a few fixes.	JD.com
good friend	Battery Reconditioner	Cell phone battery charging repairer received. Not too big. Chic appearance. Fine workmanship. The digital display is clear. Hope to achieve the actual effect. Five stars. The use of the situation later review.	JD.com
good friend	Battery Reconditioner	Repairer is good, mecha style, voltage and current stable output, display, battery repair god, full five praise!	JD.com
good friend	Battery Reconditioner	the repair of two cell phones, both significantly faster than the original, wait for a period of time to see the effect of the battery, thanks to customer service patience and effective help.	JD.com
good friend	Battery Reconditioner	Current and voltage, when monitoring. Peace of mind, efficient heat dissipation.	JD.com
good friend	Battery Reconditioner	Baby received, logistics God speed. Multiple functions, battery repair. Trendy shape, extremely cool. Dazzling light, more attention.	JD.com
good friend	Battery Reconditioner	Trial1month, power a lot more, a day and a half are used up, now able to stop for a day, good good, worth buying	JD.com
good friend	Battery Reconditioner	Goods received! After a few days of charging experiments, the battery control to stabilize the fast discharge, charging speed adjustment to strengthen a lot. And the previous charging effect is not the same.	JD.com
good friend	Battery Reconditioner	battery repairer, the cell phone life is extended a lot! Powerful!	JD.com

good friend	Battery Reconditioner	The previous cell phone battery does not work, what do not do on the drop power, which stored a lot of things do not want to change the phone, by chance searched for this product, buy to try the effectiveness of the	JD.com
good friend	Battery Reconditioner	As time goes by, the cell phone charge once the use of time is getting shorter and shorter, saw this device and bought it, beautifully packaged, exquisite device, I believe that, as the instructions say, it will have the effect of extending the battery life and improve the performance of the phone.	JD.com
good friend	Battery Reconditioner	have a good repair effect relative to the cell phone battery, you need to use the repairer for a long time, charging the cell phone, the	JD.com
good friend	Battery Reconditioner	Fast logistics, baby is very good, charging fast, can fix the battery is better, in the use of the results after seeing.	JD.com
good friend	Battery Reconditioner	logistics is fast, color digital display, beautiful and exquisite, repair batteries, more durable, thanks to the business.	JD.com
good friend	Battery Reconditioner	good, my cell phone original full of electricity can only use three hours, use this repairer charge an hour later can use a day, Jiao Ge magic, worth buying, thanks to the business good product!	JD.com
good friend	Battery Reconditioner	Have just tested it on my laptop and the results have improved. Booting up is faster than it was, opening web pages, files and videos have all been sped up. Ready to test a few more times to see if there is still an improvement.	JD.com
good friend	Battery Reconditioner	This product has been tried. Tried three old cell phones and felt like they opened and surfed the web faster than they did. Now trying an old laptop to see how it works. The product does not fail to deliver, good	JD.com
good friend	Battery Reconditioner	smoother. Customer service is very good and explains very clearly	JD.com
good friend	Battery Reconditioner	Express received, the quality of goods is also very good, the packaging is very strong, no damage, style and	JD.com
good friend	Battery Reconditioner	In kind is very good, with the seller's description of the exact same match, logistics fast seller's service attitude is also good, the most important quality is very good, this is a very satisfied with the shopping.	JD.com
good friend	Battery Reconditioner	My friend recommended it, and it didn't disappoint me, it's what I wanted, so if you like it, you can go ahead and buy it, you won't be disappointed.	JD.com
good friend	Battery Reconditioner	the quality or other are very, like, and the seller described exactly the same, like friends can recommend buying, very satisfied.	JD.com
good friend	Battery Reconditioner	Quite good, inexpensive, before the other home also bought, but the quality of this one is still better, workmanship and other things are more fine, it is worth getting!	JD.com
good friend	Battery Reconditioner	Because of online shopping for fear of return and replacement trouble, so it is directly consult the customer service to buy, customer service is very good recommended very good!	JD.com
good friend	Battery Reconditioner	The first time to buy things in this, of course, is still very hesitant, did not expect to receive a very pleasant surprise, the goods are consistent with the description, the logistics is also very powerful, give a favorable review to support.	JD.com
good friend	Battery Reconditioner	Express is very powerful, not a few days to arrive, the style of fashion atmosphere, the quality is very good, the seller shipped fast. Very satisfied.	JD.com
good friend	Battery Reconditioner	Merchants are very good to talk to oh, very polite ah, wish merchants more and more prosperous business.	JD.com
good friend	Battery Reconditioner	Fixer2weeks now, it's working great and the phone lag problem is solved	JD.com
good friend	Battery Reconditioner	Jitterbug someone sells288, Poundlandmore than70, you know, whether it works or not, saving money is happy	JD.com
good friend	Battery Reconditioner	Racer brush over, Racer178a, heremore than70dollars a, really Pinduoduo cheap ah haha	JD.com
good friend	Battery Reconditioner	Indeed very good, is genuine, with2weeks, the effect is very obvious, the battery repair is very good	JD.com
good friend	Battery Reconditioner	Really can be oh, good stuff to come, good comments	JD.com
good friend	Battery Reconditioner	It's noticeable after a week of use, the range is longer, good review!	JD.com
good friend	Battery Reconditioner	Things are good, is why the boss does not sell higher, a few cheap make people feel can not believe that there is an effect, in general it does work!	JD.com
good friend	Battery Reconditioner	Really nice this thing, no sound, ready to plug in and use, convenient	JD.com
good friend	Battery Reconditioner	Repair the battery good helper, the old phone can also be used, the price is not expensive, than spend thousands of dollars to buy a cell phone is worth it!	JD.com

good friend	Battery Reconditioner	Thought it was aUSBflash drive, but it turned out to be a black technology to repair the battery, it is really nothing strange, I have to say, a little effect	JD.com
good friend	Battery Reconditioner	Apple12battery is not working right, so I came to buy one to test it, looking forward to the result	JD.com
good friend	Battery Reconditioner	Poor can't afford a new phone, stuff tested, should the phone last another2years, hey the mouthpiece issue is hurting me	JD.com
good friend	Battery Reconditioner	continue to use for a period of time, good use in the come to buy	JD.com
good friend	Battery Reconditioner	Estimated test is not an IQ tax, bought2, deliberately used2old cell phone test,1week down, the battery has a little bit of prolonged effect, continue to use see	JD.com
good friend	Battery Reconditioner	Indeed is a good product, can repair effective, can maintain the battery, but also can charge, praise!	JD.com
good friend	Battery Reconditioner	Things are good, the price can be, customer service attitude is very good, very patient to me, praise!	JD.com
good friend	Battery Reconditioner	knowledge, my old man is out of date, this thing is very good, good use	JD.com
good friend	Battery Reconditioner	Never thought, a small thing, actually can help me quite a lot, long useless Samsung tablet, because of battery problems, can not open the machine, connected to this product charging, actually can open the machine, the information can be taken out and used, praise praise praise!	JD.com
good friend	Battery Reconditioner	Looking forward to the effect,5days to arrive, looking quite high-tech,1month later in the evaluation	JD.com
good friend	Battery Reconditioner	How to say, I also think it's quite amazing, just used1week, plugged in2times, Tencent video was watching5hours without electricity, now you can6hours	JD.com
good friend	Battery Reconditioner	I'm a poor player, a Huawei METE20with now do not want to change, the battery is just a little worse, this thing can really help, open a League of Legends10won't shut down, good	JD.com
good friend	Battery Reconditioner	warrant5years, guarantee to do, with a month in the back to follow up	JD.com
good friend	Battery Reconditioner	Things are good, is to receive the goods in Shanghai, a whole week, the overall is still effective, Apple14repair and maintenance up, I hope my14can use a4years	JD.com
good friend	Battery Reconditioner	of5fixes, the battery wasn't heating up, the range was extended, it was very good, bought another one to maintainmy Apple13	JD.com
good friend	Battery Reconditioner	at night when plugged in, the product digital display constantly jumping, the phone will be very stable repair, no other adverse reactions, continue to try to see, later in the follow-up comment!	JD.com
good friend	Battery Reconditioner	Operation is simple, things look high, just used a week, samsungNOTE10fever less often, especially a open king of glory, the game opens faster, not bad dozens of things	JD.com
good friend	Battery Reconditioner	It should not be an illusion, just use1week, the phone did not heat up playing games, and the battery life becomes a little longer, anyway, try, do not use on the return, the boss promises thatwithin1year can be returned	JD.com
good friend	Battery Reconditioner	It's okay,3chicken dropped60%of the electricity, used2weeks, now3chicken dropped40%of the electricity, in the use of a period of time in the follow-up comment	JD.com
good friend	Battery Reconditioner	It's not bad, battery health80%,1month or80%, my small7and live, extend battery life good helper, this repairer is genuine, over2years in the replacement of cell phones	JD.com
good friend	Battery Reconditioner	week, the effect is very good, the battery life really increased a lot, the battery is not like before to play a while on the heat of the hot hand, and now play for a long time will not be hot, the black science and technology products	JD.com
good friend	Battery Reconditioner	really works, I thought there was no saving, my OPPOis now barreling along.one back with the intention of trying it out, followed the tutorial and it really worked, the battery lasted twice as long and last3weeks, really great deal	JD.com
good friend	Battery Reconditioner	Repair is very good, the effect is powerful, the use of the battery is not so fast dead, dozens of dollars can be repaired, more cost-effective than the replacement of the phone, hahaha save a lot of money, is the genuine it!	JD.com
good friend	Battery Reconditioner	Follow the tutorials to do quite simple, the phone and the same before, is to sleep at night when plugged in can be automatically repaired, in fact, buy the time to try to see the mentality, after getting overjoyed than imagined, dozens of dollars to solve the problem, you do not have to thousands of dollars to change a cell phone!	JD.com

good friend	Battery Reconditioner	use of the effect is very obvious, it is indeed a very cost-effective thing to save thousands of dollars to change the phone, the operation is also particularly easy, not for a while on the good recommended!	JD.com
good friend	Battery Reconditioner	using this restorer, now almost can watch fast5hours it, receive calls1day no problem it, this phone is very old, can use so long, really happy. This is a genuine product, I really recommend it!	JD.com
good friend	Battery Reconditioner	the hands of a, indeed the battery with a long time, the phone will not be so stuck, the boss said with90days no effect can be returned, something useful on the return, the times are changing ah, technology in the development of our countryside are an eye-opener	JD.com
good friend	Battery Reconditioner	to change the battery, after repairing the phone with a longer time some, and does not heat up, and also automatically clean up the phone system cache garbage, the phone has a significantly smooth, the boss said that often used to repair will be better.	JD.com
good friend	Battery Reconditioner	AppleXbattery life before a month to drop5percent, with the previous80percent, used4weeks, is still80percent, really awesome, recommend recommended	JD.com
good friend	Battery Reconditioner	High black technology products, the store said it can protect the phone battery by controlling the current, in fact, it is right, many times the voltage is not stable we do not know. Now the cell phone battery with a little bit of the next began to decline in performance, that is, the current is not stable, this try a day is indeed very good.	JD.com
good friend	Battery Reconditioner	battery does not heat up, and is very resistant to electricity, savingmore than100pieces of battery replacement, according to the business said tutorials to do the phone system has become smooth, it seems that the phone can also last2years	JD.com
good friend	Battery Repairer	I got it yesterday. I think it's working.	JD.com
good friend	Battery Repairer	After receiving the goods, used for four days, feel that should be useful, electric car riding for two years, the battery is not durable, full of electricity a power to go down two cells of electricity, now not so fast, with a month and then look at the situation	JD.com
good friend	Battery Repairer	I just got it. I haven't used it yet. It looks good.	JD.com
good friend	Battery Repairer	Received it, just used it not sure how it turned out?	JD.com
good friend	Battery Repairer	Special thanks for the delivery. I'll comment after the charger is used	JD.com
good friend	Battery Repairer	Received the baby and cuttings used, very good and effective. Will be pushing others to buy.	JD.com
good friend	Battery Repairer	The product is beautiful, the service is good, the price is reasonable and the performance is safe.	JD.com
good friend	Battery Repairer	Very good, very practical	JD.com
good friend	Battery Repairer	an excellent	JD.com
good friend	Battery Repairer	tax.	JD.com
good friend	Battery Repairer	It doesn't work. It's all talk and no action.	JD.com
good friend	Battery Repairer	I'll comment after using it.	JD.com
good friend	Battery Repairer	You haven't used it yet? I don't know if it's any good.	JD.com
good friend	Battery Repairer	It works. I've used it for a few days to evaluate it.	JD.com
good friend	Battery Repairer	and powerful at a glance handy[love]to see if full will automatically cut off the power, as well as repair the battery🔋this powerful effect[cool]thatbe perfect nothing to say la! The only thing is that the main plug🔌a little bit can not completely fill the socket of your product, it should not affect it[stay]	JD.com
good friend	Battery Repairer	not bad	JD.com
good friend	Battery Repairer	Let's use it for now, then we'll follow up if it's good	JD.com
good friend	Battery Repairer	is quite convenient to use, used a few times performance is still safe,, the business introduced to have to use a few weeks to have the effect, looking forward to[kudos]	JD.com
good friend	Battery Repairer	Just received the goods are undergoing a trial period, the effect of how to wait for me to try to do evaluation!	JD.com
good friend	Battery Repairer	Very good, charging overnight made a difference in the battery	JD.com
good friend	Battery Repairer	I don't know if it works or not, it's too easy in here, right? I'll try the effect by charging it once at night.	JD.com
good friend	Battery Repairer	Very good Fast charging Fix it for a while and then add more Trustworthy merchant	JD.com
good friend	Battery Repairer	This user feels the product is better	JD.com
good friend	Battery Repairer	The logistics was fast and I am very happy with it.	JD.com
good friend	Battery Repairer	Nice, started using it! Support	JD.com

good friend	Battery Repairer	Loan received, quite good-looking, give a favorable review, to be used in the follow-up comment in the addition	JD.com
good friend	Battery Repairer	Used a few days must come to evaluate, value for money, recommended to a close friend, the	JD.com
good friend	Battery Repairer	fours still not charged	JD.com
good friend	Battery Repairer	Works great.	JD.com
good friend	Battery Repairer	serviceable	JD.com
good friend	Battery Repairer	Received, not yet used do not know how	JD.com
good friend	Battery Repairer	Has received the goods, the packaging is intact, consistent with the description of the merchant, recommended to buy!	JD.com
good friend	Battery Repairer	Fingers crossed for results, sollgive it all5starsfor now!	JD.com
good friend	Battery Repairer	It looks okay! I do not know whether the effect is as good as the business said, can repair the battery Oh!	JD.com
good friend	Battery Repairer	Baby received, get back to use, asked customer service, customer service attitude is very good, a handful of handful of handful of handful of handful of handful of handful of handful of handful of handful. Tea singing is quite suitable. Today rushed up to see the effect tomorrow	JD.com
good friend	Battery Repairer	The one on my cell phone worked noticeably well, this one works and I look forward to being surprised! Easy to use	JD.com
good friend	Battery Repairer	Just used it today, not sure if it works. Use it for a few days and see	JD.com
good friend	Battery Repairer	This user feels the product is better	JD.com
good friend	Battery Repairer	appearance and compact, charging also has the role of repairing the battery.	JD.com
good friend	Battery Repairer	Used it expecting good results now don't feel the car is faster than before	JD.com
good friend	Battery Repairer	It's just as described above and charges well	JD.com
good friend	Battery Repairer	I haven't started using it yet, so I won't comment on it yet, I'll try it in a few days and then come back with pictures	JD.com
good friend	Battery Repairer	Good quality , repair function……	JD.com
good friend	Battery Repairer	Not bad, the battery is not going to go very far, I expect the battery to function properly.	JD.com
good friend	Battery Repairer	results!	JD.com
good friend	Battery Repairer	I haven't tried it yet.	JD.com
good friend	Battery Repairer	This is a scam, what German imported chips, open and see, this is a fraudulent product	JD.com
good friend	Battery Repairer	to say.	JD.com
good friend	Battery Repairer	This user feels the product is better	JD.com
good friend	Battery Repairer	I'll comment after I've used it.	JD.com
good friend	Battery Repairer	Very good quality very good and the boss said almost just received the goods but do not know how effective	JD.com
good friend	Battery Repairer	It's easy to use, and after using it two or three times, I feel a little better than when I first didn't use it	JD.com
good friend	Battery Repairer	Works really well, I've fixed it	JD.com
good friend	Battery Repairer	Goods received, now do not know whether it is good or not, and so use in the evaluation	JD.com
good friend	Battery Repairer	Pretty good, fast delivery and well packed. Wait for a few days to use and then follow up.	JD.com
good friend	Battery Repairer	Goods received, easy to use, fine workmanship, beautiful appearance Thank you boss!	JD.com
good friend	Battery Repairer	high satisfaction, working properly, stable voltage, fast logistics.	JD.com
good friend	Battery Repairer	It looks good, it's easy to use, it's worth buying, I'm very happy with it.	JD.com
good friend	Battery Repairer	Shipped quickly, will evaluate after use.	JD.com
good friend	Battery Repairer	Feels good, rushing to try it today	JD.com
good friend	Battery Repairer	life renewal pulse charger48V60V72Vuniversal received, this I bought a charging recovery is very good, and bought a um in the old home	JD.com
good friend	Battery Repairer	Received first use for a period of time to see, good if the follow-up evaluation	JD.com
good friend	Battery Repairer	Used it once, felt it worked, continue to use it and hope to reach my goal	JD.com
good friend	Battery Repairer	packaged, beautiful appearance, very easy to use, fast charging, thanks to the owner and logistics of the hard service👍	JD.com
good friend	Battery Repairer	I haven't used it yet.	JD.com
good friend	Battery Repairer	(of an unmarried couple) be close	JD.com
good friend	Battery Repairer	two years of lithium batteries, I believe that the damaged part of the repair, to achieve sufficient power, more mileage. To be charged after two weeks to see the effect and then follow up!	JD.com
good friend	Battery Repairer	Works great, full auto power off, also comes with repair	JD.com

good friend	Battery Repairer	Electric car battery repairer activation to extend battery life renewal pulse charger48V60V72Vuniversal received, this battery car repairer, the power is quite large, and convenient, can protect the battery	JD.com
good friend	Battery Repairer	an excellent	JD.com
good friend	Battery Repairer	first charge is okay Looking forward to future results	JD.com
good friend	Battery Repairer	Received the goods! Its looking to fix my battery, will let everyone know if it works.	JD.com
good friend	Battery Repairer	This repair battery device is quite powerful, originally my battery rushed seven hours on the three-grid power, this time rushed to open up a look full of four-grid, purposely one day to open up all the electricity can drive thirty-five kilometers, the original drive twenty-five kilometers, try again or so, this shopping satisfaction. Give five stars	JD.com
good friend	Battery Repairer	Received, opened the carton looked very, compact has not been used, do not know how.	JD.com
good friend	Battery Repairer	Good stuff, used it for a few days before commenting	JD.com
good friend	Battery Repairer	Battery Repairer arrived just used, look good quality, there is no sound, do not understand the place, customer service patience guide, thank you boss!	JD.com
good friend	Battery Repairer	very good	JD.com
good friend	Battery Repairer	It's easy to use, well made, and looks great!	JD.com
good friend	Battery Repairer	Received the pulse charger, it charges fast and works well.	JD.com
good friend	Battery Repairer	Repair the battery back to the door, just charged a back, hope half a month to see the effect, should be able to	JD.com
good friend	Battery Repairer	First time I've used it, it's ok, I hope to use it a few more times to see how well it repairs itself	JD.com
good friend	Battery Repairer	Electric car battery repair device received, well packaged, by using, pulse indicator light is bright, by eight hours of charging, stable work, no heat, normal temperature, according to the instructions and business customer service guidance, to be repaired to improve the effect, must be more positive feedback, and see acquaintances to buy!	JD.com
good friend	Battery Repairer	Charged it as soon as I received it, the sound is smaller, looking forward to fixing it better	JD.com
good friend	Battery Repairer	The goods have been received, good quality, satisfied!	JD.com
good friend	Battery Repairer	It's good. I'll buy it again.	JD.com
good friend	Battery Repairer	Very good auto power off	JD.com
good friend	Battery Repairer	It's easy to use, I just bought it and I don't know how well it works. Customer service promises90packages, I hope so	JD.com
good friend	Battery Repairer	Use it, you can enhance the point is always more affordable than changing the battery.	JD.com
good friend	Battery Repairer	did not expect to charge once to significantly restore to as good as new, great this, before using a day to charge once, now three days a charge are many days! Restoration period three days a charge! Thank you to the merchant for providing a quality product!	JD.com
good friend	Battery Repairer	Haven't tried it yet, don't know the effect	JD.com
good friend	Battery Repairer	It's good. I've used it.	JD.com
good friend	Battery Repairer	Try it on.	JD.com
good friend	Battery Repairer	Received the goods, will comment again after using it	JD.com
good friend	Battery Repairer	I haven't tested it yet.	JD.com
good friend	Battery Repairer	It's easy to use.	JD.com
good friend	Battery Repairer	Genuine, nice. Thanks a lot.	JD.com
good friend	Battery Repairer	Fine workmanship, works fine	JD.com
good friend	Battery Repairer	Second time I've purchased it and it's working great.	JD.com
good friend	Battery Repairer	It works.	JD.com
good friend	Battery Repairer	I got it back and charged it right away to see if it was as advertised. It really does cut off the power automatically	JD.com
good friend	Battery Repairer	Works great, worth the money	JD.com
good friend	Battery Repairer	in use	JD.com
good friend	Battery Repairer	Safety features: very good next time to repurchase	JD.com
good friend	Battery Repairer	Special use a few days to evaluate, the effect can indeed be used a few<br>days, feel the battery strong point, I hope the time is longer have better results<br>, after all, change the battery is too uneconomical	JD.com
good friend	Battery Repairer	Baby is good, I believe it works well, I'll review later	JD.com
good friend	Battery Repairer	It's good, convenient and effective.	JD.com
good friend	Battery Repairer	Hopefully it will work well! It's working! Will follow up with a review![Smiles]	JD.com
good friend	Battery Repairer	received	JD.com
good friend	Battery Repairer	This user did not fill in a rating	JD.com

good friend	Battery Repairer	I'll use it first and see how it goes.	JD.com
good friend	Battery Repairer	stable, or effect, long time use should have effect[cool][cool][cool]	JD.com
good friend	Battery Repairer	Things received, not yet used, do not know the effect of how to use and then come back to the positive feedback	JD.com
good friend	Battery Repairer	I haven't used it yet. I'll comment afterward.	JD.com
good friend	Battery Repairer	I've received the goods and I'll comment in detail when I've used them.	JD.com
good friend	Battery Repairer	performance is not bad logistics is very fast, give five-star praise!	JD.com
good friend	Battery Repairer	I'm trying it out and will comment after I've used it.	JD.com
good friend	Battery Repairer	Buy this product before the battery car fully charged to the unit to run a round trip there is electricity, with this product fully charged to the unit to run a trip are barely, not useful at all, those positive reviews should be brushed single brush out, proper IQ tax!	JD.com
good friend	Battery Repairer	It's useless! Fake as hell!	JD.com
good friend	Battery Repairer	Very good quality.	JD.com
good friend	Battery Repairer	I'll follow up with a review after I've used it, it looks like it's working a bit.	JD.com
good friend	Battery Repairer	It works just as described and works great!	JD.com
good friend	Battery Repairer	It's easy to use,, looks great, and charges quickly!	JD.com
good friend	Battery Repairer	I haven't used it yet. I'll comment after I've used it.	JD.com
good friend	Battery Repairer	evaluated	JD.com
good friend	Battery Repairer	Successful rescue, but will have to keep plugging this in for a few more subsequent charges or the battery won't charge halfway through again	JD.com
good friend	Battery Repairer	Still continuing to use it! Haven't noticed any changes yet! Not sure what will happen if I use it for a while longer!	JD.com
good friend	Battery Repairer	I haven't used it yet.	JD.com
good friend	Battery Repairer	Ha ha ha ha ha ha ha ha ha ha ha ha ha ha ha ha ha ha ha ha ha ha ha ha ha ha ha ha ha ha ha ha ha ha ha ha ha ha ha ha ha	JD.com
good friend	Battery Repairer	acceptable	JD.com
good friend	Battery Repairer	Charging, repairing the battery, received the goods are trying, whether it is useful is still being verified.	JD.com
good friend	Battery Repairer	Poorly restored.	JD.com
good friend	Battery Repairer	days feel the battery run farther a little bit, hope for better results	JD.com
good friend	Battery Repairer	very happy	JD.com
good friend	Battery Repairer	Electric car restorer received, the item is the same as the picture, very satisfied with a shopping	JD.com
good friend	Battery Repairer	I don't know if it's working yet, let's give it some time!	JD.com
good friend	Battery Repairer	It's really quite good, feels like the charge goes farther than it used to jogging	JD.com
good friend	Battery Repairer	The goods have been received in good condition and are ready for use in the next couple of days!	JD.com
good friend	Battery Repairer	Just got it back today and it's in perfect condition. It's charging now, how effective it is, wait for a few days to use it and then comment, in short, thanks to the merchant.	JD.com
good friend	Battery Repairer	I'll comment after using it.	JD.com
good friend	Battery Repairer	It doesn't feel good.	JD.com
good friend	Battery Repairer	I love this, so good[big love][big love][big love][big love]	JD.com
good friend	Battery Repairer	s	JD.com
good friend	Battery Repairer	days, and he says there are noticeable results, which is great.	JD.com
good friend	Battery Repairer	Charging method: just used it, hope it works<br>Charging speed: much faster than before<br>Safety: good	JD.com
good friend	Battery Repairer	Goods received.	JD.com
good friend	Battery Repairer	Good review! Good quality, well made, worth buying.	JD.com
good friend	Battery Repairer	I haven't used it yet and can't comment on it, I'll do so after I use it!	JD.com
good friend	Battery Repairer	This user feels the product is better	JD.com
good friend	Battery Repairer	I just started using it, I don't know if it works yet, I'll add a comment if it does!	JD.com
good friend	Battery Repairer	Bought back to charge a week, did not feel the battery can run farther than one before, feel fooled again, here is one to say one. Now also used if you can as you say, must give a follow-up review, if not repair the battery also follow-up review, I am one to say one.	JD.com
good friend	Battery Repairer	Economical and practical, very good|	JD.com
good friend	Battery Repairer	A satisfactory online purchase	JD.com
good friend	Battery Repairer	No comment for now, after using it.	JD.com

good friend	Battery Repairer	It's okay, I feel that charging with this is quite good, as for the battery effect if you still have to wait with a period of time to know whether to repair the battery.	JD.com
good friend	Battery Repairer	Look at the surface may not be used for four-wheeled electric vehicles.	JD.com
good friend	Battery Repairer	It's ok, I've been using it for a few days now, hopefully the battery will make a difference!	JD.com
good friend	Battery Repairer	Goods have been received. Already used it twice. If it works well and really fixes the battery. I will introduce friends to buy. I've already bookmarked the store.	JD.com
good friend	Battery Repairer	Very good quality, very satisfied with the purchase	JD.com
good friend	Battery Repairer	serviceable	JD.com
good friend	Battery Repairer	working. It's great[Lots of love]	JD.com
good friend	Battery Repairer	review after use	JD.com
good friend	Battery Repairer	Charged once, works great, new ebike, maintain the battery with, hopefully it will last a few more years!!!!	JD.com
good friend	Battery Repairer	favorable criticism	JD.com
good friend	Battery Repairer	The goods have been received, the packaging is intact, the product looks good, praise!	JD.com
good friend	Battery Repairer	The product is beautiful and the quality feels good. It feels like the battery has been repaired. Have to use it again to see then come back to evaluate	JD.com
good friend	Battery Repairer	The stuff can be[big love][smile]and the logistics is fast[big love][big love	JD.com
good friend	Battery Repairer	Baby has received, back home and used it, can be	JD.com
good friend	Battery Repairer	I just bought it and it's working fine.	JD.com
good friend	Battery Repairer	The product has been received has not yet been used, such as used in the evaluation, first give five-star praise!	JD.com
good friend	Battery Repairer	Logistics is very fast, the boss has a good service attitude, next time will buy back!	JD.com
good friend	Battery Repairer	Electric car repairer received, the packaging is intact, logistics is very fast, the price is not expensive, tried the charger repair normal, I do not know how the effect, always better than changing a battery, look forward to good results!	JD.com
good friend	Battery Repairer	advertised, has been used, the effect to be used for a period of time to say.	JD.com
good friend	Battery Repairer	I don't know if it's any good, I'll comment after using it.	JD.com
good friend	Battery Repairer	It's still possible.	JD.com
good friend	Battery Repairer	Merchants and logistics service attitude is very good, first give a five-star.	JD.com
good friend	Battery Repairer	I feel fine.	JD.com
good friend	Battery Repairer	I got the baby. It's good. It's good.	JD.com
good friend	Battery Repairer	Peace of mind with this electric car, great customer service and trustworthy.	JD.com
good friend	Battery Repairer	It is being used. Hope it works.	JD.com
good friend	Battery Repairer	an excellent	JD.com
good friend	Battery Repairer	The goods have been received, the quality is good, and the logistics is very fast.	JD.com
good friend	Battery Repairer	Haven't used it yet.	JD.com
good friend	Battery Repairer	I'm happy with it.	JD.com
good friend	Battery Repairer	after using it once I felt it did work. I was afraid that the charge outlet wouldn't work, but I was overly concerned and it all worked.	JD.com
good friend	Battery Repairer	pretty good	JD.com
good friend	Battery Repairer	I used the item when I received it and I think it works well.	JD.com
good friend	Battery Repairer	The quality of this product is really good, and it is really strong for battery repairing.	JD.com
good friend	Battery Repairer	Goods received, the courier is also fast, business service is very good, to be used and then make additional comments.	JD.com
good friend	Battery Repairer	Goods can be logistics quickly got back immediately installed and used	JD.com
good friend	Battery Repairer	buy!	JD.com
good friend	Battery Repairer	This user feels the product is better	JD.com
good friend	Battery Repairer	Fine workmanship, beautiful appearance, easy to use, the main thing is still useful, it does have an effect on the battery for a long time	JD.com
good friend	Battery Repairer	It's easy to use, well made and looks great! Inexpensive and beautiful!	JD.com
good friend	Battery Repairer	Not yet. Looks okay.	JD.com
good friend	Battery Repairer	It works.	JD.com
good friend	Battery Repairer	Send it to you later, too busy right now. I have used it last night and it is very stable	JD.com
good friend	Battery Repairer	It's good. I'm using it.	JD.com
good friend	Battery Repairer	I haven't used it yet, so I'll follow up later when I do.	JD.com

good friend	Battery Repairer	Used it today and it feels good	JD.com
good friend	Battery Repairer	Battery, restorer received, immediately use, do not know not can not, first give a five-star evaluation, such as repair and then chase level.	JD.com
good friend	Battery Repairer	It's easy to use.	JD.com
good friend	Battery Repairer	Why isn't it energized?	JD.com
good friend	Battery Repairer	Things have been received, look quite good, well-packaged fast question is also quite fast	JD.com
good friend	Battery Repairer	Appearance is beautiful Repair battery effect is very good Logistics is also very fast 5 stars praise	JD.com
good friend	Battery Repairer	The repairer works great, automatically cuts off the power, so you don't have to worry about charging up the battery anymore!	JD.com
good friend	Battery Repairer	I don't know how it works, I'm used to 5 stars	JD.com
good friend	Battery Repairer	The goods have been received thank you boss! I'll buy it again next time.	JD.com
good friend	Battery Repairer	Haven't used it yet, don't understand how it works. Is it connected to the punch when you charge it?	JD.com
good friend	Battery Repairer	I've received the goods and tried them out.	JD.com
good friend	Battery Repairer	I'll tell you when I use it.	JD.com
good friend	Battery Repairer	Repairer received, the box is intact, Offer factory replicator small and exquisite, easy to use, very satisfied, praise!	JD.com
good friend	Battery Repairer	Received the goods type used is not bad to the live said the same quality is very good price each affordable logistics very quickly good value for money is very satisfied with the purchase of straight old iron assured that the purchase of wishing the boss a prosperous business next time to buy again!	JD.com
good friend	Battery Repairer	Pro, the loan received, the packaging is intact, the logistics is very fast, the boss service attitude is also quite good, it is an ideal shopping, thanks boss of the good products, I wish you prosperous business[][][[[点赞][点赞][点赞][点赞][点赞][点赞][点赞][点赞][点赞]	JD.com
good friend	Battery Repairer	evaluation	JD.com
good friend	Battery Repairer	Goods have been received, small and beautiful, have not yet used, after the use of comment, first give five-star praise!	JD.com
good friend	Battery Repairer	This user feels the product is better	JD.com
good friend	Battery Repairer	five-star review	JD.com
good friend	Battery Repairer	Baby received the first five stars, wait a few days to try to comment.	JD.com
good friend	Battery Repairer	Signed yesterday, electric car repairer, last night began to use, with a period of time with the effect, must praise, customer service also promises to repair the repairer 5-year warrantfor a new one, 90days to return	JD.com
good friend	Battery Repairer	Inexpensive and as advertised.	JD.com
good friend	Battery Repairer	Just like the picture, very satisfied	JD.com
good friend	Battery Repairer	I've just started using it again. It looks fine.	JD.com
good friend	Battery Repairer	This stuff can make the battery as good as new, don't need to change the battery every year	JD.com
good friend	Battery Repairer	The ding-sheet was received quickly, feels good and is being used satisfactorily!	JD.com
good friend	Battery Repairer	A very good purchase	JD.com
good friend	Battery Repairer	Easy to use and effective.	JD.com
good friend	Battery Repairer	This user feels the product is better	JD.com
good friend	Battery Repairer	This user feels the product is better	JD.com
good friend	Battery Repairer	fast logistics	JD.com
good friend	Battery Repairer	Just used it, it's okay, I can't see the effect yet.	JD.com
good friend	Battery Repairer	Item is great, right on the description. Merchant service was also excellent and will be back for the next purchase!	JD.com
good friend	Battery Repairer	This user feels the product is better	JD.com
good friend	Battery Repairer	review	JD.com
good friend	Battery Repairer	Not used yet, can't comment so far, wait to use it for a while	JD.com
good friend	Battery Repairer	Better than the last one I bought.	JD.com
good friend	Battery Repairer	received	JD.com
good friend	Battery Repairer	The logistics is fast and the quality is good.	JD.com
good friend	Battery Repairer	Baby has received, very good, very good! Excellent quality and practical, great, great, really great! 👍💐😀😀	JD.com
good friend	Battery Repairer	is perfect and the quality is reliable. It is a very good product! The courier worked hard! Good comment, give five stars!	JD.com
good friend	Battery Repairer	Easy to use,, good response	JD.com
good friend	Battery Repairer	Charging speed: pretty fast.<br>Safety features: also very good.	JD.com

good friend	Battery Repairer	The weather is getting cooler, the electric car more and more run less miles, with this battery repair artifact I do not have to worry about the	JD.com
good friend	Battery Repairer	hope it will help the battery. Just in case, be responsible for yourself.	JD.com
good friend	Battery Repairer	Received and working properly. Love it. Positive brand to order with confidence. Good service! Logistics fast!	JD.com
good friend	Battery Repairer	I received the goods, the workmanship is very rough, I do not know the shelf life of ï¿½ï¿½ï¿½ï¿½ï¿½ï¿½ï¿½ï¿½ï¿½ï¿½.	JD.com
good friend	Battery Repairer	Just received.	JD.com
good friend	Battery Repairer	Good to use a few days also charged a few times obviously improved, continue to use later in the evaluation.	JD.com
good friend	Battery Repairer	I received the restorer, I don't know how it will work, so I hope it works well.	JD.com
good friend	Battery Repairer	I haven't tried it yet.	JD.com
good friend	Battery Repairer	Small east, received, well packaged, satisfied, looking forward to good results, five-star praise!	JD.com
good friend	Battery Repairer	favorable criticism	JD.com
good friend	Battery Repairer	The restorer has been received and is being used, hopefully with good returns.	JD.com
good friend	Battery Repairer	I haven't used it yet, I'll comment afterward	JD.com
good friend	Battery Repairer	I just received it and haven't used it yet, hope it works well	JD.com
good friend	Battery Repairer	New products should be used to see how they work.	JD.com
good friend	Battery Repairer	Courier boy thanks a lot bro.[Kudos][Rose]	JD.com
good friend	Battery Repairer	This user feels the product is better	JD.com
good friend	Battery Repairer	It feels okay. It works.	JD.com
good friend	Battery Repairer	This user feels the product is better	JD.com
good friend	Battery Repairer	It's good. It works.	JD.com
good friend	Battery Repairer	Logistics is fast, the product is intact is very satisfied.	JD.com
good friend	Battery Repairer	Automatic trolley repairer received, plugged in to try, feel the effect is quite good, observe on a few days, the effect is obvious, then continue to follow up!	JD.com
good friend	Battery Repairer	Pro, the loan received, the packaging is intact, the logistics is very fast, the boss service attitude is also quite good, it is an ideal shopping, thank😜boss of the good products, I wish you a prosperous business💰💰💰💰💰💯👍	JD.com
good friend	Battery Repairer	It's kinda nice and charges fast. Automatically powers off when full.	JD.com
good friend	Battery Repairer	Picked up the goods and used it, looking forward to the effect, customer service reply promptly, praise.	JD.com
good friend	Battery Repairer	good!	JD.com
good friend	Battery Repairer	door	JD.com
good friend	Battery Repairer	use?	JD.com
good friend	Battery Repairer	This user feels the product is better	JD.com
good friend	Battery Repairer	Good workmanship! Looking forward to the results! 5 out of 5!	JD.com
good friend	Battery Repairer	month really repair success, before the electric car can only run20kilometers, after a month of use yesterday to run a round trip to Shenzhen27kilometers of road only half of the electricity, last night to help the dance friends under a single, hope that his also used, I wish the boss a prosperous business	JD.com
good friend	Battery Repairer	buying!	JD.com
good friend	Battery Repairer	Electric car battery restorer rushed twice the electricity has effect, more than a few times will be better, have an electric bike Mingyou, this restorer is really good, worth buying......	JD.com
good friend	Battery Repairer	Received the product and haven't used it yet! I don't know if the quality assurance works?	JD.com
good friend	Battery Repairer	I've been using it since I got it. It's good. It's good.	JD.com
good friend	Battery Repairer	use it.	JD.com
good friend	Battery Repairer	It's good. It's worth it.	JD.com
good friend	Battery Repairer	The description matches, the logistics are fast, the price is low, and the quality is guaranteed.	JD.com
good friend	Battery Repairer	Fast shipping, well packaged. Have not yet used it, do not know how the quality is, later follow-up evaluation.	JD.com
good friend	Battery Repairer	Charging method: convenient andfast!Charging speed: fast Safety: exceptionally good	JD.com
good friend	Battery Repairer	Goods just received, and then try, use to know the good or bad, after a period of time to say!	JD.com
good friend	Battery Repairer	I haven't used it yet.	JD.com
good friend	Battery Repairer	Repair of the battery arrived, customer service attentive to solve the problem, for the service attitude praise, repair effect later comment[smile]	JD.com

good friend	Battery Repairer	For safety reasons, charging the electric car do not let it catch fire, fully charged automatically will trip the battery, this can make the battery can be repaired, can let him time to live a little longer?	JD.com
good friend	Battery Repairer	It's good. I'll comment when I've used it.	JD.com
good friend	Battery Repairer	The Battery Repairer works.Safe and effective.Effective.Logistics quickly.Good service	JD.com
good friend	Battery Repairer	I just received the item and will evaluate it after charging it a bit.	JD.com
good friend	Battery Repairer	It works.	JD.com
good friend	Battery Repairer	This user feels the product is better	JD.com
good friend	Battery Repairer	Very good, your goods this is very good, can protect the battery car, thank you for your goods, thank you!	JD.com
good friend	Battery Repairer	I can't tell you how effective it is yet, I'll comment later.	JD.com
good friend	Battery Repairer	Boss in? Things received, get back at night to insert the battery, an electric original switch, the red light flashes a flash to start working, very its to be used after the effect.	JD.com
good friend	Battery Repairer	Goods have been received, have not yet used, for the time being, do not evaluate	JD.com
good friend	Battery Repairer	I thought it was wired, but it's not.	JD.com
good friend	Battery Repairer	Just arrived, don't know if it's good or not, I'll evaluate it after using it	JD.com
good friend	Battery Repairer	Looks great, easy to use customer service is spot on and recommended! Five stars praise!	JD.com
good friend	Battery Repairer	Best quality, good home delivery service, fast shipping satisfaction is very high!	JD.com
good friend	Battery Repairer	I haven't used it yet, I'll tell you what I think after I've tried it	JD.com
good friend	Battery Repairer	It should be fine. It's worth it.	JD.com
good friend	Battery Repairer	Great stuff, very happy with it.	JD.com
good friend	Battery Repairer	Good very good I love it!	JD.com
good friend	Battery Repairer	The goods have been received thanks! Packaging is good quality is very good! But not yet used to be followed up later.	JD.com
good friend	Battery Repairer	Goods have been received, the logistics service is very good to try and then evaluate the results	JD.com
good friend	Battery Repairer	I haven't used it yet. I'll buy it again if it's good.	JD.com
good friend	Battery Repairer	Specially used a few days to evaluate, the effect can indeed be used for a few days, the feeling of the battery is stronger, I hope that a longer time has a better effect, after all, change the battery is too uneconomical!	JD.com
good friend	Battery Repairer	Received the goods, trying it out	JD.com
good friend	Battery Repairer	very happy	JD.com
good friend	Battery Repairer	It works. It's good. It's good.	JD.com
good friend	Battery Repairer	Received and looking forward to good results	JD.com
good friend	Battery Repairer	Logistics is very fast, after receiving the goods with the same as advertised, after receiving and using the quality is very good, you are welcome to buy a use!	JD.com
good friend	Battery Repairer	This user feels the product is better	JD.com
good friend	Battery Repairer	Baby has received the goods, very practical. Logistics is also very fast.	JD.com
good friend	Battery Repairer	Logistics is fast! It looks great, so I'll give it a favorable review!	JD.com
good friend	Battery Repairer	serviceable	JD.com
good friend	Battery Repairer	an excellent	JD.com
good friend	Battery Repairer	Good!	JD.com
good friend	Battery Repairer	love it!	JD.com
good friend	Battery Repairer	not very useful	JD.com
good friend	Battery Repairer	I'll give it 5 stars for now, I'll comment after I use it	JD.com
good friend	Battery Repairer	Nice and dingy.	JD.com
good friend	Battery Repairer	In the charging logistics is also very fast to save the purchase of batteries	JD.com
good friend	Battery Repairer	Very satisfied, good quality and beautiful!	JD.com
good friend	Battery Repairer	Fast logistics, business description carefully. The product is small and exquisite, automatic battery car repairer, to be tried and then follow up the effect.	JD.com
good friend	Battery Repairer	Fast delivery looks good, hope the restoration is good.	JD.com
good friend	Battery Repairer	'Just received the goods on the chase as soon as possible to plug in the electric car.soon as I pressed the power switch, the red pulse light flashed and started to work, and I was looking forward to the effect after I made it	JD.com
good friend	Battery Repairer	I got it. Thank you.	JD.com
good friend	Battery Repairer	Smooth and fast charging, good safety features.	JD.com
good friend	Battery Repairer	Just got it. Hope it comes in handy.	JD.com

good friend	Battery Repairer	Fast logistics, good quality	JD.com
good friend	Battery Repairer	durable.	JD.com
good friend	Battery Repairer	Very good👍	JD.com
good friend	Battery Repairer	Received today! Packaging is intact! Not yet used! Hopefully with the merchant description of the same1good use to push the expansion of friends!	JD.com
good friend	Battery Repairer	tried.	JD.com
good friend	Battery Repairer	I have only used it for the past two days, and it looks okay for the time being, so I will wait for three to five weeks to see the actual results.	JD.com
good friend	Battery Repairer	Looks good, hope it works, thanks!	JD.com
good friend	Battery Repairer	Use it for a while and then comment	JD.com
good friend	Battery Repairer	Good to use, quite good, good logistics service	JD.com
good friend	Battery Repairer	Received the goods, looks pretty good, wait for me to use once and then follow up reviews	JD.com
good friend	Battery Repairer	satisfaction	JD.com
good friend	Battery Repairer	It's okay, waiting for results!	JD.com
good friend	Battery Repairer	I'll give you a good review first, and then I'll follow up after I've used it well	JD.com
good friend	Battery Repairer	ability	JD.com
good friend	Battery Repairer	The product is really good	JD.com
good friend	Battery Repairer	Product appearance is beautiful and generous[skimming], fine workmanship is okay, use and then evaluate in detail	JD.com
good friend	Battery Repairer	Not bad.	JD.com
good friend	Battery Repairer	Repurchase item, nice one.[kudos][kudos][kudos]	JD.com
good friend	Battery Repairer	Looking at this it should be in good shape, love it.	JD.com
good friend	Battery Repairer	evaluation	JD.com
good friend	Battery Repairer	The boss shipped fast, it's pretty good	JD.com
good friend	Battery Repairer	I received the baby a long time ago and have used it, it seems to be more powerful.	JD.com
good friend	Battery Repairer	Goods goods have been received very well packaged well logistics very quickly thanks to the courier company.	JD.com
good friend	Battery Repairer	pretty good	JD.com
good friend	Battery Repairer	Just received it, haven't used it yet, hope it works!	JD.com
good friend	Battery Repairer	Electric car full of electricity to run the road obviously less mileage, the battery with almost two years, online to see the store commitment, hold the heart to try, I believe it can have an effect, just received this product today, after using in the follow-up evaluation!	JD.com
good friend	Battery Repairer	Used to evaluate the later, the quality is very good, effective, we can rest assured that the order	JD.com
good friend	Battery Repairer	it.	JD.com
good friend	Battery Repairer	Not bad.	JD.com
good friend	Battery Repairer	Arrived quickly, well packaged, easy and quick to use	JD.com
good friend	Battery Repairer	It was received in perfect condition and is recommended,,...	JD.com
good friend	Battery Repairer	introduced to use the effect is very good. Customer service attitude is very good. A very satisfactory online shopping. Next time will buy back.	JD.com
good friend	Battery Repairer	Electric Smart Outlet Repairer Plug and Charge Good Efficiency Good Quality	JD.com
good friend	Battery Repairer	Goods received the effect of how to use the good if you come back to the order!	JD.com
good friend	Battery Repairer	Hello:Baby received, the quality is very good logistics quickly satisfied to give you a five-star praise!	JD.com
good friend	Battery Repairer	Safety: Satisfactory	JD.com
good friend	Battery Repairer	Not yet used, evaluation after use	JD.com
good friend	Battery Repairer	This product is very good genuine worth buying!	JD.com
good friend	Battery Repairer	pretty good	JD.com
good friend	Battery Repairer	The quality is very over the top, quality is guaranteed, quite a good choice, a lot of friends see me use all want to buy, I have recommended.	JD.com
good friend	Battery Repairer	You won't know until you use it.	JD.com
good friend	Battery Repairer	Charging is fast and economical, and the product works well and satisfactorily	JD.com
good friend	Battery Repairer	It's easy to use, genuine, effective and guaranteed!	JD.com
good friend	Battery Repairer	received, the appearance is very beautiful, with characteristics, and charging repair effect is very good, good quality, and have a warranty, thank you for the business service, praise praise praise!	JD.com
good friend	Battery Repairer	A few years old batteries with the product reconditioning really have a very good improvement, really good!	JD.com
good friend	Battery Repairer	convenient, the key is that the effect is very good, it is really worth buying!	JD.com

good friend	Battery Repairer	Electric car repairer received in the afternoon, well-packed, open the package immediately began to use, looks good quality, and charging effect is also good, repair should not be bad, useful will buy again to send someone!	JD.com
good friend	Battery Repairer	features!	JD.com
good friend	Battery Repairer	Very good quality, very satisfied with the service and answered all questions	JD.com
good friend	Battery Repairer	A battery is a few hundred dollars, with the electric car restorer can save a lot, especially affordable and worth recommending the use of people around!	JD.com
good friend	Battery Repairer	product!	JD.com
good friend	Battery Repairer	repurchase discount, recommended to relatives have bought, all praise good!	JD.com
good friend	Battery Repairer	I filled in the wrong address, customer service patience to deal with, quickly received, the appearance is very good, electric car repair god is really very good!	JD.com
good friend	Battery Repairer	Neighbor recommended to buy, used for a long time, the battery is basically the same as a new battery, truly recommended!	JD.com
good friend	Battery Repairer	The service is very good, the customer service answer is also very detailed, according to customer service said the use of a period of time after the effect is really much better!	JD.com
good friend	Battery Repairer	Used more than half a year, because they accidentally lost, with the business actually gave me a lot of discounts, and then bought a, really thank the business service after sale!	JD.com
good friend	Battery Repairer	Thank you for the business and logistics services, after-sales very good and guaranteed, the product is really good!	JD.com
good friend	Battery Repairer	Battery Repairer received, logistics is very fast, the quality is also very good, used a few days, it is indeed very conscientious, very recommended!	JD.com
good friend	Battery Repairer	Exquisite workmanship, good quality, the effect is very good, the key is affordable, the purchase is well worth it!	JD.com
good friend	Battery Repairer	And can charge and can repair the battery, it is indeed very convenient, the follow-up to see how the effect, customer service guarantee said very heartwarming!	JD.com
good friend	Battery Repairer	Received the goods, electric car repairer appearance is very beautiful, logistics is also very fast	JD.com
good friend	Battery Repairer	This multi-functional battery repair device effect is really good, used for a while obviously a lot of battery durability!	JD.com
good friend	Battery Repairer	Charging function: very good. Product appearance: beautiful and generous. Logistics speed: very fast not dragging	JD.com
good friend	Battery Repairer	Just received the use of the feeling of a slight enhancement, the product design is still quite simple, very easy to operate	JD.com
good friend	Battery Repairer	Electric car battery repairer received a very convenient and quick to use the effect is very satisfied with the battery before you can open four hours now can be used for six hours very surprised very good-looking!	JD.com
good friend	Battery Repairer	Things received product appearance cool multi-device compatible, especially after powering up is very beautiful description consistent quality, the use of simple effect is good, the battery repair is improved, it is worth having, I wish the boss a prosperous business!	JD.com
good friend	Battery Repairer	fine workmanship; store service patience and meticulous, ask a hundred questions; express delivery quickly; courier is serious and responsible; packaging is simple and strict, the goods are intact.	JD.com
good friend	Battery Repairer	Shipping is very fast, the baby received after the trial, indeed can effectively repair the old battery, with a long time, it is worth buying!	JD.com
good friend	Battery Repairer	Battery repair is very good, before full can not use for a long time, now a full charge can be used for a long time!	JD.com
good friend	Battery Repairer	used for a period of time, the effect is good and simple and convenient!	JD.com
good friend	Battery Repairer	Received, thanks to the boss, the effect of using a few days immediately a lot better, really good Oh!	JD.com
good friend	Battery Repairer	I used it a few times for a friend and asked me to buy it for him, and now I've bought it for so many people, it's a really good product!	JD.com
good friend	Battery Repairer	drive it for a day and a half after I use the restorer, it's really good!	JD.com
good friend	Battery Repairer	The packaging is intact, the product was received and started to use, the charging effect is also very good, there is a slight boost, customer service said to use for a while longer, but also guaranteed!	JD.com
good friend	Battery Repairer	After using the repairer the battery lasts a lot longer, good and recommendable!	JD.com
good friend	Battery Repairer	The effect is really good, the logistics is also very fast, a few days to arrive	JD.com
good friend	Battery Repairer	Sold out.	JD.com

Henri New Energy Restoration	Battery Reconditioner	Received it and have not used it yet, looking forward to the effect, use it and then evaluate.	JD.com
Henri New Energy Restoration	Battery Reconditioner	Customer service does not respond to messages after ordering	JD.com
Henri New Energy Restoration	Battery Reconditioner	Nice product, very happy with it, highly recommended	JD.com
Henri New Energy Restoration	Battery Reconditioner	some of use	JD.com
Henri New Energy Restoration	Battery Reconditioner	Just do activities, the price is much cheaper, cost-effective, really special value for money!	JD.com
Henri New Energy Restoration	Battery Reconditioner	Good stuff!	JD.com
Henri New Energy Restoration	Battery Reconditioner	This user feels the product is better	JD.com
Henri New Energy Restoration Technology	Battery Reconditioner	used it once It seems to really work The power drop is not so fast , thanks for the good product I've been a little worried about not using it[smile] Recommended to buy, use it for a period of time and then follow up	JD.com
Henri New Energy Restoration	Battery Reconditioner	A little disappointment.	JD.com
Henri New Energy Restoration	Battery Reconditioner	Received the item ordered. Looks basically as advertised. The quality of workmanship is average.	JD.com
Henri New Energy Restoration	Battery Reconditioner	Look at this. It's very high-tech.	JD.com
Henri New Energy Restoration	Battery Reconditioner	It's good. It works.	JD.com
Henri New Energy Restoration	Battery Reconditioner	Functional features: no exterior design: can	JD.com
Henri New Energy Restoration	Battery Reconditioner	It doesn't work at all. It's a lie.	JD.com
Henri New Energy Restoration	Battery Reconditioner	The item received is not the same as the picture, and there is no actual repair function	JD.com
Henri New Energy Restoration	Battery Reconditioner	The restorer has been received and is being used to finally see how well it works before following up.	JD.com
Henri New Energy Restoration	Battery Reconditioner	Unused products, use]to know the effect	JD.com
Henri New Energy Restoration	Battery Reconditioner	Fooled Products	JD.com
Henri New Energy Restoration	Battery Reconditioner	Fine workmanship, but the effect is yet to be known after use	JD.com
Henri New Energy Restoration	Battery Reconditioner	I haven't used it yet. I'll comment afterward.	JD.com
Henri New Energy Restoration	Battery Reconditioner	Just arrived, will comment after trying it out	JD.com
Henri New Energy Restoration	Battery Reconditioner	Use it and evaluate.	JD.com
Henri New Energy Restoration	Battery Reconditioner	It's at home.	JD.com
Henri New Energy Restoration	Battery Reconditioner	Very good. The measurements are good.	JD.com
Henri New Energy Restoration	Battery Reconditioner	It's good. I'll comment after I use it.	JD.com
Henri New Energy Restoration	Battery Reconditioner	Beautiful and practical	JD.com

Henri New Energy Restoration	Battery Reconditioner	Express received, phone charging with lights to show math, phone charging fast	JD.com
Henri New Energy Restoration	Battery Reconditioner	Received the product used, the effect is really good, good worth repurchase again!	JD.com
Henri New Energy Restoration	Battery Reconditioner	The goods received, small and exquisite, logistics is also very fast,,, packaging is very good.	JD.com
Henri New Energy Restoration	Battery Reconditioner	I don't know how to fix a cell phone? There are numbers on the go!	JD.com
Henri New Energy Restoration	Battery Reconditioner	This user feels the product is better	JD.com
Henri New Energy Restoration Technology	Battery Reconditioner	also particularly fast, while the phone does not heat up, a lot better than the products I purchased elsewhere, the shopkeeper's service is enthusiastic and thoughtful and meticulous, trustworthy products and businesses. Next time will continue to cooperate.	JD.com
Henri New Energy Restoration	Battery Reconditioner	Cell phone repair battery capacity rechargeable stability function is powerful, full of automatic power-off	JD.com
Henri New Energy Restoration	Battery Reconditioner	Great style, good quality, awesome!	JD.com
Henri New Energy Restoration	Battery Reconditioner	Very good, received the goods on the use, than I previously bought a lot stronger, recommended to buy Oh!	JD.com
Henri New Energy Restoration	Battery Reconditioner	Compact and easy to use, yet portable	JD.com
Henri New Energy Restoration	Battery Reconditioner	Deeply cleans your phone's memory junk, improves your phone's speed, and increases your phone's battery life.	JD.com
Henri New Energy Restoration	Battery Reconditioner	I love this product and it works great!	JD.com
Henri New Energy Restoration	Battery Reconditioner	The goods have been received I love it next time I will buy from you again.	JD.com
Henri New Energy Restoration	Battery Reconditioner	Repairer has received, very delicate, the phone is good again, worth buying!	JD.com
Henri New Energy Restoration	Battery Reconditioner	Baby has been received and is charging, will comment after use. First5out5for fast shipping, etc.	JD.com
Henri New Energy Restoration	Battery Reconditioner	It's in the process of being fixed, but hopefully with good results.	JD.com
Henri New Energy Restoration	Battery Reconditioner	Very good, bought with the intention of trying, received the quality is very good, worth buying!	JD.com
Henri New Energy Restoration	Battery Reconditioner	The effect is awesome very very much like it	JD.com
Henri New Energy Restoration	Battery Reconditioner	Baby arrived really good. The price/performance ratio is superb and the logistics are great!	JD.com
Henri New Energy Restoration	Battery Reconditioner	Received the goods, tried to charge very quickly, the quality is also very good, very satisfied with the next will continue to buy!	JD.com
Henri New Energy Restoration	Battery Reconditioner	Fast shipping, good quality, good value	JD.com
Henri New Energy Restoration	Battery Reconditioner	Old cell phone, after restoration, standby for a day, and more than sixty power, rare good product, worth buying	JD.com
Henri New Energy Restoration	Battery Reconditioner	The quality of the goods is very good, the charging display is being repaired, used a few days the effect is good looking forward to better later.	JD.com
Henri New Energy Restoration	Battery Reconditioner	The data cable is beautiful and delicate, three charging heads can meet different needs, charging is also fast fantastic!	JD.com
Henri New Energy Restoration	Battery Reconditioner	Charged, has been changing the number, very technological sense, received on the home phone with, now adhere to the time does last longer than before, very good products	JD.com

Henri New Energy Restoration	Battery Reconditioner	It’s the real deal. It works pretty well.		JD.com
Henri New Energy Restoration	Battery Reconditioner	It’s very good, the battery is much more durable after restoration, and the price is affordable, satisfied.		JD.com
Henri New Energy Restoration	Battery Reconditioner	Nice and compact, easy to carry on the go!		JD.com
Henri New Energy Restoration	Battery Reconditioner	You can. Fix it. The phone isn’t lagging anymore.		JD.com
Henri New Energy Restoration	Battery Reconditioner	Fast shipping, cost effective, good quality for battery repair, good quality.		JD.com
Henri New Energy Restoration	Battery Reconditioner	A cell phone that’s been broken for over a year plugged in and turned on in half an hour. Amazing.		JD.com
Henri New Energy Restoration	Battery Reconditioner	Baby received, thank you boss, the quality is very good, very happy with a shopping next time will come back to patronize!		JD.com
Henri New Energy Restoration	Battery Reconditioner	The price is not expensive. Just what was needed, I will be watching your house and will come back if needed.		JD.com
Henri New Energy Restoration	Battery Reconditioner	Baby has been received. Worth having. The 3 in 1 works great. Buy with confidence if you need it		JD.com
Henri New Energy Restoration	Battery Reconditioner	The product is of good quality, easy to use, to be seen the results.		JD.com
Henri New Energy Restoration	Battery Reconditioner	Have not used it and will comment in the future when I do.		JD.com
Henri New Energy Restoration	Battery Reconditioner	I’ll tell you later.		JD.com
Henri New Energy Restoration	Battery Reconditioner	Got the stuff, pretty good quality just arrived and tried it out. It works well.		JD.com
Henri New Energy Restoration	Battery Reconditioner	Baby received, logistics super fast! Baby appearance is also good, try a few days to follow up, good if recommended to friends.		JD.com
Henri New Energy Restoration	Battery Reconditioner	Display voltage and current when charging, it is dynamic, the current after charging0		JD.com
Henri New Energy Restoration	Battery Reconditioner	The quality is okay. A little effect. Give the merchant the same description. Mainly cheap. Worth buying if needed. Customer service attitude is very good. Give full stars.		JD.com
Henri New Energy Restoration	Battery Reconditioner	I just used it. It’s not bad.		JD.com
Henri New Energy Restoration Technology	Battery Reconditioner	The product has been received, the shape is cool, charging can always monitor the charging voltage, current, according to the instructions, there is a clear effect of repairing the battery, the logistics speed is also very fast, 2days to arrive		JD.com
Henri New Energy Restoration	Battery Reconditioner	Very good use, logistics is also very fast, very practical, satisfactory		JD.com
Henri New Energy Restoration Technology	Battery Reconditioner	Charging a few times, my cell phone battery, play the same time, feel the cell phone battery standby time is much longer than before, can play the role of repairing the cell phone battery, is a good product.		JD.com
Henri New Energy Restoration	Battery Reconditioner	Cell phone battery repairer, beautiful appearance. It feels good to use, five points praise.		JD.com
Henri New Energy Restoration	Battery Reconditioner	Works pretty well, good repair, fast shipping, and not too expensive, Nes		JD.com
Henri New Energy Restoration	Battery Reconditioner	The old phone that was previously shutting down while charging seems to have improved a bit, so I can play for relatively long periods of time, so I hope to build on that!		JD.com
Henri New Energy Restoration	Battery Reconditioner	Charging is pretty fast, and the store ships quickly, so that’s good~~		JD.com
Henri New Energy Restoration	Battery Reconditioner	The quality is good and the color is nice and the charging is relatively fast.		JD.com

Henri New Energy Restoration	Battery Reconditioner	Fast charging, silicone, soft and unbreakable	JD.com
Henri New Energy Restoration	Battery Reconditioner	Not yet used, first praise	JD.com
Henri New Energy Restoration	Battery Reconditioner	The stuff is here, I'll try it out and I'll come back with additional comments in a few days	JD.com
Henri New Energy Restoration Technology	Battery Reconditioner	time I felt there was a problem. After communicating with the store, it was realized that the product itself structure. Try again, can be used now use the effect is good, use longer than before!	JD.com
Henri New Energy Restoration	Battery Reconditioner	Fast shipping, fast charging, not too expensive, happy with it	JD.com
Henri New Energy Restoration	Battery Reconditioner	Nice, works pretty good, pretty good, fast shipping	JD.com
Henri New Energy Restoration	Battery Reconditioner	Baby received, logistics is very fast, inexpensive and beautiful, worth having!	JD.com
Henri New Energy Restoration	Battery Reconditioner	Great material, good quality and arrived quickly!	JD.com
Henri New Energy Restoration	Battery Reconditioner	The battery adapter works, the battery now lasts longer than before and playing games is much smoother!	JD.com
Henri New Energy Restoration	Battery Reconditioner	I can't believe there's this kind of artifact, it's so advanced, take out all my cell phones and fix them again	JD.com
Henri New Energy Restoration	Battery Reconditioner	As described by the store, fast shipping good quality, affordable price very good use	JD.com
Henri New Energy Restoration	Battery Reconditioner	Great quality. Amazing baby.	JD.com
Henri New Energy Restoration Technology	Battery Reconditioner	evaluate, the effect is still there, one of the cell phone original charging charge can not be in, with this now can be charged very quickly, there is a cell phone must have been plugged in order to use, not plugged in the power will automatically shut down, repair a few times, not plugged in can be used for a period of time, the effect is good, recommended the purchase of the German imported chip! This, indeed good	JD.com
Henri New Energy Restoration	Battery Reconditioner	Recommended by a friend, good quality at a good price, very satisfied	JD.com
Henri New Energy Restoration	Battery Reconditioner	The goods have been received, the logistics is very fast, has not been used, later review, first give five stars!	JD.com
Henri New Energy Restoration	Battery Reconditioner	Very good quality, satisfied with a shopping	JD.com
Henri New Energy Restoration Technology	Battery Reconditioner	The quality of this product is very good! The original cell phone battery power consumption is quite powerful, almost can not store electricity, yesterday used the product only once, power consumption greatly improved, the battery repair is very good! Really worth buying a good product! Great!	JD.com
Henri New Energy Restoration	Battery Reconditioner	The fixer does work, after following the instructions a few times, the phone standby time is noticeably longer!	JD.com
Henri New Energy Restoration	Battery Reconditioner	Good quality, good comments.	JD.com
Henri New Energy Restoration Technology	Battery Reconditioner	Received the product is very happy, the night according to the requirements of the shutdown plugged in the product after charging, when the product shows a messy code, to this morning the product shows5000it seems that the product is credible to give a five-star review.	JD.com
Henri New Energy Restoration	Battery Reconditioner	Used. The effect is good, the imported chip is good, but also shows the time of repair, today the battery use obviously extended, the effect is amazing!	JD.com
Henri New Energy Restoration	Battery Reconditioner	It's okay, you can see the voltage and current! Looks good too.	JD.com

Henri New Energy Restoration Technology	Battery Reconditioner	Baby has been received, has been used for two days before coming to evaluate, baby is really good to use, it can repair the battery that has been damaged, when charging, the number in it has been changing constantly, before I have to charge many times a day, after using it once, it has effect, yesterday there was no more charging for a day. Kudos!	JD.com
Henri New Energy Restoration	Battery Reconditioner	Friends introduced to buy, very good, logistics is also very fast!	JD.com
Henri New Energy Restoration	Battery Reconditioner	Received the goods, the quality is very good, worth recommending to everyone.	JD.com
Henri New Energy Restoration	Battery Reconditioner	We'll have to see if we can do it.	JD.com
Henri New Energy Restoration	Battery Reconditioner	Quality is very good, repair cell phone battery has effect, the old cell phone battery with a long time, value for money, cost-effective	JD.com
Henri New Energy Restoration	Battery Reconditioner	It's a great one, it charges faster and doesn't heat up, it has a visible effect, it's a great value for money, the	JD.com
Henri New Energy Restoration	Battery Reconditioner	Received and used a few times the phone battery than the original a few percent more points feel very good satisfied!	JD.com
Henri New Energy Restoration	Battery Reconditioner	Professional cell phone battery repair received to try the effect is good to extend the life of the battery	JD.com
Henri New Energy Restoration Technology	Battery Reconditioner	phone battery. It is a tool for cruising cell phone battery life. Although the complaint has been used but I believe it will be effective. I would recommend it to my friends if it works well.	JD.com
Henri New Energy Restoration	Battery Reconditioner	Logistics is quite fast, business service attitude is also good	JD.com
Henri New Energy Restoration	Battery Reconditioner	Used it for two days, it's not bad, seems like the cell phone battery is durable	JD.com
Henri New Energy Restoration	Battery Reconditioner	Received the goods and tried it okay, I guess, use it for a while.	JD.com
Henri New Energy Restoration	Battery Reconditioner	Goods received, good quality, fast charging, very satisfied.	JD.com
Henri New Energy Restoration	Battery Reconditioner	Shipped pretty fast, packed with care, received in perfect condition, overall feel good! Customer service are quite good, communication is pleasant!	JD.com
Henri New Energy Restoration	Battery Reconditioner	Sticking with cell phones does play longer than before	JD.com
Henri New Energy Restoration	Battery Reconditioner	Not yet used, any special requirements for the use of this product, no instructions.	JD.com
Henri New Energy Restoration	Battery Reconditioner	Good quality and fast shipping	JD.com
Henri New Energy Restoration	Battery Reconditioner	Baby received, love it, worth buying.	JD.com
Henri New Energy Restoration	Battery Reconditioner	Received the goods, the data cable quality is great, very satisfied.	JD.com
Henri New Energy Restoration	Battery Reconditioner	The cell phone battery is very poorly durable, saw this product and ordered, the packaging is intact, the logistics is very fast, just used a few times, it's okay.	JD.com
Henri New Energy Restoration	Battery Reconditioner	Copy that.	JD.com
Henri New Energy Restoration	Battery Reconditioner	Cell Phone Battery Repair Godspeed, Digital Display Screen, Black Tech Repair Cell Phone Battery and Memory	JD.com
Henri New Energy Restoration	Battery Reconditioner	Baby received, tested it and it works well. Extend the life of cell phone battery.	JD.com
Henri New Energy Restoration	Battery Reconditioner	Fixes cell phones well and charges much better.	JD.com
Henri New Energy Restoration	Battery Reconditioner	Pretty good workmanship, fast charging speed, satisfactory	JD.com

Henri New Energy Restoration Technology	Battery Reconditioner	The current activated charger is pretty good quality! Tried out the repair and it works well!	JD.com
Henri New Energy Restoration Technology	Battery Reconditioner	Received and tried once before coming to evaluate, feel standby time is indeed longer than before, adhere to the instructions to use, I hope that after two months will have better results, look forward to!	JD.com
Henri New Energy Restoration Technology	Battery Reconditioner	Received, the courier is quite fast, opened and looked at, the quality can be, not yet used, used to say again	JD.com
Henri New Energy Restoration Technology	Battery Reconditioner	Purchased the smart chip this, last night has rushed, unlike the advertised full natural power off, need to manually power off. Product quality is too poor, sbsplug quality with very poor, gently touch on deformation crooked. Is indiscriminate feeling. In the future buyers should be small	JD.com
Henri New Energy Restoration Technology	Battery Reconditioner	The data cable has a nice color, charges quickly, has several ports, and is reasonably priced.	JD.com
Henri New Energy Restoration Technology	Battery Reconditioner	Packaging is intact, quality is guaranteed, practical and convenient, will buy back!	JD.com
Henri New Energy Restoration Technology	Battery Reconditioner	This baby logistics fast, the appearance of the brand is beautiful, chip repair for a period of time to see the effect. First give five-star praise!	JD.com
Henri New Energy Restoration Technology	Battery Reconditioner	Pretty good, fast shipping, fine workmanship pretty good, used it2times, put a lot of durability before.	JD.com
Henri New Energy Restoration Technology	Battery Reconditioner	Works great, tried it a couple times and fast shipping!	JD.com
Henri New Energy Restoration Technology	Battery Reconditioner	Good quality, fast charging, cheap	JD.com
Henri New Energy Restoration Technology	Battery Reconditioner	Received the goods used for two days, feel still very good, look forward to look forward to	JD.com
Henri New Energy Restoration Technology	Battery Reconditioner	Sleeping at night plugged in can, also repaired for a period of time, before10King of Glory phone is dead, currently can open to13, the effect is good. Charge charge a few times. The battery life is better than before. Satisfied with a shopping	JD.com
Henri New Energy Restoration Technology	Battery Reconditioner	The stuff is affordable and beautiful. Merchants are good too.	JD.com
Henri New Energy Restoration Technology	Battery Reconditioner	Cell phone battery repair device, very good, used a few days before coming to evaluation	JD.com
Henri New Energy Restoration Technology	Battery Reconditioner	and then look at the evaluation are good to use, I hold to buy to try, this is not try a little bit, but really good than the original charge is much faster and really a little effect. In the repair a few more times to see the battery durability in the evaluation.	JD.com
Henri New Energy Restoration Technology	Battery Reconditioner	Received, shipped quickly packed tightly, used well very satisfied!	JD.com
Henri New Energy Restoration Technology	Battery Reconditioner	The quality is particularly good, inexpensive, the shopkeeper has a very good attitude towards service	JD.com
Henri New Energy Restoration Technology	Battery Reconditioner	Great fixer, detailed instructions, easy to use, plug and play, full and stop!	JD.com
Henri New Energy Restoration Technology	Battery Reconditioner	Just received, logistics is quite fast, plugged in the display is normal looking forward to a better experience effect!	JD.com
Henri New Energy Restoration Technology	Battery Reconditioner	Very fast charging, very durable and very cost effective.	JD.com
Henri New Energy Restoration Technology	Battery Reconditioner	Good quality, easy to use and affordable.	JD.com
Henri New Energy Restoration Technology	Battery Reconditioner	Fast shipping, the product is small and exquisite, go out and carry does not take up space, has been used to feel good, satisfied.	JD.com
Henri New Energy Restoration Technology	Battery Reconditioner	The item has been received and is well packaged. Opened it up and it was very well packaged and had the instructions as well. It is in use now and has repaired an old cell phone battery. Works well on old cell phone batteries.	JD.com

Henri New Energy Restoration Technology	Battery Reconditioner	This repairer really good use, hungry to charge the phone, with several kinds of charging line did not charge, before also charge a day when, also did not charge open. Buy his family restorer, to hand charging twenty minutes, on the boot, but also rushed on twenty percent, rushed quite quickly. The price is affordable, the main good use.	JD.com
Henri New Energy Restoration	Battery Reconditioner	It is very easy to use! Worth recommending! Works great!	JD.com
Henri New Energy Restoration	Battery Reconditioner	Battery restoration is much more durable and affordable, satisfied.	JD.com
Henri New Energy Restoration	Battery Reconditioner	I bought a wrong product I bought a fixer and he sent me an impulse line and didn't reply to my messages.	JD.com
Henri New Energy Restoration	Battery Reconditioner	Received good quality, fast charging, nice and satisfied.	JD.com
Henri New Energy Restoration Technology	Battery Reconditioner	Goods received a look very beautiful, and the advertisement Miao described the same, with a very good, logistics is also very fast customer service is also very good, I am very satisfied with the goods.	JD.com
Henri New Energy Restoration	Battery Reconditioner	You need to read the instructions carefully to use it, and the service is great!	JD.com
Henri New Energy Restoration Technology	Battery Reconditioner	Good to use, the previous cell phone charging is particularly difficult to repair cell phone stores to replace the battery are very expensive, with this cell phone battery repairer, no longer need to spend money, and do not have to beg, since they can do, not only charging stable in the fast, but also to care for cell phone batteries do not hurt the phone, the use of security is also reliable, there is a need for you to get!	JD.com
Henri New Energy Restoration	Battery Reconditioner	Received, very good, I bought back several times, really good	JD.com
Henri New Energy Restoration	Battery Reconditioner	The quality is good, charging quite fast, and three plugs are very convenient, good value for money	JD.com
Henri New Energy Restoration	Battery Reconditioner	Very good good worth buying fast logistics good service reasonable price very satisfied overall very like it	JD.com
Henri New Energy Restoration	Battery Reconditioner	Baby arrived in good quality, very satisfied, cost-effective logistics is also strong	JD.com
Henri New Energy Restoration	Battery Reconditioner	Cell phone fixer is worth buying, it improves the battery life of your cell phone and also fixes problems such as lagging, making your cell phone more durable.	JD.com
Henri New Energy Restoration	Battery Reconditioner	Pretty good quality, affordable price, fast shipping	JD.com
Henri New Energy Restoration	Battery Reconditioner	Received, good quality, fast charging, cheap price too	JD.com
Henri New Energy Restoration	Battery Reconditioner	Affordable price, fast delivery, satisfied!	JD.com
Henri New Energy Restoration	Battery Reconditioner	The battery repairer is convenient to apply, good quality, good price, is a good product, the cell phone battery tube soon have a better repair effect.	JD.com
Henri New Energy Restoration	Battery Reconditioner	more durable, and I hope that with continued use, the battery will regain its original durability, so that I won't have to replace it again.	JD.com
Henri New Energy Restoration	Battery Reconditioner	will not be easy to break the cell phone battery maintenance is really good very like this cell phone battery repair God truly love it!	JD.com
Henri New Energy Restoration	Battery Reconditioner	Got the stuff, works great, still fixing it. Very happy with it.	JD.com
Henri New Energy Restoration	Battery Reconditioner	Good stuff, worth having.	JD.com
Henri New Energy Restoration	Battery Reconditioner	Just started to use, whether it is good or not, after a period of time to know, the shape is quite good-looking.	JD.com
Henri New Energy Restoration	Battery Reconditioner	Nice and fast charging	JD.com

Henli New Energy Restoration	Battery Reconditioner	Logistics to give a strong, tried it, charging speed is fast, good quality!	JD.com
Henli New Energy Restoration	Battery Reconditioner	The charging cable arrived, a 3-in-1 universal product for Android and Apple, and the cable is long enough. Satisfied	JD.com
Henli New Energy Restoration	Battery Reconditioner	The stuff works great the quality is great the workmanship is fine it's very easy to use!	JD.com
Henli New Energy Restoration	Battery Reconditioner	Cell phone repair charger is good, after cell phone repair charging, cell phone use longer, worth to buy a good charger.	JD.com
Henli New Energy Restoration	Battery Reconditioner	Baby is basically the same as the description, fast shipping, worth it!	JD.com
Henli New Energy Restoration	Battery Reconditioner	Express received, very good, very convenient, the quality is also good	JD.com
Henli New Energy Restoration	Battery Reconditioner	The cell phone repair charger works great and the battery lasts longer than before!	JD.com
Henli New Energy Restoration	Battery Reconditioner	Data cable received no bad, all can be used, the night to use the charging	JD.com
Henli New Energy Restoration	Battery Reconditioner	Cute shape, the repair effect is OK, value for money	JD.com
Henli New Energy Restoration	Battery Reconditioner	Arrived quickly, meticulous workmanship, test results, good to come back!	JD.com
Henli New Energy Restoration	Battery Reconditioner	Nice, recommended by a friend. Trustworthy.	JD.com
Henli New Energy Restoration	Battery Reconditioner	Works well. Charged the battery a couple times. Battery life is better than before. Satisfied with the shopping experience.	JD.com
Henli New Energy Restoration	Battery Reconditioner	The data cable is of good quality, charges quickly and is easy to use	JD.com
Henli New Energy Restoration	Battery Reconditioner	That cell phone battery repairer, lightweight, new style, shopping satisfaction.	JD.com
Henli New Energy Restoration	Battery Reconditioner	Fast shipping good quality good value for money	JD.com
Henli New Energy Restoration	Battery Reconditioner	According to the description of the introduction is very good, good service, heat less often, especially once open the King of Glory, the game opens faster, not bad dozens of things	JD.com
Henli New Energy Restoration	Battery Reconditioner	Really super like it, very good quality, as described by the seller!	JD.com
Henli New Energy Restoration	Battery Reconditioner	Nice, good quality, well made	JD.com
Henli New Energy Restoration	Battery Reconditioner	Battery Repair Magic received, very good, logistics is also very fast.	JD.com
Henli New Energy Restoration	Battery Reconditioner	Things received, well packaged, tried the effect is not bad	JD.com
Henli New Energy Restoration	Battery Reconditioner	The quality is great and the shipping was fast the price was very affordable.	JD.com
Henli New Energy Restoration	Battery Reconditioner	It's not bad, following the instructions the phone is already more durable than before	JD.com
Henli New Energy Restoration	Battery Reconditioner	The color of the data cable is very beautiful, multiple sockets are very convenient to use, fast charging and not hot, good!	JD.com
Henli New Energy Restoration	Battery Reconditioner	Satisfied with good cheap!	JD.com
Henli New Energy Restoration	Battery Reconditioner	Received the quality is very good very like very satisfied with a shopping!	JD.com
Henli New Energy Restoration	Battery Reconditioner	Baby received, especially good, repair effect is good, recommended to buy!	JD.com

Henri New Energy Restoration	Battery Reconditioner	The data cable feels good, the charging speed is very fast not hot praise	JD.com
Henri New Energy Restoration	Battery Reconditioner	Exquisite workmanship, cost-effective, very satisfactory	JD.com
Henri New Energy Restoration	Battery Reconditioner	Not bad, the delivery is quite fast, the packaging is quite good, the stuff is good	JD.com
Henri New Energy Restoration Technology	Battery Reconditioner	Used for a period of time, my ipad could only be plugged into the power supply to turn on, with this charge a few times, you can leave the power supply to turn on a more than ten minutes, is also an improvement, the instructions say that the longer the use of time, the better the repair	JD.com
Henri New Energy Restoration	Battery Reconditioner	Okay[Chuckles]	JD.com
Henri New Energy Restoration	Battery Reconditioner	I just got it. It feels good.	JD.com
Henri New Energy Restoration	Battery Reconditioner	It's good. I just used it. I don't know what it's like.	JD.com
Henri New Energy Restoration	Battery Reconditioner	It's good, the logistics is quite fast	JD.com
Henri New Energy Restoration	Battery Reconditioner	I received it and couldn't wait to try it out, it felt good!	JD.com
Henri New Energy Restoration	Battery Reconditioner	Good quality and exceptionally fast collection.	JD.com
Henri New Energy Restoration	Battery Reconditioner	It's good. Just used it yesterday. I'm using it. I'll buy a couple more when I'm done.	JD.com
Henri New Energy Restoration	Battery Reconditioner	Things received a good use, logistics fast quality, good service praise	JD.com
Henri New Energy Restoration	Battery Reconditioner	It's good. It's good.	JD.com
Henri New Energy Restoration Technology	Battery Reconditioner	used, no evaluation of the most excellent merchants and product quality assiduous, fast shipping, service attitude communication is very good, thank you, please. [hugs] [hugs] [hugs] [hugs] [kudos] [kudos][kudos	JD.com
Henri New Energy Restoration	Battery Reconditioner	It feels like it should work. Relatively 1% longer to watch videos on electricity	JD.com
Henri New Energy Restoration	Battery Reconditioner	Very good use,, really good, logistics is also very fast very satisfied	JD.com
Henri New Energy Restoration	Battery Reconditioner	The product was received, the logistics is very fast, the product quality is guaranteed!	JD.com
Henri New Energy Restoration	Battery Reconditioner	Really useful, I a Pingguo, a Hua办, used formore than3years, would have to charge every day, with the battery repairer two days before charging, good goods!	JD.com
Henri New Energy Restoration	Battery Reconditioner	The appearance of the new trend, a sense of weight, try it out first, wait for a few times after charging to see the effect and then follow up.	JD.com
Henri New Energy Restoration	Battery Reconditioner	Role experience: good display feeling: very good appearance description: good-looking design evaluation: good rechargeable battery: should be good	JD.com
Henri New Energy Restoration	Battery Reconditioner	The logistics is fast, the quality of the product should be tried before evaluating.	JD.com
Henri New Energy Restoration	Battery Reconditioner	I got it. It feels good.	JD.com
Henri New Energy Restoration	Battery Reconditioner	Well just received expecting results, waiting for the repair to work!	JD.com
Henri New Energy Restoration	Battery Reconditioner	I use after the evaluation, charging once or twice can not see the more fruitful	JD.com
Henri New Energy Restoration	Battery Reconditioner	Works well, no more hot charging	JD.com

Henri New Energy Restoration	Battery Reconditioner	The stuff is good and affordable. Save on battery replacement. The phone does not get stuck and the logistics are fast. Shipped on the same day.	JD.com
Henri New Energy Restoration	Battery Reconditioner	Goods have been received, the product quality is good, I'm this is a repurchase, worth promoting to give you praise!	JD.com
Henri New Energy Restoration	Battery Reconditioner	Works very well, excellent quality.	JD.com
Henri New Energy Restoration	Battery Reconditioner	Received, fast shipping! Great value for money, using it and will definitely follow up with a review if it works well! Recommended!	JD.com
Henri New Energy Restoration	Battery Reconditioner	Just received and used, do not know if it is good ah wait ah good in the evaluation	JD.com
Henri New Energy Restoration	Battery Reconditioner	Received. It's good. I'll comment after I use it.	JD.com
Henri New Energy Restoration	Battery Reconditioner	The product is good!	JD.com
Henri New Energy Restoration	Battery Reconditioner	Goods have been received, can charge the quality is very good	JD.com
Henri New Energy Restoration	Battery Reconditioner	Received the product, the logistics is very fast, after the trial and then give a follow-up comment!	JD.com
Henri New Energy Restoration	Battery Reconditioner	Trying it out.	JD.com
Henri New Energy Restoration	Battery Reconditioner	Received the goods, quite good, looking forward to a while to repair the battery	JD.com
Henri New Energy Restoration	Battery Reconditioner	Good quality of goods, details, quality, workmanship are very good, shipping thief, express delivery, great~❀❀❀❀❀	JD.com
Henri New Energy Restoration	Battery Reconditioner	Good quality, very practical, and shipped in a hurry, with excellent service.	JD.com
Henri New Energy Restoration	Battery Reconditioner	I used it once, I don't know if it worked or not. I'll use it again in a few days!	JD.com
Henri New Energy Restoration	Battery Reconditioner	Cell phone battery repair, buy and try how effective	JD.com
Henri New Energy Restoration	Battery Reconditioner	Used it right away, flickering changes, works well. Good review!	JD.com
Henri New Energy Restoration	Battery Reconditioner	Received a few days to evaluate the goods, the service attitude is very good has been collected store auxiliary	JD.com
Henri New Energy Restoration Technology	Battery Reconditioner	recovery initially, but there are two points should have homework. First, the cell phone battery can play a protective role; second, for the old phone false full, can do a real full, many people feel that the battery is not durable, in fact, not necessarily entirely due to battery aging, there is also a possibility of a false full state, seems to be full, but the battery is often in the display of the last 20%, will drop the power very quickly, and even part of the cell phone will be suddenly shut down. This thing does not charge fast, but will steadily fill your battery, the most obvious place to use is the last 20% will be more "durable" than before. That's when it really fills up.	JD.com
Henri New Energy Restoration	Battery Reconditioner	Very good use of logistics is also very fast, 亻, give you praise five red stars	JD.com
Henri New Energy Restoration	Battery Reconditioner	Product received. Good quality. Shipping was fast. Better than the original purchase. One price for one item. Hope it helps. Good reviews in advance.	JD.com
Henri New Energy Restoration	Battery Reconditioner	Received the product and will follow up with a review after use.	JD.com
Henri New Energy Restoration	Battery Reconditioner	It's working well. Keep using it.	JD.com
Henri New Energy Restoration	Battery Reconditioner	Dad said that the introduction said that it is very good, try it first, the follow-up evaluation	JD.com

Henri New Energy Restoration	Battery Reconditioner	Great product, beautifully chic and priced for what it is!	JD.com
Henri New Energy Restoration	Battery Reconditioner	This is a good product. Kudos.	JD.com
Henri New Energy Restoration	Battery Reconditioner	The quality of this product is guaranteed to be good and inexpensive, so you can trust and buy the product.	JD.com
Henri New Energy Restoration	Battery Reconditioner	The logistics is fast, the instrument is small and compact, and the quality is good.	JD.com
Henri New Energy Restoration	Battery Reconditioner	Very satisfied with the purchase	JD.com
Henri New Energy Restoration	Battery Reconditioner	I just received the baby today, it charges quickly and the phone doesn't get hot!	JD.com
Henri New Energy Restoration	Battery Reconditioner	Commodities received, look and feel good logistics is also very fast service attitude is very good, first give five-star praise, make additional comments after the completion of the	JD.com
Henri New Energy Restoration	Battery Reconditioner	I am very satisfied with this product, please recognize this product to buy with confidence.	JD.com
Henri New Energy Restoration	Battery Reconditioner	This user did not fill in a rating	JD.com
Henri New Energy Restoration Technology	Battery Reconditioner	Goods received, open a look at the quality is very good, I have used two back, and the previous did not use this product is not the same, the previous flush when the phone is always hot, with it after the phone is not hot, really very good and do not worry about it, logistics Zhang often fast, straight to us believe that your home production Pang to 100praise Oh.	JD.com
Henri New Energy Restoration	Battery Reconditioner	The product has been received as described-the product is trustworthy	JD.com
Henri New Energy Restoration	Battery Reconditioner	Design evaluation: the first time to see how to use, there are instructions to look at the will be	JD.com
Henri New Energy Restoration	Battery Reconditioner	The restorer looks good and displays the numbers clearly, so it's recommended!	JD.com
Henri New Energy Restoration	Battery Reconditioner	Just started using it, seems to be working, use it and see what happens!	JD.com
Henri New Energy Restoration	Battery Reconditioner	Goods have been received logistics quickly and good quality	JD.com
Henri New Energy Restoration	Battery Reconditioner	Fast logistics and good after-sales service	JD.com
Henri New Energy Restoration	Battery Reconditioner	It's really good, just bought it and used it	JD.com
Henri New Energy Restoration	Battery Reconditioner	It charges quickly and the battery is durable.	JD.com
Henri New Energy Restoration	Battery Reconditioner	X	JD.com
Henri New Energy Restoration	Battery Reconditioner	Looked good quality, don't know how well it works yet, just received the goods.	JD.com
Henri New Energy Restoration	Battery Reconditioner	Good baby, fine workmanship, arrived and used, looked good, the seller shipped quickly, 5stars praise	JD.com
Henri New Energy Restoration	Battery Reconditioner	At work, ask customer service if you don't understand something and get back in seconds[kudos]	JD.com
Henri New Energy Restoration	Battery Reconditioner	The quality of the product is very good, the idea is good, the service	JD.com
Henri New Energy Restoration	Battery Reconditioner	Beautiful appearance is very cool, the material is light but solid, the repair effect will come back to the review after the test	JD.com
Henri New Energy Restoration	Battery Reconditioner	Good service attitude, received the goods immediately tried, charging quickly, good use	JD.com

Henri New Energy Restoration	Battery Reconditioner	We'll talk about it after we use it.	JD.com
Henri New Energy Restoration	Battery Reconditioner	Charger is very bright atmosphere atmosphere things also fast should be a good charger	JD.com
Henri New Energy Restoration	Battery Reconditioner	The restorer is great, with a five year warranty, so feel free to use it.	JD.com
Henri New Energy Restoration	Battery Reconditioner	The restorer was received and works well after a couple of uses.	JD.com
Henri New Energy Restoration	Battery Reconditioner	This product is the same as described on the video, I received it to try it works great!	JD.com
Henri New Energy Restoration	Battery Reconditioner	Goods have received the quality is very good, will repurchase again!	JD.com
Henri New Energy Restoration	Battery Reconditioner	Repairer is indeed very useful, just bought back and used once, the phone used to feel the battery life a lot longer, very recommended to use!	JD.com
Henri New Energy Restoration	Battery Reconditioner	The long awaited thing finally received, how long customer service help!	JD.com
Henri New Energy Restoration	Battery Reconditioner	Appearance is in good condition! I don't know how well it works, use it and then say, if it's good, I highly recommend it!	JD.com
Henri New Energy Restoration	Battery Reconditioner	The goods have been received, immediately used to try, feel or repair effect.	JD.com
Henri New Energy Restoration	Battery Reconditioner	Practical! Worth having!	JD.com
Henri New Energy Restoration	Battery Reconditioner	continue to follow the instructions and then use a few times, really can achieve the desired effect, but also really save time and money.	JD.com
Henri New Energy Restoration	Battery Reconditioner	Super good use, logistics is fast, the merchants keep their word, worth buying!	JD.com
Henri New Energy Restoration	Battery Reconditioner	The item works, it's affordable and works well, and it has a generous appearance	JD.com
Henri New Energy Restoration	Battery Reconditioner	Cell phone repairer received not only the effect of how to make good use of the chemical continue to repurchase the first five-star praise!	JD.com
Henri New Energy Restoration	Battery Reconditioner	Fast delivery, well-packed, try to feel good, looking forward to the later practical effects	JD.com
Henri New Energy Restoration	Battery Reconditioner	I'm using it now on a charge, charge it a few times and see if it works	JD.com
Henri New Energy Restoration	Battery Reconditioner	The goods have arrived[smile], on the use of good will be introduced to friends, I hope it works well,	JD.com
Henri New Energy Restoration	Battery Reconditioner	The line universal charger, like, genuine[love][love][love][love][love][love]	JD.com
Henri New Energy Restoration	Battery Reconditioner	The product was received and the logistics was fast. The effect is quite good.	JD.com
Henri New Energy Restoration	Battery Reconditioner	Good quality and fast logistics, trustworthy[kudos][kudos][kudos][kudos	JD.com
Henri New Energy Restoration	Battery Reconditioner	It's so good! Solves phone lagging problems!	JD.com
Henri New Energy Restoration	Battery Reconditioner	It's pretty good, good value for money. Extremely easy to use, recommend buying	JD.com
Henri New Energy Restoration	Battery Reconditioner	Received it and used it! Should be just as the store said it would be! 5 stars!	JD.com
Henri New Energy Restoration	Battery Reconditioner	Used it a couple of times with some results, will comment after a few more days of use.	JD.com
Henri New Energy Restoration	Battery Reconditioner	Works great, fast shipping, will buy from here again next time!	JD.com

Henri New Energy Restoration	Battery Reconditioner	Product received, quite good, good quality	JD.com
Henri New Energy Restoration	Battery Reconditioner	The cell phone battery recovery is good and recommendable to buy.	JD.com
Henri New Energy Restoration	Battery Reconditioner	Received it and used it for two days! The battery does have a little more standby time than before I didn't use it!	JD.com
Henri New Energy Restoration	Battery Reconditioner	I received the cell phone repairer, and I repaired it once, but I didn't feel how effective it is? Need to repair many times to see the effect, right? The logistics is fast.	JD.com
Henri New Energy Restoration	Battery Reconditioner	Used it, works well, value for money	JD.com
Henri New Energy Restoration	Battery Reconditioner	Merchant shipped quickly, good value, 5 stars.	JD.com
Henri New Energy Restoration	Battery Reconditioner	The outside off is very delicate, generous and beautiful. It is worth buying!	JD.com
Henri New Energy Restoration	Battery Reconditioner	Works great, fixes cell phones	JD.com
Henri New Energy Restoration	Battery Reconditioner	The logistics is fast, the stuff is really good, and it's recommendable.	JD.com
Henri New Energy Restoration	Battery Reconditioner	The goods have been received, the logistics is very fast, the packaging is intact. It is being used and hopefully it will fix my cell phone.	JD.com
Henri New Energy Restoration	Battery Reconditioner	As described by Dodo, the product is of good quality and easy to use!	JD.com
Henri New Energy Restoration	Battery Reconditioner	Received it, looks good, will follow up later.	JD.com
Henri New Energy Restoration	Battery Reconditioner	Fast charging and the phone is less likely to heat up	JD.com
Henri New Energy Restoration	Battery Reconditioner	I hope it works[cool]	JD.com
Henri New Energy Restoration	Battery Reconditioner	Received, very good quality, satisfied with the use, praise, praise, praise.	JD.com
Henri New Energy Restoration	Battery Reconditioner	Good quality, as advertised, logistics is also fast, back to start using, the use of the effect will be evaluated later!	JD.com
Henri New Energy Restoration	Battery Reconditioner	It's nice and compact, and it's a great fix.	JD.com
Henri New Energy Restoration	Battery Reconditioner	cheap and affordableandworks[big love]big love][big	JD.com
Henri New Energy Restoration	Battery Reconditioner	Very happy with it, hopefully it will be used.	JD.com
Henri New Energy Restoration	Battery Reconditioner	Received the item and used it once with good results.	JD.com
Henri New Energy Restoration	Battery Reconditioner	Good value for money good value for money cheap try it out and come back for more reviews	JD.com
Henri New Energy Restoration	Battery Reconditioner	Yes, the first time I used it, I saw results.	JD.com
Henri New Energy Restoration	Battery Reconditioner	Battery restorer received. Good quality, good price. Logistics is also very fast, kudos.	JD.com
Henri New Energy Restoration	Battery Reconditioner	Fast logistics, good price and good service.	JD.com
Henri New Energy Restoration	Battery Reconditioner	Use very good, the service is also very good, there is a wire contact is not good, immediately send a replacement, thank you!	JD.com
Henri New Energy Restoration	Battery Reconditioner	Fast shipping, pretty good quality, as described by the seller, very satisfied!	JD.com

Henri New Energy Restoration	Battery Reconditioner	Very good quality, fixes cell phone batteries fast!	JD.com
Henri New Energy Restoration	Battery Reconditioner	The baby was received and given 5 stars!	JD.com
Henri New Energy Restoration	Battery Reconditioner	Good! This has a data display, look is technology, wait to use some time to appreciate the benefits it brings to the phone, fast delivery, praise!	JD.com
Henri New Energy Restoration	Battery Reconditioner	Baby received, too good to use, before the phone-day rushed two back to power, now rushed-back can use a day, so give five stars.	JD.com
Henri New Energy Restoration	Battery Reconditioner	The shape of the product is unique, the box is intact, the repairer will repair the cell phone battery.	JD.com
Henri New Energy Restoration Technology	Battery Reconditioner	with facts, it is really good, it can eliminate a lot of traffic. It's a great way to optimize your phone while repairing it, and it also has a function to organize the debris in your phone.	JD.com
Henri New Energy Restoration	Battery Reconditioner	On the mend, not sure how well it works? After using it, I will come back to make a follow-up comment. After-sales service is very good, trustworthy.	JD.com
Henri New Energy Restoration	Battery Reconditioner	Baby just received and used it right away, charging is four times faster.	JD.com
Henri New Energy Restoration	Battery Reconditioner	The goods have been received, well packaged, to be tried to see how the effect!	JD.com
Henri New Energy Restoration	Battery Reconditioner	Used it. Pretty good. I give it 5 stars.	JD.com
Henri New Energy Restoration	Battery Reconditioner	Cell phone battery repairer received, the product packaging is exquisite, the appearance of beautiful quality is very good.	JD.com
Henri New Energy Restoration	Battery Reconditioner	The product is pretty good, good experience with my phone.	JD.com
Henri New Energy Restoration	Battery Reconditioner	The product is good and effective……can be worth having!	JD.com
Henri New Energy Restoration	Battery Reconditioner	Try it first and if it works, will repurchase!	JD.com
Henri New Energy Restoration	Battery Reconditioner	Received the cell phone battery repairer, now in the process of repairing, hope it is a good experience, looking forward to it!	JD.com
Henri New Energy Restoration	Battery Reconditioner	The restorer is of good quality, looks great, well made, and looks forward to refreshing the battery.	JD.com
Henri New Energy Restoration	Battery Reconditioner	[heehee][][hee][hee]cell phone charging repairer received, logistics is very fast well-packed, I wish business is prosperous, the financial resources of the retailers much more	JD.com
Henri New Energy Restoration	Battery Reconditioner	The item was received, good quality, easy to use and good delivery.	JD.com
Henri New Energy Restoration	Battery Reconditioner	Goods received to the same description, service enthusiasm, well-packed, logistics is also very fast. Full five-star praise!	JD.com
Henri New Energy Restoration	Battery Reconditioner	Cell Phone. Battery Repairer received. It is the same as described. Great quality, great logistics, great customer service, great.	JD.com
Henri New Energy Restoration	Battery Reconditioner	The goods have been received, fast shipping, tightly packaged, must praise!!!!	JD.com
Henri New Energy Restoration	Battery Reconditioner	The product has been received, tried it, very good, good cell phone battery repair, and there are voltage and current display, really good.	JD.com
Henri New Energy Restoration	Battery Reconditioner	Fast delivery, well packaged, good value, works great, thanks!	JD.com
Henri New Energy Restoration	Battery Reconditioner	The cell phone battery repairer is easy to use and works well.	JD.com
Henri New Energy Restoration	Battery Reconditioner	Works great, inexpensive, won't know how it works until a few days later	JD.com
Henri New Energy Restoration	Battery Reconditioner	Baby received, love it👍with it the phone is easy, thanks to the business! Good luck with business👌👌👌👌👌	JD.com

Tenfi New Energy Restoration	Battery Reconditioner	Baby received, good quality, easy to use, easy to use, express safe and reliable, worth buying!	JD.com
Tenfi New Energy Restoration	Battery Reconditioner	Received the goods, logistics is very fast, back to try quite good, worth buying.	JD.com
Tenfi New Energy Restoration	Battery Reconditioner	This cell phone repairer is small, exquisite appearance, easy to use, worth buying.	JD.com
Tenfi New Energy Restoration	Battery Reconditioner	The product is very satisfied with the streamlined look of the Digital Function Display Automotive.	JD.com
Tenfi New Energy Restoration	Battery Reconditioner	delivery, packaging is very good, logistics is also very fast, five-star praise to a, the use of good results and then additional comments.	JD.com
Tenfi New Energy Restoration	Battery Reconditioner	Item received try it works great thanks to seller service.	JD.com
Tenfi New Energy Restoration	Battery Reconditioner	I just received the item, tried it out and the results are still unknown.	JD.com
Tenfi New Energy Restoration	Battery Reconditioner	Goods received good quality, logistics is also very fast	JD.com
Tenfi New Energy Restoration	Battery Reconditioner	Goods are very good, well-packed not damaged at all, logistics is fast and trustworthy!	JD.com
Tenfi New Energy Restoration	Battery Reconditioner	Baby received, the logistics is very fast, the product quality is very good, as described, especially customer service Miss is very provincial patience, very enthusiastic.	JD.com
Tenfi New Energy Restoration	Battery Reconditioner	I received it today and I started using it then and it is indeed a good product[kudos][kudos]! Worth having!	JD.com
Tenfi New Energy Restoration	Battery Reconditioner	The logistics is very fast and the customer service after-sales attitude is very good.	JD.com
Tenfi New Energy Restoration	Battery Reconditioner	Smart Fix little machine received, small and compact, works great!	JD.com
Tenfi New Energy Restoration	Battery Reconditioner	New style and easy to use.	JD.com
Tenfi New Energy Restoration	Battery Reconditioner	Very nice looking well made nice nice nice	JD.com
Tenfi New Energy Restoration	Battery Reconditioner	Good quality to repair cell phone batteries	JD.com
Tenfi New Energy Restoration	Battery Reconditioner	Nice product, trustworthy……	JD.com
Tenfi New Energy Restoration	Battery Reconditioner	couldn't wait to test the goods when I received them, now charging▯phone is not hot; it's a genuine product worthy of recommendation and praise!	JD.com
Tenfi New Energy Restoration	Battery Reconditioner	Goods and business publicity and promotion of the same, fast logistics, business service is good.	JD.com
Tenfi New Energy Restoration	Battery Reconditioner	After turning it off, I charged my phone and it felt good!	JD.com
Tenfi New Energy Restoration	Battery Reconditioner	This is a real high tech product, with it you don't have to worry about changing batteries!	JD.com
Tenfi New Energy Restoration	Battery Reconditioner	Baby received, as described, looks great, first five star review a...	JD.com
Tenfi New Energy Restoration	Battery Reconditioner	logistics is very fast. Cell phone battery repairer, beautiful appearance, easy to use, good quality, fast repair, five-star praise!	JD.com
Tenfi New Energy Restoration	Battery Reconditioner	Received quickly after ordering, customer service patience and guidance, good quality material! Repair batteries fast!	JD.com
Tenfi New Energy Restoration	Battery Reconditioner	time being there is not much change carry description: very convenient product design: very like display experience: display is very clear	JD.com
Tenfi New Energy Restoration	Battery Reconditioner	Tried, because the time is short did not see the effect of it, charging are normal, it should be no problem, I wish the boss prosperous business ah!	JD.com

Henri New Energy Restoration	Battery Reconditioner	Logistics is very fast, the item is exquisite, after getting the goods and try to feel very good, customer service personnel attitude is very good	JD.com
Henri New Energy Restoration	Battery Reconditioner	Repairer received, fast logistics, good packaging, repair effect, to be used after the evaluation, looking forward to good results.	JD.com
Henri New Energy Restoration	Battery Reconditioner	Fast logistics, beautiful appearance, good service	JD.com
Henri New Energy Restoration	Battery Reconditioner	Nice stuff, good material, charges fast.	JD.com
Henri New Energy Restoration	Battery Reconditioner	Good quality, fast logistics and good service.	JD.com
Henri New Energy Restoration	Battery Reconditioner	Logistics was fast! Good value for money! Novel shape!	JD.com
Henri New Energy Restoration	Battery Reconditioner	Cell phone fixer is good, worth buying.	JD.com
Henri New Energy Restoration	Battery Reconditioner	Fast logistics, good packaging, product-specific use a few days, the phone battery improved quite a lot, is a good product!	JD.com
Henri New Energy Restoration	Battery Reconditioner	The stuff is really good, repair the battery is pretty good, need friends go buy it!	JD.com
Henri New Energy Restoration	Battery Reconditioner	Logistics fast service attitude is good, the arrival of timely, intact packaging, the physical and publicity of the full compliance, special to give praise!	JD.com
Henri New Energy Restoration Technology	Battery Reconditioner	Display experience: very good product design: very good product appearance: very good looking rechargeable battery: very good looking product function: very good carrying description: very convenient	JD.com
Henri New Energy Restoration	Battery Reconditioner	The product is exquisitely packaged, easy and quick to use, and has outstanding functionality. Value for money.	JD.com
Henri New Energy Restoration	Battery Reconditioner	Great purchase received great quality!	JD.com
Henri New Energy Restoration	Battery Reconditioner	Fast logistics, convenient and practical, fast charging!	JD.com
Henri New Energy Restoration	Battery Reconditioner	Logistics is very fast, the price is affordable, the stuff is very good, five stars, next time to come back!	JD.com
Henri New Energy Restoration	Battery Reconditioner	The battery restorer was received. It is very atmospheric, and the instructions are very detailed. Logistics is very fast, praise.	JD.com
Henri New Energy Restoration	Battery Reconditioner	Tried, the quality is very good, logistics is also very fast, the business attitude is good everyone rest assured to buy it!	JD.com
Henri New Energy Restoration	Battery Reconditioner	I received the goods and used it, it is more durable than before, and the phone is not so bad.	JD.com
Henri New Energy Restoration	Battery Reconditioner	The product is affordable. The quality is in use.	JD.com
Henri New Energy Restoration	Battery Reconditioner	Goods have been received, well-packed, logistics is also very fast, used two days the phone is indeed not hot before, the speed is also faster[praise]	JD.com
Henri New Energy Restoration	Battery Reconditioner	Cell phone battery repairer received very good quality logistics fast next time to buy!	JD.com
Henri New Energy Restoration	Battery Reconditioner	Design quality: feels like a fix the first time around Charging speed: stable and fast charging Maintenance cost: low cost	JD.com
Henri New Energy Restoration	Battery Reconditioner	Multi-functional cell phone battery repairer received, the quality is very good, I like it very much.	JD.com
Henri New Energy Restoration Technology	Battery Reconditioner	Things have been received without damage such as pieces, repair device work is very peculiar, feel very good, there is also a display, for the genuine, to be used after the follow-up comment, to the merchant praise, five-star praise.	JD.com
Henri New Energy Restoration	Battery Reconditioner	effect is also good, the next time will be back to buy, I wish the boss business is booming, the source of wealth to make a fortune.	JD.com

Henri New Energy Restoration	Battery Reconditioner	Good product quality, easy to carry, with environmental protection for the battery less pollution of the environment.	JD.com
Henri New Energy Restoration	Battery Reconditioner	Beautifully packaged, beautiful appearance, reliable quality, easy to use, worth having.	JD.com
Henri New Energy Restoration	Battery Reconditioner	The product is compact, affordable, durable and effective! Good value for money. Shipping is timely and on point.	JD.com
Henri New Energy Restoration	Battery Reconditioner	Satisfied with the item, good value, economical and timely delivery.	JD.com
Henri New Energy Restoration	Battery Reconditioner	Good, beautiful atmosphere, the effect should be good, just pick up the courier, see the effect, late in the additional evaluation	JD.com
Henri New Energy Restoration	Battery Reconditioner	Works great, feels great, and is very well made	JD.com
Henri New Energy Restoration	Battery Reconditioner	Fast logistics, good service attitude, fine products, compact Linglong.	JD.com
Henri New Energy Restoration	Battery Reconditioner	The goods have been received,,, fine workmanship, beautiful appearance, good quality, I am satisfied. I am satisfied.	JD.com
Henri New Energy Restoration	Battery Reconditioner	Just received the goods, packaging is good, exquisite workmanship, fast logistics, good service, give praise!	JD.com
Henri New Energy Restoration	Battery Reconditioner	Baby is good, value for money, business integrity, customer service Yujia, logistics!	JD.com
Henri New Energy Restoration	Battery Reconditioner	The baby is quite good, I like the charging is also very fast. Packaging is also very good	JD.com
Henri New Energy Restoration	Battery Reconditioner	Portable, inexpensive, and a godsend for fixing batteries on your own.	JD.com
Henri New Energy Restoration	Battery Reconditioner	Battery Repairer received, logistics is also very fast, used a few days is very good, with the business description of the same, the battery is durable, give praise.	JD.com
Henri New Energy Restoration	Battery Reconditioner	Received the goods, logistics is very fast, customer service is also very patient, very enthusiastic, tell how to use the detailed steps, the effect must be good!	JD.com
Henri New Energy Restoration	Battery Reconditioner	Good value for money, very practical and effective!	JD.com
Henri New Energy Restoration	Battery Reconditioner	[Lots of love][Lots of love]	JD.com
Henri New Energy Restoration	Battery Reconditioner	Baby received a very delicate and beautiful, immediately used to see the effect! I will share it with my friends as soon as it works!	JD.com
Henri New Energy Restoration	Battery Reconditioner	Can, quality can, cell phone repair electric board	JD.com
Henri New Energy Restoration	Battery Reconditioner	Logistics is very fast, the service attitude is very good, immediately after receiving the goods to take an old cell phone to try to fix it, the	JD.com
Henri New Energy Restoration	Battery Reconditioner	Fast shipping, the product is very delicate, immediately tried to charge, feel good, trustworthy.	JD.com
Henri New Energy Restoration Technology	Battery Reconditioner	logistics is very fast, well-packed, I have been all charged for an hour, looking very satisfied, I also consulted the store, people told me a repair to three hours, my phone is good is the battery does not work, I hope that my cell phone to repair and normal as well, will also come back to buy a, will recommend to friends.	JD.com
Henri New Energy Restoration Technology	Battery Reconditioner	Baby received, logistics is very strong, this product I have been concerned about for a long time, that can be trusted, it seems that I choose is right! The product is good value for money, exquisite and beautiful practical and generous, very satisfied with a shopping, recommended, five-star praise!	JD.com
Henri New Energy Restoration	Battery Reconditioner	The effect is being tested, I don't know what the quality is like, I'll give it a good review first and wait for it.	JD.com
Henri New Energy Restoration	Battery Reconditioner	Things received, good-looking, well-packed, as described, worth recommending the purchase.	JD.com

Henri New Energy Restoration	Battery Reconditioner	The current shows that the repair has some effect, use it a few more times and expect the effect to be more pronounced	JD.com
Henri New Energy Restoration	Battery Reconditioner	Fast shipping, affordable price, good value for money, 5 stars!	JD.com
Henri New Energy Restoration	Battery Reconditioner	Product Appearance: cool shapeRechargeable Battery: charges quicklyProduct Function: as advertised description, give 5 stars.	JD.com
Henri New Energy Restoration	Battery Reconditioner	Received the goods yesterday, the evening phone charged to try, feel charged more slightly faster, but also more secure, continue to experience, should be fixed better.	JD.com
Henri New Energy Restoration Technology	Battery Reconditioner	save money to buy, do not hesitate Baby is very good, I have to say, the customer service attitude is also too good, right! A very good shopping experience, attentive service, fast logistics, delivery is also very thoughtful, attitude is also very good, praise!	JD.com
Henri New Energy Restoration	Battery Reconditioner	This has been received, feels great, fast delivery, will see how it works when I charge it at night.	JD.com
Henri New Energy Restoration Technology	Battery Reconditioner	phone battery has been undercharged, each time only charged to90%can not be charged, now finally can be fully charged, adhere to the requirements to do, looking forward to better results.	JD.com
Henri New Energy Restoration Technology	Battery Reconditioner	really works, I thought there was no saving, my OPPO is now barreling along. one back with the intention of trying it out, followed the tutorial and it really worked, the battery lasted twice as long and last3weeks, really great deal	JD.com
Henri New Energy Restoration	Battery Reconditioner	The packaging is perfect, logistics is fast, the appearance is well designed, it is easy to use, and it is effective in repairing the effect of the cell phone.	JD.com
Henri New Energy Restoration Technology	Battery Reconditioner	the repair effect is very good, repair one day, the next day to try, cell phone standby is obviously longer than before not repair, the effect is good, did not lay hands on the hurry to order!	JD.com
Henri New Energy Restoration Technology	Battery Reconditioner	Goods have been received to take home to open to try the effect I do not understand what it means only to see the digital display constantly changing[smile]and so onuse of the effect of good and then give you a follow-up evaluation	JD.com
Henri New Energy Restoration	Battery Reconditioner	I wonder how it works?	JD.com
Henri New Energy Restoration Technology	Battery Reconditioner	appearance, practicality, easy to operate, the phone battery has a maintenance function, logistics and express speed fast, customer service attitude thoughtful and patient, I'm very satisfied Thank you.	JD.com
Henri New Energy Restoration	Battery Reconditioner	Battery Repairer received, the product appearance exquisite, first give five-star praise, service to see the effect of giving a follow-up.	JD.com
Henri New Energy Restoration	Battery Reconditioner	carry, repair powerful, cost-effective, inexpensive, has been shared with friends and family, worth recommending and repurchase.	JD.com
Henri New Energy Restoration	Battery Reconditioner	East received, immediately used, look at the jumping numbers, should be useful, with a period of time to see, have the effect to come back to the follow-up comment!	JD.com
Henri New Energy Restoration	Battery Reconditioner	The item is very good, the logistics is fast, honoring the sincerity of the promise, deserves praise.	JD.com
Henri New Energy Restoration	Battery Reconditioner	This is a great way to fix your cell phone battery! The battery is charged for hours!	JD.com
Henri New Energy Restoration	Battery Reconditioner	Cell phone repair device received, try the effect can be, well-packed! The appearance still looks good! There is a need for friends can choose, courier brother responsible!	JD.com
Henri New Energy Restoration	Battery Reconditioner	Battery Repairer Skewer, can effectively improve the battery life more durable clean up cell phone garbage, improve the running speed of the phone. Very good.	JD.com
Henri New Energy Restoration Technology	Battery Reconditioner	Fast shipping. After receiving the goods, check the packaging is intact, open the package to take out the device, the device looks good, easy to use, according to the instructions guide immediately inserted into the power supply, the device smoothly into the normal working state. Kudos.	JD.com
Henri New Energy Restoration Technology	Battery Reconditioner	Baby just received, the seller's attitude is very good, logistics is also very fast, the baby and the business description is consistent, is genuine, will continue to patronize your store. Intentionally friends can order.	JD.com

Henri New Energy Restoration	Battery Reconditioner	Cell phone battery restorer received, charged once, feel the effect is very good, thank you boss!	JD.com
Henri New Energy Restoration Technology	Battery Reconditioner	to change the battery, after repairing the phone with a longer time some, and does not heat up, and also automatically clean up the phone system cache garbage, the phone has a significantly smooth, the boss said that often used to repair will be better.	JD.com
Henri New Energy Restoration	Battery Reconditioner	I just got it this morning and the delivery was at the door last night, couldn't wait to bring it in to charge my phone, all is well, hopefully it works.	JD.com
Henri New Energy Restoration	Battery Reconditioner	This smart charge is very good indeed is the same as advertised above, the service and good rest assured to buy!	JD.com
Henri New Energy Restoration	Battery Reconditioner	Cell phone repairer received, especially delicate, so that more durable, and energy-saving and time-saving, convenient and affordable! 5stars praise!	JD.com
Henri New Energy Restoration Technology	Battery Reconditioner	Used a few days do not know, too old phone is impossible to repair, because the battery has been bad or deep sleep, there is a Huawei phone with6years in the middle of the original factory replaced the battery with three years, so far can not see, need to observe, and then tell you	JD.com
Henri New Energy Restoration	Battery Reconditioner	It looks good, try charging it twice, pulse charging activates the battery, it should be reliable.	JD.com
Henri New Energy Restoration	Battery Reconditioner	It works well, my phone kept prompting battery performance drop, after using it, it's fine[kudos][kudos][kudos][kudos	JD.com
Henri New Energy Restoration Technology	Battery Reconditioner	The logistics is very fast the package is intact. Cell phone battery repairer. Tried some work,, the quality is very good, beautiful and practical, the appearance of the perfect design, the light is good, very convenient, good.	JD.com
Henri New Energy Restoration Technology	Battery Reconditioner	Today's arrival, the logistics is quite fast, the business delivery is quite fast, pick up the courier I opened, the packaging is quite tight, the battery shell is quite beautiful, the appearance is quite fine, and so I have what I need in the future also go to your home to buy.	JD.com
Henri New Energy Restoration Technology	Battery Reconditioner	When you get the courier, the first moment was stunned, I did not expect the appearance of such a good-looking, as if a small car model, loved by young people, the quality is also very good, it is very good, the businessman's service attitude is particularly good, it is very good.	JD.com
Henri New Energy Restoration Technology	Battery Reconditioner	see there can be repair Ai Pai battery artifacts, because the battery life is longer, discharge faster, see there is this artifact to buy to try to see the home of the Ai Pai batteries can not be repaired to repair a few times to come back to follow up on the effect. The first repair a few times and then evaluate the effect. Roundtone Express Shenzhen delivery Beijing West Banner more than two days on delivery received the first five-star praise, the effect of the need to see the guest to see the additional evaluation. There are WeChat 3yuan red packets to incomeafter evaluation	JD.com
Henri New Energy Restoration Technology	Battery Reconditioner	Due to the busy courier brother put into the delivery box on the day did not have time to go pick up. After two days to get. To charge[stay] [skimming] [skimming] [skimming] [skimming] [skimming]to get back to plug in to the charging treasure light is normal. I hope it works to repair the battery	JD.com
Henri New Energy Restoration Technology	Battery Reconditioner	to view the box intact, repair the effect of the use of the repair device to follow up after the use of logistics, fast service is good, very satisfied with a shopping, give five-star praise!	JD.com
Henri New Energy Restoration Technology	Battery Reconditioner	Cell Phone Battery Repairer received, the merchant shipped quickly, express delivery. The packaging is intact. The manual inside is very detailed, and there are cell phone cleaning instructions, very practical.	JD.com
Henri New Energy Restoration Technology	Battery Reconditioner	Harvest is used, the value of jumping, the surrounding bright flash, feel in the non-stop work, the past charge can only be up to ninety like, now can reach one hundred, and speed up, and then charge a few days certainly have the effect!	JD.com
Henri New Energy Restoration Technology	Battery Reconditioner	Cell phone battery repairer received very satisfied, packaging is very good, logistics is very fast, cell phone battery is not durable just used to repair the cell phone battery, I hope the cell phone battery durable.	JD.com
Henri New Energy Restoration Technology	Battery Reconditioner	This charging repairer is very good, my cell phone can not turn off the power, and a long time the phone is hot, use this repairer a few days later, the phone this phenomenon has significantly improved, it is worth having.	JD.com

Henri New Energy Restoration	Battery Reconditioner	Cell phone battery repairer received to use four hours, now do not know how effective, with a period of time in the follow-up, customer attitude is very good point👍	JD.com
Henri New Energy Restoration	Battery Reconditioner	With it, my Huawei and Apple phones are considered beneficial, from full charge to99%is about ten minutes longer than before, which can be given a good review!	JD.com
Henri New Energy Restoration Technology	Battery Reconditioner	Used two days, feel the quality is still very good, before charging the phone will be hot, after using this repairer there is no heat, the phone is not stuck, in short, purchased this product value, businessmen shipment is fast, courier brother delivery is also timely, must give five stars.	JD.com
Henri New Energy Restoration Technology	Battery Reconditioner	Baby received, very good, packaging is very good no defects, with the description in line. Baby is very beautiful, can repair the cell phone battery, I have a flat version, charging can not be charged, with this baby, bidding can make the electric signal shine, this baby is quite good, like. Thank you for mentioning the good goods, thank you courier boy, good service attitude, fast delivery. Thank you!	JD.com
Henri New Energy Restoration Technology	Battery Reconditioner	Cell phone battery repairer received, well-packaged, good style and quality, small and exquisite very practical, the cell phone battery to play a role in repairing and protecting the role of the phone, it is worth buying, the logistics courier is very timely, give five-star praise.	JD.com
Henri New Energy Restoration Technology	Battery Reconditioner	Cell phone repairer just received, get back to install to the phone charged, do not know how to repair the effect, the Internet also has a cheaper than this are there, I bought this is to be a little more expensive, I hope to have the effect of the cell phone has a big help.	JD.com
Henri New Energy Restoration Technology	Battery Reconditioner	Cell phone battery repairer received, I used it to repair the glory and Apple, used3days to evaluate, off the charge is really effective! Merchants shipping very quickly logistics is also fast, five-star praise!	JD.com
Henri New Energy Restoration Technology	Battery Reconditioner	logistics is very fast, the data at a glance, the product is inexpensive, exquisite and beautiful practical and generous, very satisfied with a shopping, recommendable, five-star praise!👍👍	JD.com
Henri New Energy Restoration Technology	Battery Reconditioner	good quality. Received the goods I immediately used to charge, the result is really cell phone one day down the power than not use the restorer when more very each, is true, for you to praise.	JD.com
Henri New Energy Restoration Technology	Battery Reconditioner	Did not start the baby, you can place an order to buy a to try, repair your second wife, female second husband cutie, cell phone📱battery repairer, repair battery and lagging, October listing of the artifacts, hahaha😆	JD.com
Henri New Energy Restoration Technology	Battery Reconditioner	packaging is very good, received immediately to the cell phone battery repair for three hours, feel the phone mom like clear, thanks to customer service is not afraid of trouble to answer Yan how to plug in the original how to use, so give five-star praise.	JD.com
Henri New Energy Restoration Technology	Battery Reconditioner	Charge protection device has been received, installed after charging very satisfied! Able to protect the phone battery is very good and practical, worth buying five stars and beautiful appearance with digital display very satisfied!	JD.com
Henri New Energy Restoration Technology	Battery Reconditioner	Received [Universal] cell phone battery repairer to extend battery life optimize the system without lagging voltage regulator charger, the use of the results, it is really great[kudos] [clap] [handshake] [shake]	JD.com
Henri New Energy Restoration Technology	Battery Reconditioner	shipping quite fast thanks, in the jitterbug inside to see this repairer is very useful and convenient, received the goods plugged in all normal, repair the phone is not a day or two, rushed for a period of time in order to have the effect, looking forward to.	JD.com
Henri New Energy Restoration	Battery Reconditioner	The real thing is in line with the picture, small and practical. Fast delivery, excellent service delivery.	JD.com
Henri New Energy Restoration Technology	Battery Reconditioner	the4 cell phone charging repair, the effect is quite ideal, which has a cell phone has been unable to charge into the electricity, charging10hours10%of the way, last night with this charging repairer2hours or so on the char98% of thespeed of fast at the same time on the cell phone to pull the garbage cleanup and do not Katun, really straight to buy a good product, the full five-star praise!	JD.com
Henri New Energy Restoration Technology	Battery Reconditioner	[General] cell phone battery repairer received, the product is well made, beautiful appearance, the display of the digital in the constant output changes, while playing a role in the charging inches output voltage regulator, so as to protect the charging of the cell phone battery plays a major role, have a cell phone old iron can have Oh.	JD.com

Henri New Energy Restoration Technology	Battery Reconditioner	Very good product powering up with display pulse powering up	JD.com
Henri New Energy Restoration Technology	Battery Reconditioner	product doesn't restore the battery to its original state in one go you have to keep using it for a while to get better results!	JD.com
Henri New Energy Restoration Technology	Battery Reconditioner	Commodities received, the appearance of the exact same as described, is testing, if indeed the same as the business said, and then follow up, logistics is very fast service attitude is good, give five-star praise.	JD.com
Henri New Energy Restoration Technology	Battery Reconditioner	Fast charging, after fixing it, the phone is much faster and not laggy anymore	JD.com
Henri New Energy Restoration Technology	Battery Reconditioner	Cell phone battery repairer received, logistics is fast, well-packed, compact, easy to use, for repairing cell phone batteries have a greater role.	JD.com
Henri New Energy Restoration Technology	Battery Reconditioner	The goods and the description of[smile]in the charging of the data is constantly changing[smile]logistics is also very fast[smile]customer service is warm and thoughtful[smile]this style is also very beautiful[cool]	JD.com
Henri New Energy Restoration Technology	Battery Reconditioner	Cell phone battery repairer received today, also tried it, the effect is good, worth buying.	JD.com
Henri New Energy Restoration Technology	Battery Reconditioner	Cell phone repairer has received,, fine workmanship,, affordable, on the cell phone charging repair function is very good, the phone is also very good with the packaging is very good, the businessmen shipment fast, logistics speed, on time, service attitude are very good, logistics courier hard work thank you, rated five-pointed star, worth buying, I wish the businessmen to prosper and develop their business.	JD.com
Henri New Energy Restoration Technology	Battery Reconditioner	Sent quickly, well-packed, charging repairer appearance of good workmanship, just when the phone battery is not durable, see if there is no use.	JD.com
Henri New Energy Restoration Technology	Battery Reconditioner	view, found that the outer packaging (box) is intact, the repair device is also very good, no damage phenomenon, as for how to repair the effect, to be used to follow up, very satisfied with a shopping.	JD.com
Henri New Energy Restoration Technology	Battery Reconditioner	Cell phone battery board capacity is insufficient, buy this repairer to repair it, the effect is not clear, but also need to repair a period of time to see the effect, looking forward to, I hope a good result.	JD.com
Henri New Energy Restoration Technology	Battery Reconditioner	Battery Repairer received, well-packaged, good-looking, data at a glance, I hope it can be useful to repair the battery, customer service attitude is very good, logistics is fast.	JD.com
Henri New Energy Restoration Technology	Battery Reconditioner	Yesterday after receiving the goods to test an old cell phone, repair3hoursand 20minutes, I feel that the Internet Internet speed is slightly faster than before, and then continue to repair in two days, two months after the follow-up evaluation.	JD.com
Henri New Energy Restoration Technology	Battery Reconditioner	The goods have been received, the packaging is intact, the product quality is very good. The logistics is very fast, the service attitude is very good	JD.com
Henri New Energy Restoration Technology	Battery Reconditioner	Boss:I ordered to buy a cell phone repair device has been received, the description matches the physical object, the logistics is strong, to see how the effect of the use of the cycle? Favorable comments satisfied5points.	JD.com
Henri New Energy Restoration Technology	Battery Reconditioner	thing, but also feel good play, but also good-looking, ask the customer service attitude is also good, use a few days to evaluate.	JD.com
Henri New Energy Restoration Technology	Battery Reconditioner	I feel that the battery life of the phone has improved significantly, and the power consumption is not so fast when using the phone, and the power change display is very clear when charging, and I will continue to use it in anticipation of getting a better experience! Good review!!!	JD.com
Henri New Energy Restoration Technology	Battery Reconditioner	Received a cell phone battery repairer, asked customer service to use the method according to the instructions began to do repair work, showing repair data in the change, I hope that after5-8weeks to repair my cell phone battery performance is improved, five points.	JD.com
Henri New Energy Restoration Technology	Battery Reconditioner	Baby received, logistics is very fast, well packaged! Tried it in the comments! Repair hand machine works great! Now the phone good not card! Customer service attitude is very very good too powerful! Affordable	JD.com
Henri New Energy Restoration Technology	Battery Reconditioner	It's useless! It's just a current monitor that has no effect at all!proper IQ tax.[Skimming][Skimming][Skimming]	JD.com
Henri New Energy Restoration Technology	Battery Reconditioner	After using it for so long, I personally don't feel it's very useful and I don't recommend it[cool]	JD.com

Henri New Energy Restoration Technology	Battery Reconditioner	Really like it, completely exceeded expectations, shipping very fast, packaging very carefully, strict, fast shipping, very satisfied with a shopping authentic, buy assured, the price is also fair, satisfied!	JD.com
Henri New Energy Restoration	Battery Reconditioner	The logistics is very fast, the business explanation is careful, received the goods are in use!	JD.com
Henri New Energy Restoration	Battery Reconditioner	This user feels the product is better	JD.com
Henri New Energy Restoration	Battery Reconditioner	Restorer received, well packaged, fast shipping, with a certificate, genuine, with a period of time to see the effect, then evaluate.	JD.com
Henri New Energy Restoration	Battery Reconditioner	Very good, fast logistics and excellent service!	JD.com
Henri New Energy Restoration	Battery Reconditioner	It's pretty good. It's finally working.	JD.com
Henri New Energy Restoration	Battery Reconditioner	Baby received, like yo! It's a great platform to get what you need, and good luck to the store!	JD.com
Henri New Energy Restoration Technology	Battery Reconditioner	It's very powerful, very good, very good, it's very smooth to use, and it's fast to charge, so give him credit for that, and please go for it, even though it's a little bit more expensive, and especially good for the batteries	JD.com
Henri New Energy Restoration	Battery Reconditioner	Good quality goods, very good and affordable, logistics is also very fast!	JD.com
Henri New Energy Restoration	Battery Reconditioner	Baby received, the merchant shipment in a timely manner, logistics is also very fast, I hope that there is a door in.	JD.com
Henri New Energy Restoration	Battery Reconditioner	Beautiful appearance, a sense of technology, just charge a night battery capacity than before, the phone is not to be smooth	JD.com
Henri New Energy Restoration	Battery Reconditioner	Restorers that work well, are aesthetically pleasing, and repair with results.	JD.com
Henri New Energy Restoration	Battery Reconditioner	The product arrived in good condition and matched the seller's description	JD.com
Henri New Energy Restoration	Battery Reconditioner	Goods have been received logistics is also very fast packaging intact, the effect of how to use a few days after the follow-up, first give a basic praise!	JD.com
Henri New Energy Restoration	Battery Reconditioner	Good, fast logistics, simple operation, easy to use	JD.com
Henri New Energy Restoration	Battery Reconditioner	It's easy and quick to use! The effect is also very good![Smile][Smile]customer servicealso very good!	JD.com
Henri New Energy Restoration	Battery Reconditioner	Logistics was fast, arrived in three days and used it right away.	JD.com
Henri New Energy Restoration	Battery Reconditioner	Quality can't be beat, just like the store, especially like, and the price is favorable!	JD.com
Henri New Energy Restoration	Battery Reconditioner	The stuff is good, cost-effective, and will continue to support, praise	JD.com
Henri New Energy Restoration	Battery Reconditioner	Goods received, used a period of time before evaluation, is indeed quite good, cell phone battery charging a period of time, the battery with a long time.	JD.com
Henri New Energy Restoration	Battery Reconditioner	I need to use it to know if it's any good	JD.com
Henri New Energy Restoration	Battery Reconditioner	Cell phone repair without asking for a set received, fast logistics, look good.	JD.com
Henri New Energy Restoration	Battery Reconditioner	Things received really good ah workmanship value are very good hope that can play a role	JD.com
Henri New Energy Restoration	Battery Reconditioner	Kudos to the logistics for being so fast despite the epidemic!	JD.com
Henri New Energy Restoration	Battery Reconditioner	The quality is very good, the logistics is very fast, the customer service is very good, it is worth to buy in the next time.	JD.com

Henri New Energy Restoration	Battery Reconditioner	Received the item, just tried it out and will give more review tomorrow.	JD.com
Henri New Energy Restoration	Battery Reconditioner	Beyond time and space super value enjoyment! Great value for money, worth owning and recommending!	JD.com
Henri New Energy Restoration	Battery Reconditioner	Baby received! Will use it to see how it works, just plugged it in and it seems good.	JD.com
Henri New Energy Restoration	Battery Reconditioner	The logistics is also very fast, the service is very good, it is the kind I want.	JD.com
Henri New Energy Restoration	Battery Reconditioner	Fast shipping and very good quality	JD.com
Henri New Energy Restoration	Battery Reconditioner	The baby is very good, as described by the seller, trustworthy.	JD.com
Henri New Energy Restoration	Battery Reconditioner	sent after receipt. 7night after contacting the merchant, overnight replenishment, has received today. Very good service, kudos!	JD.com
Henri New Energy Restoration	Battery Reconditioner	Baby received, logistics God speed. Multiple functions, battery repair. Trendy shape, extremely cool. Dazzling light, more attention.	JD.com
Henri New Energy Restoration	Battery Reconditioner	The cell phone battery is not working, so I'm looking forward to getting good results,[smile]	JD.com
Henri New Energy Restoration	Battery Reconditioner	Logistics is very fast, the battery repairer received, immediately according to the instructions to start charging repair, I hope to have results!	JD.com
Henri New Energy Restoration	Battery Reconditioner	Good value Easy to use Against old cell phones	JD.com
Henri New Energy Restoration	Battery Reconditioner	It works very well, the cell phone battery is more durable after charging, it is worth buying.	JD.com
Henri New Energy Restoration	Battery Reconditioner	It's pretty new, feels pretty good, use it twice and see.	JD.com
Henri New Energy Restoration	Battery Reconditioner	Things received, very delicate, to the phone charging trial a few hours, the effect is good!	JD.com
Henri New Energy Restoration	Battery Reconditioner	Very good, quite good, logistics is very fast, business service is very good	JD.com
Henri New Energy Restoration Technology	Battery Reconditioner	Quality is very good, with the seller's description of the exact same, very satisfied, really like it, completely exceeded expectations, the delivery speed is very fast, the packaging is very careful and strict, the logistics company has a very good service attitude, the delivery speed is very fast, very satisfied with a shopping.	JD.com
Henri New Energy Restoration Technology	Battery Reconditioner	Exquisite design, fine workmanship, compact, good looking and good use, feel very good, fast delivery from the merchant, nice pink color, good workmanship, tight package, good store service, good color.	JD.com
Henri New Energy Restoration	Battery Reconditioner	Great results, very happy with the product	JD.com
Henri New Energy Restoration	Battery Reconditioner	Things match the picture, genuine, affordable, use to see the effect!	JD.com
Henri New Energy Restoration	Battery Reconditioner	Things have been received, logistics is very fast, beautiful appearance, charging stability, worth buying Oh!	JD.com
Henri New Energy Restoration	Battery Reconditioner	charging feels smooth and the battery is noticeably longer. Very satisfied with the shopping, wish the boss a prosperous business!	JD.com
Henri New Energy Restoration	Battery Reconditioner	Big brand, with a very assured, the quality is very good, in time for the event to buy very cost,customer service is really very good Oh ~!	JD.com
Henri New Energy Restoration	Battery Reconditioner	Baby received can not wait to try, feel good, look forward to the effect!	JD.com
Henri New Energy Restoration	Battery Reconditioner	Restorer has received, fast shipping, quality is quite good, and the seller describes the same, very satisfied, with a period of time to see how the results	JD.com
Henri New Energy Restoration	Battery Reconditioner	First-class quality, packaging is also good, logistics is very fast, and the price is reasonable, very affordable, I like it!	JD.com

Henri New Energy Restoration	Battery Reconditioner	Received the item and tried it out. The quality is very good!	JD.com
Henri New Energy Restoration	Battery Reconditioner	Logistics is very fast, packaging is very good, product quality is very good, is genuine, worth buying!	JD.com
Henri New Energy Restoration	Battery Reconditioner	The baby has been received, the logistics is very fast, the seller's service is very good!	JD.com
Henri New Energy Restoration	Battery Reconditioner	Fast shipping, received easy to use to extend battery life and standby time, this product praise!	JD.com
Henri New Energy Restoration	Battery Reconditioner	Fast logistics, baby is very good, charging fast, can fix the battery is better, in the use of the results after seeing.	JD.com
Henri New Energy Restoration	Battery Reconditioner	Available!	JD.com
Henri New Energy Restoration	Battery Reconditioner	Beautiful appearance, fine workmanship, and good effect of use	JD.com
Henri New Energy Restoration Technology	Battery Reconditioner	with other chargers charging is very slow, go to the customer service repair people also said that the model is too aging not that, in addition to the charging port is bad charging slow no other problems, with this charging I feel faster still other still in use, do not comment, the current feeling is good	JD.com
Henri New Energy Restoration	Battery Reconditioner	Received looked good, the effect of the first use to be observed should be able to.	JD.com
Henri New Energy Restoration	Battery Reconditioner	Clever design, small things that do a lot, love it!	JD.com
Henri New Energy Restoration	Battery Reconditioner	Baby received, is charging, really hope to repair the battery, praise!	JD.com
Henri New Energy Restoration	Battery Reconditioner	High-end atmosphere, good effect, voltage display, current display, at a glance	JD.com
Henri New Energy Restoration	Battery Reconditioner	Goods have been received, packaging is very good, deliberately use2days before evaluation, indeed with the business description of the same, effective, good.	JD.com
Henri New Energy Restoration	Battery Reconditioner	This baby is very good to use, to the phone charging is very smooth, but also often the battery life, full of since the booth, very satisfactory worth five stars![Big Love]	JD.com
Henri New Energy Restoration	Battery Reconditioner	Good to see see see see so much use of brands	JD.com
Henri New Energy Restoration	Battery Reconditioner	Has arrived, the effect of performance is quite good and very chic feel quite good worth going to buy	JD.com
Henri New Energy Restoration	Battery Reconditioner	Cell phone battery repairer open the package immediately try, very good, the effect is good good to make, the future need will come back to buy.	JD.com
Henri New Energy Restoration	Battery Reconditioner	Battery Repairer received,, the logistics is very fast, and as described,.	JD.com
Henri New Energy Restoration	Battery Reconditioner	logistics is fast, color digital display, beautiful and exquisite, repair batteries, more durable, thanks to the business.	JD.com
Henri New Energy Restoration	Battery Reconditioner	Look at the quality is good, exquisite workmanship, fast charging, digital display is clear, it is worth buying, the back of the old phone to see the effect of using.	JD.com
Henri New Energy Restoration	Battery Reconditioner	Would recommend friends to buy,, looks good, feels good, works well. Received the baby, logistics express fast. Good service. Stick drop!	JD.com
Henri New Energy Restoration	Battery Reconditioner	Repairer received, has been used for a few days, charging fast, old phone charging is not hot, and then use some days to give you a report on the use of it.	JD.com
Henri New Energy Restoration	Battery Reconditioner	The battery restorer has been received, looks high-end atmosphere, charging speed is also very fast.	JD.com
Henri New Energy Restoration	Battery Reconditioner	using the repair battery device when the phone is hot improved, to be used a few more times to come back to the review.	JD.com
Henri New Energy Restoration	Battery Reconditioner	Very well packaged, fast shipping, top quality, nice good seller	JD.com

Henri New Energy Restoration	Battery Reconditioner	Bought the set, a battery restorer, a charging cable (head), well packaged, the restorer is chic.	JD.com
Henri New Energy Restoration	Battery Reconditioner	Fast logistics, received the goods the next day, very satisfied with the great!	JD.com
Henri New Energy Restoration Technology	Battery Reconditioner	Intelligent Fixer Receive Liu, the physical object is consistent with the description of the advertisement, the appearance is beautiful and cool, and the feel is good. After the order, the logistics is fast.	JD.com
Henri New Energy Restoration	Battery Reconditioner	Cell phone battery repairer received, logistics is very fast, looking very good very like. A very satisfactory online shopping. Wish the boss a prosperous business	JD.com
Henri New Energy Restoration	Battery Reconditioner	Things received, the appearance of the feel of the quality is very good, the use of simple methods, the effect is good, the cell phone battery repair has improved, worth having.	JD.com
Henri New Energy Restoration	Battery Reconditioner	Small and convenient, full of technology, excellent performance and good conscience![Kudos]	JD.com
Henri New Energy Restoration	Battery Reconditioner	Well made and easy to use. Repair function is good, worth buying. Five stars praise.	JD.com
Henri New Energy Restoration	Battery Reconditioner	This cell phone battery repairer has fine workmanship, accurate tree data, there is a gradual recovery effect, recommended purchase	JD.com
Henri New Energy Restoration	Battery Reconditioner	detailed operating instructions, the boss has a very good attitude, patiently answer the questions raised, logistics fast.	JD.com
Henri New Energy Restoration	Battery Reconditioner	The logistics is fast and the store service is on point, answering questions carefully.	JD.com
Henri New Energy Restoration	Battery Reconditioner	Good price, fine workmanship, repair battery function is good, worth buying.	JD.com
Henri New Energy Restoration	Battery Reconditioner	Goods received, logistics is very fast, good-looking, high cost-effective, good quality, this time do not have to change the battery, thanks to the business.	JD.com
Henri New Energy Restoration	Battery Reconditioner	Very fast delivery and great service!	JD.com
Henri New Energy Restoration	Battery Reconditioner	Just used it once and the next day the phone was a lot smoother. Customer service is very good and explains very clearly	JD.com
Henri New Energy Restoration Technology	Battery Reconditioner	more than two hundredGdata, to restore the factory value of the way to repair too much trouble. Can only use the most tube convenient shutdown many times a long time to charge the way to repair, in the end, how effective, can only after a period of time to evaluate it, I hope to be able to describe the same effect.	JD.com
Henri New Energy Restoration	Battery Reconditioner	Bought back and used, easy to operate, charging is also fast, in a few days and then look at the battery repair situation	JD.com
Henri New Energy Restoration	Battery Reconditioner	Fast logistics Well-packed Looks great Authentic I'll comment after I use it	JD.com
Henri New Energy Restoration Technology	Battery Reconditioner	Purposefully received the goods four or five days before the evaluation, the repairer charging stability, the battery has a certain repair, the phone is also smoother than the other day, play time is much longer than the previous time	JD.com
Henri New Energy Restoration Technology	Battery Reconditioner	Received, very satisfied from the appearance and workmanship, and the business description is consistent. Has begun to use, to be used after the follow-up comment, to give you a real feeling of use[shake hands]	JD.com
Henri New Energy Restoration Technology	Battery Reconditioner	ten dollars or so, the whole repair process is very gentle, the charger and the phone did not heat up, only the pulse data is jumping. Feel the quality is very good, cost-effective, logistics and delivery is very fast, the seller's service is very good.	JD.com
Henri New Energy Restoration Technology	Battery Reconditioner	Used for a period of time, at first I used it as a charger, did not follow the instructions as such with the appearance of the message does not support accessories, I thought it was broken, the boss patiently explained to me that it can not be used as a charger, you need to three days at a time for three hours so that it works! I try and then follow up! I also gave my blood pressure meter battery repair, did not think that surprisingly battery repair is good indeed good worth having!	JD.com

Henri New Energy Restoration Technology	Battery Reconditioner	To be honest, this repair agent back, there are three or four days, this shopkeeper is also quite good attitude, do not understand the place with him, he taught me a little bit, I wish him a prosperous business!	JD.com
Henri New Energy Restoration	Battery Reconditioner	Logistics is also very fast, genuine, fine workmanship, function as the anchor introduction is true, repair works!	JD.com
Henri New Energy Restoration Technology	Battery Reconditioner	Battery Repairer received, used for a few days suburb fruit is very good, my cell phone was full of electricity can only be used for three hours, the use of this repair device can be used for a day after charging for an hour, suburb fruit magical, worth buying, thanks to the business good product!	JD.com
Henri New Energy Restoration Technology	Battery Reconditioner	Cell Phone Battery Repair Magic, Universal Android Apple Tablet Extended Battery Life, this repair magic with quite good, charged a few times with the effect of a few times, the appearance of a beautiful and beautiful, the quality is also good, the logistics is fast customer service attitude is good.	JD.com
Henri New Energy Restoration	Battery Reconditioner	also does not close, baby received on the trial, feel well-made, easy to use, small and exquisite. Is the old cell phone old battery protector.	JD.com
Henri New Energy Restoration Technology	Battery Reconditioner	Cell phone battery repairer has been received, the packaging is very good, the repairer is very stylish, the cell phone plugged into it to charge, the cell phone power saving effect is really much better, a very satisfactory shopping.	JD.com
Henri New Energy Restoration Technology	Battery Reconditioner	Finally received the baby I need, the stuff is very good, the price is good and cheap, thanks to!The owner's attitude is very professional and enthusiastic, ask questions and reply quickly, I asked a lot of questions, he does not feel annoyed, they will seriously answer me. And then say baby, just what I need, when I received the packaging is complete, after opening the surprise, baby than I imagined even better!Had to have to give a thumbs up. I will come back next time I need it!	JD.com
Henri New Energy Restoration	Battery Reconditioner	Exquisite packaging, design texture, fast delivery, with detailed instructions for use in English and Chinese.	JD.com
Henri New Energy Restoration	Battery Reconditioner	Very good, received the goods on the use, than I previously bought a lot stronger, recommended to buy Oh!	JD.com
Henri New Energy Restoration	Battery Reconditioner	It's good. I'll comment after I use it.	JD.com
Henri New Energy Restoration	Battery Reconditioner	Very good. The measurements are good.	JD.com
Henri New Energy Restoration	Battery Reconditioner	Repairer has received, very delicate, the phone is good again, worth buying!	JD.com
Henri New Energy Restoration	Battery Reconditioner	It's good. It works.	JD.com
Henri New Energy Restoration	Battery Reconditioner	Use it and evaluate.	JD.com
Henri New Energy Restoration	Battery Reconditioner	Already received it thanks a lot[smile]	JD.com
Henri New Energy Restoration	Battery Reconditioner	Especially especially satisfied, super nice, customer service is very polite, very polite, conscientious business, support!	JD.com
Henri New Energy Restoration	Battery Reconditioner	Deeply cleans your phone's memory junk, improves your phone's speed, and increases your phone's battery life.	JD.com
Henri New Energy Restoration	Battery Reconditioner	Cell phone repair battery capacity rechargeable stability function is powerful, full of automatic power-off	JD.com
Henri New Energy Restoration	Battery Reconditioner	an excellent	JD.com
Henri New Energy Restoration	Battery Reconditioner	Fine workmanship, but the effect is yet to be known after use	JD.com
Henri New Energy Restoration	Battery Reconditioner	Unused products, use]to know the effect	JD.com
Henri New Energy Restoration Technology	Battery Reconditioner	also particularly fast, while the phone does not heat up, a lot better than the products I purchased elsewhere, the shopkeeper's service is enthusiastic and thoughtful and meticulous, trustworthy products and businesses. Next time will continue to cooperate.	JD.com

Henri New Energy Restoration	Battery Reconditioner	Goods received.	JD.com
Henri New Energy Restoration	Battery Reconditioner	Express received, phone charging with lights to show math, phone charging fast	JD.com
Henri New Energy Restoration	Battery Reconditioner	It's at home.	JD.com
Henri New Energy Restoration	Battery Reconditioner	I love this product and it works great!	JD.com
Henri New Energy Restoration	Battery Reconditioner	Compact and easy to use, yet portable	JD.com
Henri New Energy Restoration	Battery Reconditioner	Great style, good quality, awesome!	JD.com
Henri New Energy Restoration	Battery Reconditioner	The restorer has been received and is being used to finally see how well it works before following up.	JD.com
Henri New Energy Restoration	Battery Reconditioner	Received the product used, the effect is really good, good worth repurchase again!	JD.com
Henri New Energy Restoration	Battery Reconditioner	I haven't used it yet. I'll comment afterward.	JD.com
Henri New Energy Restoration	Battery Reconditioner	The goods received, small and exquisite, logistics is also very fast,,, packaging is very good.	JD.com
Henri New Energy Restoration	Battery Reconditioner	The goods have been received I love it next time I will buy from you again.	JD.com
Henri New Energy Restoration	Battery Reconditioner	Beautiful and practical	JD.com
Henri New Energy Restoration	Battery Reconditioner	Goods have been received, do not know how to use it, just asked the customer service, first use after evaluation	JD.com
Henri New Energy Restoration	Battery Reconditioner	Good service attitude. Logistics is fast.	JD.com
Henri New Energy Restoration	Battery Reconditioner	Look really good, and the root of the host speak the same, good service, fast logistics, quality is very good.	JD.com
Henri New Energy Restoration	Battery Reconditioner	It's a great product and the logistics are fast, thanks for buying it!	JD.com
Henri New Energy Restoration	Battery Reconditioner	The item is so good, I like this item the most, it is very excellent and the quality is also very good.	JD.com
Henri New Energy Restoration	Battery Reconditioner	The quality of the product is very good worth repurchasing	JD.com
Henri New Energy Restoration	Battery Reconditioner	Goods have been received thanks, the courier is very fast, things are very good, five stars, good service!	JD.com
Henri New Energy Restoration	Battery Reconditioner	It works.	JD.com
Henri New Energy Restoration	Battery Reconditioner	Received the shipment and it works fine! Works great! Express delivery is also very powerful!	JD.com
Henri New Energy Restoration	Battery Reconditioner	Kudos. Kudos. Kudos.	JD.com
Henri New Energy Restoration	Battery Reconditioner	goods received, and cell phone charging trial a few days, the effect of the bar, the phone is not lagging, the network speed is very good!	JD.com
Henri New Energy Restoration	Battery Reconditioner	give5stars first, I'll follow up after I've used it	JD.com
Henri New Energy Restoration	Battery Reconditioner	Excellent service Fast delivery Works	JD.com
Henri New Energy Restoration	Battery Reconditioner	Okay.	JD.com

Henri New Energy Restoration	Battery Reconditioner	Good stuff worth buying 5 stars	JD.com
Henri New Energy Restoration	Battery Reconditioner	received	JD.com
Henri New Energy Restoration	Battery Reconditioner	Great style, top quality, awesome![Kudos]	JD.com
Henri New Energy Restoration	Battery Reconditioner	The goods have been received and the logistics are fast.	JD.com
Henri New Energy Restoration	Battery Reconditioner	This is a really good restorer, straight up buy!	JD.com
Henri New Energy Restoration	Battery Reconditioner	Received the goods, especially like, logistics is very fast	JD.com
Henri New Energy Restoration	Battery Reconditioner	Goods received, very good, inexpensive and beautiful, used the phone does not heat up, courier force, thank you boss shipping fast.	JD.com
Henri New Energy Restoration	Battery Reconditioner	Buy again if it works well during use. Great service from the drop shipper We are all happy with it.	JD.com
Henri New Energy Restoration Technology	Battery Reconditioner	Repair received, strong packaging, fast logistics, do not know how to use it is not good, waiting to use again, if it is good, I will ask my friends to your home to buy, wish the boss a prosperous business, muah da!	JD.com
Henri New Energy Restoration	Battery Reconditioner	The product is fine and works well	JD.com
Henri New Energy Restoration	Battery Reconditioner	The product is great and I love it!	JD.com
Henri New Energy Restoration	Battery Reconditioner	Goods received good quality quite good customer service attitude is very good	JD.com
Henri New Energy Restoration	Battery Reconditioner	The merchant shipped quickly and the courier service was great. I love it, I personally love it. friends order soon.	JD.com
Henri New Energy Restoration	Battery Reconditioner	(of an unmarried couple) be close	JD.com
Henri New Energy Restoration	Battery Reconditioner	This is beautiful and practical, love it!	JD.com
Henri New Energy Restoration	Battery Reconditioner	Good quality, fast logistics	JD.com
Henri New Energy Restoration	Battery Reconditioner	Very nice. It works.	JD.com
Henri New Energy Restoration	Battery Reconditioner	Contact sensitivity: good, Exterior material: beautiful, Charging speed: very fast Delivery: door-to-door, very good attitude!	JD.com
Henri New Energy Restoration Technology	Battery Reconditioner	From the packaging of the product, to the texture of the product in my hand, I think it's a great product. Look at its work seems to be balancing the current! Should be a good product. Later in the supplement battery repair effect it!	JD.com
Henri New Energy Restoration	Battery Reconditioner	Logistics was also very strong! Received the item very quickly! First give a favorable comment! Other use and then evaluate!	JD.com
Henri New Energy Restoration	Battery Reconditioner	The quality of workmanship is exquisite	JD.com
Henri New Energy Restoration	Battery Reconditioner	The charging effect is OK	JD.com
Henri New Energy Restoration	Battery Reconditioner	There is value in using Convenient Good to use !	JD.com
Henri New Energy Restoration	Battery Reconditioner	It's working. The batteries do last longer than before.	JD.com
Henri New Energy Restoration	Battery Reconditioner	Charging speed: very, very fast. It's the same as the seller's description. It's really a black technology, it's breathtaking.	JD.com

Henri New Energy Restoration	Battery Reconditioner	Exquisite packaging, design texture, fast delivery, with detailed instructions for use in English and Chinese.	JD.com
Henri New Energy Restoration	Battery Reconditioner	Charging is steady, and you can also take care of your cell phone battery without hurting your phone.	JD.com
Henri New Energy Restoration Technology	Battery Reconditioner	Finally received the baby I need, the stuff is very good, the boss attitude is very professional and enthusiastic, ask questions and reply quickly, I asked a lot of questions, he does not feel annoyed, they will seriously answer me.	JD.com
Henri New Energy Restoration	Battery Reconditioner	Used a few days, now the phone battery is also much more durable, very good product, recommended!	JD.com
Henri New Energy Restoration Technology	Battery Reconditioner	This product has been tried. Tried three old cell phones and felt like they opened and surfed the web faster than they did. Now trying an old laptop to see how it works. The product does not fail to deliver.	JD.com
Henri New Energy Restoration	Battery Reconditioner	Baby safe delivery, packaging is quite good, first try to use, do not know how the effect habit of praise!	JD.com
Henri New Energy Restoration	Battery Reconditioner	The restorer charges steadily, has fixed the battery somewhat, and the phone is smoother than it was a few days ago, and plays much longer than it did before time	JD.com
Henri New Energy Restoration	Battery Reconditioner	The lens is also protected by a raised design, and it's easy to remove and install, so I'm very satisfied!	JD.com
Henri New Energy Restoration	Battery Reconditioner	Works well charging process doesn't burn your hands anymore. Effective and noticeable! Thanks for the kudos!	JD.com
Henri New Energy Restoration	Battery Reconditioner	Baby received, very good, satisfied!	JD.com
Henri New Energy Restoration	Battery Reconditioner	The store shipped very quickly, baby received after the trial, feel well-made, easy to use, small and exquisite. It is the protector of old cell phones and old batteries.	JD.com
Henri New Energy Restoration Technology	Battery Reconditioner	Tried it for a few days before coming to evaluate, the service is very good. My old cell phone could not be used for an hour, after using this restorer once it really works, now it can be used for more than two hours!	JD.com
Henri New Energy Restoration Technology	Battery Reconditioner	thank you! As described in the workmanship looks good rules standard! The effect of the test charging in the future to evaluate it! Satisfaction! Thank you for the speed of delivery, I wish the boss business is getting better and better, health and happiness!	JD.com
Henri New Energy Restoration	Battery Reconditioner	Only after using it to evaluate, the appearance is exquisite, charging speed is very fast, satisfied with the	JD.com
Henri New Energy Restoration	Battery Reconditioner	Logistics is very fast, product quality can be, satisfied	JD.com
Henri New Energy Restoration Technology	Battery Reconditioner	charging can always monitor the charging voltage, current, according to the instructions, there is a clear effect of repairing the battery. Good customer service and fast logistics.	JD.com
Henri New Energy Restoration	Battery Reconditioner	Extraordinarily good restorers, the quality of workmanship can't be beat, very delicate	JD.com
Henri New Energy Restoration	Battery Reconditioner	Logistics is very fast, and the power off and on charge, really effective, full of boot	JD.com
Henri New Energy Restoration	Battery Reconditioner	In use, the display is normal, now with the store description of the match, friends have the effect before buying, with a longer time than before	JD.com
Henri New Energy Restoration	Battery Reconditioner	The battery adapter works, the battery now lasts longer than before and playing games is much smoother!	JD.com
Henri New Energy Restoration	Battery Reconditioner	The results are much better than what you can buy at other places!	JD.com
Henri New Energy Restoration Technology	Battery Reconditioner	Bought cell phone battery repairer received more**, have been using, the effect is good, the seller's service is hospitable, after receiving do not know how to use the seller also sent a detailed description, trustworthy business.	JD.com
Henri New Energy Restoration	Battery Reconditioner	Cell phone battery repair device has been received, with this repair device on the cell phone battery repair comparative	JD.com
Henri New Energy Restoration	Battery Reconditioner	Fast shipping, good quality Contact s:good sensitivity Appearance material:comfortable feel Charging speed:very fast	JD.com

Henri New Energy Restoration	Battery Reconditioner	Repairer shipment received. Going to try it on an unused phone to see how it works.	JD.com
Henri New Energy Restoration Technology	Battery Reconditioner	Battery Repairer received, fast logistics, the use of a period of time to evaluate, the effect is very good, the battery use time increased significantly a lot, in accordance with the instructions required by the shutdown repair effect, without interference, the effect can reach more than ninety percent!	JD.com
Henri New Energy Restoration Technology	Battery Reconditioner	Cell phone battery repairer after hours of charging and on a cell phone that can not be charged into the repair, a few hours to repair the success, kudos! Commodity description is consistent, logistics is so fast, worth buying!	JD.com
Henri New Energy Restoration	Battery Reconditioner	Used for a period of time, from the appearance and workmanship is very satisfied, and the business description-consistent, have effect, and now charging has not been hot	JD.com
Henri New Energy Restoration Technology	Battery Reconditioner	durable, by cheap recommended to buy this fixer, did not expect to use cell phone is indeed smooth the battery feels also durable to, do not have to always take the charging treasure, very satisfied with an online shopping , there is a need to also come to buy.	JD.com
Henri New Energy Restoration Technology	Battery Reconditioner	Cell phone battery repairer received, looked good, as described, there are instructions have the use of methods, plugged in the words of the repair, I hope the effect is obvious, or to give five-star praise.	JD.com
Henri New Energy Restoration	Battery Reconditioner	phone battery repairer after repairing the cell phone battery is obviously faster than before when charging, logistics is also very fast.	JD.com
Henri New Energy Restoration Technology	Battery Reconditioner	This cell phone battery repairer was received very quickly! Its quality, really good! This baby, its appearance, exquisite production, fashionable technology, small and beautiful, convenient and good use! Efficient top quality, repair effect, worth looking forward to and buying!	JD.com
Henri New Energy Restoration	Battery Reconditioner	Baymax received, used the effect is still very good, the appearance of the material is also very good	JD.com
Henri New Energy Restoration	Battery Reconditioner	I got it. Try it.	JD.com
German science and technology	Battery Repairer	This thing is still a problem, fully charged a value does not extinguish the light, and the description of the goods is not the same, I do not know the battery should have a response no	JD.com
German science and technology	Battery Repairer	It's the wrong one, but it works.	JD.com
German science and technology	Battery Repairer	I've been using it for almost a month now It doesn't work at all Not recommended	JD.com
German science and technology	Battery Repairer	This user feels the product is better	JD.com
German science and technology	Battery Repairer	Feel no effect, charged twice, are open less than two days on the powerless, a little use is useless!	JD.com
German science and technology	Battery Repairer	This user feels the product is better	JD.com
German science and technology	Battery Repairer	Fully charged, still green light, won't go out	JD.com
German science and technology	Battery Repairer	Goods have received the quality of the effect are quite very satisfied must give a five-star praise!	JD.com
German science and technology	Battery Repairer	This user feels the product is better	JD.com
German science and technology	Battery Repairer	This user feels the product is better	JD.com
German science and technology	Battery Repairer	Used it for the second time did not experience the battery repair, I hope to use it a few more times for the repair.	JD.com
German science and technology	Battery Repairer	I'll comment after using it.	JD.com
German science and technology	Battery Repairer	I don't know if it's good or not. It doesn't feel good.	JD.com
German science and technology	Battery Repairer	This user feels the product is better	JD.com
German science and technology	Battery Repairer	This user feels the product is better	JD.com
German science and technology	Battery Repairer	This user feels the product is better	JD.com
German science and technology	Battery Repairer	then out of the car is very obvious that the power increased a lot.	JD.com
German science and technology	Battery Repairer	This user feels the product is better	JD.com
German science and technology	Battery Repairer	This user feels the product is better	JD.com
German science and technology	Battery Repairer	also very fast, repair can be, first use a period of time to follow up.	JD.com

German science and technology	Battery Repairer	can	JD.com
German science and technology	Battery Repairer	the fake goods are here, I was fooled. I have asked the master to fix it, but the wiring was burnt out. I want to return the product. I can't find the return address in the logistics, one is a fake loan, one is a fake factory, please Pinduoduo give me an understanding.	JD.com
German science and technology	Battery Repairer	Use normal, do not know whether there is a repair will have to be used at a later stage to be able to draw conclusions.	JD.com
German science and technology	Battery Repairer	Electrical quality is very good, logistics is very fast, the next time will buy, I hope you come!	JD.com
German science and technology	Battery Repairer	Baby received, this electric car battery, the retractor can be, easy to connectors, logistics is very fast, inexpensive, value for money, I like it, very satisfied.	JD.com
German science and technology	Battery Repairer	Battery activator. It's been in the house for a few days now. Works great. Thanks again to customer service and the owner.	JD.com
German science and technology	Battery Repairer	This user feels the product is better	JD.com
German science and technology	Battery Repairer	We'll see how it works tomorrow when it's charged.	JD.com
German science and technology	Battery Repairer	[big love][big love][]biglove[big][biglove]	JD.com
German science and technology	Battery Repairer	Working on it, waiting for results	JD.com
German science and technology	Battery Repairer	Goods to receive, fast, I opened it with the same as said on the Internet, very satisfied.	JD.com
German science and technology	Battery Repairer	Little baby to receive, the effect of how do not know first give a favorable comment on it!	JD.com
German science and technology	Battery Repairer	This user feels the product is better	JD.com
German science and technology	Battery Repairer	This user feels the product is better	JD.com
German science and technology	Battery Repairer	I don't see any effect now, I'll use it for a while and then come back to the flat price	JD.com
German science and technology	Battery Repairer	This user feels the product is better	JD.com
German science and technology	Battery Repairer	Very fast logistics, fast delivery, good service from the manufacturer. The new superwill received only used once, can not see the effect for the time being, may need to repair a few times to get the effect, please ask the manufacturer how long it needs to be repaired at a time is best.	JD.com
German science and technology	Battery Repairer	Looking forward to the effect	JD.com
German science and technology	Battery Repairer	This user feels the product is better	JD.com
German science and technology	Battery Repairer	This user feels the product is better	JD.com
German science and technology	Battery Repairer	This user feels the product is better	JD.com
German science and technology	Battery Repairer	poor evaluation	JD.com
German science and technology	Battery Repairer	I'm trying it out, and Iwon't knowthe results until I've used itfor 2days!	JD.com
German science and technology	Battery Repairer	as advertised any battery can be used, the voltage is 36V-96V,12Vcan not be used. In addition the product also need to match their own link cable, before all are not aware of it is no way to try, whether it is good or not is not clear. Lazy to return the product to deal with.<br>Boss delivery is still quite fast. Items were squeezed and smashed deformation, good space in the inner box, no big problem.	JD.com
German science and technology	Battery Repairer	Very dissatisfied, just received in accordance with the instructions to operate the power on the smoke, burned, can not be used. Can such a product be used?	JD.com
German science and technology	Battery Repairer	Baby received, have not yet used, habitually praise it	JD.com
German science and technology	Battery Repairer	Received in use has no effect in the future to know and then praise!	JD.com
German science and technology	Battery Repairer	Boss pulse repair charger received the goods, I'm too satisfied happy, especially to recommend you my friends who have battery cars, to give you five red heart, I hope to keep the quality and quantity you have a good product.	JD.com
German science and technology	Battery Repairer	Logistics is fast, packaging is very good, the restorer is good, please buy if you need.	JD.com
German science and technology	Battery Repairer	I'll give it a 5-star review first, and then I'll comment after I use it	JD.com
German science and technology	Battery Repairer	This user feels the product is better	JD.com

German science and technology	Battery Repairer	This user feels the product is better	JD.com
German science and technology	Battery Repairer	Starting to use it, hope it works	JD.com
German science and technology	Battery Repairer	This user feels the product is better	JD.com
German science and technology	Battery Repairer	Express is not like the packaging are broken not star side packaging are broken.... The good thing is that the product is not broken.	JD.com
German science and technology	Battery Repairer	an excellent	JD.com
German science and technology	Battery Repairer	This user feels the product is better	JD.com
German science and technology	Battery Repairer	The models are not the same.	JD.com
German science and technology	Battery Repairer	It tookmore than10hoursrepair, I can't feel the effect after using it once, the merchant said that it takes a month to repair before I can see the effect..	JD.com
German science and technology	Battery Repairer	This user feels the product is better	JD.com
German science and technology	Battery Repairer	I haven't used it yet.	JD.com
German science and technology	Battery Repairer	The mouth can be right, but it doesn't work?	JD.com
German science and technology	Battery Repairer	Goods have been received to see the effect after use, first give a five-star praise before.	JD.com
German science and technology	Battery Repairer	Your electric car intelligent pulse regulator goods have been received logistics and your service attitude is very good.	JD.com
German science and technology	Battery Repairer	This user feels the product is better	JD.com
German science and technology	Battery Repairer	Bad review, bought it and it doesn't work	JD.com
German science and technology	Battery Repairer	evaluation after use	JD.com
German science and technology	Battery Repairer	use, fully repair the battery, value for money, give you praise.	JD.com
German science and technology	Battery Repairer	Just received, used once, the effect is not obvious, to be used for a period of time to evaluate.	JD.com
German science and technology	Battery Repairer	This user feels the product is better	JD.com
German science and technology	Battery Repairer	There's no way to use it without a special adapter.	JD.com
German science and technology	Battery Repairer	This user feels the product is better	JD.com
German science and technology	Battery Repairer	The restorer was received well packaged hopefully it will work satisfied with 5 stars	JD.com
German science and technology	Battery Repairer	Fast logistics, good service, very satisfied with a shopping.	JD.com
German science and technology	Battery Repairer	This user feels the product is better	JD.com
German science and technology	Battery Repairer	Finally found a good store, good service, good quality, next time I have a chance to buy again.	JD.com
German science and technology	Battery Repairer	same, very satisfied,really like, completely exceeded expectations, the delivery speed is very fast, the packaging is very careful and strict, the logistics company has a very good service attitude, the delivery speed is very fast, very satisfied with a purchase	JD.com
German science and technology	Battery Repairer	Very happy with the purchase!	JD.com
German science and technology	Battery Repairer	Great price, great service, nice stuff, great customer service!	JD.com
German science and technology	Battery Repairer	Shipping was very fast!	JD.com
German science and technology	Battery Repairer	Finally received the baby I need, the stuff is very good, the price is good and cheap, thanks to the shopkeeper! Honestly, this is the most satisfying shopping I've ever done in my Poundland shopping. Whether it's the shopkeeper's attitude or the item, I'm very satisfied. Cabinet attitude is very professional and enthusiastic, ask questions, reply quickly, I asked a lot of questions, he does not feel annoyed, will seriously answer me, this point I express my heartfelt respect to the cabinet, such a good cabinet can not be more!	JD.com
German science and technology	Battery Repairer	90This has gone on my unlimited repurchase list!	JD.com
German science and technology	Battery Repairer	Just unpacked it and really happy with it, highly recommended!	JD.com
German science and technology	Battery Repairer	Very happy with the purchase!	JD.com

German science and technology	Battery Repairer	I received it today and I'm very happy with it! Remember to get a discount next time you come!	JD.com
German science and technology	Battery Repairer	The owner was really nice and shipped quickly. The quality of the item is also quite good. Love it so much, thanks!	JD.com
German science and technology	Battery Repairer	I'm going to give a good review……review until everyone sees it!	JD.com
German science and technology	Battery Repairer	Baby received, very satisfied, come back in the future.	JD.com
German science and technology	Battery Repairer	owner is good, warm service, fast delivery, next time will come to your home, will also recommend others to your home, full five praise!!!!	JD.com
German science and technology	Battery Repairer	Baby received! Very unexpected, will be back next time~great seller, stay tuned!	JD.com
German science and technology	Battery Repairer	The store is very reputable, and it's great to buy things from this store.	JD.com
German science and technology	Battery Repairer	Very happy with the purchase!	JD.com
German science and technology	Battery Repairer	Who says you can't get good stuff at Poundland!	JD.com
German science and technology	Battery Repairer	I have no hesitation in bookmarking the seller's store.	JD.com
German science and technology	Battery Repairer	Very good seller, very enthusiastic ah, next time will come back~baby is very good!	JD.com
German science and technology	Battery Repairer	Very satisfied,really like it, completely exceeded expectations.	JD.com
German science and technology	Battery Repairer	This has gone on my infinite repurchase list!	JD.com
German science and technology	Battery Repairer	Arrived very quickly, good quality, good store, recommended~	JD.com
German science and technology	Battery Repairer	I am thankful that Hades made me come across this store!	JD.com
German science and technology	Battery Repairer	Compared several, really not disappointed, very good quality, very detailed workmanship, very satisfied, super like.	JD.com
German science and technology	Battery Repairer	The quality is excellent and exactly as described by the seller!	JD.com
German science and technology	Battery Repairer	Anyone who says it's bad I want to spell it out!	JD.com
German science and technology	Battery Repairer	The logistics company has a great service and fast delivery!	JD.com
German science and technology	Battery Repairer	The delivery was fast, the guy was polite and didn't mind waiting a few extra minutes for me.	JD.com
German science and technology	Battery Repairer	After the receipt of things immediately check, found with the picture description consistent, super like, sellers shipping speed, service is also very in place, to the boss point a praise, the next time will come to buy.	JD.com
German science and technology	Battery Repairer	This is one of the most satisfying purchases I've made since I've been shopping.	JD.com
German science and technology	Battery Repairer	The packaging is very careful and tight!	JD.com
German science and technology	Battery Repairer	This store is okay. Came to buy a few times, very good service to regular customers, and will come back often!	JD.com
German science and technology	Battery Repairer	Very patient seller, will repurchase if needed in the future.	JD.com
German science and technology	Battery Repairer	Shipping was very fast!	JD.com
German science and technology	Battery Repairer	Great seller, so patient, I finally got what I wanted. Thanks to the seller.	JD.com
German science and technology	Battery Repairer	worthwhile. Not too early, not too late, just the right time to meet. So I stopped and waited for this baby to give you something to look forward to, time will not fail to wait. This world, the only way to have love is to exchange sincerity for sincerity. Those sweet words, fame, fortune and vain promises are no match for a profound affirmation. Simple and ordinary words are enough to warm a whole winter. The corners of my mouth must be upturned right now, with deep dimples floating on my cheeks. So beautiful smile, must be to receive this baby let me unconsciously born. ve bookmarked the store,soI hope you'll be happy and happy every day when you see this comment.	JD.com
German science and technology	Battery Repairer	Such a great seller, don't want to share!	JD.com
German science and technology	Battery Repairer	The answer to the questions is relentless, detailed and serious, the key is good stuff, and the goods are sent super fast, carefully packaged, and trustworthy!	JD.com
German science and technology	Battery Repairer	Online shopping is so intense, I didn't realize that the store's service is so good, the goods are of good quality but inexpensive, I thank you so much!	JD.com
German science and technology	Battery Repairer	storebaifamily is very credible,duand very good oh, in this store to buy things,zhiI am very satisfied!	JD.com

German science and technology	Battery Repairer	I was in a good mood when I came to the live stream and found my favorite treasure!	JD.com
German science and technology	Battery Repairer	Receivedbaigoods, the first time to unpack, feel thequality is still relatively gooddu	JD.com
German science and technology	Battery Repairer	There is no question of exaggeration or deception.	JD.com
German science and technology	Battery Repairer	All in all, a really satisfying shopping experience yet.	JD.com
German science and technology	Battery Repairer	Really hype and love it, totally exceeded expectations.	JD.com
German science and technology	Battery Repairer	First of allbaiquality, I really super like it	JD.com
German science and technology	Battery Repairer	Family and friends also say it is good. If you like it, just chop your hand, you won't regret it.	JD.com
German science and technology	Battery Repairer	protect very well, not a bit of damage, packaging is very tight, shipping is also quite fast, thank you thank you, thank you very much!	JD.com
German science and technology	Battery Repairer	Baby is a great value for money, I always try it before I evaluate it!	JD.com
German science and technology	Battery Repairer	While the baby isn't necessarily the best, it's definitely the best performer inside the same price point.	JD.com
German science and technology	Battery Repairer	Give three good reviews and be satisfied! Satisfied! Satisfied!	JD.com
German science and technology	Battery Repairer	Give my thanks and respect inside the baby reviews of sellers that I feel get my 5-star reviews!	JD.com
German science and technology	Battery Repairer	The baby has been received, very satisfied, wish the store a great sale!	JD.com
German science and technology	Battery Repairer	It has been received and the logistics was fast. Packaging is ok, but there are some small creases on the cover. The baby and the freebie are good, first time buying here, feel ok, overall rating is good. That's about it.	JD.com
German science and technology	Battery Repairer	Instant answers to questions!Awesome!The key is to be happy with!	JD.com
German science and technology	Battery Repairer	Customer service is also very responsible and patient to give recommendations	JD.com
German science and technology	Battery Repairer	Recommended!Those who are still on the fence can take the plunge!	JD.com
German science and technology	Battery Repairer	finally chose this one after comparing a number of them, and I was so relieved!	JD.com
German science and technology	Battery Repairer	I would like to wish the store owner the best of luck and many repeat customers.	JD.com
German science and technology	Battery Repairer	Fast shipping good value for money, give it full marks!	JD.com
German science and technology	Battery Repairer	I'm in the habit of buying things and taking pictures of them.	JD.com
German science and technology	Battery Repairer	Good review!	JD.com
German science and technology	Battery Repairer	It's a bargain.	JD.com
German science and technology	Battery Repairer	Great quality, will be back next time!Express is very strong	JD.com
German science and technology	Battery Repairer	The baby is really good, it matches the picture, there is no difference, it's really worth it!	JD.com
German science and technology	Battery Repairer	I can see the merchant is very attentive	JD.com
German science and technology	Battery Repairer	There is also a beautiful packaging, high-end atmosphere;	JD.com
German science and technology	Battery Repairer	Customer servicebaiexcellent attitude, very patient, givedupeople a kind of affection, good like.	JD.com
German science and technology	Battery Repairer	and attentive, careful and sincere……	JD.com
German science and technology	Battery Repairer	Answers questions relentlessly and carefully, and the key is good stuff!	JD.com
German science and technology	Battery Repairer	Fast shipping, very happy with the purchase	JD.com
German science and technology	Battery Repairer	It's okay.	JD.com
German science and technology	Battery Repairer	Fake**.	JD.com
German science and technology	Battery Repairer	Instructions on how to use it are not clear.	JD.com
German science and technology	Battery Repairer	I've only used it twice and I don't know if it's fixable or not	JD.com
German science and technology	Battery Repairer	follow up. First give a five-star praise! Will repurchase if it's good!	JD.com
German science and technology	Battery Repairer	Pro I buy our baby has received, the quality of the very Heath home to visit.	JD.com
German science and technology	Battery Repairer	unscrupulous businessman	JD.com

German science and technology	Battery Repairer	This user feels the product is better	JD.com
German science and technology	Battery Repairer	ll comment on the quality after use.	JD.com
German science and technology	Battery Repairer	This product looks ok don't know if it will work	JD.com
German science and technology	Battery Repairer	This user feels the product is better	JD.com
German science and technology	Battery Repairer	Don't know the effect yet, looks good, also 有 voice Try it and comment later	JD.com
German science and technology	Battery Repairer	Just received, has not come to the urgent use, I looked at the goods, the inside is empty, is a circuit board, there is nothing else, the weight is gently, I do not know how to use it, use it, no matter how to use it does not matter, is not just more than twenty dollars.	JD.com
German science and technology	Battery Repairer	This user feels the product is better	JD.com
German science and technology	Battery Repairer	Electric bike restorer received very satisfied thanks	JD.com
German science and technology	Battery Repairer	It'll talk when it's energized.	JD.com
German science and technology	Battery Repairer	dissatisfied	JD.com
German science and technology	Battery Repairer	Great business, great quality, thanks.	JD.com
German science and technology	Battery Repairer	It's the effect I was looking for, perfect, love it.	JD.com
German science and technology	Battery Repairer	Arrived, this packaging business is attentive, second praise!	JD.com
German science and technology	Battery Repairer	Shipping was so fast, it's the effect I was looking for, very happy with it, kudos.	JD.com
German science and technology	Battery Repairer	Arrived too soon, right, baby is very good, next time is to come to your home, come on!	JD.com
German science and technology	Battery Repairer	The goods have been received, the product is very good, I am very satisfied, give5points.	JD.com
German science and technology	Battery Repairer	It's pretty good, and my friends love it, and when my friends compliment me, I'm cross merchant.	JD.com
German science and technology	Battery Repairer	I didn't expect it to arrive so quickly, love it, love it!	JD.com
German science and technology	Battery Repairer	What a great seller, will definitely repurchase.	JD.com
German science and technology	Battery Repairer	Real quite good, match the picture, cherish to recommend to friends.	JD.com
German science and technology	Battery Repairer	The baby is genuinely nice, matches the picture and is a real value for money.	JD.com
German science and technology	Battery Repairer	The speed is the biggest plus, keep it up, don't even have to go to a brick and mortar store!	JD.com
German science and technology	Battery Repairer	Shipping was very fast!	JD.com
German science and technology	Battery Repairer	same, very satisfied,really like, completely exceeded expectations, the delivery speed is very fast, the packaging is very careful and strict, the logistics company has a very good service attitude, the delivery speed is very fast, very satisfied with a purchase	JD.com
German science and technology	Battery Repairer	Great price, great service, nice stuff, great customer service!	JD.com
German science and technology	Battery Repairer	Finally found a good store, good service, good quality, next time I have a chance to buy again.	JD.com
German science and technology	Battery Repairer	Second repurchase, truly love it, recommend a friend.	JD.com
German science and technology	Battery Repairer	Shipping was very fast!	JD.com
German science and technology	Battery Repairer	Very happy with the purchase!	JD.com
German science and technology	Battery Repairer	I received it today and I'm very happy with it! Remember to get a discount next time you come!	JD.com
German science and technology	Battery Repairer	I'm going to give a good review……review until everyone sees it!	JD.com
German science and technology	Battery Repairer	The owner was really nice and shipped quickly. The quality of the item is also quite good. Love it so much, thanks!	JD.com
German science and technology	Battery Repairer	Baby received! Very unexpected, will be back next time~great seller, stay tuned!	JD.com
German science and technology	Battery Repairer	Baby received, very satisfied, come back in the future.	JD.com
German science and technology	Battery Repairer	The store is very reputable, and it's great to buy things from this store.	JD.com
German science and technology	Battery Repairer	Very happy with the purchase!	JD.com

German science and technology	Battery Repairer	Who says you can't get good stuff at Poundland!	JD.com
German science and technology	Battery Repairer	I have no hesitation in bookmarking the seller's store.	JD.com
German science and technology	Battery Repairer	This has gone on my infinite repurchase list!	JD.com
German science and technology	Battery Repairer	Very good seller, very enthusiastic ah, next time will come back~baby is very good!	JD.com
German science and technology	Battery Repairer	I am thankful that Hades made me come across this store!	JD.com
German science and technology	Battery Repairer	Arrived very quickly, good quality, good store, recommended~	JD.com
German science and technology	Battery Repairer	Anyone who says it's bad I want to spell it out!	JD.com
German science and technology	Battery Repairer	Compared several, really not disappointed, very good quality, very detailed workmanship, very satisfied, super like.	JD.com
German science and technology	Battery Repairer	The logistics company has a great service and fast delivery!	JD.com
German science and technology	Battery Repairer	The delivery was fast, the guy was polite and didn't mind waiting a few extra minutes for me.	JD.com
German science and technology	Battery Repairer	The quality is excellent and exactly as described by the seller!	JD.com
German science and technology	Battery Repairer	After the receipt of things immediately check, found with the picture description consistent, super like, sellers shipping speed, service is also very in place, to the boss point a praise, the next time will come to buy.	JD.com
German science and technology	Battery Repairer	The packaging is very careful and tight!	JD.com
German science and technology	Battery Repairer	This is one of the most satisfying purchases I've made since I've been shopping.	JD.com
German science and technology	Battery Repairer	Very patient seller, will repurchase if needed in the future.	JD.com
German science and technology	Battery Repairer	Great seller, so patient, I finally got what I wanted. Thanks to the seller.	JD.com
German science and technology	Battery Repairer	This store is okay. Came to buy a few times, very good service to regular customers, and will come back often!	JD.com
German science and technology	Battery Repairer	Such a great seller, don't want to share!	JD.com
German science and technology	Battery Repairer	Shipping was very fast!	JD.com
German science and technology	Battery Repairer	worthwhile. Not too early, not too late, just the right time to meet. So I stopped and waited for this baby to give you something to look forward to, time will not fail to wait. This world, the only way to have love is to exchange sincerity for sincerity. Those sweet words, fame, fortune and vain promises are no match for a profound affirmation. Simple and ordinary words are enough to warm a whole winter. The corners of my mouth must be upturned right now, with deep dimples floating on my cheeks. So beautiful smile, must be to receive this baby let me unconsciously born. ve bookmarked the store,soI hope you'll be happy and happy every day when you see this comment .	JD.com
German science and technology	Battery Repairer	Shipping was very fast!	JD.com
German science and technology	Battery Repairer	Very satisfied,really like it, completely exceeded expectations.	JD.com
German science and technology	Battery Repairer	owner is good, warm service, fast delivery, next time will come to your home, will also recommend others to your home, full five praise!!!!	JD.com
Yawang Digital	Battery Reconditioner	Digital display is clear, charging is quite fast	JD.com
Yawang Digital	Battery Reconditioner	Please do not go to buy I have let when ding`use at all, but also to take the opposite effect`now my cell phone to make even more can not week, merchants are really scammer,	JD.com
Yawang Digital	Battery Reconditioner	serviceable	JD.com
Yawang Digital	Battery Reconditioner	I'll review it after I use it.	JD.com
Yawang Digital	Battery Reconditioner	I'm using it now, and I'm looking for a good result.	JD.com
Yawang Digital	Battery Reconditioner	The cell phone repairer works well and effectively extends the life of the cell phone battery	JD.com
Yawang Digital	Battery Reconditioner	Evaluate after use	JD.com
Yawang Digital	Battery Reconditioner	voltage, a flash of cheap observation, easy to use, read the instructions if you want to achieve the best results have to restore the phone to the factory settings, which is a little complicated can not really get down, and therefore not good too many comments, thank you!	JD.com

Yawang Digital	Battery Reconditioner	[smiles[cool][cool] [cool][shy]shy] [shy] [shy] [shy] [shy] [shy] [shy] [shy] [shy] [big love[love] [big love] [big love] [big love] [big love] [big love] [big love] [big love] [big love] [big love] [big love] [big love] [big love] [big love][big love] [big love][big love][big love][big love]	JD.com
Yawang Digital	Battery Reconditioner	I've used a cell phone and it doesn't look like it's molten.	JD.com
Yawang Digital	Battery Reconditioner	The product is very good and the logistics is very fast	JD.com
Yawang Digital	Battery Reconditioner	Being used, to be evaluated.	JD.com
Yawang Digital	Battery Reconditioner	Baby has been received. The quality is very good. I like the fast logistics. My second time to put together an order.	JD.com
Yawang Digital	Battery Reconditioner	This time the purchase is only genuine, the stuff is very satisfied, very grateful to the courier	JD.com
Yawang Digital	Battery Reconditioner	This user feels the product is better	JD.com
Yawang Digital	Battery Reconditioner	It works great.	JD.com
Yawang Digital	Battery Reconditioner	The product is beautifully showy, works fine, genuine.	JD.com
Yawang Digital	Battery Reconditioner	don't know the effectafter usingfirstll know after using it for a long time.	JD.com
Yawang Digital	Battery Reconditioner	Cell phone battery repairer received, received last night, is genuine, charging while using no hot, I do not know whether the future use of balance charging, business service attitude! Logistics is also very fast!	JD.com
Yawang Digital	Battery Reconditioner	Feeling okay, cuttings plugged into the phone on the phone does not heat up, cell phone Internet speed accelerated	JD.com
Yawang Digital	Battery Reconditioner	Do not evaluate it first. Just received the goods, not yet used, wait for the use of evaluation again	JD.com
Yawang Digital	Battery Reconditioner	This user feels the product is better	JD.com
Yawang Digital	Battery Reconditioner	Works well and is affordable	JD.com
Yawang Digital	Battery Reconditioner	Repairer received, packaging exquisite operation is simple and easy to understand, I hope to use the battery repair to extend the use of the phone to use smooth.	JD.com
Yawang Digital	Battery Reconditioner	Goods received yet do not know how effective use after the evaluation:	JD.com
Yawang Digital	Battery Reconditioner	The digital display is clear, charging is pretty fast, features to be tried.	JD.com
Yawang Digital	Battery Reconditioner	Cell phone battery repairer received. The shape is beautiful and generous. Did not use, such as the use of the later continue to bash, give the business five is favorable.	JD.com
Yawang Digital	Battery Reconditioner	I received the stuff and feel good after using it	JD.com
Yawang Digital	Battery Reconditioner	Small goods arrive assuredly used.	JD.com
Yawang Digital	Battery Reconditioner	Received the baby, feel good, logistics is very fast packaging intact	JD.com
Yawang Digital	Battery Reconditioner	Baby received, can't wait to use it at night, should be good, after charging the power on a lot faster	JD.com
Yawang Digital	Battery Reconditioner	After charging, the phone turned on fast, thank you very much, love it!	JD.com
Yawang Digital	Battery Reconditioner	Both the material and workmanship exceeded expectations, great value, and looks great on oh. The seller also shipped much earlier than expected, and will come back again. Buy it, will not regret it! All five points.	JD.com
Yawang Digital	Battery Reconditioner	It doesn't work very well and is slow to charge.	JD.com
Yawang Digital	Battery Reconditioner	Fast logistics and good service	JD.com
Yawang Digital	Battery Reconditioner	It didn't work. I hope you don't fall for it.	JD.com
Yawang Digital	Battery Reconditioner	Not bad. It's worth it.	JD.com
Yawang Digital	Battery Reconditioner	It works.	JD.com
Yawang Digital	Battery Reconditioner	Not really.	JD.com
Yawang Digital	Battery Reconditioner	This user feels the product is better	JD.com
Yawang Digital	Battery Reconditioner	short time, one price for one item. Read the reviews and buy this should be good. First give a five-star praise da full marks!	JD.com
Yawang Digital	Battery Reconditioner	It's okay.	JD.com

Yawang Digital	Battery Reconditioner	This store is worth buying, used a week to evaluate, I do not know how long to use it! Is it as good as the description and instructions? If so, come back to add positive feedback, and then repurchase, and then give you free advertising.	JD.com
Yawang Digital	Battery Reconditioner	Charging time: not sure yet.	JD.com
Yawang Digital	Battery Reconditioner	I just received it and tried it out.	JD.com
Yawang Digital	Battery Reconditioner	I haven't used it yet, I'll comment again after using it	JD.com
Yawang Digital	Battery Reconditioner	The digital display is clear, charging is quite fast, the product is beautiful, looks good, the battery is durable	JD.com
Yawang Digital	Battery Reconditioner	Not as described	JD.com
Yawang Digital	Battery Reconditioner	good Looks good Practical Very good[love it][love it] Love it	JD.com
Yawang Digital	Battery Reconditioner	This user feels the product is better	JD.com
Yawang Digital	Battery Reconditioner	The stuff is great.	JD.com
Yawang Digital	Battery Reconditioner	As with the publicity display, the shape is beautiful, looking at the pulse data flashes cheerfully, the heart is more open, the phone has an additional layer of protection, how good! Customer service is patient and answers all questions.	JD.com
Yawang Digital	Battery Reconditioner	Fast shipping, standardized packaging, excellent service, and affordable prices.	JD.com
Yawang Digital	Battery Reconditioner	Things received logistics quickly, looking forward to	JD.com
Yawang Digital	Battery Reconditioner	Cell phone battery repairer has received, used a few days did not see obvious results, feel not very satisfied!	JD.com
Yawang Digital	Battery Reconditioner	The goods arrived, the packaging is perfect, using up is okay, only used once, always feel the phone a lot faster. Everyone rest assured to buy.	JD.com
Yawang Digital	Battery Reconditioner	I haven't used it yet, I'll comment after using it.	JD.com
Yawang Digital	Battery Reconditioner	Charging is too slow4hours did not charge slowly, other do not know yet	JD.com
Yawang Digital	Battery Reconditioner	not bad	JD.com
Yawang Digital	Battery Reconditioner	Let's try it first.	JD.com
Yawang Digital	Battery Reconditioner	General.	JD.com
Yawang Digital	Battery Reconditioner	It's not bad at all.	JD.com
Yawang Digital	Battery Reconditioner	[Smiles][Smiles][Smiles]	JD.com
Yawang Digital	Battery Reconditioner	This user feels the product is better	JD.com
Yawang Digital	Battery Reconditioner	sent by the business and the description of the same, extended good	JD.com
Yawang Digital	Battery Reconditioner	The restorer works great and the customer service is great 👍	JD.com
Yawang Digital	Battery Reconditioner	Received the goods, don't know if it works? Good use will recommend to friends 👭the	JD.com
Yawang Digital	Battery Reconditioner	It's good, as described, whether it works or not, I don't know.	JD.com
Yawang Digital	Battery Reconditioner	Hello, in your home to buy the battery repairer has received, really good, especially good, very satisfied, thank you boss, wish boss business prosperity!	JD.com
Yawang Digital	Battery Reconditioner	It doesn't work well, the phone gets stuck after using it and now I'm afraid to use it.	JD.com
Yawang Digital	Battery Reconditioner	I haven't used it yet, let's wait until it's good	JD.com
Yawang Digital	Battery Reconditioner	It's not much use.	JD.com
Yawang Digital	Battery Reconditioner	(of an unmarried couple) be close	JD.com
Yawang Digital	Battery Reconditioner	evaluation, buy before reading the evaluation of friends who have bought basically positive feedback this is to buy. May be their own cell phone is too card with a few days at present the effect is not obvious, it may be too short a time it has to go through a period of time to repair in order to have a significant effect but hopefully it can be realized.	JD.com
Yawang Digital	Battery Reconditioner	can	JD.com
Yawang Digital	Battery Reconditioner	I haven't used it yet. I won't know until I do.	JD.com

Yawang Digital	Battery Reconditioner	Just do activities, the price is much cheaper, cost-effective, really special cost-effective really like, completely exceeded expectations, shipping very fast, packaging very carefully, strict, fast delivery, very satisfied with a shopping	JD.com
Yawang Digital	Battery Reconditioner	review after use	JD.com
Yawang Digital	Battery Reconditioner	The goods have been received! I'm using it now, I'll comment later if it works!	JD.com
Yawang Digital	Battery Reconditioner	Let's see what happens after using it, it's not a bad purchase for the price point yada yada yada!	JD.com
Yawang Digital	Battery Reconditioner	This user feels the product is better	JD.com
Yawang Digital	Battery Reconditioner	False.	JD.com
Yawang Digital	Battery Reconditioner	charge the phone twice not hot while charging the use of cell phone batteries can calm the sparkling value of the purchase of logistics services to the point Thank you business owners Thank you!	JD.com
Yawang Digital	Battery Reconditioner	Got it Love it Nice style Very useful Great for battery repair Really like it	JD.com
Yawang Digital	Battery Reconditioner	I've never used it.	JD.com
Yawang Digital	Battery Reconditioner	Average!	JD.com
Yawang Digital	Battery Reconditioner	The product is slow to charge, did not reflect the ∴ what effect, apply for a refund	JD.com
Yawang Digital	Battery Reconditioner	Received the goods to try a few days is very good next time to come back	JD.com
Yawang Digital	Battery Reconditioner	Just used it, don't know what effect it has yet	JD.com
Yawang Digital	Battery Reconditioner	The product is great.	JD.com
Yawang Digital	Battery Reconditioner	Just received the goods. Scanning code is authentic. Looking forward to the effect.[Smiles][Smiles]	JD.com
Yawang Digital	Battery Reconditioner	The goods have been received and they work well.	JD.com
Yawang Digital	Battery Reconditioner	Received the product is very beautiful, charging quickly fine workmanship, beautiful style, good service attitude, logistics is also fast!	JD.com
Yawang Digital	Battery Reconditioner	Goods received very good Baoshuang is very good, logistics is very fast.	JD.com
Yawang Digital	Battery Reconditioner	Let's see how it works.	JD.com
Yawang Digital	Battery Reconditioner	My phone doesn't have a fever anymore the speed of the phone is wildly normal.	JD.com
Yawang Digital	Battery Reconditioner	Well done phone doesn't heat up, plugged in and running.	JD.com
Yawang Digital	Battery Reconditioner	This charger and repair battery all-in-one baby is very good to use, the appearance of the design sense is very good, the quality is very good, the need of the pro buy with confidence.	JD.com
Yawang Digital	Battery Reconditioner	I can only say now that the service state is good, the quality of the product and the use of the effect is not good, only began to use, can not see, and then follow up later!	JD.com
Yawang Digital	Battery Reconditioner	Satisfied, good service, fast logistics, praise	JD.com
Yawang Digital	Battery Reconditioner	I'll comment after I use it.	JD.com
Yawang Digital	Battery Reconditioner	Great Hope to keep it	JD.com
Yawang Digital	Battery Reconditioner	This product is really good. If you want to buy it, you should order it now.	JD.com
Yawang Digital	Battery Reconditioner	It works fine and it's normal.	JD.com
Yawang Digital	Battery Reconditioner	Not found to help with battery repair	JD.com
Yawang Digital	Battery Reconditioner	This new black tech fixer works great and the delivery was fast.	JD.com
Yawang Digital	Battery Reconditioner	This user feels the product is better	JD.com
Yawang Digital	Battery Reconditioner	Good price, good quality, continue to buy next time	JD.com
Yawang Digital	Battery Reconditioner	We'll talk about it in a few days.	JD.com
Yawang Digital	Battery Reconditioner	Just received the goods, are using do not know the effect, if effective and then evaluation, logistics fast to give them five-star praise!	JD.com
Yawang Digital	Battery Reconditioner	The product is very good to use to repair the battery effect is good very satisfied!	JD.com

Yawang Digital	Battery Reconditioner	This user feels the product is better	JD.com
Yawang Digital	Battery Reconditioner	the charging speed is relatively fast, and the charging is not hot while using! Long-term use can balance the charging pulse current!	JD.com
Yawang Digital	Battery Reconditioner	I'll comment after using it.	JD.com
Yawang Digital	Battery Reconditioner	I haven't used it yet, I'll comment afterward.	JD.com
Yawang Digital	Battery Reconditioner	This user feels the product is better	JD.com
Yawang Digital	Battery Reconditioner	I don't know if it's working.	JD.com
Yawang Digital	Battery Reconditioner	Hello! Charge repair device received, I heard that after using this repair device after the phone does not heat up and not card can also clean up the phone memory garbage, there are friends who want to buy buy to try it!	JD.com
Yawang Digital	Battery Reconditioner	The service is superb, the product is beautiful and durable, and I'm very satisfied with it.	JD.com
Yawang Digital	Battery Reconditioner	The goods have been received, have not yet used, the effect is not known. However, the packaging is very good, in general, should be able to.	JD.com
Yawang Digital	Battery Reconditioner	Clear digital display	JD.com
Yawang Digital	Battery Reconditioner	This user feels the product is better	JD.com
Yawang Digital	Battery Reconditioner	Watch and listen.	JD.com
Yawang Digital	Battery Reconditioner	Goods have been received, not yet used, evaluation after use	JD.com
Yawang Digital	Battery Reconditioner	Slow logistics	JD.com
Yawang Digital	Battery Reconditioner	Matches the description, tried it yesterday, will see the effect in a few days, if it works well, will recommend friends	JD.com
Yawang Digital	Battery Reconditioner	This user feels the product is better	JD.com
Yawang Digital	Battery Reconditioner	It works. It's good. Thanks.	JD.com
Yawang Digital	Battery Reconditioner	It looks high class and has a great look	JD.com
Yawang Digital	Battery Reconditioner	Goods to receive, the quality is very good, real material, logistics is very fast, it is worth recommending.	JD.com
Yawang Digital	Battery Reconditioner	Charges pretty fast.	JD.com
Yawang Digital	Battery Reconditioner	The appearance is ok, and it is very convenient that it automatically cuts off when it is full![Big Love][Big Love][Big Love][Big Love]	JD.com
Yawang Digital	Battery Reconditioner	Nice packaging Yellow and tender Hope it works	JD.com
Yawang Digital	Battery Reconditioner	Cell phone repairer received, the style is quite good-looking, easy to operate, can automatically repair the cell phone battery problems, and can be carried around, a very good choice, like it will buy back!	JD.com
Yawang Digital	Battery Reconditioner	I just used it and I don't know if it works?	JD.com
Yawang Digital	Battery Reconditioner	Things received two days logistics very quickly customer service attitude is very good with the okay	JD.com
Yawang Digital	Battery Reconditioner	Item is back, powered up and just used.	JD.com
Yawang Digital	Battery Reconditioner	Timely shipping, good service attitude, small and exquisite, very like, have friends need, can be promoted	JD.com
Yawang Digital	Battery Reconditioner	This cell phone repairer works great and I'm very happy with the fast shipping from the merchant!	JD.com
Yawang Digital	Battery Reconditioner	It's not the same as in the video. It's a little too small.	JD.com
Yawang Digital	Battery Reconditioner	Fast shipping, good quality, fast logistics.	JD.com
Yawang Digital	Battery Reconditioner	It looks good. I'll comment on it when I've used it.	JD.com
Yawang Digital	Battery Reconditioner	Battery repair is very good, back to open and use, businessmen shipping fast, logistics is strong, it is worth getting to buy, next time will patronize your store.	JD.com
Yawang Digital	Battery Reconditioner	The product has been used for three days since it was received, and the effect feels very good, so I give it a five-star review. Good! Good! Good!	JD.com
Yawang Digital	Battery Reconditioner	I don't know if it works yet, I'll review it then	JD.com
Yawang Digital	Battery Reconditioner	very satisfied	JD.com

Yawang Digital	Battery Reconditioner	It's good. It's fast.	JD.com
Yawang Digital	Battery Reconditioner	received	JD.com
Yawang Digital	Battery Reconditioner	I just got it back today, how long did it take to charge? Before fixing it?	JD.com
Yawang Digital	Battery Reconditioner	Received and used, feel the phone speed faster, immediately to evaluate, customer service is very patient, explained how to use[kudos]praise!	JD.com
Yawang Digital	Battery Reconditioner	It's a lie. Fix it, my ass.	JD.com
Yawang Digital	Battery Reconditioner	So far it feels like it's charging👍👍👍👍👍	JD.com
Yawang Digital	Battery Reconditioner	Bought2of these, hope they work well, great, if they work well, will buy more[smile]	JD.com
Yawang Digital	Battery Reconditioner	It's just faster.	JD.com
Yawang Digital	Battery Reconditioner	This is a good product.	JD.com
Yawang Digital	Battery Reconditioner	Straight volume of good friends can be enough to buy	JD.com
Yawang Digital	Battery Reconditioner	This restorer doesn't work, is it a fake product	JD.com
Yawang Digital	Battery Reconditioner	It works, it's cozy, the courier service is excellent, give it a 5 star rating, thanks!	JD.com
Yawang Digital	Battery Reconditioner	Received a few days, used to feel okay, give5stars praise, praise, customer service and I said this product will protect5years, hope the quality bar good	JD.com
Yawang Digital	Battery Reconditioner	Good quality, quite good, seller service attitude is also very good	JD.com
Yawang Digital	Battery Reconditioner	(of an unmarried couple) be close	JD.com
Yawang Digital	Battery Reconditioner	The item is great, I love it and the delivery was fast! The service is also very good!	JD.com
Yawang Digital	Battery Reconditioner	This user feels the product is better	JD.com
Yawang Digital	Battery Reconditioner	Roger that. Thanks. That's great.	JD.com
Yawang Digital	Battery Reconditioner	It works. It works right away.	JD.com
Yawang Digital	Battery Reconditioner	The restorer is well made, but hopefully it will work. Great customer service and patience in answering customer questions! Five star reviews!	JD.com
Yawang Digital	Battery Reconditioner	How can you underpunch a powerhead? Poor, poor, poor.	JD.com
Yawang Digital	Battery Reconditioner	this product	JD.com
Yawang Digital	Battery Reconditioner	Good quality, came back and tried it out, it works quite well, next time I will come back.	JD.com
Yawang Digital	Battery Reconditioner	Cell phone repair artifact aesthetics look very good, small and exquisite, I hope it can play a role in the repair of cell phone batteries, five-star praise.	JD.com
Yawang Digital	Battery Reconditioner	The digital display is clear, the charging is quite fast and the product is beautiful	JD.com
Yawang Digital	Battery Reconditioner	It's good.	JD.com
Yawang Digital	Battery Reconditioner	It's good. It works.	JD.com
Yawang Digital	Battery Reconditioner	The product is quite good, not bad	JD.com
Yawang Digital	Battery Reconditioner	This user feels the product is better	JD.com
Yawang Digital	Battery Reconditioner	Received, do not know whether it is good or not, wait for a good use and then come back to the review	JD.com
Yawang Digital	Battery Reconditioner	me how to do, thank you very much, have not repaired the phone at night to do the repair, the product is very beautiful, trustworthy!	JD.com
Yawang Digital	Battery Reconditioner	Product received. Still happy with the delivery from order to receipt. Is being used. Don't know how the quality of the product is yet no assessment for now	JD.com
Yawang Digital	Battery Reconditioner	an excellent	JD.com
Yawang Digital	Battery Reconditioner	All fakes can't be re-circulated0points[big love]	JD.com
Yawang Digital	Battery Reconditioner	Good quality product good grasp, good quality, easy to use very satisfactory	JD.com
Yawang Digital	Battery Reconditioner	I think it's okay. I just used it today.	JD.com

Yawang Digital	Battery Reconditioner	Habitually favorable reviews!	JD.com
Yawang Digital	Battery Reconditioner	Clear digital display	JD.com
Yawang Digital	Battery Reconditioner	It works.	JD.com
Yawang Digital	Battery Reconditioner	charging is very effective and extends battery life after reconditioning.	JD.com
Yawang Digital	Battery Reconditioner	This user feels the product is better	JD.com
Yawang Digital	Battery Reconditioner	I used it and it works well. I'll buy more next time.	JD.com
Yawang Digital	Battery Reconditioner	cheap, it is worth buying, just started to try, after repairing the effect is good, if the next time, but also back to buy.	JD.com
Yawang Digital	Battery Reconditioner	This is a pretty good restoration and a beautiful style, need to get another one.	JD.com
Yawang Digital	Battery Reconditioner	See the results after use	JD.com
Yawang Digital	Battery Reconditioner	Repair effect is very good style also like, very satisfied with an online shopping!	JD.com
Yawang Digital	Battery Reconditioner	Let's use it first.	JD.com
Yawang Digital	Battery Reconditioner	It works well.	JD.com
Yawang Digital	Battery Reconditioner	Loved it very satisfied with everyone's support thank you thank you thank you	JD.com
Yawang Digital	Battery Reconditioner	I just received it and haven't used it yet, I'll comment after I use it.	JD.com
Yawang Digital	Battery Reconditioner	Baby received, previously bought a cheap price, not very good, this time the arrival immediately use, this price is more expensive, really a penny a penny. Charge fast, novel style. Convenient operation. Friends in need, I recommend buying this is still a good choice. Five-star praise!	JD.com
Yawang Digital	Battery Reconditioner	Great quality quick charge phone is smooth really works well.	JD.com
Yawang Digital	Battery Reconditioner	The batteries do last longer.	JD.com
Yawang Digital	Battery Reconditioner	Very much. Very much.	JD.com
Yawang Digital	Battery Reconditioner	Very good. How to use there is also live scanning teaching. Attentive service, express delivery is also very fast!	JD.com
Yawang Digital	Battery Reconditioner	It's great.[Smiles][Smiles]	JD.com
Yawang Digital	Battery Reconditioner	dissatisfied	JD.com
Yawang Digital	Battery Reconditioner	can	JD.com
Yawang Digital	Battery Reconditioner	Liar.	JD.com
Yawang Digital	Battery Reconditioner	Good quality product very much like	JD.com
Yawang Digital	Battery Reconditioner	I'm using it, I don't know if it's effective for repairing, I'll follow up after a while.	JD.com
Yawang Digital	Battery Reconditioner	Received last night, immediately try, charging fast, very good, and so repair, see the effect, and then follow up! First deceive a five-star praise it!	JD.com
Yawang Digital	Battery Reconditioner	very happy	JD.com
Yawang Digital	Battery Reconditioner	Seems like an IQ tax to me now😒😒😒	JD.com
Yawang Digital	Battery Reconditioner	okay!	JD.com
Yawang Digital	Battery Reconditioner	Let's give it a good review and use it.	JD.com
Yawang Digital	Battery Reconditioner	Who can say what this is like when you receive it?	JD.com
Yawang Digital	Battery Reconditioner	Baby received, to be used. Good reviews.	JD.com
Yawang Digital	Battery Reconditioner	It's okay.	JD.com
Yawang Digital	Battery Reconditioner	Charging speed is fast, the phone does not get hot while charging! Genuine	JD.com
Yawang Digital	Battery Reconditioner	Already received it, trying it out, hope it can really fix the battery, good to use and then come back to evaluate.	JD.com
Yawang Digital	Battery Reconditioner	The goods are good ah used to comment again.	JD.com
Yawang Digital	Battery Reconditioner	Goods arrived to use the feeling quite good thanks to the pro to bring the gift💥💥💥💥💥	JD.com

Yawang Digital	Battery Reconditioner	This user feels the product is better	JD.com
Yawang Digital	Battery Reconditioner	Received the product, just used it, feel good!	JD.com
Yawang Digital	Battery Reconditioner	It doesn't work. It's like paying 50 bucks for a charger.	JD.com
Yawang Digital	Battery Reconditioner	Goods received.	JD.com
Yawang Digital	Battery Reconditioner	It's amazing, theoPphonebought last yearcharged once a day, I used this fixer three times and now it charges once every two days, whoever buys it earns	JD.com
Yawang Digital	Battery Reconditioner	This Charge Repair Scam	JD.com
Yawang Digital	Battery Reconditioner	The effect is not	JD.com
Yawang Digital	Battery Reconditioner	Looked at the quality very good next time continue to buy	JD.com
Yawang Digital	Battery Reconditioner	logistics, as for the effect, wait a few days to see how to say, first praise! The effect is good and then make a follow-up comment.	JD.com
Yawang Digital	Battery Reconditioner	The restorer was bought and tried a few times, no response at all, please friends do not fall for the scam.	JD.com
Yawang Digital	Battery Reconditioner	and convenient, with the seller's description in line with the seller's fast shipping, logistics company is also strong, give five points!	JD.com
Yawang Digital	Battery Reconditioner	acceptable	JD.com
Yawang Digital	Battery Reconditioner	(of an unmarried couple) be close	JD.com
Yawang Digital	Battery Reconditioner	(of an unmarried couple) be close	JD.com
Yawang Digital	Battery Reconditioner	Logistics is fast, service is good, product quality to be verified	JD.com
Yawang Digital	Battery Reconditioner	Battery Reconditioner is good quality, just using it, don't know how effective it is	JD.com
Yawang Digital	Battery Reconditioner	I just received it. I'll try it later.	JD.com
Yawang Digital	Battery Reconditioner	BatteryIRepairer received and the results should work.	JD.com
Yawang Digital	Battery Reconditioner	Packaging is quite good, charging is quite fast, the phone battery has been charged twice the effect is being observed, but hopefully it will have good results!	JD.com
Yawang Digital	Battery Reconditioner	I'll comment after I've used it and it's good	JD.com
Yawang Digital	Battery Reconditioner	This is the best and most useful baby I've ever bought on the Poundland shopping platform! Will definitely come back when I have the chance!	JD.com
Yawang Digital	Battery Reconditioner	Baby received Trying to fix it I'll comment after using it Thank you boss	JD.com
Yawang Digital	Battery Reconditioner	Baby received, last night after charging and using the effect is good, as described, trustworthy.	JD.com
Yawang Digital	Battery Reconditioner	Doesn't seem to work, doesn't speed up the phone	JD.com
Yawang Digital	Battery Reconditioner	Cell phone battery repairer received, logistics faster, well-packed. After the trial, I feel that the repair effect is very good, more satisfied, give five-star praise.	JD.com
Yawang Digital	Battery Reconditioner	This user feels the product is better	JD.com
Yawang Digital	Battery Reconditioner	Express very timely effect and so use a period of time in the evaluation it if satisfied with the pleading will certainly recommend to a good circle of friends inside!	JD.com
Yawang Digital	Battery Reconditioner	Black tech, tried charging much faster, but not lagging this will have to be spent to find out	JD.com
Yawang Digital	Battery Reconditioner	This is a great product.	JD.com
Yawang Digital	Battery Reconditioner	an excellent	JD.com
Yawang Digital	Battery Reconditioner	Didn't work at all, used it for a week, threw it away. IQ tax to buy a lesson	JD.com
Yawang Digital	Battery Reconditioner	Battery description: express timely, good quality, service another thoughtful	JD.com
Yawang Digital	Battery Reconditioner	It is indeed quite good and the logistics is fast.	JD.com
Yawang Digital	Battery Reconditioner	The goods have been received and are quite satisfactory.	JD.com
Yawang Digital	Battery Reconditioner	I've been using it for two days now and it's still looking good. But haven't seen the effect yet. I'll comment again after using it for two days	JD.com

Yawang Digital	Battery Reconditioner	The goods are very good, the logistics is very fast. Charging suburbia fruit is very good.	JD.com
Yawang Digital	Battery Reconditioner	Yes, you can.	JD.com
Yawang Digital	Battery Reconditioner	The logistics is fast, the knowledge is good, too bleached too beautiful, too much good.	JD.com
Yawang Digital	Battery Reconditioner	Already used two or three times, no obvious effect yet, continue to use to see!	JD.com
Yawang Digital	Battery Reconditioner	Very good, there are advantages over regular chargers.	JD.com
Yawang Digital	Battery Reconditioner	Repair has received, well-packed logistics very quickly, the first to use to see how the effect, and then follow up, first give a five-star praise!	JD.com
Yawang Digital	Battery Reconditioner	Good quality, easy to use.	JD.com
Yawang Digital	Battery Reconditioner	handy	JD.com
Yawang Digital	Battery Reconditioner	Goods have been received, purchase satisfaction five-star praise, thank you seller!	JD.com
Yawang Digital	Battery Reconditioner	Take back the night to an old phone, repair a moment, to the next morning, really an advertisement are not, don't five-star praise, quickly, logistics courier hard work!	JD.com
Yawang Digital	Battery Reconditioner	Have aniphone4s, we all know, very classic, the battery repair effect is very good, praise	JD.com
Yawang Digital	Battery Reconditioner	I just started using it10days and brought it in to recharge, and I feel as if the battery has lengthened a bit.	JD.com
Yawang Digital	Battery Reconditioner	Things are very satisfied Oh, beautiful and stylish atmosphere, customer service is super patient, service is meticulous and attentive, the big brands are not the same!	JD.com
Yawang Digital	Battery Reconditioner	resurrection, originally wanted to change the phone now do not have to change!	JD.com
Yawang Digital	Battery Reconditioner	The product received, genuine, the use of the effect is very good, fine workmanship and beautiful, Overseas Chinese Gog affordable, thoughtful service, has given5stars	JD.com
Yawang Digital	Battery Reconditioner	Bought this hoping it works so I don't have to spend more money on a new phone	JD.com
Yawang Digital	Battery Reconditioner	have time, my cell phone battery drains strangely, up to 30 or 40 pcs of power will drop off at once, it's just annoying, and there's still a problem with charging it when it's turned off, the	JD.com
Yawang Digital	Battery Reconditioner	After using the reconditioned battery two or three times, it still feels better than when it was not reconditioned, so I hope it will be better in the future!	JD.com
Yawang Digital	Battery Reconditioner	prompted to replace, no power to boot can not open, used the repairer to repair, but also can charge and boot up, really can be	JD.com
Yawang Digital	Battery Reconditioner	Vivocell phone a grid of electricity is also gone, can not charge into the electricity is this battery repair really activate the phone, very happy thanks to the business of good products! May this good product can help more people in need, I wish the boss a prosperous business!	JD.com
Yawang Digital	Battery Reconditioner	before anyway. Whatever the principle is, it's good if it works well	JD.com
Yawang Digital	Battery Reconditioner	the effect is not very obvious, I hope that after a few more times will have the effect, the later with a good will recommend to friends.	JD.com
Yawang Digital	Battery Reconditioner	Is a repairer just bought back, always thought can not have no effect, but after the boss general patience guide, charge twice a week, more than three weeks on the effect, and now it is true that the cell phone is more durable than the previous battery, it is worth buying!	JD.com
Yawang Digital	Battery Reconditioner	Used two two charging3have six hours, the effect is still some, see the back of the use and then Lei evaluation, but I hope that as described useful, first to give a good review	JD.com
Yawang Digital	Battery Reconditioner	Good to use originally fully charged with3hours, yesterday just started to use Doting two hours. Thank you for the express customer service teaching and understanding, stage yi have staff, in special times adhere to the work. Wish the boss a prosperous business and prosperity!	JD.com
Yawang Digital	Battery Reconditioner	Baby high usage, comparable, applicable to all types of cell phones, the store has a good service attitude, has recommended the baby to friends, thanks to the store! Five-star praise!	JD.com
Yawang Digital	Battery Reconditioner	Over the past few days, according to the business and the instructions to come up to operate, not only the phone can be repaired, even my Xiaomi Tablet can be repaired. This is still my experience with the merchant on the customer service, and it is really good! The service patience and attitude of the merchant is great!	JD.com

Yawang Digital	Battery Reconditioner	Battery Repairer is okay, it's just a little bit slightly troublesome to do, it can fix the battery and cell phone lagging, in general it's okay	JD.com
Yawang Digital	Battery Reconditioner	After charging it once I feel that my cell phone standby time has increased. Hopefully it will work very well after a few fixes.	JD.com
Yawang Digital	Battery Reconditioner	Cell phone battery charging repairer received. Not too big. Chic appearance. Fine workmanship. The digital display is clear. Hope to achieve the actual effect. Five stars. The use of the situation later review.	JD.com
Yawang Digital	Battery Reconditioner	Repairer is good, mecha style, voltage and current stable output, display, battery repair god, full five praise!	JD.com
Yawang Digital	Battery Reconditioner	the repair of two cell phones, both significantly faster than the original, wait for a period of time to see the effect of the battery, thanks to customer service patience and effective help.	JD.com
Yawang Digital	Battery Reconditioner	Current and voltage, when monitoring. Peace of mind, efficient heat dissipation.	JD.com
Yawang Digital	Battery Reconditioner	Baby received, logistics God speed. Multiple functions, battery repair. Trendy shape, extremely cool. Dazzling light, more attention.	JD.com
Yawang Digital	Battery Reconditioner	Trial1month, power a lot more, a day and a half are used up, now able to stop for a day, good good, worth buying	JD.com
Yawang Digital	Battery Reconditioner	Goods received! After a few days of charging experiments, the battery control to stabilize the fast discharge, charging speed adjustment to strengthen a lot. And the previous charging effect is not the same.	JD.com
Yawang Digital	Battery Reconditioner	battery repairer, the cell phone life is extended a lot! Powerful!	JD.com
Yawang Digital	Battery Reconditioner	The previous cell phone battery does not work, what do not do on the drop power, which stored a lot of things do not want to change the phone, by chance searched for this product, buy to try the effectiveness of the	JD.com
Yawang Digital	Battery Reconditioner	As time goes by, the cell phone charge once the use of time is getting shorter and shorter, saw this device and bought it, beautifully packaged, exquisite device, I believe that, as the instructions say, it will have the effect of extending the battery life and improve the performance of the phone.	JD.com
Yawang Digital	Battery Reconditioner	have a good repair effect relative to the cell phone battery, you need to use the repairer for a long time, charging the cell phone, the	JD.com
Yawang Digital	Battery Reconditioner	Fast logistics, baby is very good, charging fast, can fix the battery is better, in the use of the results after seeing.	JD.com
Yawang Digital	Battery Reconditioner	logistics is fast, color digital display, beautiful and exquisite, repair batteries, more durable, thanks to the business.	JD.com
Yawang Digital	Battery Reconditioner	good, my cell phone original full of electricity can only use three hours, use this repairer charge an hour later can use a day, Jiao Ge magic, worth buying, thanks to the business good product!	JD.com
Yawang Digital	Battery Reconditioner	Have just tested it on my laptop and the results have improved. Booting up is faster than it was, opening web pages, files and videos have all been sped up. Ready to test a few more times to see if there is still an improvement.	JD.com
Yawang Digital	Battery Reconditioner	This product has been tried. Tried three old cell phones and felt like they opened and surfed the web faster than they did. Now trying an old laptop to see how it works. The product does not fail to deliver, good	JD.com
Yawang Digital	Battery Reconditioner	smoother. Customer service is very good and explains very clearly	JD.com
Yawang Digital	Battery Reconditioner	Express received, the quality of goods is also very good, the packaging is very strong, no damage, style and	JD.com
Yawang Digital	Battery Reconditioner	In kind is very good, with the seller's description of the exact same match, logistics fast seller's service attitude is also good, the most important quality is very good, this is a very satisfied with the shopping.	JD.com
Yawang Digital	Battery Reconditioner	My friend recommended it, and it didn't disappoint me, it's what I wanted, so if you like it, you can go ahead and buy it, you won't be disappointed.	JD.com
Yawang Digital	Battery Reconditioner	the quality or other are very, like, and the seller described exactly the same, like friends can recommend buying, very satisfied.	JD.com
Yawang Digital	Battery Reconditioner	Quite good, inexpensive, before the other home also bought, but the quality of this one is still better, workmanship and other things are more fine, it is worth getting!	JD.com
Yawang Digital	Battery Reconditioner	Because of online shopping for fear of return and replacement trouble, so it is directly consult the customer service to buy, customer service is very good recommended very good!	JD.com

Yawang Digital	Battery Reconditioner	The first time to buy things in this, of course, is still very hesitant, did not expect to receive a very pleasant surprise, the goods are consistent with the description, the logistics is also very powerful, give a favorable review to support.	JD.com
Yawang Digital	Battery Reconditioner	Express is very powerful, not a few days to arrive, the style of fashion atmosphere, the quality is very good, the seller shipped fast. Very satisfied.	JD.com
Yawang Digital	Battery Reconditioner	Merchants are very good to talk to oh, very polite ah, wish merchants more and more prosperous business.	JD.com
Yawang Digital	Battery Reconditioner	Fixer2weeks now, it's working great and the phone lag problem is solved	JD.com
Yawang Digital	Battery Reconditioner	Jitterbug someone sells288, Poundlandmore than70, you know, whether it works or not, saving money is happy	JD.com
Yawang Digital	Battery Reconditioner	Racer brush over, Racer178a, heremore than70dollars a, really Pinduoduo cheap ah haha	JD.com
Yawang Digital	Battery Reconditioner	Indeed very good, is genuine, with2weeks, the effect is very obvious, the battery repair is very good	JD.com
Yawang Digital	Battery Reconditioner	Really can be oh, good stuff to come, good comments	JD.com
Yawang Digital	Battery Reconditioner	It's noticeable after a week of use, the range is longer, good review!	JD.com
Yawang Digital	Battery Reconditioner	Things are good, is why the boss does not sell higher, a few cheap make people feel can not believe that there is an effect, in general it does work!	JD.com
Yawang Digital	Battery Reconditioner	Really nice this thing, no sound, ready to plug in and use, convenient	JD.com
Yawang Digital	Battery Reconditioner	Repair the battery good helper, the old phone can also be used, the price is not expensive, than spend thousands of dollars to buy a cell phone is worth it!	JD.com
Yawang Digital	Battery Reconditioner	Thought it was aUSBflash drive, but it turned out to be a black technology to repair the battery, it is really nothing strange, I have to say, a little effect	JD.com
Yawang Digital	Battery Reconditioner	Apple12battery is not working right, so I came to buy one to test it, looking forward to the result	JD.com
Yawang Digital	Battery Reconditioner	Poor can't afford a new phone, stuff tested, should the phone last another2years, hey the mouthpiece issue is hurting me	JD.com
Yawang Digital	Battery Reconditioner	continue to use for a period of time, good use in the come to buy	JD.com
Yawang Digital	Battery Reconditioner	Estimated test is not an IQ tax, bought2, deliberately used2old cell phone test,1week down, the battery has a little bit of prolonged effect, continue to use see	JD.com
Yawang Digital	Battery Reconditioner	Indeed is a good product, can repair effective, can maintain the battery, but also can charge, praise!	JD.com
Yawang Digital	Battery Reconditioner	Things are good, the price can be, customer service attitude is very good, very patient to me, praise!	JD.com
Yawang Digital	Battery Reconditioner	knowledge, my old man is out of date, this thing is very good, good use	JD.com
Yawang Digital	Battery Reconditioner	Never thought, a small thing, actually can help me quite a lot, long useless Samsung tablet, because of battery problems, can not open the machine, connected to this product charging, actually can open the machine, the information can be taken out and used, praise praise praise!	JD.com
Yawang Digital	Battery Reconditioner	Looking forward to the effect,5days to arrive, looking quite high-tech,1month later in the evaluation	JD.com
Yawang Digital	Battery Reconditioner	How to say, I also think it's quite amazing, just used1week, plugged in2times, Tencent video was watching5hours without electricity, now you can6hours	JD.com
Yawang Digital	Battery Reconditioner	I'm a poor player, a HuaweiMETE20with now do not want to change, the battery is just a little worse, this thing can really help, open a League of Legends10won't shut down, good	JD.com
Yawang Digital	Battery Reconditioner	warrant5years, guarantee to do, with a month in the back to follow up	JD.com
Yawang Digital	Battery Reconditioner	Things are good, is to receive the goods in Shanghai, a whole week, the overall is still effective, Apple14repair and maintenance up, I hope my14can use a4years	JD.com
Yawang Digital	Battery Reconditioner	of5fixes, the battery wasn't heating up, the range was extended, it was very good, bought another one tomaintainmy Apple13	JD.com
Yawang Digital	Battery Reconditioner	at night when plugged in, the product digital display constantly jumping, the phone will be very stable repair, no other adverse reactions, continue to try to see, later in the follow-up comment!	JD.com
Yawang Digital	Battery Reconditioner	Operation is simple, things look high, just used a week, samsungNOTE10fever less often, especially a open king of glory, the game opens faster, not bad dozens of things	JD.com

Yawang Digital	Battery Reconditioner	It should not be an illusion, just use1week, the phone did not heat up playing games, and the battery life becomes a little longer, anyway, try, do not use on the return, the boss promises thatwithin1year can be returned	JD.com
Yawang Digital	Battery Reconditioner	It's okay,3chicken dropped60%of the electricity, used2weeks, now3chicken dropped40%of the electricity, in the use of a period of time in the follow-up comment	JD.com
Yawang Digital	Battery Reconditioner	It's not bad, battery health80%,1month or80%, my small7and live, extend battery life good helper, this repairer is genuine, over2years in the replacement of cell phones	JD.com
Yawang Digital	Battery Reconditioner	week, the effect is very good, the battery life really increased a lot, the battery is not like before to play a while on the heat of the hot hand, and now play for a long time will not be hot, the black science and technology products	JD.com
Yawang Digital	Battery Reconditioner	really works, I thought there was no saving, my OPPOis now barreling along.one back with the intention of trying it out, followed the tutorial and it really worked, the battery lasted twice as long and last3weeks, really great deal	JD.com
Yawang Digital	Battery Reconditioner	Repair is very good, the effect is powerful, the use of the battery is not so fast dead, dozens of dollars can be repaired, more cost-effective than the replacement of the phone, hahaha save a lot of money, is the genuine it!	JD.com
Yawang Digital	Battery Reconditioner	Follow the tutorials to do quite simple, the phone and the same before, is to sleep at night when plugged in can be automatically repaired, in fact, buy the time to try to see the mentality, after getting overjoyed than imagined, dozens of dollars to solve the problem, you do not have to thousands of dollars to change a cell phone!	JD.com
Yawang Digital	Battery Reconditioner	use of the effect is very obvious, it is indeed a very cost-effective thing to save thousands of dollars to change the phone, the operation is also particularly easy, not for a while on the good recommended!	JD.com
Yawang Digital	Battery Reconditioner	using this restorer, now almost can watch fast5hours it, receive calls1day no problem it, this phone is very old, can use so long, really happy. This is a genuine product, I really recommend it!	JD.com
Yawang Digital	Battery Reconditioner	the hands of a, indeed the battery with a long time, the phone will not be so stuck, the boss said with90days no effect can be returned, something useful on the return, the times are changing ah, technology in the development of our countryside are an eye-opener	JD.com
Yawang Digital	Battery Reconditioner	have to change the battery, after repairing the phone with a longer time some, and does not heat up, and also automatically clean up the phone system cache garbage, the phone has a significantly smooth, the boss said that often used to repair will be better.	JD.com
Yawang Digital	Battery Reconditioner	Apple X battery life before a month to drop 5 percent, with the previous 80 percent, used 4 weeks, is still 80 percent, really awesome, recommend recommended!	JD.com
Yawang Digital	Battery Reconditioner	High black technology products, the store said it can protect the phone battery by controlling the current, in fact, it is right, many times the voltage is not stable we do not know. Now the cell phone battery with a little bit of the next began to decline in performance, that is, the current is not stable, this try a day is indeed very good.	JD.com
Yawang Digital	Battery Reconditioner	phone battery does not heat up, and is very resistant to electricity, saving more than 100 pieces of battery replacement, according to the business said tutorials to do the phone system has become smooth, it seems that the phone can also last 2 years!	JD.com
Royal Preferences	Battery Reconditioner	The store's gone.	JD.com
Night Prairie Life	Battery Reconditioner	This user did not fill in a rating	JD.com
Night Prairie Life	Battery Reconditioner	It didn't work, no response at all, it's not what it actually describes,[fumes][fumes][fumes][fumes][fumes]	JD.com
Night Prairie Life	Battery Reconditioner	incompetent	JD.com
Night Prairie Life	Battery Reconditioner	Very satisfied with the use of12hours of electricity charged in, riding for two days nothing strange	JD.com
Night Prairie Life	Battery Reconditioner	instructions to repair the24hours, but also the same as before, there is no hanging, but also quite expensive, the most pitiful a shopping, poor evaluation, do not recommend buying	JD.com
Night Prairie Life	Battery Reconditioner	The battery's all messed up.	JD.com

Night Prairie Life	Battery Reconditioner	This product can't repair the battery, the whole thing is a piece of junk!	JD.com
Night Prairie Life	Battery Reconditioner	Good quality will buy back	JD.com
Night Prairie Life	Battery Reconditioner	This activation artifact, not so good	JD.com
Night Prairie Life	Battery Reconditioner	electric put loss almost spendmore than300to replace the battery with this charger to save back the boss is really good especially patient savemore than200boss hair big money[love][love] [love]	JD.com
Night Prairie Life	Battery Reconditioner	Charging method: convenient|Charging speed: fast Safety performance: especially great	JD.com
Night Prairie Life	Battery Reconditioner	Electric car a winter ride, the battery starved to death, replace the battery to more than three hundred, fortunately bought this, immediately activated, praise	JD.com
Night Prairie Life	Battery Reconditioner	Successful repair of starved batteries is very good	JD.com
Night Prairie Life	Battery Reconditioner	It works. It's just a little too slow to fix.	JD.com
Night Prairie Life	Battery Reconditioner	Starved battery successfully activated	JD.com
Night Prairie Life	Battery Reconditioner	results yet. Use it more than twice before submitting a review.	JD.com
Night Prairie Life	Battery Reconditioner	It doesn't work. It's still the same.	JD.com
Night Prairie Life	Battery Reconditioner	the merchants build the question of their own return, but they insisted on leaving the future with the power to activate1~2timesa month	JD.com
Night Prairie Life	Battery Reconditioner	acceptable	JD.com
Night Prairie Life	Battery Reconditioner	I haven't used it yet, I'll follow up when I use it	JD.com
Night Prairie Life	Battery Reconditioner	Bullseye, fixed it for over two hours automatically jumped, then took the original charger and charged it. 666	JD.com
Night Prairie Life	Battery Reconditioner	Electric car battery repairer used, the effect is really great, before the battery can not charge how much electricity, after using the repairer, the battery charging power much more, must follow up, must point a praise!	JD.com
Night Prairie Life	Battery Reconditioner	I used it once. It's not ideal. It doesn't seem to work.	JD.com
Night Prairie Life	Battery Reconditioner	Electric car battery repairer received, the quality is very good, the effect is great, the battery's big savior, buy is to earn, must give five-star praise, must!	JD.com
Night Prairie Life	Battery Reconditioner	I haven't used it yet, I'll say later I should trust the technology	JD.com
Night Prairie Life	Battery Reconditioner	I haven't used it yet, I don't know what's going on, I'll comment after I use it.	JD.com
Night Prairie Life	Battery Reconditioner	Poor functionality.	JD.com
Night Prairie Life	Battery Reconditioner	Charged two sets of batteries. But can't see any results yet.	JD.com
Night Prairie Life	Battery Reconditioner	The goods have been received, have not yet tried, try to come back to the review	JD.com
Night Prairie Life	Battery Reconditioner	After using it for a few days, it will automatically power off and the repair function is to be tested.	JD.com
Night Prairie Life	Battery Reconditioner	I haven't used it yet, I don't know how it works.	JD.com
Night Prairie Life	Battery Reconditioner	Quite good, thanks, love it, very happy with a shopping, next time back to buy, like the small friends quickly, and the picture is the same.	JD.com
Night Prairie Life	Battery Reconditioner	been testeddoesn't work on a battery car that hasn't been used3months and can't berecharged.	JD.com
Night Prairie Life	Battery Reconditioner	What's that? It burned out my charger.	JD.com
Night Prairie Life	Battery Reconditioner	Haven't used it yet, don't know the effect.	JD.com
Night Prairie Life	Battery Reconditioner	description, looks good has not yet been used, after using it to follow up.	JD.com
Night Prairie Life	Battery Reconditioner	fast, safety performance: safe, safe performance, worth buying.	JD.com
Night Prairie Life	Battery Reconditioner	It looks okay, I'll try it later.	JD.com
Night Prairie Life	Battery Reconditioner	I think it's just a charger.	JD.com
Night Prairie Life	Battery Reconditioner	It's okay. I'll read it after a while.	JD.com
Night Prairie Life	Battery Reconditioner	pretty good	JD.com

Night Prairie Life	Battery Reconditioner	This user feels the product is better	JD.com
Night Prairie Life	Battery Reconditioner	I used it once last night and it didn't get hot all night charging, not bad, looking forward to the back of the	JD.com
Night Prairie Life	Battery Reconditioner	Nice and fast logistics, haven't used it yet, hope it works.	JD.com
Night Prairie Life	Battery Reconditioner	and got50km, which is1/4increasecompared to the original which was only40km. don't look at it as a big thing, but it does work.	JD.com
Night Prairie Life	Battery Reconditioner	The quality is very good and fine workmanship will be back to buy Thank you boss is genuine!	JD.com
Night Prairie Life	Battery Reconditioner	This user did not fill in a rating	JD.com
Night Prairie Life	Battery Reconditioner	[Chuckles]It doesn't work	JD.com
Night Prairie Life	Battery Reconditioner	Used a little bit is okay, first give a5stars, after a few days to use again to see after, then come back to the comment	JD.com
Night Prairie Life	Battery Reconditioner	This user did not fill in a rating	JD.com
Night Prairie Life	Battery Reconditioner	Looks good, complete packaging and fast logistics.	JD.com
Night Prairie Life	Battery Reconditioner	Baby quality is good, repair function is there, but too slow, logistics is very fast, good service attitude	JD.com
Night Prairie Life	Battery Reconditioner	Comment later!	JD.com
Night Prairie Life	Battery Reconditioner	Very good quality and effective	JD.com
Night Prairie Life	Battery Reconditioner	fully charged, in the repair machine repair, repair process no sound, is the red light green light flashing at the same time, the business said24hours, a month to repair once, waiting for a miracle	JD.com
Night Prairie Life	Battery Reconditioner	(of an unmarried couple) be close	JD.com
Night Prairie Life	Battery Reconditioner	Shipping and logistics are very block, the appearance is also good not yet had time to use, after using and then praise.	JD.com
Night Prairie Life	Battery Reconditioner	There is a little effect also used	JD.com
Night Prairie Life	Battery Reconditioner	Used with effect, back to help relatives to buy, recommendable!	JD.com
Night Prairie Life	Battery Reconditioner	Bought received, well-packaged, logistics is fast, the price is also very affordable, if it is good, will give follow-up comments, thanks to the seller!	JD.com
Night Prairie Life	Battery Reconditioner	has a restorative effect on the battery, which is worth buying.	JD.com
Night Prairie Life	Battery Reconditioner	It doesn't seem to be working. Try it again.	JD.com
Night Prairie Life	Battery Reconditioner	and repair batteries, multi-purpose, affordable and convenient.	JD.com
Night Prairie Life	Battery Reconditioner	I'll comment after using it.	JD.com
Night Prairie Life	Battery Reconditioner	The workmanship looks good, I haven't actually used it yet, so I'll follow up after I've used it a few times!	JD.com
Night Prairie Life	Battery Reconditioner	Originally is not much evaluation, but the product effect is really good, and is more niche, more hope that this kind of useful products can be more people know, recommended	JD.com
Night Prairie Life	Battery Reconditioner	It doesn't help, people buy tight	JD.com
Night Prairie Life	Battery Reconditioner	I don't really feel it. It's like it's so-so.	JD.com
Night Prairie Life	Battery Reconditioner	Try it and keep it if it works well, return it if it doesn't, and review it later[smile]	JD.com
Night Prairie Life	Battery Reconditioner	Used to evaluate the later, the quality is very good, effective, we can rest assured that the order	JD.com
Night Prairie Life	Battery Reconditioner	electricity, repair more than four hours, it is fixed, and then I am full again to repair, with the use of really can, great!	JD.com
Night Prairie Life	Battery Reconditioner	There is a restorative effect.	JD.com
Night Prairie Life	Battery Reconditioner	Not yet used	JD.com
Night Prairie Life	Battery Reconditioner	Function: reliable and amazingly effective.	JD.com
Night Prairie Life	Battery Reconditioner	My battery starved to death for over half a month and was completely repaired	JD.com
Night Prairie Life	Battery Reconditioner	Received it, looking forward to using it!	JD.com
Night Prairie Life	Battery Reconditioner	This user feels the product is better	JD.com

Night Prairie Life	Battery Reconditioner	Evaluation after use. battery starvedmore than2weeks, the charger could not charge, the repairer received, immediately used, repair charging for15hours, and then charged with the original charger, the battery back to normal. Very good!	JD.com
Night Prairie Life	Battery Reconditioner	It's genuine, so don't worry about buying it.	JD.com
Night Prairie Life	Battery Reconditioner	The product has been received, not yet used, used to comment again	JD.com
Night Prairie Life	Battery Reconditioner	Experimented with three sets of batteries have no effect at all. Before buying on the Internet checked a few dozen dollars of no effect, holding the mindset of trying now believe it	JD.com
Night Prairie Life	Battery Reconditioner	Fast logistics and practical.	JD.com
Night Prairie Life	Battery Reconditioner	Starvation battery can be activated	JD.com
Night Prairie Life	Battery Reconditioner	Repair of the battery arrived, customer service attentive to solve the problem, for the service attitude praise, repair effect later comment[smile]	JD.com
Night Prairie Life	Battery Reconditioner	The baby is really good, the starved battery activated and working properly	JD.com
Night Prairie Life	Battery Reconditioner	Used for a long time, to comment on it, I hope to help people who need it, do a reference. Newly purchased electric car because of the epidemic has not been riding also did not charge the results of battery starvation, see this charger holding a dead horse as a doctor's idea of the order, did not look carefully to buy a universal results back to see the head does not match the merchants, the merchants told me to buy a converter online3yuan, back to charge theresults of the24hours is still no electricity and ask the merchants, the merchants told me to recharge and charged again24hours as a result of batteryWell, and new as durable, really incredible, are going to replace the new battery, the results of the repair is good, save money[kudos]Thank you merchants, business is bo[clasp]	JD.com
Night Prairie Life	Battery Reconditioner	Friends recommended the purchase, at first not quite believe, used for a period of time after the real effect, which came to the evaluation	JD.com
Night Prairie Life	Battery Reconditioner	Electric car battery repairer received. Fast logistics and good service! Give a five-star praise!1	JD.com
Night Prairie Life	Battery Reconditioner	This user did not fill in a rating	JD.com
Night Prairie Life	Battery Reconditioner	I can't see the effect yet! We won't know until after a while!	JD.com
Night Prairie Life	Battery Reconditioner	Let's see if it works.	JD.com
Night Prairie Life	Battery Reconditioner	Can be saved, charging depends on the original charger	JD.com
Night Prairie Life	Battery Reconditioner	[big love] [big love] [big]love]big[love] [big love] [big love] [biglove]	JD.com
Night Prairie Life	Battery Reconditioner	batteryafterplugging in24hours, good product. [Kudos] [Kudos] [Kudos]	JD.com
Night Prairie Life	Battery Reconditioner	The battery has been used for a long time, quickly run out of power, after using the restorer can be opened for a long time, not afraid of not durable	JD.com
Night Prairie Life	Battery Reconditioner	It works.	JD.com
Night Prairie Life	Battery Reconditioner	It just happened that the family electric car battery used a long time is not very durable, buy the repair of the god after the battery does not have to change, save a lot of money!	JD.com
Night Prairie Life	Battery Reconditioner	I'll comment after I've used it	JD.com
Night Prairie Life	Battery Reconditioner	[Big Love]	JD.com
Night Prairie Life	Battery Reconditioner	I'll comment after I've used it.	JD.com
Night Prairie Life	Battery Reconditioner	The stuff works.	JD.com
Night Prairie Life	Battery Reconditioner	Received yesterday's goods a convenient good stool, there is a clear role in the minimum increase of20%or more, good quality - cheap price, logistics is very fast, the service is also very on point - satisfied	JD.com
Night Prairie Life	Battery Reconditioner	I have received the goods and I will use it again. If it's good, I'll keep it. If it's bad, I'll return it.	JD.com
Night Prairie Life	Battery Reconditioner	This user feels the product is better	JD.com
Night Prairie Life	Battery Reconditioner	The restorer was received and feels good and worth having.	JD.com
Night Prairie Life	Battery Reconditioner	The item is as described, inexpensive and will be back next time.	JD.com

Night Prairie Life	Battery Reconditioner	I've been using it for a while and the results have really improved and the after-sales service is very good	JD.com
Night Prairie Life	Battery Reconditioner	Although the item has been received, I haven't used it yet, and I just saw that it's another $2a piececheaper. 38when I bought it, now it's only$36.	JD.com
Night Prairie Life	Battery Reconditioner	You'll know when you've used it.	JD.com
Night Prairie Life	Battery Reconditioner	Can fix starved batteries	JD.com
Night Prairie Life	Battery Reconditioner	Good product deserves more promotion, hope more people use it on	JD.com
Night Prairie Life	Battery Reconditioner	Fine workmanship	JD.com
Night Prairie Life	Battery Reconditioner	Great quality and great results, worth buying!	JD.com
Night Prairie Life	Battery Reconditioner	Baby to receive, with the supermarket to sell the same, fine workmanship, electric car repair fast, simple packaging, practical, good value for money!	JD.com
Night Prairie Life	Battery Reconditioner	The battery starved to death with this fix for days and now it finally charges properly[hee][hee][hee hee][hee hee][hee	JD.com
Night Prairie Life	Battery Reconditioner	For safety reasons, charging the electric car do not let it catch fire, fully charged automatically will trip the battery, this can make the battery can be repaired, can let him time to live a little longer?	JD.com
Night Prairie Life	Battery Reconditioner	This battery repair is really good, my battery is a loss of power for half a month, the charger can not into the power, after24hours of repair, the battery is full again, really good	JD.com
Night Prairie Life	Battery Reconditioner	I hope it works out well!	JD.com
Night Prairie Life	Battery Reconditioner	The battery is three years old and performs great, it's just that the battery isn't working. The original can use two days, now less than a day. I saw this product from the Internet, the response is good, buy to try, the effect is really good!	JD.com
Night Prairie Life	Battery Reconditioner	dissatisfied	JD.com
Night Prairie Life	Battery Reconditioner	This user feels the product is better	JD.com
Night Prairie Life	Battery Reconditioner	Mine are broken.	JD.com
Night Prairie Life	Battery Reconditioner	I have only used it for the past two days, and it looks okay for the time being, so I will wait for three to five weeks to see the actual results.	JD.com
Night Prairie Life	Battery Reconditioner	Good product, recommendable, very good results and good service!	JD.com
Night Prairie Life	Battery Reconditioner	a long time to find this product, holding a try mentality, I did not expect to repair successfully, has been normal charging use, do not have to spend hundreds of dollars to replace the battery[cool]	JD.com
Night Prairie Life	Battery Reconditioner	I haven't used it yet.	JD.com
Night Prairie Life	Battery Reconditioner	charged. Bought this repair24hours, the repair is complete, it can be charged normally, now the electricity is full. Need to buy.	JD.com
Night Prairie Life	Battery Reconditioner	Repair is very good, and customer service is very patient answer, good luck with business!	JD.com
Night Prairie Life	Battery Reconditioner	It feels like it's working.	JD.com
Night Prairie Life	Battery Reconditioner	It's okay, waiting for results!	JD.com
Night Prairie Life	Battery Reconditioner	Good baby, fast logistics, good buyer service, thank you, and will come back again!	JD.com
Night Prairie Life	Battery Reconditioner	Quite good, the starved battery after repair charger charging can be normal charging again, and save a battery replacement money[praise] [praise] [praise] [praise] [praise]	JD.com
Night Prairie Life	Battery Reconditioner	as described by customer service, to see the effect and then follow up.	JD.com
Night Prairie Life	Battery Reconditioner	Works great. Got the battery activated.	JD.com
Night Prairie Life	Battery Reconditioner	Goods have been received, have not yet used, for the time being, do not evaluate	JD.com
Night Prairie Life	Battery Reconditioner	effective	JD.com
Night Prairie Life	Battery Reconditioner	I've read that he's a few dollars cheaper than others, so you're right to buy him, buy with confidence	JD.com
Night Prairie Life	Battery Reconditioner	It's still a bit easy to see, I'll let you know when I've used it.	JD.com

Night Prairie Life	Battery Reconditioner	before, this time you do not have to spend a few hundred to change the battery, every day to work to run more than ten kilometers of road, almost every year to change the battery, buy this will not have to change the battery, and can save a few hundred.	JD.com
Night Prairie Life	Battery Reconditioner	not very useful	JD.com
Night Prairie Life	Battery Reconditioner	under test	JD.com
Night Prairie Life	Battery Reconditioner	Through many tests, or effective, tested two sets of batteries, older batteries, through two super24hours of restoration, walking mileage increasedmore than20%	JD.com
Night Prairie Life	Battery Reconditioner	Goods received, good business service attitude, delivery, logistics are very fast, after receiving has not yet begun to use, looking forward to the same effect with the business publicity, to give praise!	JD.com
Night Prairie Life	Battery Reconditioner	serviceable	JD.com
Night Prairie Life	Battery Reconditioner	Not yet used, only after the use of the effect of how to know the	JD.com
Night Prairie Life	Battery Reconditioner	I've received it and tried it. I hope it works.	JD.com
Night Prairie Life	Battery Reconditioner	The customer service guide is very patient and also has an after-sales guarantee, so it should be good	JD.com
Night Prairie Life	Battery Reconditioner	It's working. The battery runs longer.	JD.com
Night Prairie Life	Battery Reconditioner	Received the product and haven't used it yet! I don't know if the quality assurance works?	JD.com
Night Prairie Life	Battery Reconditioner	The customer service is very good, there are questions and answers, the key product effect is really good!	JD.com
Night Prairie Life	Battery Reconditioner	death, can not charge, in your store to buy a battery repairer, I received immediately after the repair of the battery, the whole3days, under the guidance of the boss, last night with the original charger can be charged, the more fruit is good.	JD.com
Night Prairie Life	Battery Reconditioner	This is really useful for battery activation, now the battery car can be charged properly, good stuff!	JD.com
Night Prairie Life	Battery Reconditioner	Fast logistics, business description carefully. The product is small and exquisite, automatic battery car repairer, to be tried and then follow up the effect.	JD.com
Night Prairie Life	Battery Reconditioner	Use it for a couple days and then follow up	JD.com
Night Prairie Life	Battery Reconditioner	I thought it was wired, but it's not.	JD.com
Night Prairie Life	Battery Reconditioner	The stuff works.	JD.com
Night Prairie Life	Battery Reconditioner	I'll comment after I've used it.	JD.com
Night Prairie Life	Battery Reconditioner	Business description is very detailed, and the logistics is very fast, recommended to buy!	JD.com
Night Prairie Life	Battery Reconditioner	I hope it works.	JD.com
Night Prairie Life	Battery Reconditioner	very good	JD.com
Night Prairie Life	Battery Reconditioner	Logistics is very fast, the product appearance is very good, and the operation is simple and effective!	JD.com
Night Prairie Life	Battery Reconditioner	It works well, and you can save a lot of money with the product!	JD.com
Night Prairie Life	Battery Reconditioner	Fast shipping, well packaged. Have not yet used it, do not know how the quality is, later follow-up evaluation.	JD.com
Night Prairie Life	Battery Reconditioner	Great customer service, patient and very satisfied!	JD.com
Night Prairie Life	Battery Reconditioner	Used more than half a year, because they accidentally lost, with the business actually gave me a lot of discounts, and then bought a, really thank the business service after sale!	JD.com
Night Prairie Life	Battery Reconditioner	Baby received the same as sold outside I like it very much next time to come back to the store to buy.	JD.com
Night Prairie Life	Battery Reconditioner	Exquisite workmanship, good quality, the effect is very good, the key is affordable, the purchase is well worth it!	JD.com
Night Prairie Life	Battery Reconditioner	After using the repairer the battery lasts a lot longer, good and recommendable!	JD.com
Night Prairie Life	Battery Reconditioner	Baby received, really great ah! Quality is really nothing to say, very satisfied! Happy happy baby and the description is basically the same, fast delivery, worth a buy!	JD.com
Night Prairie Life	Battery Reconditioner	introduced to use the effect is very good. Customer service attitude is very good. A very satisfactory online shopping. Next time will buy back.	JD.com
Night Prairie Life	Battery Reconditioner	finally activated after58hoursuseand can now be charged in with the original charger, good review!	JD.com

Night Prairie Life	Battery Reconditioner	Very good quality, very satisfied with the service and answered all questions	JD.com
Night Prairie Life	Battery Reconditioner	This user feels the product is better	JD.com
Night Prairie Life	Battery Reconditioner	Not bad.	JD.com
Night Prairie Life	Battery Reconditioner	Good quality.	JD.com
Night Prairie Life	Battery Reconditioner	repurchase discount, recommended to relatives have bought, all praise good!	JD.com
Night Prairie Life	Battery Reconditioner	It worked for about48hours, you can use the charge normally	JD.com
Night Prairie Life	Battery Reconditioner	Just received the use of the feeling of a slight enhancement, the product design is still quite simple, very easy to operate	JD.com
Night Prairie Life	Battery Reconditioner	good, logistics is also very fast, has been used twice, working normally, electric car battery has been more than a year, the range is obviously reduced, but the repair will take a period of time, looking forward to using4a5weeks after the obvious results.	JD.com
Night Prairie Life	Battery Reconditioner	You can charge it normally after about48hours ofrepair, the repairer works well.	JD.com
Night Prairie Life	Battery Reconditioner	logistics is also very fast, has been used twice, plugged in the power supply to press the repair switch began to work, the electric car inside the battery has been used for more than a year, the range is significantly reduced, looking forward to using the restorer for a period of time after the repair effect can be unexpected.	JD.com
Night Prairie Life	Battery Reconditioner	The logistics is also very fast	JD.com
Night Prairie Life	Battery Reconditioner	straight away, my ebikehadn't been charged for5months and was activated successfully with this.	JD.com
Night Prairie Life	Battery Reconditioner	Mine are broken.	JD.com
Night Prairie Life	Battery Reconditioner	This is a scam. It doesn't work.	JD.com
Night Prairie Life	Battery Reconditioner	Works but not much. The activation did activate but the battery was bad. Merchant wouldn't take care of it, nor did the platform, and finally refunded me$28.[Skimming]	JD.com
Night Prairie Life	Battery Reconditioner	Poor functionality.	JD.com
Night Prairie Life	Battery Reconditioner	This restorer works well, it is stable and the restoration effect is good, Highlight! Worth buying.	JD.com
Night Prairie Life	Battery Reconditioner	run2kilometers after I give the battery three days of repair now runmore than10kilometers really good must be five-star praise	JD.com
Night Prairie Life	Battery Reconditioner	Received the goods, looks pretty good, wait for me to use once and then follow up reviews	JD.com
Night Prairie Life	Battery Reconditioner	It actually fixed my starving battery. I don't know how.	JD.com
Night Prairie Life	Battery Reconditioner	The point is useless, charged all night without power, get another one up, I just rushed to show that it was only20%after charging all nightshows that that must not be	JD.com
Night Prairie Life	Battery Reconditioner	Pro, the loan received, the packaging is intact, the logistics is very fast, the boss service attitude is also quite good, it is an ideal shopping, thank you boss good products, I wish you prosperous business!	JD.com
Night Prairie Life	Battery Reconditioner	Received it, not sure if I can successfully fix the starved battery yet	JD.com
Night Prairie Life	Battery Reconditioner	I got it! Thank you boss for shipping the goods, awesome!	JD.com
Night Prairie Life	Battery Reconditioner	Not a TO, it does activate the starvation battery.	JD.com
Night Prairie Life	Battery Reconditioner	Automatic trolley repairer received, plugged in to try, feel the effect is quite good, observe on a few days, the effect is obvious, then continue to follow up!	JD.com
Night Prairie Life	Battery Reconditioner	Logistics is fast, the product is intact is very satisfied.	JD.com
Night Prairie Life	Battery Reconditioner	Received the goods, is in the trial period. Let's see how it is, I'll comment after I use it. Can not say good or bad.	JD.com
Night Prairie Life	Battery Reconditioner	It's good. It works.	JD.com
Night Prairie Life	Battery Reconditioner	What is this? Nothing. Don't fall for it.	JD.com
Night Prairie Life	Battery Reconditioner	The commodity收到物流很快外观质量精致充电修复电瓶效果等用过再来评价先给一个五星好评吧，请问客服，智能充电器能用吗？	JD.com
Night Prairie Life	Battery Reconditioner	I received it today and I'm in the process of fixing it. I'll comment in a few days.	JD.com

Night Prairie Life	Battery Reconditioner	This user feels the product is better	JD.com
Night Prairie Life	Battery Reconditioner	This user feels the product is better	JD.com
Night Prairie Life	Battery Reconditioner	This user feels the product is better	JD.com
Night Prairie Life	Battery Reconditioner	Not yet used, help a friend to buy, there is a mission statement, good use and then follow up!	JD.com
Night Prairie Life	Battery Reconditioner	This user feels the product is better	JD.com
Night Prairie Life	Battery Reconditioner	This user feels the product is better	JD.com
Night Prairie Life	Battery Reconditioner	I've got it. Let's see how it works.	JD.com
Night Prairie Life	Battery Reconditioner	The restorer was received, well packaged, and the logistics were fast!	JD.com
Night Prairie Life	Battery Reconditioner	Good quality will buy back	JD.com
Night Prairie Life	Battery Reconditioner	Received the item, haven't used it yet, hope it's as described, looking forward to the results.	JD.com
Night Prairie Life	Battery Reconditioner	Product quality is very good product price is cheap hit five	JD.com
Night Prairie Life	Battery Reconditioner	How come it took12hours to fill up?	JD.com
Night Prairie Life	Battery Reconditioner	Safety: charging speed, baby received, logistics is also very fast, packaging is very good, five-star praise, shipping is also very fast, genuine, I do not know how effective?	JD.com
Night Prairie Life	Battery Reconditioner	seller said in the beginning of the consultation. A perfect shopping.	JD.com
Night Prairie Life	Battery Reconditioner	can not charge into the electricity, bought this charger, charging for a few days, really fix it, too powerful,. From now on, just follow the instructions to use to give the battery range. Thank you boss.	JD.com
Night Prairie Life	Battery Reconditioner	This user feels the product is better	JD.com
Night Prairie Life	Battery Reconditioner	evaluate, used to evaluate, repair effect is very good, my three electric car are repairedmore than2ten hours, straight praise.	JD.com
Night Prairie Life	Battery Reconditioner	Things are very good, so far the car has been restored to use, the boss is also very enthusiastic, timely response, the car four months do not ride, the battery is starved to death, with this charger, according to the boss's method, so far it has been full-blooded revived!	JD.com
Night Prairie Life	Battery Reconditioner	The battery is relatively new, starved battery repair came back to life4hours, the original charger lit up red again, what a great product!	JD.com
Night Prairie Life	Battery Reconditioner	It's nice and compact, you'll have to try it out to see how it works.	JD.com
Night Prairie Life	Battery Reconditioner	Not bad.	JD.com
Night Prairie Life	Battery Reconditioner	The restorer was received, inexpensive, restored, and brought the starved battery back to life in one night!	JD.com
Night Prairie Life	Battery Reconditioner	To tell you the truth, it's not bad.	JD.com
Night Prairie Life	Battery Reconditioner	It's easy to use, you just don't see the effect at the time, you don't know if it's working or not until after a long period of time. I have replaced the batteries with new ones	JD.com
Night Prairie Life	Battery Reconditioner	Picked up the goods and used it, looking forward to the effect, customer service reply promptly, praise.	JD.com
Night Prairie Life	Battery Reconditioner	It feels okay. It works.	JD.com
Night Prairie Life	Battery Reconditioner	Fast shipping, well packaged, works well with the right fit and effect	JD.com
Night Prairie Life	Battery Reconditioner	Fine workmanship, easy to use, beautiful appearance, just received has not been used, looking forward to the effect.	JD.com
Night Prairie Life	Battery Reconditioner	This battery activator works pretty well, so feel free to get it if you want.	JD.com
Night Prairie Life	Battery Reconditioner	Lead-acid battery repairer received for a long time, used before coming to comment, the repair effect is good, after repairing the battery lasting strong, praise!	JD.com
Night Prairie Life	Battery Reconditioner	Works great. Charges comfortably and quickly. Automatically cuts off when fully charged	JD.com
Night Prairie Life	Battery Reconditioner	The logistics is fast, the price is reasonable, and the effect will be followed up after using it	JD.com
Night Prairie Life	Battery Reconditioner	Good, very durable, very satisfied with a shopping, have recommended to friends	JD.com
Night Prairie Life	Battery Reconditioner	I received the charger, it looks very nice, I can see that the quality is good!	JD.com

Night Prairie Life	Battery Reconditioner	This user feels the product is better	JD.com
Night Prairie Life	Battery Reconditioner	The stuff works well and the logistics are fast and the boss is very good too!	JD.com
Night Prairie Life	Battery Reconditioner	Use it for a while and come back with a favorable review.	JD.com
Night Prairie Life	Battery Reconditioner	light, affixed with a seal, but the starved battery charged15hours with the original can be charged, good use on the line, the video is the original charging can not be charged into the electricity, in a few days to try to repair the other old electric chicken how.	JD.com
Night Prairie Life	Battery Reconditioner	I haven't used it yet. I'll buy it again if it's good.	JD.com
Night Prairie Life	Battery Reconditioner	not bad	JD.com
Night Prairie Life	Battery Reconditioner	Open the packaging product quality is good. Fine workmanship, genuine, to be used after further praise.	JD.com
Night Prairie Life	Battery Reconditioner	Works great, the battery activates straight away	JD.com
Night Prairie Life	Battery Reconditioner	Good service too[Smiles]	JD.com
Night Prairie Life	Battery Reconditioner	Seller shipped quickly and well packaged. Satisfied. All five stars!	JD.com
Night Prairie Life	Battery Reconditioner	month really repair success, before the electric car can only run20kilometers, after a month of use yesterday to run a round trip to Shenzhen27kilometers of road only half of the electricity, last night to help the dance friends under a single, hope that his also used, I wish the boss a prosperous business	JD.com
Night Prairie Life	Battery Reconditioner	Boss in? Things received, get back at night to insert the battery, an electric original switch, the red light flashes a flash to start working, very its to be used after the effect.	JD.com
Night Prairie Life	Battery Reconditioner	I received the stuff, try to see how well it works, and see if starving the battery for three months might fix it!	JD.com
Night Prairie Life	Battery Reconditioner	Can fix starved batteries	JD.com
Night Prairie Life	Battery Reconditioner	serviceable	JD.com
Night Prairie Life	Battery Reconditioner	It's very good, I'll come back again, mamma mia!	JD.com
Night Prairie Life	Battery Reconditioner	Thank you very much, very happy with it! Battery starved2times (this time for the second time), fixed it for24hoursyesterday to todayand now it's charging fine! Must praise! Restoring other ebikes again to see if I can extend the usage time!	JD.com
Night Prairie Life	Battery Reconditioner	Logistics is fast! It looks great, so I'll give it a favorable review!	JD.com
Night Prairie Life	Battery Reconditioner	Noise is small, big fine, small as the size of a cigarette box, with or without effect to be used and then evaluate.	JD.com
Night Prairie Life	Battery Reconditioner	It's great, no toggle switch, foolproof operation, and the power does improve after trying it[clapping]	JD.com
Night Prairie Life	Battery Reconditioner	[Lots of love] [Lof[Lotslove][Lots[LotsLotsloveof] [Lots of love] [Lotsoflove]]	JD.com
Night Prairie Life	Battery Reconditioner	The quality is great.	JD.com
Night Prairie Life	Battery Reconditioner	Quality is very good, with the seller's description of the exact same, very satisfied, really like it, completely exceeded expectations, the delivery speed is very fast, the packaging is very careful and strict, the logistics company has a very good service attitude, the delivery speed is very fast, very satisfied with a shopping.	JD.com
Night Prairie Life	Battery Reconditioner	than thirty hours of restoration! The battery charge really lasts longer! Personally, I think it's worth a try! After all, it is more affordable than replacing the battery with a new one.	JD.com
Night Prairie Life	Battery Reconditioner	The logistics company is very powerful Use it and then come back to the follow-up evaluation	JD.com
Night Prairie Life	Battery Reconditioner	Not yet used, evaluation after use	JD.com
Night Prairie Life	Battery Reconditioner	I don't know how effective it is, the indicator light is not on, let's charge it first to see if it can be activated, I'm a little worried, it's been parked for too long[sad]	JD.com
Night Prairie Life	Battery Reconditioner	in use	JD.com
Night Prairie Life	Battery Reconditioner	Fast logistics and practical.	JD.com
Night Prairie Life	Battery Reconditioner	Not yet used, the effect should be good, if there is a problem in the follow-up comment!	JD.com

Night Prairie Life	Battery Reconditioner	serviceable	JD.com
Night Prairie Life	Battery Reconditioner	Looks good, hope it works, thanks!	JD.com
Night Prairie Life	Battery Reconditioner	Fast shipping logistics is also very fast, the quality of goods is good with a good use of the role!	JD.com
Night Prairie Life	Battery Reconditioner	Haven't used it yet, posting a picture to fill in the blanks.	JD.com
Night Prairie Life	Battery Reconditioner	Good stuff, waiting for the effect, easy to operate, I believe it will be good results	JD.com
Night Prairie Life	Battery Reconditioner	Received the baby, have not yet used it, expect to be useful	JD.com
Night Prairie Life	Battery Reconditioner	About the same as the charger cable.	JD.com
Night Prairie Life	Battery Reconditioner	I haven't used it yet, I'll tell you what I think after I've tried it	JD.com
Night Prairie Life	Battery Reconditioner	Electric donkey battery starved to death, starved to death for five months, bought this charger, Kaka activated, very happy, save money to change the battery, really thank you boss, thank you for this piece of activator, kudos, recommend that everyone buy, really good, the	JD.com
Night Prairie Life	Battery Reconditioner	The quality is very over the top, quality is guaranteed, quite a good choice, a lot of friends see me use all want to buy, I have recommended.	JD.com
Night Prairie Life	Battery Reconditioner	Battery activation successful, charger is very powerful, the small electric donkey can run again, haha, boss, people are good, after-sales very good, very powerful	JD.com
Night Prairie Life	Battery Reconditioner	Goods beautifully designed logistics very fast business service attitude is good	JD.com
Night Prairie Life	Battery Reconditioner	Battery repairer small, exquisite workmanship, easy to use, the battery does have voltage stabilization, repair role, the price is not expensive, worth buying.	JD.com
Night Prairie Life	Battery Reconditioner	quality is very good! Goods real price! Really good use ah! Very convenient and practical ah! Very satisfied ah! Halla less!	JD.com
Night Prairie Life	Battery Reconditioner	Specially used a few days to evaluate, the effect can indeed be used for a few days, the feeling of the battery is stronger, I hope that a longer time has a better effect, after all, change the battery is too uneconomical!	JD.com
Night Prairie Life	Battery Reconditioner	fixing it. Started to have a little problem and customer service caught it and fixed it in a timely manner! Thank you very much!	JD.com
Night Prairie Life	Battery Reconditioner	Really want to return, and feel sorry conscience, other people's electric car last winter December in my garage, seven months without charging battery loss. Buy this object and the boss agreed that if the excitement does not live can be returned, the results of a miracle, after 44 hours of repair, finally able to use the original charger to charge, but have to buy a conversion head, modern technology genuine good!	JD.com
Night Prairie Life	Battery Reconditioner	Very satisfied, good quality and beautiful!	JD.com
Night Prairie Life	Battery Reconditioner	Really activated! Charged the repair charger charging almost exactly 24 hours, arrived home from work and tried to light up, direct replacement of the regular charger charging, tomorrow morning you can ride your bike to work ~	JD.com
Night Prairie Life	Battery Reconditioner	results of the test, after two days and two nights of charging repair, finally my eight-month battery repair success, cheap and easy to use, and the business description of the same, searched for a lot of homes in Jindo are several hundred dollars, this is the cheapest one, can repair the lead-acid batteries, the long term does not charge charge can not charge a good product ，，，，，，，	JD.com
Night Prairie Life	Battery Reconditioner	'Just received the goods on the chase as soon as possible to plug in the electric car.soon as I pressed the power switch, the red pulse light flashed and started to work, and I was looking forward to the effect after I made it	JD.com
Night Prairie Life	Battery Reconditioner	Electric car full of electricity to run the road obviously less mileage, the battery with almost two years, online to see the store commitment, hold the heart to try, I believe it can have an effect, just received this product today, after using in the follow-up evaluation!	JD.com
Night Prairie Life	Battery Reconditioner	repair, can be charged normally, great [love] [love] [love] [love], with dozens of dollars to repair hundreds of dollars of battery, very satisfied, good things worth having and recommend to good friends [smile] [smile] [smile] [smile], next time to continue to buy other things in his family [smile] [smile] [smile] [smile].	JD.com

Fei Fan Goodies Preferred	Battery Reconditioner	Only those with high IQs can buy it.	JD.com
Fei Fan Goodies Preferred	Battery Reconditioner	ÔÇ£Please, my friends, donÔÇÖt buy any more.	JD.com
Fei Fan Goodies Preferred	Battery Reconditioner	**	JD.com
Fei Fan Goodies Preferred	Battery Reconditioner	This user feels the product is better	JD.com
Fei Fan Goodies Preferred	Battery Reconditioner	Now I know how to charge it, the rest of the restoration junk I don't understand!	JD.com
Fei Fan Goodies Preferred	Battery Reconditioner	This user feels the product is better	JD.com
Fei Fan Goodies Preferred	Battery Reconditioner	differ from	JD.com
Fei Fan Goodies Preferred	Battery Reconditioner	This user feels the product is better	JD.com
Fei Fan Goodies Preferred	Battery Reconditioner	I want to return this item if I'm not happy with it, it doesn't work well	JD.com
Fei Fan Goodies Preferred	Battery Reconditioner	This user feels the product is better	JD.com
Fei Fan Goodies Preferred	Battery Reconditioner	This user feels the product is better	JD.com
Fei Fan Goodies Preferred	Battery Reconditioner	Affordable, powerful, beautifully designed, great shape, fast charging, very practical, aesthetically pleasing, very practical, great shape	JD.com
Fei Fan Goodies Preferred	Battery Reconditioner	I bought it and haven't used it yet, so I'll give it a good review first!	JD.com
Fei Fan Goodies Preferred	Battery Reconditioner	This user feels the product is better	JD.com
Fei Fan Goodies Preferred	Battery Reconditioner	This user feels the product is better	JD.com
Fei Fan Goodies Preferred	Battery Reconditioner	This user feels the product is better	JD.com
Fei Fan Goodies Preferred	Battery Reconditioner	This user feels the product is better	JD.com
Fei Fan Goodies Preferred	Battery Reconditioner	This user feels the product is better	JD.com
Fei Fan Goodies Preferred	Battery Reconditioner	The item is complete, I don't know how well it works, I'll wait and see. Tentative four stars.	JD.com
Fei Fan Goodies Preferred	Battery Reconditioner	Received. Unknown if it works well or not. I'm using it.	JD.com
Fei Fan Goodies Preferred	Battery Reconditioner	Not bad!	JD.com
Fei Fan Goodies Preferred	Battery Reconditioner	This user feels the product is better	JD.com
Fei Fan Goodies Preferred	Battery Reconditioner	This user feels the product is better	JD.com
Fei Fan Goodies Preferred	Battery Reconditioner	I got it.	JD.com
Fei Fan Goodies Preferred	Battery Reconditioner	This user feels the product is better	JD.com
Fei Fan Goodies Preferred	Battery Reconditioner	Haven't used it yet, to be evaluated	JD.com
Fei Fan Goodies Preferred	Battery Reconditioner	It doesn't work very well.	JD.com
Fei Fan Goodies Preferred	Battery Reconditioner	Goods are real, business service is very good logistics is also very fast, straight to trust!	JD.com
Fei Fan Goodies Preferred	Battery Reconditioner	Baby received, logistics is very fast, the service attitude is very good!	JD.com
Fei Fan Goodies Preferred	Battery Reconditioner	The logistics is very fast, worth repurchasing, and I will come back next time.	JD.com
Fei Fan Goodies Preferred	Battery Reconditioner	Beautifully designed, clear to use, economical and very effective.	JD.com
Fei Fan Goodies Preferred	Battery Reconditioner	space<br>Product design:'new style, good visibility, cost-effective<br>Charging function: fast, practical, really able to repair cell phone batteries	JD.com
Fei Fan Goodies Preferred	Battery Reconditioner	Great shape and practical	JD.com
Fei Fan Goodies Preferred	Battery Reconditioner	Good quality and charges much faster.	JD.com
Fei Fan Goodies Preferred	Battery Reconditioner	The logistics is fast and the goods are cheap	JD.com
Fei Fan Goodies Preferred	Battery Reconditioner	The goods have been received, logistics is very fast, is using, the effect is good then buy!	JD.com
Fei Fan Goodies Preferred	Battery Reconditioner	It is being used and fixes cell phone batteries.	JD.com

Fei Fan Goodies Preferred	Battery Reconditioner	Baby quality is very good, not only small, beautiful and generous, but also inexpensive. Got it back and tried it out, it's really good. The seller shipped quickly, logistics is strong, five-star praise, the next time to patronize!	JD.com
Fei Fan Goodies Preferred	Battery Reconditioner	Good results, fast logistics and good service!	JD.com
Fei Fan Goodies Preferred	Battery Reconditioner	Fast delivery and already in use.	JD.com
Fei Fan Goodies Preferred	Battery Reconditioner	Express speed, complete packaging, something small, can help the phone battery is really worth buying. Waiting to try down to see if the battery is strong enough, I hope the black technology can make a difference.	JD.com
Fei Fan Goodies Preferred	Battery Reconditioner	Goods to receive, good quality, inexpensive, logistics is also very fast satisfaction.	JD.com
Fei Fan Goodies Preferred	Battery Reconditioner	Consistent with the description, fast logistics, good service, after using the product, charging is much better than when not in use, charging time narrowed down a bit, value for money	JD.com
Fei Fan Goodies Preferred	Battery Reconditioner	Received the cell phone adapter, can not wait to fix on the logistics is very fast, good value for money, after use and then comment!	JD.com
Fei Fan Goodies Preferred	Battery Reconditioner	I've received it. I haven't used it yet.	JD.com
Fei Fan Goodies Preferred	Battery Reconditioner	Goods have received a very good use, can repair the battery, than before the power lasting, only used once quite good, thanks to the business.	JD.com
Fei Fan Goodies Preferred	Battery Reconditioner	Things received, the operation is simple and practical, logistics is also very strong, satisfied!	JD.com
Mokoyi Digital Selection	Electric Vehicle Repair	That's good!	JD.com
Mokoyi Digital Selection	Electric Vehicle Repair	This user feels the product is better	JD.com
Mokoyi Digital Selection	Electric Vehicle Repair	This user feels the product is better	JD.com
Mokoyi Digital Selection	Electric Vehicle Repair	very timely, thanks to the platform to provide such a good product.	JD.com
Mokoyi Digital Selection	Electric Vehicle Repair	Fast logistics, beautiful appearancel, the effect does not know, use and then evaluate	JD.com
Mokoyi Digital Selection	Electric Vehicle Repair	Do not evaluate the good or bad for the time being, after using it, then evaluate	JD.com
Mokoyi Digital Selection	Electric Vehicle Repair	This user feels the product is better	JD.com
Mokoyi Digital Selection	Electric Vehicle Repair	acceptable	JD.com
Mokoyi Digital Selection	Electric Vehicle Repair	This user feels the product is better	JD.com
Mokoyi Digital Selection	Electric Vehicle Repair	Baby received! Used it straight away when I got home! Let's see how it works out! Will also follow up the review!	JD.com
Mokoyi Digital Selection	Electric Vehicle Repair	Bought the delivery arrived, now charging on the electric car to try how to	JD.com
Mokoyi Digital Selection	Electric Vehicle Repair	This user feels the product is better	JD.com
Mokoyi Digital Selection	Electric Vehicle Repair	The logistics was fast, the item was complete at the time, and it was easy to use this.	JD.com
Mokoyi Digital Selection	Electric Vehicle Repair	I am using it and the delivery was fast! It's just as good as the instructions say it should be!	JD.com
Mokoyi Digital Selection	Electric Vehicle Repair	's quite good, very satisfied[big love][big love]	JD.com
Mokoyi Digital Selection	Electric Vehicle Repair	This user feels the product is better	JD.com
Mokoyi Digital Selection	Electric Vehicle Repair	item description is too simple, I72volt 4x4, will it work?	JD.com
Mokoyi Digital Selection	Electric Vehicle Repair	This user feels the product is better	JD.com
Mokoyi Digital Selection	Electric Vehicle Repair	This user feels the product is better	JD.com
Mokoyi Digital Selection	Electric Vehicle Repair	The goods have been received! Quite good next次 to come back to buy!	JD.com
Mokoyi Digital Selection	Electric Vehicle Repair Magic	Charger received, buy back can not wait to give their own electric car charged. The same as the merchant description of the switch to turn on the light up three lights. I believe that the old battery will slowly get repaired.	JD.com
Mokoyi Digital Selection	Electric Vehicle Repair	Great service, value for money and easy to use.	JD.com
Mokoyi Digital Selection	Electric Vehicle Repair	This user feels the product is better	JD.com
Mokoyi Digital Selection	Electric Vehicle Repair	Works great worth buying 5 stars	JD.com
Mokoyi Digital Selection	Electric Vehicle Repair	I'll comment after I use it.	JD.com

Mokoyi Digital Selection	Electric Vehicle Repair	I started using it last night, I don't know if it's any good.	JD.com
Mokoyi Digital Selection	Electric Vehicle Repair	Fast shipping, received the physical quality is very good, convenient and practical effect is ideal, trustworthy!	JD.com
Mokoyi Digital Selection	Electric Vehicle Repair Magic	Logistics is very fast, the goods are also okay, is still do not know the effect, good and then buy. [smile] [smile] [smile] [smile] [smile] [smile] [smile].	JD.com
Mokoyi Digital Selection	Electric Vehicle Repair	This user feels the product is better	JD.com
Mokoyi Digital Selection	Electric Vehicle Repair	It's small and compact. My cell phone battery battery repair works.	JD.com
Mokoyi Digital Selection	Electric Vehicle Repair	The logistics is very fast already received the goods	JD.com
Mokoyi Digital Selection	Electric Vehicle Repair	Logistics is fast, compact repair my electric car battery a little	JD.com
Blossom and Fortune Shop	Repair Adapter	The store's gone.	JD.com
Smart 3C Digital	Battery Reconditioner	Very good, very satisfied with a shopping, the effect is good, will buy again![Kudos] [Kudos] [Buy] [Buy] [Buy it] [Buy] [Rose]	JD.com
Smart 3C Digital	Battery Reconditioner	It works very well and is worth buying.	JD.com
Smart 3C Digital	Battery Reconditioner	Goods received, logistics quickly, well packaged	JD.com
Smart 3C Digital	Battery Reconditioner	today much more durable than before, good product, worth having!	JD.com
Smart 3C Digital	Battery Reconditioner	rare	JD.com
Smart 3C Digital	Battery Reconditioner	It's okay. Worth buying. I introduced friends. It's really not bad. I like it.	JD.com
Smart 3C Digital Furniture AD	Battery Reconditioner	have charged three times. Including a cell phone restored to factory settings after charging, and see no effect, according to the instructions, charging five or six times six or seven times, Fang Xian effect. The display shows a bunch of numbers over and over again, I don't know what it means. As the online evaluation of this battery restorer, there are Chinese also got a whole lot of foreign languages, I do not know why, and there is no manufacturer, there is no safety instructions, on the display of the digital representation of what is not explained, so this cell phone charging restorer, in the end, what role in the repair of the battery, doubtful! That's a reason to give a three star.	JD.com
Smart 3C Digital	Battery Reconditioner	a feel pretty good, tried for a while feel no problem pretty good, very like[big love] [big love][big love] [big love][big love	JD.com
Smart 3C Digital	Battery Reconditioner	Very good, with the description of the match, logistics is fast, good service attitude, have friends need to recommend	JD.com
Smart 3C Digital	Battery Reconditioner	It's really good.	JD.com
Smart 3C Digital	Battery Reconditioner	Trying it now, hope it works	JD.com
Smart 3C Digital	Battery Reconditioner	Fast shipping, fine workmanship, unique and beautiful shape, with voltage and current display, very satisfied!	JD.com
Smart 3C Digital	Battery Reconditioner	It's being activated with a display, and I expect it to be as good as the anchor narrated.	JD.com
Smart 3C Digital	Battery Reconditioner	Smart charger received good quality worth buying!	JD.com
Smart 3C Digital	Battery Reconditioner	[big love] [big love] [big]love]big[] [big love] [biglove]	JD.com
Smart 3C Digital	Battery Reconditioner	The quality of the product is excellent and I love using you as a brand. The service is top notch.	JD.com
Smart 3C Digital	Battery Reconditioner	I won the prize, thanks to the store, business is booming!	JD.com
Smart 3C Digital Furniture AD	Battery Reconditioner	Baby received, logistics fast,, now technology is really developed, things affordable, fine workmanship, beautiful appearance, easy to use, full five-star praise.	JD.com
Smart 3C Digital	Battery Reconditioner	Quite good, easy to use, fast charging, repair battery, good price, worth buying.	JD.com
Smart 3C Digital	Battery Reconditioner	Charge Fixer Received,<br>Charging Speed: Very Fast Worth the money	JD.com
Smart 3C Digital	Battery Reconditioner	Charging is not hot, the style is beautiful, now the technology is really advanced I love it!	JD.com
Smart 3C Digital	Battery Reconditioner	I received the cell phone fixer but have not used it, it looks like it should be good!	JD.com
Smart 3C Digital	Battery Reconditioner	Don't know how it works. Plugged in. Shows charging. I don't know if that's right.	JD.com
Smart 3C Digital	Battery Reconditioner	Works pretty well, use it a few more times, hope the battery lasts longer	JD.com
Smart 3C Digital	Battery Reconditioner	Cell Phone Battery Smart Repairer Received, Cleaning Junk, Repairing Battery	JD.com

Smart 3C Digital Furniture AD	Battery Reconditioner	Goods received logistics is also very fast quality, beautiful and generous I like.	JD.com
Smart 3C Digital Furniture AD	Battery Reconditioner	recovered the role of the battery, nearly two months in the cell phone every day shows that the battery has a problem should be sent to the test, with this baby charged the second time there is no prompt to test,.There is a need to be assured that the spell.	JD.com
Smart 3C Digital Furniture AD	Battery Reconditioner	Technical performance: good enough to buy	JD.com
Smart 3C Digital Furniture AD	Battery Reconditioner	Just received the goods, whether it is workmanship, material, appearance, can be called a perfect! Absolutely conscientious business! Five-star praise!	JD.com
Smart 3C Digital Furniture AD	Battery Reconditioner	Charging speed: still quite fast<br>Ease of operation: convenient and practical<br>Safety: relatively safe<br>Other, to be verified[smile]	JD.com
Smart 3C Digital Furniture AD	Battery Reconditioner	This repair gas is very good to use, charging speed is very fast, charging the phone is not hot, give five stars!	JD.com
Smart 3C Digital Furniture AD	Battery Reconditioner	not wait to give the phone plugged in, full of later, with a whole day, than the previous resistance, before it can be used for half a day, and now with a whole day, really good, I got the unit, we all say good, there are four comrades said to buy, I'm not a toto, really good to use, must give the businessman five points positive feedback!	JD.com
Smart 3C Digital Furniture AD	Battery Reconditioner	I received the converter, at first I did not know how to use it. Asked the merchant sent a video, quite good.	JD.com
Smart 3C Digital Furniture AD	Battery Reconditioner	Baby is great and fixes well! Worth buying.	JD.com
Smart 3C Digital Furniture AD	Battery Reconditioner	Great service from the merchant, answers to questions, and great quality!	JD.com
Smart 3C Digital Furniture AD	Battery Reconditioner	Received yesterday, the logistics is also very fast, in the trial observation a few days before commenting.	JD.com
Smart 3C Digital Furniture AD	Battery Reconditioner	Follow the instructions to install, charge the phone, the effect is really good, really black technology, cost-effective, very satisfied.	JD.com
Smart 3C Digital Furniture AD	Battery Reconditioner	The packaging is intact, nice…………	JD.com
Smart 3C Digital Furniture AD	Battery Reconditioner	Exactly as described, fast charging, stable performance and great price!	JD.com
Smart 3C Digital Furniture AD	Battery Reconditioner	Repairer received, immediately charge the phone, to see how the effect, first give a positive feedback.	JD.com
Smart 3C Digital Furniture AD	Battery Reconditioner	It has a high class feeling, with voltage and current display, just used it, see how durable the phone is!	JD.com
Smart 3C Digital Furniture AD	Battery Reconditioner	[Lots of love][Lots of love]	JD.com
Smart 3C Digital Furniture AD	Battery Reconditioner	Received, thank you boss, fast delivery, packaging intact.	JD.com
Smart 3C Digital Furniture AD	Battery Reconditioner	The product is of good quality and works well, good value for money.	JD.com
Smart 3C Digital Furniture AD	Battery Reconditioner	Charging speed: fast<br>Stable performance: good	JD.com
Smart 3C Digital Furniture AD	Battery Reconditioner	Battery Reconditioner works great!	JD.com
Smart 3C Digital Furniture AD	Battery Reconditioner	The goods have been received, well packaged, open the box, the effect is good, very like, next time again.	JD.com
Smart 3C Digital Furniture AD	Battery Reconditioner	Received the goods have been practical, very good worth getting!	JD.com
Smart 3C Digital Furniture AD	Battery Reconditioner	I'm on a trial period, so far I'm feeling pretty good	JD.com
Smart 3C Digital Furniture AD	Battery Reconditioner	Nice and fast charging	JD.com
Smart 3C Digital Furniture AD	Battery Reconditioner	effect is not yet seen, wait a few days to use down to comment on it	JD.com
Smart 3C Digital Furniture AD	Battery Reconditioner	It's okay, charging is not lagging, try two days have effect, better than the original, need to rest assured that the purchase, logistics fast service is good, five-star praise.	JD.com
Smart 3C Digital Furniture AD	Battery Reconditioner	I hope there really is a fix for cell phones	JD.com
Smart 3C Digital Furniture AD	Battery Reconditioner	Beautiful style and reasonable design and fine workmanship after receiving the trial can control the current to ensure that the charging is not hot in fact, a trickle charging and fast charging is the opposite of fast charging is often damage to the battery trickle charging is to care for the battery feel good the price is also acceptable all five-star praise!	JD.com
Smart 3C Digital Furniture AD	Battery Reconditioner	Pretty good! Received it and used it, looking forward to amazing results	JD.com
Smart 3C Digital Furniture AD	Battery Reconditioner	can	JD.com

Smart 3C Digital Furniture AD	Battery Reconditioner	The goods have been received, the logistics is very fast, the charging speed is very fast, the	JD.com
Smart 3C Digital Furniture AD	Battery Reconditioner	Just received the goods on the plug to charge, do not know if there is no effect, try it first	JD.com
Smart 3C Digital Furniture AD	Battery Reconditioner	Received the goods, looks particularly good, take it back tonight to try[cool]	JD.com
Smart 3C Digital Furniture AD	Battery Reconditioner	Can be fast-charged, can be super fast-charged, can repair the battery, good reviews	JD.com
Smart 3C Digital Furniture AD	Battery Reconditioner	Waiting for results before commenting	JD.com
Smart 3C Digital Furniture AD	Battery Reconditioner	This is good quality, much stronger than the last product I bought, trustworthy. Fast logistics and good service.	JD.com
Smart 3C Digital Furniture AD	Battery Reconditioner	Quality can't be beat, just like the store, especially like, and the price is favorable!	JD.com
Smart 3C Digital Furniture AD	Battery Reconditioner	Beautiful and practical, very affordable, thanks for the compliments.	JD.com
Smart 3C Digital Furniture AD	Battery Reconditioner	Good quality, hope it works well, thanks!	JD.com
Smart 3C Digital Furniture AD	Battery Reconditioner	It works well.	JD.com
Smart 3C Digital Furniture AD	Battery Reconditioner	The stuff is pretty good, the month is pretty good, the restorers are pretty good	JD.com
Smart 3C Digital Furniture AD	Battery Reconditioner	Technology is so advanced nowadays.[Lotsof love]	JD.com
Smart 3C Digital Furniture AD	Battery Reconditioner	inexpensive, much cheaper than the supermarket, very satisfied with a shopping, I wish the boss a prosperous business wealth roll.	JD.com
Smart 3C Digital Furniture AD	Battery Reconditioner	Exquisite and compact, the instructions are very detailed, will repurchase!	JD.com
Smart 3C Digital Furniture AD	Battery Reconditioner	Fast logistics Well-packed Compact and exquisite Easy to use Trustworthy	JD.com
Smart 3C Digital Furniture AD	Battery Reconditioner	Have not yet used it, do not know how the effect is, or give you5points praise, logistics is quite fast. Same as depicted.	JD.com
Smart 3C Digital Furniture AD	Battery Reconditioner	Fooling around! You can't fix a bad battery! I bought it to return it and the kids lost the cover!	JD.com
Smart 3C Digital Furniture AD	Battery Reconditioner	The merchandise is great, exactly as described by the merchant, the	JD.com
Smart 3C Digital Furniture AD	Battery Reconditioner	Not bad.	JD.com
Smart 3C Digital Furniture AD	Battery Reconditioner	Likes and Likes and Likes and Likes and Likes and Likes and Likes and Likes and Likes and Likes and Likes and Likes and Likes.	JD.com
Smart 3C Digital Furniture AD	Battery Reconditioner	I'm trying it out, so I'll wait and see.	JD.com
Smart 3C Digital Furniture AD	Battery Reconditioner	Repair punch is very good, was charging when the phone is hot, now with this repairer charging does not heat, give positive feedback5stars.	JD.com
Smart 3C Digital Furniture AD	Battery Reconditioner	First time buyer and first time user it's ok, recommend it!	JD.com
Smart 3C Digital Furniture AD	Battery Reconditioner	Charging speed: not great	JD.com
Smart 3C Digital Furniture AD	Battery Reconditioner	not heat, but also boosted1hour<br>Charging speed: charging speed<br>Stability performance: stable current does not heat<br>Safety: very good<br>Operation convenience: anytime with<br>Charging port design: charging socket, and there is an internal s	JD.com
Smart 3C Digital Furniture AD	Battery Reconditioner	Quite good service is in place, I hope everyone to buy it, quite affordable, baby received it thank you!	JD.com
Smart 3C Digital Furniture AD	Battery Reconditioner	Well, well, well, well.	JD.com
Smart 3C Digital Furniture AD	Battery Reconditioner	First time to use the old phone first try the effect looks good	JD.com
Smart 3C Digital Furniture AD	Battery Reconditioner	Received reconditioned batteries□can continue every day	JD.com
Smart 3C Digital Furniture AD	Battery Reconditioner	First time using it and it works great!	JD.com
Smart 3C Digital Furniture AD	Battery Reconditioner	It's restorative.	JD.com
Smart 3C Digital Furniture AD	Battery Reconditioner	Received baby, shipping can be one fix two beautiful three afford[kudos] [kudos] [kudos] [] [kudos][kudos	JD.com
Smart 3C Digital Furniture AD	Battery Reconditioner	I got it. It works.	JD.com
Smart 3C Digital Furniture AD	Battery Reconditioner	Very nice Works very well Good	JD.com

Smart 3C Digital Furniture AD	Battery Reconditioner	Express has been received, the quality is really good, the use can also see the size of the working voltage and current, it is also very convenient to use, intuitive, next time to buy!	JD.com
Smart 3C Digital Furniture AD	Battery Reconditioner	I don't know how well it works. I'll follow up with a review if it works well.	JD.com
Smart 3C Digital Furniture AD	Battery Reconditioner	Received the cell phone rechargeable battery repairer. Follow the instructions in use to see how it works. Looking forward to good results. Good results will buy back again.	JD.com
Smart 3C Digital Furniture AD	Battery Reconditioner	The quality is very good, received the goods are easy to use and convenient, charging can also improve the cell phone battery life is very good to use	JD.com
Smart 3C Digital Furniture AD	Battery Reconditioner	Good with that junk good fast, that power charge amount fast	JD.com
Smart 3C Digital Furniture AD	Battery Reconditioner	thing really works, I wish your factory business prosperity prosperity,.	JD.com
Smart 3C Digital Furniture AD	Battery Reconditioner	Technical performance: good<br>Charging speed: fast<br>Stable performance: good Please buy with confidence.	JD.com
Smart 3C Digital Furniture AD	Battery Reconditioner	Use the phone does not burn, only used once, do not know how the effect, should be able to	JD.com
Smart 3C Digital Furniture AD	Battery Reconditioner	Great product, second repurchase. Express delivery is fast. Worth buying.	JD.com
Smart 3C Digital Furniture AD	Battery Reconditioner	This cell phone battery repair is a product qualification documents, very good, high-end atmosphere five-star praise.	JD.com
Smart 3C Digital Furniture AD	Battery Reconditioner	Just started using it. Looks good.	JD.com
Smart 3C Digital Furniture AD	Battery Reconditioner	I've used it a couple times and it's fine.	JD.com
Smart 3C Digital Furniture AD	Battery Reconditioner	It's great! Charging is faster too! Battery is slowly being fixed!	JD.com
Smart 3C Digital Furniture AD	Battery Reconditioner	Charging doesn't get hot works great	JD.com
Smart 3C Digital Furniture AD	Battery Reconditioner	It works great, when you charge it, the case is cool and it should protect the phone well.	JD.com
Smart 3C Digital Furniture AD	Battery Reconditioner	It's spot on. It's very good.	JD.com
Smart 3C Digital Furniture AD	Battery Reconditioner	Has been used, has a digital display, feels effective, will comment again after charging is complete and in use.	JD.com
Smart 3C Digital Furniture AD	Battery Reconditioner	probationary period	JD.com
Smart 3C Digital Furniture AD	Battery Reconditioner	Received the goods, the light emitted when charging is also quite nice,[cool] [cool]	JD.com
Smart 3C Digital Furniture AD	Battery Reconditioner	Goods received is good fast, I started the first day of the bed is afraid to use later asked the business, the next day three days is really cell phone on the fluent more than a little, this thing is worth buying.	JD.com
Smart 3C Digital Furniture AD	Battery Reconditioner	speed is also exceptionally fast, will continue to follow up later	JD.com
Smart 3C Digital Furniture AD	Battery Reconditioner	Good product, fast logistics, will buy again if it's good.	JD.com
Smart 3C Digital Furniture AD	Battery Reconditioner	Practicality and value are very awesome, beautifully packaged, super love this series, wall to wall recommendation! 有需要再回购的[大爱]	JD.com
Smart 3C Digital Furniture AD	Battery Reconditioner	Let's see.	JD.com
Smart 3C Digital Furniture AD	Battery Reconditioner	I won't know if it's good until I've used it for a while	JD.com
Smart 3C Digital Furniture AD	Battery Reconditioner	Received the cell phone battery repairer! Used it for a while and it works fine! Very satisfied with a shopping! 👍	JD.com
Smart 3C Digital Furniture AD	Battery Reconditioner	Received: looks good, pretty good. Hope it works.	JD.com
Smart 3C Digital Furniture AD	Battery Reconditioner	It's nice nice charging it overnight and finding out the next day that the phone lasts half an hour longer	JD.com
Smart 3C Digital Furniture AD	Battery Reconditioner	It's good, it's really noticeable, it saves money on battery replacements.	JD.com
Smart 3C Digital Furniture AD	Battery Reconditioner	Repairer received, please use feel the effect is okay, will recommend the need of friends to buy. five-star[kudos] [kudos] [kudos] [kudos]	JD.com
Smart 3C Digital Furniture AD	Battery Reconditioner	It's good.	JD.com
Smart 3C Digital Furniture AD	Battery Reconditioner	buy, after the purchase of no regrets at all, and the logistics is also fast, you can get into a very good shopping experience, thoughtful service, fast logistics, distribution is also thoughtful, attitude is also very good, value for money, ah, decisive praise!	JD.com

Smart 3C Digital Furniture AD	Battery Reconditioner	Roger that. - Thanks! Give it a try.	JD.com
Smart 3C Digital Furniture AD	Battery Reconditioner	I'll give it a good review first, I don't know if it works or not	JD.com
Smart 3C Digital Furniture AD	Battery Reconditioner	: very good<br>Stable performance: very good	JD.com
Smart 3C Digital Furniture AD	Battery Reconditioner	I just received it today, I don't know if the repairing effect is good, looking forward to the result	JD.com
Smart 3C Digital Furniture AD	Battery Reconditioner	Customer service was very patient showed me how to use it<br>Thank you	JD.com
Smart 3C Digital Furniture AD	Battery Reconditioner	Yes[Smiles]	JD.com
Smart 3C Digital Furniture AD	Battery Reconditioner	Works like a charm, good quality and works great.	JD.com
Smart 3C Digital Furniture AD	Battery Reconditioner	The baby was received very quickly, tried it for a few days and it works great!	JD.com
Smart 3C Digital Furniture AD	Battery Reconditioner	Finally received the baby I need, the stuff is very good, the price is good and cheap, thanks to!The owner's attitude is very professional and enthusiastic, ask questions and reply quickly, I asked a lot of questions, he does not feel annoyed, they will seriously answer me. And then say baby, just what I need, when I received the packaging is complete, after opening the surprise, baby than I imagined even better!Had to have to give a thumbs up. I will come back next time I need it!	JD.com
Smart 3C Digital Furniture AD	Battery Reconditioner	I have received the goods and I feel good after using it	JD.com
Smart 3C Digital Furniture AD	Battery Reconditioner	very convenient and simple[stay]effect is also very good👍worth having worth looking forward to recommend oh[love][love][love][love]	JD.com
Smart 3C Digital Furniture AD	Battery Reconditioner	It works well!	JD.com
Smart 3C Digital Furniture AD	Battery Reconditioner	The logistics is fast, and I have used it.	JD.com
Smart 3C Digital Furniture AD	Battery Reconditioner	Not bad. Not bad.[Lots of love][Lots of love]	JD.com
Smart 3C Digital Furniture AD	Battery Reconditioner	I don't know if it works, but I don't feel the battery is fixed anyway, I guess it's an IQ tax	JD.com
Smart 3C Digital Furniture AD	Battery Reconditioner	can	JD.com
Smart 3C Digital Furniture AD	Battery Reconditioner	It still feels good and well made	JD.com
Smart 3C Digital Furniture AD	Battery Reconditioner	Repair summer device received, opened a look at the beautiful style, immediately began to use, in the process of using the same as described in the video, the data in the change is genuine. Can make the phone to extend the use of life, cost-effective worth buying, must rate five stars!	JD.com
Smart 3C Digital Furniture AD	Battery Reconditioner	very powerful	JD.com
Smart 3C Digital Furniture AD	Battery Reconditioner	Charge protector received, good quality	JD.com
Smart 3C Digital Furniture AD	Battery Reconditioner	Faster charging, but battery repair is not obvious	JD.com
Smart 3C Digital Furniture AD	Battery Reconditioner	Goods received feel pretty good, power on the appearance of more beautifuldo not know if the use of any effect:if the effect of Dali publicity!	JD.com
Smart 3C Digital Furniture AD	Battery Reconditioner	Goods have been received, very good use of fast delivery, after-sales service first-class!	JD.com
Smart 3C Digital Furniture AD	Battery Reconditioner	Tried several times. It doesn't show results.	JD.com
Smart 3C Digital Furniture AD	Battery Reconditioner	received	JD.com
Smart 3C Digital Furniture AD	Battery Reconditioner	Baby has received, open the package to find the appearance of the design of small and exquisite, fast charging speed, strong technical performance, good efficiency, maintenance costs can be considerable, it is worth buying!	JD.com
Smart 3C Digital Furniture AD	Battery Reconditioner	I got it. I don't know how it's gonna work.	JD.com
Smart 3C Digital Furniture AD	Battery Reconditioner	This thing works really well, I bought two at once and gave one to my son who also said it was pretty good. Good review!	JD.com
Smart 3C Digital Furniture AD	Battery Reconditioner	Arrived at home immediately used, I hope there are effects, looks good, good and then follow up reviews	JD.com
Smart 3C Digital Furniture AD	Battery Reconditioner	Quality is very good, good value, have to rest assured that the order it!	JD.com
Smart 3C Digital Furniture AD	Battery Reconditioner	Just received the item, this is the second time I've bought it and it works well.	JD.com
Smart 3C Digital Furniture AD	Battery Reconditioner	The goods have been received, the service attitude. Awesome.	JD.com

Smart 3C Digital Furniture AD	Battery Reconditioner	This user feels the product is better	JD.com
Smart 3C Digital Furniture AD	Battery Reconditioner	In use, will follow up after a while	JD.com
Smart 3C Digital Furniture AD	Battery Reconditioner	to evaluate. Charging speed is very fast, the key is to be able to maintain the cell phone battery. A purchase and recommended.	JD.com
Smart 3C Digital Furniture AD	Battery Reconditioner	I'm using it now. I'll comment after I use it.	JD.com
Smart 3C Digital Furniture AD	Battery Reconditioner	Nowadays, technology is so advanced, the battery is really durable, and the price of the stuff is affordable.	JD.com
Smart 3C Digital Furniture AD	Battery Reconditioner	Additional comments after use	JD.com
Smart 3C Digital Furniture AD	Battery Reconditioner	Works well, tried the charge not as hot as before.	JD.com
Smart 3C Digital Furniture AD	Battery Reconditioner	Charging is normal, looking forward to a good repair of the battery, logistics is fast.	JD.com
Smart 3C Digital Furniture AD	Battery Reconditioner	The cell phone adapter has been received, it works well and charges fast, I will recommend my friends to buy it!	JD.com
Smart 3C Digital Furniture AD	Battery Reconditioner	last night does have the effect , he repair ability is quite powerful , cell phone lagging charging can not be charged he will help you to repair, I do not know how the quality of the future First praise!	JD.com
Smart 3C Digital Furniture AD	Battery Reconditioner	The item is good, just as the merchant said it would be.	JD.com
Smart 3C Digital Furniture AD	Battery Reconditioner	Received, charging, expect good results, follow up with evaluation	JD.com
Smart 3C Digital Furniture AD	Battery Reconditioner	This user feels the product is better	JD.com
Smart 3C Digital Furniture AD	Battery Reconditioner	This product is quite good, the phone charging is not hot anymore	JD.com
Smart 3C Digital Furniture AD	Battery Reconditioner	It's here, it feels good, I'll charge it a couple times and see.	JD.com
Smart 3C Digital Furniture AD	Battery Reconditioner	the root of the business description of the same, thank you business!	JD.com
Smart 3C Digital Furniture AD	Battery Reconditioner	After a couple of flushes, the cell phone battery is good to go	JD.com
Smart 3C Digital Furniture AD	Battery Reconditioner	effect, after the use of follow-up comments, I hope it is a good shopping choice, although the reviews are mixed, more trust in the store.	JD.com
Smart 3C Digital Furniture AD	Battery Reconditioner	It's good, it's genuine, I used it right away when I received it, and it has a digital display.	JD.com
Smart 3C Digital Furniture AD	Battery Reconditioner	Works well, good quality, charges quickly, looking forward to good results.	JD.com
Smart 3C Digital Furniture AD	Battery Reconditioner	Fast shipping, pretty good quality, as described by the seller, very satisfied!	JD.com
Smart 3C Digital Furniture AD	Battery Reconditioner	Easy to use, not too effective.	JD.com
Smart 3C Digital Furniture AD	Battery Reconditioner	It works well, I will come back next time if I need it, 5 stars!	JD.com
Smart 3C Digital Furniture AD	Battery Reconditioner	Cell phone battery protector received, easy to use, good price. Give five stars praise.	JD.com
Smart 3C Digital Furniture AD	Battery Reconditioner	Can show the current and voltage of the number of sub, nowadays the science is advanced, on I believe that can repair the phone, looking forward to will be successfully repaired. Thank you buyer for providing	JD.com
Smart 3C Digital Furniture AD	Battery Reconditioner	After the payment, I am using it, andcan only seespecific resultsafterI use itfirsttime. If it is really good, I will review it. I hope it can solve the problem of hot cell phone battery plate and prolong the service life.	JD.com
Smart 3C Digital Furniture AD	Battery Reconditioner	Goods to receive, very good, need to come back to your store to buy.	JD.com
Smart 3C Digital Furniture AD	Battery Reconditioner	prompted by the decline in health status, buy back to see the instructions, the need to shut down the repair, shut down before going to bed at night to repair, the morning boot actually that annoying prompts are really no longer, the battery also feels good to use. The photo above is the second repair last night. It's a pleasant surprise, and I recommend buying it.	JD.com
Smart 3C Digital Furniture AD	Battery Reconditioner	I haven't used it yet, it feels good, but I hope it works!	JD.com
Smart 3C Digital Furniture AD	Battery Reconditioner	It's good, and the cell phone battery has been extended, so I'm glad you guys bought it.	JD.com
Smart 3C Digital Furniture AD	Battery Reconditioner	This user feels the product is better	JD.com
Smart 3C Digital Furniture AD	Battery Reconditioner	I bought it and tried it for a few days, it works quite well, I recommend buying it!	JD.com

Smart 3C Digital Furniture AD	Battery Reconditioner	Charge very quickly, now technology is really developed, the battery is really durable, the value is super high, the price of the stuff is affordable, exquisite workmanship	JD.com
Smart 3C Digital Furniture AD	Battery Reconditioner	Waiting to try the results before commenting	JD.com
Smart 3C Digital Furniture AD	Battery Reconditioner	Very good, fast, beautiful, I like it very much, thanks to the buyer, next time I will definitely come to your store.	JD.com
Smart 3C Digital Furniture AD	Battery Reconditioner	I just got it and used it. It feels fine. It's good.	JD.com
Smart 3C Digital Furniture AD	Battery Reconditioner	Received the item, shipped quickly, and am trying it out.	JD.com
Smart 3C Digital Furniture AD	Battery Reconditioner	Merchants shipping fast, logistics is also strong, has been used, looking at the quality is very good, repair a period of time to see the effect and then follow up.	JD.com
Smart 3C Digital Furniture AD	Battery Reconditioner	quite good, is the merchant said that there is a repair function	JD.com
Smart 3C Digital Furniture AD	Battery Reconditioner	Let's see how it works and then we'll have a proper review	JD.com
Smart 3C Digital Furniture AD	Battery Reconditioner	It feels good.	JD.com
Smart 3C Digital Furniture AD	Battery Reconditioner	It's great, it's worth buying[big love][big love][big love][big love	JD.com
Smart 3C Digital Furniture AD	Battery Reconditioner	Charging is fast, technology is so advanced now!	JD.com
Smart 3C Digital Furniture AD	Battery Reconditioner	Baby is good, received it and used it, two cell phone rotation, feel pretty good.	JD.com
Smart 3C Digital Furniture AD	Battery Reconditioner	Received have not started to use, read the instructions and deep repair function, hope that good use hey!	JD.com
Smart 3C Digital Furniture AD	Battery Reconditioner	The goods have been received, it works well, the phone does not get hot when charging, thank you boss.	JD.com
Smart 3C Digital Furniture AD	Battery Reconditioner	Battery Repairer received, the effect is good, very satisfied, five stars!	JD.com
Smart 3C Digital Furniture AD	Battery Reconditioner	This user did not fill in a rating	JD.com
Smart 3C Digital Furniture AD	Battery Reconditioner	Haha pretty amazing little thing, good use of modern tech.	JD.com
Smart 3C Digital Furniture AD	Battery Reconditioner	's working.	JD.com
Smart 3C Digital Furniture AD	Battery Reconditioner	It looks good.	JD.com
Smart 3C Digital Furniture AD	Battery Reconditioner	Goods have been received, the quality is very good.	JD.com
Smart 3C Digital Furniture AD	Battery Reconditioner	I used it today after I got it, I don't know how it works yet	JD.com
Smart 3C Digital Furniture AD	Battery Reconditioner	The charger has a display light and charges quickly, which is nice.	JD.com
Smart 3C Digital Furniture AD	Battery Reconditioner	You goods received, well-packed, shipping is also very fast, the service is also good, the next time to buy!	JD.com
Smart 3C Digital Furniture AD	Battery Reconditioner	Just used it, how the effect is still please chu, wait for a period of time to follow up the evaluation.	JD.com
Smart 3C Digital Furniture AD	Battery Reconditioner	The Battery Reconditioner is small and compact, easy to use and has a repair digital display, use it2to3timesa week.	JD.com
Smart 3C Digital Furniture AD	Battery Reconditioner	an excellent	JD.com
Smart 3C Digital Furniture AD	Battery Reconditioner	It's very practical and[smile] [smile] [smilesmile] [smile] [smile]	JD.com
Smart 3C Digital Furniture AD	Battery Reconditioner	The goods have been received, the quality is very good oh!	JD.com
Smart 3C Digital Furniture AD	Battery Reconditioner	pretty good	JD.com
Smart 3C Digital Furniture AD	Battery Reconditioner	Rechargeable battery: very fast, feel the effect can be, there is an improvement work.<br>Face appearance: exquisite<br>Product appearance: exquisite	JD.com
Smart 3C Digital Furniture AD	Battery Reconditioner	The product has received, the logistics is very fast, used two days really have the effect, the phone does not heat up, charge is also fast, very satisfied with the product.	JD.com
Smart 3C Digital Furniture AD	Battery Reconditioner	非常满意的一次购物，[点赞]五星好评	JD.com
Smart 3C Digital Furniture AD	Battery Reconditioner	Item looks ok! Still has data when charging! Battery has yet to be tested to see if it is repaired! I'll comment later.	JD.com
Smart 3C Digital Furniture AD	Battery Reconditioner	Charger received good quality, used it for a long time	JD.com
Smart 3C Digital Furniture AD	Battery Reconditioner	Baby received The logistics was fast I tried it today and it's a little better than it used to be	JD.com
Smart 3C Digital Furniture AD	Battery Reconditioner	an excellent	JD.com

Smart 3C Digital	Battery Reconditioner	Not bad, using it is very good for hand eletric leakage	JD.com
Smart 3C Digital Furniture AD	Battery Reconditioner	I received the goods, the repair effect is obvious, the pulse charging effect is very good, repair cell phone battery good and fast, really good, very satisfied.	JD.com
Smart 3C Digital	Battery Reconditioner	long time. I'm not a trustworthy person, I'm just telling the truth.	JD.com
Smart 3C Digital Furniture AD	Battery Reconditioner	power consumption is fast, the phone is easy to heat, slow charging, using the repairer after the video screen for pulse display voltage and current, charging time and heating phenomenon has improved, just started using, the effect needs to be further observed.	JD.com
Smart 3C Digital	Battery Reconditioner	Received. Logistics was fast. Very good. Same as the business description.	JD.com
Smart 3C Digital	Battery Reconditioner	I don't feel like it's working very well, I'm using it for a few days to comment on it	JD.com
Smart 3C Digital Furniture AD	Battery Reconditioner	than three years, and the battery doesn't last anymore. Used this fixer, effect personal feeling bar[big love][big love][big[big][big love][big] [big love] [big love] [big love] [big love] [big love] [big love] [big love] [big love] [big love] [big love] [big love] [big love][big love][big love	JD.com
Smart 3C Digital Furniture AD	Battery Reconditioner	the appearance of the product packaging, certificate of conformity, product technical parameters are complete, according to the instructions to do, looking forward to the effect.	JD.com
Smart 3C Digital	Battery Reconditioner	favorable criticism	JD.com
Smart 3C Digital	Battery Reconditioner	dissatisfied	JD.com
Smart 3C Digital	Battery Reconditioner	This is great because I had to use it to fix it very well!	JD.com
Smart 3C Digital	Battery Reconditioner	Logistics is very fast, packaging and installation, exquisite workmanship, a very satisfactory shopping, give five stars!	JD.com
Smart 3C Digital	Battery Reconditioner	It really works super well, the phone doesn't get hot while charging	JD.com
Smart 3C Digital	Battery Reconditioner	Received, to be used.	JD.com
Smart 3C Digital Furniture AD	Battery Reconditioner	The merchant shipped quickly. Logistics is also fast. After receiving the two cell phones were repaired, the effect is very good. Merchants and logistics are given five-star praise.	JD.com
Smart 3C Digital Furniture AD	Battery Reconditioner	Cell phone smart battery repairer received, try a day quite good, charging the phone is not hot, faster than the previous charge. The first thing I'd like to say is that I'm not sure how much I'm going to be able to do.	JD.com
Smart 3C Digital	Battery Reconditioner	Charge and repair cell phone batteries quickly, charging fast	JD.com
Smart 3C Digital	Battery Reconditioner	It's really good. It works.	JD.com
Smart 3C Digital	Battery Reconditioner	Received the product. I don't know if it's good! Never used it.	JD.com
Smart 3C Digital	Battery Reconditioner	dissatisfied	JD.com
Smart 3C Digital	Battery Reconditioner	Very good. Much smoother phone	JD.com
Smart 3C Digital	Battery Reconditioner	The goods have been received, not yet officially used, did not see the dowel port, hope it works!	JD.com
Smart 3C Digital	Battery Reconditioner	Looks good good quality worth buying fast delivery	JD.com
Smart 3C Digital	Battery Reconditioner	It has been receivedfor15days, ask for a review!	JD.com
Smart 3C Digital Furniture AD	Battery Reconditioner	To receive the charger to use the good, the beginning will not use customer service patiently told, thanks to the business customer service	JD.com
Smart 3C Digital	Battery Reconditioner	phone, so far it feels okay, or quite like it[love][love][love][love]	JD.com
Smart 3C Digital Furniture AD	Battery Reconditioner	Merchant service is thoughtful, express logistics quickly, the product looks good, to be used for a period of time after the follow-up effect!	JD.com
Smart 3C Digital	Battery Reconditioner	Goods received, just used for a period of time to know the effect is good or not!	JD.com
Smart 3C Digital Furniture AD	Battery Reconditioner	The logistics is very fast, the service attitude is very good, and the charging effect is very good. Used two or three days, the phone battery back to normal, much more durable than ever!	JD.com
Smart 3C Digital Furniture AD	Battery Reconditioner	I've been using it for a while before coming to comment, I feel it works, I tried it with my cell phone which I can no longer turn on, and after all this time, the phone has been working for an hour, so you can buy it.	JD.com

Smart 3C Digital Furniture AD	Battery Reconditioner	整挺好的嘿嘿嘿，不知道会不会补电池	JD.com
Smart 3C Digital Furniture AD	Battery Reconditioner	Merchant product quality is very good, beautiful	JD.com
Smart 3C Digital Furniture AD	Battery Reconditioner	said it's five to eight weeks to see the effect so many people buy it 👍	JD.com
Smart 3C Digital Furniture AD	Battery Reconditioner	Excellent service and fast delivery, good quality and low price, easy to use, very satisfied!	JD.com
Smart 3C Digital Furniture AD	Battery Reconditioner	Goods just received, is trying, feel okay, use a few days and then follow up.	JD.com
Smart 3C Digital Furniture AD	Battery Reconditioner	It works.	JD.com
Smart 3C Digital Furniture AD	Battery Reconditioner	The instructions are very detailed. Follow the instructions for a while and then follow up.	JD.com
Smart 3C Digital Furniture AD	Battery Reconditioner	Great value for money and long lasting use after charging the phone.	JD.com
Smart 3C Digital Furniture AD	Battery Reconditioner	Cell phone battery repair received, the phone will be turned off, the official charging, charging normal, battery repair effect to be followed up.<br>Packaging seriously, no damage. Good comment.	JD.com
Smart 3C Digital Furniture AD	Battery Reconditioner	Got it back and used it several times, charging and repairing all together, no additional switch, charging is fast, battery life is enhanced, phone lagging is a little less, repairing a few more times, it may be better.	JD.com
Smart 3C Digital Furniture AD	Battery Reconditioner	The goods have been received! It works really well.	JD.com
Smart 3C Digital Furniture AD	Battery Reconditioner	I just used it and I don't know if it's working yet[looking forward to]	JD.com
Smart 3C Digital Furniture AD	Battery Reconditioner	Charge to repair the original power and your own cell phone's standby time becomes longer	JD.com
Smart 3C Digital Furniture AD	Battery Reconditioner	Haven't used it yet, 3starsfor now.	JD.com
Smart 3C Digital Furniture AD	Battery Reconditioner	Good, can, repair cell phone battery fast, good satisfaction, want to order partners hurry up!	JD.com
Smart 3C Digital Furniture AD	Battery Reconditioner	Baby is very good, the quality is very good, very satisfied with a shopping, is to charge for a period of time to see if you can achieve satisfactory results 100% the battery capacity, I this phone is now 83%	JD.com
Smart 3C Digital Furniture AD	Battery Reconditioner	Charges quickly.	JD.com
Smart 3C Digital Furniture AD	Battery Reconditioner	I'm going to It feels good	JD.com
Smart 3C Digital Furniture AD	Battery Reconditioner	Easy to use, satisfied, fast shipping good reviews not sold.	JD.com
Smart 3C Digital Furniture AD	Battery Reconditioner	Has received, logistics is also very fast, customer service attitude is very good, five-star praise!	JD.com
Smart 3C Digital Furniture AD	Battery Reconditioner	Now in the process of fixing the charge, looks good, give it 5 stars!	JD.com
Smart 3C Digital Furniture AD	Battery Reconditioner	use, look at the instructions to use also worried about the inside of the stuff messed up, I hope the battery can get a better repair!	JD.com
Smart 3C Digital Furniture AD	Battery Reconditioner	It's pretty good and the baby looks good, and looking at the digital display, it should work!	JD.com
Smart 3C Digital Furniture AD	Battery Reconditioner	Goods have been received, conscientious businessmen, trustworthy	JD.com
Smart 3C Digital Furniture AD	Battery Reconditioner	Goods received product quality is ok came back and used it will repurchase.	JD.com
Smart 3C Digital Furniture AD	Battery Reconditioner	We'll see what it looks like later.	JD.com
Smart 3C Digital Furniture AD	Battery Reconditioner	Battery Repairer received, is testing, can see the voltage and current digital changes, feel very very good, try to comment again after good!	JD.com
Smart 3C Digital Furniture AD	Battery Reconditioner	I just used it twice, I'm not sure if it's effective yet, it's very convenient to use it	JD.com
Smart 3C Digital Furniture AD	Battery Reconditioner	The quality of the bar, packaging and quality can not be said, the quality of business and courier service can not be said, are quite good, the next time you will come to buy!	JD.com
Smart 3C Digital Furniture AD	Battery Reconditioner	deal with the cell phone is very good to use genuinely good will introduce friends so that everyone can use a good product boss service is very good logistics is also very fast all five praise!	JD.com
Smart 3C Digital Furniture AD	Battery Reconditioner	It works well.	JD.com
Smart 3C Digital Furniture AD	Battery Reconditioner	helper for repairing and maintaining cell phone batteries., safe and easy to use is a good choice for cell phone users.Five-star praise!	JD.com
Smart 3C Digital Furniture AD	Battery Reconditioner	Received the pieces, tried them out, they are ok, good quality.	JD.com

Smart 3C Digital Furniture AD	Battery Reconditioner	Product appearance: can<br>Face appearance: small size<br>Lighting effect: soft	JD.com
Smart 3C Digital Furniture AD	Battery Reconditioner	The cell phone adapter has been received and the logistics were fast!	JD.com
Smart 3C Digital Furniture AD	Battery Reconditioner	Goods have been received! Packaging intact; match the description; business integrity, patience to explain, help, guide to try, not bad; very satisfied!	JD.com
Smart 3C Digital Furniture AD	Battery Reconditioner	working great. Thank you so much, Merchant, for having such a great product.	JD.com
Smart 3C Digital Furniture AD	Battery Reconditioner	Repair has received, try, repair normal, a pleasant shopping. Wish merchants prosperous business.	JD.com
Smart 3C Digital Furniture AD	Battery Reconditioner	see how well it works	JD.com
Smart 3C Digital Furniture AD	Battery Reconditioner	Not bad, it still works pretty well and the phone doesn't get hot.	JD.com
Smart 3C Digital Furniture AD	Battery Reconditioner	The quality is great the workmanship is exquisite recommendable!	JD.com
Smart 3C Digital Furniture AD	Battery Reconditioner	trying it out, it's a good choice if it extends battery repair and life.	JD.com
Smart 3C Digital Furniture AD	Battery Reconditioner	It's good to use it again.	JD.com
Smart 3C Digital Furniture AD	Battery Reconditioner	It's good, using the battery once started to extend the working time, and the results are obviously good!	JD.com
Smart 3C Digital Furniture AD	Battery Reconditioner	used, more than half a day will not have much power. In order to have a comparison, with this high-tech product is also fully charged after not using, nearly two days plus one night, there are still25power. This shows that you really bought the right one.	JD.com
Smart 3C Digital Furniture AD	Battery Reconditioner	it works well, I believe it should work well. Logistics is fast.	JD.com
Smart 3C Digital Furniture AD	Battery Reconditioner	First of all, the merchant shipment is very timely, express is very powerful. Shenzhen to Henan two days, I this cell phone is already more than a year because the battery charging is very slow, storage capacity is too small abandoned almost two years. At noon after getting the baby charged, fifty minutes fast full. Charging speed satisfaction (this cell phone normal charging almost five hours), charging process temperature is normal. Not hot hand not hot. The back side looking forward to the satisfactory use of the effect. Information repeatedly repair a few times to harvest satisfactory results.	JD.com
Smart 3C Digital Furniture AD	Battery Reconditioner	Fine workmanship, looking forward to having results	JD.com
Smart 3C Digital Furniture AD	Battery Reconditioner	The item was received and has not been used yet.	JD.com
Smart 3C Digital Furniture AD	Battery Reconditioner	tablet phone is too stuck, after charging the speed is obviously accelerated, great, this shopping the most ideal shopping!	JD.com
Smart 3C Digital Furniture AD	Battery Reconditioner	Received the goods: very satisfied will repurchase	JD.com
Smart 3C Digital Furniture AD	Battery Reconditioner	try, the appearance is very beautiful, hold in the hand silky smooth, very feel, take pictures really live up to its name, the photos taken out of the super texture, too like! The battery is also very good, no problem with a day, charging is also very fast, no charging heat, running speed is also very fast.	JD.com
Smart 3C Digital Furniture AD	Battery Reconditioner	The original phone charging is very slow, now there is a significant improvement, the price is good, worth[loveit]	JD.com
Smart 3C Digital Furniture AD	Battery Reconditioner	Things received a look at the high feeling, get back to try a little, good quite good	JD.com
Smart 3C Digital Furniture AD	Battery Reconditioner	Received and use two days feel good, charging time than before short, use time is also long, logistics is very fast, value for money, worth buying, need not consider.	JD.com
Smart 3C Digital Furniture AD	Battery Reconditioner	It works.	JD.com
Smart 3C Digital Furniture AD	Battery Reconditioner	I've been using it for a few days now, and it feels good, standby and use is significantly longer, the	JD.com
Smart 3C Digital Furniture AD	Battery Reconditioner	light is showing repair, the appearance is also quite good, the light also looks good to the business point five-star praise!	JD.com
Smart 3C Digital Furniture AD	Battery Reconditioner	The goods received a few days ago, used a few days, feel pretty good, kudos!	JD.com
Smart 3C Digital Furniture AD	Battery Reconditioner	Just received the goods today that can not wait to try charging, feel good charging is genuine, to be repaired and then see the effect.	JD.com
Smart 3C Digital Furniture AD	Battery Reconditioner	Repair charger received, look good, a little sense of technology, do not know what kind of use, used in said	JD.com
Smart 3C Digital Furniture AD	Battery Reconditioner	I used it once and it felt good.	JD.com

Smart 3C Digital Furniture AD	Battery Reconditioner	would have been charging heat, power consumption fast. Charging time is long. After using the restorer, the deactivated machine charging is not molten, and the charging time is also shortened. But when charging while using, the charge is also gradually reduced, it seems that only the best deactivation of the phone charging, to see if the subsequent use can restore the durability of the battery and then evaluate!	JD.com
Smart 3C Digital Furniture AD	Battery Reconditioner	The charger restorer works very well, the battery is restored quickly, the charger charges quickly, the restored battery is also durable, the shipping is also very fast, straight to own.	JD.com
Smart 3C Digital [illegible]	Battery Reconditioner	It works great it's nice and practical it's worth buying it's good	JD.com
Smart 3C Digital [illegible]	Battery Reconditioner	Goods received, thanks!	JD.com
Smart 3C Digital Furniture AD	Battery Reconditioner	fine. The heating of the phone has improved, and the phone battery also feels a little more resistant than before, and then use it to see.	JD.com
Smart 3C Digital [illegible]	Battery Reconditioner	The data cable is soft and the battery restorer is nice and works well, 5 stars.	JD.com
Smart 3C Digital [illegible]	Battery Reconditioner	Can really repair, charging is also very fast, the object is also fine workmanship, regular manufacturers	JD.com
Smart 3C Digital [illegible]	Battery Reconditioner	Battery Repairer received, is testing, test good to follow up, you can see the voltage and current digital changes, feel very	JD.com
Smart 3C Digital Furniture AD	Battery Reconditioner	The product and the actual picture matches, I tried a few days to evaluate, it does have this aspect of the function, the battery is now more durable than before, the quality is very good, to the store products all five-star praise.	JD.com
Smart 3C Digital [illegible]	Battery Reconditioner	as described. Friends in need can rest assured that the purchase.	JD.com
Smart 3C Digital [illegible]	Battery Reconditioner	Goods received the quality is very good, repair cell phone battery	JD.com
Smart 3C Digital [illegible]	Battery Reconditioner	Good price, good quality, easy to use, very popular with customers	JD.com
Smart 3C Digital Furniture AD	Battery Reconditioner	really durable, now technology is really developed, the value is super high, the light is very cool, the color style is great, exquisite workmanship, easy to use. It is worth buying and selling!	JD.com
Smart 3C Digital [illegible]	Battery Reconditioner	Baby received immediately use, feel the effect is very good, goods value for money.	JD.com
Smart 3C Digital [illegible]	Battery Reconditioner	Beautiful appearance, large storage capacity, easy to charge, cost-effective, good[kudos]	JD.com
Smart 3C Digital [illegible]	Battery Reconditioner	Good, like, good value for money, will come back often!	JD.com
Smart 3C Digital [illegible]	Battery Reconditioner	I received the baby, it's really good! It's a great device to protect your cell phone battery! I give it a million kudos!	JD.com
Smart 3C Digital [illegible]	Battery Reconditioner	Received the product and started using it last night. Works like a charm, just like the instructions.Five stars.	JD.com
Smart 3C Digital [illegible]	Battery Reconditioner	Good stuff. It's good to try it out. I'll see you later.	JD.com
Smart 3C Digital Furniture AD	Battery Reconditioner	Received the flat machine fixer. Plugged it in right away and tried it out. The Fixer was able to see changes in the running data while in use. Lamp brother is very cool. Merchant sales first place. Now technology is really developed. My old man saw bought your product. Satisfied after using it. Quality is very good. Appearance thick new. Thank you for your products.	JD.com
Smart 3C Digital Furniture AD	Battery Reconditioner	Received the goods, tried a few days, the effect of power can be, is a good helper to repair the cell phone battery, so that the old cell phone battery to restore the youth, there is a need to play friends, hurry to order, five stars.	JD.com
Smart 3C Digital Furniture AD	Battery Reconditioner	very good packaging exquisite intact[praise] received and can not wait to try put a long time of the battery since the repair charged up	JD.com
Smart 3C Digital Furniture AD	Battery Reconditioner	Commodities just received, logistics is very fast and punctual, just tried, the effect is good, charging ok, value for money, worthy of a battery repair artifact.	JD.com
Smart 3C Digital Furniture AD	Battery Reconditioner	and the seller described exactly the same, very satisfied, completely exceeded expectations, the delivery speed is very fast, the packaging is very careful and rigorous, the logistics company has a very good service attitude, the delivery speed is very fast, very satisfied with a shopping it~~~	JD.com
Smart 3C Digital [illegible]	Battery Reconditioner	Only after using it to evaluate, just use it once to see the effect,[kudos][kudos][kudos][kudos], quite good, very satisfied	JD.com
Smart 3C Digital [illegible]	Battery Reconditioner	Look good, use to see how the effect, good if the family back to buy!	JD.com
Smart 3C Digital [illegible]	Battery Reconditioner	Great shopping experience classroom assignments.	JD.com

Smart 3C Digital Furniture AD	Battery Reconditioner	Received, the effect of waiting for the use of how to know, looking forward to perfect!	JD.com
Smart 3C Digital Furniture AD	Battery Reconditioner	I don't know if it works or not, I'll comment after I use it.	JD.com
Smart 3C Digital Furniture AD	Battery Reconditioner	Logistics is fast, packaging is fine. I charged once, the goods are real, while charging, while repairing, bright electricity cell phone does not heat up, the effect is obvious. It is worth buying.	JD.com
Smart 3C Digital Furniture AD	Battery Reconditioner	I've been using it for two days and I don't feel any effect, so I hope it works.	JD.com
Smart 3C Digital Furniture AD	Battery Reconditioner	Works very well and is of good quality as the product description! Good value for money, buy again next time.	JD.com
Smart 3C Digital Furniture AD	Battery Reconditioner	Value Appearance: beautiful<br>Rechargeable Battery: more abundant, the service attitude is also very good, worth to buy	JD.com
Smart 3C Digital Furniture AD	Battery Reconditioner	The logistics is fast and the quality is good.	JD.com
Smart 3C Digital Furniture AD	Battery Reconditioner	maintenance of cell phone battery good helper, inexpensive, safe and fast, easy to use is a good choice for cell phone users, five-star praise!	JD.com
Smart 3C Digital Furniture AD	Battery Reconditioner	The phone is fixed. It's really quite good. Fixed it too.	JD.com
Smart 3C Digital Furniture AD	Battery Reconditioner	The service was superb the item was received the next day	JD.com
Smart 3C Digital Furniture AD	Battery Reconditioner	The goods received, it is very good, thank you for the platform without a staff, you have worked hard.	JD.com
Smart 3C Digital Furniture AD	Battery Reconditioner	This little thing, received it, tried it, before coming to sign for it, also wrong oh.	JD.com
Smart 3C Digital Furniture AD	Battery Reconditioner	Received the goods immediately use, found that the phone charging much faster. Originally charging is very slow, this adapter works well worth buying!	JD.com
Smart 3C Digital Furniture AD	Battery Reconditioner	Received the item this morning, great price, worth the money, very happy with the purchase!	JD.com
Smart 3C Digital Furniture AD	Battery Reconditioner	Things received a very good quality, the effect of the use of the end of the comment.	JD.com
Smart 3C Digital Furniture AD	Battery Reconditioner	Fast logistics, good quality, so far the use of okay, cell phone battery repair is okay	JD.com
Smart 3C Digital Furniture AD	Battery Reconditioner	effect, logistics is very fast, satisfied with the shopping[handshake]	JD.com
Smart 3C Digital Furniture AD	Battery Reconditioner	The goods have been received, really_very satisfied	JD.com
Smart 3C Digital Furniture AD	Battery Reconditioner	Charging quickly, the battery is being tried, the value is durable, wait for a few days after the use of a few days to come back to the review, give merchants praise!	JD.com
Smart 3C Digital Furniture AD	Battery Reconditioner	Bargain item, just in stock. Phone usually doesn't hold up to a full charge for the day, hopefully it will improve with use!	JD.com
Smart 3C Digital Furniture AD	Battery Reconditioner	Pulse charging charging effect is very good, fast, repair effect is obvious, care for the battery, extend the service life, very satisfied.	JD.com
Smart 3C Digital Furniture AD	Battery Reconditioner	'Goods have been received to see the contents of a very complex, now charged whether the role of repair is not known, ask customer service to say it again	JD.com
Smart 3C Digital Furniture AD	Battery Reconditioner	Cell phone repairer received, nice style, good quality very satisfied, praise!	JD.com
Smart 3C Digital Furniture AD	Battery Reconditioner	Look very good, logistics is also very fast, you can know how much voltage, how much current, should be good effect	JD.com
Smart 3C Digital Furniture AD	Battery Reconditioner	looking.<br>Battery charging: used a few times, it works.	JD.com
Smart 3C Digital Furniture AD	Battery Reconditioner	The product has been received, the logistics is also very fast, but also with the display function, good to see! Kudos!	JD.com
Smart 3C Digital Furniture AD	Battery Reconditioner	The store is very nice fast shipping I give 5stars good flat	JD.com
Smart 3C Digital Furniture AD	Battery Reconditioner	evaluate, the original cell phone full of electricity, with less than5 6hours, a large part of it is gone, last night with this contraption full of electricity, to the present half of the day cell phone and73%of the electricity, that the phone repair function is still quite good, you can rest assured that the order,,	JD.com
Smart 3C Digital Furniture AD	Battery Reconditioner	is affordable, the lights are cool, it's compact and quite nice.	JD.com
Smart 3C Digital Furniture AD	Battery Reconditioner	I just received the goods today and used the effect is quite good, but also repair is also quite fast!	JD.com
Smart 3C Digital Furniture AD	Battery Reconditioner	Now technology is really advanced, charging quickly, things are affordable, the goods are very good, quite good-looking, and match the picture very well!	JD.com
Smart 3C Digital Furniture AD	Battery Reconditioner	Battery Repairer has received, the logistics is quite fast, stagecoach is good to find, will give friends to your store to do recommendations, give your store five-star praise 👍	JD.com

Smart 3C Digital Furniture AD	Battery Reconditioner	battery life optimization system, not lagging stable charger, did not see the obvious effect, said it can protect the battery, first praise!	JD.com
Smart 3C Digital Furniture AD	Battery Reconditioner	Goods have been received, tried a few days. The effect is still really good, showing the level of high-tech, charging is also very fast, the effect of repair is also very good, it is really value for money, more users can rest assured to buy!	JD.com
Smart 3C Digital Furniture AD	Battery Reconditioner	I just received it, I'm using it now, I'll comment again when I'm done.	JD.com
Smart 3C Digital Furniture AD	Battery Reconditioner	always easy to heat, and charging is getting worse, often poor contact, and the battery is not durable, at least two or three times a day to charge. Inadvertently saw this repairer online, comparison of three, hesitated for a long time to choose this one. Shipping is very fast, two or three days to receive, things did not disappoint, repair charging digital change display, although not quite understand what it means, but I hope it will really have a repair effect Oh!	JD.com
Smart 3C Digital Furniture AD	Battery Reconditioner	It really works, the battery is really durable ah, five stars praise praise praise praise	JD.com
Smart 3C Digital Furniture AD	Battery Reconditioner	I received the stuff, it's very cartoonish, I just started using it, I'm really impressed with the technology nowadays! Thanks for the invention of the gods!	JD.com
Smart 3C Digital Furniture AD	Battery Reconditioner	It is the ideal product, good quality, logistics is also very fast, worthy of praise!	JD.com
Smart 3C Digital Furniture AD	Battery Reconditioner	good to be here[smiles]smiles]smilessmiles] [smiles] [smiles]	JD.com
Smart 3C Digital Furniture AD	Battery Reconditioner	The product has been received, try a very good, repair effect is very good, it is worth buying!	JD.com
Smart 3C Digital Furniture AD	Battery Reconditioner	The item is as described by the merchant, and I was given a free Apple charging cable for my old cell phone Thanks 🙏	JD.com
Smart 3C Digital Furniture AD	Battery Reconditioner	The stuff is very good, the battery time of the phone is longer after using it, recommend to buy.	JD.com
Smart 3C Digital Furniture AD	Battery Reconditioner	Already used to buy for my father, the price is more expensive to have a guarantee ~ to see how the results	JD.com
Smart 3C Digital Furniture AD	Battery Reconditioner	My delivery has been received, the business courier logistics service is good.	JD.com
Smart 3C Digital Furniture AD	Battery Reconditioner	Manual, full of little excitement, use it and then add more!	JD.com
Smart 3C Digital Furniture AD	Battery Reconditioner	Things received, feel good, charging 鈪 night phone does not heat up, it's okay.	JD.com
Smart 3C Digital Furniture AD	Battery Reconditioner	Quite good, it is genuine, recommend everyone to buy, later will buy back	JD.com
Smart 3C Digital Furniture AD	Battery Reconditioner	Good quality, works fine, phone cleans up about 90%, good overall	JD.com
Smart 3C Digital Furniture AD	Battery Reconditioner	Superb, highly recommended, quick fix, very patient and professional customer service!	JD.com
Smart 3C Digital Furniture AD	Battery Reconditioner	first time I used the cell phone battery intelligent repairer, full of black technology three major functions "charging ➕ repair ➕ maintenance", very practical! After using it, it is obvious that the cell phone battery is more durable, the whole charging system of the Apple phone has been optimized, and the battery capacity has increased by 1%!	JD.com
Smart 3C Digital Furniture AD	Battery Reconditioner	Received today on the use, really the effect is particularly obvious, really black technology, play the game very fast, but also not stuck[praise] [praise] [praise] [praise] [praise]	JD.com
Smart 3C Digital Furniture AD	Battery Reconditioner	ready to replace the new phone, listen to friends say this restorer, try the mentality, did not think I used a week after the battery is obviously durable, dozens of dollars is too cost-effective!	JD.com
Smart 3C Digital Furniture AD	Battery Reconditioner	It works very well.	JD.com
Smart 3C Digital Furniture AD	Battery Reconditioner	It's great, fast charging, plenty of power, and can be used as a torch🔦and lighting	JD.com
Smart 3C Digital Furniture AD	Battery Reconditioner	It works.	JD.com
Smart 3C Digital Furniture AD	Battery Reconditioner	It's a great deal, and after using it for a while the cell phone battery lasts all day!	JD.com
Home Life Equipment	Cell Phone Battery Adapter	Cell phone charging does not hot, very good, has been used to see how the later	JD.com
Home Life Equipment	Cell Phone Battery Adapter	I just used it, I don't know if it's good or not, I'll come back to comment after using it for a while.	JD.com
Home Life Equipment	Cell Phone Battery Adapter	Cell phone repairer received, and has been used for a few days, the effect of how, at present do not know, I believe that Jackie Chan endorsement of the product, customer service is very on point, ask and answer. First give five-star praise, the effect is good and then follow up.	JD.com

Home Life Equipment	Cell Phone Battery Adapter	Smart Fixer works real No scam No false advertising Worth buying I have a few cell phones in my hand The battery doesn't work at all It doesn't turn on at all It all fixes It's worth buying Trust It's also cheap and afford The merchant ships fast	JD.com
Home Life Equipment	Cell Phone Battery	Not yet used. Evaluation after use. First five-star praise!	JD.com
Home Life Equipment	Cell Phone Battery	IQ tax	JD.com
Home Life Equipment	Cell Phone Battery Adapter	charger does not charge, the phone charging is also slow, charging the android phone the more charging power less, the opposite effect, the	JD.com
Home Life Equipment	Cell Phone Battery	Cell phone smart repair received quality is still good very good very satisfied praise	JD.com
Home Life Equipment	Cell Phone Battery	Logistics is very fast, the product performance effect, to be used after the evaluation.	JD.com
Home Life Equipment	Cell Phone Battery	Works fine, battery lasts	JD.com
Home Life Equipment	Cell Phone Battery	condition has improved, try again for a while before commenting.	JD.com
Home Life Equipment	Cell Phone Battery	I just plugged it in and used it, I don't know how effective it is, hopefully it can repair the battery	JD.com
Home Life Equipment	Cell Phone Battery	Fake and shoddy products claiming refunds and returns	JD.com
Home Life Equipment	Cell Phone Battery	This user feels the product is better	JD.com
Home Life Equipment	Cell Phone Battery	Received the goods and tried quite good, worth buying!	JD.com
Home Life Equipment	Cell Phone Battery Adapter	This cell phone repairer received, the small thing looks pretty good, fast shipping, fast logistics, there are instructions very good, to be used and then follow up. Merchant service is good, all five-star praise.	JD.com
Home Life Equipment	Cell Phone Battery	Running speed: Nice and effective. I'm sleeping.	JD.com
Home Life Equipment	Cell Phone Battery	This user feels the product is better	JD.com
Home Life Equipment	Cell Phone Battery	Plugging in the boot prompts protection, and it's hard to find out from the surface how well it actually works.	JD.com
Home Life Equipment	Cell Phone Battery	Fast delivery, good service, and the medication is very efficacious	JD.com
Home Life Equipment	Cell Phone Battery	Charged a few times, the phone is not so hot, continue to charge, pretty good five stars!	JD.com
Home Life Equipment	Cell Phone Battery	Received, in use, affordable price, good comments, next time to buy back.	JD.com
Home Life Equipment	Cell Phone Battery	This user feels the product is better	JD.com
Home Life Equipment	Cell Phone Battery	Haven't seen very noticeable results yet, keep it for further use	JD.com
Home Life Equipment	Cell Phone Battery	(of an unmarried couple) be close	JD.com
Home Life Equipment	Cell Phone Battery Adapter	Cell phone smart repair device has received, my cell phone this year has come to full power can only be used for a day on the power, there is a bit of lag, since the use of this cell phone smart repair device after charging for four times there is a significant effect, the phone is not stuck, normal use of full power available for more than two days, good.	JD.com
Home Life Equipment	Cell Phone Battery	The logistics are fast and the price is suitable.	JD.com
Home Life Equipment	Cell Phone Battery	Good stuff, 5 stars now!	JD.com
Home Life Equipment	Cell Phone Battery	Logistics is very fast and the service is attentive. Genuine products will be evaluated after use.	JD.com
Home Life Equipment	Cell Phone Battery	feeling after the repair, logistics is also very fast no damage, the	JD.com
Home Life Equipment	Cell Phone Battery Adapter	Yesterday afternoon received a cell phone with a repair battery charger, used more than a day, have not yet seen the effect, have the effect to come back to follow up on it!	JD.com
Home Life Equipment	Cell Phone Battery Adapter	Repair device received, logistics is very fast, received yesterday, Yun Dian tried, the phone does not heat, there is a voice broadcast, as for whether to repair the electric plate, to be used to decide, first five-star praise.	JD.com
Home Life Equipment	Cell Phone Battery	It's great. I've been using it for a couple days now. It works great.	JD.com
Home Life Equipment	Cell Phone Battery	The voice one feels pretty good and is commendable for its good value for money	JD.com
Home Life Equipment	Cell Phone Battery	I've received the goods, I'll comment after use	JD.com
Home Life Equipment	Cell Phone Battery	It's not much use.	JD.com

Home Life Equipment	Cell Phone Battery Adapter	I received it and felt more comfortable seeing that it had a security marking.	JD.com
Home Life Equipment	Cell Phone Battery Adapter	It doesn't seem to be working.	JD.com
Home Life Equipment	Cell Phone Battery Adapter	Used once, there is a voice feel good, with a period of time and then look at the hope that the cell phone battery repair is useful, if good will recommend friends to buy!	JD.com
Home Life Equipment	Cell Phone Battery Adapter	it works later. First of all, I would like to give a favorable comment.	JD.com
Home Life Equipment	Cell Phone Battery Adapter	This will take time to verify, will follow up later through use	JD.com
Home Life Equipment	Cell Phone Battery Adapter	immediately upon insertion, just what a poor quality product,[stammers]	JD.com
Home Life Equipment	Cell Phone Battery Adapter	Baby is too amazing, the phone with a few years of SamsungS21U, before full only85, using this repairer, repair full100, recommended, satisfied! Full marks!	JD.com
Home Life Equipment	Cell Phone Battery Adapter	East East received a very good quality worth buying Oh!	JD.com
Home Life Equipment	Cell Phone Battery Adapter	Repairer received the goods, try a period of time to see, to be good to buy a	JD.com
Home Life Equipment	Cell Phone Battery Adapter	As described by the seller, very satisfied and effective	JD.com
Home Life Equipment	Cell Phone Battery Adapter	It's charging up! It's talking and saying it's restored! But I don't think it's working at all.	JD.com
Home Life Equipment	Cell Phone Battery Adapter	I've just started using it and I don't know how well it works!	JD.com
Home Life Equipment	Cell Phone Battery Adapter	Have started to use, will voice instructions, how effective wait for a follow-up review later.	JD.com
Home Life Equipment	Cell Phone Battery Adapter	few days to evaluate,, the effect of repair is good, the phone is not hot, there are voice prompts, very like, fast logistics, good business attitude, the need to hurry to order it.	JD.com
Home Life Equipment	Cell Phone Battery Adapter	Logistics is very fast, the appearance of the goods regular, to be used after the follow-up evaluation.	JD.com
Home Life Equipment	Cell Phone Battery Adapter	Got the stuff, works great, worth the money!	JD.com
Home Life Equipment	Cell Phone Battery Adapter	The goods have been received, pulse charging, voice prompts, text prompts, the price is okay! Wait until after the try to know the effect. By that time then follow up the evaluation!	JD.com
Home Life Equipment	Cell Phone Battery Adapter	Receive the product and use it before commenting	JD.com
Home Life Equipment	Cell Phone Battery Adapter	This product is really good , I received it yesterday and started to use it , originally my cell phone was charging and got hot , yesterday I used this multi-functional charging treasure and it didn't get hot	JD.com
Home Life Equipment	Cell Phone Battery Adapter	After using it, the effect is good, the phone does not lag anymore, it is worth promoting.	JD.com
Home Life Equipment	Cell Phone Battery Adapter	I don't know yet.	JD.com
Home Life Equipment	Cell Phone Battery Adapter	Simple to use, customer service is very patient, do not understand, ask and do not bother, one by one to answer the product, fine workmanship, good use	JD.com
Home Life Equipment	Cell Phone Battery Adapter	Satisfaction.	JD.com
Home Life Equipment	Cell Phone Battery Adapter	The product arrived at my home I'm very happy with it thank you	JD.com
Home Life Equipment	Cell Phone Battery Adapter	The product arrived at my home I'm very happy with it thank you	JD.com
Home Life Equipment	Cell Phone Battery Adapter	Evaluate after you've used it.	JD.com
Home Life Equipment	Cell Phone Battery Adapter	The Smart Repair Charger was received in perfect packaging. Used it right after receiving. Charging fast. The phone does not get hot. Works well, recommended.	JD.com
Home Life Equipment	Cell Phone Battery Adapter	Cell phone battery adapter received. Merchant shipped very quickly. Packaging is intact. Goods are very formal. The price is reasonable. The effect is good. In the repurchase. Ah logistics is also fast. Satisfied with the five-star praise.	JD.com
Home Life Equipment	Cell Phone Battery Adapter	Goods received, the quality is very good, is genuine, is using the effect to be used for a period of time can be seen! Store service attitude good degree of good!	JD.com
Home Life Equipment	Cell Phone Battery Adapter	used. The effect of how to use a period of time and then in the follow-up evaluation. At this time on customer service, logistics, packaging and other aspects are satisfied. Five-star praise!	JD.com
Home Life Equipment	Cell Phone Battery Adapter	Bought back a few days, charged the phone a few times, feel the phone battery is much more durable than before, a lot smoother, heat slowly reduced, worth buying a good product.	JD.com

Home Life Equipment	Cell Phone Battery	I haven't felt if it functions as well as presented yet, I'll say more after using it for a while.	JD.com
Home Life Equipment	Cell Phone Battery	Good value for money, economical, generous and beautiful	JD.com
Home Life Equipment	Cell Phone Battery Adapter	try a few hours, before this because the phone class, the phone will often be hot, today with this east side of the charging side with the phone class, it seems to be a little bit of a role, the phone is not so hot. Hopefully, it has been good, but the business gave a guarantee, you can try 180days, not good enough to use the package back. So we consumers have protection. We can buy with confidence!	JD.com
Home Life Equipment	Cell Phone Battery Adapter	The quality is great[love] [great love]great]love[great] [great love] [great love] [great love] [great love] [great love] [great love] [great love] [great love] [great love] [great love] [great love]	JD.com
Home Life Equipment	Cell Phone Battery	The goods are consistent with the description, test for a period of time to evaluate, the first five stars	JD.com
Home Life Equipment	Cell Phone Battery	The restorer works great, got the right one. It's genuine.	JD.com
Home Life Equipment	Cell Phone Battery Adapter	Fast, fast shipping fast logistics, the receipt of goods, tried, there is a voice function, see the description of the effect should be very good, thank you.	JD.com
Home Life Equipment	Cell Phone Battery	It didn't feel restorative!	JD.com
Home Life Equipment	Cell Phone Battery	effective	JD.com
Home Life Equipment	Cell Phone Battery	Goods have been received Packaging is flawless Quality is very good Courier service is good Thank you!	JD.com
Home Life Equipment	Cell Phone Battery	Very happy with the purchase!	JD.com
Home Life Equipment	Cell Phone Battery	serviceable	JD.com
Home Life Equipment	Cell Phone Battery	I received it, the logistics were fast, the color was good, and it was a good review.	JD.com
Home Life Equipment	Cell Phone Battery	A proper IQ tax!	JD.com
Home Life Equipment	Cell Phone Battery	Fast shipping, stable packaging, compact, powerful, voice prompts, easy to use, worth buying!	JD.com
Home Life Equipment	Cell Phone Battery	Works great, good review.	JD.com
Home Life Equipment	Cell Phone Battery	Shipping? also in a hurry. Awesome!!!	JD.com
Home Life Equipment	Cell Phone Battery	review full of kudos! I hope the store serves customers well as always!	JD.com
Home Life Equipment	Cell Phone Battery Adapter	The goods have been received, the packaging is complete, the goods are not extruded phenomenon… intact, thanks for the cooperation happy!	JD.com
Home Life Equipment	Cell Phone Battery	Running speed: Very good, a real cell phone fixer! [Kudos] [Kudos] [Kudos] [Kudos]	JD.com
Home Life Equipment	Cell Phone Battery Adapter	After receiving the goods, Lang carries on the charging repair to the cell phone, first of all, I feel that the charging is a little faster than before, and the length of use is extended after full. Root manual introduced, after a few days of repair to complete, I hope to have good results!	JD.com
Home Life Equipment	Cell Phone Battery	It's great, love it!	JD.com
Home Life Equipment	Cell Phone Battery Adapter	product, regular packaging, manuals and qualification trusts should be complete, with voice broadcast function and text display; logistics and delivery is very fast, three days to the home.	JD.com
Home Life Equipment	Cell Phone Battery	Convenient, simple and works well!	JD.com
Home Life Equipment	Cell Phone Battery	The new charger, which will bring new life to the newborn cell phone, is worth buying.	JD.com
Home Life Equipment	Cell Phone Battery	I got it. - Thank you.	JD.com
Home Life Equipment	Cell Phone Battery	Trustworthy product and genuine goods! I am very satisfied!	JD.com
Home Life Equipment	Cell Phone Battery	I got it. It's working[Kudos] [Kudos]	JD.com
Home Life Equipment	Cell Phone Battery	Product performance: favorable comments	JD.com
Home Life Equipment	Cell Phone Battery	Just want to say IQ tax.[Lots of love] [Lots of love]	JD.com
Home Life Equipment	Cell Phone Battery	Voice cell phone repairer has been received, logistics is very fast, the effect is good, five-star praise!	JD.com
Home Life Equipment	Cell Phone Battery	Good price, logistics is very fast, used a few days feel good, the old phone is a little bit of electricity, continue to use.	JD.com

Home Life Equipment	Cell Phone Battery	I don't know if it works.	JD.com
Home Life Equipment	Cell Phone Battery	:	JD.com
Home Life Equipment	Cell Phone Battery	Baby received, used2times to feel the phone smooth, battery durable, is genuine!	JD.com
Home Life Equipment	Cell Phone Battery	Goods received well packaged. Use it to see if it is repaired. Little brother service is good.	JD.com
Home Life Equipment	Cell Phone Battery	The product is good and the logistics is fast	JD.com
Home Life Equipment	Cell Phone Battery	It is being used with the late effect of how, to be observed.	JD.com
Home Life Equipment	Cell Phone Battery	Use it and evaluate again	JD.com
Home Life Equipment	Cell Phone Battery	Have been using it for a while before commenting	JD.com
Home Life Equipment	Cell Phone Battery Adapter	Has been used, the battery repair is very good, the power is more durable, the effect is very good, there are like you can rest assured that purchase	JD.com
Home Life Equipment	Cell Phone Battery	The quality of the product is good also affordable really good	JD.com
Home Life Equipment	Cell Phone Battery	Fast shipping and packaging good business to give a real smart fix need to grab the order!	JD.com
Home Life Equipment	Cell Phone Battery	Baby received pretty good[smile]is a satisfied shopping!	JD.com
Home Life Equipment	Cell Phone Battery	The quality is great! Small and delicate! Protective for cell phones! Extend the life! Worth recommending to buy!	JD.com
Home Life Equipment	Cell Phone Battery	I don't know if it works yet.	JD.com
Home Life Equipment	Cell Phone Battery	Product quality is good love it	JD.com
Home Life Equipment	Cell Phone Battery	Baby has received! Logistics is very fast, well-packed tried the effect is very good!	JD.com
Home Life Equipment	Cell Phone Battery	Tried it for a day and it seems to be working a little bit	JD.com
Home Life Equipment	Cell Phone Battery	Easy to use, charging doesn't interfere, should respond to cell phones .	JD.com
Home Life Equipment	Cell Phone Battery	cell phone port	JD.com
Home Life Equipment	Cell Phone Battery	an excellent	JD.com
Home Life Equipment	Cell Phone Battery	use? It's not good for 3-4 days. It's not24hours	JD.com
Home Life Equipment	Cell Phone Battery	'm using it,'t knowif it's any good until I use it	JD.com
Home Life Equipment	Cell Phone Battery	Received.Satisfied.	JD.com
Home Life Equipment	Cell Phone Battery	I'm not going to rate it yet, I'll wait to use it.	JD.com
Home Life Equipment	Cell Phone Battery Adapter	Fast shipping, packaging intact, original genuine, read the instruction manual, to be used for a few weeks will effectively improve the battery life, optimize the phone system, affordable price, give praise.	JD.com
Home Life Equipment	Cell Phone Battery	It's good.	JD.com
Home Life Equipment	Cell Phone Battery Adapter	I don't know whether it works or not, I won't know until I use it, but I hope it works, I believe in black technology, and I will give it a five-star review first!	JD.com
Home Life Equipment	Cell Phone Battery	Received, have not yet used, after using it to evaluate again	JD.com
Home Life Equipment	Cell Phone Battery Adapter	Running Speed: Smooth<br>Operating System: Simple<br>Product Performance: Reliable is a very good cell phone battery big savior protector, Golden Shield! Golden Shield! Golden Shield! The big brand is trustworthy!	JD.com
Home Life Equipment	Cell Phone Battery	Not used yet, see the effect and then evaluate	JD.com
Home Life Equipment	Cell Phone Battery	The merchandise is okay.	JD.com
Home Life Equipment	Cell Phone Battery Adapter	hindered the charging, because I2sets of cell phones, was just a battery can not be charged, after using this, the other can not charge……unplugged this is able to charge normally[grinning]	JD.com
Home Life Equipment	Cell Phone Battery	I don't know the effect.	JD.com
Home Life Equipment	Cell Phone Battery	I'm going to give you a 5 star rating first, I'll follow up after I've used it for a while	JD.com

Home Life Equipment	Cell Phone Battery Adapter	Goods have been received, has been used twice, before the phone will be hot, the first time to use did not feel any difference, the second time to use the obvious place, the phone is not hot, and the description of the same, good value for money, the next time will come back to patronize!	JD.com
Home Life Equipment	Cell Phone Battery Adapter	Logistics is very fast, the item is intact, with a very comfortable goods received, do not know if there is no effect, after using in said.	JD.com
Home Life Equipment	Cell Phone Battery	Will follow up with a review after I use it!	JD.com
Home Life Equipment	Cell Phone Battery	The charger works.	JD.com
Home Life Equipment	Cell Phone Battery Adapter	Repair device has been received, the logistics is very fast, opened to try a good effect, charging try a cell phone does not heat up, there are Chinese and English voice broadcast, as to whether or not the repair of the electric board to be used to decide, it looks like there is a certain effect, if it is good and then back to buy, five-star praise!	JD.com
Home Life Equipment	Cell Phone Battery	Second purchase, bought for a friend	JD.com
Home Life Equipment	Cell Phone Battery	cell phone	JD.com
Home Life Equipment	Cell Phone Battery	The product received, the quality is very good, as the anchor introduced, it is genuine, thank you!	JD.com
Home Life Equipment	Cell Phone Battery	Have not yet used the quality to be verified good or bad, later comments	JD.com
Home Life Equipment	Cell Phone Battery	Just got it and will comment after using it for a while.	JD.com
Home Life Equipment	Cell Phone Battery	This user feels the product is better	JD.com
Home Life Equipment	Cell Phone Battery	Received the goods, use it first and then add a follow-up review.	JD.com
Home Life Equipment	Cell Phone Battery	This user feels the product is better	JD.com
Home Life Equipment	Cell Phone Battery	It's good, it works, I love it, I[big love],works	JD.com
Home Life Equipment	Cell Phone Battery	Shipping and charging is fast, easy to operate, and a full charge lasts longer	JD.com
Home Life Equipment	Cell Phone Battery Adapter	is to try to charge, from the effect, obviously the phone a change in charging fever, and the phone no longer appear jamming, as the page description is very satisfied![Kudos][Kudos][Kudos][Kudos]	JD.com
Home Life Equipment	Cell Phone Battery	Copy that. It's good!	JD.com
Home Life Equipment	Cell Phone Battery	It's good, with voice function. Use it first.	JD.com
Home Life Equipment	Cell Phone Battery	. Affordable	JD.com
Home Life Equipment	Cell Phone Battery	Not yet used	JD.com
Home Life Equipment	Cell Phone Battery Adapter	Receive to, the photographed items in line, after a few days of trial before coming to evaluate, repair the phone heat, card tons of problems have improved the effect (note my phone has been used for two or three years long). First of all, I affirm that I wrote a full five-star praise is not the store's drag". It is worth promoting the use of.	JD.com
Home Life Equipment	Cell Phone Battery	of cell phone heat, over-soul garbage, speeding up cell phone charging.	JD.com
Home Life Equipment	Cell Phone Battery	This product is very good, next time I buy it with my friends	JD.com
Home Life Equipment	Cell Phone Battery	I just received it, I don't know how it works, but it doesn't automatically power off when it's full, which is not ideal.	JD.com
Home Life Equipment	Cell Phone Battery	Goods have been received, easy to use and well made.	JD.com
Home Life Equipment	Cell Phone Battery	I've been using it since I got it back. I don't know if it works!	JD.com
Home Life Equipment	Cell Phone Battery	It's not as described, it says recognizable repairable battery, the fake doesn't work at all.	JD.com
Home Life Equipment	Cell Phone Battery	received	JD.com
Home Life Equipment	Cell Phone Battery	Received it last night, forgot to comment, in use, will follow up in the future!	JD.com
Home Life Equipment	Cell Phone Battery	Evaluate after use	JD.com
Home Life Equipment	Cell Phone Battery	Baby loves it [illegible]!	JD.com
Home Life Equipment	Cell Phone Battery	The cell phone adapter was received, fast shipping, quick delivery, is experiencing, looking forward to the effect!	JD.com

Home Life Equipment	Cell Phone Battery	I don't know how it is yet, I'll use it first and then I'll comment.	JD.com
Home Life Equipment	Cell Phone Battery	Battery adapter received. Works well. Good value for money. Logistics is also fast.	JD.com
Home Life Equipment	Cell Phone Battery	With the description is the same, very high-grade, good value, to buy friends can order Oh!	JD.com
Home Life Equipment	Cell Phone Battery Adapter	Packaging is very solid, logistics is also particularly fast, things get very satisfied, consistent with the description. Very satisfied with a shopping.	JD.com
Home Life Equipment	Cell Phone Battery Adapter	The little thing is pretty good, I came back and charged my cell phone with it once, and it felt like the battery lasted longer than before.	JD.com
Home Life Equipment	Cell Phone Battery	Small positive feedback, if the results are ok, I will definitely follow up!	JD.com
Home Life Equipment	Cell Phone Battery Adapter	Baby especially like, the value of high-quality good, a very good shopping experience, attentive service, fast logistics, distribution is also very thoughtful, attitude is also very good, value for money ah, decisive praise!<br>Quality is quite good, logistics is also very fast, praise!	JD.com
Home Life Equipment	Cell Phone Battery	Fast courier delivery doesn't work	JD.com
Home Life Equipment	Cell Phone Battery	Repairer received immediately after the use of good results[Rose][Kudos]logistics quickly[Rose][Kudos]	JD.com
Home Life Equipment	Cell Phone Battery	It's not open.	JD.com
Home Life Equipment	Cell Phone Battery	Runs fast and easy to operate the phone really does not get hot!	JD.com
Home Life Equipment	Cell Phone Battery	an excellent	JD.com
Home Life Equipment	Cell Phone Battery	Fast shipping, good logistics, no damage, looking forward to results	JD.com
Home Life Equipment	Cell Phone Battery	before, no change at all, the phone is still hot after a long time.	JD.com
Home Life Equipment	Cell Phone Battery	Affordable, durable battery, normal use, fine workmanship	JD.com
Home Life Equipment	Cell Phone Battery	It's genuine, works well, and the logistics is fast.	JD.com
Home Life Equipment	Cell Phone Battery	Prompt delivery, as described, looks good.	JD.com
Home Life Equipment	Cell Phone Battery	Affordable, works fine, well made, great product, very good, good quality	JD.com
Home Life Equipment	Cell Phone Battery	Item received, the boss shipped quickly, express delivery, very satisfied!	JD.com
Home Life Equipment	Cell Phone Battery	Product received, packaging is very good, logistics is very fast, thank you boss!	JD.com
Home Life Equipment	Cell Phone Battery	This user feels the product is better	JD.com
Home Life Equipment	Cell Phone Battery	Received a few days, the use of the cell phone battery effect is really great👍👍👍👍👍	JD.com
Home Life Equipment	Cell Phone Battery	Received their own started to use, good workmanship small and exquisite, good to recommend friends to buy.	JD.com
Home Life Equipment	Cell Phone Battery	Just started using it, expecting full marks, waiting for the good news, & follow up reviews!	JD.com
Home Life Equipment	Cell Phone Battery	I got it. Thank you.	JD.com
Home Life Equipment	Cell Phone Battery Adapter	Is genuine. With the description of the same, warranty box formal manufacturer and instructions, very good, because the use of electric car battery repairer using the process is very good, this should also be the same, the subsequent use of the process for follow-up Oh!	JD.com
Home Life Equipment	Cell Phone Battery Adapter	Goods received. Quality qualified. There is a voice prompt after powering on. Prevent the phone from heating up, fast logistics. Five-star praise.	JD.com
Home Life Equipment	Cell Phone Battery Adapter	The cell phone repairer is of good quality and fine workmanship, such as the cell phone is hot and becomes stuck can use this repairer to repair, can effectively improve these symptoms. Worth buying.	JD.com
Home Life Equipment	Cell Phone Battery	I just received the item and will try it out before commenting.	JD.com
Home Life Equipment	Cell Phone Battery	Very good, logistics is also very fast, next time to go back to buy	JD.com
Home Life Equipment	Cell Phone Battery	Received, a small one, do not know if it is useful, give a five-star praise	JD.com
Home Life Equipment	Cell Phone Battery	Thank you boss received the goods, but the quality is quite good. Buy with confidence, everyone. Thank you!	JD.com
Home Life Equipment	Cell Phone Battery	Baby received, satisfied, all 5 stars.	JD.com
Home Life Equipment	Cell Phone Battery	Received the goods, is in use, very satisfied!	JD.com

Home Life Equipment	Cell Phone Battery Adapter	okay, I hope that the future will be able to please to the logo requirements. Fast logistics. I like it! Five stars praise!	JD.com
Home Life Equipment	Cell Phone Battery Adapter	It's all working, really good, worth buying, give it a good review!	JD.com
Home Life Equipment	Cell Phone Battery Adapter	started with the phone only thirty-nine percent, and then contacted the business, he asked me to try a few more times, I felt useless, and then a colleague to help me use his charger to try, charging well, and then come back at night to try again or useless, and then change the charging line, and then can be, and then I tried to charge with the original charger and can be, I don't know if it's a coincidence, or the repair device! Now I don't know if it's a coincidence or if the fixer is working, I can't figure it out. I don't know whether it's a coincidence or the repairer is working.	JD.com
Home Life Equipment	Cell Phone Battery Adapter	Tried it and it didn't work at all, just plugged it in for the first time and then it didn't work for the second time, and it didn't fix the battery.	JD.com
Home Life Equipment	Cell Phone Battery Adapter	Just received the baby, feel pretty good, logistics is also very fast, now use it, looking forward to the effect, good use, later come back to evaluate, first give merchants praise!	JD.com
Home Life Equipment	Cell Phone Battery Adapter	It's good. It's got voice prompts.	JD.com
Home Life Equipment	Cell Phone Battery Adapter	Received the goods logistics fast, good service attitude, give full five-star praise.	JD.com
Home Life Equipment	Cell Phone Battery Adapter	such as shattered screens and damaged batteries. This is the case with my cell phone. Fixers usually use technology to repair hardware problems with cell phones, avoiding the time and expense of going to a professional repair store. There are also some unscrupulous people who use cell phone fixers to commit fraud. They may offer false fixes, charge high fees without solving the problem, and may even cause more damage to the phone.<br><br>When choosing a cell phone fixer, pay attention to the following points: choose a regular brand and channel to buy a cell phone fixer to avoid buying counterfeit products. Understand the specific functions and scope of application of the fixer to ensure that it can solve your cell phone problems. If you are not familiar with the use of cell phone repairer, it is recommended that you try to find a professional repairer to repair your cell phone so as not to cause more damage.<br><br>My cell phone recently went to the repair department entity store, the repair staff said the battery has a bulge, take out the screen may be shattered, the repair fee in more than four hundred, the final consideration or not repair. This cell phone repairer used two days, looked at the bulging screen cracks did go down. The cell phone repairer can be charged,	JD.com
Home Life Equipment	Cell Phone Battery Adapter	We'll talk about it later.	JD.com
Home Life Equipment	Cell Phone Battery Adapter	Logistics is fast worth buying.	JD.com
Home Life Equipment	Cell Phone Battery Adapter	Confirmation of receipt. Logistics was pretty fast too, try charging it for two nights first. See if there is a possible boost to the phone battery	JD.com
Home Life Equipment	Cell Phone Battery Adapter	Goods received logistics fast, cheap, next time I will buy.	JD.com
Home Life Equipment	Cell Phone Battery Adapter	Very good and satisfied with the service	JD.com
Home Life Equipment	Cell Phone Battery Adapter	The goods received, thank you, cell phone charging repairer is very good everyone who use can buy yo.	JD.com
Home Life Equipment	Cell Phone Battery Adapter	I received the baby, haven't tried it yet, love it.	JD.com
Home Life Equipment	Cell Phone Battery Adapter	really solve the big problem, very good. Thank you for your business.	JD.com
Home Life Equipment	Cell Phone Battery Adapter	Go outside to buy are not this price, business attitude is very good, line. Service attitude is quite good.	JD.com
Home Life Equipment	Cell Phone Battery Adapter	Online shopping is so intense, I did not expect the store's service is so good, the goods are good quality and cheap, the seller is very enthusiastic, next time come back!	JD.com
Home Life Equipment	Cell Phone Battery Adapter	The owner is very nice, I will come back again good seller awesome, fast shipping, very nice, very satisfied! Awesome!	JD.com
Home Life Equipment	Cell Phone Battery Adapter	quickly, very satisfied, shopping quality is very good, hope more friends trust.	JD.com
Home Life Equipment	Cell Phone Battery Adapter	strict, very strict, the logistics company has a very good service attitude	JD.com

Home Life Equipment	Cell Phone Battery	Great quality, exactly as described by the seller, really like it, completely exceeded expectations!	JD.com
Home Life Equipment	Cell Phone Battery	It's really not easy to find a top quality business, I'll keep buying from your house and give you 5 stars!	JD.com
Home Life Equipment	Cell Phone Battery Adapter	speed of the courier is also powerful, after receiving open to see, all kinds of things put very complete, complete packaging.	JD.com
Home Life Equipment	Cell Phone Battery Adapter	Packaging is very careful, strict, logistics company service attitude is very good, fast delivery, very satisfied with a shoppingit~~~	JD.com
Home Life Equipment	Cell Phone Battery Adapter	the same as the seller's description, very satisfied, completely exceeded expectations, the shipping speed is very fast!	JD.com
Home Life Equipment	Cell Phone Battery	family in the future. In short: satisfied! Satisfied! Satisfied!	JD.com
Home Life Equipment	Cell Phone Battery	hesitant friends hurry up and get down, conscientious recommendation!	JD.com
Home Life Equipment	Cell Phone Battery	recommended~still waiting for friends can not hesitate to get down!	JD.com
Home Life Equipment	Cell Phone Battery Adapter	Quality is very good, and the seller described exactly the same, completely exceeded expectations, packaging is very careful, very satisfied with a purchase	JD.com
Home Life Equipment	Cell Phone Battery Adapter	The packaging is very good, the courier is strong, the seller is very reputable, and the shipping speed is fast. The price is even more affordable than others!	JD.com
Home Life Equipment	Cell Phone Battery Adapter	Genuine, good quality, low price, high sex ratio, good value for money, value for money, friends look at all say good. Customer service enthusiasm are to. Logistics is very strong	JD.com
Home Life Equipment	Cell Phone Battery Adapter	my expectations, the packaging is very careful and strict, in general this is a very satisfactory shopping, thanks to the seller.	JD.com
Home Life Equipment	Cell Phone Battery	The thing received, fix the phone memory shortage, very good, the phone is hot also have good results.	JD.com
Home Life Equipment	Cell Phone Battery	Inexpensive, affordable, convenient and practical.	JD.com
Home Life Equipment	Cell Phone Battery	Good quality, good use	JD.com
Home Life Equipment	Cell Phone Battery	Good service good goods good logistics very fast	JD.com
Home Life Equipment	Cell Phone Battery	It's great. It's got voice prompts. It's great.	JD.com
Home Life Equipment	Cell Phone Battery	The logistics are fast, the charger works well.	JD.com
Home Life Equipment	Cell Phone Battery	Received the goods soon after the order Business fast shipping logistics fast arrival in a timely manner well-packed	JD.com
Home Life Equipment	Cell Phone Battery	Baby received the logistics very quickly well-packed good quality points praise!	JD.com
Home Life Equipment	Cell Phone Battery Adapter	When your phone is used for a long time, it will become hot and become stuck, you can use this fixer to fix it, plug it directly into your phone and it will work, it can effectively improve these symptoms	JD.com
Home Life Equipment	Cell Phone Battery	Bought home and tried, okay not bad, should be a good quality product, logistics is fast, worth having.	JD.com
Home Life Equipment	Cell Phone Battery	very good, the courier is also very powerful, super satisfied with the	JD.com
Home Life Equipment	Cell Phone Battery Adapter	packaging is very good, still do not know how the effect? I'll come back after using it! Thank you for the hard work of the courier boy! Thank you!	JD.com
Home Life Equipment	Cell Phone Battery	Just received the goods, the logistics is very fast, try it, after a few days to say.	JD.com
Home Life Equipment	Cell Phone Battery	Repairer received, not yet used, with a period of time to add evaluation, but hopefully effective!	JD.com
Home Life Equipment	Cell Phone Battery	This repair machine, very good, factory direct very cheap, good	JD.com
Home Life Equipment	Cell Phone Battery Adapter	Goods have been received, there is no effect currently do not know, has been plugged into the charging, it looks good, first give a favorable review of it	JD.com
Home Life Equipment	Cell Phone Battery	The product performance is okay, the feel is also good, the function is okay, the effect is good. A satisfactory shopping.	JD.com
Home Life Equipment	Cell Phone Battery	Works fine, battery is durable and economical, charger works well, great product!	JD.com
Home Life Equipment	Cell Phone Battery	Thanks to the merchant and thanks to the delivery guy."	JD.com
Home Life Equipment	Cell Phone Battery	"Received the item and looking forward to using it.	JD.com
Home Life Equipment	Cell Phone Battery	Good goods received, fast logistics, good quality	JD.com

Home Life Equipment	Cell Phone Battery Adapter	don't know how well the repair works We'll see if the repair works afterward	JD.com
Home Life Equipment	Cell Phone Battery Adapter	Courier service is good, packaging is very good, repair effect comment after use.	JD.com
Home Life Equipment	Cell Phone Battery Adapter	Used normally, good, works well, used before commenting	JD.com
Home Life Equipment	Cell Phone Battery Adapter	particularly good, customer service attitude is better, and customer service introduced the same, the quality is quite good, integrity first, thank you. Will buy your goods in the future	JD.com
Home Life Equipment	Cell Phone Battery Adapter	Voice prompts, smart repair of the phone, good! Have recommended to friends to buy!	JD.com
Home Life Equipment	Cell Phone Battery Adapter	Received the goods are trying out, praise five points.	JD.com
Home Life Equipment	Cell Phone Battery Adapter	The logistics is quite fast, the quality is quite good, very satisfied	JD.com
Home Life Equipment	Cell Phone Battery Adapter	Fast logistics, good packaging, goods intact. Easy to use, the appearance is quite good	JD.com
Home Life Equipment	Cell Phone Battery Adapter	It works great. The phone is often hot, with this it is not hot anymore and the battery is durable.	JD.com
Home Life Equipment	Cell Phone Battery Adapter	plays a role in off building, for the majority of cell phone friends made a very big contribution to thank the developer, made a great contribution. Thank you boss and courier brother, next time again.	JD.com
Home Life Equipment	Cell Phone Battery Adapter	Cell phone repairer received, used for two days, charging is not hot, the quality is quite good, fast logistics, good service attitude, give a five-star praise!	JD.com
Home Life Equipment	Battery Adapter	I have not used it, I don't know if it is good or not, I am looking forward to a good result!	JD.com
Home Life Equipment	Cell Phone Battery Adapter	This user feels the product is better	JD.com
Home Life Equipment	Cell Phone Battery Adapter	aspects of normal, feel the effect is not very obvious, wait a few days to see the effect and then make up the comment. First give five stars.	JD.com
Home Life Equipment	Cell Phone Battery Adapter	Goods received, have not tried, it is difficult to evaluate its effect. Fast logistics, good service.	JD.com
Home Life Equipment	Cell Phone Battery Adapter	This user feels the product is better	JD.com
Home Life Equipment	Cell Phone Battery Adapter	Just got the shipment, thanks!	JD.com
Home Life Equipment	Cell Phone Battery Adapter	Goods good quality and excellent service and fast logistics is genuine cell phone repairer.	JD.com
Home Life Equipment	Cell Phone Battery Adapter	This user feels the product is better	JD.com
Home Life Equipment	Cell Phone Battery Adapter	Delivered quickly the first time to use do not know the effect but the standard is genuine for the elderly to speak very good often renewable power does not cost the battery!	JD.com
Home Life Equipment	Cell Phone Battery Adapter	Testimonials	JD.com
Home Life Equipment	Cell Phone Battery Adapter	This product is ok, running speed is ok, easy to operate, I am very satisfied, so 5 stars!	JD.com
Home Life Equipment	Cell Phone Battery Adapter	Not used. Don't know how it turned out. Not good to comment.	JD.com
Home Life Equipment	Cell Phone Battery Adapter	This user feels the product is better	JD.com
Home Life Equipment	Cell Phone Battery Adapter	This user feels the product is better	JD.com
Home Life Equipment	Cell Phone Battery Adapter	Not applicable! Returnable?	JD.com
Home Life Equipment	Cell Phone Battery Adapter	Haven't used it yet!	JD.com
Home Life Equipment	Cell Phone Battery Adapter	very happy	JD.com
Home Life Equipment	Cell Phone Battery Adapter	This user feels the product is better	JD.com
Home Life Equipment	Cell Phone Battery Adapter	(of an unmarried couple) be close	JD.com
Home Life Equipment	Cell Phone Battery Adapter	serviceable	JD.com
Home Life Equipment	Cell Phone Battery Adapter	The charger is compact and easy to use, with voice prompts and introductory features such as garbage removal, which can improve battery performance	JD.com
Home Life Equipment	Cell Phone Battery Adapter	This user feels the product is better	JD.com
Home Life Equipment	Cell Phone Battery Adapter	It's nice and easy to use.	JD.com
Home Life Equipment	Cell Phone Battery Adapter	Very good.	JD.com

Home Life Equipment	Cell Phone Battery	Poor express delivery	JD.com
Home Life Equipment	Cell Phone Battery	Goods have been received logistics very fast packaging is very good	JD.com
Home Life Equipment	Cell Phone Battery Adapter	Received, indeed very good, get back can not wait to try, the original phone can not open the phone, and now can open, very good, thank you!	JD.com
Home Life Equipment	Cell Phone Battery	Very good Thanks to the owner ,2thanks to the courier .	JD.com
Home Life Equipment	Cell Phone Battery	Very satisfied with a shopping logistics is also very fast	JD.com
Home Life Equipment	Cell Phone Battery	appliances with lithium batteries at home can be brought back from the dead!	JD.com
Home Life Equipment	Cell Phone Battery	Works well, only used it once felt good, will buy another one if needed.	JD.com
Home Life Equipment	Cell Phone Battery	The goods have been received, the logistics is also very fast, the stuff is good, the next time to come back to visit.	JD.com
Home Life Equipment	Cell Phone Battery	This user feels the product is better	JD.com
Home Life Equipment	Cell Phone Battery	Good value for money. Good service from the courier guy.	JD.com
Home Life Equipment	Cell Phone Battery	This user feels the product is better	JD.com
Home Life Equipment	Cell Phone Battery	It's faster to charge with this fixer.	JD.com
Home Life Equipment	Cell Phone Battery	This user feels the product is better	JD.com
Home Life Equipment	Cell Phone Battery Adapter	appearance of all normal. Short-term use has not seen obvious results.(The cell phonestill sometimes hot and lagging when using, I hope that soon there will be an improvement in the situation.	JD.com
Home Life Equipment	Cell Phone Battery	Good value for money, well worth the money!	JD.com
Home Life Equipment	Cell Phone Battery	Baby's fine. Looks like it's working.	JD.com
Home Life Equipment	Cell Phone Battery	The thing has been received, charged once, do not know how the effect, looking forward to.	JD.com
Home Life Equipment	Cell Phone Battery	This user feels the product is better	JD.com
Home Life Equipment	Cell Phone Battery	Goods to receive the logistics is also very fast, first give five stars praise, and so use2days later to add comments.	JD.com
Home Life Equipment	Cell Phone Battery	It feels like it's working.	JD.com
Home Life Equipment	Cell Phone Battery	It looks good.	JD.com
Home Life Equipment	Cell Phone Battery	This user feels the product is better	JD.com
Home Life Equipment	Cell Phone Battery	Fast logistics, good service, easy to use, will repurchase and recommend to friends to buy!	JD.com
Home Life Equipment	Cell Phone Battery	I don't know if it's good or not yet, but I'm using it, so I'll give it a good review first.	JD.com
Home Life Equipment	Cell Phone Battery	Works great, worth the money	JD.com
Home Life Equipment	Cell Phone Battery	This user feels the product is better	JD.com
Home Life Equipment	Battery Adapter	This user feels the product is better	JD.com
Home Life Equipment	Battery Adapter	Received a good one to use right away.	JD.com
Home Life Equipment	Battery Adapter	Received, satisfied with the excellent after the review	JD.com
Home Life Equipment	Battery Adapter	Thank you.	JD.com
Home Life Equipment	Battery Adapter	It feels okay, well worth the money.	JD.com
Home Life Equipment	Battery Adapter	This user feels the product is better	JD.com
Home Life Equipment	Battery Adapter	evaluation after use	JD.com
Home Life Equipment	Cell Phone Battery Adapter	first time to use this kind of equipment how the effect is yet to be verified but looks okay and also voice intelligent broadcast look forward to it to bring my surprise	JD.com
Home Life Equipment	Battery Adapter	This user feels the product is better	JD.com
Home Life Equipment	Battery Adapter	The stuff is good, use it and hope for good results	JD.com
Home Life Equipment	Battery Adapter	This user feels the product is better	JD.com

Home Life Equipment	Battery Adapter	ve used it a couple times and it doesn't feel like it.	JD.com
Home Life Equipment	Cell Phone Battery Adapter	The description matches. Used it as soon as I received it and my phone really doesn't get hot.Everyone says it's great! Friends can go shoot it! Customer service 🙎‍♂️service attitude is very good👍Kudos	JD.com
Home Life Equipment	Battery Adapter	This user feels the product is better	JD.com
Home Life Equipment	Battery Adapter	pretty good	JD.com
Home Life Equipment	Cell Phone Battery Adapter	Cell phone repairer received, it rushed to try, but also with voice it, quite convenient, the period of time to get the effect of the future, the first five-star praise it.	JD.com
Home Life Equipment	Battery Adapter	role, no matter how long it has been charged, the phone will not be hot.	JD.com
Home Life Equipment	Battery Adapter	This user feels the product is better	JD.com
Home Life Equipment	Battery Adapter	I don't feel it. It's working.	JD.com
Home Life Equipment	Battery Adapter	This user feels the product is better	JD.com
Home Life Equipment	Battery Adapter	(of an unmarried couple) be close	JD.com
Home Life Equipment	Battery Adapter	no good	JD.com
Home Life Equipment	Cell Phone Battery Adapter	This cell phone battery repairer is quite good, can be used as a charger, but also can repair the battery, there are voice prompts, logistics is very fast Oh.	JD.com
Home Life Equipment	Battery Adapter	Repair can machine battery effect is good, make the cell phone battery life long, good value for money	JD.com
Shelf open outdoor equipment franchise	Battery Reconditioner	not have	JD.com
Xiao Hai Home Dept.	Air Conditioner Intelligent Inverter	Products off the shelves	JD.com
XINSHUHEIMA	Air Conditioner Intelligent Inverter	The store's gone.	JD.com
Scientific Research Beauty & Personal Care	Air Conditioner Intelligent Inverter	The store's gone.	JD.com
Geek Goodies	Electric Vehicle Repair	Charge quickly, beautifully made, affordable, durable battery, beautiful appearance of the product	JD.com
Geek Goodies	Electric Vehicle Repair	Got it back and used it, plugged in charging voltage is quite stable, or effect, long time use effect will be better	JD.com
Geek Goodies	Electric Vehicle Repair	Thank you for the business and logistics services, after-sales very good and guaranteed, the product is really good!	JD.com
Geek Goodies	Electric Vehicle Repair	Business description is very detailed, and the logistics is very fast, recommended to buy!	JD.com
Geek Goodies	Electric Vehicle Repair	Compact and space-saving, it can charge and repair batteries, the best of both worlds!	JD.com
Geek Goodies	Electric Vehicle Repair	Great quality and great results, worth buying!	JD.com
Geek Goodies	Electric Vehicle Repair	The customer service is very good, there are questions and answers, the key product effect is really good!	JD.com
Geek Goodies	Electric Vehicle Repair	Good experience, full of automatic power off, also with repair	JD.com
Geek Goodies	Electric Vehicle Repair	Well packaged, very easy to use, fast charging speeds	JD.com
Geek Goodies	Electric Vehicle Repair	Cost-effective, very effective for old battery reconditioning	JD.com
Geek Goodies	Electric Vehicle Repair	Goods have been received, good quality, satisfied	JD.com
Geek Goodies	Electric Vehicle Repair	Received the item, very happy with it, love it, the product works great!	JD.com
Geek Goodies	Electric Vehicle Repair	It works well, and you can save a lot of money with the product!	JD.com
Geek Goodies	Electric Vehicle Repair	Great customer service and trustworthy	JD.com
Geek Goodies	Electric Vehicle Repair	Repairs batteries very well	JD.com
Geek Goodies	Electric Vehicle Repair	It works well and is quite convenient	JD.com

Geek Goodies	Electric Vehicle Repair	Affordable price, fast charging, easy to operate	JD.com
Geek Goodies	Electric Vehicle Repair	After using the repairer the battery lasts a lot longer, good and recommendable!	JD.com
Geek Goodies	Electric Vehicle Repair Magic	received, the appearance is very beautiful, with characteristics, and charging repair effect is very good, good quality, and have a warranty, thank you for the business service, praise praise praise!	JD.com
Geek Goodies	Electric Vehicle Repair	Battery repair is very good, before full can not use for a long time, now a full charge can be used for a long time!	JD.com
Geek Goodies	Electric Vehicle Repair	Pulse received, fast charging, works great!	JD.com
Geek Goodies	Electric Vehicle Repair	A few years old batteries do get a good boost with the reconditioner	JD.com
Geek Goodies	Electric Vehicle Repair	Charge repair battery, used it a few times, works pretty well	JD.com
Geek Goodies	Electric Vehicle Repair Magic	Specially used a few days to evaluate, the effect can indeed be used for a few days, the battery is indeed a little stronger, I hope that the time is long with better results, after all, to replace a car but also a few thousand	JD.com
Geek Goodies	Electric Vehicle Repair Magic	Electric car repairer received in the afternoon, well-packed, open the package immediately began to use, looks good quality, and charging effect is also good, repair should not be bad, useful will buy again to send someone!	JD.com
Geek Goodies	Electric Vehicle Repair	My friend has been using it and said it works well, so I bought it to try.	JD.com
Geek Goodies	Electric Vehicle Repair	Charging is fast and economical, and the product works well and satisfactorily	JD.com
Geek Goodies	Electric Vehicle Repair	Received the goods, electric car repairer appearance is very beautiful, logistics is also very fast	JD.com
Geek Goodies	Electric Vehicle Repair	Fast logistics, good quality, charging automatic power off	JD.com
Geek Goodies	Electric Vehicle Repair	Charging is fast, if you want to buy it, you can feel free to get it!	JD.com
Geek Goodies	Electric Vehicle Repair	Goods can, logistics is fast, back to use immediately!	JD.com
Geek Goodies	Electric Vehicle Repair Magic	Used more than half a year, because they accidentally lost, with the business actually gave me a lot of discounts, and then bought a, really thank the business service after sale!	JD.com
Geek Goodies	Electric Vehicle Repair	used for a period of time, the effect is good and simple and convenient!	JD.com
Geek Goodies	Electric Vehicle Repair	I've been using it for a long time and the battery is basically as good as new, I truly recommend it!	JD.com
Geek Goodies	Electric Vehicle Repair	drive it for a day and a half after I use the restorer, it's really good!	JD.com
Geek Goodies	Electric Vehicle Repair Magic	And can charge and can repair the battery, it is indeed very convenient, the follow-up to see how the effect, customer service guarantee said very heartwarming!	JD.com
Geek Goodies	Electric Vehicle Repair	I've used it twice already and it feels fine	JD.com
Geek Goodies	Electric Vehicle Repair	Repairable batteries, complete repair during charging, very convenient	JD.com
Geek Goodies	Electric Vehicle Repair	Repairer received, the quality is very good, very use recommended to buy!	JD.com
Geek Goodies	Electric Vehicle Repair	Good product, can repair on the battery	JD.com
Geek Goodies	Electric Vehicle Repair	Used it once, felt it worked	JD.com
Geek Goodies	Electric Vehicle Repair	Very good, safe and effective, the effect is obvious, logistics is very fast	JD.com
Geek Goodies	Electric Vehicle Repair	Very good quality, very satisfied with the service and answered all questions	JD.com
Geek Goodies	Electric Vehicle Repair Magic	It just happened that the family electric car battery used a long time is not very durable, buy the repair of the god after the battery does not have to change, save a lot of money!	JD.com
Geek Goodies	Electric Vehicle Repair	Goods received, well-packed, logistics is very fast, the boss's attitude is also very good!	JD.com
Geek Goodies	Electric Vehicle Repair	Battery Repair Magic is well-designed, fine workmanship, tightly packaged, very good	JD.com
Geek Goodies	Electric Vehicle Repair	It's an upgrade to battery reconditioning, so you can just close your eyes and enter the	JD.com
Geek Goodies	Electric Vehicle Repair	Received, thanks to the boss, the effect of using a few days immediately a lot better, really good Oh!	JD.com
Geek Goodies	Electric Vehicle Repair	The restorer has been received and is in use	JD.com
Geek Goodies	Electric Vehicle Repair	Fully automatic trolley repairer received, feel the effect is quite good	JD.com
Geek Goodies	Electric Vehicle Repair	After using it to evaluate, the quality is very good, effective, you can rest assured that the punch	JD.com

Geek Goodies	Electric Vehicle Repair Magic	Received the goods, as described by the merchant, quite well	JD.com
Geek Goodies	Electric Vehicle Repair Magic	Very good, very easy to use	JD.com
Geek Goodies	Electric Vehicle Repair Magic	Battery Repairer received, logistics is very fast, the quality is also very good, used a few days, it is indeed very conscientious, very recommended!	JD.com
Geek Goodies	Electric Vehicle Repair Magic	convenient, the key is that the effect is very good, it is really worth buying!	JD.com
Geek Goodies	Electric Vehicle Repair Magic	fine workmanship; store service patience and meticulous, ask a hundred questions; express delivery quickly; courier is serious and responsible; packaging is simple and strict, the goods are intact.	JD.com
Geek Goodies	Electric Vehicle Repair Magic	Repair is very good, and customer service is very patient answer, good luck with business!	JD.com
Geek Goodies	Electric Vehicle Repair Magic	I used it a few times for a friend and asked me to buy it for him, and now I've bought it for so many people, it's a really good product!	JD.com
Geek Goodies	Electric Vehicle Repair Magic	Logistics is very fast, the product appearance is very good, and the operation is simple and effective!	JD.com
Geek Goodies	Electric Vehicle Repair Magic	Friends recommended the purchase, at first not quite believe, used for a period of time after the real effect, which came to the evaluation	JD.com
Geek Goodies	Electric Vehicle Repair Magic	Good product, recommendable, very good results and good service!	JD.com
Geek Goodies	Electric Vehicle Repair Magic	Shipping is very fast, the baby received after the trial, indeed can effectively repair the old battery, with a long time, it is worth buying!	JD.com
Geek Goodies	Electric Vehicle Repair Magic	Battery repairer use really have repaired, power to improve a little, the effect is good!	JD.com
Geek Goodies	Electric Vehicle Repair Magic	Exquisite workmanship, good quality, the effect is very good, the key is affordable, the purchase is well worth it!	JD.com
Geek Goodies	Electric Vehicle Repair Magic	The effect is really good, the logistics is also very fast, a few days to arrive	JD.com
Geek Goodies	Electric Vehicle Repair Magic	This multi-functional battery repair device effect is really good, used for a while obviously a lot of battery durability!	JD.com
Geek Goodies	Electric Vehicle Repair Magic	Neighbor recommended to buy, used for a long time, the battery is basically the same as a new battery, truly recommended!	JD.com
Geek Goodies	Electric Vehicle Repair Magic	Fast logistics, good product, functionality	JD.com
Geek Goodies	Electric Vehicle Repair Magic	I've been using it for a while and the results have really improved and the after-sales service is very good	JD.com
Geek Goodies	Electric Vehicle Repair Magic	The customer service guide is very patient and also has an after-sales guarantee, so it should be good	JD.com
Geek Goodies	Electric Vehicle Repair Magic	The battery has been used for a long time, quickly run out of power, after using the restorer can be opened for a long time, not afraid of not durable	JD.com
Geek Goodies	Electric Vehicle Repair Magic	After I bought it and used it once, I didn't see any results, by the time the car went out, or something?	JD.com
Geek Goodies	Electric Vehicle Repair Magic	Received the goods today can not wait to take out the charge, looks good, the logistics is very fast!	JD.com
Geek Goodies	Electric Vehicle Repair Magic	Not yet used, looking small and exquisite, logistics is too slow	JD.com
Geek Goodies	Electric Vehicle Repair Magic	Electric car repair charger has received logistics is also very fast price affordable.	JD.com
Geek Goodies	Electric Vehicle Repair Magic	an excellent	JD.com
Geek Goodies	Electric Vehicle Repair Magic	It works well. It's genuine.	JD.com
Geek Goodies	Electric Vehicle Repair Magic	Good workmanship, easy to use, clear instructions, use and then evaluate	JD.com
Geek Goodies	Electric Vehicle Repair Magic	This user feels the product is better	JD.com
Geek Goodies	Electric Vehicle Repair Magic	Convenient, to be used to experience the effect	JD.com
Geek Goodies	Electric Vehicle Repair Magic	Fast shipping,3 to7weeks to review	JD.com
Geek Goodies	Electric Vehicle Repair Magic	This user feels the product is better	JD.com
Geek Goodies	Electric Vehicle Repair Magic	The fake can't be plugged in it's all plastic inside and has no seat bottom. How do you energize it?	JD.com
Geek Goodies	Electric Vehicle Repair Magic	Very poor quality, can't cut off the power automatically, blowing fiercely	JD.com
Geek Goodies	Electric Vehicle Repair Magic	This user feels the product is better	JD.com
Geek Goodies	Electric Vehicle Repair Magic	Charges quickly.	JD.com
Geek Goodies	Electric Vehicle Repair Magic	Received, has been used for two days, looks good, to be used for a period of time and then come back to the evaluation.	JD.com

Geek Goodies	Electric Vehicle Repair Magic	This user feels the product is better	JD.com
Geek Goodies	Electric Vehicle Repair Magic	This user feels the product is better	JD.com
Geek Goodies	Electric Vehicle Repair Magic	I don't know how it is, but I'll comment later. I'll give it 5 stars first.	JD.com
Geek Goodies	Electric Vehicle Repair Magic	an excellent	JD.com
Geek Goodies	Electric Vehicle Repair Magic	The stuff is great and worth having	JD.com
Geek Goodies	Electric Vehicle Repair Magic	Received the goods verification code2535 8203 0167 3198check authenticity	JD.com
Geek Goodies	Electric Vehicle Repair Magic	Goods received logistics is also fast not yet used do not know whether it is the same as the description of the situation first to give a favorable comment on it	JD.com
Geek Goodies	Electric Vehicle Repair Magic	This item is not self-powered.	JD.com
Geek Goodies	Electric Vehicle Repair Magic	Item just received as described by the manufacturer	JD.com
Geek Goodies	Electric Vehicle Repair Magic	Received the product and it works great, great service, will buy again in the future!	JD.com
Geek Goodies	Electric Vehicle Repair Magic	This user feels the product is better	JD.com
Geek Goodies	Electric Vehicle Repair Magic	I'll comment after I've had some success with it.	JD.com
Geek Goodies	Electric Vehicle Repair Magic	The goods have been received, well-packed, fast logistics satisfied!	JD.com
Geek Goodies	Electric Vehicle Repair Magic	Received the restorer, not sure how well it works? Let's use it first.	JD.com
Geek Goodies	Electric Vehicle Repair Magic	This user feels the product is better	JD.com
Geek Goodies	Electric Vehicle Repair Magic	This user feels the product is better	JD.com
Geek Goodies	Electric Vehicle Repair Magic	Expecting good results and giving good reviews!	JD.com
Geek Goodies	Electric Vehicle Repair Magic	The goods have just been received and are as described. Logistics is also very fast. Awesome!!!	JD.com
Geek Goodies	Electric Vehicle Repair Magic	Just received.	JD.com
Geek Goodies	Electric Vehicle Repair Magic	Review in a couple weeks.	JD.com
Geek Goodies	Electric Vehicle Repair Magic	Very satisfied with the five-star review	JD.com
Geek Goodies	Electric Vehicle Repair Magic	Let's use it and see.	JD.com
Geek Goodies	Electric Vehicle Repair Magic	The service is very good, the customer service answer is also very detailed, according to customer service said the use of a period of time after the effect is really much better!	JD.com
Geek Goodies	Electric Vehicle Repair Magic	After using the repairer the battery lasts a lot longer, good and recommendable!	JD.com
Geek Goodies	Electric Vehicle Repair Magic	convenient, the key is that the effect is very good, it is really worth buying!	JD.com
Geek Goodies	Electric Vehicle Repair Magic	I've been using it for a while and the results have really improved and the after-sales service is very good	JD.com
Geek Goodies	Electric Vehicle Repair Magic	Just received the use of the feeling of a slight enhancement, the product design is still quite simple, very easy to operate	JD.com
Geek Goodies	Electric Vehicle Repair Magic	The customer service is very good, there are questions and answers, the key product effect is really good!	JD.com
Geek Goodies	Electric Vehicle Repair Magic	The store is very enthusiastic to reply to my questions, the shopkeeper's service attitude is really OK, the shipment is very fast!	JD.com
Geek Goodies	Electric Vehicle Repair Magic	Love it, very tightly packaged and affordable.	JD.com
Geek Goodies	Electric Vehicle Repair Magic	No problem, I finally got what I wanted. Thanks to the seller and it's also very ok	JD.com
Geek Goodies	Electric Vehicle Repair Magic	Charging function: very good. Product appearance: beautiful and generous. Logistics speed: very fast not dragging	JD.com
Geek Goodies	Electric Vehicle Repair Magic	Great quality and great results, worth buying!	JD.com
Geek Goodies	Electric Vehicle Repair Magic	Well worth the money, affordable and must be praised	JD.com
Geek Goodies	Electric Vehicle Repair Magic	It works well, and you can save a lot of money with the product!	JD.com
Geek Goodies	Electric Vehicle Repair Magic	Logistics is fast. Affordable, love it. Properly give you praise, with this price can buy such, affordable. Love it. Praise, praise, praise.	JD.com
Geek Goodies	Electric Vehicle Repair Magic	And can charge and can repair the battery, it is indeed very convenient, the follow-up to see how the effect, customer service guarantee said very heartwarming!	JD.com

Geek Goodies	Electric Vehicle Repair Magic	Recommended purchase, awesome, great seller service!	JD.com
Geek Goodies	Electric Vehicle Repair Magic	A few years old batteries with the product reconditioning really have a very good improvement, really good!	JD.com
Geek Goodies	Electric Vehicle Repair Magic	Cost-effective Oh very much like the future will continue to patronize, buy a very can be a baby, something line	JD.com
Geek Goodies	Electric Vehicle Repair Magic	Used more than half a year, because they accidentally lost, with the business actually gave me a lot of discounts, and then bought a, really thank the business service after sale!	JD.com
Geek Goodies	Electric Vehicle Repair Magic	Battery repair is very good, before full can not use for a long time, now a full charge can be used for a long time!	JD.com
Geek Goodies	Electric Vehicle Repair Magic	The battery has been used for a long time, quickly run out of power, after using the restorer can be opened for a long time, not afraid of not durable	JD.com
Geek Goodies	Electric Vehicle Repair Magic	It's easy to use, genuine, effective and guaranteed!	JD.com
Geek Goodies	Electric Vehicle Repair Magic	Great value, just like the picture, and it's nice and really affordable!	JD.com
Geek Goodies	Electric Vehicle Repair Magic	commendable, inadvertently came to you to buy the heart of the baby!	JD.com
Geek Goodies	Electric Vehicle Repair Magic	The packaging is intact, the product was received and started to use, the charging effect is also very good, there is a slight boost, customer service said to use for a while longer, but also guaranteed!	JD.com
Geek Goodies	Electric Vehicle Repair Magic	The customer service guide is very patient and also has an after-sales guarantee, so it should be good	JD.com
Geek Goodies	Electric Vehicle Repair Magic	It's good. All in all, it's okay. It's good quality.	JD.com
Geek Goodies	Electric Vehicle Repair Magic	It's even better than expected. The price was fair and the shipping was pretty fast kudos, love it!	JD.com
Geek Goodies	Electric Vehicle Repair Magic	Quality is fine, baby received, fast shipping!	JD.com
Geek Goodies	Electric Vehicle Repair Magic	Worth buying, good seller service, very unexpected	JD.com
Geek Goodies	Electric Vehicle Repair Magic	Affordable, very fast very fast very fast thank you for the opportunity to work together again, overall a good buy at this price!	JD.com
Geek Goodies	Electric Vehicle Repair Magic	Friends recommended the purchase, at first not quite believe, used for a period of time after the real effect, which came to the evaluation	JD.com
Geek Goodies	Electric Vehicle Repair Magic	All in all great praise, good customer service, very patient store owner arrived in a timely manner, can, never a TO!	JD.com
Geek Goodies	Electric Vehicle Repair Magic	Good product, recommendable, very good results and good service!	JD.com
Geek Goodies	Electric Vehicle Repair Magic	Shipping was fast. The quality of the item is also quite okay. Love it so much so also, recommend it!	JD.com
Geek Goodies	Electric Vehicle Repair Magic	Can, line, packaging with care. Express delivery is also quite OK.	JD.com
Geek Goodies	Electric Vehicle Repair Magic	Personally, I think it's worth it and I like it, the quality of the goods is good but the price is low, affordable!	JD.com
Geek Goodies	Electric Vehicle Repair Magic	The seller shipped quickly, it is the kind I want Thank you very happy shopping, affordable Oh!	JD.com
Geek Goodies	Electric Vehicle Repair Magic	Express very can seller packaged very can service attitude very can also quite can, is very like.	JD.com
Geek Goodies	Electric Vehicle Repair Magic	I used it a few times for a friend and asked me to buy it for him, and now I've bought it for so many people, it's a really good product!	JD.com
Geek Goodies	Electric Vehicle Repair Magic	Exquisite workmanship, good quality, the effect is very good, the key is affordable, the purchase is well worth it!	JD.com
Geek Goodies	Electric Vehicle Repair Magic	The quality is fine, I had bought it with the intention of trying it out and it was very affordable. Overall very like	JD.com
Geek Goodies	Electric Vehicle Repair Magic	drive it for a day and a half after I use the restorer, it's really good!	JD.com
Geek Goodies	Electric Vehicle Repair Magic	The battery is three years old and performs great, it's just that the battery isn't working. The original can use two days, now less than a day. I saw this product from the Internet, the response is good, buy to try, the effect is really good!	JD.com
Geek Goodies	Electric Vehicle Repair Magic	It's fine, overall it's a good buy for the price.	JD.com
Geek Goodies	Electric Vehicle Repair Magic	Battery Repairer received, logistics is very fast, the quality is also very good, used a few days, it is indeed very conscientious, very recommended!	JD.com
Geek Goodies	Electric Vehicle Repair Magic	Received the goods, electric car repairer appearance is very beautiful, logistics is also very fast	JD.com
Geek Goodies	Electric Vehicle Repair Magic	Charging is fast and economical, and the product works well and satisfactorily	JD.com
Geek Goodies	Electric Vehicle Repair Magic	Shipping is very fast, the baby received after the trial, indeed can effectively repair the old battery, with a long time, it is worth buying!	JD.com

Geek Goodies	Electric Vehicle Repair	The shipping speed is very fast, the quality of the line of oh, are very affordable, very praise.	JD.com
Geek Goodies	Electric Vehicle Repair	The effect is really good, the logistics is also very fast, a few days to arrive	JD.com
Geek Goodies	Electric Vehicle Repair	I like it very much. Thank you boss, logistics can be fast ah, value for money, thank you!	JD.com
Geek Goodies	Electric Vehicle Repair	Logistics is very fast, the product appearance is very good, and the operation is simple and effective!	JD.com
Geek Goodies	Electric Vehicle Repair Magic	Things received product appearance cool multi-device compatible, especially after powering up is very beautiful description consistent quality, the use of simple effect is good, the battery repair is improved, it is worth having, I wish the boss a prosperous business!	JD.com
Geek Goodies	Electric Vehicle Repair	Baby received, affordable, cheap	JD.com
Geek Goodies	Electric Vehicle Repair	The line is as described, a pleasant shopping, can comment	JD.com
Geek Goodies	Electric Vehicle Repair	It's good. It's all right.	JD.com
Geek Goodies	Electric Vehicle Repair	Totally out of expectation, want to buy, love it!	JD.com
Geek Goodies	Electric Vehicle Repair	Very good quality, very satisfied with the service and answered all questions	JD.com
Geek Goodies	Electric Vehicle Repair	Neighbor recommended to buy, used for a long time, the battery is basically the same as a new battery, truly recommended!	JD.com
Geek Goodies	Electric Vehicle Repair	Logistics is very fast, service attitude level can be, praise!	JD.com
Geek Goodies	Electric Vehicle Repair	Business description is very detailed, and the logistics is very fast, recommended to buy!	JD.com
Geek Goodies	Electric Vehicle Repair Magic	Electric car battery repairer received a very convenient and quick to use the effect is very satisfied with the battery before you can open four hours now can be used for six hours very surprised very good-looking!	JD.com
Geek Goodies	Electric Vehicle Repair	The price is very affordable, the logistics is very fast, I will buy it again next time, the quality is as I imagined	JD.com
Geek Goodies	Electric Vehicle Repair Magic	I filled in the wrong address, customer service patience to deal with, quickly received, the appearance is very good, electric car repair god is really very good!	JD.com
Geek Goodies	Electric Vehicle Repair Magic	fine workmanship; store service patience and meticulous, ask a hundred questions; express delivery quickly; courier is serious and responsible; packaging is simple and strict, the goods are intact.	JD.com
Geek Goodies	Electric Vehicle Repair	Repairer received and used for a few days the effect has increased, use it for a while to see if it will be better	JD.com
Geek Goodies	Electric Vehicle Repair	Very useful a shopping, will often visit this store in the future, there is a need to come back to the store to buy!	JD.com
Geek Goodies	Electric Vehicle Repair Magic	Electric car repairer received in the afternoon, well-packed, open the package immediately began to use, looks good quality, and charging effect is also good, repair should not be bad, useful will buy again to send someone!	JD.com
Geek Goodies	Electric Vehicle Repair	The stuff works. Trustworthy.	JD.com
Geek Goodies	Electric Vehicle Repair	Cost-effective, fast logistics, praise the merchant, the	JD.com
Geek Goodies	Electric Vehicle Repair	Thank you for the business and logistics services, after-sales very good and guaranteed, the product is really good!	JD.com
Geek Goodies	Electric Vehicle Repair	Good value, excellent results, easy to use and lots of features!	JD.com
Geek Goodies	Electric Vehicle Repair	Fast logistics will buy again next time, can buy such a good price is really earned!	JD.com
Geek Goodies	Electric Vehicle Repair	will be resolved in a timely manner, like friends do not hesitate!	JD.com
Geek Goodies	Electric Vehicle Repair	It's worth buying for a good price, so if you like it, do it now!	JD.com
Geek Goodies	Electric Vehicle Repair	Very awesome, packed with care. Express delivery is also quite OK and fast. Next time will come again.	JD.com
Geek Goodies	Electric Vehicle Repair	Repair is very good, and customer service is very patient answer, good luck with business!	JD.com
Geek Goodies	Electric Vehicle Repair	I finally got what I wanted. Thanks to the seller, who has good service and very god speed delivery.	JD.com
Geek Goodies	Electric Vehicle Repair	The price is very affordable, and the quality is quite acceptable praise bar, all very affordable, very praise, the	JD.com
Geek Goodies	Electric Vehicle Repair	Shipping was quick. Merchandise is also quite okay. Love it so much, it's really nice	JD.com
Geek Goodies	Electric Vehicle Repair	This multi-functional battery repair device effect is really good, used for a while obviously a lot of battery durability!	JD.com
Geek Goodies	Electric Vehicle Repair	Received, thanks to the boss, the effect of using a few days immediately a lot better, really good Oh!	JD.com

Geek Goodies	Electric Vehicle Repair Magic	A battery is a few hundred dollars, with the electric car restorer can save a lot, especially affordable and worth recommending the use of people around!	JD.com
Geek Goodies	Electric Vehicle Repair	Very good quality and affordable	JD.com
Geek Goodies	Electric Vehicle Repair	repurchase discount, recommended to relatives have bought, all praise good!	JD.com
Geek Goodies	Electric Vehicle Repair Magic	received, the appearance is very beautiful, with characteristics, and charging repair effect is very good, good quality, and have a warranty, thank you for the business service, praise praise praise!	JD.com
Geek Goodies	Electric Vehicle Repair	All in all, it's great and affordable.	JD.com
Geek Goodies	Electric Vehicle Repair	used for a period of time, the effect is good and simple and convenient!	JD.com
Tekla Intelligence	Battery Reconditioner	Offer a cell phone repair device to offer, and the same as described, plug in the charging repair normal work!	JD.com
Tekla Intelligence	Battery Reconditioner	To the description is completely different, after using the phone is still hot, not heat dissipation. Feel duped to publicity all do not pay, period of fraud consumers.	JD.com
Tekla Intelligence	Battery Reconditioner	This user feels the product is better	JD.com
Tekla Intelligence	Battery Reconditioner	(of an unmarried couple) be close	JD.com
Tekla Intelligence	Battery Reconditioner	Easy to charge, old cell phone charged and fully charged works much better!	JD.com
Tekla Intelligence	Battery Reconditioner	The item is cheap, good quality, fast logistics, good service attitude, worth having and recommending, next time to buy!	JD.com
Tekla Intelligence	Battery Reconditioner	This user feels the product is better	JD.com
Tekla Intelligence	Battery Reconditioner	This user feels the product is better	JD.com
Tekla Intelligence	Battery Reconditioner	[Smiles]	JD.com
Tekla Intelligence	Battery Reconditioner	Cell phone repairer what day received, has been used to charge very quickly, today tried to have the effect, good quality, small and exquisite. Easy to use very satisfied.	JD.com
Tekla Intelligence	Battery Reconditioner	This user feels the product is better	JD.com
Tekla Intelligence	Battery Reconditioner	This user feels the product is better	JD.com
Tekla Intelligence	Battery Reconditioner	This user feels the product is better	JD.com
Tekla Intelligence	Battery Reconditioner	This user feels the product is better	JD.com
Tekla Intelligence	Battery Reconditioner	CHARGING: Charging is fast, when fast charging is possible, but still does not extend the battery life.	JD.com
Tekla Intelligence	Battery Reconditioner	Good to use, really can repair the battery, a cell phone battery does not work, charging especially hot, can not boot, after repair, charging is not hot, but also can boot, good!	JD.com
Tekla Intelligence	Battery Reconditioner	I don't know if it's working.	JD.com
Tekla Intelligence	Battery Reconditioner	I haven't used it yet, I'll follow up after using it.	JD.com
Tekla Intelligence	Battery Reconditioner	Cell phone battery🔋repairer received, this is the second time to buy it, repair the battery is still good👍. Give merchants five-star praise!	JD.com
Tekla Intelligence	Battery Reconditioner	I just got it and I'm using it, let's see how it works.	JD.com
Tekla Intelligence	Battery Reconditioner	Inconsistent with the publicity, used did not feel the effect, the business explained to be5a8weeks to be effective, hahaha! Purchase with caution.	JD.com
Tekla Intelligence	Battery Reconditioner	It's really good, works well, and is worth the money.	JD.com
Tekla Intelligence	Battery Reconditioner	This user feels the product is better	JD.com
Tekla Intelligence	Battery Reconditioner	This user feels the product is better	JD.com
Tekla Intelligence	Battery Reconditioner	Cell phone battery repairer received, logistics is very fast, first give five stars. Expect to be able to repair the battery, and then follow up.	JD.com
Tekla Intelligence	Battery Reconditioner	Cell phone fixer, well made, technologically advanced, with clear instructions for use, at the right price.	JD.com
Tekla Intelligence	Battery Reconditioner	Product packaging is good, small and exquisite, new style, easy to carry, looking forward to the effect, repurchase.	JD.com
Tekla Intelligence	Battery Reconditioner	Just received the goods, wait for a period of time to use again cheap	JD.com
Tekla Intelligence	Battery Reconditioner	This user feels the product is better	JD.com

Tekla Intelligence	Battery Reconditioner	Satisfaction Shopping	JD.com
Tekla Intelligence	Battery Reconditioner	Satisfaction.	JD.com
Tekla Intelligence	Battery Reconditioner	The baby was received and the logistics was fast.	JD.com
Tekla Intelligence	Battery Reconditioner	This user feels the product is better	JD.com
Tekla Intelligence	Battery Reconditioner	favorable criticism	JD.com
Tekla Intelligence	Battery Reconditioner	The product works well, the charge retouching was quick and finished in about three hours, which is great.	JD.com
Tekla Intelligence	Battery Reconditioner	This user feels the product is better	JD.com
Tekla Intelligence	Battery Reconditioner	This user feels the product is better	JD.com
Tekla Intelligence	Battery Reconditioner	This user feels the product is better	JD.com
Tekla Intelligence	Battery Reconditioner	Cell phone repairer received, with a period of time such as the battery can play a role in repairing the words will repurchase. First give merchants five-star praise!	JD.com
Tekla Intelligence	Battery Reconditioner	Baby received very satisfied thank you boss 5	JD.com
Tekla Intelligence	Battery Reconditioner	(of an unmarried couple) be close	JD.com
Tekla Intelligence	Battery Reconditioner	so that	JD.com
Tekla Intelligence	Battery Reconditioner	I have seen the effect of visible repair of the cell phone, and the logistics are fast!	JD.com
Tekla Intelligence	Battery Reconditioner	In use- Not sure when it will work/. It's working. will comment again.	JD.com
Tekla Intelligence	Battery Reconditioner	Haven't found it useful so far, waiting for a follow up review.	JD.com
Tekla Intelligence	Battery Reconditioner	classifier for objects in rows such as words	JD.com
Tekla Intelligence	Battery Reconditioner	general	JD.com
Tekla Intelligence	Battery Reconditioner	It doesn't help at all!!! Pitbulls.	JD.com
Tekla Intelligence	Battery Reconditioner	It feels okay.	JD.com
Tekla Intelligence	Battery Reconditioner	This user feels the product is better	JD.com
Tekla Intelligence	Battery Reconditioner	Feels good, high utility value and good value for money! Use it for a while and see!	JD.com
Tekla Intelligence	Battery Reconditioner	s	JD.com
Tekla Intelligence	Battery Reconditioner	in use	JD.com
Tekla Intelligence	Battery Reconditioner	Everything was satisfactory!	JD.com
Tekla Intelligence	Battery Reconditioner	123456789123456789	JD.com
Tekla Intelligence	Battery Reconditioner	Have purchased many times and it is really good.[Smiles]	JD.com
Tekla Intelligence	Battery Reconditioner	It's not working.	JD.com
Tekla Intelligence	Battery Reconditioner	I hope it works.	JD.com
Tekla Intelligence	Battery Reconditioner	Good value for money worth buying three five star reviews1	JD.com
Tekla Intelligence	Battery Reconditioner	an excellent	JD.com
Tekla Intelligence	Battery Reconditioner	being carried out, the main quality is guaranteed, it is worth buying!	JD.com
Tekla Intelligence	Battery Reconditioner	Thank you!	JD.com
Tekla Intelligence	Battery Reconditioner	It's good stuff. It's good.	JD.com
Tekla Intelligence	Battery Reconditioner	I just arrived and tested it right away.	JD.com
Tekla Intelligence	Battery Reconditioner	Evaluate after use	JD.com
Tekla Intelligence	Battery Reconditioner	Baby received, logistics is very fast, packaging is very good, very satisfied!	JD.com
Tekla Intelligence	Battery Reconditioner	It's okay.	JD.com
Tekla Intelligence	Battery Reconditioner	It works great. The phone doesn't get hot.	JD.com

Tekla Intelligence	Battery Reconditioner	Try it for a week and then follow up	JD.com
Tekla Intelligence	Battery Reconditioner	I hope it's not an IQ tax.	JD.com
Tekla Intelligence	Battery Reconditioner	can	JD.com
Tekla Intelligence	Battery Reconditioner	This user feels the product is better	JD.com
Tekla Intelligence	Battery Reconditioner	Not bad, the voltage is a bit more stable, and the phone will last longer than it did before the charge was used	JD.com
Tekla Intelligence	Battery Reconditioner	Rip-off, no effect, I have used it for ten days.	JD.com
Tekla Intelligence	Battery Reconditioner	Quality is very good use it works use it again will recommend to buy[Smile]	JD.com
Tekla Intelligence	Battery Reconditioner	This user feels the product is better	JD.com
Tekla Intelligence	Battery Reconditioner	Great value for money and a lovely looking little item	JD.com
Tekla Intelligence	Battery Reconditioner	dissatisfied	JD.com
Tekla Intelligence	Battery Reconditioner	Used it once, it works, phone is not so hot	JD.com
Tekla Intelligence	Battery Reconditioner	Why do I feel like this is paying an IQ tax.	JD.com
Tekla Intelligence	Battery Reconditioner	Use it and then evaluate it.	JD.com
Tekla Intelligence	Battery Reconditioner	[Smiles][Smiles][Smiles]	JD.com
Tekla Intelligence	Battery Reconditioner	Battery Repairer received, the phone used once, feel good, satisfied with a shopping	JD.com
Tekla Intelligence	Battery Reconditioner	This user did not fill in a rating	JD.com
Tekla Intelligence	Battery Reconditioner	I can't charge it, I bought a new phone and it's slow to charge the new one, I don't know what's going on!	JD.com
Tekla Intelligence	Battery Reconditioner	Good, good, good, good, good, good, good, good, good, good, good, good, good.	JD.com
Tekla Intelligence	Battery Reconditioner	Fixing the battery should be okay, I feel like fixing the lag shouldn't be useful, I haven't used it yet, I'll talk about it later.	JD.com
Tekla Intelligence	Battery Reconditioner	Product quality is very good, worth buying	JD.com
Tekla Intelligence	Battery Reconditioner	Cell phone repairer received, open the package is very satisfied, is using, give five stars!	JD.com
Tekla Intelligence	Battery Reconditioner	The goods have been received, wait until after use to see if there is no effect to comment again!	JD.com
Tekla Intelligence	Battery Reconditioner	cell phone it was full the next day after using this one it works great!	JD.com
Tekla Intelligence	Battery Reconditioner	[Big Love]	JD.com
Tekla Intelligence	Battery Reconditioner	Baby received, I do not know how effective wait for a follow-up review!	JD.com
Tekla Intelligence	Battery Reconditioner	Good, fast, satisfied, kudos 5 stars☆oh. Thanks for the honesty of the merchant.	JD.com
Tekla Intelligence	Battery Reconditioner	(of an unmarried couple) be close	JD.com
Tekla Intelligence	Battery Reconditioner	high, use a period of time well continue to buy for the family.	JD.com
Tekla Intelligence	Battery Reconditioner	It's good, my phone used to get hot when charging, but today I got it back and it didn't get hot when charging!	JD.com
Tekla Intelligence	Battery Reconditioner	This user feels the product is better	JD.com
Tekla Intelligence	Battery Reconditioner	Just arrived haven't tried it yet. Hope it works well!	JD.com
Tekla Intelligence	Battery Reconditioner	It's not good at all.	JD.com
Tekla Intelligence	Battery Reconditioner	now it's still85% Are those of you who say it works dragging your feet? Can I get a refund for this and a charging cable for over 40 bucks?	JD.com
Tekla Intelligence	Battery Reconditioner	repairer has received, with the description of the match, the package is intact, the price is favorable, has not been tried, waiting for a follow-up evaluation. Fast logistics, good service attitude, satisfied!	JD.com
Tekla Intelligence	Battery Reconditioner	Satisfied well well well	JD.com
Tekla Intelligence	Battery Reconditioner	Received it and am using it. Not sure what all the numbers that change back and forth mean.	JD.com
Tekla Intelligence	Battery Reconditioner	Can't see the effect	JD.com

Tekla Intelligence	Battery Reconditioner	I've used it three times, but I haven't seen any effect yet.	JD.com
Tekla Intelligence	Battery Reconditioner	I don't know the effect of repairing without using it, so I'll give it a good review first!	JD.com
Tekla Intelligence	Battery Reconditioner	Not bad.	JD.com
Tekla Intelligence	Battery Reconditioner	Seems to work fine so far, will follow up with more time! 5out of5first	JD.com
Tekla Intelligence	Battery Reconditioner	Two weeks in and no results at all, the publicity is a total lie, will never trust advertising again.	JD.com
Tekla Intelligence	Battery Reconditioner	Things received, the appearance of technology full of sense of use is very convenient and fast, the price is also very affordable, the delivery speed is also very fast!	JD.com
Tekla Intelligence	Battery Reconditioner	pretty good	JD.com
Tekla Intelligence	Battery Reconditioner	Good quality at a good price give a good review	JD.com
Tekla Intelligence	Battery Reconditioner	Superb and top notch repair👍👍👍👍👍	JD.com
Tekla Intelligence	Battery Reconditioner	Satisfaction!	JD.com
Tekla Intelligence	Battery Reconditioner	Repairer received, used twice the effect is very good, indeed not false, really high-tech ah, after repairing the phone runs very quickly, to be8weeks after the effect may be even better, it is worth buying, friends quickly order it!	JD.com
Tekla Intelligence	Battery Reconditioner	Logistics is very fast, business service attitude is good, serious and responsible to solve every problem.	JD.com
Tekla Intelligence	Battery Reconditioner	Haven't used it yet, will follow up if it works well.	JD.com
Tekla Intelligence	Battery Reconditioner	Use it for a while and then evaluate further.	JD.com
Tekla Intelligence	Battery Reconditioner	This product is very good and worth buying.	JD.com
Tekla Intelligence	Battery Reconditioner	It's pretty good, I'll follow up after using it for a while.	JD.com
Tekla Intelligence	Battery Reconditioner	Cell phone repairer received ah, logistics fast, inexpensive, to the business five-star praise!	JD.com
Tekla Intelligence	Battery Reconditioner	Seems to be working. Standby time is a little longer.	JD.com
Tekla Intelligence	Battery Reconditioner	Good stuff hope it works well for my cell phone	JD.com
Tekla Intelligence	Battery Reconditioner	This works very well, to my surprise, originally the phone was very laggy, and now it's all good!	JD.com
Tekla Intelligence	Battery Reconditioner	I haven't used it yet, I'll comment afterward.	JD.com
Tekla Intelligence	Battery Reconditioner	Things received very good, tightly packaged without damage, the boss is very good and careful, the next time will come back, I wish the store business prosperity!	JD.com
Tekla Intelligence	Battery Reconditioner	Logistics is fast.Good service attitude and product quality!	JD.com
Tekla Intelligence	Battery Reconditioner	plugged in all over the world, I feel that it is effective, and then insist on using it, I hope to use it for a longer period of time.	JD.com
Tekla Intelligence	Battery Reconditioner	Nice machine, in use, looking forward to the results, will comment after some time!	JD.com
Tekla Intelligence	Battery Reconditioner	Goods have been received, logistics is very fast, well-packed, used two days, the effect is very good, the battery has improved significantly, the next time will patronize!	JD.com
Tekla Intelligence	Battery Reconditioner	Haven't had a chance to try it out yet! Hopefully it works!	JD.com
Tekla Intelligence	Battery Reconditioner	Cell phone battery repairer has been received, is trying, with very good	JD.com
Tekla Intelligence	Battery Reconditioner	The goods received, logistics is very fast, looks good quality, will use it later, worth buying.	JD.com
Tekla Intelligence	Battery Reconditioner	Baby has received, the appearance is good, logistics is also quite fast, is using the expected results	JD.com
Tekla Intelligence	Battery Reconditioner	Express quite fast, bought once before, used the effect is good, the phone less self-heating phenomenon, easy to use, plug in the charging treasure can also be used, like.	JD.com
Tekla Intelligence	Battery Reconditioner	I just used it once and I can't feel it so I can't comment on it.	JD.com
Tekla Intelligence	Battery Reconditioner	Things received, well-packed, baby compact, after using a follow-up review.	JD.com
Tekla Intelligence	Battery Reconditioner	Looks good! Don't know if it's good to use! I hope it's as described! I hope it works.	JD.com
Tekla Intelligence	Battery Reconditioner	Tried a few cell phones, the original old cell phone does not charge into the electricity, now there is a can charge sixty percent of the electricity, another or charge into, or have a certain effect, try a few days in the follow-up!	JD.com

Tekla Intelligence	Battery Reconditioner	The item has been received and I hope it does work to repair my cell phone battery.	JD.com
Tekla Intelligence	Battery Reconditioner	Very good, worth buying oh	JD.com
Tekla Intelligence	Battery Reconditioner	can	JD.com
Tekla Intelligence	Battery Reconditioner	usefulness of the real intelligence (idiom); efficient use of resources	JD.com
Tekla Intelligence	Battery Reconditioner	Cell phone repairer received a very good price will be the second time to buy enough to give a good review!	JD.com
Tekla Intelligence	Battery Reconditioner	The cell phone repairer received, the appearance of the design is good, logistics is very fast!	JD.com
Tekla Intelligence	Battery Reconditioner	Good compatibility, multiple types of cell phones available, fast charging, and repaired cell phones, great.	JD.com
Tekla Intelligence	Battery Reconditioner	The phone had been heating up from time to time before and had to be turned off periodically, bought this fixer to try it out, have not found the phone heating up on its own for the time being, will continue to use it and see how it works out.	JD.com
Tekla Intelligence	Battery Reconditioner	Goods received, fast logistics, first five stars, what is the quality of use after saying.	JD.com
Tekla Intelligence	Battery Reconditioner	Used it for a few days and it worked a bit. Use it for a while longer and comment later	JD.com
Tekla Intelligence	Battery Reconditioner	Cell phone battery smart repairer, repair battery to improve life. Technology chip multiple protection, detect current and voltage, repair and maintain the battery, do not hurt the battery, intelligent repair from the inside out.	JD.com
Tekla Intelligence	Battery Reconditioner	goods to open the first time to see, it is very good👍, very satisfied😃, customer service also have a question and answer, thank you🙏	JD.com
Tekla Intelligence	Battery Reconditioner	Goods received, do not know how effective?	JD.com
Tekla Intelligence	Battery Reconditioner	Baby has been received, use a day feel okay, another two days to see.	JD.com
Tekla Intelligence	Battery Reconditioner	The merchandise is great and looks great, it's worth getting one if you need one!	JD.com
Tekla Intelligence	Battery Reconditioner	What the hell is wrong?	JD.com
Tekla Intelligence	Battery Reconditioner	merchant wishing the merchant a prosperous business year after year!	JD.com
Tekla Intelligence	Battery Reconditioner	Used it and hopefully it will fix the battery	JD.com
Tekla Intelligence	Battery Reconditioner	packaged, did not use the high-tech products, with a use in the follow-up.	JD.com
Tekla Intelligence	Battery Reconditioner	The stuff is good quality and high tech. The merchant is reputable and the courier is very hardworking.	JD.com
Tekla Intelligence	Battery Reconditioner	Received yesterday, feel good, beautiful design, good shopping, fast logistics, thanks to the courier boy	JD.com
Tekla Intelligence	Battery Reconditioner	Super good logistics fast customer service is good this in the purchase of your products!	JD.com
Tekla Intelligence	Battery Reconditioner	Hand experience: good<br>Product design: beautiful<br>Product appearance: bright<br>Face appearance: worth buying<br>Gray Pink: black<br>Fit: good<br>Product function: used feasible, helpful enhancement<br>Joint description: good<br>Elasticity evaluation: good<br>Charging cable: do not need<br>Utility: practical<br>Protective case: good	JD.com
Tekla Intelligence	Battery Reconditioner	Goods received. Business enthusiasm logistics is very fast. Give five-star praise.	JD.com
Tekla Intelligence	Battery Reconditioner	Function: very good<br>Design: nice<br>Elasticity Description: very good	JD.com
Tekla Intelligence	Battery Reconditioner	Cell phone battery repairer, has received, high value, affordable gifts, the effect is in progress, overall, very satisfied! Five-star praise! Worth recommending and buying!!!	JD.com
Tekla Intelligence	Battery Reconditioner	Just received a try, the effect is very good, a very good one over the shopping, on the phone to help a lot, good-looking style, logistics is very fast, business service attitude is very good, give five stars!	JD.com
Tekla Intelligence	Battery Reconditioner	It's a bit of a snub.	JD.com
Tekla Intelligence	Battery Reconditioner	Works absolutely great, does repair batteries, worth the money, buy again in the future if needed.	JD.com

Tekla Intelligence	Battery Reconditioner	Started to repair my phone under the guidance of customer service, customer service is very good, see the data on the repairer has been changing, it should be in the process of repairing, very good, very satisfied.	JD.com
Tekla Intelligence	Battery Reconditioner	view, found that the outer packaging (box) is intact, the repair device is also very good, no damage phenomenon, as for how to repair the effect, to be used to follow up, very satisfied with a shopping.	JD.com
Tekla Intelligence	Battery Reconditioner	I plugged it in at night when I was sleeping, the product number display constantly jumped, the phone will be very peaceful to repair, no other adverse reactions, very good, more repair a few times better results.	JD.com
Tekla Intelligence	Battery Reconditioner	of good workmanship, easy to carry, inexpensive and beautiful, they can repair the battery magic weapon. The instruction manual is clear, the operation is very simple to use, everyone will use.	JD.com
Tekla Intelligence	Battery Reconditioner	Started to repair my phone under the guidance of customer service, customer service is very good, see the data on the repairer has been changing, it should be in the process of repairing, very good, very satisfied.	JD.com
Tekla Intelligence	Battery Reconditioner	of good workmanship, easy to carry, inexpensive and beautiful, they can repair the battery magic weapon. The instruction manual is clear, the operation is very simple to use, everyone will use.	JD.com
Tekla Intelligence	Battery Reconditioner	Works absolutely great, does repair batteries, worth the money, buy again in the future if needed.	JD.com
Tekla Intelligence	Battery Reconditioner	Charge once, use cell phone time increased by 2hours, it does work. Merchants ship quickly, good price, five stars.	JD.com
Tekla Intelligence	Battery Reconditioner	there is no current sound, the stability is very good, deliberately used for a few days, the phone feels really good, praise!	JD.com
Tekla Intelligence	Battery Reconditioner	Appearance is beautiful, there is a digital display, although it can not read[snicker], tried a few times did not have a great change, read the instructions for a week with 2,3times, is to be commonly used, will not be instantly visible, more than a few times to come back to the review.	JD.com
Tekla Intelligence	Battery Reconditioner	I plugged it in at night when I was sleeping, the product number display constantly jumped, the phone will be very peaceful to repair, no other adverse reactions, very good, more repair a few times better results.	JD.com
Tekla Intelligence	Battery Reconditioner	Powerful, strong repair force, the effect is immediately visible, top quality, not enough, but also want to buy, point of praise, due to the repair effect, decided to buy another, also everyone recommended the past, really good.	JD.com
Tekla Intelligence	Battery Reconditioner	number in the continuous output changes, while playing a role in the charging output voltage regulator, so as to protect the charging of the cell phone battery plays a significant role, worth buying!	JD.com
Tekla Intelligence	Battery Reconditioner	Cell phone battery repairer received, I used it to repair the glory and Apple, used 3days to evaluate, off the charge is really effective! Merchants shipping very quickly logistics is also fast, five-star praise!	JD.com
Tekla Intelligence	Battery Reconditioner	This cell phone repairer is small and exquisite in appearance, easy to use, worth buying. This item is so practical, super quality, worth buying.	JD.com
Tekla Intelligence	Battery Reconditioner	view, found that the outer packaging (box) is intact, the repair device is also very good, no damage phenomenon, as for how to repair the effect, to be used to follow up, very satisfied with a shopping.	JD.com
Tekla Intelligence	Battery Reconditioner	Fast logistics Beautiful appearance and thorough service Good repair effect Reliable quality Worth having, small things play a big role, good value for money!	JD.com
Tekla Intelligence	Battery Reconditioner	are real, small things have a big role, good value for money! Very interesting stuff, protect the cell phone battery kudos collection.	JD.com
Yearly pop-up store	Battery Reconditioner	The store's gone.	JD.com
Manxuan Technology	Battery Reconditioner	It's fast.	JD.com
Manxuan Technology	Battery Reconditioner	This user feels the product is better	JD.com
Manxuan Technology	Battery Reconditioner	It can also be repaired before the phone began to run out of power at about 4, after the repair to 8or 9pm before needing to charge, indicating that there is an effect, before buying has not been convinced, but also saw a comment that is the IQ tax, holding 10dollars less than try the mentality of shooting not, the results can also be	JD.com

Manxuan Technology	Battery Reconditioner	I just received it. I just tried it out. I'm happy with it.	JD.com
Manxuan Technology	Battery Reconditioner	It doesn't work It won't charge all night It's a scam	JD.com
Manxuan Technology	Battery Reconditioner	This user feels the product is better	JD.com
Manxuan Technology	Battery Reconditioner	convenient, multiple protection. It's really a battery protector.	JD.com
Manxuan Technology	Battery Reconditioner	This user feels the product is better	JD.com
Manxuan Technology	Battery Reconditioner	[Big Love]	JD.com
Manxuan Technology	Battery Reconditioner	This user feels the product is better	JD.com
Manxuan Technology	Battery Reconditioner	Very pleased![Big love][Big love][love][love][Big love][Big love][Big love][Big love][Big love]	JD.com
Manxuan Technology	Battery Reconditioner	This user feels the product is better	JD.com
Manxuan Technology	Battery Reconditioner	This user feels the product is better	JD.com
Manxuan Technology	Battery Reconditioner	Logistics is fast, value for money, good quality, beautiful appearance, after the test is very good!	JD.com
Manxuan Technology	Battery Reconditioner	Goods have been received, have not yet used, there will be later evaluation	JD.com
Manxuan Technology	Battery Reconditioner	Goods have been received, good! Logistics is fast	JD.com
Manxuan Technology	Battery Reconditioner	logistics is fast, the effect will be followed up, introduced to friends.	JD.com
Manxuan Technology	Battery Reconditioner	This user feels the product is better	JD.com
Manxuan Technology	Battery Reconditioner	This user feels the product is better	JD.com
Manxuan Technology	Battery Reconditioner	This user feels the product is better	JD.com
Manxuan Technology	Battery Reconditioner	Can display charging current and voltage, more intuitive. The maintenance of the old battery is not a few times can be restored, still trying. Receive first	JD.com
Manxuan Technology	Battery Reconditioner	Looking forward to the results	JD.com
Manxuan Technology	Battery Reconditioner	This user feels the product is better	JD.com
Manxuan Technology	Battery Reconditioner	Battery Repairer to receive the quality is very good as described logistics is also very fast service too degree is also very good satisfaction.	JD.com
Manxuan Technology	Battery Reconditioner	This user feels the product is better	JD.com
Manxuan Technology	Battery Reconditioner	It seems to be working.	JD.com
Manxuan Technology	Battery Reconditioner	I don't know how well it works yet, I'd rate it average.	JD.com
Manxuan Technology	Battery Reconditioner	(of an unmarried couple) be close	JD.com
Manxuan Technology	Battery Reconditioner	This user feels the product is better	JD.com
Manxuan Technology	Battery Reconditioner	This cell phone battery repairer is tightly packaged. The trial effect is very good. My Huawei Honor NOTE8with more years, charging with it to try two days a little effect.	JD.com
Manxuan Technology	Battery Reconditioner	非常好好好好好好好好好好好好好好好好好好好好好好好好好[微笑][微笑][微笑][微笑][微笑][微笑][微笑][微笑][微笑][微笑][微笑]	JD.com
Manxuan Technology	Battery Reconditioner	Merchants shipping speed, for cell phone charging is quite good	JD.com
Manxuan Technology	Battery Reconditioner	This user feels the product is better	JD.com
Manxuan Technology	Battery Reconditioner	Received the goods very good quality is good.	JD.com
Manxuan Technology	Battery Reconditioner	effect is being used after the evaluation, the logistics is fast!	JD.com
Manxuan Technology	Battery Reconditioner	This user feels the product is better	JD.com
Manxuan Technology	Battery Reconditioner	Well made, effective and very satisfied.	JD.com
Manxuan Technology	Battery Reconditioner	I used to always buy from physical stores, but this time I chose to buy online, not only is it authentic, but the price is also affordable!,🎁Baby received, the color is very nice, the texture is also very good, the look is simple!😍It's what I want! Will come back next time! Give me 5 stars!	JD.com
Manxuan Technology	Battery Reconditioner	It's great and it works, thanks[kudos][kudos][kudoshandshake][handshake][handshakehugs][hugs][hug][hugs]	JD.com

Manxuan Technology	Battery Reconditioner	This user feels the product is better	JD.com
Manxuan Technology	Battery Reconditioner	Cell phone battery repairer received, beautiful and beautiful design, logistics is also very fast. Very good!	JD.com
Manxuan Technology	Battery Reconditioner	useless	JD.com
Manxuan Technology	Battery Reconditioner	As described, fast shipping and logistics courier service.	JD.com
Manxuan Technology	Battery Reconditioner	The quality of the product is very good, the customer service is very high quality, enthusiastic and level, know very comprehensive, thank you very much!	JD.com
Manxuan Technology	Battery Reconditioner	I haven't used it yet.	JD.com
Manxuan Technology	Battery Reconditioner	The goods have received, do not know if it is not good to use, use and then come back to evaluate. Logistics service are very satisfied, thank you!	JD.com
Manxuan Technology	Battery Reconditioner	affordable than the store , , , , to be used to see the effect of the	JD.com
Manxuan Technology	Battery Reconditioner	This user feels the product is better	JD.com
Manxuan Technology	Battery Reconditioner	I've been using it for a few days now and it's working.	JD.com
Manxuan Technology	Battery Reconditioner	Just received the goods do not know if it is good to use it habit of praise	JD.com
Manxuan Technology	Battery Reconditioner	The goods arrived, the appearance of the quality is good, to be used after the evaluation.	JD.com
Manxuan Technology	Battery Reconditioner	I just got it, and it will be a month or two before I know how it works.	JD.com
Manxuan Technology	Battery Reconditioner	is good or not, I am afraid of clearing the memory information!	JD.com
Manxuan Technology	Battery Reconditioner	hot, the power long enough to increase the length, praise praise praise	JD.com
Manxuan Technology	Battery Reconditioner	Received, don't know how to open it?	JD.com
Manxuan Technology	Battery Reconditioner	IQ tax	JD.com
Manxuan Technology	Battery Reconditioner	my phone has been working for years. The original 59just shut down repaired twice. I was able to credit the phone for more than ten hours. This repair battery my cell phone works great FYI!	JD.com
Manxuan Technology	Battery Reconditioner	Let's try it first, I don't know how it works yet	JD.com
Manxuan Technology	Battery Reconditioner	I'm using it now, I'll follow up if it works	JD.com
Manxuan Technology	Battery Reconditioner	I just used it. I don't know.	JD.com
Manxuan Technology	Battery Reconditioner	I received the goods, whether it is good or not, I will try and then comment.	JD.com
Manxuan Technology	Battery Reconditioner	Just as described, works pretty well, all 5 stars!	JD.com
Manxuan Technology	Battery Reconditioner	Goods received.	JD.com
Manxuan Technology	Battery Reconditioner	Cell phone battery repair device received, immediately charged on the test, look should be quite good, temporarily do not know how the effect, and then follow up later, fast logistics, business service attitude is good!	JD.com
Manxuan Technology	Battery Reconditioner	It works great.	JD.com
Manxuan Technology	Battery Reconditioner	Good and effective. Can be purchased.	JD.com
Manxuan Technology	Battery Reconditioner	I don't know if it works or not, looking forward to the results	JD.com
Manxuan Technology	Battery Reconditioner	Just received do not know the effect, accustomed to praise, after a period of time in the additional.	JD.com
Manxuan Technology	Battery Reconditioner	Workmanship is fair. Trying it out yet to see results to be followed up with results!	JD.com
Manxuan Technology	Battery Reconditioner	Very good stuff, recommended, worth buying oh! Good review!!!	JD.com
Manxuan Technology	Battery Reconditioner	It's good, it's good.	JD.com
Manxuan Technology	Battery Reconditioner	I can't tell anything yet, so I'll use it and add more comments when I'm done!	JD.com
Manxuan Technology	Battery Reconditioner	acceptable	JD.com
Manxuan Technology	Battery Reconditioner	Completed the operation as requested, started at 12pmlast nightto8amthis morning,, don't know about the rest, opened the software a lot smoother, that's it	JD.com
Manxuan Technology	Battery Reconditioner	Courier service is good, have not yet begun to use, with the use of the praise in the	JD.com

Manxuan Technology	Battery Reconditioner	It's being tested. It's a good price.	JD.com
Manxuan Technology	Battery Reconditioner	It works well and the battery durability is much better after using it.	JD.com
Manxuan Technology	Battery Reconditioner	Not bad.	JD.com
Manxuan Technology	Battery Reconditioner	Works great Convenient and practical, great stuff	JD.com
Manxuan Technology	Battery Reconditioner	(of an unmarried couple) be close	JD.com
Manxuan Technology	Battery Reconditioner	Logistics is very fast, cell phone battery repairer received, used once, see the effect, first five-star praise!	JD.com
Manxuan Technology	Battery Reconditioner	Good review!	JD.com
Manxuan Technology	Battery Reconditioner	Very enthusiastic seller, the next time to come back to hope that the next time there will be an opportunity to cooperate with you to wish you a prosperous business Quality is very good really out of my expectations Packaging very carefully Thank you very much. Wish you prosperous business	JD.com
Manxuan Technology	Battery Reconditioner	The program's still messed up.	JD.com
Manxuan Technology	Battery Reconditioner	I've got it, I haven't used it yet. I have to turn it off to use it. It's a pain in the ass.	JD.com
Manxuan Technology	Battery Reconditioner	Used it for two days, tried to charge a couple things, can't say how well it works, use it for a while and see.	JD.com
Manxuan Technology	Battery Reconditioner	I don't know if it works or not, I'll give it a good review first and come back after using it	JD.com
Manxuan Technology	Battery Reconditioner	Good quality and fast logistics I like it very much	JD.com
Manxuan Technology	Battery Reconditioner	Expectations in use	JD.com
Manxuan Technology	Battery Reconditioner	Must take8weeks to repair,will comment after8weeks, first give a good review.	JD.com
Manxuan Technology	Battery Reconditioner	Logistics is fast, the goods have been received, to be used.	JD.com
Manxuan Technology	Battery Reconditioner	Fine workmanship, beautiful appearance, good quality, with voltage and current display, very good, satisfied!	JD.com
Manxuan Technology	Battery Reconditioner	evaluation after use	JD.com
Manxuan Technology	Battery Reconditioner	This cell phone battery repairer is really good for those who have faith to buy with confidence.	JD.com
Manxuan Technology	Battery Reconditioner	Charging effect is very good, charging time is short, charging effect is very good, the phone is durable	JD.com
Manxuan Technology	Battery Reconditioner	It's in great condition so far! Not only does it look great it's also very techy and cool! Plugs in tight and secure! The charging function can be turned off and repaired! At the same time to buy2different brands, this product is not only the price is lower, the function is also stronger than the high price! I marvel at Made in China![Kudos]In order not to mislead other spellers, later will follow up the review!	JD.com
Manxuan Technology	Battery Reconditioner	It's a little useful.	JD.com
Manxuan Technology	Battery Reconditioner	I'm getting used to it.	JD.com
Manxuan Technology	Battery Reconditioner	Habitual praise	JD.com
Manxuan Technology	Battery Reconditioner	Restorers are very good very good very good	JD.com
Manxuan Technology	Battery Reconditioner	Exquisite workmanship, beautiful appearance, normal display	JD.com
Manxuan Technology	Battery Reconditioner	Use it.	JD.com
Manxuan Technology	Battery Reconditioner	Works great, charges quickly	JD.com
Manxuan Technology	Battery Reconditioner	Customer service patiently answered questions because I didn't know how to use it. Thank you very much!	JD.com
Manxuan Technology	Battery Reconditioner	is very good, repair the phone fast, business service attitude is good!	JD.com
Manxuan Technology	Battery Reconditioner	Not at this time.	JD.com
Manxuan Technology	Battery Reconditioner	The display is normal and it looks cool	JD.com
Manxuan Technology	Battery Reconditioner	I don't know if it works.	JD.com
Manxuan Technology	Battery Reconditioner	The logistics is quite fast, the quality is average, not much use	JD.com
Manxuan Technology	Battery Reconditioner	Satisfied, unused.	JD.com

Manxuan Technology	Battery Reconditioner	Just received the goods, not yet used to wait until after the use of evaluation	JD.com
Manxuan Technology	Battery Reconditioner	I just used it and don't know the effect yet, waiting for a follow up review.	JD.com
Manxuan Technology	Battery Reconditioner	battery and felt it did the trick. Keep flushing for a couple weeks and see if there is a big change in the battery before I follow up.	JD.com
Manxuan Technology	Battery Reconditioner	lagging, repairer arrived, a small look can be, as the need to shut down the repair, the business said2timesa week to repair, a3-hour, continuous repair of8weeks16times, that only operate at night when you go to bed, look at the evaluation of the good to use, hopefully, repair the good phone is not in the heating, lagging, the first positive feedback.2months after the follow-up comment[hee hee]	JD.com
Manxuan Technology	Battery Reconditioner	Product received, first praise5points, the effect of how, to be used and then comment.	JD.com
Manxuan Technology	Battery Reconditioner	Fast logistics, easy to use, very satisfied.	JD.com
Manxuan Technology	Battery Reconditioner	Received, in the test, the process has a pulse action, whether it works or not have to see in a few days, first praise. Really hope to succeed, even if the IQ tax also dare to try, ha, old iron do not spray.	JD.com
Manxuan Technology	Battery Reconditioner	[Big Love]	JD.com
Manxuan Technology	Battery Reconditioner	Not yet used	JD.com
Manxuan Technology	Battery Reconditioner	I've been using it for a while now and I don't feel it's working	JD.com
Manxuan Technology	Battery Reconditioner	attitude is very good, logistics is also very fast, give five stars!	JD.com
Manxuan Technology	Battery Reconditioner	Exquisite workmanship	JD.com
Manxuan Technology	Battery Reconditioner	I'll use it right away and see how it works	JD.com
Manxuan Technology	Battery Reconditioner	Goods are well packaged, logistics is fast, the goods look good. Customer service attitude is very good! To be tried after the effect and then evaluate the practical situation. Thank you for the business also delivered a manual!	JD.com
Manxuan Technology	Battery Reconditioner	I received the goods look okay specific effect how to use the follow-up comment!	JD.com
Manxuan Technology	Battery Reconditioner	I don't know if it works well, it's good quality! Come back later to evaluate it!	JD.com
Manxuan Technology	Battery Reconditioner	The item was received and is of good quality. It's what I wanted.	JD.com
Manxuan Technology	Battery Reconditioner	twice. Can't see the effect. To use eight weeks. Later to see the effect, do not know exactly how the situation. First praise.	JD.com
Manxuan Technology	Battery Reconditioner	battery charges faster, the capacity is increased and it lasts longer.	JD.com
Manxuan Technology	Battery Reconditioner	I don't know if it works, but I'll use it first	JD.com
Manxuan Technology	Battery Reconditioner	Logistics is very fast packaging satisfaction, I wish the owner a prosperous business and prosperity.	JD.com
Manxuan Technology	Battery Reconditioner	Good quality, I'll comment after I use it for restoration.	JD.com
Manxuan Technology	Battery Reconditioner	It feels effective use it for some more days to see, let's give it a good review first.	JD.com
Manxuan Technology	Battery Reconditioner	Received it, haven't used it yet.	JD.com
Manxuan Technology	Battery Reconditioner	It's working fine. It's charging faster than the previous one.	JD.com
Manxuan Technology	Battery Reconditioner	Use it a couple of times to see if it works and have another positive review.	JD.com
Manxuan Technology	Battery Reconditioner	Baby received, well-packed, very tight, fast courier delivery, quality is good, very like, full five-star praise! Thanks to the seller, to provide good value for money baby! Wish the seller a prosperous business!	JD.com
Manxuan Technology	Battery Reconditioner	(of an unmarried couple) be close	JD.com
Manxuan Technology	Battery Reconditioner	Looked stop good thanks boss wish you good luck with your business.	JD.com
Manxuan Technology	Battery Reconditioner	serviceable	JD.com
Manxuan Technology	Battery Reconditioner	It does work well, there is a ZTE folding screen noggin, years put aside without charging without any response, with this fixer immediately see the effect! Good!	JD.com
Manxuan Technology	Battery Reconditioner	Goods received, in the test, the specific effect of the later to know, but customer service is very good, there is no clear customer service quickly reply!	JD.com

Manxuan Technology	Battery Reconditioner	Good workmanship, looking forward to the results.	JD.com
Manxuan Technology	Battery Reconditioner	I didn't look at it when I bought it, and I still need to format my phone to use this, so if that's not necessary it can also serve the purpose of fast charging the phone, right? That's it! I don't want to say anything else.	JD.com
Manxuan Technology	Battery Reconditioner	Not bad, not bad, it works so well, it's called Crooked Gudet in our American parlance!	JD.com
Manxuan Technology	Battery Reconditioner	Even if you pay the IQ tax.	JD.com
Manxuan Technology	Battery Reconditioner	Cell phone fixer received, thank you boss!	JD.com
Manxuan Technology	Battery Reconditioner	Don't know how to use it yet? You can't reach out the front, you can't use it?	JD.com
Manxuan Technology	Battery Reconditioner	pretty good	JD.com
Manxuan Technology	Battery Reconditioner	It's good[Laughs] [Laughs] [Laughs]	JD.com
Manxuan Technology	Battery Reconditioner	I didn't feel any results after using it for two days! Looking forward to follow up with results I guess!	JD.com
Manxuan Technology	Battery Reconditioner	German chip, battery cell phone charger repairer is genuine, good quality, the thing that fast, high price ratio.	JD.com
Manxuan Technology	Battery Reconditioner	I haven't used it yet, if it's good, I'll buy a few more.	JD.com
Manxuan Technology	Battery Reconditioner	general	JD.com
Manxuan Technology	Battery Reconditioner	Use it for a few days and then evaluate	JD.com
Manxuan Technology	Battery Reconditioner	Have received it and tried it can work well next time thanks.	JD.com
Manxuan Technology	Battery Reconditioner	Very good👍	JD.com
Manxuan Technology	Battery Reconditioner	I'll try it and then I'll tell you more.	JD.com
Manxuan Technology	Battery Reconditioner	I'll give it a 5 star review first and use it to see what happens!	JD.com
Manxuan Technology	Battery Reconditioner	How to use my Huawei tablet	JD.com
Manxuan Technology	Battery Reconditioner	This user feels the product is better	JD.com
Manxuan Technology	Battery Reconditioner	There is no effect, the power feels still use fast, after using the fast charging function is no longer, charging slow as hell,5.1Vcharging	JD.com
Manxuan Technology	Battery Reconditioner	I haven't used it yet, I don't know how it works.	JD.com
Manxuan Technology	Battery Reconditioner	Goods received, packaging and instructions on even the minimum product manufacturer, address, date of production, batch, certificate of conformity, warranty card, etc. are not, to be tried to see how the effect say it.	JD.com
Manxuan Technology	Battery Reconditioner	It's not working.	JD.com
Manxuan Technology	Battery Reconditioner	I haven't seen any results, so I'll give you a good review first	JD.com
Manxuan Technology	Battery Reconditioner	quality.<br>Packaging specification, easy to use, good value for money, recommendable.	JD.com
Manxuan Technology	Battery Reconditioner	The goods arrived! I don't know if it works after use!	JD.com
Manxuan Technology	Battery Reconditioner	Let's use it and see if it works.	JD.com
Manxuan Technology	Battery Reconditioner	Cell phone battery repairer received, logistics fast service is very satisfied.	JD.com
Manxuan Technology	Battery Reconditioner	Exquisite workmanship, beautiful shape, display normal price and good quality praise	JD.com
Manxuan Technology	Battery Reconditioner	It looks good, I don't know if it works or not, I haven't tried it yet.	JD.com
Manxuan Technology	Battery Reconditioner	(of an unmarried couple) be close	JD.com
Manxuan Technology	Battery Reconditioner	Charging up is slower than the original	JD.com
Manxuan Technology	Battery Reconditioner	A little bit🔥IQ[tears]	JD.com
Manxuan Technology	Battery Reconditioner	Miserable, slower charging, can't see any results at all	JD.com
Manxuan Technology	Battery Reconditioner	Just received it, fast shipping, thanks!	JD.com

Manxuan Technology	Battery Reconditioner	deliberately used for a period of time to evaluate, room shell exquisite workmanship is simple and convenient to use, the use of good results, cell phone battery power than the original durable, very satisfied with a purchase, full five-star praise!	JD.com
Manxuan Technology	Battery Reconditioner	[Big Love]Ivery satisfied with this product of yours? The logistics and courier service is also very satisfied? Thank you. I'm very happy with this product!	JD.com
Manxuan Technology	Battery Reconditioner	Still trying it out, hope it works	JD.com
Manxuan Technology	Battery Reconditioner	Just used it, first praise, expect to have the effect	JD.com
Manxuan Technology	Battery Reconditioner	senseless	JD.com
Manxuan Technology	Battery Reconditioner	I don't know if it works well. I haven't used it yet.	JD.com
Manxuan Technology	Battery Reconditioner	classifier for objects in rows such as words	JD.com
Manxuan Technology	Battery Reconditioner	Tried it but don't know the effect yet, will reevaluate some other time	JD.com
Manxuan Technology	Battery Reconditioner	product are very formal, fine workmanship no odor, try it first, looking forward to good results and thank you for customer service help[cool]	JD.com
Manxuan Technology	Battery Reconditioner	Logistics is very fast, but do not know how to repair the battery, waiting to use a period of time to come back to a follow-up review, the first five-star praise[smile]	JD.com
Manxuan Technology	Battery Reconditioner	I got it. - Thank you.	JD.com
Manxuan Technology	Battery Reconditioner	Great platform to buy from, good quality, especially with a brand name, trustworthy	JD.com
Manxuan Technology	Battery Reconditioner	(of an unmarried couple) be close	JD.com
Manxuan Technology	Battery Reconditioner	Exquisite workmanship, beautiful shape, display normal, just do not know how effective	JD.com
Manxuan Technology	Battery Reconditioner	Received the goods, last night began to use according to the instructions, to say the effect of the first time to use a moment is not good to say, may have to be many times before we can see the effect of it!	JD.com
Manxuan Technology	Battery Reconditioner	Baby value for money, love it, will come back next time, especially especially especially good, there is no breakage, the appearance also looks good, praise! Express really fast, packaging high, very good!	JD.com
Manxuan Technology	Battery Reconditioner	This is really good, the material and workmanship is particularly exquisite, the workmanship is really very good, I think I can recommend to my friends to buy	JD.com
Manxuan Technology	Battery Reconditioner	Review after use.	JD.com
Manxuan Technology	Battery Reconditioner	Received, have not yet used, looking forward to the effect, first favorable comments	JD.com
Manxuan Technology	Battery Reconditioner	acceptable	JD.com
Manxuan Technology	Battery Reconditioner	It works great.	JD.com
Manxuan Technology	Battery Reconditioner	Nothing special when charging for the time being	JD.com
Manxuan Technology	Battery Reconditioner	can	JD.com
Manxuan Technology	Battery Reconditioner	Received the goods linked to the phone to try, useful again to evaluate	JD.com
Manxuan Technology	Battery Reconditioner	favorable criticism	JD.com
Manxuan Technology	Battery Reconditioner	It works well. You.	JD.com
Manxuan Technology	Battery Reconditioner	Fixing the machine won't work, think twice!	JD.com
Manxuan Technology	Battery Reconditioner	Affordable quality assurance and great results worth buying.	JD.com
Manxuan Technology	Battery Reconditioner	It's still working [Loves it]	JD.com
Manxuan Technology	Battery Reconditioner	The item was received and I am very happy with it.	JD.com
Manxuan Technology	Battery Reconditioner	We'll see how it works before we add stars	JD.com
Manxuan Technology	Battery Reconditioner	Very good.[stammers][stammers][stammers]	JD.com
Manxuan Technology	Battery Reconditioner	The product has been used many times and does have a durable cell phone battery.(The cell phone is an old smart phone)	JD.com
Manxuan Technology	Battery Reconditioner	Received the product looks pretty good not yet used to wait for a good result!	JD.com

Manxuan Technology	Battery Reconditioner	Very good quality, very satisfied, next time to buy your products	JD.com
Manxuan Technology	Battery Reconditioner	Fine workmanship good customer service attitude five-star praise has not been used to use in the evaluation	JD.com
Manxuan Technology	Battery Reconditioner	of no use	JD.com
Manxuan Technology	Battery Reconditioner	look at the phone is guaranteed, logistics is also very fast, very good.	JD.com
Manxuan Technology	Battery Reconditioner	Just received it and am trying it out. It repeatedly shows the voltage and current of the charge. Pretty good!	JD.com
Manxuan Technology	Battery Reconditioner	Logistics and express very quickly, all services are very good! Wish the boss a prosperous business prosperity and wealth!	JD.com
Manxuan Technology	Battery Reconditioner	I'll review it after I use it.	JD.com
Manxuan Technology	Battery Reconditioner	Fast shipping, good quality item, nice!	JD.com
Manxuan Technology	Battery Reconditioner	Received the goods, is trying, waiting for follow-up evaluation	JD.com
Manxuan Technology	Battery Reconditioner	It's not working yet.	JD.com
Manxuan Technology	Battery Reconditioner	Workmanship is very good, like a big youtuber, hard material, looking forward to the effect in	JD.com
Manxuan Technology	Battery Reconditioner	The goods received, not yet used, first praise.	JD.com
Manxuan Technology	Battery Reconditioner	Repair in progress don't know how well it works try it out and reevaluate.	JD.com
Manxuan Technology	Battery Reconditioner	I'm talking too fast.	JD.com
Manxuan Technology	Battery Reconditioner	the more you do better and better, very good shopping experience!	JD.com
Manxuan Technology	Battery Reconditioner	Received.s charging now[Kudos]	JD.com
Manxuan Technology	Battery Reconditioner	Baby received. Will follow up after using it for a while.	JD.com
Manxuan Technology	Battery Reconditioner	I received the restorer, and I'm using it now, I don't know how it works yet, if it works I'll recommend it to my friends.	JD.com
Manxuan Technology	Battery Reconditioner	Good use! Thank you guys[hugs]the courier worked hard[hugs]	JD.com
Manxuan Technology	Battery Reconditioner	Roger that. Can't tell how it's gonna work.	JD.com
Manxuan Technology	Battery Reconditioner	[Big Love]	JD.com
Manxuan Technology	Battery Reconditioner	Use it and talk about it.	JD.com
Manxuan Technology	Battery Reconditioner	Good courier service and well packaged. Good value for money. Give five stars praise!	JD.com
Manxuan Technology	Battery Reconditioner	The baby I bought has been received, thank you!	JD.com
Manxuan Technology	Battery Reconditioner	Packaging is intact, the courier service attitude is very good. The quality is good, the price is more affordable, it is worth recommending!	JD.com
Manxuan Technology	Battery Reconditioner	Packaging is intact, the courier service attitude is very good. The quality is good, the price is more affordable, it is worth recommending!	JD.com
Manxuan Technology	Battery Reconditioner	I haven't used it yet, I'll comment after I use it.	JD.com
Manxuan Technology	Battery Reconditioner	Things are received, do not know good use bad use, with a period of time to know oh. ? ? ? ? ? ? ?	JD.com
Manxuan Technology	Battery Reconditioner	don't know the effect yet. I'll see you in a few days	JD.com
Manxuan Technology	Battery Reconditioner	It doesn't work very well.	JD.com
Manxuan Technology	Battery Reconditioner	phone battery repairer received, very satisfied, good customer service, logistics is also very fast, good value for money, can extend the use of cell phone battery life, the ideal shopping experience. This must be recommended to the majority of customers.	JD.com
Manxuan Technology	Battery Reconditioner	Value for money.	JD.com
Manxuan Technology	Battery Reconditioner	Trying it out for a week before evaluating it	JD.com
Manxuan Technology	Battery Reconditioner	The item is very formal well made.	JD.com
Manxuan Technology	Battery Reconditioner	(of an unmarried couple) be close	JD.com
Manxuan Technology	Battery Reconditioner	(of an unmarried couple) be close	JD.com

Manxuan Technology	Battery Reconditioner	Well made, beautifully shaped, displays properly. Good review.	JD.com
Manxuan Technology	Battery Reconditioner	Packaging is very good, mecha spin. I don't know if it's fixable, I'll follow up after I use it.	JD.com
Manxuan Technology	Battery Reconditioner	This user did not fill in a rating	JD.com
Manxuan Technology	Battery Reconditioner	received	JD.com
Manxuan Technology	Battery Reconditioner	This user did not fill in a rating	JD.com
Manxuan Technology	Battery Reconditioner	Received the cell phone battery repairer, the cell phone has been used for years and charging started to be frequent, hopefully it will repair the battery.	JD.com
Manxuan Technology	Battery Reconditioner	Nice shape, normal display, okay	JD.com
Manxuan Technology	Battery Reconditioner	Very good, get it is not yet used, first give a favorable comment, wait for later use and then make up the comment!	JD.com
Manxuan Technology	Battery Reconditioner	I'm using it. I won't know the effect until I use it.	JD.com
Manxuan Technology	Battery Reconditioner	It's working well.	JD.com
Manxuan Technology	Battery Reconditioner	cell phone battery has protection and repair function, no longer need to worry about the cell phone battery standby short!	JD.com
Manxuan Technology	Battery Reconditioner	I'll follow up after using it for a while.	JD.com
Manxuan Technology	Battery Reconditioner	looking forward to fixing the battery, good comments satisfied.	JD.com
Manxuan Technology	Battery Reconditioner	Pro goods have been received thanks!	JD.com
Manxuan Technology	Battery Reconditioner	rare	JD.com
Manxuan Technology	Battery Reconditioner	Haven't used it yet, don't know how it is	JD.com
Manxuan Technology	Battery Reconditioner	's great to use the product is so simple and rough directly recommend this store owner future buyers friends and family to buy family said it's really good do not have to go out of the house great	JD.com
Manxuan Technology	Battery Reconditioner	I'll comment after using it.	JD.com
Manxuan Technology	Battery Reconditioner	It is possible to use	JD.com
Manxuan Technology	Battery Reconditioner	I'm using it now, hopefully it works, will follow up later.	JD.com
Manxuan Technology	Battery Reconditioner	favorable criticism	JD.com
Manxuan Technology	Battery Reconditioner	It doesn't feel like much!	JD.com
Manxuan Technology	Battery Reconditioner	review after use	JD.com
Manxuan Technology	Battery Reconditioner	I don't know if it's good or not, let's give it a good review and see how it goes	JD.com
Manxuan Technology	Battery Reconditioner	(of an unmarried couple) be close	JD.com
Manxuan Technology	Battery Reconditioner	好好 Good, good.	JD.com
Manxuan Technology	Battery Reconditioner	Great stuff, compact and easy to carry. I'll comment after I use it.	JD.com
Manxuan Technology	Battery Reconditioner	Good quality product looking forward to miracles with use Great customer service!	JD.com
Manxuan Technology	Battery Reconditioner	Cell phone repair device has received after using the follow-up evaluation	JD.com
Manxuan Technology	Battery Reconditioner	(of an unmarried couple) be close	JD.com
Manxuan Technology	Battery Reconditioner	Good, satisfied, good review.	JD.com
Manxuan Technology	Battery Reconditioner	You can't buy a cell phone where the information is easy to steal. Don't even know it yourself. Security is poor.	JD.com
Manxuan Technology	Battery Reconditioner	Very satisfied.	JD.com
Manxuan Technology	Battery Reconditioner	Solid material, good quality, very satisfied, worth buying.	JD.com

Manxuan Technology	Battery Reconditioner	It's okay.	JD.com
Manxuan Technology	Battery Reconditioner	Use it at a flat rate first 5 stars	JD.com
Manxuan Technology	Battery Reconditioner	Adaptation: I'm very happy with it pretty well[smile]	JD.com
Manxuan Technology	Battery Reconditioner	Just used it, will rate it after use	JD.com
Manxuan Technology	Battery Reconditioner	favorable criticism	JD.com
Manxuan Technology	Battery Reconditioner	The German Chip Fixer Supreme Edition has been received. Delivery is very timely. Packaging is very good. No damage on the way of delivery. If the effect is good, I will recommend it to my friends. Good logistics service.	JD.com
Manxuan Technology	Battery Reconditioner	It feels like an IQ tax.	JD.com
Manxuan Technology	Battery Reconditioner	It's so smart it's several times better than the regular one, and I'm really happy with it! Next group buy refueling!	JD.com
Manxuan Technology	Battery Reconditioner	It doesn't feel like it's working.	JD.com
Manxuan Technology	Battery Reconditioner	Fast logistics, just arrived, do not know how to operate on the phone!	JD.com
Manxuan Technology	Battery Reconditioner	favorable criticism	JD.com
Manxuan Technology	Battery Reconditioner	I don't feel much of an effect.	JD.com
Manxuan Technology	Battery Reconditioner	Doesn't work well, no feeling from charging the cell phone battery. Feels like I've been ripped off	JD.com
Manxuan Technology	Battery Reconditioner	Baby is good, love it, it's nice and nice.	JD.com
Manxuan Technology	Battery Reconditioner	I'm using it. I'll tell you if it's any good after a while.	JD.com
Manxuan Technology	Battery Reconditioner	I don't know if it's effective or not, I'll comment after using it	JD.com
Manxuan Technology	Battery Reconditioner	The use of the effect of waiting for time to test, for the time being praise	JD.com
Manxuan Technology	Battery Reconditioner	It didn't work after two uses.	JD.com
Manxuan Technology	Battery Reconditioner	I received the goods, it's okay!	JD.com
Manxuan Technology	Battery Reconditioner	I just got it, I don't know if it works well, I'll try it out and comment.	JD.com
Manxuan Technology	Battery Reconditioner	It works.	JD.com
Manxuan Technology	Battery Reconditioner	Works great, results to follow up after use!	JD.com
Manxuan Technology	Battery Reconditioner	Battery Repairer is great, great logistics, great value for money!	JD.com
Manxuan Technology	Battery Reconditioner	Still in use	JD.com
Manxuan Technology	Battery Reconditioner	Shipping was fast and the quality of the item was pretty good	JD.com
Manxuan Technology	Battery Reconditioner	After charging it a few times, the phone is much better, and it really does work	JD.com
Manxuan Technology	Battery Reconditioner	Exquisite workmanship, beautifully shaped, beautifully shaped	JD.com
Manxuan Technology	Battery Reconditioner	Expect a good restoration.	JD.com
Manxuan Technology	Battery Reconditioner	battery wasn't used much<br>The first charge with this wasn't noticeable The second charge was completed The battery has been dropping off very slowly It was fully charged the night before There are still41chargesleft It used to be that when I woke up overnight I'd lose almost20charges I don't know how it works It's amazing I swear I'm not a trustworthy person It's just a couple of bucks There's no need to lie and cheat people	JD.com
Manxuan Technology	Battery Reconditioner	Good price, fast delivery, praise	JD.com
Manxuan Technology	Battery Reconditioner	The battery retractor was received quickly and works very well, very good	JD.com
Manxuan Technology	Battery Reconditioner	Received, well packaged and satisfied.	JD.com
Manxuan Technology	Battery Reconditioner	Fast logistics, quality is okay	JD.com
Manxuan Technology	Battery Reconditioner	I've got it. It's good.	JD.com
Manxuan Technology	Battery Reconditioner	Exquisite workmanship	JD.com

Manxuan Technology	Battery Reconditioner	looks pretty good, and the restorer has a good face value. .	JD.com
Manxuan Technology	Battery Reconditioner	Baby received, fast shipping, well packaged, affordable price.	JD.com
Manxuan Technology	Battery Reconditioner	Cell phone repairer received, very good, I tried a few cell phone effect is very good, convenient and practical.	JD.com
Manxuan Technology	Battery Reconditioner	Cell phone battery repairer received, packaging is very good, logistics is very fast, how late to start using, temporarily invisible effect, first give five-star praise!	JD.com
Manxuan Technology	Battery Reconditioner	Goods received, complete packaging, appearance with the tide, inexpensive, easy to use, monitoring in place, value for money, looking forward to the effect.	JD.com
Manxuan Technology	Battery Reconditioner	The charger is very good, the logistics is very fast love this product use it a little before you say thank you.	JD.com
Manxuan Technology	Battery Reconditioner	Received on the use, plug in the digital has been changing, do not understand how to see, this battery repairer check the QR code is genuine, first use a period of time to see if there is no effect in the end to say.	JD.com
Manxuan Technology	Battery Reconditioner	Quite good! With digital display, you can ask the boss if you don't understand it	JD.com
Manxuan Technology	Battery Reconditioner	Cell phone charging speeds up	JD.com
Manxuan Technology	Battery Reconditioner	Charging speed: very good charging<br>Adaptability: very good<br>Efficiency: very effective	JD.com
Manxuan Technology	Battery Reconditioner	Fast shipping and great service!	JD.com
Manxuan Technology	Battery Reconditioner	Great to plug in and start fixing. Seller details the method. Adherence to the repair will be effective.	JD.com
Manxuan Technology	Battery Reconditioner	Exactly as described! Fast shipping and good quality! Worth repurchasing!	JD.com
Manxuan Technology	Battery Reconditioner	the second one I bought, it really works, I'm telling the truth!	JD.com
Manxuan Technology	Battery Reconditioner	phone charging a day can not charge, with this charging repair damaged battery, the phone is really full of electricity, quite good!	JD.com
Manxuan Technology	Battery Reconditioner	Fixer received, logistics fast, in use, can be	JD.com
Manxuan Technology	Battery Reconditioner	Very satisfied with the delivery received	JD.com
Manxuan Technology	Battery Reconditioner	Goods received, are two days, have not yet evaluated, quality can be, cheap, repair okay, trustworthy, welcome friends to buy this product. Directly give a five-star praise!	JD.com
Manxuan Technology	Battery Reconditioner	Goods to receive, logistics is very fast, use it to see.	JD.com
Manxuan Technology	Battery Reconditioner	Very good workmanship, beautiful appearance, good effect, good value for money	JD.com
Manxuan Technology	Battery Reconditioner	It's not working, IQ tax.	JD.com
Manxuan Technology	Battery Reconditioner	The goods have been received.	JD.com
Manxuan Technology	Battery Reconditioner	Goods received, quite fast, give a favorable comment, the use of the effect of the later review it	JD.com
Manxuan Technology	Battery Reconditioner	Use it and then come back to evaluate	JD.com
Manxuan Technology	Battery Reconditioner	Just received it, not sure how well it works.	JD.com
Manxuan Technology	Battery Reconditioner	The item was received and is very nice aesthetically pleasing to look at. It is very convenient to use.	JD.com
Manxuan Technology	Battery Reconditioner	(of an unmarried couple) be close	JD.com
Manxuan Technology	Battery Reconditioner	We'll see if it works.	JD.com
Manxuan Technology	Battery Reconditioner	Charge charge very well, quite satisfied, quite good, quite good, quite good, quite good.	JD.com
Manxuan Technology	Battery Reconditioner	This cell phone charging repairer fixes well!	JD.com
Manxuan Technology	Battery Reconditioner	It's good. It's good. [static] [static] [static] [static]	JD.com
Manxuan Technology	Battery Reconditioner	Logistics is quite fast, charged twice the feeling of the phone battery is longer than when not used, baby appearance design is more beautiful, quite good.	JD.com
Manxuan Technology	Battery Reconditioner	Nice, love it thanks to the merchant, thanks to the logistics.	JD.com
Manxuan Technology	Battery Reconditioner	Thequality of the goods is very good business service enthusiasm logistics is also very fast	JD.com

Manxuan Technology	Battery Reconditioner	Fast logistics, tight packaging, easy to use<br>Compatibility: good;<br>Safety: good<br>Adaptability: strong<br>Charging speed: faster	JD.com
Manxuan Technology	Battery Reconditioner	Goods received, not bad, do not know how to use a good next time to come back to patronize	JD.com
Manxuan Technology	Battery Reconditioner	The appearance is very handsome, exquisite workmanship, the boss attitude is also good,, small and lovely, the use of carrying are very convenient logistics is very fast.	JD.com
Manxuan Technology	Battery Reconditioner	I haven't used it yet, I don't know the effect, but I'll give it 5 stars first.	JD.com
Manxuan Technology	Battery Reconditioner	Very good battery restorer, good quality, nice style, worth buying!	JD.com
Manxuan Technology	Battery Reconditioner	[smilehope it will achieve the result[big love] [big love] [big love][big love	JD.com
Manxuan Technology	Battery Reconditioner	The goods are genuine and I am satisfied with the purchase!	JD.com
Manxuan Technology	Battery Reconditioner	phone with a dying battery, and after a few charges and discharges, it seems to be working a little bit, but it will take some time to test it out, so I'll follow up with a review then.	JD.com
Manxuan Technology	Battery Reconditioner	The texture is very good, whether it's good or not, we'll know after using it.	JD.com
Manxuan Technology	Battery Reconditioner	Normal display, nice shape, works fine	JD.com
Manxuan Technology	Battery Reconditioner	Logistics is fast, very responsible, just received the goods, in the test, have you? Yes, I can't say.	JD.com
Manxuan Technology	Battery Reconditioner	Immediately wired to charge the machine, you can see the charging voltage, current and charge to the power, several values are changing, the power value is increasing, should be useful, to be charged to see the results and then follow up.	JD.com
Manxuan Technology	Battery Reconditioner	I don't know if it's working[Smiles] [Smiles]	JD.com
Manxuan Technology	Battery Reconditioner	Received the goods immediately unpacked to check, and then tried, really good!	JD.com
Manxuan Technology	Battery Reconditioner	Very good, fast logistics, service is also thoughtful, the first time to start using, charging repair can be seen	JD.com
Manxuan Technology	Battery Reconditioner	Sturdy packaging at an affordable price	JD.com
Manxuan Technology	Battery Reconditioner	Good! Works very well! Great value for money! Logistics is fast! Recommended to buy!	JD.com
Manxuan Technology	Battery Reconditioner	The good ones are really good, they work, good reviews	JD.com
Manxuan Technology	Battery Reconditioner	I'll comment after I use it.	JD.com
Manxuan Technology	Battery Reconditioner	💗catching up withtime to do activities to buy, the price of a penny♡, from the outside looks very beautiful♡, the material workmanship is good💗, I feel that the purchase is worth it, and the logistics is super fast, point of praise!	JD.com
Manxuan Technology	Battery Reconditioner	Good review first, follow up later	JD.com
Manxuan Technology	Battery Reconditioner	Received the goods, good stuff, worth repurchase!	JD.com
Manxuan Technology	Battery Reconditioner	The goods have been received and are as described by the seller.	JD.com
Manxuan Technology	Battery Reconditioner	Charging speed: average, just46, now44<br>Efficiency: not good, wait for me to use it<br><br>Adaptability: this is very difficult to comment ah, the line is like a mountain<br><br>Safety: wait for me to give you guys to test the water ha	JD.com
Manxuan Technology	Battery Reconditioner	It's working.	JD.com
Manxuan Technology	Battery Reconditioner	This battery restorer is really good and recommended.	JD.com
Manxuan Technology	Battery Reconditioner	Real goods, fast logistics, good service, five-star praise!	JD.com
Manxuan Technology	Battery Reconditioner	I tried! No change in the battery! I've flushed it twice and it doesn't fix the battery! How do you do it?	JD.com
Manxuan Technology	Battery Reconditioner	Fast logistics, good packaging, next time!!!!	JD.com
Manxuan Technology	Battery Reconditioner	Not yet used, first look at the packaging is quite good	JD.com
Manxuan Technology	Battery Reconditioner	Let's use it.	JD.com

Manxuan Technology	Battery Reconditioner	The shape is very cool, the appearance looks good, the quality should be good, charging can not repair the battery that have to wait to see yo	JD.com
Manxuan Technology	Battery Reconditioner	I used to buy them from physical stores, but this time I chose to buy them online, not only are they authentic, but they are also affordable!	JD.com
Manxuan Technology	Battery Reconditioner	effect is good, customer service is in place, also very satisfied, praise.	JD.com
Manxuan Technology	Battery Reconditioner	This is a very handy restorer with a nice refined look, I'll comment again when I use it!	JD.com
Manxuan Technology	Battery Reconditioner	It looks cool, not sure if it works try picking it.	JD.com
Manxuan Technology	Battery Reconditioner	It's great, it's easy, it's a great experience	JD.com
Manxuan Technology	Battery Reconditioner	business service is thoughtful, the appearance of good design, convenient and practical, tried, the effect is good, it can monitor the current, voltage and so on, it is worth recommending.	JD.com
Manxuan Technology	Battery Reconditioner	Goods have been received, the packaging is intact, logistics and courier service is good, cost-effective, worth buying, very satisfied!	JD.com
Manxuan Technology	Battery Reconditioner	It works very well.[Smiles]	JD.com
Manxuan Technology	Battery Reconditioner	pointless	JD.com
Manxuan Technology	Battery Reconditioner	Logistics is very fast, the packaging is very good, see the reviews say good use to buy a to try to see how the effect, good use and then buy back!	JD.com
Manxuan Technology	Battery Reconditioner	with a use to know, packaging is very good, very pleasant shopping.	JD.com
Manxuan Technology	Battery Reconditioner	Goods have been received, the use of very good, fast logistics security. Good quality and price. Very good.	JD.com
Manxuan Technology	Battery Reconditioner	It's not bad to use, cheap and affordable.	JD.com
Manxuan Technology	Battery Reconditioner	Logistics is very fast product looks pretty good, read a lot of reviews before buying this I hope it works.	JD.com
Manxuan Technology	Battery Reconditioner	I have used it, and I am looking forward to good results, as good as described!	JD.com
Manxuan Technology	Battery Reconditioner	Try it all night while you sleep at night. It's easy to carry and pretty nice.	JD.com
Manxuan Technology	Battery Reconditioner	Received the cell phone repairer sent to feel good logistics is also good!	JD.com
Manxuan Technology	Battery Reconditioner	just got it I'll try it out first It looks good	JD.com
Manxuan Technology	Battery Reconditioner	It's not effective, it's not useful, it's just a charging device.	JD.com
Manxuan Technology	Battery Reconditioner	It works, the charger charges fast! Value!	JD.com
Manxuan Technology	Battery Reconditioner	Good quality and low price, battery repair to increase standby time, good comments.	JD.com
Manxuan Technology	Battery Reconditioner	time to buy such expensive things from the Internet, began to be very apprehensive, really afraid of problems, after receiving the goods, really relieved, with the present really good👌	JD.com
Manxuan Technology	Battery Reconditioner	Packaging is very good, logistics is also fast, the shape is exquisite, fine workmanship, compact and applicable, inexpensive and beautiful.	JD.com
Manxuan Technology	Battery Reconditioner	Let's hope it works.	JD.com
Manxuan Technology	Battery Reconditioner	Received: looks good, doesn't work, will judge after use.	JD.com
Manxuan Technology	Battery Reconditioner	Works great Will be back	JD.com
Manxuan Technology	Battery Reconditioner	It doesn't work.	JD.com
Manxuan Technology	Battery Reconditioner	Battery type: first use to see, the baby has received very satisfied, the courier is also strong, very fast, there is a genuine sense of Tongxiao thank you store!	JD.com
Manxuan Technology	Battery Reconditioner	It's so good.	JD.com
Manxuan Technology	Battery Reconditioner	dissatisfied	JD.com
Manxuan Technology	Battery Reconditioner	Received it, looking forward to the results, will recommend to friends if it works well.	JD.com
Manxuan Technology	Battery Reconditioner	Goods and online description of the same, shipping and logistics is very fast, beautifully packaged, with instruction manuals, anti-counterfeiting code, the main function is to repair the rechargeable battery with pulse current.	JD.com

Manxuan Technology	Battery Reconditioner	The effect doesn't feel like a power regulator yet, it feels more like a power regulator.	JD.com
Manxuan Technology	Battery Reconditioner	It doesn't work with the cable head. It's an IQ tax.	JD.com
Manxuan Technology	Battery Reconditioner	First time using it, can't see any effect yet	JD.com
Manxuan Technology	Battery Reconditioner	The replacement has been received, thank you! Customer service attitude is very good, know the bad, rushed to send a replacement, will introduce friends to come.	JD.com
Manxuan Technology	Battery Reconditioner	very happy	JD.com
Manxuan Technology	Battery Reconditioner	serviceable	JD.com
Manxuan Technology	Battery Reconditioner	Received, well-packed, logistics is fast, see the effect in the follow-up comment!	JD.com
Manxuan Technology	Battery Reconditioner	It's working a little bit.	JD.com
Manxuan Technology	Battery Reconditioner	I hope it works, it's good quality and displays properly	JD.com
Manxuan Technology	Battery Reconditioner	After using 90% repair ability, good product, worth five-star praise, logistics and fast!	JD.com
Manxuan Technology	Battery Reconditioner	Repair device has received, installed on the charging cell phone to try to see the effect, first praise. Thanks to the merchants and logistics companies.	JD.com
Manxuan Technology	Battery Reconditioner	Goods have been received, the quality is very good, will buy again next time	JD.com
Manxuan Technology	Battery Reconditioner	Not bad I guess, trying it out at the moment.	JD.com
Manxuan Technology	Battery Reconditioner	Fast shipping and great results!	JD.com
Manxuan Technology	Battery Reconditioner	Goods have been received, logistics is very fast, the quality of the products of this store is quite good, do not know how to use the boss and customer service, but also very polite to explain to us to buy pro, hurry to order, praise five stars!	JD.com
Manxuan Technology	Battery Reconditioner	dissatisfied	JD.com
Manxuan Technology	Battery Reconditioner	This user feels the product is better	JD.com
Manxuan Technology	Battery Reconditioner	Exquisite workmanship, high value, so much better than similar products!	JD.com
Manxuan Technology	Battery Reconditioner	service is thoughtful, the appearance of the design is very good, convenient and practical, charging to try to see how the effect?	JD.com
Manxuan Technology	Battery Reconditioner	good	JD.com
Manxuan Technology	Battery Reconditioner	favorable criticism	JD.com
Manxuan Technology	Battery Reconditioner	The service is too good and the logistics is fast. The appearance of the product is the same as the description.	JD.com
Manxuan Technology	Battery Reconditioner	The item is very good and nice, I like it very much, the shape is cool like a sports car, will buy back, the	JD.com
Manxuan Technology	Battery Reconditioner	Good review.	JD.com
Manxuan Technology	Battery Reconditioner	Logistics is very fast, packaging is very good, use it and then comment!	JD.com
Manxuan Technology	Battery Reconditioner	Logistics is very fast, the packaging is also solid, the manual is complete and detailed, used once at night as instructed, the plug is solid, science, this morning with the phone has a smooth feeling, the	JD.com
Manxuan Technology	Battery Reconditioner	Fast shipping and good packaging, battery restorer after use and then follow up first give a positive feedback, the courier to the home in a timely manner, praiseworthy.	JD.com
Manxuan Technology	Battery Reconditioner	Received and things look good. Product is well manufactured.	JD.com
Manxuan Technology	Battery Reconditioner	Very good👍	JD.com
Manxuan Technology	Battery Reconditioner	Genuine, good quality	JD.com
Manxuan Technology	Battery Reconditioner	Logistics is very fast, looks very good, use and then comment!	JD.com
Manxuan Technology	Battery Reconditioner	Baby has received, very satisfied, logistics, express delivery is also strong, very fast, there are genuine Dongcheng, thanks to the store.	JD.com
Manxuan Technology	Battery Reconditioner	Evaluate after use	JD.com
Manxuan Technology	Battery Reconditioner	Goods have been received, the product is well packaged, .The price is beautiful.	JD.com
Manxuan Technology	Battery Reconditioner	I haven't used it yet.	JD.com

Manxuan Technology	Battery Reconditioner	The baby received, tried to feel good seems to be a little 効果, after a few weeks of use and then review it!	JD.com
Manxuan Technology	Battery Reconditioner	Good quality, effective, fast logistics.	JD.com
Manxuan Technology	Battery Reconditioner	service attitude is very good👌logistics is very fast five-star praise.	JD.com
Manxuan Technology	Battery Reconditioner	Purchase received, quite good	JD.com
Manxuan Technology	Battery Reconditioner	It's good. It's good.	JD.com
Manxuan Technology	Battery Reconditioner	I received the baby and tried it immediately, it looks great and I'm looking forward to good results!	JD.com
Manxuan Technology	Battery Reconditioner	Received the goods have not yet used it	JD.com
Manxuan Technology	Battery Reconditioner	I got it.	JD.com
Manxuan Technology	Battery Reconditioner	Matches the description, good quality	JD.com
Manxuan Technology	Battery Reconditioner	The goods have been received and feel quite good.	JD.com
Manxuan Technology	Battery Reconditioner	Use it over to rate it.	JD.com
Manxuan Technology	Battery Reconditioner	[LOVE] [LOVE]Could be	JD.com
Manxuan Technology	Battery Reconditioner	Time will tell if it works.	JD.com
Manxuan Technology	Battery Reconditioner	The cell phone repairer has been received fast logistics.	JD.com
Manxuan Technology	Battery Reconditioner	good price. Use two days to test. Not bad. Give five stars to praise.	JD.com
Manxuan Technology	Battery Reconditioner	Android Battery Repairer received, very perfect, rationalization of the price, simple to use, prolong the aging of the battery and can deep clean the garbage generated by the use, in short, many benefits!	JD.com
Manxuan Technology	Battery Reconditioner	Copy that.	JD.com
Manxuan Technology	Battery Reconditioner	Battery type: currently have begun to use the results in the end how to feedback later, the overall feeling is okay	JD.com
Manxuan Technology	Battery Reconditioner	in the future will continue to patronize the purchase of thank you!	JD.com
Manxuan Technology	Battery Reconditioner	This fixer is real and works great, I've tried it on three cell phones with good results! Even better it monitors current, voltage, etc! I like very much satisfied!	JD.com
Manxuan Technology	Battery Reconditioner	It's really cool.'s okay if you use it.4~5stars.	JD.com
Manxuan Technology	Battery Reconditioner	Cell phone repair battery received, and the business description of the same, fast shipping worth buying	JD.com
Manxuan Technology	Battery Reconditioner	Just received, not quite know how to use, contacted the store, immediately explained. Good service!	JD.com
Manxuan Technology	Battery Reconditioner	Baby received, very like very satisfied	JD.com
Manxuan Technology	Battery Reconditioner	Change did not use later evaluation, business courier are very powerful! Five-star praise!	JD.com
Manxuan Technology	Battery Reconditioner	an excellent	JD.com
Manxuan Technology	Battery Reconditioner	Matches the description, good quality, awesome!	JD.com
Manxuan Technology	Battery Reconditioner	Baby received, in kind and the same as the picture, the quality is good, very satisfied with a shopping, logistics is also very strong Oh!	JD.com
Manxuan Technology	Battery Reconditioner	The product is well made with it to charge the phone is very smooth and does not lag the original phone has not worked for many years now a lot better but I do not know how to use nothing to get to understand now feel good to use first to give a good review thanks to the manufacturer and the store!	JD.com
Manxuan Technology	Battery Reconditioner	Don't be fooled. It's useless. It's a fake.	JD.com
Manxuan Technology	Battery Reconditioner	This user did not fill in a rating	JD.com
Manxuan Technology	Battery Reconditioner	Very good, worth buying Oh, thanks for such a good product[smile]smile[smile]smilesmile[smile] [smile] [smile] [smile] [smile] [smile] [smile] [smile] [smile] [smile] [smile] [smile] [smile] [smile] [smile] [smile] [smile] [smile][smile]	JD.com
Manxuan Technology	Battery Reconditioner	This user feels the product is better	JD.com
Ling's Goodies Preferred	Battery Reconditioner	There are more similar products on the Internet, compared to the feeling of bias in favor of this one, more detailed workmanship, tight sockets, more like.	JD.com

Ling's Goodies Preferred	Battery Reconditioner	Design: good Quality: good Ease of handling: good Compatibility: good Charging speed: good Interface: good Appearance: good Portability: good	JD.com
Ling's Goodies Preferred	Battery Reconditioner	as described, but also particularly good, business service attitude is very good, is my favorite, please Friends assured purchase!	JD.com
Ling's Goodies Preferred	Battery Reconditioner	This restorer works very well and I especially like it. Low cost, high efficiency, beautiful design, friends also praise👍, everyone said good, I am also particularly happy, please friends feel free to buy it!	JD.com
Ling's Goodies Preferred	Battery Reconditioner	It's not good at all, the picture is nothing like the item	JD.com
Ling's Goodies Preferred	Battery Reconditioner	It's good quality and works great.	JD.com
Ling's Goodies Preferred	Battery Reconditioner	Fast shipping, fast logistics and great service!	JD.com
Ling's Goodies Preferred	Battery Reconditioner	can	JD.com
Ling's Goodies Preferred	Battery Reconditioner	C0	JD.com
Ling's Goodies Preferred	Battery Reconditioner	(of an unmarried couple) be close	JD.com
Ling's Goodies Preferred	Battery Reconditioner	Good service from the owner Great stuff	JD.com
Ling's Goodies Preferred	Battery Reconditioner	Small goods received, logistics is very fast, well-packed, good service, small goods fine workmanship, the appearance is very beautiful, first buy a try, will come back to buy.	JD.com
Ling's Goodies Preferred	Battery Reconditioner	Small goods received, logistics is very fast, well-packed, good service, small goods fine workmanship, the appearance is very beautiful, first buy a try, will come back to buy.	JD.com
Ling's Goodies Preferred	Battery Reconditioner	charger quality is very good, according to the product information according to the installation of the use of ease of use, cell phone electricity he repair effect is good, the price is affordable.	JD.com
Ling's Goodies Preferred	Battery Reconditioner	Shipped very quickly, well packaged, the battery with a long time, would like to change the battery, saw this on the Internet, hold to try, anyway, is not expensive, back to use, before the phone charge, the battery is hot, this time no more, I hope to be able to use a longer period of time.	JD.com
Ling's Goodies Preferred	Battery Reconditioner	Poundland.comis so good. Fast shipping from the manufacturer. The quality is very good. The service is attentive. I am very satisfied. The price is reasonable. Superb! Give a rating of 5 stars!👍	JD.com
Ling's Goodies Preferred	Battery Reconditioner	Charge Time: Receipt has been confirmed. Prompt shipping, well packaged, and excellent service. Very satisfied with the initial trial, after charging and repairing, the original battery heat is weakened, and the power is sufficient.	JD.com
Ling's Goodies Preferred	Battery Reconditioner	Cell phone repairer is small and exquisite, multi-functional, novel design, easy to operate, business enthusiasm, fast logistics. Very like, worth buying, need to play the old iron people in order to repair their own cell phone battery and the complete removal of garbage to buy a bar.	JD.com
Ling's Goodies Preferred	Battery Reconditioner	Very good, authentic, good quality, small and cute! Very convenient,the effect is awesome.	JD.com
Ling's Goodies Preferred	Battery Reconditioner	Baby has received a good packaging logistics fast, order the next day to receive the goods! The function is very good👌easy to operate. Very good👍phone becomes new thanks🙏boss	JD.com
Ling's Goodies Preferred	Battery Reconditioner	This user feels the product is better	JD.com
Ling's Goodies Preferred	Battery Reconditioner	Good quality and very easy to use. Little things work great.	JD.com
Ling's Goodies Preferred	Battery Reconditioner	Goods received, good business service, fast logistics, satisfied.	JD.com
Ling's Goodies Preferred	Battery Reconditioner	operation, easy to use, repairer charging stable, clear display,, on the old battery has a certain repair function,, it is very good!	JD.com
Ling's Goodies Preferred	Battery Reconditioner	The quality is very good, make also quite convenient, charging effect is also very good, the courier boy service is also quite in place, next time I also buy back!	JD.com
Ling's Goodies Preferred	Battery Reconditioner	Express just received can't wait to open, plug in the phone is indeed very good, the instructions for the operation is also written in detail, looking forward to fixing the phone sooner!	JD.com
Ling's Goodies Preferred	Battery Reconditioner	This user feels the product is better	JD.com
Ling's Goodies Preferred	Battery Reconditioner	This user feels the product is better	JD.com
Ling's Goodies Preferred	Battery Reconditioner	This user feels the product is better	JD.com

Ling's Goodies Preferred	Battery Reconditioner	Cell phone repairer received, small and exquisite, with screen display quality is good, charging while repairing really good, tried it is very satisfied.	JD.com
Ling's Goodies Preferred	Battery Reconditioner	Received the cell phone adapter am using it hopefully with good results	JD.com
Ling's Goodies Preferred	Battery Reconditioner	classifier for objects in rows such as words	JD.com
Ling's Goodies Preferred	Battery Reconditioner	This user feels the product is better	JD.com
Ling's Goodies Preferred	Battery Reconditioner	This user feels the product is better	JD.com
Ling's Goodies Preferred	Battery Reconditioner	Parts have been received Logistics is fast Have not officially used Not sure if it is good Kudos first[kudos]	JD.com
Ling's Goodies Preferred	Battery Reconditioner	Nice item and fast flushing all 5 stars!	JD.com
Ling's Goodies Preferred	Battery Reconditioner	It feels good so far!	JD.com
Ling's Goodies Preferred	Battery Reconditioner	This user feels the product is better	JD.com
Ling's Goodies Preferred	Battery Reconditioner	also very fast, the business attitude is very good, five-star praise.	JD.com
Ling's Goodies Preferred	Battery Reconditioner	Received the goods very quickly and are already starting to use them.	JD.com
Ling's Goodies Preferred	Battery Reconditioner	I'll add a comment after I use it.	JD.com
Ling's Goodies Preferred	Battery Reconditioner	Item has been received in perfect packaging.	JD.com
Ling's Goodies Preferred	Battery Reconditioner	Express received, thank you boss, logistics is very fast, the business service attitude is good[kudos]courier brother hard work[kudos], to the business five-star praise[kudos]	JD.com
Ling's Goodies Preferred	Battery Reconditioner	The quality of this item is good, customer service is very good, give no stars, logistics is fast, the goods are not damaged, give, five stars!	JD.com
Ling's Goodies Preferred	Battery Reconditioner	Receive the baby and use it to evaluate	JD.com
Ling's Goodies Preferred	Battery Reconditioner	This user feels the product is better	JD.com
Ling's Goodies Preferred	Battery Reconditioner	Good quality!	JD.com
Ling's Goodies Preferred	Battery Reconditioner	Little fixer received, inexpensive, easy to use, plug and play, small things to solve big problems, the shell is thin, the shell snap is not very tight.[Kudos][Rose]	JD.com
Ling's Goodies Preferred	Battery Reconditioner	The goods have been received and the delivery was fast.	JD.com
Ling's Goodies Preferred	Battery Reconditioner	This item works well, the phone really doesn't get stuck anymore, and it takes a long time to charge once about!	JD.com
Ling's Goodies Preferred	Battery Reconditioner	self-acceptance	JD.com
Ling's Goodies Preferred	Battery Reconditioner	Has received, product quality is good, the store is very enthusiastic! The use of the effect to be tried and then make a follow-up comment!	JD.com
Ling's Goodies Preferred	Battery Reconditioner	Incoming goods received has been more than favorable comments, try and then follow up.	JD.com
Ling's Goodies Preferred	Battery Reconditioner	It doesn't work. It doesn't work.	JD.com
Ling's Goodies Preferred	Battery Reconditioner	This cell phone adapter works really well and will repurchase.	JD.com
Ling's Goodies Preferred	Battery Reconditioner	Good, that's great.	JD.com
Ling's Goodies Preferred	Battery Reconditioner	The adapter has been received, tried a few days feel good, businessmen shipping in a timely manner is very satisfied, five-star praise!	JD.com
Ling's Goodies Preferred	Battery Reconditioner	Received probably used, the effect is not yet obvious out, wait a few days to see, it must be good, good value for money.	JD.com
Ling's Goodies Preferred	Battery Reconditioner	quick fix	JD.com
Ling's Goodies Preferred	Battery Reconditioner	Got the cell phone adapter. Nice. Thanks.	JD.com
Ling's Goodies Preferred	Battery Reconditioner	This user feels the product is better	JD.com
Ling's Goodies Preferred	Battery Reconditioner	Fixed it2days and it's still the same.	JD.com
Ling's Goodies Preferred	Battery Reconditioner	The quality of the goods is very good I am very satisfied	JD.com

Ling's Goodies Preferred	Battery Reconditioner	Good service from the merchant! Good attitude! Courier delivery. Thank you ~ multifunctional cell phone battery adapter, clean up cell phone memory, make your cell phone memory, restore factory settings, suburb fruit more obvious, cell phone memory can be cleaned up, it is very good to use.	JD.com
Ling's Goodies Preferred	Battery Reconditioner	Compact and easy to carry. Convenient to fix the cell phone. Use under power can see the finger亓 light on. You can use the cell phone without limitations.	JD.com
Ling's Goodies Preferred	Battery Reconditioner	fine workmanship, easy to use, affordable, fast delivery, after using, additional evaluation. Give full five-star praise!	JD.com
Ling's Goodies Preferred	Battery Reconditioner	Baby phone adapter received, the color is my favorite good-looking, small and exquisite, fast charging.	JD.com
Ling's Goodies Preferred	Battery Reconditioner	effect is very good, after repairing the phone speed to improve much, like!	JD.com
Ling's Goodies Preferred	Battery Reconditioner	Cell phone adapter received, clean up the phone memory is very good to use, the original cell phone is too stuck, through this adapter after charging gui in the cell phone network number of fast and not stuck, value for money, satisfied with the shopping, fast delivery force!	JD.com
Ling's Goodies Preferred	Battery Reconditioner	The product is very good, the second time to buy back, recommended friends, used up to buy again, customer service attitude is very good!	JD.com
Ling's Goodies Preferred	Battery Reconditioner	commenting. Feels great. The phone doesn't get hot when charging, and it doesn't get hot when watching videos, and it doesn't skip stations. Trustworthy, buy with confidence. Good customer service, fast shipping, complete packaging, no damage.	JD.com
Ling's Goodies Preferred	Battery Reconditioner	In-kind and in line with the description, fast shipping, fast logistics, the boss has a good service attitude, the quality of the product can repair the battery, extend the battery life so that the phone becomes the same as the new machine, the need to rest assured that the courage to snatch.	JD.com
Ling's Goodies Preferred	Battery Reconditioner	This item is great for repairing cell phone batteries and gets straight 5 stars.	JD.com
Ling's Goodies Preferred	Battery Reconditioner	The goods have been received, complete packaging. Logistics is fast.	JD.com
Ling's Goodies Preferred	Battery Reconditioner	The item has been received and is good value for money. Though the thing is small, the role is great. Can be a good solution to the ills of the phone, such as running speed, cell phone lag, battery life, cell phone junk, etc., fast also quickly, worth everyone to buy!	JD.com
Ling's Goodies Preferred	Battery Reconditioner	Fast shipping and logistics. Packaging is very good. Received plugged in and used up, as if a little effect, charging is also a little faster, in use, later review it.	JD.com
Ling's Goodies Preferred	Battery Reconditioner	Battery repair is effective, my original cell phone went out for a day the battery is not enough to use, now go out, come back there are50power, recommend everyone rest assured to buy	JD.com
Ling's Goodies Preferred	Battery Reconditioner	repairer is well packaged. Logistics is fast. The cell phone adapter is made with fine craftsmanship. The use of the party invasion. How its effect, used to know. Good value for money. Worth buying. ', 1	JD.com
Ling's Goodies Preferred	Battery Reconditioner	The cell phone charging adapter has been received, charged and used in the morning, felt a little faster than before. Five ♥ praise to give encouragement!	JD.com
Ling's Goodies Preferred	Battery Reconditioner	not have	JD.com
promise that everything will turn out well	Battery Reconditioner	The quality is terrible. It's useless.	JD.com
promise that everything will turn out well	Battery Reconditioner	useless	JD.com
promise that everything will turn out well	Battery Reconditioner	I don't know if it works.	JD.com
promise that everything will turn out well	Battery Reconditioner	Baby has been received, the quality is very good, very satisfied. The price is cheap and reasonable and practical service is first-class, very thoughtful. Logistics is also very fast, only have to buy.	JD.com
promise that everything will turn out well	Battery Reconditioner	Repairer received, very good, fast charging, and then use a period of time to see what the effect of repairing the battery and then follow up......	JD.com
promise that everything will turn out well	Battery Reconditioner	This user feels the product is better	JD.com
promise that everything will turn out well	Battery Reconditioner	This user feels the product is better	JD.com

promise that everything will turn out well	Battery Reconditioner	Repairer received, not yet used it, and so on good use will follow up the evaluation	JD.com
promise that everything will turn out well	Battery Reconditioner	I got the stuff. It's good. It's really good.	JD.com
promise that everything will turn out well	Battery Reconditioner	The goods have been received,,, affordable, charging especially fast, please feel free to buy.	JD.com
promise that everything will turn out well	Battery Reconditioner	It doesn't do much good.	JD.com
promise that everything will turn out well	Battery Reconditioner	What the internet says is all bragging, not as good as the internet says and it gets hot and the phone can't stand it.	JD.com
promise that everything will turn out well	Battery Reconditioner	Not sure if the product is good or bad	JD.com
promise that everything will turn out well	Battery Reconditioner	This user feels the product is better	JD.com
promise that everything will turn out well	Battery Reconditioner	unavailable	JD.com
promise that everything will turn out well	Battery Reconditioner	dissatisfied	JD.com
promise that everything will turn out well	Battery Reconditioner	****Whoever buys it will be fooled	JD.com
promise that everything will turn out well	Battery Reconditioner	This user feels the product is better	JD.com
promise that everything will turn out well	Battery Reconditioner	Received, good, fast logistics, easy to use, worth having, all five stars!	JD.com
promise that everything will turn out well	Battery Reconditioner	This user feels the product is better	JD.com
promise that everything will turn out well	Battery Reconditioner	I used it as soon as I received it, and itlooksOK	JD.com
promise that everything will turn out well	Battery Reconditioner	It doesn't look sturdy at all. The switch is broken.	JD.com
promise that everything will turn out well	Battery Reconditioner	This user feels the product is better	JD.com
promise that everything will turn out well	Battery Reconditioner	It's just a light.	JD.com
promise that everything will turn out well	Battery Reconditioner	It's not good. It's not good at all.	JD.com
promise that everything will turn out well	Battery Reconditioner	Goods have been received, good business service , good quality	JD.com
promise that everything will turn out well	Battery Reconditioner	Used to feel the effect gave five stars now full use of the phone a day still50percent ofthe battery feel more durable than before	JD.com
promise that everything will turn out well	Battery Reconditioner	IQ tax	JD.com
promise that everything will turn out well	Battery Reconditioner	very happy	JD.com
promise that everything will turn out well	Battery Reconditioner	Charging overnight doesn't matter, this multi-functional cell phone battery adapter it's a scam!	JD.com
promise that everything will turn out well	Battery Reconditioner	Received just tried it, effect to be followed up.	JD.com
promise that everything will turn out well	Battery Reconditioner	Compact and lightweight, cheap and affordable. Cell phone power consumption, preferred must-have. The logistics is very fast, the service is in place!	JD.com
promise that everything will turn out well	Battery Reconditioner	This user feels the product is better	JD.com

promise that everything will turn out well	Battery Reconditioner	an excellent	JD.com
promise that everything will turn out well	Battery Reconditioner	This user feels the product is better	JD.com
promise that everything will turn out well	Battery Reconditioner	It works great. It's inexpensive.	JD.com
promise that everything will turn out well	Battery Reconditioner	Goods received, quite good.	JD.com
promise that everything will turn out well	Battery Reconditioner	The logistics is particularly fast, and the charging is also particularly fast,, it really works!	JD.com
promise that everything will turn out well	Battery Reconditioner	very good	JD.com
promise that everything will turn out well	Battery Reconditioner	I got the stuff and it's okay now I'm starting to use it I think it's good too	JD.com
promise that everything will turn out well	Battery Reconditioner	That's good!	JD.com
promise that everything will turn out well	Battery Reconditioner	Goods received, the boss gave you manufacturers evaluation, good use, thank you very much, the boss	JD.com
promise that everything will turn out well	Battery Reconditioner	This user feels the product is better	JD.com
promise that everything will turn out well	Battery Reconditioner	This user feels the product is better	JD.com
promise that everything will turn out well	Battery Reconditioner	It's awesome, very useful, worth buying and coming back next time.	JD.com
promise that everything will turn out well	Battery Reconditioner	Good value for money and fast logistics.	JD.com
promise that everything will turn out well	Battery Reconditioner	evaluate, the original my cell phone battery can not use a day, I tried now can use a day and a half it, really good then give five stars!	JD.com
promise that everything will turn out well	Battery Reconditioner	This user feels the product is better	JD.com
promise that everything will turn out well	Battery Reconditioner	charging slow, power consumption is too fast problem, I hope to help improve, just received yet not used wait for the use of good words will be back to buy and recommend friends to[kudos][kudos][kudos]	JD.com
promise that everything will turn out well	Battery Reconditioner	This user feels the product is better	JD.com
promise that everything will turn out well	Battery Reconditioner	No effect, totally skinny product, not working at all on charging it up	JD.com
promise that everything will turn out well	Battery Reconditioner	This user feels the product is better	JD.com
promise that everything will turn out well	Battery Reconditioner	This user feels the product is better	JD.com
promise that everything will turn out well	Battery Reconditioner	We'll see.	JD.com
promise that everything will turn out well	Battery Reconditioner	The baby has received not yet used to use the good and then follow up reviews	JD.com
promise that everything will turn out well	Battery Reconditioner	Affordable, easy to use, baby works pretty well, the bag is intact	JD.com
promise that everything will turn out well	Battery Reconditioner	fraudulent	JD.com
promise that everything will turn out well	Battery Reconditioner	This user feels the product is better	JD.com
promise that everything will turn out well	Battery Reconditioner	Baby, received, is using, first give 5 stars good	JD.com

promise that everything will turn out well	Battery Reconditioner	to use, with results in the follow-up.	JD.com
promise that everything will turn out well	Battery Reconditioner	This user feels the product is better	JD.com
promise that everything will turn out well	Battery Reconditioner	This user feels the product is better	JD.com
promise that everything will turn out well	Battery Reconditioner	Small products are quite good to apply, recognize the product, the business service attitude is good, give praise	JD.com
promise that everything will turn out well	Battery Reconditioner	It's just a charging plug that doesn't work at all. Doesn't work.	JD.com
promise that everything will turn out well	Battery Reconditioner	Received the goods to give five-star praise	JD.com
promise that everything will turn out well	Battery Reconditioner	Very good quality worth having	JD.com
promise that everything will turn out well	Battery Reconditioner	unsatisfactory result	JD.com
promise that everything will turn out well	Battery Reconditioner	This user feels the product is better	JD.com
promise that everything will turn out well	Battery Reconditioner	Already received, very good workmanship	JD.com
promise that everything will turn out well	Battery Reconditioner	This item works well and is very popular, thanks!	JD.com
promise that everything will turn out well	Battery Reconditioner	It doesn't feel very useful. Don't be silly.	JD.com
promise that everything will turn out well	Battery Reconditioner	This user feels the product is better	JD.com
promise that everything will turn out well	Battery Reconditioner	This user feels the product is better	JD.com
promise that everything will turn out well	Battery Reconditioner	Not yet used! I'll comment after I use it.	JD.com
promise that everything will turn out well	Battery Reconditioner	Item received. Merchant shipped quickly, logistics company fast all five stars.	JD.com
promise that everything will turn out well	Battery Reconditioner	Baby received, packaging is very good, logistics is also very fast, the	JD.com
promise that everything will turn out well	Battery Reconditioner	t know how well it works but's "affordable	JD.com
promise that everything will turn out well	Battery Reconditioner	The product is used, the phone consumes more power than before, and the battery is not as durable as before	JD.com
promise that everything will turn out well	Battery Reconditioner	The goods have been received, thanks to the store business is prosperous,[smile]	JD.com
promise that everything will turn out well	Battery Reconditioner	The plug is bad and doesn't work.	JD.com
promise that everything will turn out well	Battery Reconditioner	Good value for money cheap price, and then what honesty, business conscience[smile]	JD.com
promise that everything will turn out well	Battery Reconditioner	Good review!	JD.com
promise that everything will turn out well	Battery Reconditioner	This user feels the product is better	JD.com
promise that everything will turn out well	Battery Reconditioner	It's not good, but it's all in the repair, and the two times I used it it didn't work, so I don't know if I'm scamming my customers?	JD.com
promise that everything will turn out well	Battery Reconditioner	Satisfied, five stars for the review. Very good. Effects to be seen in a few days.	JD.com

promise that everything will turn out well	Battery Reconditioner	Arrived quickly, good business service, just started using it, should help with the phone	JD.com
promise that everything will turn out well	Battery Reconditioner	Fast logistics, good quality, practical, satisfied.	JD.com
promise that everything will turn out well	Battery Reconditioner	The price is right, the logistics is fast, the customer service is good, and it works well.	JD.com
promise that everything will turn out well	Battery Reconditioner	It works great.	JD.com
promise that everything will turn out well	Battery Reconditioner	Good use, fast logistics, very affordable, recommended purchase	JD.com
promise that everything will turn out well	Battery Reconditioner	The effect is too inconspicuous	JD.com
promise that everything will turn out well	Battery Reconditioner	Goods have been received, logistics is fast, packaging fine, very satisfied with the	JD.com
promise that everything will turn out well	Battery Reconditioner	Baby is pretty good, not expensive, worth buying, will buy back.	JD.com
promise that everything will turn out well	Battery Reconditioner	Good quality product love this product	JD.com
promise that everything will turn out well	Battery Reconditioner	It doesn't work at all. It's very bad.	JD.com
promise that everything will turn out well	Battery Reconditioner	This is a fake inside the repair is affixed does not work at all, put on the start is also charging, off is also charging	JD.com
promise that everything will turn out well	Battery Reconditioner	The baby came quickly, works well, and glows on its own, which is nice.	JD.com
promise that everything will turn out well	Battery Reconditioner	Cell phone battery repairer received, very easy to use, according to the instructions have to charge a few more times.	JD.com
promise that everything will turn out well	Battery Reconditioner	This user feels the product is better	JD.com
promise that everything will turn out well	Battery Reconditioner	Use it and then evaluate.	JD.com
promise that everything will turn out well	Battery Reconditioner	receivable	JD.com
promise that everything will turn out well	Battery Reconditioner	well, good and like it, give you praise[praise][praise[praise[praise[praise] [praise] [praise] [praise] [praise] [praise] [praise] [praise] [praise] [praise] [praise] [praise] [praise] [praise][praise] [praise] [praise] [praise]	JD.com
promise that everything will turn out well	Battery Reconditioner	General, if the best in the post a look at that restoration of that one, this I also tried, and the restoration of the engineering section is not good, there is something else?	JD.com
promise that everything will turn out well	Battery Reconditioner	[cool]	JD.com
promise that everything will turn out well	Battery Reconditioner	Good merchandise, with the use of the phone to reduce the state of heat, praise!	JD.com
promise that everything will turn out well	Battery Reconditioner	The cell phone repairer has been received, after using it to evaluate, the effect is good, really good!	JD.com
promise that everything will turn out well	Battery Reconditioner	Charging function: very good, logistics is also very fast, full of praise, I hope the store more billing!	JD.com
promise that everything will turn out well	Battery Reconditioner	It looks good. I just don't know how it works.	JD.com
promise that everything will turn out well	Battery Reconditioner	Goods received, very often	JD.com
promise that everything will turn out well	Battery Reconditioner	Use it and then evaluate.	JD.com
promise that everything will turn out well	Battery Reconditioner	The logistics is fast, the product is very good, and the business service is very good.	JD.com

promise that everything will turn out well	Battery Reconditioner	[love]biglove[big love] [big]bigbig love] [big love] [big love][big love][big love][big love][big love][big love][big love][big love][big love][big love][big love][big love][big love][big love] [big love]	JD.com
promise that everything will turn out well	Battery Reconditioner	Let's have a good Pindo, everyone.	JD.com
promise that everything will turn out well	Battery Reconditioner	I haven't used it yet.	JD.com
promise that everything will turn out well	Battery Reconditioner	It's no use at all.	JD.com
promise that everything will turn out well	Battery Reconditioner	Received and very satisfied.Genuine.Next time to buy a few more.I can recommend it to my friends.	JD.com
promise that everything will turn out well	Battery Reconditioner	Goods to receive, not yet used, looks okay.	JD.com
promise that everything will turn out well	Battery Reconditioner	Fast shipping and good service! In the process of fixing the phone.	JD.com
promise that everything will turn out well	Battery Reconditioner	Baby has been received, affordable and good, highly recommended!	JD.com
promise that everything will turn out well	Battery Reconditioner	Product received. It is very delicately made. Logistics is also very fast. Used it twice and it works well.	JD.com
promise that everything will turn out well	Battery Reconditioner	Timely delivery	JD.com
promise that everything will turn out well	Battery Reconditioner	Functionality not clear but easy to open	JD.com
promise that everything will turn out well	Battery Reconditioner	Looked good, will patronize again!	JD.com
promise that everything will turn out well	Battery Reconditioner	Received the goods, logistics is very fast, packaging intact. Looking forward to using the effect. Favorable comments!	JD.com
promise that everything will turn out well	Battery Reconditioner	It doesn't work. It doesn't work at all.	JD.com
promise that everything will turn out well	Battery Reconditioner	Easy to use and economical	JD.com
promise that everything will turn out well	Battery Reconditioner	Very good! Works well without being expensive and reasonably priced. Thanks!	JD.com
promise that everything will turn out well	Battery Reconditioner	It's just a slide show.	JD.com
promise that everything will turn out well	Battery Reconditioner	Cell phone adapter received, received to try, the effect is very good, thank you business, give a five-star praise.	JD.com
promise that everything will turn out well	Battery Reconditioner	Marry****, what****?	JD.com
promise that everything will turn out well	Battery Reconditioner	Have not tried to have any effect, if you try to have a certain effect and then supplement the evaluation.	JD.com
promise that everything will turn out well	Battery Reconditioner	It doesn't work, people, this stuff doesn't work.	JD.com
promise that everything will turn out well	Battery Reconditioner	Good price, good performance, fast delivery!	JD.com
promise that everything will turn out well	Battery Reconditioner	Baby is basically the same as the description, fast shipping, worth it!	JD.com
promise that everything will turn out well	Battery Reconditioner	Baby received a very good, tried to fix in, good business service attitude, fast delivery, five-star praise.	JD.com
promise that everything will turn out well	Battery Reconditioner	quality for the money[smile]	JD.com

promise that everything will turn out well	Battery Reconditioner	I've got it.	JD.com
promise that everything will turn out well	Battery Reconditioner	Goods received, fast delivery, packaging is very good	JD.com
promise that everything will turn out well	Battery Reconditioner	Very practical, good quality and effective.	JD.com
promise that everything will turn out well	Battery Reconditioner	economical	JD.com
promise that everything will turn out well	Battery Reconditioner	Affordable, easy to use and should work well.	JD.com
promise that everything will turn out well	Battery Reconditioner	Received. It's good!	JD.com
promise that everything will turn out well	Battery Reconditioner	The goods have been received, the quality is very good to use the bar.	JD.com
promise that everything will turn out well	Battery Reconditioner	economical	JD.com
promise that everything will turn out well	Battery Reconditioner	repair the battery, the original cell phone lag after charging the Internet a lot faster, I've been the second time to buy a good price!	JD.com
promise that everything will turn out well	Battery Reconditioner	The quality is very good, very satisfied with a shopping, give 5 stars!	JD.com
promise that everything will turn out well	Battery Reconditioner	Used it a bit, it works well, really good, recommend!	JD.com
promise that everything will turn out well	Battery Reconditioner	The logistics is fast and the goods have arrived. Good worth buying.	JD.com
promise that everything will turn out well	Battery Reconditioner	The product is quite good and can repair cell phone batteries	JD.com
promise that everything will turn out well	Battery Reconditioner	The goods aren't great. It's still a fever.	JD.com
promise that everything will turn out well	Battery Reconditioner	Good reviews all five stars	JD.com
promise that everything will turn out well	Battery Reconditioner	I just received it, I don't know how effective it is, let's talk about it after using it	JD.com
promise that everything will turn out well	Battery Reconditioner	Charge repairer received very good, cost-effective, after using the phone does not card is very smooth, very happy, happy happy happy	JD.com
promise that everything will turn out well	Battery Reconditioner	Battery reconditioning is effective, reconditioning batteries is a good choice, good	JD.com
promise that everything will turn out well	Battery Reconditioner	Baby received, logistics is very fast, perfect packaging, easy to use! The effect is very good!	JD.com
promise that everything will turn out well	Battery Reconditioner	This user feels the product is better	JD.com
promise that everything will turn out well	Battery Reconditioner	doesn't light up. It's fake[Cool]	JD.com
promise that everything will turn out well	Battery Reconditioner	Very good quality and good value for money	JD.com
promise that everything will turn out well	Battery Reconditioner	The stuff is good logistics very fast service is very good	JD.com
promise that everything will turn out well	Battery Reconditioner	Received the goods. Unknown how effective it is.	JD.com
promise that everything will turn out well	Battery Reconditioner	Received the cell phone adapter, picked it up and used it on my phone, screen repair in progress, all good. Quite good!	JD.com

promise that everything will turn out well	Battery Reconditioner	I can't rate it at the moment because I don't understand it, so let's find out before we decide.<br>Non let evaluation, then give a two star first.<br>To be clear: it's not about the product or delivery, but about the setup of this evaluation program, which can't be passed without evaluation. But I don't know enough about the product to really make an accurate evaluation.	JD.com
promise that everything will turn out well	Battery Reconditioner	Only just used it, will follow up after using it	JD.com
promise that everything will turn out well	Battery Reconditioner	very good	JD.com
promise that everything will turn out well	Battery Reconditioner	Should work great. Should work great. Should work great.	JD.com
promise that everything will turn out well	Battery Reconditioner	The quality of the product is very good and the delivery is very fast.	JD.com
promise that everything will turn out well	Battery Reconditioner	The product is not spared, it works well	JD.com
promise that everything will turn out well	Battery Reconditioner	do what is advertised at all. Anyone knows, in the use of the phone can not be restored to factory settings, so will lose a lot of files can not be recovered, and then read it another several instructions can not be used, and again, to close this close that, I want it why? Just operate it directly in the settings, why do I need this cheat? Why do I need this tricky thing? Isn't it just a pantsless fart?<br>So this thing is useless, it's a scam.<br>Don't buy it, don't believe the black hearted businessmen doing black hearted advertising.	JD.com
promise that everything will turn out well	Battery Reconditioner	This charger is very quirky and should work very well	JD.com
promise that everything will turn out well	Battery Reconditioner	It works well. After two days of fixing it, the phone doesn't get stuck anymore, it's very good.	JD.com
promise that everything will turn out well	Battery Reconditioner	Received the goods on the use of the use of also stop good, this repair orchestration can be used is a good product	JD.com
promise that everything will turn out well	Battery Reconditioner	I like it very much, it's affordable, good quality, good to use, worth buying.	JD.com
promise that everything will turn out well	Battery Reconditioner	Received the product is very good good value for money	JD.com
promise that everything will turn out well	Battery Reconditioner	Cell phone adapter received, the bag is intact, logistics is also very fast, evaluation of five stars.	JD.com
promise that everything will turn out well	Battery Reconditioner	Goods have been received, tested, the use of the process is normal, whether the actual effect can be achieved, it is not convenient to say more, I guess it is good!	JD.com
promise that everything will turn out well	Battery Reconditioner	an excellent	JD.com
promise that everything will turn out well	Battery Reconditioner	Received, tightly packaged. Used it right away and it worked, after a couple hours, we'll see	JD.com
promise that everything will turn out well	Battery Reconditioner	We've got the goods.	JD.com
promise that everything will turn out well	Battery Reconditioner	Cell phone battery repairer received. Immediately connect the cell phone to try. Turn on the power, press the repairer switch. Repairer immediately lit up the word is repairing. After the activation of the battery. Battery use time is longer than before repair. All five stars praise.	JD.com
promise that everything will turn out well	Battery Reconditioner	It's so nice that it's as described by customer service, simple and functional.	JD.com
promise that everything will turn out well	Battery Reconditioner	Merchants shipping speed, packaging tight good no damage, good use worth buying!	JD.com
promise that everything will turn out well	Battery Reconditioner	Received the goods, seems to be good should be very good	JD.com
promise that everything will turn out well	Battery Reconditioner	Goods received, the quality is very good, you can use it if you need it, you can also come back to patronize the	JD.com

promise that everything will turn out well	Battery Reconditioner	Charge fix, good quality, works pretty well, worth having.	JD.com
promise that everything will turn out well	Battery Reconditioner	Good quality of goods cheap price is okay ah.	JD.com
promise that everything will turn out well	Battery Reconditioner	the charging display effect is good, according to the instructions have to charge a few more times, the merchant shipped very quickly service attitude is very good, in order to ensure that the long-lasting use of cell phone batteries, it is worth buying.	JD.com
promise that everything will turn out well	Battery Reconditioner	Easy to use, complete goods	JD.com
promise that everything will turn out well	Battery Reconditioner	The goods have been received, the logistics is very fast, the quality is very good, the effect is very good, I am very satisfied.	JD.com
promise that everything will turn out well	Battery Reconditioner	The product is as described and functions OK.	JD.com
promise that everything will turn out well	Battery Reconditioner	I'm using it and will comment later.	JD.com
promise that everything will turn out well	Battery Reconditioner	phone battery repair adapter has received, open the bag to see, to the online business description of the same goods, but also with instructions and certificates of conformity, and company profiles, etc., inserted into the charger head a try, quite useful, is the original genuine, I hope to be able to use the better and better to the cell phone and rechargeable treasure can be repaired great. thank the owner of the business and customer service !	JD.com
promise that everything will turn out well	Battery Reconditioner	The cell phone repairer looks good, but I haven't used it yet for fear of deleting my useful memory, so I'll put it away for now!	JD.com
promise that everything will turn out well	Battery Reconditioner	This user feels the product is better	JD.com
promise that everything will turn out well	Battery Reconditioner	thankfully	JD.com
promise that everything will turn out well	Battery Reconditioner	I don't know if it works or not, I'll come back with a review after using it	JD.com
promise that everything will turn out well	Battery Reconditioner	This user feels the product is better	JD.com
promise that everything will turn out well	Battery Reconditioner	This user feels the product is better	JD.com
promise that everything will turn out well	Battery Reconditioner	This user feels the product is better	JD.com
promise that everything will turn out well	Battery Reconditioner	This user feels the product is better	JD.com
promise that everything will turn out well	Battery Reconditioner	Received, the logistics is also very fast, the quality of use to know, or first to give a favorable review!	JD.com
promise that everything will turn out well	Battery Reconditioner	I just received it and will follow up after trying it out.	JD.com
promise that everything will turn out well	Battery Reconditioner	This user feels the product is better	JD.com
promise that everything will turn out well	Battery Reconditioner	I just got it. I don't know how long it will last.	JD.com
promise that everything will turn out well	Battery Reconditioner	I'll comment after I use it, for now.	JD.com
promise that everything will turn out well	Battery Reconditioner	Something small, easy to carry, voice prompts with advanced sense of how effective, to observe the comparison to know, the manufacturer's manual is complete, should be a regular product! Effect to be time tested.	JD.com
promise that everything will turn out well	Battery Reconditioner	Well-packed, logistics给力, the items received, and then comment after use.	JD.com

promise that everything will turn out well	Battery Reconditioner	Charges quickly very nice and works fine	JD.com
promise that everything will turn out well	Battery Reconditioner	Logistics is very fast, business service attitude is very good, I used a few days, can indeed repair[praise] [praise] [praise[praise] [praise] [praise] [praise] [praise] [praise] [praise] [praise] [praise] [praise][praise] [praise	JD.com
promise that everything will turn out well	Battery Reconditioner	Well made, easy to use, inexpensive, small and delicate[skimming]	JD.com
promise that everything will turn out well	Battery Reconditioner	The quality is good, as described, and the logistics is fast.	JD.com
promise that everything will turn out well	Battery Reconditioner	This user feels the product is better	JD.com
promise that everything will turn out well	Battery Reconditioner	Great battery repairer,"charging is repairing"and voice prompts when it turns on, very nice.	JD.com
promise that everything will turn out well	Battery Reconditioner	This user feels the product is better	JD.com
promise that everything will turn out well	Battery Reconditioner	The product is fine, everyone can go for it	JD.com
promise that everything will turn out well	Battery Reconditioner	This user feels the product is better	JD.com
promise that everything will turn out well	Battery Reconditioner	This user feels the product is better	JD.com
promise that everything will turn out well	Battery Reconditioner	Good quality and fast logistics	JD.com
promise that everything will turn out well	Battery Reconditioner	The goods received, logistics is very fast, five-star praise.	JD.com
promise that everything will turn out well	Battery Reconditioner	The goods have been received, the quality of use to know, quantity, logistics and service is very satisfied, thank you!	JD.com
promise that everything will turn out well	Battery Reconditioner	Genuine, merchants shipping fast , the goods are genuine , the majority of friends to rest assured that the purchase .	JD.com
promise that everything will turn out well	Battery Reconditioner	And voice announcements. Smart fixes.	JD.com
promise that everything will turn out well	Battery Reconditioner	.2024Model Smart Battery Repairer, high quality and low price. Dozfine tenant, nice color, sturdy and durable effective worth buying.	JD.com
promise that everything will turn out well	Battery Reconditioner	Goods received the quality is good, use is also quite good	JD.com
promise that everything will turn out well	Battery Reconditioner	The product is too good, logistics is also very fast, straight to buy!	JD.com
promise that everything will turn out well	Battery Reconditioner	I'm using it now and will wait a while to see how it works.	JD.com
promise that everything will turn out well	Battery Reconditioner	Cell phone battery repairer received, received a few days after the trial to give evaluation, things can be, fixed can not be a good repair to observe a period of time to say it.	JD.com
promise that everything will turn out well	Battery Reconditioner	This user feels the product is better	JD.com
promise that everything will turn out well	Battery Reconditioner	Baby received Just as described on the platform Let's see how it functions and continue to follow up	JD.com
promise that everything will turn out well	Battery Reconditioner	This thing received, used a few days before evaluating no feeling, is a fast charger that can not be directly plugged in, but also another plug in the USBcharging head to use, superfluous. Give three stars because:Rhyme Express courier does not deliver, directly dumped in the stage self-take, convenient for the courier bitter consumers, consumers are also helpless to complain about the fruitless. There are objections to you to find Rhymer Express complaints.	JD.com
promise that everything will turn out well	Battery Reconditioner	Works fine, charges quickly very nice and easy to use	JD.com

promise that everything will turn out well	Battery Reconditioner	Good value, cheap and easy to use	JD.com
promise that everything will turn out well	Battery Reconditioner	Like to give a good review, come back in a couple of days to follow up	JD.com
promise that everything will turn out well	Battery Reconditioner	It's good. It really works. Plugging it in makes the cell phone so much faster.	JD.com
promise that everything will turn out well	Battery Reconditioner	I bought it and used it and it feels good. I feel that the temperature of the phone has dropped. Use it for a while to see the effect. Should be a good product.	JD.com
promise that everything will turn out well	Battery Reconditioner	This user feels the product is better	JD.com
promise that everything will turn out well	Battery Reconditioner	Good quality product and easy to use!	JD.com
promise that everything will turn out well	Battery Reconditioner	Baby received, the quality is very good, the courier is awesome, so far away in three days, very good.	JD.com
promise that everything will turn out well	Battery Reconditioner	serviceable	JD.com
promise that everything will turn out well	Battery Reconditioner	Goods have been received, the quality of many good products, easy to use.	JD.com
promise that everything will turn out well	Battery Reconditioner	Cell phone repairer received, tried, charging fast, I do not know how to repair the effect, use a period of time to follow up the evaluation, the	JD.com
promise that everything will turn out well	Battery Reconditioner	Okay, thanks!	JD.com
promise that everything will turn out well	Battery Reconditioner	This user feels the product is better	JD.com
promise that everything will turn out well	Battery Reconditioner	Cell phone repair, expulsion of lagging to clear the garbage, a punch to fix, the phone is no burden to use without worrying about easy and smooth, really smart punch hey.	JD.com
promise that everything will turn out well	Battery Reconditioner	This user feels the product is better	JD.com
promise that everything will turn out well	Battery Reconditioner	like in the picture. The courier is very strong. Has been recommended to friends, there is a genuine logo, use a few days later to follow up!	JD.com
promise that everything will turn out well	Battery Reconditioner	This user feels the product is better	JD.com
promise that everything will turn out well	Battery Reconditioner	Cell phone battery repairer received, the logistics is very fast, the battery repair effect is very good Thank you!	JD.com
promise that everything will turn out well	Battery Reconditioner	This user feels the product is better	JD.com
promise that everything will turn out well	Battery Reconditioner	Works great, fast shipping, great features!	JD.com
promise that everything will turn out well	Battery Reconditioner	This user feels the product is better	JD.com
promise that everything will turn out well	Battery Reconditioner	Evaluate after use	JD.com
promise that everything will turn out well	Battery Reconditioner	Logistics is very fast, arrived and used and the description of the same, worth repurchase!	JD.com
promise that everything will turn out well	Battery Reconditioner	This user feels the product is better	JD.com
promise that everything will turn out well	Battery Reconditioner	🍓♡ Item received as described ♡♡🍓<br>🍓🍓♡ Logistics quickly served ♡🍓🍓🍓<br>🍓🍓🍓♡ 务很好 ♡🍓🍓🍓	JD.com
promise that everything will turn out well	Battery Reconditioner	Merchandise, fine workmanship, commodity integrity, after use in making additional comments	JD.com
promise that everything will turn out well	Battery Reconditioner	Cell phone battery repairer was cut off, cheap, after using the evaluation	JD.com

promise that everything will turn out well	Battery Reconditioner	This user feels the product is better	JD.com
promise that everything will turn out well	Battery Reconditioner	The product is of good quality and works, thank you.	JD.com
promise that everything will turn out well	Battery Reconditioner	This user feels the product is better	JD.com
promise that everything will turn out well	Battery Reconditioner	This user feels the product is better	JD.com
promise that everything will turn out well	Battery Reconditioner	This user feels the product is better	JD.com
promise that everything will turn out well	Battery Reconditioner	This user feels the product is better	JD.com
promise that everything will turn out well	Battery Reconditioner	Not yet used, do not evaluate, responsible for themselves and others, starting with me, good use will come back to evaluation	JD.com
promise that everything will turn out well	Battery Reconditioner	Things received the packaging good logistics fast things look good is genuine hope effect	JD.com
promise that everything will turn out well	Battery Reconditioner	Got it Haven't used it yet Hope it's as good as the video says it	JD.com
promise that everything will turn out well	Battery Reconditioner	Genuine, with language announcements in use.	JD.com
promise that everything will turn out well	Battery Reconditioner	This user feels the product is better	JD.com
promise that everything will turn out well	Battery Reconditioner	Goods received very satisfied.	JD.com
promise that everything will turn out well	Battery Reconditioner	exactly as it is in kind, with diagrams and written instructions as well as precautions for use. Clearly labeled the scope of use. The logistics company is also very fast and responsible.	JD.com
promise that everything will turn out well	Battery Reconditioner	This user feels the product is better	JD.com
promise that everything will turn out well	Battery Reconditioner	You can use this quality with good results	JD.com
promise that everything will turn out well	Battery Reconditioner	Okay, okay, okay, okay.	JD.com
promise that everything will turn out well	Battery Reconditioner	(of an unmarried couple) be close	JD.com
promise that everything will turn out well	Battery Reconditioner	Testimonials	JD.com
promise that everything will turn out well	Battery Reconditioner	Cell phone electricity also repair clamor,, fine workmanship, charging very quickly very good, working properly, goods complete, easy to use worth buying.	JD.com
promise that everything will turn out well	Battery Reconditioner	Shipped promptly and started using it.	JD.com
promise that everything will turn out well	Battery Reconditioner	an excellent	JD.com
promise that everything will turn out well	Battery Reconditioner	Logistics is fast, there is a voice function, but how the work efficiency, to be recharged and then supplement the evaluation.	JD.com
promise that everything will turn out well	Battery Reconditioner	This user feels the product is better	JD.com
promise that everything will turn out well	Battery Reconditioner	Charges quickly very nice and works fine	JD.com
promise that everything will turn out well	Battery Reconditioner	Goods have been received, the function is okay, good value for money, next time to introduce friends to buy good!	JD.com
promise that everything will turn out well	Battery Reconditioner	to use it and see the results.	JD.com

promise that everything will turn out well	Battery Reconditioner	Copy that, pretty good.	JD.com
promise that everything will turn out well	Battery Reconditioner	This user feels the product is better	JD.com
promise that everything will turn out well	Battery Reconditioner	This user feels the product is better	JD.com
promise that everything will turn out well	Battery Reconditioner	This user feels the product is better	JD.com
promise that everything will turn out well	Battery Reconditioner	First time using it, not sure if it's really good or not, to be followed up!	JD.com
promise that everything will turn out well	Battery Reconditioner	Cell phone repairer received, packaging, products are intact, the product is genuine, courier service is very good.	JD.com
promise that everything will turn out well	Battery Reconditioner	This user feels the product is better	JD.com
promise that everything will turn out well	Battery Reconditioner	Good value for money.	JD.com
promise that everything will turn out well	Battery Reconditioner	The product is of fast logistics and excellent quality! The product is a new product with high technological content. It is very practical after trying and should be praised!	JD.com
promise that everything will turn out well	Battery Reconditioner	twisting	JD.com
promise that everything will turn out well	Battery Reconditioner	It's a great deal for the price.	JD.com
promise that everything will turn out well	Battery Reconditioner	Cell phone battery repairer I received, have not yet used, the shopkeeper's service attitude is very good, fast delivery, express is very strong, give five-star praise!	JD.com
promise that everything will turn out well	Battery Reconditioner	This user feels the product is better	JD.com
promise that everything will turn out well	Battery Reconditioner	This user feels the product is better	JD.com
promise that everything will turn out well	Battery Reconditioner	Try it out.	JD.com
promise that everything will turn out well	Battery Reconditioner	It's not bad. It works as soon as it's plugged in.	JD.com
promise that everything will turn out well	Battery Reconditioner	I haven't used it yet, I'll comment when it's good.	JD.com
promise that everything will turn out well	Battery Reconditioner	very good	JD.com
promise that everything will turn out well	Battery Reconditioner	Trying it out and will comment after using it!	JD.com
promise that everything will turn out well	Battery Reconditioner	Nice product, very easy to use	JD.com
promise that everything will turn out well	Battery Reconditioner	Baby received very quickly, cell phone battery repair have purchased three repair is not ideal, a long time are difficult to achieve the effect of repair and charging, this time to buy the genuine logo and the original genuine seal. Only slightly more money or buy high-grade product value. I would like to give a five-star praise, thanks to the business good product.	JD.com
promise that everything will turn out well	Battery Reconditioner	This user feels the product is better	JD.com
promise that everything will turn out well	Battery Reconditioner	Received thanks!	JD.com
promise that everything will turn out well	Battery Reconditioner	This user feels the product is better	JD.com
promise that everything will turn out well	Battery Reconditioner	Received by courier. Hope it works.	JD.com

promise that everything will turn out well	Battery Reconditioner	That's good!	JD.com
promise that everything will turn out well	Battery Reconditioner	Received this cell phone battery adapter, by charging the cell phone to test, the original cell phone power consumption fast problem has a repair effect, can extend the battery use time, with the product description.	JD.com
promise that everything will turn out well	Battery Reconditioner	Great quality, fast logistics, five stars.	JD.com
promise that everything will turn out well	Battery Reconditioner	Battery Repairer received, small and exquisite, company profile, complete instructions, inexpensive, trustworthy and recommend friends to buy.	JD.com
promise that everything will turn out well	Battery Reconditioner	Has been used, compact and convenient, and then see the effect of use and then follow up.	JD.com
promise that everything will turn out well	Battery Reconditioner	Good for boosting old batteries	JD.com
promise that everything will turn out well	Battery Reconditioner	Pretty good! With voice function! Charges and fixes both!	JD.com
promise that everything will turn out well	Battery Reconditioner	There are voice prompts, the use of ease of use, the power of high-tech to be used after the follow-up evaluation!	JD.com
promise that everything will turn out well	Battery Reconditioner	The goods have been received, fast logistics, well packaged, and instructions, easy to use!	JD.com
promise that everything will turn out well	Battery Reconditioner	the last time I bought a little better, according to the instructions prompted to use a period of time to show its effect, the current first initial evaluation, and later according to the effect of the use of the re-assessment of the follow-up.	JD.com
promise that everything will turn out well	Battery Reconditioner	Fast shipping, affordable and easy to use	JD.com
promise that everything will turn out well	Battery Reconditioner	This user feels the product is better	JD.com
promise that everything will turn out well	Battery Reconditioner	Good goods logistics very fast service is very good worth buying	JD.com
promise that everything will turn out well	Battery Reconditioner	It's not working.	JD.com
promise that everything will turn out well	Battery Reconditioner	an excellent	JD.com
promise that everything will turn out well	Battery Reconditioner	The quality is great[love][great love]great]love[great] [great love] [great love] [great love] [great love] [great love] [great love] [great love] [great love] [great love] [great love] [great love]	JD.com
promise that everything will turn out well	Battery Reconditioner	Merchant shipped promptly, well packaged, and good value for the money.	JD.com
promise that everything will turn out well	Battery Reconditioner	Black tech products that work well and are worth using	JD.com
promise that everything will turn out well	Battery Reconditioner	and practical worth looking at and owning……	JD.com
promise that everything will turn out well	Battery Reconditioner	This user feels the product is better	JD.com
promise that everything will turn out well	Battery Reconditioner	This user feels the product is better	JD.com
promise that everything will turn out well	Battery Reconditioner	The repair charger was received, has voice prompts and works fine. Very pleasant shopping.	JD.com
promise that everything will turn out well	Battery Reconditioner	I'll give you a good review first and follow up after using it.	JD.com
promise that everything will turn out well	Battery Reconditioner	How can I use it? I don't know. I'll know after I use it.	JD.com
promise that everything will turn out well	Battery Reconditioner	This user feels the product is better	JD.com

promise that everything will turn out well	Battery Reconditioner	Battery adapter received, business fast delivery, good service, consistent with the description, logistics is also strong!	JD.com
promise that everything will turn out well	Battery Reconditioner	This user feels the product is better	JD.com
promise that everything will turn out well	Battery Reconditioner	I've received it, I'll comment after I use it.	JD.com
promise that everything will turn out well	Battery Reconditioner	Prompt delivery Aff Good!	JD.com
promise that everything will turn out well	Battery Reconditioner	I'm using it. I don't know how it works.	JD.com
promise that everything will turn out well	Battery Reconditioner	This user feels the product is better	JD.com
promise that everything will turn out well	Battery Reconditioner	2024model cell phone smart repair cell phone lagging hot memory insufficient charger received, I hope the hand motor board is helpful, good will recommend friends.	JD.com
promise that everything will turn out well	Battery Reconditioner	Battery Repairer received, product quality and description match, good service attitude, timely delivery.	JD.com
promise that everything will turn out well	Battery Reconditioner	Baby received pretty good[smile]is a satisfied shopping!	JD.com
promise that everything will turn out well	Battery Reconditioner	I'll comment after I use it. - Thanks.	JD.com
promise that everything will turn out well	Battery Reconditioner	Things received, more like satisfied. Has been five-star praise.	JD.com
promise that everything will turn out well	Battery Reconditioner	charging speed can be, in the end, how much repair on the cell phone battery is unknown, anyway, the repairer is not expensive, buy a try, there is always a little bit of work, give five stars!	JD.com
promise that everything will turn out well	Battery Reconditioner	Cell phone battery adapter received, packaging is good, although this use, still first five stars. To be used and then make up the comment.	JD.com
promise that everything will turn out well	Battery Reconditioner	This item is genuine and works very well, I'll buy another one in a few days	JD.com
promise that everything will turn out well	Battery Reconditioner	Fast shipping, well made, is using it, works great, happy with it!	JD.com
promise that everything will turn out well	Battery Reconditioner	I'm trying it out. I'll comment when it works.	JD.com
promise that everything will turn out well	Battery Reconditioner	Received the product small and cute to try the effect is okay!	JD.com
promise that everything will turn out well	Battery Reconditioner	The restorer was received and is very good, I love it and am very happy with it!	JD.com
promise that everything will turn out well	Battery Reconditioner	The quality of the product is very good, it is worth everyone's trust and enough to buy.	JD.com
promise that everything will turn out well	Battery Reconditioner	Affordable good use good-looking inexpensive, logistics is very fast packaging intact no damage bad. Economical and affordable good-looking and inexpensive, logistics is very fast packaging intact without damage bad.	JD.com
promise that everything will turn out well	Battery Reconditioner	Practical and fast charging. Buy again next time.	JD.com
promise that everything will turn out well	Battery Reconditioner	Intelligent Charge Repair Repairer received, the logistics is very fast, with a very good.	JD.com
promise that everything will turn out well	Battery Reconditioner	Feel the effect is not very ideal, sincere use for a period of time before evaluation, payment first.	JD.com
promise that everything will turn out well	Battery Reconditioner	(of an unmarried couple) be close	JD.com
promise that everything will turn out well	Battery Reconditioner	Goods received, well-packaged, took back and used it, the effect is good, praise.	JD.com

promise that everything will turn out well	Battery Reconditioner	Fast arrival, great service, great price, awesome!	JD.com
promise that everything will turn out well	Battery Reconditioner	Charges quickly very good, works fine, well made	JD.com
promise that everything will turn out well	Battery Reconditioner	Honest business, prompt shipping, very well packaged and effective.	JD.com
promise that everything will turn out well	Battery Reconditioner	[Kudos][Kudos][Kudos]	JD.com
promise that everything will turn out well	Battery Reconditioner	The item is consistent with the description, small and exquisite, and with the instruction manual, convenient and practical.	JD.com
promise that everything will turn out well	Battery Reconditioner	This user feels the product is better	JD.com
promise that everything will turn out well	Battery Reconditioner	Scared me, suddenly talking, well, kudos👍kudos👍kudos👍kudos	JD.com
promise that everything will turn out well	Battery Reconditioner	This user feels the product is better	JD.com
promise that everything will turn out well	Battery Reconditioner	logistics, with two days of good results and then give a follow-up comment, expect customers to rest assured that purchase!	JD.com
promise that everything will turn out well	Battery Reconditioner	Charges quickly very good, works fine, well made	JD.com
promise that everything will turn out well	Battery Reconditioner	This user feels the product is better	JD.com
promise that everything will turn out well	Battery Reconditioner	Cell phone battery adapter two to receive. Wait for the use of the effect and then come back to evaluate. First give five-star praise.	JD.com
promise that everything will turn out well	Battery Reconditioner	I haven't used it yet, I don't know how it performs.	JD.com
promise that everything will turn out well	Battery Reconditioner	Fixer received, looks good, voice works fine, can have it.	JD.com
promise that everything will turn out well	Battery Reconditioner	Wait to use it before you have a say.	JD.com
promise that everything will turn out well	Battery Reconditioner	This user feels the product is better	JD.com
promise that everything will turn out well	Battery Reconditioner	This user feels the product is better	JD.com
promise that everything will turn out well	Battery Reconditioner	Very good and especially satisfied Recommended to buy	JD.com
promise that everything will turn out well	Battery Reconditioner	Electric Ground Repairer received to repair batteries, extend life and multiple protections.	JD.com
promise that everything will turn out well	Battery Reconditioner	This user feels the product is better	JD.com
promise that everything will turn out well	Battery Reconditioner	And the voice prompts, nice[Loves it]	JD.com
promise that everything will turn out well	Battery Reconditioner	👍👍	JD.com
promise that everything will turn out well	Battery Reconditioner	(of an unmarried couple) be close	JD.com
promise that everything will turn out well	Battery Reconditioner	Cell phone battery adapter received, well packaged, the product is labeled with genuine logo and anti-counterfeiting identification code, very formal. First give five stars. Use for a period of time before making in-depth evaluation.	JD.com
promise that everything will turn out well	Battery Reconditioner	The cell phone battery adapter just arrived. Looking at the description it's a great piece of appliance. If it works well, I will repurchase a few more and refer friends and family to purchase this product with me!	JD.com

promise that everything will turn out well	Battery Reconditioner	It's good, fast charging, has the function of repairing the phone battery, I'll comment after using it a few more times.	JD.com
promise that everything will turn out well	Battery Reconditioner	Fast shipping, good packaging, good quality, ready to broadcast, worth buying.	JD.com
promise that everything will turn out well	Battery Reconditioner	Just received the item and haven't used it yet can't comment	JD.com
promise that everything will turn out well	Battery Reconditioner	This user feels the product is better	JD.com
promise that everything will turn out well	Battery Reconditioner	Repairer received, logistics is very fast, packaging is very good, good value for money, recommended to buy[loveit]	JD.com
promise that everything will turn out well	Battery Reconditioner	Fast shipping, good quality, felt good trying it out	JD.com
promise that everything will turn out well	Battery Reconditioner	without damage, the goods received immediately after the trial, the current charging is normal, yet to watch its effect.	JD.com
promise that everything will turn out well	Battery Reconditioner	Commodity received, tried it, it is true, the phone is good card, fix it. Good, good, straight to buy, logistics is also very fast.	JD.com
promise that everything will turn out well	Battery Reconditioner	a good service attitude, fast logistics, courier service is also good! All in all a more satisfactory shopping experience🏀! Kudos! Give five-star praise! If there is a next time to buy your home!	JD.com
promise that everything will turn out well	Battery Reconditioner	Received very good, and now are using looking forward to the effect, praise!	JD.com
promise that everything will turn out well	Battery Reconditioner	It's been planted.	JD.com
promise that everything will turn out well	Battery Reconditioner	This user feels the product is better	JD.com
promise that everything will turn out well	Battery Reconditioner	The e-restorer is great and will be back next time!	JD.com
promise that everything will turn out well	Battery Reconditioner	Received, well-packaged, timely delivery, used hope and anchor introduction of a kind, effective. First five-star praise, after using the chase home reviews.	JD.com
promise that everything will turn out well	Battery Reconditioner	open ads out of this buy back two days of the phone is not stuck can see WeChat, can also play fast hand, and not jumping good use. Buy a used cell phone only to spend two hundred and twenty-four dollars for more than a month can not be used, and now it is good to use thanks to this charging restorer helped me a great deal, give power!	JD.com
promise that everything will turn out well	Battery Reconditioner	Goods received, logistics is very fast oh! Thank you!	JD.com
promise that everything will turn out well	Battery Reconditioner	Charges quickly very good, product works well	JD.com
promise that everything will turn out well	Battery Reconditioner	I don't know if it's good or bad.	JD.com
promise that everything will turn out well	Battery Reconditioner	This user feels the product is better	JD.com
promise that everything will turn out well	Battery Reconditioner	OK, good value for money, great.	JD.com
promise that everything will turn out well	Battery Reconditioner	Merchandise received. Very good!	JD.com
promise that everything will turn out well	Battery Reconditioner	First according to do with the manual test charging mainly to extend the battery life other fearless look forward to results	JD.com
promise that everything will turn out well	Battery Reconditioner	Not tried, will comment after use.	JD.com
promise that everything will turn out well	Battery Reconditioner	favorable criticism	JD.com
promise that everything will turn out well	Battery Reconditioner	This user feels the product is better	JD.com

promise that everything will turn out well	Battery Reconditioner	Take delivery first.	JD.com
promise that everything will turn out well	Battery Reconditioner	Intelligent repair of cell phone batteries have received, can not wait to go back to the plug socket inserted, immediately came the voice broadcast, intelligent repair has started, this is the best product I have bought, and no longer need to worry about cell phone batteries do not last long, after repairing the good at least can be used for a day!	JD.com
promise that everything will turn out well	Battery Reconditioner	I just used it and I don't know how it works first 5stars	JD.com
promise that everything will turn out well	Battery Reconditioner	The product works well, is good value for money, and the service and logistics are excellent.	JD.com
promise that everything will turn out well	Battery Reconditioner	Received, goods complete, good use would recommend.	JD.com
promise that everything will turn out well	Battery Reconditioner	This user feels the product is better	JD.com
promise that everything will turn out well	Battery Reconditioner	Baby is good Applicable price is cheap Worth to shoot the treasure Bong Zang	JD.com
promise that everything will turn out well	Battery Reconditioner	manner, logistics come quickly. The product looked okay, happy cooperation, attention to your store to continue to cooperate in the future	JD.com
promise that everything will turn out well	Battery Reconditioner	This user feels the product is better	JD.com
promise that everything will turn out well	Battery Reconditioner	This user feels the product is better	JD.com
promise that everything will turn out well	Battery Reconditioner	Fast logistics and distribution, good product quality, perfect and good packaging, the original authentic logo, you can check the authenticity.	JD.com
promise that everything will turn out well	Battery Reconditioner	This user feels the product is better	JD.com
promise that everything will turn out well	Battery Reconditioner	Holy shit. Junk items. They don't even light up.	JD.com
promise that everything will turn out well	Battery Reconditioner	Looking forward to its efficacy!	JD.com
promise that everything will turn out well	Battery Reconditioner	This cell phone battery adapter works well, works well, I like it.	JD.com
promise that everything will turn out well	Battery Reconditioner	I hope it works.	JD.com
promise that everything will turn out well	Battery Reconditioner	[Smiles]	JD.com
promise that everything will turn out well	Battery Reconditioner	Don't know how well it works to be evaluated after use. -I don't know how it works.	JD.com
promise that everything will turn out well	Battery Reconditioner	The item works and the effect is still present.	JD.com
promise that everything will turn out well	Battery Reconditioner	Charge Fixer received, works great kudos👍👍👍👍👍	JD.com
promise that everything will turn out well	Battery Reconditioner	This product works great.	JD.com
promise that everything will turn out well	Battery Reconditioner	Goods received, fast delivery, good service, good quality!	JD.com
promise that everything will turn out well	Battery Reconditioner	It works great.	JD.com
promise that everything will turn out well	Battery Reconditioner	With voice broadcasting, repairing the battery, multiple protection, improving life, and fast logistics. Worth pointing[kudos]	JD.com
promise that everything will turn out well	Battery Reconditioner	The goods have been received in perfect condition, very satisfied with the purchase!	JD.com

promise that everything will turn out well	Battery Reconditioner	I just received the item and will review it after I use it.	JD.com
promise that everything will turn out well	Battery Reconditioner	quality of his cell phone charger is really good and affordable welcome to come and buy it[smile]	JD.com
promise that everything will turn out well	Battery Reconditioner	Baby received, fine workmanship, and business description of the same, worth buying!	JD.com
promise that everything will turn out well	Battery Reconditioner	The product works very well , and the logistics are fast , and I like it very much.	JD.com
promise that everything will turn out well	Battery Reconditioner	Logistics is very fast, this evening to repair the battery, try to see how the effect, and then follow up!	JD.com
promise that everything will turn out well	Battery Reconditioner	It's good stuff. It's worth having.	JD.com
promise that everything will turn out well	Battery Reconditioner	Works well, good value for money, worth the money.	JD.com
Mazda Motor Corporation	Battery Repairer	Very good.	JD.com
Mazda Motor Corporation	Battery Repairer	Received not yet used wait to use after the evaluation	JD.com
Mazda Motor Corporation	Battery Repairer	Received the goods and feel good after using it.	JD.com
Mazda Motor Corporation	Battery Repairer	an excellent	JD.com
Mazda Motor Corporation	Battery Repairer	This user feels the product is better	JD.com
Mazda Motor Corporation	Battery Repairer	have to have a bad phone to use to know, look pretty good, first praise.	JD.com
Mazda Motor Corporation	Battery Repairer	This user did not fill in a rating	JD.com
Mazda Motor Corporation	Battery Repairer	It works great.	JD.com
Mazda Motor Corporation	Battery Repairer	In use	JD.com
Mazda Motor Corporation	Battery Repairer	dissatisfied	JD.com
Mazda Motor Corporation	Battery Repairer	It's being tested.	JD.com
Mazda Motor Corporation	Battery Repairer	Logistics and express quite fast, quite good, try to charge quite fast, the	JD.com
Mazda Motor Corporation	Battery Repairer	packaging is intact, the quality is very good, straight to buy.	JD.com
Mazda Motor Corporation	Battery Repairer	Cell phone charging repair plug received, the packaging is undamaged. Look at the appearance is quite good, wait to use to see the effect and then follow up.	JD.com
Mazda Motor Corporation	Battery Repairer	This user feels the product is better	JD.com
Mazda Motor Corporation	Battery Repairer	And impersonate.	JD.com
Mazda Motor Corporation	Battery Repairer	I started using it after I received it, and it's pretty good with the voice announcements. The customer service lady is also very enthusiastic like fire!	JD.com
Mazda Motor Corporation	Battery Repairer	Bought for a friend first time buy use good will buy back!	JD.com
Mazda Motor Corporation	Battery Repairer	This user feels the product is better	JD.com
Mazda Motor Corporation	Battery Repairer	This user feels the product is better	JD.com
Mazda Motor Corporation	Battery Repairer	I don't know if it's fixable, but I'll give you a good review first.	JD.com
Mazda Motor Corporation	Battery Repairer	Very good. - Some good.	JD.com
Mazda Motor Corporation	Battery Repairer	That's good.	JD.com
Mazda Motor Corporation	Battery Repairer	Merchants shipping strong logistics very quickly packaged very well, try to have a sound with a display, as for the effect can not be confirmed, only with the display to get a good heart, well no heart disease, the impact is not great, pay IQ tax is, customary five-star praise!	JD.com
Mazda Motor Corporation	Battery Repairer	How do you know if the product is good or not when you haven't even used it yet?	JD.com
Mazda Motor Corporation	Battery Repairer	This user did not fill in a rating	JD.com

Mazda Motor Corporation	Battery Repairer	(of an unmarried couple) be close	JD.com
Mazda Motor Corporation	Battery Repairer	The quality of the merchandise is so good.	JD.com
Mazda Motor Corporation	Battery Repairer	This user feels the product is better	JD.com
Mazda Motor Corporation	Battery Repairer	Five stars in advance Product is in use	JD.com
Mazda Motor Corporation	Battery Repairer	Voice intelligent broadcasting, very good and good, next time to buy again	JD.com
Mazda Motor Corporation	Battery Repairer	This user feels the product is better	JD.com
Mazda Motor Corporation	Battery Repairer	Appearance is beautiful, fine workmanship, easy to use, clear display, indeed have the effect of repair, two days the battery seems to be a little more durable	JD.com
Mazda Motor Corporation	Battery Repairer	Just received, useful or not yet know, first praise!	JD.com
Mazda Motor Corporation	Battery Repairer	Just received the goods! Will comment further when I've used it. Five stars first! Because the appearance is good.	JD.com
Mazda Motor Corporation	Battery Repairer	This user feels the product is better	JD.com
Mazda Motor Corporation	Battery Repairer	I've just used it once and I can't comment on it.	JD.com
Mazda Motor Corporation	Battery Repairer	Let's try it first.	JD.com
Mazda Motor Corporation	Battery Repairer	very much	JD.com
Mazda Motor Corporation	Battery Repairer	A product that doesn't work, caution buying!	JD.com
Mazda Motor Corporation	Battery Repairer	This user did not fill in a rating	JD.com
Mazda Motor Corporation	Battery Repairer	This user did not fill in a rating	JD.com
Mazda Motor Corporation	Battery Repairer	This user did not fill in a rating	JD.com
Mazda Motor Corporation	Battery Repairer	Affordable, good charging cleanup and fast logistics	JD.com
Mazda Motor Corporation	Battery Repairer	I haven't used it yet.	JD.com
Mazda Motor Corporation	Battery Repairer	I don't know how well the repair works, but I'll give it a good review first and follow up after use	JD.com
Mazda Motor Corporation	Battery Repairer	an excellent	JD.com
Mazda Motor Corporation	Battery Repairer	Genuine 'Rechargeable Repair Batteries': Increase power and also refresh memory: battery durability, clean up trash, etc.	JD.com
Mazda Motor Corporation	Battery Repairer	Received it and used it according to the instructions, I don't know how effective it is, I hope that the problem of the cell phone charging heat can be solved. First give 4 stars, use three weeks time to see the effect and then follow up.	JD.com
Mazda Motor Corporation	Battery Repairer	Received the product is trying, thank you merchants and courier boy!	JD.com
Mazda Motor Corporation	Battery Repairer	Yes, yes, customer service explained very clearly, as presented	JD.com
Mazda Motor Corporation	Battery Repairer	Battery Repairer received, well-packed, accustomed to praise. Try a period of time to see the effect and then follow up!	JD.com
Mazda Motor Corporation	Battery Repairer	It's good quality.	JD.com
Mazda Motor Corporation	Battery Repairer	Effective[hee]	JD.com
Mazda Motor Corporation	Battery Repairer	It's good. It's very good.	JD.com
Mazda Motor Corporation	Battery Repairer	The goods are real, thanks to the merchant, thanks to the express delivery	JD.com
Mazda Motor Corporation	Battery Repairer	an excellent	JD.com
Mazda Motor Corporation	Battery Repairer	The display is clear, nice looking and well made.	JD.com
Mazda Motor Corporation	Battery Repairer	Received. Very pleased.	JD.com
Mazda Motor Corporation	Battery Repairer	This user feels the product is better	JD.com
Mazda Motor Corporation	Battery Repairer	voice broadcasting, very suitable for cell phone companion Lv, the next time will come back to buy, I hope everyone fast enough to buy it!	JD.com
Mazda Motor Corporation	Battery Repairer	The logistics is also fast and the service too degree is very good.	JD.com
Mazda Motor Corporation	Battery Repairer	Very well made, with voice prompts and authentic labeling	JD.com

Mazda Motor Corporation	Battery Repairer	At first it would flush in. Then a few times after using it, it actually charged all night and was still at the same value as when it was low!I didn't believe it and charged another cell phone for 1hour without flushing in the slightest bit of electricity. Realistically it could be an IQ tax.	JD.com
Mazda Motor Corporation	Battery Repairer	This user feels the product is better	JD.com
Mazda Motor Corporation	Battery Repairer	This product is great received it today and used it as soon as I received it	JD.com
Mazda Motor Corporation	Battery Repairer	This user feels the product is better	JD.com
Mazda Motor Corporation	Battery Repairer	IQ tax. It's useless.	JD.com
Mazda Motor Corporation	Battery Repairer	The logistics is very fast things have to be used to know	JD.com
Mazda Motor Corporation	Battery Repairer	This item is great very happy with it	JD.com
Mazda Motor Corporation	Battery Repairer	This product is very good genuine worth buying!	JD.com
Mazda Motor Corporation	Battery Repairer	Received quickly, genuine good, recommended to friends to buy!	JD.com
Mazda Motor Corporation	Battery Repairer	Let's see and then we'll talk.	JD.com
Mazda Motor Corporation	Battery Repairer	The goods have been received, tried, feel good, praise!	JD.com
Mazda Motor Corporation	Battery Repairer	I don't know. Does it matter?	JD.com
Mazda Motor Corporation	Battery Repairer	The product received, well-packed, power normal, but the function of repair is not yet known, there may be some problems to the present power after being repaired after the battery does not light, I do not know how it happened!	JD.com
Mazda Motor Corporation	Battery Repairer	This user feels the product is better	JD.com
Mazda Motor Corporation	Battery Repairer	Contact evaluation: Yesterday, when I picked up the goods brand new can not see the effect, to be used for a period of time to see and then follow up!	JD.com
Mazda Motor Corporation	Battery Repairer	I don't know how well it works.	JD.com
Mazda Motor Corporation	Battery Repairer	serviceable	JD.com
Mazda Motor Corporation	Battery Repairer	dissatisfied	JD.com
Mazda Motor Corporation	Battery Repairer	This user feels the product is better	JD.com
Mazda Motor Corporation	Battery Repairer	It doesn't feel like it's doing much of anything!	JD.com
Mazda Motor Corporation	Battery Repairer	Everything is fine! I'll comment after the effect is used!	JD.com
Mazda Motor Corporation	Battery Repairer	Arrived quickly, packaging is also good, the product is as good as the description, customer service attitude of patience have asked quickly back, repair charger has been in use very good, affordable, worth buying.	JD.com
Mazda Motor Corporation	Battery Repairer	Goods have been received, recovery battery is very good, is genuine	JD.com
Mazda Motor Corporation	Battery Repairer	is very good, plug in the power on the voice broadcast, small things to solve big problems is really great, business service is very good, fast delivery, this shopping is very satisfied, give a big praise.	JD.com
Mazda Motor Corporation	Battery Repairer	Average, not seeing results	JD.com
Mazda Motor Corporation	Battery Repairer	Things are good, there are discounts, you can rest assured to save money to buy, do not hesitate to quality is very good, received the first time to open the goods to see, it is very good, very satisfied, will be back to buy!	JD.com
Mazda Motor Corporation	Battery Repairer	This user feels the product is better	JD.com
Mazda Motor Corporation	Battery Repairer	Just received the item today. Love the stuff and very happy with it.	JD.com
Mazda Motor Corporation	Battery Repairer	okay, charging is quite fast, and the seller described as good, the seller's service attitude is also very good, have shared the link to my friends, the next time will come back to buy.	JD.com
Mazda Motor Corporation	Battery Repairer	favorable criticism	JD.com
Mazda Motor Corporation	Battery Repairer	The cell phone number repairer has been received. Beautifully constructed, simple, practical and effective.	JD.com

Mazda Motor Corporation	Battery Repairer	Got it last night and rushed to plug it in and try it out! The owner said to fix it at night when I went to bed ! I did! I did as I was told. I may not be able to do it once, but I'll fix it in a couple of days! Overall, it's not bad.	JD.com
Mazda Motor Corporation	Battery Repairer	This user feels the product is better	JD.com
Mazda Motor Corporation	Battery Repairer	Charge restorer has received, the quality of workmanship are very good, has begun to put into use, there are voice function prompts, and now use a short time, have not seen the effect, well after a period of time to follow up on the evaluation, good value for money worth buying.	JD.com
Mazda Motor Corporation	Battery Repairer	This user feels the product is better	JD.com
Mazda Motor Corporation	Battery Repairer	The quality of the goods is exceptionally good and the logistics are fast.	JD.com
Mazda Motor Corporation	Battery Repairer	Cell phone battery adapter received.	JD.com
Mazda Motor Corporation	Battery Repairer	Good quality, genuine, good service, courier delivery is better.	JD.com
Mazda Motor Corporation	Battery Repairer	The goods were received and the logistics were fast!	JD.com
Mazda Motor Corporation	Battery Repairer	The quality of the product is very good, the logistics is fast and the service is good.	JD.com
Mazda Motor Corporation	Battery Repairer	This user feels the product is better	JD.com
Mazda Motor Corporation	Battery Repairer	All good, all 5 stars.	JD.com
Mazda Motor Corporation	Battery Repairer	not yet used to give five stars first, but I hope it works well.	JD.com
Mazda Motor Corporation	Battery Repairer	This user feels the product is better	JD.com
Mazda Motor Corporation	Battery Repairer	Good review, fixed it overnight and really the phone is not lagging, awesome.	JD.com
Mazda Motor Corporation	Battery Repairer	can	JD.com
Mazda Motor Corporation	Battery Repairer	This user feels the product is better	JD.com
Mazda Motor Corporation	Battery Repairer	Haven't used it yet don't know how it works	JD.com
Mazda Motor Corporation	Battery Repairer	As described, logistics fast, good business service attitude, is a satisfactory shopping	JD.com
Mazda Motor Corporation	Battery Repairer	It's good quality.	JD.com
Mazda Motor Corporation	Battery Repairer	This user feels the product is better	JD.com
Mazda Motor Corporation	Battery Repairer	This user feels the product is better	JD.com
Mazda Motor Corporation	Battery Repairer	I used it for a day before evaluating it, it does work on the battery of the old cell phone, the battery lasts longer than the original one	JD.com
Mazda Motor Corporation	Battery Repairer	The quality of the charger is excellent, the logistics is fast, and it is the ideal item.	JD.com
Mazda Motor Corporation	Battery Repairer	This user feels the product is better	JD.com
Mazda Motor Corporation	Battery Repairer	Baby received and looking forward to using it!	JD.com
Mazda Motor Corporation	Battery Repairer	Received the item today and the quality is as good as the store! Great value for money and shared with friends!	JD.com
Mazda Motor Corporation	Battery Repairer	I'll wait to use it before I ask for a price!	JD.com
Mazda Motor Corporation	Battery Repairer	Merchants are not good.	JD.com
Mazda Motor Corporation	Battery Repairer	Fast logistics, great quality, reasonably priced, great fixes, the	JD.com
Mazda Motor Corporation	Battery Repairer	Looking forward to being as good as the item describes and reclaiming the aging battery.	JD.com
Mazda Motor Corporation	Battery Repairer	All good, all 5 stars.	JD.com
Mazda Motor Corporation	Battery Repairer	I've received the goods but I don't know how it works.	JD.com
Mazda Motor Corporation	Battery Repairer	Not bad.The effect of charging once is not yet visible, it takes several times to work.	JD.com
Mazda Motor Corporation	Battery Repairer	Buy one to try and expect results. Kudos to CMT!	JD.com
Mazda Motor Corporation	Battery Repairer	Am using it to see how it works.	JD.com
Mazda Motor Corporation	Battery Repairer	Cell phone battery repairer received, see the introduction is quite good, immediately try!	JD.com

Mazda Motor Corporation	Battery Repairer	it really doesn't turn on, it still doesn't turn on the frequency.	JD.com
Mazda Motor Corporation	Battery Repairer	I have received it, 5 stars first, and will follow up after using it!	JD.com
Mazda Motor Corporation	Battery Repairer	Come back when you're ready.	JD.com
Mazda Motor Corporation	Battery Repairer	I'm glad to finally find a regular cell phone battery repairer also generation of voice prompts and anti-counterfeiting marking same day shipment thanks to the merchant.	JD.com
Mazda Motor Corporation	Battery Repairer	I haven't used it yet.	JD.com
Mazda Motor Corporation	Battery Repairer	Good use next time again! Please support this product more!	JD.com
Mazda Motor Corporation	Battery Repairer	This user feels the product is better	JD.com
Mazda Motor Corporation	Battery Repairer	not used bad evaluation, logistics is also very fast, courier brother hard ah[cool][cool]	JD.com
Mazda Motor Corporation	Battery Repairer	Charges well, worth the money	JD.com
Mazda Motor Corporation	Battery Repairer	It's good quality.	JD.com
Mazda Motor Corporation	Battery Repairer	(of an unmarried couple) be close	JD.com
Mazda Motor Corporation	Battery Repairer	Goods received, looks good after use to evaluate	JD.com
Mazda Motor Corporation	Battery Repairer	I rated it well in my former self.	JD.com
Mazda Motor Corporation	Battery Repairer	Things received logistics very quickly, just do not know how to use the effect of the use of the function of the effect of the temporary do not comment!	JD.com
Mazda Motor Corporation	Battery Repairer	I used it once and I'll review it later.	JD.com
Mazda Motor Corporation	Battery Repairer	Cell phone battery repairer received, as described,,, easy to use, really good, give you praise five stars.	JD.com
Mazda Motor Corporation	Battery Repairer	It's really not bad Battery capacity fixed Nice Nice Worth it	JD.com
Mazda Motor Corporation	Battery Repairer	Repairer received, the appearance is very beautiful, now in use, use a period of time to evaluate. Fast logistics, good customer service, affordable!	JD.com
Mazda Motor Corporation	Battery Repairer	This user feels the product is better	JD.com
Mazda Motor Corporation	Battery Repairer	I've been using it for a few days, but it doesn't seem to be working!	JD.com
Mazda Motor Corporation	Battery Repairer	Good review worth buying	JD.com
Mazda Motor Corporation	Battery Repairer	The store shipped very quickly, good service attitude, should be genuine, a very pleasant shopping.	JD.com
Mazda Motor Corporation	Battery Repairer	The goods have been received thank you pro!	JD.com
Mazda Motor Corporation	Battery Repairer	Battery adapter received, used and described the same, easy to use, versatile, good things are not expensive, value for money, praise10points	JD.com
Mazda Motor Corporation	Battery Repairer	It doesn't work. It's a lie.	JD.com
Mazda Motor Corporation	Battery Repairer	Goods have been received, feel good, first try a period of time, if the effect is good then come back!	JD.com
Mazda Motor Corporation	Battery Repairer	Pro, the goods have been received, exactly as you said, brought home to try, the overall feeling of the effect is very good, the speed of the phone has become faster, the quality of goods is very good, give five-star praise!	JD.com
Mazda Motor Corporation	Battery Repairer	With great hope, the phone suddenly does not turn on the phone Anzhao requirements docking good charging point3, a little response is not, a little bit of electricity, how could it happen? Boss bragging🐂right?	JD.com
Mazda Motor Corporation	Battery Repairer	Just received, immediately use, looks like it is, genuine, looking forward to the effect, really with voice mode[yay]	JD.com
Mazda Motor Corporation	Battery Repairer	Beautiful appearance, small sparrow,, easy to use, clear display, economical and practical, easy to carry	JD.com
Mazda Motor Corporation	Battery Repairer	Received the goods and looked at the goods is very good next time to buy!	JD.com
Mazda Motor Corporation	Battery Repairer	Logistics is fast	JD.com
Mazda Motor Corporation	Battery Repairer	It's very sensitive! Plugged it into a fast charger that I just unplugged from the plug and it voiced out. I hope it works as well as the merchant promised!	JD.com
Mazda Motor Corporation	Battery Repairer	Let's use it first.	JD.com

Mazda Motor Corporation	Battery Repairer	The battery restorer was received in perfect condition and is satisfactory.	JD.com
Mazda Motor Corporation	Battery Repairer	On trial.	JD.com
Mazda Motor Corporation	Battery Repairer	Goods received as in the picture, logistics is very fast!	JD.com
Mazda Motor Corporation	Battery Repairer	I have received the goods, how effective, after using it to say!	JD.com
Mazda Motor Corporation	Battery Repairer	Good quality, authentic, reasonable price, worth buying!	JD.com
Mazda Motor Corporation	Battery Repairer	Good!	JD.com
Mazda Motor Corporation	Battery Repairer	The goods have been received, well packaged exquisite, immediately used, waiting for the effect, hope it will be good. First give a favorable comment!	JD.com
Mazda Motor Corporation	Battery Repairer	It's already charged.	JD.com
Mazda Motor Corporation	Battery Repairer	or not, first give five stars to the follow-up management evaluation	JD.com
Mazda Motor Corporation	Battery Repairer	This user feels the product is better	JD.com
Mazda Motor Corporation	Battery Repairer	received	JD.com
Mazda Motor Corporation	Battery Repairer	Item received, cell phone battery adapter repairer, logistics is very block, service is very good, thank you,!	JD.com
Mazda Motor Corporation	Battery Repairer	Cell phone battery repairer received, fast delivery, thank you!	JD.com
Mazda Motor Corporation	Battery Repairer	This user feels the product is better	JD.com
Mazda Motor Corporation	Battery Repairer	This user feels the product is better	JD.com
Mazda Motor Corporation	Battery Repairer	The quality is quite good and the logistics is fast, praise!	JD.com
Mazda Motor Corporation	Battery Repairer	It doesn't work, it charges very slowly and doesn't do much.	JD.com
Mazda Motor Corporation	Battery Repairer	very happy	JD.com
Mazda Motor Corporation	Battery Repairer	restorer has been received, not sure how well it works[smile][smile][smile][smile	JD.com
Mazda Motor Corporation	Battery Repairer	This user feels the product is better	JD.com
Mazda Motor Corporation	Battery Repairer	connected to the repairer to charge the phone, there are voice prompts. Repair effect use a period of time to say. Good review!	JD.com
Mazda Motor Corporation	Battery Repairer	Repairer received, small and exquisite, now the technology is awesome, so small can repair the cell phone battery, look forward to the effect!	JD.com
Mazda Motor Corporation	Battery Repairer	Cell phone repair device received,, the price is cheap, good quality, quality is guaranteed, the repair device battery is good, the phone is fast effect is very good, with the repair device with the phone becomes new, logistics services are very fast, worthy of praise, I hope that we all go to buy!	JD.com
Mazda Motor Corporation	Battery Repairer	Very good quality recommended purchase	JD.com
Mazda Motor Corporation	Battery Repairer	I don't know if it works or not. I'll give it a try.	JD.com
Mazda Motor Corporation	Battery Repairer	[Big Love][Cool]	JD.com
Mazda Motor Corporation	Battery Repairer	Big brand, with a very assured, the quality is very good, in time for the event to buy very cost,customer service is really very good Oh ~!	JD.com
Mazda Motor Corporation	Battery Repairer	This is a great product at a great price!	JD.com
Mazda Motor Corporation	Battery Repairer	🆒It's authentic, it's the same as the ones you buy in the store, it's easy and fast to buy online here, I'm assured of the quality, I'll be back!!.^◡^.,🌈quality is very good, buy very much to my liking💕, awesome! ✨ Pros who need it can rest assured to buy, give me five stars!	JD.com
Mazda Motor Corporation	Battery Repairer	It doesn't seem to be any different than a regular charger.	JD.com
Mazda Motor Corporation	Battery Repairer	received	JD.com
Mazda Motor Corporation	Battery Repairer	This user feels the product is better	JD.com
Mazda Motor Corporation	Battery Repairer	an excellent	JD.com
Mazda Motor Corporation	Battery Repairer	Fine workmanship, fast logistics, to be used and then follow up!	JD.com

Mazda Motor Corporation	Battery Repairer	Received the goods, should be genuine, looking forward to the effect. Five-star praise.	JD.com
Mazda Motor Corporation	Battery Repairer	Charge repair device has received the goods today is genuine, above the anti-counterfeiting code by, over the charging trial does have the effect of repairing the cell phone battery than before there is a significant continuation of the long and very practical recommended to buy!	JD.com
Mazda Motor Corporation	Battery Repairer	Just received, the product looks very good, manuals, certificates of conformity warranty card all complete, the key logistics is quite fast! First use to see, later follow-up.	JD.com
Mazda Motor Corporation	Battery Repairer	After plugging it in, it will also voice broadcast, really full of technology. Customer service attitude is also very good, to buy a restorer recommended to buy this one. Really good.	JD.com
Mazda Motor Corporation	Battery Repairer	This merchant's stuff works great, my cell phone was fixed to the point where it can last2days before charging.it had to be charged2timesa day, thanks to the merchant for producing such a great product. Giving you5star review.	JD.com
Mazda Motor Corporation	Battery Repairer	Looks great, plugs in and talks as a reminder. The repair effect is very good, worth buying. The logistics is fast and the quality is very good.	JD.com
Mazda Motor Corporation	Battery Repairer	Husband's razor every time after shaving on no power, see online video introduction razor battery can also be repaired on the buy a back to his razor with a look, not to mention the use of repair a few days, the battery is really durable quite a lot. Technology products I want to recommend it.	JD.com
Mazda Motor Corporation	Battery Repairer	Looks great, restorer works great, been using it for a while. Only today to evaluate. Fast logistics, good customer service. The price is affordable.	JD.com
Mazda Motor Corporation	Battery Repairer	After looking at many, I finally chose this one and it didn't disappoint, significantly improved the durability of my batteries, great quality and easy after sales. I didn't make a mistake. If you want to buy it, I suggest you to get it.	JD.com
Mazda Motor Corporation	Battery Repairer	repairer to try, really can repair the battery capacity. Customer service attitude is also good. A good shopping experience.	JD.com
Mazda Motor Corporation	Battery Repairer	The goods received, the appearance of small and beautiful, with voice reminder start function. The main thing is that it also solves my cell phone lag problem. Really good.	JD.com
Mazda Motor Corporation	Battery Repairer	I've been using it for a week now, my phone is not as stuck as it was before, this fixer still works. Don't hesitate to buy it, I tested it myself. The quality is also very good.	JD.com
Mazda Motor Corporation	Battery Repairer	recent618activities to hand as long as26decisively bought, arrived at home and immediately measured. Indeed good use. The quality is great.	JD.com
Mazda Motor Corporation	Battery Repairer	The product looks great, good quality, nice appearance, especially when plugged in with LCD display and voice announcement function. It is friendly to our elderly.	JD.com
Mazda Motor Corporation	Battery Repairer	Just got it and used it, great quality and fast logistics. Let's see when I get my phone fixed. Special	JD.com
Mazda Motor Corporation	Battery Repairer	I bought two from him and they both look beautiful. One can display numbers to detect current and voltage, and one has a liquid crystal display and voice announcement. All high-tech products. I'll try to see which one has the best repair effect. Tough courier boy to my door, good service attitude.	JD.com
Mazda Motor Corporation	Battery Repairer	merchant said this effect is more powerful decisively bought this, get the hand feeling is still good, the sparrow is small, all the organs are complete. Tried my cell phone that could not be turned on after charging for a night really turned on. It's amazing!	JD.com
Mazda Motor Corporation	Battery Repairer	This is black, with my phone charger is not very match if the business more production a few colors to choose from would be good. But the quality is very good, the repair effect is very good, buy not lose, the service attitude is also very good.	JD.com
Mazda Motor Corporation	Battery Repairer	cheaper than physical stores. I think it is very good, I am very satisfied!	JD.com
Mazda Motor Corporation	Battery Repairer	of6boughtbusiness did not give me to count cheaper, it looks like the quality is still very good, I hope the effect will not let us down. Good use I give colleagues to help you recommend, give me to count cheaper!	JD.com
Mazda Motor Corporation	Battery Repairer	logistics, such a store is worth recommending. Next time to buy	JD.com
Mazda Motor Corporation	Battery Repairer	Just received, logistics speed is very fast, will go back to try to see if it is good. Look at the evaluation is very good quality, I hope the effect is very good.	JD.com

Mazda Motor Corporation	Battery Repairer	advantage communicate with customer service to give her a replacement payment. The quality of goods is very good. Future merchants shipping to pay more attention, not meet me you lose.	JD.com
Mazda Motor Corporation	Battery Repairer	I have to love, this product is really too good to say, I have to love, the small appearance is beautiful as always, I have to love, the repair effect shows immediately. Logistics is fast, the quality is great.	JD.com
Mazda Motor Corporation	Battery Repairer	The repair works great, my cell phone that wouldn't turn on turned on. Tomorrow, I will fix my dad's cell phone too. Thanks to the scientists who have made so many high-tech products to make our life more and more convenient.	JD.com
Mazda Motor Corporation	Battery Repairer	The item was received, well packaged, fast logistics and good quality. I think it's worth buying. The effect is added after use. First full,5points praise.	JD.com
Mazda Motor Corporation	Battery Repairer	phone in the electric car inside forget to take, a moment who do not know who took it for me. Buy a cell phone to a few thousand dollars, bought this fixer to see if you can help me fix a use. If the repair is good, I want to give you a big praise.	JD.com
Mazda Motor Corporation	Battery Repairer	My cell phone is always stuck and dead, I saw this fixer online, bought it and tried it really solved the problem of lagging for me, it's quite good, it seems I didn't try wrong. The quality is very good.	JD.com
Mazda Motor Corporation	Battery Repairer	The Poundland stuff is pretty good, cheap and reliable. I bought this fixer and it works quite well. Helped me solve the problem of my cell phone card and battery not lasting long.	JD.com
Mazda Motor Corporation	Battery Repairer	conformity manual is complete. There is also the anti-counterfeiting code, I swept is genuine. Quality is very good, I just accidentally fell on the ground to pick up still intact. Need family members can buy	JD.com
Mazda Motor Corporation	Battery Repairer	Product qualification certificate warranty card all complete, seems to be very good quality, absolutely genuine. The price is really high cost-effective. Buy value	JD.com
Mazda Motor Corporation	Battery Repairer	Just went to Fengchao to get back, looks like the quality is very good, logistics is also very fast. Use it to see how it works, and recommend it to you if it works well!	JD.com
Mazda Motor Corporation	Battery Repairer	light is soft and does not sting the eyes. Plugged in at the bedside, you can charge your cell phone while fixing it. It's great.	JD.com
Mazda Motor Corporation	Battery Repairer	asked the business that can be repaired. So I bought it to try. Repair with the repairer repaired for more than a week. A lot longer than before, slowly repair estimates can be restored to the level of the new machine. Give a favorable first	JD.com
Mazda Motor Corporation	Battery Repairer	See next door neighbor's family is using this, she said it works well I asked her to share and buy me one, got it exactly the same as hers. My cell phone usually have a little bit of card with this hope can improve. Overall feel that the delivery speed is very fast, logistics speed, customer service attitude is good, the quality of goods is also good.	JD.com
Mazda Motor Corporation	Battery Repairer	I bought it a long time ago and just used it today. Let's see how it works. Looks like good quality. Just asked customer service how to use it she replied very quickly, helped me solve a lot of unclear problems, give customer service points!	JD.com
Mazda Motor Corporation	Battery Repairer	I bought two at once to compare to see which one works better, and I prefer the clear one for appearance. It looks more fashionable. The black one has a display when plugged in and is more high-end than the transparent one. Logistics is very fast, may be the same city, bought yesterday today arrived.	JD.com
Mazda Motor Corporation	Battery Repairer	store description, fine workmanship, beautiful appearance, easy to carry, economical and practical. The product is on trial looking forward to good results, praise! Logistics worthy of praise, five praise!	JD.com
Mazda Motor Corporation	Battery Repairer	Two repairer received in good condition, unpacking to see, each fine workmanship. Tried all can use, I did not think there is such a tool can repair the phone, really authentic.	JD.com
Mazda Motor Corporation	Battery Repairer	Things received, in the test, the effect is good, will recommend friends to buy, logistics is very fast also send a year warranty, cost-effective.	JD.com
Mazda Motor Corporation	Battery Repairer	relatively fine workmanship, logistics is quite strong. Good reviews, good reviews!	JD.com
Mazda Motor Corporation	Battery Repairer	Cell phone electricity does not last long, bought this repairer, used it has some effect, worth recommending.	JD.com
Mazda Motor Corporation	Battery Repairer	Simple to use and great repair. Buy no regrets. Logistics is also very fast	JD.com
Mazda Motor Corporation	Battery Repairer	platform service attitude is excellent, shopping on the platform6years. Haven't bought a single thing I regret. Kudos to the platform.	JD.com

Mazda Motor Corporation	Battery Repairer	been used once according to the instructions, and I feel that the phone is not as stuck as before, and I bought two at a time, and both of them work very well. Checked that security code is genuine.	JD.com
Mazda Motor Corporation	Battery Repairer	Received the goods, very satisfied! The previous phone with aging battery is finally saved! Technology changes life. Finally bought the baby that I like. Give 5stars praise!	JD.com
Mazda Motor Corporation	Battery Repairer	Nowadays, there are more and more high-tech products, and the cell phone battery is old and useless. Unintentionally found this tech product, looking forward to give my cell phone repair as before. The logistics speed is very fast.	JD.com
Mazda Motor Corporation	Battery Repairer	the battery can play a role in repairing, I feel that after charging with your home repairer is more durable. Looking forward to the phone back as new. Good reviews! Logistics is very fast worth praise	JD.com
Mazda Motor Corporation	Battery Repairer	you get it. How well the repair works will be evaluated later. Because just received. Logistics is very fast. I am very satisfied.	JD.com
Mazda Motor Corporation	Battery Repairer	It's fake. It doesn't work after a day or two.	JD.com
Mazda Motor Corporation	Battery Repairer	This cell phone protector is good and gives 5 stars[kudos][kudos[kudos[kudos] [kudos] [kudos] [kudos] [kudos] [kudos] [kudos] [kudos] [kudos] [kudos] [kudos] [kudos][kudos	JD.com
Mazda Motor Corporation	Battery Repairer	Works great Works Phone doesn't get hot anymore	JD.com
Mazda Motor Corporation	Battery Repairer	Good quality products, worth buying	JD.com
Mazda Motor Corporation	Battery Repairer	The goods have been received, the language broadcast, the repair is available.	JD.com
Mazda Motor Corporation	Battery Repairer	I just got it. I'll comment when I use it.	JD.com
Mazda Motor Corporation	Battery Repairer	Charging is very stable, very fast, good quality, fast logistics, worth having	JD.com
Mazda Motor Corporation	Battery Repairer	This user feels the product is better	JD.com
Mazda Motor Corporation	Battery Repairer	This user feels the product is better	JD.com
Mazda Motor Corporation	Battery Repairer	Being used, first5stars praise, wish the merchant business prosperity! Prosperity!	JD.com
Mazda Motor Corporation	Battery Repairer	This user feels the product is better	JD.com
Mazda Motor Corporation	Battery Repairer	To be used and then evaluated.	JD.com
Mazda Motor Corporation	Battery Repairer	Fast shipping, good packaging, good quality, worth buying.	JD.com
Mazda Motor Corporation	Battery Repairer	Battery Repairer received, fast logistics, repairer I used three days before giving positive feedback, really good, before the phone charging hot, now the phone charging is not hot, and feel the phone running faster than before, not long, not a long time, not a circle, the repairer is genuine, but also has a true and false identification code, along with the purchase. If you need it, order it quickly!	JD.com
Mazda Motor Corporation	Battery Repairer	This user feels the product is better	JD.com
Mazda Motor Corporation	Battery Repairer	Genuine, cheaper than other stores, trustworthy, looking forward to the effect, five stars!	JD.com
Mazda Motor Corporation	Battery Repairer	This user feels the product is better	JD.com
Mazda Motor Corporation	Battery Repairer	Feeling it out.	JD.com
Mazda Motor Corporation	Battery Repairer	This user feels the product is better	JD.com
Mazda Motor Corporation	Battery Repairer	This user feels the product is better	JD.com
Mazda Motor Corporation	Battery Repairer	This user feels the product is better	JD.com
Mazda Motor Corporation	Battery Repairer	an excellent	JD.com
Mazda Motor Corporation	Battery Repairer	The product is very good! Like the description in the picture, the delivery is also fast and well packaged, the business is very serious about what they do and are willing to work together often!😉😉	JD.com
Mazda Motor Corporation	Battery Repairer	This user feels the product is better	JD.com
Mazda Motor Corporation	Battery Repairer	can	JD.com
Mazda Motor Corporation	Battery Repairer	I got it. Thank you![Smiles]	JD.com

Mazda Motor Corporation	Battery Repairer	Used it for a few days with no results returned it.	JD.com
Mazda Motor Corporation	Battery Repairer	Fast logistics is genuine	JD.com
Mazda Motor Corporation	Battery Repairer	This user feels the product is better	JD.com
Mazda Motor Corporation	Battery Repairer	Logistics quickly delivered, the quality is good, the next purchase[cool]	JD.com
Mazda Motor Corporation	Battery Repairer	Good for you.	JD.com
Mazda Motor Corporation	Battery Repairer	Roger that. - Thanks.	JD.com
Mazda Motor Corporation	Battery Repairer	It doesn't help at all.	JD.com
Mazda Motor Corporation	Battery Repairer	It's working!	JD.com
Mazda Motor Corporation	Battery Repairer	This user feels the product is better	JD.com
Mazda Motor Corporation	Battery Repairer	This user feels the product is better	JD.com
Mazda Motor Corporation	Battery Repairer	This user feels the product is better	JD.com
Mazda Motor Corporation	Battery Repairer	I received the goods, I like it, very satisfied, very reputable.	JD.com
Mazda Motor Corporation	Battery Repairer	Goods have been received logistics quickly and good service attitude, affordable and good value for money	JD.com
Mazda Motor Corporation	Battery Repairer	This user feels the product is better	JD.com
Mazda Motor Corporation	Battery Repairer	This user feels the product is better	JD.com
Mazda Motor Corporation	Battery Repairer	Received it quickly by courier, the restorer is really nice and good quality, nice stuff!	JD.com
Mazda Motor Corporation	Battery Repairer	Just received the goods have not yet used it, do not know the effect of how it, first give a favorable comment	JD.com
Mazda Motor Corporation	Battery Repairer	Charger received thanks to the delivery.	JD.com
Mazda Motor Corporation	Battery Repairer	Too good 。。。。。。。。。。。。。。。。。。。。。。。 It's so good.	JD.com
Mazda Motor Corporation	Battery Repairer	Received, good, is genuine, thanks to the merchants	JD.com
Mazda Motor Corporation	Battery Repairer	I've received the goods, I'll use it right away	JD.com
Mazda Motor Corporation	Battery Repairer	This user feels the product is better	JD.com
Mazda Motor Corporation	Battery Repairer	I'm satisfied. Thank you.	JD.com
Mazda Motor Corporation	Battery Repairer	Product quality is very good, please friends rest assured to buy!	JD.com
Mazda Motor Corporation	Battery Repairer	Enthusiastic service and fast delivery, just received the goods to thank the courier boy you worked hard!	JD.com
Mazda Motor Corporation	Battery Repairer	Received the goods today, very good to use, logistics is very fast, charging is very good to use, next time to buy again	JD.com
Mazda Motor Corporation	Battery Repairer	This user feels the product is better	JD.com
Mazda Motor Corporation	Battery Repairer	This user feels the product is better	JD.com
Mazda Motor Corporation	Battery Repairer	This user feels the product is better	JD.com
Mazda Motor Corporation	Battery Repairer	This user feels the product is better	JD.com
Mazda Motor Corporation	Battery Repairer	very happy	JD.com
Mazda Motor Corporation	Battery Repairer	Cell phone battery repairer received, look good, good quality, like.	JD.com
Mazda Motor Corporation	Battery Repairer	This user feels the product is better	JD.com
Mazda Motor Corporation	Battery Repairer	very happy	JD.com
Mazda Motor Corporation	Battery Repairer	The goods have been received, the logistics is very fast, the packaging is very good, give a favorable comment!	JD.com
Mazda Motor Corporation	Battery Repairer	Received the goods, the night off to try to see to see before giving a favorable review	JD.com
Mazda Motor Corporation	Battery Reconditioner	Nice product, very solid, much better than my last purchase, I'm very happy with it, it's worth buying!	JD.com
Mazda Motor Corporation	Battery Reconditioner	effective	JD.com
Mazda Motor Corporation	Battery Reconditioner	Second time I put together an order, works great, good value, next time	JD.com

Mazda Motor Corporation	Battery Reconditioner	I've bought this stuff before, it's advertised by Coat, features a very noticeable effect, but it's good for battery action, so I'll buy a second coat this time and hope it's a little better than last time.	JD.com
Mazda Motor Corporation	Battery Reconditioner	The product has a beautiful appearance, good charging functionality and fast logistics.	JD.com
Mazda Motor Corporation	Battery Reconditioner	Battery repair in progress! Let's see how it works!	JD.com
Mazda Motor Corporation	Battery Reconditioner	Not the same as the video's, didn't feel effective	JD.com
Mazda Motor Corporation	Battery Reconditioner	's not working. It's getting less and less[Stammering]	JD.com
Mazda Motor Corporation	Battery Reconditioner	Logistics was fast during the epidemic and the goods have been received. Trial and then follow up. Thank you boss!	JD.com
Mazda Motor Corporation	Battery Reconditioner	Baby received, well packaged, five stars!	JD.com
Mazda Motor Corporation	Battery Reconditioner	This user did not fill in a rating	JD.com
Mazda Motor Corporation	Battery Reconditioner	Count me unlucky, bought this thing want to cell phone better, do not use it cell phone a moment full of electricity, use directly after the cell phone to get a half-finished, a night charging38%every day, cell phone electricity charging is not full.[Love] [Love] [Love]Love[Love] [Love] [Love] [Love][Love	JD.com
Mazda Motor Corporation	Battery Reconditioner	Why'tchargeto80%?What's the reasonI can chargeto 100%before I start using it?	JD.com
Mazda Motor Corporation	Battery Reconditioner	Very satisfied!	JD.com
Mazda Motor Corporation	Battery Reconditioner	The results are okay, with an increase in range	JD.com
Mazda Motor Corporation	Battery Reconditioner	Digital display Convenient and easy to use	JD.com
Mazda Motor Corporation	Battery Reconditioner	I received the goods, but I won't know how it works until I use it.	JD.com
Mazda Motor Corporation	Battery Reconditioner	Item has been received and is a great value for the money.	JD.com
Mazda Motor Corporation	Battery Reconditioner	This product is good, repairing the battery automatically, logistics is particularly fast	JD.com
Mazda Motor Corporation	Battery Reconditioner	The baby has received, quite a good online shopping	JD.com
Mazda Motor Corporation	Battery Reconditioner	The price is very beautiful, the stuff is still good and will continue to repurchase! All five points! Nessie Nessie~	JD.com
Mazda Motor Corporation	Battery Reconditioner	It's okay.	JD.com
Mazda Motor Corporation	Battery Reconditioner	clear display	JD.com
Mazda Motor Corporation	Battery Reconditioner	It doesn't work. It doesn't work at all.	JD.com
Mazda Motor Corporation	Battery Reconditioner	serviceable	JD.com
Mazda Motor Corporation	Battery Reconditioner	It's high quality. It's good.	JD.com
Mazda Motor Corporation	Battery Reconditioner	It's working well, the power feels like it's taking longer.	JD.com
Mazda Motor Corporation	Battery Reconditioner	The items work well.	JD.com
Mazda Motor Corporation	Battery Reconditioner	There is no repair battery male energy.	JD.com
Mazda Motor Corporation	Battery Reconditioner	The store's products are as advertised and are of good quality give a shout out!	JD.com
Mazda Motor Corporation	Battery Reconditioner	effect of the Huawei phone self-test did not show the replacement of the battery. Previously there has been a display to replace the battery	JD.com
Mazda Motor Corporation	Battery Reconditioner	not bad	JD.com
Mazda Motor Corporation	Battery Reconditioner	Just like the store said.	JD.com
Mazda Motor Corporation	Battery Reconditioner	The message on my cell phone prompting for a battery change is gone for now! My super fast charger turned into a slow charger, how long will it take to fix it! Will add more later!	JD.com
Mazda Motor Corporation	Battery Reconditioner	wait for the repair to take place for a while to see if it works well.	JD.com
Mazda Motor Corporation	Battery Reconditioner	Immediately used, good, really a good, current and voltage display clearly, praise must.	JD.com
Mazda Motor Corporation	Battery Reconditioner	Good quality, fast shipping, great service, very satisfied!	JD.com
Mazda Motor Corporation	Battery Reconditioner	Logistics is very fast, product quality is good, good price, very good, everyone quickly come to the sobuy it!	JD.com
Mazda Motor Corporation	Battery Reconditioner	The quality of the goods is very good, and the logistics is also very fast	JD.com

Mazda Motor Corporation	Battery Reconditioner	holiday rushed on the phone will still be hot, I use 3times not dry use, so I want a refund, please give support to the platform, thank you for the platform support thank you ！！！！！！！！！！！！.	JD.com
Mazda Motor Corporation	Battery Reconditioner	It's just an IQ tax.	JD.com
Mazda Motor Corporation	Battery Reconditioner	It could be even better than I thought.	JD.com
Mazda Motor Corporation	Battery Reconditioner	No effect at all.	JD.com
Mazda Motor Corporation	Battery Reconditioner	Charging speed: pretty fast and safe	JD.com
Mazda Motor Corporation	Battery Reconditioner	Very good, small and handy, come in handy, easy to carry, easy to use, at a glance, praise.	JD.com
Mazda Motor Corporation	Battery Reconditioner	Display experience: good, worth buying oh	JD.com
Mazda Motor Corporation	Battery Reconditioner	Very good and very satisfied with the logistics is also very fast good quality cheap price	JD.com
Mazda Motor Corporation	Battery Reconditioner	Five stars, why don't you reply?	JD.com
Mazda Motor Corporation	Battery Reconditioner	It doesn't help at all.	JD.com
Mazda Motor Corporation	Battery Reconditioner	Quality is good wait to use it and then come back to the review	JD.com
Mazda Motor Corporation	Battery Reconditioner	an excellent	JD.com
Mazda Motor Corporation	Battery Reconditioner	I don't know if it works even after I've used it!	JD.com
Mazda Motor Corporation	Battery Reconditioner	relatively fine, just started to use, do not know how the effect. Logistics is also very fast, the habit of five-star praise!	JD.com
Mazda Motor Corporation	Battery Reconditioner	Not as useful as described	JD.com
Mazda Motor Corporation	Battery Reconditioner	very fast, makingZespecially exquisite, charging especially fast! The display shows fast. Repair while charging, hope the repair effect is excellent, really like it. Wish my husband a prosperous business!	JD.com
Mazda Motor Corporation	Battery Reconditioner	Very good very good, fast logistics, good service attitude	JD.com
Mazda Motor Corporation	Battery Reconditioner	The charger has been received very well	JD.com
Mazda Motor Corporation	Battery Reconditioner	I have received it and I have praised it.	JD.com
Mazda Motor Corporation	Battery Reconditioner	Product use effect is good, the beginning is not familiar with the use of the method, the store timely, serious guidance, the product is indeed good.	JD.com
Mazda Motor Corporation	Battery Reconditioner	good quality, more convenient to use, inexpensive, this thing also has the role of repairing the battery, well-packaged, good service attitude, good stuff, logistics is very fast, it is worth buying.	JD.com
Mazda Motor Corporation	Battery Reconditioner	a good, good, after the cell phone battery compound durable, thanks to the store wishing the store business prosperity, prosperity.	JD.com
Mazda Motor Corporation	Battery Reconditioner	Received it, unwrapped it to try it out, don't know how well it works, some small expectations.	JD.com
Mazda Motor Corporation	Battery Reconditioner	Received, satisfied, waiting to use to see results.	JD.com
Mazda Motor Corporation	Battery Reconditioner	It's not expensive and it works well.	JD.com
Mazda Motor Corporation	Battery Reconditioner	This product is genuine and works as advertised!	JD.com
Mazda Motor Corporation	Battery Reconditioner	Zhongtong Express Delivery Good service The quality of the goods is quite good.	JD.com
Mazda Motor Corporation	Battery Reconditioner	Good quality, satisfied!	JD.com
Mazda Motor Corporation	Battery Reconditioner	Nice as described 5 out of 5!	JD.com
Mazda Motor Corporation	Battery Reconditioner	"Charging is very good very fast logistics is also very fast convenient and can repair the cell phone battery	JD.com
Mazda Motor Corporation	Battery Reconditioner	It worked after one use.	JD.com
Mazda Motor Corporation	Battery Reconditioner	Arrived in a timely manner, through the use of the quality is quite good, attentive service, please feel free to buy.	JD.com
Mazda Motor Corporation	Battery Reconditioner	pretty good	JD.com
Mazda Motor Corporation	Battery Reconditioner	As soon as I see it, I'll buy it. Good review. Good.	JD.com
Mazda Motor Corporation	Battery Reconditioner	No visible effect	JD.com

Mazda Motor Corporation	Battery Reconditioner	[smiles][smiles][smiles][smiles]	JD.com
Mazda Motor Corporation	Battery Reconditioner	It works.	JD.com
Mazda Motor Corporation	Battery Reconditioner	The product is quite good, the use of safe and reliable, clear display, workmanship is relatively fine, very good, too good!	JD.com
Mazda Motor Corporation	Battery Reconditioner	Exquisite appearance of the product, transparent digital display, display voltage and current changes during charging.	JD.com
Mazda Motor Corporation	Battery Reconditioner	(of an unmarried couple) be close	JD.com
Mazda Motor Corporation	Battery Reconditioner	Goods received logistics is also very fast very good, good value for money very good worth having! Great!	JD.com
Mazda Motor Corporation	Battery Reconditioner	Express received, tried, very good, logistics is very fast, thank you boss!	JD.com
Mazda Motor Corporation	Battery Reconditioner	[Clasping fists]	JD.com
Mazda Motor Corporation	Battery Reconditioner	The product has been received, test charging a few days before evaluation. This product does repair the battery, should be the more you charge the more you repair the battery, also economical and cheap, worth buying!	JD.com
Mazda Motor Corporation	Battery Reconditioner	Baby has been received, very satisfied, want to get it.	JD.com
Mazda Motor Corporation	Battery Reconditioner	If you use it and see that it's good, you'll introduce other friends to buy it.	JD.com
Mazda Motor Corporation	Battery Reconditioner	Very good, very happy with the service of this product.	JD.com
Mazda Motor Corporation	Battery Reconditioner	IQ tax, not useful at all, just a charging current voltmeter, whoever buys it will be fooled	JD.com
Mazda Motor Corporation	Battery Reconditioner	I received the goods and it should be fine.	JD.com
Mazda Motor Corporation	Battery Reconditioner	Received the goods, tried it today and it's good.	JD.com
Mazda Motor Corporation	Battery Reconditioner	I think it's okay[Smiles][Smiles][SmilesSmilesSmilesSmiles][Smiles]	JD.com
Mazda Motor Corporation	Battery Reconditioner	This user feels the product is better	JD.com
Mazda Motor Corporation	Battery Reconditioner	Quite good, do not know if it can repair the battery, is using, can show the charging voltage and current.	JD.com
Mazda Motor Corporation	Battery Reconditioner	it can repair the battery use it for a while and then follow up.	JD.com
Mazda Motor Corporation	Battery Reconditioner	Good. Practical.	JD.com
Mazda Motor Corporation	Battery Reconditioner	is as durable battery as the new one, it is worth recommending!	JD.com
Mazda Motor Corporation	Battery Reconditioner	Trying it out. I don't know if it's working or not.	JD.com
Mazda Motor Corporation	Battery Reconditioner	It's easy to use.	JD.com
Mazda Motor Corporation	Battery Reconditioner	Cell phone repairer has been received, do not know how effective, just use it today, if it is good, will recommend it to everyone, let us look forward to him that effect.	JD.com
Mazda Motor Corporation	Battery Reconditioner	The product received, with the more fruit how, do not know yet, thanks!	JD.com
Mazda Motor Corporation	Battery Reconditioner	pretty good	JD.com
Mazda Motor Corporation	Battery Reconditioner	The display is a little dim, but it works	JD.com
Mazda Motor Corporation	Battery Reconditioner	Works great, must be 5 stars	JD.com
Mazda Motor Corporation	Battery Reconditioner	Packaging is good, logistics is very fast, the specific function, to be used after the follow-up comment.	JD.com
Mazda Motor Corporation	Battery Reconditioner	This user feels the product is better	JD.com
Mazda Motor Corporation	Battery Reconditioner	The product was received and used! Very good!	JD.com
Mazda Motor Corporation	Battery Reconditioner	Works great and worth the money.	JD.com
Mazda Motor Corporation	Battery Reconditioner	The item was received and the results are yet to be proven.	JD.com
Mazda Motor Corporation	Battery Reconditioner	Battery Reconditioner received Nice color!	JD.com
Mazda Motor Corporation	Battery Reconditioner	It matches the picture and looks so good, so keep coming back for more! Will follow up with a review! Five stars☆☆☆☆☆favorable review! Courier worked hard to deliver to the home five stars☆☆☆☆☆praise	JD.com
Mazda Motor Corporation	Battery Reconditioner	This product works well and the logistics is fast, genuine.	JD.com
Mazda Motor Corporation	Battery Reconditioner	Roger that. I'm gonna try it out first.	JD.com

Mazda Motor Corporation	Battery Reconditioner	Safety: did not use after or give yourself before the same	JD.com
Mazda Motor Corporation	Battery Reconditioner	Cheap and affordable, good for batteries, optimized batteries worth buying	JD.com
Mazda Motor Corporation	Battery Reconditioner	Goods received, fast logistics, packaging intact, whether it is the same as described, after the use of follow-up comments, I wish the boss a prosperous business.	JD.com
Mazda Motor Corporation	Battery Reconditioner	Product received An customer service said the requirements Try for a period of time Whether there is a repair function Not quite sure Customer service is surely useful I hope it works The logistics is fast The service is good!	JD.com
Mazda Motor Corporation	Battery Reconditioner	Received last night, immediately connected to the phone charged, today the phone battery use feel longer, before the phone battery a day are not used. This thing can really extend the life of the battery, good.	JD.com
Mazda Motor Corporation	Battery Reconditioner	Nice baby and fast shipping.	JD.com
Mazda Motor Corporation	Battery Reconditioner	The product is of high quality, easy to use and durable.	JD.com
Mazda Motor Corporation	Battery Reconditioner	Logistics is very fast, have not yet started to use, hope that like the description of the match!	JD.com
Mazda Motor Corporation	Battery Reconditioner	good! Good quality! Beautiful modeling! Easy to use! The effect is very good! Charging and repairing both! Worth buying!	JD.com
Mazda Motor Corporation	Battery Reconditioner	[点赞][点赞][点赞][点赞][点赞]，做工比较精细，显示清晰	JD.com
Mazda Motor Corporation	Battery Reconditioner	Why not?	JD.com
Mazda Motor Corporation	Battery Reconditioner	for the use of good and then come back to the follow-up, first praise,	JD.com
Mazda Motor Corporation	Battery Reconditioner	It's okay!	JD.com
Mazda Motor Corporation	Battery Reconditioner	charging display, charging is more intuitive. There are so many varieties on the market. I have also used for many years. Plug this not only can display the voltage and current, but also can improve the DC ripple and continuous pulse to prevent overcharging. At present, some better cell phones, tablets, players, etc. are with internal charging protection, generally do not have to worry about overcharging! There is something to be said for charging. My old players and tablets from more than a decade ago are outdated and the software can't be upgraded, but I still generally have no problem reading, watching videos, listening to music, taking notes, etc. Sales promotion over-exaggerate the function of goods is flashy! And the real thing is not voltage and current dual display!	JD.com
Mazda Motor Corporation	Battery Reconditioner	Fast logistics, good quality, good service attitude	JD.com
Mazda Motor Corporation	Battery Reconditioner	It's better looking. It seems to be working.	JD.com
Mazda Motor Corporation	Battery Reconditioner	Really great boutique rechargeable restorers that work quite well and ship quickly, super awesome 👍	JD.com
Mazda Motor Corporation	Battery Reconditioner	Roger that, thanks!	JD.com
Mazda Motor Corporation	Battery Reconditioner	was good, and I am currently trying it out to see how it works.	JD.com
Mazda Motor Corporation	Battery Reconditioner	Cell Phone Fixer is great!	JD.com
Mazda Motor Corporation	Battery Reconditioner	exquisite, looking forward to good results, give a five-star praise.	JD.com
Mazda Motor Corporation	Battery Reconditioner	Not bad, used on the field, cell phone battery repair rely on it	JD.com
Mazda Motor Corporation	Battery Reconditioner	Looks good, I'll follow up after I use it to see how it works.	JD.com
Mazda Motor Corporation	Battery Reconditioner	The product has been received and is being used to see the results before evaluating.	JD.com
Mazda Motor Corporation	Battery Reconditioner	Display experience: good<br>Charging function: good<br>Product appearance: good	JD.com
Mazda Motor Corporation	Battery Reconditioner	Packaging is very good, logistics is very fast, has been used	JD.com
Mazda Motor Corporation	Battery Reconditioner	acceptable	JD.com
Mazda Motor Corporation	Battery Reconditioner	Good Very good	JD.com
Mazda Motor Corporation	Battery Reconditioner	Logistics is very fast, the quality is good, the display number is clear, used in the follow-up comment.	JD.com
Mazda Motor Corporation	Battery Reconditioner	Restorer(cellphone/dynamics received, thanks.	JD.com

Mazda Motor Corporation	Battery Reconditioner	Received the goods are quite satisfied with a shopping	JD.com
Mazda Motor Corporation	Battery Reconditioner	Good packaging, good workmanship, good repair, very good quality, very satisfied	JD.com
Mazda Motor Corporation	Battery Reconditioner	Received and satisfied.	JD.com
Mazda Motor Corporation	Battery Reconditioner	Voltage and current are displayed alternately, can not repair the battery, we will see the effect later.	JD.com
Mazda Motor Corporation	Battery Reconditioner	This stuff is great, I love it.	JD.com
Mazda Motor Corporation	Battery Reconditioner	Very good Oh good After all, a penny a penny Super satisfied[big love][big love][big love][big love][big love	JD.com
Mazda Motor Corporation	Battery Reconditioner	There is some restorative effect, and at 100% it can take a long time to become	JD.com
Mazda Motor Corporation	Battery Reconditioner	Goods received and used for a few days, the battery charging faster, effective, positive feedback	JD.com
Mazda Motor Corporation	Battery Reconditioner	I used it today for one day but I don't know the effect, I'll tell you in a few days.	JD.com
Mazda Motor Corporation	Battery Reconditioner	It's okay. It's good.	JD.com
Mazda Motor Corporation	Battery Reconditioner	The cell phone repairer works pretty well, after using it twice, the phone works much better, and charging it once lasts all day. Previously have not purchased a cell phone repairer, a day to charge a few times. Cell phone repairer is not expensive, use the effect is very good Oh!	JD.com
Mazda Motor Corporation	Battery Reconditioner	Good workmanship legible handwriting	JD.com
Mazda Motor Corporation	Battery Reconditioner	Plugging this in feels like the charging is slowing down, it should be repairing the battery, then use it afterwards to see if you need it before you get it!	JD.com
Mazda Motor Corporation	Battery Reconditioner	Very intuitive and great value for money	JD.com
Mazda Motor Corporation	Battery Reconditioner	Charged once, feel good, use some time to comment again	JD.com
Mazda Motor Corporation	Battery Reconditioner	Display experience: the quality is very good, logistics is also very fast conscientious business!	JD.com
Mazda Motor Corporation	Battery Reconditioner	aging repair artifact, arrived just used once, for the time being can not see a significant effect, and then use a few times to follow up.	JD.com
Mazda Motor Corporation	Battery Reconditioner	Just received the goods have not yet used it to use a good and then come back to the review.	JD.com
Mazda Motor Corporation	Battery Reconditioner	Charge Repairer received, then used once, the effect is not obvious, due to seven days no reason to return, I will try a few more days, or have the effect, then under it, thank you for the seven days no reason to return, thanks to the live beauty of the patience of the consolation, bless your business is good, the invention of better products.	JD.com
Mazda Motor Corporation	Battery Reconditioner	I got it. It works.	JD.com
Mazda Motor Corporation	Battery Reconditioner	It's here. Thank you.	JD.com
Mazda Motor Corporation	Battery Reconditioner	Things received the logistics very quickly service is very good, using quite good very convenient.	JD.com
Mazda Motor Corporation	Battery Reconditioner	Charging is too slow, it was fast charging.Now it's slow charging.	JD.com
Mazda Motor Corporation	Battery Reconditioner	使用安全可靠，显示清晰，做工比较精细，外观很美，正品无疑，作工精细，手机充电快，容量特别大，实用性非常强，经济实惠，电池相当耐用，体积小，耗电慢，网速快[点赞][点赞][点赞][玫瑰][玫瑰][玫瑰][买买买][买买买][买买买]	JD.com
Mazda Motor Corporation	Battery Reconditioner	it repaired with the new one is really good, logistics fast service.	JD.com
Mazda Motor Corporation	Battery Reconditioner	I don't see it at the moment More on that later	JD.com
Mazda Motor Corporation	Battery Reconditioner	Works well, recommend good stuff.	JD.com
Mazda Motor Corporation	Battery Reconditioner	The cell phone repairer works great and the logistics are fast. It is worth recommending to buy!	JD.com
Mazda Motor Corporation	Battery Reconditioner	business service attitude! Waiting to use it and then follow up.<br>Product appearance: looked similar to the live room!	JD.com
Mazda Motor Corporation	Battery Reconditioner	Just received the goods, plugged in the charger is using, feel very good!	JD.com
Mazda Motor Corporation	Battery Reconditioner	Looks good. Try it.	JD.com
Mazda Motor Corporation	Battery Reconditioner	This user feels the product is better	JD.com
Mazda Motor Corporation	Battery Reconditioner	Item received, can display voltage and current, after using it, then comment.	JD.com

Mazda Motor Corporation	Battery Reconditioner	This user feels the product is better	JD.com
Mazda Motor Corporation	Battery Reconditioner	experiments, the restorer charging normal, not hot, the role of the restorer is not too obvious, but also continue to repair an observation! Logistics are also fast to give five stars praise it!	JD.com
Mazda Motor Corporation	Battery Reconditioner	Good goods, use a few days before evaluation, the effect is good, good value, logistics is very fast.	JD.com
Mazda Motor Corporation	Battery Reconditioner	Not good. Not as advertised.	JD.com
Mazda Motor Corporation	Battery Reconditioner	It's a good product.	JD.com
Mazda Motor Corporation	Battery Reconditioner	serviceable	JD.com
Mazda Motor Corporation	Battery Reconditioner	It's okay, I just don't know how well it's going to fix it yet	JD.com
Mazda Motor Corporation	Battery Reconditioner	Artifacts! Bull! Male alliance!	JD.com
Mazda Motor Corporation	Battery Reconditioner	The logistics is fast, the quality is good, the effect is good.	JD.com
Mazda Motor Corporation	Battery Reconditioner	the display is clear, just use don't know how the effect of it!	JD.com
Mazda Motor Corporation	Battery Reconditioner	I'm already using it and it feels good, yo.	JD.com
Mazda Motor Corporation	Battery Reconditioner	This user feels the product is better	JD.com
Mazda Motor Corporation	Battery Reconditioner	It's working well.	JD.com
Mazda Motor Corporation	Battery Reconditioner	I'll comment after I use it.	JD.com
Mazda Motor Corporation	Battery Reconditioner	Goods received, first five-star praise, to be used after additional evaluation	JD.com
Mazda Motor Corporation	Battery Reconditioner	Works well! Charges fast too!	JD.com
Mazda Motor Corporation	Battery Reconditioner	This repairer can be used only half a day after one charge, but after three repairs, it can now be used for a day.	JD.com
Mazda Motor Corporation	Battery Reconditioner	Beautiful, repair battery no problem, repair cell phone batteries and toy aircraft✈️ battery, are repaired can be used, thanks to the store and more and more to what you want!	JD.com
Mazda Motor Corporation	Battery Reconditioner	Received, logistics and express very quickly, the shopkeeper has a good service attitude, repair beautiful, clear display, worth buying, five-star praise!	JD.com
Mazda Motor Corporation	Battery Reconditioner	Logistics is very fast, packaging is also good, small and exquisite, the ability is not small, plug and play, the effect is very good, it is worth buying!	JD.com
Mazda Motor Corporation	Battery Reconditioner	Goods have been received, five-star praise logistics very quickly, I hope the effect is very good!	JD.com
Mazda Motor Corporation	Battery Reconditioner	The logistics is very fast, customer service is very good, tried it without problems.	JD.com
Mazda Motor Corporation	Battery Reconditioner	Received, all 5 stars.	JD.com
Mazda Motor Corporation	Battery Reconditioner	an excellent	JD.com
Mazda Motor Corporation	Battery Reconditioner	Cell phone battery repairer is not bad, have the effect to buy a few more	JD.com
Mazda Motor Corporation	Battery Reconditioner	Baby received, in kind and the same as the picture, the quality is good, very satisfied with a shopping, logistics is also very strong Oh!	JD.com
Mazda Motor Corporation	Battery Reconditioner	made. Affordable, good value for money. It is a good handle for cell phone users, if needed will continue to buy back, five stars.	JD.com
Mazda Motor Corporation	Battery Reconditioner	battery do not need to change, cell phone do not need to buy. Slow power consumption, fast network speed, purchased it, save big money. I like it so much! Thank you, boss! Wish you good business and more money! If you want to buy one, don't wait to see it!	JD.com
Mazda Motor Corporation	Battery Reconditioner	[Big Love]Bullshit, it doesn't work at all!!!	JD.com
Mazda Motor Corporation	Battery Reconditioner	[smiles][smiles][smiles][smiles]	JD.com
Mazda Motor Corporation	Battery Reconditioner	Not very useful, not obvious, when you buy a toy, the pool is not much money, try it first!	JD.com
Mazda Motor Corporation	Battery Reconditioner	Good value for money and worth buying is a pleasant enough experience	JD.com
Mazda Motor Corporation	Battery Reconditioner	The goods have been received, use it to evaluate	JD.com
Mazda Motor Corporation	Battery Reconditioner	Worked, very useful, numbers keep changing again, will comment later	JD.com

Mazda Motor Corporation	Battery Reconditioner	Tried it out for a day and found that it charges better off. Used it for significantly longer standby time!	JD.com
Mazda Motor Corporation	Battery Reconditioner	The goods have been received, easy to use, the price is affordable, good for cell phone battery, worth having.	JD.com
Mazda Motor Corporation	Battery Reconditioner	The battery repairer has been received, it works well, very affordable easy to use, worth buying.	JD.com
Mazda Motor Corporation	Battery Reconditioner	Logistics is fast service is very good	JD.com
Mazda Motor Corporation	Battery Reconditioner	Safe and reliable to use, clear display, beautiful to look at, works well.	JD.com
Mazda Motor Corporation	Battery Reconditioner	Charge repairer is okay, quite good, good value for money is worth buying, next time to come back to patronize!	JD.com
Mazda Motor Corporation	Battery Reconditioner	I don't know if it works.	JD.com
Mazda Motor Corporation	Battery Reconditioner	Just received the goods. After using it and feeling that it did no harm to my cell phone battery.	JD.com
Mazda Motor Corporation	Battery Reconditioner	Well packaged, good quality, works well	JD.com
Mazda Motor Corporation	Battery Reconditioner	I received it and used it again before commenting	JD.com
Mazda Motor Corporation	Battery Reconditioner	Goods in received, mounted to try very good is genuine, very satisfied.	JD.com
Mazda Motor Corporation	Battery Reconditioner	This user feels the product is better	JD.com
Mazda Motor Corporation	Battery Reconditioner	The display is clear, the workmanship is fine, and it feels OK.	JD.com
Mazda Motor Corporation	Battery Reconditioner	packaging is very delicate, it really opens the door to a new world......The baby has been received, it is what I want, the feel is very good, the quality is also quite good, there is a problem customer service is also very fast to solve the problem, the attitude is very good, it must be praised! Very satisfied😘, the material workmanship is very good, and versatile, feel great, verynice, very practical, praise👍	JD.com
Mazda Motor Corporation	Battery Reconditioner	Good quality, fast logistics, worth buying!	JD.com
Mazda Motor Corporation	Battery Reconditioner	Fast shipping and good quality	JD.com
Mazda Motor Corporation	Battery Reconditioner	Just got it.	JD.com
Mazda Motor Corporation	Battery Reconditioner	I just used it, I don't know if it's effective yet, I'll comment later.	JD.com
Mazda Motor Corporation	Battery Reconditioner	Goods I received a long time ago, because of busy work, until now to evaluate, the quality is very good, as advertised, the service attitude is good, all aspects are quite good, on the logistics of a little slow, in short, worthy of the user's trust! I will also introduce friends to buy now.	JD.com
Mazda Motor Corporation	Battery Reconditioner	Good quality, give it 5 out of 5!	JD.com
Mazda Motor Corporation	Battery Reconditioner	I hope it works.	JD.com
Mazda Motor Corporation	Battery Reconditioner	fast delivery	JD.com
Mazda Motor Corporation	Battery Reconditioner	This repairer really works well, charging are no longer hot, charging will change the value, very pleasant shopping!	JD.com
Mazda Motor Corporation	Battery Reconditioner	Quite delicate, in the process of use	JD.com
Mazda Motor Corporation	Battery Reconditioner	This user feels the product is better	JD.com
Mazda Motor Corporation	Battery Reconditioner	Repair clamor received, very good logistics quickly thank you!	JD.com
Mazda Motor Corporation	Battery Reconditioner	Things received, good quality, good price, good service, logistics is also very fast.	JD.com
Mazda Motor Corporation	Battery Reconditioner	Through2days of cell phone charging compared to the actual charging voltage and current, indeed the phone charging is not hot, of course, charging time will be longer, the old cell phone charging repair does have a certain effect	JD.com
Mazda Motor Corporation	Battery Reconditioner	The goods have been received and feel good	JD.com
Mazda Motor Corporation	Battery Reconditioner	I received it last night and will try it today to see how it works......then I'll comment......	JD.com
Mazda Motor Corporation	Battery Reconditioner	It's good. It's good.	JD.com
Mazda Motor Corporation	Battery Reconditioner	It's working fine. I'm happy with it.	JD.com
Mazda Motor Corporation	Battery Reconditioner	It shows the voltage and current level! I just don't know why it won't activate the fast charger after I use it!!!!	JD.com
Mazda Motor Corporation	Battery Reconditioner	Baby received. Same as described in the picture. The price is real.	JD.com

Mazda Motor Corporation	Battery Reconditioner	Good stuff, will buy again next time	JD.com
Mazda Motor Corporation	Battery Reconditioner	Not bad.	JD.com
Mazda Motor Corporation	Battery Reconditioner	Goods received! Quite fast! Price is higher than high! Not expensive! Practical is good!	JD.com
Mazda Motor Corporation	Battery Reconditioner	You guys this stuff is not as advertised. I found an old cell phone and charged it overnight, but the next day the phone turned on and opened its eyes and slept as usual. Please make sure you understand the black technology before you put it on the market. Don't let the junk products harm the people!	JD.com
Mazda Motor Corporation	Battery Reconditioner	Well packaged, looks good, happy with 5 stars!	JD.com
Mazda Motor Corporation	Battery Reconditioner	Baby received, the quality is good, the function is intact, the logistics is very fast, habitual praise it!	JD.com
Mazda Motor Corporation	Battery Reconditioner	Logistics is fast. Will comment later.	JD.com
Mazda Motor Corporation	Battery Reconditioner	Affordable price good quality well packaged and easy to use	JD.com
Mazda Motor Corporation	Battery Reconditioner	Seems like the school fruit isn't very good.	JD.com
Mazda Motor Corporation	Battery Reconditioner	Goods received, because there is no instruction manual and business communication, by pointing out some understanding, the actual use of the follow-up, this habitual praise.	JD.com
Mazda Motor Corporation	Battery Reconditioner	I don't know how it works yet.	JD.com
Mazda Motor Corporation	Battery Reconditioner	give4starsfor nowand review it in a few days to see how it works out	JD.com
Mazda Motor Corporation	Battery Reconditioner	Compatibility: logistics is very fast, five-star praise	JD.com
Mazda Motor Corporation	Battery Reconditioner	Very trustworthy brand, quality is guaranteed, the price is still affordable, quite satisfied!	JD.com
Mazda Motor Corporation	Battery Reconditioner	Item is great affordable worth the money, good charger with a digital display.	JD.com
Mazda Motor Corporation	Battery Reconditioner	show charging, as for the effect, after a period of time to evaluate!	JD.com
Mazda Motor Corporation	Battery Reconditioner	Nice product, good quality, worth buying!	JD.com
Mazda Motor Corporation	Battery Reconditioner	Digital display, smart phone battery charging repairer, very good!	JD.com
Mazda Motor Corporation	Battery Reconditioner	Just received it, experimenting with it, don't know if it's good or bad. I think the bar should be very good. Very good!	JD.com
Mazda Motor Corporation	Battery Reconditioner	Goods received quite quickly, the appearance and goods as good or not I do not know, try to come back to evaluate.	JD.com
Mazda Motor Corporation	Battery Reconditioner	It's great, give it a good review!	JD.com
Mazda Motor Corporation	Battery Reconditioner	Pro goods have received the courier is very helpful use after the evaluation!	JD.com
Mazda Motor Corporation	Battery Reconditioner	the effect of the same, thanks to the merchant cooperation happy.	JD.com
Mazda Motor Corporation	Battery Reconditioner	This user feels the product is better	JD.com
Mazda Motor Corporation	Battery Reconditioner	Appearance saw the item is quite good, after using it, I will comment again.	JD.com
Mazda Motor Corporation	Battery Reconditioner	ah, the life of the battery is the life of the phone, with this restorer, cell phone batteries will live forever ah! Give a five-star praise.	JD.com
Mazda Motor Corporation	Battery Reconditioner	The logistics service is excellent, five stars.	JD.com
Mazda Motor Corporation	Battery Reconditioner	Item received and works great for charging old cell phones!	JD.com
Mazda Motor Corporation	Battery Reconditioner	The product received, the packaging is not humane, no scissors can not open, no instructions, I provide a photo please look at the business, I do not know what is the meaning of[face], please give guidance to the business as a hope!	JD.com
Mazda Motor Corporation	Battery Reconditioner	Fast logistics, good quality, good logistics attitude, good performance of the boy, next purchase.	JD.com
Mazda Motor Corporation	Battery Reconditioner	The item was shipped quickly, well packaged and of good quality.	JD.com
Mazda Motor Corporation	Battery Reconditioner	terrific	JD.com
Mazda Motor Corporation	Battery Reconditioner	Safe and reliable to use, genuine no doubt, clear display, very beautiful appearance, small size.	JD.com
Mazda Motor Corporation	Battery Reconditioner	Battery repair can be, and is worth collecting.	JD.com
Mazda Motor Corporation	Battery Reconditioner	Commodity received, the appearance of the feeling is still good, charging a few numbers in the exchange of jumps, I do not know if I can repair the phone battery, buy to play it.	JD.com

Mazda Motor Corporation	Battery Reconditioner	The item has been received, as described, unused, the courier is very fast and powerful[praise]	JD.com
Mazda Motor Corporation	Battery Reconditioner	I don't know how well it works, will follow up later.	JD.com
Mazda Motor Corporation	Battery Reconditioner	simple and practical	JD.com
Mazda Motor Corporation	Battery Reconditioner	Goods have been received quality is very good👌have want to buy rest assured that the order, I wish the boss business is prosperous, the financial resources[skimming]	JD.com
Mazda Motor Corporation	Battery Reconditioner	Cell phone charger received.	JD.com
Mazda Motor Corporation	Battery Reconditioner	Safe and reliable to use, more fine workmanship	JD.com
Mazda Motor Corporation	Battery Reconditioner	Just used stay for two days to see how it works.	JD.com
Mazda Motor Corporation	Battery Reconditioner	The use of safe and reliable, relatively fine workmanship, clear display, easy to use, after the initial use, the old phone is slightly more durable than before, continue to use it to see the effect	JD.com
Mazda Motor Corporation	Battery Reconditioner	This manufacturer is too good this man's hands are too technologically advanced. Thanks to the manufacturer	JD.com
Mazda Motor Corporation	Battery Reconditioner	The real deal!	JD.com
Mazda Motor Corporation	Battery Reconditioner	Got it, just received it and trying it out.	JD.com
Mazda Motor Corporation	Battery Reconditioner	It's great, good value for money. I love it.	JD.com
Mazda Motor Corporation	Battery Reconditioner	packaging is very good, the price is very affordable, thank you!	JD.com
Mazda Motor Corporation	Battery Reconditioner	This user feels the product is better	JD.com
Mazda Motor Corporation	Battery Reconditioner	Not bad!	JD.com
Mazda Motor Corporation	Battery Reconditioner	Charging function: battery restorer received the goods, no damage, satisfied. Looking forward to effective.	JD.com
Mazda Motor Corporation	Battery Reconditioner	Good and practical, easy to use and inexpensive.	JD.com
Mazda Motor Corporation	Battery Reconditioner	I'm very happy with it.	JD.com
Mazda Motor Corporation	Battery Reconditioner	Charging function: good, easy to use,<br>safety: good, good results after use, heat is significantly reduced. The price is right.	JD.com
Mazda Motor Corporation	Battery Reconditioner	And the picture describes the same, good value for money, whether it can repair the phone battery, later chase evaluation, give5stars praise! Recommended purchase.	JD.com
Mazda Motor Corporation	Battery Reconditioner	Is trying to see the effect, first five-star praise, after using in additional evaluation	JD.com
Mazda Motor Corporation	Battery Reconditioner	faster than it was. And I still don't understand what that digital display stands for, I'll follow up in a couple of days.	JD.com
Mazda Motor Corporation	Battery Reconditioner	ok	JD.com
Mazda Motor Corporation	Battery Reconditioner	Seems to work well to fix	JD.com
Mazda Motor Corporation	Battery Reconditioner	Baby received, consistent with the description, good service, fast delivery, logistics, praise ! ! ! ! !	JD.com
Mazda Motor Corporation	Battery Reconditioner	Logistics is fast and honest! Keeping promises! Good service attitude!	JD.com
Mazda Motor Corporation	Battery Reconditioner	Good use of the actual Hui, fast logistics,2days are received goods, good service.	JD.com
Mazda Motor Corporation	Battery Reconditioner	Cell phone charging repairer received, easy to use, but also can play a role in repairing the cell phone battery, and the description of the same	JD.com
Mazda Motor Corporation	Battery Reconditioner	Logistics very fast service good delivery fast give five good comments	JD.com
Mazda Motor Corporation	Battery Reconditioner	not bad	JD.com
Mazda Motor Corporation	Battery Reconditioner	Goods have been received, fast logistics and business description of the same, good service attitude	JD.com
Mazda Motor Corporation	Battery Reconditioner	The goods have been received. Thank you.	JD.com
Mazda Motor Corporation	Battery Reconditioner	Good quality, fast delivery of goods, kudos to Wingstar, good courier service!	JD.com
Mazda Motor Corporation	Battery Reconditioner	Good price How good is it and how will it work	JD.com
Mazda Motor Corporation	Battery Reconditioner	Where will it be fixed?	JD.com

Mazda Motor Corporation	Battery Reconditioner	It looks good. I don't know if it works.	JD.com
Mazda Motor Corporation	Battery Reconditioner	The stuff is great!	JD.com
Mazda Motor Corporation	Battery Reconditioner	charge, and after charging it for three hours with this item, it is now fully charged, and after a long time of use the battery shows a full charge. Must be a top quality item if it stands the test of time.	JD.com
Mazda Motor Corporation	Battery Reconditioner	Goods received and the video is the same, very satisfied with the evening to try Thanks!	JD.com
Mazda Motor Corporation	Battery Reconditioner	Charging function: good	JD.com
Mazda Motor Corporation	Battery Reconditioner	Cell phone battery is too costly/cell phone charger battery repairer battery drain too fast aging repair god Received, got it to see, and the same as in the video, good packaging, fast logistics, may the boss prosper in business and prosperity	JD.com
Mazda Motor Corporation	Battery Reconditioner	Received the goods, the logistics is very fast, try the effect and then say!	JD.com
Mazda Motor Corporation	Battery Reconditioner	[Loves]Love it	JD.com
Mazda Motor Corporation	Battery Reconditioner	began to rise, the eighth day is more normal, showing full, through the recent period of time to observe the use of view, the phone speed than the original a little bit better, or very useful, I have that old cell phone before they can not be charged into the electricity is broken, with this restorer can be, very good.	JD.com
Mazda Motor Corporation	Battery Reconditioner	The logistics is very fast, the quality of the goods is very good, repairing the cell phone battery of the gods, but also a good fast charger, does not hurt the phone, charging fast, the appearance is very beautiful, but also equipped with a display, it is very convenient to use. The same as described on the product introduction, and the logistics is very fast, thoughtful service, praise!	JD.com
Mazda Motor Corporation	Battery Reconditioner	The digital display is clear and accurate, the trial of the cell phone battery has a certain repair effect, easy to use, the cell phone range time is obviously long, very practical and strong sense of technology, it is worth recommending.	JD.com
Mazda Motor Corporation	Battery Reconditioner	Cell phone battery repairer quickly received, workmanship is relatively fine, just used a little, can be charged, if you can repair damaged appliances that would be worth it, the back will be followed up, first to give a good review!	JD.com
Mazda Motor Corporation	Battery Reconditioner	instructions, LCD display, according to customer service guidance to insert the charging head connected to the data line can be slow charging fast power consumption or charging not into the power of the phone to repair the first time to repair the cell phone shutdown, with the effect that after the use of the phone does, now my phone does not have to be before the power consumption!	JD.com
Mazda Motor Corporation	Battery Reconditioner	Have repaired several cell phones, Apple and Android can be used, my family's cell phone let me repair once, and now usually charging time can also be used!	JD.com
Mazda Motor Corporation	Battery Reconditioner	A use for many years of bluetooth speaker battery discharge deficit, charger direct charging has been unable to fill, so bought this repair charging equipment to try, after using, the battery power to restore 100%!	JD.com
Mazda Motor Corporation	Battery Reconditioner	Product received. The outer packaging is intact and the product is undamaged. Product products are being tried. Thank you very much for your store's warm and thoughtful service, give your store five-star praise! The store shipped very quickly, the logistics is also very strong.	JD.com
Mazda Motor Corporation	Battery Reconditioner	There is an effect, the phone power consumption is not so fast. At first not too obvious, after using a few days, only to feel the effect, not in vain to buy, really good, I also want to recommend relatives and friends.	JD.com
Mazda Motor Corporation	Battery Reconditioner	God, the phone isGalaxy-On7is2016to buy the product, the battery itself years in this, but there is indeed a clear ability to repair the stability of the battery, such as this old phone, the power once below40% will immediately shut down automatically, through the repair device to repair the two times, the amount of power is below18% has not been shut down, enough to show that the repair device is! There is a certain good effect	JD.com
Mazda Motor Corporation	Battery Reconditioner	of the charging process of the equipment, directly enabling us to observe the results of the charging current and voltage, reflecting the effectiveness of the equipment and good or bad, reducing the blind charging method and equipment damage. Very worth having!	JD.com

Mazda Motor Corporation	Battery Reconditioner	Previously, cell phone charging is particularly difficult to repair cell phone store to change the battery repair are very expensive, with this cell phone battery repairer, no longer need to spend money, no need to beg, self can do. Not only charging stable in the fast, but also can maintain the phone battery does not hurt the phone, the use of safe and reliable.	JD.com
Mazda Motor Corporation	Battery Reconditioner	Logistics and shipping are fast, also studying this specialty, the theory is really good for the battery, and very affordable compared to changing the battery	JD.com
Mazda Motor Corporation	Battery Reconditioner	description matches, packaging is beautiful and undamaged! The rest of the situation to be used a few days later to do a follow-up review!	JD.com
Mazda Motor Corporation	Battery Reconditioner	Received it, it works fine, I'll give it a good review first! Whether it is effective to use a period of time after evaluation, the battery can only be charged to 84%	JD.com
Mazda Motor Corporation	Battery Reconditioner	fairly well	JD.com
Mazda Motor Corporation	Battery Reconditioner	received, the repairer is very useful, the phone plugged into it after charging, the phone power saving effect is really much better!	JD.com
Mazda Motor Corporation	Battery Reconditioner	requirements for the operation of the use, the effect is still quite obvious, affordable, physical and picture description of a	JD.com
Mazda Motor Corporation	Battery Reconditioner	Battery is not durable power consumption rate is very fast, buy this repair artifact try really good results, now full of electricity can be used for a long time, charging speed is very fast, charging power range is obviously longer than before, the business also said that you can slowly repair the cell phone battery, really good ah, really super good!	JD.com
Mazda Motor Corporation	Battery Reconditioner	The effect is very good, samsung note8 repair effect is very good, this time do not have to change the battery, after repairing the phone with a longer time some, and does not heat up, and also automatically clean up the phone system cache garbage, the phone has a significantly smoother, the boss said that often used to repair will be better.	JD.com
Mazda Motor Corporation	Battery Reconditioner	resistant to electricity is to change the battery Or change the phone The first time to see this kind of repair battery Repair is very good I just Meadow not long Now the battery is healthy is a full score Every time you charge the battery to connect a bit It is not a lot of effort It is very hassle-free Not only can you charge the battery but also repair the TV A one-step two-fold Now the thing is doing more and more advanced magic weapon There is no major problem with the phone It is just the battery's good I'll give it a good review	JD.com
Mazda Motor Corporation	Battery Reconditioner	Hello, the cell phone repairer received to try the effect is very good and online said the same, indeed good, genuine cheap price, good quality, the effect is very good, this time the cell phone can battery have on, before fully charged an hour on the powerless, you see, a word fantastic, thank you.	JD.com
Mazda Motor Corporation	Battery Reconditioner	always go to work and ask my colleagues to borrow a charger My colleague said that my phone is so intolerant of electricity The battery repairer he bought is very good He charges it once a day in the evening The next day the battery is enough The charger I bought before is in the company Recently, I can't use it I asked him to push the link to me I bought it as well Sure enough, it works just as good as he said It's just that the power is obviously longer than before It is good for repairing batteries It protects the cell phone Very good I also give positive feedback Now most of my colleagues in the	JD.com
Mazda Motor Corporation	Battery Reconditioner	Cell phone battery repairer received very quickly, the workmanship is relatively fine, just used a little, can charge, can repair damaged batteries, it's worth it!	JD.com
Mazda Motor Corporation	Battery Reconditioner	Received after the use, my VIVO cell phone battery is not durable, with other chargers charging is very slow, go to customer service repair people also said that the model is too aging, the need to change the battery, I bought this slowly repair, and now it is very durable.	JD.com

Mazda Motor Corporation	Battery Reconditioner	you charge your cell phone but also repair the battery After I used it on the same day the next day I went to work and it felt significantly more resistant to electricity than the first day We use our cell phones about the same frequency Welook at our cell phones only so much time every day It is the function of repairing This is a few dozen dollars of things to save the money of replacing batteries Nowadays, there are a lot of places where you use your cell phone especially not resistant to electricity Replacement of the battery with the original one is also very expensive this productstill good. It's good	JD.com
Mazda Motor Corporation	Battery Reconditioner	This repairer after using the phone directly newborn, it is very good, the phone will not be hot, the response are becoming sensitive, with hands-on can be completed, the battery is quite durable, the capacity is particularly large, the response effect is also super agile, the phone does not appear to be lagging phenomenon, the charging effect is also super fast, and there is no mismatch.	JD.com
Mazda Motor Corporation	Battery Reconditioner	change the battery, the phone charge fast, full of electricity are able to use some more time, or satisfied with the product, this price love!	JD.com
Mazda Motor Corporation	Battery Reconditioner	particularly difficult to repair cell phone stores to replace the battery repair are very expensive, with this cell phone battery repairer, no longer need to spend money, and do not have to beg, since they can do, not only charging stable in the fast, but also to maintain cell phone batteries do not hurt the phone, the use of security is also reliable, there is a need for you to enter!	JD.com
Mazda Motor Corporation	Battery Reconditioner	Baby and the description of the match, business fast delivery and good attitude, logistics quickly, the courier swift and good attitude, give full marks!	JD.com
Mazda Motor Corporation	Battery Reconditioner	Battery Repairer received, the appearance of the work is very good, praise, just started to use do not know how, later used and then follow up!	JD.com
Mazda Motor Corporation	Battery Reconditioner	ineffective	JD.com
Mazda Motor Corporation	Battery Reconditioner	I was to see the cell phone repair store master with it to repair the battery (cell phone long unused leakage loss) think very practical! So[love]	JD.com
Mazda Motor Corporation	Battery Reconditioner	should work[hee]	JD.com
Mazda Motor Corporation	Battery Reconditioner	Logistics, good service attitude, cell phone repairer is good, the effect is good, satisfied!	JD.com
Mazda Motor Corporation	Battery Reconditioner	Good results after use	JD.com
Mazda Motor Corporation	Battery Reconditioner	can	JD.com
Mazda Motor Corporation	Battery Reconditioner	convenient to operate, looking forward to a good repair effect.	JD.com
Mazda Motor Corporation	Battery Reconditioner	Good quality and fast shipping received in a few days well	JD.com
Mazda Motor Corporation	Battery Reconditioner	Pro, cell phone battery repairer received, the goods are well made, the user thinks the goods are very good. Wish merchants prosperous business and prosperity. Give five-star praise!	JD.com
Mazda Motor Corporation	Battery Reconditioner	Nice baby, very satisfied, good price!	JD.com
Mazda Motor Corporation	Battery Reconditioner	Logistics is fast, the baby is consistent with the description, business service attitude is very good, the baby is worth the next time to help others to buy again.	JD.com
Mazda Motor Corporation	Battery Reconditioner	The seller shipped quickly! The baby is very good! Good value for money! Hope it works! All five good comments!	JD.com
Mazda Motor Corporation	Battery Reconditioner	This user feels the product is better	JD.com
Mazda Motor Corporation	Battery Reconditioner	Cell phone battery repairer, just have received, packaging intact, logistics is very fast, and the same as described in the picture, the quality is very good Oh, get back to charge, repair the Apple cell phone battery, the need for friends can order, give five-star praise!	JD.com
Mazda Motor Corporation	Battery Reconditioner	The workmanship is fine, I do not know if it works well, if it works then repurchase.	JD.com
Mazda Motor Corporation	Battery Reconditioner	This user feels the product is better	JD.com
Mazda Motor Corporation	Battery Reconditioner	Goods can be repaired quickly with the use of	JD.com
Mazda Motor Corporation	Battery Reconditioner	Excuse me.	JD.com
Mazda Motor Corporation	Battery Reconditioner	This user feels the product is better	JD.com

Mazda Motor Corporation	Battery Reconditioner	Can't see if there's a fix for it	JD.com
Mazda Motor Corporation	Battery Reconditioner	This user feels the product is better	JD.com
Mazda Motor Corporation	Battery Reconditioner	the quality is quite good, I am very satisfied. The product is beautiful, affordable and individually packaged. Worth recommending.	JD.com
Mazda Motor Corporation	Battery Reconditioner	This user feels the product is better	JD.com
Mazda Motor Corporation	Battery Reconditioner	Poor quality exaggerates the role of shoddy products	JD.com
Mazda Motor Corporation	Battery Reconditioner	I haven't used it yet.	JD.com
Mazda Motor Corporation	Battery Reconditioner	Use it and then price it.	JD.com
Mazda Motor Corporation	Battery Reconditioner	The packaging is very good and the logistics is fast.	JD.com
Mazda Motor Corporation	Battery Reconditioner	Packed completely, normal use, the effect will be known only after use, the	JD.com
Mazda Motor Corporation	Battery Reconditioner	The cell phone charging repairer works so well, it really doesn't jam the shield anymore	JD.com
Mazda Motor Corporation	Battery Reconditioner	That's a fast delivery!	JD.com
Mazda Motor Corporation	Battery Reconditioner	praise first five-star praise it night to use in order to evaluate the good or bad hope to use a very good in the purchase of a!	JD.com
Mazda Motor Corporation	Battery Reconditioner	Comments	JD.com
Mazda Motor Corporation	Battery Reconditioner	This user feels the product is better	JD.com
Mazda Motor Corporation	Battery Reconditioner	Poor quality exaggerates the role of shoddy products	JD.com
Mazda Motor Corporation	Battery Reconditioner	I haven't used it yet.	JD.com
Mazda Motor Corporation	Battery Reconditioner	Use it and then price it.	JD.com
Mazda Motor Corporation	Battery Reconditioner	The packaging is very good and the logistics is fast.	JD.com
Mazda Motor Corporation	Battery Reconditioner	Packed completely, normal use, the effect will be known only after use, the	JD.com
Mazda Motor Corporation	Battery Reconditioner	The cell phone charging repairer works so well, it really doesn't jam the shield anymore	JD.com
Mazda Motor Corporation	Battery Reconditioner	That's a fast delivery!	JD.com
Mazda Motor Corporation	Battery Reconditioner	praise first five-star praise it night to use in order to evaluate the good or bad hope to use a very good in the purchase of a!	JD.com
Mazda Motor Corporation	Battery Reconditioner	Comments	JD.com
Mazda Motor Corporation	Battery Reconditioner	This user feels the product is better	JD.com
Mazda Motor Corporation	Battery Reconditioner	Poor quality exaggerates the role of shoddy products	JD.com
Mazda Motor Corporation	Battery Reconditioner	I haven't used it yet.	JD.com
Mazda Motor Corporation	Battery Reconditioner	Use it and then price it.	JD.com
Mazda Motor Corporation	Battery Reconditioner	The packaging is very good and the logistics is fast.	JD.com
Mazda Motor Corporation	Battery Reconditioner	Packed completely, normal use, the effect will be known only after use, the	JD.com
Mazda Motor Corporation	Battery Reconditioner	The cell phone charging repairer works so well, it really doesn't jam the shield anymore	JD.com
Mazda Motor Corporation	Battery Reconditioner	That's a fast delivery!	JD.com
Mazda Motor Corporation	Battery Reconditioner	praise first five-star praise it night to use in order to evaluate the good or bad hope to use a very good in the purchase of a!	JD.com
Mazda Motor Corporation	Battery Reconditioner	Comments	JD.com
Mazda Motor Corporation	Battery Reconditioner	This user feels the product is better	JD.com
Mazda Motor Corporation	Battery Reconditioner	Poor quality exaggerates the role of shoddy products	JD.com
Mazda Motor Corporation	Battery Reconditioner	I haven't used it yet.	JD.com
Mazda Motor Corporation	Battery Reconditioner	Use it and then price it.	JD.com

Mazda Motor Corporation	Battery Reconditioner	The packaging is very good and the logistics is fast.	JD.com
Mazda Motor Corporation	Battery Reconditioner	Packed completely, normal use, the effect will be known only after use, the	JD.com
Mazda Motor Corporation	Battery Reconditioner	The cell phone charging repairer works so well, it really doesn't jam the shield anymore	JD.com
Mazda Motor Corporation	Battery Reconditioner	That's a fast delivery!	JD.com
Mazda Motor Corporation	Battery Reconditioner	praise first five-star praise it night to use in order to evaluate the good or bad hope to use a very good in the purchase of a!	JD.com
Mazda Motor Corporation	Battery Reconditioner	Comments	JD.com
Mazda Motor Corporation	Battery Reconditioner	This user feels the product is better	JD.com
Mazda Motor Corporation	Battery Reconditioner	Poor quality exaggerates the role of shoddy products	JD.com
Mazda Motor Corporation	Battery Reconditioner	I haven't used it yet.	JD.com
Mazda Motor Corporation	Battery Reconditioner	Use it and then price it.	JD.com
Mazda Motor Corporation	Battery Reconditioner	The packaging is very good and the logistics is fast.	JD.com
Mazda Motor Corporation	Battery Reconditioner	Packed completely, normal use, the effect will be known only after use, the	JD.com
Mazda Motor Corporation	Battery Reconditioner	The cell phone charging repairer works so well, it really doesn't jam the shield anymore	JD.com
Mazda Motor Corporation	Battery Reconditioner	That's a fast delivery!	JD.com
Mazda Motor Corporation	Battery Reconditioner	praise first five-star praise it night to use in order to evaluate the good or bad hope to use a very good in the purchase of a!	JD.com
Mazda Motor Corporation	Battery Reconditioner	Comments	JD.com
Mazda Motor Corporation	Battery Reconditioner	This user feels the product is better	JD.com
Mazda Motor Corporation	Battery Reconditioner	Poor quality exaggerates the role of shoddy products	JD.com
Mazda Motor Corporation	Battery Reconditioner	I haven't used it yet.	JD.com
Mazda Motor Corporation	Battery Reconditioner	Use it and then price it.	JD.com
Mazda Motor Corporation	Battery Reconditioner	The packaging is very good and the logistics is fast.	JD.com
Mazda Motor Corporation	Battery Reconditioner	Packed completely, normal use, the effect will be known only after use, the	JD.com
Mazda Motor Corporation	Battery Reconditioner	The cell phone charging repairer works so well, it really doesn't jam the shield anymore	JD.com
Mazda Motor Corporation	Battery Reconditioner	That's a fast delivery!	JD.com
Mazda Motor Corporation	Battery Reconditioner	praise first five-star praise it night to use in order to evaluate the good or bad hope to use a very good in the purchase of a!	JD.com
Mazda Motor Corporation	Battery Reconditioner	Comments	JD.com
Mazda Motor Corporation	Battery Reconditioner	This user feels the product is better	JD.com
Mazda Motor Corporation	Battery Reconditioner	Poor quality exaggerates the role of shoddy products	JD.com
Mazda Motor Corporation	Battery Reconditioner	I haven't used it yet.	JD.com
Mazda Motor Corporation	Battery Reconditioner	Use it and then price it.	JD.com
Mazda Motor Corporation	Battery Reconditioner	The packaging is very good and the logistics is fast.	JD.com
Mazda Motor Corporation	Battery Reconditioner	Packed completely, normal use, the effect will be known only after use, the	JD.com
Mazda Motor Corporation	Battery Reconditioner	The cell phone charging repairer works so well, it really doesn't jam the shield anymore	JD.com
Mazda Motor Corporation	Battery Reconditioner	That's a fast delivery!	JD.com
Mazda Motor Corporation	Battery Reconditioner	praise first five-star praise it night to use in order to evaluate the good or bad hope to use a very good in the purchase of a!	JD.com
Mazda Motor Corporation	Battery Reconditioner	Comments	JD.com
Mazda Motor Corporation	Battery Reconditioner	This user feels the product is better	JD.com

Mazda Motor Corporation	Battery Reconditioner	Poor quality exaggerates the role of shoddy products	JD.com
Mazda Motor Corporation	Battery Reconditioner	I haven't used it yet.	JD.com
Mazda Motor Corporation	Battery Reconditioner	Use it and then price it.	JD.com
Mazda Motor Corporation	Battery Reconditioner	The packaging is very good and the logistics is fast.	JD.com
Mazda Motor Corporation	Battery Reconditioner	Packed completely, normal use, the effect will be known only after use, the	JD.com
Mazda Motor Corporation	Battery Reconditioner	The cell phone charging repairer works so well, it really doesn't jam the shield anymore	JD.com
Mazda Motor Corporation	Battery Reconditioner	That's a fast delivery!	JD.com
Mazda Motor Corporation	Battery Reconditioner	praise first five-star praise it night to use in order to evaluate the good or bad hope to use a very good in the purchase of a!	JD.com
Mazda Motor Corporation	Battery Reconditioner	Comments	JD.com
Mazda Motor Corporation	Battery Reconditioner	This user feels the product is better	JD.com
Mazda Motor Corporation	Battery Reconditioner	Poor quality exaggerates the role of shoddy products	JD.com
Mazda Motor Corporation	Battery Reconditioner	I haven't used it yet.	JD.com
Mazda Motor Corporation	Battery Reconditioner	Use it and then price it.	JD.com
Mazda Motor Corporation	Battery Reconditioner	The packaging is very good and the logistics is fast.	JD.com
Mazda Motor Corporation	Battery Reconditioner	Packed completely, normal use, the effect will be known only after use, the	JD.com
Mazda Motor Corporation	Battery Reconditioner	The cell phone charging repairer works so well, it really doesn't jam the shield anymore	JD.com
Mazda Motor Corporation	Battery Reconditioner	That's a fast delivery!	JD.com
Mazda Motor Corporation	Battery Reconditioner	praise first five-star praise it night to use in order to evaluate the good or bad hope to use a very good in the purchase of a!	JD.com
Mazda Motor Corporation	Battery Reconditioner	Comments	JD.com
Mazda Motor Corporation	Battery Reconditioner	This user feels the product is better	JD.com
Mazda Motor Corporation	Battery Reconditioner	Poor quality exaggerates the role of shoddy products	JD.com
Mazda Motor Corporation	Battery Reconditioner	I haven't used it yet.	JD.com
Mazda Motor Corporation	Battery Reconditioner	Use it and then price it.	JD.com
Mazda Motor Corporation	Battery Reconditioner	The packaging is very good and the logistics is fast.	JD.com
Mazda Motor Corporation	Battery Reconditioner	Packed completely, normal use, the effect will be known only after use, the	JD.com
Mazda Motor Corporation	Battery Reconditioner	The cell phone charging repairer works so well, it really doesn't jam the shield anymore	JD.com
Mazda Motor Corporation	Battery Reconditioner	That's a fast delivery!	JD.com
Mazda Motor Corporation	Battery Reconditioner	praise first five-star praise it night to use in order to evaluate the good or bad hope to use a very good in the purchase of a!	JD.com
Mazda Motor Corporation	Battery Reconditioner	Comments	JD.com
Mazda Motor Corporation	Battery Reconditioner	This user feels the product is better	JD.com
Mazda Motor Corporation	Battery Reconditioner	Poor quality exaggerates the role of shoddy products	JD.com
Mazda Motor Corporation	Battery Reconditioner	I haven't used it yet.	JD.com
Mazda Motor Corporation	Battery Reconditioner	Use it and then price it.	JD.com
Mazda Motor Corporation	Battery Reconditioner	The packaging is very good and the logistics is fast.	JD.com
Mazda Motor Corporation	Battery Reconditioner	Packed completely, normal use, the effect will be known only after use, the	JD.com
Mazda Motor Corporation	Battery Reconditioner	The cell phone charging repairer works so well, it really doesn't jam the shield anymore	JD.com
Mazda Motor Corporation	Battery Reconditioner	That's a fast delivery!	JD.com

Mazda Motor Corporation	Battery Reconditioner	praise first five-star praise it night to use in order to evaluate the good or bad hope to use a very good in the purchase of a!	JD.com
Mazda Motor Corporation	Battery Reconditioner	Comments	JD.com
Mazda Motor Corporation	Battery Reconditioner	This user feels the product is better	JD.com
Mazda Motor Corporation	Battery Reconditioner	Poor quality exaggerates the role of shoddy products	JD.com
Mazda Motor Corporation	Battery Reconditioner	I haven't used it yet.	JD.com
Mazda Motor Corporation	Battery Reconditioner	Use it and then price it.	JD.com
Mazda Motor Corporation	Battery Reconditioner	The packaging is very good and the logistics is fast.	JD.com
Mazda Motor Corporation	Battery Reconditioner	Packed completely, normal use, the effect will be known only after use, the	JD.com
Mazda Motor Corporation	Battery Reconditioner	The cell phone charging repairer works so well, it really doesn't jam the shield anymore	JD.com
Mazda Motor Corporation	Battery Reconditioner	That's a fast delivery!	JD.com
Mazda Motor Corporation	Battery Reconditioner	praise first five-star praise it night to use in order to evaluate the good or bad hope to use a very good in the purchase of a!	JD.com
Mazda Motor Corporation	Battery Reconditioner	Comments	JD.com
Mazda Motor Corporation	Battery Reconditioner	This user feels the product is better	JD.com
Mazda Motor Corporation	Battery Reconditioner	Poor quality exaggerates the role of shoddy products	JD.com
Mazda Motor Corporation	Battery Reconditioner	I haven't used it yet.	JD.com
Mazda Motor Corporation	Battery Reconditioner	Use it and then price it.	JD.com
Mazda Motor Corporation	Battery Reconditioner	The packaging is very good and the logistics is fast.	JD.com
Mazda Motor Corporation	Battery Reconditioner	Packed completely, normal use, the effect will be known only after use, the	JD.com
Mazda Motor Corporation	Battery Reconditioner	The cell phone charging repairer works so well, it really doesn't jam the shield anymore	JD.com
Mazda Motor Corporation	Battery Reconditioner	That's a fast delivery!	JD.com
Mazda Motor Corporation	Battery Reconditioner	praise first five-star praise it night to use in order to evaluate the good or bad hope to use a very good in the purchase of a!	JD.com
Mazda Motor Corporation	Battery Reconditioner	Comments	JD.com
Mazda Motor Corporation	Battery Reconditioner	This user feels the product is better	JD.com
Mazda Motor Corporation	Battery Reconditioner	Poor quality exaggerates the role of shoddy products	JD.com
Mazda Motor Corporation	Battery Reconditioner	I haven't used it yet.	JD.com
Mazda Motor Corporation	Battery Reconditioner	Use it and then price it.	JD.com
Mazda Motor Corporation	Battery Reconditioner	The packaging is very good and the logistics is fast.	JD.com
Mazda Motor Corporation	Battery Reconditioner	Packed completely, normal use, the effect will be known only after use, the	JD.com
Mazda Motor Corporation	Battery Reconditioner	The cell phone charging repairer works so well, it really doesn't jam the shield anymore	JD.com
Mazda Motor Corporation	Battery Reconditioner	That's a fast delivery!	JD.com
Mazda Motor Corporation	Battery Reconditioner	praise first five-star praise it night to use in order to evaluate the good or bad hope to use a very good in the purchase of a!	JD.com
Mazda Motor Corporation	Battery Reconditioner	Comments	JD.com
Mazda Motor Corporation	Battery Reconditioner	This user feels the product is better	JD.com
Mazda Motor Corporation	Battery Reconditioner	Poor quality exaggerates the role of shoddy products	JD.com
Mazda Motor Corporation	Battery Reconditioner	I haven't used it yet.	JD.com
Mazda Motor Corporation	Battery Reconditioner	Use it and then price it.	JD.com
Mazda Motor Corporation	Battery Reconditioner	The packaging is very good and the logistics is fast.	JD.com

Mazda Motor Corporation	Battery Reconditioner	Packed completely, normal use, the effect will be known only after use, the	JD.com
Mazda Motor Corporation	Battery Reconditioner	The cell phone charging repairer works so well, it really doesn't jam the shield anymore	JD.com
Mazda Motor Corporation	Battery Reconditioner	That's a fast delivery!	JD.com
Mazda Motor Corporation	Battery Reconditioner	praise first five-star praise it night to use in order to evaluate the good or bad hope to use a very good in the purchase of a!	JD.com
Mazda Motor Corporation	Battery Reconditioner	Comments	JD.com
Mazda Motor Corporation	Battery Reconditioner	This user feels the product is better	JD.com
Mazda Motor Corporation	Battery Reconditioner	Poor quality exaggerates the role of shoddy products	JD.com
Mazda Motor Corporation	Battery Reconditioner	I haven't used it yet.	JD.com
Mazda Motor Corporation	Battery Reconditioner	Use it and then price it.	JD.com
Mazda Motor Corporation	Battery Reconditioner	The packaging is very good and the logistics is fast.	JD.com
Mazda Motor Corporation	Battery Reconditioner	Packed completely, normal use, the effect will be known only after use, the	JD.com
Mazda Motor Corporation	Battery Reconditioner	The cell phone charging repairer works so well, it really doesn't jam the shield anymore	JD.com
Mazda Motor Corporation	Battery Reconditioner	That's a fast delivery!	JD.com
Mazda Motor Corporation	Battery Reconditioner	praise first five-star praise it night to use in order to evaluate the good or bad hope to use a very good in the purchase of a!	JD.com
Mazda Motor Corporation	Battery Reconditioner	Comments	JD.com
Mazda Motor Corporation	Battery Reconditioner	This user feels the product is better	JD.com
Mazda Motor Corporation	Battery Reconditioner	Poor quality exaggerates the role of shoddy products	JD.com
Mazda Motor Corporation	Battery Reconditioner	I haven't used it yet.	JD.com
Mazda Motor Corporation	Battery Reconditioner	Use it and then price it.	JD.com
Mazda Motor Corporation	Battery Reconditioner	The packaging is very good and the logistics is fast.	JD.com
Mazda Motor Corporation	Battery Reconditioner	Packed completely, normal use, the effect will be known only after use, the	JD.com
Mazda Motor Corporation	Battery Reconditioner	The cell phone charging repairer works so well, it really doesn't jam the shield anymore	JD.com
Mazda Motor Corporation	Battery Reconditioner	That's a fast delivery!	JD.com
Mazda Motor Corporation	Battery Reconditioner	praise first five-star praise it night to use in order to evaluate the good or bad hope to use a very good in the purchase of a!	JD.com
Mazda Motor Corporation	Battery Reconditioner	Comments	JD.com
Mazda Motor Corporation	Battery Reconditioner	This user feels the product is better	JD.com
Mazda Motor Corporation	Battery Reconditioner	Poor quality exaggerates the role of shoddy products	JD.com
Mazda Motor Corporation	Battery Reconditioner	I haven't used it yet.	JD.com
Mazda Motor Corporation	Battery Reconditioner	Use it and then price it.	JD.com
Mazda Motor Corporation	Battery Reconditioner	The packaging is very good and the logistics is fast.	JD.com
Mazda Motor Corporation	Battery Reconditioner	Packed completely, normal use, the effect will be known only after use, the	JD.com
Mazda Motor Corporation	Battery Reconditioner	The cell phone charging repairer works so well, it really doesn't jam the shield anymore	JD.com
Mazda Motor Corporation	Battery Reconditioner	That's a fast delivery!	JD.com
Mazda Motor Corporation	Battery Reconditioner	praise first five-star praise it night to use in order to evaluate the good or bad hope to use a very good in the purchase of a!	JD.com
Mazda Motor Corporation	Battery Reconditioner	Comments	JD.com
Mazda Motor Corporation	Battery Reconditioner	This user feels the product is better	JD.com
Mazda Motor Corporation	Battery Reconditioner	Poor quality exaggerates the role of shoddy products	JD.com

Mazda Motor Corporation	Battery Reconditioner	I haven't used it yet.	JD.com
Mazda Motor Corporation	Battery Reconditioner	Use it and then price it.	JD.com
Mazda Motor Corporation	Battery Reconditioner	The packaging is very good and the logistics is fast.	JD.com
Mazda Motor Corporation	Battery Reconditioner	Packed completely, normal use, the effect will be known only after use, the	JD.com
Mazda Motor Corporation	Battery Reconditioner	The cell phone charging repairer works so well, it really doesn't jam the shield anymore	JD.com
Mazda Motor Corporation	Battery Reconditioner	That's a fast delivery!	JD.com
Mazda Motor Corporation	Battery Reconditioner	praise first five-star praise it night to use in order to evaluate the good or bad hope to use a very good in the purchase of a!	JD.com
Mazda Motor Corporation	Battery Reconditioner	Comments	JD.com
Mazda Motor Corporation	Battery Reconditioner	This user feels the product is better	JD.com
Mazda Motor Corporation	Battery Reconditioner	Poor quality exaggerates the role of shoddy products	JD.com
Mazda Motor Corporation	Battery Reconditioner	I haven't used it yet.	JD.com
Mazda Motor Corporation	Battery Reconditioner	Use it and then price it.	JD.com
Mazda Motor Corporation	Battery Reconditioner	The packaging is very good and the logistics is fast.	JD.com
Mazda Motor Corporation	Battery Reconditioner	Packed completely, normal use, the effect will be known only after use, the	JD.com
Mazda Motor Corporation	Battery Reconditioner	The cell phone charging repairer works so well, it really doesn't jam the shield anymore	JD.com
Mazda Motor Corporation	Battery Reconditioner	That's a fast delivery!	JD.com
Mazda Motor Corporation	Battery Reconditioner	praise first five-star praise it night to use in order to evaluate the good or bad hope to use a very good in the purchase of a!	JD.com
Mazda Motor Corporation	Battery Reconditioner	Comments	JD.com
Mazda Motor Corporation	Battery Reconditioner	This user feels the product is better	JD.com
Mazda Motor Corporation	Battery Reconditioner	Poor quality exaggerates the role of shoddy products	JD.com
Mazda Motor Corporation	Battery Reconditioner	I haven't used it yet.	JD.com
Mazda Motor Corporation	Battery Reconditioner	Use it and then price it.	JD.com
Mazda Motor Corporation	Battery Reconditioner	The packaging is very good and the logistics is fast.	JD.com
Mazda Motor Corporation	Battery Reconditioner	Packed completely, normal use, the effect will be known only after use, the	JD.com
Mazda Motor Corporation	Battery Reconditioner	The cell phone charging repairer works so well, it really doesn't jam the shield anymore	JD.com
Mazda Motor Corporation	Battery Reconditioner	That's a fast delivery!	JD.com
Mazda Motor Corporation	Battery Reconditioner	praise first five-star praise it night to use in order to evaluate the good or bad hope to use a very good in the purchase of a!	JD.com
Mazda Motor Corporation	Battery Reconditioner	Comments	JD.com
Mazda Motor Corporation	Battery Reconditioner	This user feels the product is better	JD.com
Mazda Motor Corporation	Battery Reconditioner	Poor quality exaggerates the role of shoddy products	JD.com
Mazda Motor Corporation	Battery Reconditioner	I haven't used it yet.	JD.com
Mazda Motor Corporation	Battery Reconditioner	Use it and then price it.	JD.com
Mazda Motor Corporation	Battery Reconditioner	The packaging is very good and the logistics is fast.	JD.com
Mazda Motor Corporation	Battery Reconditioner	Packed completely, normal use, the effect will be known only after use, the	JD.com
Mazda Motor Corporation	Battery Reconditioner	The cell phone charging repairer works so well, it really doesn't jam the shield anymore	JD.com
Mazda Motor Corporation	Battery Reconditioner	That's a fast delivery!	JD.com
Mazda Motor Corporation	Battery Reconditioner	praise first five-star praise it night to use in order to evaluate the good or bad hope to use a very good in the purchase of a!	JD.com

Mazda Motor Corporation	Battery Reconditioner	Comments	JD.com
Mazda Motor Corporation	Battery Reconditioner	This user feels the product is better	JD.com
Mazda Motor Corporation	Battery Reconditioner	Poor quality exaggerates the role of shoddy products	JD.com
Mazda Motor Corporation	Battery Reconditioner	I haven't used it yet.	JD.com
Mazda Motor Corporation	Battery Reconditioner	Use it and then price it.	JD.com
Mazda Motor Corporation	Battery Reconditioner	The packaging is very good and the logistics is fast.	JD.com
Mazda Motor Corporation	Battery Reconditioner	Packed completely, normal use, the effect will be known only after use, the	JD.com
Mazda Motor Corporation	Battery Reconditioner	The cell phone charging repairer works so well, it really doesn't jam the shield anymore	JD.com
Mazda Motor Corporation	Battery Reconditioner	That's a fast delivery!	JD.com
Mazda Motor Corporation	Battery Reconditioner	praise first five-star praise it night to use in order to evaluate the good or bad hope to use a very good in the purchase of a!	JD.com
Mazda Motor Corporation	Battery Reconditioner	Comments	JD.com
Mazda Motor Corporation	Battery Reconditioner	This user feels the product is better	JD.com
Mazda Motor Corporation	Battery Reconditioner	Poor quality exaggerates the role of shoddy products	JD.com
Mazda Motor Corporation	Battery Reconditioner	I haven't used it yet.	JD.com
Mazda Motor Corporation	Battery Reconditioner	Use it and then price it.	JD.com
Mazda Motor Corporation	Battery Reconditioner	The packaging is very good and the logistics is fast.	JD.com
Mazda Motor Corporation	Battery Reconditioner	Packed completely, normal use, the effect will be known only after use, the	JD.com
Mazda Motor Corporation	Battery Reconditioner	The cell phone charging repairer works so well, it really doesn't jam the shield anymore	JD.com
Mazda Motor Corporation	Battery Reconditioner	That's a fast delivery!	JD.com
Mazda Motor Corporation	Battery Reconditioner	praise first five-star praise it night to use in order to evaluate the good or bad hope to use a very good in the purchase of a!	JD.com
Mazda Motor Corporation	Battery Reconditioner	Comments	JD.com
Mazda Motor Corporation	Battery Reconditioner	This user feels the product is better	JD.com
Mazda Motor Corporation	Battery Reconditioner	Poor quality exaggerates the role of shoddy products	JD.com
Mazda Motor Corporation	Battery Reconditioner	I haven't used it yet.	JD.com
Mazda Motor Corporation	Battery Reconditioner	Use it and then price it.	JD.com
Mazda Motor Corporation	Battery Reconditioner	The packaging is very good and the logistics is fast.	JD.com
Mazda Motor Corporation	Battery Reconditioner	Packed completely, normal use, the effect will be known only after use, the	JD.com
Mazda Motor Corporation	Battery Reconditioner	The cell phone charging repairer works so well, it really doesn't jam the shield anymore	JD.com
Mazda Motor Corporation	Battery Reconditioner	That's a fast delivery!	JD.com
Mazda Motor Corporation	Battery Reconditioner	praise first five-star praise it night to use in order to evaluate the good or bad hope to use a very good in the purchase of a!	JD.com
Mazda Motor Corporation	Battery Reconditioner	Comments	JD.com
Mazda Motor Corporation	Battery Reconditioner	This user feels the product is better	JD.com
Mazda Motor Corporation	Battery Reconditioner	Poor quality exaggerates the role of shoddy products	JD.com
Mazda Motor Corporation	Battery Reconditioner	I haven't used it yet.	JD.com
Mazda Motor Corporation	Battery Reconditioner	Use it and then price it.	JD.com
Mazda Motor Corporation	Battery Reconditioner	The packaging is very good and the logistics is fast.	JD.com
Mazda Motor Corporation	Battery Reconditioner	Packed completely, normal use, the effect will be known only after use, the	JD.com
Mazda Motor Corporation	Battery Reconditioner	The cell phone charging repairer works so well, it really doesn't jam the shield anymore	JD.com

Mazda Motor Corporation	Battery Reconditioner	That's a fast delivery!	JD.com
Mazda Motor Corporation	Battery Reconditioner	praise first five-star praise it night to use in order to evaluate the good or bad hope to use a very good in the purchase of a!	JD.com
Mazda Motor Corporation	Battery Reconditioner	Comments	JD.com
Mazda Motor Corporation	Battery Reconditioner	This user feels the product is better	JD.com
Mazda Motor Corporation	Battery Reconditioner	Poor quality exaggerates the role of shoddy products	JD.com
Mazda Motor Corporation	Battery Reconditioner	I haven't used it yet.	JD.com
Mazda Motor Corporation	Battery Reconditioner	Use it and then price it.	JD.com
Mazda Motor Corporation	Battery Reconditioner	The packaging is very good and the logistics is fast.	JD.com
Mazda Motor Corporation	Battery Reconditioner	Packed completely, normal use, the effect will be known only after use, the	JD.com
Mazda Motor Corporation	Battery Reconditioner	The cell phone charging repairer works so well, it really doesn't jam the shield anymore	JD.com
Mazda Motor Corporation	Battery Reconditioner	That's a fast delivery!	JD.com
Mazda Motor Corporation	Battery Reconditioner	praise first five-star praise it night to use in order to evaluate the good or bad hope to use a very good in the purchase of a!	JD.com
Mazda Motor Corporation	Battery Reconditioner	Comments	JD.com
Mazda Motor Corporation	Battery Reconditioner	This user feels the product is better	JD.com
Mazda Motor Corporation	Battery Reconditioner	Poor quality exaggerates the role of shoddy products	JD.com
Mazda Motor Corporation	Battery Reconditioner	I haven't used it yet.	JD.com
Mazda Motor Corporation	Battery Reconditioner	Use it and then price it.	JD.com
Mazda Motor Corporation	Battery Reconditioner	The packaging is very good and the logistics is fast.	JD.com
Mazda Motor Corporation	Battery Reconditioner	Packed completely, normal use, the effect will be known only after use, the	JD.com
Mazda Motor Corporation	Battery Reconditioner	The cell phone charging repairer works so well, it really doesn't jam the shield anymore	JD.com
Mazda Motor Corporation	Battery Reconditioner	That's a fast delivery!	JD.com
Mazda Motor Corporation	Battery Reconditioner	praise first five-star praise it night to use in order to evaluate the good or bad hope to use a very good in the purchase of a!	JD.com
Mazda Motor Corporation	Battery Reconditioner	Comments	JD.com
Mazda Motor Corporation	Battery Reconditioner	This user feels the product is better	JD.com
Mazda Motor Corporation	Battery Reconditioner	Poor quality exaggerates the role of shoddy products	JD.com
Mazda Motor Corporation	Battery Reconditioner	I haven't used it yet.	JD.com
Mazda Motor Corporation	Battery Reconditioner	Use it and then price it.	JD.com
Mazda Motor Corporation	Battery Reconditioner	The packaging is very good and the logistics is fast.	JD.com
Mazda Motor Corporation	Battery Reconditioner	Packed completely, normal use, the effect will be known only after use, the	JD.com
Mazda Motor Corporation	Battery Reconditioner	The cell phone charging repairer works so well, it really doesn't jam the shield anymore	JD.com
Mazda Motor Corporation	Battery Reconditioner	That's a fast delivery!	JD.com
Mazda Motor Corporation	Battery Reconditioner	praise first five-star praise it night to use in order to evaluate the good or bad hope to use a very good in the purchase of a!	JD.com
Mazda Motor Corporation	Battery Reconditioner	Comments	JD.com
Mazda Motor Corporation	Battery Reconditioner	This user feels the product is better	JD.com
Mazda Motor Corporation	Battery Reconditioner	Poor quality exaggerates the role of shoddy products	JD.com
Mazda Motor Corporation	Battery Reconditioner	I haven't used it yet.	JD.com
Mazda Motor Corporation	Battery Reconditioner	Use it and then price it.	JD.com

Mazda Motor Corporation	Battery Reconditioner	The packaging is very good and the logistics is fast.	JD.com
Mazda Motor Corporation	Battery Reconditioner	Packed completely, normal use, the effect will be known only after use, the	JD.com
Mazda Motor Corporation	Battery Reconditioner	The cell phone charging repairer works so well, it really doesn't jam the shield anymore	JD.com
Mazda Motor Corporation	Battery Reconditioner	That's a fast delivery!	JD.com
Mazda Motor Corporation	Battery Reconditioner	praise first five-star praise it night to use in order to evaluate the good or bad hope to use a very good in the purchase of a!	JD.com
Mazda Motor Corporation	Battery Reconditioner	Comments	JD.com
Mazda Motor Corporation	Battery Reconditioner	This user feels the product is better	JD.com
Mazda Motor Corporation	Battery Reconditioner	Poor quality exaggerates the role of shoddy products	JD.com
Mazda Motor Corporation	Battery Reconditioner	I haven't used it yet.	JD.com
Mazda Motor Corporation	Battery Reconditioner	Use it and then price it.	JD.com
Mazda Motor Corporation	Battery Reconditioner	The packaging is very good and the logistics is fast.	JD.com
Mazda Motor Corporation	Battery Reconditioner	Packed completely, normal use, the effect will be known only after use, the	JD.com
Mazda Motor Corporation	Battery Reconditioner	The cell phone charging repairer works so well, it really doesn't jam the shield anymore	JD.com
Mazda Motor Corporation	Battery Reconditioner	That's a fast delivery!	JD.com
Mazda Motor Corporation	Battery Reconditioner	praise first five-star praise it night to use in order to evaluate the good or bad hope to use a very good in the purchase of a!	JD.com
Mazda Motor Corporation	Battery Reconditioner	Comments	JD.com
Mazda Motor Corporation	Battery Reconditioner	This user feels the product is better	JD.com
Mazda Motor Corporation	Battery Reconditioner	Poor quality exaggerates the role of shoddy products	JD.com
Mazda Motor Corporation	Battery Reconditioner	I haven't used it yet.	JD.com
Mazda Motor Corporation	Battery Reconditioner	Use it and then price it.	JD.com
Mazda Motor Corporation	Battery Reconditioner	The packaging is very good and the logistics is fast.	JD.com
Mazda Motor Corporation	Battery Reconditioner	Packed completely, normal use, the effect will be known only after use, the	JD.com
Mazda Motor Corporation	Battery Reconditioner	The cell phone charging repairer works so well, it really doesn't jam the shield anymore	JD.com
Mazda Motor Corporation	Battery Reconditioner	That's a fast delivery!	JD.com
Mazda Motor Corporation	Battery Reconditioner	praise first five-star praise it night to use in order to evaluate the good or bad hope to use a very good in the purchase of a!	JD.com
Mazda Motor Corporation	Battery Reconditioner	Comments	JD.com
Mazda Motor Corporation	Battery Reconditioner	This user feels the product is better	JD.com
Mazda Motor Corporation	Battery Reconditioner	Poor quality exaggerates the role of shoddy products	JD.com
Mazda Motor Corporation	Battery Reconditioner	I haven't used it yet.	JD.com
Mazda Motor Corporation	Battery Reconditioner	Use it and then price it.	JD.com
Mazda Motor Corporation	Battery Reconditioner	The packaging is very good and the logistics is fast.	JD.com
Mazda Motor Corporation	Battery Reconditioner	Packed completely, normal use, the effect will be known only after use, the	JD.com
Mazda Motor Corporation	Battery Reconditioner	The cell phone charging repairer works so well, it really doesn't jam the shield anymore	JD.com
Mazda Motor Corporation	Battery Reconditioner	That's a fast delivery!	JD.com
Mazda Motor Corporation	Battery Reconditioner	praise first five-star praise it night to use in order to evaluate the good or bad hope to use a very good in the purchase of a!	JD.com
Mazda Motor Corporation	Battery Reconditioner	Comments	JD.com
Mazda Motor Corporation	Battery Reconditioner	This user feels the product is better	JD.com

Mazda Motor Corporation	Battery Reconditioner	Poor quality exaggerates the role of shoddy products	JD.com
Mazda Motor Corporation	Battery Reconditioner	I haven't used it yet.	JD.com
Mazda Motor Corporation	Battery Reconditioner	Use it and then price it.	JD.com
Mazda Motor Corporation	Battery Reconditioner	The packaging is very good and the logistics is fast.	JD.com
Mazda Motor Corporation	Battery Reconditioner	Packed completely, normal use, the effect will be known only after use, the	JD.com
Mazda Motor Corporation	Battery Reconditioner	The cell phone charging repairer works so well, it really doesn't jam the shield anymore	JD.com
Mazda Motor Corporation	Battery Reconditioner	That's a fast delivery!	JD.com
Mazda Motor Corporation	Battery Reconditioner	praise first five-star praise it night to use in order to evaluate the good or bad hope to use a very good in the purchase of a!	JD.com
Mazda Motor Corporation	Battery Reconditioner	Comments	JD.com
Mazda Motor Corporation	Battery Reconditioner	This user feels the product is better	JD.com
Mazda Motor Corporation	Battery Reconditioner	Poor quality exaggerates the role of shoddy products	JD.com
Mazda Motor Corporation	Battery Reconditioner	I haven't used it yet.	JD.com
Mazda Motor Corporation	Battery Reconditioner	Use it and then price it.	JD.com
Mazda Motor Corporation	Battery Reconditioner	The packaging is very good and the logistics is fast.	JD.com
Mazda Motor Corporation	Battery Reconditioner	Packed completely, normal use, the effect will be known only after use, the	JD.com
Mazda Motor Corporation	Battery Reconditioner	The cell phone charging repairer works so well, it really doesn't jam the shield anymore	JD.com
Mazda Motor Corporation	Battery Reconditioner	That's a fast delivery!	JD.com
Mazda Motor Corporation	Battery Reconditioner	praise first five-star praise it night to use in order to evaluate the good or bad hope to use a very good in the purchase of a!	JD.com
Mazda Motor Corporation	Battery Reconditioner	Comments	JD.com
Mazda Motor Corporation	Battery Reconditioner	This user feels the product is better	JD.com
Mazda Motor Corporation	Battery Reconditioner	Poor quality exaggerates the role of shoddy products	JD.com
Mazda Motor Corporation	Battery Reconditioner	I haven't used it yet.	JD.com
Mazda Motor Corporation	Battery Reconditioner	Use it and then price it.	JD.com
Mazda Motor Corporation	Battery Reconditioner	The packaging is very good and the logistics is fast.	JD.com
Mazda Motor Corporation	Battery Reconditioner	Packed completely, normal use, the effect will be known only after use, the	JD.com
Mazda Motor Corporation	Battery Reconditioner	The cell phone charging repairer works so well, it really doesn't jam the shield anymore	JD.com
Mazda Motor Corporation	Battery Reconditioner	That's a fast delivery!	JD.com
Mazda Motor Corporation	Battery Reconditioner	praise first five-star praise it night to use in order to evaluate the good or bad hope to use a very good in the purchase of a!	JD.com
Mazda Motor Corporation	Battery Reconditioner	Comments	JD.com
Mazda Motor Corporation	Battery Reconditioner	This user feels the product is better	JD.com
Mazda Motor Corporation	Battery Reconditioner	Poor quality exaggerates the role of shoddy products	JD.com
Mazda Motor Corporation	Battery Reconditioner	I haven't used it yet.	JD.com
Mazda Motor Corporation	Battery Reconditioner	Use it and then price it.	JD.com
Mazda Motor Corporation	Battery Reconditioner	The packaging is very good and the logistics is fast.	JD.com
Mazda Motor Corporation	Battery Reconditioner	Packed completely, normal use, the effect will be known only after use, the	JD.com
Mazda Motor Corporation	Battery Reconditioner	The cell phone charging repairer works so well, it really doesn't jam the shield anymore	JD.com
Mazda Motor Corporation	Battery Reconditioner	That's a fast delivery!	JD.com

Mazda Motor Corporation	Battery Reconditioner	praise first five-star praise it night to use in order to evaluate the good or bad hope to use a very good in the purchase of a!	JD.com
Mazda Motor Corporation	Battery Reconditioner	Comments	JD.com
Mazda Motor Corporation	Battery Reconditioner	This user feels the product is better	JD.com
Mazda Motor Corporation	Battery Reconditioner	Poor quality exaggerates the role of shoddy products	JD.com
Mazda Motor Corporation	Battery Reconditioner	I haven't used it yet.	JD.com
Mazda Motor Corporation	Battery Reconditioner	Use it and then price it.	JD.com
Mazda Motor Corporation	Battery Reconditioner	The packaging is very good and the logistics is fast.	JD.com
Mazda Motor Corporation	Battery Reconditioner	Packed completely, normal use, the effect will be known only after use, the	JD.com
Mazda Motor Corporation	Battery Reconditioner	The cell phone charging repairer works so well, it really doesn't jam the shield anymore	JD.com
Mazda Motor Corporation	Battery Reconditioner	That's a fast delivery!	JD.com
Mazda Motor Corporation	Battery Reconditioner	praise first five-star praise it night to use in order to evaluate the good or bad hope to use a very good in the purchase of a!	JD.com
Mazda Motor Corporation	Battery Reconditioner	Comments	JD.com
Mazda Motor Corporation	Battery Reconditioner	This user feels the product is better	JD.com
Mazda Motor Corporation	Battery Reconditioner	Poor quality exaggerates the role of shoddy products	JD.com
Mazda Motor Corporation	Battery Reconditioner	I haven't used it yet.	JD.com
Mazda Motor Corporation	Battery Reconditioner	Use it and then price it.	JD.com
Mazda Motor Corporation	Battery Reconditioner	The packaging is very good and the logistics is fast.	JD.com
Mazda Motor Corporation	Battery Reconditioner	Packed completely, normal use, the effect will be known only after use, the	JD.com
Mazda Motor Corporation	Battery Reconditioner	The cell phone charging repairer works so well, it really doesn't jam the shield anymore	JD.com
Mazda Motor Corporation	Battery Reconditioner	That's a fast delivery!	JD.com
Mazda Motor Corporation	Battery Reconditioner	praise first five-star praise it night to use in order to evaluate the good or bad hope to use a very good in the purchase of a!	JD.com
Mazda Motor Corporation	Battery Reconditioner	Comments	JD.com
Mazda Motor Corporation	Battery Reconditioner	This user feels the product is better	JD.com
Mazda Motor Corporation	Battery Reconditioner	Poor quality exaggerates the role of shoddy products	JD.com
Mazda Motor Corporation	Battery Reconditioner	I haven't used it yet.	JD.com
Mazda Motor Corporation	Battery Reconditioner	Use it and then price it.	JD.com
Mazda Motor Corporation	Battery Reconditioner	The packaging is very good and the logistics is fast.	JD.com
Mazda Motor Corporation	Battery Reconditioner	Packed completely, normal use, the effect will be known only after use, the	JD.com
Mazda Motor Corporation	Battery Reconditioner	The cell phone charging repairer works so well, it really doesn't jam the shield anymore	JD.com
Mazda Motor Corporation	Battery Reconditioner	That's a fast delivery!	JD.com
Mazda Motor Corporation	Battery Reconditioner	praise first five-star praise it night to use in order to evaluate the good or bad hope to use a very good in the purchase of a!	JD.com
Mazda Motor Corporation	Battery Reconditioner	Comments	JD.com
Mazda Motor Corporation	Battery Reconditioner	This user feels the product is better	JD.com
Mazda Motor Corporation	Battery Reconditioner	Poor quality exaggerates the role of shoddy products	JD.com
Mazda Motor Corporation	Battery Reconditioner	I haven't used it yet.	JD.com
Mazda Motor Corporation	Battery Reconditioner	Use it and then price it.	JD.com
Mazda Motor Corporation	Battery Reconditioner	The packaging is very good and the logistics is fast.	JD.com

Mazda Motor Corporation	Battery Reconditioner	Packed completely, normal use, the effect will be known only after use, the	JD.com
Mazda Motor Corporation	Battery Reconditioner	The cell phone charging repairer works so well, it really doesn't jam the shield anymore	JD.com
Mazda Motor Corporation	Battery Reconditioner	That's a fast delivery!	JD.com
Mazda Motor Corporation	Battery Reconditioner	praise first five-star praise it night to use in order to evaluate the good or bad hope to use a very good in the purchase of a!	JD.com
Mazda Motor Corporation	Battery Reconditioner	Comments	JD.com
Mazda Motor Corporation	Battery Reconditioner	This user feels the product is better	JD.com
Mazda Motor Corporation	Battery Reconditioner	Poor quality exaggerates the role of shoddy products	JD.com
Mazda Motor Corporation	Battery Reconditioner	I haven't used it yet.	JD.com
Mazda Motor Corporation	Battery Reconditioner	Use it and then price it.	JD.com
Mazda Motor Corporation	Battery Reconditioner	The packaging is very good and the logistics is fast.	JD.com
Mazda Motor Corporation	Battery Reconditioner	Packed completely, normal use, the effect will be known only after use, the	JD.com
Mazda Motor Corporation	Battery Reconditioner	The cell phone charging repairer works so well, it really doesn't jam the shield anymore	JD.com
Mazda Motor Corporation	Battery Reconditioner	That's a fast delivery!	JD.com
Mazda Motor Corporation	Battery Reconditioner	praise first five-star praise it night to use in order to evaluate the good or bad hope to use a very good in the purchase of a!	JD.com
Mazda Motor Corporation	Battery Reconditioner	Comments	JD.com
Mazda Motor Corporation	Battery Reconditioner	This user feels the product is better	JD.com
Mazda Motor Corporation	Battery Reconditioner	Poor quality exaggerates the role of shoddy products	JD.com
Mazda Motor Corporation	Battery Reconditioner	I haven't used it yet.	JD.com
Mazda Motor Corporation	Battery Reconditioner	Use it and then price it.	JD.com
Mazda Motor Corporation	Battery Reconditioner	The packaging is very good and the logistics is fast.	JD.com
Mazda Motor Corporation	Battery Reconditioner	Packed completely, normal use, the effect will be known only after use, the	JD.com
Mazda Motor Corporation	Battery Reconditioner	The cell phone charging repairer works so well, it really doesn't jam the shield anymore	JD.com
Mazda Motor Corporation	Battery Reconditioner	That's a fast delivery!	JD.com
Mazda Motor Corporation	Battery Reconditioner	praise first five-star praise it night to use in order to evaluate the good or bad hope to use a very good in the purchase of a!	JD.com
Mazda Motor Corporation	Battery Reconditioner	Comments	JD.com
Mazda Motor Corporation	Battery Reconditioner	This user feels the product is better	JD.com
Mazda Motor Corporation	Battery Reconditioner	Poor quality exaggerates the role of shoddy products	JD.com
Mazda Motor Corporation	Battery Reconditioner	I haven't used it yet.	JD.com
Mazda Motor Corporation	Battery Reconditioner	Use it and then price it.	JD.com
Mazda Motor Corporation	Battery Reconditioner	The packaging is very good and the logistics is fast.	JD.com
Mazda Motor Corporation	Battery Reconditioner	Packed completely, normal use, the effect will be known only after use, the	JD.com
Mazda Motor Corporation	Battery Reconditioner	The cell phone charging repairer works so well, it really doesn't jam the shield anymore	JD.com
Mazda Motor Corporation	Battery Reconditioner	That's a fast delivery!	JD.com
Mazda Motor Corporation	Battery Reconditioner	praise first five-star praise it night to use in order to evaluate the good or bad hope to use a very good in the purchase of a!	JD.com
Mazda Motor Corporation	Battery Reconditioner	Comments	JD.com
Mazda Motor Corporation	Battery Reconditioner	This user feels the product is better	JD.com
Mazda Motor Corporation	Battery Reconditioner	Poor quality exaggerates the role of shoddy products	JD.com

Mazda Motor Corporation	Battery Reconditioner	I haven't used it yet.	JD.com
Mazda Motor Corporation	Battery Reconditioner	Use it and then price it.	JD.com
Mazda Motor Corporation	Battery Reconditioner	The packaging is very good and the logistics is fast.	JD.com
Mazda Motor Corporation	Battery Reconditioner	Packed completely, normal use, the effect will be known only after use, the	JD.com
Mazda Motor Corporation	Battery Reconditioner	The cell phone charging repairer works so well, it really doesn't jam the shield anymore	JD.com
Mazda Motor Corporation	Battery Reconditioner	That's a fast delivery!	JD.com
Mazda Motor Corporation	Battery Reconditioner	praise first five-star praise it night to use in order to evaluate the good or bad hope to use a very good in the purchase of a!	JD.com
Mazda Motor Corporation	Battery Reconditioner	Comments	JD.com
Mazda Motor Corporation	Battery Reconditioner	This user feels the product is better	JD.com
Mazda Motor Corporation	Battery Reconditioner	Poor quality exaggerates the role of shoddy products	JD.com
Mazda Motor Corporation	Battery Reconditioner	I haven't used it yet.	JD.com
Mazda Motor Corporation	Battery Reconditioner	Use it and then price it.	JD.com
Mazda Motor Corporation	Battery Reconditioner	The packaging is very good and the logistics is fast.	JD.com
Mazda Motor Corporation	Battery Reconditioner	Packed completely, normal use, the effect will be known only after use, the	JD.com
Mazda Motor Corporation	Battery Reconditioner	The cell phone charging repairer works so well, it really doesn't jam the shield anymore	JD.com
Mazda Motor Corporation	Battery Reconditioner	That's a fast delivery!	JD.com
Mazda Motor Corporation	Battery Reconditioner	praise first five-star praise it night to use in order to evaluate the good or bad hope to use a very good in the purchase of a!	JD.com
Mazda Motor Corporation	Battery Reconditioner	Comments	JD.com
Mazda Motor Corporation	Battery Reconditioner	This user feels the product is better	JD.com
Mazda Motor Corporation	Battery Reconditioner	Poor quality exaggerates the role of shoddy products	JD.com
Mazda Motor Corporation	Battery Reconditioner	I haven't used it yet.	JD.com
Mazda Motor Corporation	Battery Reconditioner	Use it and then price it.	JD.com
Mazda Motor Corporation	Battery Reconditioner	The packaging is very good and the logistics is fast.	JD.com
Mazda Motor Corporation	Battery Reconditioner	Packed completely, normal use, the effect will be known only after use, the	JD.com
Mazda Motor Corporation	Battery Reconditioner	The cell phone charging repairer works so well, it really doesn't jam the shield anymore	JD.com
Mazda Motor Corporation	Battery Reconditioner	That's a fast delivery!	JD.com
Mazda Motor Corporation	Battery Reconditioner	praise first five-star praise it night to use in order to evaluate the good or bad hope to use a very good in the purchase of a!	JD.com
Mazda Motor Corporation	Battery Reconditioner	Comments	JD.com
Mazda Motor Corporation	Battery Reconditioner	This user feels the product is better	JD.com
Mazda Motor Corporation	Battery Reconditioner	Poor quality exaggerates the role of shoddy products	JD.com
Mazda Motor Corporation	Battery Reconditioner	I haven't used it yet.	JD.com
Mazda Motor Corporation	Battery Reconditioner	Use it and then price it.	JD.com
Mazda Motor Corporation	Battery Reconditioner	The packaging is very good and the logistics is fast.	JD.com
Mazda Motor Corporation	Battery Reconditioner	Packed completely, normal use, the effect will be known only after use, the	JD.com
Mazda Motor Corporation	Battery Reconditioner	The cell phone charging repairer works so well, it really doesn't jam the shield anymore	JD.com
Mazda Motor Corporation	Battery Reconditioner	That's a fast delivery!	JD.com
Mazda Motor Corporation	Battery Reconditioner	praise first five-star praise it night to use in order to evaluate the good or bad hope to use a very good in the purchase of a!	JD.com

Mazda Motor Corporation	Battery Reconditioner	Comments	JD.com
Mazda Motor Corporation	Battery Reconditioner	This user feels the product is better	JD.com
Mazda Motor Corporation	Battery Reconditioner	Poor quality exaggerates the role of shoddy products	JD.com
Mazda Motor Corporation	Battery Reconditioner	I haven't used it yet.	JD.com
Mazda Motor Corporation	Battery Reconditioner	Use it and then price it.	JD.com
Mazda Motor Corporation	Battery Reconditioner	The packaging is very good and the logistics is fast.	JD.com
Mazda Motor Corporation	Battery Reconditioner	Packed completely, normal use, the effect will be known only after use, the	JD.com
Mazda Motor Corporation	Battery Reconditioner	The cell phone charging repairer works so well, it really doesn't jam the shield anymore	JD.com
Mazda Motor Corporation	Battery Reconditioner	That's a fast delivery!	JD.com
Mazda Motor Corporation	Battery Reconditioner	praise first five-star praise it night to use in order to evaluate the good or bad hope to use a very good in the purchase of a!	JD.com
Mazda Motor Corporation	Battery Reconditioner	Comments	JD.com
Mazda Motor Corporation	Battery Reconditioner	This user feels the product is better	JD.com
Mazda Motor Corporation	Battery Reconditioner	Poor quality exaggerates the role of shoddy products	JD.com
Mazda Motor Corporation	Battery Reconditioner	I haven't used it yet.	JD.com
Mazda Motor Corporation	Battery Reconditioner	Use it and then price it.	JD.com
Mazda Motor Corporation	Battery Reconditioner	The packaging is very good and the logistics is fast.	JD.com
Mazda Motor Corporation	Battery Reconditioner	Packed completely, normal use, the effect will be known only after use, the	JD.com
Mazda Motor Corporation	Battery Reconditioner	The cell phone charging repairer works so well, it really doesn't jam the shield anymore	JD.com
Mazda Motor Corporation	Battery Reconditioner	That's a fast delivery!	JD.com
Mazda Motor Corporation	Battery Reconditioner	praise first five-star praise it night to use in order to evaluate the good or bad hope to use a very good in the purchase of a!	JD.com
Mazda Motor Corporation	Battery Reconditioner	Comments	JD.com
Mazda Motor Corporation	Battery Reconditioner	This user feels the product is better	JD.com
Mazda Motor Corporation	Battery Reconditioner	Poor quality exaggerates the role of shoddy products	JD.com
Mazda Motor Corporation	Battery Reconditioner	I haven't used it yet.	JD.com
Mazda Motor Corporation	Battery Reconditioner	Use it and then price it.	JD.com
Mazda Motor Corporation	Battery Reconditioner	The packaging is very good and the logistics is fast.	JD.com
Mazda Motor Corporation	Battery Reconditioner	Packed completely, normal use, the effect will be known only after use, the	JD.com
Mazda Motor Corporation	Battery Reconditioner	The cell phone charging repairer works so well, it really doesn't jam the shield anymore	JD.com
Mazda Motor Corporation	Battery Reconditioner	That's a fast delivery!	JD.com
Mazda Motor Corporation	Battery Reconditioner	praise first five-star praise it night to use in order to evaluate the good or bad hope to use a very good in the purchase of a!	JD.com
Mazda Motor Corporation	Battery Reconditioner	Comments	JD.com
Mazda Motor Corporation	Battery Reconditioner	This user feels the product is better	JD.com
Mazda Motor Corporation	Battery Reconditioner	Poor quality exaggerates the role of shoddy products	JD.com
Mazda Motor Corporation	Battery Reconditioner	I haven't used it yet.	JD.com
Mazda Motor Corporation	Battery Reconditioner	Use it and then price it.	JD.com
Mazda Motor Corporation	Battery Reconditioner	The packaging is very good and the logistics is fast.	JD.com
Mazda Motor Corporation	Battery Reconditioner	Packed completely, normal use, the effect will be known only after use, the	JD.com
Mazda Motor Corporation	Battery Reconditioner	The cell phone charging repairer works so well, it really doesn't jam the shield anymore	JD.com

Mazda Motor Corporation	Battery Reconditioner	That's a fast delivery!	JD.com
Mazda Motor Corporation	Battery Reconditioner	praise first five-star praise it night to use in order to evaluate the good or bad hope to use a very good in the purchase of a!	JD.com
Mazda Motor Corporation	Battery Reconditioner	Comments	JD.com
Mazda Motor Corporation	Battery Reconditioner	This user feels the product is better	JD.com
Mazda Motor Corporation	Battery Reconditioner	Poor quality exaggerates the role of shoddy products	JD.com
Mazda Motor Corporation	Battery Reconditioner	I haven't used it yet.	JD.com
Mazda Motor Corporation	Battery Reconditioner	Use it and then price it.	JD.com
Mazda Motor Corporation	Battery Reconditioner	The packaging is very good and the logistics is fast.	JD.com
Mazda Motor Corporation	Battery Reconditioner	Packed completely, normal use, the effect will be known only after use, the	JD.com
Mazda Motor Corporation	Battery Reconditioner	The cell phone charging repairer works so well, it really doesn't jam the shield anymore	JD.com
Mazda Motor Corporation	Battery Reconditioner	That's a fast delivery!	JD.com
Mazda Motor Corporation	Battery Reconditioner	praise first five-star praise it night to use in order to evaluate the good or bad hope to use a very good in the purchase of a!	JD.com
Mazda Motor Corporation	Battery Reconditioner	Comments	JD.com
Mazda Motor Corporation	Battery Reconditioner	This user feels the product is better	JD.com
Mazda Motor Corporation	Battery Reconditioner	Poor quality exaggerates the role of shoddy products	JD.com
Mazda Motor Corporation	Battery Reconditioner	I haven't used it yet.	JD.com
Mazda Motor Corporation	Battery Reconditioner	Use it and then price it.	JD.com
Mazda Motor Corporation	Battery Reconditioner	The packaging is very good and the logistics is fast.	JD.com
Mazda Motor Corporation	Battery Reconditioner	Packed completely, normal use, the effect will be known only after use, the	JD.com
Mazda Motor Corporation	Battery Reconditioner	The cell phone charging repairer works so well, it really doesn't jam the shield anymore	JD.com
Mazda Motor Corporation	Battery Reconditioner	That's a fast delivery!	JD.com
Mazda Motor Corporation	Battery Reconditioner	praise first five-star praise it night to use in order to evaluate the good or bad hope to use a very good in the purchase of a!	JD.com
Mazda Motor Corporation	Battery Reconditioner	Comments	JD.com
Mazda Motor Corporation	Battery Reconditioner	This user feels the product is better	JD.com
Mazda Motor Corporation	Battery Reconditioner	Poor quality exaggerates the role of shoddy products	JD.com
Mazda Motor Corporation	Battery Reconditioner	I haven't used it yet.	JD.com
Mazda Motor Corporation	Battery Reconditioner	Use it and then price it.	JD.com
Mazda Motor Corporation	Battery Reconditioner	The packaging is very good and the logistics is fast.	JD.com
Mazda Motor Corporation	Battery Reconditioner	Packed completely, normal use, the effect will be known only after use, the	JD.com
Mazda Motor Corporation	Battery Reconditioner	The cell phone charging repairer works so well, it really doesn't jam the shield anymore	JD.com
Mazda Motor Corporation	Battery Reconditioner	That's a fast delivery!	JD.com
Mazda Motor Corporation	Battery Reconditioner	praise first five-star praise it night to use in order to evaluate the good or bad hope to use a very good in the purchase of a!	JD.com
Mazda Motor Corporation	Battery Reconditioner	Comments	JD.com
Mazda Motor Corporation	Battery Reconditioner	This user feels the product is better	JD.com
Mazda Motor Corporation	Battery Reconditioner	Poor quality exaggerates the role of shoddy products	JD.com
Mazda Motor Corporation	Battery Reconditioner	I haven't used it yet.	JD.com
Mazda Motor Corporation	Battery Reconditioner	Use it and then price it.	JD.com

Mazda Motor Corporation	Battery Reconditioner	The packaging is very good and the logistics is fast.	JD.com
Mazda Motor Corporation	Battery Reconditioner	Packed completely, normal use, the effect will be known only after use, the	JD.com
Mazda Motor Corporation	Battery Reconditioner	The cell phone charging repairer works so well, it really doesn't jam the shield anymore	JD.com
Mazda Motor Corporation	Battery Reconditioner	That's a fast delivery!	JD.com
Mazda Motor Corporation	Battery Reconditioner	praise first five-star praise it night to use in order to evaluate the good or bad hope to use a very good in the purchase of a!	JD.com
Mazda Motor Corporation	Battery Reconditioner	Comments	JD.com
Mazda Motor Corporation	Battery Reconditioner	This user feels the product is better	JD.com
Mazda Motor Corporation	Battery Reconditioner	Poor quality exaggerates the role of shoddy products	JD.com
Mazda Motor Corporation	Battery Reconditioner	I haven't used it yet.	JD.com
Mazda Motor Corporation	Battery Reconditioner	Use it and then price it.	JD.com
Mazda Motor Corporation	Battery Reconditioner	The packaging is very good and the logistics is fast.	JD.com
Mazda Motor Corporation	Battery Reconditioner	Packed completely, normal use, the effect will be known only after use, the	JD.com
Mazda Motor Corporation	Battery Reconditioner	The cell phone charging repairer works so well, it really doesn't jam the shield anymore	JD.com
Mazda Motor Corporation	Battery Reconditioner	That's a fast delivery!	JD.com
Mazda Motor Corporation	Battery Reconditioner	praise first five-star praise it night to use in order to evaluate the good or bad hope to use a very good in the purchase of a!	JD.com
Mazda Motor Corporation	Battery Reconditioner	Comments	JD.com
Mazda Motor Corporation	Battery Reconditioner	This user feels the product is better	JD.com
Mazda Motor Corporation	Battery Reconditioner	Poor quality exaggerates the role of shoddy products	JD.com
Mazda Motor Corporation	Battery Reconditioner	I haven't used it yet.	JD.com
Mazda Motor Corporation	Battery Reconditioner	Use it and then price it.	JD.com
Mazda Motor Corporation	Battery Reconditioner	The packaging is very good and the logistics is fast.	JD.com
Mazda Motor Corporation	Battery Reconditioner	Packed completely, normal use, the effect will be known only after use, the	JD.com
Mazda Motor Corporation	Battery Reconditioner	The cell phone charging repairer works so well, it really doesn't jam the shield anymore	JD.com
Mazda Motor Corporation	Battery Reconditioner	That's a fast delivery!	JD.com
Mazda Motor Corporation	Battery Reconditioner	praise first five-star praise it night to use in order to evaluate the good or bad hope to use a very good in the purchase of a!	JD.com
Mazda Motor Corporation	Battery Reconditioner	Comments	JD.com
Mazda Motor Corporation	Battery Reconditioner	This user feels the product is better	JD.com
Mazda Motor Corporation	Battery Reconditioner	Poor quality exaggerates the role of shoddy products	JD.com
Mazda Motor Corporation	Battery Reconditioner	I haven't used it yet.	JD.com
Mazda Motor Corporation	Battery Reconditioner	Use it and then price it.	JD.com
Mazda Motor Corporation	Battery Reconditioner	The packaging is very good and the logistics is fast.	JD.com
Mazda Motor Corporation	Battery Reconditioner	Packed completely, normal use, the effect will be known only after use, the	JD.com
Mazda Motor Corporation	Battery Reconditioner	The cell phone charging repairer works so well, it really doesn't jam the shield anymore	JD.com
Mazda Motor Corporation	Battery Reconditioner	That's a fast delivery!	JD.com
Mazda Motor Corporation	Battery Reconditioner	praise first five-star praise it night to use in order to evaluate the good or bad hope to use a very good in the purchase of a!	JD.com
Mazda Motor Corporation	Battery Reconditioner	Comments	JD.com
Mazda Motor Corporation	Battery Reconditioner	This user feels the product is better	JD.com

Mazda Motor Corporation	Battery Reconditioner	Poor quality exaggerates the role of shoddy products	JD.com
Mazda Motor Corporation	Battery Reconditioner	I haven't used it yet.	JD.com
Mazda Motor Corporation	Battery Reconditioner	Use it and then price it.	JD.com
Mazda Motor Corporation	Battery Reconditioner	The packaging is very good and the logistics is fast.	JD.com
Mazda Motor Corporation	Battery Reconditioner	Packed completely, normal use, the effect will be known only after use, the	JD.com
Mazda Motor Corporation	Battery Reconditioner	The cell phone charging repairer works so well, it really doesn't jam the shield anymore	JD.com
Mazda Motor Corporation	Battery Reconditioner	That's a fast delivery!	JD.com
Mazda Motor Corporation	Battery Reconditioner	praise first five-star praise it night to use in order to evaluate the good or bad hope to use a very good in the purchase of a!	JD.com
Mazda Motor Corporation	Battery Reconditioner	Comments	JD.com
Mazda Motor Corporation	Battery Reconditioner	This user feels the product is better	JD.com
Mazda Motor Corporation	Battery Reconditioner	Poor quality exaggerates the role of shoddy products	JD.com
Mazda Motor Corporation	Battery Reconditioner	I haven't used it yet.	JD.com
Mazda Motor Corporation	Battery Reconditioner	Use it and then price it.	JD.com
Mazda Motor Corporation	Battery Reconditioner	The packaging is very good and the logistics is fast.	JD.com
Mazda Motor Corporation	Battery Reconditioner	Packed completely, normal use, the effect will be known only after use, the	JD.com
Mazda Motor Corporation	Battery Reconditioner	The cell phone charging repairer works so well, it really doesn't jam the shield anymore	JD.com
Mazda Motor Corporation	Battery Reconditioner	That's a fast delivery!	JD.com
Mazda Motor Corporation	Battery Reconditioner	praise first five-star praise it night to use in order to evaluate the good or bad hope to use a very good in the purchase of a!	JD.com
Mazda Motor Corporation	Battery Reconditioner	Comments	JD.com
Mazda Motor Corporation	Battery Reconditioner	This user feels the product is better	JD.com
Mazda Motor Corporation	Battery Reconditioner	Poor quality exaggerates the role of shoddy products	JD.com
Mazda Motor Corporation	Battery Reconditioner	I haven't used it yet.	JD.com
Mazda Motor Corporation	Battery Reconditioner	Use it and then price it.	JD.com
Mazda Motor Corporation	Battery Reconditioner	The packaging is very good and the logistics is fast.	JD.com
Mazda Motor Corporation	Battery Reconditioner	Packed completely, normal use, the effect will be known only after use, the	JD.com
Mazda Motor Corporation	Battery Reconditioner	The cell phone charging repairer works so well, it really doesn't jam the shield anymore	JD.com
Mazda Motor Corporation	Battery Reconditioner	That's a fast delivery!	JD.com
Mazda Motor Corporation	Battery Reconditioner	praise first five-star praise it night to use in order to evaluate the good or bad hope to use a very good in the purchase of a!	JD.com
Mazda Motor Corporation	Battery Reconditioner	Comments	JD.com
Mazda Motor Corporation	Battery Reconditioner	This user feels the product is better	JD.com
Mazda Motor Corporation	Battery Reconditioner	Poor quality exaggerates the role of shoddy products	JD.com
Mazda Motor Corporation	Battery Reconditioner	I haven't used it yet.	JD.com
Mazda Motor Corporation	Battery Reconditioner	Use it and then price it.	JD.com
Mazda Motor Corporation	Battery Reconditioner	The packaging is very good and the logistics is fast.	JD.com
Mazda Motor Corporation	Battery Reconditioner	Packed completely, normal use, the effect will be known only after use, the	JD.com
Mazda Motor Corporation	Battery Reconditioner	The cell phone charging repairer works so well, it really doesn't jam the shield anymore	JD.com
Mazda Motor Corporation	Battery Reconditioner	That's a fast delivery!	JD.com

Mazda Motor Corporation	Battery Reconditioner	praise first five-star praise it night to use in order to evaluate the good or bad hope to use a very good in the purchase of a!	JD.com
Mazda Motor Corporation	Battery Reconditioner	Comments	JD.com
Mazda Motor Corporation	Battery Reconditioner	This user feels the product is better	JD.com
Mazda Motor Corporation	Battery Reconditioner	Poor quality exaggerates the role of shoddy products	JD.com
Mazda Motor Corporation	Battery Reconditioner	I haven't used it yet.	JD.com
Mazda Motor Corporation	Battery Reconditioner	Use it and then price it.	JD.com
Mazda Motor Corporation	Battery Reconditioner	The packaging is very good and the logistics is fast.	JD.com
Mazda Motor Corporation	Battery Reconditioner	Packed completely, normal use, the effect will be known only after use, the	JD.com
Mazda Motor Corporation	Battery Reconditioner	The cell phone charging repairer works so well, it really doesn't jam the shield anymore	JD.com
Mazda Motor Corporation	Battery Reconditioner	That's a fast delivery!	JD.com
Mazda Motor Corporation	Battery Reconditioner	praise first five-star praise it night to use in order to evaluate the good or bad hope to use a very good in the purchase of a!	JD.com
Mazda Motor Corporation	Automatic Battery Repair	This user feels the product is better	JD.com
Mazda Motor Corporation	Automatic Battery Repair	This user feels the product is better	JD.com
Mazda Motor Corporation	Automatic Battery Repair	Just tried three bells, seems no difference with the charger, it's a couple days to see	JD.com
Mazda Motor Corporation	Automatic Battery Repair	It's working fine.	JD.com
Mazda Motor Corporation	Automatic Battery Repair	Tried it and it worked, will try it again for a while and follow up!	JD.com
Mazda Motor Corporation	Automatic Battery Repair	Average, mine doesn't have the features described	JD.com
Mazda Motor Corporation	Automatic Battery Repair	Parameter display is quite comprehensive, use to see the effect and then follow up[cool][cool]	JD.com
Mazda Motor Corporation	Automatic Battery Repair	This user feels the product is better	JD.com
Mazda Motor Corporation	Automatic Battery Repair	Let's use it for a while.	JD.com
Mazda Motor Corporation	Automatic Battery Repair	This user feels the product is better	JD.com
Mazda Motor Corporation	Automatic Battery Repair	Said certificate of conformity complete look quite suitable to use and then follow up evaluation	JD.com
Mazda Motor Corporation	Automatic Battery Repair	I haven't used it yet.[Lots of love][Lots of love]	JD.com
Mazda Motor Corporation	Automatic Battery Repair	This cell phone repairer is really good, small size, big role, really can repair the old cell phone battery, in the past only40% .Now there are89%, and the repairer is stable charging, clear display, and has a certain repair function for the battery. The cell phone is much smoother than before, it's really good value for money.	JD.com
Mazda Motor Corporation	Automatic Battery Repair	the appearance and workmanship is very satisfied, and the business description is consistent with the customer service attitude is very good, the account is very clear, the use of charging effect is good, the speed is also very fast, the quality is very good, this does not have to change the battery, thanks to the business.	JD.com
Mazda Motor Corporation	Automatic Battery Repair	The logistics is very fast, the quality of the goods is very good, repairing the cell phone battery of the gods, but also a good fast charger, does not hurt the phone, charging fast, the appearance is very beautiful, but also equipped with a display, it is very convenient to use. The same as described on the product introduction, and the logistics is very fast, thoughtful service, praise!	JD.com
Mazda Motor Corporation	Automatic Battery Repair	could only be plugged into the power supply to boot, with this charging a few times, you can leave the power supply to boot a eight or nine hours, there has been a big improvement in more than a few times will be better, customer service attitude is also very good, sent me the use of the method, very satisfied with the o	JD.com

Mazda Motor Corporation	Automatic Battery Repair	repair cell phone store to replace the battery repair are very expensive, with this cell phone battery repairer, no longer need to spend money, and do not have to beg, self can do. Not only charging stable in the fast, but also can maintain the phone battery does not hurt the phone, the use of safe and reliable!	JD.com
Mazda Motor Corporation	Automatic Battery Repair	Timely delivery, the product has a certain repair function for the old cell phone battery, and the use of the special square	JD.com
Mazda Motor Corporation	Automatic Battery Repair	Have repaired several cell phones, Apple and Android can be used, my family's cell phone let me repair once, and now usually charging time can also be used!	JD.com
Mazda Motor Corporation	Automatic Battery Repair	Especially good, this section has been charging in use, has repaired a cell phone, now is repairing the second cell phone, when they do not use it can also be lent to others.	JD.com
Mazda Motor Corporation	Automatic Battery Repair	the goods I tried it does have a certain effect of my a car recorder can not open the repair really open and can be used again	JD.com
Mazda Motor Corporation	Automatic Battery Repair	The restorer is charging steadily, it's repairing the battery, and the phone is smoother and lasts much longer than it did in the last few days	JD.com
Mazda Motor Corporation	Automatic Battery Repair	Cell phone battery repairer received, logistics is very fast, the physical look is very good, cell phone battery repairer fine workmanship, precision, there are cell phones have a gradual recovery effect, very like.	JD.com
Mazda Motor Corporation	Automatic Battery Repair	use a lot of days, there is a screen display, according to customer service guidance to insert the charging head to connect the data line can be slow charging power consumption fast or charging into the power of the cell phone repair, but also one time to repair the two cell phones, the first time to repair the phone shutdown, used after the effect of the effect is indeed, and now my two cell phones do not have the previous power consumption.	JD.com
Mazda Motor Corporation	Automatic Battery Repair	artifact finally arrived, after arriving home to open the package, look at the product is very good, immediately began to use up, the effect is really very good, do not have to charge every time you play a cell phone, now the battery durability more durable than before, the future out of the world do not have to worry about the power, a good product, the merchant is also very good to ask a lot of questions are timely response to deal with!	JD.com
Mazda Motor Corporation	Automatic Battery Repair	too powerful, the use of the battery is not so fast waste power, before full power can only be used for two hours, and now normal to play a little bit and then can stick to the evening home from work. Dozens of dollars can be repaired, more cost-effective than changing the phone for the battery, hahaha , really super recommended.	JD.com
Mazda Motor Corporation	Automatic Battery Repair	of time, before10King of Glory phone is out of power, currently can open to13, continued to use, look at the effect of the	JD.com
Mazda Motor Corporation	Automatic Battery Repair	repair artifact, the effect is really not blowing, I now go out and dare to use the phone to play jitterbug, before it is not even dare to think, a small repair artifact can actually play such a great effect, has been recommended to my old sister!	JD.com
Mazda Motor Corporation	Automatic Battery Repair	Battery is not durable power consumption rate is very fast, buy this repair artifact try really good results, now full of electricity can be used for a long time, charging speed is very fast, charging power range is obviously longer than before, the business also said that you can slowly repair the cell phone battery, really good ah, really super good!	JD.com
Mazda Motor Corporation	Automatic Battery Repair	particularly difficult to repair cell phone stores to replace the battery repair are very expensive, with this cell phone battery repairer, no longer need to spend money, and do not have to beg, since they can do, not only charging stable in the fast, but also to care for cell phone batteries do not hurt the phone, the use of security is also reliable, there is a need for you to get started.	JD.com
Mazda Motor Corporation	Automatic Battery Repair	period of time, my phone could only be plugged into the power supply to boot, with this charge a few times, you can leave the power supply to boot a eight or nine hours, there has been a big improvement, in more than a few times will be better, customer service attitude is also very good, very satisfied with the	JD.com

Mazda Motor Corporation	Automatic Battery Repair	This repairer after using the phone directly newborn, it is very good, the phone will not be hot, the response are becoming sensitive, with hands-on can be completed, the battery is quite durable, the capacity is particularly large, the response effect is also super agile, the phone does not appear to be lagging phenomenon, the charging effect is also super fast, and there is no mismatch.	JD.com
Mazda Motor Corporation	Automatic Battery Repair	Cell phone battery repairer quickly received, workmanship is relatively fine, just used a little, can be charged, if you can repair damaged appliances that would be worth it, the back will be followed up, first to give a good review!	JD.com
Mazda Motor Corporation	Automatic Battery Repair	specifically called to tell me that this repair artifact is good, said that the phone battery is more durable than before, are planning to give the elderly to replace the phone. Inadvertently saw this repair device, I did not expect it to work, not	JD.com
Mazda Motor Corporation	Automatic Battery Repair	durable, with other chargers charging is very slow, go to customer service repair people also said that the model is too aging, need to change the battery, I bought this slowly repair, now very durable.	JD.com
Mazda Motor Corporation	Automatic Battery Repair	Battery Repair Magic has been received, used once, now standby effect is very	JD.com
Mazda Motor Corporation	Automatic Battery Repair	Just used once, the next day the cell phone battery durable a lot. From the appearance and workmanship is very satisfied, and the business description is consistent with the customer service attitude is very good, the account is very clear, after the use of charging armor effect is good, speed is also very fast, the quality is very good, this does not have to change the battery, thanks to the business!	JD.com
Mazda Motor Corporation	Automatic Battery Repair	Logistics and shipping are fast, also studying this specialty, the theory is really good for the battery, and very affordable compared to changing the battery	JD.com
Mazda Motor Corporation	Automatic Battery Repair	The effect is very good, note8repair effect is very good, this time do not have to change the battery, after the repair of the phone with a longer time some, and does not heat up, and also automatically clean up the phone system cache garbage, the phone has a significantly smoother, the boss said that often used to repair will be better.	JD.com
Mazda Motor Corporation	Automatic Battery Repair	This cell phone repairer is really good, small size, big role, really can repair the old cell phone battery, in the past only40%now89%, and the repairer is stable charging, clear display, and has a certain repair function for the battery. The cell phone is much smoother than before, it's really good value for money.	JD.com
Mazda Motor Corporation	Automatic Battery Repair	General cell phone batteries will slowly decay after a period of time, this charger if the battery has a repair function on the battery can extend the use of the phone is too good, the development of science and technology to enhance the effectiveness of the praise.	JD.com
Mazda Motor Corporation	Automatic Battery Repair	loss of power consumption is too fast, go to repair cell phone store to replace the battery repair are very expensive, with this cell phone battery restorer, no longer need to spend money, since you can do charging stable and fast, the amount of batteries recovered tomore than90%, truly recommended to the needs of the people	JD.com
Mazda Motor Corporation	Automatic Battery Repair	capacity can charge100/100 now, things are very good thank you boss.	JD.com
Mazda Motor Corporation	Automatic Battery Repair	Battery Repairer received, used this section is very good, my cell phone was full of electricity can only be used for 2hours battery left 80%, repair cell phone batteries play a big role, the effect is really especially magical, has been repaired to100, than the replacement of batteries strong.	JD.com
Mazda Motor Corporation	Automatic Battery Repair	Battery Repairer with a very good cell phone just bought a short time is not resistant to electricity every day need to charge several times a day sometimes go out two rechargeable treasure is not enough to listen to a friend to say there is this thing, then rush to order to buy buy back to use once the battery repaired5%and charging very quickly now I bought back a day or so back to the cell phone battery is already 100%go out and do not have to be afraid of cell phone out of battery battery is very resistant to electricity is very good I'm very satisfied! I am very satisfied with the purchase!	JD.com
Mazda Motor Corporation	Automatic Battery Repair	This user feels the product is better	JD.com
Mazda Motor Corporation	Automatic Battery Repair		JD.com
Mazda Motor Corporation	Automatic Battery Repair	The logistics is fast, the goods are real, simple and good.	JD.com

Mazda Motor Corporation	Automatic Battery Repair	It works well, the repair is okay	JD.com
Mazda Motor Corporation	Automatic Battery Repair	The goods have been received, the logistics is very fast, the service attitude is also very good, the goods and the picture is the same	JD.com
Mazda Motor Corporation	Battery Repairer	Received yesterday, just used yet do not know how the effect of packaging some extrusion inside not how to use after the evaluation, first give a favorable review, the service is very good courier fast against the	JD.com
Mazda Motor Corporation	Battery Repairer	your products back, no matter how to use, to the battery car can not be inserted into, forget the charger also can not be inserted into, you do not rely on people? My time you also do not tell me, you are the car what product, what brand, I sent you what plug, now know, you tell others to buy again when you ask people, what brand of your car, I will send what plug to you, you do not rely on people again.	JD.com
Mazda Motor Corporation	Battery Repairer	This fixer an advertising process to use, I found no work, 不 light, I do not know what is the inertia, ask to deal with	JD.com
Mazda Motor Corporation	Battery Repairer	This fixer works great.	JD.com
Mazda Motor Corporation	Battery Repairer	It looks good it's okay to use it I guess give it 5 stars.	JD.com
Mazda Motor Corporation	Battery Repairer	I just received the item and will comment after using it.	JD.com
Mazda Motor Corporation	Battery Repairer	This user feels the product is better	JD.com
Mazda Motor Corporation	Battery Repairer	Charging is safe, looks like good quality, product works, nice features, aesthetically pleasing	JD.com
Mazda Motor Corporation	Battery Repairer	We don't know if it's fixable yet, let's use it first and see	JD.com
Mazda Motor Corporation	Battery Repairer	It's okay, but it's not obvious that it's working.	JD.com
Mazda Motor Corporation	Battery Repairer	The more you flush, the less you get.	JD.com
Mazda Motor Corporation	Battery Repairer	Goods are very good charging safe and reliable do not worry about charging out of the security will be accidents	JD.com
Mazda Motor Corporation	Battery Repairer	(of an unmarried couple) be close	JD.com
Mazda Motor Corporation	Battery Repairer	Good quality.	JD.com
Mazda Motor Corporation	Battery Repairer	I returned it. Refund back.	JD.com
Mazda Motor Corporation	Battery Repairer	Looks like good quality, will follow up after I'm satisfied with it. It's beautiful.	JD.com
Mazda Motor Corporation	Battery Repairer	This repairer fine to fast delivery to give force but not yet on, people are not in the mound, wait to come back in the test it must border this plaything not see hoo!	JD.com
Mazda Motor Corporation	Battery Repairer	Satisfied with the product	JD.com
Mazda Motor Corporation	Battery Repairer	Use it for a while and then evaluate	JD.com
Mazda Motor Corporation	Battery Repairer	It works great. Thanks.	JD.com
Mazda Motor Corporation	Battery Repairer	Let's see if it works.	JD.com
Mazda Motor Corporation	Battery Repairer	Very good satisfactory favorable comments!	JD.com
Mazda Motor Corporation	Battery Repairer	an excellent	JD.com
Mazda Motor Corporation	Battery Repairer	I received the product and it works just as he said it would. I can trust it.	JD.com
Mazda Motor Corporation	Battery Repairer	Arrival of its fast, only installed on the power, the future effect is good if you give to do selected pass.	JD.com
Mazda Motor Corporation	Battery Repairer	Practical, lightweight, good functionality and fast delivery 5 stars!	JD.com
Mazda Motor Corporation	Battery Repairer	It's working fine. - Kudos.	JD.com
Mazda Motor Corporation	Battery Repairer	Received, good quality, just don't know if it works well.	JD.com
Mazda Motor Corporation	Battery Repairer	The product is as good as described, beautiful and good value for money.	JD.com
Mazda Motor Corporation	Battery Repairer	Fixed, what is the reaction of that fixed light, or how much time does it usually take, and is it fully charged and fixed as well, please enlighten me please.	JD.com
Mazda Motor Corporation	Battery Repairer	Baby has received, logistics is also very fast, with good re-purchase.	JD.com
Mazda Motor Corporation	Battery Repairer	worthwhile	JD.com

Mazda Motor Corporation	Battery Repairer	Superb family buy with confidence	JD.com
Mazda Motor Corporation	Battery Repairer	It's not working yet?	JD.com
Mazda Motor Corporation	Battery Repairer	The product is okay.	JD.com
Mazda Motor Corporation	Battery Repairer	desirable	JD.com
Mazda Motor Corporation	Battery Repairer	Intelligent electric car regulator, really works well, worth buying, give 5 stars.	JD.com
Mazda Motor Corporation	Battery Repairer	The logistics is fast, well packaged and of good quality.	JD.com
Mazda Motor Corporation	Battery Repairer	I'm a pincushion, you're two branches that don't fit together, and the mother's head is a pincushion.	JD.com
Mazda Motor Corporation	Battery Repairer	I received my baby, good quality goods, more satisfied. But the root of my incompatibility does not work.	JD.com
Mazda Motor Corporation	Battery Repairer	Logistics is very fast service attitude is good. It's just that you can't connect this smart pulse regulator with the original charger of the electric car.	JD.com
Mazda Motor Corporation	Battery Repairer	Just received it, looks good quality	JD.com
Mazda Motor Corporation	Battery Repairer	Used it and it was good!	JD.com
Mazda Motor Corporation	Battery Repairer	Plugged in this is the way the lights do not respond at all I do not know what the use of driving today to try to see how the battery reacts if it can then in the evaluation	JD.com
Mazda Motor Corporation	Battery Repairer	Five stars for now! See how it works and then follow up!	JD.com
Mazda Motor Corporation	Battery Repairer	Stocks are fraudulent fakes	JD.com
Mazda Motor Corporation	Battery Repairer	Just received, the packaging is intact, not yet used it, after using and then follow up, first praise!	JD.com
Mazda Motor Corporation	Battery Repairer	Product received, the product is as good as advertised whether or not it works. Is it an IQ tax, use it for a while to know before commenting on the review.	JD.com
Mazda Motor Corporation	Battery Repairer	What fixer, are all cheating, a little use repair effect is not, everyone do not buy this fixer!	JD.com
Mazda Motor Corporation	Battery Repairer	Fast logistics, well packed, good quality, fine workmanship, thank you boss. Great luck. Make a big fortune.	JD.com
Mazda Motor Corporation	Battery Repairer	Product feels good	JD.com
Mazda Motor Corporation	Battery Repairer	I received the goods, just as the anchor said.	JD.com
Mazda Motor Corporation	Battery Repairer	This user feels the product is better	JD.com
Mazda Motor Corporation	Battery Repairer	No voice prompts	JD.com
Mazda Motor Corporation	Battery Repairer	Goods received, logistics is very fast, the quality is very good, very satisfied!	JD.com
Mazda Motor Corporation	Battery Repairer	I don't know if it's any good.	JD.com
Mazda Motor Corporation	Battery Repairer	I'm very happy with the results.	JD.com
Mazda Motor Corporation	Battery Repairer	work at all, I hope the world won't fall for it again, scammer, scam]son.	JD.com
Mazda Motor Corporation	Battery Repairer	This user feels the product is better	JD.com
Mazda Motor Corporation	Battery Repairer	The plugs don't fit mine.	JD.com
Mazda Motor Corporation	Battery Repairer	Logistics is very fast, has not been used, after using the effect, then add a good spell. Thank you boss!	JD.com
Mazda Motor Corporation	Battery Repairer	It's good.	JD.com
Mazda Motor Corporation	Battery Repairer	This user feels the product is better	JD.com
Mazda Motor Corporation	Battery Repairer	This user feels the product is better	JD.com
Mazda Motor Corporation	Battery Repairer	Charges very quickly[big love] [big love] [big love] [big love]	JD.com
Mazda Motor Corporation	Battery Repairer	This user feels the product is better	JD.com
Mazda Motor Corporation	Battery Repairer	The product is a fake children's toy	JD.com
Mazda Motor Corporation	Battery Repairer	Baby received the quality very good reviews 5 stars?	JD.com
Mazda Motor Corporation	Battery Repairer	Looks good! It's in use, so I'll have to watch and see how it works?	JD.com
Mazda Motor Corporation	Battery Repairer	It's fine when you use it, but the two plugs don't fit quite right	JD.com

Mazda Motor Corporation	Battery Repairer	This user feels the product is better	JD.com
Mazda Motor Corporation	Battery Repairer	Just received it. I don't know if it's any good.	JD.com
Mazda Motor Corporation	Battery Repairer	This user feels the product is better	JD.com
Mazda Motor Corporation	Battery Repairer	This user feels the product is better	JD.com
Mazda Motor Corporation	Battery Repairer	This user feels the product is better	JD.com
Mazda Motor Corporation	Battery Repairer	This user feels the product is better	JD.com
Mazda Motor Corporation	Battery Repairer	This user feels the product is better	JD.com
Mazda Motor Corporation	Battery Repairer	This stuff is good!	JD.com
Mazda Motor Corporation	Battery Repairer	Very satisfied. Worth the money, this is a great product[love it!]	JD.com
Mazda Motor Corporation	Battery Repairer	The quality is pretty good.	JD.com
Mazda Motor Corporation	Battery Repairer	This user feels the product is better	JD.com
Mazda Motor Corporation	Battery Repairer	What about all that good stuff you said you'd fix the battery?	JD.com
Mazda Motor Corporation	Battery Repairer	This user feels the product is better	JD.com
Mazda Motor Corporation	Battery Repairer	Haven't used it yet.	JD.com
Mazda Motor Corporation	Battery Repairer	This user feels the product is better	JD.com
Mazda Motor Corporation	Battery Repairer	Cost effective, easy to use and should work well!	JD.com
Mazda Motor Corporation	Battery Repairer	Very good quality inexpensive product very good	JD.com
Mazda Motor Corporation	Battery Repairer	This user feels the product is better	JD.com
Mazda Motor Corporation	Battery Repairer	The logistics is very fast and the product is very good	JD.com
Mazda Motor Corporation	Battery Repairer	The battery reconditioner has been received. It is as described by the merchant, and the use of the square 1	JD.com
Mazda Motor Corporation	Battery Repairer	This user feels the product is better	JD.com
Mazda Motor Corporation	Battery Repairer	This user feels the product is better	JD.com
Mazda Motor Corporation	Battery Repairer	Charging is fine, using it quite well, it works well	JD.com
Mazda Motor Corporation	Battery Repairer	Charging is fine, using it quite well, it works well and is worth having!	JD.com
Mazda Motor Corporation	Battery Repairer	Charging works, it works well:, using it quite well:	JD.com
Mazda Motor Corporation	Battery Repairer	use of the effect and then evaluate, the first evaluation is good!	JD.com
Mazda Motor Corporation	Battery Repairer	Charging can be, on the battery to see a few days to see if there is no effect, before deciding whether it is good or not	JD.com
Mazda Motor Corporation	Battery Repairer	How did so many positive reviews come about, it doesn't help at all. People don't buy so bad.	JD.com
Mazda Motor Corporation	Battery Repairer	This user feels the product is better	JD.com
Mazda Motor Corporation	Battery Repairer	not see, but the electricity is full of no automatic power off!	JD.com
Mazda Motor Corporation	Battery Repairer	This user feels the product is better	JD.com
Mazda Motor Corporation	Battery Repairer	I used to save inches between, can run 10 kilometers more power a lot of uphill very powerful, the price is not expensive, very good use!	JD.com
Mazda Motor Corporation	Battery Repairer	Let's use it for a while.	JD.com
Mazda Motor Corporation	Battery Repairer	This user feels the product is better	JD.com
Mazda Motor Corporation	Battery Repairer	It's working fine.	JD.com
Mazda Motor Corporation	Battery Repairer	acceptable	JD.com
Mazda Motor Corporation	Battery Repairer	It still works, but we'll just have to wait and see if it fixes the battery.	JD.com
Mazda Motor Corporation	Battery Repairer	It has not been used yet.	JD.com
Mazda Motor Corporation	Battery Repairer	Baby received, as described, love it!	JD.com

Mazda Motor Corporation	Battery Repairer	This user feels the product is better	JD.com
Mazda Motor Corporation	Battery Repairer	Review after use!	JD.com
Mazda Motor Corporation	Battery Repairer	It looks like it fits so far, waiting to see how it looks in the car tomorrow.	JD.com
Mazda Motor Corporation	Battery Repairer	The charge is super fast worth it [big love] [big love] [big love] [big love] Families, charge it!!!! [Big Love]	JD.com
Mazda Motor Corporation	Battery Repairer	This product cannot be automatically disconnected	JD.com
Mazda Motor Corporation	Battery Repairer	I'm using it now, so we'll see how it works.	JD.com
Mazda Motor Corporation	Battery Repairer	The merchandise works great.	JD.com
Mazda Motor Corporation	Battery Repairer	This user feels the product is better	JD.com
Mazda Motor Corporation	Battery Repairer	This product has been received, the effect will have to see how the late situation, fast shipping logistics is also very fast, the effect is good next time will patronize! I hope to have good expectations!	JD.com
Mazda Motor Corporation	Battery Repairer	It looks okay! Thanks!	JD.com
Mazda Motor Corporation	Battery Repairer	This user feels the product is better	JD.com
UOHCGFA Digital Appliance Flagship Store	Battery Reconditioner	I never thought I could get such a good baby at such an affordable price, it's very good value for money, I love it!	JD.com
UOHCGFA Digital Appliance Flagship Store	Battery Reconditioner	Logistics is fast, is charging, hope it works well	JD.com
UOHCGFA Digital Appliance Flagship Store	Battery Reconditioner	This user feels the product is better	JD.com
UOHCGFA Digital Appliance Flagship Store	Battery Reconditioner	I have received the cell phone repairer adapter. Used it for a while. Very satisfied.	JD.com
UOHCGFA Digital Appliance Flagship Store	Battery Reconditioner	This user feels the product is better	JD.com
UOHCGFA Digital Appliance Flagship Store	Battery Reconditioner	This user feels the product is better	JD.com
UOHCGFA Digital Appliance Flagship Store	Battery Reconditioner	The goods have been received, very satisfied, with the picture sent exactly the same, the service is also good, fast delivery!	JD.com
UOHCGFA Digital Appliance Flagship Store	Battery Reconditioner	Received thanks to the boss rated 5 stars but still have to use it don't know how effective it is	JD.com
UOHCGFA Digital Appliance Flagship Store	Battery Reconditioner	an excellent	JD.com
UOHCGFA Digital Appliance Flagship Store	Battery Reconditioner	repair the battery and clean up cell phone garbage, the phone runs like new well yo! [smile] [smile] [smile] [smile] [smile].	JD.com
UOHCGFA Digital Appliance Flagship Store	Battery Reconditioner	The package has been received, the packaging is complete, used once, can not see the effect, more use a few times may be better. Every thing is not so fast effect.	JD.com
UOHCGFA Digital Appliance Flagship Store	Battery Reconditioner	This user feels the product is better	JD.com
UOHCGFA Digital Appliance Flagship Store	Battery Reconditioner	This user feels the product is better	JD.com
UOHCGFA Digital Appliance Flagship Store	Battery Reconditioner	This user feels the product is better	JD.com
UOHCGFA Digital Appliance Flagship Store	Battery Reconditioner	Received the goods look good, do not know if it is good to use	JD.com
UOHCGFA Digital Appliance Flagship Store	Battery Reconditioner	Charger received good quality, cheaper than the physical store	JD.com
UOHCGFA Digital Appliance Flagship Store	Battery Reconditioner	Baby received packaging did not break the business also sent a data line value for money so cheap price can buy affordable things, five-star praise!	JD.com
UOHCGFA Digital Appliance Flagship Store	Battery Reconditioner	Ships quickly, is easy to use, and boosts the speed of your phone.	JD.com

UOHCGFA Digital Appliance Flagship Store	Battery Reconditioner	can also be distributed a data line, a tube of three plugs is particularly convenient conscience boss trustworthy. Thank you!	JD.com
UOHCGFA Digital Appliance Flagship Store	Battery Reconditioner	Carrying description: I looked at your product I really don't want to use sleepy for the inside mentioned that will lose part of the content	JD.com
UOHCGFA Digital Appliance Flagship Store	Battery Reconditioner	This user feels the product is better	JD.com
UOHCGFA Digital Appliance Flagship Store	Battery Reconditioner	I don't know how well it works, I'll tell you after I use it	JD.com
UOHCGFA Digital Appliance Flagship Store	Battery Reconditioner	Experience the effect is average, not as god as blown on the video, doubt if it is regular goods.	JD.com
UOHCGFA Digital Appliance Flagship Store	Battery Reconditioner	This user feels the product is better	JD.com
UOHCGFA Digital Appliance Flagship Store	Battery Reconditioner	Just received this item and all is well! Have yet to try it out! Hopefully it will fix your cell phone battery as stated online! Will add more comments if I wish! Thanks!	JD.com
UOHCGFA Digital Appliance Flagship Store	Battery Reconditioner	Received it not sure if it works or not, it's sandwiched in the middle anyway.	JD.com
UOHCGFA Digital Appliance Flagship Store	Battery Reconditioner	This user feels the product is better	JD.com
UOHCGFA Digital Appliance Flagship Store	Battery Reconditioner	A great cell phone adapter, worth every penny.	JD.com
UOHCGFA Digital Appliance Flagship Store	Battery Reconditioner	This user feels the product is better	JD.com
UOHCGFA Digital Appliance Flagship Store	Battery Reconditioner	good quality	JD.com
UOHCGFA Digital Appliance Flagship Store	Battery Reconditioner	It works.	JD.com
UOHCGFA Digital Appliance Flagship Store	Battery Reconditioner	Things received logistics very fast service to good looking things are quite beautiful, I used in the follow-up, have to buy friends quickly come to hold it.	JD.com
UOHCGFA Digital Appliance Flagship Store	Battery Reconditioner	This user feels the product is better	JD.com
UOHCGFA Digital Appliance Flagship Store	Battery Reconditioner	There's no point in repairing the battery.	JD.com
UOHCGFA Digital Appliance Flagship Store	Battery Reconditioner	Product received, fast logistics, used a few times, charging is very good, rechargeable batteries are not hot, and power consumption is slow, durable a lot of	JD.com
UOHCGFA Digital Appliance Flagship Store	Battery Reconditioner	Fast shipping, use it and then evaluate	JD.com
UOHCGFA Digital Appliance Flagship Store	Battery Reconditioner	Very satisfied with the charging effect is particularly good worth recommending	JD.com
UOHCGFA Digital Appliance Flagship Store	Battery Reconditioner	This restorer works exceptionally well and works exceptionally well. This way the battery is not easy to be broken. It can be used for a long time	JD.com
UOHCGFA Digital Appliance Flagship Store	Battery Reconditioner	Compact, that can charge your cell phone, but also for hand repair electric chi, good function, I'm going to tell my friends to buy it too.	JD.com
UOHCGFA Digital Appliance Flagship Store	Battery Reconditioner	Baby received. Great quality and not too expensive. Repair effect is very strong. Works great, exactly like the picture.	JD.com
UOHCGFA Digital Appliance Flagship Store	Battery Reconditioner	problem came, looked at a few buy this configuration of high effect cell phone battery repair maintenance cell phone adapter, easy to operate, plug in the power supply will start charging repair, after a night of use repair, power on slow power drop, cell phone electricity durable, repair the phone after the Internet is not lagging, and does not get hot, satisfied!	JD.com
UOHCGFA Digital Appliance Flagship Store	Battery Reconditioner	with a good price is cheap, things look good, use two before coming to the evaluation, the battery is a little more durable than before	JD.com
UOHCGFA Digital Appliance Flagship Store	Battery Reconditioner	Goods received, used, but also really to the description of the same, hope that friends quickly buy!	JD.com

UOHCGFA Digital Appliance Flagship Store	Battery Reconditioner	Battery Repairer is good, very good to use a plug can repair the cell phone battery, after repairing the battery is durable a lot of	JD.com
UOHCGFA Digital Appliance Flagship Store	Battery Reconditioner	Received the cell phone repair clamor to use, simple and good, good operation, five-star praise!	JD.com
UOHCGFA Digital Appliance Flagship Store	Battery Reconditioner	The battery reconditioner works great, good quality, good repair.	JD.com
UOHCGFA Digital Appliance Flagship Store	Battery Reconditioner	not have	JD.com
Fuyi Living	Battery Reconditioner	not have	JD.com
Puning Dental	Battery Repairer	The restorer has been received. Works very, very well, very satisfied!	JD.com
Puning Dental	Cell Phone Battery Repairer	This user feels the product is better	JD.com
Puning Dental	Cell Phone Battery Repairer	I haven't received the shipment till today.	JD.com
Puning Dental	Cell Phone Battery Repairer	anymore. Have returned it to the neighborhood collection point.	JD.com
Puning Dental	Cell Phone Battery Repairer	This user did not fill in a rating	JD.com
Puning Dental	Cell Phone Battery Repairer	The logistics was fast! The sockets on both ends fit, and I was able to use it tonight, and the merchant describes that it should be used for a couple of weeks in a row!	JD.com
Puning Dental	Cell Phone Battery Repairer	This user feels the product is better	JD.com
Puning Dental	Cell Phone Battery Repairer	This user feels the product is better	JD.com
Puning Dental	Cell Phone Battery Repairer	The goods have been received, very satisfied, the service is excellent, recommended, thank you!	JD.com
Puning Dental	Cell Phone Battery Repairer	This user feels the product is better	JD.com
Puning Dental	Cell Phone Battery Repairer	I've only used it once, and I don't see any results, so I'll comment again after a few uses.	JD.com
Puning Dental	Cell Phone Battery Repairer	The software is unsupported, doesn't work, too lazy to return it.	JD.com
Puning Dental	Cell Phone Battery Repairer	This user feels the product is better	JD.com
Puning Dental	Cell Phone Battery Repairer	Scammed,**used to be useless, Dash scammer	JD.com
Puning Dental	Cell Phone Battery Repairer	This user feels the product is better	JD.com
Puning Dental	Cell Phone Battery Repairer	Use it before you comment.	JD.com
Puning Dental	Cell Phone Battery Repairer	This user feels the product is better	JD.com
Puning Dental	Cell Phone Battery Repairer	This user feels the product is better	JD.com
Puning Dental	Cell Phone Battery Repairer	IQ tax	JD.com
Puning Dental	Cell Phone Battery Repairer	Received 5 stars I'll be back for more!	JD.com
Puning Dental	Cell Phone Battery Repairer	Product performance: good price, economical and practical, very good function, fast logistics, good service attitude!	JD.com
Puning Dental	Cell Phone Battery Repairer	Received the cell phone adapter, used it once as requested, works well, following the store.	JD.com
Puning Dental	Cell Phone Battery Repairer	This baby is not good, it's not what you guys said, I want to return it	JD.com
Puning Dental	Cell Phone Battery Repairer	I'm using it. I don't know how it works.	JD.com
Puning Dental	Cell Phone Battery Repairer	Logistics is fast, packaging is good, is genuine, worth buying	JD.com
Puning Dental	Cell Phone Battery Repairer	No effect scam product	JD.com
Puning Dental	Cell Phone Battery Repairer	It was like this from the time it was plugged in until the end of the charge, and it's been in the process of being fixed. Can't see the effect.	JD.com
Puning Dental	Cell Phone Battery Repairer	You're ripping me off. I'm returning it.	JD.com
Puning Dental	Cell Phone Battery Repairer	This user feels the product is better	JD.com
Puning Dental	Cell Phone Battery Repairer	The cell phone battery has been used for a long time, the charge is not enough, use this restorer to restore the battery power. Use a period of time to evaluate.	JD.com
Puning Dental	Cell Phone Battery Repairer	Just received it today don't know how it works, use it for a few days to see the results.	JD.com

Puning Dental	Cell Phone Battery	This user feels the product is better	JD.com
Puning Dental	Cell Phone Battery	Fast charging and compact appearance	JD.com
Puning Dental	Cell Phone Battery	This user feels the product is better	JD.com
Puning Dental	Cell Phone Battery	Received it, tried it out, don't know how it works yet.	JD.com
Puning Dental	Cell Phone Battery	This one doesn't work, it came back to use as broken, and very originally it didn't work. Bragger.	JD.com
Puning Dental	Cell Phone Battery	Sowed up and burned out, requesting a new one.	JD.com
Puning Dental	Cell Phone Battery Repairer	Fast shipping and logistics. Packaging is very good. Received plugged in and used up, as if a little effect, charging is also a little faster, in use, later review it.	JD.com
Puning Dental	Cell Phone Battery	Fast delivery and great customer service, [rose	JD.com
Puning Dental	Cell Phone Battery	This user feels the product is better	JD.com
Puning Dental	Cell Phone Battery	It works.	JD.com
Puning Dental	Cell Phone Battery	This user feels the product is better	JD.com
Puning Dental	Cell Phone Battery	The goods have been received, the repair is very good, charging quickly, good use	JD.com
Puning Dental	Cell Phone Battery	This user feels the product is better	JD.com
Puning Dental	Cell Phone Battery	The effect is good business service attitude good logistics is very fast	JD.com
Puning Dental	Cell Phone Battery	This user feels the product is better	JD.com
Puning Dental	Cell Phone Battery	This product works well	JD.com
Puning Dental	Cell Phone Battery	After getting it back and using it, it works great and I recommend getting it!	JD.com
Puning Dental	Cell Phone Battery	The time it takes to charge my old phone has really been shortened.	JD.com
Puning Dental	Cell Phone Battery	[Lots of love] [Lots of love] [Lof love] [Lotsof love]	JD.com
Puning Dental	Cell Phone Battery	Speechless😒la! Doesn't work, both ends move, won't stand up straight, connection doesn't charge.	JD.com
Puning Dental	Cell Phone Battery	(of an unmarried couple) be close	JD.com
Puning Dental	Cell Phone Battery	This user feels the product is better	JD.com
Puning Dental	Cell Phone Battery	This user feels the product is better	JD.com
Puning Dental	Cell Phone Battery	Looks ok, haven't officially used it yet, hopefully it works	JD.com
Puning Dental	Cell Phone Battery	(of an unmarried couple) be close	JD.com
Puning Dental	Cell Phone Battery	Goods received and very pleased. Good value for money. Try to see the effect in the follow-up.	JD.com
Puning Dental	Cell Phone Battery	poor quality	JD.com
Puning Dental	Cell Phone Battery	This user feels the product is better	JD.com
Puning Dental	Cell Phone Battery Repairer	conscience of the dog ate it? There are also celebrities advertisements you are not afraid to go out and get into a car accident?	JD.com
Puning Dental	Cell Phone Battery	This user feels the product is better	JD.com
Puning Dental	Cell Phone Battery	Just got it now and am using it	JD.com
Puning Dental	Cell Phone Battery	The product has unique properties, kudos for supporting the awesome!	JD.com
Puning Dental	Cell Phone Battery	The stuff is okay. And I'm using it right now to use it to see if it's good or not before I use it.	JD.com
Puning Dental	Cell Phone Battery	This user feels the product is better	JD.com
Puning Dental	Cell Phone Battery	This user feels the product is better	JD.com
Puning Dental	Cell Phone Battery	Received the cell phone protector and it is now in use.	JD.com
Puning Dental	Cell Phone Battery	There's no restorative power at all! Doesn't work! Ask for a return for a refund!	JD.com
Puning Dental	Cell Phone Battery	Compact appearance	JD.com

Puning Dental	Cell Phone Battery	These things are all lies. They don't work.	JD.com
Puning Dental	Cell Phone Battery	light board inside and nothing? What does it do? Don't be fooled!	JD.com
Puning Dental	Cell Phone Battery	This user feels the product is better	JD.com
Puning Dental	Cell Phone Battery	This user feels the product is better	JD.com
Puning Dental	Cell Phone Battery	Logistics is also very fast, good service, now received the goods have not used it	JD.com
Puning Dental	Cell Phone Battery	The packaging is fine, the product is great, and I will be back next time!	JD.com
Puning Dental	Cell Phone Battery	It works It really works It's OK It's OK	JD.com
Puning Dental	Cell Phone Battery	Very good, charges quickly, nice design	JD.com
Puning Dental	Cell Phone Battery	This fixer is great does wonders for cell phones	JD.com
Puning Dental	Cell Phone Battery	Loose plug.	JD.com
Puning Dental	Cell Phone Battery	You guys said there's a switch. There's no switch.	JD.com
Puning Dental	Cell Phone Battery	I can't say whether it's good or not. I can't say how I feel.	JD.com
Puning Dental	Cell Phone Battery	Quite good, the packaging is very good(▽), like, I hope there are effects	JD.com
Puning Dental	Cell Phone Battery	Things received, small and exquisite so cute, I hope to help the phone battery	JD.com
Puning Dental	Cell Phone Battery	Product quality is good, beautiful and good-looking, is a very satisfied with a shopping, I wish the store business Xing Oh!	JD.com
Puning Dental	Cell Phone Battery	Received, the appearance of small, easy to carry	JD.com
Puning Dental	Cell Phone Battery	Repairs quickly, works wonders, brings the phone back to life, good stuff!	JD.com
Puning Dental	Cell Phone Battery	You won't know the quality until you try it!	JD.com
Puning Dental	Cell Phone Battery	very happy	JD.com
Puning Dental	Cell Phone Battery	This user feels the product is better	JD.com
Puning Dental	Cell Phone Battery	This user feels the product is better	JD.com
Puning Dental	Cell Phone Battery	Perfectly designed, easy to use and worth having.	JD.com
Puning Dental	Cell Phone Battery	This user feels the product is better	JD.com
Puning Dental	Cell Phone Battery	This user feels the product is better	JD.com
Puning Dental	Cell Phone Battery	No effect of use	JD.com
Puning Dental	Cell Phone Battery	This cell phone repairer is very good, inexpensive, worth enough to buy!	JD.com
Puning Dental	Cell Phone Battery	Received the goods Fine workmanship Looking forward to the results Hope to achieve satisfaction	JD.com
Puning Dental	Cell Phone Battery	Good stuff Inexpensive Will repurchase if used well	JD.com
Puning Dental	Cell Phone Battery	The cell phone adapter has been received, normal use in the case as described, to Hou good results!	JD.com
Puning Dental	Cell Phone Battery	Cheap price, fast logistics, good service attitude, is in use	JD.com
Puning Dental	Cell Phone Battery	This user feels the product is better	JD.com
Puning Dental	Cell Phone Battery	This user feels the product is better	JD.com
Puning Dental	Cell Phone Battery Repairer	Still don't know the countryside fruit, plug in the power shows repair. And plug no cell phone no difference, now do not give the star, because if it is not good to give five stars to deceive consumers, if you give one or two stars, if the goods are real goods, it is unfair to the business, sorry business, so after a while and then follow up the evaluation.	JD.com
Puning Dental	Cell Phone Battery	This user feels the product is better	JD.com
Puning Dental	Cell Phone Battery	the courier service attitude good next time to continue to buy[kudos]	JD.com
Puning Dental	Cell Phone Battery	Works great, very effective at cleaning up phone junk!	JD.com
Puning Dental	Cell Phone Battery	Goods have been received, tried the effect is good, logistics is very fast!	JD.com

Puning Dental	Cell Phone Battery	I got it. It's powered up.	JD.com
Puning Dental	Cell Phone Battery	The merchandise works great.	JD.com
Puning Dental	Cell Phone Battery	Good product, great for repairing batteries, will buy again next time!	JD.com
Puning Dental	Cell Phone Battery	This user feels the product is better	JD.com
Puning Dental	Cell Phone Battery Repairer	Goods I have received, just tried the effect is very good, is an ideal shopping. Logistics is very fast, the merchant is also good, give five-star praise.	JD.com
Puning Dental	Cell Phone Battery	This user feels the product is better	JD.com
Puning Dental	Cell Phone Battery	incompetent	JD.com
Puning Dental	Cell Phone Battery	serviceable	JD.com
Puning Dental	Cell Phone Battery	Goods received, received and charged, small and exquisite, applicable and convenient.	JD.com
Puning Dental	Cell Phone Battery	(of an unmarried couple) be close	JD.com
Puning Dental	Cell Phone Battery	This user feels the product is better	JD.com
Puning Dental	Cell Phone Battery	This user feels the product is better	JD.com
Puning Dental	Cell Phone Battery	The charging speed is still the same as before, and it's very convenient to connect the two sides directly.	JD.com
Puning Dental	Cell Phone Battery Repairer	Good and affordable Good and affordable Good and affordable Good and affordable Good and affordable Good andaffordable Good and affordable GoodandaffordableGoodaffordableaffordable	JD.com
Puning Dental	Cell Phone Battery	This user feels the product is better	JD.com
Puning Dental	Cell Phone Battery	Just received the cell phone adapter, item is unused, may be good but a little small.	JD.com
Puning Dental	Cell Phone Battery	Fast logistics, good value for money, very full! Will recommend to friends	JD.com
Puning Dental	Cell Phone Battery	Goods received, are using, cheap logistics very quickly!	JD.com
Puning Dental	Cell Phone Battery	Works great, great for cell phone junk cleaning!	JD.com
Puning Dental	Cell Phone Battery Repairer	ofmore than5hours, the battery consumption does not seem to fast, the phone performance also seems to be faster, because there is no instrument to measure, I can not say exactly how much betterThere is a little doubt, I follow the instructions of the instruction manual"shut down"and then charging, but a charge on the I followed the instructions to "turn off" the phone and then charge it, but as soon as I charged it, the phone turned on automatically. So I fixed it by charging it with the screen off (Huawei). Can you please tell me if this is harmful to the phone?	JD.com
Puning Dental	Cell Phone Battery Repairer	You're charging that uh, what's-being-restored plug? I bought one last time, this is the second one I don't think I've used this one yet, the last one was pretty good, I think this one is good too, it's good, it's good, it's pretty affordable.	JD.com
Puning Dental	Cell Phone Battery	This user feels the product is better	JD.com
Puning Dental	Cell Phone Battery	Good courier service and good use.	JD.com
Puning Dental	Cell Phone Battery	Goods have been received good delivery is very good good not a few days goods received very good	JD.com
Puning Dental	Cell Phone Battery	This user feels the product is better	JD.com
Puning Dental	Cell Phone Battery	Received the cell phone fixer. I'll follow up after using it.	JD.com
Puning Dental	Cell Phone Battery Repairer	Well, well.	JD.com
Puning Dental	Cell Phone Battery	It doesn't seem to be fitting in very well.	JD.com
Puning Dental	Cell Phone Battery	ineffective	JD.com
Puning Dental	Cell Phone Battery	Plugged in, it flickered for a second and never lit up again. It doesn't work.	JD.com
Puning Dental	Cell Phone Battery	Merchant service is thoughtful, fast delivery, logistics is also very strong, just received is trying, five-star praise.	JD.com
Puning Dental	Cell Phone Battery	This cell phone repairer works really well is great for cell phone battery maintenance	JD.com

Puning Dental	Cell Phone Battery Repairer	Repair device received, fast logistics, beautiful packaging. Goods inexpensive and beautiful, repair effect on the phone, this is my second purchase.	JD.com
Puning Dental	Cell Phone Battery Repairer	Goods received, very good, I am also very satisfied, to your store to say good reviews and then forward you out, there are more friends in the shoot!	JD.com
Puning Dental	Cell Phone Battery	[LOVE] [LOVE] [LOVELOVELOVE[LLOVE]LOVE]]	JD.com
Puning Dental	Cell Phone Battery	The stuff is great. Good to look at and use. Very good purchase.	JD.com
Puning Dental	Cell Phone Battery	The goods are very good, last time also bought, good use, courier Road goods in time, thank you!	JD.com
Puning Dental	Cell Phone Battery	It's not very effective. It doesn't work.	JD.com
Puning Dental	Cell Phone Battery	Baby has received, have to a few days may see the effect. Thank you boss! Thanks to the courier boy!	JD.com
Puning Dental	Cell Phone Battery	overcharged and saying it can fix the phone doesn't help at all	JD.com
Puning Dental	Cell Phone Battery	I haven't used it yet I'll comment after I try it	JD.com
Puning Dental	Cell Phone Battery	Merchant service is good, logistics is fast, the product does work to repair, see how long it can be later, then evaluate!	JD.com
Puning Dental	Cell Phone Battery	This user feels the product is better	JD.com
Puning Dental	Cell Phone Battery	very good	JD.com
Puning Dental	Cell Phone Battery	The goods are small and effective	JD.com
Puning Dental	Cell Phone Battery	This user feels the product is better	JD.com
Puning Dental	Cell Phone Battery	It's good. It's true.	JD.com
Puning Dental	Cell Phone Battery	The item has been received and the customer service is excellent. Use it.	JD.com
Puning Dental	Cell Phone Battery	Express has been received, the logistics is quite strong, with quite suitable, next time to continue to buy!	JD.com
Puning Dental	Cell Phone Battery	This one works exceptionally well? Fast delivery.	JD.com
Puning Dental	Cell Phone Battery	This user feels the product is better	JD.com
Puning Dental	Cell Phone Battery	This user feels the product is better	JD.com
Puning Dental	Cell Phone Battery	and low price, logistics is very fast, next time continue to buy.	JD.com
Puning Dental	Cell Phone Battery	This user feels the product is better	JD.com
Puning Dental	Cell Phone Battery Repairer	repairer is well packaged. Logistics is fast. The cell phone adapter is made with fine craftsmanship. The use of the party invasion. How its effect, used to know. Good value for money. Worth buying.', 1	JD.com
Puning Dental	Cell Phone Battery	Operation: ok[smile]Product performance: ok	JD.com
Puning Dental	Cell Phone Battery Repairer	Arrived quickly, well-packed, cost-effective, well adapter is very easy to use, but also can refuse to repair the battery, overall quite good, satisfied with the praise.	JD.com
Puning Dental	Cell Phone Battery	Average quality, function not yet known	JD.com
Puning Dental	Cell Phone Battery	、非常滿意快递也較快!	JD.com
Puning Dental	Cell Phone Battery Repairer	Cell phone adapter just take today to plug in the repair, look forward to the effect, the appearance of small, easy to carry, back just when the phone is out of power on the plug, good use will also buy back, with a few days of good to make and then give a follow-up comment!	JD.com
Puning Dental	Cell Phone Battery Repairer	Used ten minutes display light does not light up, customer service said can charge on the line, about 20minutes, automatically shut down, such a product who still buy?	JD.com
Puning Dental	Cell Phone Battery	Still trying it out, don't know how well it works, feels pretty cheap?	JD.com
Puning Dental	Cell Phone Battery	The merchant is very trustworthy, I like it very much	JD.com
Puning Dental	Cell Phone Battery	The goods have been received, I do not know whether it is good or not, first give you five-star reviews	JD.com
Puning Dental	Cell Phone Battery	This user feels the product is better	JD.com
Puning Dental	Cell Phone Battery	[FURIOUS]	JD.com

Puning Dental	Cell Phone Battery	It works.	JD.com
Puning Dental	Cell Phone Battery	The item works great and is just as described by the merchant.	JD.com
Puning Dental	Cell Phone Battery	Good quality product, worth buying.	JD.com
Puning Dental	Battery Repairer	Well, I can't say yet. Let's just use it and try it out! Uh-huh!	JD.com
Puning Dental	Cell Phone Battery	It looks good.	JD.com
Puning Dental	Cell Phone Battery	Repair has received, use good re-purchase wish the store business prosperity!	JD.com
Puning Dental	Cell Phone Battery	It works well.	JD.com
Puning Dental	Cell Phone Battery Repairer	Conversion efficiency: good<br>Compatibility: strong<br>Security: good	JD.com
Puning Dental	Cell Phone Battery	Cell phone adapter received looks good used well in buy a	JD.com
Puning Dental	Cell Phone Battery	Received the item and it looks great. The style is also beautiful and okay.	JD.com
Puning Dental	Cell Phone Battery	Friends introduced to buy, on the cell phone pool repair remarkable, worth buying.	JD.com
Puning Dental	Cell Phone Battery	pretty good	JD.com
Puning Dental	Cell Phone Battery Repairer	The goods are very delicate, logistics is also fast, the business service is enthusiastic<br>Give five-star praise	JD.com
Puning Dental	Cell Phone Battery Repairer	Great price for what you get. Will buy the good stuff again in the future. Probably would work better at a higher price. Recommended purchase has been sent to the Poundland coterie.	JD.com
Puning Dental	Cell Phone Battery	I am very happy with your merchandise and the product is great.	JD.com
Puning Dental	Cell Phone Battery	Useful to repair fast charging good quality	JD.com
Puning Dental	Cell Phone Battery	products also fraudulent consumer business or unlicensed operation!	JD.com
Puning Dental	Cell Phone Battery Repairer	Authentic battery activator, cell phone repair is very easy, product quality is quite over the top, very satisfied with the special like, the price is cheap things are good, fine workmanship style is good, the service is thoughtful shipping fast, full-star praise a hundred praise!	JD.com
Puning Dental	Cell Phone Battery Repairer	This store's repairer is good, the cell phone battery activation rate is high, authentic product stuff is good, exquisite and beautiful high quality, fast shipping and thoughtful service, praise a full star high!	JD.com
Puning Dental	Cell Phone Battery Repairer	Cell phone repairer has received, genuine activator effect, product over the quality of high, something special good spend less, very satisfied with the super like, thoughtful service delivery fast, full of stars!	JD.com
Puning Dental	Cell Phone Battery Repairer	smooth, fine workmanship kind of beautiful, product quality is quite over the top, the price is cheap things are good, the service is enthusiastic and thoughtful, fast delivery full of stars high light!	JD.com
Puning Dental	Cell Phone Battery Repairer	Cheap and affordable<br>Stability: good<br>Will repurchase again	JD.com
Puning Dental	Cell Phone Battery	Quality can be, the courier boy is very enthusiastic please you can rest assured to buy!	JD.com
Puning Dental	Cell Phone Battery	This item is great quality and fast shipping.	JD.com
Puning Dental	Cell Phone Battery	classifier for objects in rows such as words	JD.com
Puning Dental	Cell Phone Battery Repairer	Received a cell phone battery repairer, the quality is quite good, exquisite packaging, good seller service attitude, logistics and express timely, all five-star praise. Wish merchants prosperous business.	JD.com
Puning Dental	Cell Phone Battery	Well packaged, inexpensive and easy to use.	JD.com
Puning Dental	Cell Phone Battery	Charging is average, charging is so slow	JD.com
Puning Dental	Cell Phone Battery	Very good satisfied next time come back[buy buy buy][kudos][kudos]	JD.com
Puning Dental	Cell Phone Battery	This user feels the product is better	JD.com
Puning Dental	Cell Phone Battery	Review after use.	JD.com

Puning Dental	Cell Phone Battery Repairer	Battery Repairer received, quite good, good quality, carefully packaged, intact, good business attitude, logistics and express timely, all five-star praise.	JD.com
Puning Dental	Cell Phone Battery	Plus this is similar to slow charging, it's good for battery protection	JD.com
Puning Dental	Cell Phone Battery	Great price and fast shipping! Packaging was great!	JD.com
Puning Dental	Cell Phone Battery	Works great and fits perfectly. It also plugs in.	JD.com
Puning Dental	Cell Phone Battery	The quality is pretty good.	JD.com
Puning Dental	Cell Phone Battery	The stuff works great, I'll buy another one later	JD.com
Puning Dental	Cell Phone Battery	IQ tax	JD.com
Puning Dental	Cell Phone Battery	This user feels the product is better	JD.com
Puning Dental	Cell Phone Battery	[Smiles][Skimming][Big Love]	JD.com
Puning Dental	Cell Phone Battery Repairer	Baby is very good, the logistics is very fast, the quality of workmanship is very good, cheaper than the entity store, trustworthy, like friends hurry to get down to it , , , , , , , , , , , , , , ,	JD.com
Puning Dental	Cell Phone Battery	False advertising, false products	JD.com
Puning Dental	Cell Phone Battery	I've got more memory with this. It's not laggy.	JD.com
Puning Dental	Cell Phone Battery	! ! ! ! ! ! ! ! ! ! !	JD.com
Puning Dental	Cell Phone Battery	This user feels the product is better	JD.com
Puning Dental	Cell Phone Battery	This user feels the product is better	JD.com
Puning Dental	Cell Phone Battery	Use it right now and punch a laptop.	JD.com
Puning Dental	Cell Phone Battery	Good quality, very cheap, good object	JD.com
Puning Dental	Cell Phone Battery	Support the new technology, the goods have been received. The effect of the use of follow-up comments!	JD.com
Puning Dental	Cell Phone Battery Repairer	especially well, thanks to the merchant's stuff works especially well, this is especially good.[Smiles] This very satisfied. This is very, very good, this satisfaction to you to play, I good praise.	JD.com
Puning Dental	Cell Phone Battery	Thanks to the merchant's stuff works exceptionally well, really exceptionally well possesses no problems at all!	JD.com
Puning Dental	Cell Phone Battery	Please return the product if there is any problem with the quality	JD.com
Puning Dental	Cell Phone Battery	I'm using it now with good use over and over and over again.	JD.com
Puning Dental	Cell Phone Battery	Logistics is very powerful Quality is very good Immediately repurchase	JD.com
Puning Dental	Cell Phone Battery	This user feels the product is better	JD.com
Puning Dental	Cell Phone Battery Repairer	is very fast, the customer service attitude is very positive, and questions are answered in a timely manner! Overall very satisfied!	JD.com
Puning Dental	Cell Phone Battery	This user feels the product is better	JD.com
Puning Dental	Cell Phone Battery	inexpensive and good quality, fast delivery, customer service is very good too!	JD.com
Puning Dental	Cell Phone Battery	I'll wait to use it before I say how I feel about it.	JD.com
Puning Dental	Cell Phone Battery Repairer	beautifully packaged/product! It is a good adapter to improve the battery life of mobilephone/optimize cell phonesystem/clean up cell phone garbage and improverunning speed of cell phone!Trustworthy/purchaseuse! I wish the manufacturer a prosperous business! We wish you a prosperous business! Popularity is strong and prosperous![Kudos][Buy]][Good][Buy]][Clap][Hands[Ah Huang][Handshake][Tongue]Rose]	JD.com
Puning Dental	Cell Phone Battery Repairer	The item has been received, the courier is also very fast, the product quality is very good, the workmanship is very refined, is genuine, the price is also very cheap, and the description is exactly the same, I give five-star praise.	JD.com
Puning Dental	Cell Phone Battery	Comments	JD.com
Puning Dental	Cell Phone Battery	Well, I can't say yet. Let's just use it and try it out! Uh-huh!	JD.com

Puning Dental	Cell Phone Battery	It looks good.	JD.com
Puning Dental	Cell Phone Battery	Repair has received, use good re-purchase wish the store business prosperity!	JD.com
Puning Dental	Cell Phone Battery	It works well.	JD.com
Puning Dental	Cell Phone Battery Repairer	Conversion efficiency: good<br>Compatibility: strong<br>Security: good	JD.com
Puning Dental	Cell Phone Battery	Cell phone adapter received looks good used well in buy a|	JD.com
Puning Dental	Cell Phone Battery	Received the item and it looks great. The style is also beautiful and okay.	JD.com
Puning Dental	Cell Phone Battery	Friends introduced to buy, on the cell phone pool repair remarkable, worth buying.	JD.com
Puning Dental	Cell Phone Battery	pretty good	JD.com
Puning Dental	Cell Phone Battery Repairer	The goods are very delicate, logistics is also fast, the business service is enthusiastic<br>Give five-star praise	JD.com
Puning Dental	Cell Phone Battery Repairer	Great price for what you get. Will buy the good stuff again in the future. Probably would work better at a higher price. Recommended purchase has been sent to the Poundland coterie.	JD.com
Puning Dental	Cell Phone Battery	I am very happy with your merchandise and the product is great.	JD.com
Puning Dental	Cell Phone Battery	Useful to repair fast charging good quality	JD.com
Puning Dental	Cell Phone Battery	products also fraudulent consumer business or unlicensed operation!	JD.com
Puning Dental	Cell Phone Battery Repairer	Authentic battery activator, cell phone repair is very easy, product quality is quite over the top, very satisfied with the special like, the price is cheap things are good, fine workmanship style is good, the service is thoughtful shipping fast, full-star praise a hundred praise!	JD.com
Puning Dental	Cell Phone Battery Repairer	This store's repairer is good, the cell phone battery activation rate is high, authentic product stuff is good, exquisite and beautiful high quality, fast shipping and thoughtful service, praise a full star high!	JD.com
Puning Dental	Cell Phone Battery Repairer	Cell phone repairer has received, genuine activator effect, product over the quality of high, something special good spend less, very satisfied with the super like, thoughtful service delivery fast, full of stars!	JD.com
Puning Dental	Cell Phone Battery Repairer	smooth, fine workmanship kind of beautiful, product quality is quite over the top, the price is cheap things are good, the service is enthusiastic and thoughtful, fast delivery full of stars high light!	JD.com
Puning Dental	Cell Phone Battery Repairer	Cheap and affordable<br>Stability: good<br>Will repurchase again	JD.com
Puning Dental	Cell Phone Battery	Quality can be, the courier boy is very enthusiastic please you can rest assured to buy!	JD.com
Puning Dental	Cell Phone Battery	This item is great quality and fast shipping.	JD.com
Puning Dental	Cell Phone Battery	classifier for objects in rows such as words	JD.com
Puning Dental	Cell Phone Battery Repairer	Received a cell phone battery repairer, the quality is quite good, exquisite packaging, good seller service attitude, logistics and express timely, all five-star praise. Wish merchants prosperous business.	JD.com
Puning Dental	Cell Phone Battery	Well packaged, inexpensive and easy to use.	JD.com
Puning Dental	Cell Phone Battery	Charging is average, charging is so slow	JD.com
Puning Dental	Cell Phone Battery	Very good satisfied next time come back[buy buy buy][kudos][kudos]	JD.com
Puning Dental	Cell Phone Battery	This user feels the product is better	JD.com
Puning Dental	Cell Phone Battery	Review after use.	JD.com
Puning Dental	Cell Phone Battery Repairer	Battery Repairer received, quite good, good quality, carefully packaged, intact, good business attitude, logistics and express timely, all five-star praise.	JD.com
Puning Dental	Cell Phone Battery	Plus this is similar to slow charging, it's good for battery protection	JD.com
Puning Dental	Cell Phone Battery	Great price and fast shipping! Packaging was great!	JD.com
Puning Dental	Cell Phone Battery	Works great and fits perfectly. It also plugs in.	JD.com

Puning Dental	Cell Phone Battery	The quality is pretty good.	JD.com
Puning Dental	Cell Phone Battery	The stuff works great, I'll buy another one later	JD.com
Puning Dental	Cell Phone Battery	IQ tax	JD.com
Puning Dental	Cell Phone Battery	This user feels the product is better	JD.com
Puning Dental	Cell Phone Battery	[Smiles][Skimming][Big Love]	JD.com
Puning Dental	Cell Phone Battery Repairer	Baby is very good, the logistics is very fast, the quality of workmanship is very good, cheaper than the entity store, trustworthy, like friends hurry to get down to it , , , , , , , , , , , , , , ,	JD.com
Puning Dental	Cell Phone Battery	False advertising, false products	JD.com
Puning Dental	Cell Phone Battery	I've got more memory with this. It's not laggy.	JD.com
Puning Dental	Cell Phone Battery	！！！！！！！！！！！	JD.com
Puning Dental	Cell Phone Battery	This user feels the product is better	JD.com
Puning Dental	Cell Phone Battery	This user feels the product is better	JD.com
Puning Dental	Cell Phone Battery	Use it right now and punch a laptop.	JD.com
Puning Dental	Cell Phone Battery	Good quality, very cheap, good object	JD.com
Puning Dental	Cell Phone Battery	Support the new technology, the goods have been received. The effect of the use of follow-up comments!	JD.com
Puning Dental	Cell Phone Battery Repairer	especially well, thanks to the merchant's stuff works especially well, this is especially good.[Smiles]This very satisfied. This is very, very good, this satisfaction to you to play, I good praise.	JD.com
Puning Dental	Cell Phone Battery	Thanks to the merchant's stuff works exceptionally well, really exceptionally well possesses no problems at all!	JD.com
Puning Dental	Cell Phone Battery	Please return the product if there is any problem with the quality	JD.com
Puning Dental	Cell Phone Battery	I'm using it now with good use over and over and over again.	JD.com
Puning Dental	Cell Phone Battery	Logistics is very powerful Quality is very good Immediately repurchase	JD.com
Puning Dental	Cell Phone Battery	This user feels the product is better	JD.com
Puning Dental	Cell Phone Battery Repairer	is very fast, the customer service attitude is very positive, and questions are answered in a timely manner! Overall very satisfied!	JD.com
Puning Dental	Cell Phone Battery	This user feels the product is better	JD.com
Puning Dental	Cell Phone Battery	inexpensive and good quality, fast delivery, customer service is very good too!	JD.com
Puning Dental	Cell Phone Battery	I'll wait to use it before I say how I feel about it.	JD.com
Puning Dental	Cell Phone Battery Repairer	beautifully packaged/product! It is a good adapter to improve the battery life of mobilephone/optimize cell phonesystem/clean up cell phone garbage and improverunning speed of cell phone!Trustworthy/purchaseuse! I wish the manufacturer a prosperous business! We wish you a prosperous business! Popularity is strong and prosperous![Kudos][Buy]][Good][Buy]][Clap][Hands[Ah Huang][Handshake][Tongue]Rose]	JD.com
Puning Dental	Cell Phone Battery Repairer	The item has been received, the courier is also very fast, the product quality is very good, the workmanship is very refined, is genuine, the price is also very cheap, and the description is exactly the same, I give five-star praise.	JD.com
Puning Dental	Cell Phone Battery	Comments	JD.com
Puning Dental	Cell Phone Battery	Well, I can't say yet. Let's just use it and try it out! Uh-huh!	JD.com
Puning Dental	Cell Phone Battery	It looks good.	JD.com
Puning Dental	Cell Phone Battery	Repair has received, use good re-purchase wish the store business prosperity!	JD.com
Puning Dental	Cell Phone Battery	It works well.	JD.com
Puning Dental	Cell Phone Battery Repairer	Conversion efficiency: good<br>Compatibility: strong<br>Security: good	JD.com

Puning Dental	Cell Phone Battery	Cell phone adapter received looks good used well in buy a	JD.com
Puning Dental	Cell Phone Battery	Received the item and it looks great. The style is also beautiful and okay.	JD.com
Puning Dental	Cell Phone Battery	Friends introduced to buy, on the cell phone pool repair remarkable, worth buying.	JD.com
Puning Dental	Cell Phone Battery	pretty good	JD.com
Puning Dental	Cell Phone Battery Repairer	The goods are very delicate, logistics is also fast, the business service is enthusiastic<br>Give five-star praise	JD.com
Puning Dental	Cell Phone Battery Repairer	Great price for what you get. Will buy the good stuff again in the future. Probably would work better at a higher price. Recommended purchase has been sent to the Poundland coterie.	JD.com
Puning Dental	Cell Phone Battery	I am very happy with your merchandise and the product is great.	JD.com
Puning Dental	Cell Phone Battery	Useful to repair fast charging good quality	JD.com
Puning Dental	Cell Phone Battery	products also fraudulent consumer business or unlicensed operation!	JD.com
Puning Dental	Cell Phone Battery Repairer	Authentic battery activator, cell phone repair is very easy, product quality is quite over the top, very satisfied with the special like, the price is cheap things are good, fine workmanship style is good, the service is thoughtful shipping fast, full-star praise a hundred praise!	JD.com
Puning Dental	Cell Phone Battery Repairer	This store's repairer is good, the cell phone battery activation rate is high, authentic product stuff is good, exquisite and beautiful high quality, fast shipping and thoughtful service, praise a full star high!	JD.com
Puning Dental	Cell Phone Battery Repairer	Cell phone repairer has received, genuine activator effect, product over the quality of high, something special good spend less, very satisfied with the super like, thoughtful service delivery fast, full of stars!	JD.com
Puning Dental	Cell Phone Battery Repairer	smooth, fine workmanship kind of beautiful, product quality is quite over the top, the price is cheap things are good, the service is enthusiastic and thoughtful, fast delivery full of stars high light!	JD.com
Puning Dental	Cell Phone Battery Repairer	Cheap and affordable<br>Stability: good<br>Will repurchase again	JD.com
Puning Dental	Cell Phone Battery	Quality can be, the courier boy is very enthusiastic please you can rest assured to buy!	JD.com
Puning Dental	Cell Phone Battery	This item is great quality and fast shipping.	JD.com
Puning Dental	Cell Phone Battery	classifier for objects in rows such as words	JD.com
Puning Dental	Cell Phone Battery Repairer	Received a cell phone battery repairer, the quality is quite good, exquisite packaging, good seller service attitude, logistics and express timely, all five-star praise. Wish merchants prosperous business.	JD.com
Puning Dental	Cell Phone Battery	Well packaged, inexpensive and easy to use.	JD.com
Puning Dental	Cell Phone Battery	Charging is average, charging is so slow	JD.com
Puning Dental	Cell Phone Battery	Very good satisfied next time come back[buy buy buy][kudos][kudos]	JD.com
Puning Dental	Cell Phone Battery	This user feels the product is better	JD.com
Puning Dental	Cell Phone Battery	Review after use.	JD.com
Puning Dental	Cell Phone Battery Repairer	Battery Repairer received, quite good, good quality, carefully packaged, intact, good business attitude, logistics and express timely, all five-star praise.	JD.com
Puning Dental	Cell Phone Battery	Plus this is similar to slow charging, it's good for battery protection	JD.com
Puning Dental	Cell Phone Battery	Great price and fast shipping! Packaging was great!	JD.com
Puning Dental	Cell Phone Battery	Works great and fits perfectly. It also plugs in.	JD.com
Puning Dental	Cell Phone Battery	The quality is pretty good.	JD.com
Puning Dental	Cell Phone Battery	The stuff works great, I'll buy another one later	JD.com
Puning Dental	Cell Phone Battery	IQ tax	JD.com
Puning Dental	Cell Phone Battery	This user feels the product is better	JD.com
Puning Dental	Cell Phone Battery	[Smiles][Skimming][Big Love]	JD.com

Puning Dental	Cell Phone Battery Repairer	Baby is very good, the logistics is very fast, the quality of workmanship is very good, cheaper than the entity store, trustworthy, like friends hurry to get down to it , , , , , , , , , , , , , , ,	JD.com
Puning Dental	Cell Phone Battery	False advertising, false products	JD.com
Puning Dental	Cell Phone Battery	I've got more memory with this. It's not laggy.	JD.com
Puning Dental	Cell Phone Battery	! ! ! ! ! ! ! ! ! ! !	JD.com
Puning Dental	Cell Phone Battery	This user feels the product is better	JD.com
Puning Dental	Cell Phone Battery	This user feels the product is better	JD.com
Puning Dental	Cell Phone Battery	Use it right now and punch a laptop.	JD.com
Puning Dental	Cell Phone Battery	Good quality, very cheap, good object	JD.com
Puning Dental	Cell Phone Battery	Support the new technology, the goods have been received. The effect of the use of follow-up comments!	JD.com
Puning Dental	Cell Phone Battery Repairer	especially well, thanks to the merchant's stuff works especially well, this is especially good.[Smiles]This very satisfied. This is very, very good, this satisfaction to you to play, I good praise.	JD.com
Puning Dental	Cell Phone Battery	Thanks to the merchant's stuff works exceptionally well, really exceptionally well possesses no problems at all!	JD.com
Puning Dental	Cell Phone Battery	Please return the product if there is any problem with the quality	JD.com
Puning Dental	Cell Phone Battery	I'm using it now with good use over and over and over again.	JD.com
Puning Dental	Cell Phone Battery	Logistics is very powerful Quality is very good Immediately repurchase	JD.com
Puning Dental	Cell Phone Battery	This user feels the product is better	JD.com
Puning Dental	Cell Phone Battery Repairer	is very fast, the customer service attitude is very positive, and questions are answered in a timely manner! Overall very satisfied!	JD.com
Puning Dental	Cell Phone Battery	This user feels the product is better	JD.com
Puning Dental	Cell Phone Battery	inexpensive and good quality, fast delivery, customer service is very good too!	JD.com
Puning Dental	Cell Phone Battery	I'll wait to use it before I say how I feel about it.	JD.com
Puning Dental	Cell Phone Battery Repairer	beautifully packaged/product! It is a good adapter to improve the battery life of mobilephone/optimize cell phonesystem/clean up cell phone garbage and improverunning speed of cell phone!Trustworthy/purchaseuse! I wish the manufacturer a prosperous business! We wish you a prosperous business! Popularity is strong and prosperous![Kudos][Buy]][Good][Buy]][Clap][Hands[Ah Huang][Handshake][Tongue]Rose]	JD.com
Puning Dental	Cell Phone Battery Repairer	The item has been received, the courier is also very fast, the product quality is very good, the workmanship is very refined, is genuine, the price is also very cheap, and the description is exactly the same, I give five-star praise.	JD.com
Puning Dental	Cell Phone Battery	Comments	JD.com
Puning Dental	Cell Phone Battery	Well, I can't say yet. Let's just use it and try it out! Uh-huh!	JD.com
Puning Dental	Cell Phone Battery	It looks good.	JD.com
Puning Dental	Cell Phone Battery	Repair has received, use good re-purchase wish the store business prosperity!	JD.com
Puning Dental	Cell Phone Battery	It works well.	JD.com
Puning Dental	Cell Phone Battery Repairer	Conversion efficiency: good<br>Compatibility: strong<br>Security: good	JD.com
Puning Dental	Cell Phone Battery	Cell phone adapter received looks good used well in buy a |	JD.com
Puning Dental	Cell Phone Battery	Received the item and it looks great. The style is also beautiful and okay.	JD.com
Puning Dental	Cell Phone Battery	Friends introduced to buy, on the cell phone pool repair remarkable, worth buying.	JD.com
Puning Dental	Cell Phone Battery	pretty good	JD.com

Puning Dental	Cell Phone Battery Repairer	The goods are very delicate, logistics is also fast, the business service is enthusiastic<br>Give five-star praise	JD.com
Puning Dental	Cell Phone Battery Repairer	Great price for what you get. Will buy the good stuff again in the future. Probably would work better at a higher price. Recommended purchase has been sent to the Poundland coterie.	JD.com
Puning Dental	Cell Phone Battery	I am very happy with your merchandise and the product is great.	JD.com
Puning Dental	Cell Phone Battery	Useful to repair fast charging good quality	JD.com
Puning Dental	Cell Phone Battery	products also fraudulent consumer business or unlicensed operation!	JD.com
Puning Dental	Cell Phone Battery Repairer	Authentic battery activator, cell phone repair is very easy, product quality is quite over the top, very satisfied with the special like, the price is cheap things are good, fine workmanship style is good, the service is thoughtful shipping fast, full-star praise a hundred praise!	JD.com
Puning Dental	Cell Phone Battery Repairer	This store's repairer is good, the cell phone battery activation rate is high, authentic product stuff is good, exquisite and beautiful high quality, fast shipping and thoughtful service, praise a full star high!	JD.com
Puning Dental	Cell Phone Battery Repairer	Cell phone repairer has received, genuine activator effect, product over the quality of high, something special good spend less, very satisfied with the super like, thoughtful service delivery fast, full of stars!	JD.com
Puning Dental	Cell Phone Battery Repairer	smooth, fine workmanship kind of beautiful, product quality is quite over the top, the price is cheap things are good, the service is enthusiastic and thoughtful, fast delivery full of stars high light!	JD.com
Puning Dental	Cell Phone Battery Repairer	Cheap and affordable<br>Stability: good<br>Will repurchase again	JD.com
Puning Dental	Cell Phone Battery	Quality can be, the courier boy is very enthusiastic please you can rest assured to buy!	JD.com
Puning Dental	Cell Phone Battery	This item is great quality and fast shipping.	JD.com
Puning Dental	Cell Phone Battery	classifier for objects in rows such as words	JD.com
Puning Dental	Cell Phone Battery Repairer	Received a cell phone battery repairer, the quality is quite good, exquisite packaging, good seller service attitude, logistics and express timely, all five-star praise. Wish merchants prosperous business.	JD.com
Puning Dental	Cell Phone Battery	Well packaged, inexpensive and easy to use.	JD.com
Puning Dental	Cell Phone Battery	Charging is average, charging is so slow	JD.com
Puning Dental	Cell Phone Battery	Very good satisfied next time come back[buy buy buy][kudos][kudos]	JD.com
Puning Dental	Cell Phone Battery	This user feels the product is better	JD.com
Puning Dental	Cell Phone Battery	Review after use.	JD.com
Puning Dental	Cell Phone Battery Repairer	Battery Repairer received, quite good, good quality, carefully packaged, intact, good business attitude, logistics and express timely, all five-star praise.	JD.com
Puning Dental	Cell Phone Battery	Plus this is similar to slow charging, it's good for battery protection	JD.com
Puning Dental	Cell Phone Battery	Great price and fast shipping! Packaging was great!	JD.com
Puning Dental	Cell Phone Battery	Works great and fits perfectly. It also plugs in.	JD.com
Puning Dental	Cell Phone Battery	The quality is pretty good.	JD.com
Puning Dental	Cell Phone Battery	The stuff works great, I'll buy another one later	JD.com
Puning Dental	Cell Phone Battery	IQ tax	JD.com
Puning Dental	Cell Phone Battery	This user feels the product is better	JD.com
Puning Dental	Cell Phone Battery	[Smiles][Skimming][Big Love]	JD.com
Puning Dental	Cell Phone Battery Repairer	Baby is very good, the logistics is very fast, the quality of workmanship is very good, cheaper than the entity store, trustworthy, like friends hurry to get down to it<br>, , , , , , , , , , , , , , ,	JD.com
Puning Dental	Cell Phone Battery	False advertising, false products	JD.com
Puning Dental	Cell Phone Battery	I've got more memory with this. It's not laggy.	JD.com

Puning Dental	Cell Phone Battery	！！！！！！！！！！！	JD.com
Puning Dental	Cell Phone Battery	This user feels the product is better	JD.com
Puning Dental	Cell Phone Battery	This user feels the product is better	JD.com
Puning Dental	Cell Phone Battery	Use it right now and punch a laptop.	JD.com
Puning Dental	Cell Phone Battery	Good quality, very cheap, good object	JD.com
Puning Dental	Cell Phone Battery	Support the new technology, the goods have been received. The effect of the use of follow-up comments!	JD.com
Puning Dental	Cell Phone Battery Repairer	especially well, thanks to the merchant's stuff works especially well, this is especially good.[Smiles] This very satisfied. This is very, very good, this satisfaction to you to play, I good praise.	JD.com
Puning Dental	Cell Phone Battery	Thanks to the merchant's stuff works exceptionally well, really exceptionally well possesses no problems at all!	JD.com
Puning Dental	Cell Phone Battery	Please return the product if there is any problem with the quality	JD.com
Puning Dental	Cell Phone Battery	I'm using it now with good use over and over and over again.	JD.com
Puning Dental	Cell Phone Battery	Logistics is very powerful Quality is very good Immediately repurchase	JD.com
Puning Dental	Cell Phone Battery	This user feels the product is better	JD.com
Puning Dental	Cell Phone Battery Repairer	is very fast, the customer service attitude is very positive, and questions are answered in a timely manner! Overall very satisfied!	JD.com
Puning Dental	Cell Phone Battery	This user feels the product is better	JD.com
Puning Dental	Cell Phone Battery	inexpensive and good quality, fast delivery, customer service is very good too!	JD.com
Puning Dental	Cell Phone Battery	I'll wait to use it before I say how I feel about it.	JD.com
Puning Dental	Cell Phone Battery Repairer	beautifully packaged/product! It is a good adapter to improve the battery life of mobilephone/optimize cell phonesystem/clean up cell phone garbage and improverunning speed of cell phone!Trustworthy/purchaseuse! I wish the manufacturer a prosperous business! We wish you a prosperous business! Popularity is strong and prosperous![Kudos][Buy]][Good][Buy]][Clap][Hands[Ah Huang][Handshake][Tongue]Rose]	JD.com
Puning Dental	Cell Phone Battery Repairer	The item has been received, the courier is also very fast, the product quality is very good, the workmanship is very refined, is genuine, the price is also very cheap, and the description is exactly the same, I give five-star praise.	JD.com
Puning Dental	Cell Phone Battery	Comments	JD.com
Puning Dental	Cell Phone Battery	Well, I can't say yet. Let's just use it and try it out! Uh-huh!	JD.com
Puning Dental	Cell Phone Battery	It looks good.	JD.com
Puning Dental	Cell Phone Battery	Repair has received, use good re-purchase wish the store business prosperity!	JD.com
Puning Dental	Cell Phone Battery	It works well.	JD.com
Puning Dental	Cell Phone Battery Repairer	Conversion efficiency: good Compatibility: strong Security: good	JD.com
Puning Dental	Cell Phone Battery	Cell phone adapter received looks good used well in buy a|	JD.com
Puning Dental	Cell Phone Battery	Received the item and it looks great. The style is also beautiful and okay.	JD.com
Puning Dental	Cell Phone Battery	Friends introduced to buy, on the cell phone pool repair remarkable, worth buying.	JD.com
Puning Dental	Cell Phone Battery	pretty good	JD.com
Puning Dental	Cell Phone Battery Repairer	The goods are very delicate, logistics is also fast, the business service is enthusiastic Give five-star praise	JD.com
Puning Dental	Cell Phone Battery Repairer	Great price for what you get. Will buy the good stuff again in the future. Probably would work better at a higher price. Recommended purchase has been sent to the Poundland coterie.	JD.com
Puning Dental	Cell Phone Battery	I am very happy with your merchandise and the product is great.	JD.com
Puning Dental	Cell Phone Battery	Useful to repair fast charging good quality	JD.com

Puning Dental	Cell Phone Battery	products also fraudulent consumer business or unlicensed operation!	JD.com
Puning Dental	Cell Phone Battery Repairer	Authentic battery activator, cell phone repair is very easy, product quality is quite over the top, very satisfied with the special like, the price is cheap things are good, fine workmanship style is good, the service is thoughtful shipping fast, full-star praise a hundred praise!	JD.com
Puning Dental	Cell Phone Battery Repairer	This store's repairer is good, the cell phone battery activation rate is high, authentic product stuff is good, exquisite and beautiful high quality, fast shipping and thoughtful service, praise a full star high!	JD.com
Puning Dental	Cell Phone Battery Repairer	Cell phone repairer has received, genuine activator effect, product over the quality of high, something special good spend less, very satisfied with the super like, thoughtful service delivery fast, full of stars!	JD.com
Puning Dental	Cell Phone Battery Repairer	smooth, fine workmanship kind of beautiful, product quality is quite over the top, the price is cheap things are good, the service is enthusiastic and thoughtful, fast delivery full of stars high light!	JD.com
Puning Dental	Cell Phone Battery Repairer	Cheap and affordable<br>Stability: good<br>Will repurchase again	JD.com
Puning Dental	Cell Phone Battery	Quality can be, the courier boy is very enthusiastic please you can rest assured to buy!	JD.com
Puning Dental	Cell Phone Battery	This item is great quality and fast shipping.	JD.com
Puning Dental	Cell Phone Battery	classifier for objects in rows such as words	JD.com
Puning Dental	Cell Phone Battery Repairer	Received a cell phone battery repairer, the quality is quite good, exquisite packaging, good seller service attitude, logistics and express timely, all five-star praise. Wish merchants prosperous business.	JD.com
Puning Dental	Cell Phone Battery	Well packaged, inexpensive and easy to use.	JD.com
Puning Dental	Cell Phone Battery	Charging is average, charging is so slow	JD.com
Puning Dental	Cell Phone Battery	Very good satisfied next time come back[buy buy buy][kudos][kudos]	JD.com
Puning Dental	Cell Phone Battery	This user feels the product is better	JD.com
Puning Dental	Cell Phone Battery	Review after use.	JD.com
Puning Dental	Cell Phone Battery Repairer	Battery Repairer received, quite good, good quality, carefully packaged, intact, good business attitude, logistics and express timely, all five-star praise.	JD.com
Puning Dental	Cell Phone Battery	Plus this is similar to slow charging, it's good for battery protection	JD.com
Puning Dental	Cell Phone Battery	Great price and fast shipping! Packaging was great!	JD.com
Puning Dental	Cell Phone Battery	Works great and fits perfectly. It also plugs in.	JD.com
Puning Dental	Cell Phone Battery	The quality is pretty good.	JD.com
Puning Dental	Cell Phone Battery	The stuff works great, I'll buy another one later	JD.com
Puning Dental	Cell Phone Battery	IQ tax	JD.com
Puning Dental	Cell Phone Battery	This user feels the product is better	JD.com
Puning Dental	Cell Phone Battery	[Smiles][Skimming][Big Love]	JD.com
Puning Dental	Cell Phone Battery Repairer	Baby is very good, the logistics is very fast, the quality of workmanship is very good, cheaper than the entity store, trustworthy, like friends hurry to get down to it ,,,,,,,,,,,,,,,	JD.com
Puning Dental	Cell Phone Battery	False advertising, false products	JD.com
Puning Dental	Cell Phone Battery	I've got more memory with this. It's not laggy.	JD.com
Puning Dental	Cell Phone Battery	!!!!!!!!!!!	JD.com
Puning Dental	Cell Phone Battery	This user feels the product is better	JD.com
Puning Dental	Cell Phone Battery	This user feels the product is better	JD.com
Puning Dental	Cell Phone Battery	Use it right now and punch a laptop.	JD.com
Puning Dental	Cell Phone Battery	Good quality, very cheap, good object	JD.com

Puning Dental	Cell Phone Battery Repairer	Support the new technology, the goods have been received. The effect of the use of follow-up comments!	JD.com
Puning Dental	Cell Phone Battery Repairer	especially well, thanks to the merchant's stuff works especially well, this is especially good.[Smiles]This very satisfied. This is very, very good, this satisfaction to you to play, I good praise.	JD.com
Puning Dental	Cell Phone Battery Repairer	Thanks to the merchant's stuff works exceptionally well, really exceptionally well possesses no problems at all!	JD.com
Puning Dental	Cell Phone Battery Repairer	Please return the product if there is any problem with the quality	JD.com
Puning Dental	Cell Phone Battery Repairer	I'm using it now with good use over and over and over again.	JD.com
Puning Dental	Cell Phone Battery Repairer	Logistics is very powerful Quality is very good Immediately repurchase	JD.com
Puning Dental	Cell Phone Battery Repairer	This user feels the product is better	JD.com
Puning Dental	Cell Phone Battery Repairer	is very fast, the customer service attitude is very positive, and questions are answered in a timely manner! Overall very satisfied!	JD.com
Puning Dental	Cell Phone Battery Repairer	This user feels the product is better	JD.com
Puning Dental	Cell Phone Battery Repairer	inexpensive and good quality, fast delivery, customer service is very good too!	JD.com
Puning Dental	Cell Phone Battery Repairer	I'll wait to use it before I say how I feel about it.	JD.com
Puning Dental	Cell Phone Battery Repairer	beautifully packaged/product! It is a good adapter to improve the battery life of mobilephone/optimize cell phonesystem/clean up cell phone garbage and improverunning speed of cell phone!Trustworthy/purchaseuse! I wish the manufacturer a prosperous business! We wish you a prosperous business! Popularity is strong and prosperous![Kudos][Buy]][Good][Buy]][Clap][Hands[Ah Huang][Handshake][Tongue]Rose]	JD.com
Puning Dental	Cell Phone Battery Repairer	The item has been received, the courier is also very fast, the product quality is very good, the workmanship is very refined, is genuine, the price is also very cheap, and the description is exactly the same, I give five-star praise.	JD.com
Puning Dental	Cell Phone Battery Repairer	Comments	JD.com
Puning Dental	Cell Phone Battery Repairer	Well, I can't say yet. Let's just use it and try it out! Uh-huh!	JD.com
Puning Dental	Cell Phone Battery Repairer	It looks good.	JD.com
Puning Dental	Cell Phone Battery Repairer	Repair has received, use good re-purchase wish the store business prosperity!	JD.com
Puning Dental	Cell Phone Battery Repairer	It works well.	JD.com
Puning Dental	Cell Phone Battery Repairer	Conversion efficiency: good<br>Compatibility: strong<br>Security: good	JD.com
Puning Dental	Cell Phone Battery Repairer	Cell phone adapter received looks good used well in buy a|	JD.com
Puning Dental	Cell Phone Battery Repairer	Received the item and it looks great. The style is also beautiful and okay.	JD.com
Puning Dental	Cell Phone Battery Repairer	Friends introduced to buy, on the cell phone pool repair remarkable, worth buying.	JD.com
Puning Dental	Cell Phone Battery Repairer	pretty good	JD.com
Puning Dental	Cell Phone Battery Repairer	The goods are very delicate, logistics is also fast, the business service is enthusiastic<br>Give five-star praise	JD.com
Puning Dental	Cell Phone Battery Repairer	Great price for what you get. Will buy the good stuff again in the future. Probably would work better at a higher price. Recommended purchase has been sent to the Poundland coterie.	JD.com
Puning Dental	Cell Phone Battery Repairer	I am very happy with your merchandise and the product is great.	JD.com
Puning Dental	Cell Phone Battery Repairer	Useful to repair fast charging good quality	JD.com
Puning Dental	Cell Phone Battery Repairer	products also fraudulent consumer business or unlicensed operation!	JD.com
Puning Dental	Cell Phone Battery Repairer	Authentic battery activator, cell phone repair is very easy, product quality is quite over the top, very satisfied with the special like, the price is cheap things are good, fine workmanship style is good, the service is thoughtful shipping fast, full-star praise a hundred praise!	JD.com

Puning Dental	Cell Phone Battery Repairer	This store's repairer is good, the cell phone battery activation rate is high, authentic product stuff is good, exquisite and beautiful high quality, fast shipping and thoughtful service, praise a full star high!	JD.com
Puning Dental	Cell Phone Battery Repairer	Cell phone repairer has received, genuine activator effect, product over the quality of high, something special good spend less, very satisfied with the super like, thoughtful service delivery fast, full of stars!	JD.com
Puning Dental	Cell Phone Battery Repairer	smooth, fine workmanship kind of beautiful, product quality is quite over the top, the price is cheap things are good, the service is enthusiastic and thoughtful, fast delivery full of stars high light!	JD.com
Puning Dental	Cell Phone Battery Repairer	Cheap and affordable<br>Stability: good<br>Will repurchase again	JD.com
Puning Dental	Cell Phone Battery Repairer	Quality can be, the courier boy is very enthusiastic please you can rest assured to buy!	JD.com
Puning Dental	Cell Phone Battery Repairer	This item is great quality and fast shipping.	JD.com
Puning Dental	Cell Phone Battery Repairer	classifier for objects in rows such as words	JD.com
Puning Dental	Cell Phone Battery Repairer	Received a cell phone battery repairer, the quality is quite good, exquisite packaging, good seller service attitude, logistics and express timely, all five-star praise. Wish merchants prosperous business.	JD.com
Puning Dental	Cell Phone Battery Repairer	Well packaged, inexpensive and easy to use.	JD.com
Puning Dental	Cell Phone Battery Repairer	Charging is average, charging is so slow	JD.com
Puning Dental	Cell Phone Battery Repairer	Very good satisfied next time come back[buy buy buy][kudos][kudos]	JD.com
Puning Dental	Cell Phone Battery Repairer	This user feels the product is better	JD.com
Puning Dental	Cell Phone Battery Repairer	Review after use.	JD.com
Puning Dental	Cell Phone Battery Repairer	Battery Repairer received, quite good, good quality, carefully packaged, intact, good business attitude, logistics and express timely, all five-star praise.	JD.com
Puning Dental	Cell Phone Battery Repairer	Plus this is similar to slow charging, it's good for battery protection	JD.com
Puning Dental	Cell Phone Battery Repairer	Great price and fast shipping! Packaging was great!	JD.com
Puning Dental	Cell Phone Battery Repairer	Works great and fits perfectly. It also plugs in.	JD.com
Puning Dental	Cell Phone Battery Repairer	The quality is pretty good.	JD.com
Puning Dental	Cell Phone Battery Repairer	The stuff works great, I'll buy another one later	JD.com
Puning Dental	Cell Phone Battery Repairer	IQ tax	JD.com
Puning Dental	Cell Phone Battery Repairer	This user feels the product is better	JD.com
Puning Dental	Cell Phone Battery Repairer	[Smiles][Skimming][Big Love]	JD.com
Puning Dental	Cell Phone Battery Repairer	Baby is very good, the logistics is very fast, the quality of workmanship is very good, cheaper than the entity store, trustworthy, like friends hurry to get down to it , , , , , , , , , , , , , , ,	JD.com
Puning Dental	Cell Phone Battery Repairer	False advertising, false products	JD.com
Puning Dental	Cell Phone Battery Repairer	I've got more memory with this. It's not laggy.	JD.com
Puning Dental	Cell Phone Battery Repairer	! ! ! ! ! ! ! ! ! ! !	JD.com
Puning Dental	Cell Phone Battery Repairer	This user feels the product is better	JD.com
Puning Dental	Cell Phone Battery Repairer	This user feels the product is better	JD.com
Puning Dental	Cell Phone Battery Repairer	Use it right now and punch a laptop.	JD.com
Puning Dental	Cell Phone Battery Repairer	Good quality, very cheap, good object	JD.com
Puning Dental	Cell Phone Battery Repairer	Support the new technology, the goods have been received. The effect of the use of follow-up comments!	JD.com
Puning Dental	Cell Phone Battery Repairer	especially well, thanks to the merchant's stuff works especially well, this is especially good.[Smiles]This very satisfied. This is very, very good, this satisfaction to you to play, I good praise.	JD.com

Puning Dental	Cell Phone Battery	Thanks to the merchant's stuff works exceptionally well, really exceptionally well possesses no problems at all!	JD.com
Puning Dental	Cell Phone Battery	Please return the product if there is any problem with the quality	JD.com
Puning Dental	Cell Phone Battery	I'm using it now with good use over and over and over again.	JD.com
Puning Dental	Cell Phone Battery	Logistics is very powerful Quality is very good Immediately repurchase	JD.com
Puning Dental	Cell Phone Battery	This user feels the product is better	JD.com
Puning Dental	Cell Phone Battery Repairer	is very fast, the customer service attitude is very positive, and questions are answered in a timely manner! Overall very satisfied!	JD.com
Puning Dental	Cell Phone Battery	This user feels the product is better	JD.com
Puning Dental	Cell Phone Battery	inexpensive and good quality, fast delivery, customer service is very good too!	JD.com
Puning Dental	Cell Phone Battery	I'll wait to use it before I say how I feel about it.	JD.com
Puning Dental	Cell Phone Battery Repairer	beautifully packaged/product! It is a good adapter to improve the battery life of mobilephone/optimize cell phonesystem/clean up cell phone garbage and improverunning speed of cell phone!Trustworthy/purchaseuse! I wish the manufacturer a prosperous business! We wish you a prosperous business! Popularity is strong and prosperous![Kudos][Buy]][Good][Buy]][Clap][Hands[Ah Huang][Handshake][Tongue]Rose]	JD.com
Puning Dental	Cell Phone Battery Repairer	The item has been received, the courier is also very fast, the product quality is very good, the workmanship is very refined, is genuine, the price is also very cheap, and the description is exactly the same, I give five-star praise.	JD.com
Puning Dental	Cell Phone Battery	Comments	JD.com
Puning Dental	Cell Phone Battery	Well, I can't say yet. Let's just use it and try it out! Uh-huh!	JD.com
Puning Dental	Cell Phone Battery	It looks good.	JD.com
Puning Dental	Cell Phone Battery	Repair has received, use good re-purchase wish the store business prosperity!	JD.com
Puning Dental	Cell Phone Battery	It works well.	JD.com
Puning Dental	Cell Phone Battery Repairer	Conversion efficiency: good<br>Compatibility: strong<br>Security: good	JD.com
Puning Dental	Cell Phone Battery	Cell phone adapter received looks good used well in buy a	JD.com
Puning Dental	Cell Phone Battery	Received the item and it looks great. The style is also beautiful and okay.	JD.com
Puning Dental	Cell Phone Battery	Friends introduced to buy, on the cell phone pool repair remarkable, worth buying.	JD.com
Puning Dental	Cell Phone Battery	pretty good	JD.com
Puning Dental	Cell Phone Battery Repairer	The goods are very delicate, logistics is also fast, the business service is enthusiastic<br>Give five-star praise	JD.com
Puning Dental	Cell Phone Battery Repairer	Great price for what you get. Will buy the good stuff again in the future. Probably would work better at a higher price. Recommended purchase has been sent to the Poundland coterie.	JD.com
Puning Dental	Cell Phone Battery	I am very happy with your merchandise and the product is great.	JD.com
Puning Dental	Cell Phone Battery	Useful to repair fast charging good quality	JD.com
Puning Dental	Cell Phone Battery	products also fraudulent consumer business or unlicensed operation!	JD.com
Puning Dental	Cell Phone Battery Repairer	Authentic battery activator, cell phone repair is very easy, product quality is quite over the top, very satisfied with the special like, the price is cheap things are good, fine workmanship style is good, the service is thoughtful shipping fast, full-star praise a hundred praise!	JD.com
Puning Dental	Cell Phone Battery Repairer	This store's repairer is good, the cell phone battery activation rate is high, authentic product stuff is good, exquisite and beautiful high quality, fast shipping and thoughtful service, praise a full star high!	JD.com
Puning Dental	Cell Phone Battery Repairer	Cell phone repairer has received, genuine activator effect, product over the quality of high, something special good spend less, very satisfied with the super like, thoughtful service delivery fast, full of stars!	JD.com

Puning Dental	Cell Phone Battery Repairer	smooth, fine workmanship kind of beautiful, product quality is quite over the top, the price is cheap things are good, the service is enthusiastic and thoughtful, fast delivery full of stars high light!	JD.com
Puning Dental	Cell Phone Battery Repairer	Cheap and affordable<br>Stability: good<br>Will repurchase again	JD.com
Puning Dental	Cell Phone Battery	Quality can be, the courier boy is very enthusiastic please you can rest assured to buy!	JD.com
Puning Dental	Cell Phone Battery	This item is great quality and fast shipping.	JD.com
Puning Dental	Cell Phone Battery	classifier for objects in rows such as words	JD.com
Puning Dental	Cell Phone Battery Repairer	Received a cell phone battery repairer, the quality is quite good, exquisite packaging, good seller service attitude, logistics and express timely, all five-star praise. Wish merchants prosperous business.	JD.com
Puning Dental	Cell Phone Battery	Well packaged, inexpensive and easy to use.	JD.com
Puning Dental	Cell Phone Battery	Charging is average, charging is so slow	JD.com
Puning Dental	Cell Phone Battery	Very good satisfied next time come back[buy buy buy][kudos][kudos]	JD.com
Puning Dental	Cell Phone Battery	This user feels the product is better	JD.com
Puning Dental	Cell Phone Battery	Review after use.	JD.com
Puning Dental	Cell Phone Battery Repairer	Battery Repairer received, quite good, good quality, carefully packaged, intact, good business attitude, logistics and express timely, all five-star praise.	JD.com
Puning Dental	Cell Phone Battery	Plus this is similar to slow charging, it's good for battery protection	JD.com
Puning Dental	Cell Phone Battery	Great price and fast shipping! Packaging was great!	JD.com
Puning Dental	Cell Phone Battery	Works great and fits perfectly. It also plugs in.	JD.com
Puning Dental	Cell Phone Battery	The quality is pretty good.	JD.com
Puning Dental	Cell Phone Battery	The stuff works great, I'll buy another one later	JD.com
Puning Dental	Cell Phone Battery	IQ tax	JD.com
Puning Dental	Cell Phone Battery	This user feels the product is better	JD.com
Puning Dental	Cell Phone Battery	[Smiles][Skimming][Big Love]	JD.com
Puning Dental	Cell Phone Battery Repairer	Baby is very good, the logistics is very fast, the quality of workmanship is very good, cheaper than the entity store, trustworthy, like friends hurry to get down to it , , , , , , , , , , , , , , ,	JD.com
Puning Dental	Cell Phone Battery	False advertising, false products	JD.com
Puning Dental	Cell Phone Battery	I've got more memory with this. It's not laggy.	JD.com
Puning Dental	Cell Phone Battery	! ! ! ! ! ! ! ! ! ! !	JD.com
Puning Dental	Cell Phone Battery	This user feels the product is better	JD.com
Puning Dental	Cell Phone Battery	This user feels the product is better	JD.com
Puning Dental	Cell Phone Battery	Use it right now and punch a laptop.	JD.com
Puning Dental	Cell Phone Battery	Good quality, very cheap, good object	JD.com
Puning Dental	Cell Phone Battery	Support the new technology, the goods have been received. The effect of the use of follow-up comments!	JD.com
Puning Dental	Cell Phone Battery Repairer	especially well, thanks to the merchant's stuff works especially well, this is especially good.[Smiles]This very satisfied. This is very, very good, this satisfaction to you to play, I good praise.	JD.com
Puning Dental	Cell Phone Battery	Thanks to the merchant's stuff works exceptionally well, really exceptionally well possesses no problems at all!	JD.com
Puning Dental	Cell Phone Battery	Please return the product if there is any problem with the quality	JD.com
Puning Dental	Cell Phone Battery	I'm using it now with good use over and over and over again.	JD.com
Puning Dental	Cell Phone Battery	Logistics is very powerful Quality is very good Immediately repurchase	JD.com

Puning Dental	Cell Phone Battery Repairer	This user feels the product is better	JD.com
Puning Dental	Cell Phone Battery Repairer	is very fast, the customer service attitude is very positive, and questions are answered in a timely manner! Overall very satisfied!	JD.com
Puning Dental	Cell Phone Battery Repairer	This user feels the product is better	JD.com
Puning Dental	Cell Phone Battery Repairer	inexpensive and good quality, fast delivery, customer service is very good too!	JD.com
Puning Dental	Cell Phone Battery Repairer	I'll wait to use it before I say how I feel about it.	JD.com
Puning Dental	Cell Phone Battery Repairer	beautifully packaged/product! It is a good adapter to improve the battery life of mobilephone/optimize cell phonesystem/clean up cell phone garbage and improverunning speed of cell phone!Trustworthy/purchaseuse! I wish the manufacturer a prosperous business! We wish you a prosperous business! Popularity is strong and prosperous![Kudos][Buy]][Good][Buy]][Clap][Hands[Ah Huang][Handshake][Tongue]Rose]	JD.com
Puning Dental	Cell Phone Battery Repairer	The item has been received, the courier is also very fast, the product quality is very good, the workmanship is very refined, is genuine, the price is also very cheap, and the description is exactly the same, I give five-star praise.	JD.com
Puning Dental	Cell Phone Battery Repairer	Comments	JD.com
Puning Dental	Cell Phone Battery Repairer	Well, I can't say yet. Let's just use it and try it out! Uh-huh!	JD.com
Puning Dental	Cell Phone Battery Repairer	It looks good.	JD.com
Puning Dental	Cell Phone Battery Repairer	Repair has received, use good re-purchase wish the store business prosperity!	JD.com
Puning Dental	Cell Phone Battery Repairer	It works well.	JD.com
Puning Dental	Cell Phone Battery Repairer	Conversion efficiency: good<br>Compatibility: strong<br>Security: good	JD.com
Puning Dental	Cell Phone Battery Repairer	Cell phone adapter received looks good used well in buy a	JD.com
Puning Dental	Cell Phone Battery Repairer	Received the item and it looks great. The style is also beautiful and okay.	JD.com
Puning Dental	Cell Phone Battery Repairer	Friends introduced to buy, on the cell phone pool repair remarkable, worth buying.	JD.com
Puning Dental	Cell Phone Battery Repairer	pretty good	JD.com
Puning Dental	Cell Phone Battery Repairer	The goods are very delicate, logistics is also fast, the business service is enthusiastic<br>Give five-star praise	JD.com
Puning Dental	Cell Phone Battery Repairer	Great price for what you get. Will buy the good stuff again in the future. Probably would work better at a higher price. Recommended purchase has been sent to the Poundland coterie.	JD.com
Puning Dental	Cell Phone Battery Repairer	I am very happy with your merchandise and the product is great.	JD.com
Puning Dental	Cell Phone Battery Repairer	Useful to repair fast charging good quality	JD.com
Puning Dental	Cell Phone Battery Repairer	products also fraudulent consumer business or unlicensed operation!	JD.com
Puning Dental	Cell Phone Battery Repairer	Authentic battery activator, cell phone repair is very easy, product quality is quite over the top, very satisfied with the special like, the price is cheap things are good, fine workmanship style is good, the service is thoughtful shipping fast, full-star praise a hundred praise!	JD.com
Puning Dental	Cell Phone Battery Repairer	This store's repairer is good, the cell phone battery activation rate is high, authentic product stuff is good, exquisite and beautiful high quality, fast shipping and thoughtful service, praise a full star high!	JD.com
Puning Dental	Cell Phone Battery Repairer	Cell phone repairer has received, genuine activator effect, product over the quality of high, something special good spend less, very satisfied with the super like, thoughtful service delivery fast, full of stars!	JD.com
Puning Dental	Cell Phone Battery Repairer	smooth, fine workmanship kind of beautiful, product quality is quite over the top, the price is cheap things are good, the service is enthusiastic and thoughtful, fast delivery full of stars high light!	JD.com
Puning Dental	Cell Phone Battery Repairer	Cheap and affordable<br>Stability: good<br>Will repurchase again	JD.com
Puning Dental	Cell Phone Battery Repairer	Quality can be, the courier boy is very enthusiastic please you can rest assured to buy!	JD.com

Puning Dental	Cell Phone Battery	This item is great quality and fast shipping.	JD.com
Puning Dental	Cell Phone Battery	classifier for objects in rows such as words	JD.com
Puning Dental	Cell Phone Battery Repairer	Received a cell phone battery repairer, the quality is quite good, exquisite packaging, good seller service attitude, logistics and express timely, all five-star praise. Wish merchants prosperous business.	JD.com
Puning Dental	Cell Phone Battery	Well packaged, inexpensive and easy to use.	JD.com
Puning Dental	Cell Phone Battery	Charging is average, charging is so slow	JD.com
Puning Dental	Cell Phone Battery	Very good satisfied next time come back[buy buy buy][kudos][kudos]	JD.com
Puning Dental	Cell Phone Battery	This user feels the product is better	JD.com
Puning Dental	Cell Phone Battery	Review after use.	JD.com
Puning Dental	Cell Phone Battery Repairer	Battery Repairer received, quite good, good quality, carefully packaged, intact, good business attitude, logistics and express timely, all five-star praise.	JD.com
Puning Dental	Cell Phone Battery	Plus this is similar to slow charging, it's good for battery protection	JD.com
Puning Dental	Cell Phone Battery	Great price and fast shipping! Packaging was great!	JD.com
Puning Dental	Cell Phone Battery	Works great and fits perfectly. It also plugs in.	JD.com
Puning Dental	Cell Phone Battery	The quality is pretty good.	JD.com
Puning Dental	Cell Phone Battery	The stuff works great, I'll buy another one later	JD.com
Puning Dental	Cell Phone Battery	IQ tax	JD.com
Puning Dental	Cell Phone Battery	This user feels the product is better	JD.com
Puning Dental	Cell Phone Battery	[Smiles][Skimming][Big Love]	JD.com
Puning Dental	Cell Phone Battery Repairer	Baby is very good, the logistics is very fast, the quality of workmanship is very good, cheaper than the entity store, trustworthy, like friends hurry to get down to it , , , , , , , , , , , , , , ,	JD.com
Puning Dental	Cell Phone Battery	False advertising, false products	JD.com
Puning Dental	Cell Phone Battery	I've got more memory with this. It's not laggy.	JD.com
Puning Dental	Cell Phone Battery	! ! ! ! ! ! ! ! ! ! !	JD.com
Puning Dental	Cell Phone Battery	This user feels the product is better	JD.com
Puning Dental	Cell Phone Battery	This user feels the product is better	JD.com
Puning Dental	Cell Phone Battery	Use it right now and punch a laptop.	JD.com
Puning Dental	Cell Phone Battery	Good quality, very cheap, good object	JD.com
Puning Dental	Cell Phone Battery	Support the new technology, the goods have been received. The effect of the use of follow-up comments!	JD.com
Puning Dental	Cell Phone Battery Repairer	especially well, thanks to the merchant's stuff works especially well, this is especially good.[Smiles]This very satisfied. This is very, very good, this satisfaction to you to play, I good praise.	JD.com
Puning Dental	Cell Phone Battery	Thanks to the merchant's stuff works exceptionally well, really exceptionally well possesses no problems at all!	JD.com
Puning Dental	Cell Phone Battery	Please return the product if there is any problem with the quality	JD.com
Puning Dental	Cell Phone Battery	I'm using it now with good use over and over and over again.	JD.com
Puning Dental	Cell Phone Battery	Logistics is very powerful Quality is very good Immediately repurchase	JD.com
Puning Dental	Cell Phone Battery	This user feels the product is better	JD.com
Puning Dental	Cell Phone Battery Repairer	is very fast, the customer service attitude is very positive, and questions are answered in a timely manner! Overall very satisfied!	JD.com
Puning Dental	Cell Phone Battery	This user feels the product is better	JD.com
Puning Dental	Cell Phone Battery	inexpensive and good quality, fast delivery, customer service is very good too!	JD.com

Puning Dental	Cell Phone Battery	I'll wait to use it before I say how I feel about it.	JD.com
Puning Dental	Cell Phone Battery Repairer	beautifully packaged/product! It is a good adapter to improve the battery life of mobilephone/optimize cell phonesystem/clean up cell phone garbage and improverunning speed of cell phone!Trustworthy/purchaseuse! I wish the manufacturer a prosperous business! We wish you a prosperous business! Popularity is strong and prosperous![Kudos][Buy]][Good][Buy]][Clap][Hands[Ah Huang][Handshake][Tongue]Rose]	JD.com
Puning Dental	Cell Phone Battery Repairer	The item has been received, the courier is also very fast, the product quality is very good, the workmanship is very refined, is genuine, the price is also very cheap, and the description is exactly the same, I give five-star praise.	JD.com
Puning Dental	Cell Phone Battery	Comments	JD.com
Puning Dental	Cell Phone Battery	Well, I can't say yet. Let's just use it and try it out! Uh-huh!	JD.com
Puning Dental	Cell Phone Battery	It looks good.	JD.com
Puning Dental	Cell Phone Battery	Repair has received, use good re-purchase wish the store business prosperity!	JD.com
Puning Dental	Cell Phone Battery	It works well.	JD.com
Puning Dental	Cell Phone Battery Repairer	Conversion efficiency: good<br>Compatibility: strong<br>Security: good	JD.com
Puning Dental	Cell Phone Battery	Cell phone adapter received looks good used well in buy a|	JD.com
Puning Dental	Cell Phone Battery	Received the item and it looks great. The style is also beautiful and okay.	JD.com
Puning Dental	Cell Phone Battery	Friends introduced to buy, on the cell phone pool repair remarkable, worth buying.	JD.com
Puning Dental	Cell Phone Battery	pretty good	JD.com
Puning Dental	Cell Phone Battery Repairer	The goods are very delicate, logistics is also fast, the business service is enthusiastic<br>Give five-star praise	JD.com
Puning Dental	Cell Phone Battery Repairer	Great price for what you get. Will buy the good stuff again in the future. Probably would work better at a higher price. Recommended purchase has been sent to the Poundland coterie.	JD.com
Puning Dental	Cell Phone Battery	I am very happy with your merchandise and the product is great.	JD.com
Puning Dental	Cell Phone Battery	Useful to repair fast charging good quality	JD.com
Puning Dental	Cell Phone Battery	products also fraudulent consumer business or unlicensed operation!	JD.com
Puning Dental	Cell Phone Battery Repairer	Authentic battery activator, cell phone repair is very easy, product quality is quite over the top, very satisfied with the special like, the price is cheap things are good, fine workmanship style is good, the service is thoughtful shipping fast, full-star praise a hundred praise!	JD.com
Puning Dental	Cell Phone Battery Repairer	This store's repairer is good, the cell phone battery activation rate is high, authentic product stuff is good, exquisite and beautiful high quality, fast shipping and thoughtful service, praise a full star high!	JD.com
Puning Dental	Cell Phone Battery Repairer	Cell phone repairer has received, genuine activator effect, product over the quality of high, something special good spend less, very satisfied with the super like, thoughtful service delivery fast, full of stars!	JD.com
Puning Dental	Cell Phone Battery Repairer	smooth, fine workmanship kind of beautiful, product quality is quite over the top, the price is cheap things are good, the service is enthusiastic and thoughtful, fast delivery full of stars high light!	JD.com
Puning Dental	Cell Phone Battery Repairer	Cheap and affordable<br>Stability: good<br>Will repurchase again	JD.com
Puning Dental	Cell Phone Battery	Quality can be, the courier boy is very enthusiastic please you can rest assured to buy!	JD.com
Puning Dental	Cell Phone Battery	This item is great quality and fast shipping.	JD.com
Puning Dental	Cell Phone Battery	classifier for objects in rows such as words	JD.com
Puning Dental	Cell Phone Battery Repairer	Received a cell phone battery repairer, the quality is quite good, exquisite packaging, good seller service attitude, logistics and express timely, all five-star praise. Wish merchants prosperous business.	JD.com

Puning Dental	Cell Phone Battery	Well packaged, inexpensive and easy to use.	JD.com
Puning Dental	Cell Phone Battery	Charging is average, charging is so slow	JD.com
Puning Dental	Cell Phone Battery	Very good satisfied next time come back[buy buy buy][kudos][kudos]	JD.com
Puning Dental	Cell Phone Battery	This user feels the product is better	JD.com
Puning Dental	Cell Phone Battery	Review after use.	JD.com
Puning Dental	Cell Phone Battery Repairer	Battery Repairer received, quite good, good quality, carefully packaged, intact, good business attitude, logistics and express timely, all five-star praise.	JD.com
Puning Dental	Cell Phone Battery	Plus this is similar to slow charging, it's good for battery protection	JD.com
Puning Dental	Cell Phone Battery	Great price and fast shipping! Packaging was great!	JD.com
Puning Dental	Cell Phone Battery	Works great and fits perfectly. It also plugs in.	JD.com
Puning Dental	Cell Phone Battery	The quality is pretty good.	JD.com
Puning Dental	Cell Phone Battery	The stuff works great, I'll buy another one later	JD.com
Puning Dental	Cell Phone Battery	IQ tax	JD.com
Puning Dental	Cell Phone Battery	This user feels the product is better	JD.com
Puning Dental	Cell Phone Battery	[Smiles][Skimming][Big Love]	JD.com
Puning Dental	Cell Phone Battery Repairer	Baby is very good, the logistics is very fast, the quality of workmanship is very good, cheaper than the entity store, trustworthy, like friends hurry to get down to it , , , , , , , , , , , , , , ,	JD.com
Puning Dental	Cell Phone Battery	False advertising, false products	JD.com
Puning Dental	Cell Phone Battery	I've got more memory with this. It's not laggy.	JD.com
Puning Dental	Cell Phone Battery	! ! ! ! ! ! ! ! ! ! !	JD.com
Puning Dental	Cell Phone Battery	This user feels the product is better	JD.com
Puning Dental	Cell Phone Battery	This user feels the product is better	JD.com
Puning Dental	Cell Phone Battery	Use it right now and punch a laptop.	JD.com
Puning Dental	Cell Phone Battery	Good quality, very cheap, good object	JD.com
Puning Dental	Cell Phone Battery	Support the new technology, the goods have been received. The effect of the use of follow-up comments!	JD.com
Puning Dental	Cell Phone Battery Repairer	especially well, thanks to the merchant's stuff works especially well, this is especially good.[Smiles]This very satisfied. This is very, very good, this satisfaction to you to play, I good praise.	JD.com
Puning Dental	Cell Phone Battery	Thanks to the merchant's stuff works exceptionally well, really exceptionally well possesses no problems at all!	JD.com
Puning Dental	Cell Phone Battery	Please return the product if there is any problem with the quality	JD.com
Puning Dental	Cell Phone Battery	I'm using it now with good use over and over and over again.	JD.com
Puning Dental	Cell Phone Battery	Logistics is very powerful Quality is very good Immediately repurchase	JD.com
Puning Dental	Cell Phone Battery	This user feels the product is better	JD.com
Puning Dental	Cell Phone Battery Repairer	is very fast, the customer service attitude is very positive, and questions are answered in a timely manner! Overall very satisfied!	JD.com
Puning Dental	Cell Phone Battery	This user feels the product is better	JD.com
Puning Dental	Cell Phone Battery	inexpensive and good quality, fast delivery, customer service is very good too!	JD.com
Puning Dental	Cell Phone Battery	I'll wait to use it before I say how I feel about it.	JD.com

Puning Dental	Cell Phone Battery Repairer	beautifully packaged/product! It is a good adapter to improve the battery life of mobilephone/optimize cell phonesystem/clean up cell phone garbage and improverunning speed of cell phone!Trustworthy/purchaseuse! I wish the manufacturer a prosperous business! We wish you a prosperous business! Popularity is strong and prosperous![Kudos][Buy]][Good][Buy]][Clap][Hands[Ah Huang][Handshake][Tongue]Rose]	JD.com
Puning Dental	Cell Phone Battery Repairer	The item has been received, the courier is also very fast, the product quality is very good, the workmanship is very refined, is genuine, the price is also very cheap, and the description is exactly the same, I give five-star praise.	JD.com
Puning Dental	Cell Phone Battery	Comments	JD.com
Puning Dental	Cell Phone Battery	Well, I can't say yet. Let's just use it and try it out! Uh-huh!	JD.com
Puning Dental	Cell Phone Battery	It looks good.	JD.com
Puning Dental	Cell Phone Battery	Repair has received, use good re-purchase wish the store business prosperity!	JD.com
Puning Dental	Cell Phone Battery	It works well.	JD.com
Puning Dental	Cell Phone Battery Repairer	Conversion efficiency: good<br>Compatibility: strong<br>Security: good	JD.com
Puning Dental	Cell Phone Battery	Cell phone adapter received looks good used well in buy a		JD.com
Puning Dental	Cell Phone Battery	Received the item and it looks great. The style is also beautiful and okay.	JD.com
Puning Dental	Cell Phone Battery	Friends introduced to buy, on the cell phone pool repair remarkable, worth buying.	JD.com
Puning Dental	Cell Phone Battery	pretty good	JD.com
Puning Dental	Cell Phone Battery Repairer	The goods are very delicate, logistics is also fast, the business service is enthusiastic<br>Give five-star praise	JD.com
Puning Dental	Cell Phone Battery Repairer	Great price for what you get. Will buy the good stuff again in the future. Probably would work better at a higher price. Recommended purchase has been sent to the Poundland coterie.	JD.com
Puning Dental	Cell Phone Battery	I am very happy with your merchandise and the product is great.	JD.com
Puning Dental	Cell Phone Battery	Useful to repair fast charging good quality	JD.com
Puning Dental	Cell Phone Battery	products also fraudulent consumer business or unlicensed operation!	JD.com
Puning Dental	Cell Phone Battery Repairer	Authentic battery activator, cell phone repair is very easy, product quality is quite over the top, very satisfied with the special like, the price is cheap things are good, fine workmanship style is good, the service is thoughtful shipping fast, full-star praise a hundred praise!	JD.com
Puning Dental	Cell Phone Battery Repairer	This store's repairer is good, the cell phone battery activation rate is high, authentic product stuff is good, exquisite and beautiful high quality, fast shipping and thoughtful service, praise a full star high!	JD.com
Puning Dental	Cell Phone Battery Repairer	Cell phone repairer has received, genuine activator effect, product over the quality of high, something special good spend less, very satisfied with the super like, thoughtful service delivery fast, full of stars!	JD.com
Puning Dental	Cell Phone Battery Repairer	smooth, fine workmanship kind of beautiful, product quality is quite over the top, the price is cheap things are good, the service is enthusiastic and thoughtful, fast delivery full of stars high light!	JD.com
Puning Dental	Cell Phone Battery Repairer	Cheap and affordable<br>Stability: good<br>Will repurchase again	JD.com
Puning Dental	Cell Phone Battery	Quality can be, the courier boy is very enthusiastic please you can rest assured to buy!	JD.com
Puning Dental	Cell Phone Battery	This item is great quality and fast shipping.	JD.com
Puning Dental	Cell Phone Battery	classifier for objects in rows such as words	JD.com
Puning Dental	Cell Phone Battery Repairer	Received a cell phone battery repairer, the quality is quite good, exquisite packaging, good seller service attitude, logistics and express timely, all five-star praise. Wish merchants prosperous business.	JD.com
Puning Dental	Cell Phone Battery	Well packaged, inexpensive and easy to use.	JD.com

Puning Dental	Cell Phone Battery Repairer	Charging is average, charging is so slow	JD.com
Puning Dental	Cell Phone Battery Repairer	Very good satisfied next time come back[buy buy buy][kudos][kudos]	JD.com
Puning Dental	Cell Phone Battery Repairer	This user feels the product is better	JD.com
Puning Dental	Cell Phone Battery Repairer	Review after use.	JD.com
Puning Dental	Cell Phone Battery Repairer	Battery Repairer received, quite good, good quality, carefully packaged, intact, good business attitude, logistics and express timely, all five-star praise.	JD.com
Puning Dental	Cell Phone Battery Repairer	Plus this is similar to slow charging, it's good for battery protection	JD.com
Puning Dental	Cell Phone Battery Repairer	Great price and fast shipping! Packaging was great!	JD.com
Puning Dental	Cell Phone Battery Repairer	Works great and fits perfectly. It also plugs in.	JD.com
Puning Dental	Cell Phone Battery Repairer	The quality is pretty good.	JD.com
Puning Dental	Cell Phone Battery Repairer	The stuff works great, I'll buy another one later	JD.com
Puning Dental	Cell Phone Battery Repairer	IQ tax	JD.com
Puning Dental	Cell Phone Battery Repairer	This user feels the product is better	JD.com
Puning Dental	Cell Phone Battery Repairer	[Smiles][Skimming][Big Love]	JD.com
Puning Dental	Cell Phone Battery Repairer	Baby is very good, the logistics is very fast, the quality of workmanship is very good, cheaper than the entity store, trustworthy, like friends hurry to get down to it , , , , , , , , , , , , , , ,	JD.com
Puning Dental	Cell Phone Battery Repairer	False advertising, false products	JD.com
Puning Dental	Cell Phone Battery Repairer	I've got more memory with this. It's not laggy.	JD.com
Puning Dental	Cell Phone Battery Repairer	! ! ! ! ! ! ! ! ! ! !	JD.com
Puning Dental	Cell Phone Battery Repairer	This user feels the product is better	JD.com
Puning Dental	Cell Phone Battery Repairer	This user feels the product is better	JD.com
Puning Dental	Cell Phone Battery Repairer	Use it right now and punch a laptop.	JD.com
Puning Dental	Cell Phone Battery Repairer	Good quality, very cheap, good object	JD.com
Puning Dental	Cell Phone Battery Repairer	Support the new technology, the goods have been received. The effect of the use of follow-up comments!	JD.com
Puning Dental	Cell Phone Battery Repairer	especially well, thanks to the merchant's stuff works especially well, this is especially good.[Smiles]This very satisfied. This is very, very good, this satisfaction to you to play, I good praise.	JD.com
Puning Dental	Cell Phone Battery Repairer	Thanks to the merchant's stuff works exceptionally well, really exceptionally well possesses no problems at all!	JD.com
Puning Dental	Cell Phone Battery Repairer	Please return the product if there is any problem with the quality	JD.com
Puning Dental	Cell Phone Battery Repairer	I'm using it now with good use over and over and over again.	JD.com
Puning Dental	Cell Phone Battery Repairer	Logistics is very powerful Quality is very good Immediately repurchase	JD.com
Puning Dental	Cell Phone Battery Repairer	This user feels the product is better	JD.com
Puning Dental	Cell Phone Battery Repairer	is very fast, the customer service attitude is very positive, and questions are answered in a timely manner! Overall very satisfied!	JD.com
Puning Dental	Cell Phone Battery Repairer	This user feels the product is better	JD.com
Puning Dental	Cell Phone Battery Repairer	inexpensive and good quality, fast delivery, customer service is very good too!	JD.com
Puning Dental	Cell Phone Battery Repairer	I'll wait to use it before I say how I feel about it.	JD.com
Puning Dental	Cell Phone Battery Repairer	beautifully packaged/product! It is a good adapter to improve the battery life of mobilephone/optimize cell phonesystem/clean up cell phone garbage and improverunning speed of cell phone!Trustworthy/purchaseuse! I wish the manufacturer a prosperous business! We wish you a prosperous business! Popularity is strong and prosperous![Kudos][Buy]][Good][Buy]][Clap][Hands[Ah Huang][Handshake][Tongue]Rose]	JD.com

Puning Dental	Cell Phone Battery Repairer	The item has been received, the courier is also very fast, the product quality is very good, the workmanship is very refined, is genuine, the price is also very cheap, and the description is exactly the same, I give five-star praise.	JD.com
Puning Dental	Cell Phone Battery	Comments	JD.com
Puning Dental	Cell Phone Battery	Well, I can't say yet. Let's just use it and try it out! Uh-huh!	JD.com
Puning Dental	Cell Phone Battery	It looks good.	JD.com
Puning Dental	Cell Phone Battery	Repair has received, use good re-purchase wish the store business prosperity!	JD.com
Puning Dental	Cell Phone Battery	It works well.	JD.com
Puning Dental	Cell Phone Battery Repairer	Conversion efficiency: good<br>Compatibility: strong<br>Security: good	JD.com
Puning Dental	Cell Phone Battery	Cell phone adapter received looks good used well in buy a	JD.com
Puning Dental	Cell Phone Battery	Received the item and it looks great. The style is also beautiful and okay.	JD.com
Puning Dental	Cell Phone Battery	Friends introduced to buy, on the cell phone pool repair remarkable, worth buying.	JD.com
Puning Dental	Cell Phone Battery	pretty good	JD.com
Puning Dental	Cell Phone Battery Repairer	The goods are very delicate, logistics is also fast, the business service is enthusiastic<br>Give five-star praise	JD.com
Puning Dental	Cell Phone Battery Repairer	Great price for what you get. Will buy the good stuff again in the future. Probably would work better at a higher price. Recommended purchase has been sent to the Poundland coterie.	JD.com
Puning Dental	Cell Phone Battery	I am very happy with your merchandise and the product is great.	JD.com
Puning Dental	Cell Phone Battery	Useful to repair fast charging good quality	JD.com
Puning Dental	Cell Phone Battery	products also fraudulent consumer business or unlicensed operation!	JD.com
Puning Dental	Cell Phone Battery Repairer	Authentic battery activator, cell phone repair is very easy, product quality is quite over the top, very satisfied with the special like, the price is cheap things are good, fine workmanship style is good, the service is thoughtful shipping fast, full-star praise a hundred praise!	JD.com
Puning Dental	Cell Phone Battery Repairer	This store's repairer is good, the cell phone battery activation rate is high, authentic product stuff is good, exquisite and beautiful high quality, fast shipping and thoughtful service, praise a full star high!	JD.com
Puning Dental	Cell Phone Battery Repairer	Cell phone repairer has received, genuine activator effect, product over the quality of high, something special good spend less, very satisfied with the super like, thoughtful service delivery fast, full of stars!	JD.com
Puning Dental	Cell Phone Battery Repairer	smooth, fine workmanship kind of beautiful, product quality is quite over the top, the price is cheap things are good, the service is enthusiastic and thoughtful, fast delivery full of stars high light!	JD.com
Puning Dental	Cell Phone Battery Repairer	Cheap and affordable<br>Stability: good<br>Will repurchase again	JD.com
Puning Dental	Cell Phone Battery	Quality can be, the courier boy is very enthusiastic please you can rest assured to buy!	JD.com
Puning Dental	Cell Phone Battery	This item is great quality and fast shipping.	JD.com
Puning Dental	Cell Phone Battery	classifier for objects in rows such as words	JD.com
Puning Dental	Cell Phone Battery Repairer	Received a cell phone battery repairer, the quality is quite good, exquisite packaging, good seller service attitude, logistics and express timely, all five-star praise. Wish merchants prosperous business.	JD.com
Puning Dental	Cell Phone Battery	Well packaged, inexpensive and easy to use.	JD.com
Puning Dental	Cell Phone Battery	Charging is average, charging is so slow	JD.com
Puning Dental	Cell Phone Battery	Very good satisfied next time come back[buy buy buy][kudos][kudos]	JD.com
Puning Dental	Cell Phone Battery	This user feels the product is better	JD.com
Puning Dental	Cell Phone Battery	Review after use.	JD.com

Puning Dental	Cell Phone Battery Repairer	Battery Repairer received, quite good, good quality, carefully packaged, intact, good business attitude, logistics and express timely, all five-star praise.	JD.com
Puning Dental	Cell Phone Battery	Plus this is similar to slow charging, it's good for battery protection	JD.com
Puning Dental	Cell Phone Battery	Great price and fast shipping! Packaging was great!	JD.com
Puning Dental	Cell Phone Battery	Works great and fits perfectly. It also plugs in.	JD.com
Puning Dental	Cell Phone Battery	The quality is pretty good.	JD.com
Puning Dental	Cell Phone Battery	The stuff works great, I'll buy another one later	JD.com
Puning Dental	Cell Phone Battery	IQ tax	JD.com
Puning Dental	Cell Phone Battery	This user feels the product is better	JD.com
Puning Dental	Cell Phone Battery	[Smiles][Skimming][Big Love]	JD.com
Puning Dental	Cell Phone Battery Repairer	Baby is very good, the logistics is very fast, the quality of workmanship is very good, cheaper than the entity store, trustworthy, like friends hurry to get down to it , , , , , , , , , , , , , , ,	JD.com
Puning Dental	Cell Phone Battery	False advertising, false products	JD.com
Puning Dental	Cell Phone Battery	I've got more memory with this. It's not laggy.	JD.com
Puning Dental	Cell Phone Battery	! ! ! ! ! ! ! ! ! ! !	JD.com
Puning Dental	Cell Phone Battery	This user feels the product is better	JD.com
Puning Dental	Cell Phone Battery	This user feels the product is better	JD.com
Puning Dental	Cell Phone Battery	Use it right now and punch a laptop.	JD.com
Puning Dental	Cell Phone Battery	Good quality, very cheap, good object	JD.com
Puning Dental	Cell Phone Battery	Support the new technology, the goods have been received. The effect of the use of follow-up comments!	JD.com
Puning Dental	Cell Phone Battery Repairer	especially well, thanks to the merchant's stuff works especially well, this is especially good. [Smiles] This very satisfied. This is very, very good, this satisfaction to you to play, I good praise.	JD.com
Puning Dental	Cell Phone Battery	Thanks to the merchant's stuff works exceptionally well, really exceptionally well possesses no problems at all!	JD.com
Puning Dental	Cell Phone Battery	Please return the product if there is any problem with the quality	JD.com
Puning Dental	Cell Phone Battery	I'm using it now with good use over and over and over again.	JD.com
Puning Dental	Cell Phone Battery	Logistics is very powerful Quality is very good Immediately repurchase	JD.com
Puning Dental	Cell Phone Battery	This user feels the product is better	JD.com
Puning Dental	Cell Phone Battery Repairer	is very fast, the customer service attitude is very positive, and questions are answered in a timely manner! Overall very satisfied!	JD.com
Puning Dental	Cell Phone Battery	This user feels the product is better	JD.com
Puning Dental	Cell Phone Battery	inexpensive and good quality, fast delivery, customer service is very good too!	JD.com
Puning Dental	Cell Phone Battery	I'll wait to use it before I say how I feel about it.	JD.com
Puning Dental	Cell Phone Battery Repairer	beautifully packaged/product! It is a good adapter to improve the battery life of mobilephone/optimize cell phonesystem/clean up cell phone garbage and improverunning speed of cell phone!Trustworthy/purchaseuse! I wish the manufacturer a prosperous business! We wish you a prosperous business! Popularity is strong and prosperous![Kudos][Buy]][Good][Buy]][Clap][Hands[Ah Huang][Handshake][Tongue]Rose]	JD.com
Puning Dental	Cell Phone Battery Repairer	The item has been received, the courier is also very fast, the product quality is very good, the workmanship is very refined, is genuine, the price is also very cheap, and the description is exactly the same, I give five-star praise.	JD.com
Puning Dental	Cell Phone Battery	Comments	JD.com
Puning Dental	Cell Phone Battery	Well, I can't say yet. Let's just use it and try it out! Uh-huh!	JD.com

Puning Dental	Cell Phone Battery	It looks good.	JD.com
Puning Dental	Cell Phone Battery	Repair has received, use good re-purchase wish the store business prosperity!	JD.com
Puning Dental	Cell Phone Battery	It works well.	JD.com
Puning Dental	Cell Phone Battery Repairer	Conversion efficiency: good<br>Compatibility: strong<br>Security: good	JD.com
Puning Dental	Cell Phone Battery	Cell phone adapter received looks good used well in buy a|	JD.com
Puning Dental	Cell Phone Battery	Received the item and it looks great. The style is also beautiful and okay.	JD.com
Puning Dental	Cell Phone Battery	Friends introduced to buy, on the cell phone pool repair remarkable, worth buying.	JD.com
Puning Dental	Cell Phone Battery	pretty good	JD.com
Puning Dental	Cell Phone Battery Repairer	The goods are very delicate, logistics is also fast, the business service is enthusiastic<br>Give five-star praise	JD.com
Puning Dental	Cell Phone Battery Repairer	Great price for what you get. Will buy the good stuff again in the future. Probably would work better at a higher price. Recommended purchase has been sent to the Poundland coterie.	JD.com
Puning Dental	Cell Phone Battery	I am very happy with your merchandise and the product is great.	JD.com
Puning Dental	Cell Phone Battery	Useful to repair fast charging good quality	JD.com
Puning Dental	Cell Phone Battery	products also fraudulent consumer business or unlicensed operation!	JD.com
Puning Dental	Cell Phone Battery Repairer	Authentic battery activator, cell phone repair is very easy, product quality is quite over the top, very satisfied with the special like, the price is cheap things are good, fine workmanship style is good, the service is thoughtful shipping fast, full-star praise a hundred praise!	JD.com
Puning Dental	Cell Phone Battery Repairer	This store's repairer is good, the cell phone battery activation rate is high, authentic product stuff is good, exquisite and beautiful high quality, fast shipping and thoughtful service, praise a full star high!	JD.com
Puning Dental	Cell Phone Battery Repairer	Cell phone repairer has received, genuine activator effect, product over the quality of high, something special good spend less, very satisfied with the super like, thoughtful service delivery fast, full of stars!	JD.com
Puning Dental	Cell Phone Battery Repairer	smooth, fine workmanship kind of beautiful, product quality is quite over the top, the price is cheap things are good, the service is enthusiastic and thoughtful, fast delivery full of stars high light!	JD.com
Puning Dental	Cell Phone Battery Repairer	Cheap and affordable<br>Stability: good<br>Will repurchase again	JD.com
Puning Dental	Cell Phone Battery	Quality can be, the courier boy is very enthusiastic please you can rest assured to buy!	JD.com
Puning Dental	Cell Phone Battery	This item is great quality and fast shipping.	JD.com
Puning Dental	Cell Phone Battery	classifier for objects in rows such as words	JD.com
Puning Dental	Cell Phone Battery Repairer	Received a cell phone battery repairer, the quality is quite good, exquisite packaging, good seller service attitude, logistics and express timely, all five-star praise. Wish merchants prosperous business.	JD.com
Puning Dental	Cell Phone Battery	Well packaged, inexpensive and easy to use.	JD.com
Puning Dental	Cell Phone Battery	Charging is average, charging is so slow	JD.com
Puning Dental	Cell Phone Battery	Very good satisfied next time come back[buy buy buy][kudos][kudos]	JD.com
Puning Dental	Cell Phone Battery	This user feels the product is better	JD.com
Puning Dental	Cell Phone Battery	Review after use.	JD.com
Puning Dental	Cell Phone Battery Repairer	Battery Repairer received, quite good, good quality, carefully packaged, intact, good business attitude, logistics and express timely, all five-star praise.	JD.com
Puning Dental	Cell Phone Battery	Plus this is similar to slow charging, it's good for battery protection	JD.com
Puning Dental	Cell Phone Battery	Great price and fast shipping! Packaging was great!	JD.com
Puning Dental	Cell Phone Battery	Works great and fits perfectly. It also plugs in.	JD.com

Puning Dental	Cell Phone Battery Repairer	The quality is pretty good.	JD.com
Puning Dental	Cell Phone Battery Repairer	The stuff works great, I'll buy another one later	JD.com
Puning Dental	Cell Phone Battery Repairer	IQ tax	JD.com
Puning Dental	Cell Phone Battery Repairer	This user feels the product is better	JD.com
Puning Dental	Cell Phone Battery Repairer	[Smiles][Skimming][Big Love]	JD.com
Puning Dental	Cell Phone Battery Repairer	Baby is very good, the logistics is very fast, the quality of workmanship is very good, cheaper than the entity store, trustworthy, like friends hurry to get down to it , , , , , , , , , , , , , , ,	JD.com
Puning Dental	Cell Phone Battery Repairer	False advertising, false products	JD.com
Puning Dental	Cell Phone Battery Repairer	I've got more memory with this. It's not laggy.	JD.com
Puning Dental	Cell Phone Battery Repairer	! ! ! ! ! ! ! ! ! ! !	JD.com
Puning Dental	Cell Phone Battery Repairer	This user feels the product is better	JD.com
Puning Dental	Cell Phone Battery Repairer	This user feels the product is better	JD.com
Puning Dental	Cell Phone Battery Repairer	Use it right now and punch a laptop.	JD.com
Puning Dental	Cell Phone Battery Repairer	Good quality, very cheap, good object	JD.com
Puning Dental	Cell Phone Battery Repairer	Support the new technology, the goods have been received. The effect of the use of follow-up comments!	JD.com
Puning Dental	Cell Phone Battery Repairer	especially well, thanks to the merchant's stuff works especially well, this is especially good.[Smiles]This very satisfied. This is very, very good, this satisfaction to you to play, I good praise.	JD.com
Puning Dental	Cell Phone Battery Repairer	Thanks to the merchant's stuff works exceptionally well, really exceptionally well possesses no problems at all!	JD.com
Puning Dental	Cell Phone Battery Repairer	Please return the product if there is any problem with the quality	JD.com
Puning Dental	Cell Phone Battery Repairer	I'm using it now with good use over and over and over again.	JD.com
Puning Dental	Cell Phone Battery Repairer	Logistics is very powerful Quality is very good Immediately repurchase	JD.com
Puning Dental	Cell Phone Battery Repairer	This user feels the product is better	JD.com
Puning Dental	Cell Phone Battery Repairer	is very fast, the customer service attitude is very positive, and questions are answered in a timely manner! Overall very satisfied!	JD.com
Puning Dental	Cell Phone Battery Repairer	This user feels the product is better	JD.com
Puning Dental	Cell Phone Battery Repairer	inexpensive and good quality, fast delivery, customer service is very good too!	JD.com
Puning Dental	Cell Phone Battery Repairer	I'll wait to use it before I say how I feel about it.	JD.com
Puning Dental	Cell Phone Battery Repairer	beautifully packaged/product! It is a good adapter to improve the battery life of mobilephone/optimize cell phonesystem/clean up cell phone garbage and improverunning speed of cell phone!Trustworthy/purchaseuse! I wish the manufacturer a prosperous business! We wish you a prosperous business! Popularity is strong and prosperous![Kudos][Buy]][Good][Buy]][Clap][Hands[Ah Huang][Handshake][Tongue]Rose]	JD.com
Puning Dental	Cell Phone Battery Repairer	The item has been received, the courier is also very fast, the product quality is very good, the workmanship is very refined, is genuine, the price is also very cheap, and the description is exactly the same, I give five-star praise.	JD.com
Puning Dental	Cell Phone Battery Repairer	Comments	JD.com
Puning Dental	Cell Phone Battery Repairer	Well, I can't say yet. Let's just use it and try it out! Uh-huh!	JD.com
Puning Dental	Cell Phone Battery Repairer	It looks good.	JD.com
Puning Dental	Cell Phone Battery Repairer	Repair has received, use good re-purchase wish the store business prosperity!	JD.com
Puning Dental	Cell Phone Battery Repairer	It works well.	JD.com
Puning Dental	Cell Phone Battery Repairer	Conversion efficiency: good<br>Compatibility: strong<br>Security: good	JD.com

Puning Dental	Cell Phone Battery	Cell phone adapter received looks good used well in buy a	JD.com
Puning Dental	Cell Phone Battery	Received the item and it looks great. The style is also beautiful and okay.	JD.com
Puning Dental	Cell Phone Battery	Friends introduced to buy, on the cell phone pool repair remarkable, worth buying.	JD.com
Puning Dental	Cell Phone Battery	pretty good	JD.com
Puning Dental	Cell Phone Battery Repairer	The goods are very delicate, logistics is also fast, the business service is enthusiastic<br>Give five-star praise	JD.com
Puning Dental	Cell Phone Battery Repairer	Great price for what you get. Will buy the good stuff again in the future. Probably would work better at a higher price. Recommended purchase has been sent to the Poundland coterie.	JD.com
Puning Dental	Cell Phone Battery	I am very happy with your merchandise and the product is great.	JD.com
Puning Dental	Cell Phone Battery	Useful to repair fast charging good quality	JD.com
Puning Dental	Cell Phone Battery	products also fraudulent consumer business or unlicensed operation!	JD.com
Puning Dental	Cell Phone Battery Repairer	Authentic battery activator, cell phone repair is very easy, product quality is quite over the top, very satisfied with the special like, the price is cheap things are good, fine workmanship style is good, the service is thoughtful shipping fast, full-star praise a hundred praise!	JD.com
Puning Dental	Cell Phone Battery Repairer	This store's repairer is good, the cell phone battery activation rate is high, authentic product stuff is good, exquisite and beautiful high quality, fast shipping and thoughtful service, praise a full star high!	JD.com
Puning Dental	Cell Phone Battery Repairer	Cell phone repairer has received, genuine activator effect, product over the quality of high, something special good spend less, very satisfied with the super like, thoughtful service delivery fast, full of stars!	JD.com
Puning Dental	Cell Phone Battery Repairer	smooth, fine workmanship kind of beautiful, product quality is quite over the top, the price is cheap things are good, the service is enthusiastic and thoughtful, fast delivery full of stars high light!	JD.com
Puning Dental	Cell Phone Battery Repairer	Cheap and affordable<br>Stability: good<br>Will repurchase again	JD.com
Puning Dental	Cell Phone Battery	Quality can be, the courier boy is very enthusiastic please you can rest assured to buy!	JD.com
Puning Dental	Cell Phone Battery	This item is great quality and fast shipping.	JD.com
Puning Dental	Cell Phone Battery	classifier for objects in rows such as words	JD.com
Puning Dental	Cell Phone Battery Repairer	Received a cell phone battery repairer, the quality is quite good, exquisite packaging, good seller service attitude, logistics and express timely, all five-star praise. Wish merchants prosperous business.	JD.com
Puning Dental	Cell Phone Battery	Well packaged, inexpensive and easy to use.	JD.com
Puning Dental	Cell Phone Battery	Charging is average, charging is so slow	JD.com
Puning Dental	Cell Phone Battery	Very good satisfied next time come back[buy buy buy][kudos][kudos]	JD.com
Puning Dental	Cell Phone Battery	This user feels the product is better	JD.com
Puning Dental	Cell Phone Battery	Review after use.	JD.com
Puning Dental	Cell Phone Battery Repairer	Battery Repairer received, quite good, good quality, carefully packaged, intact, good business attitude, logistics and express timely, all five-star praise.	JD.com
Puning Dental	Cell Phone Battery	Plus this is similar to slow charging, it's good for battery protection	JD.com
Puning Dental	Cell Phone Battery	Great price and fast shipping! Packaging was great!	JD.com
Puning Dental	Cell Phone Battery	Works great and fits perfectly. It also plugs in.	JD.com
Puning Dental	Cell Phone Battery	The quality is pretty good.	JD.com
Puning Dental	Cell Phone Battery	The stuff works great, I'll buy another one later	JD.com
Puning Dental	Cell Phone Battery	IQ tax	JD.com
Puning Dental	Cell Phone Battery	This user feels the product is better	JD.com
Puning Dental	Cell Phone Battery	[Smiles][Skimming][Big Love]	JD.com

Puning Dental	Cell Phone Battery Repairer	Baby is very good, the logistics is very fast, the quality of workmanship is very good, cheaper than the entity store, trustworthy, like friends hurry to get down to it , , , , , , , , , , , , , , ,	JD.com
Puning Dental	Cell Phone Battery	False advertising, false products	JD.com
Puning Dental	Cell Phone Battery	I've got more memory with this. It's not laggy.	JD.com
Puning Dental	Cell Phone Battery	! ! ! ! ! ! ! ! ! ! !	JD.com
Puning Dental	Cell Phone Battery	This user feels the product is better	JD.com
Puning Dental	Cell Phone Battery	This user feels the product is better	JD.com
Puning Dental	Cell Phone Battery	Use it right now and punch a laptop.	JD.com
Puning Dental	Cell Phone Battery	Good quality, very cheap, good object	JD.com
Puning Dental	Cell Phone Battery	Support the new technology, the goods have been received. The effect of the use of follow-up comments!	JD.com
Puning Dental	Cell Phone Battery Repairer	especially well, thanks to the merchant's stuff works especially well, this is especially good.[Smiles]This very satisfied. This is very, very good, this satisfaction to you to play, I good praise.	JD.com
Puning Dental	Cell Phone Battery	Thanks to the merchant's stuff works exceptionally well, really exceptionally well possesses no problems at all!	JD.com
Puning Dental	Cell Phone Battery	Please return the product if there is any problem with the quality	JD.com
Puning Dental	Cell Phone Battery	I'm using it now with good use over and over and over again.	JD.com
Puning Dental	Cell Phone Battery	Logistics is very powerful Quality is very good Immediately repurchase	JD.com
Puning Dental	Cell Phone Battery	This user feels the product is better	JD.com
Puning Dental	Cell Phone Battery Repairer	is very fast, the customer service attitude is very positive, and questions are answered in a timely manner! Overall very satisfied!	JD.com
Puning Dental	Cell Phone Battery	This user feels the product is better	JD.com
Puning Dental	Cell Phone Battery	inexpensive and good quality, fast delivery, customer service is very good too!	JD.com
Puning Dental	Cell Phone Battery	I'll wait to use it before I say how I feel about it.	JD.com
Puning Dental	Cell Phone Battery Repairer	beautifully packaged/product! It is a good adapter to improve the battery life of mobilephone/optimize cell phonesystem/clean up cell phone garbage and improverunning speed of cell phone!Trustworthy/purchaseuse! I wish the manufacturer a prosperous business! We wish you a prosperous business! Popularity is strong and prosperous![Kudos][Buy]][Good][Buy]][Clap][Hands[Ah Huang][Handshake][Tongue]Rose]	JD.com
Puning Dental	Cell Phone Battery Repairer	The item has been received, the courier is also very fast, the product quality is very good, the workmanship is very refined, is genuine, the price is also very cheap, and the description is exactly the same, I give five-star praise.	JD.com
Puning Dental	Cell Phone Battery	Comments	JD.com
Puning Dental	Cell Phone Battery	Well, I can't say yet. Let's just use it and try it out! Uh-huh!	JD.com
Puning Dental	Cell Phone Battery	It looks good.	JD.com
Puning Dental	Cell Phone Battery	Repair has received, use good re-purchase wish the store business prosperity!	JD.com
Puning Dental	Cell Phone Battery	It works well.	JD.com
Puning Dental	Cell Phone Battery Repairer	Conversion efficiency: good<br>Compatibility: strong<br>Security: good	JD.com
Puning Dental	Cell Phone Battery	Cell phone adapter received looks good used well in buy a|	JD.com
Puning Dental	Cell Phone Battery	Received the item and it looks great. The style is also beautiful and okay.	JD.com
Puning Dental	Cell Phone Battery	Friends introduced to buy, on the cell phone pool repair remarkable, worth buying.	JD.com
Puning Dental	Cell Phone Battery	pretty good	JD.com

Puning Dental	Cell Phone Battery Repairer	The goods are very delicate, logistics is also fast, the business service is enthusiastic<br>Give five-star praise	JD.com
Puning Dental	Cell Phone Battery Repairer	Great price for what you get. Will buy the good stuff again in the future. Probably would work better at a higher price. Recommended purchase has been sent to the Poundland coterie.	JD.com
Puning Dental	Cell Phone Battery	I am very happy with your merchandise and the product is great.	JD.com
Puning Dental	Cell Phone Battery	Useful to repair fast charging good quality	JD.com
Puning Dental	Cell Phone Battery	products also fraudulent consumer business or unlicensed operation!	JD.com
Puning Dental	Cell Phone Battery Repairer	Authentic battery activator, cell phone repair is very easy, product quality is quite over the top, very satisfied with the special like, the price is cheap things are good, fine workmanship style is good, the service is thoughtful shipping fast, full-star praise a hundred praise!	JD.com
Puning Dental	Cell Phone Battery Repairer	This store's repairer is good, the cell phone battery activation rate is high, authentic product stuff is good, exquisite and beautiful high quality, fast shipping and thoughtful service, praise a full star high!	JD.com
Puning Dental	Cell Phone Battery Repairer	Cell phone repairer has received, genuine activator effect, product over the quality of high, something special good spend less, very satisfied with the super like, thoughtful service delivery fast, full of stars!	JD.com
Puning Dental	Cell Phone Battery Repairer	smooth, fine workmanship kind of beautiful, product quality is quite over the top, the price is cheap things are good, the service is enthusiastic and thoughtful, fast delivery full of stars high light!	JD.com
Puning Dental	Cell Phone Battery Repairer	Cheap and affordable<br>Stability: good<br>Will repurchase again	JD.com
Puning Dental	Cell Phone Battery	Quality can be, the courier boy is very enthusiastic please you can rest assured to buy!	JD.com
Puning Dental	Cell Phone Battery	This item is great quality and fast shipping.	JD.com
Puning Dental	Cell Phone Battery	classifier for objects in rows such as words	JD.com
Puning Dental	Cell Phone Battery Repairer	Received a cell phone battery repairer, the quality is quite good, exquisite packaging, good seller service attitude, logistics and express timely, all five-star praise. Wish merchants prosperous business.	JD.com
Puning Dental	Cell Phone Battery	Well packaged, inexpensive and easy to use.	JD.com
Puning Dental	Cell Phone Battery	Charging is average, charging is so slow	JD.com
Puning Dental	Cell Phone Battery	Very good satisfied next time come back[buy buy buy][kudos][kudos]	JD.com
Puning Dental	Cell Phone Battery	This user feels the product is better	JD.com
Puning Dental	Cell Phone Battery	Review after use.	JD.com
Puning Dental	Cell Phone Battery Repairer	Battery Repairer received, quite good, good quality, carefully packaged, intact, good business attitude, logistics and express timely, all five-star praise.	JD.com
Puning Dental	Cell Phone Battery	Plus this is similar to slow charging, it's good for battery protection	JD.com
Puning Dental	Cell Phone Battery	Great price and fast shipping! Packaging was great!	JD.com
Puning Dental	Cell Phone Battery	Works great and fits perfectly. It also plugs in.	JD.com
Puning Dental	Cell Phone Battery	The quality is pretty good.	JD.com
Puning Dental	Cell Phone Battery	The stuff works great, I'll buy another one later	JD.com
Puning Dental	Cell Phone Battery	IQ tax	JD.com
Puning Dental	Cell Phone Battery	This user feels the product is better	JD.com
Puning Dental	Cell Phone Battery	[Smiles][Skimming][Big Love]	JD.com
Puning Dental	Cell Phone Battery Repairer	Baby is very good, the logistics is very fast, the quality of workmanship is very good, cheaper than the entity store, trustworthy, like friends hurry to get down to it<br>, , , , , , , , , , , , , , ,	JD.com
Puning Dental	Cell Phone Battery	False advertising, false products	JD.com
Puning Dental	Cell Phone Battery	I've got more memory with this. It's not laggy.	JD.com

Puning Dental	Cell Phone Battery	！！！！！！！！！！！	JD.com
Puning Dental	Cell Phone Battery	This user feels the product is better	JD.com
Puning Dental	Cell Phone Battery	This user feels the product is better	JD.com
Puning Dental	Cell Phone Battery	Use it right now and punch a laptop.	JD.com
Puning Dental	Cell Phone Battery	Good quality, very cheap, good object	JD.com
Puning Dental	Cell Phone Battery	Support the new technology, the goods have been received. The effect of the use of follow-up comments!	JD.com
Puning Dental	Cell Phone Battery Repairer	especially well, thanks to the merchant's stuff works especially well, this is especially good.[Smiles] This very satisfied. This is very, very good, this satisfaction to you to play, I good praise.	JD.com
Puning Dental	Cell Phone Battery	Thanks to the merchant's stuff works exceptionally well, really exceptionally well possesses no problems at all!	JD.com
Puning Dental	Cell Phone Battery	Please return the product if there is any problem with the quality	JD.com
Puning Dental	Cell Phone Battery	I'm using it now with good use over and over and over again.	JD.com
Puning Dental	Cell Phone Battery	Logistics is very powerful Quality is very good Immediately repurchase	JD.com
Puning Dental	Cell Phone Battery	This user feels the product is better	JD.com
Puning Dental	Cell Phone Battery Repairer	is very fast, the customer service attitude is very positive, and questions are answered in a timely manner! Overall very satisfied!	JD.com
Puning Dental	Cell Phone Battery	This user feels the product is better	JD.com
Puning Dental	Cell Phone Battery	inexpensive and good quality, fast delivery, customer service is very good too!	JD.com
Puning Dental	Cell Phone Battery	I'll wait to use it before I say how I feel about it.	JD.com
Puning Dental	Cell Phone Battery Repairer	beautifully packaged/product! It is a good adapter to improve the battery life of mobilephone/optimize cell phonesystem/clean up cell phone garbage and improverunning speed of cell phone!Trustworthy/purchaseuse! I wish the manufacturer a prosperous business! We wish you a prosperous business! Popularity is strong and prosperous![Kudos][Buy]][Good][Buy]][Clap][Hands[Ah Huang][Handshake][Tongue]Rose]	JD.com
Puning Dental	Cell Phone Battery Repairer	The item has been received, the courier is also very fast, the product quality is very good, the workmanship is very refined, is genuine, the price is also very cheap, and the description is exactly the same, I give five-star praise.	JD.com
Puning Dental	Cell Phone Battery	Comments	JD.com
Puning Dental	Cell Phone Battery	Well, I can't say yet. Let's just use it and try it out! Uh-huh!	JD.com
Puning Dental	Cell Phone Battery	It looks good.	JD.com
Puning Dental	Cell Phone Battery	Repair has received, use good re-purchase wish the store business prosperity!	JD.com
Puning Dental	Cell Phone Battery	It works well.	JD.com
Puning Dental	Cell Phone Battery Repairer	Conversion efficiency: good Compatibility: strong Security: good	JD.com
Puning Dental	Cell Phone Battery	Cell phone adapter received looks good used well in buy a|	JD.com
Puning Dental	Cell Phone Battery	Received the item and it looks great. The style is also beautiful and okay.	JD.com
Puning Dental	Cell Phone Battery	Friends introduced to buy, on the cell phone pool repair remarkable, worth buying.	JD.com
Puning Dental	Cell Phone Battery	pretty good	JD.com
Puning Dental	Cell Phone Battery Repairer	The goods are very delicate, logistics is also fast, the business service is enthusiastic Give five-star praise	JD.com
Puning Dental	Cell Phone Battery Repairer	Great price for what you get. Will buy the good stuff again in the future. Probably would work better at a higher price. Recommended purchase has been sent to the Poundland coterie.	JD.com
Puning Dental	Cell Phone Battery	I am very happy with your merchandise and the product is great.	JD.com
Puning Dental	Cell Phone Battery	Useful to repair fast charging good quality	JD.com

Puning Dental	Cell Phone Battery	products also fraudulent consumer business or unlicensed operation!	JD.com
Puning Dental	Cell Phone Battery Repairer	Authentic battery activator, cell phone repair is very easy, product quality is quite over the top, very satisfied with the special like, the price is cheap things are good, fine workmanship style is good, the service is thoughtful shipping fast, full-star praise a hundred praise!	JD.com
Puning Dental	Cell Phone Battery Repairer	This store's repairer is good, the cell phone battery activation rate is high, authentic product stuff is good, exquisite and beautiful high quality, fast shipping and thoughtful service, praise a full star high!	JD.com
Puning Dental	Cell Phone Battery Repairer	Cell phone repairer has received, genuine activator effect, product over the quality of high, something special good spend less, very satisfied with the super like, thoughtful service delivery fast, full of stars!	JD.com
Puning Dental	Cell Phone Battery Repairer	smooth, fine workmanship kind of beautiful, product quality is quite over the top, the price is cheap things are good, the service is enthusiastic and thoughtful, fast delivery full of stars high light!	JD.com
Puning Dental	Cell Phone Battery Repairer	Cheap and affordable<br>Stability: good<br>Will repurchase again	JD.com
Puning Dental	Cell Phone Battery	Quality can be, the courier boy is very enthusiastic please you can rest assured to buy!	JD.com
Puning Dental	Cell Phone Battery	This item is great quality and fast shipping.	JD.com
Puning Dental	Cell Phone Battery	classifier for objects in rows such as words	JD.com
Puning Dental	Cell Phone Battery Repairer	Received a cell phone battery repairer, the quality is quite good, exquisite packaging, good seller service attitude, logistics and express timely, all five-star praise. Wish merchants prosperous business.	JD.com
Puning Dental	Cell Phone Battery	Well packaged, inexpensive and easy to use.	JD.com
Puning Dental	Cell Phone Battery	Charging is average, charging is so slow	JD.com
Puning Dental	Cell Phone Battery	Very good satisfied next time come back[buy buy buy][kudos][kudos]	JD.com
Puning Dental	Cell Phone Battery	This user feels the product is better	JD.com
Puning Dental	Cell Phone Battery	Review after use.	JD.com
Puning Dental	Cell Phone Battery Repairer	Battery Repairer received, quite good, good quality, carefully packaged, intact, good business attitude, logistics and express timely, all five-star praise.	JD.com
Puning Dental	Cell Phone Battery	Plus this is similar to slow charging, it's good for battery protection	JD.com
Puning Dental	Cell Phone Battery	Great price and fast shipping! Packaging was great!	JD.com
Puning Dental	Cell Phone Battery	Works great and fits perfectly. It also plugs in.	JD.com
Puning Dental	Cell Phone Battery	The quality is pretty good.	JD.com
Puning Dental	Cell Phone Battery	The stuff works great, I'll buy another one later	JD.com
Puning Dental	Cell Phone Battery	IQ tax	JD.com
Puning Dental	Cell Phone Battery	This user feels the product is better	JD.com
Puning Dental	Cell Phone Battery	[Smiles][Skimming][Big Love]	JD.com
Puning Dental	Cell Phone Battery Repairer	Baby is very good, the logistics is very fast, the quality of workmanship is very good, cheaper than the entity store, trustworthy, like friends hurry to get down to it , , , , , , , , , , , , , ,	JD.com
Puning Dental	Cell Phone Battery	False advertising, false products	JD.com
Puning Dental	Cell Phone Battery	I've got more memory with this. It's not laggy.	JD.com
Puning Dental	Cell Phone Battery	! ! ! ! ! ! ! ! ! ! !	JD.com
Puning Dental	Cell Phone Battery	This user feels the product is better	JD.com
Puning Dental	Cell Phone Battery	This user feels the product is better	JD.com
Puning Dental	Cell Phone Battery	Use it right now and punch a laptop.	JD.com
Puning Dental	Cell Phone Battery	Good quality, very cheap, good object	JD.com

Puning Dental	Cell Phone Battery	Support the new technology, the goods have been received. The effect of the use of follow-up comments!	JD.com
Puning Dental	Cell Phone Battery Repairer	especially well, thanks to the merchant's stuff works especially well, this is especially good.[Smiles]This very satisfied. This is very, very good, this satisfaction to you to play, I good praise.	JD.com
Puning Dental	Cell Phone Battery	Thanks to the merchant's stuff works exceptionally well, really exceptionally well possesses no problems at all!	JD.com
Puning Dental	Cell Phone Battery	Please return the product if there is any problem with the quality	JD.com
Puning Dental	Cell Phone Battery	I'm using it now with good use over and over and over again.	JD.com
Puning Dental	Cell Phone Battery	Logistics is very powerful Quality is very good Immediately repurchase	JD.com
Puning Dental	Cell Phone Battery	This user feels the product is better	JD.com
Puning Dental	Cell Phone Battery Repairer	is very fast, the customer service attitude is very positive, and questions are answered in a timely manner! Overall very satisfied!	JD.com
Puning Dental	Cell Phone Battery	This user feels the product is better	JD.com
Puning Dental	Cell Phone Battery	inexpensive and good quality, fast delivery, customer service is very good too!	JD.com
Puning Dental	Cell Phone Battery	I'll wait to use it before I say how I feel about it.	JD.com
Puning Dental	Cell Phone Battery Repairer	beautifully packaged/product! It is a good adapter to improve the battery life of mobilephone/optimize cell phonesystem/clean up cell phone garbage and improverunning speed of cell phone!Trustworthy/purchaseuse! I wish the manufacturer a prosperous business! We wish you a prosperous business! Popularity is strong and prosperous![Kudos][Buy]][Good][Buy]][Clap][Hands[Ah Huang][Handshake][Tongue]Rose]	JD.com
Puning Dental	Cell Phone Battery Repairer	The item has been received, the courier is also very fast, the product quality is very good, the workmanship is very refined, is genuine, the price is also very cheap, and the description is exactly the same, I give five-star praise.	JD.com
Puning Dental	Cell Phone Battery	Comments	JD.com
Puning Dental	Cell Phone Battery	Well, I can't say yet. Let's just use it and try it out! Uh-huh!	JD.com
Puning Dental	Cell Phone Battery	It looks good.	JD.com
Puning Dental	Cell Phone Battery	Repair has received, use good re-purchase wish the store business prosperity!	JD.com
Puning Dental	Cell Phone Battery	It works well.	JD.com
Puning Dental	Cell Phone Battery Repairer	Conversion efficiency: good<br>Compatibility: strong<br>Security: good	JD.com
Puning Dental	Cell Phone Battery	Cell phone adapter received looks good used well in buy a|	JD.com
Puning Dental	Cell Phone Battery	Received the item and it looks great. The style is also beautiful and okay.	JD.com
Puning Dental	Cell Phone Battery	Friends introduced to buy, on the cell phone pool repair remarkable, worth buying.	JD.com
Puning Dental	Cell Phone Battery	pretty good	JD.com
Puning Dental	Cell Phone Battery Repairer	The goods are very delicate, logistics is also fast, the business service is enthusiastic<br>Give five-star praise	JD.com
Puning Dental	Cell Phone Battery Repairer	Great price for what you get. Will buy the good stuff again in the future. Probably would work better at a higher price. Recommended purchase has been sent to the Poundland coterie.	JD.com
Puning Dental	Cell Phone Battery	I am very happy with your merchandise and the product is great.	JD.com
Puning Dental	Cell Phone Battery	Useful to repair fast charging good quality	JD.com
Puning Dental	Cell Phone Battery	products also fraudulent consumer business or unlicensed operation!	JD.com
Puning Dental	Cell Phone Battery Repairer	Authentic battery activator, cell phone repair is very easy, product quality is quite over the top, very satisfied with the special like, the price is cheap things are good, fine workmanship style is good, the service is thoughtful shipping fast, full-star praise a hundred praise!	JD.com

Puning Dental	Cell Phone Battery Repairer	This store's repairer is good, the cell phone battery activation rate is high, authentic product stuff is good, exquisite and beautiful high quality, fast shipping and thoughtful service, praise a full star high!	JD.com
Puning Dental	Cell Phone Battery Repairer	Cell phone repairer has received, genuine activator effect, product over the quality of high, something special good spend less, very satisfied with the super like, thoughtful service delivery fast, full of stars!	JD.com
Puning Dental	Cell Phone Battery Repairer	smooth, fine workmanship kind of beautiful, product quality is quite over the top, the price is cheap things are good, the service is enthusiastic and thoughtful, fast delivery full of stars high light!	JD.com
Puning Dental	Cell Phone Battery Repairer	Cheap and affordable<br>Stability: good<br>Will repurchase again	JD.com
Puning Dental	Cell Phone Battery Repairer	Quality can be, the courier boy is very enthusiastic please you can rest assured to buy!	JD.com
Puning Dental	Cell Phone Battery Repairer	This item is great quality and fast shipping.	JD.com
Puning Dental	Cell Phone Battery Repairer	classifier for objects in rows such as words	JD.com
Puning Dental	Cell Phone Battery Repairer	Received a cell phone battery repairer, the quality is quite good, exquisite packaging, good seller service attitude, logistics and express timely, all five-star praise. Wish merchants prosperous business.	JD.com
Puning Dental	Cell Phone Battery Repairer	Well packaged, inexpensive and easy to use.	JD.com
Puning Dental	Cell Phone Battery Repairer	Charging is average, charging is so slow	JD.com
Puning Dental	Cell Phone Battery Repairer	Very good satisfied next time come back[buy buy buy][kudos][kudos]	JD.com
Puning Dental	Cell Phone Battery Repairer	This user feels the product is better	JD.com
Puning Dental	Cell Phone Battery Repairer	Review after use.	JD.com
Puning Dental	Cell Phone Battery Repairer	Battery Repairer received, quite good, good quality, carefully packaged, intact, good business attitude, logistics and express timely, all five-star praise.	JD.com
Puning Dental	Cell Phone Battery Repairer	Plus this is similar to slow charging, it's good for battery protection	JD.com
Puning Dental	Cell Phone Battery Repairer	Great price and fast shipping! Packaging was great!	JD.com
Puning Dental	Cell Phone Battery Repairer	Works great and fits perfectly. It also plugs in.	JD.com
Puning Dental	Cell Phone Battery Repairer	The quality is pretty good.	JD.com
Puning Dental	Cell Phone Battery Repairer	The stuff works great, I'll buy another one later	JD.com
Puning Dental	Cell Phone Battery Repairer	IQ tax	JD.com
Puning Dental	Cell Phone Battery Repairer	This user feels the product is better	JD.com
Puning Dental	Cell Phone Battery Repairer	[Smiles][Skimming][Big Love]	JD.com
Puning Dental	Cell Phone Battery Repairer	Baby is very good, the logistics is very fast, the quality of workmanship is very good, cheaper than the entity store, trustworthy, like friends hurry to get down to it , , , , , , , , , , , , , , ,	JD.com
Puning Dental	Cell Phone Battery Repairer	False advertising, false products	JD.com
Puning Dental	Cell Phone Battery Repairer	I've got more memory with this. It's not laggy.	JD.com
Puning Dental	Cell Phone Battery Repairer	! ! ! ! ! ! ! ! ! ! !	JD.com
Puning Dental	Cell Phone Battery Repairer	This user feels the product is better	JD.com
Puning Dental	Cell Phone Battery Repairer	This user feels the product is better	JD.com
Puning Dental	Cell Phone Battery Repairer	Use it right now and punch a laptop.	JD.com
Puning Dental	Cell Phone Battery Repairer	Good quality, very cheap, good object	JD.com
Puning Dental	Cell Phone Battery Repairer	Support the new technology, the goods have been received. The effect of the use of follow-up comments!	JD.com
Puning Dental	Cell Phone Battery Repairer	especially well, thanks to the merchant's stuff works especially well, this is especially good.[Smiles]This very satisfied. This is very, very good, this satisfaction to you to play, I good praise.	JD.com

Puning Dental	Cell Phone Battery	Thanks to the merchant's stuff works exceptionally well, really exceptionally well possesses no problems at all!	JD.com
Puning Dental	Cell Phone Battery	Please return the product if there is any problem with the quality	JD.com
Puning Dental	Cell Phone Battery	I'm using it now with good use over and over and over again.	JD.com
Puning Dental	Cell Phone Battery	Logistics is very powerful Quality is very good Immediately repurchase	JD.com
Puning Dental	Cell Phone Battery	This user feels the product is better	JD.com
Puning Dental	Cell Phone Battery Repairer	is very fast, the customer service attitude is very positive, and questions are answered in a timely manner! Overall very satisfied!	JD.com
Puning Dental	Cell Phone Battery	This user feels the product is better	JD.com
Puning Dental	Cell Phone Battery	inexpensive and good quality, fast delivery, customer service is very good too!	JD.com
Puning Dental	Cell Phone Battery	I'll wait to use it before I say how I feel about it.	JD.com
Puning Dental	Cell Phone Battery Repairer	beautifully packaged/product! It is a good adapter to improve the battery life of mobilephone/optimize cell phonesystem/clean up cell phone garbage and improverunning speed of cell phone!Trustworthy/purchaseuse! I wish the manufacturer a prosperous business! We wish you a prosperous business! Popularity is strong and prosperous![Kudos][Buy]][Good][Buy]][Clap][Hands[Ah Huang][Handshake][Tongue]Rose]	JD.com
Puning Dental	Cell Phone Battery Repairer	The item has been received, the courier is also very fast, the product quality is very good, the workmanship is very refined, is genuine, the price is also very cheap, and the description is exactly the same, I give five-star praise.	JD.com
Puning Dental	Cell Phone Battery	Comments	JD.com
Puning Dental	Cell Phone Battery	Well, I can't say yet. Let's just use it and try it out! Uh-huh!	JD.com
Puning Dental	Cell Phone Battery	It looks good.	JD.com
Puning Dental	Cell Phone Battery	Repair has received, use good re-purchase wish the store business prosperity!	JD.com
Puning Dental	Cell Phone Battery	It works well.	JD.com
Puning Dental	Cell Phone Battery Repairer	Conversion efficiency: good<br>Compatibility: strong<br>Security: good	JD.com
Puning Dental	Cell Phone Battery	Cell phone adapter received looks good used well in buy a	JD.com
Puning Dental	Cell Phone Battery	Received the item and it looks great. The style is also beautiful and okay.	JD.com
Puning Dental	Cell Phone Battery	Friends introduced to buy, on the cell phone pool repair remarkable, worth buying.	JD.com
Puning Dental	Cell Phone Battery	pretty good	JD.com
Puning Dental	Cell Phone Battery Repairer	The goods are very delicate, logistics is also fast, the business service is enthusiastic<br>Give five-star praise	JD.com
Puning Dental	Cell Phone Battery Repairer	Great price for what you get. Will buy the good stuff again in the future. Probably would work better at a higher price. Recommended purchase has been sent to the Poundland coterie.	JD.com
Puning Dental	Cell Phone Battery	I am very happy with your merchandise and the product is great.	JD.com
Puning Dental	Cell Phone Battery	Useful to repair fast charging good quality	JD.com
Puning Dental	Cell Phone Battery	products also fraudulent consumer business or unlicensed operation!	JD.com
Puning Dental	Cell Phone Battery Repairer	Authentic battery activator, cell phone repair is very easy, product quality is quite over the top, very satisfied with the special like, the price is cheap things are good, fine workmanship style is good, the service is thoughtful shipping fast, full-star praise a hundred praise!	JD.com
Puning Dental	Cell Phone Battery Repairer	This store's repairer is good, the cell phone battery activation rate is high, authentic product stuff is good, exquisite and beautiful high quality, fast shipping and thoughtful service, praise a full star high!	JD.com
Puning Dental	Cell Phone Battery Repairer	Cell phone repairer has received, genuine activator effect, product over the quality of high, something special good spend less, very satisfied with the super like, thoughtful service delivery fast, full of stars!	JD.com

Puning Dental	Cell Phone Battery Repairer	smooth, fine workmanship kind of beautiful, product quality is quite over the top, the price is cheap things are good, the service is enthusiastic and thoughtful, fast delivery full of stars high light!	JD.com
Puning Dental	Cell Phone Battery Repairer	Cheap and affordable<br>Stability: good<br>Will repurchase again	JD.com
Puning Dental	Cell Phone Battery	Quality can be, the courier boy is very enthusiastic please you can rest assured to buy!	JD.com
Puning Dental	Cell Phone Battery	This item is great quality and fast shipping.	JD.com
Puning Dental	Cell Phone Battery	classifier for objects in rows such as words	JD.com
Puning Dental	Cell Phone Battery Repairer	Received a cell phone battery repairer, the quality is quite good, exquisite packaging, good seller service attitude, logistics and express timely, all five-star praise. Wish merchants prosperous business.	JD.com
Puning Dental	Cell Phone Battery	Well packaged, inexpensive and easy to use.	JD.com
Puning Dental	Cell Phone Battery	Charging is average, charging is so slow	JD.com
Puning Dental	Cell Phone Battery	Very good satisfied next time come back[buy buy buy][kudos][kudos]	JD.com
Puning Dental	Cell Phone Battery	This user feels the product is better	JD.com
Puning Dental	Cell Phone Battery	Review after use.	JD.com
Puning Dental	Cell Phone Battery Repairer	Battery Repairer received, quite good, good quality, carefully packaged, intact, good business attitude, logistics and express timely, all five-star praise.	JD.com
Puning Dental	Cell Phone Battery	Plus this is similar to slow charging, it's good for battery protection	JD.com
Puning Dental	Cell Phone Battery	Great price and fast shipping! Packaging was great!	JD.com
Puning Dental	Cell Phone Battery	Works great and fits perfectly. It also plugs in.	JD.com
Puning Dental	Cell Phone Battery	The quality is pretty good.	JD.com
Puning Dental	Cell Phone Battery	The stuff works great, I'll buy another one later	JD.com
Puning Dental	Cell Phone Battery	IQ tax	JD.com
Puning Dental	Cell Phone Battery	This user feels the product is better	JD.com
Puning Dental	Cell Phone Battery	[Smiles][Skimming][Big Love]	JD.com
Puning Dental	Cell Phone Battery Repairer	Baby is very good, the logistics is very fast, the quality of workmanship is very good, cheaper than the entity store, trustworthy, like friends hurry to get down to it , , , , , , , , , , , , , , ,	JD.com
Puning Dental	Cell Phone Battery	False advertising, false products	JD.com
Puning Dental	Cell Phone Battery	I've got more memory with this. It's not laggy.	JD.com
Puning Dental	Cell Phone Battery	! ! ! ! ! ! ! ! ! ! !	JD.com
Puning Dental	Cell Phone Battery	This user feels the product is better	JD.com
Puning Dental	Cell Phone Battery	This user feels the product is better	JD.com
Puning Dental	Cell Phone Battery	Use it right now and punch a laptop.	JD.com
Puning Dental	Cell Phone Battery	Good quality, very cheap, good object	JD.com
Puning Dental	Cell Phone Battery	Support the new technology, the goods have been received. The effect of the use of follow-up comments!	JD.com
Puning Dental	Cell Phone Battery Repairer	especially well, thanks to the merchant's stuff works especially well, this is especially good.[Smiles]This very satisfied. This is very, very good, this satisfaction to you to play, I good praise.	JD.com
Puning Dental	Cell Phone Battery	Thanks to the merchant's stuff works exceptionally well, really exceptionally well possesses no problems at all!	JD.com
Puning Dental	Cell Phone Battery	Please return the product if there is any problem with the quality	JD.com
Puning Dental	Cell Phone Battery	I'm using it now with good use over and over and over again.	JD.com
Puning Dental	Cell Phone Battery	Logistics is very powerful Quality is very good Immediately repurchase	JD.com

Puning Dental	Cell Phone Battery [illegible]	This user feels the product is better	JD.com
Puning Dental	Cell Phone Battery Repairer	is very fast, the customer service attitude is very positive, and questions are answered in a timely manner! Overall very satisfied!	JD.com
Puning Dental	Cell Phone Battery [illegible]	This user feels the product is better	JD.com
Puning Dental	Cell Phone Battery [illegible]	inexpensive and good quality, fast delivery, customer service is very good too!	JD.com
Puning Dental	Cell Phone Battery [illegible]	I'll wait to use it before I say how I feel about it.	JD.com
Puning Dental	Cell Phone Battery Repairer	beautifully packaged/product! It is a good adapter to improve the battery life of mobilephone/optimize cell phonesystem/clean up cell phone garbage and improverunning speed of cell phone!Trustworthy/purchaseuse! I wish the manufacturer a prosperous business! We wish you a prosperous business! Popularity is strong and prosperous![Kudos][Buy]][Good][Buy]][Clap][Hands[Ah Huang][Handshake][Tongue]Rose]	JD.com
Puning Dental	Cell Phone Battery Repairer	The item has been received, the courier is also very fast, the product quality is very good, the workmanship is very refined, is genuine, the price is also very cheap, and the description is exactly the same, I give five-star praise.	JD.com
Puning Dental	Cell Phone Battery [illegible]	Comments	JD.com
Puning Dental	Cell Phone Battery [illegible]	Well, I can't say yet. Let's just use it and try it out! Uh-huh!	JD.com
Puning Dental	Cell Phone Battery [illegible]	It looks good.	JD.com
Puning Dental	Cell Phone Battery [illegible]	Repair has received, use good re-purchase wish the store business prosperity!	JD.com
Puning Dental	Cell Phone Battery [illegible]	It works well.	JD.com
Puning Dental	Cell Phone Battery Repairer	Conversion efficiency: good<br>Compatibility: strong<br>Security: good	JD.com
Puning Dental	Cell Phone Battery [illegible]	Cell phone adapter received looks good used well in buy a	JD.com
Puning Dental	Cell Phone Battery [illegible]	Received the item and it looks great. The style is also beautiful and okay.	JD.com
Puning Dental	Cell Phone Battery [illegible]	Friends introduced to buy, on the cell phone pool repair remarkable, worth buying.	JD.com
Puning Dental	Cell Phone Battery [illegible]	pretty good	JD.com
Puning Dental	Cell Phone Battery Repairer	The goods are very delicate, logistics is also fast, the business service is enthusiastic<br>Give five-star praise	JD.com
Puning Dental	Cell Phone Battery Repairer	Great price for what you get. Will buy the good stuff again in the future. Probably would work better at a higher price. Recommended purchase has been sent to the Poundland coterie.	JD.com
Puning Dental	Cell Phone Battery [illegible]	I am very happy with your merchandise and the product is great.	JD.com
Puning Dental	Cell Phone Battery [illegible]	Useful to repair fast charging good quality	JD.com
Puning Dental	Cell Phone Battery [illegible]	products also fraudulent consumer business or unlicensed operation!	JD.com
Puning Dental	Cell Phone Battery Repairer	Authentic battery activator, cell phone repair is very easy, product quality is quite over the top, very satisfied with the special like, the price is cheap things are good, fine workmanship style is good, the service is thoughtful shipping fast, full-star praise a hundred praise!	JD.com
Puning Dental	Cell Phone Battery Repairer	This store's repairer is good, the cell phone battery activation rate is high, authentic product stuff is good, exquisite and beautiful high quality, fast shipping and thoughtful service, praise a full star high!	JD.com
Puning Dental	Cell Phone Battery Repairer	Cell phone repairer has received, genuine activator effect, product over the quality of high, something special good spend less, very satisfied with the super like, thoughtful service delivery fast, full of stars!	JD.com
Puning Dental	Cell Phone Battery Repairer	smooth, fine workmanship kind of beautiful, product quality is quite over the top, the price is cheap things are good, the service is enthusiastic and thoughtful, fast delivery full of stars high light!	JD.com
Puning Dental	Cell Phone Battery Repairer	Cheap and affordable<br>Stability: good<br>Will repurchase again	JD.com
Puning Dental	Cell Phone Battery [illegible]	Quality can be, the courier boy is very enthusiastic please you can rest assured to buy!	JD.com

Puning Dental	Cell Phone Battery Repairer	This item is great quality and fast shipping.	JD.com
Puning Dental	Cell Phone Battery Repairer	classifier for objects in rows such as words	JD.com
Puning Dental	Cell Phone Battery Repairer	Received a cell phone battery repairer, the quality is quite good, exquisite packaging, good seller service attitude, logistics and express timely, all five-star praise. Wish merchants prosperous business.	JD.com
Puning Dental	Cell Phone Battery Repairer	Well packaged, inexpensive and easy to use.	JD.com
Puning Dental	Cell Phone Battery Repairer	Charging is average, charging is so slow	JD.com
Puning Dental	Cell Phone Battery Repairer	Very good satisfied next time come back[buy buy buy][kudos][kudos]	JD.com
Puning Dental	Cell Phone Battery Repairer	This user feels the product is better	JD.com
Puning Dental	Cell Phone Battery Repairer	Review after use.	JD.com
Puning Dental	Cell Phone Battery Repairer	Battery Repairer received, quite good, good quality, carefully packaged, intact, good business attitude, logistics and express timely, all five-star praise.	JD.com
Puning Dental	Cell Phone Battery Repairer	Plus this is similar to slow charging, it's good for battery protection	JD.com
Puning Dental	Cell Phone Battery Repairer	Great price and fast shipping! Packaging was great!	JD.com
Puning Dental	Cell Phone Battery Repairer	Works great and fits perfectly. It also plugs in.	JD.com
Puning Dental	Cell Phone Battery Repairer	The quality is pretty good.	JD.com
Puning Dental	Cell Phone Battery Repairer	The stuff works great, I'll buy another one later	JD.com
Puning Dental	Cell Phone Battery Repairer	IQ tax	JD.com
Puning Dental	Cell Phone Battery Repairer	This user feels the product is better	JD.com
Puning Dental	Cell Phone Battery Repairer	[Smiles][Skimming][Big Love]	JD.com
Puning Dental	Cell Phone Battery Repairer	Baby is very good, the logistics is very fast, the quality of workmanship is very good, cheaper than the entity store, trustworthy, like friends hurry to get down to it , , , , , , , , , , , , , , ,	JD.com
Puning Dental	Cell Phone Battery Repairer	False advertising, false products	JD.com
Puning Dental	Cell Phone Battery Repairer	I've got more memory with this. It's not laggy.	JD.com
Puning Dental	Cell Phone Battery Repairer	！！！！！！！！！！！	JD.com
Puning Dental	Cell Phone Battery Repairer	This user feels the product is better	JD.com
Puning Dental	Cell Phone Battery Repairer	This user feels the product is better	JD.com
Puning Dental	Cell Phone Battery Repairer	Use it right now and punch a laptop.	JD.com
Puning Dental	Cell Phone Battery Repairer	Good quality, very cheap, good object	JD.com
Puning Dental	Cell Phone Battery Repairer	Support the new technology, the goods have been received. The effect of the use of follow-up comments!	JD.com
Puning Dental	Cell Phone Battery Repairer	especially well, thanks to the merchant's stuff works especially well, this is especially good.[Smiles]This very satisfied. This is very, very good, this satisfaction to you to play, I good praise.	JD.com
Puning Dental	Cell Phone Battery Repairer	Thanks to the merchant's stuff works exceptionally well, really exceptionally well possesses no problems at all!	JD.com
Puning Dental	Cell Phone Battery Repairer	Please return the product if there is any problem with the quality	JD.com
Puning Dental	Cell Phone Battery Repairer	I'm using it now with good use over and over and over again.	JD.com
Puning Dental	Cell Phone Battery Repairer	Logistics is very powerful Quality is very good Immediately repurchase	JD.com
Puning Dental	Cell Phone Battery Repairer	This user feels the product is better	JD.com
Puning Dental	Cell Phone Battery Repairer	is very fast, the customer service attitude is very positive, and questions are answered in a timely manner! Overall very satisfied!	JD.com
Puning Dental	Cell Phone Battery Repairer	This user feels the product is better	JD.com
Puning Dental	Cell Phone Battery Repairer	inexpensive and good quality, fast delivery, customer service is very good too!	JD.com

Puning Dental	Cell Phone Battery	I'll wait to use it before I say how I feel about it.	JD.com
Puning Dental	Cell Phone Battery Repairer	beautifully packaged/product! It is a good adapter to improve the battery life of mobilephone/optimize cell phonesystem/clean up cell phone garbage and improverunning speed of cell phone!Trustworthy/purchaseuse! I wish the manufacturer a prosperous business! We wish you a prosperous business! Popularity is strong and prosperous![Kudos][Buy]][Good][Buy]][Clap][Hands[Ah Huang][Handshake][Tongue]Rose]	JD.com
Puning Dental	Cell Phone Battery Repairer	The item has been received, the courier is also very fast, the product quality is very good, the workmanship is very refined, is genuine, the price is also very cheap, and the description is exactly the same, I give five-star praise.	JD.com
Puning Dental	Cell Phone Battery	Comments	JD.com
Puning Dental	Cell Phone Battery	Well, I can't say yet. Let's just use it and try it out! Uh-huh!	JD.com
Puning Dental	Cell Phone Battery	It looks good.	JD.com
Puning Dental	Cell Phone Battery	Repair has received, use good re-purchase wish the store business prosperity!	JD.com
Puning Dental	Cell Phone Battery	It works well.	JD.com
Puning Dental	Cell Phone Battery Repairer	Conversion efficiency: good<br>Compatibility: strong<br>Security: good	JD.com
Puning Dental	Cell Phone Battery	Cell phone adapter received looks good used well in buy a	JD.com
Puning Dental	Cell Phone Battery	Received the item and it looks great. The style is also beautiful and okay.	JD.com
Puning Dental	Cell Phone Battery	Friends introduced to buy, on the cell phone pool repair remarkable, worth buying.	JD.com
Puning Dental	Cell Phone Battery	pretty good	JD.com
Puning Dental	Cell Phone Battery Repairer	The goods are very delicate, logistics is also fast, the business service is enthusiastic<br>Give five-star praise	JD.com
Puning Dental	Cell Phone Battery Repairer	Great price for what you get. Will buy the good stuff again in the future. Probably would work better at a higher price. Recommended purchase has been sent to the Poundland coterie.	JD.com
Puning Dental	Cell Phone Battery	I am very happy with your merchandise and the product is great.	JD.com
Puning Dental	Cell Phone Battery	Useful to repair fast charging good quality	JD.com
Puning Dental	Cell Phone Battery	products also fraudulent consumer business or unlicensed operation!	JD.com
Puning Dental	Cell Phone Battery Repairer	Authentic battery activator, cell phone repair is very easy, product quality is quite over the top, very satisfied with the special like, the price is cheap things are good, fine workmanship style is good, the service is thoughtful shipping fast, full-star praise a hundred praise!	JD.com
Puning Dental	Cell Phone Battery Repairer	This store's repairer is good, the cell phone battery activation rate is high, authentic product stuff is good, exquisite and beautiful high quality, fast shipping and thoughtful service, praise a full star high!	JD.com
Puning Dental	Cell Phone Battery Repairer	Cell phone repairer has received, genuine activator effect, product over the quality of high, something special good spend less, very satisfied with the super like, thoughtful service delivery fast, full of stars!	JD.com
Puning Dental	Cell Phone Battery Repairer	smooth, fine workmanship kind of beautiful, product quality is quite over the top, the price is cheap things are good, the service is enthusiastic and thoughtful, fast delivery full of stars high light!	JD.com
Puning Dental	Cell Phone Battery Repairer	Cheap and affordable<br>Stability: good<br>Will repurchase again	JD.com
Puning Dental	Cell Phone Battery	Quality can be, the courier boy is very enthusiastic please you can rest assured to buy!	JD.com
Puning Dental	Cell Phone Battery	This item is great quality and fast shipping.	JD.com
Puning Dental	Cell Phone Battery	classifier for objects in rows such as words	JD.com
Puning Dental	Cell Phone Battery Repairer	Received a cell phone battery repairer, the quality is quite good, exquisite packaging, good seller service attitude, logistics and express timely, all five-star praise. Wish merchants prosperous business.	JD.com

Puning Dental	Cell Phone Battery Repairer	Well packaged, inexpensive and easy to use.	JD.com
Puning Dental	Cell Phone Battery Repairer	Charging is average, charging is so slow	JD.com
Puning Dental	Cell Phone Battery Repairer	Very good satisfied next time come back[buy buy buy][kudos][kudos]	JD.com
Puning Dental	Cell Phone Battery Repairer	This user feels the product is better	JD.com
Puning Dental	Cell Phone Battery Repairer	Review after use.	JD.com
Puning Dental	Cell Phone Battery Repairer	Battery Repairer received, quite good, good quality, carefully packaged, intact, good business attitude, logistics and express timely, all five-star praise.	JD.com
Puning Dental	Cell Phone Battery Repairer	Plus this is similar to slow charging, it's good for battery protection	JD.com
Puning Dental	Cell Phone Battery Repairer	Great price and fast shipping! Packaging was great!	JD.com
Puning Dental	Cell Phone Battery Repairer	Works great and fits perfectly. It also plugs in.	JD.com
Puning Dental	Cell Phone Battery Repairer	The quality is pretty good.	JD.com
Puning Dental	Cell Phone Battery Repairer	The stuff works great, I'll buy another one later	JD.com
Puning Dental	Cell Phone Battery Repairer	IQ tax	JD.com
Puning Dental	Cell Phone Battery Repairer	This user feels the product is better	JD.com
Puning Dental	Cell Phone Battery Repairer	[Smiles][Skimming][Big Love]	JD.com
Puning Dental	Cell Phone Battery Repairer	Baby is very good, the logistics is very fast, the quality of workmanship is very good, cheaper than the entity store, trustworthy, like friends hurry to get down to it , , , , , , , , , , , , , , ,	JD.com
Puning Dental	Cell Phone Battery Repairer	False advertising, false products	JD.com
Puning Dental	Cell Phone Battery Repairer	I've got more memory with this. It's not laggy.	JD.com
Puning Dental	Cell Phone Battery Repairer	! ! ! ! ! ! ! ! ! ! !	JD.com
Puning Dental	Cell Phone Battery Repairer	This user feels the product is better	JD.com
Puning Dental	Cell Phone Battery Repairer	This user feels the product is better	JD.com
Puning Dental	Cell Phone Battery Repairer	Use it right now and punch a laptop.	JD.com
Puning Dental	Cell Phone Battery Repairer	Good quality, very cheap, good object	JD.com
Puning Dental	Cell Phone Battery Repairer	Support the new technology, the goods have been received. The effect of the use of follow-up comments!	JD.com
Puning Dental	Cell Phone Battery Repairer	especially well, thanks to the merchant's stuff works especially well, this is especially good.[Smiles]This very satisfied. This is very, very good, this satisfaction to you to play, I good praise.	JD.com
Puning Dental	Cell Phone Battery Repairer	Thanks to the merchant's stuff works exceptionally well, really exceptionally well possesses no problems at all!	JD.com
Puning Dental	Cell Phone Battery Repairer	Please return the product if there is any problem with the quality	JD.com
Puning Dental	Cell Phone Battery Repairer	I'm using it now with good use over and over and over again.	JD.com
Puning Dental	Cell Phone Battery Repairer	Logistics is very powerful Quality is very good Immediately repurchase	JD.com
Puning Dental	Cell Phone Battery Repairer	This user feels the product is better	JD.com
Puning Dental	Cell Phone Battery Repairer	is very fast, the customer service attitude is very positive, and questions are answered in a timely manner! Overall very satisfied!	JD.com
Puning Dental	Cell Phone Battery Repairer	This user feels the product is better	JD.com
Puning Dental	Cell Phone Battery Repairer	inexpensive and good quality, fast delivery, customer service is very good too!	JD.com
Puning Dental	Cell Phone Battery Repairer	I'll wait to use it before I say how I feel about it.	JD.com

Puning Dental	Cell Phone Battery Repairer	beautifully packaged/product! It is a good adapter to improve the battery life of mobilephone/optimize cell phonesystem/clean up cell phone garbage and improverunning speed of cell phone!Trustworthy/purchaseuse! I wish the manufacturer a prosperous business! We wish you a prosperous business! Popularity is strong and prosperous![Kudos][Buy]][Good][Buy]][Clap][Hands[Ah Huang][Handshake][Tongue]Rose]	JD.com
Puning Dental	Cell Phone Battery Repairer	The item has been received, the courier is also very fast, the product quality is very good, the workmanship is very refined, is genuine, the price is also very cheap, and the description is exactly the same, I give five-star praise.	JD.com
Puning Dental	Cell Phone Battery	Comments	JD.com
Puning Dental	Cell Phone Battery	Well, I can't say yet. Let's just use it and try it out! Uh-huh!	JD.com
Puning Dental	Cell Phone Battery	It looks good.	JD.com
Puning Dental	Cell Phone Battery	Repair has received, use good re-purchase wish the store business prosperity!	JD.com
Puning Dental	Cell Phone Battery	It works well.	JD.com
Puning Dental	Cell Phone Battery Repairer	Conversion efficiency: good<br>Compatibility: strong<br>Security: good	JD.com
Puning Dental	Cell Phone Battery	Cell phone adapter received looks good used well in buy a	JD.com
Puning Dental	Cell Phone Battery	Received the item and it looks great. The style is also beautiful and okay.	JD.com
Puning Dental	Cell Phone Battery	Friends introduced to buy, on the cell phone pool repair remarkable, worth buying.	JD.com
Puning Dental	Cell Phone Battery	pretty good	JD.com
Puning Dental	Cell Phone Battery Repairer	The goods are very delicate, logistics is also fast, the business service is enthusiastic<br>Give five-star praise	JD.com
Puning Dental	Cell Phone Battery Repairer	Great price for what you get. Will buy the good stuff again in the future. Probably would work better at a higher price. Recommended purchase has been sent to the Poundland coterie.	JD.com
Puning Dental	Cell Phone Battery	I am very happy with your merchandise and the product is great.	JD.com
Puning Dental	Cell Phone Battery	Useful to repair fast charging good quality	JD.com
Puning Dental	Cell Phone Battery	products also fraudulent consumer business or unlicensed operation!	JD.com
Puning Dental	Cell Phone Battery Repairer	Authentic battery activator, cell phone repair is very easy, product quality is quite over the top, very satisfied with the special like, the price is cheap things are good, fine workmanship style is good, the service is thoughtful shipping fast, full-star praise a hundred praise!	JD.com
Puning Dental	Cell Phone Battery Repairer	This store's repairer is good, the cell phone battery activation rate is high, authentic product stuff is good, exquisite and beautiful high quality, fast shipping and thoughtful service, praise a full star high!	JD.com
Puning Dental	Cell Phone Battery Repairer	Cell phone repairer has received, genuine activator effect, product over the quality of high, something special good spend less, very satisfied with the super like, thoughtful service delivery fast, full of stars!	JD.com
Puning Dental	Cell Phone Battery Repairer	smooth, fine workmanship kind of beautiful, product quality is quite over the top, the price is cheap things are good, the service is enthusiastic and thoughtful, fast delivery full of stars high light!	JD.com
Puning Dental	Cell Phone Battery Repairer	Cheap and affordable<br>Stability: good<br>Will repurchase again	JD.com
Puning Dental	Cell Phone Battery	Quality can be, the courier boy is very enthusiastic please you can rest assured to buy!	JD.com
Puning Dental	Cell Phone Battery	This item is great quality and fast shipping.	JD.com
Puning Dental	Cell Phone Battery	classifier for objects in rows such as words	JD.com
Puning Dental	Cell Phone Battery Repairer	Received a cell phone battery repairer, the quality is quite good, exquisite packaging, good seller service attitude, logistics and express timely, all five-star praise. Wish merchants prosperous business.	JD.com
Puning Dental	Cell Phone Battery	Well packaged, inexpensive and easy to use.	JD.com

Puning Dental	Cell Phone Battery Repairer	Charging is average, charging is so slow	JD.com
Puning Dental	Cell Phone Battery Repairer	Very good satisfied next time come back[buy buy buy][kudos][kudos]	JD.com
Puning Dental	Cell Phone Battery Repairer	This user feels the product is better	JD.com
Puning Dental	Cell Phone Battery Repairer	Review after use.	JD.com
Puning Dental	Cell Phone Battery Repairer	Battery Repairer received, quite good, good quality, carefully packaged, intact, good business attitude, logistics and express timely, all five-star praise.	JD.com
Puning Dental	Cell Phone Battery Repairer	Plus this is similar to slow charging, it's good for battery protection	JD.com
Puning Dental	Cell Phone Battery Repairer	Great price and fast shipping! Packaging was great!	JD.com
Puning Dental	Cell Phone Battery Repairer	Works great and fits perfectly. It also plugs in.	JD.com
Puning Dental	Cell Phone Battery Repairer	The quality is pretty good.	JD.com
Puning Dental	Cell Phone Battery Repairer	The stuff works great, I'll buy another one later	JD.com
Puning Dental	Cell Phone Battery Repairer	IQ tax	JD.com
Puning Dental	Cell Phone Battery Repairer	This user feels the product is better	JD.com
Puning Dental	Cell Phone Battery Repairer	[Smiles][Skimming][Big Love]	JD.com
Puning Dental	Cell Phone Battery Repairer	Baby is very good, the logistics is very fast, the quality of workmanship is very good, cheaper than the entity store, trustworthy, like friends hurry to get down to it , , , , , , , , , , , , , , ,	JD.com
Puning Dental	Cell Phone Battery Repairer	False advertising, false products	JD.com
Puning Dental	Cell Phone Battery Repairer	I've got more memory with this. It's not laggy.	JD.com
Puning Dental	Cell Phone Battery Repairer	! ! ! ! ! ! ! ! ! ! !	JD.com
Puning Dental	Cell Phone Battery Repairer	This user feels the product is better	JD.com
Puning Dental	Cell Phone Battery Repairer	This user feels the product is better	JD.com
Puning Dental	Cell Phone Battery Repairer	Use it right now and punch a laptop.	JD.com
Puning Dental	Cell Phone Battery Repairer	Good quality, very cheap, good object	JD.com
Puning Dental	Cell Phone Battery Repairer	Support the new technology, the goods have been received. The effect of the use of follow-up comments!	JD.com
Puning Dental	Cell Phone Battery Repairer	especially well, thanks to the merchant's stuff works especially well, this is especially good.[Smiles]This very satisfied. This is very, very good, this satisfaction to you to play, I good praise.	JD.com
Puning Dental	Cell Phone Battery Repairer	Thanks to the merchant's stuff works exceptionally well, really exceptionally well possesses no problems at all!	JD.com
Puning Dental	Cell Phone Battery Repairer	Please return the product if there is any problem with the quality	JD.com
Puning Dental	Cell Phone Battery Repairer	I'm using it now with good use over and over and over again.	JD.com
Puning Dental	Cell Phone Battery Repairer	Logistics is very powerful Quality is very good Immediately repurchase	JD.com
Puning Dental	Cell Phone Battery Repairer	This user feels the product is better	JD.com
Puning Dental	Cell Phone Battery Repairer	is very fast, the customer service attitude is very positive, and questions are answered in a timely manner! Overall very satisfied!	JD.com
Puning Dental	Cell Phone Battery Repairer	This user feels the product is better	JD.com
Puning Dental	Cell Phone Battery Repairer	inexpensive and good quality, fast delivery, customer service is very good too!	JD.com
Puning Dental	Cell Phone Battery Repairer	I'll wait to use it before I say how I feel about it.	JD.com
Puning Dental	Cell Phone Battery Repairer	beautifully packaged/product! It is a good adapter to improve the battery life of mobilephone/optimize cell phonesystem/clean up cell phone garbage and improverunning speed of cell phone!Trustworthy/purchaseuse! I wish the manufacturer a prosperous business! We wish you a prosperous business! Popularity is strong and prosperous![Kudos][Buy]][Good][Buy]][Clap][Hands[Ah Huang][Handshake][Tongue]Rose]	JD.com

Puning Dental	Cell Phone Battery Repairer	The item has been received, the courier is also very fast, the product quality is very good, the workmanship is very refined, is genuine, the price is also very cheap, and the description is exactly the same, I give five-star praise.	JD.com
Puning Dental	Cell Phone Battery	Comments	JD.com
Puning Dental	Cell Phone Battery	Well, I can't say yet. Let's just use it and try it out! Uh-huh!	JD.com
Puning Dental	Cell Phone Battery	It looks good.	JD.com
Puning Dental	Cell Phone Battery	Repair has received, use good re-purchase wish the store business prosperity!	JD.com
Puning Dental	Cell Phone Battery	It works well.	JD.com
Puning Dental	Cell Phone Battery Repairer	Conversion efficiency: good<br>Compatibility: strong<br>Security: good	JD.com
Puning Dental	Cell Phone Battery	Cell phone adapter received looks good used well in buy a	JD.com
Puning Dental	Cell Phone Battery	Received the item and it looks great. The style is also beautiful and okay.	JD.com
Puning Dental	Cell Phone Battery	Friends introduced to buy, on the cell phone pool repair remarkable, worth buying.	JD.com
Puning Dental	Cell Phone Battery	pretty good	JD.com
Puning Dental	Cell Phone Battery Repairer	The goods are very delicate, logistics is also fast, the business service is enthusiastic<br>Give five-star praise	JD.com
Puning Dental	Cell Phone Battery Repairer	Great price for what you get. Will buy the good stuff again in the future. Probably would work better at a higher price. Recommended purchase has been sent to the Poundland coterie.	JD.com
Puning Dental	Cell Phone Battery	I am very happy with your merchandise and the product is great.	JD.com
Puning Dental	Cell Phone Battery	Useful to repair fast charging good quality	JD.com
Puning Dental	Cell Phone Battery	products also fraudulent consumer business or unlicensed operation!	JD.com
Puning Dental	Cell Phone Battery Repairer	Authentic battery activator, cell phone repair is very easy, product quality is quite over the top, very satisfied with the special like, the price is cheap things are good, fine workmanship style is good, the service is thoughtful shipping fast, full-star praise a hundred praise!	JD.com
Puning Dental	Cell Phone Battery Repairer	This store's repairer is good, the cell phone battery activation rate is high, authentic product stuff is good, exquisite and beautiful high quality, fast shipping and thoughtful service, praise a full star high!	JD.com
Puning Dental	Cell Phone Battery Repairer	Cell phone repairer has received, genuine activator effect, product over the quality of high, something special good spend less, very satisfied with the super like, thoughtful service delivery fast, full of stars!	JD.com
Puning Dental	Cell Phone Battery Repairer	smooth, fine workmanship kind of beautiful, product quality is quite over the top, the price is cheap things are good, the service is enthusiastic and thoughtful, fast delivery full of stars high light!	JD.com
Puning Dental	Cell Phone Battery Repairer	Cheap and affordable<br>Stability: good<br>Will repurchase again	JD.com
Puning Dental	Cell Phone Battery	Quality can be, the courier boy is very enthusiastic please you can rest assured to buy!	JD.com
Puning Dental	Cell Phone Battery	This item is great quality and fast shipping.	JD.com
Puning Dental	Cell Phone Battery	classifier for objects in rows such as words	JD.com
Puning Dental	Cell Phone Battery Repairer	Received a cell phone battery repairer, the quality is quite good, exquisite packaging, good seller service attitude, logistics and express timely, all five-star praise. Wish merchants prosperous business.	JD.com
Puning Dental	Cell Phone Battery	Well packaged, inexpensive and easy to use.	JD.com
Puning Dental	Cell Phone Battery	Charging is average, charging is so slow	JD.com
Puning Dental	Cell Phone Battery	Very good satisfied next time come back[buy buy buy][kudos][kudos]	JD.com
Puning Dental	Cell Phone Battery	This user feels the product is better	JD.com
Puning Dental	Cell Phone Battery	Review after use.	JD.com

Puning Dental	Cell Phone Battery Repairer	Battery Repairer received, quite good, good quality, carefully packaged, intact, good business attitude, logistics and express timely, all five-star praise.	JD.com
Puning Dental	Cell Phone Battery	Plus this is similar to slow charging, it's good for battery protection	JD.com
Puning Dental	Cell Phone Battery	Great price and fast shipping! Packaging was great!	JD.com
Puning Dental	Cell Phone Battery	Works great and fits perfectly. It also plugs in.	JD.com
Puning Dental	Cell Phone Battery	The quality is pretty good.	JD.com
Puning Dental	Cell Phone Battery	The stuff works great, I'll buy another one later	JD.com
Puning Dental	Cell Phone Battery	IQ tax	JD.com
Puning Dental	Cell Phone Battery	This user feels the product is better	JD.com
Puning Dental	Cell Phone Battery	[Smiles][Skimming][Big Love]	JD.com
Puning Dental	Cell Phone Battery Repairer	Baby is very good, the logistics is very fast, the quality of workmanship is very good, cheaper than the entity store, trustworthy, like friends hurry to get down to it , , , , , , , , , , , , , , ,	JD.com
Puning Dental	Cell Phone Battery	False advertising, false products	JD.com
Puning Dental	Cell Phone Battery	I've got more memory with this. It's not laggy.	JD.com
Puning Dental	Cell Phone Battery	! ! ! ! ! ! ! ! ! ! !	JD.com
Puning Dental	Cell Phone Battery	This user feels the product is better	JD.com
Puning Dental	Cell Phone Battery	This user feels the product is better	JD.com
Puning Dental	Cell Phone Battery	Use it right now and punch a laptop.	JD.com
Puning Dental	Cell Phone Battery	Good quality, very cheap, good object	JD.com
Puning Dental	Cell Phone Battery	Support the new technology, the goods have been received. The effect of the use of follow-up comments!	JD.com
Puning Dental	Cell Phone Battery Repairer	especially well, thanks to the merchant's stuff works especially well, this is especially good.[Smiles]This very satisfied. This is very, very good, this satisfaction to you to play, I good praise.	JD.com
Puning Dental	Cell Phone Battery	Thanks to the merchant's stuff works exceptionally well, really exceptionally well possesses no problems at all!	JD.com
Puning Dental	Cell Phone Battery	Please return the product if there is any problem with the quality	JD.com
Puning Dental	Cell Phone Battery	I'm using it now with good use over and over and over again.	JD.com
Puning Dental	Cell Phone Battery	Logistics is very powerful Quality is very good Immediately repurchase	JD.com
Puning Dental	Cell Phone Battery	This user feels the product is better	JD.com
Puning Dental	Cell Phone Battery Repairer	is very fast, the customer service attitude is very positive, and questions are answered in a timely manner! Overall very satisfied!	JD.com
Puning Dental	Cell Phone Battery	This user feels the product is better	JD.com
Puning Dental	Cell Phone Battery	inexpensive and good quality, fast delivery, customer service is very good too!	JD.com
Puning Dental	Cell Phone Battery	I'll wait to use it before I say how I feel about it.	JD.com
Puning Dental	Cell Phone Battery Repairer	beautifully packaged/product! It is a good adapter to improve the battery life of mobilephone/optimize cell phonesystem/clean up cell phone garbage and improverunning speed of cell phone!Trustworthy/purchaseuse! I wish the manufacturer a prosperous business! We wish you a prosperous business! Popularity is strong and prosperous![Kudos][Buy]][Good][Buy]][Clap][Hands[Ah Huang][Handshake][Tongue]Rose]	JD.com
Puning Dental	Cell Phone Battery Repairer	The item has been received, the courier is also very fast, the product quality is very good, the workmanship is very refined, is genuine, the price is also very cheap, and the description is exactly the same, I give five-star praise.	JD.com
Puning Dental	Cell Phone Battery	Comments	JD.com
Puning Dental	Cell Phone Battery	Well, I can't say yet. Let's just use it and try it out! Uh-huh!	JD.com

Puning Dental	Cell Phone Battery	It looks good.	JD.com
Puning Dental	Cell Phone Battery	Repair has received, use good re-purchase wish the store business prosperity!	JD.com
Puning Dental	Cell Phone Battery	It works well.	JD.com
Puning Dental	Cell Phone Battery Repairer	Conversion efficiency: good<br>Compatibility: strong<br>Security: good	JD.com
Puning Dental	Cell Phone Battery	Cell phone adapter received looks good used well in buy a	JD.com
Puning Dental	Cell Phone Battery	Received the item and it looks great. The style is also beautiful and okay.	JD.com
Puning Dental	Cell Phone Battery	Friends introduced to buy, on the cell phone pool repair remarkable, worth buying.	JD.com
Puning Dental	Cell Phone Battery	pretty good	JD.com
Puning Dental	Cell Phone Battery Repairer	The goods are very delicate, logistics is also fast, the business service is enthusiastic<br>Give five-star praise	JD.com
Puning Dental	Cell Phone Battery Repairer	Great price for what you get. Will buy the good stuff again in the future. Probably would work better at a higher price. Recommended purchase has been sent to the Poundland coterie.	JD.com
Puning Dental	Cell Phone Battery	I am very happy with your merchandise and the product is great.	JD.com
Puning Dental	Cell Phone Battery	Useful to repair fast charging good quality	JD.com
Puning Dental	Cell Phone Battery	products also fraudulent consumer business or unlicensed operation!	JD.com
Puning Dental	Cell Phone Battery Repairer	Authentic battery activator, cell phone repair is very easy, product quality is quite over the top, very satisfied with the special like, the price is cheap things are good, fine workmanship style is good, the service is thoughtful shipping fast, full-star praise a hundred praise!	JD.com
Puning Dental	Cell Phone Battery Repairer	This store's repairer is good, the cell phone battery activation rate is high, authentic product stuff is good, exquisite and beautiful high quality, fast shipping and thoughtful service, praise a full star high!	JD.com
Puning Dental	Cell Phone Battery Repairer	Cell phone repairer has received, genuine activator effect, product over the quality of high, something special good spend less, very satisfied with the super like, thoughtful service delivery fast, full of stars!	JD.com
Puning Dental	Cell Phone Battery Repairer	smooth, fine workmanship kind of beautiful, product quality is quite over the top, the price is cheap things are good, the service is enthusiastic and thoughtful, fast delivery full of stars high light!	JD.com
Puning Dental	Cell Phone Battery Repairer	Cheap and affordable<br>Stability: good<br>Will repurchase again	JD.com
Puning Dental	Cell Phone Battery	Quality can be, the courier boy is very enthusiastic please you can rest assured to buy!	JD.com
Puning Dental	Cell Phone Battery	This item is great quality and fast shipping.	JD.com
Puning Dental	Cell Phone Battery	classifier for objects in rows such as words	JD.com
Puning Dental	Cell Phone Battery Repairer	Received a cell phone battery repairer, the quality is quite good, exquisite packaging, good seller service attitude, logistics and express timely, all five-star praise. Wish merchants prosperous business.	JD.com
Puning Dental	Cell Phone Battery	Well packaged, inexpensive and easy to use.	JD.com
Puning Dental	Cell Phone Battery	Charging is average, charging is so slow	JD.com
Puning Dental	Cell Phone Battery	Very good satisfied next time come back[buy buy buy][kudos][kudos]	JD.com
Puning Dental	Cell Phone Battery	This user feels the product is better	JD.com
Puning Dental	Cell Phone Battery	Review after use.	JD.com
Puning Dental	Cell Phone Battery Repairer	Battery Repairer received, quite good, good quality, carefully packaged, intact, good business attitude, logistics and express timely, all five-star praise.	JD.com
Puning Dental	Cell Phone Battery	Plus this is similar to slow charging, it's good for battery protection	JD.com
Puning Dental	Cell Phone Battery	Great price and fast shipping! Packaging was great!	JD.com
Puning Dental	Cell Phone Battery	Works great and fits perfectly. It also plugs in.	JD.com

Puning Dental	Cell Phone Battery	The quality is pretty good.	JD.com
Puning Dental	Cell Phone Battery	The stuff works great, I'll buy another one later	JD.com
Puning Dental	Cell Phone Battery	IQ tax	JD.com
Puning Dental	Cell Phone Battery	This user feels the product is better	JD.com
Puning Dental	Cell Phone Battery	[Smiles][Skimming][Big Love]	JD.com
Puning Dental	Cell Phone Battery Repairer	Baby is very good, the logistics is very fast, the quality of workmanship is very good, cheaper than the entity store, trustworthy, like friends hurry to get down to it , , , , , , , , , , , , , , ,	JD.com
Puning Dental	Cell Phone Battery	False advertising, false products	JD.com
Puning Dental	Cell Phone Battery	I've got more memory with this. It's not laggy.	JD.com
Puning Dental	Cell Phone Battery	! ! ! ! ! ! ! ! ! ! !	JD.com
Puning Dental	Cell Phone Battery	This user feels the product is better	JD.com
Puning Dental	Cell Phone Battery	This user feels the product is better	JD.com
Puning Dental	Cell Phone Battery	Use it right now and punch a laptop.	JD.com
Puning Dental	Cell Phone Battery	Good quality, very cheap, good object	JD.com
Puning Dental	Cell Phone Battery	Support the new technology, the goods have been received. The effect of the use of follow-up comments!	JD.com
Puning Dental	Cell Phone Battery Repairer	especially well, thanks to the merchant's stuff works especially well, this is especially good.[Smiles]This very satisfied. This is very, very good, this satisfaction to you to play, I good praise.	JD.com
Puning Dental	Cell Phone Battery	Thanks to the merchant's stuff works exceptionally well, really exceptionally well possesses no problems at all!	JD.com
Puning Dental	Cell Phone Battery	Please return the product if there is any problem with the quality	JD.com
Puning Dental	Cell Phone Battery	I'm using it now with good use over and over and over again.	JD.com
Puning Dental	Cell Phone Battery	Logistics is very powerful Quality is very good Immediately repurchase	JD.com
Puning Dental	Cell Phone Battery	This user feels the product is better	JD.com
Puning Dental	Cell Phone Battery Repairer	is very fast, the customer service attitude is very positive, and questions are answered in a timely manner! Overall very satisfied!	JD.com
Puning Dental	Cell Phone Battery	This user feels the product is better	JD.com
Puning Dental	Cell Phone Battery	inexpensive and good quality, fast delivery, customer service is very good too!	JD.com
Puning Dental	Cell Phone Battery	I'll wait to use it before I say how I feel about it.	JD.com
Puning Dental	Cell Phone Battery Repairer	beautifully packaged/product! It is a good adapter to improve the battery life of mobilephone/optimize cell phonesystem/clean up cell phone garbage and improverunning speed of cell phone!Trustworthy/purchaseuse! I wish the manufacturer a prosperous business! We wish you a prosperous business! Popularity is strong and prosperous![Kudos][Buy]][Good][Buy]][Clap][Hands[Ah Huang][Handshake][Tongue]Rose]	JD.com
Puning Dental	Cell Phone Battery Repairer	The item has been received, the courier is also very fast, the product quality is very good, the workmanship is very refined, is genuine, the price is also very cheap, and the description is exactly the same, I give five-star praise.	JD.com
Puning Dental	Cell Phone Battery	Comments	JD.com
Puning Dental	Cell Phone Battery	Well, I can't say yet. Let's just use it and try it out! Uh-huh!	JD.com
Puning Dental	Cell Phone Battery	It looks good.	JD.com
Puning Dental	Cell Phone Battery	Repair has received, use good re-purchase wish the store business prosperity!	JD.com
Puning Dental	Cell Phone Battery	It works well.	JD.com
Puning Dental	Cell Phone Battery Repairer	Conversion efficiency: good<br>Compatibility: strong<br>Security: good	JD.com

Puning Dental	Cell Phone Battery	Cell phone adapter received looks good used well in buy a	JD.com
Puning Dental	Cell Phone Battery	Received the item and it looks great. The style is also beautiful and okay.	JD.com
Puning Dental	Cell Phone Battery	Friends introduced to buy, on the cell phone pool repair remarkable, worth buying.	JD.com
Puning Dental	Cell Phone Battery	pretty good	JD.com
Puning Dental	Cell Phone Battery Repairer	The goods are very delicate, logistics is also fast, the business service is enthusiastic<br>Give five-star praise	JD.com
Puning Dental	Cell Phone Battery Repairer	Great price for what you get. Will buy the good stuff again in the future. Probably would work better at a higher price. Recommended purchase has been sent to the Poundland coterie.	JD.com
Puning Dental	Cell Phone Battery	I am very happy with your merchandise and the product is great.	JD.com
Puning Dental	Cell Phone Battery	Useful to repair fast charging good quality	JD.com
Puning Dental	Cell Phone Battery	products also fraudulent consumer business or unlicensed operation!	JD.com
Puning Dental	Cell Phone Battery Repairer	Authentic battery activator, cell phone repair is very easy, product quality is quite over the top, very satisfied with the special like, the price is cheap things are good, fine workmanship style is good, the service is thoughtful shipping fast, full-star praise a hundred praise!	JD.com
Puning Dental	Cell Phone Battery Repairer	This store's repairer is good, the cell phone battery activation rate is high, authentic product stuff is good, exquisite and beautiful high quality, fast shipping and thoughtful service, praise a full star high!	JD.com
Puning Dental	Cell Phone Battery Repairer	Cell phone repairer has received, genuine activator effect, product over the quality of high, something special good spend less, very satisfied with the super like, thoughtful service delivery fast, full of stars!	JD.com
Puning Dental	Cell Phone Battery Repairer	smooth, fine workmanship kind of beautiful, product quality is quite over the top, the price is cheap things are good, the service is enthusiastic and thoughtful, fast delivery full of stars high light!	JD.com
Puning Dental	Cell Phone Battery Repairer	Cheap and affordable<br>Stability: good<br>Will repurchase again	JD.com
Puning Dental	Cell Phone Battery	Quality can be, the courier boy is very enthusiastic please you can rest assured to buy!	JD.com
Puning Dental	Cell Phone Battery	This item is great quality and fast shipping.	JD.com
Puning Dental	Cell Phone Battery	classifier for objects in rows such as words	JD.com
Puning Dental	Cell Phone Battery Repairer	Received a cell phone battery repairer, the quality is quite good, exquisite packaging, good seller service attitude, logistics and express timely, all five-star praise. Wish merchants prosperous business.	JD.com
Puning Dental	Cell Phone Battery	Well packaged, inexpensive and easy to use.	JD.com
Puning Dental	Cell Phone Battery	Charging is average, charging is so slow	JD.com
Puning Dental	Cell Phone Battery	Very good satisfied next time come back[buy buy buy][kudos][kudos]	JD.com
Puning Dental	Cell Phone Battery	This user feels the product is better	JD.com
Puning Dental	Cell Phone Battery	Review after use.	JD.com
Puning Dental	Cell Phone Battery Repairer	Battery Repairer received, quite good, good quality, carefully packaged, intact, good business attitude, logistics and express timely, all five-star praise.	JD.com
Puning Dental	Cell Phone Battery	Plus this is similar to slow charging, it's good for battery protection	JD.com
Puning Dental	Cell Phone Battery	Great price and fast shipping! Packaging was great!	JD.com
Puning Dental	Cell Phone Battery	Works great and fits perfectly. It also plugs in.	JD.com
Puning Dental	Cell Phone Battery	The quality is pretty good.	JD.com
Puning Dental	Cell Phone Battery	The stuff works great, I'll buy another one later	JD.com
Puning Dental	Cell Phone Battery	IQ tax	JD.com
Puning Dental	Cell Phone Battery	This user feels the product is better	JD.com
Puning Dental	Cell Phone Battery	[Smiles][Skimming][Big Love]	JD.com

Puning Dental	Cell Phone Battery Repairer	Baby is very good, the logistics is very fast, the quality of workmanship is very good, cheaper than the entity store, trustworthy, like friends hurry to get down to it , , , , , , , , , , , , , , ,	JD.com
Puning Dental	Cell Phone Battery	False advertising, false products	JD.com
Puning Dental	Cell Phone Battery	I've got more memory with this. It's not laggy.	JD.com
Puning Dental	Cell Phone Battery	! ! ! ! ! ! ! ! ! ! !	JD.com
Puning Dental	Cell Phone Battery	This user feels the product is better	JD.com
Puning Dental	Cell Phone Battery	This user feels the product is better	JD.com
Puning Dental	Cell Phone Battery	Use it right now and punch a laptop.	JD.com
Puning Dental	Cell Phone Battery	Good quality, very cheap, good object	JD.com
Puning Dental	Cell Phone Battery	Support the new technology, the goods have been received. The effect of the use of follow-up comments!	JD.com
Puning Dental	Cell Phone Battery Repairer	especially well, thanks to the merchant's stuff works especially well, this is especially good.[Smiles]This very satisfied. This is very, very good, this satisfaction to you to play, I good praise.	JD.com
Puning Dental	Cell Phone Battery	Thanks to the merchant's stuff works exceptionally well, really exceptionally well possesses no problems at all!	JD.com
Puning Dental	Cell Phone Battery	Please return the product if there is any problem with the quality	JD.com
Puning Dental	Cell Phone Battery	I'm using it now with good use over and over and over again.	JD.com
Puning Dental	Cell Phone Battery	Logistics is very powerful Quality is very good Immediately repurchase	JD.com
Puning Dental	Cell Phone Battery	This user feels the product is better	JD.com
Puning Dental	Cell Phone Battery Repairer	is very fast, the customer service attitude is very positive, and questions are answered in a timely manner! Overall very satisfied!	JD.com
Puning Dental	Cell Phone Battery	This user feels the product is better	JD.com
Puning Dental	Cell Phone Battery	inexpensive and good quality, fast delivery, customer service is very good too!	JD.com
Puning Dental	Cell Phone Battery	I'll wait to use it before I say how I feel about it.	JD.com
Puning Dental	Cell Phone Battery Repairer	beautifully packaged/product! It is a good adapter to improve the battery life of mobilephone/optimize cell phonesystem/clean up cell phone garbage and improverunning speed of cell phone!Trustworthy/purchaseuse! I wish the manufacturer a prosperous business! We wish you a prosperous business! Popularity is strong and prosperous![Kudos][Buy]][Good][Buy]][Clap][Hands[Ah Huang][Handshake][Tongue]Rose]	JD.com
Puning Dental	Cell Phone Battery Repairer	The item has been received, the courier is also very fast, the product quality is very good, the workmanship is very refined, is genuine, the price is also very cheap, and the description is exactly the same, I give five-star praise.	JD.com
Puning Dental	Cell Phone Battery	Comments	JD.com
Puning Dental	Cell Phone Battery	Well, I can't say yet. Let's just use it and try it out! Uh-huh!	JD.com
Puning Dental	Cell Phone Battery	It looks good.	JD.com
Puning Dental	Cell Phone Battery	Repair has received, use good re-purchase wish the store business prosperity!	JD.com
Puning Dental	Cell Phone Battery	It works well.	JD.com
Puning Dental	Cell Phone Battery Repairer	Conversion efficiency: good<br>Compatibility: strong<br>Security: good	JD.com
Puning Dental	Cell Phone Battery	Cell phone adapter received looks good used well in buy a|	JD.com
Puning Dental	Cell Phone Battery	Received the item and it looks great. The style is also beautiful and okay.	JD.com
Puning Dental	Cell Phone Battery	Friends introduced to buy, on the cell phone pool repair remarkable, worth buying.	JD.com
Puning Dental	Cell Phone Battery	pretty good	JD.com

Puning Dental	Cell Phone Battery Repairer	The goods are very delicate, logistics is also fast, the business service is enthusiastic<br>Give five-star praise	JD.com
Puning Dental	Cell Phone Battery Repairer	Great price for what you get. Will buy the good stuff again in the future. Probably would work better at a higher price. Recommended purchase has been sent to the Poundland coterie.	JD.com
Puning Dental	Cell Phone Battery	I am very happy with your merchandise and the product is great.	JD.com
Puning Dental	Cell Phone Battery	Useful to repair fast charging good quality	JD.com
Puning Dental	Cell Phone Battery	products also fraudulent consumer business or unlicensed operation!	JD.com
Puning Dental	Cell Phone Battery Repairer	Authentic battery activator, cell phone repair is very easy, product quality is quite over the top, very satisfied with the special like, the price is cheap things are good, fine workmanship style is good, the service is thoughtful shipping fast, full-star praise a hundred praise!	JD.com
Puning Dental	Cell Phone Battery Repairer	This store's repairer is good, the cell phone battery activation rate is high, authentic product stuff is good, exquisite and beautiful high quality, fast shipping and thoughtful service, praise a full star high!	JD.com
Puning Dental	Cell Phone Battery Repairer	Cell phone repairer has received, genuine activator effect, product over the quality of high, something special good spend less, very satisfied with the super like, thoughtful service delivery fast, full of stars!	JD.com
Puning Dental	Cell Phone Battery Repairer	smooth, fine workmanship kind of beautiful, product quality is quite over the top, the price is cheap things are good, the service is enthusiastic and thoughtful, fast delivery full of stars high light!	JD.com
Puning Dental	Cell Phone Battery Repairer	Cheap and affordable<br>Stability: good<br>Will repurchase again	JD.com
Puning Dental	Cell Phone Battery	Quality can be, the courier boy is very enthusiastic please you can rest assured to buy!	JD.com
Puning Dental	Cell Phone Battery	This item is great quality and fast shipping.	JD.com
Puning Dental	Cell Phone Battery	classifier for objects in rows such as words	JD.com
Puning Dental	Cell Phone Battery Repairer	Received a cell phone battery repairer, the quality is quite good, exquisite packaging, good seller service attitude, logistics and express timely, all five-star praise. Wish merchants prosperous business.	JD.com
Puning Dental	Cell Phone Battery	Well packaged, inexpensive and easy to use.	JD.com
Puning Dental	Cell Phone Battery	Charging is average, charging is so slow	JD.com
Puning Dental	Cell Phone Battery	Very good satisfied next time come back[buy buy buy][kudos][kudos]	JD.com
Puning Dental	Cell Phone Battery	This user feels the product is better	JD.com
Puning Dental	Cell Phone Battery	Review after use.	JD.com
Puning Dental	Cell Phone Battery Repairer	Battery Repairer received, quite good, good quality, carefully packaged, intact, good business attitude, logistics and express timely, all five-star praise.	JD.com
Puning Dental	Cell Phone Battery	Plus this is similar to slow charging, it's good for battery protection	JD.com
Puning Dental	Cell Phone Battery	Great price and fast shipping! Packaging was great!	JD.com
Puning Dental	Cell Phone Battery	Works great and fits perfectly. It also plugs in.	JD.com
Puning Dental	Cell Phone Battery	The quality is pretty good.	JD.com
Puning Dental	Cell Phone Battery	The stuff works great, I'll buy another one later	JD.com
Puning Dental	Cell Phone Battery	IQ tax	JD.com
Puning Dental	Cell Phone Battery	This user feels the product is better	JD.com
Puning Dental	Cell Phone Battery	[Smiles][Skimming][Big Love]	JD.com
Puning Dental	Cell Phone Battery Repairer	Baby is very good, the logistics is very fast, the quality of workmanship is very good, cheaper than the entity store, trustworthy, like friends hurry to get down to it<br>, , , , , , , , , , , , , , , ,	JD.com
Puning Dental	Cell Phone Battery	False advertising, false products	JD.com
Puning Dental	Cell Phone Battery	I've got more memory with this. It's not laggy.	JD.com

Puning Dental	Cell Phone Battery	！！！！！！！！！！！	JD.com
Puning Dental	Cell Phone Battery	This user feels the product is better	JD.com
Puning Dental	Cell Phone Battery	This user feels the product is better	JD.com
Puning Dental	Cell Phone Battery	Use it right now and punch a laptop.	JD.com
Puning Dental	Cell Phone Battery	Good quality, very cheap, good object	JD.com
Puning Dental	Cell Phone Battery	Support the new technology, the goods have been received. The effect of the use of follow-up comments!	JD.com
Puning Dental	Cell Phone Battery Repairer	especially well, thanks to the merchant's stuff works especially well, this is especially good.[Smiles] This very satisfied. This is very, very good, this satisfaction to you to play, I good praise.	JD.com
Puning Dental	Cell Phone Battery	Thanks to the merchant's stuff works exceptionally well, really exceptionally well possesses no problems at all!	JD.com
Puning Dental	Cell Phone Battery	Please return the product if there is any problem with the quality	JD.com
Puning Dental	Cell Phone Battery	I'm using it now with good use over and over and over again.	JD.com
Puning Dental	Cell Phone Battery	Logistics is very powerful Quality is very good Immediately repurchase	JD.com
Puning Dental	Cell Phone Battery	This user feels the product is better	JD.com
Puning Dental	Cell Phone Battery Repairer	is very fast, the customer service attitude is very positive, and questions are answered in a timely manner! Overall very satisfied!	JD.com
Puning Dental	Cell Phone Battery	This user feels the product is better	JD.com
Puning Dental	Cell Phone Battery	inexpensive and good quality, fast delivery, customer service is very good too!	JD.com
Puning Dental	Cell Phone Battery	I'll wait to use it before I say how I feel about it.	JD.com
Puning Dental	Cell Phone Battery Repairer	beautifully packaged/product! It is a good adapter to improve the battery life of mobilephone/optimize cell phonesystem/clean up cell phone garbage and improverunning speed of cell phone!Trustworthy/purchaseuse! I wish the manufacturer a prosperous business! We wish you a prosperous business! Popularity is strong and prosperous![Kudos][Buy]][Good][Buy]][Clap][Hands[Ah Huang][Handshake][Tongue]Rose]	JD.com
Puning Dental	Cell Phone Battery Repairer	The item has been received, the courier is also very fast, the product quality is very good, the workmanship is very refined, is genuine, the price is also very cheap, and the description is exactly the same, I give five-star praise.	JD.com
Puning Dental	Cell Phone Battery	Comments	JD.com
Puning Dental	Cell Phone Battery	Well, I can't say yet. Let's just use it and try it out! Uh-huh!	JD.com
Puning Dental	Cell Phone Battery	It looks good.	JD.com
Puning Dental	Cell Phone Battery	Repair has received, use good re-purchase wish the store business prosperity!	JD.com
Puning Dental	Cell Phone Battery	It works well.	JD.com
Puning Dental	Cell Phone Battery Repairer	Conversion efficiency: good<br>Compatibility: strong<br>Security: good	JD.com
Puning Dental	Cell Phone Battery	Cell phone adapter received looks good used well in buy a|	JD.com
Puning Dental	Cell Phone Battery	Received the item and it looks great. The style is also beautiful and okay.	JD.com
Puning Dental	Cell Phone Battery	Friends introduced to buy, on the cell phone pool repair remarkable, worth buying.	JD.com
Puning Dental	Cell Phone Battery	pretty good	JD.com
Puning Dental	Cell Phone Battery Repairer	The goods are very delicate, logistics is also fast, the business service is enthusiastic<br>Give five-star praise	JD.com
Puning Dental	Cell Phone Battery Repairer	Great price for what you get. Will buy the good stuff again in the future. Probably would work better at a higher price. Recommended purchase has been sent to the Poundland coterie.	JD.com
Puning Dental	Cell Phone Battery	I am very happy with your merchandise and the product is great.	JD.com
Puning Dental	Cell Phone Battery	Useful to repair fast charging good quality	JD.com

Puning Dental	Cell Phone Battery	products also fraudulent consumer business or unlicensed operation!	JD.com
Puning Dental	Cell Phone Battery Repairer	Authentic battery activator, cell phone repair is very easy, product quality is quite over the top, very satisfied with the special like, the price is cheap things are good, fine workmanship style is good, the service is thoughtful shipping fast, full-star praise a hundred praise!	JD.com
Puning Dental	Cell Phone Battery Repairer	This store's repairer is good, the cell phone battery activation rate is high, authentic product stuff is good, exquisite and beautiful high quality, fast shipping and thoughtful service, praise a full star high!	JD.com
Puning Dental	Cell Phone Battery Repairer	Cell phone repairer has received, genuine activator effect, product over the quality of high, something special good spend less, very satisfied with the super like, thoughtful service delivery fast, full of stars!	JD.com
Puning Dental	Cell Phone Battery Repairer	smooth, fine workmanship kind of beautiful, product quality is quite over the top, the price is cheap things are good, the service is enthusiastic and thoughtful, fast delivery full of stars high light!	JD.com
Puning Dental	Cell Phone Battery Repairer	Cheap and affordable<br>Stability: good<br>Will repurchase again	JD.com
Puning Dental	Cell Phone Battery	Quality can be, the courier boy is very enthusiastic please you can rest assured to buy!	JD.com
Puning Dental	Cell Phone Battery	This item is great quality and fast shipping.	JD.com
Puning Dental	Cell Phone Battery	classifier for objects in rows such as words	JD.com
Puning Dental	Cell Phone Battery Repairer	Received a cell phone battery repairer, the quality is quite good, exquisite packaging, good seller service attitude, logistics and express timely, all five-star praise. Wish merchants prosperous business.	JD.com
Puning Dental	Cell Phone Battery	Well packaged, inexpensive and easy to use.	JD.com
Puning Dental	Cell Phone Battery	Charging is average, charging is so slow	JD.com
Puning Dental	Cell Phone Battery	Very good satisfied next time come back[buy buy buy][kudos][kudos]	JD.com
Puning Dental	Cell Phone Battery	This user feels the product is better	JD.com
Puning Dental	Cell Phone Battery	Review after use.	JD.com
Puning Dental	Cell Phone Battery Repairer	Battery Repairer received, quite good, good quality, carefully packaged, intact, good business attitude, logistics and express timely, all five-star praise.	JD.com
Puning Dental	Cell Phone Battery	Plus this is similar to slow charging, it's good for battery protection	JD.com
Puning Dental	Cell Phone Battery	Great price and fast shipping! Packaging was great!	JD.com
Puning Dental	Cell Phone Battery	Works great and fits perfectly. It also plugs in.	JD.com
Puning Dental	Cell Phone Battery	The quality is pretty good.	JD.com
Puning Dental	Cell Phone Battery	The stuff works great, I'll buy another one later	JD.com
Puning Dental	Cell Phone Battery	IQ tax	JD.com
Puning Dental	Cell Phone Battery	This user feels the product is better	JD.com
Puning Dental	Cell Phone Battery	[Smiles][Skimming][Big Love]	JD.com
Puning Dental	Cell Phone Battery Repairer	Baby is very good, the logistics is very fast, the quality of workmanship is very good, cheaper than the entity store, trustworthy, like friends hurry to get down to it , , , , , , , , , , , , , , ,	JD.com
Puning Dental	Cell Phone Battery	False advertising, false products	JD.com
Puning Dental	Cell Phone Battery	I've got more memory with this. It's not laggy.	JD.com
Puning Dental	Cell Phone Battery	! ! ! ! ! ! ! ! ! ! !	JD.com
Puning Dental	Cell Phone Battery	This user feels the product is better	JD.com
Puning Dental	Cell Phone Battery	This user feels the product is better	JD.com
Puning Dental	Cell Phone Battery	Use it right now and punch a laptop.	JD.com
Puning Dental	Cell Phone Battery	Good quality, very cheap, good object	JD.com

Puning Dental	Cell Phone Battery	Support the new technology, the goods have been received. The effect of the use of follow-up comments!	JD.com
Puning Dental	Cell Phone Battery Repairer	especially well, thanks to the merchant's stuff works especially well, this is especially good.[Smiles] This very satisfied. This is very, very good, this satisfaction to you to play, I good praise.	JD.com
Puning Dental	Cell Phone Battery	Thanks to the merchant's stuff works exceptionally well, really exceptionally well possesses no problems at all!	JD.com
Puning Dental	Cell Phone Battery	Please return the product if there is any problem with the quality	JD.com
Puning Dental	Cell Phone Battery	I'm using it now with good use over and over and over again.	JD.com
Puning Dental	Cell Phone Battery	Logistics is very powerful Quality is very good Immediately repurchase	JD.com
Puning Dental	Cell Phone Battery	This user feels the product is better	JD.com
Puning Dental	Cell Phone Battery Repairer	is very fast, the customer service attitude is very positive, and questions are answered in a timely manner! Overall very satisfied!	JD.com
Puning Dental	Cell Phone Battery	This user feels the product is better	JD.com
Puning Dental	Cell Phone Battery	inexpensive and good quality, fast delivery, customer service is very good too!	JD.com
Puning Dental	Cell Phone Battery	I'll wait to use it before I say how I feel about it.	JD.com
Puning Dental	Cell Phone Battery Repairer	beautifully packaged/product! It is a good adapter to improve the battery life of mobilephone/optimize cell phonesystem/clean up cell phone garbage and improverunning speed of cell phone!Trustworthy/purchaseuse! I wish the manufacturer a prosperous business! We wish you a prosperous business! Popularity is strong and prosperous![Kudos][Buy]][Good][Buy]][Clap][Hands[Ah Huang][Handshake][Tongue]Rose]	JD.com
Puning Dental	Cell Phone Battery Repairer	The item has been received, the courier is also very fast, the product quality is very good, the workmanship is very refined, is genuine, the price is also very cheap, and the description is exactly the same, I give five-star praise.	JD.com
Puning Dental	Cell Phone Battery	Comments	JD.com
Puning Dental	Cell Phone Battery	Well, I can't say yet. Let's just use it and try it out! Uh-huh!	JD.com
Puning Dental	Cell Phone Battery	It looks good.	JD.com
Puning Dental	Cell Phone Battery	Repair has received, use good re-purchase wish the store business prosperity!	JD.com
Puning Dental	Cell Phone Battery	It works well.	JD.com
Puning Dental	Cell Phone Battery Repairer	Conversion efficiency: good<br>Compatibility: strong<br>Security: good	JD.com
Puning Dental	Cell Phone Battery	Cell phone adapter received looks good used well in buy a|	JD.com
Puning Dental	Cell Phone Battery	Received the item and it looks great. The style is also beautiful and okay.	JD.com
Puning Dental	Cell Phone Battery	Friends introduced to buy, on the cell phone pool repair remarkable, worth buying.	JD.com
Puning Dental	Cell Phone Battery	pretty good	JD.com
Puning Dental	Cell Phone Battery Repairer	The goods are very delicate, logistics is also fast, the business service is enthusiastic<br>Give five-star praise	JD.com
Puning Dental	Cell Phone Battery Repairer	Great price for what you get. Will buy the good stuff again in the future. Probably would work better at a higher price. Recommended purchase has been sent to the Poundland coterie.	JD.com
Puning Dental	Cell Phone Battery	I am very happy with your merchandise and the product is great.	JD.com
Puning Dental	Cell Phone Battery	Useful to repair fast charging good quality	JD.com
Puning Dental	Cell Phone Battery	products also fraudulent consumer business or unlicensed operation!	JD.com
Puning Dental	Cell Phone Battery Repairer	Authentic battery activator, cell phone repair is very easy, product quality is quite over the top, very satisfied with the special like, the price is cheap things are good, fine workmanship style is good, the service is thoughtful shipping fast, full-star praise a hundred praise!	JD.com

Puning Dental	Cell Phone Battery Repairer	This store's repairer is good, the cell phone battery activation rate is high, authentic product stuff is good, exquisite and beautiful high quality, fast shipping and thoughtful service, praise a full star high!	JD.com
Puning Dental	Cell Phone Battery Repairer	Cell phone repairer has received, genuine activator effect, product over the quality of high, something special good spend less, very satisfied with the super like, thoughtful service delivery fast, full of stars!	JD.com
Puning Dental	Cell Phone Battery Repairer	smooth, fine workmanship kind of beautiful, product quality is quite over the top, the price is cheap things are good, the service is enthusiastic and thoughtful, fast delivery full of stars high light!	JD.com
Puning Dental	Cell Phone Battery Repairer	Cheap and affordable<br>Stability: good<br>Will repurchase again	JD.com
Puning Dental	Cell Phone Battery	Quality can be, the courier boy is very enthusiastic please you can rest assured to buy!	JD.com
Puning Dental	Cell Phone Battery	This item is great quality and fast shipping.	JD.com
Puning Dental	Cell Phone Battery	classifier for objects in rows such as words	JD.com
Puning Dental	Cell Phone Battery Repairer	Received a cell phone battery repairer, the quality is quite good, exquisite packaging, good seller service attitude, logistics and express timely, all five-star praise. Wish merchants prosperous business.	JD.com
Puning Dental	Cell Phone Battery	Well packaged, inexpensive and easy to use.	JD.com
Puning Dental	Cell Phone Battery	Charging is average, charging is so slow	JD.com
Puning Dental	Cell Phone Battery	Very good satisfied next time come back[buy buy buy][kudos][kudos]	JD.com
Puning Dental	Cell Phone Battery	This user feels the product is better	JD.com
Puning Dental	Cell Phone Battery	Review after use.	JD.com
Puning Dental	Cell Phone Battery Repairer	Battery Repairer received, quite good, good quality, carefully packaged, intact, good business attitude, logistics and express timely, all five-star praise.	JD.com
Puning Dental	Cell Phone Battery	Plus this is similar to slow charging, it's good for battery protection	JD.com
Puning Dental	Cell Phone Battery	Great price and fast shipping! Packaging was great!	JD.com
Puning Dental	Cell Phone Battery	Works great and fits perfectly. It also plugs in.	JD.com
Puning Dental	Cell Phone Battery	The quality is pretty good.	JD.com
Puning Dental	Cell Phone Battery	The stuff works great, I'll buy another one later	JD.com
Puning Dental	Cell Phone Battery	IQ tax	JD.com
Puning Dental	Cell Phone Battery	This user feels the product is better	JD.com
Puning Dental	Cell Phone Battery	[Smiles][Skimming][Big Love]	JD.com
Puning Dental	Cell Phone Battery Repairer	Baby is very good, the logistics is very fast, the quality of workmanship is very good, cheaper than the entity store, trustworthy, like friends hurry to get down to it ,,,,,,,,,,,,,,,	JD.com
Puning Dental	Cell Phone Battery	False advertising, false products	JD.com
Puning Dental	Cell Phone Battery	I've got more memory with this. It's not laggy.	JD.com
Puning Dental	Cell Phone Battery	!!!!!!!!!!!	JD.com
Puning Dental	Cell Phone Battery	This user feels the product is better	JD.com
Puning Dental	Cell Phone Battery	This user feels the product is better	JD.com
Puning Dental	Cell Phone Battery	Use it right now and punch a laptop.	JD.com
Puning Dental	Cell Phone Battery	Good quality, very cheap, good object	JD.com
Puning Dental	Cell Phone Battery	Support the new technology, the goods have been received. The effect of the use of follow-up comments!	JD.com
Puning Dental	Cell Phone Battery Repairer	especially well, thanks to the merchant's stuff works especially well, this is especially good.[Smiles]This very satisfied. This is very, very good, this satisfaction to you to play, I good praise.	JD.com

Puning Dental	Cell Phone Battery	Thanks to the merchant's stuff works exceptionally well, really exceptionally well possesses no problems at all!	JD.com
Puning Dental	Cell Phone Battery	Please return the product if there is any problem with the quality	JD.com
Puning Dental	Cell Phone Battery	I'm using it now with good use over and over and over again.	JD.com
Puning Dental	Cell Phone Battery	Logistics is very powerful Quality is very good Immediately repurchase	JD.com
Puning Dental	Cell Phone Battery	This user feels the product is better	JD.com
Puning Dental	Cell Phone Battery Repairer	is very fast, the customer service attitude is very positive, and questions are answered in a timely manner! Overall very satisfied!	JD.com
Puning Dental	Cell Phone Battery	This user feels the product is better	JD.com
Puning Dental	Cell Phone Battery	inexpensive and good quality, fast delivery, customer service is very good too!	JD.com
Puning Dental	Cell Phone Battery	I'll wait to use it before I say how I feel about it.	JD.com
Puning Dental	Cell Phone Battery Repairer	beautifully packaged/product! It is a good adapter to improve the battery life of mobilephone/optimize cell phonesystem/clean up cell phone garbage and improverunning speed of cell phone!Trustworthy/purchaseuse! I wish the manufacturer a prosperous business! We wish you a prosperous business! Popularity is strong and prosperous![Kudos][Buy]][Good][Buy]][Clap][Hands[Ah Huang][Handshake][Tongue]Rose]	JD.com
Puning Dental	Cell Phone Battery Repairer	The item has been received, the courier is also very fast, the product quality is very good, the workmanship is very refined, is genuine, the price is also very cheap, and the description is exactly the same, I give five-star praise.	JD.com
Puning Dental	Cell Phone Battery	Comments	JD.com
Puning Dental	Cell Phone Battery	Well, I can't say yet. Let's just use it and try it out! Uh-huh!	JD.com
Puning Dental	Cell Phone Battery	It looks good.	JD.com
Puning Dental	Cell Phone Battery	Repair has received, use good re-purchase wish the store business prosperity!	JD.com
Puning Dental	Cell Phone Battery	It works well.	JD.com
Puning Dental	Cell Phone Battery Repairer	Conversion efficiency: good<br>Compatibility: strong<br>Security: good	JD.com
Puning Dental	Cell Phone Battery	Cell phone adapter received looks good used well in buy a	JD.com
Puning Dental	Cell Phone Battery	Received the item and it looks great. The style is also beautiful and okay.	JD.com
Puning Dental	Cell Phone Battery	Friends introduced to buy, on the cell phone pool repair remarkable, worth buying.	JD.com
Puning Dental	Cell Phone Battery	pretty good	JD.com
Puning Dental	Cell Phone Battery Repairer	The goods are very delicate, logistics is also fast, the business service is enthusiastic<br>Give five-star praise	JD.com
Puning Dental	Cell Phone Battery Repairer	Great price for what you get. Will buy the good stuff again in the future. Probably would work better at a higher price. Recommended purchase has been sent to the Poundland coterie.	JD.com
Puning Dental	Cell Phone Battery	I am very happy with your merchandise and the product is great.	JD.com
Puning Dental	Cell Phone Battery	Useful to repair fast charging good quality	JD.com
Puning Dental	Cell Phone Battery	products also fraudulent consumer business or unlicensed operation!	JD.com
Puning Dental	Cell Phone Battery Repairer	Authentic battery activator, cell phone repair is very easy, product quality is quite over the top, very satisfied with the special like, the price is cheap things are good, fine workmanship style is good, the service is thoughtful shipping fast, full-star praise a hundred praise!	JD.com
Puning Dental	Cell Phone Battery Repairer	This store's repairer is good, the cell phone battery activation rate is high, authentic product stuff is good, exquisite and beautiful high quality, fast shipping and thoughtful service, praise a full star high!	JD.com
Puning Dental	Cell Phone Battery Repairer	Cell phone repairer has received, genuine activator effect, product over the quality of high, something special good spend less, very satisfied with the super like, thoughtful service delivery fast, full of stars!	JD.com

Puning Dental	Cell Phone Battery Repairer	smooth, fine workmanship kind of beautiful, product quality is quite over the top, the price is cheap things are good, the service is enthusiastic and thoughtful, fast delivery full of stars high light!	JD.com
Puning Dental	Cell Phone Battery Repairer	Cheap and affordable<br>Stability: good<br>Will repurchase again	JD.com
Puning Dental	Cell Phone Battery	Quality can be, the courier boy is very enthusiastic please you can rest assured to buy!	JD.com
Puning Dental	Cell Phone Battery	This item is great quality and fast shipping.	JD.com
Puning Dental	Cell Phone Battery	classifier for objects in rows such as words	JD.com
Puning Dental	Cell Phone Battery Repairer	Received a cell phone battery repairer, the quality is quite good, exquisite packaging, good seller service attitude, logistics and express timely, all five-star praise. Wish merchants prosperous business.	JD.com
Puning Dental	Cell Phone Battery	Well packaged, inexpensive and easy to use.	JD.com
Puning Dental	Cell Phone Battery	Charging is average, charging is so slow	JD.com
Puning Dental	Cell Phone Battery	Very good satisfied next time come back[buy buy buy][kudos][kudos]	JD.com
Puning Dental	Cell Phone Battery	This user feels the product is better	JD.com
Puning Dental	Cell Phone Battery	Review after use.	JD.com
Puning Dental	Cell Phone Battery Repairer	Battery Repairer received, quite good, good quality, carefully packaged, intact, good business attitude, logistics and express timely, all five-star praise.	JD.com
Puning Dental	Cell Phone Battery	Plus this is similar to slow charging, it's good for battery protection	JD.com
Puning Dental	Cell Phone Battery	Great price and fast shipping! Packaging was great!	JD.com
Puning Dental	Cell Phone Battery	Works great and fits perfectly. It also plugs in.	JD.com
Puning Dental	Cell Phone Battery	The quality is pretty good.	JD.com
Puning Dental	Cell Phone Battery	The stuff works great, I'll buy another one later	JD.com
Puning Dental	Cell Phone Battery	IQ tax	JD.com
Puning Dental	Cell Phone Battery	This user feels the product is better	JD.com
Puning Dental	Cell Phone Battery	[Smiles][Skimming][Big Love]	JD.com
Puning Dental	Cell Phone Battery Repairer	Baby is very good, the logistics is very fast, the quality of workmanship is very good, cheaper than the entity store, trustworthy, like friends hurry to get down to it , , , , , , , , , , , , , , ,	JD.com
Puning Dental	Cell Phone Battery	False advertising, false products	JD.com
Puning Dental	Cell Phone Battery	I've got more memory with this. It's not laggy.	JD.com
Puning Dental	Cell Phone Battery	! ! ! ! ! ! ! ! ! ! !	JD.com
Puning Dental	Cell Phone Battery	This user feels the product is better	JD.com
Puning Dental	Cell Phone Battery	This user feels the product is better	JD.com
Puning Dental	Cell Phone Battery	Use it right now and punch a laptop.	JD.com
Puning Dental	Cell Phone Battery	Good quality, very cheap, good object	JD.com
Puning Dental	Cell Phone Battery	Support the new technology, the goods have been received. The effect of the use of follow-up comments!	JD.com
Puning Dental	Cell Phone Battery Repairer	especially well, thanks to the merchant's stuff works especially well, this is especially good.[Smiles]This very satisfied. This is very, very good, this satisfaction to you to play, I good praise.	JD.com
Puning Dental	Cell Phone Battery	Thanks to the merchant's stuff works exceptionally well, really exceptionally well possesses no problems at all!	JD.com
Puning Dental	Cell Phone Battery	Please return the product if there is any problem with the quality	JD.com
Puning Dental	Cell Phone Battery	I'm using it now with good use over and over and over again.	JD.com
Puning Dental	Cell Phone Battery	Logistics is very powerful Quality is very good Immediately repurchase	JD.com

Puning Dental	Cell Phone Battery Repairer	This user feels the product is better	JD.com
Puning Dental	Cell Phone Battery Repairer	is very fast, the customer service attitude is very positive, and questions are answered in a timely manner! Overall very satisfied!	JD.com
Puning Dental	Cell Phone Battery Repairer	This user feels the product is better	JD.com
Puning Dental	Cell Phone Battery Repairer	inexpensive and good quality, fast delivery, customer service is very good too!	JD.com
Puning Dental	Cell Phone Battery Repairer	I'll wait to use it before I say how I feel about it.	JD.com
Puning Dental	Cell Phone Battery Repairer	beautifully packaged/product! It is a good adapter to improve the battery life of mobilephone/optimize cell phonesystem/clean up cell phone garbage and improverunning speed of cell phone!Trustworthy/purchaseuse! I wish the manufacturer a prosperous business! We wish you a prosperous business! Popularity is strong and prosperous![Kudos][Buy]][Good][Buy]][Clap][Hands[Ah Huang][Handshake][Tongue]Rose]	JD.com
Puning Dental	Cell Phone Battery Repairer	The item has been received, the courier is also very fast, the product quality is very good, the workmanship is very refined, is genuine, the price is also very cheap, and the description is exactly the same, I give five-star praise.	JD.com
Puning Dental	Cell Phone Battery Repairer	Comments	JD.com
Puning Dental	Cell Phone Battery Repairer	Well, I can't say yet. Let's just use it and try it out! Uh-huh!	JD.com
Puning Dental	Cell Phone Battery Repairer	It looks good.	JD.com
Puning Dental	Cell Phone Battery Repairer	Repair has received, use good re-purchase wish the store business prosperity!	JD.com
Puning Dental	Cell Phone Battery Repairer	It works well.	JD.com
Puning Dental	Cell Phone Battery Repairer	Conversion efficiency: good<br>Compatibility: strong<br>Security: good	JD.com
Puning Dental	Cell Phone Battery Repairer	Cell phone adapter received looks good used well in buy a	JD.com
Puning Dental	Cell Phone Battery Repairer	Received the item and it looks great. The style is also beautiful and okay.	JD.com
Puning Dental	Cell Phone Battery Repairer	Friends introduced to buy, on the cell phone pool repair remarkable, worth buying.	JD.com
Puning Dental	Cell Phone Battery Repairer	pretty good	JD.com
Puning Dental	Cell Phone Battery Repairer	The goods are very delicate, logistics is also fast, the business service is enthusiastic<br>Give five-star praise	JD.com
Puning Dental	Cell Phone Battery Repairer	Great price for what you get. Will buy the good stuff again in the future. Probably would work better at a higher price. Recommended purchase has been sent to the Poundland coterie.	JD.com
Puning Dental	Cell Phone Battery Repairer	I am very happy with your merchandise and the product is great.	JD.com
Puning Dental	Cell Phone Battery Repairer	Useful to repair fast charging good quality	JD.com
Puning Dental	Cell Phone Battery Repairer	products also fraudulent consumer business or unlicensed operation!	JD.com
Puning Dental	Cell Phone Battery Repairer	Authentic battery activator, cell phone repair is very easy, product quality is quite over the top, very satisfied with the special like, the price is cheap things are good, fine workmanship style is good, the service is thoughtful shipping fast, full-star praise a hundred praise!	JD.com
Puning Dental	Cell Phone Battery Repairer	This store's repairer is good, the cell phone battery activation rate is high, authentic product stuff is good, exquisite and beautiful high quality, fast shipping and thoughtful service, praise a full star high!	JD.com
Puning Dental	Cell Phone Battery Repairer	Cell phone repairer has received, genuine activator effect, product over the quality of high, something special good spend less, very satisfied with the super like, thoughtful service delivery fast, full of stars!	JD.com
Puning Dental	Cell Phone Battery Repairer	smooth, fine workmanship kind of beautiful, product quality is quite over the top, the price is cheap things are good, the service is enthusiastic and thoughtful, fast delivery full of stars high light!	JD.com
Puning Dental	Cell Phone Battery Repairer	Cheap and affordable<br>Stability: good<br>Will repurchase again	JD.com
Puning Dental	Cell Phone Battery Repairer	Quality can be, the courier boy is very enthusiastic please you can rest assured to buy!	JD.com

Puning Dental	Cell Phone Battery	This item is great quality and fast shipping.	JD.com
Puning Dental	Cell Phone Battery	classifier for objects in rows such as words	JD.com
Puning Dental	Cell Phone Battery Repairer	Received a cell phone battery repairer, the quality is quite good, exquisite packaging, good seller service attitude, logistics and express timely, all five-star praise. Wish merchants prosperous business.	JD.com
Puning Dental	Cell Phone Battery	Well packaged, inexpensive and easy to use.	JD.com
Puning Dental	Cell Phone Battery	Charging is average, charging is so slow	JD.com
Puning Dental	Cell Phone Battery	Very good satisfied next time come back[buy buy buy][kudos][kudos]	JD.com
Puning Dental	Cell Phone Battery	This user feels the product is better	JD.com
Puning Dental	Cell Phone Battery	Review after use.	JD.com
Puning Dental	Cell Phone Battery Repairer	Battery Repairer received, quite good, good quality, carefully packaged, intact, good business attitude, logistics and express timely, all five-star praise.	JD.com
Puning Dental	Cell Phone Battery	Plus this is similar to slow charging, it's good for battery protection	JD.com
Puning Dental	Cell Phone Battery	Great price and fast shipping! Packaging was great!	JD.com
Puning Dental	Cell Phone Battery	Works great and fits perfectly. It also plugs in.	JD.com
Puning Dental	Cell Phone Battery	The quality is pretty good.	JD.com
Puning Dental	Cell Phone Battery	The stuff works great, I'll buy another one later	JD.com
Puning Dental	Cell Phone Battery	IQ tax	JD.com
Puning Dental	Cell Phone Battery	This user feels the product is better	JD.com
Puning Dental	Cell Phone Battery	[Smiles][Skimming][Big Love]	JD.com
Puning Dental	Cell Phone Battery Repairer	Baby is very good, the logistics is very fast, the quality of workmanship is very good, cheaper than the entity store, trustworthy, like friends hurry to get down to it , , , , , , , , , , , , , , ,	JD.com
Puning Dental	Cell Phone Battery	False advertising, false products	JD.com
Puning Dental	Cell Phone Battery	I've got more memory with this. It's not laggy.	JD.com
Puning Dental	Cell Phone Battery	! ! ! ! ! ! ! ! ! ! !	JD.com
Puning Dental	Cell Phone Battery	This user feels the product is better	JD.com
Puning Dental	Cell Phone Battery	This user feels the product is better	JD.com
Puning Dental	Cell Phone Battery	Use it right now and punch a laptop.	JD.com
Puning Dental	Cell Phone Battery	Good quality, very cheap, good object	JD.com
Puning Dental	Cell Phone Battery	Support the new technology, the goods have been received. The effect of the use of follow-up comments!	JD.com
Puning Dental	Cell Phone Battery Repairer	especially well, thanks to the merchant's stuff works especially well, this is especially good.[Smiles]This very satisfied. This is very, very good, this satisfaction to you to play, I good praise.	JD.com
Puning Dental	Cell Phone Battery	Thanks to the merchant's stuff works exceptionally well, really exceptionally well possesses no problems at all!	JD.com
Puning Dental	Cell Phone Battery	Please return the product if there is any problem with the quality	JD.com
Puning Dental	Cell Phone Battery	I'm using it now with good use over and over and over again.	JD.com
Puning Dental	Cell Phone Battery	Logistics is very powerful Quality is very good Immediately repurchase	JD.com
Puning Dental	Cell Phone Battery	This user feels the product is better	JD.com
Puning Dental	Cell Phone Battery Repairer	is very fast, the customer service attitude is very positive, and questions are answered in a timely manner! Overall very satisfied!	JD.com
Puning Dental	Cell Phone Battery	This user feels the product is better	JD.com
Puning Dental	Cell Phone Battery	inexpensive and good quality, fast delivery, customer service is very good too!	JD.com

Puning Dental	Cell Phone Battery	I'll wait to use it before I say how I feel about it.	JD.com
Puning Dental	Cell Phone Battery Repairer	beautifully packaged/product! It is a good adapter to improve the battery life of mobilephone/optimize cell phonesystem/clean up cell phone garbage and improverunning speed of cell phone!Trustworthy/purchaseuse! I wish the manufacturer a prosperous business! We wish you a prosperous business! Popularity is strong and prosperous![Kudos][Buy]][Good][Buy]][Clap][Hands[Ah Huang][Handshake][Tongue]Rose]	JD.com
Puning Dental	Cell Phone Battery Repairer	The item has been received, the courier is also very fast, the product quality is very good, the workmanship is very refined, is genuine, the price is also very cheap, and the description is exactly the same, I give five-star praise.	JD.com
Puning Dental	Cell Phone Battery	Comments	JD.com
Puning Dental	Cell Phone Battery	Well, I can't say yet. Let's just use it and try it out! Uh-huh!	JD.com
Puning Dental	Cell Phone Battery	It looks good.	JD.com
Puning Dental	Cell Phone Battery	Repair has received, use good re-purchase wish the store business prosperity!	JD.com
Puning Dental	Cell Phone Battery	It works well.	JD.com
Puning Dental	Cell Phone Battery Repairer	Conversion efficiency: good<br>Compatibility: strong<br>Security: good	JD.com
Puning Dental	Cell Phone Battery	Cell phone adapter received looks good used well in buy a	JD.com
Puning Dental	Cell Phone Battery	Received the item and it looks great. The style is also beautiful and okay.	JD.com
Puning Dental	Cell Phone Battery	Friends introduced to buy, on the cell phone pool repair remarkable, worth buying.	JD.com
Puning Dental	Cell Phone Battery	pretty good	JD.com
Puning Dental	Cell Phone Battery Repairer	The goods are very delicate, logistics is also fast, the business service is enthusiastic<br>Give five-star praise	JD.com
Puning Dental	Cell Phone Battery Repairer	Great price for what you get. Will buy the good stuff again in the future. Probably would work better at a higher price. Recommended purchase has been sent to the Poundland coterie.	JD.com
Puning Dental	Cell Phone Battery	I am very happy with your merchandise and the product is great.	JD.com
Puning Dental	Cell Phone Battery	Useful to repair fast charging good quality	JD.com
Puning Dental	Cell Phone Battery	products also fraudulent consumer business or unlicensed operation!	JD.com
Puning Dental	Cell Phone Battery Repairer	Authentic battery activator, cell phone repair is very easy, product quality is quite over the top, very satisfied with the special like, the price is cheap things are good, fine workmanship style is good, the service is thoughtful shipping fast, full-star praise a hundred praise!	JD.com
Puning Dental	Cell Phone Battery Repairer	This store's repairer is good, the cell phone battery activation rate is high, authentic product stuff is good, exquisite and beautiful high quality, fast shipping and thoughtful service, praise a full star high!	JD.com
Puning Dental	Cell Phone Battery Repairer	Cell phone repairer has received, genuine activator effect, product over the quality of high, something special good spend less, very satisfied with the super like, thoughtful service delivery fast, full of stars!	JD.com
Puning Dental	Cell Phone Battery Repairer	smooth, fine workmanship kind of beautiful, product quality is quite over the top, the price is cheap things are good, the service is enthusiastic and thoughtful, fast delivery full of stars high light!	JD.com
Puning Dental	Cell Phone Battery Repairer	Cheap and affordable<br>Stability: good<br>Will repurchase again	JD.com
Puning Dental	Cell Phone Battery	Quality can be, the courier boy is very enthusiastic please you can rest assured to buy!	JD.com
Puning Dental	Cell Phone Battery	This item is great quality and fast shipping.	JD.com
Puning Dental	Cell Phone Battery	classifier for objects in rows such as words	JD.com
Puning Dental	Cell Phone Battery Repairer	Received a cell phone battery repairer, the quality is quite good, exquisite packaging, good seller service attitude, logistics and express timely, all five-star praise. Wish merchants prosperous business.	JD.com

Puning Dental	Cell Phone Battery	Well packaged, inexpensive and easy to use.	JD.com
Puning Dental	Cell Phone Battery	Charging is average, charging is so slow	JD.com
Puning Dental	Cell Phone Battery	Very good satisfied next time come back[buy buy buy][kudos][kudos]	JD.com
Puning Dental	Cell Phone Battery	This user feels the product is better	JD.com
Puning Dental	Cell Phone Battery	Review after use.	JD.com
Puning Dental	Cell Phone Battery Repairer	Battery Repairer received, quite good, good quality, carefully packaged, intact, good business attitude, logistics and express timely, all five-star praise.	JD.com
Puning Dental	Cell Phone Battery	Plus this is similar to slow charging, it's good for battery protection	JD.com
Puning Dental	Cell Phone Battery	Great price and fast shipping! Packaging was great!	JD.com
Puning Dental	Cell Phone Battery	Works great and fits perfectly. It also plugs in.	JD.com
Puning Dental	Cell Phone Battery	The quality is pretty good.	JD.com
Puning Dental	Cell Phone Battery	The stuff works great, I'll buy another one later	JD.com
Puning Dental	Cell Phone Battery	IQ tax	JD.com
Puning Dental	Cell Phone Battery	This user feels the product is better	JD.com
Puning Dental	Cell Phone Battery	[Smiles][Skimming][Big Love]	JD.com
Puning Dental	Cell Phone Battery Repairer	Baby is very good, the logistics is very fast, the quality of workmanship is very good, cheaper than the entity store, trustworthy, like friends hurry to get down to it , , , , , , , , , , , , , , ,	JD.com
Puning Dental	Cell Phone Battery	False advertising, false products	JD.com
Puning Dental	Cell Phone Battery	I've got more memory with this. It's not laggy.	JD.com
Puning Dental	Cell Phone Battery	! ! ! ! ! ! ! ! ! ! !	JD.com
Puning Dental	Cell Phone Battery	This user feels the product is better	JD.com
Puning Dental	Cell Phone Battery	This user feels the product is better	JD.com
Puning Dental	Cell Phone Battery	Use it right now and punch a laptop.	JD.com
Puning Dental	Cell Phone Battery	Good quality, very cheap, good object	JD.com
Puning Dental	Cell Phone Battery	Support the new technology, the goods have been received. The effect of the use of follow-up comments!	JD.com
Puning Dental	Cell Phone Battery Repairer	especially well, thanks to the merchant's stuff works especially well, this is especially good.[Smiles]This very satisfied. This is very, very good, this satisfaction to you to play, I good praise.	JD.com
Puning Dental	Cell Phone Battery	Thanks to the merchant's stuff works exceptionally well, really exceptionally well possesses no problems at all!	JD.com
Puning Dental	Cell Phone Battery	Please return the product if there is any problem with the quality	JD.com
Puning Dental	Cell Phone Battery	I'm using it now with good use over and over and over again.	JD.com
Puning Dental	Cell Phone Battery	Logistics is very powerful Quality is very good Immediately repurchase	JD.com
Puning Dental	Cell Phone Battery	This user feels the product is better	JD.com
Puning Dental	Cell Phone Battery Repairer	is very fast, the customer service attitude is very positive, and questions are answered in a timely manner! Overall very satisfied!	JD.com
Puning Dental	Cell Phone Battery	This user feels the product is better	JD.com
Puning Dental	Cell Phone Battery	inexpensive and good quality, fast delivery, customer service is very good too!	JD.com
Puning Dental	Cell Phone Battery	I'll wait to use it before I say how I feel about it.	JD.com

Puning Dental	Cell Phone Battery Repairer	beautifully packaged/product! It is a good adapter to improve the battery life of mobilephone/optimize cell phonesystem/clean up cell phone garbage and improverunning speed of cell phone! Trustworthy/purchaseuse! I wish the manufacturer a prosperous business! We wish you a prosperous business! Popularity is strong and prosperous! [Kudos][Buy]][Good][Buy]][Clap][Hands[Ah Huang][Handshake][Tongue]Rose]	JD.com
Puning Dental	Cell Phone Battery Repairer	The item has been received, the courier is also very fast, the product quality is very good, the workmanship is very refined, is genuine, the price is also very cheap, and the description is exactly the same, I give five-star praise.	JD.com
Puning Dental	Cell Phone Battery	Comments	JD.com
Puning Dental	Cell Phone Battery	Well, I can't say yet. Let's just use it and try it out! Uh-huh!	JD.com
Puning Dental	Cell Phone Battery	It looks good.	JD.com
Puning Dental	Cell Phone Battery	Repair has received, use good re-purchase wish the store business prosperity!	JD.com
Puning Dental	Cell Phone Battery	It works well.	JD.com
Puning Dental	Cell Phone Battery Repairer	Conversion efficiency: good<br>Compatibility: strong<br>Security: good	JD.com
Puning Dental	Cell Phone Battery	Cell phone adapter received looks good used well in buy a|	JD.com
Puning Dental	Cell Phone Battery	Received the item and it looks great. The style is also beautiful and okay.	JD.com
Puning Dental	Cell Phone Battery	Friends introduced to buy, on the cell phone pool repair remarkable, worth buying.	JD.com
Puning Dental	Cell Phone Battery	pretty good	JD.com
Puning Dental	Cell Phone Battery Repairer	The goods are very delicate, logistics is also fast, the business service is enthusiastic<br>Give five-star praise	JD.com
Puning Dental	Cell Phone Battery Repairer	Great price for what you get. Will buy the good stuff again in the future. Probably would work better at a higher price. Recommended purchase has been sent to the Poundland coterie.	JD.com
Puning Dental	Cell Phone Battery	I am very happy with your merchandise and the product is great.	JD.com
Puning Dental	Cell Phone Battery	Useful to repair fast charging good quality	JD.com
Puning Dental	Cell Phone Battery	products also fraudulent consumer business or unlicensed operation!	JD.com
Puning Dental	Cell Phone Battery Repairer	Authentic battery activator, cell phone repair is very easy, product quality is quite over the top, very satisfied with the special like, the price is cheap things are good, fine workmanship style is good, the service is thoughtful shipping fast, full-star praise a hundred praise!	JD.com
Puning Dental	Cell Phone Battery Repairer	This store's repairer is good, the cell phone battery activation rate is high, authentic product stuff is good, exquisite and beautiful high quality, fast shipping and thoughtful service, praise a full star high!	JD.com
Puning Dental	Cell Phone Battery Repairer	Cell phone repairer has received, genuine activator effect, product over the quality of high, something special good spend less, very satisfied with the super like, thoughtful service delivery fast, full of stars!	JD.com
Puning Dental	Cell Phone Battery Repairer	smooth, fine workmanship kind of beautiful, product quality is quite over the top, the price is cheap things are good, the service is enthusiastic and thoughtful, fast delivery full of stars high light!	JD.com
Puning Dental	Cell Phone Battery Repairer	Cheap and affordable<br>Stability: good<br>Will repurchase again	JD.com
Puning Dental	Cell Phone Battery	Quality can be, the courier boy is very enthusiastic please you can rest assured to buy!	JD.com
Puning Dental	Cell Phone Battery	This item is great quality and fast shipping.	JD.com
Puning Dental	Cell Phone Battery	classifier for objects in rows such as words	JD.com
Puning Dental	Cell Phone Battery Repairer	Received a cell phone battery repairer, the quality is quite good, exquisite packaging, good seller service attitude, logistics and express timely, all five-star praise. Wish merchants prosperous business.	JD.com
Puning Dental	Cell Phone Battery	Well packaged, inexpensive and easy to use.	JD.com

Puning Dental	Cell Phone Battery	Charging is average, charging is so slow	JD.com
Puning Dental	Cell Phone Battery	Very good satisfied next time come back[buy buy buy][kudos][kudos]	JD.com
Puning Dental	Cell Phone Battery	This user feels the product is better	JD.com
Puning Dental	Cell Phone Battery	Review after use.	JD.com
Puning Dental	Cell Phone Battery Repairer	Battery Repairer received, quite good, good quality, carefully packaged, intact, good business attitude, logistics and express timely, all five-star praise.	JD.com
Puning Dental	Cell Phone Battery	Plus this is similar to slow charging, it's good for battery protection	JD.com
Puning Dental	Cell Phone Battery	Great price and fast shipping! Packaging was great!	JD.com
Puning Dental	Cell Phone Battery	Works great and fits perfectly. It also plugs in.	JD.com
Puning Dental	Cell Phone Battery	The quality is pretty good.	JD.com
Puning Dental	Cell Phone Battery	The stuff works great, I'll buy another one later	JD.com
Puning Dental	Cell Phone Battery	IQ tax	JD.com
Puning Dental	Cell Phone Battery	This user feels the product is better	JD.com
Puning Dental	Cell Phone Battery	[Smiles][Skimming][Big Love]	JD.com
Puning Dental	Cell Phone Battery Repairer	Baby is very good, the logistics is very fast, the quality of workmanship is very good, cheaper than the entity store, trustworthy, like friends hurry to get down to it , , , , , , , , , , , , , , ,	JD.com
Puning Dental	Cell Phone Battery	False advertising, false products	JD.com
Puning Dental	Cell Phone Battery	I've got more memory with this. It's not laggy.	JD.com
Puning Dental	Cell Phone Battery	! ! ! ! ! ! ! ! ! ! !	JD.com
Puning Dental	Cell Phone Battery	This user feels the product is better	JD.com
Puning Dental	Cell Phone Battery	This user feels the product is better	JD.com
Puning Dental	Cell Phone Battery	Use it right now and punch a laptop.	JD.com
Puning Dental	Cell Phone Battery	Good quality, very cheap, good object	JD.com
Puning Dental	Cell Phone Battery	Support the new technology, the goods have been received. The effect of the use of follow-up comments!	JD.com
Puning Dental	Cell Phone Battery Repairer	especially well, thanks to the merchant's stuff works especially well, this is especially good.[Smiles]This very satisfied. This is very, very good, this satisfaction to you to play, I good praise.	JD.com
Puning Dental	Cell Phone Battery	Thanks to the merchant's stuff works exceptionally well, really exceptionally well possesses no problems at all!	JD.com
Puning Dental	Cell Phone Battery	Please return the product if there is any problem with the quality	JD.com
Puning Dental	Cell Phone Battery	I'm using it now with good use over and over and over again.	JD.com
Puning Dental	Cell Phone Battery	Logistics is very powerful Quality is very good Immediately repurchase	JD.com
Puning Dental	Cell Phone Battery	This user feels the product is better	JD.com
Puning Dental	Cell Phone Battery Repairer	is very fast, the customer service attitude is very positive, and questions are answered in a timely manner! Overall very satisfied!	JD.com
Puning Dental	Cell Phone Battery	This user feels the product is better	JD.com
Puning Dental	Cell Phone Battery	inexpensive and good quality, fast delivery, customer service is very good too!	JD.com
Puning Dental	Cell Phone Battery	I'll wait to use it before I say how I feel about it.	JD.com
Puning Dental	Cell Phone Battery Repairer	beautifully packaged/product! It is a good adapter to improve the battery life of mobilephone/optimize cell phonesystem/clean up cell phone garbage and improverunning speed of cell phone!Trustworthy/purchaseuse! I wish the manufacturer a prosperous business! We wish you a prosperous business! Popularity is strong and prosperous![Kudos][Buy]][Good][Buy]][Clap][Hands[Ah Huang][Handshake][Tongue]Rose]	JD.com

Puning Dental	Cell Phone Battery Repairer	The item has been received, the courier is also very fast, the product quality is very good, the workmanship is very refined, is genuine, the price is also very cheap, and the description is exactly the same, I give five-star praise.	JD.com
Puning Dental	Cell Phone Battery	Comments	JD.com
Puning Dental	Cell Phone Battery	Well, I can't say yet. Let's just use it and try it out! Uh-huh!	JD.com
Puning Dental	Cell Phone Battery	It looks good.	JD.com
Puning Dental	Cell Phone Battery	Repair has received, use good re-purchase wish the store business prosperity!	JD.com
Puning Dental	Cell Phone Battery	It works well.	JD.com
Puning Dental	Cell Phone Battery Repairer	Conversion efficiency: good<br>Compatibility: strong<br>Security: good	JD.com
Puning Dental	Cell Phone Battery	Cell phone adapter received looks good used well in buy a	JD.com
Puning Dental	Cell Phone Battery	Received the item and it looks great. The style is also beautiful and okay.	JD.com
Puning Dental	Cell Phone Battery	Friends introduced to buy, on the cell phone pool repair remarkable, worth buying.	JD.com
Puning Dental	Cell Phone Battery	pretty good	JD.com
Puning Dental	Cell Phone Battery Repairer	The goods are very delicate, logistics is also fast, the business service is enthusiastic<br>Give five-star praise	JD.com
Puning Dental	Cell Phone Battery Repairer	Great price for what you get. Will buy the good stuff again in the future. Probably would work better at a higher price. Recommended purchase has been sent to the Poundland coterie.	JD.com
Puning Dental	Cell Phone Battery	I am very happy with your merchandise and the product is great.	JD.com
Puning Dental	Cell Phone Battery	Useful to repair fast charging good quality	JD.com
Puning Dental	Cell Phone Battery	products also fraudulent consumer business or unlicensed operation!	JD.com
Puning Dental	Cell Phone Battery Repairer	Authentic battery activator, cell phone repair is very easy, product quality is quite over the top, very satisfied with the special like, the price is cheap things are good, fine workmanship style is good, the service is thoughtful shipping fast, full-star praise a hundred praise!	JD.com
Puning Dental	Cell Phone Battery Repairer	This store's repairer is good, the cell phone battery activation rate is high, authentic product stuff is good, exquisite and beautiful high quality, fast shipping and thoughtful service, praise a full star high!	JD.com
Puning Dental	Cell Phone Battery Repairer	Cell phone repairer has received, genuine activator effect, product over the quality of high, something special good spend less, very satisfied with the super like, thoughtful service delivery fast, full of stars!	JD.com
Puning Dental	Cell Phone Battery Repairer	smooth, fine workmanship kind of beautiful, product quality is quite over the top, the price is cheap things are good, the service is enthusiastic and thoughtful, fast delivery full of stars high light!	JD.com
Puning Dental	Cell Phone Battery Repairer	Cheap and affordable<br>Stability: good<br>Will repurchase again	JD.com
Puning Dental	Cell Phone Battery	Quality can be, the courier boy is very enthusiastic please you can rest assured to buy!	JD.com
Puning Dental	Cell Phone Battery	This item is great quality and fast shipping.	JD.com
Puning Dental	Cell Phone Battery	classifier for objects in rows such as words	JD.com
Puning Dental	Cell Phone Battery Repairer	Received a cell phone battery repairer, the quality is quite good, exquisite packaging, good seller service attitude, logistics and express timely, all five-star praise. Wish merchants prosperous business.	JD.com
Puning Dental	Cell Phone Battery	Well packaged, inexpensive and easy to use.	JD.com
Puning Dental	Cell Phone Battery	Charging is average, charging is so slow	JD.com
Puning Dental	Cell Phone Battery	Very good satisfied next time come back[buy buy buy][kudos][kudos]	JD.com
Puning Dental	Cell Phone Battery	This user feels the product is better	JD.com
Puning Dental	Cell Phone Battery	Review after use.	JD.com

Puning Dental	Cell Phone Battery Repairer	Battery Repairer received, quite good, good quality, carefully packaged, intact, good business attitude, logistics and express timely, all five-star praise.	JD.com
Puning Dental	Cell Phone Battery	Plus this is similar to slow charging, it's good for battery protection	JD.com
Puning Dental	Cell Phone Battery	Great price and fast shipping! Packaging was great!	JD.com
Puning Dental	Cell Phone Battery	Works great and fits perfectly. It also plugs in.	JD.com
Puning Dental	Cell Phone Battery	The quality is pretty good.	JD.com
Puning Dental	Cell Phone Battery	The stuff works great, I'll buy another one later	JD.com
Puning Dental	Cell Phone Battery	IQ tax	JD.com
Puning Dental	Cell Phone Battery	This user feels the product is better	JD.com
Puning Dental	Cell Phone Battery	[Smiles][Skimming][Big Love]	JD.com
Puning Dental	Cell Phone Battery Repairer	Baby is very good, the logistics is very fast, the quality of workmanship is very good, cheaper than the entity store, trustworthy, like friends hurry to get down to it , , , , , , , , , , , , , , ,	JD.com
Puning Dental	Cell Phone Battery	False advertising, false products	JD.com
Puning Dental	Cell Phone Battery	I've got more memory with this. It's not laggy.	JD.com
Puning Dental	Cell Phone Battery	! ! ! ! ! ! ! ! ! ! !	JD.com
Puning Dental	Cell Phone Battery	This user feels the product is better	JD.com
Puning Dental	Cell Phone Battery	This user feels the product is better	JD.com
Puning Dental	Cell Phone Battery	Use it right now and punch a laptop.	JD.com
Puning Dental	Cell Phone Battery	Good quality, very cheap, good object	JD.com
Puning Dental	Cell Phone Battery	Support the new technology, the goods have been received. The effect of the use of follow-up comments!	JD.com
Puning Dental	Cell Phone Battery Repairer	especially well, thanks to the merchant's stuff works especially well, this is especially good.[Smiles] This very satisfied. This is very, very good, this satisfaction to you to play, I good praise.	JD.com
Puning Dental	Cell Phone Battery	Thanks to the merchant's stuff works exceptionally well, really exceptionally well possesses no problems at all!	JD.com
Puning Dental	Cell Phone Battery	Please return the product if there is any problem with the quality	JD.com
Puning Dental	Cell Phone Battery	I'm using it now with good use over and over and over again.	JD.com
Puning Dental	Cell Phone Battery	Logistics is very powerful Quality is very good Immediately repurchase	JD.com
Puning Dental	Cell Phone Battery	This user feels the product is better	JD.com
Puning Dental	Cell Phone Battery Repairer	is very fast, the customer service attitude is very positive, and questions are answered in a timely manner! Overall very satisfied!	JD.com
Puning Dental	Cell Phone Battery	This user feels the product is better	JD.com
Puning Dental	Cell Phone Battery	inexpensive and good quality, fast delivery, customer service is very good too!	JD.com
Puning Dental	Cell Phone Battery	I'll wait to use it before I say how I feel about it.	JD.com
Puning Dental	Cell Phone Battery Repairer	beautifully packaged/product! It is a good adapter to improve the battery life of mobilephone/optimize cell phonesystem/clean up cell phone garbage and improverunning speed of cell phone!Trustworthy/purchaseuse! I wish the manufacturer a prosperous business! We wish you a prosperous business! Popularity is strong and prosperous![Kudos][Buy]][Good][Buy]][Clap][Hands[Ah Huang][Handshake][Tongue]Rose]	JD.com
Puning Dental	Cell Phone Battery Repairer	The item has been received, the courier is also very fast, the product quality is very good, the workmanship is very refined, is genuine, the price is also very cheap, and the description is exactly the same, I give five-star praise.	JD.com
Puning Dental	Cell Phone Battery	Comments	JD.com
Puning Dental	Cell Phone Battery	Well, I can't say yet. Let's just use it and try it out! Uh-huh!	JD.com

Puning Dental	Cell Phone Battery	It looks good.	JD.com
Puning Dental	Cell Phone Battery	Repair has received, use good re-purchase wish the store business prosperity!	JD.com
Puning Dental	Cell Phone Battery	It works well.	JD.com
Puning Dental	Cell Phone Battery Repairer	Conversion efficiency: good<br>Compatibility: strong<br>Security: good	JD.com
Puning Dental	Cell Phone Battery	Cell phone adapter received looks good used well in buy a|	JD.com
Puning Dental	Cell Phone Battery	Received the item and it looks great. The style is also beautiful and okay.	JD.com
Puning Dental	Cell Phone Battery	Friends introduced to buy, on the cell phone pool repair remarkable, worth buying.	JD.com
Puning Dental	Cell Phone Battery	pretty good	JD.com
Puning Dental	Cell Phone Battery Repairer	The goods are very delicate, logistics is also fast, the business service is enthusiastic<br>Give five-star praise	JD.com
Puning Dental	Cell Phone Battery Repairer	Great price for what you get. Will buy the good stuff again in the future. Probably would work better at a higher price. Recommended purchase has been sent to the Poundland coterie.	JD.com
Puning Dental	Cell Phone Battery	I am very happy with your merchandise and the product is great.	JD.com
Puning Dental	Cell Phone Battery	Useful to repair fast charging good quality	JD.com
Puning Dental	Cell Phone Battery	products also fraudulent consumer business or unlicensed operation!	JD.com
Puning Dental	Cell Phone Battery Repairer	Authentic battery activator, cell phone repair is very easy, product quality is quite over the top, very satisfied with the special like, the price is cheap things are good, fine workmanship style is good, the service is thoughtful shipping fast, full-star praise a hundred praise!	JD.com
Puning Dental	Cell Phone Battery Repairer	This store's repairer is good, the cell phone battery activation rate is high, authentic product stuff is good, exquisite and beautiful high quality, fast shipping and thoughtful service, praise a full star high!	JD.com
Puning Dental	Cell Phone Battery Repairer	Cell phone repairer has received, genuine activator effect, product over the quality of high, something special good spend less, very satisfied with the super like, thoughtful service delivery fast, full of stars!	JD.com
Puning Dental	Cell Phone Battery Repairer	smooth, fine workmanship kind of beautiful, product quality is quite over the top, the price is cheap things are good, the service is enthusiastic and thoughtful, fast delivery full of stars high light!	JD.com
Puning Dental	Cell Phone Battery Repairer	Cheap and affordable<br>Stability: good<br>Will repurchase again	JD.com
Puning Dental	Cell Phone Battery	Quality can be, the courier boy is very enthusiastic please you can rest assured to buy!	JD.com
Puning Dental	Cell Phone Battery	This item is great quality and fast shipping.	JD.com
Puning Dental	Cell Phone Battery	classifier for objects in rows such as words	JD.com
Puning Dental	Cell Phone Battery Repairer	Received a cell phone battery repairer, the quality is quite good, exquisite packaging, good seller service attitude, logistics and express timely, all five-star praise. Wish merchants prosperous business.	JD.com
Puning Dental	Cell Phone Battery	Well packaged, inexpensive and easy to use.	JD.com
Puning Dental	Cell Phone Battery	Charging is average, charging is so slow	JD.com
Puning Dental	Cell Phone Battery	Very good satisfied next time come back[buy buy buy][kudos][kudos]	JD.com
Puning Dental	Cell Phone Battery	This user feels the product is better	JD.com
Puning Dental	Cell Phone Battery	Review after use.	JD.com
Puning Dental	Cell Phone Battery Repairer	Battery Repairer received, quite good, good quality, carefully packaged, intact, good business attitude, logistics and express timely, all five-star praise.	JD.com
Puning Dental	Cell Phone Battery	Plus this is similar to slow charging, it's good for battery protection	JD.com
Puning Dental	Cell Phone Battery	Great price and fast shipping! Packaging was great!	JD.com
Puning Dental	Cell Phone Battery	Works great and fits perfectly. It also plugs in.	JD.com

Puning Dental	Cell Phone Battery	The quality is pretty good.	JD.com
Puning Dental	Cell Phone Battery	The stuff works great, I'll buy another one later	JD.com
Puning Dental	Cell Phone Battery	IQ tax	JD.com
Puning Dental	Cell Phone Battery	This user feels the product is better	JD.com
Puning Dental	Cell Phone Battery	[Smiles][Skimming][Big Love]	JD.com
Puning Dental	Cell Phone Battery Repairer	Baby is very good, the logistics is very fast, the quality of workmanship is very good, cheaper than the entity store, trustworthy, like friends hurry to get down to it , , , , , , , , , , , , , , ,	JD.com
Puning Dental	Cell Phone Battery	False advertising, false products	JD.com
Puning Dental	Cell Phone Battery	I've got more memory with this. It's not laggy.	JD.com
Puning Dental	Cell Phone Battery	! ! ! ! ! ! ! ! ! ! !	JD.com
Puning Dental	Cell Phone Battery	This user feels the product is better	JD.com
Puning Dental	Cell Phone Battery	This user feels the product is better	JD.com
Puning Dental	Cell Phone Battery	Use it right now and punch a laptop.	JD.com
Puning Dental	Cell Phone Battery	Good quality, very cheap, good object	JD.com
Puning Dental	Cell Phone Battery	Support the new technology, the goods have been received. The effect of the use of follow-up comments!	JD.com
Puning Dental	Cell Phone Battery Repairer	especially well, thanks to the merchant's stuff works especially well, this is especially good.[Smiles]This very satisfied. This is very, very good, this satisfaction to you to play, I good praise.	JD.com
Puning Dental	Cell Phone Battery	Thanks to the merchant's stuff works exceptionally well, really exceptionally well possesses no problems at all!	JD.com
Puning Dental	Cell Phone Battery	Please return the product if there is any problem with the quality	JD.com
Puning Dental	Cell Phone Battery	I'm using it now with good use over and over and over again.	JD.com
Puning Dental	Cell Phone Battery	Logistics is very powerful Quality is very good Immediately repurchase	JD.com
Puning Dental	Cell Phone Battery	This user feels the product is better	JD.com
Puning Dental	Cell Phone Battery Repairer	is very fast, the customer service attitude is very positive, and questions are answered in a timely manner! Overall very satisfied!	JD.com
Puning Dental	Cell Phone Battery	This user feels the product is better	JD.com
Puning Dental	Cell Phone Battery	inexpensive and good quality, fast delivery, customer service is very good too!	JD.com
Puning Dental	Cell Phone Battery	I'll wait to use it before I say how I feel about it.	JD.com
Puning Dental	Cell Phone Battery Repairer	beautifully packaged/product! It is a good adapter to improve the battery life of mobilephone/optimize cell phonesystem/clean up cell phone garbage and improverunning speed of cell phone!Trustworthy/purchaseuse! I wish the manufacturer a prosperous business! We wish you a prosperous business! Popularity is strong and prosperous![Kudos][Buy]][Good][Buy]][Clap][Hands[Ah Huang][Handshake][Tongue]Rose]	JD.com
Puning Dental	Cell Phone Battery Repairer	The item has been received, the courier is also very fast, the product quality is very good, the workmanship is very refined, is genuine, the price is also very cheap, and the description is exactly the same, I give five-star praise.	JD.com
Puning Dental	Cell Phone Battery	Comments	JD.com
Puning Dental	Cell Phone Battery	Well, I can't say yet. Let's just use it and try it out! Uh-huh!	JD.com
Puning Dental	Cell Phone Battery	It looks good.	JD.com
Puning Dental	Cell Phone Battery	Repair has received, use good re-purchase wish the store business prosperity!	JD.com
Puning Dental	Cell Phone Battery	It works well.	JD.com
Puning Dental	Cell Phone Battery Repairer	Conversion efficiency: good<br>Compatibility: strong<br>Security: good	JD.com

Puning Dental	Cell Phone Battery	Cell phone adapter received looks good used well in buy a	JD.com
Puning Dental	Cell Phone Battery	Received the item and it looks great. The style is also beautiful and okay.	JD.com
Puning Dental	Cell Phone Battery	Friends introduced to buy, on the cell phone pool repair remarkable, worth buying.	JD.com
Puning Dental	Cell Phone Battery	pretty good	JD.com
Puning Dental	Cell Phone Battery Repairer	The goods are very delicate, logistics is also fast, the business service is enthusiastic<br>Give five-star praise	JD.com
Puning Dental	Cell Phone Battery Repairer	Great price for what you get. Will buy the good stuff again in the future. Probably would work better at a higher price. Recommended purchase has been sent to the Poundland coterie.	JD.com
Puning Dental	Cell Phone Battery	I am very happy with your merchandise and the product is great.	JD.com
Puning Dental	Cell Phone Battery	Useful to repair fast charging good quality	JD.com
Puning Dental	Cell Phone Battery	products also fraudulent consumer business or unlicensed operation!	JD.com
Puning Dental	Cell Phone Battery Repairer	Authentic battery activator, cell phone repair is very easy, product quality is quite over the top, very satisfied with the special like, the price is cheap things are good, fine workmanship style is good, the service is thoughtful shipping fast, full-star praise a hundred praise!	JD.com
Puning Dental	Cell Phone Battery Repairer	This store's repairer is good, the cell phone battery activation rate is high, authentic product stuff is good, exquisite and beautiful high quality, fast shipping and thoughtful service, praise a full star high!	JD.com
Puning Dental	Cell Phone Battery Repairer	Cell phone repairer has received, genuine activator effect, product over the quality of high, something special good spend less, very satisfied with the super like, thoughtful service delivery fast, full of stars!	JD.com
Puning Dental	Cell Phone Battery Repairer	smooth, fine workmanship kind of beautiful, product quality is quite over the top, the price is cheap things are good, the service is enthusiastic and thoughtful, fast delivery full of stars high light!	JD.com
Puning Dental	Cell Phone Battery Repairer	Cheap and affordable<br>Stability: good<br>Will repurchase again	JD.com
Puning Dental	Cell Phone Battery	Quality can be, the courier boy is very enthusiastic please you can rest assured to buy!	JD.com
Puning Dental	Cell Phone Battery	This item is great quality and fast shipping.	JD.com
Puning Dental	Cell Phone Battery	classifier for objects in rows such as words	JD.com
Puning Dental	Cell Phone Battery Repairer	Received a cell phone battery repairer, the quality is quite good, exquisite packaging, good seller service attitude, logistics and express timely, all five-star praise. Wish merchants prosperous business.	JD.com
Puning Dental	Cell Phone Battery	Well packaged, inexpensive and easy to use.	JD.com
Puning Dental	Cell Phone Battery	Charging is average, charging is so slow	JD.com
Puning Dental	Cell Phone Battery	Very good satisfied next time come back[buy buy buy][kudos][kudos]	JD.com
Puning Dental	Cell Phone Battery	This user feels the product is better	JD.com
Puning Dental	Cell Phone Battery	Review after use.	JD.com
Puning Dental	Cell Phone Battery Repairer	Battery Repairer received, quite good, good quality, carefully packaged, intact, good business attitude, logistics and express timely, all five-star praise.	JD.com
Puning Dental	Cell Phone Battery	Plus this is similar to slow charging, it's good for battery protection	JD.com
Puning Dental	Cell Phone Battery	Great price and fast shipping! Packaging was great!	JD.com
Puning Dental	Cell Phone Battery	Works great and fits perfectly. It also plugs in.	JD.com
Puning Dental	Cell Phone Battery	The quality is pretty good.	JD.com
Puning Dental	Cell Phone Battery	The stuff works great, I'll buy another one later	JD.com
Puning Dental	Cell Phone Battery	IQ tax	JD.com
Puning Dental	Cell Phone Battery	This user feels the product is better	JD.com
Puning Dental	Cell Phone Battery	[Smiles][Skimming][Big Love]	JD.com

Puning Dental	Cell Phone Battery Repairer	Baby is very good, the logistics is very fast, the quality of workmanship is very good, cheaper than the entity store, trustworthy, like friends hurry to get down to it , , , , , , , , , , , , , , ,	JD.com
Puning Dental	Cell Phone Battery	False advertising, false products	JD.com
Puning Dental	Cell Phone Battery	I've got more memory with this. It's not laggy.	JD.com
Puning Dental	Cell Phone Battery	! ! ! ! ! ! ! ! ! ! !	JD.com
Puning Dental	Cell Phone Battery	This user feels the product is better	JD.com
Puning Dental	Cell Phone Battery	This user feels the product is better	JD.com
Puning Dental	Cell Phone Battery	Use it right now and punch a laptop.	JD.com
Puning Dental	Cell Phone Battery	Good quality, very cheap, good object	JD.com
Puning Dental	Cell Phone Battery	Support the new technology, the goods have been received. The effect of the use of follow-up comments!	JD.com
Puning Dental	Cell Phone Battery Repairer	especially well, thanks to the merchant's stuff works especially well, this is especially good.[Smiles]This very satisfied. This is very, very good, this satisfaction to you to play, I good praise.	JD.com
Puning Dental	Cell Phone Battery	Thanks to the merchant's stuff works exceptionally well, really exceptionally well possesses no problems at all!	JD.com
Puning Dental	Cell Phone Battery	Please return the product if there is any problem with the quality	JD.com
Puning Dental	Cell Phone Battery	I'm using it now with good use over and over and over again.	JD.com
Puning Dental	Cell Phone Battery	Logistics is very powerful Quality is very good Immediately repurchase	JD.com
Puning Dental	Cell Phone Battery	This user feels the product is better	JD.com
Puning Dental	Cell Phone Battery Repairer	is very fast, the customer service attitude is very positive, and questions are answered in a timely manner! Overall very satisfied!	JD.com
Puning Dental	Cell Phone Battery	This user feels the product is better	JD.com
Puning Dental	Cell Phone Battery	inexpensive and good quality, fast delivery, customer service is very good too!	JD.com
Puning Dental	Cell Phone Battery	I'll wait to use it before I say how I feel about it.	JD.com
Puning Dental	Cell Phone Battery Repairer	beautifully packaged/product! It is a good adapter to improve the battery life of mobilephone/optimize cell phonesystem/clean up cell phone garbage and improverunning speed of cell phone!Trustworthy/purchaseuse! I wish the manufacturer a prosperous business! We wish you a prosperous business! Popularity is strong and prosperous![Kudos][Buy]][Good][Buy]][Clap][Hands[Ah Huang][Handshake][Tongue]Rose]	JD.com
Puning Dental	Cell Phone Battery Repairer	The item has been received, the courier is also very fast, the product quality is very good, the workmanship is very refined, is genuine, the price is also very cheap, and the description is exactly the same, I give five-star praise.	JD.com
Puning Dental	Cell Phone Battery	Comments	JD.com
Puning Dental	Cell Phone Battery	Well, I can't say yet. Let's just use it and try it out! Uh-huh!	JD.com
Puning Dental	Cell Phone Battery	It looks good.	JD.com
Puning Dental	Cell Phone Battery	Repair has received, use good re-purchase wish the store business prosperity!	JD.com
Puning Dental	Cell Phone Battery	It works well.	JD.com
Puning Dental	Cell Phone Battery Repairer	Conversion efficiency: good<br>Compatibility: strong<br>Security: good	JD.com
Puning Dental	Cell Phone Battery	Cell phone adapter received looks good used well in buy a|	JD.com
Puning Dental	Cell Phone Battery	Received the item and it looks great. The style is also beautiful and okay.	JD.com
Puning Dental	Cell Phone Battery	Friends introduced to buy, on the cell phone pool repair remarkable, worth buying.	JD.com
Puning Dental	Cell Phone Battery	pretty good	JD.com

Puning Dental	Cell Phone Battery Repairer	The goods are very delicate, logistics is also fast, the business service is enthusiastic Give five-star praise	JD.com
Puning Dental	Cell Phone Battery Repairer	Great price for what you get. Will buy the good stuff again in the future. Probably would work better at a higher price. Recommended purchase has been sent to the Poundland coterie.	JD.com
Puning Dental	Cell Phone Battery	I am very happy with your merchandise and the product is great.	JD.com
Puning Dental	Cell Phone Battery	Useful to repair fast charging good quality	JD.com
Puning Dental	Cell Phone Battery	products also fraudulent consumer business or unlicensed operation!	JD.com
Puning Dental	Cell Phone Battery Repairer	Authentic battery activator, cell phone repair is very easy, product quality is quite over the top, very satisfied with the special like, the price is cheap things are good, fine workmanship style is good, the service is thoughtful shipping fast, full-star praise a hundred praise!	JD.com
Puning Dental	Cell Phone Battery Repairer	This store's repairer is good, the cell phone battery activation rate is high, authentic product stuff is good, exquisite and beautiful high quality, fast shipping and thoughtful service, praise a full star high!	JD.com
Puning Dental	Cell Phone Battery Repairer	Cell phone repairer has received, genuine activator effect, product over the quality of high, something special good spend less, very satisfied with the super like, thoughtful service delivery fast, full of stars!	JD.com
Puning Dental	Cell Phone Battery Repairer	smooth, fine workmanship kind of beautiful, product quality is quite over the top, the price is cheap things are good, the service is enthusiastic and thoughtful, fast delivery full of stars high light!	JD.com
Puning Dental	Cell Phone Battery Repairer	Cheap and affordable Stability: good Will repurchase again	JD.com
Puning Dental	Cell Phone Battery	Quality can be, the courier boy is very enthusiastic please you can rest assured to buy!	JD.com
Puning Dental	Cell Phone Battery	This item is great quality and fast shipping.	JD.com
Puning Dental	Cell Phone Battery	classifier for objects in rows such as words	JD.com
Puning Dental	Cell Phone Battery Repairer	Received a cell phone battery repairer, the quality is quite good, exquisite packaging, good seller service attitude, logistics and express timely, all five-star praise. Wish merchants prosperous business.	JD.com
Puning Dental	Cell Phone Battery	Well packaged, inexpensive and easy to use.	JD.com
Puning Dental	Cell Phone Battery	Charging is average, charging is so slow	JD.com
Puning Dental	Cell Phone Battery	Very good satisfied next time come back[buy buy buy][kudos][kudos]	JD.com
Puning Dental	Cell Phone Battery	This user feels the product is better	JD.com
Puning Dental	Cell Phone Battery	Review after use.	JD.com
Puning Dental	Cell Phone Battery Repairer	Battery Repairer received, quite good, good quality, carefully packaged, intact, good business attitude, logistics and express timely, all five-star praise.	JD.com
Puning Dental	Cell Phone Battery	Plus this is similar to slow charging, it's good for battery protection	JD.com
Puning Dental	Cell Phone Battery	Great price and fast shipping! Packaging was great!	JD.com
Puning Dental	Cell Phone Battery	Works great and fits perfectly. It also plugs in.	JD.com
Puning Dental	Cell Phone Battery	The quality is pretty good.	JD.com
Puning Dental	Cell Phone Battery	The stuff works great, I'll buy another one later	JD.com
Puning Dental	Cell Phone Battery	IQ tax	JD.com
Puning Dental	Cell Phone Battery	This user feels the product is better	JD.com
Puning Dental	Cell Phone Battery	[Smiles][Skimming][Big Love]	JD.com
Puning Dental	Cell Phone Battery Repairer	Baby is very good, the logistics is very fast, the quality of workmanship is very good, cheaper than the entity store, trustworthy, like friends hurry to get down to it , , , , , , , , , , , , , , ,	JD.com
Puning Dental	Cell Phone Battery	False advertising, false products	JD.com
Puning Dental	Cell Phone Battery	I've got more memory with this. It's not laggy.	JD.com

Puning Dental	Cell Phone Battery	! ! ! ! ! ! ! ! ! ! !	JD.com
Puning Dental	Cell Phone Battery	This user feels the product is better	JD.com
Puning Dental	Cell Phone Battery	This user feels the product is better	JD.com
Puning Dental	Cell Phone Battery	Use it right now and punch a laptop.	JD.com
Puning Dental	Cell Phone Battery	Good quality, very cheap, good object	JD.com
Puning Dental	Cell Phone Battery	Support the new technology, the goods have been received. The effect of the use of follow-up comments!	JD.com
Puning Dental	Cell Phone Battery Repairer	especially well, thanks to the merchant's stuff works especially well, this is especially good.[Smiles] This very satisfied. This is very, very good, this satisfaction to you to play, I good praise.	JD.com
Puning Dental	Cell Phone Battery	Thanks to the merchant's stuff works exceptionally well, really exceptionally well possesses no problems at all!	JD.com
Puning Dental	Cell Phone Battery	Please return the product if there is any problem with the quality	JD.com
Puning Dental	Cell Phone Battery	I'm using it now with good use over and over and over again.	JD.com
Puning Dental	Cell Phone Battery	Logistics is very powerful Quality is very good Immediately repurchase	JD.com
Puning Dental	Cell Phone Battery	This user feels the product is better	JD.com
Puning Dental	Cell Phone Battery Repairer	is very fast, the customer service attitude is very positive, and questions are answered in a timely manner! Overall very satisfied!	JD.com
Puning Dental	Cell Phone Battery	This user feels the product is better	JD.com
Puning Dental	Cell Phone Battery	inexpensive and good quality, fast delivery, customer service is very good too!	JD.com
Puning Dental	Cell Phone Battery	I'll wait to use it before I say how I feel about it.	JD.com
Puning Dental	Cell Phone Battery Repairer	beautifully packaged/product! It is a good adapter to improve the battery life of mobilephone/optimize cell phonesystem/clean up cell phone garbage and improverunning speed of cell phone!Trustworthy/purchaseuse! I wish the manufacturer a prosperous business! We wish you a prosperous business! Popularity is strong and prosperous![Kudos][Buy]][Good][Buy]][Clap][Hands[Ah Huang][Handshake][Tongue]Rose]	JD.com
Puning Dental	Cell Phone Battery Repairer	The item has been received, the courier is also very fast, the product quality is very good, the workmanship is very refined, is genuine, the price is also very cheap, and the description is exactly the same, I give five-star praise.	JD.com
Puning Dental	Cell Phone Battery	Comments	JD.com
Puning Dental	Cell Phone Battery	Well, I can't say yet. Let's just use it and try it out! Uh-huh!	JD.com
Puning Dental	Cell Phone Battery	It looks good.	JD.com
Puning Dental	Cell Phone Battery	Repair has received, use good re-purchase wish the store business prosperity!	JD.com
Puning Dental	Cell Phone Battery	It works well.	JD.com
Puning Dental	Cell Phone Battery Repairer	Conversion efficiency: good<br>Compatibility: strong<br>Security: good	JD.com
Puning Dental	Cell Phone Battery	Cell phone adapter received looks good used well in buy a	JD.com
Puning Dental	Cell Phone Battery	Received the item and it looks great. The style is also beautiful and okay.	JD.com
Puning Dental	Cell Phone Battery	Friends introduced to buy, on the cell phone pool repair remarkable, worth buying.	JD.com
Puning Dental	Cell Phone Battery	pretty good	JD.com
Puning Dental	Cell Phone Battery Repairer	The goods are very delicate, logistics is also fast, the business service is enthusiastic<br>Give five-star praise	JD.com
Puning Dental	Cell Phone Battery Repairer	Great price for what you get. Will buy the good stuff again in the future. Probably would work better at a higher price. Recommended purchase has been sent to the Poundland coterie.	JD.com
Puning Dental	Cell Phone Battery	I am very happy with your merchandise and the product is great.	JD.com
Puning Dental	Cell Phone Battery	Useful to repair fast charging good quality	JD.com

Puning Dental	Cell Phone Battery Repairer	products also fraudulent consumer business or unlicensed operation!	JD.com
Puning Dental	Cell Phone Battery Repairer	Authentic battery activator, cell phone repair is very easy, product quality is quite over the top, very satisfied with the special like, the price is cheap things are good, fine workmanship style is good, the service is thoughtful shipping fast, full-star praise a hundred praise!	JD.com
Puning Dental	Cell Phone Battery Repairer	This store's repairer is good, the cell phone battery activation rate is high, authentic product stuff is good, exquisite and beautiful high quality, fast shipping and thoughtful service, praise a full star high!	JD.com
Puning Dental	Cell Phone Battery Repairer	Cell phone repairer has received, genuine activator effect, product over the quality of high, something special good spend less, very satisfied with the super like, thoughtful service delivery fast, full of stars!	JD.com
Puning Dental	Cell Phone Battery Repairer	smooth, fine workmanship kind of beautiful, product quality is quite over the top, the price is cheap things are good, the service is enthusiastic and thoughtful, fast delivery full of stars high light!	JD.com
Puning Dental	Cell Phone Battery Repairer	Cheap and affordable<br>Stability: good<br>Will repurchase again	JD.com
Puning Dental	Cell Phone Battery Repairer	Quality can be, the courier boy is very enthusiastic please you can rest assured to buy!	JD.com
Puning Dental	Cell Phone Battery Repairer	This item is great quality and fast shipping.	JD.com
Puning Dental	Cell Phone Battery Repairer	classifier for objects in rows such as words	JD.com
Puning Dental	Cell Phone Battery Repairer	Received a cell phone battery repairer, the quality is quite good, exquisite packaging, good seller service attitude, logistics and express timely, all five-star praise. Wish merchants prosperous business.	JD.com
Puning Dental	Cell Phone Battery Repairer	Well packaged, inexpensive and easy to use.	JD.com
Puning Dental	Cell Phone Battery Repairer	Charging is average, charging is so slow	JD.com
Puning Dental	Cell Phone Battery Repairer	Very good satisfied next time come back[buy buy buy][kudos][kudos]	JD.com
Puning Dental	Cell Phone Battery Repairer	This user feels the product is better	JD.com
Puning Dental	Cell Phone Battery Repairer	Review after use.	JD.com
Puning Dental	Cell Phone Battery Repairer	Battery Repairer received, quite good, good quality, carefully packaged, intact, good business attitude, logistics and express timely, all five-star praise.	JD.com
Puning Dental	Cell Phone Battery Repairer	Plus this is similar to slow charging, it's good for battery protection	JD.com
Puning Dental	Cell Phone Battery Repairer	Great price and fast shipping! Packaging was great!	JD.com
Puning Dental	Cell Phone Battery Repairer	Works great and fits perfectly. It also plugs in.	JD.com
Puning Dental	Cell Phone Battery Repairer	The quality is pretty good.	JD.com
Puning Dental	Cell Phone Battery Repairer	The stuff works great, I'll buy another one later	JD.com
Puning Dental	Cell Phone Battery Repairer	IQ tax	JD.com
Puning Dental	Cell Phone Battery Repairer	This user feels the product is better	JD.com
Puning Dental	Cell Phone Battery Repairer	[Smiles][Skimming][Big Love]	JD.com
Puning Dental	Cell Phone Battery Repairer	Baby is very good, the logistics is very fast, the quality of workmanship is very good, cheaper than the entity store, trustworthy, like friends hurry to get down to it , , , , , , , , , , , , , , ,	JD.com
Puning Dental	Cell Phone Battery Repairer	False advertising, false products	JD.com
Puning Dental	Cell Phone Battery Repairer	I've got more memory with this. It's not laggy.	JD.com
Puning Dental	Cell Phone Battery Repairer	! ! ! ! ! ! ! ! ! ! !	JD.com
Puning Dental	Cell Phone Battery Repairer	This user feels the product is better	JD.com
Puning Dental	Cell Phone Battery Repairer	This user feels the product is better	JD.com
Puning Dental	Cell Phone Battery Repairer	Use it right now and punch a laptop.	JD.com
Puning Dental	Cell Phone Battery Repairer	Good quality, very cheap, good object	JD.com

Puning Dental	Cell Phone Battery Repairer	Support the new technology, the goods have been received. The effect of the use of follow-up comments!	JD.com
Puning Dental	Cell Phone Battery Repairer	especially well, thanks to the merchant's stuff works especially well, this is especially good.[Smiles]This very satisfied. This is very, very good, this satisfaction to you to play, I good praise.	JD.com
Puning Dental	Cell Phone Battery Repairer	Thanks to the merchant's stuff works exceptionally well, really exceptionally well possesses no problems at all!	JD.com
Puning Dental	Cell Phone Battery Repairer	Please return the product if there is any problem with the quality	JD.com
Puning Dental	Cell Phone Battery Repairer	I'm using it now with good use over and over and over again.	JD.com
Puning Dental	Cell Phone Battery Repairer	Logistics is very powerful Quality is very good Immediately repurchase	JD.com
Puning Dental	Cell Phone Battery Repairer	This user feels the product is better	JD.com
Puning Dental	Cell Phone Battery Repairer	is very fast, the customer service attitude is very positive, and questions are answered in a timely manner! Overall very satisfied!	JD.com
Puning Dental	Cell Phone Battery Repairer	This user feels the product is better	JD.com
Puning Dental	Cell Phone Battery Repairer	inexpensive and good quality, fast delivery, customer service is very good too!	JD.com
Puning Dental	Cell Phone Battery Repairer	I'll wait to use it before I say how I feel about it.	JD.com
Puning Dental	Cell Phone Battery Repairer	beautifully packaged/product! It is a good adapter to improve the battery life of mobilephone/optimize cell phonesystem/clean up cell phone garbage and improverunning speed of cell phone!Trustworthy/purchaseuse! I wish the manufacturer a prosperous business! We wish you a prosperous business! Popularity is strong and prosperous![Kudos][Buy]][Good][Buy]][Clap][Hands[Ah Huang][Handshake][Tongue]Rose]	JD.com
Puning Dental	Cell Phone Battery Repairer	The item has been received, the courier is also very fast, the product quality is very good, the workmanship is very refined, is genuine, the price is also very cheap, and the description is exactly the same, I give five-star praise.	JD.com
Puning Dental	Cell Phone Battery Repairer	Comments	JD.com
Puning Dental	Cell Phone Battery Repairer	Well, I can't say yet. Let's just use it and try it out! Uh-huh!	JD.com
Puning Dental	Cell Phone Battery Repairer	It looks good.	JD.com
Puning Dental	Cell Phone Battery Repairer	Repair has received, use good re-purchase wish the store business prosperity!	JD.com
Puning Dental	Cell Phone Battery Repairer	It works well.	JD.com
Puning Dental	Cell Phone Battery Repairer	Conversion efficiency: good<br>Compatibility: strong<br>Security: good	JD.com
Puning Dental	Cell Phone Battery Repairer	Cell phone adapter received looks good used well in buy a|	JD.com
Puning Dental	Cell Phone Battery Repairer	Received the item and it looks great. The style is also beautiful and okay.	JD.com
Puning Dental	Cell Phone Battery Repairer	Friends introduced to buy, on the cell phone pool repair remarkable, worth buying.	JD.com
Puning Dental	Cell Phone Battery Repairer	pretty good	JD.com
Puning Dental	Cell Phone Battery Repairer	The goods are very delicate, logistics is also fast, the business service is enthusiastic<br>Give five-star praise	JD.com
Puning Dental	Cell Phone Battery Repairer	Great price for what you get. Will buy the good stuff again in the future. Probably would work better at a higher price. Recommended purchase has been sent to the Poundland coterie.	JD.com
Puning Dental	Cell Phone Battery Repairer	I am very happy with your merchandise and the product is great.	JD.com
Puning Dental	Cell Phone Battery Repairer	Useful to repair fast charging good quality	JD.com
Puning Dental	Cell Phone Battery Repairer	products also fraudulent consumer business or unlicensed operation!	JD.com
Puning Dental	Cell Phone Battery Repairer	Authentic battery activator, cell phone repair is very easy, product quality is quite over the top, very satisfied with the special like, the price is cheap things are good, fine workmanship style is good, the service is thoughtful shipping fast, full-star praise a hundred praise!	JD.com

Puning Dental	Cell Phone Battery Repairer	This store's repairer is good, the cell phone battery activation rate is high, authentic product stuff is good, exquisite and beautiful high quality, fast shipping and thoughtful service, praise a full star high!	JD.com
Puning Dental	Cell Phone Battery Repairer	Cell phone repairer has received, genuine activator effect, product over the quality of high, something special good spend less, very satisfied with the super like, thoughtful service delivery fast, full of stars!	JD.com
Puning Dental	Cell Phone Battery Repairer	smooth, fine workmanship kind of beautiful, product quality is quite over the top, the price is cheap things are good, the service is enthusiastic and thoughtful, fast delivery full of stars high light!	JD.com
Puning Dental	Cell Phone Battery Repairer	Cheap and affordable<br>Stability: good<br>Will repurchase again	JD.com
Puning Dental	Cell Phone Battery	Quality can be, the courier boy is very enthusiastic please you can rest assured to buy!	JD.com
Puning Dental	Cell Phone Battery	This item is great quality and fast shipping.	JD.com
Puning Dental	Cell Phone Battery	classifier for objects in rows such as words	JD.com
Puning Dental	Cell Phone Battery Repairer	Received a cell phone battery repairer, the quality is quite good, exquisite packaging, good seller service attitude, logistics and express timely, all five-star praise. Wish merchants prosperous business.	JD.com
Puning Dental	Cell Phone Battery	Well packaged, inexpensive and easy to use.	JD.com
Puning Dental	Cell Phone Battery	Charging is average, charging is so slow	JD.com
Puning Dental	Cell Phone Battery	Very good satisfied next time come back[buy buy buy][kudos][kudos]	JD.com
Puning Dental	Cell Phone Battery	This user feels the product is better	JD.com
Puning Dental	Cell Phone Battery	Review after use.	JD.com
Puning Dental	Cell Phone Battery Repairer	Battery Repairer received, quite good, good quality, carefully packaged, intact, good business attitude, logistics and express timely, all five-star praise.	JD.com
Puning Dental	Cell Phone Battery	Plus this is similar to slow charging, it's good for battery protection	JD.com
Puning Dental	Cell Phone Battery	Great price and fast shipping! Packaging was great!	JD.com
Puning Dental	Cell Phone Battery	Works great and fits perfectly. It also plugs in.	JD.com
Puning Dental	Cell Phone Battery	The quality is pretty good.	JD.com
Puning Dental	Cell Phone Battery	The stuff works great, I'll buy another one later	JD.com
Puning Dental	Cell Phone Battery	IQ tax	JD.com
Puning Dental	Cell Phone Battery	This user feels the product is better	JD.com
Puning Dental	Cell Phone Battery	[Smiles][Skimming][Big Love]	JD.com
Puning Dental	Cell Phone Battery Repairer	Baby is very good, the logistics is very fast, the quality of workmanship is very good, cheaper than the entity store, trustworthy, like friends hurry to get down to it , , , , , , , , , , , , , , ,	JD.com
Puning Dental	Cell Phone Battery	False advertising, false products	JD.com
Puning Dental	Cell Phone Battery	I've got more memory with this. It's not laggy.	JD.com
Puning Dental	Cell Phone Battery	! ! ! ! ! ! ! ! ! ! !	JD.com
Puning Dental	Cell Phone Battery	This user feels the product is better	JD.com
Puning Dental	Cell Phone Battery	This user feels the product is better	JD.com
Puning Dental	Cell Phone Battery	Use it right now and punch a laptop.	JD.com
Puning Dental	Cell Phone Battery	Good quality, very cheap, good object	JD.com
Puning Dental	Cell Phone Battery	Support the new technology, the goods have been received. The effect of the use of follow-up comments!	JD.com
Puning Dental	Cell Phone Battery Repairer	especially well, thanks to the merchant's stuff works especially well, this is especially good.[Smiles]This very satisfied. This is very, very good, this satisfaction to you to play, I good praise.	JD.com

Puning Dental	Cell Phone Battery	Thanks to the merchant's stuff works exceptionally well, really exceptionally well possesses no problems at all!	JD.com
Puning Dental	Cell Phone Battery	Please return the product if there is any problem with the quality	JD.com
Puning Dental	Cell Phone Battery	I'm using it now with good use over and over and over again.	JD.com
Puning Dental	Cell Phone Battery	Logistics is very powerful Quality is very good Immediately repurchase	JD.com
Puning Dental	Cell Phone Battery	This user feels the product is better	JD.com
Puning Dental	Cell Phone Battery Repairer	is very fast, the customer service attitude is very positive, and questions are answered in a timely manner! Overall very satisfied!	JD.com
Puning Dental	Cell Phone Battery	This user feels the product is better	JD.com
Puning Dental	Cell Phone Battery	inexpensive and good quality, fast delivery, customer service is very good too!	JD.com
Puning Dental	Cell Phone Battery	I'll wait to use it before I say how I feel about it.	JD.com
Puning Dental	Cell Phone Battery Repairer	beautifully packaged/product! It is a good adapter to improve the battery life of mobilephone/optimize cell phonesystem/clean up cell phone garbage and improverunning speed of cell phone!Trustworthy/purchaseuse! I wish the manufacturer a prosperous business! We wish you a prosperous business! Popularity is strong and prosperous![Kudos][Buy]][Good][Buy]][Clap][Hands[Ah Huang][Handshake][Tongue]Rose]	JD.com
Puning Dental	Cell Phone Battery Repairer	The item has been received, the courier is also very fast, the product quality is very good, the workmanship is very refined, is genuine, the price is also very cheap, and the description is exactly the same, I give five-star praise.	JD.com
Puning Dental	Cell Phone Battery	Comments	JD.com
Puning Dental	Cell Phone Battery	Well, I can't say yet. Let's just use it and try it out! Uh-huh!	JD.com
Puning Dental	Cell Phone Battery	It looks good.	JD.com
Puning Dental	Cell Phone Battery	Repair has received, use good re-purchase wish the store business prosperity!	JD.com
Puning Dental	Cell Phone Battery	It works well.	JD.com
Puning Dental	Cell Phone Battery Repairer	Conversion efficiency: good<br>Compatibility: strong<br>Security: good	JD.com
Puning Dental	Cell Phone Battery	Cell phone adapter received looks good used well in buy a	JD.com
Puning Dental	Cell Phone Battery	Received the item and it looks great. The style is also beautiful and okay.	JD.com
Puning Dental	Cell Phone Battery	Friends introduced to buy, on the cell phone pool repair remarkable, worth buying.	JD.com
Puning Dental	Cell Phone Battery	pretty good	JD.com
Puning Dental	Cell Phone Battery Repairer	The goods are very delicate, logistics is also fast, the business service is enthusiastic<br>Give five-star praise	JD.com
Puning Dental	Cell Phone Battery Repairer	Great price for what you get. Will buy the good stuff again in the future. Probably would work better at a higher price. Recommended purchase has been sent to the Poundland coterie.	JD.com
Puning Dental	Cell Phone Battery	I am very happy with your merchandise and the product is great.	JD.com
Puning Dental	Cell Phone Battery	Useful to repair fast charging good quality	JD.com
Puning Dental	Cell Phone Battery	products also fraudulent consumer business or unlicensed operation!	JD.com
Puning Dental	Cell Phone Battery Repairer	Authentic battery activator, cell phone repair is very easy, product quality is quite over the top, very satisfied with the special like, the price is cheap things are good, fine workmanship style is good, the service is thoughtful shipping fast, full-star praise a hundred praise!	JD.com
Puning Dental	Cell Phone Battery Repairer	This store's repairer is good, the cell phone battery activation rate is high, authentic product stuff is good, exquisite and beautiful high quality, fast shipping and thoughtful service, praise a full star high!	JD.com
Puning Dental	Cell Phone Battery Repairer	Cell phone repairer has received, genuine activator effect, product over the quality of high, something special good spend less, very satisfied with the super like, thoughtful service delivery fast, full of stars!	JD.com

Puning Dental	Cell Phone Battery Repairer	smooth, fine workmanship kind of beautiful, product quality is quite over the top, the price is cheap things are good, the service is enthusiastic and thoughtful, fast delivery full of stars high light!	JD.com
Puning Dental	Cell Phone Battery Repairer	Cheap and affordable<br>Stability: good<br>Will repurchase again	JD.com
Puning Dental	Cell Phone Battery	Quality can be, the courier boy is very enthusiastic please you can rest assured to buy!	JD.com
Puning Dental	Cell Phone Battery	This item is great quality and fast shipping.	JD.com
Puning Dental	Cell Phone Battery	classifier for objects in rows such as words	JD.com
Puning Dental	Cell Phone Battery Repairer	Received a cell phone battery repairer, the quality is quite good, exquisite packaging, good seller service attitude, logistics and express timely, all five-star praise. Wish merchants prosperous business.	JD.com
Puning Dental	Cell Phone Battery	Well packaged, inexpensive and easy to use.	JD.com
Puning Dental	Cell Phone Battery	Charging is average, charging is so slow	JD.com
Puning Dental	Cell Phone Battery	Very good satisfied next time come back[buy buy buy][kudos][kudos]	JD.com
Puning Dental	Cell Phone Battery	This user feels the product is better	JD.com
Puning Dental	Cell Phone Battery	Review after use.	JD.com
Puning Dental	Cell Phone Battery Repairer	Battery Repairer received, quite good, good quality, carefully packaged, intact, good business attitude, logistics and express timely, all five-star praise.	JD.com
Puning Dental	Cell Phone Battery	Plus this is similar to slow charging, it's good for battery protection	JD.com
Puning Dental	Cell Phone Battery	Great price and fast shipping! Packaging was great!	JD.com
Puning Dental	Cell Phone Battery	Works great and fits perfectly. It also plugs in.	JD.com
Puning Dental	Cell Phone Battery	The quality is pretty good.	JD.com
Puning Dental	Cell Phone Battery	The stuff works great, I'll buy another one later	JD.com
Puning Dental	Cell Phone Battery	IQ tax	JD.com
Puning Dental	Cell Phone Battery	This user feels the product is better	JD.com
Puning Dental	Cell Phone Battery	[Smiles][Skimming][Big Love]	JD.com
Puning Dental	Cell Phone Battery Repairer	Baby is very good, the logistics is very fast, the quality of workmanship is very good, cheaper than the entity store, trustworthy, like friends hurry to get down to it , , , , , , , , , , , , , , ,	JD.com
Puning Dental	Cell Phone Battery	False advertising, false products	JD.com
Puning Dental	Cell Phone Battery	I've got more memory with this. It's not laggy.	JD.com
Puning Dental	Cell Phone Battery	! ! ! ! ! ! ! ! ! ! !	JD.com
Puning Dental	Cell Phone Battery	This user feels the product is better	JD.com
Puning Dental	Cell Phone Battery	This user feels the product is better	JD.com
Puning Dental	Cell Phone Battery	Use it right now and punch a laptop.	JD.com
Puning Dental	Cell Phone Battery	Good quality, very cheap, good object	JD.com
Puning Dental	Cell Phone Battery	Support the new technology, the goods have been received. The effect of the use of follow-up comments!	JD.com
Puning Dental	Cell Phone Battery Repairer	especially well, thanks to the merchant's stuff works especially well, this is especially good.[Smiles]This very satisfied. This is very, very good, this satisfaction to you to play, I good praise.	JD.com
Puning Dental	Cell Phone Battery	Thanks to the merchant's stuff works exceptionally well, really exceptionally well possesses no problems at all!	JD.com
Puning Dental	Cell Phone Battery	Please return the product if there is any problem with the quality	JD.com
Puning Dental	Cell Phone Battery	I'm using it now with good use over and over and over again.	JD.com
Puning Dental	Cell Phone Battery	Logistics is very powerful Quality is very good Immediately repurchase	JD.com

Puning Dental	Cell Phone Battery	This user feels the product is better	JD.com
Puning Dental	Cell Phone Battery Repairer	is very fast, the customer service attitude is very positive, and questions are answered in a timely manner! Overall very satisfied!	JD.com
Puning Dental	Cell Phone Battery	This user feels the product is better	JD.com
Puning Dental	Cell Phone Battery	inexpensive and good quality, fast delivery, customer service is very good too!	JD.com
Puning Dental	Cell Phone Battery	I'll wait to use it before I say how I feel about it.	JD.com
Puning Dental	Cell Phone Battery Repairer	beautifully packaged/product! It is a good adapter to improve the battery life of mobilephone/optimize cell phonesystem/clean up cell phone garbage and improverunning speed of cell phone!Trustworthy/purchaseuse! I wish the manufacturer a prosperous business! We wish you a prosperous business! Popularity is strong and prosperous![Kudos][Buy]][Good][Buy]][Clap][Hands[Ah Huang][Handshake][Tongue]Rose]	JD.com
Puning Dental	Cell Phone Battery Repairer	The item has been received, the courier is also very fast, the product quality is very good, the workmanship is very refined, is genuine, the price is also very cheap, and the description is exactly the same, I give five-star praise.	JD.com
Puning Dental	Cell Phone Battery	Comments	JD.com
Puning Dental	Cell Phone Battery	Well, I can't say yet. Let's just use it and try it out! Uh-huh!	JD.com
Puning Dental	Cell Phone Battery	It looks good.	JD.com
Puning Dental	Cell Phone Battery	Repair has received, use good re-purchase wish the store business prosperity!	JD.com
Puning Dental	Cell Phone Battery	It works well.	JD.com
Puning Dental	Cell Phone Battery Repairer	Conversion efficiency: good<br>Compatibility: strong<br>Security: good	JD.com
Puning Dental	Cell Phone Battery	Cell phone adapter received looks good used well in buy a	JD.com
Puning Dental	Cell Phone Battery	Received the item and it looks great. The style is also beautiful and okay.	JD.com
Puning Dental	Cell Phone Battery	Friends introduced to buy, on the cell phone pool repair remarkable, worth buying.	JD.com
Puning Dental	Cell Phone Battery	pretty good	JD.com
Puning Dental	Cell Phone Battery Repairer	The goods are very delicate, logistics is also fast, the business service is enthusiastic<br>Give five-star praise	JD.com
Puning Dental	Cell Phone Battery Repairer	Great price for what you get. Will buy the good stuff again in the future. Probably would work better at a higher price. Recommended purchase has been sent to the Poundland coterie.	JD.com
Puning Dental	Cell Phone Battery	I am very happy with your merchandise and the product is great.	JD.com
Puning Dental	Cell Phone Battery	Useful to repair fast charging good quality	JD.com
Puning Dental	Cell Phone Battery	products also fraudulent consumer business or unlicensed operation!	JD.com
Puning Dental	Cell Phone Battery Repairer	Authentic battery activator, cell phone repair is very easy, product quality is quite over the top, very satisfied with the special like, the price is cheap things are good, fine workmanship style is good, the service is thoughtful shipping fast, full-star praise a hundred praise!	JD.com
Puning Dental	Cell Phone Battery Repairer	This store's repairer is good, the cell phone battery activation rate is high, authentic product stuff is good, exquisite and beautiful high quality, fast shipping and thoughtful service, praise a full star high!	JD.com
Puning Dental	Cell Phone Battery Repairer	Cell phone repairer has received, genuine activator effect, product over the quality of high, something special good spend less, very satisfied with the super like, thoughtful service delivery fast, full of stars!	JD.com
Puning Dental	Cell Phone Battery Repairer	smooth, fine workmanship kind of beautiful, product quality is quite over the top, the price is cheap things are good, the service is enthusiastic and thoughtful, fast delivery full of stars high light!	JD.com
Puning Dental	Cell Phone Battery Repairer	Cheap and affordable<br>Stability: good<br>Will repurchase again	JD.com
Puning Dental	Cell Phone Battery	Quality can be, the courier boy is very enthusiastic please you can rest assured to buy!	JD.com

Puning Dental	Cell Phone Battery Repairer	This item is great quality and fast shipping.	JD.com
Puning Dental	Cell Phone Battery Repairer	classifier for objects in rows such as words	JD.com
Puning Dental	Cell Phone Battery Repairer	Received a cell phone battery repairer, the quality is quite good, exquisite packaging, good seller service attitude, logistics and express timely, all five-star praise. Wish merchants prosperous business.	JD.com
Puning Dental	Cell Phone Battery Repairer	Well packaged, inexpensive and easy to use.	JD.com
Puning Dental	Cell Phone Battery Repairer	Charging is average, charging is so slow	JD.com
Puning Dental	Cell Phone Battery Repairer	Very good satisfied next time come back[buy buy buy][kudos][kudos]	JD.com
Puning Dental	Cell Phone Battery Repairer	This user feels the product is better	JD.com
Puning Dental	Cell Phone Battery Repairer	Review after use.	JD.com
Puning Dental	Cell Phone Battery Repairer	Battery Repairer received, quite good, good quality, carefully packaged, intact, good business attitude, logistics and express timely, all five-star praise.	JD.com
Puning Dental	Cell Phone Battery Repairer	Plus this is similar to slow charging, it's good for battery protection	JD.com
Puning Dental	Cell Phone Battery Repairer	Great price and fast shipping! Packaging was great!	JD.com
Puning Dental	Cell Phone Battery Repairer	Works great and fits perfectly. It also plugs in.	JD.com
Puning Dental	Cell Phone Battery Repairer	The quality is pretty good.	JD.com
Puning Dental	Cell Phone Battery Repairer	The stuff works great, I'll buy another one later	JD.com
Puning Dental	Cell Phone Battery Repairer	IQ tax	JD.com
Puning Dental	Cell Phone Battery Repairer	This user feels the product is better	JD.com
Puning Dental	Cell Phone Battery Repairer	[Smiles][Skimming][Big Love]	JD.com
Puning Dental	Cell Phone Battery Repairer	Baby is very good, the logistics is very fast, the quality of workmanship is very good, cheaper than the entity store, trustworthy, like friends hurry to get down to it , , , , , , , , , , , , , , ,	JD.com
Puning Dental	Cell Phone Battery Repairer	False advertising, false products	JD.com
Puning Dental	Cell Phone Battery Repairer	I've got more memory with this. It's not laggy.	JD.com
Puning Dental	Cell Phone Battery Repairer	！！！！！！！！！！！	JD.com
Puning Dental	Cell Phone Battery Repairer	This user feels the product is better	JD.com
Puning Dental	Cell Phone Battery Repairer	This user feels the product is better	JD.com
Puning Dental	Cell Phone Battery Repairer	Use it right now and punch a laptop.	JD.com
Puning Dental	Cell Phone Battery Repairer	Good quality, very cheap, good object	JD.com
Puning Dental	Cell Phone Battery Repairer	Support the new technology, the goods have been received. The effect of the use of follow-up comments!	JD.com
Puning Dental	Cell Phone Battery Repairer	especially well, thanks to the merchant's stuff works especially well, this is especially good.[Smiles]This very satisfied. This is very, very good, this satisfaction to you to play, I good praise.	JD.com
Puning Dental	Cell Phone Battery Repairer	Thanks to the merchant's stuff works exceptionally well, really exceptionally well possesses no problems at all!	JD.com
Puning Dental	Cell Phone Battery Repairer	Please return the product if there is any problem with the quality	JD.com
Puning Dental	Cell Phone Battery Repairer	I'm using it now with good use over and over and over again.	JD.com
Puning Dental	Cell Phone Battery Repairer	Logistics is very powerful Quality is very good Immediately repurchase	JD.com
Puning Dental	Cell Phone Battery Repairer	This user feels the product is better	JD.com
Puning Dental	Cell Phone Battery Repairer	is very fast, the customer service attitude is very positive, and questions are answered in a timely manner! Overall very satisfied!	JD.com
Puning Dental	Cell Phone Battery Repairer	This user feels the product is better	JD.com
Puning Dental	Cell Phone Battery Repairer	inexpensive and good quality, fast delivery, customer service is very good too!	JD.com

Puning Dental	Cell Phone Battery	I'll wait to use it before I say how I feel about it.	JD.com
Puning Dental	Cell Phone Battery Repairer	beautifully packaged/product! It is a good adapter to improve the battery life of mobilephone/optimize cell phonesystem/clean up cell phone garbage and improverunning speed of cell phone!Trustworthy/purchaseuse! I wish the manufacturer a prosperous business! We wish you a prosperous business! Popularity is strong and prosperous![Kudos][Buy]][Good][Buy]][Clap][Hands[Ah Huang][Handshake][Tongue]Rose]	JD.com
Puning Dental	Cell Phone Battery Repairer	The item has been received, the courier is also very fast, the product quality is very good, the workmanship is very refined, is genuine, the price is also very cheap, and the description is exactly the same, I give five-star praise.	JD.com
Puning Dental	Cell Phone Battery	Comments	JD.com
Puning Dental	Cell Phone Battery	Well, I can't say yet. Let's just use it and try it out! Uh-huh!	JD.com
Puning Dental	Cell Phone Battery	It looks good.	JD.com
Puning Dental	Cell Phone Battery	Repair has received, use good re-purchase wish the store business prosperity!	JD.com
Puning Dental	Cell Phone Battery	It works well.	JD.com
Puning Dental	Cell Phone Battery Repairer	Conversion efficiency: good<br>Compatibility: strong<br>Security: good	JD.com
Puning Dental	Cell Phone Battery	Cell phone adapter received looks good used well in buy a	JD.com
Puning Dental	Cell Phone Battery	Received the item and it looks great. The style is also beautiful and okay.	JD.com
Puning Dental	Cell Phone Battery	Friends introduced to buy, on the cell phone pool repair remarkable, worth buying.	JD.com
Puning Dental	Cell Phone Battery	pretty good	JD.com
Puning Dental	Cell Phone Battery Repairer	The goods are very delicate, logistics is also fast, the business service is enthusiastic<br>Give five-star praise	JD.com
Puning Dental	Cell Phone Battery Repairer	Great price for what you get. Will buy the good stuff again in the future. Probably would work better at a higher price. Recommended purchase has been sent to the Poundland coterie.	JD.com
Puning Dental	Cell Phone Battery	I am very happy with your merchandise and the product is great.	JD.com
Puning Dental	Cell Phone Battery	Useful to repair fast charging good quality	JD.com
Puning Dental	Cell Phone Battery	products also fraudulent consumer business or unlicensed operation!	JD.com
Puning Dental	Cell Phone Battery Repairer	Authentic battery activator, cell phone repair is very easy, product quality is quite over the top, very satisfied with the special like, the price is cheap things are good, fine workmanship style is good, the service is thoughtful shipping fast, full-star praise a hundred praise!	JD.com
Puning Dental	Cell Phone Battery Repairer	This store's repairer is good, the cell phone battery activation rate is high, authentic product stuff is good, exquisite and beautiful high quality, fast shipping and thoughtful service, praise a full star high!	JD.com
Puning Dental	Cell Phone Battery Repairer	Cell phone repairer has received, genuine activator effect, product over the quality of high, something special good spend less, very satisfied with the super like, thoughtful service delivery fast, full of stars!	JD.com
Puning Dental	Cell Phone Battery Repairer	smooth, fine workmanship kind of beautiful, product quality is quite over the top, the price is cheap things are good, the service is enthusiastic and thoughtful, fast delivery full of stars high light!	JD.com
Puning Dental	Cell Phone Battery Repairer	Cheap and affordable<br>Stability: good<br>Will repurchase again	JD.com
Puning Dental	Cell Phone Battery	Quality can be, the courier boy is very enthusiastic please you can rest assured to buy!	JD.com
Puning Dental	Cell Phone Battery	This item is great quality and fast shipping.	JD.com
Puning Dental	Cell Phone Battery	classifier for objects in rows such as words	JD.com
Puning Dental	Cell Phone Battery Repairer	Received a cell phone battery repairer, the quality is quite good, exquisite packaging, good seller service attitude, logistics and express timely, all five-star praise. Wish merchants prosperous business.	JD.com

Puning Dental	Cell Phone Battery	Well packaged, inexpensive and easy to use.	JD.com
Puning Dental	Cell Phone Battery	Charging is average, charging is so slow	JD.com
Puning Dental	Cell Phone Battery	Very good satisfied next time come back[buy buy buy][kudos][kudos]	JD.com
Puning Dental	Cell Phone Battery	This user feels the product is better	JD.com
Puning Dental	Cell Phone Battery	Review after use.	JD.com
Puning Dental	Cell Phone Battery Repairer	Battery Repairer received, quite good, good quality, carefully packaged, intact, good business attitude, logistics and express timely, all five-star praise.	JD.com
Puning Dental	Cell Phone Battery	Plus this is similar to slow charging, it's good for battery protection	JD.com
Puning Dental	Cell Phone Battery	Great price and fast shipping! Packaging was great!	JD.com
Puning Dental	Cell Phone Battery	Works great and fits perfectly. It also plugs in.	JD.com
Puning Dental	Cell Phone Battery	The quality is pretty good.	JD.com
Puning Dental	Cell Phone Battery	The stuff works great, I'll buy another one later	JD.com
Puning Dental	Cell Phone Battery	IQ tax	JD.com
Puning Dental	Cell Phone Battery	This user feels the product is better	JD.com
Puning Dental	Cell Phone Battery	[Smiles][Skimming][Big Love]	JD.com
Puning Dental	Cell Phone Battery Repairer	Baby is very good, the logistics is very fast, the quality of workmanship is very good, cheaper than the entity store, trustworthy, like friends hurry to get down to it , , , , , , , , , , , , , , ,	JD.com
Puning Dental	Cell Phone Battery	False advertising, false products	JD.com
Puning Dental	Cell Phone Battery	I've got more memory with this. It's not laggy.	JD.com
Puning Dental	Cell Phone Battery	! ! ! ! ! ! ! ! ! ! !	JD.com
Puning Dental	Cell Phone Battery	This user feels the product is better	JD.com
Puning Dental	Cell Phone Battery	This user feels the product is better	JD.com
Puning Dental	Cell Phone Battery	Use it right now and punch a laptop.	JD.com
Puning Dental	Cell Phone Battery	Good quality, very cheap, good object	JD.com
Puning Dental	Cell Phone Battery	Support the new technology, the goods have been received. The effect of the use of follow-up comments!	JD.com
Puning Dental	Cell Phone Battery Repairer	especially well, thanks to the merchant's stuff works especially well, this is especially good.[Smiles]This very satisfied. This is very, very good, this satisfaction to you to play, I good praise.	JD.com
Puning Dental	Cell Phone Battery	Thanks to the merchant's stuff works exceptionally well, really exceptionally well possesses no problems at all!	JD.com
Puning Dental	Cell Phone Battery	Please return the product if there is any problem with the quality	JD.com
Puning Dental	Cell Phone Battery	I'm using it now with good use over and over and over again.	JD.com
Puning Dental	Cell Phone Battery	Logistics is very powerful Quality is very good Immediately repurchase	JD.com
Puning Dental	Cell Phone Battery	This user feels the product is better	JD.com
Puning Dental	Cell Phone Battery Repairer	is very fast, the customer service attitude is very positive, and questions are answered in a timely manner! Overall very satisfied!	JD.com
Puning Dental	Cell Phone Battery	This user feels the product is better	JD.com
Puning Dental	Cell Phone Battery	inexpensive and good quality, fast delivery, customer service is very good too!	JD.com
Puning Dental	Cell Phone Battery	I'll wait to use it before I say how I feel about it.	JD.com

Puning Dental	Cell Phone Battery Repairer	beautifully packaged/product! It is a good adapter to improve the battery life of mobilephone/optimize cell phonesystem/clean up cell phone garbage and improverunning speed of cell phone!Trustworthy/purchaseuse! I wish the manufacturer a prosperous business! We wish you a prosperous business! Popularity is strong and prosperous![Kudos][Buy]][Good][Buy]][Clap][Hands[Ah Huang][Handshake][Tongue]Rose]	JD.com
Puning Dental	Cell Phone Battery Repairer	The item has been received, the courier is also very fast, the product quality is very good, the workmanship is very refined, is genuine, the price is also very cheap, and the description is exactly the same, I give five-star praise.	JD.com
Puning Dental	Cell Phone Battery	Comments	JD.com
Puning Dental	Cell Phone Battery	Well, I can't say yet. Let's just use it and try it out! Uh-huh!	JD.com
Puning Dental	Cell Phone Battery	It looks good.	JD.com
Puning Dental	Cell Phone Battery	Repair has received, use good re-purchase wish the store business prosperity!	JD.com
Puning Dental	Cell Phone Battery	It works well.	JD.com
Puning Dental	Cell Phone Battery Repairer	Conversion efficiency: good<br>Compatibility: strong<br>Security: good	JD.com
Puning Dental	Cell Phone Battery	Cell phone adapter received looks good used well in buy a	JD.com
Puning Dental	Cell Phone Battery	Received the item and it looks great. The style is also beautiful and okay.	JD.com
Puning Dental	Cell Phone Battery	Friends introduced to buy, on the cell phone pool repair remarkable, worth buying.	JD.com
Puning Dental	Cell Phone Battery	pretty good	JD.com
Puning Dental	Cell Phone Battery Repairer	The goods are very delicate, logistics is also fast, the business service is enthusiastic<br>Give five-star praise	JD.com
Puning Dental	Cell Phone Battery Repairer	Great price for what you get. Will buy the good stuff again in the future. Probably would work better at a higher price. Recommended purchase has been sent to the Poundland coterie.	JD.com
Puning Dental	Cell Phone Battery	I am very happy with your merchandise and the product is great.	JD.com
Puning Dental	Cell Phone Battery	Useful to repair fast charging good quality	JD.com
Puning Dental	Cell Phone Battery	products also fraudulent consumer business or unlicensed operation!	JD.com
Puning Dental	Cell Phone Battery Repairer	Authentic battery activator, cell phone repair is very easy, product quality is quite over the top, very satisfied with the special like, the price is cheap things are good, fine workmanship style is good, the service is thoughtful shipping fast, full-star praise a hundred praise!	JD.com
Puning Dental	Cell Phone Battery Repairer	This store's repairer is good, the cell phone battery activation rate is high, authentic product stuff is good, exquisite and beautiful high quality, fast shipping and thoughtful service, praise a full star high!	JD.com
Puning Dental	Cell Phone Battery Repairer	Cell phone repairer has received, genuine activator effect, product over the quality of high, something special good spend less, very satisfied with the super like, thoughtful service delivery fast, full of stars!	JD.com
Puning Dental	Cell Phone Battery Repairer	smooth, fine workmanship kind of beautiful, product quality is quite over the top, the price is cheap things are good, the service is enthusiastic and thoughtful, fast delivery full of stars high light!	JD.com
Puning Dental	Cell Phone Battery Repairer	Cheap and affordable<br>Stability: good<br>Will repurchase again	JD.com
Puning Dental	Cell Phone Battery	Quality can be, the courier boy is very enthusiastic please you can rest assured to buy!	JD.com
Puning Dental	Cell Phone Battery	This item is great quality and fast shipping.	JD.com
Puning Dental	Cell Phone Battery	classifier for objects in rows such as words	JD.com
Puning Dental	Cell Phone Battery Repairer	Received a cell phone battery repairer, the quality is quite good, exquisite packaging, good seller service attitude, logistics and express timely, all five-star praise. Wish merchants prosperous business.	JD.com
Puning Dental	Cell Phone Battery	Well packaged, inexpensive and easy to use.	JD.com

Puning Dental	Cell Phone Battery Repairer	Charging is average, charging is so slow	JD.com
Puning Dental	Cell Phone Battery Repairer	Very good satisfied next time come back[buy buy buy][kudos][kudos]	JD.com
Puning Dental	Cell Phone Battery Repairer	This user feels the product is better	JD.com
Puning Dental	Cell Phone Battery Repairer	Review after use.	JD.com
Puning Dental	Cell Phone Battery Repairer	Battery Repairer received, quite good, good quality, carefully packaged, intact, good business attitude, logistics and express timely, all five-star praise.	JD.com
Puning Dental	Cell Phone Battery Repairer	Plus this is similar to slow charging, it's good for battery protection	JD.com
Puning Dental	Cell Phone Battery Repairer	Great price and fast shipping! Packaging was great!	JD.com
Puning Dental	Cell Phone Battery Repairer	Works great and fits perfectly. It also plugs in.	JD.com
Puning Dental	Cell Phone Battery Repairer	The quality is pretty good.	JD.com
Puning Dental	Cell Phone Battery Repairer	The stuff works great, I'll buy another one later	JD.com
Puning Dental	Cell Phone Battery Repairer	IQ tax	JD.com
Puning Dental	Cell Phone Battery Repairer	This user feels the product is better	JD.com
Puning Dental	Cell Phone Battery Repairer	[Smiles][Skimming][Big Love]	JD.com
Puning Dental	Cell Phone Battery Repairer	Baby is very good, the logistics is very fast, the quality of workmanship is very good, cheaper than the entity store, trustworthy, like friends hurry to get down to it ，，，，，，，，，，，，，，，，	JD.com
Puning Dental	Cell Phone Battery Repairer	False advertising, false products	JD.com
Puning Dental	Cell Phone Battery Repairer	I've got more memory with this. It's not laggy.	JD.com
Puning Dental	Cell Phone Battery Repairer	！！！！！！！！！！！	JD.com
Puning Dental	Cell Phone Battery Repairer	This user feels the product is better	JD.com
Puning Dental	Cell Phone Battery Repairer	This user feels the product is better	JD.com
Puning Dental	Cell Phone Battery Repairer	Use it right now and punch a laptop.	JD.com
Puning Dental	Cell Phone Battery Repairer	Good quality, very cheap, good object	JD.com
Puning Dental	Cell Phone Battery Repairer	Support the new technology, the goods have been received. The effect of the use of follow-up comments!	JD.com
Puning Dental	Cell Phone Battery Repairer	especially well, thanks to the merchant's stuff works especially well, this is especially good.[Smiles]This very satisfied. This is very, very good, this satisfaction to you to play, I good praise.	JD.com
Puning Dental	Cell Phone Battery Repairer	Thanks to the merchant's stuff works exceptionally well, really exceptionally well possesses no problems at all!	JD.com
Puning Dental	Cell Phone Battery Repairer	Please return the product if there is any problem with the quality	JD.com
Puning Dental	Cell Phone Battery Repairer	I'm using it now with good use over and over and over again.	JD.com
Puning Dental	Cell Phone Battery Repairer	Logistics is very powerful Quality is very good Immediately repurchase	JD.com
Puning Dental	Cell Phone Battery Repairer	This user feels the product is better	JD.com
Puning Dental	Cell Phone Battery Repairer	is very fast, the customer service attitude is very positive, and questions are answered in a timely manner! Overall very satisfied!	JD.com
Puning Dental	Cell Phone Battery Repairer	This user feels the product is better	JD.com
Puning Dental	Cell Phone Battery Repairer	inexpensive and good quality, fast delivery, customer service is very good too!	JD.com
Puning Dental	Cell Phone Battery Repairer	I'll wait to use it before I say how I feel about it.	JD.com
Puning Dental	Cell Phone Battery Repairer	beautifully packaged/product! It is a good adapter to improve the battery life of mobilephone/optimize cell phonesystem/clean up cell phone garbage and improverunning speed of cell phone!Trustworthy/purchaseuse! I wish the manufacturer a prosperous business! We wish you a prosperous business! Popularity is strong and prosperous![Kudos][Buy]][Good][Buy]][Clap][Hands[Ah Huang][Handshake][Tongue]Rose]	JD.com

Puning Dental	Cell Phone Battery Repairer	The item has been received, the courier is also very fast, the product quality is very good, the workmanship is very refined, is genuine, the price is also very cheap, and the description is exactly the same, I give five-star praise.	JD.com
Puning Dental	Cell Phone Battery	Comments	JD.com
Puning Dental	Cell Phone Battery	Well, I can't say yet. Let's just use it and try it out! Uh-huh!	JD.com
Puning Dental	Cell Phone Battery	It looks good.	JD.com
Puning Dental	Cell Phone Battery	Repair has received, use good re-purchase wish the store business prosperity!	JD.com
Puning Dental	Cell Phone Battery	It works well.	JD.com
Puning Dental	Cell Phone Battery Repairer	Conversion efficiency: good<br>Compatibility: strong<br>Security: good	JD.com
Puning Dental	Cell Phone Battery	Cell phone adapter received looks good used well in buy a	JD.com
Puning Dental	Cell Phone Battery	Received the item and it looks great. The style is also beautiful and okay.	JD.com
Puning Dental	Cell Phone Battery	Friends introduced to buy, on the cell phone pool repair remarkable, worth buying.	JD.com
Puning Dental	Cell Phone Battery	pretty good	JD.com
Puning Dental	Cell Phone Battery Repairer	The goods are very delicate, logistics is also fast, the business service is enthusiastic<br>Give five-star praise	JD.com
Puning Dental	Cell Phone Battery Repairer	Great price for what you get. Will buy the good stuff again in the future. Probably would work better at a higher price. Recommended purchase has been sent to the Poundland coterie.	JD.com
Puning Dental	Cell Phone Battery	I am very happy with your merchandise and the product is great.	JD.com
Puning Dental	Cell Phone Battery	Useful to repair fast charging good quality	JD.com
Puning Dental	Cell Phone Battery	products also fraudulent consumer business or unlicensed operation!	JD.com
Puning Dental	Cell Phone Battery Repairer	Authentic battery activator, cell phone repair is very easy, product quality is quite over the top, very satisfied with the special like, the price is cheap things are good, fine workmanship style is good, the service is thoughtful shipping fast, full-star praise a hundred praise!	JD.com
Puning Dental	Cell Phone Battery Repairer	This store's repairer is good, the cell phone battery activation rate is high, authentic product stuff is good, exquisite and beautiful high quality, fast shipping and thoughtful service, praise a full star high!	JD.com
Puning Dental	Cell Phone Battery Repairer	Cell phone repairer has received, genuine activator effect, product over the quality of high, something special good spend less, very satisfied with the super like, thoughtful service delivery fast, full of stars!	JD.com
Puning Dental	Cell Phone Battery Repairer	smooth, fine workmanship kind of beautiful, product quality is quite over the top, the price is cheap things are good, the service is enthusiastic and thoughtful, fast delivery full of stars high light!	JD.com
Puning Dental	Cell Phone Battery Repairer	Cheap and affordable<br>Stability: good<br>Will repurchase again	JD.com
Puning Dental	Cell Phone Battery	Quality can be, the courier boy is very enthusiastic please you can rest assured to buy!	JD.com
Puning Dental	Cell Phone Battery	This item is great quality and fast shipping.	JD.com
Puning Dental	Cell Phone Battery	classifier for objects in rows such as words	JD.com
Puning Dental	Cell Phone Battery Repairer	Received a cell phone battery repairer, the quality is quite good, exquisite packaging, good seller service attitude, logistics and express timely, all five-star praise. Wish merchants prosperous business.	JD.com
Puning Dental	Cell Phone Battery	Well packaged, inexpensive and easy to use.	JD.com
Puning Dental	Cell Phone Battery	Charging is average, charging is so slow	JD.com
Puning Dental	Cell Phone Battery	Very good satisfied next time come back[buy buy buy][kudos][kudos]	JD.com
Puning Dental	Cell Phone Battery	This user feels the product is better	JD.com
Puning Dental	Cell Phone Battery	Review after use.	JD.com

Puning Dental	Cell Phone Battery Repairer	Battery Repairer received, quite good, good quality, carefully packaged, intact, good business attitude, logistics and express timely, all five-star praise.	JD.com
Puning Dental	Cell Phone Battery Repairer	Plus this is similar to slow charging, it's good for battery protection	JD.com
Puning Dental	Cell Phone Battery Repairer	Great price and fast shipping! Packaging was great!	JD.com
Puning Dental	Cell Phone Battery Repairer	Works great and fits perfectly. It also plugs in.	JD.com
Puning Dental	Cell Phone Battery Repairer	The quality is pretty good.	JD.com
Puning Dental	Cell Phone Battery Repairer	The stuff works great, I'll buy another one later	JD.com
Puning Dental	Cell Phone Battery Repairer	IQ tax	JD.com
Puning Dental	Cell Phone Battery Repairer	This user feels the product is better	JD.com
Puning Dental	Cell Phone Battery Repairer	[Smiles][Skimming][Big Love]	JD.com
Puning Dental	Cell Phone Battery Repairer	Baby is very good, the logistics is very fast, the quality of workmanship is very good, cheaper than the entity store, trustworthy, like friends hurry to get down to it , , , , , , , , , , , , , , ,	JD.com
Puning Dental	Cell Phone Battery Repairer	False advertising, false products	JD.com
Puning Dental	Cell Phone Battery Repairer	I've got more memory with this. It's not laggy.	JD.com
Puning Dental	Cell Phone Battery Repairer	! ! ! ! ! ! ! ! ! ! !	JD.com
Puning Dental	Cell Phone Battery Repairer	This user feels the product is better	JD.com
Puning Dental	Cell Phone Battery Repairer	This user feels the product is better	JD.com
Puning Dental	Cell Phone Battery Repairer	Use it right now and punch a laptop.	JD.com
Puning Dental	Cell Phone Battery Repairer	Good quality, very cheap, good object	JD.com
Puning Dental	Cell Phone Battery Repairer	Support the new technology, the goods have been received. The effect of the use of follow-up comments!	JD.com
Puning Dental	Cell Phone Battery Repairer	especially well, thanks to the merchant's stuff works especially well, this is especially good.[Smiles]This very satisfied. This is very, very good, this satisfaction to you to play, I good praise.	JD.com
Puning Dental	Cell Phone Battery Repairer	Thanks to the merchant's stuff works exceptionally well, really exceptionally well possesses no problems at all!	JD.com
Puning Dental	Cell Phone Battery Repairer	Please return the product if there is any problem with the quality	JD.com
Puning Dental	Cell Phone Battery Repairer	I'm using it now with good use over and over and over again.	JD.com
Puning Dental	Cell Phone Battery Repairer	Logistics is very powerful Quality is very good Immediately repurchase	JD.com
Puning Dental	Cell Phone Battery Repairer	This user feels the product is better	JD.com
Puning Dental	Cell Phone Battery Repairer	is very fast, the customer service attitude is very positive, and questions are answered in a timely manner! Overall very satisfied!	JD.com
Puning Dental	Cell Phone Battery Repairer	This user feels the product is better	JD.com
Puning Dental	Cell Phone Battery Repairer	inexpensive and good quality, fast delivery, customer service is very good too!	JD.com
Puning Dental	Cell Phone Battery Repairer	I'll wait to use it before I say how I feel about it.	JD.com
Puning Dental	Cell Phone Battery Repairer	beautifully packaged/product! It is a good adapter to improve the battery life of mobilephone/optimize cell phonesystem/clean up cell phone garbage and improverunning speed of cell phone!Trustworthy/purchaseuse! I wish the manufacturer a prosperous business! We wish you a prosperous business! Popularity is strong and prosperous![Kudos][Buy]][Good][Buy]][Clap][Hands[Ah Huang][Handshake][Tongue]Rose]	JD.com
Puning Dental	Cell Phone Battery Repairer	The item has been received, the courier is also very fast, the product quality is very good, the workmanship is very refined, is genuine, the price is also very cheap, and the description is exactly the same, I give five-star praise.	JD.com
Puning Dental	Cell Phone Battery Repairer	Comments	JD.com
Puning Dental	Cell Phone Battery Repairer	Well, I can't say yet. Let's just use it and try it out! Uh-huh!	JD.com

Puning Dental	Cell Phone Battery	It looks good.	JD.com
Puning Dental	Cell Phone Battery	Repair has received, use good re-purchase wish the store business prosperity!	JD.com
Puning Dental	Cell Phone Battery	It works well.	JD.com
Puning Dental	Cell Phone Battery Repairer	Conversion efficiency: good<br>Compatibility: strong<br>Security: good	JD.com
Puning Dental	Cell Phone Battery	Cell phone adapter received looks good used well in buy a		JD.com
Puning Dental	Cell Phone Battery	Received the item and it looks great. The style is also beautiful and okay.	JD.com
Puning Dental	Cell Phone Battery	Friends introduced to buy, on the cell phone pool repair remarkable, worth buying.	JD.com
Puning Dental	Cell Phone Battery	pretty good	JD.com
Puning Dental	Cell Phone Battery Repairer	The goods are very delicate, logistics is also fast, the business service is enthusiastic<br>Give five-star praise	JD.com
Puning Dental	Cell Phone Battery Repairer	Great price for what you get. Will buy the good stuff again in the future. Probably would work better at a higher price. Recommended purchase has been sent to the Poundland coterie.	JD.com
Puning Dental	Cell Phone Battery	I am very happy with your merchandise and the product is great.	JD.com
Puning Dental	Cell Phone Battery	Useful to repair fast charging good quality	JD.com
Puning Dental	Cell Phone Battery	products also fraudulent consumer business or unlicensed operation!	JD.com
Puning Dental	Cell Phone Battery Repairer	Authentic battery activator, cell phone repair is very easy, product quality is quite over the top, very satisfied with the special like, the price is cheap things are good, fine workmanship style is good, the service is thoughtful shipping fast, full-star praise a hundred praise!	JD.com
Puning Dental	Cell Phone Battery Repairer	This store's repairer is good, the cell phone battery activation rate is high, authentic product stuff is good, exquisite and beautiful high quality, fast shipping and thoughtful service, praise a full star high!	JD.com
Puning Dental	Cell Phone Battery Repairer	Cell phone repairer has received, genuine activator effect, product over the quality of high, something special good spend less, very satisfied with the super like, thoughtful service delivery fast, full of stars!	JD.com
Puning Dental	Cell Phone Battery Repairer	smooth, fine workmanship kind of beautiful, product quality is quite over the top, the price is cheap things are good, the service is enthusiastic and thoughtful, fast delivery full of stars high light!	JD.com
Puning Dental	Cell Phone Battery Repairer	Cheap and affordable<br>Stability: good<br>Will repurchase again	JD.com
Puning Dental	Cell Phone Battery	Quality can be, the courier boy is very enthusiastic please you can rest assured to buy!	JD.com
Puning Dental	Cell Phone Battery	This item is great quality and fast shipping.	JD.com
Puning Dental	Cell Phone Battery	classifier for objects in rows such as words	JD.com
Puning Dental	Cell Phone Battery Repairer	Received a cell phone battery repairer, the quality is quite good, exquisite packaging, good seller service attitude, logistics and express timely, all five-star praise. Wish merchants prosperous business.	JD.com
Puning Dental	Cell Phone Battery	Well packaged, inexpensive and easy to use.	JD.com
Puning Dental	Cell Phone Battery	Charging is average, charging is so slow	JD.com
Puning Dental	Cell Phone Battery	Very good satisfied next time come back[buy buy buy][kudos][kudos]	JD.com
Puning Dental	Cell Phone Battery	This user feels the product is better	JD.com
Puning Dental	Cell Phone Battery	Review after use.	JD.com
Puning Dental	Cell Phone Battery Repairer	Battery Repairer received, quite good, good quality, carefully packaged, intact, good business attitude, logistics and express timely, all five-star praise.	JD.com
Puning Dental	Cell Phone Battery	Plus this is similar to slow charging, it's good for battery protection	JD.com
Puning Dental	Cell Phone Battery	Great price and fast shipping! Packaging was great!	JD.com
Puning Dental	Cell Phone Battery	Works great and fits perfectly. It also plugs in.	JD.com

Puning Dental	Cell Phone Battery	The quality is pretty good.	JD.com
Puning Dental	Cell Phone Battery	The stuff works great, I'll buy another one later	JD.com
Puning Dental	Cell Phone Battery	IQ tax	JD.com
Puning Dental	Cell Phone Battery	This user feels the product is better	JD.com
Puning Dental	Cell Phone Battery	[Smiles][Skimming][Big Love]	JD.com
Puning Dental	Cell Phone Battery Repairer	Baby is very good, the logistics is very fast, the quality of workmanship is very good, cheaper than the entity store, trustworthy, like friends hurry to get down to it ，，，，，，，，，，，，，，，	JD.com
Puning Dental	Cell Phone Battery	False advertising, false products	JD.com
Puning Dental	Cell Phone Battery	I've got more memory with this. It's not laggy.	JD.com
Puning Dental	Cell Phone Battery	！！！！！！！！！！！	JD.com
Puning Dental	Cell Phone Battery	This user feels the product is better	JD.com
Puning Dental	Cell Phone Battery	This user feels the product is better	JD.com
Puning Dental	Cell Phone Battery	Use it right now and punch a laptop.	JD.com
Puning Dental	Cell Phone Battery	Good quality, very cheap, good object	JD.com
Puning Dental	Cell Phone Battery	Support the new technology, the goods have been received. The effect of the use of follow-up comments!	JD.com
Puning Dental	Cell Phone Battery Repairer	especially well, thanks to the merchant's stuff works especially well, this is especially good.[Smiles]This very satisfied. This is very, very good, this satisfaction to you to play, I good praise.	JD.com
Puning Dental	Cell Phone Battery	Thanks to the merchant's stuff works exceptionally well, really exceptionally well possesses no problems at all!	JD.com
Puning Dental	Cell Phone Battery	Please return the product if there is any problem with the quality	JD.com
Puning Dental	Cell Phone Battery	I'm using it now with good use over and over and over again.	JD.com
Puning Dental	Cell Phone Battery	Logistics is very powerful Quality is very good Immediately repurchase	JD.com
Puning Dental	Cell Phone Battery	This user feels the product is better	JD.com
Puning Dental	Cell Phone Battery Repairer	is very fast, the customer service attitude is very positive, and questions are answered in a timely manner! Overall very satisfied!	JD.com
Puning Dental	Cell Phone Battery	This user feels the product is better	JD.com
Puning Dental	Cell Phone Battery	inexpensive and good quality, fast delivery, customer service is very good too!	JD.com
Puning Dental	Cell Phone Battery	I'll wait to use it before I say how I feel about it.	JD.com
Puning Dental	Cell Phone Battery Repairer	beautifully packaged/product! It is a good adapter to improve the battery life of mobilephone/optimize cell phonesystem/clean up cell phone garbage and improverunning speed of cell phone!Trustworthy/purchaseuse! I wish the manufacturer a prosperous business! We wish you a prosperous business! Popularity is strong and prosperous![Kudos][Buy]][Good][Buy]][Clap][Hands[Ah Huang][Handshake][Tongue]Rose]	JD.com
Puning Dental	Cell Phone Battery Repairer	The item has been received, the courier is also very fast, the product quality is very good, the workmanship is very refined, is genuine, the price is also very cheap, and the description is exactly the same, I give five-star praise.	JD.com
Puning Dental	Cell Phone Battery	Comments	JD.com
Puning Dental	Cell Phone Battery	Well, I can't say yet. Let's just use it and try it out! Uh-huh!	JD.com
Puning Dental	Cell Phone Battery	It looks good.	JD.com
Puning Dental	Cell Phone Battery	Repair has received, use good re-purchase wish the store business prosperity!	JD.com
Puning Dental	Cell Phone Battery	It works well.	JD.com
Puning Dental	Cell Phone Battery Repairer	Conversion efficiency: good Compatibility: strong Security: good	JD.com

Puning Dental	Cell Phone Battery	Cell phone adapter received looks good used well in buy a	JD.com
Puning Dental	Cell Phone Battery	Received the item and it looks great. The style is also beautiful and okay.	JD.com
Puning Dental	Cell Phone Battery	Friends introduced to buy, on the cell phone pool repair remarkable, worth buying.	JD.com
Puning Dental	Cell Phone Battery	pretty good	JD.com
Puning Dental	Cell Phone Battery Repairer	The goods are very delicate, logistics is also fast, the business service is enthusiastic<br>Give five-star praise	JD.com
Puning Dental	Cell Phone Battery Repairer	Great price for what you get. Will buy the good stuff again in the future. Probably would work better at a higher price. Recommended purchase has been sent to the Poundland coterie.	JD.com
Puning Dental	Cell Phone Battery	I am very happy with your merchandise and the product is great.	JD.com
Puning Dental	Cell Phone Battery	Useful to repair fast charging good quality	JD.com
Puning Dental	Cell Phone Battery	products also fraudulent consumer business or unlicensed operation!	JD.com
Puning Dental	Cell Phone Battery Repairer	Authentic battery activator, cell phone repair is very easy, product quality is quite over the top, very satisfied with the special like, the price is cheap things are good, fine workmanship style is good, the service is thoughtful shipping fast, full-star praise a hundred praise!	JD.com
Puning Dental	Cell Phone Battery Repairer	This store's repairer is good, the cell phone battery activation rate is high, authentic product stuff is good, exquisite and beautiful high quality, fast shipping and thoughtful service, praise a full star high!	JD.com
Puning Dental	Cell Phone Battery Repairer	Cell phone repairer has received, genuine activator effect, product over the quality of high, something special good spend less, very satisfied with the super like, thoughtful service delivery fast, full of stars!	JD.com
Puning Dental	Cell Phone Battery Repairer	smooth, fine workmanship kind of beautiful, product quality is quite over the top, the price is cheap things are good, the service is enthusiastic and thoughtful, fast delivery full of stars high light!	JD.com
Puning Dental	Cell Phone Battery Repairer	Cheap and affordable<br>Stability: good<br>Will repurchase again	JD.com
Puning Dental	Cell Phone Battery	Quality can be, the courier boy is very enthusiastic please you can rest assured to buy!	JD.com
Puning Dental	Cell Phone Battery	This item is great quality and fast shipping.	JD.com
Puning Dental	Cell Phone Battery	classifier for objects in rows such as words	JD.com
Puning Dental	Cell Phone Battery Repairer	Received a cell phone battery repairer, the quality is quite good, exquisite packaging, good seller service attitude, logistics and express timely, all five-star praise. Wish merchants prosperous business.	JD.com
Puning Dental	Cell Phone Battery	Well packaged, inexpensive and easy to use.	JD.com
Puning Dental	Cell Phone Battery	Charging is average, charging is so slow	JD.com
Puning Dental	Cell Phone Battery	Very good satisfied next time come back[buy buy buy][kudos][kudos]	JD.com
Puning Dental	Cell Phone Battery	This user feels the product is better	JD.com
Puning Dental	Cell Phone Battery	Review after use.	JD.com
Puning Dental	Cell Phone Battery Repairer	Battery Repairer received, quite good, good quality, carefully packaged, intact, good business attitude, logistics and express timely, all five-star praise.	JD.com
Puning Dental	Cell Phone Battery	Plus this is similar to slow charging, it's good for battery protection	JD.com
Puning Dental	Cell Phone Battery	Great price and fast shipping! Packaging was great!	JD.com
Puning Dental	Cell Phone Battery	Works great and fits perfectly. It also plugs in.	JD.com
Puning Dental	Cell Phone Battery	The quality is pretty good.	JD.com
Puning Dental	Cell Phone Battery	The stuff works great, I'll buy another one later	JD.com
Puning Dental	Cell Phone Battery	IQ tax	JD.com
Puning Dental	Cell Phone Battery	This user feels the product is better	JD.com
Puning Dental	Cell Phone Battery	[Smiles][Skimming][Big Love]	JD.com

Puning Dental	Cell Phone Battery Repairer	Baby is very good, the logistics is very fast, the quality of workmanship is very good, cheaper than the entity store, trustworthy, like friends hurry to get down to it , , , , , , , , , , , , , , ,	JD.com
Puning Dental	Cell Phone Battery	False advertising, false products	JD.com
Puning Dental	Cell Phone Battery	I've got more memory with this. It's not laggy.	JD.com
Puning Dental	Cell Phone Battery	! ! ! ! ! ! ! ! ! ! !	JD.com
Puning Dental	Cell Phone Battery	This user feels the product is better	JD.com
Puning Dental	Cell Phone Battery	This user feels the product is better	JD.com
Puning Dental	Cell Phone Battery	Use it right now and punch a laptop.	JD.com
Puning Dental	Cell Phone Battery	Good quality, very cheap, good object	JD.com
Puning Dental	Cell Phone Battery	Support the new technology, the goods have been received. The effect of the use of follow-up comments!	JD.com
Puning Dental	Cell Phone Battery Repairer	especially well, thanks to the merchant's stuff works especially well, this is especially good.[Smiles]This very satisfied. This is very, very good, this satisfaction to you to play, I good praise.	JD.com
Puning Dental	Cell Phone Battery	Thanks to the merchant's stuff works exceptionally well, really exceptionally well possesses no problems at all!	JD.com
Puning Dental	Cell Phone Battery	Please return the product if there is any problem with the quality	JD.com
Puning Dental	Cell Phone Battery	I'm using it now with good use over and over and over again.	JD.com
Puning Dental	Cell Phone Battery	Logistics is very powerful Quality is very good Immediately repurchase	JD.com
Puning Dental	Cell Phone Battery	This user feels the product is better	JD.com
Puning Dental	Cell Phone Battery Repairer	is very fast, the customer service attitude is very positive, and questions are answered in a timely manner! Overall very satisfied!	JD.com
Puning Dental	Cell Phone Battery	This user feels the product is better	JD.com
Puning Dental	Cell Phone Battery	inexpensive and good quality, fast delivery, customer service is very good too!	JD.com
Puning Dental	Cell Phone Battery	I'll wait to use it before I say how I feel about it.	JD.com
Puning Dental	Cell Phone Battery Repairer	beautifully packaged/product! It is a good adapter to improve the battery life of mobilephone/optimize cell phonesystem/clean up cell phone garbage and improverunning speed of cell phone!Trustworthy/purchaseuse! I wish the manufacturer a prosperous business! We wish you a prosperous business! Popularity is strong and prosperous![Kudos][Buy]][Good][Buy]][Clap][Hands[Ah Huang][Handshake][Tongue]Rose]	JD.com
Puning Dental	Cell Phone Battery Repairer	The item has been received, the courier is also very fast, the product quality is very good, the workmanship is very refined, is genuine, the price is also very cheap, and the description is exactly the same, I give five-star praise.	JD.com
Puning Dental	Cell Phone Battery	Comments	JD.com
Puning Dental	Cell Phone Battery	Well, I can't say yet. Let's just use it and try it out! Uh-huh!	JD.com
Puning Dental	Cell Phone Battery	It looks good.	JD.com
Puning Dental	Cell Phone Battery	Repair has received, use good re-purchase wish the store business prosperity!	JD.com
Puning Dental	Cell Phone Battery	It works well.	JD.com
Puning Dental	Cell Phone Battery Repairer	Conversion efficiency: good Compatibility: strong Security: good	JD.com
Puning Dental	Cell Phone Battery	Cell phone adapter received looks good used well in buy a|	JD.com
Puning Dental	Cell Phone Battery	Received the item and it looks great. The style is also beautiful and okay.	JD.com
Puning Dental	Cell Phone Battery	Friends introduced to buy, on the cell phone pool repair remarkable, worth buying.	JD.com
Puning Dental	Cell Phone Battery	pretty good	JD.com

Puning Dental	Cell Phone Battery Repairer	The goods are very delicate, logistics is also fast, the business service is enthusiastic<br>Give five-star praise	JD.com
Puning Dental	Cell Phone Battery Repairer	Great price for what you get. Will buy the good stuff again in the future. Probably would work better at a higher price. Recommended purchase has been sent to the Poundland coterie.	JD.com
Puning Dental	Cell Phone Battery	I am very happy with your merchandise and the product is great.	JD.com
Puning Dental	Cell Phone Battery	Useful to repair fast charging good quality	JD.com
Puning Dental	Cell Phone Battery	products also fraudulent consumer business or unlicensed operation!	JD.com
Puning Dental	Cell Phone Battery Repairer	Authentic battery activator, cell phone repair is very easy, product quality is quite over the top, very satisfied with the special like, the price is cheap things are good, fine workmanship style is good, the service is thoughtful shipping fast, full-star praise a hundred praise!	JD.com
Puning Dental	Cell Phone Battery Repairer	This store's repairer is good, the cell phone battery activation rate is high, authentic product stuff is good, exquisite and beautiful high quality, fast shipping and thoughtful service, praise a full star high!	JD.com
Puning Dental	Cell Phone Battery Repairer	Cell phone repairer has received, genuine activator effect, product over the quality of high, something special good spend less, very satisfied with the super like, thoughtful service delivery fast, full of stars!	JD.com
Puning Dental	Cell Phone Battery Repairer	smooth, fine workmanship kind of beautiful, product quality is quite over the top, the price is cheap things are good, the service is enthusiastic and thoughtful, fast delivery full of stars high light!	JD.com
Puning Dental	Cell Phone Battery Repairer	Cheap and affordable<br>Stability: good<br>Will repurchase again	JD.com
Puning Dental	Cell Phone Battery	Quality can be, the courier boy is very enthusiastic please you can rest assured to buy!	JD.com
Puning Dental	Cell Phone Battery	This item is great quality and fast shipping.	JD.com
Puning Dental	Cell Phone Battery	classifier for objects in rows such as words	JD.com
Puning Dental	Cell Phone Battery Repairer	Received a cell phone battery repairer, the quality is quite good, exquisite packaging, good seller service attitude, logistics and express timely, all five-star praise. Wish merchants prosperous business.	JD.com
Puning Dental	Cell Phone Battery	Well packaged, inexpensive and easy to use.	JD.com
Puning Dental	Cell Phone Battery	Charging is average, charging is so slow	JD.com
Puning Dental	Cell Phone Battery	Very good satisfied next time come back[buy buy buy][kudos][kudos]	JD.com
Puning Dental	Cell Phone Battery	This user feels the product is better	JD.com
Puning Dental	Cell Phone Battery	Review after use.	JD.com
Puning Dental	Cell Phone Battery Repairer	Battery Repairer received, quite good, good quality, carefully packaged, intact, good business attitude, logistics and express timely, all five-star praise.	JD.com
Puning Dental	Cell Phone Battery	Plus this is similar to slow charging, it's good for battery protection	JD.com
Puning Dental	Cell Phone Battery	Great price and fast shipping! Packaging was great!	JD.com
Puning Dental	Cell Phone Battery	Works great and fits perfectly. It also plugs in.	JD.com
Puning Dental	Cell Phone Battery	The quality is pretty good.	JD.com
Puning Dental	Cell Phone Battery	The stuff works great, I'll buy another one later	JD.com
Puning Dental	Cell Phone Battery	IQ tax	JD.com
Puning Dental	Cell Phone Battery	This user feels the product is better	JD.com
Puning Dental	Cell Phone Battery	[Smiles][Skimming][Big Love]	JD.com
Puning Dental	Cell Phone Battery Repairer	Baby is very good, the logistics is very fast, the quality of workmanship is very good, cheaper than the entity store, trustworthy, like friends hurry to get down to it<br>, , , , , , , , , , , , , , ,	JD.com
Puning Dental	Cell Phone Battery	False advertising, false products	JD.com
Puning Dental	Cell Phone Battery	I've got more memory with this. It's not laggy.	JD.com

Puning Dental	Cell Phone Battery	！！！！！！！！！！！	JD.com
Puning Dental	Cell Phone Battery	This user feels the product is better	JD.com
Puning Dental	Cell Phone Battery	This user feels the product is better	JD.com
Puning Dental	Cell Phone Battery	Use it right now and punch a laptop.	JD.com
Puning Dental	Cell Phone Battery	Good quality, very cheap, good object	JD.com
Puning Dental	Cell Phone Battery	Support the new technology, the goods have been received. The effect of the use of follow-up comments!	JD.com
Puning Dental	Cell Phone Battery Repairer	especially well, thanks to the merchant's stuff works especially well, this is especially good.[Smiles] This very satisfied. This is very, very good, this satisfaction to you to play, I good praise.	JD.com
Puning Dental	Cell Phone Battery	Thanks to the merchant's stuff works exceptionally well, really exceptionally well possesses no problems at all!	JD.com
Puning Dental	Cell Phone Battery	Please return the product if there is any problem with the quality	JD.com
Puning Dental	Cell Phone Battery	I'm using it now with good use over and over and over again.	JD.com
Puning Dental	Cell Phone Battery	Logistics is very powerful Quality is very good Immediately repurchase	JD.com
Puning Dental	Cell Phone Battery	This user feels the product is better	JD.com
Puning Dental	Cell Phone Battery Repairer	is very fast, the customer service attitude is very positive, and questions are answered in a timely manner! Overall very satisfied!	JD.com
Puning Dental	Cell Phone Battery	This user feels the product is better	JD.com
Puning Dental	Cell Phone Battery	inexpensive and good quality, fast delivery, customer service is very good too!	JD.com
Puning Dental	Cell Phone Battery	I'll wait to use it before I say how I feel about it.	JD.com
Puning Dental	Cell Phone Battery Repairer	beautifully packaged/product! It is a good adapter to improve the battery life of mobilephone/optimize cell phonesystem/clean up cell phone garbage and improverunning speed of cell phone!Trustworthy/purchaseuse! I wish the manufacturer a prosperous business! We wish you a prosperous business! Popularity is strong and prosperous![Kudos][Buy]][Good][Buy]][Clap][Hands[Ah Huang][Handshake][Tongue]Rose]	JD.com
Puning Dental	Cell Phone Battery Repairer	The item has been received, the courier is also very fast, the product quality is very good, the workmanship is very refined, is genuine, the price is also very cheap, and the description is exactly the same, I give five-star praise.	JD.com
Puning Dental	Cell Phone Battery	Comments	JD.com
Puning Dental	Cell Phone Battery	Well, I can't say yet. Let's just use it and try it out! Uh-huh!	JD.com
Puning Dental	Cell Phone Battery	It looks good.	JD.com
Puning Dental	Cell Phone Battery	Repair has received, use good re-purchase wish the store business prosperity!	JD.com
Puning Dental	Cell Phone Battery	It works well.	JD.com
Puning Dental	Cell Phone Battery Repairer	Conversion efficiency: good<br>Compatibility: strong<br>Security: good	JD.com
Puning Dental	Cell Phone Battery	Cell phone adapter received looks good used well in buy a |	JD.com
Puning Dental	Cell Phone Battery	Received the item and it looks great. The style is also beautiful and okay.	JD.com
Puning Dental	Cell Phone Battery	Friends introduced to buy, on the cell phone pool repair remarkable, worth buying.	JD.com
Puning Dental	Cell Phone Battery	pretty good	JD.com
Puning Dental	Cell Phone Battery Repairer	The goods are very delicate, logistics is also fast, the business service is enthusiastic<br>Give five-star praise	JD.com
Puning Dental	Cell Phone Battery Repairer	Great price for what you get. Will buy the good stuff again in the future. Probably would work better at a higher price. Recommended purchase has been sent to the Poundland coterie.	JD.com
Puning Dental	Cell Phone Battery	I am very happy with your merchandise and the product is great.	JD.com
Puning Dental	Cell Phone Battery	Useful to repair fast charging good quality	JD.com

Puning Dental	Cell Phone Battery	products also fraudulent consumer business or unlicensed operation!	JD.com
Puning Dental	Cell Phone Battery Repairer	Authentic battery activator, cell phone repair is very easy, product quality is quite over the top, very satisfied with the special like, the price is cheap things are good, fine workmanship style is good, the service is thoughtful shipping fast, full-star praise a hundred praise!	JD.com
Puning Dental	Cell Phone Battery Repairer	This store's repairer is good, the cell phone battery activation rate is high, authentic product stuff is good, exquisite and beautiful high quality, fast shipping and thoughtful service, praise a full star high!	JD.com
Puning Dental	Cell Phone Battery Repairer	Cell phone repairer has received, genuine activator effect, product over the quality of high, something special good spend less, very satisfied with the super like, thoughtful service delivery fast, full of stars!	JD.com
Puning Dental	Cell Phone Battery Repairer	smooth, fine workmanship kind of beautiful, product quality is quite over the top, the price is cheap things are good, the service is enthusiastic and thoughtful, fast delivery full of stars high light!	JD.com
Puning Dental	Cell Phone Battery Repairer	Cheap and affordable<br>Stability: good<br>Will repurchase again	JD.com
Puning Dental	Cell Phone Battery	Quality can be, the courier boy is very enthusiastic please you can rest assured to buy!	JD.com
Puning Dental	Cell Phone Battery	This item is great quality and fast shipping.	JD.com
Puning Dental	Cell Phone Battery	classifier for objects in rows such as words	JD.com
Puning Dental	Cell Phone Battery Repairer	Received a cell phone battery repairer, the quality is quite good, exquisite packaging, good seller service attitude, logistics and express timely, all five-star praise. Wish merchants prosperous business.	JD.com
Puning Dental	Cell Phone Battery	Well packaged, inexpensive and easy to use.	JD.com
Puning Dental	Cell Phone Battery	Charging is average, charging is so slow	JD.com
Puning Dental	Cell Phone Battery	Very good satisfied next time come back[buy buy buy][kudos][kudos]	JD.com
Puning Dental	Cell Phone Battery	This user feels the product is better	JD.com
Puning Dental	Cell Phone Battery	Review after use.	JD.com
Puning Dental	Cell Phone Battery Repairer	Battery Repairer received, quite good, good quality, carefully packaged, intact, good business attitude, logistics and express timely, all five-star praise.	JD.com
Puning Dental	Cell Phone Battery	Plus this is similar to slow charging, it's good for battery protection	JD.com
Puning Dental	Cell Phone Battery	Great price and fast shipping! Packaging was great!	JD.com
Puning Dental	Cell Phone Battery	Works great and fits perfectly. It also plugs in.	JD.com
Puning Dental	Cell Phone Battery	The quality is pretty good.	JD.com
Puning Dental	Cell Phone Battery	The stuff works great, I'll buy another one later	JD.com
Puning Dental	Cell Phone Battery	IQ tax	JD.com
Puning Dental	Cell Phone Battery	This user feels the product is better	JD.com
Puning Dental	Cell Phone Battery	[Smiles][Skimming][Big Love]	JD.com
Puning Dental	Cell Phone Battery Repairer	Baby is very good, the logistics is very fast, the quality of workmanship is very good, cheaper than the entity store, trustworthy, like friends hurry to get down to it , , , , , , , , , , , , , , ,	JD.com
Puning Dental	Cell Phone Battery	False advertising, false products	JD.com
Puning Dental	Cell Phone Battery	I've got more memory with this. It's not laggy.	JD.com
Puning Dental	Cell Phone Battery	! ! ! ! ! ! ! ! ! ! !	JD.com
Puning Dental	Cell Phone Battery	This user feels the product is better	JD.com
Puning Dental	Cell Phone Battery	This user feels the product is better	JD.com
Puning Dental	Cell Phone Battery	Use it right now and punch a laptop.	JD.com
Puning Dental	Cell Phone Battery	Good quality, very cheap, good object	JD.com

Puning Dental	Cell Phone Battery	Support the new technology, the goods have been received. The effect of the use of follow-up comments!	JD.com
Puning Dental	Cell Phone Battery Repairer	especially well, thanks to the merchant's stuff works especially well, this is especially good.[Smiles]This very satisfied. This is very, very good, this satisfaction to you to play, I good praise.	JD.com
Puning Dental	Cell Phone Battery	Thanks to the merchant's stuff works exceptionally well, really exceptionally well possesses no problems at all!	JD.com
Puning Dental	Cell Phone Battery	Please return the product if there is any problem with the quality	JD.com
Puning Dental	Cell Phone Battery	I'm using it now with good use over and over and over again.	JD.com
Puning Dental	Cell Phone Battery	Logistics is very powerful Quality is very good Immediately repurchase	JD.com
Puning Dental	Cell Phone Battery	This user feels the product is better	JD.com
Puning Dental	Cell Phone Battery Repairer	is very fast, the customer service attitude is very positive, and questions are answered in a timely manner! Overall very satisfied!	JD.com
Puning Dental	Cell Phone Battery	This user feels the product is better	JD.com
Puning Dental	Cell Phone Battery	inexpensive and good quality, fast delivery, customer service is very good too!	JD.com
Puning Dental	Cell Phone Battery	I'll wait to use it before I say how I feel about it.	JD.com
Puning Dental	Cell Phone Battery Repairer	beautifully packaged/product! It is a good adapter to improve the battery life of mobilephone/optimize cell phonesystem/clean up cell phone garbage and improverunning speed of cell phone!Trustworthy/purchaseuse! I wish the manufacturer a prosperous business! We wish you a prosperous business! Popularity is strong and prosperous![Kudos][Buy]][Good][Buy]][Clap][Hands[Ah Huang][Handshake][Tongue]Rose]	JD.com
Puning Dental	Cell Phone Battery Repairer	The item has been received, the courier is also very fast, the product quality is very good, the workmanship is very refined, is genuine, the price is also very cheap, and the description is exactly the same, I give five-star praise.	JD.com
Puning Dental	Cell Phone Battery	Comments	JD.com
Puning Dental	Cell Phone Battery	Well, I can't say yet. Let's just use it and try it out! Uh-huh!	JD.com
Puning Dental	Cell Phone Battery	It looks good.	JD.com
Puning Dental	Cell Phone Battery	Repair has received, use good re-purchase wish the store business prosperity!	JD.com
Puning Dental	Cell Phone Battery	It works well.	JD.com
Puning Dental	Cell Phone Battery Repairer	Conversion efficiency: good<br>Compatibility: strong<br>Security: good	JD.com
Puning Dental	Cell Phone Battery	Cell phone adapter received looks good used well in buy a|	JD.com
Puning Dental	Cell Phone Battery	Received the item and it looks great. The style is also beautiful and okay.	JD.com
Puning Dental	Cell Phone Battery	Friends introduced to buy, on the cell phone pool repair remarkable, worth buying.	JD.com
Puning Dental	Cell Phone Battery	pretty good	JD.com
Puning Dental	Cell Phone Battery Repairer	The goods are very delicate, logistics is also fast, the business service is enthusiastic<br>Give five-star praise	JD.com
Puning Dental	Cell Phone Battery Repairer	Great price for what you get. Will buy the good stuff again in the future. Probably would work better at a higher price. Recommended purchase has been sent to the Poundland coterie.	JD.com
Puning Dental	Cell Phone Battery	I am very happy with your merchandise and the product is great.	JD.com
Puning Dental	Cell Phone Battery	Useful to repair fast charging good quality	JD.com
Puning Dental	Cell Phone Battery	products also fraudulent consumer business or unlicensed operation!	JD.com
Puning Dental	Cell Phone Battery Repairer	Authentic battery activator, cell phone repair is very easy, product quality is quite over the top, very satisfied with the special like, the price is cheap things are good, fine workmanship style is good, the service is thoughtful shipping fast, full-star praise a hundred praise!	JD.com

Puning Dental	Cell Phone Battery Repairer	This store's repairer is good, the cell phone battery activation rate is high, authentic product stuff is good, exquisite and beautiful high quality, fast shipping and thoughtful service, praise a full star high!	JD.com
Puning Dental	Cell Phone Battery Repairer	Cell phone repairer has received, genuine activator effect, product over the quality of high, something special good spend less, very satisfied with the super like, thoughtful service delivery fast, full of stars!	JD.com
Puning Dental	Cell Phone Battery Repairer	smooth, fine workmanship kind of beautiful, product quality is quite over the top, the price is cheap things are good, the service is enthusiastic and thoughtful, fast delivery full of stars high light!	JD.com
Puning Dental	Cell Phone Battery Repairer	Cheap and affordable<br>Stability: good<br>Will repurchase again	JD.com
Puning Dental	Cell Phone Battery	Quality can be, the courier boy is very enthusiastic please you can rest assured to buy!	JD.com
Puning Dental	Cell Phone Battery	This item is great quality and fast shipping.	JD.com
Puning Dental	Cell Phone Battery	classifier for objects in rows such as words	JD.com
Puning Dental	Cell Phone Battery Repairer	Received a cell phone battery repairer, the quality is quite good, exquisite packaging, good seller service attitude, logistics and express timely, all five-star praise. Wish merchants prosperous business.	JD.com
Puning Dental	Cell Phone Battery	Well packaged, inexpensive and easy to use.	JD.com
Puning Dental	Cell Phone Battery	Charging is average, charging is so slow	JD.com
Puning Dental	Cell Phone Battery	Very good satisfied next time come back[buy buy buy][kudos][kudos]	JD.com
Puning Dental	Cell Phone Battery	This user feels the product is better	JD.com
Puning Dental	Cell Phone Battery	Review after use.	JD.com
Puning Dental	Cell Phone Battery Repairer	Battery Repairer received, quite good, good quality, carefully packaged, intact, good business attitude, logistics and express timely, all five-star praise.	JD.com
Puning Dental	Cell Phone Battery	Plus this is similar to slow charging, it's good for battery protection	JD.com
Puning Dental	Cell Phone Battery	Great price and fast shipping! Packaging was great!	JD.com
Puning Dental	Cell Phone Battery	Works great and fits perfectly. It also plugs in.	JD.com
Puning Dental	Cell Phone Battery	The quality is pretty good.	JD.com
Puning Dental	Cell Phone Battery	The stuff works great, I'll buy another one later	JD.com
Puning Dental	Cell Phone Battery	IQ tax	JD.com
Puning Dental	Cell Phone Battery	This user feels the product is better	JD.com
Puning Dental	Cell Phone Battery	[Smiles][Skimming][Big Love]	JD.com
Puning Dental	Cell Phone Battery Repairer	Baby is very good, the logistics is very fast, the quality of workmanship is very good, cheaper than the entity store, trustworthy, like friends hurry to get down to it , , , , , , , , , , , , , , ,	JD.com
Puning Dental	Cell Phone Battery	False advertising, false products	JD.com
Puning Dental	Cell Phone Battery	I've got more memory with this. It's not laggy.	JD.com
Puning Dental	Cell Phone Battery	! ! ! ! ! ! ! ! ! ! !	JD.com
Puning Dental	Cell Phone Battery	This user feels the product is better	JD.com
Puning Dental	Cell Phone Battery	This user feels the product is better	JD.com
Puning Dental	Cell Phone Battery	Use it right now and punch a laptop.	JD.com
Puning Dental	Cell Phone Battery	Good quality, very cheap, good object	JD.com
Puning Dental	Cell Phone Battery	Support the new technology, the goods have been received. The effect of the use of follow-up comments!	JD.com
Puning Dental	Cell Phone Battery Repairer	especially well, thanks to the merchant's stuff works especially well, this is especially good.[Smiles]This very satisfied. This is very, very good, this satisfaction to you to play, I good praise.	JD.com

Puning Dental	Cell Phone Battery	Thanks to the merchant's stuff works exceptionally well, really exceptionally well possesses no problems at all!	JD.com
Puning Dental	Cell Phone Battery	Please return the product if there is any problem with the quality	JD.com
Puning Dental	Cell Phone Battery	I'm using it now with good use over and over and over again.	JD.com
Puning Dental	Cell Phone Battery	Logistics is very powerful Quality is very good Immediately repurchase	JD.com
Puning Dental	Cell Phone Battery	This user feels the product is better	JD.com
Puning Dental	Cell Phone Battery Repairer	is very fast, the customer service attitude is very positive, and questions are answered in a timely manner! Overall very satisfied!	JD.com
Puning Dental	Cell Phone Battery	This user feels the product is better	JD.com
Puning Dental	Cell Phone Battery	inexpensive and good quality, fast delivery, customer service is very good too!	JD.com
Puning Dental	Cell Phone Battery	I'll wait to use it before I say how I feel about it.	JD.com
Puning Dental	Cell Phone Battery Repairer	beautifully packaged/product! It is a good adapter to improve the battery life of mobilephone/optimize cell phonesystem/clean up cell phone garbage and improverunning speed of cell phone! Trustworthy/purchaseuse! I wish the manufacturer a prosperous business! We wish you a prosperous business! Popularity is strong and prosperous! [Kudos][Buy]][Good][Buy]][Clap][Hands[Ah Huang][Handshake][Tongue]Rose]	JD.com
Puning Dental	Cell Phone Battery Repairer	The item has been received, the courier is also very fast, the product quality is very good, the workmanship is very refined, is genuine, the price is also very cheap, and the description is exactly the same, I give five-star praise.	JD.com
Puning Dental	Cell Phone Battery	Comments	JD.com
Puning Dental	Cell Phone Battery	Well, I can't say yet. Let's just use it and try it out! Uh-huh!	JD.com
Puning Dental	Cell Phone Battery	It looks good.	JD.com
Puning Dental	Cell Phone Battery	Repair has received, use good re-purchase wish the store business prosperity!	JD.com
Puning Dental	Cell Phone Battery	It works well.	JD.com
Puning Dental	Cell Phone Battery Repairer	Conversion efficiency: good<br>Compatibility: strong<br>Security: good	JD.com
Puning Dental	Cell Phone Battery	Cell phone adapter received looks good used well in buy a	JD.com
Puning Dental	Cell Phone Battery	Received the item and it looks great. The style is also beautiful and okay.	JD.com
Puning Dental	Cell Phone Battery	Friends introduced to buy, on the cell phone pool repair remarkable, worth buying.	JD.com
Puning Dental	Cell Phone Battery	pretty good	JD.com
Puning Dental	Cell Phone Battery Repairer	The goods are very delicate, logistics is also fast, the business service is enthusiastic<br>Give five-star praise	JD.com
Puning Dental	Cell Phone Battery Repairer	Great price for what you get. Will buy the good stuff again in the future. Probably would work better at a higher price. Recommended purchase has been sent to the Poundland coterie.	JD.com
Puning Dental	Cell Phone Battery	I am very happy with your merchandise and the product is great.	JD.com
Puning Dental	Cell Phone Battery	Useful to repair fast charging good quality	JD.com
Puning Dental	Cell Phone Battery	products also fraudulent consumer business or unlicensed operation!	JD.com
Puning Dental	Cell Phone Battery Repairer	Authentic battery activator, cell phone repair is very easy, product quality is quite over the top, very satisfied with the special like, the price is cheap things are good, fine workmanship style is good, the service is thoughtful shipping fast, full-star praise a hundred praise!	JD.com
Puning Dental	Cell Phone Battery Repairer	This store's repairer is good, the cell phone battery activation rate is high, authentic product stuff is good, exquisite and beautiful high quality, fast shipping and thoughtful service, praise a full star high!	JD.com
Puning Dental	Cell Phone Battery Repairer	Cell phone repairer has received, genuine activator effect, product over the quality of high, something special good spend less, very satisfied with the super like, thoughtful service delivery fast, full of stars!	JD.com

Puning Dental	Cell Phone Battery Repairer	smooth, fine workmanship kind of beautiful, product quality is quite over the top, the price is cheap things are good, the service is enthusiastic and thoughtful, fast delivery full of stars high light!	JD.com
Puning Dental	Cell Phone Battery Repairer	Cheap and affordable<br>Stability: good<br>Will repurchase again	JD.com
Puning Dental	Cell Phone Battery	Quality can be, the courier boy is very enthusiastic please you can rest assured to buy!	JD.com
Puning Dental	Cell Phone Battery	This item is great quality and fast shipping.	JD.com
Puning Dental	Cell Phone Battery	classifier for objects in rows such as words	JD.com
Puning Dental	Cell Phone Battery Repairer	Received a cell phone battery repairer, the quality is quite good, exquisite packaging, good seller service attitude, logistics and express timely, all five-star praise. Wish merchants prosperous business.	JD.com
Puning Dental	Cell Phone Battery	Well packaged, inexpensive and easy to use.	JD.com
Puning Dental	Cell Phone Battery	Charging is average, charging is so slow	JD.com
Puning Dental	Cell Phone Battery	Very good satisfied next time come back[buy buy buy][kudos][kudos]	JD.com
Puning Dental	Cell Phone Battery	This user feels the product is better	JD.com
Puning Dental	Cell Phone Battery	Review after use.	JD.com
Puning Dental	Cell Phone Battery Repairer	Battery Repairer received, quite good, good quality, carefully packaged, intact, good business attitude, logistics and express timely, all five-star praise.	JD.com
Puning Dental	Cell Phone Battery	Plus this is similar to slow charging, it's good for battery protection	JD.com
Puning Dental	Cell Phone Battery	Great price and fast shipping! Packaging was great!	JD.com
Puning Dental	Cell Phone Battery	Works great and fits perfectly. It also plugs in.	JD.com
Puning Dental	Cell Phone Battery	The quality is pretty good.	JD.com
Puning Dental	Cell Phone Battery	The stuff works great, I'll buy another one later	JD.com
Puning Dental	Cell Phone Battery	IQ tax	JD.com
Puning Dental	Cell Phone Battery	This user feels the product is better	JD.com
Puning Dental	Cell Phone Battery	[Smiles][Skimming][Big Love]	JD.com
Puning Dental	Cell Phone Battery Repairer	Baby is very good, the logistics is very fast, the quality of workmanship is very good, cheaper than the entity store, trustworthy, like friends hurry to get down to it , , , , , , , , , , , , , , ,	JD.com
Puning Dental	Cell Phone Battery	False advertising, false products	JD.com
Puning Dental	Cell Phone Battery	I've got more memory with this. It's not laggy.	JD.com
Puning Dental	Cell Phone Battery	! ! ! ! ! ! ! ! ! ! !	JD.com
Puning Dental	Cell Phone Battery	This user feels the product is better	JD.com
Puning Dental	Cell Phone Battery	This user feels the product is better	JD.com
Puning Dental	Cell Phone Battery	Use it right now and punch a laptop.	JD.com
Puning Dental	Cell Phone Battery	Good quality, very cheap, good object	JD.com
Puning Dental	Cell Phone Battery	Support the new technology, the goods have been received. The effect of the use of follow-up comments!	JD.com
Puning Dental	Cell Phone Battery Repairer	especially well, thanks to the merchant's stuff works especially well, this is especially good.[Smiles]This very satisfied. This is very, very good, this satisfaction to you to play, I good praise.	JD.com
Puning Dental	Cell Phone Battery	Thanks to the merchant's stuff works exceptionally well, really exceptionally well possesses no problems at all!	JD.com
Puning Dental	Cell Phone Battery	Please return the product if there is any problem with the quality	JD.com
Puning Dental	Cell Phone Battery	I'm using it now with good use over and over and over again.	JD.com
Puning Dental	Cell Phone Battery	Logistics is very powerful Quality is very good Immediately repurchase	JD.com

Puning Dental	Cell Phone Battery	This user feels the product is better	JD.com
Puning Dental	Cell Phone Battery Repairer	is very fast, the customer service attitude is very positive, and questions are answered in a timely manner! Overall very satisfied!	JD.com
Puning Dental	Cell Phone Battery	This user feels the product is better	JD.com
Puning Dental	Cell Phone Battery	inexpensive and good quality, fast delivery, customer service is very good too!	JD.com
Puning Dental	Cell Phone Battery	I'll wait to use it before I say how I feel about it.	JD.com
Puning Dental	Cell Phone Battery Repairer	beautifully packaged/product! It is a good adapter to improve the battery life of mobilephone/optimize cell phonesystem/clean up cell phone garbage and improverunning speed of cell phone!Trustworthy/purchaseuse! I wish the manufacturer a prosperous business! We wish you a prosperous business! Popularity is strong and prosperous![Kudos][Buy]][Good][Buy]][Clap][Hands[Ah Huang][Handshake][Tongue]Rose]	JD.com
Puning Dental	Cell Phone Battery Repairer	The item has been received, the courier is also very fast, the product quality is very good, the workmanship is very refined, is genuine, the price is also very cheap, and the description is exactly the same, I give five-star praise.	JD.com
Puning Dental	Cell Phone Battery	Comments	JD.com
Puning Dental	Cell Phone Battery	Well, I can't say yet. Let's just use it and try it out! Uh-huh!	JD.com
Puning Dental	Cell Phone Battery	It looks good.	JD.com
Puning Dental	Cell Phone Battery	Repair has received, use good re-purchase wish the store business prosperity!	JD.com
Puning Dental	Cell Phone Battery	It works well.	JD.com
Puning Dental	Cell Phone Battery Repairer	Conversion efficiency: good<br>Compatibility: strong<br>Security: good	JD.com
Puning Dental	Cell Phone Battery	Cell phone adapter received looks good used well in buy a	JD.com
Puning Dental	Cell Phone Battery	Received the item and it looks great. The style is also beautiful and okay.	JD.com
Puning Dental	Cell Phone Battery	Friends introduced to buy, on the cell phone pool repair remarkable, worth buying.	JD.com
Puning Dental	Cell Phone Battery	pretty good	JD.com
Puning Dental	Cell Phone Battery Repairer	The goods are very delicate, logistics is also fast, the business service is enthusiastic<br>Give five-star praise	JD.com
Puning Dental	Cell Phone Battery Repairer	Great price for what you get. Will buy the good stuff again in the future. Probably would work better at a higher price. Recommended purchase has been sent to the Poundland coterie.	JD.com
Puning Dental	Cell Phone Battery	I am very happy with your merchandise and the product is great.	JD.com
Puning Dental	Cell Phone Battery	Useful to repair fast charging good quality	JD.com
Puning Dental	Cell Phone Battery	products also fraudulent consumer business or unlicensed operation!	JD.com
Puning Dental	Cell Phone Battery Repairer	Authentic battery activator, cell phone repair is very easy, product quality is quite over the top, very satisfied with the special like, the price is cheap things are good, fine workmanship style is good, the service is thoughtful shipping fast, full-star praise a hundred praise!	JD.com
Puning Dental	Cell Phone Battery Repairer	This store's repairer is good, the cell phone battery activation rate is high, authentic product stuff is good, exquisite and beautiful high quality, fast shipping and thoughtful service, praise a full star high!	JD.com
Puning Dental	Cell Phone Battery Repairer	Cell phone repairer has received, genuine activator effect, product over the quality of high, something special good spend less, very satisfied with the super like, thoughtful service delivery fast, full of stars!	JD.com
Puning Dental	Cell Phone Battery Repairer	smooth, fine workmanship kind of beautiful, product quality is quite over the top, the price is cheap things are good, the service is enthusiastic and thoughtful, fast delivery full of stars high light!	JD.com
Puning Dental	Cell Phone Battery Repairer	Cheap and affordable<br>Stability: good<br>Will repurchase again	JD.com
Puning Dental	Cell Phone Battery	Quality can be, the courier boy is very enthusiastic please you can rest assured to buy!	JD.com

Puning Dental	Cell Phone Battery	This item is great quality and fast shipping.	JD.com
Puning Dental	Cell Phone Battery	classifier for objects in rows such as words	JD.com
Puning Dental	Cell Phone Battery Repairer	Received a cell phone battery repairer, the quality is quite good, exquisite packaging, good seller service attitude, logistics and express timely, all five-star praise. Wish merchants prosperous business.	JD.com
Puning Dental	Cell Phone Battery	Well packaged, inexpensive and easy to use.	JD.com
Puning Dental	Cell Phone Battery	Charging is average, charging is so slow	JD.com
Puning Dental	Cell Phone Battery	Very good satisfied next time come back[buy buy buy][kudos][kudos]	JD.com
Puning Dental	Cell Phone Battery	This user feels the product is better	JD.com
Puning Dental	Cell Phone Battery	Review after use.	JD.com
Puning Dental	Cell Phone Battery Repairer	Battery Repairer received, quite good, good quality, carefully packaged, intact, good business attitude, logistics and express timely, all five-star praise.	JD.com
Puning Dental	Cell Phone Battery	Plus this is similar to slow charging, it's good for battery protection	JD.com
Puning Dental	Cell Phone Battery	Great price and fast shipping! Packaging was great!	JD.com
Puning Dental	Cell Phone Battery	Works great and fits perfectly. It also plugs in.	JD.com
Puning Dental	Cell Phone Battery	The quality is pretty good.	JD.com
Puning Dental	Cell Phone Battery	The stuff works great, I'll buy another one later	JD.com
Puning Dental	Cell Phone Battery	IQ tax	JD.com
Puning Dental	Cell Phone Battery	This user feels the product is better	JD.com
Puning Dental	Cell Phone Battery	[Smiles][Skimming][Big Love]	JD.com
Puning Dental	Cell Phone Battery Repairer	Baby is very good, the logistics is very fast, the quality of workmanship is very good, cheaper than the entity store, trustworthy, like friends hurry to get down to it , , , , , , , , , , , , , , ,	JD.com
Puning Dental	Cell Phone Battery	False advertising, false products	JD.com
Puning Dental	Cell Phone Battery	I've got more memory with this. It's not laggy.	JD.com
Puning Dental	Cell Phone Battery	! ! ! ! ! ! ! ! ! ! !	JD.com
Puning Dental	Cell Phone Battery	This user feels the product is better	JD.com
Puning Dental	Cell Phone Battery	This user feels the product is better	JD.com
Puning Dental	Cell Phone Battery	Use it right now and punch a laptop.	JD.com
Puning Dental	Cell Phone Battery	Good quality, very cheap, good object	JD.com
Puning Dental	Cell Phone Battery	Support the new technology, the goods have been received. The effect of the use of follow-up comments!	JD.com
Puning Dental	Cell Phone Battery Repairer	especially well, thanks to the merchant's stuff works especially well, this is especially good.[Smiles]This very satisfied. This is very, very good, this satisfaction to you to play, I good praise.	JD.com
Puning Dental	Cell Phone Battery	Thanks to the merchant's stuff works exceptionally well, really exceptionally well possesses no problems at all!	JD.com
Puning Dental	Cell Phone Battery	Please return the product if there is any problem with the quality	JD.com
Puning Dental	Cell Phone Battery	I'm using it now with good use over and over and over again.	JD.com
Puning Dental	Cell Phone Battery	Logistics is very powerful Quality is very good Immediately repurchase	JD.com
Puning Dental	Cell Phone Battery	This user feels the product is better	JD.com
Puning Dental	Cell Phone Battery Repairer	is very fast, the customer service attitude is very positive, and questions are answered in a timely manner! Overall very satisfied!	JD.com
Puning Dental	Cell Phone Battery	This user feels the product is better	JD.com
Puning Dental	Cell Phone Battery	inexpensive and good quality, fast delivery, customer service is very good too!	JD.com

Puning Dental	Cell Phone Battery	I'll wait to use it before I say how I feel about it.	JD.com
Puning Dental	Cell Phone Battery Repairer	beautifully packaged/product! It is a good adapter to improve the battery life of mobilephone/optimize cell phonesystem/clean up cell phone garbage and improverunning speed of cell phone!Trustworthy/purchaseuse! I wish the manufacturer a prosperous business! We wish you a prosperous business! Popularity is strong and prosperous![Kudos][Buy]][Good][Buy]][Clap][Hands[Ah Huang][Handshake][Tongue]Rose]	JD.com
Puning Dental	Cell Phone Battery Repairer	The item has been received, the courier is also very fast, the product quality is very good, the workmanship is very refined, is genuine, the price is also very cheap, and the description is exactly the same, I give five-star praise.	JD.com
Puning Dental	Cell Phone Battery	Comments	JD.com
Puning Dental	Cell Phone Battery	Well, I can't say yet. Let's just use it and try it out! Uh-huh!	JD.com
Puning Dental	Cell Phone Battery	It looks good.	JD.com
Puning Dental	Cell Phone Battery	Repair has received, use good re-purchase wish the store business prosperity!	JD.com
Puning Dental	Cell Phone Battery	It works well.	JD.com
Puning Dental	Cell Phone Battery Repairer	Conversion efficiency: good<br>Compatibility: strong<br>Security: good	JD.com
Puning Dental	Cell Phone Battery	Cell phone adapter received looks good used well in buy a	JD.com
Puning Dental	Cell Phone Battery	Received the item and it looks great. The style is also beautiful and okay.	JD.com
Puning Dental	Cell Phone Battery	Friends introduced to buy, on the cell phone pool repair remarkable, worth buying.	JD.com
Puning Dental	Cell Phone Battery	pretty good	JD.com
Puning Dental	Cell Phone Battery Repairer	The goods are very delicate, logistics is also fast, the business service is enthusiastic<br>Give five-star praise	JD.com
Puning Dental	Cell Phone Battery Repairer	Great price for what you get. Will buy the good stuff again in the future. Probably would work better at a higher price. Recommended purchase has been sent to the Poundland coterie.	JD.com
Puning Dental	Cell Phone Battery	I am very happy with your merchandise and the product is great.	JD.com
Puning Dental	Cell Phone Battery	Useful to repair fast charging good quality	JD.com
Puning Dental	Cell Phone Battery	products also fraudulent consumer business or unlicensed operation!	JD.com
Puning Dental	Cell Phone Battery Repairer	Authentic battery activator, cell phone repair is very easy, product quality is quite over the top, very satisfied with the special like, the price is cheap things are good, fine workmanship style is good, the service is thoughtful shipping fast, full-star praise a hundred praise!	JD.com
Puning Dental	Cell Phone Battery Repairer	This store's repairer is good, the cell phone battery activation rate is high, authentic product stuff is good, exquisite and beautiful high quality, fast shipping and thoughtful service, praise a full star high!	JD.com
Puning Dental	Cell Phone Battery Repairer	Cell phone repairer has received, genuine activator effect, product over the quality of high, something special good spend less, very satisfied with the super like, thoughtful service delivery fast, full of stars!	JD.com
Puning Dental	Cell Phone Battery Repairer	smooth, fine workmanship kind of beautiful, product quality is quite over the top, the price is cheap things are good, the service is enthusiastic and thoughtful, fast delivery full of stars high light!	JD.com
Puning Dental	Cell Phone Battery Repairer	Cheap and affordable<br>Stability: good<br>Will repurchase again	JD.com
Puning Dental	Cell Phone Battery	Quality can be, the courier boy is very enthusiastic please you can rest assured to buy!	JD.com
Puning Dental	Cell Phone Battery	This item is great quality and fast shipping.	JD.com
Puning Dental	Cell Phone Battery	classifier for objects in rows such as words	JD.com
Puning Dental	Cell Phone Battery Repairer	Received a cell phone battery repairer, the quality is quite good, exquisite packaging, good seller service attitude, logistics and express timely, all five-star praise. Wish merchants prosperous business.	JD.com

Puning Dental	Cell Phone Battery	Well packaged, inexpensive and easy to use.	JD.com
Puning Dental	Cell Phone Battery	Charging is average, charging is so slow	JD.com
Puning Dental	Cell Phone Battery	Very good satisfied next time come back[buy buy buy][kudos][kudos]	JD.com
Puning Dental	Cell Phone Battery	This user feels the product is better	JD.com
Puning Dental	Cell Phone Battery	Review after use.	JD.com
Puning Dental	Cell Phone Battery Repairer	Battery Repairer received, quite good, good quality, carefully packaged, intact, good business attitude, logistics and express timely, all five-star praise.	JD.com
Puning Dental	Cell Phone Battery	Plus this is similar to slow charging, it's good for battery protection	JD.com
Puning Dental	Cell Phone Battery	Great price and fast shipping! Packaging was great!	JD.com
Puning Dental	Cell Phone Battery	Works great and fits perfectly. It also plugs in.	JD.com
Puning Dental	Cell Phone Battery	The quality is pretty good.	JD.com
Puning Dental	Cell Phone Battery	The stuff works great, I'll buy another one later	JD.com
Puning Dental	Cell Phone Battery	IQ tax	JD.com
Puning Dental	Cell Phone Battery	This user feels the product is better	JD.com
Puning Dental	Cell Phone Battery	[Smiles][Skimming][Big Love]	JD.com
Puning Dental	Cell Phone Battery Repairer	Baby is very good, the logistics is very fast, the quality of workmanship is very good, cheaper than the entity store, trustworthy, like friends hurry to get down to it , , , , , , , , , , , , , , ,	JD.com
Puning Dental	Cell Phone Battery	False advertising, false products	JD.com
Puning Dental	Cell Phone Battery	I've got more memory with this. It's not laggy.	JD.com
Puning Dental	Cell Phone Battery	! ! ! ! ! ! ! ! ! ! !	JD.com
Puning Dental	Cell Phone Battery	This user feels the product is better	JD.com
Puning Dental	Cell Phone Battery	This user feels the product is better	JD.com
Puning Dental	Cell Phone Battery	Use it right now and punch a laptop.	JD.com
Puning Dental	Cell Phone Battery	Good quality, very cheap, good object	JD.com
Puning Dental	Cell Phone Battery	Support the new technology, the goods have been received. The effect of the use of follow-up comments!	JD.com
Puning Dental	Cell Phone Battery Repairer	especially well, thanks to the merchant's stuff works especially well, this is especially good.[Smiles]This very satisfied. This is very, very good, this satisfaction to you to play, I good praise.	JD.com
Puning Dental	Cell Phone Battery	Thanks to the merchant's stuff works exceptionally well, really exceptionally well possesses no problems at all!	JD.com
Puning Dental	Cell Phone Battery	Please return the product if there is any problem with the quality	JD.com
Puning Dental	Cell Phone Battery	I'm using it now with good use over and over and over again.	JD.com
Puning Dental	Cell Phone Battery	Logistics is very powerful Quality is very good Immediately repurchase	JD.com
Puning Dental	Cell Phone Battery	This user feels the product is better	JD.com
Puning Dental	Cell Phone Battery Repairer	is very fast, the customer service attitude is very positive, and questions are answered in a timely manner! Overall very satisfied!	JD.com
Puning Dental	Cell Phone Battery	This user feels the product is better	JD.com
Puning Dental	Cell Phone Battery	inexpensive and good quality, fast delivery, customer service is very good too!	JD.com
Puning Dental	Cell Phone Battery	I'll wait to use it before I say how I feel about it.	JD.com

Puning Dental	Cell Phone Battery Repairer	beautifully packaged/product! It is a good adapter to improve the battery life of mobilephone/optimize cell phonesystem/clean up cell phone garbage and improverunning speed of cell phone!Trustworthy/purchaseuse! I wish the manufacturer a prosperous business! We wish you a prosperous business! Popularity is strong and prosperous![Kudos][Buy]][Good][Buy]][Clap][Hands[Ah Huang][Handshake][Tongue]Rose]	JD.com
Puning Dental	Cell Phone Battery Repairer	The item has been received, the courier is also very fast, the product quality is very good, the workmanship is very refined, is genuine, the price is also very cheap, and the description is exactly the same, I give five-star praise.	JD.com
Puning Dental	Cell Phone Battery	Comments	JD.com
Puning Dental	Cell Phone Battery	Well, I can't say yet. Let's just use it and try it out! Uh-huh!	JD.com
Puning Dental	Cell Phone Battery	It looks good.	JD.com
Puning Dental	Cell Phone Battery	Repair has received, use good re-purchase wish the store business prosperity!	JD.com
Puning Dental	Cell Phone Battery	It works well.	JD.com
Puning Dental	Cell Phone Battery Repairer	Conversion efficiency: good<br>Compatibility: strong<br>Security: good	JD.com
Puning Dental	Cell Phone Battery	Cell phone adapter received looks good used well in buy a	JD.com
Puning Dental	Cell Phone Battery	Received the item and it looks great. The style is also beautiful and okay.	JD.com
[truncated: 462,407 more chars]
